# The altered transcriptome of pediatric myelodysplastic syndrome revealed by RNA sequencing

**DOI:** 10.1186/s13045-020-00974-3

**Published:** 2020-10-12

**Authors:** Lorena Zubovic, Silvano Piazza, Toma Tebaldi, Luca Cozzuto, Giuliana Palazzo, Viktoryia Sidarovich, Veronica De Sanctis, Roberto Bertorelli, Tim Lammens, Mattias Hofmans, Barbara De Moerloose, Julia Ponomarenko, Martina Pigazzi, Riccardo Masetti, Cristina Mecucci, Giuseppe Basso, Paolo Macchi

**Affiliations:** 1 Laboratory of Molecular and Cellular Neurobiology, Department of Cellular, Computational and Integrative Biology - CIBIO, University of Trento, Trento, Italy; 2 Bioinformatics Facility, Department of Cellular, Computational and Integrative Biology - CIBIO, University of Trento, Trento, Italy; 3 Section of Hematology, Department of Internal Medicine and Yale Comprehensive Cancer Center, Yale University School of Medicine, New Haven, CT USA; 4 Centre for Genomic Regulation (CRG), Barcelona, Spain; 5 High Throughput Screening (HTS) and Validation, CIBIO, University of Trento, Trento, Italy; 6 Next Generation Sequencing Core Facility LaBSSAH - CIBIO, University of Trento, Trento, Italy; 7 Department of Pediatric Hematology-Oncology and Stem Cell Transplantation, Ghent University Hospital, Ghent, Belgium; 8 University Pompeu Fabra, Barcelona, Spain; 9 Istituto Di Ricerca Pediatrica Città Della Speranza, Padua, Italy; 10 Associazione italiana ematologia e oncologia pediatrica (AIEOP), Bologna, Italy; 11 Centro di Ricerca Emato-Oncologico (CREO), Perugia, Italy; 12 Maternal and Child Health Department, Padua University, Padua, Italy; 13 IIGM-Italian Institute for Genomic Medicine, Turin, Italy

**Keywords:** Differentially expressed genes, Transcriptome, Pediatrics, Myelodysplastic syndrome

## Abstract

Pediatric myelodysplastic syndrome (PMDS) is a very rare and still poorly characterized disorder. In this work, we identified novel potential targets of PMDS by determining genes with aberrant expression, which can be correlated with PMDS pathogenesis. We identified 291 differentially expressed genes (DEGs) in PMDS patients, comprising genes involved in the regulation of apoptosis and the cell cycle, ribosome biogenesis, inflammation and adaptive immunity. Ten selected DEGs were then validated, confirming the sequencing data. These DEGs will potentially represent new molecular biomarkers and therapeutic targets for PMDS.

## To the Editor

**Fig. 1:**
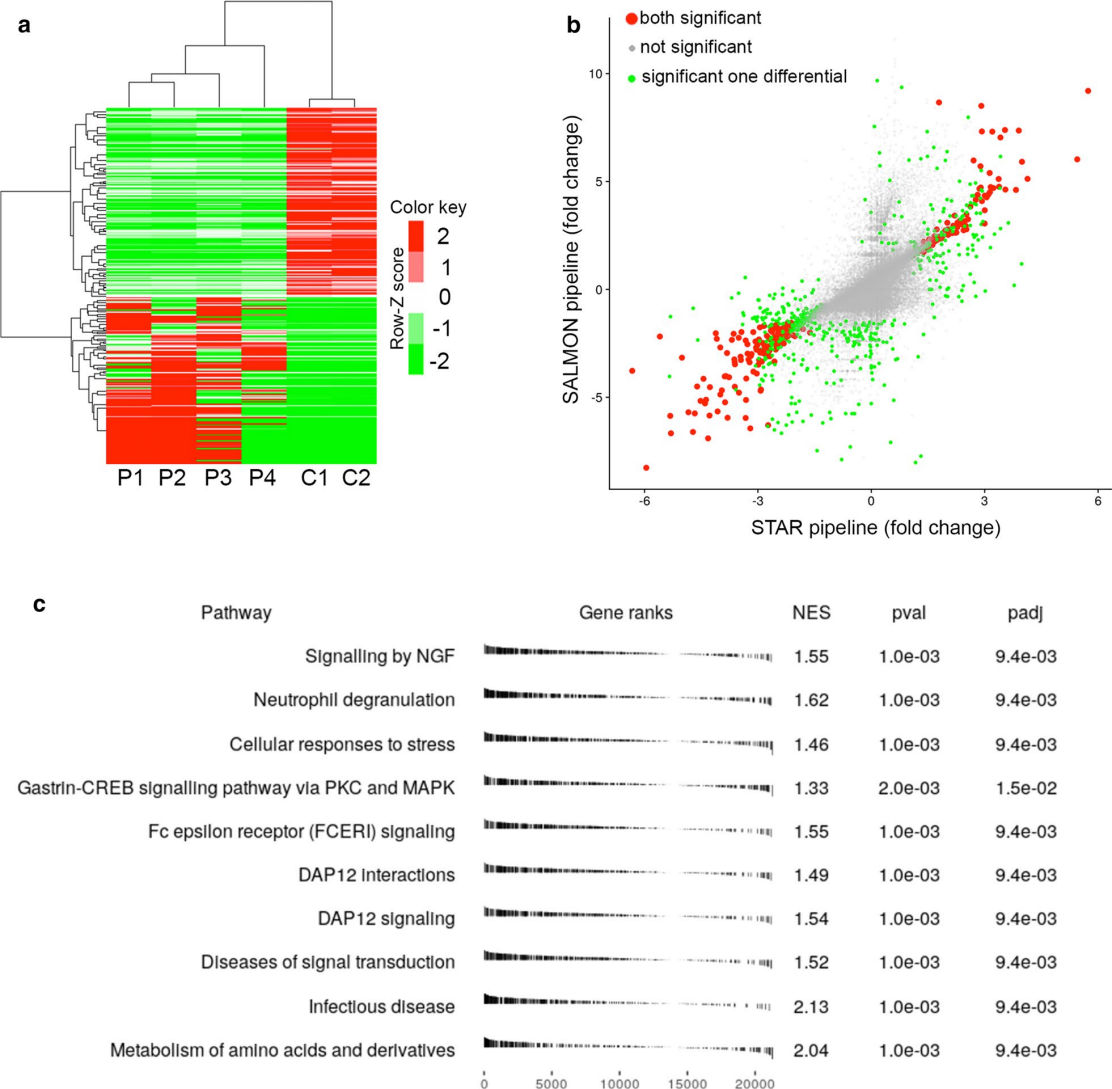
**a** Z-score hierarchical clustering analysis and heatmap of differentially expressed genes. The color scale means the gene expression standard deviations from the mean green. **b** Scatterplot of the differentially expressed genes obtained using the SALMON and STAR pipelines (different colors highlight genes identified as differentially expressed in none, one, or both pipelines). **c** Gene set enrichment analysis (GSEA) rank plots for top statistically significant Reactome pathways with Normalized Enrichment Score (NES)

MDSs are a heterogeneous group of clonal hematopoietic neoplasms. Although recent studies have shown that MDS and AML patients had different gene mutation patterns [1–4], the molecular underpinnings remain unknown [5–10]. To identify DEGs related to the PMDS, we performed RNA-seq in 4 patients with primary PMDS and in 2 control pediatric samples (Additional file 1: Figures S1A-B). Because of the limited number of samples and to limit the false positives, we used two independent bioinformatics pipelines, STAR + DESeq2 and SALMON + edgeR, and considered only genes differentially expressed in both pipelines. Hierarchical clustering showed that PMDS patients and controls clustered in two distinct groups (Fig. 1a). In total, 651 DEGs were identified by STAR + DESeq2 and 616 DEGs by SALMON + edgeR (Fig. 1B; Additional file 1: Figures S1C-D). 291 DEGs were identified by both pipelines among which 136 genes were upregulated and 155 downregulated in patients (Additional file 1: Table 1). As a further validation, we used the LPEseq method. The concordance of the genes in the ranks of the differential gene lists was remarkably high (Additional file 1: Figures S1E-G). We then used GSEA to identify altered pathways from the Reactome database (Web reference 1) (Fig. 1c). The Enrichr enrichment analysis tool revealed that DEGs in PMDS are mainly related to pathways associated with the cell abnormal activity, immune and inflammatory systems and erythropoiesis (Additional file 1: Figure S2A).

**Fig. 2:**
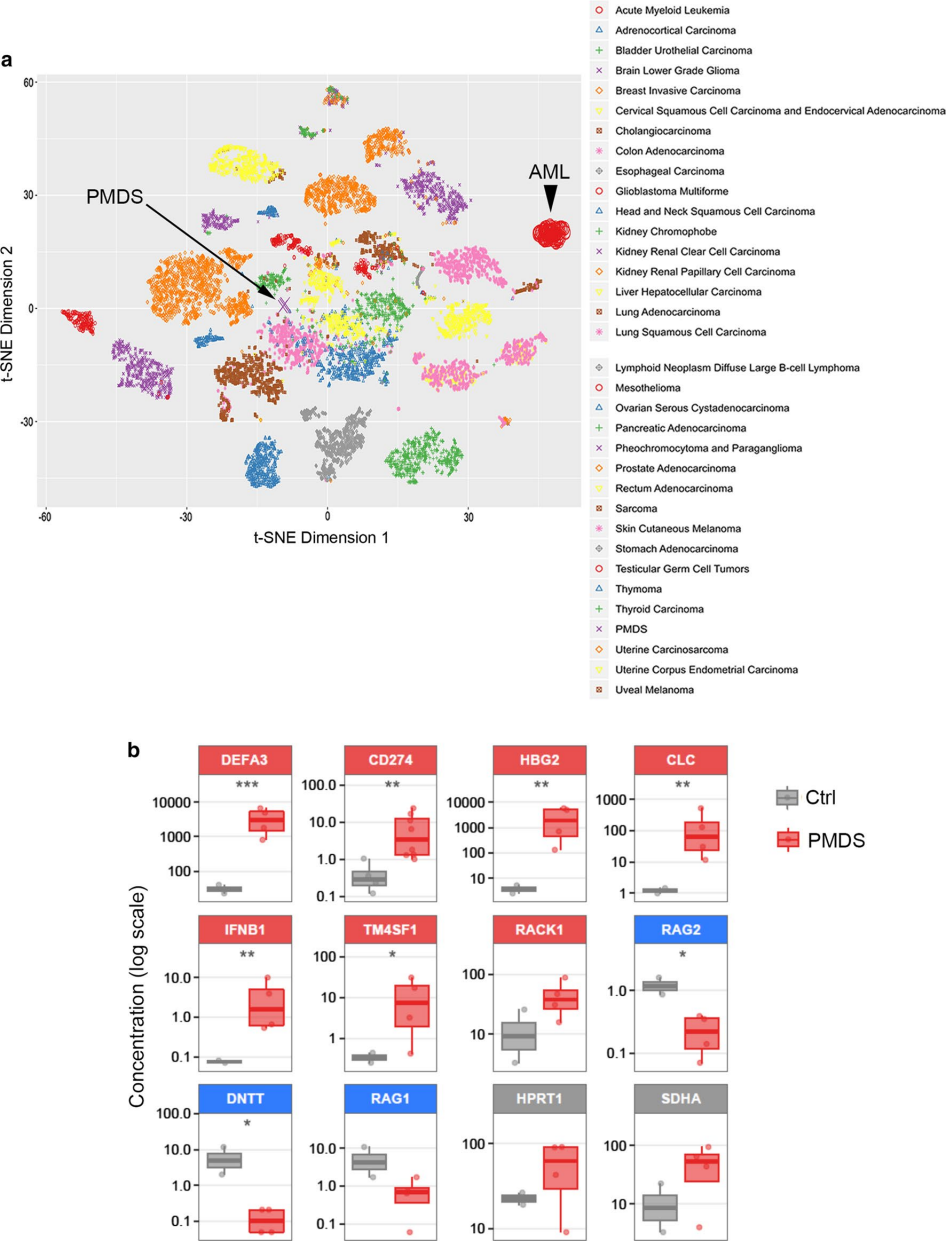
**a** T-distributed stochastic neighbor embedding (t-SNE) plot in the expression space of several cancer datasets, plotting the results of the two principal dimensions. The data were obtained from the GDC-PAN cancer data Portal. The PMDS samples do not cluster near other tumor types, AML in particular (black arrowhead), showing a distinct profile. **b **Boxplot: ddPCR analysis of twelve genes, comparing expression levels between controls and PMDS patients. For each gene, box–whisker plots of concentration values are shown. Genes are classified as upregulated (red), downregulated (blue) and reference (grey). Significant changes in cDNA concentration between control and patients are highlighted (one-tailed *t* test, corrected for unequal variances *p < 0.05, **p < 0.01, ***p < 0.001)

Further, we compared our data with the transcriptomic profiles from TCGA database. Interestingly, we found a clear distinction of PMDS from all other types of tumors (Fig. 2a; Additional file 1: Figure S2B). Moreover, the DEGs profile was able to divide tumors into three distinct groups (Additional file 1: Figure S3A). As for control samples, we integrated the transcriptomic data from the GTEx (Web reference 2) and observed a clear separation between blood related tissues and other normal tissues (Additional file 1: Figures S3B). Finally, we compared the DEGs gene list with the gene sets available in the Enrichr database specifically for “Diseases/Drugs” and “Cell types “categories (Additional file 1: Tables 2–3). We confirmed that the DEGs identified in PMDS are significantly connected with blood tissues and blood disorders (Additional file 1: Figure S3C).

A comparison of our PMDS DEGs with multiple RNA-seq datasets from adult MDS samples revealed a statistically significant overlap (67 out of 136 DEGs). Nonetheless, 69 upregulated genes and almost all downregulated genes were unique in PMDS (Additional file 1: Figure S4A-B; Additional file 1: Table 4).

Then, we validated the most statistically significant and biologically relevant DEGs either up- or downregulated. Analysis by ddPCR showed significant differences between patient and control samples (Fig. 2b). The log2 fold-change values for all 10 genes were highly correlated (Additional file 1: Figure S5). We also validated the DEGs in 6 new PMDS patients (Additional file 1: Figure S6). Additionally, we compared our data with 36 pediatric patients (3). The comparative data on 10 DEGs in PMDS and validation are shown in the Additional file 1: Figure S7.

In conclusion, we have identified 291 DEGs that correlate with the PMDS which might represent novel candidate genes for therapeutic intervention. Although a larger study cohort would be desirable, our data suggest that at the level of gene expression the PMDS is indeed a distinct disorder.

## Supplementary information

**Additional file 1.** The altered transcriptome of pediatric myelodysplastic syndrome revealed by RNA sequencing.

## Data Availability

All data generated or analysed during this study are included in this published article and its supplementary information files.

## Abbreviations

PMDS: Pediatric Myelodysplastic Syndrome
BM: Bone marrow
DEGs: : Differentially expressed genes
RNA-seq: RNA sequencing
MDSs: Myelodysplastic syndromes
AML: Acute myeloid leukemia
GSEA: Gene set enrichment analysis
GO: Gene ontology
HPO: Human phenotype ontologies
TCGA: The cancer genome atlas
GTEx: Genotype tissue expression
ddPCR: Digital droplet PCR

## References

[1] Rau AT, Shreedhara AK, Kumar S (2012). Myelodysplastic syndromes in children: where are we today?. Ochsner J.

[2] Yu J, Li Y, Li T, Li Y, Xing H, Sun H, Sun L, Wan D, Liu Y, Xie X, Jiang Z (2020). Gene mutational analysis by NGS and its clinical significance in patients with myelodysplastic syndrome and acute myeloid leukemia. Exp Hematol Oncol.

[3] Schwartz JR, Ma J, Lamprecht T, Walsh M, Wang S, Bryant V (2017). The genomic landscape of pediatric myelodysplastic syndromes. Nature communications.

[4] Locatelli F, Zecca M, Pession A, Maserati E, De Stefano P, Severi F (1995). Myelodysplastic syndromes: the pediatric point of view. Haematologica.

[5] Bresolin S, Trentin L, Zecca M, Giordan M, Sainati L, Locatelli F (2012). Gene expression signatures of pediatric myelodysplastic syndromes are associated with risk of evolution into acute myeloid leukemia. Leukemia.

[6] Papaemmanuil E, Gerstung M, Malcovati L, Tauro S, Gundem G, Van Loo P (2013). Clinical and biological implications of driver mutations in myelodysplastic syndromes. Blood.

[7] Cazzola M, Della Porta MG, Malcovati L (2013). The genetic basis of myelodysplasia and its clinical relevance. Blood.

[8] Hrustincova A, Krejcik Z, Kundrat D, Szikszai K, Belickova M, Pecherkova P, Klema J, Vesela J, Hruba M, Cermak J, Hrdinova T, Krijt M, Valka J, Jonasova A, Merkerova MD (2020). Circulating small noncoding RNAs have specific expression patterns in plasma and extracellular vesicles in myelodysplastic syndromes and are predictive of patient outcome. Cells.

[9] Yu J, Li Y, Zhang D, Wan D, Jiang Z (2020). Clinical implications of recurrent gene mutations in acute myeloid leukemia. Exp Hematol Oncol.

[10] Bejar R (2017). Implications of molecular genetic diversity in myelodysplastic syndromes. Curr Opin Hematol.

