# Supplementary material for: The altered transcriptome of pediatric myelodysplastic syndrome revealed by RNA sequencing
Source: J Hematol Oncol. 2020 Oct 12;13:135. doi: 10.1186/s13045-020-00974-3 (PMC7552545; doi:10.1186/s13045-020-00974-3)
Supplement: Supplementary file 1 — Additional file 1. The altered transcriptome of pediatric myelodysplastic syndrome revealed by RNA sequencing. [file 13045_2020_974_MOESM1_ESM.pdf]

SUPPLEMENTARY DATA

The altered transcriptome of pediatric myelodysplastic syndrome revealed by RNA sequencing

Lorena Zubovic, Silvano Piazza, Toma Tebaldi, Luca Cozzuto, Giuliana Palazzo, Viktoryia Sidarovich, Veronica De Sanctis, Roberto Bertorelli, Tim Lammens, Mattias Hofmans, Barbara De Moerloose, Julia Ponomarenko, Martina Pigazzi, Ricardo Masetti, Cristina Mecucci, Giuseppe Basso and Paolo Macchi.

Supplementary Methods

Patient material. For the RNA-seq analysis we used samples taken at the time of diagnosis from four pediatric patients with MDS and two control samples, containing three Refractory Anemia with Excess Blasts (MDS EB-1, P1, P2 (monosomy, -7) and P4), one MDS EB in transformation (MDS EB-2, P3) and two non-neoplastic pediatric control samples (C1 and C2). All 4 PMDS patient samples were obtained from the Associazione Italiana di Ematologia e Oncologia Pediatrica (AIEOP). Two control samples were obtained from the Centro Ricerche Emato-Oncologiche (CREO). All RNA samples from PMDS patients and controls were obtained from the BM blood. Informed consent was obtained from their legal guardians, as part of sample collection by the European Working Organization Group (EWOG). Institutional review board approval was granted by the Comitato Etico per la Sperimentazione con l'Essere Umano (CESP) of the University of Trento.

To validate further ten DEGs, we analyzed RNA samples from six new patients: P5 (MDS EB-1) and P6 (MDS-EB1, monosomy -7) from the Associazione Italiana di Ematologia e Oncologia Pediatrica (AIEOP), B1, B2, B4 (MDS EB-1) and B4 (MDS EB-2) obtained from Department of Pediatric Hematology-Oncology and Stem Cell Transplantation, Ghent University Hospital, Belgium. All RNAs were obtained from the BM blood (**Supplementary Figure S6A**). For the patients P5 and P6 informed consent was obtained from their legal guardians, as part of sample collection by the European Working Organization Group (EWOG). For the B1, B2, B3 and B4 informed consent was obtained from their legal guardians, as part of sample collection by the Department of Pediatric Hematology-Oncology and Stem Cell Transplantation, Ghent University Hospital, Belgium.

RNA sequencing. IlluminaHiSeq 2500 (Illumina, Inc., San Diego, CA, USA) was used for the sequencing process. Briefly, Ribosomal RNA depleted strand-specific RNA libraries were generated with the TruSeq Stranded Total RNA HT sample preparation kit with Ribo-Zero Gold starting from 500ng of total RNA.rRNA-depleted RNA recovered was fragmented for 4 min and processed in a random-primed, first-strand cDNA synthesis, followed by second strand cDNA (dUTP-marking) synthesis using DNA Polymerase I and RNaseH. A single 'A' base was added to these cDNA fragments and, in a subsequent ligation, Illumina-specific barcoded sequencing adapters were incorporated. The products were purified and enriched with PCR to create the final cDNA library. Each individual library was quantified, and quality controlled using QubitFluorometer (Thermo Scientific), LabChip GX (Perkin Elmer). After libraries equimolar pooling, the final pool was quality checked again with Qubit, LabChip GX and qPCR (KAPA and BIORAD). The adaptor-tagged pool of libraries was loaded in the Illumina Hiseq2500 for cluster generation and sequencing. 50 bp paired end reads were generated for all samples.

Computational analysis. For every sample, at least 80 million reads were sequenced. Raw sequence files were subjected to quality control analysis using FastQC (v 1.3) (Web reference 4). Gene quantification was conducted using two different methodologies: STAR (v2.5.3a) (13) (assigned to a gene using the GENCODE annotation (v27) (**Supplementary reference 1**) using the STAR function "quantModeGeneCounts" (**Supplementary reference 2)** and SALMON (**Supplementary reference 3**) both mapped to the human genome version GRCh38. Salmon performs transcript-level quantification estimates from RNA-seq data; it achieves its accuracy and speed via a number of different innovations, including the use of quasi-mapping (an accurate but fast-to-compute proxy for traditional read alignments) and a two-phase inference procedure that makes use of massively-parallel stochastic collapsed variation inference. Reads are mapped over exons by STAR/HT-SEQ COUNT pipeline and are proportionally assigned to the human transcripts by SALMON pipeline. Read counts generated by STAR were analyzed by using the DESeq2 package (**Supplementary reference 4**) for detecting genes that were differentially expressed. An adjusted p-value cut off of 0.05 was decided as threshold for detection of DEGs. Similarly, read counts generated by SALMON were analysed using edgeR, and applying the same cutoffs. Genes with absolute log2 fold change (FC) >1 and false discovery rate (FDR) <0.05 were considered to be differentially expressed. Starting from the SALMON expression matrix, genes that were considered as differentially expressed were analyzed using hierarchical clustering method (Cluster 3.0, (Web reference 6). Visualization of the clustering and heatmap of log2-normalized values were obtained using Java Treeview. Gene set enrichment analysis was performed starting from the common DEGs, using fgsea package, performing 10000 permutations and using as database the combination of the Reactome dataset (human profile, accessed in January 2018, from fgsea package) and the Molecular signature database (msigdb, V6), a comprehensive knowledge base for gene

sets (**Supplementary reference 5**). For the functional annotations analyses we used the Enrichr web tool (**Supplementary reference 6**). The enriched annotation table results obtained were then downloaded to be processed and visualized using the ggplot2 packages. The Cancer Genome Atlas (TCGA) gene expression data and the GTEX data were obtained via the GDC data portal hub (https://portal.gdc.cancer.gov/, last accessed July 2019) and integrated with our data using the COMBAT algorithm (**Supplementary reference 7**). To explore the high-dimensional property of the Integrated data we used the non-linear dimensionality reduction algorithm t-SNE (t-Distributed Stochastic Neighbor Embedding) https://github.com/jkrijthe/Rtsne. To evaluate the peculiar properties of the DEGs against the TCGA and GTEX dataset, we used GEPIA2 web tool using standard parameters (**Supplementary reference 8**). We also integrated our results with the LPEseq method (**Supplementary reference 9**). Since it starts from the raw counts, we applied LPE-seq in parallel to the Salmon quantifications and HT-seq derived counts. Since the LPEseq table results of the differential analysis does not include an actual estimation of the fold change but only the log2 means of the two groups, we used the difference between them as log fold change. In order to compare in a proper manner the differential gene lists, we decided to employ a concordance strategy using the correspondence at the top (CAT) plots. To create the CAT plot (**Supplementary reference 10**), we ranked genes by log fold change using the all four lists (LPEseq_HTSEQ, LPEseq_SALMON, SALMON_pipeline and EDGER_pipeline). We then created lists of candidate genes for a range of list sizes. CAT curves show the proportion of genes in common plotted against the size of the lists. The correlations were much higher (30% higher on average) in LPEseq_STAR_vs_STAR_pipeline and in LPEseq_SALMON_vs_SALMON_pipeline compared to our original comparison STAR_pipeline_vs_SALMON_pipeline

For the integrative analysis, we retrieved RNA-seq gene expression values (FPKM) from 36 pediatric patients with MDS, previously sequenced in *Schwartz JR et al., 2017* (3). To remove batch effects, the integration of the datasets was performed with the ComBat algorithm.

Droplet Digital PCR. Droplet Digital PCR (ddPCR) was performed according to the manufacturer's protocol. Briefly, 5ng of cDNA from each sample were added to the reaction tube. 20 µg of the mixture were loaded into a sample well of a DG8™ Cartridge for QX200 Droplet Generator followed by 70 µg of Droplet Generation Oil for Probes into the oil wells. The droplets were carefully transferred into a clean 96-well plate, sealed with the PX1 PCR Plate Sealer and put in the thermal cycler (QX200 Droplet Reader). QuantaSoft™ Software was used to set up a plate layout according to the experimental design. The automatic threshold applied to the 1-D or 2-D amplitude data was modified by establishing threshold manually. The relative expression value of each of DEG was calculated as the ratio between the droplet numbers for the studied gene and the two reference genes, Succinate Dehydrogenase Complex Flavoprotein Subunit A (*SDHA*) and Hypoxanthine Phosphoribosyltransferase 1 (*HPRT1*).

Statistical analysis. Statistical significance for droplet PCR concentrations (molecules/microliter) was tested with one-tailed t-test, unequal variances (*$P<0.05$, **$P<0.01$, ***$P<0.001$). The overall agreement between droplet PCR and RNA-seq fold changes was measured using the Pearson correlation coefficient.

Supplementary Figure Legends

Supplementary Figure SF1

A) Clinical characteristics of the patients enrolled in the study. **B)** Overview of the pipeline followed in this work. **C)** Gene expression scatterplot: example comparison of a sample gene expression performed with an alignment program (STAR) and pseudo-alignment program (SALMON). Interestingly, even if the correlation is high, some differences are present, especially for the low-intensity signals. Significantly enriched pathways were identified, four of which are related to the immune system: neutrophil degranulation, FCERI signaling, DAP12 interactions and DAP12 signaling. **D)** Correlation heatmap of the samples based on the expression obtained with SALMON and STAR. We observed that the samples could be grouped in pairs. **E)** Concordance at the top (CAT) plot of the log fold change (LFC). Starting from the four LFC lists form, LPEseq_HTSEQ, LPEseq_SALMON, SALMON_pipeline and EDGER_pipeline that were compared in pairs. The curves represent at each position (rank) in the DEG analysis the concordance value of two lists. **F)** Comparisons of the functional annotation scores in four different pipelines. We observe the density of the scores in the diagonal, in the upper part their correlation values in pairs, and in lower part the scatter plot in pairs. **G)** The heatmap of the subset of Gene Ontology score, in a simple color code: white if less than 2 (not significant), in green if less than 10 and greater than 2, and in red if greater than 5.

Supplementary Figure SF2

A) Functional Analysis: left panels: bar-plots representing the combined score, as defined in the Enrichr tool, for the GO terms significantly enriched for the upregulated (red) and downregulated (blue) genes; right panels: network map of the ontology terms from the Human Phenotype Ontology (HPO). An extensive investigation of GO and UniProt has shown that the majority of the 291 DEGs identified were previously reported to play roles in various biological processes and cellular pathways: ribosome dysfunction, inflammation, innate and adaptive immunity, oxygen transport, regulation of T-cell anergy, apoptosis and cell cycle, DNA recombination and glycolysis. **B)** T-distributed Stochastic Neighbor Embedding (t-SNE) representation of PMDS, AML and normal samples, each color corresponding to a different class. Please note that some samples are so close to each other that are not distinguishable.

Supplementary Figure SF3

A) Principal Component Analysis plot of the DEGs in TCGA dataset and **(B)** GTEx dataset. DEGs profile divides tumors into three distinct groups: one group that corresponds to AML (arrowhead), another group that even though heterogeneous comprises Thymoma tumors and lymphoid neoplasm Diffuse Large B-cell Lymphoma, and a third group that includes solid tumors only. **C)** Bar plots representing the combined score, as defined in Enrichr tool, for the Drug Signature Database (DSigDB) and the Drug Matrix database for the DEGs.

Supplementary Figure SF4

A) Network analysis of DEGs in PMDS (upregulated in red, downregulated in blue) and associated with aberrant expression in adult MDS transcriptome datasets. **B)** Heatmap displaying significant overlaps between genes differentially expressed in PMDS and tissue specific expression profiles from the ARCHS4 collection (Web reference 3).

Supplementary Figure SF5

Scatterplot: comparison between RNA-seq and ddPCR analysis. Genes are classified as upregulated (red), downregulated (blue) and reference (grey) according to RNA-seq results. The Pearson correlation coefficient is provided. Linear regression is also shown.

Supplementary Figure SF6

A) Clinical characteristics of 6 new patients used for the validation studies. **B)** Boxplot: digital PCR analysis of twelve genes, comparing expression levels between controls and PMDS patients. For each gene, box-whisker plots of concentration values are shown. Genes are classified as upregulated (red), downregulated (blue) and reference (grey) according to the RNA-seq analysis. Significant changes in cDNA concentration between control and patients are highlighted (one-tailed t-test, corrected for unequal variances *p-value <0.05, **p-value <0.01, ***p-value<0.001). **C)** Scatterplot: comparison between RNA-seq and ddPCR analysis. Genes are classified as upregulated (red), downregulated (blue) and reference (grey) according to RNA-seq results. The Pearson correlation coefficient is provided. Linear regression is also shown.

Supplementary Figure SF7

Integrative analysis of the transcript levels of 10 DEGs with altered expression in PMDS (7 up-regulated, 3 down-regulated). Data are represented as mean +-SEM for each class of samples: CTRL and PMDS patients sequenced by us, MDS-EB, MDS-RCC, MDS/MPN and AML-MRC pediatric patients sequenced in (3). The observed alterations in expression are fully confirmed by the integrative analysis.

Supplementary Table 1.DEGs list - Excel

Supplementary Table 2.DSigDB table

Supplementary Table 3. Drug Matrix table

Supplementary Table 4. Common DEGs and unique DEGs for PMDS - Excel

Supplementary References

1. Harrow J, Frankish A, Gonzalez JM, Tapanari E, Diekhans M, Kokocinski F, et al. GENCODE: the reference human genome annotation for The ENCODE Project. Genome research. 2012; 1760-74.

2. Dobin A, Davis CA, Schlesinger F, Drenkow J, Zaleski C, Jha S, et al. STAR: ultrafast universal RNA-seq aligner. Bioinformatics. 2013; 29:15-21.

3. Patro R, Duggal G, Love MI, Irizarry RA, Kingsford C. Salmon provides fast and bias-aware quantification of transcript expression. Nature methods. 2017;14:417-9.

4. Love MI, Huber W, Anders S. Moderated estimation of fold change and dispersion for RNA-seq data with DESeq2. Genome biology. 2014;15:550.

5. Subramanian A, Tamayo P, Mootha VK, Mukherjee S, Ebert BL, Gillette MA, et al. Gene set enrichment analysis: a knowledge-based approach for interpreting genome-wide expression profiles. Proceedings of the National Academy of Sciences of the United States of America. 2005;102:15545-50.

6. Chen EY, Tan CM, Kou Y, Duan Q, Wang Z, Meirelles GV, et al. Enrichr: interactive and collaborative HTML5 gene list enrichment analysis tool. BMC bioinformatics. 2013;14:128.

7. Leek JT, Storey JD. Capturing heterogeneity in gene expression studies by surrogate variable analysis. PLoS genetics. 2007; 9:1724-35.

8. Tang Z, Kang B, Li C, Chen T, Zhang Z. GEPIA2: an enhanced web server for large-scale expression profiling and interactive analysis. Nucleic acids research. 2019; 47:W556-W60.

9. Gim J, Won S, Park T. LPEseq: Local-Pooled-Error Test for RNA Sequencing Experiments with a Small Number of Replicates. PLOS ONE. 2016; 11: e0159182.

10. Bingqing L, Zhen P (2019) Stability of methods for differential expression analysis of RNA-seq data. BMC Genomics. 2019;20:35.

Supplementary Web references

1.https://reactome.org/

2.https://gtexportal.org/home/

3.https://amp.pharm.mssm.edu/archs4/

4. http://www.bioinformatics.babraham.ac.uk/projects/fastqc/

5. https://github.com/jkrijthe/Rtsne

6. http://bonsai.hgc.jp/~mdehoon/software/cluster/software.htm

Zubovic et al., Supplementary Figure S1

A

Samples	Age of diagnosis (years)	Sex	Age of birth	Caryotype	Diagnosis
P1	7	Male	1999	46 XY	MDS EB-1
P2	12	Male	1994	45 XY, -7	MDS EB-1
P3	8	Female	1999	46 XX	MDS EB-2
P4	9	Female	2001	46 XX	MDS EB-1
C1	9	Male	2007	46 XY	Non neoplastic
C2	14	Female	2003	46 XX	Non neoplastic

B

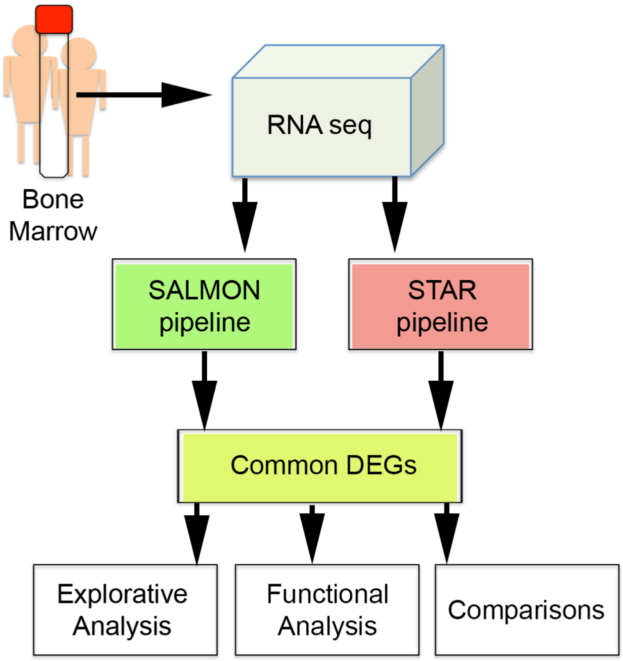

C

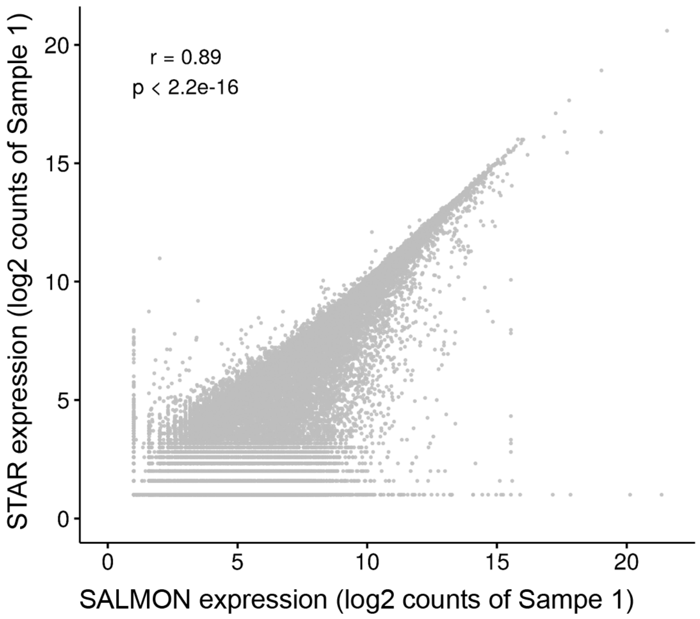

D

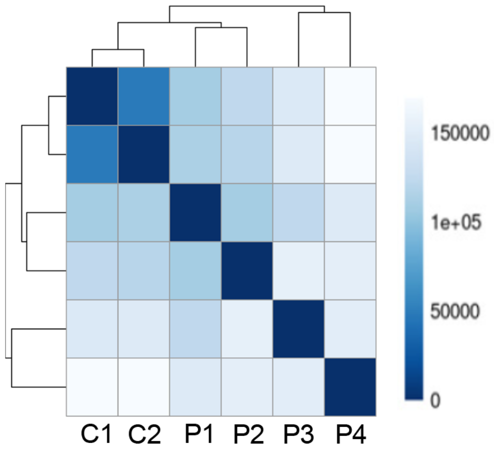

Zubovic et al., Supplementary Figure S1

E

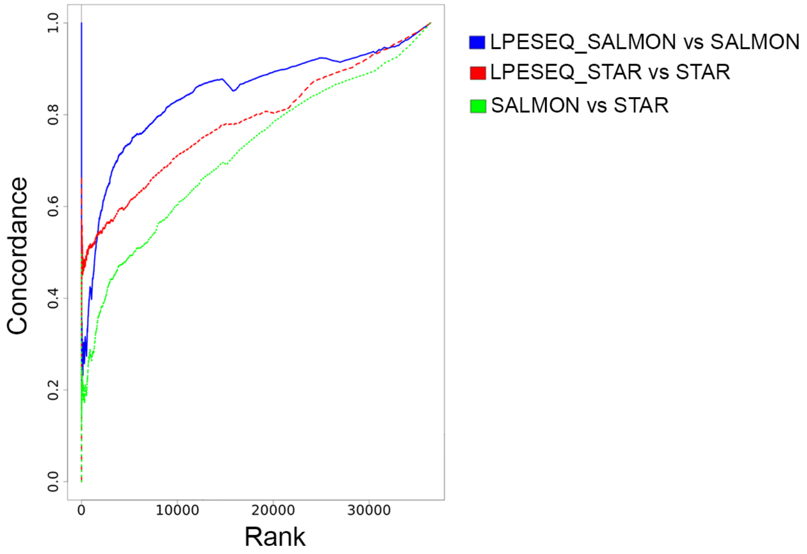

F

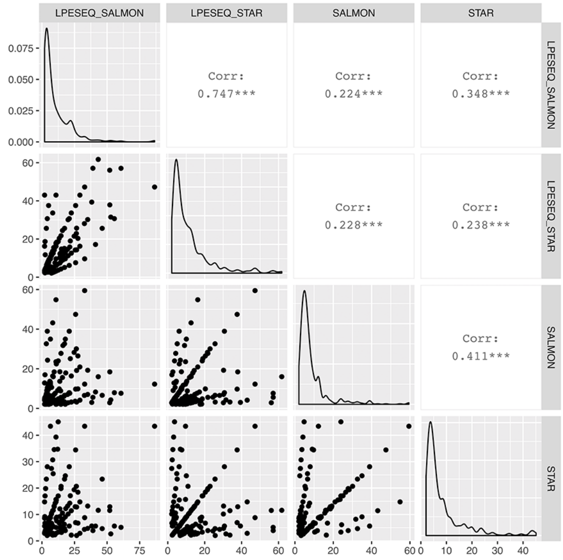

G

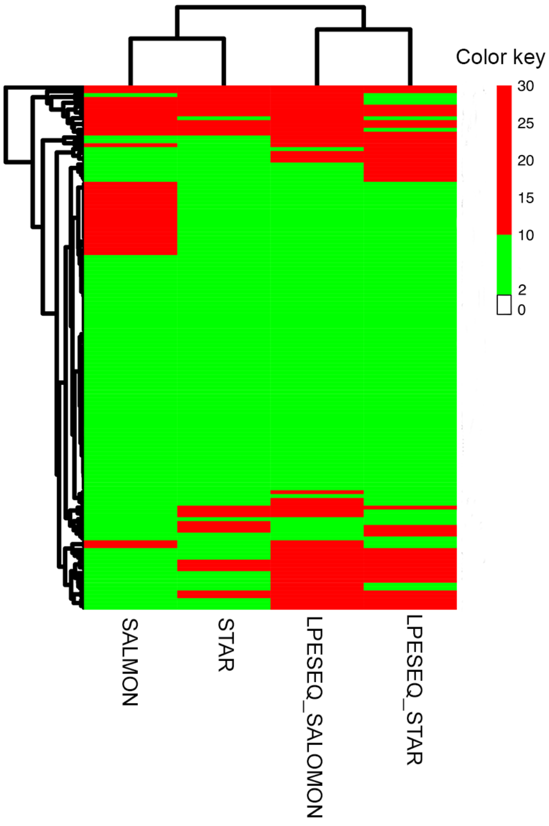

Zubovic et al., Supplementary Figure S2

A

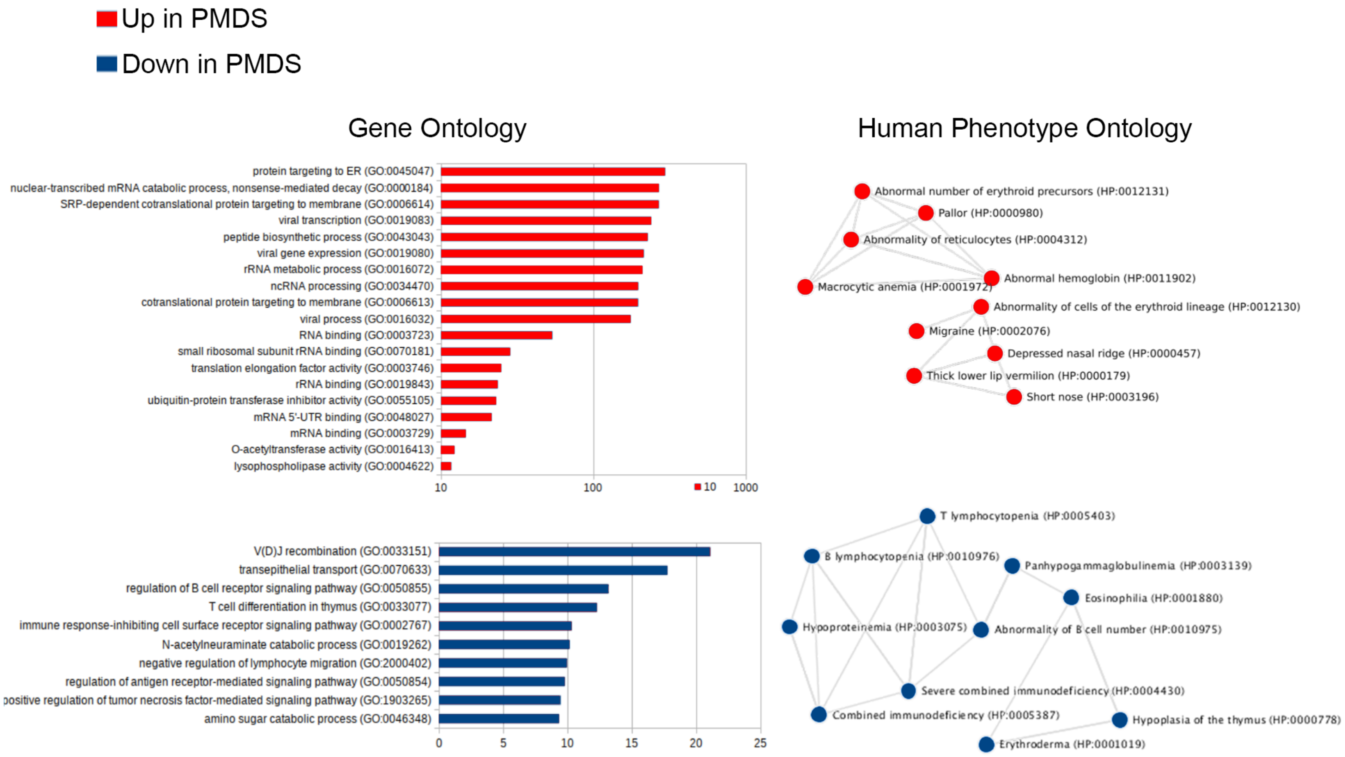

B

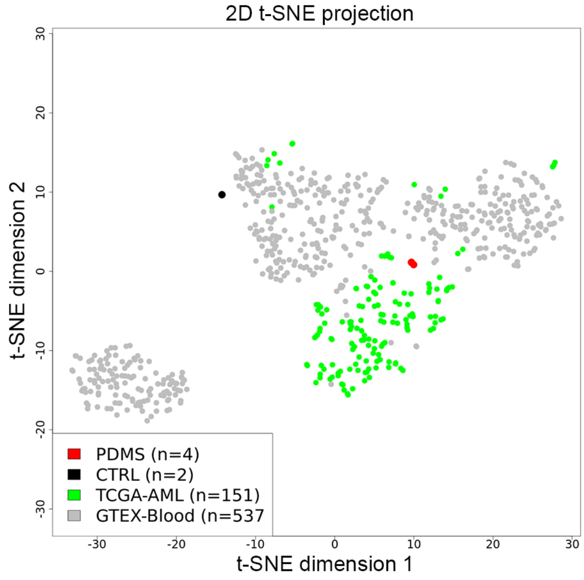

Zubovic et al., Supplementary Figure S3

A

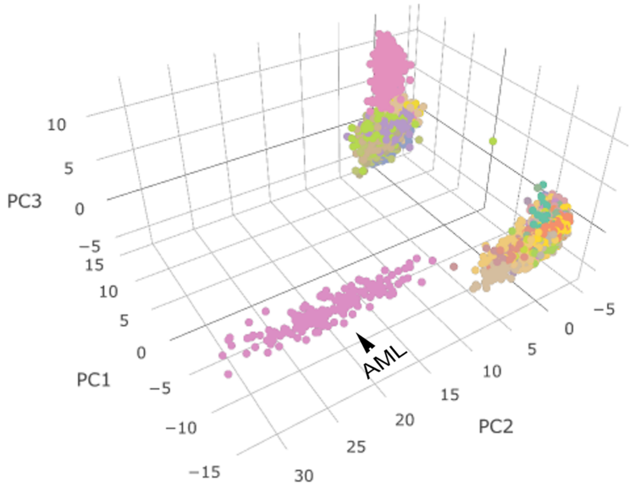

B

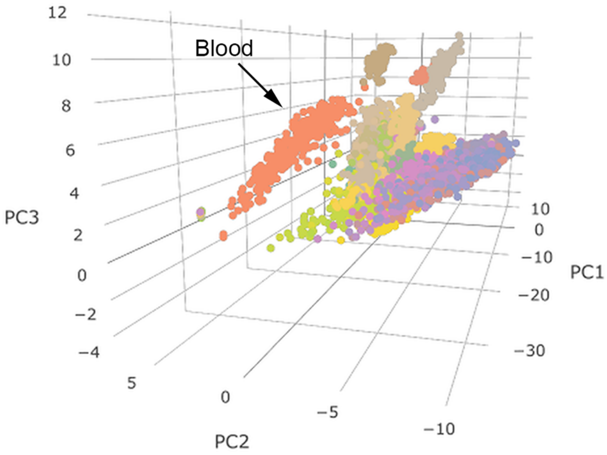

C

DSigDB

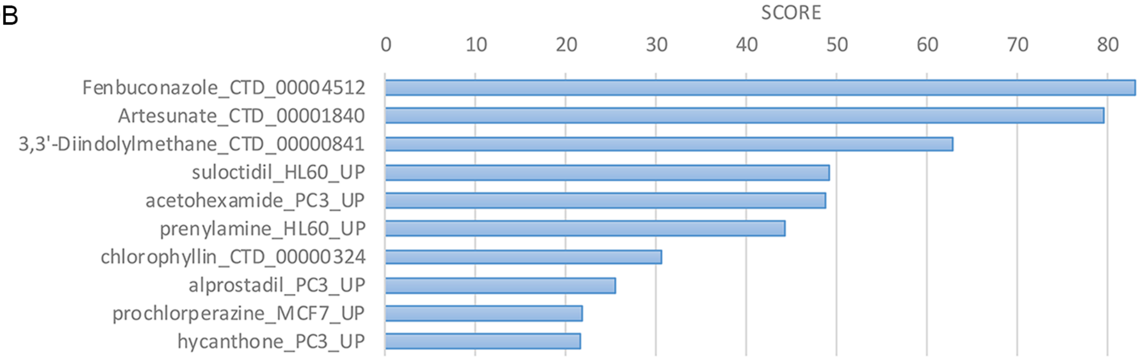

DrugMatrix

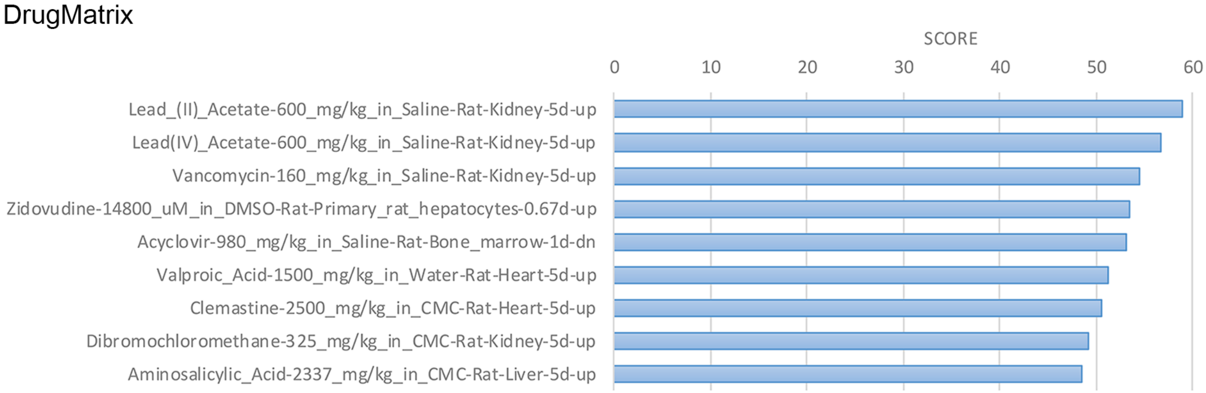

Zubovic et al., Supplementary Figure S4

A

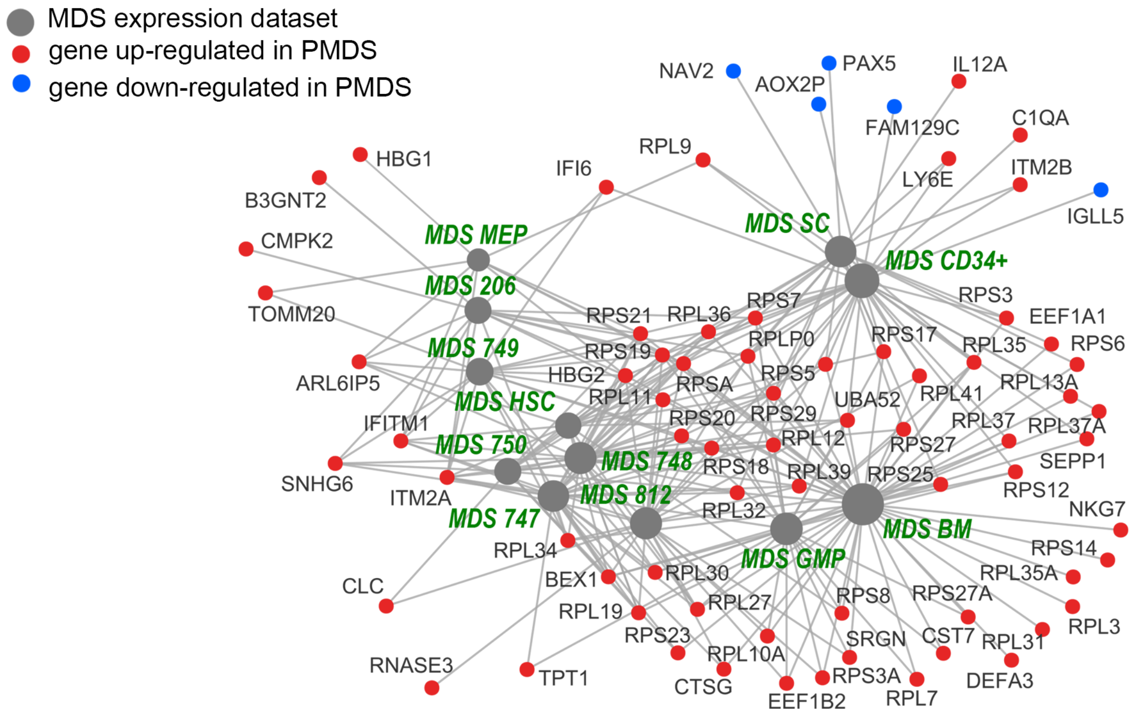

B

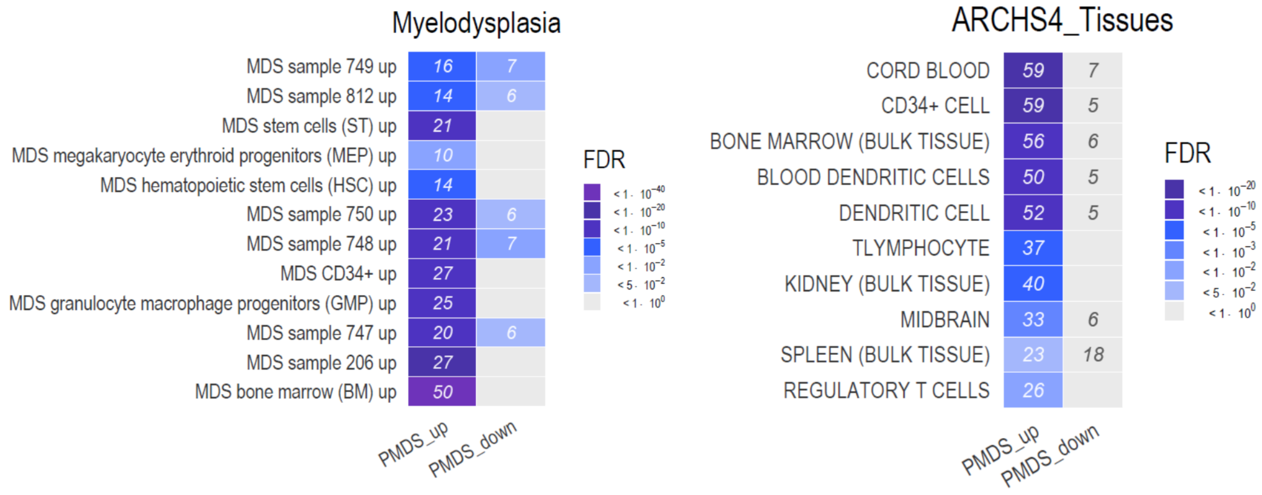

Zubovic et al., Supplementary Figure S5

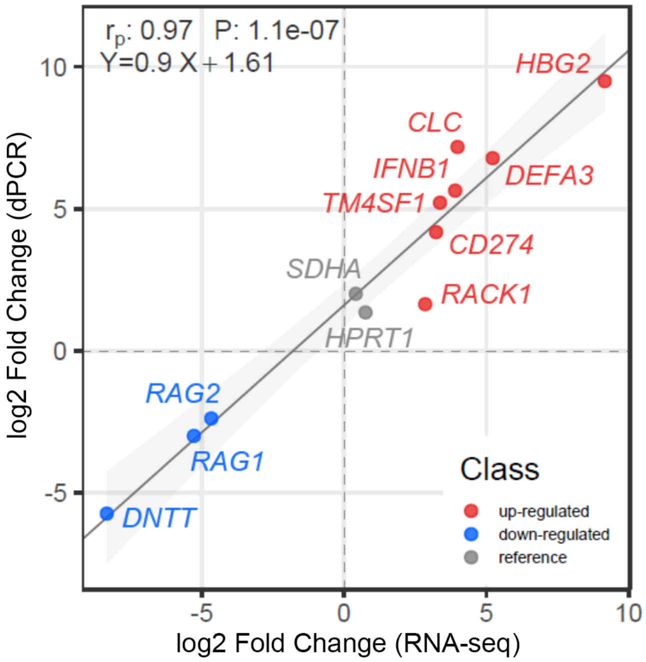

Zubovic et al., Supplementary Figure S6

A

Samples	Age of diagnosis (years)	Sex	Year of birth	Caryotype	Diagnosis
**P5**	14	Female	2006	45 XX, -7	MDS EB-1
**P6**	10	Female	2010	46 XX	MDS EB-1
**B1**	5	Female	2011	46 XX	MDS EB-1
**B2**	7	Male	2011	46 XY	MDS EB-1
**B3**	12	Male	2006	46 XY	MDS EB-2
**B4**	11	Female	2009	46 XX	MDS EB-1

B

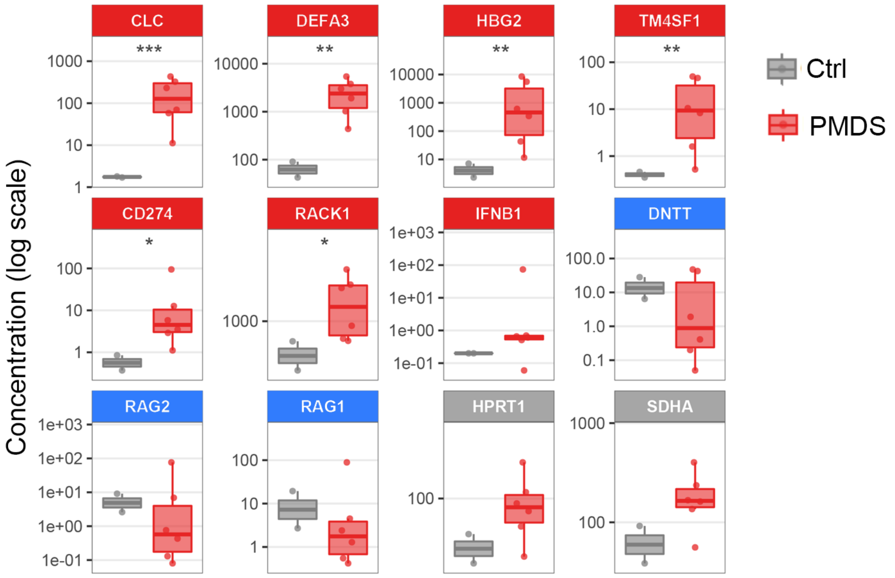

C

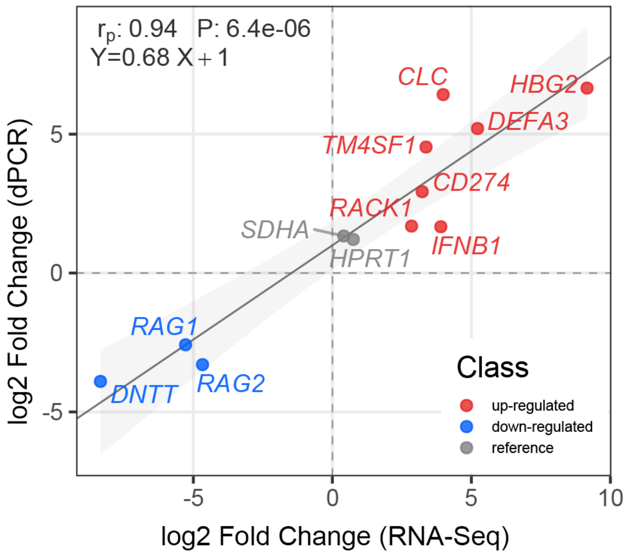

Zubovic et al., Supplementary Figure S7

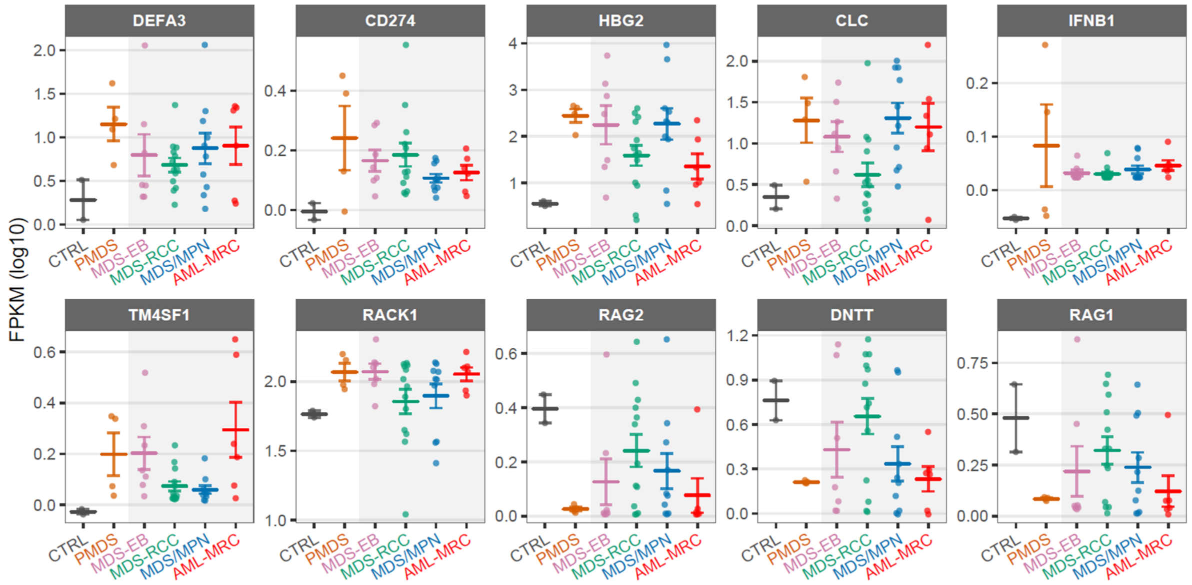

Supplementary Table 1.

Upregulated DEGs	Downregulated DEGs
RPS8	CFAP73
RPS6	MIR6075
RPS7	POU2AF1
DEFA3	RP11-206L10.2
SEPP1	POU4F1
RPSA	TSSC1-IT1
TM4SF1	FAM157C
KIAA1217	RP11-563J2.2
EEF1A1	MIR7848
CXCL11	ASH2LP1
WT1	RP4-666F24.3
DOK4	MIR3155A
COL5A3	AC084117.3
LY6E	RN7SL683P
CLC	RP4-669L17.10
LINC01218	ROR1
RPL27	MIR210HG
RP11-521I2.3	HSPA1B
ZNF792	TMEM63A
SAMD9	RP1-161N10.1
RPL11	MIR3909
IFITM1	C6ORF99
RNU6-8	IGKV1D-22
RPL12	FAM129C
IFI6	LAMP5
CCDC102A	VPREB3
TOMM20	RP11-723D22.2
PYURF	RP1-168P16.2
CLP1	VPREB1
NRBF2	RP11-452K12.6
RPS5	NPTX1
HOXA1	APBB2
RPS3	GJA6P
RPL19	SLC16A3
HBG1	RP11-553K8.2
SLC6A19	RP11-10J21.4
RPL41	NTNG2
HBG2	CTD-3137H5.4
NKG7	LINC00671

Supplementary Table 1.

DOLK	TRPC6
SNHG6	CYP4F3
COMMD6	MIR27A
RPS27	RP13-554M15.7
EFNA1	RN7SKP134
RPS29	CTC-436K13.1
HOXB5	RP11-605P22.1
RPS21	CTC-436K13.2
RPS20	CDHR2
ITM2A	HIF1A-AS2
RPS23	IGKV2D-24
ITM2B	RP11-804M7.2
RPS25	OR10AA1P
RPL3	AOX2P
RPL31	RP11-5L12.1
CD274	CEMIP
DHH	DPP10
RPL30	RN7SL600P
BEX1	RP11-164J13.1
RPL32	HHIP
RPL35	KB-208E9.1
RPL34	TPM3P7
GDE1	ABCA13
RPL9	RP11-295K3.1
RPL7	FCRLA
EEF1B2	LARP1P1
ADGRG5	TMEM104
RPS18	RP11-147L13.14
RPS17	AMDHD2
C6ORF106	RNU6-1003P
RPS19	RP11-196B3.1
EEF1A1P12	RP11-426C22.5
EEF1A1P13	RP11-123C21.3
MARC2	RP3-416J7.4
RPL37	RAG1
ARL6IP5	RAG2
RPL36	MIR646HG
RPL39	RP11-769N19.2
TNFSF10	FCRL1
IL12A	KCNJ12
GOLGA7	RP11-426C22.1

Supplementary Table 1.

RPS12	RP11-35J23.1
FAM81B	PAX5
RPS14	DNTT
RP11-561B11.1	GCSAM
SRGN	NAV2
FAM32A	RP11-87G24.3
RPL13A	RP11-87G24.2
RPS3A	METAZOA_SRP
GNG12	BMP3
KBTBD4	NME8
R3HCC1	MYO3A
ARL2BP	RP13-516M14.8
RP11-386I8.4	CCDC191
RPL37A	ARPP21
HBBP1	SLC26A6
MDFI	RAPGEF5
ZNF597	MTND5P32
CD244	HRK
PTGDR2	LINC01013
BGLT3	RP11-147L13.8
RP11-372K14.2	HILPDA
EIF4BP6	C2ORF48
RP11-152H18.3	C3ORF52
DUSP14	DTX1
FCGR3B	AEBP1
ADGRE1	RPS6KA2-IT1
MFSD5	HK2
CTD-2651B20.6	ZNF415P1
FUNDC1	RP11-16C1.2
CTSG	AC011899.9
RPS27A	RP11-87G24.6
PDRG1	C17ORF107
NPM2	RN7SL472P
FEM1B	RP11-391L3.3
VTRNA1-3	RP11-34P13.16
RPL35A	CPB2-AS1
RNASE3	SETP21
CMPK2	RP11-53B2.2
ANGPTL6	AOX3P

Supplementary Table 1.

B3GNT2	CXADR
UBA52	POLR3DP1
EEF1A1P5	LINC00472
CTD-2553L13.10	MIR23A
TPT1	GAD1
RP11-384O8.1	OR6N2
C1QA	AC130469.1
RTP4	RP11-618M23.5
EEF1A1P6	RP11-139H15.5
RPLP0	RP4-647J21.1
CTB-58E17.1	MIR24-2
RPL10A	IGLL5
RP11-217B1.2	RP13-516M14.10
CST7	DAAM2
IFIH1	CLDN9
IFNB1	RP11-631M6.2
ZNF689	RAB19
	SNRPD2P1
	RP11-216N14.7
	CLEC12B
	CTD-2179L22.1
	CEP295NL
	BEGAIN
	HMGB1P30
	FAR1-IT1
	AC068580.5
	RP4-673D20.3
	CECR2
	SMPD5
	NT5DC3
	RN7SL288P
	RPS6KA2
	VAT1L
	AL928654.7
	RP11-504I13.2
	EPHB4

SUPPLEMENTARY TABLE 2. DSigDB TABLE

Term	Overlap	P-value	Adjusted P-value	Old P-value	Old Adjusted P-value	Odds Ratio	Combined Score
3,3'-Diindolylmethane_CTD_00000841	9/105	2,2898E-05	0,092187548	0	0	5,8910162	62,94231997
suloctidil_HL60_UP	10/141	4,2074E-05	0,084694946	0	0	4,874363286	49,11447818
Fenbuconazole_CTD_00004512	5/36	0,000164121	0,220249745	0	0	9,545628102	83,18928492
Artesunate_CTD_00001840	5/37	0,000187525	0,188743686	0	0	9,287638154	79,70279177
prenylamine_HL60_UP	4/38	0,002195283	1	0	0	7,234581299	44,28608523
acetohexamide_PC3_UP	3/23	0,00435185	1	0	0	8,96458987	48,74185878
alprostadil_PC3_UP	4/53	0,007348174	1	0	0	5,18705829	25,48559148
prochlorperazine_MCF7_UP	4/58	0,010063486	1	0	0	4,739898092	21,79804059
chlorophyllin_CTD_00000324	3/31	0,010141135	1	0	0	6,651147323	30,53645065
Beryllium sulfate_CTD_00001005	3/39	0,01894004	1	0	0	5,286809411	20,97000822
LY-294002_PC3_DOWN	3/41	0,02162999	1	0	0	5,028916269	19,2792281
deferoxamine_PC3_UP	3/44	0,026035779	1	0	0	4,686035614	17,09598672
puromycin_MCF7_DOWN	5/116	0,027378506	1	0	0	2,962436308	10,65883702
2,2',4,4',5,5'-Hexachlorobiphenyl_CTD_00000731	7/209	0,033681487	1	0	0	2,301912231	7,805339959
hydralazine_CTD_00006108	18/777	0,035856216	1	0	0	1,592166541	5,2991097
hycanthone_PC3_UP	2/21	0,036935773	1	0	0	6,545573556	21,59106355
securinine_PC3_UP	3/52	0,039943221	1	0	0	3,965107058	12,7688196
2,6-DICHLORO-4-NITROPHENOL_CTD_00000815	2/22	0,040246859	1	0	0	6,248047485	20,07324784
triprolidine_PC3_UP	2/22	0,040246859	1	0	0	6,248047485	20,07324784
corbadrine_PC3_UP	2/23	0,043665437	1	0	0	5,976393247	18,71327297
propofol_MCF7_UP	3/54	0,043901983	1	0	0	3,818251241	11,93507367
protriptyline_MCF7_UP	2/24	0,047187703	1	0	0	5,727376861	17,48924374
rescinnamine_MCF7_UP	2/24	0,047187703	1	0	0	5,727376861	17,48924374
(3s)-3-Cyclopentyl-6-Methyl-7-[(4-Methylpiperazin-1-Yl)sulfonyl]-3,4-Dihy	3/57	0,05019171	1	0	0	3,617290649	10,82259146
lycorine_MCF7_DOWN	2/25	0,050809937	1	0	0	5,498281787	16,38302862
montelukast_CTD_00003205	2/25	0,050809937	1	0	0	5,498281787	16,38302862
nifuroxazide_HL60_DOWN	2/25	0,050809937	1	0	0	5,498281787	16,38302862
ampyrone_HL60_UP	10/380	0,052218424	1	0	0	1,808645325	5,339699576
celastrol_MCF7_DOWN	2/26	0,054528508	1	0	0	5,286809411	15,37949579
isoetarine_PC3_UP	2/26	0,054528508	1	0	0	5,286809411	15,37949579
3-Butylidenephthalide_CTD_00001227	3/59	0,054614772	1	0	0	3,494670627	10,16058319
dobutamine_PC3_UP	2/27	0,058339867	1	0	0	5,091001655	14,46592641
ciclopirox_HL60_DOWN	3/61	0,059217795	1	0	0	3,380091262	9,553940124
nocodazole_MCF7_UP	3/62	0,061585647	1	0	0	3,325573661	9,269459416
deferoxamine_MCF7_UP	2/29	0,066227163	1	0	0	4,739898092	12,86723347
colforsin_MCF7_UP	4/106	0,069154586	1	0	0	2,593529145	6,928382042
mebendazole_HL60_DOWN	5/151	0,07001291	1	0	0	2,275778885	6,05146816
ICN 1229_CTD_00006697	2/30	0,070296408	1	0	0	4,581901489	12,16510688
anisomycin_MCF7_DOWN	8/307	0,080797376	1	0	0	1,790971266	4,50574483
MG-262_PC3_DOWN	9/360	0,081362614	1	0	0	1,718213058	4,310720615
Cobalt sulfate_CTD_00001238	2/33	0,082968001	1	0	0	4,16536499	10,36884424
alexidine_PC3_DOWN	2/34	0,087336219	1	0	0	4,042854255	9,856438325
ritodrine_PC3_UP	2/35	0,091771664	1	0	0	3,927344134	9,380271764
benzethonium chloride_HL60_DOWN	2/36	0,096271471	1	0	0	3,818251241	8,936934929
ketoconazole_HL60_DOWN	2/38	0,105453052	1	0	0	3,617290649	8,137057087
Selenium methyl cysteine_CTD_00000103	2/38	0,105453052	1	0	0	3,617290649	8,137057087
2-Naphthoxyacetic acid_BOSS	2/38	0,105453052	1	0	0	3,617290649	8,137057087
thapsigargin_PC3_DOWN	8/329	0,108779668	1	0	0	1,67121027	3,707464395
mebendazole_MCF7_UP	2/40	0,114859393	1	0	0	3,436426117	7,436586154
aspirin_CTD_00005447	12/562	0,120423119	1	0	0	1,467512932	3,106348818
menadione_HL60_DOWN	3/84	0,123730797	1	0	0	2,454590083	5,129226974
clenbuterol_PC3_UP	2/42	0,124469957	1	0	0	3,272786778	6,819476037
IRON_CTD_00006166	5/181	0,125010906	1	0	0	1,898577965	3,94781625
NICKEL CHLORIDE_CTD_00001064	9/400	0,131039632	1	0	0	1,546391753	3,14266309
ellipticine_HL60_UP	3/87	0,133501358	1	0	0	2,369949046	4,772232792
etynodiol_HL60_DOWN	2/44	0,134265199	1	0	0	3,124023743	6,272847039
ajmaline_HL60_UP	7/294	0,138546336	1	0	0	1,636393389	3,234414095
puromycin_HL60_DOWN	6/240	0,138655017	1	0	0	1,718213058	3,394787491
chrysin_HL60_UP	2/45	0,139226228	1	0	0	3,054600993	6,022619713
fenoterol_PC3_UP	2/45	0,139226228	1	0	0	3,054600993	6,022619713
thiostrepton_PC3_UP	2/45	0,139226228	1	0	0	3,054600993	6,022619713
thapsigargin_MCF7_DOWN	4/137	0,13971036	1	0	0	2,006672185	3,949499796
harmine_MCF7_UP	3/90	0,143530018	1	0	0	2,290950745	4,447218974
Inosinic acid_BOSS	2/46	0,144226526	1	0	0	2,988196623	5,786254639
cephaeline_HL60_DOWN	9/411	0,147010105	1	0	0	1,505004139	2,885475131
5,6-Dichloro-1H-benzimidazole_CTD_00003559	1/11	0,148934309	1	0	0	6,248047485	11,89784409
IB-MECA_CTD_00003042	1/11	0,148934309	1	0	0	6,248047485	11,89784409
PRIMAQUINE_CTD_00006613	1/11	0,148934309	1	0	0	6,248047485	11,89784409
Bosentan_CTD_00003071	1/11	0,148934309	1	0	0	6,248047485	11,89784409
betulinic acid_HL60_DOWN	2/47	0,149263913	1	0	0	2,924617972	5,562738363
phenoxybenzamine_MCF7_DOWN	2/47	0,149263913	1	0	0	2,924617972	5,562738363
Bexarotene_CTD_00003225	3/92	0,150350566	1	0	0	2,241147468	4,246493959
cicloheximide_MCF7_DOWN	4/141	0,150369409	1	0	0	1,949745315	3,694105019
diltiazem_PC3_UP	2/48	0,154336259	1	0	0	2,863688431	5,351149929
dimethyloxalylglycine_PC3_UP	2/48	0,154336259	1	0	0	2,863688431	5,351149929
R-atenolol_PC3_DOWN	4/143	0,155812774	1	0	0	1,922476149	3,574075718
4-Methylhistamine_CTD_00001201	1/12	0,161324112	1	0	0	5,727376861	10,44868168
1,1-Bis(4-hydroxyphenyl)-2-phenylbut-1-ene_CTD_00002234	1/12	0,161324112	1	0	0	5,727376861	10,44868168
2-Naphthalenamine_CTD_00007026	3/97	0,167836074	1	0	0	2,125624402	3,793745411
(17S)-17-hydroxy-13,17-dimethyl-1,2,6,7,8,14,15,16-octahydrocyclopent	8/368	0,169637195	1	0	0	1,494098312	2,650669762
scopolamine_PC3_DOWN	8/368	0,169637195	1	0	0	1,494098312	2,650669762
ciclopirox_PC3_UP	3/98	0,171402475	1	0	0	2,103934357	3,710794938
amlexanox_CTD_00002035	1/13	0,173534154	1	0	0	5,286809411	9,259216728

15(R)-Prostaglandin D2_CTD_00007048	1/13	0,173534154	1	0	0	5,286809411	9,259216728
126643-37-6_CTD_00002813	1/13	0,173534154	1	0	0	5,286809411	9,259216728
MS-275_PC3_UP	9/431	0,178401246	1	0	0	1,43516636	2,473825061
piperlongumine_MCF7_DOWN	6/262	0,183752179	1	0	0	1,573935626	2,666510241
MG-132_MCF7_DOWN	5/207	0,184616136	1	0	0	1,660109235	2,804715628
HELENALIN_CTD_00000035	2/54	0,185392029	1	0	0	2,545500827	4,289888298
10'(Z),13'(E),15'(E)-Heptadecatrienylhydroquinone_CTD_00004147	1/14	0,185567037	1	0	0	4,909180167	8,268724001
bepridil_CTD_00007094	1/14	0,185567037	1	0	0	4,909180167	8,268724001
celastrol_CTD_00002186	3/103	0,18954614	1	0	0	2,001801621	3,329241919
Tanespimycin_CTD_00003582	2/56	0,195935592	1	0	0	2,454590083	4,000906447
Dimaprit_CTD_00007150	1/15	0,19742532	1	0	0	4,581901489	7,433653572
propanil_CTD_00004257	1/15	0,19742532	1	0	0	4,581901489	7,433653572
alexidine_MCF7_DOWN	3/107	0,204397713	1	0	0	1,926967916	3,059423085
metoprolol_HL60_DOWN	3/107	0,204397713	1	0	0	1,926967916	3,059423085
chrysin_HL60_DOWN	2/58	0,206551617	1	0	0	2,369949046	3,737895338
lomustine_PC3_DOWN	3/109	0,211922254	1	0	0	1,891610707	2,93490173
mometasone_PC3_UP	2/60	0,217227175	1	0	0	2,290950745	3,497850132
terfenadine_HL60_UP	2/60	0,217227175	1	0	0	2,290950745	3,497850132
curcumin II_CTD_00002181	1/17	0,220628154	1	0	0	4,042854255	6,109870857
Diethylcarbamodithioic acid_CTD_00005815	1/17	0,220628154	1	0	0	4,042854255	6,109870857
allopurinol_CTD_00005353	1/17	0,220628154	1	0	0	4,042854255	6,109870857
Phorbol 12,13-dibutyrate_CTD_00007051	1/17	0,220628154	1	0	0	4,042854255	6,109870857
leukotriene D4_CTD_00007224	1/17	0,220628154	1	0	0	4,042854255	6,109870857
Temsirolimus_CTD_00003745	1/17	0,220628154	1	0	0	4,042854255	6,109870857
cyclophosphamide_CTD_00005734	6/281	0,226727864	1	0	0	1,467512932	2,17779626
Ro 41-5253_CTD_00003040	1/18	0,231977645	1	0	0	3,818251241	5,578901374
diltiazem_CTD_00005836	1/18	0,231977645	1	0	0	3,818251241	5,578901374
N-ethylmaleimide_CTD_00005942	1/18	0,231977645	1	0	0	3,818251241	5,578901374
tyrphostin AG-825_MCF7_DOWN	9/462	0,232385291	1	0	0	1,338867318	1,953887464
dequalinium chloride_HL60_DOWN	2/63	0,233325527	1	0	0	2,181857852	3,175302872
orciprenaline_PC3_UP	2/63	0,233325527	1	0	0	2,181857852	3,175302872
Chromium(III) oxide_CTD_00001091	2/63	0,233325527	1	0	0	2,181857852	3,175302872
Glycidamide_CTD_00002776	3/115	0,23483386	1	0	0	1,792917974	2,597717606
oxygen_CTD_00006454	10/525	0,236327697	1	0	0	1,309114711	1,888444955
mycophenolate mofetil_BOSS	2/64	0,238708558	1	0	0	2,147766323	3,076700807
3-Phenyl-5H-indeno[1,2-c]pyridazin-5-one_TTD_00000814	1/19	0,24316242	1	0	0	3,617290649	5,114941807
Zinc sulfate_CTD_00007264	6/288	0,243330382	1	0	0	1,431844215	2,023675775
FCCP_CTD_00005590	2/65	0,24409776	1	0	0	2,114723764	2,982154859
ambroxol_PC3_UP	4/175	0,251180846	1	0	0	1,570937653	2,170379341
0317956-0000_MCF7_DOWN	1/20	0,254184862	1	0	0	3,436426117	4,70685042
AR-A014418_PC3_UP	1/20	0,254184862	1	0	0	3,436426117	4,70685042
TTNPB_MCF7_UP	1/20	0,254184862	1	0	0	3,436426117	4,70685042
diflorasone_PC3_UP	1/20	0,254184862	1	0	0	3,436426117	4,70685042
DIBUTYLTIN DICHLORIDE_CTD_00001008	1/20	0,254184862	1	0	0	3,436426117	4,70685042
NVP-BEZ235_CTD_00004861	1/20	0,254184862	1	0	0	3,436426117	4,70685042
pentetrazol_HL60_DOWN	2/67	0,25488946	1	0	0	2,051597682	2,804380813
luteolin_HL60_DOWN	6/293	0,255404034	1	0	0	1,407410014	1,920985946
5-azacytidine_CTD_00005455	7/353	0,255839174	1	0	0	1,362888545	1,857898196
Chromium(II) chloride_CTD_00000877	2/68	0,260289439	1	0	0	2,021427128	2,720762159
UNII-9XX54M675G_CTD_00004634	2/68	0,260289439	1	0	0	2,021427128	2,720762159
Dasatinib_MRC	1/21	0,265047319	1	0	0	3,272786778	4,345759799
GW559768X_GSK	1/21	0,265047319	1	0	0	3,272786778	4,345759799
formoterol_CTD_00000665	1/21	0,265047319	1	0	0	3,272786778	4,345759799
CP-690334-01_MCF7_DOWN	1/21	0,265047319	1	0	0	3,272786778	4,345759799
metixene_MCF7_UP	1/21	0,265047319	1	0	0	3,272786778	4,345759799
norcyclobenzaprine_MCF7_UP	1/21	0,265047319	1	0	0	3,272786778	4,345759799
securinine_MCF7_UP	1/21	0,265047319	1	0	0	3,272786778	4,345759799
TEPROTIDE_BOSS	1/21	0,265047319	1	0	0	3,272786778	4,345759799
securinine_HL60_DOWN	2/69	0,26569055	1	0	0	1,992131082	2,640416341
parthenolide_HL60_DOWN	4/181	0,270338064	1	0	0	1,518862372	1,986796551
digoxigenin_PC3_DOWN	2/70	0,271091622	1	0	0	1,963672067	2,563178063
tolnaftate_PC3_DOWN	5/240	0,271733382	1	0	0	1,431844215	1,865598376
levonorgestrel_HL60_DOWN	6/300	0,272573587	1	0	0	1,374570447	1,786730798
Dovitinib_RBC	1/22	0,275752105	1	0	0	3,124023743	4,024532923
cinchocaine_PC3_UP	1/22	0,275752105	1	0	0	3,124023743	4,024532923
24939-16-0_CTD_00001660	1/22	0,275752105	1	0	0	3,124023743	4,024532923
labetalol_HL60_UP	1/22	0,275752105	1	0	0	3,124023743	4,024532923
lasalocid_HL60_DOWN	1/22	0,275752105	1	0	0	3,124023743	4,024532923
oxyphenbutazone_MCF7_UP	1/22	0,275752105	1	0	0	3,124023743	4,024532923
4-aminobutyric acid_CTD_00006014	1/22	0,275752105	1	0	0	3,124023743	4,024532923
calyculin A_CTD_00002466	1/22	0,275752105	1	0	0	3,124023743	4,024532923
Muramyl Dipeptide_CTD_00005307	1/22	0,275752105	1	0	0	3,124023743	4,024532923
solanine_HL60_UP	3/127	0,281771426	1	0	0	1,623508402	2,056431662
bucladesine_HL60_DOWN	2/72	0,281889124	1	0	0	1,90912562	2,417414018
emetine_MCF7_DOWN	5/244	0,282883237	1	0	0	1,408371359	1,778380172
Copper(II) chloride_CTD_00001408	4/185	0,283250176	1	0	0	1,486022105	1,874505072
Dinoprostone_CTD_00007049	3/128	0,285727876	1	0	0	1,610824742	2,01790497
indomethacin	1/23	0,2863015	1	0	0	2,988196623	3,737366885
MLN-8054_Kinome Scan	1/23	0,2863015	1	0	0	2,988196623	3,737366885
XMD14-99_LINCS	1/23	0,2863015	1	0	0	2,988196623	3,737366885
BI-2536_LINCS	1/23	0,2863015	1	0	0	2,988196623	3,737366885
AY 9944_CTD_00005454	1/23	0,2863015	1	0	0	2,988196623	3,737366885
carmustine_MCF7_UP	1/23	0,2863015	1	0	0	2,988196623	3,737366885
ethaverine_PC3_UP	1/23	0,2863015	1	0	0	2,988196623	3,737366885
4,5,6,7-tetrabromobenzotriazole_CTD_00003398	1/23	0,2863015	1	0	0	2,988196623	3,737366885

IN1541_CTD_00001481	1/23	0,2863015	1	0	0	2,988196623	3,737366885
Flavopiridol hydrochloride_BOSS	1/23	0,2863015	1	0	0	2,988196623	3,737366885
lonidamine_CTD_00000839	1/23	0,2863015	1	0	0	2,988196623	3,737366885
biotin_CTD_00005513	1/23	0,2863015	1	0	0	2,988196623	3,737366885
Cladribine_BOSS	1/23	0,2863015	1	0	0	2,988196623	3,737366885
flufenamic acid_BOSS	1/23	0,2863015	1	0	0	2,988196623	3,737366885
630-60-4_TTD_00001253	1/23	0,2863015	1	0	0	2,988196623	3,737366885
GNF-PF-3832_TTD_00008267	1/23	0,2863015	1	0	0	2,988196623	3,737366885
benzene_CTD_00005481	11/616	0,286727571	1	0	0	1,227295042	1,533164881
rosiglitazone_PC3_UP	2/73	0,287283376	1	0	0	1,882973215	2,348606461
2-Bromo-3-hydroxy-4-methoxybenzaldehyde_CTD_00004641	3/129	0,289688876	1	0	0	1,598337729	1,980256972
1,5-isoquinolinediol_HL60_DOWN	2/74	0,29267323	1	0	0	1,857527631	2,282341504
perhexiline_HL60_UP	2/74	0,29267323	1	0	0	1,857527631	2,282341504
STOCK1N-35874_PC3_UP	1/24	0,296697753	1	0	0	2,863688431	3,479499787
ifosfamide_MCF7_UP	1/24	0,296697753	1	0	0	2,863688431	3,479499787
minocycline_HL60_DOWN	1/24	0,296697753	1	0	0	2,863688431	3,479499787
papaverine_HL60_UP	1/24	0,296697753	1	0	0	2,863688431	3,479499787
methylprednisolone_BOSS	2/75	0,298057674	1	0	0	1,832760596	2,218498558
Fulvestrant_CTD_00002740	6/311	0,300084382	1	0	0	1,325952199	1,596037487
emetine_HL60_DOWN	8/435	0,300638532	1	0	0	1,263972825	1,519101475
actinomycin D_CTD_00005748	4/191	0,302783842	1	0	0	1,439340782	1,719632424
GW768505A_GSK	1/25	0,306943079	1	0	0	2,749140893	3,246990952
ALW-II-49-7_LINCS	1/25	0,306943079	1	0	0	2,749140893	3,246990952
5194442_MCF7_UP	1/25	0,306943079	1	0	0	2,749140893	3,246990952
5253409_MCF7_UP	1/25	0,306943079	1	0	0	2,749140893	3,246990952
disulfiram_PC3_UP	1/25	0,306943079	1	0	0	2,749140893	3,246990952
metanephrine_PC3_UP	1/25	0,306943079	1	0	0	2,749140893	3,246990952
salbutamol_PC3_UP	1/25	0,306943079	1	0	0	2,749140893	3,246990952
Everolimus_CTD_00003479	1/25	0,306943079	1	0	0	2,749140893	3,246990952
STOCK1N-35215_PC3_UP	2/77	0,308806437	1	0	0	1,785156424	2,097631302
pyrvinium_HL60_DOWN	2/77	0,308806437	1	0	0	1,785156424	2,097631302
tetryzoline_HL60_DOWN	3/135	0,313523676	1	0	0	1,527300496	1,771485909
folic acid_CTD_00005997	4/195	0,315890867	1	0	0	1,409815843	1,624613245
ebselen_MCF7_UP	1/26	0,317039662	1	0	0	2,643404705	3,036554048
ionomycin_MCF7_DOWN	1/26	0,317039662	1	0	0	2,643404705	3,036554048
letrozole_CTD_00002692	1/26	0,317039662	1	0	0	2,643404705	3,036554048
Salbutamol sulfate_BOSS	1/26	0,317039662	1	0	0	2,643404705	3,036554048
GNF-PF-2812_TTD_00008260	1/26	0,317039662	1	0	0	2,643404705	3,036554048
apigenin_HL60_UP	4/196	0,31917573	1	0	0	1,402622905	1,601814225
morphine_CTD_00006352	2/80	0,324865434	1	0	0	1,718213058	1,931862944
withaferin A_PC3_UP	2/80	0,324865434	1	0	0	1,718213058	1,931862944
wortmannin_MCF7_UP	2/80	0,324865434	1	0	0	1,718213058	1,931862944
Bosutinib_MRC	1/27	0,326989654	1	0	0	2,545500827	2,845428911
LY-317615_Kinome Scan	1/27	0,326989654	1	0	0	2,545500827	2,845428911
alexidine_HL60_DOWN	1/27	0,326989654	1	0	0	2,545500827	2,845428911
niclosamide_MCF7_DOWN	1/27	0,326989654	1	0	0	2,545500827	2,845428911
nicotinamide_CTD_00006391	1/27	0,326989654	1	0	0	2,545500827	2,845428911
zuclopenthixol_PC3_DOWN	5/260	0,328239132	1	0	0	1,321702353	1,47239344
ursodiol_CTD_00006973	3/139	0,329445363	1	0	0	1,483349403	1,647029231
hyoscyamine_HL60_DOWN	2/81	0,33019783	1	0	0	1,697000552	1,880384062
ALW-II-38-3_LINCS	1/28	0,336795176	1	0	0	2,454590083	2,671282081
5155877_PC3_UP	1/28	0,336795176	1	0	0	2,454590083	2,671282081
bisindolylmaleimide IX_CTD_00002617	1/28	0,336795176	1	0	0	2,454590083	2,671282081
AC1L1KON_CTD_00000061	1/28	0,336795176	1	0	0	2,454590083	2,671282081
nutlin-3B_BOSS	1/28	0,336795176	1	0	0	2,454590083	2,671282081
dacarbazine_CTD_00005747	1/28	0,336795176	1	0	0	2,454590083	2,671282081
Pentadecafluorooctanoic acid_CTD_00001078	4/204	0,345530837	1	0	0	1,347618085	1,432077873
staurosporine_MCF7_DOWN	11/649	0,345939089	1	0	0	1,164890209	1,236522294
triamterene	1/29	0,346458319	1	0	0	2,369949046	2,512128831
XMD8-85_LINCS	1/29	0,346458319	1	0	0	2,369949046	2,512128831
PEPCID_CTD_00007086	1/29	0,346458319	1	0	0	2,369949046	2,512128831
Compound C (Dorsomorphin)_MRC	1/29	0,346458319	1	0	0	2,369949046	2,512128831
benfluorex_MCF7_UP	1/29	0,346458319	1	0	0	2,369949046	2,512128831
isoxsuprine_PC3_UP	1/29	0,346458319	1	0	0	2,369949046	2,512128831
mefloquine_HL60_UP	1/29	0,346458319	1	0	0	2,369949046	2,512128831
naloxone_PC3_DOWN	1/29	0,346458319	1	0	0	2,369949046	2,512128831
deltamethrin_CTD_00000882	1/29	0,346458319	1	0	0	2,369949046	2,512128831
thiotepa_CTD_00006869	1/29	0,346458319	1	0	0	2,369949046	2,512128831
IMIQUIMOD_BOSS	1/29	0,346458319	1	0	0	2,369949046	2,512128831
Disodium selenite_CTD_00007229	22/1371	0,347948823	1	0	0	1,102864691	1,164294111
ouabain_PC3_DOWN	3/144	0,349334343	1	0	0	1,431844215	1,505907521
0179445-0000_PC3_DOWN	2/85	0,351402882	1	0	0	1,617141702	1,69124221
Lead(II) acetate_CTD_00000394	2/85	0,351402882	1	0	0	1,617141702	1,69124221
SILVER_CTD_00006742	2/85	0,351402882	1	0	0	1,617141702	1,69124221
(-)-isoprenaline_HL60_DOWN	3/145	0,353306287	1	0	0	1,421969428	1,479445332
HC toxin_MCF7_UP	6/332	0,353845117	1	0	0	1,242081729	1,29039372
BI-2536_Kinome Scan	1/30	0,355981142	1	0	0	2,290950745	2,366271525
Indirubin Derivative E804_RBC	1/30	0,355981142	1	0	0	2,290950745	2,366271525
amantadine_PC3_UP	1/30	0,355981142	1	0	0	2,290950745	2,366271525
atovaquone_MCF7_UP	1/30	0,355981142	1	0	0	2,290950745	2,366271525
harmol_MCF7_UP	1/30	0,355981142	1	0	0	2,290950745	2,366271525
monorden_PC3_UP	1/30	0,355981142	1	0	0	2,290950745	2,366271525
tanespimycin_ssMCF7_UP	1/30	0,355981142	1	0	0	2,290950745	2,366271525
tonzonium bromide_PC3_UP	3/146	0,357275364	1	0	0	1,412229911	1,453535472
wortmannin_CTD_00000504	3/147	0,361241191	1	0	0	1,402622905	1,42816386

Gly-His-Lys_PC3_UP	1/31	0,365365677	1	0	0	2,217049108	2,232250463
lasalocid_HL60_UP	1/31	0,365365677	1	0	0	2,217049108	2,232250463
PI-103_CTD_00004750	1/31	0,365365677	1	0	0	2,217049108	2,232250463
vigabatrin_HL60_UP	1/31	0,365365677	1	0	0	2,217049108	2,232250463
vorinostat_HL60_UP	12/727	0,369118391	1	0	0	1,134446036	1,130631851
microcystin RR_CTD_00002594	2/89	0,372373532	1	0	0	1,544461176	1,525708036
Zinc acetate dihydrate_CTD_00007284	3/150	0,373115468	1	0	0	1,374570447	1,355144114
colforsin_PC3_UP	1/32	0,374613925	1	0	0	2,147766323	2,108804372
HMN-176_CTD_00004250	1/32	0,374613925	1	0	0	2,147766323	2,108804372
2,2',3,4,4',5'-HEXACHLOROBIPHENYL_CTD_00001404	1/32	0,374613925	1	0	0	2,147766323	2,108804372
simvastatin_PC3_UP	1/32	0,374613925	1	0	0	2,147766323	2,108804372
ranitidine_CTD_00006691	1/32	0,374613925	1	0	0	2,147766323	2,108804372
GNF-Pf-78_TTD_00008278	1/32	0,374613925	1	0	0	2,147766323	2,108804372
testosterone enanthate_CTD_00000155	9/534	0,374626377	1	0	0	1,158345882	1,137294195
gedunin_CTD_00003449	2/90	0,37757452	1	0	0	1,527300496	1,487571328
helveticoside_PC3_DOWN	2/91	0,382757603	1	0	0	1,510516974	1,450630082
digitoxin	1/33	0,383727859	1	0	0	2,082682495	1,994838443
chloroquine_CTD_00005645	1/33	0,383727859	1	0	0	2,082682495	1,994838443
Pulmicort Nebuamp_BOSS	1/33	0,383727859	1	0	0	2,082682495	1,994838443
HG-9-91-01_LINCS	3/153	0,384947386	1	0	0	1,347618085	1,286501738
tolazoline_PC3_DOWN	3/153	0,384947386	1	0	0	1,347618085	1,286501738
tamoxifen_CTD_00006827	13/803	0,386835066	1	0	0	1,112665991	1,056762161
5-Fluorouracil_CTD_00005987	19/1203	0,38952603	1	0	0	1,085487884	1,023424665
danthron_CTD_00000135	1/34	0,392709422	1	0	0	2,021427128	1,889398271
(+)-isoprenaline_PC3_UP	1/34	0,392709422	1	0	0	2,021427128	1,889398271
bisacodyl_PC3_UP	1/34	0,392709422	1	0	0	2,021427128	1,889398271
3-Aminofluoranthene_CTD_00002071	1/34	0,392709422	1	0	0	2,021427128	1,889398271
flufenamic acid_CTD_00005976	1/34	0,392709422	1	0	0	2,021427128	1,889398271
rosavin_CTD_00004837	1/34	0,392709422	1	0	0	2,021427128	1,889398271
EGTA_CTD_00005889	1/34	0,392709422	1	0	0	2,021427128	1,889398271
terbutaline_PC3_UP	1/34	0,392709422	1	0	0	2,021427128	1,889398271
Salmeterol_CTD_00002421	1/34	0,392709422	1	0	0	2,021427128	1,889398271
Inositol 1,4,5-triphosphate_BOSS	1/34	0,392709422	1	0	0	2,021427128	1,889398271
uridine triphosphate_BOSS	1/34	0,392709422	1	0	0	2,021427128	1,889398271
pyrithyldione_HL60_DOWN	3/155	0,392807188	1	0	0	1,330229465	1,243014835
KIN001-111/A770041_LINCS	2/93	0,393067759	1	0	0	1,478032738	1,380147459
dexibuprofen_PC3_DOWN	4/219	0,394974206	1	0	0	1,255315476	1,166106254
Caspan_CTD_00000180	13/809	0,397061865	1	0	0	1,104413832	1,020106392
Bortezomib	1/35	0,401560531	1	0	0	1,963672067	1,791648495
POLY I-C_CTD_00006579	1/35	0,401560531	1	0	0	1,963672067	1,791648495
Geranylgeranyl pyrophosphate_CTD_00000102	1/35	0,401560531	1	0	0	1,963672067	1,791648495
dipivefrine_HL60_UP	1/35	0,401560531	1	0	0	1,963672067	1,791648495
luteolin_PC3_UP	1/35	0,401560531	1	0	0	1,963672067	1,791648495
pyrantel_HL60_DOWN	1/35	0,401560531	1	0	0	1,963672067	1,791648495
tanespimycin_SKMEL5_UP	1/35	0,401560531	1	0	0	1,963672067	1,791648495
vanillin_CTD_00003324	1/35	0,401560531	1	0	0	1,963672067	1,791648495
beta-D-Glucose 6-phosphate_BOSS	1/35	0,401560531	1	0	0	1,963672067	1,791648495
Pentabromodiphenyl ether_CTD_00003077	7/416	0,402295272	1	0	0	1,156489559	1,053063486
methylene blue_BOSS	2/95	0,403299604	1	0	0	1,44691626	1,313909292
parthenolide_MCF7_UP	4/223	0,408086965	1	0	0	1,232798607	1,104926543
tretinoin_MCF7_UP	2/96	0,408384872	1	0	0	1,431844215	1,282281264
R547_Kinome Scan	1/36	0,410283073	1	0	0	1,90912562	1,700855165
WZ3105_LINCS	1/36	0,410283073	1	0	0	1,90912562	1,700855165
prednisone_CTD_00006606	1/36	0,410283073	1	0	0	1,90912562	1,700855165
leucovorin_CTD_00005678	1/36	0,410283073	1	0	0	1,90912562	1,700855165
Tributyltin_CTD_00000610	1/36	0,410283073	1	0	0	1,90912562	1,700855165
hyoscyamine_HL60_UP	1/36	0,410283073	1	0	0	1,90912562	1,700855165
oxolinic acid_HL60_UP	1/36	0,410283073	1	0	0	1,90912562	1,700855165
primaquine_MCF7_UP	1/36	0,410283073	1	0	0	1,90912562	1,700855165
Vandetanib_CTD_00004046	1/36	0,410283073	1	0	0	1,90912562	1,700855165
9-Anthroic acid_BOSS	1/36	0,410283073	1	0	0	1,90912562	1,700855165
EINECS 250-892-2_CTD_00001193	4/224	0,411356875	1	0	0	1,227295042	1,090198985
ellipticine_HL60_DOWN	3/161	0,41622292	1	0	0	1,280655696	1,122538639
SELENIUM_CTD_00006731	24/1560	0,418510364	1	0	0	1,057361882	0,921018899
Dasatinib_RBC	1/37	0,41887891	1	0	0	1,857527631	1,616371129
IKK Inhibitor VII_MRC	1/37	0,41887891	1	0	0	1,857527631	1,616371129
STOCK1N-35696_PC3_UP	1/37	0,41887891	1	0	0	1,857527631	1,616371129
beclometasone_HL60_UP	1/37	0,41887891	1	0	0	1,857527631	1,616371129
trolox_CTD_00000557	1/37	0,41887891	1	0	0	1,857527631	1,616371129
cloperastine_PC3_DOWN	1/37	0,41887891	1	0	0	1,857527631	1,616371129
disulfiram_HL60_UP	1/37	0,41887891	1	0	0	1,857527631	1,616371129
securinine_PC3_DOWN	1/37	0,41887891	1	0	0	1,857527631	1,616371129
semustine_MCF7_UP	1/37	0,41887891	1	0	0	1,857527631	1,616371129
edaravone_BOSS	1/37	0,41887891	1	0	0	1,857527631	1,616371129
Tenofovir_BOSS	1/37	0,41887891	1	0	0	1,857527631	1,616371129
ambroxol_PC3_DOWN	10/622	0,419301508	1	0	0	1,104960166	0,960392733
POTASSIUM DICHROMATE_CTD_00006598	9/557	0,422288839	1	0	0	1,110514724	0,957336705
alexidine_MCF7_UP	2/99	0,423512333	1	0	0	1,388454997	1,192922549
Vorinostat_CTD_00003560	7/426	0,426058866	1	0	0	1,129341916	0,963529405
Nilotinib_Kinome Scan	1/38	0,427349877	1	0	0	1,808645325	1,537623832
15-delta prostaglandin J2_MCF7_DOWN	1/38	0,427349877	1	0	0	1,808645325	1,537623832
2,2',5,5'-TETRACHLOROBIPHENYL_CTD_00000481	1/38	0,427349877	1	0	0	1,808645325	1,537623832
etilefrine_PC3_UP	1/38	0,427349877	1	0	0	1,808645325	1,537623832
verteporfin_HL60_UP	1/38	0,427349877	1	0	0	1,808645325	1,537623832
HELENALIN_TTD_00008402	1/38	0,427349877	1	0	0	1,808645325	1,537623832

strophanthidin_PC3_DOWN	3/164	0,427823651	1	0	0	1,257229067	1,067443045
okadaic acid_CTD_00007275	2/100	0,428510553	1	0	0	1,374570447	1,164865861
ACMC-20mvek_CTD_00002629	2/101	0,433485939	1	0	0	1,360960838	1,137621611
nitrofural_PC3_DOWN	4/231	0,434131583	1	0	0	1,190104283	0,993032064
Bosutinib_RBC	1/39	0,435697781	1	0	0	1,762269804	1,4641051
Nilotinib_FDA	1/39	0,435697781	1	0	0	1,762269804	1,4641051
naringin_HL60_DOWN	1/39	0,435697781	1	0	0	1,762269804	1,4641051
pyrantel_HL60_UP	1/39	0,435697781	1	0	0	1,762269804	1,4641051
Ephedrone_CTD_00002112	1/39	0,435697781	1	0	0	1,762269804	1,4641051
mitoxantrone_MCF7_DOWN	8/497	0,436366411	1	0	0	1,106294122	0,917419843
sanguinarine_HL60_DOWN	7/431	0,437907509	1	0	0	1,116240502	0,921732868
METHAMPHETAMINE_CTD_00006286	2/102	0,438438097	1	0	0	1,347618085	1,111160496
1,2,4-BENZENETRIOL_CTD_00000682	2/102	0,438438097	1	0	0	1,347618085	1,111160496
MERCURY_CTD_00006274	6/365	0,439264517	1	0	0	1,129783929	0,929420707
Clorgiline_CTD_00005690	3/168	0,443163026	1	0	0	1,227295042	0,99879427
ciclopirox_MCF7_UP	2/103	0,443366649	1	0	0	1,334534414	1,08545451
baclofen_HL60_DOWN	4/234	0,443820496	1	0	0	1,174846536	0,954369063
pentoxifylline_CTD_00006487	1/40	0,443924404	1	0	0	1,718213058	1,395362531
dexverapamil_MCF7_UP	1/40	0,443924404	1	0	0	1,718213058	1,395362531
Clofop [ISO]_CTD_00000269	1/40	0,443924404	1	0	0	1,718213058	1,395362531
1,6,7,8,9,11a,12,13,14,14a-Decahydro-1,13-dihydroxy-6-methyl-4H-cycl	1/40	0,443924404	1	0	0	1,718213058	1,395362531
trifluoperazine_MCF7_UP	1/40	0,443924404	1	0	0	1,718213058	1,395362531
Silica_BOSS	1/40	0,443924404	1	0	0	1,718213058	1,395362531
lycorine_PC3_DOWN	5/302	0,448793107	1	0	0	1,137889443	0,91166938
captopril_HL60_DOWN	3/170	0,450773028	1	0	0	1,212856277	0,966393366
MLN8054_LINCS	1/41	0,452031501	1	0	0	1,676305423	1,33099222
F0447-0125_PC3_UP	1/41	0,452031501	1	0	0	1,676305423	1,33099222
alvespimycin_HL60_UP	1/41	0,452031501	1	0	0	1,676305423	1,33099222
furazolidone_HL60_UP	1/41	0,452031501	1	0	0	1,676305423	1,33099222
mephentermine_HL60_DOWN	1/41	0,452031501	1	0	0	1,676305423	1,33099222
hesperetin_PC3_DOWN	4/237	0,453459469	1	0	0	1,159975061	0,917365565
staurosporine_CTD_00007273	3/171	0,454562238	1	0	0	1,20576355	0,950648625
flunixin_HL60_DOWN	4/238	0,456660573	1	0	0	1,155101216	0,905385535
3,3',4,4',5-Pentachlorobiphenyl_CTD_00001077	4/238	0,456660573	1	0	0	1,155101216	0,905385535
buflomedil_PC3_DOWN	2/106	0,458007118	1	0	0	1,296764572	1,01260527
Imatinib_CTD_00003267	2/106	0,458007118	1	0	0	1,296764572	1,01260527
amantadine_PC3_DOWN	1/42	0,460020804	1	0	0	1,636393389	1,270632571
monorden_HL60_UP	1/42	0,460020804	1	0	0	1,636393389	1,270632571
CHLOROGENIC ACID_CTD_00005640	1/42	0,460020804	1	0	0	1,636393389	1,270632571
rimexolone_HL60_DOWN	1/42	0,460020804	1	0	0	1,636393389	1,270632571
3'-Azido-3'-deoxythymidine_CTD_00007047	6/375	0,464864413	1	0	0	1,099656357	0,842347218
1,10-phenanthroline_CTD_00001181	2/108	0,467643156	1	0	0	1,272750414	0,967353647
calmidazolium_MCF7_DOWN	2/108	0,467643156	1	0	0	1,272750414	0,967353647
5182598_MCF7_UP	1/43	0,467894018	1	0	0	1,598337729	1,213959027
tert-Butylhydroquinone_CTD_00000961	1/43	0,467894018	1	0	0	1,598337729	1,213959027
isotretinoin_HL60_DOWN	1/43	0,467894018	1	0	0	1,598337729	1,213959027
GADOLINIUM_BOSS	1/43	0,467894018	1	0	0	1,598337729	1,213959027
zafirlukast_CTD_00002560	1/44	0,475652825	1	0	0	1,562011871	1,160679553
isoflupredone_PC3_UP	1/44	0,475652825	1	0	0	1,562011871	1,160679553
mevalolactone_HL60_UP	1/44	0,475652825	1	0	0	1,562011871	1,160679553
minocycline_HL60_UP	1/44	0,475652825	1	0	0	1,562011871	1,160679553
mycophenolic acid_MCF7_UP	1/44	0,475652825	1	0	0	1,562011871	1,160679553
Gadodiamide hydrate_CTD_00002623	1/44	0,475652825	1	0	0	1,562011871	1,160679553
oxolinic acid_HL60_DOWN	1/44	0,475652825	1	0	0	1,562011871	1,160679553
pentetrazol_HL60_UP	1/44	0,475652825	1	0	0	1,562011871	1,160679553
BARIUM_CTD_00005464	1/44	0,475652825	1	0	0	1,562011871	1,160679553
loratadine_CTD_00007174	1/44	0,475652825	1	0	0	1,562011871	1,160679553
securinine_HL60_UP	1/44	0,475652825	1	0	0	1,562011871	1,160679553
Lysergide_BOSS	1/44	0,475652825	1	0	0	1,562011871	1,160679553
POTASSIUM_CTD_00006595	1/44	0,475652825	1	0	0	1,562011871	1,160679553
sertraline_CTD_00007358	2/110	0,47717698	1	0	0	1,249609497	0,924545867
puromycin_PC3_DOWN	11/722	0,481467183	1	0	0	1,047110451	0,765351045
kojic acid_CTD_00000624	5/314	0,482498988	1	0	0	1,094403222	0,797575301
PKR Inhibitor_RBC	1/45	0,483298881	1	0	0	1,527300496	1,110530761
HG-5-88-01_LINCS	1/45	0,483298881	1	0	0	1,527300496	1,110530761
SU11652_RBC	1/45	0,483298881	1	0	0	1,527300496	1,110530761
GSK461364_LINCS	1/45	0,483298881	1	0	0	1,527300496	1,110530761
STYRENE_CTD_00001125	1/45	0,483298881	1	0	0	1,527300496	1,110530761
metoprolol_HL60_UP	1/45	0,483298881	1	0	0	1,527300496	1,110530761
semustine_PC3_UP	1/45	0,483298881	1	0	0	1,527300496	1,110530761
strophanthidin_MCF7_UP	1/45	0,483298881	1	0	0	1,527300496	1,110530761
Acetovanillone_CTD_00002374	1/45	0,483298881	1	0	0	1,527300496	1,110530761
Acridine orange_BOSS	1/45	0,483298881	1	0	0	1,527300496	1,110530761
okadaic acid_BOSS	1/45	0,483298881	1	0	0	1,527300496	1,110530761
cicloheximide_PC3_DOWN	4/247	0,485171963	1	0	0	1,113012507	0,804988398
22-Hydroxycholesterol_CTD_00000121	1/46	0,490833819	1	0	0	1,494098312	1,06327456
dorsomorphin_CTD_00004649	1/46	0,490833819	1	0	0	1,494098312	1,06327456
Bromoacetate_CTD_00000832	1/46	0,490833819	1	0	0	1,494098312	1,06327456
Org-2058_CTD_00000993	1/46	0,490833819	1	0	0	1,494098312	1,06327456
colchicine_SKMEL5_UP	1/46	0,490833819	1	0	0	1,494098312	1,06327456
geldanamycin_PC3_UP	1/46	0,490833819	1	0	0	1,494098312	1,06327456
Diquat dication_CTD_00005858	1/46	0,490833819	1	0	0	1,494098312	1,06327456
Bazedoxifene_CTD_00004022	1/46	0,490833819	1	0	0	1,494098312	1,06327456
2-deoxy-D-glucose_BOSS	1/46	0,490833819	1	0	0	1,494098312	1,06327456
montelukast_BOSS	1/46	0,490833819	1	0	0	1,494098312	1,06327456

bilirubin_BOSS	3/182	0,495479593	1	0	0	1,132887731	0,795546743
Sorafenib_CTD_00004146	2/114	0,495929594	1	0	0	1,20576355	0,845627671
pomiferin_CTD_00004186	1/47	0,498259248	1	0	0	1,462308986	1,018695268
1,4-chrysenequinone_HL60_UP	1/47	0,498259248	1	0	0	1,462308986	1,018695268
benserazide_PC3_UP	1/47	0,498259248	1	0	0	1,462308986	1,018695268
Ferriheme chloride_CTD_00006079	1/47	0,498259248	1	0	0	1,462308986	1,018695268
indometacin_PC3_UP	1/47	0,498259248	1	0	0	1,462308986	1,018695268
methylergometrine_PC3_DOWN	1/47	0,498259248	1	0	0	1,462308986	1,018695268
alpha-Neu5Ac_BOSS	1/47	0,498259248	1	0	0	1,462308986	1,018695268
Erlotinib_CTD_00003738	1/47	0,498259248	1	0	0	1,462308986	1,018695268
FURA-2_BOSS	1/47	0,498259248	1	0	0	1,462308986	1,018695268
5,6-BENZOFLAVONE_CTD_00007276	3/183	0,499124558	1	0	0	1,126697087	0,782941354
azacitidine_PC3_DOWN	9/595	0,500392726	1	0	0	1,039591094	0,719773407
cytarabine_CTD_00005743	9/595	0,500392726	1	0	0	1,039591094	0,719773407
LITHOCHOLIC ACID_CTD_00006221	2/116	0,505144702	1	0	0	1,184974523	0,809231368
Cardidigin_CTD_00005822	1/48	0,505576755	1	0	0	1,431844215	0,976597097
mitotane_CTD_00006344	1/48	0,505576755	1	0	0	1,431844215	0,976597097
primidone_PC3_UP	1/48	0,505576755	1	0	0	1,431844215	0,976597097
AMILORIDE_CTD_00005369	1/48	0,505576755	1	0	0	1,431844215	0,976597097
Crizotinib_CTD_00004714	1/48	0,505576755	1	0	0	1,431844215	0,976597097
Sildenafil citrate_BOSS	1/48	0,505576755	1	0	0	1,431844215	0,976597097
disulfiram_CTD_00005860	2/117	0,509711246	1	0	0	1,174846536	0,791741884
fendiline_MCF7_UP	2/117	0,509711246	1	0	0	1,174846536	0,791741884
mitoxantrone_PC3_DOWN	7/463	0,512503213	1	0	0	1,03909213	0,694579367
LY-333531_Kinome Scan	1/49	0,512787903	1	0	0	1,402622905	0,93680197
Staurosporine, N-benzoyl-_RBC	1/49	0,512787903	1	0	0	1,402622905	0,93680197
Go 6976_RBC	1/49	0,512787903	1	0	0	1,402622905	0,93680197
etoposide_HL60_DOWN	1/49	0,512787903	1	0	0	1,402622905	0,93680197
pioglitazone_PC3_UP	1/49	0,512787903	1	0	0	1,402622905	0,93680197
progesterone_HL60_DOWN	1/49	0,512787903	1	0	0	1,402622905	0,93680197
carboplatin_CTD_00007106	1/49	0,512787903	1	0	0	1,402622905	0,93680197
mycophenolic acid_BOSS	1/49	0,512787903	1	0	0	1,402622905	0,93680197
GDC-0941_CTD_00004868	8/534	0,516739953	1	0	0	1,029640784	0,679784828
primaquine_PC3_DOWN	8/535	0,518865911	1	0	0	1,027716222	0,674294674
Cabozantinib_FDA	1/50	0,519894232	1	0	0	1,374570447	0,899147613
lactacystin_CTD_00002698	1/50	0,519894232	1	0	0	1,374570447	0,899147613
(+)-chelidonine_HL60_DOWN	1/50	0,519894232	1	0	0	1,374570447	0,899147613
CP-863187_PC3_DOWN	1/50	0,519894232	1	0	0	1,374570447	0,899147613
STOCK1N-35696_MCF7_UP	1/50	0,519894232	1	0	0	1,374570447	0,899147613
Osajin_CTD_00004306	1/50	0,519894232	1	0	0	1,374570447	0,899147613
monorden_MCF7_UP	1/50	0,519894232	1	0	0	1,374570447	0,899147613
perphenazine_PC3_UP	1/50	0,519894232	1	0	0	1,374570447	0,899147613
Dasatinib_TTD_00007441	1/50	0,519894232	1	0	0	1,374570447	0,899147613
methacholine_BOSS	1/50	0,519894232	1	0	0	1,374570447	0,899147613
ellipticine_PC3_DOWN	6/399	0,524892684	1	0	0	1,033511614	0,666161743
Elesclomol_CTD_00004602	1/51	0,526897261	1	0	0	1,347618085	0,863485884
Leptomycin B_CTD_00001805	1/51	0,526897261	1	0	0	1,347618085	0,863485884
5155877_MCF7_UP	1/51	0,526897261	1	0	0	1,347618085	0,863485884
apigenin_MCF7_UP	1/51	0,526897261	1	0	0	1,347618085	0,863485884
mycophenolic acid_CTD_00006358	1/51	0,526897261	1	0	0	1,347618085	0,863485884
estradiol_CTD_00005920	63/4337	0,529234909	1	0	0	0,998362211	0,63528072
cyclosporin A_BOSS	3/192	0,531311936	1	0	0	1,073883162	0,679130133
staurosporine_TTD_00011086	2/122	0,53212709	1	0	0	1,126697087	0,710802691
SP600125_LINCS	1/52	0,533798486	1	0	0	1,321702353	0,829681308
(-)-isoprenaline_PC3_UP	1/52	0,533798486	1	0	0	1,321702353	0,829681308
dinoprostone_PC3_UP	1/52	0,533798486	1	0	0	1,321702353	0,829681308
isoetarine_HL60_UP	1/52	0,533798486	1	0	0	1,321702353	0,829681308
pararosaniline_MCF7_UP	1/52	0,533798486	1	0	0	1,321702353	0,829681308
budesonide_CTD_00007317	1/52	0,533798486	1	0	0	1,321702353	0,829681308
suloctidil_PC3_UP	1/52	0,533798486	1	0	0	1,321702353	0,829681308
cicloheximide_HL60_DOWN	10/684	0,539022435	1	0	0	1,004802958	0,620966305
amikacin_PC3_DOWN	5/335	0,539637626	1	0	0	1,025798841	0,632771634
LY-294002_HL60_DOWN	1/53	0,540599383	1	0	0	1,296764572	0,797609786
ellipticine_MCF7_UP	1/53	0,540599383	1	0	0	1,296764572	0,797609786
benserazide_PC3_DOWN	2/125	0,545238035	1	0	0	1,099656357	0,66697767
picrotoxinin_PC3_DOWN	3/196	0,545242537	1	0	0	1,051967179	0,638043931
sanguinarine_MCF7_UP	3/196	0,545242537	1	0	0	1,051967179	0,638043931
niclosamide_HL60_DOWN	4/267	0,546201578	1	0	0	1,029640784	0,622692955
Decitabine_CTD_00000750	26/1801	0,547068314	1	0	0	0,992194104	0,598473222
GW-5074_LINCS	1/54	0,547301405	1	0	0	1,272750414	0,767157457
acacetin_HL60_DOWN	1/54	0,547301405	1	0	0	1,272750414	0,767157457
etacrynic acid_MCF7_UP	1/54	0,547301405	1	0	0	1,272750414	0,767157457
indole_BOSS	1/54	0,547301405	1	0	0	1,272750414	0,767157457
Cyclohexanecarboxamide, 4-(1-aminoethyl)-N-4-pyridinyl-, trans-_CTD_(	1/54	0,547301405	1	0	0	1,272750414	0,767157457
metronidazole_PC3_UP	19/1316	0,548221534	1	0	0	0,992281098	0,596436168
MANGANESE CHLORIDE_CTD_00001187	3/197	0,548687575	1	0	0	1,046627244	0,628212967
clozapine_CTD_00005693	2/126	0,549551226	1	0	0	1,090928926	0,653088187
methotrexate_CTD_00006299	9/620	0,550196622	1	0	0	0,997672098	0,596088697
p-Phenylenediamine_CTD_00001400	1/55	0,553905984	1	0	0	1,249609497	0,738219695
CHEBI:18224_CTD_00001728	1/55	0,553905984	1	0	0	1,249609497	0,738219695
JAK3 Inhibitor VI_RBC	1/55	0,553905984	1	0	0	1,249609497	0,738219695
Vandetanib_Kinome Scan	1/55	0,553905984	1	0	0	1,249609497	0,738219695
Vandetanib_FDA	1/55	0,553905984	1	0	0	1,249609497	0,738219695
3,3',4,4'-Tetrachlorobiphenyl_CTD_00001342	1/55	0,553905984	1	0	0	1,249609497	0,738219695
atovaquone_HL60_DOWN	1/55	0,553905984	1	0	0	1,249609497	0,738219695

verapamil_MCF7_DOWN	1/55	0,553905984	1	0	0	1,249609497	0,738219695
azacitidine_MCF7_DOWN	7/482	0,555170091	1	0	0	0,998132067	0,587381499
Bosutinib	3/199	0,555531692	1	0	0	1,036108377	0,609055193
azathioprine_CTD_00005457	1/56	0,560414533	1	0	0	1,227295042	0,710700211
AZ-628_LINCS	1/56	0,560414533	1	0	0	1,227295042	0,710700211
fluoride_CTD_00005982	1/56	0,560414533	1	0	0	1,227295042	0,710700211
nilutamide_PC3_UP	1/56	0,560414533	1	0	0	1,227295042	0,710700211
cephaeline_MCF7_DOWN	3/201	0,562313807	1	0	0	1,025798841	0,590547479
trichostatin A_PC3_UP	6/415	0,563385623	1	0	0	0,993665383	0,570156196
acetaldehyde_CTD_00005293	2/130	0,566516062	1	0	0	1,057361882	0,600845727
acacetin_HL60_UP	1/57	0,566828442	1	0	0	1,20576355	0,68451027
terfenadine_CTD_00007125	1/57	0,566828442	1	0	0	1,20576355	0,68451027
LITHIUM_CTD_00006220	1/57	0,566828442	1	0	0	1,20576355	0,68451027
parthenolide_PC3_UP	3/203	0,569033103	1	0	0	1,015692448	0,572664333
NSC25485_CTD_00006443	2/131	0,57068495	1	0	0	1,049290417	0,588565853
TAE-684_Kinome Scan	3/204	0,572368956	1	0	0	1,010713564	0,563949331
monobenzone_PC3_DOWN	3/204	0,572368956	1	0	0	1,010713564	0,563949331
digoxin	1/58	0,573149084	1	0	0	1,184974523	0,659567975
0297417-0002B_MCF7_UP	1/58	0,573149084	1	0	0	1,184974523	0,659567975
FERROUS SULFATE_CTD_00001009	1/58	0,573149084	1	0	0	1,184974523	0,659567975
orlistat_CTD_00002339	1/58	0,573149084	1	0	0	1,184974523	0,659567975
taurine_CTD_00006829	1/58	0,573149084	1	0	0	1,184974523	0,659567975
hydroxyurea_BOSS	1/58	0,573149084	1	0	0	1,184974523	0,659567975
co-dergocrine mesilate_HL60_UP	2/132	0,574824803	1	0	0	1,041341248	0,576580209
lanatoside C_PC3_DOWN	2/133	0,57893558	1	0	0	1,033511614	0,564880313
1-Methyl-3-nitro-1-nitrosoguanidine_CTD_00006318	2/133	0,57893558	1	0	0	1,033511614	0,564880313
Tyrphostin B42_CTD_00003234	1/59	0,57937781	1	0	0	1,164890209	0,63579765
Puerarin_CTD_00001616	1/59	0,57937781	1	0	0	1,164890209	0,63579765
GW9662_CTD_00004071	1/59	0,57937781	1	0	0	1,164890209	0,63579765
Everolimus_BOSS	1/59	0,57937781	1	0	0	1,164890209	0,63579765
alpha-Tocopherol_CTD_00007387	1/59	0,57937781	1	0	0	1,164890209	0,63579765
Alvocidib_CTD_00002892	1/59	0,57937781	1	0	0	1,164890209	0,63579765
methylene blue_TTD_00009259	1/59	0,57937781	1	0	0	1,164890209	0,63579765
Caffeic acid phenethyl ester_CTD_00002352	1/60	0,585515952	1	0	0	1,145475372	0,613129268
H-89_PC3_DOWN	1/60	0,585515952	1	0	0	1,145475372	0,613129268
STOCK1N-35215_MCF7_UP	1/60	0,585515952	1	0	0	1,145475372	0,613129268
PONATINIB_CTD_00004976	1/60	0,585515952	1	0	0	1,145475372	0,613129268
zardaverine_HL60_DOWN	1/60	0,585515952	1	0	0	1,145475372	0,613129268
dihydroergotamine_HL60_UP	2/136	0,591093157	1	0	0	1,010713564	0,531414643
isotretinoin_HL60_UP	2/136	0,591093157	1	0	0	1,010713564	0,531414643
INCB18424_Kinome Scan	1/61	0,591564823	1	0	0	1,126697087	0,591497955
F0447-0125_MCF7_UP	1/61	0,591564823	1	0	0	1,126697087	0,591497955
tamoxifen_MCF7_UP	1/61	0,591564823	1	0	0	1,126697087	0,591497955
azathioprine_BOSS	1/61	0,591564823	1	0	0	1,126697087	0,591497955
ouabain_BOSS	1/61	0,591564823	1	0	0	1,126697087	0,591497955
taurine_BOSS	1/61	0,591564823	1	0	0	1,126697087	0,591497955
pyrvinium_HL60_UP	2/137	0,595087368	1	0	0	1,003336093	0,520778636
irinotecan_PC3_UP	7/501	0,596147577	1	0	0	0,960278755	0,49672054
Nickelous acetate_CTD_00003684	1/62	0,597525718	1	0	0	1,108524554	0,570843536
alvespimycin_MCF7_UP	1/62	0,597525718	1	0	0	1,108524554	0,570843536
cefotiam_PC3_UP	1/62	0,597525718	1	0	0	1,108524554	0,570843536
methylene blue_CTD_00006312	1/62	0,597525718	1	0	0	1,108524554	0,570843536
Electrocorundum_CTD_00005364	1/62	0,597525718	1	0	0	1,108524554	0,570843536
Iniprol_BOSS	1/62	0,597525718	1	0	0	1,108524554	0,570843536
Ruxolitinib_FDA	1/63	0,60339991	1	0	0	1,090928926	0,551110131
MANGANESE_CTD_00006240	3/214	0,60483543	1	0	0	0,963483958	0,48443865
Mustard gas_CTD_00006356	3/215	0,60799133	1	0	0	0,959002637	0,477194588
valproic acid_HL60_UP	4/289	0,608758817	1	0	0	0,951259825	0,472141758
Dibenziodolium_CTD_00000351	1/64	0,609188657	1	0	0	1,073883162	0,532245787
digoxin_CTD_00005825	1/64	0,609188657	1	0	0	1,073883162	0,532245787
thiostrepton_MCF7_UP	1/64	0,609188657	1	0	0	1,073883162	0,532245787
valsartan_CTD_00002971	1/64	0,609188657	1	0	0	1,073883162	0,532245787
thalidomide_CTD_00006858	2/142	0,614620935	1	0	0	0,968007357	0,471177162
arecoline_CTD_00005439	1/65	0,614893198	1	0	0	1,057361882	0,514202155
15-delta prostaglandin J2_PC3_UP	1/65	0,614893198	1	0	0	1,057361882	0,514202155
papaverine_HL60_DOWN	1/65	0,614893198	1	0	0	1,057361882	0,514202155
thioridazine_HL60_UP	1/65	0,614893198	1	0	0	1,057361882	0,514202155
Calcium phosphate_BOSS	1/65	0,614893198	1	0	0	1,057361882	0,514202155
sulfaguanidine_PC3_DOWN	8/583	0,616408938	1	0	0	0,943101507	0,456314643
glibenclamide_HL60_UP	5/366	0,618193755	1	0	0	0,93891424	0,45157395
Nilotinib	1/66	0,620514753	1	0	0	1,041341248	0,496934185
DL-Mevalonic acid_CTD_00006329	1/66	0,620514753	1	0	0	1,041341248	0,496934185
corbadrine_HL60_UP	1/66	0,620514753	1	0	0	1,041341248	0,496934185
carvedilol_CTD_00001961	1/66	0,620514753	1	0	0	1,041341248	0,496934185
VANADIUM PENTOXIDE_CTD_00002655	1/66	0,620514753	1	0	0	1,041341248	0,496934185
ellipticine_MCF7_DOWN	7/515	0,625082669	1	0	0	0,93417409	0,438941657
HG-6-64-01_LINCS	2/145	0,625991632	1	0	0	0,947979618	0,444050978
chlortetracycline_PC3_UP	1/67	0,626054526	1	0	0	1,025798841	0,480399866
ciclopirox_MCF7_DOWN	1/67	0,626054526	1	0	0	1,025798841	0,480399866
Masoprocol_CTD_00006416	1/67	0,626054526	1	0	0	1,025798841	0,480399866
FITC_BOSS	1/67	0,626054526	1	0	0	1,025798841	0,480399866
TITANIUM DIOXIDE_CTD_00000489	2/146	0,6297238	1	0	0	0,941486607	0,435413048
minoxidil_PC3_UP	5/371	0,630106344	1	0	0	0,926260409	0,427808815
5109870_MCF7_DOWN	1/68	0,631513702	1	0	0	1,010713564	0,464559976
beclomethasone_CTD_00005468	1/68	0,631513702	1	0	0	1,010713564	0,464559976

lomustine_MCF7_UP	1/68	0,631513702	1	0	0	1,010713564	0,464559976
Calcifediol_BOSS	1/68	0,631513702	1	0	0	1,010713564	0,464559976
3-methylcholanthrene_CTD_00006310	4/298	0,632782615	1	0	0	0,922530501	0,422176098
etoposide_HL60_UP	2/147	0,633427005	1	0	0	0,935081937	0,426968241
Mehp_CTD_00000849	1/69	0,63689345	1	0	0	0,996065541	0,449377864
histamine_CTD_00006100	1/69	0,63689345	1	0	0	0,996065541	0,449377864
disulfiram_MCF7_UP	1/69	0,63689345	1	0	0	0,996065541	0,449377864
glycocholic acid_PC3_DOWN	1/69	0,63689345	1	0	0	0,996065541	0,449377864
gossypol_CTD_00006052	1/69	0,63689345	1	0	0	0,996065541	0,449377864
spiperone_PC3_DOWN	1/69	0,63689345	1	0	0	0,996065541	0,449377864
trichostatin A_HL60_UP	9/667	0,637917324	1	0	0	0,927371366	0,416896635
[6-[6-(butanoylamino)purin-9-yl]-2-hydroxy-2-oxo-4a,6,7,7a-tetrahydro-4I	3/225	0,638622614	1	0	0	0,916380298	0,410943035
CHROMIUM_CTD_00005668	2/149	0,640746733	1	0	0	0,922530501	0,410637711
lomustine_PC3_UP	2/149	0,640746733	1	0	0	0,922530501	0,410637711
quercetin_HL60_UP	1/70	0,642194921	1	0	0	0,981836033	0,43481925
tonzonium bromide_PC3_DOWN	1/70	0,642194921	1	0	0	0,981836033	0,43481925
tretinoin_HL60_DOWN	1/70	0,642194921	1	0	0	0,981836033	0,43481925
brefeldin A_BOSS	1/70	0,642194921	1	0	0	0,981836033	0,43481925
digitoxigenin_MCF7_DOWN	5/377	0,644102289	1	0	0	0,911518864	0,400975081
thioguanosine_MCF7_DOWN	2/150	0,644363368	1	0	0	0,916380298	0,402742245
GW-8510_MCF7_UP	1/71	0,647419251	1	0	0	0,968007357	0,420852043
clotrimazole_CTD_00005691	1/71	0,647419251	1	0	0	0,968007357	0,420852043
nitrofural_PC3_UP	1/71	0,647419251	1	0	0	0,968007357	0,420852043
pipemidic acid_PC3_DOWN	1/71	0,647419251	1	0	0	0,968007357	0,420852043
Fludarabine_CTD_00001135	1/71	0,647419251	1	0	0	0,968007357	0,420852043
camptothecin_BOSS	1/71	0,647419251	1	0	0	0,968007357	0,420852043
HC toxin_MCF7_DOWN	2/151	0,647951268	1	0	0	0,910311554	0,395020404
rifabutin_PC3_UP	2/151	0,647951268	1	0	0	0,910311554	0,395020404
chlorzoxazone_HL60_UP	6/455	0,652501324	1	0	0	0,906310185	0,386941984
0297417-0002B_PC3_DOWN	1/72	0,652567558	1	0	0	0,95456281	0,407446171
fluoxetine_CTD_00005988	1/72	0,652567558	1	0	0	0,95456281	0,407446171
gossypol_HL60_UP	1/72	0,652567558	1	0	0	0,95456281	0,407446171
harmine_PC3_UP	1/72	0,652567558	1	0	0	0,95456281	0,407446171
levonorgestrel_HL60_UP	3/231	0,656182775	1	0	0	0,892578212	0,3760574
TBTO_CTD_00000264	1/73	0,657640944	1	0	0	0,941486607	0,394573435
PHA-00767505E_MCF7_DOWN	1/73	0,657640944	1	0	0	0,941486607	0,394573435
benzocaine_PC3_DOWN	1/73	0,657640944	1	0	0	0,941486607	0,394573435
phenoxybenzamine_PC3_UP	2/154	0,658543246	1	0	0	0,892578212	0,372852311
sanguinarine_HL60_UP	5/384	0,660009474	1	0	0	0,894902635	0,37183302
thioridazine_MCF7_UP	2/155	0,662016917	1	0	0	0,886819643	0,365781327
imidurea_PC3_DOWN	1/74	0,662640498	1	0	0	0,928763815	0,382207367
carbachol_BOSS	1/74	0,662640498	1	0	0	0,928763815	0,382207367
diethylstilbestrol_CTD_00005818	3/234	0,664730932	1	0	0	0,881134902	0,359831645
ns-398_CTD_00002958	1/75	0,667567288	1	0	0	0,916380298	0,370323105
piperlongumine_HL60_DOWN	5/389	0,671089042	1	0	0	0,88340003	0,35234715
vorinostat_MCF7_UP	8/613	0,671786963	1	0	0	0,896946458	0,356817865
N-NITROSODIETHYLAMINE_CTD_00005817	2/158	0,672268127	1	0	0	0,869981295	0,345467849
canavanine_MCF7_DOWN	1/76	0,672422371	1	0	0	0,904322662	0,358897276
tanespimycin_HL60_UP	1/76	0,672422371	1	0	0	0,904322662	0,358897276
withaferin A_MCF7_UP	1/76	0,672422371	1	0	0	0,904322662	0,358897276
Medroxyprogesterone acetate_CTD_00006623	5/390	0,673276394	1	0	0	0,881134902	0,348576389
luteolin_HL60_UP	2/159	0,675628897	1	0	0	0,864509715	0,338984048
Panobinostat_CTD_00004412	1/77	0,677206786	1	0	0	0,892578212	0,347907893
vanoxerine_PC3_UP	1/77	0,677206786	1	0	0	0,892578212	0,347907893
Pyrrolidine dithiocarbamate_CTD_00001021	2/160	0,678961675	1	0	0	0,859106529	0,332637969
8-HYDROXYQUINOLINE_CTD_00007045	3/240	0,681362756	1	0	0	0,859106529	0,329605184
mesalazine_MCF7_DOWN	1/78	0,681921559	1	0	0	0,881134902	0,337334253
primidone_PC3_DOWN	1/78	0,681921559	1	0	0	0,881134902	0,337334253
quercetin_PC3_UP	1/78	0,681921559	1	0	0	0,881134902	0,337334253
ellagic acid_TTD_00007809	1/78	0,681921559	1	0	0	0,881134902	0,337334253
digitoxigenin_PC3_DOWN	9/694	0,68386136	1	0	0	0,891292076	0,338691053
apigenin_HL60_DOWN	5/396	0,686199071	1	0	0	0,867784373	0,32679675
gefitinib_HL60_DOWN	1/79	0,686567701	1	0	0	0,869981295	0,32715685
puromycin_MCF7_UP	3/242	0,686769204	1	0	0	0,852006475	0,32014739
geldanamycin_HL60_UP	1/80	0,691146205	1	0	0	0,859106529	0,317357296
tanespimycin_PC3_UP	1/80	0,691146205	1	0	0	0,859106529	0,317357296
ANTIMONY_CTD_00005424	1/80	0,691146205	1	0	0	0,859106529	0,317357296
CARBON TETRACHLORIDE_BOSS	1/80	0,691146205	1	0	0	0,859106529	0,317357296
quinpirole_HL60_DOWN	3/245	0,694750294	1	0	0	0,841573743	0,306503503
bromocriptine_HL60_UP	1/81	0,695658055	1	0	0	0,848500276	0,307918238
Bathocuproine disulfonate_CTD_00001350	1/81	0,695658055	1	0	0	0,848500276	0,307918238
biotin_BOSS	1/81	0,695658055	1	0	0	0,848500276	0,307918238
gabexate_PC3_DOWN	2/166	0,698376326	1	0	0	0,828054486	0,297269219
cinnamaldehyde_CTD_00000671	2/166	0,698376326	1	0	0	0,828054486	0,297269219
pregnenolone_PC3_DOWN	4/325	0,698987993	1	0	0	0,845889506	0,302931399
TRICHLOROETHYLENE_CTD_00006932	1/82	0,700104217	1	0	0	0,838152711	0,298823295
Dasatinib_Kinome Scan	1/82	0,700104217	1	0	0	0,838152711	0,298823295
Dasatinib_FDA	1/82	0,700104217	1	0	0	0,838152711	0,298823295
dihydroergocristine_HL60_UP	1/82	0,700104217	1	0	0	0,838152711	0,298823295
rapamycin_CTD_00007350	3/248	0,702577524	1	0	0	0,831393415	0,293481485
Palbociclib_FDA	1/83	0,704485644	1	0	0	0,828054486	0,290056991
PD0332991_LINCS	1/83	0,704485644	1	0	0	0,828054486	0,290056991
SB 218078_RBC	1/83	0,704485644	1	0	0	0,828054486	0,290056991
Staurosporine_MRC	1/83	0,704485644	1	0	0	0,828054486	0,290056991
Apocarotenal_CTD_00000185	1/83	0,704485644	1	0	0	0,828054486	0,290056991

Vorinostat_BOSS	1/83	0,704485644	1	0	0	0,828054486	0,290056991
diphenylpyraline_BOSS	2/168	0,704628809	1	0	0	0,818196694	0,286437676
staurosporine_MCF7_UP	1/84	0,708803276	1	0	0	0,818196694	0,281604695
doxorubicin_MCF7_DOWN	13/1006	0,70903079	1	0	0	0,888141939	0,305393224
NSC94017_CTD_00005320	1/85	0,713058038	1	0	0	0,808570851	0,273452568
Rubidomycin hydrochloride	1/85	0,713058038	1	0	0	0,808570851	0,273452568
acetoacetic acid_BOSS	1/85	0,713058038	1	0	0	0,808570851	0,273452568
ouabain_MCF7_DOWN	6/486	0,713384897	1	0	0	0,848500276	0,286567543
5109870_MCF7_UP	2/171	0,713805405	1	0	0	0,803842367	0,27101135
bicalutamide_CTD_00002279	2/171	0,713805405	1	0	0	0,803842367	0,27101135
WH-4-025_LINCS	1/86	0,717250842	1	0	0	0,799168864	0,265587509
GSK1059615_LINCS	1/86	0,717250842	1	0	0	0,799168864	0,265587509
rifabutin_PC3_DOWN	1/86	0,717250842	1	0	0	0,799168864	0,265587509
Adenosine triphosphate_CTD_00005324	1/86	0,717250842	1	0	0	0,799168864	0,265587509
Edetate sodium_BOSS	1/86	0,717250842	1	0	0	0,799168864	0,265587509
Vincristine sulfate_BOSS	1/86	0,717250842	1	0	0	0,799168864	0,265587509
AZD7762_LINCS	3/254	0,717773022	1	0	0	0,811754201	0,269179224
cantharidin_HL60_DOWN	3/254	0,717773022	1	0	0	0,811754201	0,269179224
TPEN_CTD_00001994	3/254	0,717773022	1	0	0	0,811754201	0,269179224
Irinotecan hydrochloride_CTD_00002224	7/566	0,720074342	1	0	0	0,849999393	0,279140497
minoxidil_PC3_DOWN	3/255	0,720246455	1	0	0	0,808570851	0,265342088
mifepristone_CTD_00007083	4/335	0,721226761	1	0	0	0,820639073	0,268186229
chitosamine_CTD_00006030	1/87	0,721382588	1	0	0	0,789983015	0,257997115
alsterpaullone_PC3_UP	1/87	0,721382588	1	0	0	0,789983015	0,257997115
adenine_CTD_00005317	1/87	0,721382588	1	0	0	0,789983015	0,257997115
felodipine_MCF7_UP	1/87	0,721382588	1	0	0	0,789983015	0,257997115
Spectrum_001666_CTD_00000796	1/87	0,721382588	1	0	0	0,789983015	0,257997115
tanespimycin_MCF7_UP	1/87	0,721382588	1	0	0	0,789983015	0,257997115
calcitriol_BOSS	1/87	0,721382588	1	0	0	0,789983015	0,257997115
alsterpaullone_MCF7_UP	3/256	0,722703078	1	0	0	0,805412371	0,261563161
Gefitinib	1/88	0,725454161	1	0	0	0,781005936	0,250669627
GW-8510_PC3_UP	1/88	0,725454161	1	0	0	0,781005936	0,250669627
ANTIMONY POTASSIUM TARTRATE_CTD_00005425	1/88	0,725454161	1	0	0	0,781005936	0,250669627
Sunitinib	3/258	0,727566045	1	0	0	0,799168864	0,254176057
paclitaxel_CTD_00007144	6/494	0,727853963	1	0	0	0,834759381	0,265165367
midecamycin_HL60_UP	2/176	0,728569192	1	0	0	0,781005936	0,247323243
ALDOSTERONE_BOSS	2/176	0,728569192	1	0	0	0,781005936	0,247323243
paclitaxel_PC3_DOWN	5/417	0,728680137	1	0	0	0,824083002	0,260839092
IN1152_CTD_00001901	1/89	0,729466436	1	0	0	0,772230588	0,2435939
IBMX_CTD_00007018	1/89	0,729466436	1	0	0	0,772230588	0,2435939
metoclopramide_HL60_DOWN	1/89	0,729466436	1	0	0	0,772230588	0,2435939
emetine_PC3_DOWN	3/260	0,732362235	1	0	0	0,793021412	0,247010333
vincristine_CTD_00006988	16/1246	0,732922363	1	0	0	0,882549243	0,274221728
Ponatinib_FDA	1/90	0,733420273	1	0	0	0,763650248	0,236759359
azacyclonol_PC3_UP	1/90	0,733420273	1	0	0	0,763650248	0,236759359
Octa-2,4,6-trienoic acid_CTD_00002551	1/90	0,733420273	1	0	0	0,763650248	0,236759359
Dichloromercury_CTD_00006273	1/90	0,733420273	1	0	0	0,763650248	0,236759359
pergolide_HL60_DOWN	4/341	0,733977444	1	0	0	0,806199676	0,249339001
niclosamide_HL60_UP	2/178	0,734292111	1	0	0	0,772230588	0,238502149
CALCIUM_CTD_00005559	2/179	0,737114993	1	0	0	0,767916451	0,234223249
GEMCITABINE HYDROCHLORIDE_BOSS	2/179	0,737114993	1	0	0	0,767916451	0,234223249
pimozide_MCF7_UP	1/91	0,73731652	1	0	0	0,755258487	0,230155967
chrysin_CTD_00001976	1/91	0,73731652	1	0	0	0,755258487	0,230155967
lanatoside C_MCF7_DOWN	8/653	0,737654683	1	0	0	0,842003336	0,256204332
digoxin_PC3_DOWN	2/180	0,739912343	1	0	0	0,763650248	0,230029443
PhIP_CTD_00002156	2/180	0,739912343	1	0	0	0,763650248	0,230029443
histamine_BOSS	2/180	0,739912343	1	0	0	0,763650248	0,230029443
clotrimazole_PC3_UP	1/92	0,741156013	1	0	0	0,747049156	0,223774191
pyrvinium_MCF7_DOWN	1/92	0,741156013	1	0	0	0,747049156	0,223774191
9,10-Phenanthrenequinone_TTD_00001360	1/92	0,741156013	1	0	0	0,747049156	0,223774191
hemin_BOSS	2/181	0,742684304	1	0	0	0,759431186	0,225918793
Diallyl trisulfide_CTD_00001934	1/93	0,744939576	1	0	0	0,739016369	0,217604974
hexachlorophene_CTD_00006092	1/93	0,744939576	1	0	0	0,739016369	0,217604974
(-)-isoprenaline_HL60_UP	2/182	0,745431019	1	0	0	0,755258487	0,221889414
PROPIDIUM_BOSS	2/182	0,745431019	1	0	0	0,755258487	0,221889414
tolnaftate_PC3_UP	3/266	0,746353589	1	0	0	0,775133711	0,226769871
rifabutin_MCF7_UP	3/267	0,748628041	1	0	0	0,772230588	0,223570814
Deguelin_CTD_00003487	1/94	0,748668021	1	0	0	0,731154493	0,211639704
valinomycin_MCF7_UP	1/94	0,748668021	1	0	0	0,731154493	0,211639704
KW-2449_Kinome Scan	2/184	0,750849295	1	0	0	0,747049156	0,214067175
Cdk1/2 Inhibitor III_RBC	1/95	0,752342148	1	0	0	0,72345813	0,205870193
chlortetracycline_PC3_DOWN	1/95	0,752342148	1	0	0	0,72345813	0,205870193
geldanamycin_MCF7_UP	1/95	0,752342148	1	0	0	0,72345813	0,205870193
thioguanosine_MCF7_UP	1/95	0,752342148	1	0	0	0,72345813	0,205870193
valinomycin_PC3_UP	1/95	0,752342148	1	0	0	0,72345813	0,205870193
UNII-768N7QO4KH_BOSS	1/95	0,752342148	1	0	0	0,72345813	0,205870193
15-delta prostaglandin J2_HL60_UP	2/185	0,753521147	1	0	0	0,743011052	0,210270788
5224221_MCF7_UP	1/96	0,755962746	1	0	0	0,715922108	0,200288647
Gefitinib_CTD_00003879	1/96	0,755962746	1	0	0	0,715922108	0,200288647
thapsigargin_BOSS	1/96	0,755962746	1	0	0	0,715922108	0,200288647
GNF-Pf-2272_TTD_00008257	1/96	0,755962746	1	0	0	0,715922108	0,200288647
carbon monoxide_BOSS	1/96	0,755962746	1	0	0	0,715922108	0,200288647
TERT-BUTYL HYDROPEROXIDE_CTD_00007349	17/1342	0,757378602	1	0	0	0,870629568	0,241941006
Quercetin dihydrate_BOSS	2/187	0,758791022	1	0	0	0,73506441	0,202899001
15-delta prostaglandin J2_HL60_DOWN	1/97	0,759530592	1	0	0	0,708541467	0,194887646

apigenin_PC3_DOWN	1/97	0,759530592	1	0	0	0,708541467	0,194887646
phenylephrine_BOSS	1/97	0,759530592	1	0	0	0,708541467	0,194887646
beta-D-allopyranose_BOSS	1/97	0,759530592	1	0	0	0,708541467	0,194887646
hydrocortisone_CTD_00006117	1/98	0,763046452	1	0	0	0,701311452	0,189660123
lovastatin_CTD_00006225	1/98	0,763046452	1	0	0	0,701311452	0,189660123
chloroquine_BOSS	1/98	0,763046452	1	0	0	0,701311452	0,189660123
prednisolone_BOSS	1/98	0,763046452	1	0	0	0,701311452	0,189660123
camptothecin_PC3_UP	5/437	0,765152238	1	0	0	0,786367532	0,210495224
azacyclonol_HL60_UP	3/275	0,766242041	1	0	0	0,749765698	0,199630499
0297417-0002B_MCF7_DOWN	1/99	0,76651108	1	0	0	0,694227498	0,184599344
33258 Hoechst_BOSS	1/99	0,76651108	1	0	0	0,694227498	0,184599344
doxorubicin_CTD_00005874	9/751	0,76847582	1	0	0	0,823644076	0,216903521
ciglitazone_CTD_00001835	2/191	0,769039655	1	0	0	0,719670391	0,188994616
phenoxybenzamine_MCF7_UP	2/191	0,769039655	1	0	0	0,719670391	0,188994616
AGN-PC-0JHFVD_BOSS	2/191	0,769039655	1	0	0	0,719670391	0,188994616
nitric oxide_BOSS	2/191	0,769039655	1	0	0	0,719670391	0,188994616
trimethoprim_BOSS	2/191	0,769039655	1	0	0	0,719670391	0,188994616
alexidine_HL60_UP	1/100	0,769925222	1	0	0	0,687285223	0,179698889
hesperidin_PC3_DOWN	1/100	0,769925222	1	0	0	0,687285223	0,179698889
Hydroxyzine dihydrochloride_BOSS	1/100	0,769925222	1	0	0	0,687285223	0,179698889
Toxoflavin_TTD_00011503	1/100	0,769925222	1	0	0	0,687285223	0,179698889
Calcimycin_CTD_00005287	1/100	0,769925222	1	0	0	0,687285223	0,179698889
PHA-767491_LINCS	1/101	0,77328961	1	0	0	0,680480419	0,174952634
Crizotinib_Kinome Scan	1/101	0,77328961	1	0	0	0,680480419	0,174952634
cerivastatin_BOSS	2/193	0,774020842	1	0	0	0,712212667	0,182437888
Angiotensin II, human_BOSS	2/193	0,774020842	1	0	0	0,712212667	0,182437888
ethosuximide_HL60_DOWN	2/194	0,77647619	1	0	0	0,708541467	0,17925341
furosemide_BOSS	2/194	0,77647619	1	0	0	0,708541467	0,17925341
Aizen uranine_BOSS	2/194	0,77647619	1	0	0	0,708541467	0,17925341
losartan_BOSS	1/102	0,776604966	1	0	0	0,673809043	0,170354738
Phenethyl isothiocyanate_CTD_00002443	1/102	0,776604966	1	0	0	0,673809043	0,170354738
melphalan_CTD_00006262	3/280	0,776734223	1	0	0	0,736377025	0,186050841
CHIR-258/TKI-258_Kinome Scan	1/103	0,779872003	1	0	0	0,667267207	0,165899624
R-atenolol_PC3_UP	2/196	0,781317169	1	0	0	0,701311452	0,173065507
phenol_CTD_00007305	1/104	0,783091423	1	0	0	0,660851176	0,161581965
Doxorubicin Hydrochloride	1/104	0,783091423	1	0	0	0,660851176	0,161581965
phenoxybenzamine_PC3_DOWN	1/104	0,783091423	1	0	0	0,660851176	0,161581965
curcumin_CTD_00000663	6/529	0,785099864	1	0	0	0,779529554	0,188602775
0175029-0000_MCF7_UP	4/368	0,785962593	1	0	0	0,747049156	0,17992386
cyclophosphamide_BOSS	2/198	0,786066226	1	0	0	0,694227498	0,16711044
rapamycin_BOSS	2/198	0,786066226	1	0	0	0,694227498	0,16711044
raloxifene_CTD_00007367	8/687	0,786088313	1	0	0	0,800332138	0,192628849
Axitinib	1/105	0,786263917	1	0	0	0,654557356	0,157396675
Decabromodiphenyl oxide_CTD_00000574	1/105	0,786263917	1	0	0	0,654557356	0,157396675
bromocriptine_HL60_DOWN	1/105	0,786263917	1	0	0	0,654557356	0,157396675
cholecalciferol_CTD_00005655	1/105	0,786263917	1	0	0	0,654557356	0,157396675
picrotoxinin_HL60_DOWN	1/105	0,786263917	1	0	0	0,654557356	0,157396675
tyrphostin AG-1478_MCF7_UP	1/105	0,786263917	1	0	0	0,654557356	0,157396675
beta-carotene_CTD_00007253	1/105	0,786263917	1	0	0	0,654557356	0,157396675
MAGNESIUM_BOSS	1/105	0,786263917	1	0	0	0,654557356	0,157396675
Dasatinib_BOSS	1/106	0,789390167	1	0	0	0,648382286	0,153338891
doxorubicin_BOSS	2/200	0,790724612	1	0	0	0,687285223	0,161378367
chlortetracycline_HL60_UP	3/289	0,794646933	1	0	0	0,713444869	0,163990563
Chlorpyrifos oxon_CTD_00000500	1/108	0,795506613	1	0	0	0,636375207	0,14558745
OTSSP167_MRC	1/108	0,795506613	1	0	0	0,636375207	0,14558745
CHOLINE HYDROXIDE_BOSS	1/108	0,795506613	1	0	0	0,636375207	0,14558745
SODIUM BICARBONATE_BOSS	1/108	0,795506613	1	0	0	0,636375207	0,14558745
9,10-Phenanthrenequinone_CTD_00000222	1/108	0,795506613	1	0	0	0,636375207	0,14558745
co-dergocrine mesilate_HL60_DOWN	2/203	0,797544923	1	0	0	0,677128299	0,153178011
Allococaine_CTD_00005697	3/291	0,798461434	1	0	0	0,708541467	0,159470444
clonidine_HL60_DOWN	1/109	0,798498123	1	0	0	0,630536902	0,141885092
HG-14-10-04_LINCS	2/204	0,799774385	1	0	0	0,673809043	0,150546197
phenytoin_CTD_00006527	1/110	0,801446018	1	0	0	0,624804749	0,138292821
temozolomide_CTD_00002088	2/205	0,80198212	1	0	0	0,670522169	0,147963433
cicloheximide_PC3_UP	4/378	0,803048145	1	0	0	0,727285951	0,159523344
hexachlorophene	1/111	0,804350932	1	0	0	0,619175877	0,134806738
docetaxel_CTD_00002688	1/111	0,804350932	1	0	0	0,619175877	0,134806738
prenylamine_PC3_UP	1/111	0,804350932	1	0	0	0,619175877	0,134806738
etoposide_CTD_00005948	5/462	0,805393854	1	0	0	0,743815177	0,160979353
8-azaguanine_PC3_DOWN	2/207	0,806333046	1	0	0	0,664043694	0,142940992
Pioglitazone_CTD_00002515	1/112	0,80721349	1	0	0	0,613647521	0,131423109
sulforaphane_CTD_00000857	1/112	0,80721349	1	0	0	0,613647521	0,131423109
dimethyl sulfoxide_CTD_00005842	2/208	0,80847655	1	0	0	0,660851176	0,140499342
amiodarone_CTD_00005381	7/626	0,809220379	1	0	0	0,768529802	0,162685455
ionomycin_CTD_00007090	1/113	0,810034307	1	0	0	0,608217012	0,128138356
menadione_PC3_UP	1/113	0,810034307	1	0	0	0,608217012	0,128138356
Sodium dichromate_CTD_00000827	2/209	0,81059896	1	0	0	0,657689209	0,138102796
NVP-TAE684_LINCS	2/212	0,81684119	1	0	0	0,648382286	0,1311746
(-)-Epigallocatechin gallate_BOSS	2/212	0,81684119	1	0	0	0,648382286	0,1311746
puromycin_PC3_UP	6/552	0,817464404	1	0	0	0,747049156	0,150566203
Sorafenib	1/116	0,818252334	1	0	0	0,592487262	0,118843769
PD-173955_Kinome Scan	1/116	0,818252334	1	0	0	0,592487262	0,118843769
AM580_CTD_00002706	1/116	0,818252334	1	0	0	0,592487262	0,118843769
caffeine_CTD_00005556	1/116	0,818252334	1	0	0	0,592487262	0,118843769
Alitretinoin_CTD_00003402	2/213	0,818880791	1	0	0	0,645338238	0,128949396

PARAQUAT_CTD_00006471	2/214	0,820900083	1	0	0	0,642322639	0,126764864
Vandetanib	1/117	0,820912166	1	0	0	0,587423268	0,115921614
SKI-606_Kinome Scan	1/117	0,820912166	1	0	0	0,587423268	0,115921614
celecoxib_CTD_00003448	1/117	0,820912166	1	0	0	0,587423268	0,115921614
suloctidil_MCF7_UP	1/117	0,820912166	1	0	0	0,587423268	0,115921614
helveticoside_MCF7_DOWN	6/555	0,8213897	1	0	0	0,743011052	0,146193084
HG-5-113-01_LINCS	1/118	0,823533203	1	0	0	0,582445105	0,113082539
anisomycin_PC3_DOWN	6/558	0,825248267	1	0	0	0,739016369	0,141943619
1,4-chrysenequinone_PC3_UP	1/119	0,826116009	1	0	0	0,577550608	0,110323757
Nandrolone phenpropionate_BOSS	1/119	0,826116009	1	0	0	0,577550608	0,110323757
Tamibarotene_CTD_00002527	6/560	0,827783845	1	0	0	0,736377025	0,139177625
Deoxycorticosterone acetate_BOSS	1/120	0,828661141	1	0	0	0,572737686	0,107642591
mitomycin C_CTD_00007136	3/309	0,830195377	1	0	0	0,667267207	0,124174565
PP-242_Kinome Scan	1/121	0,831169145	1	0	0	0,568004317	0,105036472
luteolin_CTD_00007430	1/121	0,831169145	1	0	0	0,568004317	0,105036472
zalcitabine_PC3_DOWN	8/726	0,833186171	1	0	0	0,757339089	0,138212995
15-delta prostaglandin J2_MCF7_UP	1/122	0,833640562	1	0	0	0,563348544	0,10250293
perhexiline_MCF7_UP	1/123	0,836075923	1	0	0	0,558768474	0,100039591
deferoxamine_CTD_00005759	1/124	0,838475753	1	0	0	0,554262277	0,097644172
tolazoline_PC3_UP	2/224	0,840010319	1	0	0	0,613647521	0,106983985
PKC-412_Kinome Scan	1/125	0,840840568	1	0	0	0,549828179	0,095314481
CGP74514A_LINCS	1/125	0,840840568	1	0	0	0,549828179	0,095314481
Dronabinol_CTD_00006853	4/403	0,840964099	1	0	0	0,682168956	0,118155967
N-Acetyl-L-cysteine_CTD_00005305	3/316	0,841331852	1	0	0	0,652485972	0,112729417
methazolamide_HL60_UP	3/316	0,841331852	1	0	0	0,652485972	0,112729417
K-252a, Nocardiopsis sp._RBC	1/126	0,843170877	1	0	0	0,545464463	0,093048405
azacyclonol_MCF7_UP	1/126	0,843170877	1	0	0	0,545464463	0,093048405
Arsenenous acid_CTD_00000922	15/1284	0,843724266	1	0	0	0,802903298	0,136436986
PHENCYCLIDINE_CTD_00005881	2/227	0,845373612	1	0	0	0,605537642	0,101716158
clidinium bromide_HL60_UP	2/227	0,845373612	1	0	0	0,605537642	0,101716158
N-NITROSODIMETHYLAMINE_CTD_00005845	1/127	0,845467183	1	0	0	0,541169467	0,090843913
methyprylon_BOSS	1/127	0,845467183	1	0	0	0,541169467	0,090843913
MG-262_MCF7_DOWN	4/407	0,846427044	1	0	0	0,675464593	0,112621067
LY 294002_CTD_00003061	2/228	0,84712488	1	0	0	0,602881775	0,100022402
NICKEL SULFATE_CTD_00001417	6/577	0,848173238	1	0	0	0,714681341	0,117686844
FENRETINIDE_CTD_00007166	2/229	0,848858152	1	0	0	0,600249103	0,098358729
paclitaxel_PC3_UP	1/129	0,849959754	1	0	0	0,532779243	0,086611939
8-azaguanine_PC3_UP	3/323	0,851833201	1	0	0	0,638345409	0,102367971
colchicine_HL60_DOWN	1/130	0,852156986	1	0	0	0,528680941	0,084580763
rosiglitazone_CTD_00003139	4/412	0,853033276	1	0	0	0,667267207	0,106066607
scriptaid_PC3_UP	7/664	0,853336405	1	0	0	0,724547675	0,114914298
LITHIUM CHLORIDE_CTD_00007226	2/232	0,853951499	1	0	0	0,592487262	0,09354241
Retinoic acid_BOSS	2/232	0,853951499	1	0	0	0,592487262	0,09354241
Oxazolone_CTD_00006449	1/131	0,854322151	1	0	0	0,524645209	0,082603778
methazolamide_HL60_DOWN	1/131	0,854322151	1	0	0	0,524645209	0,082603778
bucladesine_HL60_UP	2/233	0,85561429	1	0	0	0,589944398	0,091993334
thimerosal_CTD_00006868	1/132	0,856455713	1	0	0	0,520670624	0,080679303
STOCK1N-28457_MCF7_DOWN	1/132	0,856455713	1	0	0	0,520670624	0,080679303
thiostrepton_HL60_UP	1/132	0,856455713	1	0	0	0,520670624	0,080679303
cephaeline_PC3_DOWN	2/234	0,857259832	1	0	0	0,587423268	0,090471535
vorinostat_PC3_UP	4/416	0,858144017	1	0	0	0,660851176	0,101099221
flunixin_HL60_UP	1/133	0,858558133	1	0	0	0,516755807	0,078805719
harmine_PC3_DOWN	1/133	0,858558133	1	0	0	0,516755807	0,078805719
tacrolimus_BOSS	1/133	0,858558133	1	0	0	0,516755807	0,078805719
1-NITROPYRENE_CTD_00001569	2/235	0,858888274	1	0	0	0,584923594	0,088976489
Torcetrapib_CTD_00004291	3/328	0,858959871	1	0	0	0,628614534	0,0955702
Dasatinib_CTD_00004330	5/503	0,859382043	1	0	0	0,683186107	0,103531185
irinotecan_MCF7_UP	7/671	0,860494363	1	0	0	0,716989056	0,107726325
Prestwick-983_HL60_DOWN	2/236	0,860499763	1	0	0	0,582445105	0,087507682
gemcitabine_CTD_00002382	2/236	0,860499763	1	0	0	0,582445105	0,087507682
Adenosine triphosphate_BOSS	1/134	0,860629864	1	0	0	0,51289942	0,076981463
OTSSP167_LINCS	2/237	0,862094444	1	0	0	0,57998753	0,086064611
WH-4-023_LINCS	1/135	0,862671351	1	0	0	0,509100165	0,075205031
AG-012559_PC3_DOWN	1/135	0,862671351	1	0	0	0,509100165	0,075205031
retinol_BOSS	1/135	0,862671351	1	0	0	0,509100165	0,075205031
alsterpaullone_PC3_DOWN	13/1153	0,862727147	1	0	0	0,774909619	0,114420679
dorzolamide_HL60_DOWN	2/238	0,863672465	1	0	0	0,577550608	0,084646784
midecamycin_HL60_DOWN	2/238	0,863672465	1	0	0	0,577550608	0,084646784
Crizotinib_LINCS	1/136	0,864683037	1	0	0	0,505356782	0,07347497
methylergometrine_HL60_UP	1/136	0,864683037	1	0	0	0,505356782	0,07347497
3,4-DICHLOROANILINE_CTD_00000754	1/136	0,864683037	1	0	0	0,505356782	0,07347497
cimetidine_PC3_UP	4/422	0,865526946	1	0	0	0,651455188	0,094081055
dexamethasone_CTD_00005779	4/422	0,865526946	1	0	0	0,651455188	0,094081055
proscillaridin_MCF7_DOWN	4/423	0,866725018	1	0	0	0,649915105	0,092959643
menadione_HL60_UP	3/334	0,867115362	1	0	0	0,617322057	0,088019787
medrysone_HL60_DOWN	8/760	0,867299116	1	0	0	0,72345813	0,102999718
cyclopenthiazide_PC3_UP	3/335	0,868433629	1	0	0	0,615479305	0,086822044
Crizotinib_FDA	1/138	0,868618726	1	0	0	0,498032771	0,070148414
1-chloro-2,4-dinitrobenzene_CTD_00005848	2/243	0,871317703	1	0	0	0,565666851	0,077919824
iohexol_HL60_DOWN	1/140	0,872440327	1	0	0	0,490918017	0,066991174
Uranium acetate_CTD_00000229	2/244	0,872798779	1	0	0	0,563348544	0,076643707
Bosutinib_FDA	1/141	0,874309377	1	0	0	0,487436329	0,065472929
colchicine_CTD_00005701	1/141	0,874309377	1	0	0	0,487436329	0,065472929
strophanthidin_HL60_DOWN	3/341	0,876104297	1	0	0	0,604649757	0,079977105
docetaxel_BOSS	1/142	0,876151134	1	0	0	0,484003678	0,063993357

scriptaid_MCF7_UP	7/688	0,876702055	1	0	0	0,699272756	0,092015957
URANIUM_CTD_00006964	2/247	0,877148606	1	0	0	0,556506254	0,072946202
mefloquine_MCF7_UP	1/143	0,877965994	1	0	0	0,480619037	0,062551326
ARSENIC_CTD_00005442	9/854	0,878150249	1	0	0	0,724305271	0,09411447
cefadroxil_HL60_DOWN	1/144	0,879754349	1	0	0	0,477281405	0,061145742
diuron_CTD_00005864	1/144	0,879754349	1	0	0	0,477281405	0,061145742
Decitabine_BOSS	1/144	0,879754349	1	0	0	0,477281405	0,061145742
Dasatinib	1/145	0,881516585	1	0	0	0,473989809	0,059775548
prenylamine_MCF7_UP	1/145	0,881516585	1	0	0	0,473989809	0,059775548
Cadmium sulfate_CTD_00001745	1/145	0,881516585	1	0	0	0,473989809	0,059775548
SU-14813_Kinome Scan	1/146	0,883253082	1	0	0	0,470743304	0,058439723
fulvestrant_PC3_DOWN	1/146	0,883253082	1	0	0	0,470743304	0,058439723
doxorubicin_PC3_DOWN	11/1023	0,883992097	1	0	0	0,739016369	0,091126007
lycorine_HL60_DOWN	9/862	0,884530639	1	0	0	0,71758318	0,088046111
Crizotinib	1/147	0,884964214	1	0	0	0,467540968	0,05713728
ellagic acid_CTD_00005891	1/147	0,884964214	1	0	0	0,467540968	0,05713728
daunorubicin_CTD_00005752	1/147	0,884964214	1	0	0	0,467540968	0,05713728
AST-487_Kinome Scan	1/148	0,886650351	1	0	0	0,464381908	0,055867264
EXEL-2880/GSK-1363089_Kinome Scan	1/149	0,888311857	1	0	0	0,461265251	0,054628754
mitoxantrone_CTD_00006345	2/256	0,889389737	1	0	0	0,536941581	0,062940152
tetrahydropalmatine_CTD_00000745	1/150	0,88994909	1	0	0	0,458190149	0,053420857
Cianidanol_CTD_00005600	9/871	0,891379483	1	0	0	0,710168428	0,081658742
parthenolide_HL60_UP	1/151	0,891562403	1	0	0	0,455155777	0,05224271
anisomycin_HL60_DOWN	11/1034	0,891752882	1	0	0	0,731154493	0,083765608
635-65-4_CTD_00005510	1/152	0,893152146	1	0	0	0,452161331	0,051093478
nitrate_BOSS	1/152	0,893152146	1	0	0	0,452161331	0,051093478
digoxin_MCF7_DOWN	4/449	0,894812767	1	0	0	0,612280823	0,068049369
cyclopenthiazide_PC3_DOWN	2/261	0,895693079	1	0	0	0,526655344	0,05801502
ascorbic acid_CTD_00005445	2/262	0,896913084	1	0	0	0,524645209	0,057079467
Bortezomib_CTD_00003736	9/879	0,897183226	1	0	0	0,703705007	0,076348596
meclofenoxate_HL60_DOWN	7/713	0,897687495	1	0	0	0,674754076	0,072828416
PD 98059_CTD_00003206	2/263	0,898119832	1	0	0	0,52265036	0,05615971
scopolamine_PC3_UP	4/454	0,899578488	1	0	0	0,605537642	0,064083426
Premarin_CTD_00005926	2/266	0,901661784	1	0	0	0,516755807	0,053492386
daunorubicin_MCF7_DOWN	14/1290	0,901911227	1	0	0	0,74589094	0,07700517
diltiazem_PC3_DOWN	3/364	0,90196445	1	0	0	0,566443865	0,058445775
Nintedanib_FDA	1/158	0,902214483	1	0	0	0,434990648	0,044761843
BIBF-1120 (derivative)_Kinome Scan	1/158	0,902214483	1	0	0	0,434990648	0,044761843
Vanadic sulfate_CTD_00001628	1/158	0,902214483	1	0	0	0,434990648	0,044761843
dmnq_CTD_00002569	1/158	0,902214483	1	0	0	0,434990648	0,044761843
PHOSPHINE_BOSS	1/158	0,902214483	1	0	0	0,434990648	0,044761843
raloxifene_HL60_DOWN	1/160	0,905061684	1	0	0	0,429553265	0,042848874
Sorafenib tosylate_BOSS	1/160	0,905061684	1	0	0	0,429553265	0,042848874
MG-132_MCF7_UP	2/270	0,906206321	1	0	0	0,509100165	0,050140395
parthenolide_MCF7_DOWN	1/162	0,907826258	1	0	0	0,424250138	0,041025949
CEP-701_Kinome Scan	2/272	0,908404459	1	0	0	0,505356782	0,048547382
liothyronine_CTD_00006943	1/163	0,909178309	1	0	0	0,421647376	0,040146752
Adehl_CTD_00006002	1/164	0,910510595	1	0	0	0,419076356	0,039288301
puromycin_HL60_UP	2/274	0,910554446	1	0	0	0,501668046	0,04700709
zalcitabine_PC3_UP	9/902	0,912451538	1	0	0	0,685761309	0,06282966
sanguinarine_MCF7_DOWN	3/376	0,913434566	1	0	0	0,54836587	0,049650985
GW-8510_PC3_DOWN	14/1312	0,913970826	1	0	0	0,733383622	0,065972717
niclosamide_PC3_UP	1/168	0,915647775	1	0	0	0,409098347	0,036051184
troglitazone_CTD_00002415	6/652	0,915950579	1	0	0	0,632471064	0,055526449
theophylline_CTD_00006862	4/473	0,91600054	1	0	0	0,58121372	0,050994718
methylergometrine_HL60_DOWN	1/169	0,916885468	1	0	0	0,406677647	0,035288523
tobramycin_PC3_DOWN	1/169	0,916885468	1	0	0	0,406677647	0,035288523
daunorubicin_PC3_DOWN	3/381	0,917844284	1	0	0	0,541169467	0,04639312
Coxistac_CTD_00000539	1/170	0,918105061	1	0	0	0,404285426	0,034543541
pyrvinium_MCF7_UP	1/170	0,918105061	1	0	0	0,404285426	0,034543541
carmustine_PC3_UP	1/171	0,919306819	1	0	0	0,401921183	0,03381578
dirithromycin_HL60_UP	1/171	0,919306819	1	0	0	0,401921183	0,03381578
arbutin_CTD_00005438	2/283	0,91965828	1	0	0	0,485713939	0,040680054
verteporfin_HL60_DOWN	2/283	0,91965828	1	0	0	0,485713939	0,040680054
14-Deoxy-11,12-didehydroandrographolide_CTD_00004394	1/173	0,921657863	1	0	0	0,397274696	0,032410149
astemizole_MCF7_UP	1/173	0,921657863	1	0	0	0,397274696	0,032410149
L-aspartic acid_BOSS	1/173	0,921657863	1	0	0	0,397274696	0,032410149
digoxigenin_HL60_DOWN	6/662	0,922636929	1	0	0	0,622917121	0,050156964
COBALT_CTD_00005696	1/174	0,922807657	1	0	0	0,394991508	0,031731427
meclofenoxate_HL60_UP	3/387	0,922865946	1	0	0	0,532779243	0,042766879
H-7_MCF7_DOWN	14/1332	0,923847156	1	0	0	0,722371856	0,05721809
menadione_PC3_DOWN	1/175	0,923940634	1	0	0	0,392734413	0,031068221
Phorbol 12-myristate 13-acetate_CTD_00006852	4/484	0,924376283	1	0	0	0,568004317	0,04466562
ciclopirox_HL60_UP	1/177	0,926157108	1	0	0	0,388296736	0,029786784
dimethyl sulfoxide_BOSS	1/177	0,926157108	1	0	0	0,388296736	0,029786784
dUTP_BOSS	1/179	0,928309205	1	0	0	0,383958225	0,028562808
O,P'-DDT_CTD_00000838	1/179	0,928309205	1	0	0	0,383958225	0,028562808
dihydroergotamine_HL60_DOWN	2/293	0,928751078	1	0	0	0,469136671	0,034676013
clopamide_HL60_UP	1/180	0,929361697	1	0	0	0,381825124	0,027971468
doxycycline_HL60_DOWN	1/180	0,929361697	1	0	0	0,381825124	0,027971468
4-aminobutyric acid_BOSS	1/180	0,929361697	1	0	0	0,381825124	0,027971468
L-threonine_BOSS	1/181	0,930398788	1	0	0	0,379715593	0,027393435
folic acid_BOSS	1/181	0,930398788	1	0	0	0,379715593	0,027393435
hydrogen sulfide_BOSS	1/181	0,930398788	1	0	0	0,379715593	0,027393435
digoxigenin_PC3_UP	2/296	0,931284102	1	0	0	0,464381908	0,033059762

danazol_HL60_UP	3/398	0,931351254	1	0	0	0,518054188	0,036843385
calmidazolium_MCF7_UP	1/182	0,931420705	1	0	0	0,377629244	0,026828375
1,9-Pyrazoloanthrone_CTD_00003948	1/182	0,931420705	1	0	0	0,377629244	0,026828375
methotrexate_BOSS	1/182	0,931420705	1	0	0	0,377629244	0,026828375
L-leucine_BOSS	1/182	0,931420705	1	0	0	0,377629244	0,026828375
VITAMIN E_CTD_00006994	16/1511	0,931915833	1	0	0	0,727767278	0,051316892
Velcade(Bortezomib)_BOSS	1/183	0,932427667	1	0	0	0,375565696	0,026275965
cephaeline_HL60_UP	7/767	0,93290793	1	0	0	0,627248574	0,043561639
piperlongumine_HL60_UP	1/184	0,933419894	1	0	0	0,373524578	0,025735893
terfenadine_MCF7_UP	1/184	0,933419894	1	0	0	0,373524578	0,025735893
ellagic acid_BOSS	1/184	0,933419894	1	0	0	0,373524578	0,025735893
COPPER_BOSS	1/184	0,933419894	1	0	0	0,373524578	0,025735893
Sunitinib_Kinome Scan	1/185	0,934397599	1	0	0	0,371505526	0,025207852
ZEARALENONE_CTD_00007008	1/185	0,934397599	1	0	0	0,371505526	0,025207852
IRON_BOSS	1/185	0,934397599	1	0	0	0,371505526	0,025207852
diazinon_CTD_00005790	10/1028	0,935204321	1	0	0	0,668565392	0,044787362
3-(1-methylpyrrolidin-2-yl)pyridine_CTD_00006393	4/500	0,935225726	1	0	0	0,549828179	0,036820542
zuclopenthixol_PC3_UP	2/301	0,935317979	1	0	0	0,456667923	0,030536801
lactic acid_BOSS	1/186	0,935360996	1	0	0	0,369508185	0,024691547
Sunitinib_FDA	1/187	0,936310291	1	0	0	0,367532205	0,024186688
acetic acid_BOSS	1/187	0,936310291	1	0	0	0,367532205	0,024186688
ZINC_BOSS	1/187	0,936310291	1	0	0	0,367532205	0,024186688
Imatinib mesylate_BOSS	1/187	0,936310291	1	0	0	0,367532205	0,024186688
Erythorbic acid_BOSS	1/187	0,936310291	1	0	0	0,367532205	0,024186688
17-Hydroxyandrostan-3-one_CTD_00006776	1/188	0,937245693	1	0	0	0,365577246	0,023692995
parathion_CTD_00006472	7/777	0,938118104	1	0	0	0,619175877	0,0395526
Prestwick-983_HL60_UP	1/189	0,938167402	1	0	0	0,363642975	0,023210196
metformin_BOSS	1/189	0,938167402	1	0	0	0,363642975	0,023210196
17-Ethynyl estradiol_CTD_00005932	2/305	0,9383829	1	0	0	0,450678835	0,028661914
NICKEL_CTD_00006389	1/190	0,939075618	1	0	0	0,361729065	0,022738026
L-glutamic acid_BOSS	1/190	0,939075618	1	0	0	0,361729065	0,022738026
L-glutamine_BOSS	1/190	0,939075618	1	0	0	0,361729065	0,022738026
HEXACHLOROETHANE_BOSS	1/190	0,939075618	1	0	0	0,361729065	0,022738026
cholesterol_BOSS	1/190	0,939075618	1	0	0	0,361729065	0,022738026
streptozocin_BOSS	1/191	0,93997054	1	0	0	0,359835195	0,022276226
N-Acetyl-L-cysteine_BOSS	1/191	0,93997054	1	0	0	0,359835195	0,022276226
Insulin_BOSS	1/191	0,93997054	1	0	0	0,359835195	0,022276226
GW-8510_MCF7_DOWN	13/1290	0,940713937	1	0	0	0,692613016	0,042329865
dihydroergocristine_HL60_DOWN	1/192	0,940852359	1	0	0	0,357961054	0,021824545
Isoguanine_BOSS	1/192	0,940852359	1	0	0	0,357961054	0,021824545
iopromide_HL60_UP	1/193	0,941721268	1	0	0	0,356106333	0,02138274
DICHLOROMETHANE_BOSS	1/193	0,941721268	1	0	0	0,356106333	0,02138274
Simvastatin and niacin_BOSS	1/193	0,941721268	1	0	0	0,356106333	0,02138274
17-Hydroxy-17-methylandrost-4-en-3-one_BOSS	1/194	0,942577456	1	0	0	0,354270734	0,020950573
PLATINUM_BOSS	1/194	0,942577456	1	0	0	0,354270734	0,020950573
trichostatin A_MCF7_UP	8/874	0,942752588	1	0	0	0,629094026	0,037085972
MG-262_MCF7_UP	2/312	0,943418424	1	0	0	0,440567451	0,025661018
pergolide_HL60_UP	2/312	0,943418424	1	0	0	0,440567451	0,025661018
valproic acid_HL60_DOWN	1/195	0,943421106	1	0	0	0,352453961	0,020527812
cephaeline_PC3_UP	4/514	0,943543737	1	0	0	0,534852314	0,031081637
Demecolcine_CTD_00005762	8/877	0,944084265	1	0	0	0,626942051	0,036074153
thioridazine_PC3_UP	1/196	0,944252404	1	0	0	0,350655726	0,020114234
2-Butanone_BOSS	1/196	0,944252404	1	0	0	0,350655726	0,020114234
Staurosporine, Streptomyces sp._RBC	1/197	0,945071528	1	0	0	0,348875748	0,019709618
tamoxifen_BOSS	1/197	0,945071528	1	0	0	0,348875748	0,019709618
11-cis-Retinal_BOSS	1/197	0,945071528	1	0	0	0,348875748	0,019709618
5-Fluorouracil_BOSS	1/197	0,945071528	1	0	0	0,348875748	0,019709618
Staurosporine_Kinome Scan	2/315	0,945454827	1	0	0	0,43637157	0,024475719
Acid red 87_BOSS	1/198	0,945878656	1	0	0	0,347113749	0,019313752
Bisulfite_BOSS	1/198	0,945878656	1	0	0	0,347113749	0,019313752
Andriol_CTD_00000567	1/199	0,946673964	1	0	0	0,345369459	0,018926429
MG-262_PC3_UP	2/320	0,948695013	1	0	0	0,429553265	0,022623672
Zoledronic acid_CTD_00003127	3/426	0,949229453	1	0	0	0,484003678	0,025218879
proscillaridin_HL60_DOWN	5/620	0,949518145	1	0	0	0,554262277	0,02871114
R406_Kinome Scan	1/203	0,949740406	1	0	0	0,338564149	0,017458598
Vitamin D3_BOSS	1/203	0,949740406	1	0	0	0,338564149	0,017458598
Fonofos_CTD_00005884	6/713	0,949987583	1	0	0	0,578360637	0,029673582
0179445-0000_MCF7_UP	1/204	0,95047915	1	0	0	0,336904521	0,017111062
helveticoside_PC3_UP	2/324	0,95115453	1	0	0	0,424250138	0,021245911
TERBUFOS_CTD_00000658	6/716	0,951287358	1	0	0	0,575937338	0,028761791
carmustine_CTD_00005595	3/430	0,951398325	1	0	0	0,479501319	0,023889933
cycloheximide_CTD_00005731	2/325	0,951751641	1	0	0	0,422944753	0,020915108
BX-912_LINCS	1/206	0,951924329	1	0	0	0,333633604	0,016438039
norgestrel_CTD_00006422	1/206	0,951924329	1	0	0	0,333633604	0,016438039
dexverapamil_MCF7_DOWN	2/326	0,952341797	1	0	0	0,421647376	0,02058958
Capsaicin_CTD_00005570	1/207	0,952631078	1	0	0	0,332021847	0,016112212
sulfamonomethoxine_HL60_UP	2/328	0,953501542	1	0	0	0,419076356	0,019954001
helveticoside_HL60_DOWN	4/534	0,953746942	1	0	0	0,514820392	0,024380299
deptropine_HL60_DOWN	3/435	0,953988269	1	0	0	0,473989809	0,022326771
clindamycin_HL60_UP	2/332	0,95574083	1	0	0	0,414027243	0,018742393
nocodazole_HL60_DOWN	1/212	0,956012487	1	0	0	0,324191143	0,014583513
fisetin_PC3_DOWN	4/541	0,956898467	1	0	0	0,50815913	0,022388469
trichostatin A_HL60_DOWN	4/543	0,957761938	1	0	0	0,506287457	0,021849357
piperlongumine_MCF7_UP	1/216	0,958543529	1	0	0	0,318187603	0,01347216
medrysone_HL60_UP	2/341	0,960408925	1	0	0	0,403099838	0,01628367

camptothecin_MCF7_UP	1/222	0,962070505	1	0	0	0,309587938	0,011971004
clopamide_HL60_DOWN	2/345	0,962329014	1	0	0	0,398426216	0,015299119
8-Bromo-cAMP, Na_CTD_00007044	5/653	0,963016062	1	0	0	0,526252085	0,019831909
H-7_PC3_DOWN	13/1361	0,963403909	1	0	0	0,65648111	0,024475275
pregnenolone_PC3_UP	3/456	0,963522864	1	0	0	0,452161331	0,016801891
resveratrol_BOSS	1/225	0,963720183	1	0	0	0,305460099	0,011288062
latamoxef_HL60_UP	11/1196	0,964639413	1	0	0	0,632118516	0,022756844
strophanthidin_PC3_UP	3/459	0,964723032	1	0	0	0,449206028	0,01613289
vinblastine_CTD_00006986	1/228	0,965298342	1	0	0	0,301440887	0,010646308
lanatoside C_HL60_DOWN	4/563	0,965561127	1	0	0	0,488302113	0,017112971
celastrol_MCF7_UP	1/229	0,96580904	1	0	0	0,300124552	0,010441077
SARIN_CTD_00006722	1/230	0,966312247	1	0	0	0,298819662	0,01024003
atovaquone_MCF7_DOWN	2/354	0,966328538	1	0	0	0,388296736	0,013299707
cicloheximide_MCF7_UP	2/354	0,966328538	1	0	0	0,388296736	0,013299707
vorinostat_PC3_DOWN	2/355	0,96674692	1	0	0	0,387202943	0,013094636
daunorubicin_MCF7_UP	1/232	0,967296625	1	0	0	0,296243631	0,009850125
ethanol_CTD_00005337	1/232	0,967296625	1	0	0	0,296243631	0,009850125
vorinostat_HL60_DOWN	4/569	0,967626706	1	0	0	0,483153057	0,015900036
Aflodac_CTD_00006817	1/235	0,968719696	1	0	0	0,292461797	0,00929443
tretinoin_HL60_UP	1/241	0,971383328	1	0	0	0,285180591	0,008279965
digoxigenin_HL60_UP	1/244	0,972629113	1	0	0	0,281674272	0,007817151
quinpirole_HL60_UP	1/245	0,973032243	1	0	0	0,280524581	0,007668998
digitoxigenin_PC3_UP	3/483	0,973071772	1	0	0	0,426885232	0,011652872
digoxin_PC3_UP	3/483	0,973071772	1	0	0	0,426885232	0,011652872
4-Hydroxytamoxifen_CTD_00000850	4/587	0,973153155	1	0	0	0,468337461	0,012745244
Cylindrospermopsin_CTD_00003136	3/484	0,973375483	1	0	0	0,426003238	0,011495854
ouabain_MCF7_UP	1/246	0,973429456	1	0	0	0,279384237	0,007523796
bisacodyl_MCF7_DOWN	2/373	0,973476527	1	0	0	0,368517546	0,009906329
cicloheximide_HL60_UP	3/485	0,973675954	1	0	0	0,42512488	0,01134094
harmine_MCF7_DOWN	1/247	0,973820837	1	0	0	0,278253127	0,007381482
progesterone_CTD_00006624	19/1916	0,975149739	1	0	0	0,68154589	0,017150585
topotecan_CTD_00007293	1/251	0,975329736	1	0	0	0,273818814	0,006839905
HEMATOXYLIN_BOSS	1/251	0,975329736	1	0	0	0,273818814	0,006839905
PNU-0293363_MCF7_UP	1/255	0,976751939	1	0	0	0,269523617	0,006339886
digoxigenin_MCF7_UP	1/255	0,976751939	1	0	0	0,269523617	0,006339886
emetine_PC3_UP	6/801	0,977544857	1	0	0	0,514820392	0,011692137
alprostadil_HL60_DOWN	3/500	0,977815174	1	0	0	0,412371134	0,009251386
anisomycin_PC3_UP	7/899	0,97828356	1	0	0	0,535149785	0,011749595
CUPRIC OXIDE_CTD_00001471	2/394	0,979675583	1	0	0	0,348875748	0,007163745
lycorine_PC3_UP	3/508	0,979762275	1	0	0	0,4058771	0,008298285
hydrogen peroxide_CTD_00006118	28/2674	0,979818206	1	0	0	0,719670391	0,014672805
sulpiride_PC3_DOWN	4/617	0,980449817	1	0	0	0,445565785	0,008797169
digoxin_HL60_UP	1/267	0,980546619	1	0	0	0,257410196	0,005056846
Tetradioxin_CTD_00006848	42/3769	0,980662178	1	0	0	0,765878997	0,014955506
2-Nonenal, 4-hydroxy-, (2E,4R)-_CTD_00001295	1/275	0,982726698	1	0	0	0,249921899	0,004354696
thapsigargin_MCF7_UP	2/407	0,9827844	1	0	0	0,337732296	0,005864894
alprostadil_HL60_UP	2/409	0,983219831	1	0	0	0,336080794	0,005687344
lanatoside C_PC3_UP	2/411	0,983644584	1	0	0	0,334445364	0,005515219
Retinoic acid_CTD_00006918	48/4259	0,983756575	1	0	0	0,774587714	0,012685265
PHA-00665752_MCF7_DOWN	1/280	0,983963751	1	0	0	0,245459008	0,003968145
ouabain_PC3_UP	2/416	0,984661384	1	0	0	0,330425588	0,005107543
digoxigenin_MCF7_DOWN	1/283	0,984663174	1	0	0	0,242856969	0,003753513
metronidazole_PC3_DOWN	5/745	0,985115511	1	0	0	0,461265251	0,006917307
emetine_HL60_UP	6/849	0,98581776	1	0	0	0,485713939	0,006937826
neostigmine bromide_PC3_DOWN	4/650	0,986304076	1	0	0	0,422944753	0,005832653
chlorhexidine_PC3_UP	1/293	0,98678225	1	0	0	0,234568336	0,003121139
ouabain_HL60_DOWN	3/549	0,987441416	1	0	0	0,375565696	0,004746441
piroxicam_CTD_00006571	3/550	0,987588294	1	0	0	0,374882849	0,004682052
OCHRATOXIN A_CTD_00001202	1/298	0,987729651	1	0	0	0,230632625	0,002847448
primaquine_PC3_UP	2/434	0,987838371	1	0	0	0,316721301	0,003875461
carbamazepine_CTD_00005574	2/437	0,988301577	1	0	0	0,314547013	0,003701397
testosterone_CTD_00006844	10/1248	0,988470164	1	0	0	0,550709314	0,006386477
simvastatin_CTD_00007319	1/305	0,988943362	1	0	0	0,225339417	0,002505372
scriptaid_MCF7_DOWN	4/670	0,988998106	1	0	0	0,410319536	0,004539309
Vitinoin_CTD_00007069	5/781	0,989733618	1	0	0	0,440003344	0,00454059
Bisphenol A_CTD_00000312	10/1263	0,989857216	1	0	0	0,544168823	0,005547568
digoxin_HL60_DOWN	2/452	0,990371989	1	0	0	0,304108506	0,002942146
LEAD_CTD_00006203	5/792	0,990849413	1	0	0	0,433892186	0,003988645
Silica_CTD_00006678	6/899	0,991345684	1	0	0	0,458699815	0,003987011
scriptaid_PC3_DOWN	4/692	0,991377408	1	0	0	0,397274696	0,003440392
digoxin_MCF7_UP	1/324	0,991667596	1	0	0	0,212125069	0,001774917
thiostrepton_HL60_DOWN	1/325	0,99179079	1	0	0	0,211472376	0,001743186
trichostatin A_MCF7_DOWN	3/588	0,992095398	1	0	0	0,350655726	0,002782807
digitoxigenin_MCF7_UP	1/328	0,992149583	1	0	0	0,209538178	0,001651453
indomethacin_CTD_00006147	1/336	0,993031755	1	0	0	0,204549174	0,001430338
anisomycin_MCF7_UP	4/718	0,993557774	1	0	0	0,382888704	0,002474635
strophanthidin_HL60_UP	1/344	0,993815061	1	0	0	0,199792216	0,00123954
trichostatin A_CTD_00000660	37/3585	0,993824936	1	0	0	0,70933203	0,004393751
etifenin_PC3_DOWN	3/609	0,993860058	1	0	0	0,338564149	0,002085172
digitoxigenin_HL60_DOWN	4/723	0,993911549	1	0	0	0,380240787	0,002322154
mebendazole_HL60_UP	1/346	0,993996762	1	0	0	0,198637348	0,001196061
genistein_CTD_00007324	9/1232	0,994164346	1	0	0	0,502075244	0,00293852
resveratrol_CTD_00002483	13/1602	0,994228419	1	0	0	0,557722091	0,003228263
vorinostat_MCF7_DOWN	2/492	0,994299539	1	0	0	0,279384237	0,001597175
calcitriol_CTD_00005558	17/1959	0,994424085	1	0	0	0,59641903	0,003334888

CADMIUM_CTD_00005555	8/1143	0,994532742	1	0	0	0,481039526	0,002637183
ajmaline_HL60_DOWN	2/496	0,994592536	1	0	0	0,277131138	0,001502643
helveticoside_MCF7_UP	1/355	0,994750752	1	0	0	0,193601471	0,001018939
cimetidine_PC3_DOWN	6/953	0,995004179	1	0	0	0,43270843	0,002167152
67526-95-8_CTD_00007263	6/954	0,995055483	1	0	0	0,432254857	0,002142593
captopril_PC3_DOWN	5/856	0,995377531	1	0	0	0,401451649	0,00186
POTASSIUM CHROMATE_CTD_00001284	16/1898	0,995455763	1	0	0	0,579376374	0,002638824
ZINC_CTD_00007011	13/1643	0,995904579	1	0	0	0,543804498	0,002231681
nocodazole_HL60_UP	1/373	0,995987148	1	0	0	0,184258773	0,000740891
alsterpaullone_MCF7_DOWN	11/1469	0,996244772	1	0	0	0,514645164	0,001936248
proscillaridin_MCF7_UP	1/378	0,996275767	1	0	0	0,181821488	0,00067841
0175029-0000_MCF7_DOWN	11/1488	0,996832411	1	0	0	0,508073754	0,001611923
doxycycline_HL60_UP	1/390	0,996886766	1	0	0	0,17622698	0,000549492
irinotecan_PC3_DOWN	6/999	0,996908921	1	0	0	0,412783918	0,001277924
COPPER_CTD_00005706	13/1689	0,997237956	1	0	0	0,528993955	0,001463126
lanatoside C_MCF7_UP	1/405	0,997511817	1	0	0	0,169700055	0,000422771
dirithromycin_HL60_DOWN	2/556	0,997566338	1	0	0	0,2472249	0,000602395
phenobarbital_CTD_00006510	7/1130	0,997682122	1	0	0	0,425751908	0,000987986
sulfaguanidine_PC3_UP	4/809	0,997739742	1	0	0	0,339819641	0,000768949
menadione_CTD_00007386	7/1141	0,997930591	1	0	0	0,421647376	0,000873465
digitoxigenin_HL60_UP	1/421	0,998041106	1	0	0	0,163250647	0,000320104
8-azaguanine_HL60_UP	1/428	0,998235782	1	0	0	0,16058066	0,000283549
diclofenac_CTD_00005804	1/428	0,998235782	1	0	0	0,16058066	0,000283549
lanatoside C_HL60_UP	1/432	0,998338226	1	0	0	0,159093802	0,000264598
(-)-Epigallocatechin gallate_CTD_00002033	17/2115	0,998396362	1	0	0	0,552427839	0,000886605
latamoxef_HL60_DOWN	11/1578	0,998615463	1	0	0	0,479096163	0,000663786
Enterolactone_CTD_00001393	5/972	0,998726854	1	0	0	0,353541782	0,000450397
benzo[a]pyrene_CTD_00005488	44/4425	0,999000978	1	0	0	0,683402256	0,000683075
8-azaguanine_HL60_DOWN	4/897	0,999206565	1	0	0	0,306481705	0,00024327
thapsigargin_PC3_UP	1/484	0,999236713	1	0	0	0,142001079	0,000108429
0179445-0000_MCF7_DOWN	4/911	0,999329986	1	0	0	0,301771778	0,000202259
anisomycin_HL60_UP	6/1142	0,999345933	1	0	0	0,361095564	0,000236258
chlorzoxazone_HL60_DOWN	7/1286	0,999556465	1	0	0	0,374105487	0,000165966
camptothecin_MCF7_DOWN	9/1513	0,999628892	1	0	0	0,408827958	0,000151747
chlorhexidine_PC3_DOWN	1/534	0,999638566	1	0	0	0,128705098	4,65268E-05
azacyclonol_HL60_DOWN	2/703	0,999669633	1	0	0	0,195529224	6,46071E-05
glibenclamide_HL60_DOWN	7/1333	0,999734646	1	0	0	0,360914971	9,57829E-05
lycorine_HL60_UP	3/862	0,999744645	1	0	0	0,239194393	6,10873E-05
7646-79-9_CTD_00000928	25/3095	0,999832072	1	0	0	0,555157693	9,32341E-05
formaldehyde_CTD_00006001	26/3193	0,999846103	1	0	0	0,559643464	8,61343E-05
Pirinixic acid_CTD_00000280	7/1403	0,999877247	1	0	0	0,342907809	4,20957E-05
chlortetracycline_HL60_DOWN	4/1050	0,999877542	1	0	0	0,261822942	3,20642E-05
ETHYL METHANESULFONATE_CTD_00005938	16/2316	0,999878648	1	0	0	0,474808444	5,76225E-05
clindamycin_HL60_DOWN	9/1626	0,999885558	1	0	0	0,380416175	4,35381E-05
ampyrone_HL60_DOWN	2/781	0,999886269	1	0	0	0,176001338	2,00179E-05
arsenite_CTD_00000779	6/1301	0,999891623	1	0	0	0,316964746	3,43534E-05
quercetin_CTD_00006679	25/3159	0,999900647	1	0	0	0,543910433	5,40418E-05
emetine_MCF7_UP	1/637	0,999921128	1	0	0	0,10789407	8,51012E-06
cephaeline_MCF7_UP	2/811	0,999924168	1	0	0	0,169490807	1,28533E-05
2-Methylcholine_CTD_00002006	9/1667	0,999925277	1	0	0	0,371059809	2,77277E-05
danazol_HL60_DOWN	5/1233	0,999939237	1	0	0	0,27870447	1,69353E-05
atrazine_CTD_00005450	22/2969	0,999947921	1	0	0	0,509271637	2,6523E-05
camptothecin_PC3_DOWN	7/1494	0,999954341	1	0	0	0,322021189	1,47036E-05
0175029-0000_PC3_DOWN	25/3326	0,999973445	1	0	0	0,516600438	1,37183E-05
lobeline_HL60_DOWN	6/1510	0,999987451	1	0	0	0,273093466	3,42718E-06
trichostatin A_PC3_DOWN	3/1133	0,999989495	1	0	0	0,181981966	1,91176E-06
cyclosporin A_CTD_00007121	40/4826	0,99999079	1	0	0	0,569652071	5,24666E-06
COUMESTROL_CTD_00005717	8/1813	0,99999184	1	0	0	0,303269817	2,47458E-06
AFLATOXIN B1_CTD_00007128	20/3082	0,999992218	1	0	0	0,445999496	3,47062E-06
acetaminophen_CTD_00005295	30/4136	0,999993749	1	0	0	0,498514427	3,11603E-06
Copper sulfate_CTD_00007279	51/6017	0,999993756	1	0	0	0,582541904	3,63716E-06
VALPROIC ACID_CTD_00006977	75/8313	0,999993779	1	0	0	0,620069671	3,85745E-06
METHYL METHANESULFONATE_CTD_00006307	27/3865	0,999993828	1	0	0	0,480121631	2,9635E-06
irinotecan_MCF7_DOWN	2/1272	0,999995843	1	0	0	0,108063714	4,49192E-07

**Genes**
HRK;RPS17;RPL41;RPL31;RPS6;RPLP0;RPS3;RPL13A;RPL7
LAMP5;RTP4;IFIH1;IFITM1;CXCL11;SAMD9;IFNB1;TNFSF10;IFI6;LY6E
EEF1A1;RPS19;RPS7;RPS3A;RPL27
RPL41;RPS6;TPT1;RPL7;RPS12
IFIH1;SAMD9;IFI6;TNFSF10
IFIH1;IFITM1;IFI6
CEMIP;B3GNT2;SLC16A3;TM4SF1
IFIH1;IFITM1;CEMIP;IFI6
IFITM1;BEX1;IFI6
RPL30;RPS7;RPL37A
WT1;HK2;HSPA1B
HILPDA;RPL37A;HK2
KBTBD4;RPL31;MFSD5;HILPDA;B3GNT2
CEMIP;HBG1;TNFSF10;SEPP1;MDFI;HSPA1B;HK2
RPL41;RPL31;RPL34;COMMD6;RPL10A;TOMM20;RPL7;KIAA1217;RPS27;MFSD5;RPL37A;ARL6IP5;RPL36;RPL27;RPS27A;RPL39;UBA52;RPS12
IFITM1;IFI6
C3ORF52;IL12A;HSPA1B
RPS3;RPL13A
IFITM1;IFI6
CEMIP;TM4SF1
IFIH1;IFITM1;IFI6
CEMIP;IFI6
IFITM1;IFI6
IFIH1;IFNB1;RNASE3
B3GNT2;HSPA1B
IL12A;RNASE3
HILPDA;SLC16A3
CXADR;DOK4;PTGDR2;C6ORF106;MARC2;AMDHD2;APBB2;AEBP1;CLC;HSPA1B
MFSD5;B3GNT2
CEMIP;TM4SF1
EEF1A1;RPSA;TPT1
CEMIP;TM4SF1
TMEM63A;B3GNT2;CD244
CEMIP;RPL31;NAV2
HILPDA;HK2
CEMIP;RPS6KA2;NAV2;TM4SF1
LAMP5;HILPDA;CTSG;HK2;HSPA1B
TNFSF10;IL12A
CLP1;RPL31;MFSD5;HILPDA;RPL37A;B3GNT2;DOLK;HSPA1B
EFNA1;TMEM63A;CXADR;MFSD5;HILPDA;ARL6IP5;B3GNT2;ROR1;HK2
HILPDA;HK2
HILPDA;B3GNT2
CEMIP;SLC16A3
HILPDA;SLC16A3
HILPDA;HK2
TNFSF10;SEPP1
IFIH1;TNFSF10
TMEM63A;CXADR;MFSD5;ARL6IP5;B3GNT2;DOLK;SLC16A3;HSPA1B
CEMIP;NAV2
EEF1A1;CD274;RPS19;HBG2;PTGDR2;RPL34;ARL6IP5;TNFSF10;NKG7;IL12A;CLC;RPS27A
RPS6KA2;HILPDA;HK2
CEMIP;TM4SF1
LAMP5;TNFSF10;DEFA3;IL12A;CLC
IFIH1;EFNA1;CXADR;FEM1B;HILPDA;TNFSF10;NAV2;HSPA1B;HK2
IFIH1;KBTBD4;CLP1
HILPDA;HK2
DOK4;PTGDR2;MARC2;GAD1;AMDHD2;APBB2;AEBP1
WT1;HILPDA;RPL37A;CTSG;SLC16A3;HK2
RPL31;RPS20
CEMIP;TM4SF1
C3ORF52;HSPA1B
KBTBD4;MFSD5;B3GNT2;HSPA1B
RPL31;CLP1;RPS20
IFIH1;CXCL11
TMEM63A;RPS6KA2;HILPDA;RPL37A;CTSG;RPS21;TOMM20;SLC16A3;HK2
TNFSF10
IL12A
HBG2
CXADR
HILPDA;HK2
B3GNT2;APBB2
ARL6IP5;ITM2A;TM4SF1
RPL31;RPL37A;APBB2;HSPA1B
CEMIP;RPL37A
HILPDA;HK2
BEX1;RPL31;B3GNT2;HOXA1
IL12A
TNFSF10
RPS25;POU2AF1;IFI6
RPL3;RPS8;RPL11;RPS6;RPS3;RPS20;RPSA;SEPP1
BEX1;CXADR;TMEM104;RPL31;WT1;GAD1;C3ORF52;HOXA1
HILPDA;SLC16A3;HK2
TNFSF10

PTGDR2
TRPC6
CEMIP;TMEM63A;BEX1;CXADR;GAD1;C3ORF52;HOXA1;IL12A;SEPP1
MFSD5;HILPDA;RPL37A;C3ORF52;B3GNT2;DOLK
EFNA1;MFSD5;HILPDA;ARL6IP5;B3GNT2
TNFSF10;IL12A
TNFSF10
TNFSF10
HILPDA;TNFSF10;HSPA1B
RPS6;TNFSF10
IL12A
HSPA1B
B3GNT2;DOLK;HSPA1B
MFSD5;RPL37A;HSPA1B
TNFSF10;CD244
CXADR;TMEM63A;ROR1
NAV2;HSPA1B
IFIH1;IFI6
WT1
HBG2
IFNB1
IL12A
IL12A
RPS6
RPS6KA2;HOXA1;GNG12;CST7;TM4SF1;HSPA1B
IL12A
IL12A
GAD1
EFNA1;MFSD5;FEM1B;HILPDA;RPL37A;B3GNT2;APBB2;DOLK;HSPA1B
HILPDA;HK2
CEMIP;TM4SF1
TNFSF10;TM4SF1
IFITM1;SEPP1;TM4SF1
EFNA1;CD274;WT1;HILPDA;RPS6;IL12A;NPTX1;SLC16A3;TM4SF1;HK2
IFNB1;RAPGEF5
TNFSF10
EFNA1;RPS6KA2;HILPDA;IFI6;ROR1;HSPA1B
RPL13A;HSPA1B
CEMIP;CXADR;RPL37A;HOXA1
NAV2
ROR1
HOXA1
NAV2
HSPA1B
RPS6
R3HCC1;SLC16A3
WT1;HILPDA;TNFSF10;HSPA1B;CD244;HK2
HRK;EEF1A1;CLDN9;RPS6;TNFSF10;HOXA1;RPSA
TNFSF10;TM4SF1
CD274;HSPA1B
EPHB4
EPHB4
RNASE3
SLC16A3
CEMIP
CEMIP
HSPA1B
RAPGEF5
IFIH1;HILPDA
IFIH1;WT1;HILPDA;SLC16A3
HILPDA;HK2
BEX1;RPL31;RPL37A;C3ORF52;HOXA1
LAMP5;ARL2BP;MFSD5;RPL37A;SLC16A3;HSPA1B
RPS6KA2
RPL37A
WT1
IFI6
SLC16A3
HSPA1B
GAD1
RPS6
IL12A
CXADR;RPL37A;RPL35A
LAMP5;RPS6KA2
RPL31;RPL37A;B3GNT2;TOMM20;HSPA1B
RPL32;COMMD6;SEPP1;HSPA1B
WT1;RPS6;IL12A
PTGDR2
EPHB4
RPS6KA2
RPS6KA2
IL12A
HSPA1B
CEMIP
TNFSF10

RPS6
CLP1
RPS6
CXCL11
IFNB1
TRPC6
TNFSF10
TNFSF10
FCRLA;IFITM1;MFSD5;AMDHD2;CYP4F3;IFI6;IL12A;DOLK;RPS21;SLC16A3;HSPA1B
WT1;HK2
EEF1B2;EEF1A1;HSPA1B
SLC16A3;HSPA1B
IFIH1;RPS6KA2
HSPA1B
RPL37A
HSPA1B
BEX1
IFNB1;DNTT
EFNA1;CXCL11;WT1;C6ORF106;IFI6;PAX5
RPL31;RPS6KA2;HILPDA;RPL37A;B3GNT2;CTSG;TOMM20;HK2
IFNB1;RPL34;TNFSF10;NAV2
EPHB4
EPHB4
HSPA1B
HSPA1B
HSPA1B
CEMIP
TM4SF1
RPS6
C3ORF52;HSPA1B
HILPDA;HK2
LAMP5;WT1;HSPA1B
HRK;IFITM1;RPS5;TM4SF1
HSPA1B
HSPA1B
RPS6
RNASE3
TNFSF10
KBTBD4;RPL31;CLP1;FEM1B
MIR23A;ARL6IP5
C3ORF52;HSPA1B
CEMIP;TM4SF1
EPHB4
RPS6KA2
HILPDA
HSPA1B
RPS6
CXADR;BEX1;RPL31;WT1;HOXA1
RPL32;RPL35A;RPL27
RPL36;SLC16A3
EPHB4
HSPA1B
TNFSF10
IL12A
TNFSF10
TNFSF10
MARC2;GAD1;MDFI;TPT1
KBTBD4;CLP1;MFSD5;FEM1B;CLDN9;HILPDA;RPL37A;B3GNT2;DOLK;NRBF2;HSPA1B
CTSG
RPS6KA2
IL12A
EPHB4
IFIH1
CEMIP
IFIH1
C6ORF106
UBA52
HBG2
IFNB1
RPL30;RPL32;RPS7;HBG1;RPL12;RPS5;RPLP0;DEFA3;RPL35A;RPSA;RPS3A;RPL10A;SEPP1;RPL9;RPL7;ARL2BP;RPL37A;TNFSF10;RPS3;B3GNT2;GOLGA7;UBA52
WT1;HILPDA;HK2
DOLK;HK2
TNFSF10;TM4SF1
IFNB1;TNFSF10
LAMP5;WT1;CTSG
IFITM1;CXADR;IFI6;HOXA1;NAV2;HSPA1B
RPS6KA2
RPS6KA2
IFI6
SAMD9
RPL31
HSPA1B
HSPA1B
EFNA1;C3ORF52;HSPA1B
IFNB1;RPS6;IL12A

SLC16A3
IFIH1
RPS6
TNFSF10
CXADR;ARL2BP;CLP1;RPS6KA2;R3HCC1;GDE1;B3GNT2;HOXA1;APBB2;GOLGA7;POU4F1;HSPA1B
ZNF792;HSPA1B
HRK;HILPDA;HSPA1B
CEMIP
EEF1A1
IL12A
SLC16A3
IL12A
TNFSF10
C1QA;RPL30;HBG2;HBG1;KCNJ12;TNFSF10;IFI6;HSPA1B;EPHB4
HILPDA;HSPA1B
WT1;HK2
TNFSF10
HSPA1B
RNASE3
RPS6KA2;MYO3A;EPHB4
RPL31;C3ORF52;HOXA1
HRK;IFITM1;CXADR;IFNB1;GAD1;IFI6;NAV2;TM4SF1;EEF1A1;WT1;RPS6KA2;TNFSF10;ITM2B
CD274;IFITM1;RPL3;IFNB1;IFI6;CST7;RPL10A;SEPP1;HK2;DUSP14;VPREB3;EEF1A1;ARL6IP5;TNFSF10;APBB2;RPL37;HSPA1B;MDFI;TPT1
TNFSF10
CEMIP
EFNA1
CXCL11
TRPC6
TPT1
TNFSF10
CEMIP
RNASE3
TRPC6
DNTT
LAMP5;MFSD5;TNFSF10
RPS6KA2;MYO3A
SRGN;RPL11;RPS6;SLC16A3
IFITM1;RPL31;GAD1;NAV2;SEPP1;HK2;DOK4;ARL6IP5;B3GNT2;APBB2;ITM2A;SLC16A3;HSPA1B
CTSG
IFNB1
TNFSF10
BEX1
RPL31
SLC16A3
HSPA1B
HSPA1B
HK2
CEMIP;CXADR;RPL31;FEM1B;DOLK;RPL37;HK2
HBG2;TNFSF10
EFNA1;KBTBD4;HSPA1B;DUSP14
HOXA1;NAV2
RPS6KA2
RPS6KA2
IL12A
ARL6IP5
VPREB1
RPL37A
RPL37A
RPL37A
HILPDA
CTSG
IFITM1;CLP1;TNFSF10;HSPA1B
HILPDA;SLC16A3;HK2
IFITM1;TMEM63A;RPS7;RPL31;HHIP;IFI6;NAV2;RPL10A;SEPP1;RPL7;RPS25;RPS27;RPS29;C6ORF106;RPS6KA2;RPL37A;TNFSF10;RPL27;RPS20;FAM32A;RPS21;RPL39;UBA52;LY6E
EPHB4
EPHB4
HSPA1B
RPL37A
TNFSF10
HSPA1B
HSPA1B
IFIH1
HSPA1B
DNTT
RAPGEF5
SRGN;RPS7;MFSD5;RPL11;RPS6;GDE1;RPL13A;SLC16A3;HSPA1B;LY6E
RPL41;RPS17;RPS27;RPL34;TNFSF10;RPS20;RPS27A;UBA52;TPT1
CEMIP;NAV2
EEF1A1;RPL12;HHIP;RPS6;TNFSF10;RPSA;DUSP14
EPHB4
CLDN9
IL12A
CEMIP
HSPA1B
TNFSF10

CLP1;HILPDA;HK2
TNFSF10;RPS12
TNFSF10;IL12A
SRGN;RPL11;RPS6;LY6E
EPHB4
EPHB4
SLC16A3
RPL37A
C1QA
EFNA1;MFSD5;FEM1B;HILPDA;RPL37A;DOLK;NRBF2;HSPA1B
WT1;HILPDA;RPL37A;TNFSF10;TOMM20;SLC16A3;HK2
MARC2;RAPGEF5
ARL6IP5;HSPA1B
FCRLA;RPS5;TNFSF10;SEPP1;HSPA1B;RPL19
SAMD9;HBBP1;SEPP1
HILPDA;HK2
RPS17;RPL41;RPL35;TOMM20
TNFSF10
NT5DC3
GNG12
TNFSF10
CEMIP
ARPP21
CXADR;ARL6IP5;B3GNT2;HSPA1B;EPHB4
RPL3;GDE1;SLC16A3
EPHB4
HSPA1B
HSPA1B
DOK4
LAMP5
SRGN;C6ORF106;RPL11;RPS6
WT1;RPS6KA2;TNFSF10
LAMP5;GDE1;SLC16A3;HSPA1B
C1QA;NPTX1;SEPP1;MDFI
BEX1;RPL31
WT1;RPS6
HOXA1
HSPA1B
TPT1
HILPDA
IFIH1;IFITM1;TNFSF10;IFI6;SLC16A3;LY6E
HILPDA;HK2
RPL37A;B3GNT2
HSPA1B
HBG1
LAMP5
IFNB1
RNASE3
NAV2
RPL37A
RPS6KA2
RPL31
CXCL11
SLC16A3
RPL37A
KCNJ12
RPS6
HSPA1B
DNTT
KCNJ12
HSPA1B;DUSP14
SRGN;RPS7;MFSD5;RPL11;HILPDA;RPS6;B3GNT2;RPL13A;SLC16A3;TM4SF1;LY6E
CD274;DOK4;ARL6IP5;ABCA13;MDFI
RPS6KA2
MYO3A
RPS6KA2
RPS6KA2
TNFSF10
RPL37A
HSPA1B
EFNA1
IFNB1
DNTT
DTX1
HILPDA;B3GNT2;HSPA1B;HK2
CXCL11
RPS6
TNFSF10
TM4SF1
RPL37A
HSPA1B
HSPA1B
MDFI
TNFSF10
RNASE3

HBG2;DNTT;RAPGEF5
RPS6;TNFSF10
TPT1
HSPA1B
RPL37A
ARL6IP5
GDE1
SRGN
ARL2BP
RPS6
TRPC6
DEFA3;NAV2;HSPA1B
CLP1;WT1;MFSD5;HILPDA;RPL37A;HOXA1;DOLK;HSPA1B;HOXB5
EFNA1;SRGN;CEMIP;DOK4;ARL6IP5;TNFSF10;NAV2;CCDC102A;SLC16A3
EFNA1;EPHB4
TNFSF10
IFNB1
RPL37A
TNFSF10
RPS6
TRPC6
HBG2;HSPA1B
EFNA1;IFIH1
KBTBD4;CLP1;HILPDA;RPL37A;B3GNT2;DOLK;HSPA1B
RPS6KA2
RPS6KA2
RPS6KA2
HK2
WT1
HILPDA
CD274
RAPGEF5
WT1;HILPDA;RPS6;TNFSF10;SEPP1;TM4SF1;HSPA1B;HK2
TMEM63A;BEX1;CXADR;RPL31;WT1;HILPDA;HOXA1;HSPA1B
EPHB4
TOMM20
WT1
GDE1
HSPA1B
TPT1
HSPA1B
TM4SF1
EPHB4
RNASE3
MFSD5;FEM1B;HILPDA;RPL37A;C3ORF52;HSPA1B
HSPA1B
HK2
HSPA1B
RPL31
RPS6
IFITM1;RPL32;RPL31;RPL34;RPLP0;HK2;RPL7;IFIH1;RPS14;RPS17;C6ORF106;MFSD5;RPS6KA2;ARL6IP5;RPL36;TNFSF10;HOXA1;IL12A;SLC16A3;EPHB4;SRGN;TRPC6;CXADR;RPS7;RPS6;RPL13A;RPSA;PAX5;NAV2;COMMD6;SEPP1;TM4SF1;EEF1A1;CLDN9;RPL37A;CMPK2;B3GNT2;ROR1;ZNF597;LY6E;CEMIP;SAMD9;IFI6;RPS3;RPS27A;RPL41;HBG2;HBG1;FEM1B;CYP4F3;RP
WT1;DNTT;RAPGEF5
RPS6KA2;TNFSF10
RPS6KA2
CEMIP
CEMIP
TNFSF10
NAV2
RNASE3
IFIH1
RPS14;TMEM63A;RPL31;RPS6KA2;HILPDA;RPL37A;NT5DC3;TOMM20;HSPA1B;HK2
SRGN;RPL11;RPS6;RPL13A;SLC16A3
CTSG
RPL37A
RPL11;LY6E
BEX1;NT5DC3;HOXA1
SAMD9;RPL35A;HSPA1B
WT1;HILPDA;SLC16A3;HSPA1B
HRK;ZNF792;IFITM1;HHIP;RPLP0;IFI6;AEBP1;CST7;RPS6KA2;NT5DC3;ARL6IP5;TNFSF10;NPTX1;SLC16A3;VAT1L;RAG1;SRGN;NPM2;CXADR;IFNB1;RPSA;SEPP1;EFNA1;DOK4;ZNF597;TPT1
MYO3A
CD244
HSPA1B
PTGDR2
TNFSF10
SAMD9;PTGDR2;IFNB1;RPS8;FEM1B;DEFA3;DNTT;AEBP1;POU4F1;RPS14;CXCL11;DOK4;WT1;COL5A3;AMDHD2;RPS6KA2;TNFSF10;ROR1;CLC
WT1;IL12A;SLC16A3
FCGR3B;GAD1
CXCL11;FCGR3B;GAD1;CYP4F3;CTSG;CST7;SEPP1;HSPA1B;DUSP14
CD274
CD274
RPS6KA2
EPHB4
EPHB4
RPL35A
HILPDA

HSPA1B
KBTBD4;CLP1;MFSD5;RPL37A;B3GNT2;DOLK;HSPA1B
RPS6KA2;MYO3A;EPHB4
IL12A
EPHB4
TNFSF10
RPL37A
RPL31;RPL37A;HSPA1B
CXADR;RPS6KA2;GDE1;HOXA1;TM4SF1;HSPA1B
IFNB1;HILPDA
RPL31
RPS6
HILPDA
KBTBD4;HSPA1B;DUSP14
TNFSF10;TPT1
RPS6KA2;MYO3A;EPHB4
SRGN;RPL11;RPS6
TNFSF10
IFIH1
CXCL11
TNFSF10
RPS6KA2
HBG1
BEX1;TNFSF10
HILPDA;HK2
IFITM1;RPSA
IFNB1
TPT1
IL12A
RAPGEF5
HBG2
TNFSF10
TNFSF10
RPS6KA2
GDE1
HSPA1B
EPHB4
WT1
SRGN;BEX1
HOXA1;CD244
RPS6KA2
HSPA1B
EFNA1
IFNB1
TNFSF10
GAD1
RPL37A;HSPA1B
EFNA1;CLP1;RPL31;MFSD5;HSPA1B;HOXB5;DUSP14
TPT1
HSPA1B
RPL37A
TNFSF10
TNFSF10
WT1
RPS6KA2
WT1;IL12A;SLC16A3
RPS6;TOMM20;RPS12
RPS6KA2;GDE1;POU4F1;HSPA1B
TRPC6
TNFSF10
HSPA1B
CXADR
TNFSF10;IL12A
MIR23A
HSPA1B
WT1
IFIH1
RAPGEF5
BEX1;RPL31;WT1;RPL37A;NT5DC3;C3ORF52;B3GNT2;HOXA1
DOK4;MARC2;GAD1;APBB2;ITM2A
EPHB4
TNFSF10
TNFSF10
IL12A
IFNB1
MFSD5;FEM1B;HILPDA;RPL37A;C3ORF52;B3GNT2;HSPA1B
RPS6KA2;EPHB4
RPL37A
HSPA1B
TNFSF10
DNTT
CXCL11;IL12A
IFIH1;SAMD9;CYP4F3;RPL37A;RPS3A
HSPA1B
RNASE3

HSPA1B
RAPGEF5
RPL11;IL12A;LY6E;DUSP14
IFIH1;IFI6
HSPA1B
IL12A
HSPA1B
SRGN
EPHB4
HSPA1B
CXADR;CLP1;RPS6KA2;R3HCC1;GDE1;APBB2;GOLGA7;POU4F1;HSPA1B
EEF1A1;HOXA1;RPSA
HHIP;TNFSF10
C3ORF52;HSPA1B
RPS20
WT1
WT1
SLC6A19
IFIH1;FEM1B;B3GNT2;DOLK;HSPA1B
B3GNT2;HSPA1B
C6ORF106
HK2
RPL37A
RPS6
TNFSF10
TNFSF10
KBTBD4;HK2
CXADR;HOXA1
CXADR;DOK4;PTGDR2;MARC2;GAD1;CLC
HSPA1B
HBG2
RPS6KA2
RPL31
RPL37A;RPS3A;TM4SF1
VPREB1
GDE1
HOXA1
RPL31;HSPA1B
CXADR;IFNB1;RPL37A;RPL35A;HSPA1B
CEMIP;IFI6
SAMD9
TRPC6
RPS19;DOK4;RPS3A
TNFSF10
WT1;HILPDA;RPL37A;SLC16A3;HK2
CXADR;DOK4;CLP1;RPS6KA2;HOXA1;NAV2;SEPP1;HSPA1B
IFI6;HSPA1B
GDE1
HSPA1B
HSPA1B
EEF1A1;C1QA;HOXA1;RPSA;SEPP1
RPL31;RPS20
TNFSF10
IFIH1
SRGN;NKG7
HILPDA;TNFSF10;APBB2
HSPA1B
LY6E
SAMD9
HSPA1B
KBTBD4;BEX1;CXADR;WT1;C6ORF106;FEM1B;HILPDA;RPL37A;HOXA1
WT1;HILPDA;TNFSF10;CD244;HK2
HSPA1B
IFIH1;EFNA1;HSPA1B
HSPA1B
HSPA1B
TNFSF10
DNTT
WT1;CTSG;HK2
BEX1
SRGN
DNTT
BEX1;HOXA1
CD274;HSPA1B
BEX1;RPL37A;C3ORF52;HOXA1
EEF1A1
EPHB4
EPHB4
BEX1
WT1;RPS6;HSPA1B
RPS6KA2
RPS6KA2
RPS6KA2
EPHB4
TNFSF10

TNFSF10
PTGDR2;RNASE3
NAV2
FEM1B;HILPDA;DOLK;EFNA1;KBTBD4;CLP1;MFSD5;RPL37A;C3ORF52;B3GNT2;APBB2;NRBF2;HSPA1B
IL12A
TNFSF10
DNTT
FEM1B;RPL37A;B3GNT2;APBB2;DOLK;HSPA1B
HILPDA;HK2
KIAA1217;RPS6
EPHB4
RPS6KA2
WT1
HSPA1B
RAPGEF5
DNTT
RPS6KA2;MYO3A;EPHB4
TMEM63A;MFSD5;RPL37A
RPS6KA2;IFI6;ROR1
IFITM1;SAMD9;HBG1;TNFSF10;IFI6;PAX5;COMMD6
KBTBD4;CXADR;WT1
TNFSF10;DEFA3;CST7;RPL39
RPS6
RPL31
DUSP14
EFNA1
SEPP1
HSPA1B
RAPGEF5
VPREB3;TMEM104;RPL31
EPHB4
RPL37A
TNFSF10
RPS6KA2;MYO3A;EPHB4
EFNA1;SRGN;CD274;TNFSF10;RAPGEF5;ITM2B
PTGDR2;IFI6
DNTT;RAPGEF5
SRGN;RPL11;RPS6;GDE1;LY6E
TNFSF10
ROR1
SLC16A3
RPL37A;TOMM20;HSPA1B
SRGN;CD274;RPL31;CYP4F3;HILPDA;RPL7;IFIH1;CXCL11;DOK4;MFSD5;POU2AF1;RPL37A;MYO3A;TNFSF10;HBBP1;ITM2A
EPHB4
IFIH1
HOXA1
IFNB1
LAMP5;WT1;CTSG;HSPA1B
IFIH1;FEM1B
TRPC6;PTGDR2
WT1;TNFSF10
EFNA1
TNFSF10
IFIH1;FEM1B;CLDN9;RPL37A;B3GNT2;APBB2;DOLK;HSPA1B
HILPDA;HK2
NKG7;NAV2
RNASE3;TPT1
HSPA1B
HSPA1B
TNFSF10
HBG2;CD244
TPT1
HSPA1B
BEX1;RPS6KA2
TNFSF10;DNTT
IFIH1;BMP3;RPS6
CXADR;HOXA1;SEPP1
RPS6
NAV2
RPS6KA2;MYO3A
RPS6KA2
RPS6
HSPA1B
RPL31
RPS6KA2
DNTT
HOXA1;HSPA1B
HSPA1B
IFI6
TRPC6
TNFSF10
HBG2
SRGN;IFITM1;BEX1;CXADR;HILPDA;GDE1;RPL13A;SEPP1;TM4SF1;IFIH1;RPS19;ARL6IP5;TNFSF10;NRBF2;HSPA1B;ITM2B;EPHB4
TNFSF10;DNTT
WT1

WT1
DNTT
PAX5
IFNB1
TNFSF10
IFIH1
RAPGEF5
RPL31;HOXA1;SLC16A3;HOXB5;LY6E
PTGDR2;AMDHD2;APBB2
HSPA1B
DNTT
SRGN;HBG2;GAD1;HILPDA;ARL6IP5;TNFSF10;CTSG;TOMM20;HK2
EFNA1;DOK4
RPL31;HSPA1B
PAX5;RAG1
HBG2;RNASE3
EEF1A1;RNASE3
RPS6KA2
SLC16A3
IFNB1
TNFSF10
TNFSF10
RPS6KA2
EPHB4
IFNB1;RAPGEF5
TRPC6;RAPGEF5
WT1;NRBF2
WT1;RAPGEF5
IFNB1;DNTT
RAPGEF5
RPS21
B3GNT2;IL12A;ROR1
RPS6KA2
NME8;CYP4F3
CYP4F3
TNFSF10
ROR1
RPL31;IFNB1;WT1;RPS6;TNFSF10;HOXB5
CLP1;C6ORF106;IFI6;B3GNT2
IFNB1;RAPGEF5
RPS6;RAPGEF5
CXADR;C6ORF106;GAD1;RPS6KA2;IL12A;PAX5;NAV2;HSPA1B
MYO3A
HRK
WT1
TNFSF10
WT1
EFNA1
TNFSF10
RAPGEF5
EPHB4
TNFSF10;DNTT
PTGDR2;MARC2;AEBP1
HK2
EPHB4
EEF1A1
RAPGEF5
TNFSF10
WT1;CTSG
GAD1;RPSA;EPHB4
HSPA1B
RPS6KA2;MYO3A
RPSA
TNFSF10;VAT1L
EFNA1;GAD1;C3ORF52;ROR1
HSPA1B
TNFSF10
IFIH1
CD274;IFITM1;ARL6IP5;TPT1;LY6E
HILPDA;HSPA1B
HILPDA
RPSA
CLDN9;GAD1
FCRL1;HRK;LINC00472;NME8;MYO3A;TNFSF10;CST7
NKG7
HSPA1B
RPL31;RPS3A
RPS6KA2;MYO3A
TNFSF10;DNTT
EFNA1;GAD1;RPL37A;C3ORF52;HOXA1;HSPA1B
EPHB4
EPHB4
COL5A3
RPS6
TNFSF10;IL12A

HRK;CXCL11
EPHB4
EPHB4
TNFSF10
EFNA1
IFIH1;FEM1B;B3GNT2;APBB2;DOLK;HSPA1B
RPS6KA2
MFSD5;HILPDA;ARL6IP5;DOLK;RAPGEF5;LY6E
HSPA1B
BMP3
EEF1A1;ADGRE1;TNFSF10;NKG7;SLC16A3;LY6E
RAPGEF5
SRGN;TNFSF10;RPS3A
MYO3A
TNFSF10
BEX1;CXADR;RPL31;WT1;RPL37A;NT5DC3;C3ORF52;HOXA1
HSPA1B
EFNA1
WT1
RTP4;CYP4F3
RPS6KA2
RPS6KA2
SEPP1;POU4F1;HSPA1B;TPT1
TNFSF10;HSPA1B;HK2
RPS3A;RAG2;TM4SF1
RPS6KA2
EFNA1
SRGN;IFITM1;HHIP;IFI6;TM4SF1;DUSP14;EEF1A1;RPS25;WT1;TNFSF10;C3ORF52;RAPGEF5;HSPA1B;EPHB4;LY6E
RNASE3;HSPA1B
CDHR2;TM4SF1
TNFSF10
PAX5
EFNA1;MFSD5;HILPDA;B3GNT2
RPS6;TNFSF10
EFNA1;IFNB1;HILPDA;TNFSF10;CMPK2;MDFI
ARL6IP5;TNFSF10
RPL37A
RPL37A;C3ORF52;TM4SF1
HSPA1B
COL5A3;HILPDA;IL12A;HK2
CXADR;RPS6KA2;C3ORF52;HOXA1;SLC16A3;HSPA1B;DUSP14
C6ORF106;TM4SF1
IFIH1;WT1
CMPK2
B3GNT2
RTP4;RPS3A
HSPA1B
GDE1
HSPA1B
RPL37A;HSPA1B
CXADR;HOXA1;HSPA1B;DUSP14
RPL37A
HILPDA
RAPGEF5
IFITM1;CXCL11
CXADR;APBB2;GNG12
CD274;RPS6;ROR1;SEPP1;EPHB4
CLP1;RPL31;MFSD5;CLDN9;B3GNT2;RPS20;DUSP14
WT1;CTSG
TNFSF10;RPL37
HSPA1B
RPS6KA2;EPHB4
EPHB4
GDE1
IFIH1
FEM1B;HILPDA;DOLK;NAV2;EFNA1;CLP1;WT1;MFSD5;RPL37A;C3ORF52;B3GNT2;NRBF2;HSPA1B
GDE1;SLC16A3
RPL30;GDE1
EPHB4
BEX1
CYP4F3
DOK4;PAX5;AEBP1;ITM2A
SRGN;TNFSF10;ROR1;RAG1
IFIH1;FEM1B;DOLK;HSPA1B
C6ORF106;SLC16A3;HSPA1B
LAMP5;WT1;MFSD5;RPL37A;GDE1;ARL6IP5;SLC16A3;HSPA1B
IFIH1;NME8;CYP4F3
EPHB4
CD274;CMPK2
TMEM63A
EEF1A1;TOMM20
EPHB4
DUSP14
FEM1B;HILPDA;HSPA1B
TNFSF10

CXADR;DOK4;CLP1;RPS6KA2;SEPP1;GOLGA7;HSPA1B
EEF1A1;TOMM20
EFNA1
FCRL1;ADGRE1;CLP1;PTGDR2;DAAM2;GAD1;TNFSF10;HSPA1B;HOXB5
B3GNT2
CYP4F3
WT1
EPHB4
EFNA1
FEM1B
RPS6KA2
BEX1
EFNA1;KBTBD4;CLP1;WT1;MFSD5;FEM1B;HILPDA;RPL37A;NRBF2;NAV2;HSPA1B
TMEM63A;WT1;RPS6KA2;HILPDA;TNFSF10;CTSG;HSPA1B;CD244;HK2
EPHB4
HSPA1B
TNFSF10
MYO3A
EPHB4
IFITM1;CXADR
EEF1A1
EEF1B2;AOX2P;RPL30;TRPC6;CLP1;FUNDC1;SEPP1;CD244;EPHB4
HSPA1B
WT1;RPS6KA2;R3HCC1;HILPDA;RPL37A;TNFSF10;CTSG;CST7;TOMM20;CD244;HK2
CXCL11
HBG2
FEM1B;DOLK;HSPA1B;HK2
CXADR;BEX1
TNFSF10;TM4SF1
IFITM1;RPS19;WT1;GAD1;TNFSF10;B3GNT2;NPTX1;CST7;HSPA1B
LAMP5;ARL2BP;MFSD5;RPS6KA2;ARL6IP5;SLC16A3;HSPA1B
RPS6;IL12A
RTP4;DOK4;NME8;RPS3A
EFNA1;TM4SF1
FEM1B;HILPDA;DOLK;NAV2;HK2;EFNA1;KBTBD4;CLP1;MFSD5;RPL37A;C3ORF52;APBB2;NRBF2;HSPA1B
SRGN;RPL11;RPS6
RPS6KA2
RPS6KA2
CTSG
TNFSF10
RAPGEF5
NT5DC3
TNFSF10
HRK;HSPA1B
B3GNT2
RPS6KA2;MYO3A
SLC16A3
WT1
IFIH1;HSPA1B
IFIH1;BMP3;CXCL11;FCGR3B;SAMD9;DOK4;NME8;CYP4F3;RAG2
CEMIP;RPL37A;B3GNT2
FEM1B;HILPDA;DOLK;NAV2;EFNA1;KBTBD4;CLP1;WT1;MFSD5;RPL37A;B3GNT2;HOXA1;NRBF2;HSPA1B
NRBF2
EFNA1;IFITM1;HILPDA;RPLP0;TNFSF10;RPL13A
TMEM63A;IL12A;DOLK;ITM2B
WT1
SAMD9
ROR1;TM4SF1;HK2
AEBP1
NAV2
HSPA1B
APBB2
ABCA13;MDFI
ARL6IP5;TNFSF10
WT1
EFNA1
GAD1
WT1;FEM1B;RPS6KA2;HILPDA;HSPA1B;HK2
HK2
RTP4;FCGR3B;RPL37A
TMEM63A;FEM1B;HILPDA;DOLK;NAV2;EFNA1;KBTBD4;CLP1;MFSD5;RPL37A;B3GNT2;APBB2;NRBF2;HSPA1B
NAV2
RPS6;ARL6IP5;TNFSF10;NKG7
HILPDA
DNTT
DNTT
HRK
WT1;CTSG
APBB2
B3GNT2
GAD1
EEF1A1
IFI6
HBG2
EFNA1;C3ORF52

SRGN;PTGDR2;CLC
EFNA1
TNFSF10
IFNB1
RPS6
IFITM1;TMEM63A;RPS7;IFI6;AEBP1;RPL10A;RPL7;RPS27;RPS19;DAAM2;RPS6KA2;TNFSF10;RPL27;APBB2;UBA52;LY6E
TNFSF10
IFIH1;LAMP5;TMEM63A;TNFSF10;HOXA1;NRBF2;CD244
HSPA1B
EFNA1
HSPA1B
HBG2
RPS6KA2
DOK4
HBG2
EFNA1;NTNG2;VPREB1;DOK4;DHH;RPL12;RPS5;NKG7;IL12A;HSPA1B
RPS25;POU2AF1;CYP4F3;IFI6
NME8;CYP4F3
HK2
RPS6KA2
PTGDR2
WT1
WT1
HBG2
KIAA1217
NTNG2;RPL31;HHIP;GAD1;CYP4F3;ROR1;ZNF597
BEX1
RAPGEF5
HHIP;IFI6
TRPC6
GAD1
LY6E
RAPGEF5
RAPGEF5
DNTT
TNFSF10
TNFSF10
FEM1B;HILPDA;DOLK;DUSP14;EFNA1;KBTBD4;CLP1;MFSD5;RPL37A;B3GNT2;APBB2;NRBF2;HSPA1B
WT1
RAPGEF5
TM4SF1
RAPGEF5
RAPGEF5
DNTT
TNFSF10
CXADR;DOK4;CLP1;RPS6KA2;HOXA1;NAV2;SEPP1;HSPA1B
HRK;HSPA1B
BEX1;RPS6KA2
WT1
EFNA1;GAD1;HOXA1;ROR1
SRGN;CD274;CXCL11;RPL31;CYP4F3;RPL37A;TNFSF10;HOXA1
TM4SF1
CD274
RPS6KA2
DNTT
DNTT
TNFSF10
RPS6KA2;MYO3A
DNTT
WT1
SRGN
C3ORF52;HSPA1B
FEM1B;EPHB4;RPL7
SAMD9;WT1;FEM1B;HILPDA;HK2
EPHB4
RAPGEF5
NTNG2;RPL31;HHIP;GAD1;ROR1;ZNF597
FEM1B
EFNA1;C3ORF52
NTNG2;RPL31;HHIP;GAD1;ROR1;ZNF597
WT1;TNFSF10;VAT1L
TNFSF10;IFI6
RPS6KA2
SRGN
TOMM20;HSPA1B
TNFSF10
RPS3A;RAG2
FEM1B;RPS6KA2;HILPDA;HK2
GDE1;B3GNT2;SLC16A3
PTGDR2;MARC2
HK2
CLP1;MFSD5;NRBF2;HSPA1B
WT1;CTSG;CD244;HK2
HSPA1B
SAMD9;RPL37A

CLP1
ARL6IP5;SLC16A3
PTGDR2;HBG1;ROR1;GCSAM;CD244
FEM1B;HILPDA;DOLK;NAV2;EFNA1;KBTBD4;CLP1;WT1;MFSD5;RPL37A;C3ORF52;NRBF2;HSPA1B
SAMD9;NME8;CYP4F3
TNFSF10
IFIH1;RTP4;DOK4;NME8;RPS6;APBB2;ROR1;RPS3A;HBBP1;TM4SF1;MDFI
EFNA1;C3ORF52;DUSP14
SRGN
WT1;FEM1B;HILPDA;HK2
HSPA1B
ITM2B
RPL37A;GDE1
EFNA1;CLDN9
KBTBD4;WT1
NPTX1
MIR27A
WT1;CTSG;CD244;HK2
TNFSF10
HOXA1
RPL37A
BEX1
EFNA1;NME8;CYP4F3
EFNA1;C3ORF52;B3GNT2
EFNA1;C6ORF106;B3GNT2;TM4SF1
TNFSF10;ROR1;DUSP14
EFNA1
B3GNT2;DOLK
IFIH1;TNFSF10;CD244
HSPA1B
SRGN;CEMIP;TRPC6;SAMD9;HBG2;HBG1;DEFA3;NKG7;PAX5;KIAA1217;EFNA1;CXCL11;WT1;TNFSF10;CMPK2;RPS20;IL12A;ITM2A;HSPA1B
SRGN
DNTT
GDE1
EFNA1
EFNA1;GAD1;HOXA1;ROR1;NPTX1;SLC26A6
WT1;CTSG;HK2
EFNA1;CEMIP;GAD1;C3ORF52;TM4SF1;SLC26A6;DUSP14
KBTBD4;ROR1
C3ORF52;IL12A;DUSP14
RTP4;IFITM1;BEX1;HILPDA;RPL9;HK2;RPL7;DUSP14;FCGR3B;ADGRG5;ARL6IP5;HOXA1;RPL39;SLC16A3;IFNB1;GAD1;RPL13A;DOLK;RPS3A;NAV2;SEPP1;EFNA1;MIR24-2;RPS27;CLDN9;HSPA1B;ITM2B;TPT1
SRGN;RPL37A;GDE1;TM4SF1
IFIH1
C1QA;IFITM1;CEMIP;SAMD9;RPL31;PTGDR2;RPLP0;IFI6;TOMM20;HK2;RPL7;IFIH1;VPREB1;RPS17;MARC2;FUNDC1;TNFSF10;RPS3;APBB2;NPTX1;RPL41;NPM2;CXADR;HBG1;GAD1;RPS6;RPL13A;NAV2;COMMD6;SEPP1;TM4SF1;EEF1A1;EFNA1;CXCL11;RPS27;DOK4;DAAM2;POU2AF1;FAM32A;ROR1;RAPGEF5;SLC26A6
RPS6KA2
NRBF2;NAV2
BEX1;RPS6KA2
EFNA1;C3ORF52
IFITM1;RPL30;BEX1;IFI6;DTX1;HK2;BEGAIN;LAMP5;EEF1B2;ADGRE1;RPS14;TMEM104;C6ORF106;ARL6IP5;TNFSF10;RPS3;HOXA1;APBB2;IL12A;CTSG;NPTX1;SLC16A3;VAT1L;NTNG2;ARPP21;IFNB1;KCNJ12;MIR23A;GAD1;CYP4F3;DEFA3;NKG7;RPSA;PAX5;NAV2;KIAA1217;EEF1A1;EFNA1;CXCL11;DOK4;WT1;POU2AF1;ROR1;ITM2A;ITM2B;TPT1;HOXB5;LY6E
GDE1
EFNA1;C3ORF52
DOLK
BEX1;WT1;RPLP0;GOLGA7;HSPA1B
IFIH1;LAMP5;TNFSF10;HOXA1;NRBF2;CD244
CXADR;C6ORF106;ARL6IP5;SLC16A3
CLC
FEM1B;HILPDA;HK2
ARL6IP5;TM4SF1;HSPA1B
B3GNT2
CYP4F3;RPS6
CYP4F3;CMPK2
NTNG2;CD274;CXCL11;CXADR;SAMD9;RPS19;DOK4;RPS6;B3GNT2;TM4SF1
TNFSF10
EFNA1;MFSD5;RPL37A;B3GNT2
IFITM1;CYP4F3;HILPDA;TNFSF10;ROR1
EEF1A1;HHIP;RPS6;GDE1;APBB2;RPSA;RPL37;TOMM20;SLC16A3;MDFI
FEM1B;SLC16A3
HRK;RPL31;RPL35A;SLC26A6;HK2
CXCL11;KCNJ12;NPTX1;ITM2A;POU4F1;VAT1L
KBTBD4;WT1;MFSD5;HK2
EFNA1
RPS6KA2
EFNA1;KBTBD4;MFSD5
EFNA1
PTGDR2
IFIH1;EFNA1;CEMIP;NRBF2
SLC16A3
HRK;RPL31;HHIP;RPLP0;IFI6;ABCA13;HK2;IFIH1;CECR2;CLP1;C3ORF52;APBB2;IL12A;RPL37;SLC16A3;VAT1L;RAG1;NTNG2;TRPC6;CXADR;GAD1;NAV2;GNG12;SEPP1;POU4F1;KIAA1217;EFNA1;KBTBD4;RPS27;WT1;DAAM2;CLDN9;COL5A3;ROR1;ITM2A;TPT1;RPS23
CXADR;RPL37A;SLC16A3
WT1;FEM1B;HILPDA;HK2
RPL31
RPS25;RPS19;DOK4;RPS29;RPS18;TNFSF10;SLC16A3;HK2;EPHB4
MIR23A;HHIP;RPS6;RPS3A;SEPP1;HK2;EFNA1;DOK4;TNFSF10;HOXA1;IL12A;HSPA1B;TPT1
EFNA1;KBTBD4
NTNG2;CD274;CEMIP;CXADR;SAMD9;HHIP;SEPP1;TM4SF1;EEF1A1;CXCL11;RPS19;DOK4;ARL2BP;POU2AF1;B3GNT2;ROR1;RAG1

ADGRE1;PYURF;FEM1B;AMDHD2;TNFSF10;TM4SF1;HSPA1B;HK2
GDE1;GOLGA7
EFNA1
SRGN;MFSD5;GDE1;NRBF2;GOLGA7;TM4SF1
MARC2;CYP4F3;RPL37;HSPA1B;HK2;BEGAIN
TMEM63A;CXADR;C6ORF106;ARL6IP5;SLC16A3
SAMD9;RPL34;RPS3A;TOMM20;TM4SF1;HK2;PDRG1;IFIH1;EEF1A1;EFNA1;CLP1;RPL37A;TNFSF10;APBB2;RPL37;SLC16A3
HRK;CXADR;GAD1;PDRG1;EEF1A1;RPS19;POU2AF1;RPL37A;ARL6IP5;RPL35;B3GNT2;NRBF2;HSPA1B
NRBF2
EFNA1;KBTBD4;MFSD5;FEM1B;HILPDA;RPL37A;B3GNT2;APBB2;DOLK;NRBF2;HSPA1B
EFNA1
EFNA1;KBTBD4;CLP1;MFSD5;FEM1B;HILPDA;RPL37A;DOLK;NRBF2;HSPA1B;DUSP14
RPS3A
CXADR;HILPDA;APBB2;ROR1;NAV2;HK2
SRGN;CEMIP;RPSA;ABCA13;EEF1A1;WT1;MARC2;RPS6KA2;B3GNT2;HOXA1;RPL37;RAPGEF5;LY6E
EFNA1
ARL6IP5;NRBF2
FEM1B;CYP4F3;TNFSF10;B3GNT2;DOLK;NRBF2;LY6E
FCGR3B;SAMD9;CYP4F3;CDHR2
MFSD5;C3ORF52;APBB2;SEPP1;SLC16A3;HSPA1B;PDRG1
IFIH1
CXADR
CMPK2
IFIH1
SAMD9;RPL34;RPS3A;TOMM20;TM4SF1;HK2;PDRG1;IFIH1;EEF1A1;EFNA1;CLP1;RPL37A;TNFSF10;APBB2;RPL37;SLC16A3;HSPA1B
LAMP5;CLP1;MFSD5;FEM1B;RPS6KA2;NT5DC3;B3GNT2;FAM32A;CST7;SLC16A3;HSPA1B
EEF1A1;EFNA1;APBB2;SEPP1;TM4SF1
IFITM1;CEMIP;RPL3;BEX1;SAMD9;GDE1;IFI6;CST7;ABCA13;HK2;RPL7;DUSP14;VPREB3;IFIH1;TMEM104;MFSD5;RPS6KA2;TNFSF10;C3ORF52;APBB2;NPTX1;SLC16A3;SRGN;LINC00472;NPM2;TRPC6;CXADR;GAD1;MIR27A;NAV2;SEPP1;POU4F1;TM4SF1;EEF1A1;EFNA1;RPS25;ANGPTL6;POU2AF1;ROR1;RAPGEF5;UBA52;HSPA1B;ITM2B;SLC26A6
WT1;HILPDA;HSPA1B;HK2
ROR1
APBB2;DOLK;NAV2;HSPA1B
IFIH1;C6ORF106;FEM1B;B3GNT2;NRBF2;DUSP14
WT1;GDE1;B3GNT2;NRBF2;CST7;GOLGA7;SLC16A3
KBTBD4;FEM1B;HILPDA;RPL37A;C3ORF52;B3GNT2;APBB2;DOLK;NRBF2
WT1
WT1;CST7
SRGN;RPL3;RPL37A;ARL6IP5;IFI6;CST7;SLC16A3
FEM1B;NRBF2;DUSP14
HHIP;HILPDA;HK2;RPS19;C6ORF106;RPS6KA2;APBB2;NRBF2;GOLGA7;SLC16A3;EPHB4;FEM1B;RPL35A;NAV2;GNG12;EEF1A1;WT1;RPL37A;B3GNT2;RPL27;UBA52;HSPA1B;ITM2B;MDFI;LY6E
RTP4;CD274;RPL31;HILPDA;GDE1;HK2;RPL7;DUSP14;FCRLA;IFIH1;CLP1;TNFSF10;HOXA1;FEM1B;MIR23A;CYP4F3;MIR27A;RPL35A;GCSAM;CXCL11;DOK4;RPL37A;ROR1;HBBP1;ITM2A;HSPA1B
EFNA1;C1QA;TRPC6;ARPP21;R3HCC1;TM4SF1;EPHB4
WT1;RPL37A;NRBF2;HSPA1B
RTP4;CD274;TMEM63A;CYP4F3;HK2;RPL7;DUSP14;FCRLA;IFIH1;CXCL11;CLP1;TNFSF10;IL12A;ROR1;RPL37;ITM2A
SRGN;WT1;RPL37A;GDE1;ARL6IP5;NRBF2;CST7;GOLGA7;SLC16A3
GDE1;SLC16A3
WT1;KCNJ12;MYO3A;B3GNT2;PAX5;HSPA1B
CEMIP;BEX1;SAMD9;HILPDA;IFI6;HK2;IFIH1;CLP1;ARL6IP5;TNFSF10;C3ORF52;APBB2;SLC16A3;HBG2;GAD1;RPSA;DOLK;NAV2;TM4SF1;EFNA1;KBTBD4;POU2AF1;CMPK2;HSPA1B;RPS23
EFNA1
EFNA1;GAD1
EFNA1;EEF1B2;ARL6IP5;CCDC102A;RPL9;HSPA1B;SLC26A6;EPHB4;BEGAIN
WT1;RPL37A;GDE1;ARL6IP5;SLC16A3
NTNG2;IFITM1;RPL32;RPL34;HILPDA;RPLP0;IFI6;CCDC102A;TOMM20;VPREB3;RPS17;DOK4;CLP1;RPS29;MFSD5;RPS6KA2;FAM32A;ZNF689;RPS27A;SLC16A3;HSPA1B;LY6E
WT1;HILPDA;RPL37A;C3ORF52;NRBF2;NAV2;HK2
HILPDA;HK2;DUSP14;CLP1;MFSD5;NT5DC3;C3ORF52;HOXA1;APBB2;NRBF2;SLC16A3;KCNJ12;FEM1B;GAD1;RPL35A;DOLK;NAV2;EFNA1;KBTBD4;WT1;RPL37A;B3GNT2;ROR1;HSPA1B;SLC26A6
RPL31;RPL37A;GDE1;ARL6IP5;NRBF2;SLC16A3
KBTBD4;WT1;MFSD5
RTP4;RPL30;SAMD9;HILPDA;IFI6;RPS14;RPS19;MARC2;FUNDC1;RPS6KA2;ARL6IP5;RPL36;TNFSF10;RPS3;C3ORF52;IL12A;NRBF2;NPTX1;SLC16A3;SRGN;CXADR;FEM1B;RPS6;SNHG6;NAV2;SEPP1;POU4F1;TM4SF1;PDRG1;EEF1A1;KBTBD4;DOK4;DAAM2;CDHR2;B3GNT2;RAPGEF5;RPS21;HSPA1B;SLC26A6;LY6E
EEF1A1;EFNA1;RPL31;RPL37A;C3ORF52;APBB2;SEPP1;TM4SF1
SRGN;NTNG2;SAMD9;IFI6;GNG12;SEPP1;TM4SF1;DUSP14;FCRLA;ADGRE1;CXCL11;CLP1;MARC2;RPS6KA2;C3ORF52;IL12A;ROR1;FAM81B;MDFI;SLC26A6
RTP4;IFITM1;CEMIP;DHH;RPL12;IFI6;RPL9;RPL7;IFIH1;TMEM104;MFSD5;FUNDC1;ARL6IP5;TNFSF10;APBB2;ZNF689;EPHB4;NTNG2;CYP4F3;RPS3A;NAV2;GNG12;TM4SF1;EFNA1;RPS25;CLDN9;CMPK2;B3GNT2;SLC26A6;RPS23
HRK;RPL3;RPL31;RPL34;HILPDA;RPLP0;GDE1;RPL10A;TOMM20;HK2;RPL7;EEF1B2;C6ORF106;MFSD5;RPS6KA2;ARL6IP5;TNFSF10;C3ORF52;ZNF689;RPL39;GOLGA7;SLC16A3;EPHB4;SRGN;CXADR;RPS3A;NAV2;SEPP1;PDRG1;EEF1A1;CLDN9;MYO3A;B3GNT2;FAM32A;ROR1;RAPGEF5;ZNF792;BEX1;AMDHD2;RPS3;APBB2;NPTX1;NTNG2;RPL41;NPM2;CYP4F3;DOLK;EFNA1;A
HRK;DPP10;IFITM1;RPL31;RPL34;HHIP;HILPDA;GDE1;RPL10A;CCDC102A;ABCA13;TOMM20;HK2;RPL7;IFIH1;LAMP5;EEF1B2;CLP1;MFSD5;RPS6KA2;ARL6IP5;RPL36;TNFSF10;C3ORF52;HOXA1;ZNF689;RPL37;FAM81B;RPL39;SLC16A3;RPS12;RAG1;TRPC6;CXADR;RPS3A;NAV2;COMMD6;SEPP1;TM4SF1;KIAA1217;EEF1A1;NME8;RPL37A;MYO3A;CMPK2;B3GNT2;RPL27;ROR1;R
CD274;IFITM1;TMEM63A;RPL34;GDE1;CCDC102A;TOMM20;HK2;RPL7;DUSP14;IFIH1;CLP1;TNFSF10;IL12A;ZNF689;RPL37;CYP4F3;SNHG6;PDRG1;POU2AF1;COL5A3;R3HCC1;RPL37A;ROR1;ITM2A;ZNF597;LY6E
APBB2;HK2

SUPPLEMENTARY TABLE 3: DRUG MATRIX TABLE

Term	Overlap	P-value	Adjusted P-value	Old P-value	Old Adjusted P-value	Odds Ratio	Combined Score	Genes
Lead_(II)_Acetate-600_mg/kg_in_Saline-Rat-Kidney-5d-up	19/328	3,74559E-07	0,002950024	0	0	3,981225379	58,91225243	C1QA;RPL32;RPS8;RPS5;RPL13A;RPL35A;RPSA;RPS3A;RPL10A;TOMM20;EEF1B2;RPS17;CXCL11;RPS19;ARL6IP5;RPS20;RPL39;GOLGA7;TPT1
Lead(IV)_Acetate-600_mg/kg_in_Saline-Rat-Kidney-5d-up	18/309	7,04103E-07	0,002772756	0	0	4,003603243	56,71641214	C1QA;RPL32;RPS8;RPS5;RPS6;RPL13A;RPL35A;RPSA;RPS3A;RPL10A;TOMM20;RPS17;CXCL11;RPS19;ARL6IP5;RPS20;RPL39;TPT1
Vancomycin-160_mg/kg_in_Saline-Rat-Kidney-5d-up	17/290	1,3217E-06	0,003469901	0	0	4,028913378	54,53775834	C1QA;RPL32;RPS5;RPS6;RPL13A;RPL35A;RPSA;RPS3A;RPL10A;SEPP1;RPS17;CXCL11;RPS19;ARL6IP5;RPL39;GOLGA7;TPT1
Zidovudine-14800_uM_in_DMSO-Rat-Primary_rat_hepatocytes-0.67d-up	17/293	1,52165E-06	0,002996125	0	0	3,987661706	53,41758663	RPL32;RPS8;FEM1B;RPS5;RPS6;RPL13A;RPL35A;RPS3A;PDRG1;EEF1B2;RPS17;RPS19;ARL2BP;CLP1;RPS20;RPL39;TPT1
Valproic_Acid-1500_mg/kg_in_Water-Rat-Heart-5d-up	17/299	2,00558E-06	0,003159188	0	0	3,907641738	51,26660933	C1QA;RPL32;RPS8;RPS5;RPS6;RPL35A;RPSA;RPS3A;RPL10A;SEPP1;RPS17;RPS19;FUNDC1;RPS20;RAPGEF5;RPL39;TPT1
Dibromochloromethane-325_mg/kg_in_CMC-Rat-Kidney-5d-up	17/305	2,62438E-06	0,003444933	0	0	3,830770097	49,22794991	RPL32;RPS8;RPS5;RPS6;RPL35A;RPSA;RPS3A;RPL10A;TOMM20;EEF1B2;RPS17;RPS19;ARL2BP;FUNDC1;RPS20;RPL39;TPT1
Aminosalicylic_Acid-2337_mg/kg_in_CMC-Rat-Liver-5d-up	17/307	2,86602E-06	0,003224685	0	0	3,80581394	48,57202543	RPL32;RPS8;FEM1B;RPS5;RPS6;RPL13A;RPL35A;RPSA;RPS3A;RPL10A;SEPP1;EEF1B2;RPS17;RPS19;RPS20;RPL39;TPT1
Clemastine-2500_mg/kg_in_CMC-Rat-Heart-5d-up	16/276	3,13639E-06	0,003087778	0	0	3,984262164	50,4903135	C1QA;RPL32;RPS8;RPS6;RPL35A;RPSA;RPS3A;RPL10A;SEPP1;EEF1B2;RPS17;RPS19;RPS20;RAPGEF5;RPL39;TPT1
Acyclovir-980_mg/kg_in_Saline-Rat-Bone_marrow-1d-dn	15/245	3,28747E-06	0,002876905	0	0	4,207868714	53,1259888	RPL32;RPS8;FEM1B;RPS5;NKG7;RPL13A;RPL35A;RPSA;RPS3A;RPL10A;EEF1B2;RPS17;RPS19;RPS20;RPL39
Dibromochloromethane-325_mg/kg_in_CMC-Rat-Kidney-3d-up	17/312	3,56022E-06	0,002804033	0	0	3,744823332	46,98138141	RPL32;RPS8;RPS5;RPS6;RPSA;RPS3A;RPL10A;SEPP1;EEF1B2;RPS17;CXCL11;RPS19;ARL6IP5;RPS20;RPL39;GOLGA7;TPT1
Estriol-313_mg/kg_in_CMC-Rat-Liver-1d-up	17/312	3,56022E-06	0,002549121	0	0	3,744823332	46,98138141	RPL32;RPS8;FEM1B;RPS5;RPS6;RPL13A;RPL35A;RPSA;RPS3A;RPL10A;EEF1B2;RPS17;RPS19;RPS20;RPL39;GOLGA7;TPT1
Lead(IV)_Acetate-600_mg/kg_in_Saline-Rat-Kidney-3d-up	18/347	3,66119E-06	0,002402962	0	0	3,565168306	44,62778523	RPL32;RPS8;RPS5;RPS6;RPL13A;RPL35A;RPSA;RPS3A;RPL10A;TOMM20;EEF1B2;RPS17;CXCL11;RPS19;ARL6IP5;RPS20;RPL39;TPT1
Cytarabine-23_mg/kg_in_Saline-Rat-Liver-3d-up	16/286	4,94446E-06	0,002995581	0	0	3,844952299	46,97471702	RPL32;RPS8;RPS5;RPS6;RPL35A;RPSA;RPS3A;RPL10A;SEPP1;TOMM20;EEF1B2;RPS17;RPS19;RPS20;RPL39;TPT1
Idarubicin-0.625_mg/kg_in_Saline-Rat-Heart-3d-dn	16/286	4,94446E-06	0,002781611	0	0	3,844952299	46,97471702	RPL32;RPS8;RPS5;RPS6;RPL35A;RPSA;RPS3A;TOMM20;EEF1B2;RPS17;RPS19;ARL2BP;FUNDC1;ARL6IP5;RPS20;TPT1
Cisplatin-2_mg/kg_in_Saline-Rat-Kidney-5d-up	17/322	5,41934E-06	0,002845517	0	0	3,628524471	43,99780345	RPL32;RPS8;RPS5;RPS6;RPL13A;RPL35A;RPSA;RPS3A;RPL10A;TOMM20;RPS17;CXCL11;RPS19;ARL6IP5;RPS20;RPL39;TPT1
Rolipram-75_mg/kg_in_CMC-Rat-Heart-5d-up	17/322	5,41934E-06	0,002667672	0	0	3,628524471	43,99780345	RPL32;RPS8;RPS5;RPL35A;RPS3A;RPL10A;TOMM20;EEF1B2;RPS17;ARL2BP;CLP1;FUNDC1;ARL6IP5;RPS20;RAPGEF5;RPL39;TPT1
Ketoconazole-114_mg/kg_in_Corn_Oil-Rat-Liver-3d-dn	16/292	6,43266E-06	0,002980213	0	0	3,765946429	45,01858528	C1QA;RPL32;RPS8;RPS5;RPS6;RPL35A;RPSA;RPS3A;RPL10A;SEPP1;EEF1B2;RPS17;RPS19;RPS20;RPL39;TPT1
Bromodichloromethane-430_mg/kg_in_CMC-Rat-Kidney-3d-up	16/302	9,81842E-06	0,004296106	0	0	3,641246217	41,98812019	RPL32;RPS8;RPS5;RPS6;RPSA;RPS3A;RPL10A;SEPP1;EEF1B2;RPS17;CXCL11;RPS19;CLP1;RPS20;RPL39;TPT1
Timolol-10_mg/kg_in_Water-Rat-Heart-0.25d-up	16/303	1,02319E-05	0,004241403	0	0	3,629228902	41,69983143	RPL32;RPS8;RPS5;RPL35A;RPSA;RPS3A;RPL10A;PDRG1;EEF1B2;RPS17;RPS19;ARL6IP5;RPS20;RAPGEF5;RPL39;TPT1
Aminosalicylic_Acid-2337_mg/kg_in_CMC-Rat-Liver-1d-up	16/304	1,06609E-05	0,00419826	0	0	3,617290649	41,41410147	RPL32;RPS8;RPS5;RPS6;RPSA;RPS3A;RPL10A;PDRG1;EEF1B2;RPS17;RPS19;ARL2BP;RPS20;RPL39;GOLGA7;TPT1
Cyclosporin_A-350_mg/kg_in_Corn_Oil-Rat-Kidney-5d-up	16/304	1,06609E-05	0,003998343	0	0	3,617290649	41,41410147	C1QA;RPL32;RPS8;RPS5;RPS6;RPL35A;RPSA;RPS3A;RPL10A;EEF1B2;RPS17;RPS19;ARL2BP;RPS20;RPL39;TPT1
Penicillin_G-243_mg/kg_in_Saline-Rat-Kidney-5d-up	16/304	1,06609E-05	0,0038166	0	0	3,617290649	41,41410147	C1QA;RPL32;RPS5;RPS6;RPL13A;RPL35A;RPSA;RPL10A;SEPP1;TOMM20;RPS17;RPS19;ARL6IP5;RPS20;RAPGEF5;RPL39
Tacrolimus-134_mg/kg_in_CMC-Rat-Heart-5d-up	16/306	1,15672E-05	0,003961024	0	0	3,593648227	40,85019985	RPL32;RPS8;RPS6;RPSA;RPS3A;RPL10A;SEPP1;EEF1B2;RPS17;CXCL11;RPS19;ARL2BP;RPS20;RAPGEF5;RPL39;TPT1
Dibromochloromethane-325_mg/kg_in_CMC-Rat-Kidney-1d-up	16/310	1,35885E-05	0,004459285	0	0	3,547278572	39,75182703	RPL32;RPS8;RPS5;RPS6;RPSA;RPS3A;RPL10A;SEPP1;EEF1B2;RPS17;RPS19;CLP1;ARL6IP5;RPS20;RPL39;TPT1
Idarubicin-0.625_mg/kg_in_Saline-Rat-Heart-1d-dn	16/310	1,35885E-05	0,004280913	0	0	3,547278572	39,75182703	RPL32;RPS8;RPS6;RPL35A;RPSA;RPS3A;SEPP1;TOMM20;EEF1B2;RPS17;RPS19;ARL2BP;FUNDC1;ARL6IP5;RPS20;TPT1
Diethylstilbestrol-280_mg/kg_in_Corn_Oil-Rat-Liver-5d-up	15/276	1,3803E-05	0,004181246	0	0	3,735245779	41,79973483	RPL32;RPS8;FEM1B;RPS5;RPS6;RPL35A;RPSA;RPS3A;RPL10A;EEF1B2;RPS17;RPS19;RPS20;RPL39;TPT1
Tobramycin-40_mg/kg_in_Saline-Rat-Kidney-28d-up	16/311	1,41405E-05	0,00412484	0	0	3,535872532	39,4832027	C1QA;RPL32;RPS8;FEM1B;RPS5;RPSA;RPS3A;RPL10A;EEF1B2;RPS17;CXCL11;RPS19;ARL6IP5;RPS20;RPL39;TPT1
Cytarabine-23_mg/kg_in_Saline-Rat-Liver-0.25d-up	16/313	1,53048E-05	0,004305029	0	0	3,513279097	38,95292799	RPL32;RPS8;RPS5;RPS6;RPL35A;RPSA;RPS3A;RPL10A;SEPP1;TOMM20;EEF1B2;RPS17;RPS19;RPS20;RPL39;TPT1
Estriol-313_mg/kg_in_CMC-Rat-Liver-5d-up	16/313	1,53048E-05	0,00415658	0	0	3,513279097	38,95292799	RPL32;RPS8;FEM1B;RPS6;RPSA;RPS3A;RPL10A;SEPP1;RPS17;RPS19;ARL2BP;ARL6IP5;RPS20;RPL39;GOLGA7;TPT1
Primaquine-45_mg/kg_in_CMC-Rat-Liver-5d-up	16/315	1,65537E-05	0,00434589	0	0	3,490972563	38,43177791	C1QA;RPL32;RPS8;RPS5;RPS6;RPSA;RPS3A;RPL10A;SEPP1;EEF1B2;RPS17;RPS19;ARL2BP;RPS20;RPL39;TPT1
Thioguanine-24_mg/kg_in_Corn_Oil-Rat-Spleen-5d-up	16/315	1,65537E-05	0,0042057	0	0	3,490972563	38,43177791	C1QA;RPL32;RPS8;RPS5;RPL35A;RPSA;RPS3A;RPL10A;SEPP1;EEF1B2;RPS17;CXCL11;RPS20;RPL39;GOLGA7;TPT1
Beta-Estradiol_3-Benzoate-2_mg/kg_in_Corn_Oil-Rat-Liver-3d-up	15/284	1,93136E-05	0,004753559	0	0	3,630027588	39,40286501	RPL32;RPS8;RPS5;RPS6;RPL13A;RPL35A;RPSA;RPL10A;SEPP1;TOMM20;RPS17;RPS19;RPS20;RPL39;TPT1
Betamethasone-79_mg/kg_in_Corn_Oil-Rat-Liver-3d-dn	16/319	1,93263E-05	0,004612546	0	0	3,447198613	37,41604273	C1QA;RPL32;RPS8;RPS5;RPS6;RPL35A;RPSA;RPS3A;RPL10A;SEPP1;EEF1B2;RPS17;RPS19;RPS20;RPL39;TPT1
Gabapentin-500_uM_in_DMSO-Rat-Primary_rat_hepatocytes-1d-dn	16/320	2,00809E-05	0,004651676	0	0	3,436426117	37,16750049	RPL32;RPS8;RPS5;RPS6;RPL13A;RPL35A;RPSA;RPS3A;RPL10A;SEPP1;RPS17;RPS19;ARL2BP;ARL6IP5;RPL39;GOLGA7
Diethylstilbestrol-2.8_mg/kg_in_Corn_Oil-Rat-Liver-5d-up	15/285	2,01239E-05	0,004528443	0	0	3,617290649	39,11595017	RPL32;RPS8;FEM1B;RPS5;RPS6;RPL35A;RPSA;RPS3A;RPL10A;EEF1B2;RPS17;RPS19;RPS20;RPL39;TPT1
Dexchlorpheniramine-3_mg/kg_in_Water-Rat-Heart-3d-up	16/321	2,08615E-05	0,00456403	0	0	3,42572074	36,92106728	RPL32;RPS8;RPS5;RPS6;RPL35A;RPSA;RPL10A;TOMM20;RPS17;RPS19;ARL2BP;RPS20;RAPGEF5;RPL39;GOLGA7;TPT1
Diethylstilbestrol-280_mg/kg_in_Corn_Oil-Rat-Spleen-5d-up	15/286	2,09641E-05	0,00446251	0	0	3,60464278	38,83174031	C1QA;RPL32;RPS8;RPS5;RPL35A;RPSA;RPS3A;RPL10A;SEPP1;EEF1B2;RPS17;RPS19;RPS20;GOLGA7;TPT1
Diazepam-710_mg/kg_in_CMC-Rat-Heart-3d-dn	15/288	2,2738E-05	0,004712758	0	0	3,579610538	38,27130298	RPL32;RPS6;RPSA;RPS3A;RPL10A;SEPP1;PDRG1;RPS17;RPS19;ARL2BP;COL5A3;FUNDC1;ARL6IP5;RPS20;RPL39
Hexachlorophene-8_mg/kg_in_Corn_Oil-Rat-Liver-1d-up	14/254	2,28619E-05	0,004616941	0	0	3,788186271	40,48069793	C1QA;RPL32;RPS8;RPS5;RPS6;RPL35A;RPSA;RPS3A;RPL10A;SEPP1;RPS17;RPS19;RPS20;RPL39
Fludrocortisone_Acetate-125_mg/kg_in_CMC-Rat-Liver-3d-dn	16/324	2,33675E-05	0,004601053	0	0	3,394001103	36,19419112	C1QA;RPL32;RPS8;RPS5;RPS6;RPL35A;RPSA;RPS3A;RPL10A;SEPP1;EEF1B2;RPS17;RPS19;CLP1;RPS20;TPT1
Cyclopropane_Carboxylic_Acid-375_mg/kg_in_Water-Rat-Liver-1d-dn	16/325	2,42602E-05	0,004660327	0	0	3,383558023	35,95596493	C1QA;RPL32;RPS8;RPS6;RPSA;RPS3A;RPL10A;SEPP1;EEF1B2;RPS17;RPS19;ARL2BP;CLP1;RPS20;RPL39;TPT1
Neostigmine_Bromide-11_mg/kg_in_Water-Rat-Liver-1d-up	16/325	2,42602E-05	0,004549367	0	0	3,383558023	35,95596493	RPL32;RPS8;RPS5;RPS6;RPL35A;RPSA;RPS3A;RPL10A;SEPP1;TOMM20;EEF1B2;RPS17;RPS19;RPS20;RPL39;TPT1
6-Methoxy-2-Naphthylacetic_Acid-360_mg/kg_in_Saline-Rat-Kidney-5d-up	15/291	2,5648E-05	0,004697759	0	0	3,542707337	37,45011901	C1QA;RPL32;RPS8;RPS5;RPL13A;RPSA;RPS3A;RPL10A;SEPP1;EEF1B2;RPS17;RPS19;RPS20;RPL39;TPT1
Norethindrone-75_mg/kg_in_Corn_Oil-Rat-Liver-3d-up	15/291	2,5648E-05	0,004590991	0	0	3,542707337	37,45011901	RPL32;RPS8;FEM1B;RPS5;RPS6;RPSA;RPS3A;RPL10A;SEPP1;EEF1B2;RPS17;RPS19;ARL6IP5;RPS20;RPL39
33'4'5-Tetrachlorosalicylanilide-50_mg/kg_in_Corn_Oil-Rat-Liver-1d-dn	15/293	2,77662E-05	0,0048597	0	0	3,518525034	36,91527945	RPL32;RPS8;RPS5;RPS6;RPS3A;RPL10A;SEPP1;EEF1B2;RPS17;RPS19;FUNDC1;ARL6IP5;RPS20;RPL39;GOLGA7
Doxorubicin-0.65_mg/kg_in_Saline-Rat-Spleen-3d-dn	15/294	2,88821E-05	0,00494511	0	0	3,506557262	36,65155362	C1QA;RPL32;RPS8;RPS5;RPS6;RPL13A;RPL35A;RPSA;RPS3A;EEF1B2;RPS17;ARL6IP5;RPS20;RPL39;TPT1
Meloxicam-33_mg/kg_in_Corn_Oil-Rat-Kidney-5d-up	14/261	3,08601E-05	0,005171373	0	0	3,686587405	38,2890626	C1QA;RPL32;RPS8;RPS5;RPS6;RPL35A;RPSA;RPS3A;RPL10A;RPS17;RPS19;RPS20;RPL39;TPT1
Cisplatin-1.17_mg/kg_in_Saline-Rat-Bone_marrow-3d-dn	15/297	3,24707E-05	0,005327907	0	0	3,471137492	35,87480022	RPL32;RPS8;RPS5;RPS6;NKG7;RPL13A;RPL35A;RPSA;RPS3A;RPL10A;EEF1B2;RPS17;RPS20;RPL39;TPT1
Gemfibrozil-100_mg/kg_in_Corn_Oil-Rat-Liver-0.25d-up	15/297	3,24707E-05	0,005219174	0	0	3,471137492	35,87480022	C1QA;RPL32;RPS8;RPS6;RPSA;RPS3A;RPL10A;SEPP1;EEF1B2;RPS17;CXCL11;RPS19;RPL39;GOLGA7;TPT1
Sisomicin-34_mg/kg_in_Saline-Rat-Kidney-3d-up	15/297	3,24707E-05	0,005114791	0	0	3,471137492	35,87480022	C1QA;RPL32;RPS8;RPS5;RPS6;RPL13A;RPL35A;RPSA;RPS3A;SEPP1;EEF1B2;RPS17;RPS19;ARL6IP5;RPS20
Lead(IV)_Acetate-600_mg/kg_in_Saline-Rat-Kidney-1d-up	17/371	3,37182E-05	0,005207152	0	0	3,14928539	32,42967813	RPL32;RPS8;RPS5;RPS6;RPL13A;RPL35A;RPSA;RPS3A;TOMM20;EEF1B2;RPS17;CXCL11;RPS19;ARL6IP5;RPS20;RPL39;TPT1
Benzocaine-1100_mg/kg_in_Corn_Oil-Rat-Liver-0.25d-up	15/298	3,37514E-05	0,005112036	0	0	3,459489379	35,62059534	RPL32;RPS8;RPS5;RPS6;RPL35A;RPSA;RPS3A;RPL10A;SEPP1;EEF1B2;RPS17;RPS19;CLP1;RPS20;RPL39
Lomustine-4.2_mg/kg_in_Corn_Oil-Rat-Spleen-1d-dn	15/298	3,37514E-05	0,005015582	0	0	3,459489379	35,62059534	C1QA;RPL32;RPS8;RPS5;RPS6;RPL13A;RPL35A;RPSA;RPS3A;RPL10A;TOMM20;EEF1B2;RPS17;RPS19;RPL39
Miconazole-920_mg/kg_in_Corn_Oil-Rat-Liver-5d-up	15/298	3,37514E-05	0,004922701	0	0	3,459489379	35,62059534	RPL32;RPS8;RPS5;RPS6;RPL13A;RPL35A;RPSA;RPS3A;RPL10A;EEF1B2;RPS17;RPS19;RPS20;RPL39;TPT1
N-Nitrosodiethylamine-1.67_mg/kg_in_Saline-Rat-Spleen-0.25d-up	15/298	3,37514E-05	0,004833197	0	0	3,459489379	35,62059534	C1QA;RPL32;RPS8;RPS5;RPL13A;RPL35A;RPSA;RPS3A;RPL10A;SEPP1;TOMM20;EEF1B2;RPS17;RPS19;RPS20
Norethindrone_Acetate-125_mg/kg_in_Corn_Oil-Rat-Liver-1d-up	15/298	3,37514E-05	0,00474689	0	0	3,459489379	35,62059534	RPL32;RPS8;FEM1B;RPS6;RPSA;RPS3A;RPL10A;SEPP1;EEF1B2;RPS17;RPS19;ARL6IP5;RPS20;RPL39;TPT1
44'-Methylenedianiline-81_mg/kg_in_Corn_Oil-Rat-Liver-5d-up	16/334	3,37594E-05	0,004664726	0	0	3,292384304	33,8992128	C1QA;CXADR;RPL32;RPS8;FEM1B;RPS6;RPSA;RPS3A;RPL10A;RPS17;RPS19;ARL2BP;RPS20;RPL39;GOLGA7;TPT1
Hexachloro-13-Butadiene-50_mg/kg_in_Corn_Oil-Rat-Kidney-3d-up	15/301	3,78644E-05	0,005141723	0	0	3,425009419	34,87173178	RPL32;RPS8;RPS5;RPS6;RPL35A;RPSA;RPS3A;RPL10A;TOMM20;EEF1B2;RPS17;RPS19;ARL6IP5;RPS20;TPT1
Verapamil-108_mg/kg_in_Water-Rat-Heart-3d-up	15/301	3,78644E-05	0,005054575	0	0	3,425009419	34,87173178	RPL32;RPS5;RPS6;RPL35A;RPSA;RPS3A;RPL10A;RPS17;RPS19;ARL2BP;FUNDC1;ARL6IP5;RPS20;RAPGEF5;RPL39
Chlorpropamide-269_mg/kg_in_CMC-Rat-Heart-5d-up	15/302	3,93302E-05	0,005162746	0	0	3,413668328	34,62660472	RPL32;RPS8;RPS5;RPS6;RPL35A;RPSA;RPS3A;TOMM20;EEF1B2;RPS17;RPS19;ARL2BP;FUNDC1;RPS20;TPT1
Mestranol-250_mg/kg_in_CMC-Rat-Liver-5d-up	15/302	3,93302E-05	0,005078111	0	0	3,413668328	34,62660472	RPL32;RPS8;FEM1B;RPS5;RPS6;RPSA;RPS3A;RPL10A;SEPP1;RPS17;RPS19;ARL2BP;RPS20;RPL39;TPT1
4-Nitrobenzoic_Acid-1470_mg/kg_in_CMC-Rat-Kidney-5d-up	15/303	4,08458E-05	0,005188728	0	0	3,402402096	34,38368093	RPL32;RPS8;RPS5;RPS6;RPSA;RPS3A;RPL10A;EEF1B2;RPS17;RPS19;CLP1;RPS20;RAPGEF5;RPL39;TPT1
Cadmium_Chloride-66_mg/kg_in_Corn_Oil-Rat-Kidney-3d-up	15/303	4,08458E-05	0,005106368	0	0	3,402402096	34,38368093	RPL32;RPS8;RPS5;RPS6;RPL35A;RPSA;RPS3A;RPL10A;TOMM20;RPS17;CXCL11;RPS19;ARL2BP;RAPGEF5;TPT1
Bacitracin-380_mg/kg_in_Corn_Oil-Rat-Kidney-3d-up	15/304	4,24125E-05	0,005219382	0	0	3,391209984	34,14293471	C1QA;RPL32;RPS8;RPS5;RPS6;RPL35A;RPSA;RPS3A;RPL10A;EEF1B2;RPS17;CXCL11;RPS19;RPS20;RPL39
Leflunomide-30_mg/kg_in_Corn_Oil-Rat-Liver-5d-dn	15/304	4,24125E-05	0,005139084	0	0	3,391209984	34,14293471	C1QA;RPL32;RPS8;RPS5;RPS6;RPL35A;RPSA;RPS3A;RPL10A;SEPP1;EEF1B2;RPS17;RPS19;RPS20;RPL39
Rapamycin-60_mg/kg_in_CMC-Rat-Heart-1d-up	15/306	4,57052E-05	0,005454154	0	0	3,369045213	33,66787407	RPL32;RPS8;RPS6;RPSA;RPS3A;RPL10A;EEF1B2;RPS17;RPS19;ARL2BP;FUNDC1;ARL6IP5;RPS20;RPL39;TPT1
Modafinil-17.5_mg/kg_in_Saline-Rat-Liver-0.25d-up	14/271	4,65171E-05	0,005468186	0	0	3,550550969	35,41920001	RPL32;RPS8;RPS5;RPS6;RPL35A;RPSA;RPS3A;RPL10A;SEPP1;EEF1B2;RPS17;RPS19;RPL39;TPT1
Carbon_Tetrachloride-1175_mg/kg_in_Corn_Oil-Rat-Liver-0.25d-up	15/307	4,74343E-05	0,005494008	0	0	3,358071124	33,43351011	RPL32;RPS8;RPS5;RPS6;RPL35A;RPSA;RPS3A;RPL10A;PDRG1;EEF1B2;RPS17;RPS19;RPS20;RPL39;TPT1
Gemcitabine-11_mg/kg_in_Saline-Rat-Bone_marrow-3d-up	16/344	4,80474E-05	0,005484363	0	0	3,196675458	31,78557722	C1QA;RPL32;RPS8;RPS5;RPS6;RPL35A;RPSA;RPS3A;RPL10A;SEPP1;EEF1B2;RPS17;RPS19;ARL2BP;COL5A3;RPL39
Gentamicin-40_mg/kg_in_Saline-Rat-Kidney-14d-up	15/309	5,10658E-05	0,00574563	0	0	3,336336036	32,97099377	C1QA;RPL32;RPS8;RPS5;RPS6;RPS3A;RPL10A;EEF1B2;RPS17;CXCL11;RPS19;RPS20;RPL39;GOLGA7;TPT1
Itraconazole-30_mg/kg_in_Corn_Oil-Rat-Liver-0.25d-up	15/309	5,10658E-05	0,005664705	0	0	3,336336036	32,97099377	RPL32;RPS8;RPS5;RPS6;RPSA;RPS3A;RPL10A;TOMM20;EEF1B2;RPS17;RPS19;ARL6IP5;RPS20;RPL39;TPT1
Pergolide-1.1_mg/kg_in_CMC-Rat-Liver-5d-up	15/309	5,10658E-05	0,005586029	0	0	3,336336036	32,97099377	C1QA;RPL32;RPS8;RPS5;RPS6;RPL35A;RPSA;RPS3A;RPL10A;SEPP1;RPS17;RPS19;RPS20;RPL39;TPT1
Lead_(II)_Acetate-600_mg/kg_in_Saline-Rat-Liver-0.25d-up	15/310	5,29714E-05	0,005715108	0	0	3,325573661	32,74279397	C1QA;RPL32;RPS8;RPS5;RPS6;RPL35A;RPSA;RPS3A;RPL10A;SEPP1;EEF1B2;RPS17;RPS19;RPS20;RPL39
Chlorzoxazone-763_mg/kg_in_CMC-Rat-Liver-1d-up	15/311	5,49392E-05	0,005847313	0	0	3,314880499	32,51660205	RPL32;RPS8;RPS5;RPS6;RPL13A;RPL35A;RPSA;RPS3A;RPL10A;EEF1B2;RPS17;RPS19;RPS20;RPL39;TPT1
Propylene_Glycol-2000_mg/kg_in_Water-Rat-Liver-1d-up	15/311	5,49392E-05	0,005769349	0	0	3,314880499	32,51660205	C1QA;RPL32;RPS8;RPS5;RPS6;RPL35A;RPSA;RPS3A;RPL10A;EEF1B2;RPS17;RPS19;RPS20;RPL39;TPT1
Enoxacin-750_mg/kg_in_Corn_Oil-Rat-Liver-5d-up	13/241	5,58337E-05	0,005786132	0	0	3,707347678	36,30655031	C1QA;RPL32;RPS8;RPS6;RPL35A;RPS3A;EEF1B2;RPS17;RPS19;ARL2BP;RPS20;RPL39;TPT1
Granisetron-175_mg/kg_in_Water-Rat-Liver-3d-up	14/276	5,66793E-05	0,005797484	0	0	3,486229394	34,08870424	RPL32;RPS8;RPS5;RPS6;RPL35A;RPSA;RPS3A;RPL10A;BMP3;RPS17;RPS19;RPS20;RPL39;TPT1
Diethylstilbestrol-2.8_mg/kg_in_Corn_Oil-Rat-Spleen-0.25d-up	16/349	5,70147E-05	0,005757022	0	0	3,150877815	30,79101326	C1QA;RPL32;RPS8;RPL13A;RPL35A;RPSA;RPS3A;RPL10A;SEPP1;EEF1B2;RPS17;RPS19;ARL6IP5;RPS20;RPL39;TPT1
Fenofibrate-215_mg/kg_in_Corn_Oil-Rat-Kidney-5d-up	16/349	5,70147E-05	0,005684148	0	0	3,150877815	30,79101326	RPL32;RPS8;RPS5;RPS6;RPL35A;RPSA;RPS3A;RPL10A;EEF1B2;RPS17;RPS19;ARL2BP;RPS20;RPL39;GOLGA7;TPT1
Gentamicin-267_mg/kg_in_Corn_Oil-Rat-Kidney-1d-up	16/350	5,89753E-05	0,005806122	0	0	3,141875307	30,59681083	C1QA;RPL32;RPS8;RPS5;RPS6;RPL35A;RPSA;RPS3A;SEPP1;EEF1B2;RPS17;RPS19;ARL6IP5;RPS20;RPL39;TPT1
Vinorelbine-1.5_mg/kg_in_Saline-Rat-Heart-1d-dn	16/351	6,09951E-05	0,005930833	0	0	3,132924095	30,40414128	RPL32;RPS8;RPS6;RPSA;RPS3A;RPL10A;SEPP1;EEF1B2;RPS17;RPS19;ARL2BP;FUNDC1;ARL6IP5;RPS20;RPL39;TPT1
Idarubicin-4_mg/kg_in_Saline-Rat-Kidney-14d-up	15/314	6,12329E-05	0,005881343	0	0	3,283209666	31,84984652	RPL32;RPS8;RPS6;RPSA;RPS3A;RPL10A;SEPP1;EEF1B2;RPS17;CXCL11;RPS19;RPS20;RAPGEF5;RPL39;GOLGA7
4-Chloro-2-Nitroaniline-1200_mg/kg_in_CMC-Rat-Kidney-5d-up	15/315	6,34669E-05	0,006022469	0	0	3,272786778	31,6314607	RPL32;RPS8;RPS5;RPSA;RPS3A;TOMM20;EEF1B2;RPS17;CXCL11;RPS19;ARL6IP5;RPS20;RAPGEF5;RPL39;TPT1
Sumatriptan-1100_mg/kg_in_Water-Rat-Brain-5d-up	15/315	6,34669E-05	0,005950773	0	0	3,272786778	31,6314607	RPL32;RPS8;RPS5;RPS6;RPL13A;RPL35A;RPSA;RPS3A;EEF1B2;RPS17;RPS19;ARL2BP;FUNDC1;ARL6IP5;TPT1
Venlafaxine-320_mg/kg_in_Water-Rat-Heart-3d-up	15/315	6,34669E-05	0,005880764	0	0	3,272786778	31,6314607	RPL32;RPS8;RPS6;RPSA;RPS3A;RPL10A;SEPP1;TOMM20;RPS17;RPS19;RPS20;RAPGEF5;RPL39;GOLGA7;TPT1
Cisplatin-63_uM_in_DMSO-Rat-Primary_rat_hepatocytes-1d-up	14/279	6,36656E-05	0,005830581	0	0	3,448743056	33,32129615	RPL32;RPS8;RPS5;RPS6;RPL35A;RPSA;PDRG1;EEF1B2;RPS17;CXCL11;ARL2BP;CLP1;RPS20;GOLGA7
4-Octylphenol-700_mg/kg_in_Corn_Oil-Rat-Kidney-3d-up	15/316	6,57719E-05	0,005954251	0	0	3,262429858	31,41497165	RPL32;RPS8;RPS5;RPS6;RPSA;RPS3A;RPL10A;EEF1B2;RPS17;CXCL11;RPS19;ARL6IP5;RPS20;RPL39;TPT1

Fenofibrate-250_uM_in_DMSO-Rat-Primary_rat_hepatocytes-1d-dn	15/316	6,57719E-05	0,005886589	0	0	3,262429858	31,41497165	RPL32;RPS8;RPS5;RPS6;RPL35A;RPSA;RPS3A;RPL10A;SEPP1;EEF1B2;RPS17;CXCL11;ARL2BP;ARL6IP5;RPS20
Ketoprofen-4_mg/kg_in_Corn_Oil-Rat-Kidney-5d-up	15/316	6,57719E-05	0,005820448	0	0	3,262429858	31,41497165	C1QA;RPL32;RPS8;RPS5;RPS6;RPL35A;RPSA;RPS3A;RPL10A;PDRG1;RPS17;CXCL11;RPS19;RPS20;RPL39
Modafinil-17.5_mg/kg_in_Saline-Rat-Liver-3d-up	14/281	6,87303E-05	0,006014668	0	0	3,424196842	32,82202209	RPL32;RPS8;RPS5;RPS6;RPL35A;RPSA;RPS3A;RPL10A;EEF1B2;RPS17;RPS19;RPS20;RPL39;TPT1
Cyclosporin_A-70_mg/kg_in_Corn_Oil-Rat-Kidney-3d-up	15/318	7,06032E-05	0,00611067	0	0	3,241911431	30,98759904	C1QA;RPL32;RPS8;RPS5;RPS6;RPL35A;RPSA;RPS3A;RPL10A;RPS17;RPS19;RPS20;RPL39;GOLGA7;TPT1
DActinomycin-0.06_mg/kg_in_Saline-Rat-Bone_marrow-3d-up	14/282	7,1392E-05	0,006111774	0	0	3,4120543	32,57599162	C1QA;RPL32;RPS5;NKG7;RPL35A;RPSA;RPS3A;RPL10A;SEPP1;EEF1B2;RPS17;RPS19;ARL6IP5;RPL39
Bithionol-333_mg/kg_in_Corn_Oil-Rat-Spleen-5d-up	15/319	7,31333E-05	0,006193529	0	0	3,231748699	30,7766738	C1QA;RPL32;RPS8;RPS5;NKG7;RPL35A;RPSA;RPS3A;RPL10A;EEF1B2;RPS17;CXCL11;RPS20;RPL39;TPT1
Catechol-40_mg/kg_in_Saline-Rat-Kidney-0.25d-dn	15/319	7,31333E-05	0,00612764	0	0	3,231748699	30,7766738	RPL32;RPS8;RPS6;RPL35A;RPSA;RPS3A;RPL10A;SEPP1;EEF1B2;RPS17;R3HCC1;ARL6IP5;RPS20;RPL39;TPT1
Penicillin_G-243_mg/kg_in_Saline-Rat-Kidney-3d-up	15/319	7,31333E-05	0,006063139	0	0	3,231748699	30,7766738	RPL32;RPS8;RPS5;RPS6;RPL13A;RPL35A;RPSA;RPL10A;SEPP1;TOMM20;EEF1B2;RPS17;RPS19;ARL6IP5;RPS20
Stavudine-1400_mg/kg_in_Water-Rat-Liver-3d-up	15/319	7,31333E-05	0,005999981	0	0	3,231748699	30,7766738	RPL32;RPS8;FEM1B;RPS5;RPS6;RPL35A;RPSA;RPS3A;RPL10A;EEF1B2;RPS17;RPS19;RPS20;RPL39;TPT1
Chloroform-600_mg/kg_in_Corn_Oil-Rat-Liver-5d-up	14/283	7,4143E-05	0,00602011	0	0	3,399997571	32,3323254	RPL32;RPS8;RPS5;RPS6;RPSA;RPS3A;RPL10A;EEF1B2;RPS17;RPS19;ARL2BP;RPS20;RPL39;TPT1
Naproxen-134_mg/kg_in_Corn_Oil-Rat-Liver-1d-up	14/283	7,4143E-05	0,00595868	0	0	3,399997571	32,3323254	C1QA;RPL32;RPS8;RPS5;RPS6;RPL13A;RPL35A;RPS3A;EEF1B2;RPS17;RPS19;RPS20;RPL39;TPT1
N-Nitrosodiethylamine-1.67_mg/kg_in_Saline-Rat-Spleen-3d-dn	13/248	7,47253E-05	0,005944816	0	0	3,6027048	34,23178908	C1QA;RPL32;RPS8;RPS5;RPS6;RPL13A;RPL35A;RPSA;RPS3A;RPL10A;RPS17;RPL39;TPT1
Mycophenolate_Mofetil-63_mg/kg_in_CMC-Rat-Bone_marrow-3d-up	15/321	7,84327E-05	0,006177363	0	0	3,211613193	30,36024345	C1QA;RPL32;RPS8;RPS6;NKG7;RPL35A;RPSA;RPS3A;EEF1B2;RPS17;RPS19;ARL2BP;ARL6IP5;RPS20;RPL39
Thioguanine-12_mg/kg_in_Corn_Oil-Rat-Spleen-0.25d-up	14/285	7,99236E-05	0,006232459	0	0	3,376137939	31,85196818	C1QA;RPL32;RPS8;RPS5;RPS6;RPL35A;RPSA;RPS3A;RPL10A;SEPP1;EEF1B2;RAPGEF5;RPL39;TPT1
Beta-Estradiol-150_mg/kg_in_Corn_Oil-Rat-Liver-5d-up	15/322	8,12062E-05	0,006270395	0	0	3,201639239	30,15469839	RPL32;RPS8;FEM1B;RPS5;RPS6;RPSA;RPS3A;RPL10A;EEF1B2;RPS17;RPS19;ARL6IP5;RPS20;RPL39;TPT1
Imatinib-150_mg/kg_in_Water-Rat-Liver-1d-dn	15/322	8,12062E-05	0,006209518	0	0	3,201639239	30,15469839	C1QA;RPL32;RPS8;RPS5;RPS6;RPL35A;RPSA;RPS3A;RPL10A;SEPP1;EEF1B2;RPS17;RPS19;RPS20;RPL39
6-Methoxy-2-Naphthylacetic_Acid-360_mg/kg_in_Saline-Rat-Kidney-3d-up	14/287	8,60928E-05	0,006519877	0	0	3,352610846	31,38071992	C1QA;RPL32;RPS5;RPS6;RPL13A;RPSA;RPS3A;RPL10A;SEPP1;RPS17;RPS19;RPS20;RPL39;TPT1
Chloroform-600_mg/kg_in_Corn_Oil-Rat-Liver-3d-up	14/287	8,60928E-05	0,006457783	0	0	3,352610846	31,38071992	RPL32;RPS8;FEM1B;RPS5;RPS6;RPSA;RPS3A;RPL10A;SEPP1;EEF1B2;RPS19;RPS20;RPL39;TPT1
Geraniol-1500_mg/kg_in_CMC-Rat-Liver-1d-up	14/287	8,60928E-05	0,006396861	0	0	3,352610846	31,38071992	RPL32;RPS8;RPS5;RPS6;RPL35A;RPSA;RPS3A;RPL10A;SEPP1;EEF1B2;RPS17;RPS19;RPS20;RPL39
Urethane-453_mg/kg_in_Water-Rat-Bone_marrow-5d-up	14/287	8,60928E-05	0,006337077	0	0	3,352610846	31,38071992	C1QA;RPL32;RPS8;RPS5;RPL13A;RPL35A;RPSA;RPS3A;RPL10A;EEF1B2;RPS17;RPS19;RPS20;TPT1
Tetracaine-24_mg/kg_in_Corn_Oil-Rat-Heart-3d-up	15/324	8,70116E-05	0,006345404	0	0	3,181876034	29,7488507	C1QA;RPL32;RPS8;RPS5;RPS6;RPL35A;RPSA;RPS3A;RPL10A;EEF1B2;RPS17;RPS19;ARL6IP5;RPS20;TPT1
Indomethacin-10_mg/kg_in_Corn_Oil-Rat-Liver-1d-up	14/288	8,933E-05	0,006454705	0	0	3,340969836	31,14844157	C1QA;RPL32;RPS8;RPS5;RPS6;RPSA;RPL10A;SEPP1;EEF1B2;RPS17;ARL2BP;RPS20;RPL39;TPT1
Lipopolysaccharide_E._Coli_O55:B5-1.25_mg/kg_in_Saline-Rat-Liver-0.25d-up	14/288	8,933E-05	0,006396026	0	0	3,340969836	31,14844157	RPL32;RPS8;RPS6;RPSA;RPS3A;RPL10A;EEF1B2;RPS17;CXCL11;RPS19;ARL2BP;RPS20;RPL39;TPT1
Lomustine-8.75_mg/kg_in_Corn_Oil-Rat-Spleen-1d-dn	15/325	9,0048E-05	0,006389352	0	0	3,172085646	29,54850975	C1QA;RPL32;RPS5;RPL13A;RPL35A;RPSA;RPS3A;RPL10A;SEPP1;TOMM20;EEF1B2;RPS17;RPS19;RPL39;TPT1
Roflumilast-17_mg/kg_in_CMC-Rat-Heart-3d-up	14/289	9,26725E-05	0,006516861	0	0	3,329409387	30,91835725	C1QA;RPS8;RPS6;RPSA;RPS3A;RPL10A;EEF1B2;RPS17;RPS19;ARL2BP;RPS20;RAPGEF5;RPL39;TPT1
Benzyl_Acetate-1868_mg/kg_in_CMC-Rat-Kidney-5d-up	15/326	9,31765E-05	0,006494323	0	0	3,162355322	29,34986564	RPL32;RPS8;RPS5;RPSA;RPS3A;RPL10A;TOMM20;EEF1B2;RPS17;CXCL11;RPS19;ARL6IP5;RPS20;RPL39;TPT1
Carbon_Tetrachloride-3178_mg/kg_in_Saline-Rat-Liver-1d-up	15/326	9,31765E-05	0,006437355	0	0	3,162355322	29,34986564	RPL32;RPS8;RPS5;RPS6;RPSA;RPS3A;RPL10A;PDRG1;EEF1B2;RPS17;RPS19;RPS20;RPL39;GOLGA7;TPT1
Guanethidine-132_mg/kg_in_CMC-Rat-Heart-3d-up	15/326	9,31765E-05	0,006381378	0	0	3,162355322	29,34986564	RPL32;RPS8;RPS6;RPL35A;RPSA;RPS3A;RPL10A;EEF1B2;RPS17;CXCL11;RPS19;ARL6IP5;RPS20;RAPGEF5;TPT1
Caffeine-93_mg/kg_in_Water-Rat-Brain-3d-up	13/254	9,51034E-05	0,00645719	0	0	3,517601537	32,57491198	RPL32;RPS8;RPS5;RPS6;RPL35A;RPSA;RPS3A;RPL10A;EEF1B2;RPS17;RPS19;ARL2BP;TPT1
Mitomycin_C-1.7_mg/kg_in_Saline-Rat-Bone_marrow-1d-dn	14/290	9,61232E-05	0,006470655	0	0	3,317928665	30,69044009	RPL32;RPS8;RPS5;RPS6;RPL35A;RPSA;RPS3A;RPL10A;EEF1B2;RPS17;RPS19;RPS20;RPL39;TPT1
Nimesulide-162_mg/kg_in_Corn_Oil-Rat-Liver-5d-up	14/290	9,61232E-05	0,006415819	0	0	3,317928665	30,69044009	RPL32;RPS8;RPS5;RPS6;RPL35A;RPSA;RPS3A;RPL10A;TOMM20;EEF1B2;RPS17;RPS19;RPS20;RPL39
Beta-Estradiol_3-Benzoate-25_mg/kg_in_Corn_Oil-Rat-Liver-5d-up	14/291	9,96852E-05	0,006597651	0	0	3,306526848	30,46466366	RPL32;RPS8;FEM1B;RPS5;RPS6;RPSA;RPS3A;RPL10A;EEF1B2;RPS19;ARL2BP;RPS20;RPL39;TPT1
Ethinylestradiol-10_mg/kg_in_Corn_Oil-Rat-Liver-0.25d-up	14/291	9,96852E-05	0,006542671	0	0	3,306526848	30,46466366	RPL32;RPS8;FEM1B;RPS5;RPS6;RPSA;RPS3A;RPL10A;SEPP1;EEF1B2;RPS17;RPS19;RPS20;RPL39
Sodium_Arsenite-10_mg/kg_in_Saline-Rat-Kidney-0.25d-up	14/291	9,96852E-05	0,006488599	0	0	3,306526848	30,46466366	RPL32;RPS8;RPS5;RPL13A;RPL35A;RPS3A;SEPP1;TOMM20;EEF1B2;RPS17;CXCL11;RPS20;RPL39;TPT1
Idarubicin-1.5_uM_in_DMSO-Rat-Primary_rat_hepatocytes-0.67d-dn	12/222	0,000105427	0,006806103	0	0	3,715055261	34,02058154	RPS17;CXADR;ARL2BP;RPS8;RPS6;RPS20;RPSA;RPS3A;RPL39;SLC16A3;TPT1;PDRG1
Benzyl_Acetate-1868_mg/kg_in_CMC-Rat-Kidney-3d-up	15/330	0,00010666	0,006829722	0	0	3,124023743	28,57189118	RPL32;RPS8;RPS5;RPS6;RPSA;RPS3A;RPL10A;SEPP1;EEF1B2;RPS17;CXCL11;RPS19;RPS20;RPL39;TPT1
Norethindrone-75_mg/kg_in_Corn_Oil-Rat-Liver-5d-up	15/330	0,00010666	0,006774644	0	0	3,124023743	28,57189118	RPL32;RPS8;FEM1B;RPS5;RPS6;RPL13A;RPL35A;RPSA;RPS3A;RPL10A;EEF1B2;RPS17;RPS19;RPS20;RPL39
Lovastatin-450_mg/kg_in_Corn_Oil-Rat-Liver-1d-dn	14/293	0,000107155	0,006751598	0	0	3,283956699	30,01942923	C1QA;RPL32;RPS8;RPS6;RPL35A;RPSA;RPS3A;RPL10A;SEPP1;EEF1B2;RPS17;CLP1;RPS20;RPL39;TPT1
Oxcarbazepine-1600_mg/kg_in_CMC-Rat-Brain-3d-dn	16/369	0,000109348	0,00683513	0	0	2,98009853	27,18139915	C1QA;RPL32;RPS8;RPSA;RPS3A;RPL10A;SEPP1;TOMM20;EEF1B2;RPS17;RPS19;CLP1;FUNDC1;RPS20;RPL39;TPT1
Dicyclomine-645_mg/kg_in_Water-Rat-Heart-3d-dn	15/331	0,000110286	0,006839459	0	0	3,114585604	28,38145866	RPL32;RPS8;RPS5;RPS6;RPL35A;RPSA;RPS3A;EEF1B2;RPS17;RPS19;COL5A3;ARL6IP5;RPS20;RPL39;TPT1
Camptothecin-0.24_mg/kg_in_Saline-Rat-Bone_marrow-1d-up	14/294	0,000111068	0,006834168	0	0	3,272786778	29,79992036	C1QA;RPL32;FEM1B;RPS5;RPS6;NKG7;RPL35A;RPSA;RPL10A;RPS17;RPS19;RPS20;RPL39;TPT1
Trimethadione-535_mg/kg_in_Water-Rat-Kidney-3d-dn	12/224	0,000114746	0,007005757	0	0	3,681885125	33,40495615	RPS17;RPL32;RPS19;RPS8;RPS5;RPS6;RPL35A;RPS20;RPSA;RPS3A;RPL10A;RPL39
Dicumarol-5_mg/kg_in_Corn_Oil-Rat-Liver-0.25d-up	14/295	0,000115105	0,006973616	0	0	3,261692585	29,58245043	C1QA;RPL32;FEM1B;RPS6;RPL35A;RPSA;RPS3A;RPL10A;SEPP1;EEF1B2;RPS17;RPS19;RPL39;TPT1
Cyclosporin_A-350_mg/kg_in_Corn_Oil-Rat-Kidney-3d-up	15/333	0,000117861	0,007086042	0	0	3,095879385	28,00534512	C1QA;RPL32;RPS8;RPS5;RPS6;RPL35A;RPSA;RPS3A;RPL10A;EEF1B2;RPS17;RPS19;RPS20;RAPGEF5;RPL39
Hydrazine-45_mg/kg_in_Water-Rat-Liver-3d-up	15/333	0,000117861	0,007032359	0	0	3,095879385	28,00534512	C1QA;RPL32;RPS8;RPS5;RPS6;RPL35A;RPSA;RPS3A;RPL10A;SEPP1;RPS17;RPS19;RPS20;RPL39;TPT1
Irinotecan-5_mg/kg_in_Saline-Rat-Kidney-1d-dn	15/333	0,000117861	0,006979485	0	0	3,095879385	28,00534512	RPL32;RPS8;RPS5;RPS6;RPL13A;RPL35A;RPSA;RPS3A;EEF1B2;RPS17;RPS19;ARL2BP;RPS20;RPL39;TPT1
Pralidoxime_Chloride-48_mg/kg_in_Saline-Rat-Heart-1d-dn	15/333	0,000117861	0,006927399	0	0	3,095879385	28,00534512	RPL32;RPS8;RPS5;RPL35A;RPSA;RPS3A;SEPP1;TOMM20;EEF1B2;RPS17;RPS19;FUNDC1;ARL6IP5;RPS20;TPT1
Doxorubicin-3_mg/kg_in_Saline-Rat-Heart-5d-up	14/296	0,000119269	0,006958259	0	0	3,250673354	29,36699496	RPL32;RPS6;RPSA;RPS3A;RPL10A;SEPP1;EEF1B2;RPS17;CXCL11;RPS19;FUNDC1;RPS20;RPL39;TPT1
Lead_(II)_Acetate-600_mg/kg_in_Saline-Rat-Spleen-5d-up	14/296	0,000119269	0,006907095	0	0	3,250673354	29,36699496	C1QA;RPL32;RPS8;RPS5;RPS6;RPSA;RPS3A;SEPP1;EEF1B2;RPS17;RPS19;RPS20;RAPGEF5;RPL39
Leflunomide-30_mg/kg_in_Corn_Oil-Rat-Spleen-0.25d-up	14/296	0,000119269	0,006856678	0	0	3,250673354	29,36699496	C1QA;RPL32;RPS8;RPSA;RPS3A;RPL10A;SEPP1;EEF1B2;CXCL11;CLP1;FUNDC1;RPS20;RPL39;TPT1
Gatifloxacin-770_mg/kg_in_Corn_Oil-Rat-Heart-3d-dn	14/297	0,000123563	0,007052058	0	0	3,239728326	29,15352983	RPL32;RPS8;RPS6;RPSA;RPS3A;RPL10A;SEPP1;EEF1B2;RPS17;RPS19;ARL2BP;FUNDC1;RPL39;TPT1
Betahistine-1500_mg/kg_in_Water-Rat-Kidney-5d-up	15/335	0,000125885	0,007132886	0	0	3,077396523	27,63545442	C1QA;RPL32;RPS6;RPL35A;RPSA;RPS3A;RPL10A;PDRG1;EEF1B2;RPS17;RPS19;ARL2BP;RPS20;RAPGEF5;TPT1
Piclamilast-17_mg/kg_in_CMC-Rat-Heart-5d-up	15/335	0,000125885	0,007081936	0	0	3,077396523	27,63545442	RPL32;RPS8;RPL35A;RPSA;RPS3A;RPL10A;TOMM20;EEF1B2;RPS17;ARL2BP;ARL6IP5;RPS20;RAPGEF5;RPL39;TPT1
Bezafibrate-617_mg/kg_in_Corn_Oil-Rat-Skeletal_muscle-3d-dn	14/298	0,000127991	0,007149318	0	0	3,228856754	28,94203129	RPL32;FEM1B;RPS6;RPSA;RPS3A;RPL10A;EEF1B2;RPS17;RPS19;COL5A3;ARL6IP5;RPL39;SLC16A3;TPT1
Catechol-195_mg/kg_in_Saline-Rat-Liver-5d-dn	14/298	0,000127991	0,007098971	0	0	3,228856754	28,94203129	C1QA;RPL32;RPS8;RPS5;RPS6;RPS3A;SEPP1;EEF1B2;RPS17;RPS19;ARL6IP5;RPS20;RPL39;TPT1
Hydrazine-45_mg/kg_in_Water-Rat-Liver-1d-up	14/298	0,000127991	0,007049328	0	0	3,228856754	28,94203129	RPL32;RPS8;FEM1B;RPS5;RPS6;RPL35A;RPSA;RPS3A;RPL10A;SEPP1;RPS17;RPS19;RPS20;RPL39
Allyl_Alcohol-25_mg/kg_in_Saline-Rat-Kidney-3d-up	14/299	0,000132555	0,007250012	0	0	3,218057902	28,73247593	C1QA;RPL32;RPS8;RPS5;RPL13A;RPL35A;RPSA;RPS3A;SEPP1;TOMM20;RPS17;RPS20;RPL39;TPT1
Eucalyptol-930_mg/kg_in_Corn_Oil-Rat-Kidney-5d-up	15/337	0,000134381	0,007299207	0	0	3,059133042	27,27165471	RPL32;RPS8;RPS5;RPS6;RPL13A;RPL35A;RPSA;RPS3A;EEF1B2;RPS17;RPS19;ARL2BP;ARL6IP5;RPS20;RPL39
Monocrotaline-333_uM_in_DMSO-Rat-Primary_rat_hepatocytes-0.67d-up	15/337	0,000134381	0,007249213	0	0	3,059133042	27,27165471	RPL32;RPS8;RPS5;RPL35A;RPSA;RPS3A;TOMM20;PDRG1;EEF1B2;RPS17;RPS19;ARL2BP;RPS20;RPL39;GOLGA7
Papaverine-69_mg/kg_in_CMC-Rat-Heart-1d-dn	15/337	0,000134381	0,007199898	0	0	3,059133042	27,27165471	RPL32;RPS8;RPS6;RPL35A;RPSA;RPS3A;RPL10A;EEF1B2;RPS17;RPS19;ARL2BP;ARL6IP5;RPS20;RPL39;TPT1
Tenidap-75_mg/kg_in_Corn_Oil-Rat-Kidney-0.25d-up	15/337	0,000134381	0,007151251	0	0	3,059133042	27,27165471	RPL32;RPS8;RPS5;RPS6;RPL13A;RPL35A;RPSA;RPS3A;RPL10A;RPS17;RPS19;ARL6IP5;RPS20;RPL39;TPT1
Trimethadione-535_mg/kg_in_Water-Rat-Heart-1d-dn	15/337	0,000134381	0,007103256	0	0	3,059133042	27,27165471	RPL32;RPS8;RPS5;RPS6;RPL35A;RPSA;RPS3A;RPL10A;TOMM20;EEF1B2;RPS17;RPS19;FUNDC1;RPS20;RPL39
2-Amino-4-Nitrophenol-625_mg/kg_in_CMC-Rat-Kidney-1d-up	14/300	0,000137259	0,007207035	0	0	3,207331042	28,52484071	RPL32;RPS8;RPS6;RPSA;RPS3A;RPL10A;EEF1B2;RPS17;CXCL11;RPS19;ARL6IP5;RPS20;RPL39;TPT1
Cytarabine-487_mg/kg_in_Saline-Rat-Liver-0.25d-up	14/300	0,000137259	0,007159306	0	0	3,207331042	28,52484071	C1QA;RPL32;RPS8;RPS5;RPS6;RPL35A;RPSA;RPS3A;RPL10A;TOMM20;EEF1B2;RPS17;RPL39;TPT1
Enrofloxacin-2000_mg/kg_in_CMC-Rat-Kidney-5d-up	14/300	0,000137259	0,007112205	0	0	3,207331042	28,52484071	RPL32;RPS8;RPS5;RPS6;RPL13A;RPL35A;RPSA;RPS3A;RPL10A;EEF1B2;RPS17;RPS19;RPL39;TPT1
Geraniol-1500_mg/kg_in_CMC-Rat-Liver-3d-up	14/300	0,000137259	0,00706572	0	0	3,207331042	28,52484071	RPL32;RPS8;RPS5;RPS6;RPL13A;RPL35A;RPSA;RPS3A;RPL10A;EEF1B2;RPS17;RPS19;RPS20;RPL39
Methotrexate-0.3_mg/kg_in_Corn_Oil-Rat-Liver-5d-up	14/300	0,000137259	0,007019839	0	0	3,207331042	28,52484071	RPL32;RPS8;FEM1B;RPS5;RPS6;RPSA;RPS3A;RPL10A;SEPP1;EEF1B2;RPS17;RPS19;RPS20;RPL39
Modafinil-325_mg/kg_in_Saline-Rat-Liver-3d-up	14/300	0,000137259	0,00697455	0	0	3,207331042	28,52484071	RPL32;RPS8;RPS5;RPS6;RPL13A;RPL35A;RPSA;RPS3A;RPL10A;EEF1B2;RPS17;RPS19;RPS20;RPL39
Neomycin-877_mg/kg_in_Corn_Oil-Rat-Kidney-1d-dn	13/264	0,000139796	0,007057895	0	0	3,384359054	30,03729643	RPL32;RPS8;RPL13A;RPL35A;RPSA;RPS3A;SEPP1;TOMM20;EEF1B2;RPS17;RPS19;ARL2BP;TPT1
Carbon_Tetrachloride-400_mg/kg_in_Corn_Oil-Rat-Bone_marrow-3d-up	14/301	0,000142108	0,007128933	0	0	3,196675458	28,31910291	C1QA;RPL32;RPS8;FEM1B;RPS5;RPS6;RPL13A;RPL35A;RPS3A;RPS17;FUNDC1;RPS20;RPL39;TPT1
Mycophenolate_Mofetil-63_mg/kg_in_CMC-Rat-Bone_marrow-1d-up	14/301	0,000142108	0,007083813	0	0	3,196675458	28,31910291	C1QA;RPL32;RPS6;NKG7;RPL35A;RPSA;SEPP1;EEF1B2;RPS19;FUNDC1;ARL6IP5;RPS20;RPL39;TPT1
Catechol-40_mg/kg_in_Saline-Rat-Spleen-0.25d-up	15/339	0,000143372	0,007101854	0	0	3,041085059	26,91381759	C1QA;RPL32;RPS5;RPS6;NKG7;RPL13A;RPL35A;RPSA;RPL10A;SEPP1;TOMM20;RPS17;RPS19;ARL6IP5;RPL39
Stavudine-1400_mg/kg_in_Water-Rat-Liver-1d-up	14/302	0,000147104	0,007241201	0	0	3,186090439	28,11524018	RPL32;RPS8;RPS5;RPS6;RPL35A;RPSA;RPS3A;RPL10A;EEF1B2;RPS17;RPS19;RPS20;RPL39;TPT1
Ibuprofen-263_mg/kg_in_Corn_Oil-Rat-Kidney-5d-up	15/340	0,00014806	0,007242976	0	0	3,032140691	26,73709582	C1QA;RPL32;RPS8;RPS5;RPS6;RPL35A;RPSA;RPS3A;RPL10A;EEF1B2;RPS17;CXCL11;RPS19;RPS20;RPL39
Vecuronium_Bromide-0.05_mg/kg_in_Saline-Rat-Liver-1d-dn	15/340	0,00014806	0,007198266	0	0	3,032140691	26,73709582	RPL32;RPS8;RPS5;RPS6;RPL35A;RPSA;RPS3A;RPL10A;SEPP1;EEF1B2;RPS17;RPS19;RPS20;RPL39;TPT1
NN-Dimethylformamide-1400_mg/kg_in_Saline-Rat-Bone_marrow-3d-up	16/379	0,000148633	0,007181795	0	0	2,901467961	25,5736292	C1QA;RPL32;RPS8;FEM1B;RPS5;RPS6;NKG7;RPSA;RPS3A;RPL10A;SEPP1;RPS17;RPS19;RPS20;RPL39;GOLGA7
Zidovudine-14800_uM_in_DMSO-Rat-Primary_rat_hepatocytes-1d-up	13/266	0,000150632	0,007234013	0	0	3,358912746	29,56068367	RPL32;RPS8;FEM1B;RPS5;RPL35A;RPSA;RPS3A;PDRG1;EEF1B2;RPS17;ARL2BP;CLP1;RPS20
Acyclovir-980_mg/kg_in_Saline-Rat-Spleen-3d-dn	14/303	0,000152252	0,007267478	0	0	3,175575289	27,91323046	RPL32;RPS8;RPS5;RPL13A;RPL35A;RPSA;RPS3A;RPL10A;EEF1B2;RPS17;ARL2BP;ARL6IP5;RPS20;TPT1
Interleukin_6-0.125_mg/kg_in_RSA/Saline-Rat-Liver-1d-up	14/303	0,000152252	0,007223698	0	0	3,175575289	27,91323046	C1QA;RPL32;RPS8;RPS6;RPL35A;RPSA;RPS3A;SEPP1;EEF1B2;RPS17;RPS19;RPS20;RAPGEF5;RPL39
Nisoldipine-1125_mg/kg_in_Water-Rat-Liver-3d-dn	13/267	0,000156316	0,007372134	0	0	3,346332548	29,32601943	RPL32;RPS8;RPS5;RPS6;RPL35A;RPSA;RPS3A;RPL10A;SEPP1;EEF1B2;RPS17;RPS20;TPT1
Torsemide-3_mg/kg_in_Corn_Oil-Rat-Liver-1d-up	13/267	0,000156316	0,007328252	0	0	3,346332548	29,32601943	C1QA;RPL32;RPS8;RPS5;RPS6;RPL35A;RPS3A;SEPP1;RPS19;RPS20;RPL39;GOLGA7;TPT1
Eucalyptol-930_mg/kg_in_Corn_Oil-Rat-Kidney-1d-up	16/381	0,000157821	0,007355014	0	0	2,886237158	25,26626376	RPL32;RPS8;RPS6;RPL13A;RPL35A;RPSA;RPS3A;SEPP1;TOMM20;EEF1B2;RPS17;RPS19;ARL6IP5;RPS20;RPL39;TPT1
Acetazolamide-250_mg/kg_in_Corn_Oil-Rat-Kidney-5d-up	14/305	0,000163016	0,007552427	0	0	3,154751845	27,51468353	RPL32;RPS8;RPS5;RPS6;RPL35A;RPSA;RPS3A;EEF1B2;RPS17;RPS19;ARL2BP;RPS20;RAPGEF5;RPL39
Albendazole-62_mg/kg_in_Corn_Oil-Rat-Liver-0.25d-up	14/305	0,000163016	0,007508261	0	0	3,154751845	27,51468353	C1QA;RPL32;RPS5;RPS6;RPS3A;RPL10A;SEPP1;RPS17;CXCL11;RPS19;CLP1;RPS20;GOLGA7;TPT1
Amiodarone-147_mg/kg_in_CMC-Rat-Liver-5d-dn	14/305	0,000163016	0,007464608	0	0	3,154751845	27,51468353	RPL32;RPS8;RPS5;RPS6;RPL35A;RPS3A;SEPP1;EEF1B2;RPS17;RPS19;CLP1;RPS20;RPL39;TPT1
Clomipramine-7_uM_in_DMSO-Rat-Primary_rat_hepatocytes-1d-dn	14/305	0,000163016	0,00742146	0	0	3,154751845	27,51468353	RPL32;RPS6;RPSA;RPS3A;RPL10A;SEPP1;RPS17;CXCL11;RPS19;ARL6IP5;RPS20;RPL39;GOLGA7;TPT1
Clonidine-6_mg/kg_in_Water-Rat-Heart-5d-up	14/305	0,000163016	0,007378808	0	0	3,154751845	27,51468353	C1QA;RPL32;RPS5;RPS6;RPL35A;RPSA;RPS3A;RPL10A;RPS17;RPS19;RPS20;RAPGEF5;RPL39;TPT1
Lomustine-8.75_mg/kg_in_Corn_Oil-Rat-Bone_marrow-3d-up	14/305	0,000163016	0,007336643	0	0	3,154751845	27,51468353	C1QA;RPL32;RPS8;RPS5;RPS6;RPL35A;RPS3A;RPL10A;DUSP14;EEF1B2;RPS17;RPS20;RPL39;TPT1
Aspirin-375_mg/kg_in_Corn_Oil-Rat-Kidney-5d-up	15/344	0,000168171	0,007525658	0	0	2,996883241	26,04449896	RPL32;RPS8;RPS5;RPS6;RPL13A;RPL35A;RPSA;RPS3A;RPL10A;TOMM20;EEF1B2;RPS17;RPS19;RPS20;RPL39
Diclofenac-3.5_mg/kg_in_Corn_Oil-Rat-Liver-5d-dn	13/269	0,00016824	0,007486215	0	0	3,321452752	28,86381533	C1QA;RPL32;RPS8;RPS5;RPSA;RPS3A;RPL10A;EEF1B2;RPS17;RPS19;RPS20;RPL39;TPT1

Mitomycin_C-0.5_mg/kg_in_Saline-Rat-Bone_marrow-3d-dn	13/269	0,00016824	0,007444157	0	0	3,321452752	28,86381533	RPL32;RPS8;RPS6;RPSA;RPS3A;EEF1B2;RPS19;ARL2BP;FUNDC1;ARL6IP5;RPS20;RPL39;TPT1
Bromodichloromethane-430_mg/kg_in_CMC-Rat-Kidney-5d-up	14/306	0,00016864	0,007420174	0	0	3,144442198	27,31810385	RPL32;RPS8;RPS5;RPS6;RPSA;RPS3A;RPL10A;EEF1B2;RPS17;RPS19;ARL6IP5;RPS20;RPL39;TPT1
Raloxifene-5_mg/kg_in_Corn_Oil-Rat-Liver-3d-up	14/306	0,00016864	0,007378951	0	0	3,144442198	27,31810385	C1QA;CXADR;RPL32;RPS8;RPS5;RPS6;RPL13A;RPL35A;TOMM20;EEF1B2;RPS19;RPS20;RPL39;TPT1
Catechol-40_mg/kg_in_Saline-Rat-Bone_marrow-1d-dn	14/307	0,000174432	0,007590191	0	0	3,134199716	27,12329221	RPL32;RPS8;RPS5;RPS6;RPSA;RPS3A;RPL10A;EEF1B2;RPS17;RPS19;ARL2BP;RPS20;RPL39;TPT1
Ethanol-3000_mg/kg_in_Saline-Rat-Kidney-1d-up	14/307	0,000174432	0,007548486	0	0	3,134199716	27,12329221	RPL32;RPS5;RPS6;RPL13A;RPL35A;RPSA;SEPP1;EEF1B2;RPS17;RPS19;ARL6IP5;RPS20;RPL39;TPT1
Mitomycin_C-1.7_mg/kg_in_Saline-Rat-Liver-3d-up	14/307	0,000174432	0,007507238	0	0	3,134199716	27,12329221	RPL32;RPS8;RPS5;RPS6;RPL35A;RPSA;RPS3A;RPL10A;EEF1B2;RPS17;RPS19;RPS20;RAPGEF5;RPL39
Ramipril-620_uM_in_DMSO-Rat-Primary_rat_hepatocytes-0.67d-up	14/307	0,000174432	0,007466437	0	0	3,134199716	27,12329221	RPL32;RPS8;RPS5;RPL35A;RPSA;RPS3A;RPL10A;PDRG1;EEF1B2;RPS17;RPS19;ARL2BP;ARL6IP5;RPS20
Terazosin-657_mg/kg_in_Water-Rat-Heart-3d-up	14/307	0,000174432	0,007426078	0	0	3,134199716	27,12329221	C1QA;RPL32;RPS8;RPS5;RPS6;RPL35A;RPSA;RPS3A;RPL10A;RPS17;RPS19;ARL2BP;FUNDC1;ARL6IP5
Propylene_Glycol-2000_mg/kg_in_Water-Rat-Kidney-3d-dn	11/200	0,000175024	0,007411233	0	0	3,780068729	32,69981427	RPS17;RPL32;RPS19;RPS8;RPS6;RPL35A;RPS20;RPSA;RPS3A;RPL10A;RPL39
Cisplatin-2_mg/kg_in_Saline-Rat-Liver-3d-up	14/308	0,000180394	0,00759778	0	0	3,124023743	26,93022816	C1QA;RPL32;RPS8;RPS5;RPS6;RPL35A;RPSA;RPS3A;RPL10A;EEF1B2;RPS17;RPS19;RPS20;RPL39
Tranilast-300_mg/kg_in_CMC-Rat-Heart-3d-dn	14/308	0,000180394	0,007557366	0	0	3,124023743	26,93022816	C1QA;RPL32;RPS8;RPS6;RPSA;RPS3A;RPL10A;SEPP1;RPS17;RPS19;ARL2BP;ARL6IP5;RPS20;RPL39
44'-Methylenedianiline-81_mg/kg_in_Corn_Oil-Rat-Liver-1d-up	15/347	0,000184772	0,007699805	0	0	2,970973588	25,53964379	C1QA;CXADR;RPL32;RPS8;RPS5;RPS6;RPSA;RPS3A;RPL10A;RPS17;RPS19;RPS20;RPL39;GOLGA7;TPT1
2-Amino-4-Nitrophenol-625_mg/kg_in_CMC-Rat-Kidney-3d-up	14/309	0,000186532	0,007732236	0	0	3,113913633	26,73889151	C1QA;RPL32;RPS8;RPS6;RPSA;RPS3A;RPL10A;SEPP1;EEF1B2;RPS17;RPS19;RPS20;RPL39;TPT1
Cholecalciferol-8_mg/kg_in_CMC-Rat-Liver-5d-up	14/309	0,000186532	0,007691753	0	0	3,113913633	26,73889151	C1QA;RPL32;RPS8;RPS5;RPS6;RPL35A;RPSA;RPS3A;RPL10A;EEF1B2;RPS17;RPS19;RPS20;TPT1
Estriol-313_mg/kg_in_CMC-Rat-Liver-3d-up	14/309	0,000186532	0,007651692	0	0	3,113913633	26,73889151	RPL32;RPS8;FEM1B;RPS5;RPS6;RPSA;RPS3A;RPL10A;EEF1B2;RPS17;RPS19;RPS20;RPL39;TPT1
Mevastatin-1200_mg/kg_in_CMC-Rat-Liver-1d-up	14/309	0,000186532	0,007612046	0	0	3,113913633	26,73889151	RPL32;RPS8;RPS5;RPS6;RPL35A;RPSA;RPS3A;RPL10A;SEPP1;EEF1B2;RPS17;CXCL11;RPS20;TPT1
Phenobarbital-25_mg/kg_in_Water-Rat-Liver-1d-up	14/309	0,000186532	0,007572809	0	0	3,113913633	26,73889151	RPL32;RPS8;RPS5;RPS6;RPL35A;RPSA;RPS3A;RPL10A;SEPP1;EEF1B2;RPS17;RPS19;RPS20;RPL39
Timolol-900_mg/kg_in_Water-Rat-Heart-5d-up	14/309	0,000186532	0,007533974	0	0	3,113913633	26,73889151	RPL32;RPS8;RPS5;RPS6;RPSA;RPS3A;RPL10A;SEPP1;EEF1B2;RPS17;ARL2BP;FUNDC1;ARL6IP5;RPL39
Nabumetone-2500_mg/kg_in_Corn_Oil-Rat-Kidney-3d-up	15/348	0,000190612	0,007659508	0	0	2,962436308	25,374062	C1QA;RPL32;RPS8;RPS5;RPS6;RPL35A;RPSA;RPS3A;RPL10A;PDRG1;EEF1B2;RPS17;RPS19;RPS20;RPL39
Chlortetracycline-1500_mg/kg_in_CMC-Rat-Liver-1d-up	14/310	0,000192849	0,007710041	0	0	3,103868751	26,54926237	RPL32;RPS8;RPS5;RPS6;RPSA;RPS3A;RPL10A;EEF1B2;RPS17;RPS19;ARL2BP;RPS20;RPL39;TPT1
Digitoxin-1_mg/kg_in_Corn_Oil-Rat-Heart-0.25d-dn	14/310	0,000192849	0,007671102	0	0	3,103868751	26,54926237	RPL32;RPS8;RPS6;RPL35A;RPSA;TOMM20;PDRG1;EEF1B2;RPS17;RPS19;ARL2BP;FUNDC1;RPS20;RPL39
Cyclosporin_A-350_mg/kg_in_Corn_Oil-Rat-Kidney-1d-up	16/388	0,000193994	0,007677874	0	0	2,83416587	24,22555211	C1QA;RPL32;RPS8;RPS5;RPS6;RPL35A;RPSA;RPS3A;RPL10A;EEF1B2;RPS17;CXCL11;RPS19;RPS20;RPL39;TPT1
Abamectin-10_mg/kg_in_Water-Rat-Liver-1d-up	14/311	0,00019935	0,007850393	0	0	3,093888465	26,36132117	C1QA;RPL32;RPS8;RPS5;RPS6;RPL35A;RPSA;RPS3A;EEF1B2;RPS17;RPS19;RPS20;RPL39;TPT1
Hydrazine-45_mg/kg_in_Water-Rat-Liver-5d-up	14/311	0,00019935	0,007811336	0	0	3,093888465	26,36132117	C1QA;RPL32;RPS8;RPS5;RPS6;RPSA;RPS3A;RPL10A;SEPP1;RPS17;RPS19;RPS20;RAPGEF5;RPL39
Risperidone-22_mg/kg_in_CMC-Rat-Heart-3d-up	14/311	0,00019935	0,007772666	0	0	3,093888465	26,36132117	RPL32;RPS8;RPS5;RPS3A;SEPP1;EEF1B2;RPS17;RPS19;ARL2BP;FUNDC1;RPS20;RAPGEF5;GOLGA7;TPT1
Vinblastine-0.3_mg/kg_in_Saline-Rat-Liver-5d-up	14/311	0,00019935	0,007734377	0	0	3,093888465	26,36132117	C1QA;RPL32;RPS8;RPS5;RPS6;RPSA;RPS3A;RPL10A;EEF1B2;RPS17;RPS19;RPS20;RPL39;TPT1
Fenofibrate-430_mg/kg_in_Corn_Oil-Rat-Skeletal_muscle-5d-up	13/274	0,000201526	0,007780498	0	0	3,260842301	27,74843238	C1QA;RPL32;RPS8;RPS6;RPSA;RPS3A;RPL10A;EEF1B2;RPS17;RPS19;RPS20;RPL39;TPT1
Lead(IV)_Acetate-200_mg/kg_in_Saline-Rat-Liver-3d-up	13/274	0,000201526	0,007742544	0	0	3,260842301	27,74843238	RPL32;RPS8;RPS5;RPS6;RPL35A;RPSA;RPL10A;SEPP1;EEF1B2;RPS17;RPS20;RPL39;TPT1
1-Naphthyl_Isothiocyanate-60_mg/kg_in_Corn_Oil-Rat-Liver-3d-up	14/312	0,000206039	0,00787748	0	0	3,083972156	26,17504858	C1QA;RPS8;FEM1B;RPS5;RPS6;RPSA;RPS3A;RPL10A;SEPP1;EEF1B2;RPS17;RPS19;RPS20;RPL39
Digitonin-75_mg/kg_in_Corn_Oil-Rat-Heart-3d-up	15/351	0,000209105	0,007956081	0	0	2,937116339	24,88523336	RPL32;RPS5;RPS6;RPL35A;RPSA;RPS3A;RPL10A;SEPP1;TOMM20;EEF1B2;RPS17;RPS19;ARL6IP5;RPS20;RPL39
Dimenhydrinate-165_mg/kg_in_CMC-Rat-Liver-3d-up	14/313	0,00021292	0,008062309	0	0	3,07411921	25,99042555	RPL32;RPS8;RPS5;RPS6;RPL13A;RPL35A;RPSA;RPS3A;RPL10A;EEF1B2;RPS17;RPS19;RPS20;RPL39
Procarbazine-54_mg/kg_in_Water-Rat-Bone_marrow-5d-up	14/313	0,00021292	0,008023734	0	0	3,07411921	25,99042555	C1QA;RPL32;RPS8;RPS5;RPL13A;RPL35A;RPSA;RPS3A;SEPP1;EEF1B2;RPS17;ARL2BP;RPS20;TPT1
Sulindac-23_mg/kg_in_Corn_Oil-Rat-Liver-3d-dn	14/313	0,00021292	0,007985525	0	0	3,07411921	25,99042555	C1QA;RPL32;RPS8;RPS6;RPL35A;RPSA;RPS3A;RPL10A;SEPP1;RPS17;RPS19;RPS20;RPL39;TPT1
Rofecoxib-775_mg/kg_in_Corn_Oil-Rat-Kidney-1d-dn	13/276	0,000216342	0,00807539	0	0	3,237213009	27,31771197	RPL32;RPS8;RPS6;RPL35A;RPSA;RPS3A;SEPP1;TOMM20;EEF1B2;RPS17;ARL6IP5;RPL39;TPT1
Cyclophosphamide-25_mg/kg_in_Water-Rat-Heart-3d-up	14/314	0,000219999	0,008173173	0	0	3,064329021	25,80743334	RPL32;RPS8;RPS6;RPL35A;RPSA;RPS3A;RPL10A;EEF1B2;RPS17;RPS19;ARL2BP;ARL6IP5;RPS20;RAPGEF5
Etoposide-188_mg/kg_in_Corn_Oil-Rat-Bone_marrow-3d-up	14/314	0,000219999	0,008134802	0	0	3,064329021	25,80743334	C1QA;RPL32;RPS8;RPS5;NKG7;RPL35A;RPSA;RPS3A;RPL10A;SEPP1;EEF1B2;RPS17;RPS20;RPL39
Methapyrilene-100_mg/kg_in_CMC-Rat-Liver-1d-up	14/314	0,000219999	0,008096789	0	0	3,064329021	25,80743334	RPL32;RPS8;RPS5;RPS6;RPSA;RPS3A;RPL10A;SEPP1;EEF1B2;RPS17;RPS19;RPS20;RPL39;TPT1
Aspirin-35_mg/kg_in_Corn_Oil-Rat-Kidney-1d-dn	13/277	0,000224093	0,008209104	0	0	3,225526319	27,10554579	RPL32;RPS8;RPL35A;RPSA;RPS3A;EEF1B2;RPS17;ARL2BP;R3HCC1;ARL6IP5;RPS20;RPL39;TPT1
L-Buthionine_Sulfoximine-100000_uM_in_DMSO-Rat-Primary_rat_hepatocytes-1d-up	13/277	0,000224093	0,008171098	0	0	3,225526319	27,10554579	RPL32;RPS8;FEM1B;RPS5;RPL13A;RPL35A;RPS3A;PDRG1;EEF1B2;RPS17;ARL6IP5;RPS20;TPT1
Mevastatin-1200_mg/kg_in_CMC-Rat-Liver-5d-up	14/315	0,00022728	0,0082491	0	0	3,054600993	25,62605344	RPL32;RPS8;RPS5;RPS6;RPL35A;RPSA;RPS3A;RPL10A;SEPP1;RPS17;RPS19;ARL6IP5;RPS20;TPT1
Oxcarbazepine-1600_mg/kg_in_CMC-Rat-Brain-5d-up	14/315	0,00022728	0,00821126	0	0	3,054600993	25,62605344	RPL32;RPS8;RPS6;RPL35A;RPSA;RPS3A;RPL10A;SEPP1;TOMM20;RPS17;RPS20;RAPGEF5;RPL39;GOLGA7
Teicoplanin-41_mg/kg_in_Saline-Rat-Bone_marrow-3d-dn	14/315	0,00022728	0,008173766	0	0	3,054600993	25,62605344	C1QA;RPL32;RPS8;RPS5;RPS6;NKG7;RPL13A;RPL35A;RPSA;RPS17;ARL6IP5;RPS20;RPL39;TPT1
Mitomycin_C-1.7_mg/kg_in_Saline-Rat-Bone_marrow-3d-dn	13/278	0,000232081	0,00830851	0	0	3,213923706	26,89547266	RPL32;RPS8;FEM1B;RPS6;NKG7;RPSA;RPS3A;EEF1B2;RPS17;ARL2BP;FUNDC1;RPS20;TPT1
Dimenhydrinate-165_mg/kg_in_CMC-Rat-Liver-5d-up	14/316	0,000234767	0,008366622	0	0	3,044934534	25,44626762	RPL32;RPS8;RPS5;RPS6;RPL13A;RPL35A;RPSA;RPS3A;SEPP1;RPS17;RPS19;ARL6IP5;RPS20;TPT1
Phenacemide-400_mg/kg_in_CMC-Rat-Kidney-5d-up	14/316	0,000234767	0,008328934	0	0	3,044934534	25,44626762	RPL32;RPS8;RPS5;RPS6;RPL35A;RPSA;RPS3A;RPL10A;SEPP1;EEF1B2;RPS17;CXCL11;RPS19;RPS20
Pirinixic_Acid-364_mg/kg_in_CMC-Rat-Liver-1d-up	14/316	0,000234767	0,008291585	0	0	3,044934534	25,44626762	RPL32;RPS8;RPS5;RPS6;RPL35A;RPSA;RPS3A;RPL10A;EEF1B2;RPS17;RPS19;RPS20;RPL39;TPT1
Primaquine-45_mg/kg_in_CMC-Rat-Liver-3d-up	14/316	0,000234767	0,008254569	0	0	3,044934534	25,44626762	C1QA;RPL32;RPS8;RPS5;RPS6;RPSA;RPS3A;RPL10A;EEF1B2;RPS19;ARL2BP;RPS20;RPL39;TPT1
Rifampin-125_uM_in_DMSO-Rat-Primary_rat_hepatocytes-1d-dn	15/355	0,000236164	0,008266804	0	0	2,904022071	24,25143717	RPL32;RPS8;RPS5;RPL35A;RPSA;RPL10A;SEPP1;EEF1B2;RPS17;CXCL11;RPS19;ARL2BP;ARL6IP5;RPL39;TPT1
Calcitriol-0.04_mg/kg_in_CMC-Rat-Kidney-5d-up	14/317	0,000242465	0,008449809	0	0	3,035329062	25,26805789	RPL32;RPS8;RPS6;RPSA;RPS3A;RPL10A;EEF1B2;CXCL11;RPS19;CLP1;RPS20;RAPGEF5;RPL39;TPT1
Catechol-195_mg/kg_in_Saline-Rat-Kidney-1d-up	14/317	0,000242465	0,008412585	0	0	3,035329062	25,26805789	RPL32;RPS8;RPS5;RPL13A;RPL35A;RPSA;RPS3A;SEPP1;TOMM20;EEF1B2;RPS17;ARL6IP5;RPS20;RPL39
Ethylene_Glycol-3525_mg/kg_in_Water-Rat-Bone_marrow-5d-up	14/317	0,000242465	0,008375688	0	0	3,035329062	25,26805789	C1QA;RPL32;RPS5;NKG7;RPL35A;RPSA;RPS3A;RPL10A;SEPP1;EEF1B2;RPS17;RPS20;RPL39;TPT1
Foscarnet-350_mg/kg_in_Saline-Rat-Kidney-5d-dn	14/317	0,000242465	0,008339113	0	0	3,035329062	25,26805789	RPL32;RPS5;RPS6;RPL35A;RPS3A;RPL10A;SEPP1;EEF1B2;RPS17;RPS19;ARL6IP5;RPS20;RAPGEF5;RPL39
Gabapentin-2500_mg/kg_in_Water-Rat-Heart-3d-up	14/317	0,000242465	0,008302856	0	0	3,035329062	25,26805789	RPL32;RPS6;RPSA;RPS3A;RPL10A;SEPP1;RPS17;RPS19;COL5A3;ARL6IP5;RPS20;RAPGEF5;RPL39;GOLGA7
Lamotrigine-26_mg/kg_in_CMC-Rat-Kidney-1d-dn	14/317	0,000242465	0,008266912	0	0	3,035329062	25,26805789	RPL32;RPS8;RPS6;RPSA;RPL10A;SEPP1;PDRG1;EEF1B2;RPS19;ARL2BP;ARL6IP5;RPS20;RPL39;TPT1
Ondansetron-84_mg/kg_in_Water-Rat-Intestine-3d-dn	14/317	0,000242465	0,008231279	0	0	3,035329062	25,26805789	RPL32;RPS8;RPS5;RPS6;RPL13A;RPL35A;RPSA;RPS3A;RPL10A;EEF1B2;RPS17;RPS20;RPL39;TPT1
Norethindrone-75_mg/kg_in_Corn_Oil-Rat-Liver-1d-up	12/243	0,000244657	0,008270028	0	0	3,394001103	28,2233403	EEF1B2;C1QA;RPS17;RPS19;RPS8;FEM1B;RPS6;RPS20;RPSA;RPS3A;RPL10A;RPL39
Phenacetin-619_mg/kg_in_Corn_Oil-Rat-Liver-5d-up	13/280	0,000248792	0,008373862	0	0	3,190967108	26,48149925	RPL32;RPS8;RPS5;RPS6;RPL35A;RPSA;RPS3A;RPL10A;SEPP1;RPS17;RPS19;RPS20;TPT1
Aldosterone-0.065_mg/kg_in_Saline-Rat-Kidney-1d-dn	14/318	0,00025038	0,00839146	0	0	3,025784002	25,09140654	RPL32;RPS5;RPS6;RPL35A;RPSA;RPS3A;RPL10A;EEF1B2;RPS17;ARL2BP;FUNDC1;RPS20;RPL39;TPT1
Citric_Acid-3000_mg/kg_in_Water-Rat-Liver-3d-up	14/318	0,00025038	0,008355903	0	0	3,025784002	25,09140654	RPL32;RPS8;RPS5;RPS6;RPL35A;RPSA;RPS3A;RPL10A;EEF1B2;RPS17;RPS19;ARL6IP5;RPS20;RPL39
Kanamycin-225_mg/kg_in_Saline-Rat-Kidney-3d-up	14/318	0,00025038	0,008320646	0	0	3,025784002	25,09140654	C1QA;RPL32;RPS8;RPS5;RPL13A;RPL35A;RPSA;RPS3A;RPS17;CXCL11;ARL6IP5;RPS20;RPL39;TPT1
Zidovudine-1473_uM_in_DMSO-Rat-Primary_rat_hepatocytes-1d-dn	14/319	0,000258516	0,008554924	0	0	3,016298786	24,91629609	RPL32;RPS8;RPS6;RPL35A;RPS3A;RPL10A;RPS17;CXCL11;RPS19;ARL6IP5;RPS20;RPL39;GOLGA7;TPT1
Clomipramine-115_mg/kg_in_Water-Rat-Heart-3d-dn	14/320	0,000266878	0,008794697	0	0	3,006872852	24,74270932	RPL32;RPS8;RPS6;RPSA;RPS3A;RPL10A;SEPP1;EEF1B2;RPS17;RPS19;FUNDC1;RPS20;RPL39;TPT1
Cyclosporin_A-70_mg/kg_in_Corn_Oil-Rat-Bone_marrow-3d-up	14/320	0,000266878	0,008758053	0	0	3,006872852	24,74270932	C1QA;RPL32;RPS8;RPS5;RPS6;RPL35A;RPSA;RPL10A;EEF1B2;CLP1;RPS20;RPL39;SLC16A3;TPT1
Pralidoxime_Chloride-48_mg/kg_in_Saline-Rat-Liver-1d-dn	14/320	0,000266878	0,008721712	0	0	3,006872852	24,74270932	RPL32;RPS8;RPS5;RPS6;RPL35A;RPSA;RPS3A;RPL10A;EEF1B2;RPS17;RPS19;RPS20;RPL39;TPT1
Rolipram-75_mg/kg_in_CMC-Rat-Heart-3d-up	14/320	0,000266878	0,008685672	0	0	3,006872852	24,74270932	RPS8;RPSA;RPS3A;RPL10A;TOMM20;PDRG1;EEF1B2;ARL2BP;FUNDC1;ARL6IP5;RPS20;RAPGEF5;RPL39;TPT1
Cadmium_Chloride-66_mg/kg_in_Corn_Oil-Rat-Kidney-5d-up	15/360	0,000274204	0,008887385	0	0	2,863688431	23,48693127	RPL32;RPS8;RPS5;RPS6;RPL35A;RPSA;RPS3A;RPL10A;EEF1B2;RPS17;CXCL11;RPS19;ARL6IP5;RPS20;RPL39
Diphenhydramine-100_mg/kg_in_Water-Rat-Heart-3d-up	14/321	0,000275472	0,00889187	0	0	2,997505647	24,57062923	RPL32;RPS8;RPS5;RPS6;RPL35A;RPSA;RPS3A;SEPP1;RPS17;RPS19;ARL2BP;RPS20;GOLGA7;TPT1
Leflunomide-60_mg/kg_in_Corn_Oil-Rat-Spleen-5d-up	14/321	0,000275472	0,008855576	0	0	2,997505647	24,57062923	C1QA;RPL32;RPS8;RPSA;RPS3A;RPL10A;SEPP1;EEF1B2;RPS17;CXCL11;RPS20;RAPGEF5;RPL39;TPT1
Terbutaline-130_mg/kg_in_Corn_Oil-Rat-Heart-3d-up	14/321	0,000275472	0,008819578	0	0	2,997505647	24,57062923	C1QA;RPL32;RPS8;RPS5;RPS6;RPSA;RPS3A;RPL10A;SEPP1;EEF1B2;RPS19;RPS20;RPL39;TPT1
Coumarin-111_mg/kg_in_Corn_Oil-Rat-Bone_marrow-5d-up	13/283	0,000275784	0,008793829	0	0	3,157140602	25,87558372	C1QA;RPL32;RPS8;FEM1B;RPS5;RPS6;RPL35A;RPSA;RPS3A;RPS17;CLP1;RPS20;TPT1
4-Octylphenol-50_mg/kg_in_Corn_Oil-Rat-Liver-0.25d-up	15/361	0,000282416	0,008968984	0	0	2,855755776	23,33760611	RPL32;RPS8;RPS5;RPS6;RPL35A;RPSA;RPS3A;RPL10A;SEPP1;EEF1B2;RPS17;RPS19;RPS20;RPL39;TPT1
Diclofenac-10_mg/kg_in_Corn_Oil-Rat-Kidney-5d-up	14/322	0,000284302	0,008992625	0	0	2,988196623	24,40003908	C1QA;RPL32;RPS8;RPS5;RPS6;RPL35A;RPSA;RPS3A;RPS17;RPS19;RPS20;RPL39;GOLGA7;TPT1
Sparteine-33_mg/kg_in_Water-Rat-Heart-1d-up	14/322	0,000284302	0,008956654	0	0	2,988196623	24,40003908	C1QA;RPL32;RPS8;RPS5;RPS6;RPL13A;RPL35A;RPSA;RPS3A;TOMM20;RPS17;RPS20;RPL39;TPT1
Carbon_Tetrachloride-1175_mg/kg_in_Corn_Oil-Rat-Liver-1d-up	13/284	0,000285321	0,008952929	0	0	3,14602391	25,67752326	RPL32;RPS8;RPS5;RPS6;RPSA;RPS3A;RPL10A;EEF1B2;RPS17;RPS19;RPS20;RPL39;TPT1
Diphenhydramine-10.9_mg/kg_in_Water-Rat-Heart-3d-up	15/362	0,000290839	0,009089863	0	0	2,847866948	23,18944534	RPL32;RPS8;RPS5;RPS6;RPL35A;RPSA;RPS3A;RPL10A;RPS17;RPS19;ARL2BP;RPS20;RPL39;GOLGA7;TPT1
Eucalyptol-930_mg/kg_in_Corn_Oil-Rat-Kidney-3d-up	14/323	0,000293374	0,009132872	0	0	2,978945241	24,23092236	RPL32;RPS8;RPS5;RPS6;RPL13A;RPL35A;RPSA;RPS3A;EEF1B2;RPS17;RPS19;ARL2BP;ARL6IP5;RPS20
Lomustine-560_uM_in_DMSO-Rat-Primary_rat_hepatocytes-0.67d-up	14/323	0,000293374	0,009096916	0	0	2,978945241	24,23092236	RPL32;RPS8;FEM1B;RPS5;RPL13A;PDRG1;EEF1B2;RPS17;RPS19;ARL2BP;CLP1;ARL6IP5;RPS20;GOLGA7
Carboplatin-6_mg/kg_in_Saline-Rat-Liver-0.25d-up	13/285	0,000295137	0,009115695	0	0	3,134985229	25,48137777	RPL32;RPS8;RPS5;RPS6;RPL35A;RPS3A;RPL10A;EEF1B2;RPS17;RPS19;RPS20;RPL39;TPT1
Ergocalciferol-15_mg/kg_in_CMC-Rat-Kidney-5d-up	13/285	0,000295137	0,009080087	0	0	3,134985229	25,48137777	C1QA;RPL32;RPS8;RPS6;RPL35A;RPSA;RPS3A;RPL10A;EEF1B2;RPS17;RPS19;RPS20;RAPGEF5
Lacidipine-14_mg/kg_in_Corn_Oil-Rat-Heart-3d-up	13/285	0,000295137	0,009044756	0	0	3,134985229	25,48137777	C1QA;RPL32;RPS6;RPL35A;RPS3A;RPL10A;SEPP1;RPS17;RPS19;RPS20;RAPGEF5;RPL39;TPT1
Phenelzine-27_mg/kg_in_Water-Rat-Brain-3d-up	13/285	0,000295137	0,009009699	0	0	3,134985229	25,48137777	RPL32;RPL35A;RPSA;RPS3A;RPL10A;EEF1B2;RPS17;RPS19;ARL2BP;ARL6IP5;RPS20;RPL39;TPT1
Ranitidine-1500_mg/kg_in_CMC-Rat-Heart-1d-dn	13/285	0,000295137	0,008974912	0	0	3,134985229	25,48137777	C1QA;RPL32;RPS8;RPS5;RPSA;RPS3A;TOMM20;EEF1B2;RPS17;RPS19;ARL2BP;RPL39;TPT1
Artemether-74_mg/kg_in_Corn_Oil-Rat-Liver-5d-up	14/324	0,000302694	0,0091693	0	0	2,969750965	24,06326276	RPL32;RPS8;RPS5;RPS6;RPL35A;RPSA;RPS3A;RPL10A;SEPP1;EEF1B2;RPS17;RPS19;RPS20;TPT1
Chlorambucil-4.5_mg/kg_in_Corn_Oil-Rat-Spleen-5d-dn	14/324	0,000302694	0,009134169	0	0	2,969750965	24,06326276	RPS8;RPS5;RPS6;RPL13A;RPL35A;RPSA;RPS3A;RPL10A;RPS17;RPS19;FUNDC1;RPS20;RPL39;TPT1
Penicillamine-852_mg/kg_in_Water-Rat-Bone_marrow-5d-up	14/324	0,000302694	0,009099306	0	0	2,969750965	24,06326276	C1QA;RPL32;RPS5;RPS6;NKG7;RPL35A;RPS3A;RPL10A;SEPP1;EEF1B2;RPS17;RPS20;RPL39;TPT1
Atenolol-1000_mg/kg_in_CMC-Rat-Heart-5d-up	14/325	0,000312267	0,009351374	0	0	2,96061327	23,89704424	C1QA;RPL32;RPS8;RPS5;RPS6;RPL35A;RPSA;RPS3A;TOMM20;EEF1B2;RPS17;RPS19;FUNDC1;TPT1
Bromfenac-5_mg/kg_in_CMC-Rat-Liver-3d-up	14/325	0,000312267	0,009315952	0	0	2,96061327	23,89704424	RPL32;RPS8;RPS5;RPS6;RPSA;RPS3A;RPL10A;EEF1B2;RPS17;RPS19;ARL2BP;RPS20;RPL39;TPT1
Norethindrone_Acetate-125_mg/kg_in_Corn_Oil-Rat-Liver-5d-up	14/325	0,000312267	0,009280797	0	0	2,96061327	23,89704424	RPL32;RPS8;RPS5;RPS6;RPL13A;RPL35A;RPSA;RPS3A;RPL10A;RPS17;RPS19;RPS20;RPL39;TPT1
Ethinylestradiol-10_mg/kg_in_Corn_Oil-Rat-Liver-1d-up	13/287	0,000315639	0,009345757	0	0	3,113138642	25,09473664	RPL32;RPS8;FEM1B;RPS5;RPS6;RPSA;RPS3A;RPL10A;EEF1B2;RPS17;RPS19;RPS20;RPL39
Lead_(II)_Acetate-200_mg/kg_in_Saline-Rat-Liver-1d-dn	13/287	0,000315639	0,009310754	0	0	3,113138642	25,09473664	RPL32;RPS8;RPS5;RPS6;RPSA;RPS3A;SEPP1;EEF1B2;RPS17;RPS19;RPS20;RPL39;TPT1

Oxaliplatin-8_mg/kg_in_Saline-Rat-Bone_marrow-1d-dn	13/287	0,000315639	0,009276012	0	0	3,113138642	25,09473664	RPL32;RPS8;RPS5;RPS6;RPL35A;RPSA;RPS3A;EEF1B2;RPS17;RPS19;RPS20;RPL39;TPT1
Oxytetracycline-1500_mg/kg_in_Water-Rat-Liver-5d-dn	13/287	0,000315639	0,009241529	0	0	3,113138642	25,09473664	RPL32;RPS8;RPS5;RPS6;RPL35A;RPSA;RPS3A;RPL10A;SEPP1;EEF1B2;RPS17;RPS19;RPL39
Propranolol-175_mg/kg_in_Water-Rat-Heart-1d-up	15/365	0,000317419	0,009259238	0	0	2,824459822	22,75183461	RPL32;RPS8;RPS5;RPS6;RPL35A;RPSA;RPS3A;EEF1B2;RPS17;CXCL11;RPS19;ARL6IP5;RPS20;RPL39;TPT1
Cytarabine-487_mg/kg_in_Saline-Rat-Bone_marrow-1d-up	14/326	0,000322098	0,009361034	0	0	2,951531634	23,73225095	C1QA;RPL32;RPS8;RPS5;RPS6;RPL35A;RPSA;RPS3A;RPL10A;EEF1B2;RPS17;RPS19;RPS20;TPT1
Famciclovir-1200_mg/kg_in_Saline-Rat-Liver-1d-dn	14/326	0,000322098	0,009326618	0	0	2,951531634	23,73225095	RPL32;RPS8;RPS5;RPS6;RPL35A;RPS3A;RPL10A;SEPP1;EEF1B2;RPS17;RPS19;RPS20;RPL39;TPT1
Dexamethasone-150_mg/kg_in_Corn_Oil-Rat-Liver-3d-dn	13/288	0,000326337	0,009414765	0	0	3,102329133	24,90419438	C1QA;RPL32;RPS8;RPS6;RPSA;RPS3A;RPL10A;SEPP1;EEF1B2;RPS17;RPS19;RPS20;RPL39
Megestrol_Acetate-132_mg/kg_in_CMC-Rat-Liver-3d-dn	13/288	0,000326337	0,009380405	0	0	3,102329133	24,90419438	C1QA;RPL32;RPS8;FEM1B;RPS6;RPL35A;RPS3A;SEPP1;RPS17;RPS19;CLP1;RPS20;TPT1
Dexfenfluramine-29_mg/kg_in_Water-Rat-Kidney-1d-dn	11/215	0,000326663	0,009355624	0	0	3,516343003	28,22421538	RPS17;RPL32;RPS19;RPS6;ARL6IP5;RPL35A;RPS20;RPSA;RPS3A;RPL10A;RPL39
Risperidone-400_uM_in_DMSO-Rat-Primary_rat_hepatocytes-1d-dn	16/407	0,000330736	0,009437968	0	0	2,701858372	21,65320395	RPL32;RPS8;RPS5;RPS6;RPS3A;RPL10A;SEPP1;EEF1B2;RPS17;CXCL11;RPS19;ARL2BP;ARL6IP5;RPL39;SLC16A3;TPT1
Amlodipine-19_mg/kg_in_Corn_Oil-Rat-Liver-0.25d-up	14/327	0,000332193	0,009445306	0	0	2,942505543	23,56886726	RPL32;RPS8;FEM1B;RPS5;RPL13A;RPL35A;RPSA;RPS3A;SEPP1;TOMM20;EEF1B2;RPS17;RPS19;TPT1
Cycloheximide-0.25_mg/kg_in_Water-Rat-Liver-1d-up	14/327	0,000332193	0,00941133	0	0	2,942505543	23,56886726	RPL32;RPS8;RPS5;RPS6;RPL35A;RPSA;RPS3A;RPL10A;EEF1B2;RPS17;RPS19;RPS20;RPL39;TPT1
Cyclosporin_A-350_mg/kg_in_Corn_Oil-Rat-Heart-5d-up	13/289	0,000337343	0,009522989	0	0	3,09159443	24,71547386	C1QA;RPS8;RPS6;RPSA;RPS3A;RPL10A;SEPP1;CXCL11;FUNDC1;RPS20;RAPGEF5;RPL39;TPT1
Nitrendipine-1100_mg/kg_in_Corn_Oil-Rat-Heart-3d-up	13/289	0,000337343	0,009488979	0	0	3,09159443	24,71547386	C1QA;RPL32;RPS5;RPS6;RPL35A;RPSA;RPS3A;RPL10A;SEPP1;RPS19;RPS20;RPL39;TPT1
Sodium_Arsenite-30_mg/kg_in_Saline-Rat-Liver-0.25d-up	13/289	0,000337343	0,00945521	0	0	3,09159443	24,71547386	C1QA;RPL32;RPS8;RPS5;RPS6;RPL35A;RPSA;RPS3A;SEPP1;EEF1B2;RPS17;RPS20;TPT1
Zomepirac-2.8_mg/kg_in_Saline-Rat-Kidney-1d-dn	13/289	0,000337343	0,009421681	0	0	3,09159443	24,71547386	RPL32;RPS8;RPS6;RPSA;RPS3A;TOMM20;EEF1B2;RPS17;ARL2BP;R3HCC1;RPS20;RPL39;TPT1
11-Dichloroethene-600_mg/kg_in_Water-Rat-Liver-3d-up	14/328	0,000342558	0,009533516	0	0	2,93353449	23,40687777	C1QA;RPL32;RPS8;RPS5;RPS6;RPL35A;RPSA;RPS3A;RPL10A;EEF1B2;RPS17;RPS19;RPS20;RPL39
Cadmium_Chloride-66_mg/kg_in_Corn_Oil-Rat-Kidney-1d-up	14/328	0,000342558	0,009499947	0	0	2,93353449	23,40687777	RPL32;RPS8;RPS5;RPS6;RPSA;RPS3A;RPL10A;SEPP1;TOMM20;RPS17;RPS19;RPS20;RAPGEF5;TPT1
Tacrolimus-134_mg/kg_in_CMC-Rat-Heart-1d-up	14/328	0,000342558	0,009466614	0	0	2,93353449	23,40687777	RPL32;RPS6;RPSA;RPS3A;RPL10A;SEPP1;EEF1B2;RPS17;CXCL11;RPS19;ARL6IP5;RPS20;RAPGEF5;RPL39
Vinorelbine-1.5_mg/kg_in_Saline-Rat-Bone_marrow-1d-dn	14/328	0,000342558	0,009433514	0	0	2,93353449	23,40687777	RPL32;RPS8;RPS5;RPS6;NKG7;RPSA;RPS3A;RPL10A;EEF1B2;RPS17;RPS19;RPS20;RPL39;TPT1
Mestranol-250_mg/kg_in_CMC-Rat-Liver-1d-up	13/290	0,000348664	0,009568212	0	0	3,08093376	24,52855266	RPL32;RPS8;FEM1B;RPS5;RPS6;RPSA;RPS3A;RPL10A;EEF1B2;RPS17;RPS19;RPS20;RPL39
Allopurinol-175_mg/kg_in_Corn_Oil-Rat-Kidney-3d-up	14/329	0,000353199	0,009659002	0	0	2,924617972	23,24626728	C1QA;RPL32;RPS8;RPS5;RPS6;RPSA;RPS3A;RPL10A;EEF1B2;RPS17;RPS19;RPS20;RPL39;TPT1
Rofecoxib-250_mg/kg_in_Corn_Oil-Rat-Liver-1d-dn	14/329	0,000353199	0,00962558	0	0	2,924617972	23,24626728	C1QA;RPL32;RPS6;RPL35A;RPSA;RPS3A;RPL10A;EEF1B2;RPS17;RPS19;RPS20;RPL39;GOLGA7;TPT1
Vincristine-0.05_mg/kg_in_Saline-Rat-Heart-5d-up	14/329	0,000353199	0,009592388	0	0	2,924617972	23,24626728	RPL32;RPS8;RPS6;RPL35A;RPSA;RPS3A;RPL10A;EEF1B2;RPS17;RPS19;COL5A3;RPS20;RPL39;TPT1
Mestranol-250_mg/kg_in_CMC-Rat-Liver-3d-up	13/291	0,000360307	0,009751809	0	0	3,070346359	24,34340873	RPL32;RPS8;FEM1B;RPS5;RPS6;RPSA;RPS3A;RPL10A;EEF1B2;RPS17;RPS19;RPS20;RPL39
Cisplatin-0.5_mg/kg_in_Saline-Rat-Kidney-28d-up	14/330	0,000364121	0,009821298	0	0	2,915755493	23,0870208	C1QA;RPL32;RPS8;FEM1B;RPS6;RPSA;RPS3A;RPL10A;EEF1B2;CXCL11;RPS19;ARL6IP5;RPS20;RPL39
Ofloxacin-3000_mg/kg_in_Water-Rat-Heart-3d-up	13/292	0,000372279	0,010007069	0	0	3,059831474	24,16002033	RPL32;RPS8;RPS5;RPS6;RPS3A;SEPP1;RPS17;RPS19;ARL2BP;FUNDC1;ARL6IP5;RPS20;RAPGEF5
Acetaminophen-486_mg/kg_in_Corn_Oil-Rat-Kidney-1d-dn	13/293	0,000384589	0,01030279	0	0	3,049388363	23,97836608	RPL32;RPS8;RPS6;RPL35A;RPSA;RPS3A;RPL10A;TOMM20;EEF1B2;RPS17;ARL2BP;CLP1;TPT1
Calcitriol-0.04_mg/kg_in_CMC-Rat-Heart-5d-up	13/293	0,000384589	0,010267865	0	0	3,049388363	23,97836608	RPL32;RPS8;RPS6;RPS3A;RPL10A;SEPP1;EEF1B2;RPS17;RPS19;ARL2BP;RPS20;RPL39;TPT1
Vinorelbine-1.5_mg/kg_in_Saline-Rat-Liver-5d-dn	13/293	0,000384589	0,010233176	0	0	3,049388363	23,97836608	C1QA;RPL32;RPS8;RPS5;RPS6;RPL35A;RPSA;RPS3A;SEPP1;EEF1B2;RPS17;RPS19;TPT1
Amantadine-220_mg/kg_in_Water-Rat-Liver-3d-up	14/332	0,000386836	0,01025832	0	0	2,898190701	22,77256091	RPL32;RPS8;RPS5;RPS6;RPL35A;RPSA;RPS3A;RPL10A;RPS17;RPS19;RPS20;RAPGEF5;RPL39;TPT1
1-Naphthyl_Isothiocyanate-30_mg/kg_in_Corn_Oil-Rat-Bone_marrow-1d-dn	13/294	0,000397243	0,010498937	0	0	3,039016294	23,79842489	RPL32;RPS8;RPS5;RPS6;RPL35A;RPSA;RPS3A;EEF1B2;RPS17;RPS19;ARL6IP5;RPS20;TPT1
Bisphenol_A-100_mg/kg_in_Corn_Oil-Rat-Spleen-1d-up	13/294	0,000397243	0,010463823	0	0	3,039016294	23,79842489	C1QA;RPL32;RPS8;RPL35A;RPS3A;SEPP1;RPS17;ARL2BP;FUNDC1;ARL6IP5;RPS20;GOLGA7;TPT1
Cholecalciferol-8_mg/kg_in_CMC-Rat-Liver-3d-up	13/294	0,000397243	0,010428944	0	0	3,039016294	23,79842489	C1QA;RPL32;RPS8;RPS5;RPS6;RPSA;RPS3A;RPL10A;SEPP1;EEF1B2;RPS17;RPS19;RPL39
S(-)-Carbidopa-1200_mg/kg_in_CMC-Rat-Brain-3d-dn	13/294	0,000397243	0,010394296	0	0	3,039016294	23,79842489	RPL32;RPS8;RPS6;RPL35A;RPSA;RPS3A;SEPP1;EEF1B2;RPS17;ARL6IP5;RPS20;RPL39;TPT1
44'-Methylenedianiline-81_mg/kg_in_Corn_Oil-Rat-Liver-3d-up	14/333	0,000398641	0,010396338	0	0	2,889487426	22,61731852	C1QA;RPL32;RPS8;RPS5;RPS6;RPSA;RPS3A;RPL10A;SEPP1;RPS17;RPS19;RPS20;RPL39;TPT1
Mefenamic_Acid-93_mg/kg_in_Corn_Oil-Rat-Kidney-5d-dn	14/333	0,000398641	0,010362027	0	0	2,889487426	22,61731852	RPL32;RPS8;RPS6;RPSA;RPS3A;RPL10A;SEPP1;EEF1B2;RPS17;RPS19;ARL6IP5;RPS20;RAPGEF5;RPL39
Ethanol-3000_mg/kg_in_Saline-Rat-Liver-1d-dn	12/257	0,000406655	0,010535572	0	0	3,209113883	25,05530333	RPS17;RPL32;RPS19;RPS8;RPS5;RPS6;RPL35A;RPS20;RPS3A;RPL10A;RPL39;TPT1
Ifosfamide-17_mg/kg_in_Saline-Rat-Bone_marrow-3d-dn	12/257	0,000406655	0,010501029	0	0	3,209113883	25,05530333	EEF1B2;RPS17;RPL32;RPS19;RPS6;ARL6IP5;RPL35A;RPS20;RPSA;RPS3A;RPL39;TPT1
Raloxifene-5_mg/kg_in_Corn_Oil-Rat-Liver-5d-up	12/257	0,000406655	0,010466712	0	0	3,209113883	25,05530333	EEF1B2;RPS17;RPL32;RPS19;RPS6;RPL35A;RPS20;RPSA;RPS3A;RPL10A;RPL39;TPT1
Anisindione-75_mg/kg_in_CMC-Rat-Liver-5d-up	13/295	0,000410249	0,010524825	0	0	3,028714544	23,62017604	RPL32;RPS8;RPS5;RPS6;RPL35A;RPSA;RPS3A;RPL10A;RPS17;RPS19;ARL2BP;RPS20;TPT1
Famciclovir-112_mg/kg_in_Saline-Rat-Liver-1d-dn	13/295	0,000410249	0,010490654	0	0	3,028714544	23,62017604	RPL32;RPS8;RPS5;RPS6;RPL35A;RPSA;RPS3A;RPL10A;SEPP1;EEF1B2;RPS17;RPS19;TPT1
Lipopolysaccharide_E._Coli_O55:B5-1.25_mg/kg_in_Saline-Rat-Kidney-3d-up	13/295	0,000410249	0,010456703	0	0	3,028714544	23,62017604	C1QA;RPS8;FEM1B;RPS6;RPSA;RPS3A;RPL10A;SEPP1;CXCL11;RPS19;RPS20;RPL39;TPT1
Tranilast-1100_mg/kg_in_CMC-Rat-Heart-3d-dn	13/295	0,000410249	0,010422972	0	0	3,028714544	23,62017604	RPL32;RPS6;RPSA;RPS3A;SEPP1;RPS17;CXCL11;RPS19;FUNDC1;ARL6IP5;RPS20;RAPGEF5;RPL39
Pravastatin-1200_mg/kg_in_Corn_Oil-Rat-Kidney-5d-dn	12/258	0,000421067	0,010663408	0	0	3,196675458	24,84686255	EEF1B2;RPS17;RPL32;RPS19;CLP1;RPS8;RPS6;RPSA;RPS3A;RPL10A;RPL39;TPT1
Busulfan-9_mg/kg_in_Corn_Oil-Rat-Bone_marrow-3d-up	14/335	0,000423176	0,010682481	0	0	2,872236754	22,31073789	C1QA;RPL32;RPS8;RPS5;RPL35A;RPSA;RPS3A;RPL10A;SEPP1;EEF1B2;RPS17;RPS20;RPL39;TPT1
Bromodichloromethane-430_mg/kg_in_CMC-Rat-Kidney-1d-up	13/296	0,000423616	0,010659414	0	0	3,0184824	23,44359907	RPS8;RPS5;RPS6;RPSA;RPS3A;RPL10A;EEF1B2;RPS17;RPS19;ARL6IP5;RPS20;RAPGEF5;TPT1
Carmustine-16_mg/kg_in_Corn_Oil-Rat-Spleen-3d-up	13/296	0,000423616	0,010625467	0	0	3,0184824	23,44359907	C1QA;RPL32;RPS8;RPS5;RPS6;RPL35A;RPS3A;EEF1B2;RPS17;CXCL11;RPS20;RAPGEF5;RPL39
Disulfiram-100_mg/kg_in_Water-Rat-Liver-1d-up	13/296	0,000423616	0,010591736	0	0	3,0184824	23,44359907	RPL32;RPS8;RPS5;RPS6;RPL35A;RPSA;RPS3A;RPL10A;EEF1B2;RPS17;RPS19;RPS20;RPL39
Lovastatin-1500_mg/kg_in_Corn_Oil-Rat-Skeletal_muscle-3d-dn	13/296	0,000423616	0,010558218	0	0	3,0184824	23,44359907	RPL32;RPS8;RPS5;RPS6;RPL35A;RPS3A;RPL10A;EEF1B2;RPS17;RPS19;ARL6IP5;RPS20;RPL39
Raloxifene-5_mg/kg_in_Corn_Oil-Rat-Liver-1d-up	13/296	0,000423616	0,010524911	0	0	3,0184824	23,44359907	RPL32;RPS8;RPS6;RPSA;RPS3A;RPL10A;TOMM20;EEF1B2;RPS17;RPS19;RPS20;RPL39;TPT1
Famciclovir-112_mg/kg_in_Saline-Rat-Spleen-3d-dn	12/259	0,000435907	0,010796243	0	0	3,184333081	24,64062785	EEF1B2;C1QA;RPS17;RPL32;ARL2BP;RPS8;RPS5;FUNDC1;ARL6IP5;RPL13A;RPL35A;RPSA
33'5-Triiodo-L-Thyronine-0.001_mg/kg_in_PEG400-Rat-Heart-1d-dn	13/297	0,00043735	0,010798032	0	0	3,00831916	23,26867389	C1QA;RPL32;RPS8;RPS6;RPSA;RPS3A;RPL10A;RPS17;CXCL11;RPS19;ARL6IP5;RPL39;TPT1
Carbimazole-400_mg/kg_in_Water-Rat-Liver-5d-up	13/297	0,00043735	0,010764289	0	0	3,00831916	23,26867389	RPL32;RPS8;FEM1B;RPS5;RPS6;RPSA;RPS3A;RPL10A;SEPP1;RPS17;RPS19;RPS20;RPL39
Diethylstilbestrol-2.8_mg/kg_in_Corn_Oil-Rat-Liver-1d-up	13/297	0,00043735	0,010730755	0	0	3,00831916	23,26867389	RPL32;RPS8;FEM1B;RPS5;RPS6;RPSA;RPS3A;RPL10A;EEF1B2;RPS17;RPS19;RPS20;RPL39
N-Nitrosodiethylamine-1.67_mg/kg_in_Saline-Rat-Liver-0.25d-up	15/377	0,000445738	0,010902573	0	0	2,734556592	21,09923796	C1QA;RPL32;RPS8;RPS5;RPS6;RPL35A;RPSA;RPS3A;RPL10A;SEPP1;EEF1B2;RPS17;RPS19;RPS20;TPT1
Allopurinol-175_mg/kg_in_Corn_Oil-Rat-Kidney-1d-up	14/337	0,000448989	0,010948115	0	0	2,855190839	22,00927035	RPL32;RPS8;RPS5;RPS6;RPSA;RPS3A;RPL10A;EEF1B2;RPS17;RPS19;ARL2BP;ARL6IP5;RPS20;RPL39
Fluvastatin-94_mg/kg_in_Corn_Oil-Rat-Kidney-5d-up	14/337	0,000448989	0,010914324	0	0	2,855190839	22,00927035	RPL32;RPS8;RPS5;RPS6;RPL13A;RPL35A;RPS3A;RPL10A;SEPP1;TOMM20;EEF1B2;RPS17;RPS19;RPS20
Meloxicam-33_mg/kg_in_Corn_Oil-Rat-Kidney-1d-up	14/337	0,000448989	0,010880742	0	0	2,855190839	22,00927035	RPL32;RPS8;RPS5;RPS6;RPL13A;RPL35A;RPSA;RPS3A;EEF1B2;RPS17;RPS19;RPS20;RPL39;TPT1
Rifapentine-75_mg/kg_in_Corn_Oil-Rat-Kidney-1d-up	14/337	0,000448989	0,010847365	0	0	2,855190839	22,00927035	RPL32;RPS8;RPS6;RPL35A;RPSA;RPS3A;RPL10A;EEF1B2;RPS17;RPS19;ARL2BP;ARL6IP5;RPS20;TPT1
Tamoxifen-32_mg/kg_in_Corn_Oil-Rat-Liver-3d-up	14/337	0,000448989	0,010814193	0	0	2,855190839	22,00927035	RPL32;RPS8;RPS5;RPS6;RPL35A;RPSA;RPS3A;RPL10A;EEF1B2;RPS17;RPS19;RPS20;RPL39;TPT1
Amitriptyline-160_mg/kg_in_Water-Rat-Kidney-5d-dn	12/260	0,000451187	0,010833993	0	0	3,172085646	24,43656906	RPS17;RPL32;RPS19;RPS8;RPS5;RPS6;RPL35A;RPS20;RPSA;RPS3A;RPL39;SEPP1
Atenolol-1000_mg/kg_in_CMC-Rat-Heart-3d-up	13/298	0,000451462	0,010807636	0	0	2,998224129	23,09538065	RPL32;RPS8;RPS5;RPL13A;RPL35A;RPSA;RPS3A;TOMM20;RPS17;RPS19;FUNDC1;ARL6IP5;RAPGEF5
Lead_(II)_Acetate-600_mg/kg_in_Saline-Rat-Bone_marrow-5d-up	13/298	0,000451462	0,010774885	0	0	2,998224129	23,09538065	RPL32;RPS8;FEM1B;RPS5;RPS6;RPL35A;RPSA;RPS3A;RPS17;RPS19;FUNDC1;RPS20;TPT1
Oxiconazole-1500_mg/kg_in_Corn_Oil-Rat-Liver-1d-up	13/298	0,000451462	0,010742333	0	0	2,998224129	23,09538065	C1QA;RPL32;RPS8;RPS6;RPSA;RPS3A;EEF1B2;RPS17;RPS19;ARL2BP;RPS20;RPL39;TPT1
Ramipril-1500_mg/kg_in_CMC-Rat-Kidney-5d-up	15/378	0,000458197	0,01086976	0	0	2,727322315	20,96822979	RPL32;RPS5;RPS6;RPL13A;RPL35A;RPSA;RPS3A;SEPP1;TOMM20;EEF1B2;RPS17;CXCL11;ARL6IP5;RPS20;RPL39
Fenofibrate-215_mg/kg_in_Corn_Oil-Rat-Skeletal_muscle-3d-dn	13/299	0,000465957	0,011020664	0	0	2,988196623	22,92369984	RPL32;RPS8;FEM1B;RPS5;RPL35A;RPS3A;TOMM20;EEF1B2;RPS17;ARL6IP5;RPS20;RPL39;SLC16A3
Propylene_Glycol-2000_mg/kg_in_Water-Rat-Kidney-5d-dn	10/189	0,000465975	0,010988075	0	0	3,636429753	27,89643124	RPS17;RPL32;RPS19;RPS6;RPL35A;RPSA;RPS20;RPS3A;RPL10A;RPL39
Beta-Estradiol_3-Benzoate-25_mg/kg_in_Corn_Oil-Rat-Liver-1d-up	12/261	0,000466917	0,010977417	0	0	3,159932061	24,23465653	EEF1B2;RPS17;RPL32;RPS19;RPS8;FEM1B;RPS5;RPS6;RPL35A;RPSA;RPS3A;RPL10A
Vecuronium_Bromide-0.05_mg/kg_in_Saline-Rat-Liver-3d-dn	14/339	0,000476135	0,011160823	0	0	2,838346055	21,71280754	RPL32;RPS8;RPS6;RPL35A;RPSA;RPS3A;RPL10A;SEPP1;EEF1B2;RPS17;RPS19;RPS20;RPL39;TPT1
Zileuton-450_mg/kg_in_Corn_Oil-Rat-Kidney-0.25d-up	14/339	0,000476135	0,011127705	0	0	2,838346055	21,71280754	RPL32;RPS8;RPS5;RPL13A;RPSA;RPS3A;SEPP1;TOMM20;RPS17;RPS19;FUNDC1;RPS20;RPL39;TPT1
Chlortetracycline-1500_mg/kg_in_CMC-Rat-Liver-3d-up	13/301	0,000496137	0,011560864	0	0	2,968341496	22,58509888	RPL32;RPS8;RPS5;RPS6;RPL13A;RPL35A;RPSA;RPS3A;RPL10A;RPS17;RPS19;RPS20;RPL39
Cytarabine-23_mg/kg_in_Saline-Rat-Spleen-3d-dn	13/301	0,000496137	0,011526761	0	0	2,968341496	22,58509888	C1QA;RPL32;RPS8;RPS5;RPS6;RPL35A;RPSA;RPS3A;EEF1B2;RPS17;ARL2BP;ARL6IP5;RPL39
DActinomycin-0.06_mg/kg_in_Saline-Rat-Bone_marrow-5d-up	13/301	0,000496137	0,011492859	0	0	2,968341496	22,58509888	RPL32;RPS8;RPS5;RPS6;NKG7;RPSA;RPS3A;RPS17;RPS19;ARL2BP;ARL6IP5;RPS20;TPT1
Ethanol-3000_mg/kg_in_Saline-Rat-Kidney-0.25d-up	13/301	0,000496137	0,011459156	0	0	2,968341496	22,58509888	RPL32;RPS8;RPS5;RPL13A;RPL35A;RPS3A;TOMM20;EEF1B2;RPS17;RPS19;CLP1;RPS20;TPT1
Norethindrone-375_mg/kg_in_Corn_Oil-Rat-Liver-1d-up	13/301	0,000496137	0,011425649	0	0	2,968341496	22,58509888	RPL32;RPS8;FEM1B;RPS5;RPS6;RPSA;RPS3A;RPL10A;EEF1B2;RPS17;RPS19;RPS20;RPL39
Oxfendazole-1500_mg/kg_in_CMC-Rat-Liver-1d-dn	13/301	0,000496137	0,011392338	0	0	2,968341496	22,58509888	C1QA;RPL32;RPS8;RPS6;RPL35A;RPS3A;RPL10A;SEPP1;RPS17;CLP1;RPS20;RPL39;TPT1
Oxfendazole-1500_mg/kg_in_CMC-Rat-Liver-5d-dn	13/301	0,000496137	0,011359221	0	0	2,968341496	22,58509888	C1QA;RPL32;RPS8;RPL35A;RPSA;RPS3A;RPL10A;SEPP1;EEF1B2;RPS17;RPS19;ARL6IP5;TPT1
Pirinixic_Acid-364_mg/kg_in_CMC-Rat-Liver-5d-up	13/301	0,000496137	0,011326296	0	0	2,968341496	22,58509888	RPL32;RPS8;RPS5;RPS6;RPL35A;RPSA;RPS3A;RPL10A;EEF1B2;RPS17;RPS19;RPL39;TPT1
Terbutaline-130_mg/kg_in_Corn_Oil-Rat-Heart-1d-up	13/301	0,000496137	0,011293561	0	0	2,968341496	22,58509888	C1QA;RPL32;RPS8;RPS6;RPSA;RPS3A;RPL10A;EEF1B2;RPS17;RPS19;RPS20;RPL39;TPT1
Acetone-4000_mg/kg_in_Water-Rat-Kidney-1d-dn	11/226	0,000497938	0,011301896	0	0	3,345193565	25,44031566	EEF1B2;RPS17;RPL32;RPS19;RPS6;RPL35A;RPS20;RPSA;RPS3A;RPL10A;RPL39
Prednisolone-184_mg/kg_in_Water-Rat-Liver-3d-dn	12/263	0,000499766	0,011310797	0	0	3,13590216	23,83715414	EEF1B2;C1QA;RPS17;RPL32;RPS19;RPS8;RPS6;RPS20;RPS3A;RPL10A;RPL39;TPT1
4-Methylpyrazole-155_mg/kg_in_Saline-Rat-Liver-1d-up	14/341	0,000504667	0,011388982	0	0	2,821698864	21,421244	C1QA;RPL32;RPS8;RPS5;RPS6;RPL35A;RPSA;RPS3A;EEF1B2;RPS17;RPS19;ARL6IP5;RPS20;RPL39
Gentian_Violet-18_mg/kg_in_Corn_Oil-Rat-Liver-3d-dn	14/341	0,000504667	0,011356442	0	0	2,821698864	21,421244	RPL32;RPS8;RPS5;RPS6;RPL35A;RPSA;RPS3A;RPL10A;EEF1B2;RPS17;RPS19;RPS20;RPL39;TPT1
Netilmicin-40_mg/kg_in_Saline-Rat-Kidney-14d-up	14/341	0,000504667	0,011324088	0	0	2,821698864	21,421244	C1QA;RPL32;RPS8;RPS6;RPSA;RPS3A;RPL10A;RPS17;CXCL11;RPS19;ARL6IP5;RPS20;RPL39;TPT1
Carmustine-4_mg/kg_in_Corn_Oil-Rat-Bone_marrow-0.25d-dn	13/302	0,000511837	0,011452355	0	0	2,958512551	22,41814113	RPL32;RPS8;FEM1B;RPS5;RPS6;RPL35A;RPSA;RPS3A;EEF1B2;RPS17;ARL6IP5;RPS20;TPT1
Terfenadine-750_mg/kg_in_CMC-Rat-Heart-5d-up	13/302	0,000511837	0,011419912	0	0	2,958512551	22,41814113	RPL32;RPS8;RPS6;RPSA;RPS3A;RPL10A;EEF1B2;RPS19;ARL2BP;CLP1;RPS20;RAPGEF5;RPL39
Norethindrone-375_mg/kg_in_Corn_Oil-Rat-Liver-0.25d-up	12/264	0,000516908	0,011500463	0	0	3,124023743	23,64150747	EEF1B2;RPS17;RPS19;RPS8;FEM1B;RPS6;RPS20;RPSA;RPS3A;RPL10A;RPL39;GOLGA7
Ethanol-6000_mg/kg_in_Saline-Rat-Kidney-1d-up	14/342	0,00051947	0,011524927	0	0	2,813448283	21,27726725	RPL32;RPS5;RPS6;RPL13A;RPL35A;RPSA;RPS3A;EEF1B2;RPS17;RPS19;ARL6IP5;RPS20;RPL39;TPT1
Allyl_Alcohol-16_mg/kg_in_Saline-Rat-Liver-5d-dn	13/303	0,000527957	0,011680296	0	0	2,948748483	22,25272058	RPL32;RPS8;RPS5;RPS6;RPL35A;RPSA;RPS3A;RPL10A;EEF1B2;RPS17;RPS19;RPS20;RPL39
Carbon_Tetrachloride-400_mg/kg_in_Corn_Oil-Rat-Bone_marrow-1d-up	13/303	0,000527957	0,011647578	0	0	2,948748483	22,25272058	C1QA;RPL32;RPS8;RPS5;RPS6;RPL13A;RPL35A;RPS3A;RPS17;FUNDC1;RPS20;RPL39;TPT1

Megestrol_Acetate-132_mg/kg_in_CMC-Rat-Liver-5d-dn	13/303	0,000527957	0,011615043	0	0	2,948748483	22,25272058	RPL32;RPS8;RPS5;RPS6;RPL35A;RPS3A;RPL10A;SEPP1;EEF1B2;RPS17;RPS19;RPS20;TPT1
Simvastatin-160_uM_in_DMSO-Rat-Primary_rat_hepatocytes-0.67d-dn	13/303	0,000527957	0,011582689	0	0	2,948748483	22,25272058	RPS8;RPS5;RPSA;RPS3A;RPL10A;SEPP1;RPS17;CXCL11;RPS19;ARL6IP5;RPS20;GOLGA7;SLC16A3
Citalopram-90_mg/kg_in_Corn_Oil-Rat-Heart-3d-up	14/343	0,000534642	0,011696785	0	0	2,80524581	21,134477	RPS8;RPS6;RPSA;RPS3A;RPL10A;SEPP1;EEF1B2;RPS19;FUNDC1;ARL6IP5;RPS20;RAPGEF5;RPL39;TPT1
Ergocalciferol-15_mg/kg_in_CMC-Rat-Heart-3d-dn	14/343	0,000534642	0,011664384	0	0	2,80524581	21,134477	RPL32;RPS8;RPS6;RPS3A;RPL10A;SEPP1;RPS17;RPS19;ARL2BP;FUNDC1;ARL6IP5;RPS20;RPL39;TPT1
Prednisolone-184_mg/kg_in_Water-Rat-Heart-5d-dn	14/343	0,000534642	0,011632162	0	0	2,80524581	21,134477	RPL32;RPS8;RPS6;RPSA;RPS3A;RPL10A;SEPP1;RPS17;RPS19;ARL2BP;FUNDC1;RPS20;RPL39;TPT1
4-Nitrobenzoic_Acid-1470_mg/kg_in_CMC-Rat-Kidney-3d-up	13/304	0,000544504	0,011814081	0	0	2,939048653	22,08881914	RPS8;RPS5;RPS6;RPSA;RPS3A;RPL10A;SEPP1;EEF1B2;RPS17;RPS19;RPS20;RPL39;TPT1
Acetaminophen-486_mg/kg_in_Corn_Oil-Rat-Liver-3d-up	13/304	0,000544504	0,011781625	0	0	2,939048653	22,08881914	RPL32;RPS8;RPS5;RPS6;RPSA;RPS3A;RPL10A;SEPP1;RPS17;RPS19;ARL6IP5;RPS20;RPL39
Amiodarone-147_mg/kg_in_CMC-Rat-Liver-1d-dn	13/304	0,000544504	0,011749346	0	0	2,939048653	22,08881914	RPL32;RPS8;FEM1B;RPS6;RPL35A;RPS3A;RPL10A;SEPP1;EEF1B2;RPS17;RPS19;RPS20;RPL39
Clomiphene-250_mg/kg_in_CMC-Rat-Liver-1d-up	13/304	0,000544504	0,011717244	0	0	2,939048653	22,08881914	C1QA;RPL32;RPS8;RPS6;RPSA;RPS3A;RPL10A;PDRG1;EEF1B2;RPS17;RPS19;RPL39;TPT1
Daunorubicin-3.25_mg/kg_in_Saline-Rat-Bone_marrow-1d-dn	13/304	0,000544504	0,011685317	0	0	2,939048653	22,08881914	RPL32;RPS8;RPS5;RPS6;RPSA;RPS3A;RPL10A;EEF1B2;RPS17;FUNDC1;RPS20;RPL39;TPT1
Dobutamine-43_mg/kg_in_Saline-Rat-Heart-5d-up	13/304	0,000544504	0,011653564	0	0	2,939048653	22,08881914	RPL32;RPS5;RPS6;RPL35A;RPSA;RPS3A;RPL10A;SEPP1;EEF1B2;RPS17;RPS19;RPL39;TPT1
Fluconazole-394_mg/kg_in_Corn_Oil-Rat-Liver-5d-up	13/304	0,000544504	0,011621982	0	0	2,939048653	22,08881914	RPL32;RPS8;RPS5;RPS6;RPSA;RPS3A;RPL10A;RPS19;ARL2BP;RPS20;RPL39;GOLGA7;TPT1
Granisetron-175_mg/kg_in_Water-Rat-Liver-1d-up	13/304	0,000544504	0,011590571	0	0	2,939048653	22,08881914	RPL32;RPS8;RPS5;RPS6;RPL35A;RPSA;RPS3A;RPL10A;RPS17;RPS19;RPS20;RPL39;TPT1
SCH-351591-4.6_mg/kg_in_CMC-Rat-Liver-4d-up	13/304	0,000544504	0,01155933	0	0	2,939048653	22,08881914	RPL32;RPS8;RPS6;RPL35A;RPSA;RPS3A;RPL10A;EEF1B2;RPS17;RPS19;ARL6IP5;RPS20;RPL39
Indomethacin-12_mg/kg_in_Water-Rat-Liver-5d-up	14/344	0,00055019	0,011648637	0	0	2,797091025	20,99286084	RPL32;RPS8;RPS5;RPS6;RPSA;RPS3A;RPL10A;EEF1B2;RPS17;RPS19;ARL2BP;RPS20;RPL39;GOLGA7
Bezafibrate-100_mg/kg_in_Corn_Oil-Rat-Kidney-0.25d-dn	12/266	0,000552679	0,011669972	0	0	3,100534842	23,25628473	RPS17;RPL32;ARL2BP;RPS8;RPS5;RPS6;ARL6IP5;RPL35A;RPSA;SEPP1;TOMM20;TPT1
Procainamide-755_mg/kg_in_Water-Rat-Heart-1d-up	13/305	0,000561488	0,011824273	0	0	2,929412427	21,92641894	RPL32;RPS8;RPS5;RPL35A;RPSA;RPS3A;SEPP1;EEF1B2;RPS17;RPS19;ARL6IP5;RPS20;TPT1
Atorvastatin-250_uM_in_DMSO-Rat-Primary_rat_hepatocytes-1d-dn	14/345	0,00056612	0,011890026	0	0	2,788983515	20,85240652	RPL32;RPS8;RPS5;RPSA;RPL10A;SEPP1;EEF1B2;RPS17;CXCL11;RPS19;ARL6IP5;RPL39;SLC16A3;TPT1
NN-Dimethylformamide-140_mg/kg_in_Saline-Rat-Spleen-3d-dn	14/345	0,00056612	0,011858403	0	0	2,788983515	20,85240652	C1QA;RPL32;RPS8;RPS6;RPSA;RPS3A;RPL10A;EEF1B2;RPS17;ARL2BP;ARL6IP5;RPS20;RPL39;TPT1
Neostigmine_Bromide-40_mg/kg_in_Water-Rat-Liver-5d-dn	14/345	0,00056612	0,011826949	0	0	2,788983515	20,85240652	C1QA;RPL32;RPS8;RPL35A;RPSA;RPS3A;RPL10A;SEPP1;EEF1B2;RPS17;RPS19;RPS20;RPL39;TPT1
Bithionol-333_mg/kg_in_Corn_Oil-Rat-Bone_marrow-5d-up	13/306	0,000578918	0,012062323	0	0	2,919839184	21,76550242	C1QA;RPL32;FEM1B;RPS5;NKG7;RPL35A;RPSA;RPL10A;SEPP1;EEF1B2;RPS17;RPS20;TPT1
Primidone-750_mg/kg_in_CMC-Rat-Liver-5d-dn	13/306	0,000578918	0,012030496	0	0	2,919839184	21,76550242	C1QA;RPL32;RPS6;RPL35A;RPS3A;SEPP1;EEF1B2;RPS17;CLP1;ARL6IP5;RPS20;RPL39;TPT1
Sotalol-2000_mg/kg_in_Water-Rat-Heart-3d-up	13/306	0,000578918	0,011998837	0	0	2,919839184	21,76550242	RPS8;RPS6;RPSA;RPS3A;RPL10A;RPS17;RPS19;FUNDC1;ARL6IP5;RPS20;RAPGEF5;GOLGA7;TPT1
Sulindac-23_mg/kg_in_Corn_Oil-Rat-Kidney-3d-dn	14/346	0,000582441	0,012040161	0	0	2,780922869	20,71310192	RPL32;RPS8;RPS6;RPSA;RPS3A;RPL10A;SEPP1;TOMM20;EEF1B2;RPS17;RPS19;RPS20;RPL39;TPT1
Phenobarbital-80_mg/kg_in_Water-Rat-Liver-3d-dn	12/268	0,00059051	0,012175023	0	0	3,077396523	22,87897691	EEF1B2;C1QA;RPS17;RPL32;RPS19;RPS6;RPS20;RPSA;RPS3A;RPL10A;RPL39;TPT1
Chlordiazepoxide-196_mg/kg_in_Water-Rat-Heart-3d-up	13/307	0,000596803	0,012272648	0	0	2,910328307	21,60605224	RPL32;RPS8;RPS5;RPS6;RPL35A;RPSA;RPS3A;RPS17;RPS19;ARL2BP;FUNDC1;ARL6IP5;GOLGA7
Doxorubicin-3_mg/kg_in_Saline-Rat-Liver-1d-dn	13/307	0,000596803	0,012240688	0	0	2,910328307	21,60605224	C1QA;RPL32;RPS6;RPL35A;RPSA;RPS3A;RPL10A;EEF1B2;RPS17;RPS19;CLP1;RPL39;TPT1
Etoposide-188_mg/kg_in_Corn_Oil-Rat-Spleen-1d-dn	13/307	0,000596803	0,012208894	0	0	2,910328307	21,60605224	C1QA;RPL32;RPS8;RPS6;RPL35A;RPSA;RPS3A;RPL10A;EEF1B2;RPS17;RPS20;RPL39;TPT1
Melphalan-12_mg/kg_in_CMC-Rat-Bone_marrow-5d-dn	13/307	0,000596803	0,012177265	0	0	2,910328307	21,60605224	RPL32;RPS8;FEM1B;RPS5;RPS6;NKG7;RPSA;RPL10A;RPS17;RPS19;RPS20;RPL39;TPT1
Sildenafil-14.6_mg/kg_in_Saline-Rat-Liver-5d-dn	12/269	0,000610227	0,012418991	0	0	3,065956387	22,69322558	EEF1B2;C1QA;RPS17;RPL32;RPS19;RPS8;RPS6;RPSA;RPS3A;RPL10A;RPL39;TPT1
Buflomedil-615_mg/kg_in_CMC-Rat-Heart-1d-dn	13/308	0,000615154	0,012486988	0	0	2,90087919	21,44805133	C1QA;RPL32;RPS8;RPS6;RPSA;RPS3A;RPL10A;SEPP1;RPS17;RPS19;ARL2BP;RPS20;RPL39
Ethylene_Glycol-3525_mg/kg_in_Water-Rat-Bone_marrow-1d-dn	13/308	0,000615154	0,012454888	0	0	2,90087919	21,44805133	C1QA;RPL32;RPS8;RPS6;RPSA;RPS3A;RPL10A;SEPP1;RPS19;ARL6IP5;RPS20;RPL39;TPT1
Isoniazid-50_mg/kg_in_Saline-Rat-Liver-5d-dn	13/308	0,000615154	0,012422952	0	0	2,90087919	21,44805133	RPL32;RPS8;RPS5;RPS6;RPL35A;RPSA;RPS3A;RPL10A;EEF1B2;RPS17;RPS19;RPS20;RPL39
NN-Dimethylformamide-1400_mg/kg_in_Saline-Rat-Bone_marrow-1d-dn	13/308	0,000615154	0,01239118	0	0	2,90087919	21,44805133	RPL32;RPS8;FEM1B;RPS5;RPS6;RPSA;RPL10A;EEF1B2;RPS17;RPS19;RPS20;RPL39;TPT1
Phenytoin-46_mg/kg_in_Water-Rat-Heart-3d-up	13/308	0,000615154	0,01235957	0	0	2,90087919	21,44805133	RPL32;FEM1B;RPS6;RPL35A;RPSA;RPS3A;RPL10A;RPS17;RPS19;COL5A3;ARL6IP5;RPL39;TPT1
6-Methoxy-2-Naphthylacetic_Acid-360_mg/kg_in_Saline-Rat-Kidney-1d-up	14/348	0,000616284	0,012350773	0	0	2,764940554	20,4378943	RPL32;RPS8;RPS5;RPS6;RPL13A;RPL35A;RPSA;RPS3A;SEPP1;TOMM20;RPS17;RPS19;ARL6IP5;RPS20
Diethylstilbestrol-120_uM_in_DMSO-Rat-Primary_rat_hepatocytes-1d-dn	14/349	0,000633823	0,01267002	0	0	2,757018088	20,30196786	RPL32;RPS8;RPS5;RPS6;RPL35A;RPSA;RPL10A;SEPP1;RPS17;CXCL11;RPS19;ARL6IP5;RPL39;TPT1
Leflunomide-30_mg/kg_in_Corn_Oil-Rat-Bone_marrow-3d-up	13/309	0,000633978	0,012641049	0	0	2,891491231	21,29148287	C1QA;RPL32;RPS8;RPS5;NKG7;RPL35A;RPS3A;SEPP1;EEF1B2;RPS17;RPS20;RPL39;TPT1
Allyl_Alcohol-16_mg/kg_in_Saline-Rat-Bone_marrow-3d-up	15/391	0,000649396	0,012915772	0	0	2,636644079	19,35156298	C1QA;RPS8;FEM1B;RPS5;RPS6;RPL35A;RPSA;RPS3A;RPL10A;EEF1B2;RPS17;RPS19;RPS20;RPL39;TPT1
Labetalol-250_uM_in_DMSO-Rat-Primary_rat_hepatocytes-1d-up	12/271	0,000651323	0,012921462	0	0	3,043329402	22,32740082	EEF1B2;RPS17;RPL32;ARL2BP;RPS8;RPS5;RPS6;RPL35A;RPS20;RPS3A;RPL10A;PDRG1
Oxybutynin-230_mg/kg_in_Water-Rat-Heart-3d-up	14/350	0,000651783	0,012898099	0	0	2,749140893	20,16714431	RPL32;RPS5;RPS6;RPL35A;RPSA;RPS3A;RPL10A;SEPP1;TOMM20;RPS17;RPS19;ARL6IP5;RPS20;TPT1
Beta-Estradiol-150_mg/kg_in_Corn_Oil-Rat-Liver-1d-up	13/310	0,000653287	0,012895464	0	0	2,88216384	21,13633027	RPL32;RPS8;FEM1B;RPS5;RPS6;RPSA;RPS3A;RPL10A;EEF1B2;RPS17;RPS19;RPS20;RPL39
Carbon_Tetrachloride-3178_mg/kg_in_Saline-Rat-Liver-5d-up	13/310	0,000653287	0,012863226	0	0	2,88216384	21,13633027	C1QA;RPL32;RPS8;RPS6;RPSA;RPS3A;RPL10A;EEF1B2;RPS17;RPS19;RPS20;RPL39;TPT1
Fludrocortisone_Acetate-125_mg/kg_in_CMC-Rat-Liver-5d-dn	13/310	0,000653287	0,012831148	0	0	2,88216384	21,13633027	C1QA;RPL32;RPS8;RPS5;RPS6;RPL35A;RPS3A;RPL10A;SEPP1;EEF1B2;RPS17;RPS19;TPT1
NN'-Diphenyl-P-Phenylenediamine-1000_mg/kg_in_CMC-Rat-Kidney-1d-dn	13/310	0,000653287	0,012799229	0	0	2,88216384	21,13633027	RPL32;RPS8;RPS6;RPSA;RPL10A;SEPP1;TOMM20;PDRG1;RPS19;ARL2BP;ARL6IP5;RPS20;RPL39
Lead_(II)_Acetate-600_mg/kg_in_Saline-Rat-Bone_marrow-1d-dn	12/272	0,000672726	0,013147373	0	0	3,032140691	22,14727774	EEF1B2;RPS17;RPL32;RPS19;RPS5;NKG7;RPSA;RPS3A;RPL10A;RPL39;SEPP1;TPT1
Azauridine-1500_mg/kg_in_CMC-Rat-Bone_marrow-3d-up	13/311	0,00067309	0,013121922	0	0	2,872896432	20,9825772	C1QA;RPL32;RPS8;FEM1B;RPS6;RPSA;RPS3A;SEPP1;RPS17;RPS19;ARL2BP;RPS20;RPL39
Dexamethasone-1_mg/kg_in_Corn_Oil-Rat-Heart-5d-dn	13/311	0,00067309	0,013089522	0	0	2,872896432	20,9825772	RPL32;RPS8;RPS6;RPSA;RPS3A;RPL10A;RPS17;RPS19;ARL2BP;COL5A3;RPL39;GOLGA7;TPT1
Betamethasone-79_mg/kg_in_Corn_Oil-Rat-Liver-5d-dn	13/312	0,000693397	0,01345121	0	0	2,863688431	20,83020754	C1QA;RPL32;RPS8;RPS6;RPL35A;RPS3A;RPL10A;SEPP1;EEF1B2;RPS17;RPS19;RPS20;RPL39
Indomethacin-9.6_mg/kg_in_Water-Rat-Liver-0.25d-dn	12/273	0,000694716	0,013443691	0	0	3,021033949	21,96898179	EEF1B2;C1QA;RPS17;RPL32;RPS8;RPS5;RPS6;RPL35A;RPSA;RPL39;SEPP1;TPT1
Lead_(II)_Acetate-600_mg/kg_in_Saline-Rat-Liver-3d-up	12/273	0,000694716	0,013410741	0	0	3,021033949	21,96898179	EEF1B2;RPS17;RPL32;RPS8;RPS5;RPS6;RPL35A;RPS20;RPSA;RPL10A;TOMM20;TPT1
Methotrexate-27_mg/kg_in_Corn_Oil-Rat-Kidney-3d-up	12/273	0,000694716	0,013377952	0	0	3,021033949	21,96898179	RPS17;RPL32;RPS8;RPS5;RPS6;RPL35A;RPS20;RPSA;RPS3A;RAPGEF5;RPL10A;TPT1
Allyl_Alcohol-16_mg/kg_in_Saline-Rat-Bone_marrow-1d-dn	11/236	0,000713415	0,013704535	0	0	3,203448075	23,21041246	EEF1B2;RPS17;RPL32;RPS8;RPS5;RPS6;RPL13A;RPL35A;RPS20;RPSA;TPT1
Oxaliplatin-8_mg/kg_in_Saline-Rat-Kidney-1d-dn	11/236	0,000713415	0,013671191	0	0	3,203448075	23,21041246	EEF1B2;RPS17;RPL32;RPS19;RPL35A;RPS20;RPSA;RPS3A;RPL10A;RPL39;TPT1
Sulfaphenazole-1695_mg/kg_in_Water-Rat-Kidney-1d-dn	11/236	0,000713415	0,013638008	0	0	3,203448075	23,21041246	RPS17;RPL32;RPS19;ARL2BP;RPS6;ARL6IP5;RPL35A;RPS20;RPSA;RPS3A;RPL39
Levamisole-120_mg/kg_in_Water-Rat-Liver-5d-up	13/313	0,000714217	0,013620279	0	0	2,854539266	20,67920544	RPL32;RPS8;RPS5;RPS6;RPL13A;RPL35A;RPSA;RPS3A;RPL10A;SEPP1;RPS17;RPS19;RPS20
Thiabendazole-92_mg/kg_in_Corn_Oil-Rat-Liver-3d-up	13/313	0,000714217	0,01358738	0	0	2,854539266	20,67920544	RPL32;RPS6;RPL35A;RPS3A;RPL10A;SEPP1;EEF1B2;RPS17;RPS19;CLP1;COL5A3;RPS20;RPL39
Amantadine-220_mg/kg_in_Water-Rat-Liver-1d-up	13/314	0,000735562	0,013959732	0	0	2,845448377	20,52955523	C1QA;RPL32;RPS8;FEM1B;RPS6;RPL35A;RPSA;RPS3A;RPS17;RPS19;RPS20;RPL39;TPT1
Cefotaxime-1000_mg/kg_in_Saline-Rat-Kidney-5d-up	13/314	0,000735562	0,013926175	0	0	2,845448377	20,52955523	RPL32;RPS8;RPS5;RPL13A;RPL35A;RPSA;TOMM20;RPS17;CXCL11;ARL6IP5;RPS20;RPL39;TPT1
Nitrazepam-310_mg/kg_in_CMC-Rat-Liver-1d-dn	13/314	0,000735562	0,013892779	0	0	2,845448377	20,52955523	C1QA;RPL32;RPL35A;RPSA;RPL10A;SEPP1;EEF1B2;RPS19;CLP1;ARL6IP5;RPS20;RPL39;TPT1
Gatifloxacin-770_mg/kg_in_Corn_Oil-Rat-Heart-1d-dn	13/315	0,000757442	0,014271804	0	0	2,836415208	20,38124151	RPL32;RPS8;RPS5;RPS6;RPL35A;RPSA;RPS3A;EEF1B2;RPS17;ARL2BP;RPS20;RPL39;TPT1
Itraconazole-30_mg/kg_in_Corn_Oil-Rat-Liver-1d-up	13/315	0,000757442	0,014237742	0	0	2,836415208	20,38124151	RPL32;RPS8;RPS5;RPS6;RPSA;RPS3A;RPL10A;EEF1B2;RPS17;RPS19;RPS20;RPL39;TPT1
Loratadine-10_mg/kg_in_Water-Rat-Heart-3d-up	13/315	0,000757442	0,014203843	0	0	2,836415208	20,38124151	RPL32;RPS8;RPS5;RPS6;RPL35A;RPSA;RPS3A;RPS17;RPS19;ARL2BP;RPS20;RAPGEF5;GOLGA7
NN-Dimethylformamide-140_mg/kg_in_Saline-Rat-Kidney-1d-up	13/315	0,000757442	0,014170104	0	0	2,836415208	20,38124151	RPL32;RPS8;RPS5;RPS6;RPL13A;RPL35A;RPSA;RPS3A;RPS17;RPS19;RPS20;RPL39;TPT1
Atorvastatin-300_mg/kg_in_Corn_Oil-Rat-Liver-5d-up	12/276	0,000764331	0,014265103	0	0	2,988196623	21,44482046	EEF1B2;RPS17;CXADR;RPS19;RPS8;RPS5;RPS6;RPS20;RPSA;RPS3A;RPL10A;RPL39
6-Mercaptopurine-25_mg/kg_in_Corn_Oil-Rat-Kidney-3d-dn	13/316	0,000779867	0,014520647	0	0	2,82743921	20,23424906	RPL32;RPS8;RPS6;RPSA;RPS3A;SEPP1;EEF1B2;RPS17;RPS19;ARL2BP;RPS20;RPL39;TPT1
Bithionol-333_mg/kg_in_Corn_Oil-Rat-Bone_marrow-1d-up	13/316	0,000779867	0,014486401	0	0	2,82743921	20,23424906	C1QA;RPL32;RPS5;NKG7;RPSA;RPL10A;SEPP1;EEF1B2;RPS17;RPS19;RPS20;RPL39;TPT1
N-Nitrosodiethylamine-7000_uM_in_DMSO-Rat-Primary_rat_hepatocytes-0.67d-up	13/316	0,000779867	0,014452315	0	0	2,82743921	20,23424906	RPL32;RPS8;FEM1B;RPS5;RPL35A;RPSA;PDRG1;EEF1B2;CXCL11;CLP1;RPS20;RPL39;GOLGA7
Spironolactone-300_mg/kg_in_CMC-Rat-Liver-5d-dn	13/316	0,000779867	0,014418389	0	0	2,82743921	20,23424906	C1QA;RPL32;RPS5;RPS6;RPL35A;RPSA;RPS3A;EEF1B2;RPS17;RPS19;RPS20;RPL39;TPT1
Thioridazine-63_mg/kg_in_Corn_Oil-Rat-Heart-3d-dn	13/316	0,000779867	0,014384623	0	0	2,82743921	20,23424906	RPL32;RPS8;RPS6;RPSA;RPS3A;EEF1B2;RPS17;RPS19;CLP1;R3HCC1;ARL6IP5;RPL39;TPT1
Indomethacin-12_mg/kg_in_Water-Rat-Liver-3d-up	12/277	0,000788795	0,014515303	0	0	2,97740891	21,2735987	EEF1B2;RPS17;RPL32;RPS19;RPS8;RPS5;RPS6;RPS20;RPSA;RPS3A;RPL10A;RPL39
Zomepirac-2.8_mg/kg_in_Saline-Rat-Liver-3d-up	12/277	0,000788795	0,014481468	0	0	2,97740891	21,2735987	EEF1B2;RPS17;CXADR;RPL32;RPS19;RPS8;RPS5;RPL35A;RPS20;RPSA;RPS3A;TOMM20
2-Amino-4-Nitrophenol-625_mg/kg_in_CMC-Rat-Kidney-5d-up	13/317	0,000802848	0,014705192	0	0	2,818519843	20,08856291	C1QA;RPL32;RPS8;RPS6;RPSA;RPS3A;RPL10A;SEPP1;EEF1B2;RPS17;RPS19;RPL39;TPT1
Cholecalciferol-8_mg/kg_in_CMC-Rat-Heart-3d-dn	13/317	0,000802848	0,014671074	0	0	2,818519843	20,08856291	RPL32;RPS8;RPS6;RPSA;RPS3A;SEPP1;RPS17;RPS19;FUNDC1;ARL6IP5;RPS20;RPL39;TPT1
Sodium_Arsenite-30_mg/kg_in_Saline-Rat-Kidney-1d-up	13/317	0,000802848	0,014637113	0	0	2,818519843	20,08856291	RPL32;RPS8;RPS5;RPS6;RPL13A;RPL35A;RPS3A;SEPP1;EEF1B2;RPS17;RPS19;ARL6IP5;RPS20
Tolazamide-1500_mg/kg_in_CMC-Rat-Liver-1d-up	13/317	0,000802848	0,014603309	0	0	2,818519843	20,08856291	RPL32;RPS8;RPS5;RPS6;RPL35A;RPSA;RPS3A;RPL10A;EEF1B2;RPS17;RPS19;RPS20;TPT1
Amiloride-22_mg/kg_in_CMC-Rat-Heart-5d-up	12/278	0,00081391	0,014770404	0	0	2,966698806	21,10408881	EEF1B2;RPS17;RPL32;ARL2BP;RPS8;RPS5;FUNDC1;RPS6;RPL35A;RPS3A;TOMM20;TPT1
Naproxen-134_mg/kg_in_Corn_Oil-Rat-Kidney-3d-up	12/278	0,00081391	0,014736449	0	0	2,966698806	21,10408881	EEF1B2;C1QA;RPS17;RPL32;RPS8;RPS6;RPL13A;RPL35A;RPS20;RPS3A;RAPGEF5;RPL39
Carmustine-4_mg/kg_in_Corn_Oil-Rat-Spleen-0.25d-up	13/318	0,000826396	0,014928202	0	0	2,809656574	19,94416827	RPL32;RPS8;RPS6;RPL13A;RPL35A;RPSA;RPS3A;RPL10A;RPS17;FUNDC1;ARL6IP5;RPS20;RPL39
Cholecalciferol-8_mg/kg_in_CMC-Rat-Liver-1d-up	13/318	0,000826396	0,014894041	0	0	2,809656574	19,94416827	C1QA;RPL32;RPS8;RPS5;RPS6;RPL35A;RPS3A;RPL10A;EEF1B2;RPS17;RPS19;RPS20;TPT1
Letrozole-250_mg/kg_in_Corn_Oil-Rat-Liver-1d-dn	13/318	0,000826396	0,014860036	0	0	2,809656574	19,94416827	RPL32;RPS8;RPS6;RPL35A;RPSA;RPS3A;RPL10A;EEF1B2;RPS17;RPS19;RPS20;RPL39;TPT1
Lomustine-4.2_mg/kg_in_Corn_Oil-Rat-Bone_marrow-3d-up	13/318	0,000826396	0,014826187	0	0	2,809656574	19,94416827	C1QA;RPL32;RPS8;RPS5;RPL13A;RPL35A;RPS3A;SEPP1;EEF1B2;RPS17;RPS20;RPL39;TPT1
Methapyrilene-100_mg/kg_in_CMC-Rat-Liver-3d-up	13/318	0,000826396	0,014792491	0	0	2,809656574	19,94416827	RPL32;RPS8;RPS5;RPS6;RPSA;RPS3A;RPL10A;SEPP1;EEF1B2;RPS17;RPS19;RPS20;RPL39
N-Nitrosodiethylamine-34_mg/kg_in_Saline-Rat-Kidney-1d-up	13/318	0,000826396	0,014758948	0	0	2,809656574	19,94416827	RPL32;RPS8;RPS5;RPS6;RPL13A;RPL35A;RPSA;RPS3A;SEPP1;TOMM20;EEF1B2;RPS17;RPS20
Verapamil-108_mg/kg_in_Water-Rat-Heart-1d-up	13/318	0,000826396	0,014725556	0	0	2,809656574	19,94416827	RPL32;RPS8;RPS5;RPS6;RPL35A;RPSA;RPS3A;RPL10A;RPS17;RPS19;ARL6IP5;RPS20;TPT1
Haloperidol-21_mg/kg_in_Corn_Oil-Rat-Heart-5d-up	14/359	0,000833779	0,01482358	0	0	2,680220927	19,00153833	RPL32;RPS8;RPS3A;RPL10A;SEPP1;EEF1B2;RPS19;ARL2BP;FUNDC1;ARL6IP5;RPS20;RAPGEF5;RPL39;TPT1
Dicumarol-13_mg/kg_in_Corn_Oil-Rat-Liver-1d-dn	12/279	0,00083969	0,014895038	0	0	2,956065477	20,93626884	EEF1B2;RPL32;RPS19;RPS8;RPS5;RPS6;RPL35A;RPSA;RPS3A;RPL10A;RPL39;GOLGA7
Amitraz-75_mg/kg_in_CMC-Rat-Heart-1d-dn	13/319	0,000850522	0,015053281	0	0	2,800848873	19,80105058	C1QA;RPL32;RPS8;FEM1B;RPSA;RPS3A;RPL10A;EEF1B2;RPS17;RPS19;ARL2BP;RPS20;RPL39
Ethosuximide-1200_mg/kg_in_Water-Rat-Heart-3d-dn	13/319	0,000850522	0,015019529	0	0	2,800848873	19,80105058	RPL32;RPS8;RPS6;RPSA;RPS3A;RPL10A;SEPP1;EEF1B2;RPS17;RPS19;RPS20;RPL39;TPT1
Sulfinpyrazone-269_mg/kg_in_CMC-Rat-Kidney-5d-up	13/319	0,000850522	0,014985928	0	0	2,800848873	19,80105058	RPL32;RPS6;RPSA;RPL10A;SEPP1;RPS17;CXCL11;RPS19;ARL6IP5;RPS20;RAPGEF5;RPL39;GOLGA7

Valproic_Acid-850_mg/kg_in_Water-Rat-Bone_marrow-5d-up	13/319	0,000850522	0,014952478	0	0	2,800848873	19,80105058	RPL32;RPS5;NKG7;RPL35A;RPS3A;RPL10A;SEPP1;EEF1B2;RPS17;RPS19;RPS20;RPL39;TPT1
Cadmium_Acetate-167_mg/kg_in_Water-Rat-Kidney-5d-up	12/280	0,000866148	0,015193277	0	0	2,9455081	20,77011718	RPS17;RPL32;ARL2BP;RPS6;RPS20;RPSA;RPS3A;RAPGEF5;RPL10A;RPL39;SEPP1;TPT1
Carbon_Tetrachloride-400_mg/kg_in_Corn_Oil-Rat-Liver-3d-up	12/280	0,000866148	0,015159514	0	0	2,9455081	20,77011718	EEF1B2;C1QA;RPS17;RPL32;RPS19;RPS8;RPS5;RPS20;RPSA;RPS3A;RPL39;SEPP1
Nystatin-134_mg/kg_in_Corn_Oil-Rat-Liver-1d-up	12/280	0,000866148	0,015125901	0	0	2,9455081	20,77011718	EEF1B2;C1QA;RPS17;RPL32;RPS19;ARL2BP;RPS8;RPS20;RPSA;RPL39;SEPP1;TPT1
BW-723C86-50_mg/kg_in_Saline-Rat-Heart-5d-up	13/320	0,000875236	0,015250802	0	0	2,79209622	19,65919548	C1QA;RPS8;RPS6;RPS3A;RPL10A;SEPP1;EEF1B2;RPS17;RPS19;ARL2BP;ARL6IP5;RPS20;RPL39
Cobalt(II)_Acetylacetonate-100_mg/kg_in_Corn_Oil-Rat-Kidney-5d-up	13/320	0,000875236	0,015217135	0	0	2,79209622	19,65919548	RPL32;RPS8;RPS5;RPS6;RPL35A;RPSA;RPS3A;PDRG1;EEF1B2;RPS17;ARL6IP5;RPS20;RPL39
Dicyclomine-645_mg/kg_in_Water-Rat-Heart-1d-dn	13/320	0,000875236	0,015183617	0	0	2,79209622	19,65919548	RPL32;RPS8;RPS6;RPSA;RPS3A;RPL10A;EEF1B2;RPS17;RPS19;ARL2BP;FUNDC1;RPS20;RPL39
Erythromycin-1500_mg/kg_in_CMC-Rat-Liver-5d-up	13/320	0,000875236	0,015150247	0	0	2,79209622	19,65919548	C1QA;RPL32;RPS8;RPS5;RPS6;RPSA;RPS3A;RPL10A;EEF1B2;RPS19;ARL2BP;RPS20;TPT1
Kanamycin-6584_uM_in_DMSO-Rat-Primary_rat_hepatocytes-0.67d-up	13/320	0,000875236	0,015117023	0	0	2,79209622	19,65919548	RPL32;FEM1B;RPS6;RPL35A;RPS3A;RPL10A;EEF1B2;RPS17;CXCL11;CLP1;RPS20;RPL39;TPT1
Sulindac-750_uM_in_DMSO-Rat-Primary_rat_hepatocytes-0.67d-dn	13/320	0,000875236	0,015083944	0	0	2,79209622	19,65919548	RPL32;RPS8;RPS5;RPSA;RPL10A;SEPP1;CXCL11;RPS19;FUNDC1;ARL6IP5;RPS20;GOLGA7;TPT1
Sumatriptan-1100_mg/kg_in_Water-Rat-Brain-3d-up	13/320	0,000875236	0,015051009	0	0	2,79209622	19,65919548	C1QA;RPL32;RPS8;FEM1B;RPL35A;RPS3A;RPL10A;SEPP1;EEF1B2;RPS17;RPS19;RPL39;TPT1
Tegafur-175_mg/kg_in_Water-Rat-Bone_marrow-1d-dn	13/320	0,000875236	0,015018219	0	0	2,79209622	19,65919548	C1QA;RPL32;RPS8;RPS5;RPL35A;RPSA;RPS3A;EEF1B2;RPS17;ARL6IP5;RPS20;RPL39;TPT1
Netilmicin-41_mg/kg_in_Saline-Rat-Kidney-5d-up	14/361	0,00087958	0,015059938	0	0	2,665372057	18,75373411	RPL32;RPS5;RPS6;RPL35A;RPSA;SEPP1;TOMM20;RPS17;RPS19;CLP1;FUNDC1;ARL6IP5;RAPGEF5;TPT1
Enalapril-1500_mg/kg_in_CMC-Rat-Kidney-1d-up	12/281	0,000893298	0,015261646	0	0	2,935025865	20,6056126	EEF1B2;RPL32;RPS19;ARL2BP;RPS8;RPS6;RPS20;RPSA;RPS3A;RPL10A;RPL39;TPT1
Mifepristone-3_mg/kg_in_Corn_Oil-Rat-Liver-3d-up	12/281	0,000893298	0,015228612	0	0	2,935025865	20,6056126	EEF1B2;RPS17;RPL32;RPS19;RPS8;RPS5;RPL35A;RPS20;RPS3A;RPL39;SEPP1;TPT1
Nimesulide-162_mg/kg_in_Corn_Oil-Rat-Liver-1d-up	12/281	0,000893298	0,015195721	0	0	2,935025865	20,6056126	EEF1B2;RPS17;RPS19;RPS8;RPS5;RPS20;RPSA;RPS3A;RPL10A;RPL39;SEPP1;TPT1
Roxarsone-11_mg/kg_in_Corn_Oil-Rat-Kidney-1d-up	12/281	0,000893298	0,015162972	0	0	2,935025865	20,6056126	RPS17;RPL32;RPS19;RPS8;RPS5;RPS6;RPL35A;RPSA;RPS3A;RPL10A;RPL39;TPT1
Ascorbic_Acid-2000_mg/kg_in_Water-Rat-Kidney-1d-dn	13/321	0,000900551	0,015253208	0	0	2,783398101	19,51858881	RPL32;RPL35A;RPSA;RPS3A;SEPP1;TOMM20;EEF1B2;ARL2BP;FUNDC1;ARL6IP5;RPS20;RPL39;TPT1
Cholic_Acid-1402_mg/kg_in_Water-Rat-Liver-5d-up	13/321	0,000900551	0,015220476	0	0	2,783398101	19,51858881	RPL32;RPS8;RPS6;RPL35A;RPS3A;RPL10A;SEPP1;EEF1B2;RPS17;RPS19;ARL6IP5;RPS20;RPL39
Rapamycin-60_mg/kg_in_CMC-Rat-Kidney-3d-up	13/321	0,000900551	0,015187884	0	0	2,783398101	19,51858881	RPS8;RPS5;RPS6;RPL13A;RPSA;RPS3A;EEF1B2;RPS17;RPS19;ARL2BP;RPS20;RPL39;TPT1
Clofibrate-130_mg/kg_in_Corn_Oil-Rat-Kidney-0.25d-dn	12/282	0,000921155	0,015502178	0	0	2,924617972	20,44273419	EEF1B2;RPS17;RPL32;ARL2BP;RPS8;RPS5;RPS6;ARL6IP5;RPL35A;RPSA;TOMM20;TPT1
Noscapine-570_mg/kg_in_CMC-Rat-Bone_marrow-1d-dn	12/282	0,000921155	0,015469124	0	0	2,924617972	20,44273419	RPS17;RPL32;RPS19;ARL2BP;RPS8;FEM1B;RPS5;FUNDC1;RPS6;RPL35A;RPS20;RPS3A
Pramoxine-526_mg/kg_in_Corn_Oil-Rat-Liver-1d-up	12/282	0,000921155	0,015436211	0	0	2,924617972	20,44273419	EEF1B2;C1QA;RPS17;RPL32;RPS19;ARL2BP;RPS8;RPS20;RPSA;RPL10A;RPL39;SEPP1
Vinblastine-45_uM_in_DMSO-Rat-Primary_rat_hepatocytes-1d-up	12/282	0,000921155	0,015403438	0	0	2,924617972	20,44273419	EEF1B2;RPS17;ARL2BP;RPS8;FEM1B;RPS5;RPS6;RPS3A;RPL10A;GOLGA7;TPT1;PDRG1
Chlorobenzene-500_mg/kg_in_CMC-Rat-Heart-5d-up	13/322	0,000926478	0,015459615	0	0	2,774754007	19,37921662	RPL32;RPS8;RPS5;RPSA;RPS3A;RPL10A;TOMM20;PDRG1;EEF1B2;RPS17;ARL2BP;RPS20;RAPGEF5
Famciclovir-112_mg/kg_in_Saline-Rat-Liver-0.25d-up	13/322	0,000926478	0,015426931	0	0	2,774754007	19,37921662	C1QA;RPL32;RPS8;RPS5;RPS6;RPL35A;RPSA;RPS3A;SEPP1;EEF1B2;RPS17;RPS20;RPL39
Ketoprofen-4_mg/kg_in_Corn_Oil-Rat-Kidney-0.25d-up	13/322	0,000926478	0,015394384	0	0	2,774754007	19,37921662	C1QA;RPL32;RPS8;RPS6;RPSA;RPS3A;RPL10A;SEPP1;RPS17;RPS19;RPS20;RAPGEF5;TPT1
4-Octylphenol-50_mg/kg_in_Corn_Oil-Rat-Liver-1d-dn	12/283	0,000949733	0,015747568	0	0	2,914283633	20,28146136	EEF1B2;RPS17;RPL32;RPS19;RPS8;FEM1B;RPS6;RPS20;RPS3A;RPL10A;GOLGA7;TPT1
Gemfibrozil-700_mg/kg_in_Corn_Oil-Rat-Kidney-1d-dn	12/283	0,000949733	0,015714485	0	0	2,914283633	20,28146136	EEF1B2;RPS17;RPL32;RPS8;RPS6;RPL35A;RPS20;RPSA;RPS3A;RPL10A;RPL39;TOMM20
Phenacemide-400_mg/kg_in_CMC-Rat-Heart-3d-dn	13/323	0,000953027	0,015735938	0	0	2,766163438	19,24106513	RPL32;RPS8;RPS6;RPSA;RPS3A;RPL10A;SEPP1;PDRG1;RPS17;RPS19;ARL6IP5;RPS20;RPL39
Tetracycline-520_uM_in_DMSO-Rat-Primary_rat_hepatocytes-1d-up	13/323	0,000953027	0,015703018	0	0	2,766163438	19,24106513	RPL32;RPS8;RPS6;RPL35A;RPSA;RPS3A;RPL10A;PDRG1;EEF1B2;RPS17;ARL2BP;RPL39;TPT1
Daunorubicin-3.25_mg/kg_in_Saline-Rat-Kidney-3d-dn	12/284	0,000979045	0,016098036	0	0	2,904022071	20,12177386	EEF1B2;RPS17;RPL32;RPS8;RPS5;FUNDC1;ARL6IP5;RPL35A;RPS20;RPSA;RPS3A;RPL39
Doxapram-2_mg/kg_in_Saline-Rat-Liver-3d-up	12/284	0,000979045	0,016064499	0	0	2,904022071	20,12177386	EEF1B2;C1QA;RPS17;RPL32;RPS19;RPS5;RPS6;ARL6IP5;RPL35A;RPS20;RPSA;RPL39
Theophylline-225_mg/kg_in_Water-Rat-Heart-1d-up	12/284	0,000979045	0,016031101	0	0	2,904022071	20,12177386	EEF1B2;C1QA;RPS17;RPS19;ARL2BP;RPS8;RPS6;RPS20;RPS3A;RAPGEF5;RPL39;TPT1
Busulfan-9_mg/kg_in_Corn_Oil-Rat-Liver-5d-up	13/324	0,000980212	0,016016903	0	0	2,757625896	19,10412077	C1QA;RPL32;RPS8;RPS5;RPS6;RPL35A;RPS3A;RPL10A;EEF1B2;RPS17;RPS19;RPS20;RPL39
Daunorubicin-3.25_mg/kg_in_Saline-Rat-Liver-1d-dn	13/324	0,000980212	0,015983742	0	0	2,757625896	19,10412077	C1QA;RPL32;RPS6;RPL35A;RPSA;RPS3A;RPL10A;EEF1B2;RPS17;RPS19;ARL6IP5;RPS20;RPL39
Lipopolysaccharide_E._Coli_O55:B5-1.25_mg/kg_in_Saline-Rat-Liver-5d-up	13/324	0,000980212	0,015950717	0	0	2,757625896	19,10412077	C1QA;RPL32;RPS8;RPS6;RPL35A;RPSA;RPS3A;RPL10A;SEPP1;RPS17;RPS19;RPS20;RPL39
Phenobarbital-25_mg/kg_in_Water-Rat-Liver-0.25d-up	13/324	0,000980212	0,015917829	0	0	2,757625896	19,10412077	C1QA;RPL32;RPS8;RPS5;RPS6;RPL35A;RPSA;RPS3A;RPL10A;SEPP1;RPS17;RPS19;RPS20;RPL39
Clindamycin-161_mg/kg_in_Saline-Rat-Kidney-1d-up	14/366	0,001003459	0,016261811	0	0	2,628959871	18,15113456	RPL32;RPS8;RPS5;RPS6;RPL13A;RPL35A;RPSA;RPS3A;SEPP1;TOMM20;RPS17;ARL6IP5;RPS20;TPT1
Amoxapine-313_mg/kg_in_CMC-Rat-Liver-3d-up	13/325	0,001008043	0,016302557	0	0	2,749140893	18,96837016	RPL32;RPS8;RPS5;RPS6;RPL13A;RPL35A;RPSA;RPS3A;RPL10A;RPS17;RPS19;RPS20;RPL39
Cerivastatin-7_mg/kg_in_Corn_Oil-Rat-Skeletal_muscle-3d-dn	13/325	0,001008043	0,01626915	0	0	2,749140893	18,96837016	RPL32;RPS6;RPSA;RPS3A;RPL10A;EEF1B2;RPS17;RPS19;COL5A3;ARL6IP5;RPS20;RPL39;SLC16A3
Indomethacin-9.6_mg/kg_in_Water-Rat-Kidney-5d-up	13/325	0,001008043	0,01623588	0	0	2,749140893	18,96837016	C1QA;RPL32;RPS8;RPS5;RPS6;RPL13A;RPL35A;RPS3A;EEF1B2;RPS17;RPS20;RPL39;TPT1
Valdecoxib-404_mg/kg_in_CMC-Rat-Kidney-1d-up	13/325	0,001008043	0,016202745	0	0	2,749140893	18,96837016	RPL32;RPS8;RPS5;RPS6;RPL13A;RPL35A;RPSA;RPS3A;EEF1B2;RPS17;RPS19;ARL6IP5;RPS20
Acetaminophen-972_mg/kg_in_Corn_Oil-Rat-Liver-5d-up	12/285	0,001009107	0,016186818	0	0	2,893832519	19,96365177	EEF1B2;RPS17;RPL32;RPS19;RPS8;RPS5;RPS6;RPS20;RPSA;RPS3A;RPL10A;TPT1
Fluconazole-394_mg/kg_in_Corn_Oil-Rat-Liver-0.25d-up	12/285	0,001009107	0,016153917	0	0	2,893832519	19,96365177	EEF1B2;RPS17;RPL32;RPS19;RPS8;RPS6;RPS20;RPSA;RPS3A;RPL10A;RPL39;TPT1
Diclofenac-10_mg/kg_in_Corn_Oil-Rat-Kidney-3d-up	14/367	0,001029919	0,016453636	0	0	2,621796492	18,03343748	C1QA;RPL32;RPS8;RPS5;RPS6;RPL13A;RPL35A;TOMM20;EEF1B2;RPS17;RPS20;RPL39;GOLGA7;TPT1
Cisplatin-63_uM_in_DMSO-Rat-Primary_rat_hepatocytes-0.67d-up	13/326	0,001036533	0,016525771	0	0	2,740707946	18,83380009	RPL32;RPS6;RPSA;RPL10A;PDRG1;EEF1B2;RPS17;CXCL11;ARL2BP;CLP1;ARL6IP5;RPL39;GOLGA7
Cytarabine-487_mg/kg_in_Saline-Rat-Spleen-5d-up	13/326	0,001036533	0,016492386	0	0	2,740707946	18,83380009	RPL32;RPS8;RPS5;RPL13A;RPL35A;RPSA;SEPP1;EEF1B2;RPS17;CXCL11;RPS19;FUNDC1;ARL6IP5
Mifepristone-300_mg/kg_in_Corn_Oil-Rat-Liver-5d-dn	13/326	0,001036533	0,016459135	0	0	2,740707946	18,83380009	C1QA;RPL32;RPS8;RPS6;RPSA;RPS3A;RPL10A;SEPP1;EEF1B2;RPS17;RPS19;RPS20;RPL39
Clemastine-2500_mg/kg_in_CMC-Rat-Heart-3d-up	12/286	0,001039933	0,016479911	0	0	2,883714224	19,80707545	C1QA;RPS17;RPL32;RPS19;RPS5;RPS6;RPL35A;RPSA;RPS3A;RPL10A;RPL39;TPT1
Valproic_Acid-1500_mg/kg_in_Water-Rat-Heart-3d-dn	12/286	0,001039933	0,016446818	0	0	2,883714224	19,80707545	EEF1B2;RPS17;RPL32;RPS8;COL5A3;RPS6;RPL35A;RPS20;RPSA;RPL39;TOMM20;TPT1
Cefotaxime-1000_mg/kg_in_Saline-Rat-Kidney-1d-up	14/368	0,001056964	0,016682665	0	0	2,614672045	17,91665974	C1QA;RPL32;RPS8;RPS5;RPS6;RPL13A;RPL35A;RPSA;RPS3A;TOMM20;RPS17;ARL6IP5;RPS20;TPT1
Chlortetracycline-1500_mg/kg_in_CMC-Rat-Liver-5d-up	13/327	0,001065693	0,016786801	0	0	2,732326576	18,70039754	C1QA;RPL32;RPS8;RPS6;RPL35A;RPSA;RPS3A;RPL10A;RPS17;RPS19;RPS20;RPL39;TPT1
Dexfenfluramine-29_mg/kg_in_Water-Rat-Heart-1d-dn	13/327	0,001065693	0,016753294	0	0	2,732326576	18,70039754	RPL32;RPS8;RPS6;RPSA;RPS3A;RPL10A;EEF1B2;RPS17;RPS19;COL5A3;RPS20;RPL39;TPT1
Procainamide-755_mg/kg_in_Water-Rat-Heart-3d-up	13/327	0,001065693	0,016719921	0	0	2,732326576	18,70039754	RPL32;RPS8;RPS5;RPL35A;RPSA;RPL10A;SEPP1;TOMM20;EEF1B2;RPS17;COL5A3;ARL6IP5;RPS20
Coumarin-111_mg/kg_in_Corn_Oil-Rat-Bone_marrow-1d-dn	12/287	0,001071539	0,016778214	0	0	2,873666439	19,65202561	EEF1B2;RPS17;RPL32;ARL2BP;RPS8;RPS5;RPS6;ARL6IP5;RPL35A;RPS20;RPSA;RPS3A
Isoniazid-79_mg/kg_in_Water-Rat-Liver-1d-up	12/287	0,001071539	0,016744924	0	0	2,873666439	19,65202561	EEF1B2;RPS17;RPL32;RPS19;RPS5;RPS6;RPL35A;RPS20;RPSA;RPS3A;RPL10A;RPL39
Lamotrigine-26_mg/kg_in_CMC-Rat-Heart-3d-up	12/287	0,001071539	0,016711766	0	0	2,873666439	19,65202561	RPS17;CXCL11;RPL32;RPS8;RPS5;RPL35A;RPSA;RPS3A;RAPGEF5;RPL10A;TOMM20;TPT1
N-Nitrosodiethylamine-34_mg/kg_in_Saline-Rat-Bone_marrow-3d-dn	12/287	0,001071539	0,016678739	0	0	2,873666439	19,65202561	EEF1B2;RPS17;RPL32;RPS8;RPS5;RPL13A;RPL35A;RPSA;RPS3A;RPL10A;RPL39;TPT1
Bromisovalum-250_mg/kg_in_Corn_Oil-Rat-Kidney-1d-dn	13/328	0,001095537	0,017018639	0	0	2,723996312	18,56814968	RPL32;RPS8;RPS6;RPSA;RPS3A;SEPP1;EEF1B2;RPS17;RPS19;ARL2BP;RPS20;RPL39;TPT1
Diclofenac-3.5_mg/kg_in_Corn_Oil-Rat-Liver-1d-dn	12/288	0,001103939	0,017115405	0	0	2,863688431	19,49848322	EEF1B2;C1QA;RPS17;RPL32;RPS8;RPS6;RPSA;RPS3A;RPL10A;RPL39;SEPP1;TPT1
Ouabain-144_mg/kg_in_CMC-Rat-Heart-5d-dn	12/288	0,001103939	0,017081779	0	0	2,863688431	19,49848322	RPS17;RPS19;ARL2BP;RPS8;FUNDC1;ARL6IP5;RPS20;RPSA;RPS3A;RPL39;SEPP1;TPT1
Ampiroxicam-94_mg/kg_in_CMC-Rat-Kidney-5d-up	13/329	0,001126076	0,017390152	0	0	2,715716688	18,43704385	C1QA;RPL32;RPS8;RPS6;RPSA;RPS3A;RPL10A;EEF1B2;RPS17;CXCL11;RPS19;RPS20;TPT1
Cytarabine-487_mg/kg_in_Saline-Rat-Spleen-1d-up	13/329	0,001126076	0,01735612	0	0	2,715716688	18,43704385	C1QA;RPL32;RPS5;RPL35A;RPSA;RPL10A;SEPP1;EEF1B2;RPS17;RPS19;ARL6IP5;RPS20;RPL39
Hydralazine-280_mg/kg_in_Water-Rat-Liver-1d-up	13/329	0,001126076	0,017322222	0	0	2,715716688	18,43704385	RPL32;RPS8;RPS5;RPS6;RPL35A;RPSA;RPS3A;RPS17;CXCL11;RPS19;RPS20;RPL39;TPT1
Acyclovir-330_mg/kg_in_Saline-Rat-Liver-1d-dn	12/289	0,001137149	0,017458456	0	0	2,853779474	19,34642957	EEF1B2;RPS17;RPL32;RPS19;RPS8;RPS6;RPS20;RPS3A;RPL10A;RPL39;SEPP1;TPT1
Doxorubicin-0.65_mg/kg_in_Saline-Rat-Spleen-0.25d-up	12/289	0,001137149	0,01742449	0	0	2,853779474	19,34642957	C1QA;RPS17;RPL32;RPS19;RPS8;RPS5;RPL13A;RPL35A;RPSA;RPS3A;RPL10A;TPT1
Ofloxacin-30_mg/kg_in_Water-Rat-Heart-3d-up	12/289	0,001137149	0,017390656	0	0	2,853779474	19,34642957	RPS17;RPL32;RPS19;ARL2BP;RPS8;RPS5;RPS6;ARL6IP5;RPS20;RPSA;RPS3A;RAPGEF5
Spironolactone-300_mg/kg_in_CMC-Rat-Liver-3d-dn	12/289	0,001137149	0,017356953	0	0	2,853779474	19,34642957	C1QA;RPS17;RPL32;RPS19;CLP1;RPS8;RPS6;RPL35A;RPSA;RPS3A;RPL39;SEPP1
Mercuric_Chloride-2.5_mg/kg_in_Water-Rat-Kidney-5d-dn	11/250	0,001140698	0,017377447	0	0	3,024054983	20,49134322	RPS17;RPL32;RPS19;RPS6;ARL6IP5;RPS20;RPSA;RPS3A;RPL39;SEPP1;TPT1
Ketoprofen-4_mg/kg_in_Corn_Oil-Rat-Kidney-3d-up	14/371	0,00114171	0,017359275	0	0	2,593529145	17,57175272	RPL32;RPS8;RPS5;RPS6;RPL35A;RPSA;RPS3A;RPL10A;RPS17;RPS19;ARL2BP;RPS20;RAPGEF5;TPT1
Sulindac-64_mg/kg_in_Corn_Oil-Rat-Kidney-3d-up	14/371	0,00114171	0,017325827	0	0	2,593529145	17,57175272	RPL32;RPS8;RPS5;RPS6;RPL13A;RPL35A;RPSA;RPS3A;RPL10A;TOMM20;EEF1B2;RPS17;RPS19;RPS20
Clindamycin-161_mg/kg_in_Saline-Rat-Kidney-3d-up	13/330	0,001157324	0,017529005	0	0	2,707487244	18,30706754	RPL32;RPS5;RPS6;RPL13A;RPL35A;RPSA;RPS3A;SEPP1;TOMM20;EEF1B2;RPS17;RPS20;TPT1
Methapyrilene-100_mg/kg_in_CMC-Rat-Liver-5d-up	13/330	0,001157324	0,01749536	0	0	2,707487244	18,30706754	RPL32;RPS8;RPS6;RPSA;RPS3A;RPL10A;SEPP1;EEF1B2;RPS17;RPS19;R3HCC1;RPS20;RPL39
Diethylstilbestrol-280_mg/kg_in_Corn_Oil-Rat-Liver-1d-up	12/290	0,001171185	0,017670979	0	0	2,843938855	19,19584625	EEF1B2;RPS17;RPL32;RPS19;RPS8;FEM1B;RPS5;RPS6;RPS20;RPSA;RPS3A;RPL10A
Galantamine-110_mg/kg_in_CMC-Rat-Heart-1d-dn	12/290	0,001171185	0,017637191	0	0	2,843938855	19,19584625	EEF1B2;RPS17;RPL32;RPS8;COL5A3;ARL6IP5;RPL35A;RPS20;RPSA;RPS3A;RPL39;TPT1
Indomethacin-5_mg/kg_in_Corn_Oil-Rat-Liver-3d-up	12/290	0,001171185	0,017603533	0	0	2,843938855	19,19584625	EEF1B2;C1QA;RPS17;RPS19;ARL2BP;RPS8;RPS6;RPS20;RPSA;RPL10A;RPL39;SEPP1
Norepinephrine-1.75_mg/kg_in_Saline-Rat-Heart-1d-up	12/290	0,001171185	0,017570002	0	0	2,843938855	19,19584625	C1QA;RPS17;CXCL11;RPS19;RPS8;RPS6;ARL6IP5;RPS20;RPSA;RPS3A;RPL39;TPT1
Nortriptyline-63_mg/kg_in_Water-Rat-Heart-1d-up	12/290	0,001171185	0,017536599	0	0	2,843938855	19,19584625	C1QA;RPS17;RPL32;ARL2BP;RPS8;RPS6;ARL6IP5;HOXA1;RPSA;RPS3A;RPL39;TPT1
Levamisole-120_mg/kg_in_Water-Rat-Liver-3d-up	13/331	0,001189292	0,017773937	0	0	2,699307524	18,17820845	RPL32;RPS8;RPS5;RPS6;RPL35A;RPSA;RPS3A;RPL10A;SEPP1;RPS17;RPS19;RPL39;TPT1
Metoclopramide-185_mg/kg_in_CMC-Rat-Intestine-5d-dn	13/331	0,001189292	0,017740275	0	0	2,699307524	18,17820845	RPS8;RPS6;RPL13A;RPL35A;RPSA;RPS3A;RPL10A;SEPP1;EEF1B2;RPS17;RPS19;ARL6IP5;RPL39
Kanamycin-225_mg/kg_in_Saline-Rat-Kidney-1d-up	14/373	0,001201319	0,017885799	0	0	2,579622822	17,34624867	RPL32;RPS8;RPS5;RPL13A;RPL35A;RPSA;RPS3A;SEPP1;TOMM20;RPS17;ARL6IP5;RPS20;RPL39;TPT1
6-Mercaptopurine-5_uM_in_DMSO-Rat-Primary_rat_hepatocytes-1d-dn	18/549	0,001205607	0,017915777	0	0	2,253394175	15,1445484	RPL32;RPS8;RPS5;RPS6;RPL35A;RPSA;RPS3A;RPL10A;SEPP1;EEF1B2;RPS17;RPS19;ARL2BP;FUNDC1;RPS20;RPL39;GOLGA7;TPT1
Labetalol-250_uM_in_DMSO-Rat-Primary_rat_hepatocytes-0.67d-up	12/291	0,001206061	0,017888774	0	0	2,83416587	19,0467151	EEF1B2;RPS17;RPL32;RPS19;ARL2BP;RPS5;RPS6;RPS20;RPS3A;RPL10A;RPL39;PDRG1
Allyl_Alcohol-25_mg/kg_in_Saline-Rat-Kidney-1d-up	13/332	0,001221994	0,018091027	0	0	2,691177079	18,05045441	RPL32;RPS8;RPS5;RPS6;RPL13A;RPL35A;RPSA;RPS3A;TOMM20;RPS17;RPS20;RPL39;TPT1
Cyclosporin_A-70_mg/kg_in_Corn_Oil-Rat-Kidney-5d-up	13/332	0,001221994	0,018057085	0	0	2,691177079	18,05045441	RPS8;RPS6;RPSA;RPS3A;RPL10A;SEPP1;EEF1B2;RPS17;CXCL11;RPS19;ARL6IP5;RPS20;TPT1
Bacitracin-380_mg/kg_in_Corn_Oil-Rat-Kidney-5d-up	12/292	0,001241795	0,018315313	0	0	2,824459822	18,89901827	C1QA;RPS17;RPL32;RPS19;RPS8;RPS5;RPS6;RPSA;RPS3A;RPL10A;RPL39;TPT1
Doxorubicin-0.65_mg/kg_in_Saline-Rat-Liver-0.25d-up	12/292	0,001241795	0,018281078	0	0	2,824459822	18,89901827	EEF1B2;RPS17;RPL32;RPS8;RPS5;RPL35A;RPSA;RPS3A;RPL10A;SEPP1;RPL39;TPT1
Etidronate-1005_mg/kg_in_Water-Rat-Heart-1d-up	12/292	0,001241795	0,018246972	0	0	2,824459822	18,89901827	EEF1B2;RPS17;RPL32;RPS19;ARL2BP;RPS8;RPS5;RPS6;ARL6IP5;RPL35A;RPS20;RPS3A
Furosemide-375_mg/kg_in_CMC-Rat-Kidney-3d-up	12/292	0,001241795	0,018212992	0	0	2,824459822	18,89901827	EEF1B2;C1QA;RPS17;RPL32;RPS19;RPS8;RPS5;ARL6IP5;RPL13A;RPL35A;RPS20;RPS3A

Nifedipine-1022_mg/kg_in_CMC-Rat-Heart-5d-up	12/292	0,001241795	0,018179139	0	0	2,824459822	18,89901827	C1QA;RPS17;RPL32;RPS19;RPS8;RPS6;RPS20;RPSA;RPS3A;RPL10A;SEPP1;RPL39
Pentobarbital-20_mg/kg_in_Water-Rat-Liver-1d-up	13/333	0,001255443	0,018344841	0	0	2,683095467	17,92379345	RPL32;RPS8;RPS5;RPS6;RPL35A;RPSA;RPS3A;RPL10A;SEPP1;EEF1B2;RPS17;RPS19;RPL39
Valproic_Acid-235_mg/kg_in_Water-Rat-Kidney-3d-up	14/375	0,001263518	0,018428638	0	0	2,565864834	17,12421174	RPL32;RPS8;RPS5;RPS6;RPL13A;RPL35A;RPSA;RPS3A;EEF1B2;RPS17;RPS19;ARL6IP5;RPS20;RPL39
Amoxapine-313_mg/kg_in_CMC-Rat-Heart-1d-dn	12/293	0,001278402	0,01861126	0	0	2,814820027	18,75273817	RPS17;RPL32;RPS19;ARL2BP;RPS8;COL5A3;RPS6;RPL35A;RPSA;RPS3A;GOLGA7;TPT1
Ethambutol-998_mg/kg_in_CMC-Rat-Kidney-5d-up	12/293	0,001278402	0,018576922	0	0	2,814820027	18,75273817	RPL32;RPS19;RPS8;RPS5;RPS6;RPSA;RPL10A;SEPP1;RPL39;GOLGA7;TOMM20;TPT1
Ifosfamide-143_mg/kg_in_Saline-Rat-Bone_marrow-1d-dn	12/293	0,001278402	0,018542711	0	0	2,814820027	18,75273817	EEF1B2;RPS17;RPL32;RPS19;RPS8;FEM1B;RPS6;RPS20;RPSA;RPS3A;RPL10A;RPL39
Methimazole-28_mg/kg_in_Water-Rat-Liver-1d-up	12/293	0,001278402	0,018508625	0	0	2,814820027	18,75273817	EEF1B2;RPS17;RPL32;RPS8;RPS5;RPS6;RPL13A;RPL35A;RPS20;RPSA;RPS3A;RPL39
Tacrolimus-134_mg/kg_in_CMC-Rat-Kidney-3d-up	12/293	0,001278402	0,018474664	0	0	2,814820027	18,75273817	EEF1B2;C1QA;RPL32;RPS19;ARL2BP;RPS8;RPS5;RPS6;RPL35A;RPS3A;TPT1;PDRG1
Zileuton-450_mg/kg_in_Corn_Oil-Rat-Kidney-3d-up	12/293	0,001278402	0,018440828	0	0	2,814820027	18,75273817	RPS17;RPL32;RPS19;ARL2BP;RPS8;RPS5;RPS6;RPL13A;RPL35A;RPS20;RPS3A;RPL39
Fluvastatin-94_mg/kg_in_Corn_Oil-Rat-Liver-3d-up	13/334	0,001289652	0,0185691	0	0	2,675062247	17,79821372	RPL32;RPS8;RPS5;RPS6;RPL35A;RPSA;RPS3A;RPL10A;EEF1B2;RPS17;RPS19;RPS20;TPT1
Valproic_Acid-235_mg/kg_in_Water-Rat-Bone_marrow-3d-up	13/334	0,001289652	0,018535215	0	0	2,675062247	17,79821372	C1QA;RPL32;RPS5;NKG7;RPL35A;RPSA;RPS3A;RPL10A;SEPP1;EEF1B2;RPS17;RPS20;RPL39
Sulfamethoxazole-1000_mg/kg_in_Water-Rat-Kidney-5d-up	14/376	0,001295615	0,018587002	0	0	2,559040725	17,01447252	C1QA;RPL32;RPS6;RPL13A;RPL35A;RPSA;RPS3A;RPL10A;EEF1B2;RPS17;CXCL11;RPS19;ARL6IP5;RPS20
Azathioprine-20_mg/kg_in_Corn_Oil-Rat-Spleen-3d-up	12/294	0,001315898	0,018843659	0	0	2,80524581	18,60785749	EEF1B2;C1QA;RPS17;RPL32;RPS8;RPS5;RPS6;RPL13A;RPL35A;RPS20;RPSA;RPS3A
Cadmium_Chloride-8_uM_in_DMSO-Rat-Primary_rat_hepatocytes-0.67d-dn	12/294	0,001315898	0,01880946	0	0	2,80524581	18,60785749	EEF1B2;RPS17;CXCL11;RPS19;ARL2BP;FEM1B;RPS20;RPS3A;RPL10A;SEPP1;RPL39;TPT1
Carboplatin-14_mg/kg_in_Saline-Rat-Spleen-1d-dn	12/294	0,001315898	0,018775384	0	0	2,80524581	18,60785749	EEF1B2;RPS17;RPL32;RPS19;RPS8;RPS5;FUNDC1;RPS6;RPL13A;RPL35A;RPSA;RPS3A
Ethinylestradiol-1480_mg/kg_in_Corn_Oil-Rat-Liver-1d-up	12/294	0,001315898	0,018741433	0	0	2,80524581	18,60785749	EEF1B2;RPS17;RPL32;RPS19;RPS8;FEM1B;RPS6;RPS20;RPSA;RPS3A;RPL10A;RPL39
Norethindrone-0.08_mg/kg_in_Corn_Oil-Rat-Liver-5d-up	12/294	0,001315898	0,018707603	0	0	2,80524581	18,60785749	EEF1B2;C1QA;RPS17;RPL32;RPS19;RPS8;RPS5;RPS6;RPL35A;RPS20;RPSA;RPL39
Phenylbutazone-368_mg/kg_in_CMC-Rat-Kidney-3d-up	12/294	0,001315898	0,018673896	0	0	2,80524581	18,60785749	C1QA;RPL32;RPS19;FEM1B;RPS5;RPS6;RPL35A;RPS3A;RPL10A;SEPP1;RPL39;TPT1
Candesartan-1300_mg/kg_in_CMC-Rat-Kidney-1d-up	13/335	0,001324634	0,018764058	0	0	2,667076986	17,67370358	RPL32;RPS8;RPS6;RPL35A;RPSA;RPL10A;EEF1B2;RPS17;RPS19;FUNDC1;ARL6IP5;RPS20;RPL39
Daunorubicin-3.25_mg/kg_in_Saline-Rat-Liver-5d-dn	13/335	0,001324634	0,01873037	0	0	2,667076986	17,67370358	C1QA;RPL32;RPS6;RPL35A;RPSA;RPS3A;RPL10A;EEF1B2;RPS17;RPS19;ARL6IP5;RPS20;RPL39
Lead_(II)_Acetate-2_mg/kg_in_Saline-Rat-Kidney-14d-up	13/335	0,001324634	0,018696803	0	0	2,667076986	17,67370358	RPL32;RPS8;RPS5;RPS6;RPS3A;RPL10A;EEF1B2;RPS17;CXCL11;RPS19;ARL6IP5;RPS20;RPL39
Fluconazole-10_mg/kg_in_Corn_Oil-Rat-Liver-5d-up	12/295	0,0013543	0,019081338	0	0	2,795736502	18,46435917	EEF1B2;RPS17;RPL32;RPS8;RPS5;RPS6;RPL35A;RPS20;RPSA;RPS3A;SEPP1;RPL39
Ramipril-1500_mg/kg_in_CMC-Rat-Kidney-1d-up	12/295	0,0013543	0,019047264	0	0	2,795736502	18,46435917	EEF1B2;C1QA;RPS17;RPL32;RPS8;RPS5;RPS6;ARL6IP5;RPL13A;RPL35A;RPS20;RPL10A
Torsemide-110_mg/kg_in_Corn_Oil-Rat-Liver-3d-dn	12/295	0,0013543	0,019013312	0	0	2,795736502	18,46435917	EEF1B2;RPS17;RPL32;RPS19;RPS8;RPS5;RPL35A;RPS20;RPSA;RPS3A;RPL10A;TPT1
1-Naphthyl_Isothiocyanate-30_mg/kg_in_Corn_Oil-Rat-Liver-3d-up	13/336	0,001360403	0,019065002	0	0	2,659139257	17,55025151	C1QA;RPL32;RPS8;RPS5;RPS6;RPL35A;RPSA;RPL10A;EEF1B2;RPS19;RPS20;RPL39;TPT1
Bisphenol_A-610_mg/kg_in_Corn_Oil-Rat-Kidney-5d-up	13/336	0,001360403	0,019031139	0	0	2,659139257	17,55025151	RPL32;RPS8;RPS5;RPL35A;RPSA;RPS3A;SEPP1;TOMM20;EEF1B2;RPS17;ARL2BP;RPS20;TPT1
Acetaminophen-1950_uM_in_DMSO-Rat-Primary_rat_hepatocytes-1d-up	11/256	0,00137934	0,019261847	0	0	2,953178694	19,45007828	C1QA;CXCL11;RPL32;ARL2BP;CLP1;RPS8;FEM1B;RPS5;RPS6;RPS20;RPS3A
Ethylene_Glycol-352_mg/kg_in_Water-Rat-Liver-3d-dn	11/256	0,00137934	0,019227756	0	0	2,953178694	19,45007828	RPS17;RPL32;RPS19;RPS8;RPS6;ARL6IP5;RPL35A;RPS20;RPS3A;SEPP1;TPT1
Carbon_Tetrachloride-400_mg/kg_in_Corn_Oil-Rat-Bone_marrow-0.25d-up	12/296	0,001393625	0,019392562	0	0	2,786291446	18,32222643	C1QA;RPL32;RPS8;FEM1B;RPS5;FUNDC1;RPS6;RPL35A;RPS20;RPS3A;RPL39;TPT1
Carmustine-16_mg/kg_in_Corn_Oil-Rat-Bone_marrow-3d-up	12/296	0,001393625	0,01935836	0	0	2,786291446	18,32222643	EEF1B2;C1QA;RPS17;RPL32;RPS8;RPS5;RPL13A;RPL35A;RPS20;SEPP1;TPT1;DUSP14
N-Nitrosodiethylamine-34_mg/kg_in_Saline-Rat-Kidney-5d-up	12/296	0,001393625	0,019324278	0	0	2,786291446	18,32222643	RPS17;RPL32;RPS19;RPS5;RPS6;RPL13A;RPL35A;RPS20;RPSA;RPS3A;RPL39;TPT1
Sildenafil-14.6_mg/kg_in_Saline-Rat-Liver-1d-dn	12/296	0,001393625	0,019290316	0	0	2,786291446	18,32222643	EEF1B2;RPS17;RPL32;RPS8;RPS5;RPS6;RPL35A;RPSA;RPS3A;RPL10A;SEPP1;TPT1
Valproic_Acid-850_mg/kg_in_Water-Rat-Liver-5d-up	12/296	0,001393625	0,019256474	0	0	2,786291446	18,32222643	EEF1B2;RPS17;RPL32;RPS19;RPS8;RPS5;RPS6;RPL35A;RPS20;RPS3A;RPL10A;RPL39
Zomepirac-11_mg/kg_in_Saline-Rat-Liver-5d-up	12/296	0,001393625	0,019222749	0	0	2,786291446	18,32222643	EEF1B2;RPS17;RPL32;RPS19;RPS8;RPS6;RPL35A;RPS20;RPSA;RPS3A;RPL39;TPT1
Rofecoxib-775_mg/kg_in_Corn_Oil-Rat-Heart-3d-up	14/379	0,001396034	0,019222312	0	0	2,538784466	16,69027377	C1QA;RPS6;RPSA;RPS3A;RPL10A;PDRG1;EEF1B2;RPS19;ARL2BP;ARL6IP5;RPS20;RAPGEF5;RPL39;TPT1
Sodium_Nitroprusside-6_mg/kg_in_Saline-Rat-Heart-3d-dn	14/380	0,00143092	0,019668282	0	0	2,532103455	16,58385384	RPL32;FEM1B;RPS6;RPSA;RPS3A;RPL10A;SEPP1;EEF1B2;RPS17;RPS19;COL5A3;RPS20;RPL39;TPT1
4-Chloroaniline-75_mg/kg_in_CMC-Rat-Kidney-5d-up	12/297	0,001433889	0,019674759	0	0	2,776909993	18,18144271	EEF1B2;RPS17;CXCL11;CLP1;RPS8;ARL6IP5;RPS20;RPSA;RPS3A;RAPGEF5;TOMM20;TPT1
Atorvastatin-300_mg/kg_in_Corn_Oil-Rat-Liver-3d-up	12/297	0,001433889	0,019640542	0	0	2,776909993	18,18144271	C1QA;RPS17;CXADR;RPS19;ARL2BP;RPS8;RPS6;RPS20;RPSA;RPS3A;RPL10A;RPL39
Busulfan-3_mg/kg_in_Corn_Oil-Rat-Bone_marrow-3d-up	12/297	0,001433889	0,019606444	0	0	2,776909993	18,18144271	EEF1B2;C1QA;RPS17;RPL32;RPS8;RPS5;RPL13A;RPL35A;RPS20;RPS3A;TPT1;DUSP14
Lead_(II)_Acetate-200_mg/kg_in_Saline-Rat-Kidney-0.25d-up	12/297	0,001433889	0,019572464	0	0	2,776909993	18,18144271	RPS17;RPL32;RPS8;RPS5;ARL6IP5;RPL13A;RPL35A;RPS20;RPSA;RPS3A;TOMM20;TPT1
Lomefloxacin-2000_mg/kg_in_CMC-Rat-Liver-5d-up	12/297	0,001433889	0,019538601	0	0	2,776909993	18,18144271	RPS17;RPL32;RPS19;ARL2BP;RPS8;RPS6;RPS20;RPSA;RPL10A;SEPP1;RPL39;TPT1
Quetiapine-500_mg/kg_in_CMC-Rat-Liver-3d-dn	12/297	0,001433889	0,019504856	0	0	2,776909993	18,18144271	EEF1B2;C1QA;RPS17;RPL32;RPS8;RPS5;RPL35A;RPS20;RPSA;RPS3A;SEPP1;TPT1
Vinorelbine-1.5_mg/kg_in_Saline-Rat-Liver-1d-dn	12/297	0,001433889	0,019471227	0	0	2,776909993	18,18144271	EEF1B2;RPS17;RPL32;RPS19;RPS8;RPS6;RPL35A;RPS20;RPSA;RPS3A;RPL10A;RPL39
Pyridine-223_mg/kg_in_Water-Rat-Kidney-3d-dn	10/219	0,00143901	0,019507137	0	0	3,138288691	20,53633221	RPS17;RPL32;RPS19;ARL2BP;RPS8;RPS6;RPL35A;RPSA;RPS3A;RPL39
Phenelzine-27_mg/kg_in_Water-Rat-Heart-3d-dn	11/258	0,001467447	0,019858447	0	0	2,930285836	19,11786115	EEF1B2;RPS17;RPL32;RPS19;COL5A3;RPS6;RPSA;RPS3A;RPL10A;RPL39;SEPP1
Melphalan-12_mg/kg_in_CMC-Rat-Bone_marrow-1d-dn	12/298	0,00147511	0,019927902	0	0	2,767591503	18,04199174	C1QA;RPS17;RPL32;RPS19;ARL2BP;RPS8;RPS5;RPL13A;RPL35A;RPSA;RPL10A;SEPP1
Methyl_Methanesulfonate-113_mg/kg_in_Water-Rat-Bone_marrow-1d-dn	12/298	0,00147511	0,019893779	0	0	2,767591503	18,04199174	EEF1B2;RPS17;RPL32;RPS8;RPS5;RPS6;ARL6IP5;RPL35A;RPS20;RPS3A;RPL39;TPT1
Penicillamine-100_mg/kg_in_Water-Rat-Bone_marrow-1d-dn	12/298	0,00147511	0,019859773	0	0	2,767591503	18,04199174	EEF1B2;RPS17;RPL32;RPS19;RPS8;RPS5;RPS6;ARL6IP5;RPL35A;RPS20;RPSA;DUSP14
Sodium_Arsenite-10_mg/kg_in_Saline-Rat-Kidney-3d-up	12/298	0,00147511	0,019825882	0	0	2,767591503	18,04199174	RPS17;RPL32;ARL2BP;RPS5;ARL6IP5;RPL13A;RPL35A;RPS20;RPS3A;SEPP1;RPL39;TOMM20
Prednisolone-184_mg/kg_in_Water-Rat-Heart-3d-dn	13/340	0,00151162	0,020281973	0	0	2,627855266	17,06679905	RPL32;RPS8;RPS5;RPS6;RPL35A;RPSA;RPS3A;EEF1B2;RPS17;ARL2BP;RPS20;RPL39;TPT1
Carboplatin-14_mg/kg_in_Saline-Rat-Liver-5d-dn	12/299	0,001517305	0,020323626	0	0	2,758335345	17,90385748	EEF1B2;RPS17;RPL32;RPS19;CLP1;RPS6;RPS20;RPSA;RPS3A;RPL10A;RPL39;TPT1
Loxoprofen-18_mg/kg_in_Corn_Oil-Rat-Kidney-3d-up	12/299	0,001517305	0,02028912	0	0	2,758335345	17,90385748	RPS17;RPL32;RPS19;ARL2BP;RPS5;RPS6;RPL35A;RPSA;RPS3A;RPL10A;RPL39;TOMM20
Sparfloxacin-29_mg/kg_in_Corn_Oil-Rat-Liver-5d-up	12/299	0,001517305	0,020254732	0	0	2,758335345	17,90385748	EEF1B2;C1QA;RPS17;RPL32;RPS19;CLP1;RPS8;RPS6;RPS20;RPS3A;RPL39;TPT1
Tocainide-224_mg/kg_in_Corn_Oil-Rat-Liver-3d-up	12/299	0,001517305	0,02022046	0	0	2,758335345	17,90385748	EEF1B2;RPS17;RPL32;RPS19;CLP1;RPS8;RPS5;RPS6;RPL35A;RPS20;RPS3A;RPL39
Valproic_Acid-3168_uM_in_DMSO-Rat-Primary_rat_hepatocytes-0.67d-up	12/299	0,001517305	0,020186304	0	0	2,758335345	17,90385748	EEF1B2;RPS17;RPL32;RPS19;RPS8;RPS5;RPS6;RPL35A;RPS20;RPS3A;TPT1;PDRG1
Doxazosin-5_mg/kg_in_Water-Rat-Heart-1d-dn	13/341	0,00155153	0,020606826	0	0	2,620148945	16,94846998	RPL32;RPS8;FEM1B;RPS6;RPS3A;RPL10A;EEF1B2;RPS17;RPS19;ARL2BP;RPS20;RPL39;TPT1
Hydrazine-45_mg/kg_in_Water-Rat-Liver-0.25d-up	13/341	0,00155153	0,020572134	0	0	2,620148945	16,94846998	C1QA;RPL32;RPS8;RPS5;RPS6;RPL13A;RPSA;RPS3A;RPL10A;SEPP1;RPS19;RPS20;TPT1
Catechol-195_mg/kg_in_Saline-Rat-Kidney-3d-up	12/300	0,001560491	0,020656175	0	0	2,749140893	17,76702411	RPS17;RPL32;RPS8;RPS5;ARL6IP5;RPL13A;RPL35A;RPS20;RPSA;RPS3A;RPL39;TOMM20
Clonidine-6_mg/kg_in_Water-Rat-Heart-3d-up	12/300	0,001560491	0,020621517	0	0	2,749140893	17,76702411	C1QA;RPS17;RPL32;RPS8;RPS5;RPS6;RPS20;RPS3A;RAPGEF5;RPL10A;SEPP1;TPT1
Cyclosporin_A-350_mg/kg_in_Corn_Oil-Rat-Spleen-5d-up	12/300	0,001560491	0,020586975	0	0	2,749140893	17,76702411	EEF1B2;C1QA;RPS17;RPL32;RPS19;RPS8;RPS5;RPS20;RPSA;RPS3A;RPL10A;TPT1
Diethylstilbestrol-280_mg/kg_in_Corn_Oil-Rat-Bone_marrow-1d-dn	12/300	0,001560491	0,020552548	0	0	2,749140893	17,76702411	EEF1B2;RPS17;RPL32;RPS8;FEM1B;RPS5;RPS6;ARL6IP5;RPL13A;RPL35A;RPSA;RPS3A
Ferulic_Acid-1500_mg/kg_in_CMC-Rat-Liver-1d-up	12/300	0,001560491	0,020518237	0	0	2,749140893	17,76702411	EEF1B2;RPS17;RPL32;RPS19;RPS8;RPS5;RPS6;RPSA;RPS3A;RPL10A;RPL39;TPT1
Fluvastatin-94_mg/kg_in_Corn_Oil-Rat-Skeletal_muscle-5d-up	12/300	0,001560491	0,02048404	0	0	2,749140893	17,76702411	EEF1B2;RPS17;RPL32;RPS19;RPS8;RPS6;RPS20;RPSA;RPS3A;RPL10A;RPL39;TPT1
Lead_(II)_Acetate-200_mg/kg_in_Saline-Rat-Spleen-3d-dn	12/300	0,001560491	0,020449957	0	0	2,749140893	17,76702411	EEF1B2;C1QA;RPS17;ARL2BP;RPS8;FUNDC1;RPS6;ARL6IP5;RPL35A;RPS3A;RPL39;TPT1
Methotrexate-27_mg/kg_in_Corn_Oil-Rat-Bone_marrow-1d-dn	12/300	0,001560491	0,020415987	0	0	2,749140893	17,76702411	EEF1B2;RPS17;RPL32;RPS8;FEM1B;RPS5;RPS6;ARL6IP5;RPL35A;RPS20;RPSA;RPS3A
NN-Dimethylformamide-140_mg/kg_in_Saline-Rat-Spleen-1d-dn	12/300	0,001560491	0,020382129	0	0	2,749140893	17,76702411	EEF1B2;RPS17;RPL32;RPS8;RPS5;ARL6IP5;RPL13A;RPL35A;RPS20;RPSA;RPS3A;TPT1
Norethindrone-375_mg/kg_in_Corn_Oil-Rat-Liver-5d-up	12/300	0,001560491	0,020348384	0	0	2,749140893	17,76702411	EEF1B2;RPS17;RPL32;RPS19;RPS8;RPS5;RPS6;RPL35A;RPS20;RPSA;RPS3A;RPL39
Sulfathiazole-2629_mg/kg_in_Water-Rat-Liver-3d-up	12/300	0,001560491	0,02031475	0	0	2,749140893	17,76702411	EEF1B2;RPS17;RPL32;RPS8;RPS5;RPS6;RPL35A;RPS20;RPSA;RPS3A;SEPP1;TOMM20
Trimethadione-535_mg/kg_in_Water-Rat-Heart-3d-dn	12/300	0,001560491	0,020281228	0	0	2,749140893	17,76702411	RPS17;RPL32;RPS8;COL5A3;RPS5;RPS6;RPL35A;RPS20;RPSA;RPS3A;RPL39;TPT1
Doxorubicin-3_mg/kg_in_Saline-Rat-Heart-1d-dn	13/342	0,00159231	0,020660684	0	0	2,612487691	16,83113308	RPL32;RPS8;RPS5;RPL35A;RPSA;RPS3A;TOMM20;EEF1B2;RPS17;ARL6IP5;RPS20;RPL39;TPT1
11-Dichloroethene-600_mg/kg_in_Water-Rat-Liver-5d-up	12/301	0,001604685	0,020787009	0	0	2,740007535	17,63147608	EEF1B2;C1QA;RPS17;RPL32;RPS19;RPS5;RPS6;ARL6IP5;RPL35A;RPS20;RPSA;RPS3A
Altretamine-40_mg/kg_in_Corn_Oil-Rat-Bone_marrow-5d-up	12/301	0,001604685	0,020752876	0	0	2,740007535	17,63147608	EEF1B2;C1QA;RPS17;RPL32;FEM1B;RPS5;RPL35A;RPS20;RPS3A;SEPP1;RPL39;TPT1
Atorvastatin-300_mg/kg_in_Corn_Oil-Rat-Skeletal_muscle-3d-up	12/301	0,001604685	0,020718855	0	0	2,740007535	17,63147608	EEF1B2;RPS17;RPL32;RPS19;RPS5;FUNDC1;RPL35A;RPS20;RPSA;RPS3A;RPL39;TPT1
Captopril-1750_mg/kg_in_Water-Rat-Kidney-5d-up	12/301	0,001604685	0,020684946	0	0	2,740007535	17,63147608	EEF1B2;RPS17;CXCL11;RPL32;RPS19;RPS6;RPL35A;RPS20;RPS3A;RAPGEF5;RPL10A;RPL39
Diethylstilbestrol-2.8_mg/kg_in_Corn_Oil-Rat-Liver-3d-up	13/343	0,001633976	0,021028097	0	0	2,604871109	16,71477787	C1QA;RPL32;RPS8;RPS5;RPS6;RPL35A;RPSA;RPL10A;EEF1B2;RPS19;RPS20;RPL39;TPT1
Mercuric_Chloride-2.5_mg/kg_in_Water-Rat-Kidney-3d-dn	10/223	0,00164588	0,021146741	0	0	3,081996517	19,75399502	RPS17;RPL32;RPS19;RPS6;ARL6IP5;RPL35A;RPS20;RPSA;RPS3A;TPT1
Methotrexate-0.3_mg/kg_in_Corn_Oil-Rat-Spleen-0.25d-up	12/302	0,001649907	0,021163951	0	0	2,730934662	17,49719805	EEF1B2;C1QA;RPS17;RPS8;RPS5;RPL13A;RPL35A;RPS20;RPS3A;RAPGEF5;SEPP1;TPT1
Mesna-755_mg/kg_in_Saline-Rat-Kidney-1d-up	13/344	0,001676542	0,021470639	0	0	2,597298809	16,59939399	C1QA;RPL32;RPS6;RPL13A;RPL35A;RPSA;RPS3A;SEPP1;RPS17;RPS19;ARL6IP5;RPS20;RPL39
Methotrexate-0.3_mg/kg_in_Corn_Oil-Rat-Liver-3d-up	13/344	0,001676542	0,021435784	0	0	2,597298809	16,59939399	C1QA;RPL32;RPS8;RPS5;RPS6;RPL35A;RPSA;SEPP1;EEF1B2;RPS19;RPS20;RPL39;TPT1
DActinomycin-0.06_mg/kg_in_Saline-Rat-Bone_marrow-0.25d-up	12/303	0,001696173	0,021651633	0	0	2,721921677	17,36417492	RPS17;RPL32;RPS19;FEM1B;RPS5;RPS6;ARL6IP5;RPSA;RPS3A;RPL10A;RPL39;TPT1
Lomustine-560_uM_in_DMSO-Rat-Primary_rat_hepatocytes-1d-up	12/303	0,001696173	0,021616598	0	0	2,721921677	17,36417492	EEF1B2;RPS17;RPL32;CLP1;RPS5;RPS6;ARL6IP5;RPL35A;RPSA;RPL10A;GOLGA7;PDRG1
Sertraline-210_mg/kg_in_Corn_Oil-Rat-Liver-3d-dn	12/303	0,001696173	0,021581676	0	0	2,721921677	17,36417492	EEF1B2;RPS17;RPL32;RPS19;CLP1;RPS8;RPS6;RPL35A;RPS3A;RPL10A;RPL39;TPT1
Sodium_Arsenite-10_mg/kg_in_Saline-Rat-Kidney-1d-up	12/303	0,001696173	0,021546867	0	0	2,721921677	17,36417492	RPS17;RPL32;RPS8;RPS6;ARL6IP5;RPL13A;RPL35A;RPS20;RPSA;RPS3A;SEPP1;RPL39
Caffeine-93_mg/kg_in_Water-Rat-Heart-3d-dn	13/345	0,001720022	0,021814647	0	0	2,589770407	16,48497125	RPL32;RPS8;RPS6;RPL35A;RPSA;RPS3A;RPL10A;PDRG1;EEF1B2;RPS17;RPS19;ARL6IP5;RPL39
Azathioprine-0.5_uM_in_DMSO-Rat-Primary_rat_hepatocytes-0.67d-up	12/304	0,001743502	0,022076882	0	0	2,712967987	17,23239181	EEF1B2;RPS17;RPL32;RPS19;RPS5;ARL6IP5;RPL35A;RPS3A;SEPP1;RPL39;TPT1;PDRG1
Beta-Estradiol-150_mg/kg_in_Corn_Oil-Rat-Liver-3d-up	12/304	0,001743502	0,022041446	0	0	2,712967987	17,23239181	EEF1B2;RPS17;RPL32;RPS19;FEM1B;RPS6;RPS20;RPSA;RPS3A;RPL10A;SEPP1;RPL39
Bleomycin_A2-12_mg/kg_in_Saline-Rat-Bone_marrow-3d-up	12/304	0,001743502	0,022006123	0	0	2,712967987	17,23239181	EEF1B2;RPL32;RPS8;RPS5;FUNDC1;RPL13A;RPL35A;RPS20;RPSA;RPL10A;TOMM20;TPT1
Doxorubicin-1.5_uM_in_DMSO-Rat-Primary_rat_hepatocytes-0.67d-up	12/304	0,001743502	0,021970913	0	0	2,712967987	17,23239181	EEF1B2;RPS17;CXCL11;RPL32;RPS19;RPS8;RPS6;RPS20;RPS3A;SEPP1;RPL39;GOLGA7
Ifosfamide-143_mg/kg_in_Saline-Rat-Liver-3d-dn	12/304	0,001743502	0,021935816	0	0	2,712967987	17,23239181	EEF1B2;RPS17;RPL32;RPS8;RPS6;ARL6IP5;RPL35A;RPS20;RPS3A;RPL10A;RPL39;TPT1
Lomustine-4.2_mg/kg_in_Corn_Oil-Rat-Spleen-0.25d-up	12/304	0,001743502	0,02190083	0	0	2,712967987	17,23239181	EEF1B2;CXCL11;RPS19;ARL2BP;CLP1;RPS8;FUNDC1;RPS6;RPS20;RPSA;RPL10A;RPL39

NN-Dimethylformamide-140_mg/kg_in_Saline-Rat-Bone_marrow-3d-dn	12/304	0,001743502	0,021865957	0	0	2,712967987	17,23239181	EEF1B2;RPL32;RPS19;ARL2BP;RPS8;RPS5;FUNDC1;RPL13A;RPL35A;RPS20;RPSA;TPT1
Pyrogallol-1000_mg/kg_in_Water-Rat-Liver-5d-up	12/304	0,001743502	0,021831194	0	0	2,712967987	17,23239181	EEF1B2;RPS17;RPL32;RPS19;RPS8;RPS5;RPS6;RPL35A;RPS20;RPS3A;RPL10A;SEPP1
Rifabutin-1500_mg/kg_in_CMC-Rat-Liver-1d-dn	12/304	0,001743502	0,021796541	0	0	2,712967987	17,23239181	EEF1B2;C1QA;RPS17;RPL32;RPS19;RPS6;RPS20;RPSA;RPS3A;RPL10A;RPL39;TPT1
Spironolactone-300_mg/kg_in_CMC-Rat-Heart-5d-up	12/304	0,001743502	0,021761998	0	0	2,712967987	17,23239181	EEF1B2;RPS17;RPL32;RPS19;RPS8;RPS5;RPS6;RPL35A;RPS20;RPS3A;RPL10A;RPL39
Daunorubicin-3.25_mg/kg_in_Saline-Rat-Bone_marrow-3d-dn	11/264	0,001759854	0,021931339	0	0	2,863688431	18,16301463	EEF1B2;RPS17;RPL32;RPS8;FEM1B;RPS6;NKG7;RPS20;RPSA;RPS3A;RPL39
Bezafibrate-617_mg/kg_in_Corn_Oil-Rat-Kidney-1d-dn	12/305	0,001791912	0,022295576	0	0	2,70407301	17,10183405	EEF1B2;RPS17;RPL32;ARL2BP;RPS8;FUNDC1;RPS20;RPSA;SEPP1;RPL39;TOMM20;TPT1
Crotamiton-750_mg/kg_in_Corn_Oil-Rat-Liver-5d-up	12/305	0,001791912	0,02226041	0	0	2,70407301	17,10183405	RPS17;RPL32;RPS19;ARL2BP;RPS8;RPS5;RPS6;RPS20;RPSA;RPS3A;RPL10A;TPT1
Fenofibrate-430_mg/kg_in_Corn_Oil-Rat-Skeletal_muscle-3d-dn	12/305	0,001791912	0,022225354	0	0	2,70407301	17,10183405	EEF1B2;RPS17;RPL32;RPS8;FEM1B;COL5A3;ARL6IP5;RPL35A;RPSA;RPL10A;RPL39;TPT1
Hydroxyurea-400_mg/kg_in_Saline-Rat-Spleen-3d-dn	12/305	0,001791912	0,022190409	0	0	2,70407301	17,10183405	EEF1B2;C1QA;RPS17;RPL32;RPS8;RPS5;RPS6;ARL6IP5;RPL35A;RPSA;RPS3A;TPT1
Interleukin_1_Beta-0.01_mg/kg_in_RSA/Saline-Rat-Liver-1d-up	12/305	0,001791912	0,022155573	0	0	2,70407301	17,10183405	EEF1B2;RPS17;RPL32;RPS19;RPS8;RPS6;ARL6IP5;RPL35A;RPS20;RPSA;RPS3A;RPL39
Mitomycin_C-0.5_mg/kg_in_Saline-Rat-Liver-5d-up	12/305	0,001791912	0,022120846	0	0	2,70407301	17,10183405	EEF1B2;RPS17;RPL32;RPS19;RPS8;RPS6;RPS20;RPS3A;SEPP1;RPL39;GOLGA7;TPT1
Pantoprazole-1100_mg/kg_in_Water-Rat-Liver-1d-up	12/305	0,001791912	0,022086228	0	0	2,70407301	17,10183405	EEF1B2;RPS17;RPL32;RPS19;RPS8;RPS5;RPS6;RPL35A;RPS20;RPSA;RPS3A;RPL39
Vinorelbine-1.5_mg/kg_in_Saline-Rat-Heart-3d-dn	12/305	0,001791912	0,022051719	0	0	2,70407301	17,10183405	RPS17;RPL32;RPS19;RPS8;FUNDC1;RPS6;ARL6IP5;RPS20;RPSA;RPS3A;RPL39;TPT1
Camptothecin-5_mg/kg_in_Saline-Rat-Kidney-1d-dn	13/347	0,001809788	0,022236953	0	0	2,574843776	16,258969	RPL32;RPS6;RPL35A;RPSA;RPS3A;RPL10A;EEF1B2;RPS17;CLP1;FUNDC1;RPS20;RPL39;TPT1
Cyclosporin_A-70_mg/kg_in_Corn_Oil-Rat-Spleen-0.25d-up	13/347	0,001809788	0,022202316	0	0	2,574843776	16,258969	C1QA;RPL32;RPS8;RPS6;RPL13A;RPSA;RPL10A;SEPP1;EEF1B2;RPS17;RPS20;RPL39;TPT1
Pravastatin-1200_mg/kg_in_Corn_Oil-Rat-Skeletal_muscle-5d-dn	13/347	0,001809788	0,022167786	0	0	2,574843776	16,258969	RPL32;RPS8;FEM1B;RPSA;RPS3A;RPL10A;EEF1B2;RPS19;COL5A3;ARL6IP5;RPL39;SLC16A3;TPT1
Alendronic_Acid-138_mg/kg_in_CMC-Rat-Heart-5d-up	11/265	0,001812918	0,022171653	0	0	2,852882059	18,00972348	EEF1B2;RPS17;RPL32;RPS19;ARL2BP;RPS8;RPL35A;RPS20;RPSA;RPS3A;TPT1
Azathioprine-54_mg/kg_in_Water-Rat-Bone_marrow-1d-dn	12/306	0,001841422	0,022485335	0	0	2,69523617	16,9724872	EEF1B2;RPS17;RPL32;RPS8;RPS5;RPS6;ARL6IP5;RPL35A;RPS20;RPSA;RPS3A;DUSP14
Cortisone-206_mg/kg_in_CMC-Rat-Liver-3d-dn	12/306	0,001841422	0,022450528	0	0	2,69523617	16,9724872	EEF1B2;RPL32;RPS19;RPS8;RPS5;RPS6;ARL6IP5;RPS20;RPS3A;RPL10A;SEPP1;TPT1
Coumarin-111_mg/kg_in_Corn_Oil-Rat-Bone_marrow-3d-up	12/306	0,001841422	0,022415829	0	0	2,69523617	16,9724872	C1QA;RPS19;CLP1;RPS8;RPS5;RPS6;NKG7;RPL35A;RPS20;RPS3A;RPL10A;RPL39
Cyclophosphamide-25_mg/kg_in_Water-Rat-Bone_marrow-3d-up	12/306	0,001841422	0,022381237	0	0	2,69523617	16,9724872	EEF1B2;C1QA;RPS17;RPL32;RPS19;RPS5;RPS6;NKG7;RPL35A;RPS20;RPL10A;SEPP1
Dexamethasone-1_mg/kg_in_Corn_Oil-Rat-Liver-1d-up	12/306	0,001841422	0,022346751	0	0	2,69523617	16,9724872	EEF1B2;RPL32;RPS19;RPS8;FEM1B;RPS6;RPS20;RPSA;RPS3A;RPL10A;RPL39;TPT1
Doxofylline-483_mg/kg_in_Water-Rat-Heart-1d-up	12/306	0,001841422	0,022312371	0	0	2,69523617	16,9724872	RPS17;RPL32;RPS19;ARL2BP;RPS6;ARL6IP5;RPS20;RPSA;RAPGEF5;RPL10A;SEPP1;TPT1
Metoclopramide-185_mg/kg_in_CMC-Rat-Intestine-1d-dn	12/306	0,001841422	0,022278097	0	0	2,69523617	16,9724872	EEF1B2;C1QA;RPS17;RPL32;RPS19;RPL35A;RPS20;RPSA;RPS3A;RPL10A;SEPP1;RPL39
NN'-Diphenyl-P-Phenylenediamine-1000_mg/kg_in_CMC-Rat-Kidney-3d-dn	12/306	0,001841422	0,022243928	0	0	2,69523617	16,9724872	RPS19;ARL2BP;RPS8;FEM1B;RPS6;ARL6IP5;RPS20;RPSA;RPL10A;RPL39;TPT1;PDRG1
Penicillamine-852_mg/kg_in_Water-Rat-Bone_marrow-1d-dn	12/306	0,001841422	0,022209864	0	0	2,69523617	16,9724872	EEF1B2;RPS17;RPL32;RPS8;RPS5;RPS6;ARL6IP5;RPL35A;RPS20;RPSA;RPS3A;DUSP14
Phenelzine-27_mg/kg_in_Water-Rat-Heart-5d-up	12/306	0,001841422	0,022175904	0	0	2,69523617	16,9724872	EEF1B2;RPS17;RPL32;ARL2BP;CLP1;RPS8;RPS20;RPS3A;RAPGEF5;RPL39;GOLGA7;TPT1
Pyrilamine-688_mg/kg_in_Water-Rat-Heart-3d-dn	12/306	0,001841422	0,022142048	0	0	2,69523617	16,9724872	EEF1B2;RPS17;RPL32;RPS19;RPS8;RPS6;RPS20;RPSA;RPS3A;RPL10A;RPL39;TPT1
1-Naphthyl_Isothiocyanate-30_mg/kg_in_Corn_Oil-Rat-Bone_marrow-3d-up	12/307	0,001892051	0,022716148	0	0	2,686456899	16,84433703	EEF1B2;C1QA;RPS17;RPL32;RPS8;RPS5;RPS6;RPL35A;RPS20;RPL39;SLC16A3;TPT1
4-Methylpyrazole-155_mg/kg_in_Saline-Rat-Liver-3d-up	12/307	0,001892051	0,022681572	0	0	2,686456899	16,84433703	C1QA;RPS17;RPL32;RPS19;RPS5;RPS6;ARL6IP5;RPL35A;RPS20;RPSA;RPL10A;RPL39
Bacitracin-380_mg/kg_in_Corn_Oil-Rat-Kidney-1d-up	12/307	0,001892051	0,022647102	0	0	2,686456899	16,84433703	EEF1B2;RPS17;CXCL11;RPL32;RPS8;RPS5;RPS6;RPSA;RPS3A;RAPGEF5;SEPP1;RPL39
Candesartan-1300_mg/kg_in_CMC-Rat-Heart-1d-dn	12/307	0,001892051	0,022612736	0	0	2,686456899	16,84433703	RPS17;RPL32;RPS19;RPS8;COL5A3;RPS5;RPS6;ARL6IP5;RPSA;RPS3A;SEPP1;TPT1
Cyclandelate-1500_mg/kg_in_CMC-Rat-Liver-1d-up	12/307	0,001892051	0,022578474	0	0	2,686456899	16,84433703	EEF1B2;RPS17;RPL32;RPS6;RPL35A;RPS20;RPSA;RPS3A;RPL10A;SEPP1;RPL39;TPT1
Hydrocortisone-56_mg/kg_in_Corn_Oil-Rat-Bone_marrow-3d-dn	12/307	0,001892051	0,022544316	0	0	2,686456899	16,84433703	RPS17;RPS19;FEM1B;RPS6;NKG7;RPS20;RPSA;RPS3A;RPL10A;SEPP1;RPL39;TPT1
Hydroxyurea-400_mg/kg_in_Saline-Rat-Bone_marrow-5d-up	12/307	0,001892051	0,022510261	0	0	2,686456899	16,84433703	EEF1B2;C1QA;RPL32;RPS19;ARL2BP;RPS8;RPS6;RPS20;RPS3A;SEPP1;RPL39;TPT1
Hydroxyurea-59_mg/kg_in_Saline-Rat-Spleen-3d-dn	12/307	0,001892051	0,022476309	0	0	2,686456899	16,84433703	EEF1B2;C1QA;RPS17;RPL32;ARL2BP;RPS8;RPS6;ARL6IP5;RPSA;RPS3A;RPL39;TPT1
Leflunomide-60_mg/kg_in_Corn_Oil-Rat-Bone_marrow-5d-up	12/307	0,001892051	0,022442459	0	0	2,686456899	16,84433703	EEF1B2;C1QA;RPL32;RPS19;RPS5;NKG7;RPL35A;RPS20;RPL10A;SEPP1;RPL39;GOLGA7
Oxfendazole-1500_mg/kg_in_CMC-Rat-Liver-3d-up	12/307	0,001892051	0,022408711	0	0	2,686456899	16,84433703	EEF1B2;RPS17;CXADR;RPL32;RPS19;RPS5;RPS6;RPL35A;RPS20;RPSA;RPL10A;RPL39
Tenidap-75_mg/kg_in_Corn_Oil-Rat-Kidney-5d-up	12/307	0,001892051	0,022375064	0	0	2,686456899	16,84433703	RPS17;RPL32;RPS19;ARL2BP;RPS5;RPS6;RPL35A;RPS20;RPSA;RPS3A;RPL10A;RPL39
Oxaliplatin-8_mg/kg_in_Saline-Rat-Bone_marrow-3d-dn	11/267	0,001922967	0,022706582	0	0	2,831512156	17,70795378	EEF1B2;RPS17;RPL32;RPS8;FEM1B;FUNDC1;RPS6;RPS20;RPS3A;RPL39;TPT1
Doxorubicin-1_mg/kg_in_Saline-Rat-Kidney-5d-dn	12/308	0,001943817	0,022918419	0	0	2,677734636	16,71736952	EEF1B2;RPS17;RPL32;RPS19;RPS8;RPS5;RPS6;ARL6IP5;RPSA;RPS3A;SEPP1;TPT1
TNF-alpha-0.25_mg/kg_in_RSA/Saline-Rat-Liver-1d-up	12/308	0,001943817	0,022884161	0	0	2,677734636	16,71736952	EEF1B2;RPS17;RPL32;RPS19;RPS8;RPS6;RPSA;RPS3A;RPL10A;SEPP1;RPL39;TPT1
Aminocaproic_Acid-2000_mg/kg_in_Water-Rat-Kidney-5d-up	13/350	0,001951675	0,022942379	0	0	2,552773687	15,92692651	RPL32;RPS5;RPL13A;RPL35A;RPSA;RPS3A;SEPP1;TOMM20;EEF1B2;RPS17;ARL6IP5;RPS20;RPL39
Camptothecin-2.5_mg/kg_in_Saline-Rat-Bone_marrow-1d-up	13/350	0,001951675	0,022908188	0	0	2,552773687	15,92692651	C1QA;RPL32;RPS5;NKG7;RPL35A;RPSA;RPL10A;SEPP1;EEF1B2;RPS17;RPS19;RPL39;TPT1
Idarubicin-4_mg/kg_in_Saline-Rat-Heart-14d-up	11/268	0,001979999	0,023206055	0	0	2,820946812	17,55943231	EEF1B2;C1QA;RPS19;ARL2BP;RPS8;RPS6;RPS3A;RAPGEF5;RPL10A;GOLGA7;TPT1
Ethylene_Glycol-352_mg/kg_in_Water-Rat-Kidney-3d-dn	9/191	0,001987352	0,023257633	0	0	3,238516759	20,14665737	RPL32;RPS19;ARL2BP;RPS8;RPS6;RPSA;RPL10A;TPT1;PDRG1
Bromfenac-5_mg/kg_in_CMC-Rat-Liver-1d-up	12/309	0,00199674	0,023332826	0	0	2,669068829	16,59157085	EEF1B2;RPL32;RPS19;RPS8;RPS5;RPS6;RPS20;RPSA;RPS3A;RPL10A;RPL39;TPT1
Diclofenac-3.5_mg/kg_in_Corn_Oil-Rat-Liver-0.25d-dn	12/309	0,00199674	0,023298259	0	0	2,669068829	16,59157085	EEF1B2;C1QA;RPS17;RPL32;RPS8;RPL35A;RPS20;RPSA;RPS3A;SEPP1;RPL39;TPT1
Doxorubicin-0.65_mg/kg_in_Saline-Rat-Liver-3d-up	12/309	0,00199674	0,023263794	0	0	2,669068829	16,59157085	EEF1B2;RPS17;RPL32;RPS19;RPS5;RPS6;RPL35A;RPS20;RPS3A;RPL10A;SEPP1;RPL39
Ethanol-3000_mg/kg_in_Saline-Rat-Liver-5d-dn	12/309	0,00199674	0,023229431	0	0	2,669068829	16,59157085	EEF1B2;RPS17;RPL32;RPS19;CLP1;RPS8;RPS6;RPL35A;RPS20;RPSA;RPS3A;TPT1
Ethosuximide-1200_mg/kg_in_Water-Rat-Brain-5d-up	12/309	0,00199674	0,023195169	0	0	2,669068829	16,59157085	EEF1B2;C1QA;RPS17;RPL32;RPS19;ARL2BP;RPS6;ARL6IP5;RPS20;RPS3A;GOLGA7;TPT1
Methylparaben-1500_mg/kg_in_CMC-Rat-Liver-3d-up	12/309	0,00199674	0,023161008	0	0	2,669068829	16,59157085	RPL32;RPS19;RPS5;RPS6;RPL35A;RPS20;RPSA;RPS3A;RPL10A;SEPP1;RPL39;GOLGA7
Raloxifene-650_mg/kg_in_Corn_Oil-Rat-Liver-3d-up	13/351	0,002000964	0,02317587	0	0	2,545500827	15,81806355	RPL32;RPS8;RPS5;RPS6;RPL35A;RPSA;RPS3A;RPL10A;EEF1B2;RPS17;RPS19;RPL39;TPT1
Acyclovir-980_mg/kg_in_Saline-Rat-Bone_marrow-3d-dn	11/269	0,0020384	0,023574798	0	0	2,810460021	17,41245841	EEF1B2;C1QA;RPL32;RPS19;RPS8;RPS6;ARL6IP5;RPS20;RPSA;RPL10A;SEPP1
Azathioprine-20_mg/kg_in_Corn_Oil-Rat-Liver-5d-dn	11/269	0,0020384	0,023540231	0	0	2,810460021	17,41245841	C1QA;RPS17;RPL32;RPS8;RPS6;ARL6IP5;RPS20;RPSA;RPS3A;RPL10A;SEPP1
Diethylstilbestrol-280_mg/kg_in_Corn_Oil-Rat-Liver-3d-up	11/269	0,0020384	0,023505765	0	0	2,810460021	17,41245841	EEF1B2;RPS17;RPS19;RPS8;FEM1B;RPS6;RPS20;RPSA;RPS3A;RPL10A;TPT1
Indomethacin-4.5_mg/kg_in_Water-Rat-Kidney-3d-dn	11/269	0,0020384	0,0234714	0	0	2,810460021	17,41245841	EEF1B2;RPS17;RPL32;RPS19;RPS8;FUNDC1;RPS6;RPSA;RPL10A;RPL39;SEPP1
Ethylene_Glycol-3525_mg/kg_in_Water-Rat-Bone_marrow-3d-dn	12/310	0,002050839	0,023580157	0	0	2,660458929	16,46692739	RPS19;ARL2BP;CLP1;RPS8;FEM1B;FUNDC1;RPS6;RPS20;RPSA;RPS3A;RPL39;TPT1
Ketoconazole-227_mg/kg_in_Corn_Oil-Rat-Liver-1d-up	12/310	0,002050839	0,023545783	0	0	2,660458929	16,46692739	EEF1B2;RPS17;RPL32;RPS19;RPS8;RPS5;RPS6;RPSA;RPS3A;RPL10A;SEPP1;RPL39
Olanzapine-23_mg/kg_in_CMC-Rat-Liver-1d-up	12/310	0,002050839	0,02351151	0	0	2,660458929	16,46692739	EEF1B2;RPS17;RPL32;RPS19;RPS5;RPS6;RPL35A;RPS20;RPSA;RPS3A;RPL39;TPT1
Terfenadine-750_mg/kg_in_CMC-Rat-Heart-3d-up	12/310	0,002050839	0,023477336	0	0	2,660458929	16,46692739	EEF1B2;RPL32;ARL2BP;RPS8;RPS6;RPS20;RPS3A;RAPGEF5;RPL10A;SEPP1;RPL39;TPT1
Zileuton-450_mg/kg_in_Corn_Oil-Rat-Kidney-1d-up	12/310	0,002050839	0,023443262	0	0	2,660458929	16,46692739	RPS17;RPL32;RPS19;RPS8;RPS5;RPS6;RPS20;RPSA;RPS3A;RAPGEF5;RPL10A;TOMM20
Dexfenfluramine-29_mg/kg_in_Water-Rat-Kidney-5d-dn	10/230	0,002065552	0,023577231	0	0	2,988196623	18,47410029	RPS17;RPL32;RPS8;RPS6;ARL6IP5;RPL35A;RPS20;RPSA;RPS3A;SEPP1
Rapamycin-60_mg/kg_in_CMC-Rat-Heart-5d-up	11/270	0,002098195	0,023915175	0	0	2,80005091	17,2670115	EEF1B2;RPL32;RPS19;ARL2BP;RPS8;RPS6;RPS20;RPSA;RPS3A;RPL10A;TPT1
Lead_(II)_Acetate-200_mg/kg_in_Saline-Rat-Kidney-1d-up	13/353	0,002102625	0,023931036	0	0	2,531078726	15,60300834	RPL32;RPS8;RPS5;RPL13A;RPL35A;RPSA;RPS3A;TOMM20;RPS17;ARL6IP5;RPS20;RPL39;TPT1
123-Trichloropropane-108_mg/kg_in_CMC-Rat-Heart-5d-up	12/311	0,002106133	0,023936373	0	0	2,651904399	16,34342573	EEF1B2;RPS17;RPL32;ARL2BP;RPS8;ARL6IP5;RPS20;RPSA;RPS3A;RPL10A;RPL39;TOMM20
Ceramide_C2-44_uM_in_DMSO-Rat-Primary_rat_hepatocytes-0.67d-up	12/311	0,002106133	0,023901882	0	0	2,651904399	16,34342573	ARL2BP;RPS8;RPS5;RPS6;ARL6IP5;RPL35A;RPS3A;RPL10A;RPL39;GOLGA7;TOMM20;PDRG1
Gentamicin-267_mg/kg_in_Corn_Oil-Rat-Kidney-5d-up	12/311	0,002106133	0,023867491	0	0	2,651904399	16,34342573	C1QA;RPS17;CXCL11;RPL32;RPS19;RPS8;FEM1B;RPS6;RPS3A;RPL10A;RPL39;PDRG1
Nitrofurantoin-76_mg/kg_in_CMC-Rat-Liver-1d-dn	12/311	0,002106133	0,023833199	0	0	2,651904399	16,34342573	EEF1B2;RPS17;RPL32;RPS19;RPS8;RPS5;ARL6IP5;RPL35A;RPS20;RPSA;RPS3A;RPL10A
Progesterone-11.3_mg/kg_in_Corn_Oil-Rat-Liver-0.25d-up	12/311	0,002106133	0,023799005	0	0	2,651904399	16,34342573	EEF1B2;C1QA;RPS17;RPL32;RPS19;RPS8;FEM1B;RPS6;RPS20;RPS3A;RPL10A;SEPP1
Streptomycin-120_mg/kg_in_Saline-Rat-Bone_marrow-1d-dn	10/231	0,002131975	0,024056496	0	0	2,975260707	18,29995556	EEF1B2;RPS17;RPL32;RPS19;FUNDC1;NKG7;RPL35A;RPS20;RPSA;RPL39
Cadmium_Chloride-8_uM_in_DMSO-Rat-Primary_rat_hepatocytes-1d-dn	13/354	0,00215503	0,024281858	0	0	2,523928786	15,49679781	RPL32;FEM1B;RPS5;RPS6;RPS3A;RPL10A;SEPP1;EEF1B2;RPS17;RPS19;ARL2BP;RPL39;TPT1
Carmustine-16_mg/kg_in_Corn_Oil-Rat-Spleen-5d-up	11/271	0,002159409	0,024296439	0	0	2,789718619	17,12307135	C1QA;CXCL11;RPL32;RPS19;CLP1;RPS8;FUNDC1;RPS20;RPS3A;SEPP1;TPT1
Chlorambucil-4.5_mg/kg_in_Corn_Oil-Rat-Liver-5d-up	11/271	0,002159409	0,024261779	0	0	2,789718619	17,12307135	EEF1B2;RPS17;RPL32;RPS19;RPS8;RPS6;RPS20;RPSA;RPS3A;RPL10A;RPL39
Beta-Estradiol-0.3_mg/kg_in_Corn_Oil-Rat-Liver-1d-up	12/312	0,002162643	0,024263498	0	0	2,643404705	16,22105264	EEF1B2;RPS17;RPL32;RPS19;RPS8;RPS6;RPS20;RPSA;RPL10A;SEPP1;RPL39;TPT1
Bithionol-59_mg/kg_in_Corn_Oil-Rat-Bone_marrow-3d-up	12/312	0,002162643	0,024228984	0	0	2,643404705	16,22105264	EEF1B2;C1QA;RPS17;RPL32;ARL2BP;RPS8;RPS5;RPL35A;RPS20;RPS3A;RPL39;TPT1
Calcitriol-0.04_mg/kg_in_CMC-Rat-Kidney-3d-up	12/312	0,002162643	0,024194568	0	0	2,643404705	16,22105264	EEF1B2;C1QA;CXCL11;RPS19;RPS8;RPS6;RPS20;RPSA;RPS3A;RPL10A;RPL39;TPT1
Citric_Acid-3000_mg/kg_in_Water-Rat-Liver-1d-up	12/312	0,002162643	0,02416025	0	0	2,643404705	16,22105264	EEF1B2;C1QA;RPS17;RPL32;RPS19;RPS8;RPS5;RPS6;RPL35A;RPS20;RPSA;RPS3A
Cyclosporin_A-350_mg/kg_in_Corn_Oil-Rat-Bone_marrow-3d-up	12/312	0,002162643	0,024126028	0	0	2,643404705	16,22105264	EEF1B2;C1QA;RPL32;RPS8;RPS5;RPS6;NKG7;RPL35A;RPS20;RPL10A;RPL39;TPT1
Cytarabine-487_mg/kg_in_Saline-Rat-Bone_marrow-3d-dn	12/312	0,002162643	0,024091904	0	0	2,643404705	16,22105264	EEF1B2;RPS17;RPL32;RPS8;FEM1B;RPS6;NKG7;RPS20;RPSA;RPS3A;RPL39;TPT1
Erythromycin-1500_mg/kg_in_CMC-Rat-Liver-3d-up	12/312	0,002162643	0,024057876	0	0	2,643404705	16,22105264	RPL32;RPS19;RPS8;RPS5;RPS6;RPL35A;RPS20;RPSA;RPS3A;RPL10A;RPL39;TPT1
Gemfibrozil-100_mg/kg_in_Corn_Oil-Rat-Kidney-1d-dn	12/312	0,002162643	0,024023943	0	0	2,643404705	16,22105264	EEF1B2;RPS17;RPL32;RPS19;RPS6;RPL35A;RPS20;RPSA;RPS3A;RPL10A;SEPP1;RPL39
Isoeugenol-1560_mg/kg_in_CMC-Rat-Liver-5d-dn	12/312	0,002162643	0,023990107	0	0	2,643404705	16,22105264	EEF1B2;C1QA;RPS17;RPL32;RPS8;RPS5;RPL35A;RPS20;RPSA;RPS3A;RPL10A;TPT1
Mevastatin-1200_mg/kg_in_CMC-Rat-Kidney-3d-dn	12/312	0,002162643	0,023956366	0	0	2,643404705	16,22105264	RPL32;RPS19;ARL2BP;CLP1;RPS8;FEM1B;RPS6;RPS20;RPSA;RPL10A;RPL39;TPT1
Salicylamide-1300_mg/kg_in_CMC-Rat-Kidney-5d-dn	12/312	0,002162643	0,023922719	0	0	2,643404705	16,22105264	EEF1B2;RPS17;RPL32;RPS19;RPS8;RPS5;RPS6;RPL35A;RPS20;RPSA;RPL10A;SEPP1
Sodium_Orthovanadate-83_mg/kg_in_CMC-Rat-Kidney-5d-up	12/312	0,002162643	0,023889167	0	0	2,643404705	16,22105264	EEF1B2;C1QA;RPL32;RPS19;RPS8;RPS6;RPS20;RPSA;RAPGEF5;RPL10A;RPL39;TPT1
NN-Dimethylformamide-140_mg/kg_in_Saline-Rat-Bone_marrow-1d-up	10/232	0,002200108	0,024268977	0	0	2,962436308	18,12788505	EEF1B2;C1QA;RPS17;RPS19;RPS8;RPS6;RPS20;RPS3A;RPL39;TPT1
1-Naphthyl_Isothiocyanate-60_mg/kg_in_Corn_Oil-Rat-Liver-0.25d-up	12/313	0,002220388	0,024458424	0	0	2,634959323	16,0997951	EEF1B2;RPS17;RPL32;RPS19;RPS8;RPS6;RPL35A;RPS20;RPS3A;RPL10A;SEPP1;RPL39
Bezafibrate-100_mg/kg_in_Corn_Oil-Rat-Kidney-3d-up	12/313	0,002220388	0,024424264	0	0	2,634959323	16,0997951	EEF1B2;RPS17;RPL32;RPS19;RPS8;RPS5;RPL35A;RPS20;RPS3A;RPL39;TPT1;PDRG1
Clarithromycin-476_mg/kg_in_Water-Rat-Liver-3d-up	12/313	0,002220388	0,024390199	0	0	2,634959323	16,0997951	EEF1B2;RPS17;RPL32;RPS19;RPS8;RPS5;RPS6;RPL35A;RPS20;RPS3A;RPL10A;TPT1

Epirubicin-2.7_mg/kg_in_Saline-Rat-Heart-1d-dn	12/313	0,002220388	0,02435623	0	0	2,634959323	16,0997951	EEF1B2;RPS17;RPL32;RPS8;COL5A3;ARL6IP5;RPS20;RPSA;RPS3A;RPL39;GOLGA7;TPT1
Methotrexate-0.3_mg/kg_in_Corn_Oil-Rat-Liver-0.25d-up	12/313	0,002220388	0,024322355	0	0	2,634959323	16,0997951	EEF1B2;RPS17;CXADR;RPL32;RPS19;RPS6;ARL6IP5;RPS20;RPS3A;RPL10A;SEPP1;TPT1
Piclamilast-17_mg/kg_in_CMC-Rat-Heart-3d-up	12/313	0,002220388	0,024288574	0	0	2,634959323	16,0997951	EEF1B2;RPS8;RPS5;FUNDC1;ARL6IP5;RPS20;RPSA;RPS3A;RAPGEF5;RPL10A;RPL39;TOMM20
Secobarbital-70_mg/kg_in_Water-Rat-Liver-1d-up	12/313	0,002220388	0,024254886	0	0	2,634959323	16,0997951	EEF1B2;RPS17;RPL32;RPS19;RPS8;RPS5;RPS6;RPL35A;RPSA;RPS3A;RPL39;TOMM20
Beta-Estradiol_3-Benzoate-25_mg/kg_in_Corn_Oil-Rat-Liver-3d-up	11/272	0,002222067	0,024239607	0	0	2,7794623	16,98061805	EEF1B2;RPS17;RPS19;RPS8;RPS6;RPS20;RPSA;RPS3A;RPL10A;RPL39;TPT1
Staurosporine-1.3_uM_in_DMSO-Rat-Primary_rat_hepatocytes-1d-up	11/272	0,002222067	0,02420608	0	0	2,7794623	16,98061805	EEF1B2;RPS17;CXCL11;RPL32;RPS5;RPS20;RPSA;RPS3A;RPL10A;GOLGA7;SEPP1
Sulindac-132_mg/kg_in_Corn_Oil-Rat-Heart-5d-up	11/272	0,002222067	0,024172647	0	0	2,7794623	16,98061805	EEF1B2;C1QA;RPS17;RPS19;RPS8;RPS6;RPS20;RPS3A;RPL10A;RPL39;TPT1
Chlorambucil-0.6_mg/kg_in_Corn_Oil-Rat-Spleen-0.25d-up	12/314	0,002279387	0,024762008	0	0	2,626567733	15,97964024	EEF1B2;C1QA;RPS17;RPL32;RPS5;RPS6;RPSA;RPS3A;RPL10A;SEPP1;RPL39;TPT1
Citalopram-40_mg/kg_in_Corn_Oil-Rat-Liver-5d-up	12/314	0,002279387	0,0247279	0	0	2,626567733	15,97964024	EEF1B2;RPS17;RPL32;RPS8;RPS5;RPS6;RPL35A;RPS20;RPSA;RPL10A;RPL39;TPT1
Disulfiram-500_mg/kg_in_Water-Rat-Liver-3d-up	12/314	0,002279387	0,024693887	0	0	2,626567733	15,97964024	EEF1B2;RPS17;RPL32;RPS19;RPS5;RPS6;RPS20;RPSA;RPS3A;RPL10A;SEPP1;RPL39
NN-Dimethylformamide-1400_mg/kg_in_Saline-Rat-Liver-3d-up	12/314	0,002279387	0,024659967	0	0	2,626567733	15,97964024	EEF1B2;RPS17;RPL32;RPS19;RPS6;RPS20;RPSA;RPS3A;RPL10A;SEPP1;RPL39;TPT1
Netilmicin-40_mg/kg_in_Saline-Rat-Kidney-28d-up	12/314	0,002279387	0,024626139	0	0	2,626567733	15,97964024	EEF1B2;C1QA;RPS17;RPL32;RPS19;RPS8;RPS20;RPSA;RPS3A;RPL10A;RPL39;TPT1
Vinblastine-0.3_mg/kg_in_Saline-Rat-Heart-5d-up	12/314	0,002279387	0,024592405	0	0	2,626567733	15,97964024	EEF1B2;C1QA;RPL32;RPS19;FUNDC1;RPS6;RPS20;RPSA;RPL10A;RPL39;TPT1;PDRG1
6-Mercaptopurine-25_mg/kg_in_Corn_Oil-Rat-Liver-5d-dn	11/273	0,002286192	0,024632078	0	0	2,76928112	16,83963204	EEF1B2;RPS17;RPL32;RPS8;RPS6;RPSA;RPS3A;RPL10A;RPL39;SEPP1;TPT1
Hydrocortisone-56_mg/kg_in_Corn_Oil-Rat-Heart-1d-dn	11/273	0,002286192	0,024598428	0	0	2,76928112	16,83963204	RPS17;RPL32;RPS19;ARL2BP;RPS8;RPS6;RPS20;RPS3A;RPL10A;RPL39;TPT1
Lead_(II)_Acetate-600_mg/kg_in_Saline-Rat-Liver-1d-dn	11/273	0,002286192	0,024564869	0	0	2,76928112	16,83963204	EEF1B2;RPL32;RPS19;RPS8;RPS5;RPS6;RPS20;RPSA;RPS3A;RPL10A;TPT1
4-Methylpyrazole-155_mg/kg_in_Saline-Rat-Kidney-5d-dn	12/315	0,002339663	0,025105154	0	0	2,618229422	15,8605754	EEF1B2;RPS17;RPS19;ARL2BP;RPS6;ARL6IP5;RPS20;RPS3A;RPL10A;SEPP1;RPL39;TPT1
Acetazolamide-47_mg/kg_in_Corn_Oil-Rat-Kidney-3d-up	12/315	0,002339663	0,025070998	0	0	2,618229422	15,8605754	EEF1B2;C1QA;RPS17;RPL32;RPS19;RPS8;RPS5;RPS6;RPL13A;RPL35A;RPSA;RPS3A
Guanethidine-132_mg/kg_in_CMC-Rat-Heart-5d-up	12/315	0,002339663	0,025036934	0	0	2,618229422	15,8605754	RPS17;CXCL11;RPL32;RPS5;ARL6IP5;RPL35A;RPS20;RPSA;RPS3A;RAPGEF5;TOMM20;TPT1
Ivermectin-7.5_mg/kg_in_CMC-Rat-Liver-3d-up	12/315	0,002339663	0,025002962	0	0	2,618229422	15,8605754	EEF1B2;RPS17;RPL32;RPS19;RPS6;RPL35A;RPS20;RPSA;RPS3A;RPL10A;SEPP1;RPL39
Nateglinide-625_mg/kg_in_CMC-Rat-Liver-3d-up	12/315	0,002339663	0,024969083	0	0	2,618229422	15,8605754	C1QA;RPS17;RPL32;RPS19;RPS6;RPL35A;RPS20;RPSA;RPS3A;RPL10A;SEPP1;RPL39
Sulbactam-671_mg/kg_in_Saline-Rat-Kidney-1d-up	13/358	0,002375528	0,02531753	0	0	2,495728465	15,08052844	RPL32;RPS6;RPL35A;RPSA;RPS3A;RPL10A;SEPP1;EEF1B2;RPS17;RPS19;ARL6IP5;RPL39;GOLGA7
Citric_Acid-3000_mg/kg_in_Water-Rat-Liver-5d-up	12/316	0,002401234	0,025556914	0	0	2,609943886	15,74258812	C1QA;RPS17;RPL32;RPS19;RPS8;RPS5;RPS6;RPL35A;RPSA;RPS3A;RPL10A;TPT1
Ergocalciferol-15_mg/kg_in_CMC-Rat-Liver-5d-dn	12/316	0,002401234	0,025522424	0	0	2,609943886	15,74258812	C1QA;RPS17;RPL32;RPS19;RPS8;RPS6;RPL35A;RPS20;RPSA;RPL10A;SEPP1;TPT1
Fluocinolone_Acetonide-2.5_mg/kg_in_Corn_Oil-Rat-Liver-5d-dn	12/316	0,002401234	0,025488028	0	0	2,609943886	15,74258812	EEF1B2;C1QA;RPS17;RPL32;RPS19;RPS8;RPS6;RPSA;RPS3A;RPL10A;SEPP1;RPL39
Ibuprofen-54_mg/kg_in_Corn_Oil-Rat-Liver-3d-dn	12/316	0,002401234	0,025453723	0	0	2,609943886	15,74258812	EEF1B2;C1QA;RPS17;RPL32;RPS19;RPS6;RPS3A;RPL10A;SEPP1;RPL39;GOLGA7;TPT1
Indomethacin-9.6_mg/kg_in_Water-Rat-Kidney-1d-up	12/316	0,002401234	0,025419511	0	0	2,609943886	15,74258812	C1QA;RPS17;RPL32;RPS19;RPS8;RPS5;RPS6;RPS20;RPS3A;RPL10A;GOLGA7;TPT1
Loperamide-47_mg/kg_in_CMC-Rat-Intestine-3d-dn	12/316	0,002401234	0,025385391	0	0	2,609943886	15,74258812	EEF1B2;C1QA;RPS17;RPS19;RPS8;RPS5;RPS20;RPSA;RPS3A;RPL10A;SEPP1;TPT1
Sildenafil-2.5_mg/kg_in_Water-Rat-Liver-3d-up	12/316	0,002401234	0,025351363	0	0	2,609943886	15,74258812	EEF1B2;C1QA;RPS17;RPL32;RPS19;RPS8;RPS6;ARL6IP5;RPS20;RPSA;RPS3A;SEPP1
Sotalol-2000_mg/kg_in_Water-Rat-Heart-1d-up	12/316	0,002401234	0,025317425	0	0	2,609943886	15,74258812	RPS17;CXCL11;RPL32;RPS19;RPS8;RPS6;ARL6IP5;RPS20;RPSA;RPS3A;RPL39;TPT1
Dexfenfluramine-29_mg/kg_in_Water-Rat-Kidney-3d-dn	10/235	0,002415089	0,025429464	0	0	2,924617972	17,62380421	RPS17;RPL32;RPS19;RPS6;ARL6IP5;RPL35A;RPS20;RPSA;RPL10A;RPL39
Amikacin-82_mg/kg_in_Saline-Rat-Bone_marrow-3d-dn	11/275	0,002418949	0,025436105	0	0	2,749140893	16,5619852	EEF1B2;RPS17;RPS19;ARL2BP;RPS8;FUNDC1;RPS6;RPL13A;RPS20;RPSA;RPL10A
Benzoic_Acid-1700_mg/kg_in_CMC-Rat-Liver-3d-dn	11/275	0,002418949	0,02540219	0	0	2,749140893	16,5619852	EEF1B2;C1QA;RPS17;RPL32;CLP1;RPS8;RPS5;RPL35A;RPS3A;SEPP1;TPT1
Alfacalcidol-0.043_mg/kg_in_CMC-Rat-Heart-3d-dn	12/317	0,002464121	0,025842101	0	0	2,601710625	15,62566607	EEF1B2;RPS17;RPL32;RPS19;RPS8;FUNDC1;RPS6;RPS20;RPSA;RPS3A;SEPP1;TPT1
Clindamycin-161_mg/kg_in_Saline-Rat-Kidney-5d-up	12/317	0,002464121	0,025807737	0	0	2,601710625	15,62566607	EEF1B2;RPS17;RPL32;RPS19;ARL6IP5;RPL13A;RPL35A;RPS20;RPSA;RPS3A;RPL39;TPT1
Cortisone-206_mg/kg_in_CMC-Rat-Heart-3d-up	12/317	0,002464121	0,025773464	0	0	2,601710625	15,62566607	EEF1B2;RPL32;RPS19;ARL2BP;CLP1;RPS8;RPL35A;RPS20;RPSA;RPS3A;RPL10A;SEPP1
Mitoxantrone-2_mg/kg_in_Saline-Rat-Heart-1d-up	12/317	0,002464121	0,025739281	0	0	2,601710625	15,62566607	C1QA;RPS17;RPL32;RPS19;ARL2BP;RPS8;RPS6;RPS20;RAPGEF5;RPL10A;TPT1;PDRG1
NN-Dimethylformamide-65000_uM_in_DMSO-Rat-Primary_rat_hepatocytes-0.67d-up	12/317	0,002464121	0,025705189	0	0	2,601710625	15,62566607	CXCL11;RPS19;ARL2BP;CLP1;FEM1B;ARL6IP5;RPSA;RPL10A;SEPP1;RPL39;TPT1;PDRG1
Pyrazinamide-1500_mg/kg_in_CMC-Rat-Liver-1d-up	12/317	0,002464121	0,025671188	0	0	2,601710625	15,62566607	EEF1B2;RPS17;RPL32;RPS19;RPS8;RPS5;RPS6;RPL35A;RPS20;RPS3A;RPL10A;TPT1
Sotalol-2000_mg/kg_in_Water-Rat-Liver-1d-dn	12/317	0,002464121	0,025637276	0	0	2,601710625	15,62566607	RPS17;RPL32;RPS19;RPS8;RPS5;RPS6;RPL35A;RPS20;RPS3A;RPL10A;SEPP1;RPL39
Doxorubicin-3_mg/kg_in_Saline-Rat-Bone_marrow-3d-dn	11/276	0,002487631	0,025847738	0	0	2,739180238	16,42528679	EEF1B2;RPS17;RPL32;RPS8;FEM1B;RPS6;RPSA;RPS3A;RPL10A;RPL39;TPT1
Indomethacin-9.6_mg/kg_in_Water-Rat-Heart-5d-up	11/276	0,002487631	0,025813683	0	0	2,739180238	16,42528679	EEF1B2;C1QA;RPL32;RPS19;RPS8;RPS6;ARL6IP5;RPS20;RPSA;RPL10A;RPL39
4-Methylpyrazole-155_mg/kg_in_Saline-Rat-Liver-5d-up	12/318	0,002528346	0,026201647	0	0	2,593529145	15,50979713	C1QA;RPS17;RPL32;RPS19;RPS8;RPS5;RPS6;RPL35A;RPS20;RPSA;RPS3A;RPL39
Cytarabine-487_mg/kg_in_Saline-Rat-Kidney-1d-dn	12/318	0,002528346	0,026167216	0	0	2,593529145	15,50979713	EEF1B2;C1QA;RPS17;RPL32;RPS5;RPS6;RPL35A;RPS20;RPS3A;RPL10A;RPL39;GOLGA7
DActinomycin-0.06_mg/kg_in_Saline-Rat-Bone_marrow-1d-up	12/318	0,002528346	0,026132876	0	0	2,593529145	15,50979713	C1QA;RPS17;RPL32;RPS19;FEM1B;RPS5;RPS6;ARL6IP5;RPSA;RPS3A;RPL10A;RPL39
Hydroxyurea-59_mg/kg_in_Saline-Rat-Spleen-0.25d-dn	12/318	0,002528346	0,026098626	0	0	2,593529145	15,50979713	EEF1B2;CXCL11;RPL32;RPS19;RPS6;RPS20;RPSA;RPS3A;RPL10A;SEPP1;RPL39;TPT1
Spironolactone-300_mg/kg_in_CMC-Rat-Liver-1d-dn	12/318	0,002528346	0,026064465	0	0	2,593529145	15,50979713	EEF1B2;RPS17;RPL32;RPS19;RPS8;RPS6;RPL35A;RPS20;RPS3A;RPL10A;SEPP1;RPL39
Diphenidol-300_mg/kg_in_Water-Rat-Heart-3d-up	13/361	0,002552791	0,026282068	0	0	2,474988339	14,77708621	RPL32;RPS8;RPS6;RPS3A;EEF1B2;RPS17;RPS19;ARL2BP;FUNDC1;ARL6IP5;RPS20;RAPGEF5;GOLGA7
Ethanol-6000_mg/kg_in_Saline-Rat-Bone_marrow-3d-dn	11/277	0,002557884	0,026300126	0	0	2,729291501	16,28998054	EEF1B2;C1QA;RPS17;RPL32;ARL2BP;RPS8;RPS6;NKG7;RPL35A;RPS3A;TPT1
Ethinylestradiol-1480_mg/kg_in_Corn_Oil-Rat-Liver-5d-up	11/277	0,002557884	0,026265836	0	0	2,729291501	16,28998054	RPS17;RPL32;RPS19;RPS8;FEM1B;RPS5;RPS6;ARL6IP5;RPS20;RPSA;RPS3A
Mitomycin_C-0.5_mg/kg_in_Saline-Rat-Spleen-3d-dn	11/277	0,002557884	0,026231636	0	0	2,729291501	16,28998054	EEF1B2;RPS17;RPL32;ARL2BP;RPS8;RPS5;FUNDC1;RPS6;ARL6IP5;RPSA;RPL10A
N-Nitrosodiethylamine-1.67_mg/kg_in_Saline-Rat-Kidney-3d-up	11/277	0,002557884	0,026197525	0	0	2,729291501	16,28998054	C1QA;RPS17;RPL32;RPS19;ARL2BP;ARL6IP5;RPL35A;RPS20;RPS3A;GOLGA7;SEPP1
Clemastine-5.4_mg/kg_in_CMC-Rat-Heart-5d-up	12/319	0,002593929	0,026532184	0	0	2,585398959	15,39496934	EEF1B2;RPS17;RPL32;RPS19;RPS6;RPL35A;RPS20;RPSA;RPS3A;RPL10A;RPL39;TPT1
Fludrocortisone_Acetate-125_mg/kg_in_CMC-Rat-Kidney-1d-dn	12/319	0,002593929	0,026497772	0	0	2,585398959	15,39496934	RPS17;RPL32;RPS19;ARL2BP;CLP1;RPS8;RPS5;RPL35A;RPS20;RPSA;RPS3A;SEPP1
Trihexyphenidyl-588_mg/kg_in_CMC-Rat-Heart-1d-dn	12/319	0,002593929	0,026463448	0	0	2,585398959	15,39496934	EEF1B2;RPS17;RPL32;RPS8;ARL6IP5;RPS20;RPSA;RPS3A;RPL10A;RPL39;TPT1;PDRG1
Cyclosporin_A-70_mg/kg_in_Corn_Oil-Rat-Liver-3d-up	13/362	0,002614235	0,02663611	0	0	2,468151355	14,67756261	C1QA;RPL32;RPS8;RPS5;RPS6;RPL35A;RPSA;RPL10A;EEF1B2;RPS19;RPS20;RPL39;TPT1
Fluvastatin-94_mg/kg_in_Corn_Oil-Rat-Kidney-3d-up	13/362	0,002614235	0,026601697	0	0	2,468151355	14,67756261	RPL32;RPS8;RPS5;RPS6;RPL13A;RPL35A;RPS3A;TOMM20;EEF1B2;RPS17;RPS19;ARL6IP5;RPS20
Levofloxacin-739_mg/kg_in_Water-Rat-Kidney-3d-dn	9/199	0,002621779	0,026644036	0	0	3,108325131	18,47558102	RPS17;RPL32;RPS19;RPS6;RPL35A;RPSA;RPS3A;RPL10A;RPL39
Promethazine-2.3_mg/kg_in_Saline-Rat-Liver-1d-dn	11/278	0,002629734	0,02669044	0	0	2,719473905	16,15604842	EEF1B2;RPS17;RPL32;RPS8;RPS5;RPS6;RPS20;RPS3A;RPL10A;RPL39;SEPP1
Nateglinide-625_mg/kg_in_CMC-Rat-Liver-1d-up	12/320	0,002660891	0,026971913	0	0	2,577319588	15,28117092	RPS17;RPL32;RPS8;RPS5;RPS6;RPL35A;RPS20;RPSA;RPS3A;RPL10A;SEPP1;RPL39
Norethindrone-125_uM_in_DMSO-Rat-Primary_rat_hepatocytes-1d-up	12/320	0,002660891	0,026937244	0	0	2,577319588	15,28117092	EEF1B2;RPS17;CXCL11;RPL32;RPS8;RPS5;RPS6;RPL35A;RPS3A;RPL10A;SEPP1;PDRG1
Rosiglitazone-10_mg/kg_in_Corn_Oil-Rat-Liver-1d-up	12/320	0,002660891	0,026902665	0	0	2,577319588	15,28117092	EEF1B2;C1QA;RPL32;RPS19;RPS8;RPS5;RPS20;RPSA;RPS3A;RPL10A;SEPP1;GOLGA7
Etoposide-188_mg/kg_in_Corn_Oil-Rat-Liver-3d-up	13/363	0,002676886	0,027029681	0	0	2,46135204	14,57883733	C1QA;RPL32;RPS8;RPS5;RPS6;RPL35A;RPSA;RPL10A;EEF1B2;RPS19;RPS20;RPL39;TPT1
6-Methoxy-2-Naphthylacetic_Acid-360_mg/kg_in_Saline-Rat-Kidney-0.25d-up	11/279	0,002703206	0,027260497	0	0	2,709726687	16,02347268	EEF1B2;RPS17;RPL32;RPS19;RPS8;RPS5;RPL35A;RPS20;RPSA;RPS3A;TOMM20
Carboplatin-14_mg/kg_in_Saline-Rat-Spleen-3d-dn	11/279	0,002703206	0,027225637	0	0	2,709726687	16,02347268	EEF1B2;C1QA;RPS17;RPL32;ARL2BP;RPS8;RPS6;ARL6IP5;RPSA;RPS3A;TPT1
Hydrocortisone-56_mg/kg_in_Corn_Oil-Rat-Heart-3d-dn	11/279	0,002703206	0,027190866	0	0	2,709726687	16,02347268	RPS17;RPL32;RPS19;ARL2BP;RPS8;RPS6;RPS20;RPS3A;RPL39;GOLGA7;TPT1
Levofloxacin-739_mg/kg_in_Water-Rat-Heart-1d-up	11/279	0,002703206	0,027156183	0	0	2,709726687	16,02347268	EEF1B2;RPS17;RPL32;RPS19;ARL2BP;RPS8;RPS6;RPS20;RPS3A;RPL39;TPT1
Sulindac-132_mg/kg_in_Corn_Oil-Rat-Liver-5d-up	11/279	0,002703206	0,02712159	0	0	2,709726687	16,02347268	EEF1B2;RPS17;RPL32;RPS19;RPS5;RPS6;RPS20;RPSA;RPS3A;RPL10A;RPL39
Warfarin-0.25_mg/kg_in_CMC-Rat-Liver-3d-up	11/279	0,002703206	0,027087084	0	0	2,709726687	16,02347268	C1QA;RPS17;RPL32;RPS6;RPL35A;RPS20;RPSA;RPS3A;RAPGEF5;RPL10A;RPL39
Diflunisal-207_mg/kg_in_Corn_Oil-Rat-Kidney-1d-up	12/321	0,002729254	0,027313344	0	0	2,569290555	15,16839023	C1QA;CXCL11;RPS19;RPS8;RPS5;RPS6;RPS20;RPSA;RPS3A;RAPGEF5;RPL10A;TPT1
Famciclovir-112_mg/kg_in_Saline-Rat-Spleen-0.25d-up	12/321	0,002729254	0,027278683	0	0	2,569290555	15,16839023	C1QA;RPS17;RPL32;RPS5;NKG7;RPSA;RPS3A;RAPGEF5;RPL10A;SEPP1;RPL39;TPT1
Gabapentin-100_mg/kg_in_Water-Rat-Heart-5d-up	12/321	0,002729254	0,027244109	0	0	2,569290555	15,16839023	RPS17;RPS19;FEM1B;COL5A3;ARL6IP5;RPS3A;RAPGEF5;RPL10A;RPL39;GOLGA7;TPT1;PDRG1
Idebenone-2500_mg/kg_in_CMC-Rat-Liver-0.25d-up	12/321	0,002729254	0,027209623	0	0	2,569290555	15,16839023	C1QA;RPS17;RPL32;RPS19;RPS8;RPS6;RPS20;RPSA;RPS3A;RPL10A;RPL39;TPT1
Sporidesmin_A-0.15_uM_in_DMSO-Rat-Primary_rat_hepatocytes-0.67d-dn	12/321	0,002729254	0,027175224	0	0	2,569290555	15,16839023	EEF1B2;RPS17;RPL32;ARL2BP;RPS8;FUNDC1;RPSA;RPS3A;RPL10A;SEPP1;RPL39;SLC16A3
Gentamicin-656.6_uM_in_DMSO-Rat-Primary_rat_hepatocytes-1d-dn	13/364	0,002740761	0,027255346	0	0	2,454590083	14,48090239	RPL32;RPS8;RPS5;RPS6;RPL35A;RPS3A;PDRG1;RPS17;RPS19;ARL2BP;RPS20;RPL39;TPT1
Amitriptyline-160_mg/kg_in_Water-Rat-Kidney-3d-dn	11/280	0,002778327	0,027594079	0	0	2,700049092	15,89223588	RPS17;RPL32;RPS19;RPS8;RPS6;ARL6IP5;RPL35A;RPSA;RPS3A;RPL10A;RPL39
Cyclosporin_A-350_mg/kg_in_Corn_Oil-Rat-Spleen-3d-up	11/280	0,002778327	0,027559326	0	0	2,700049092	15,89223588	EEF1B2;RPS17;RPL32;RPS8;RPS5;RPS6;RPL35A;RPS20;RPSA;RPS3A;RAPGEF5
Ethinylestradiol-10_mg/kg_in_Corn_Oil-Rat-Liver-5d-up	11/280	0,002778327	0,02752466	0	0	2,700049092	15,89223588	EEF1B2;RPS17;RPL32;RPS19;RPS8;RPS6;RPS20;RPSA;RPS3A;RPL10A;RPL39
Terazosin-657_mg/kg_in_Water-Rat-Heart-1d-up	11/280	0,002778327	0,027490081	0	0	2,700049092	15,89223588	RPS17;RPL32;RPS19;RPS8;FEM1B;RPS6;ARL6IP5;RPS20;RPSA;RAPGEF5;TPT1
Amlodipine-19_mg/kg_in_Corn_Oil-Rat-Liver-5d-up	12/322	0,002799039	0,027660262	0	0	2,561311391	15,05661583	EEF1B2;RPS17;RPL32;RPS19;RPS8;RPS5;RPL35A;RPS20;RPSA;RPS3A;TOMM20;TPT1
Busulfan-9_mg/kg_in_Corn_Oil-Rat-Bone_marrow-1d-up	12/322	0,002799039	0,0276256	0	0	2,561311391	15,05661583	EEF1B2;C1QA;RPS17;RPL32;RPS5;RPL35A;RPS20;RPSA;RPS3A;RPL10A;RPL39;TPT1
Candesartan-1300_mg/kg_in_CMC-Rat-Kidney-5d-up	12/322	0,002799039	0,027591025	0	0	2,561311391	15,05661583	RPL32;RPS19;FEM1B;RPS6;RPL35A;RPSA;RAPGEF5;RPL10A;SEPP1;RPL39;TOMM20;TPT1
Mifepristone-300_mg/kg_in_Corn_Oil-Rat-Liver-1d-up	12/322	0,002799039	0,027556536	0	0	2,561311391	15,05661583	EEF1B2;RPS17;RPL32;RPS19;RPS8;RPS5;RPS6;RPS20;RPSA;RPS3A;RPL10A;TPT1
Progesterone-11.3_mg/kg_in_Corn_Oil-Rat-Liver-1d-up	12/322	0,002799039	0,027522134	0	0	2,561311391	15,05661583	EEF1B2;RPS17;RPL32;RPS19;RPS8;RPS6;RPS20;RPSA;RPS3A;RPL10A;RPL39;TPT1
Rifampin-99_mg/kg_in_CMC-Rat-Liver-3d-up	12/322	0,002799039	0,027487817	0	0	2,561311391	15,05661583	EEF1B2;RPS17;RPL32;RPS19;RPS8;RPS6;RPS20;RPSA;RPS3A;RPL10A;SEPP1;RPL39
Aspirin-375_mg/kg_in_Corn_Oil-Rat-Kidney-1d-up	13/365	0,002805879	0,027520676	0	0	2,447865179	14,38374987	RPL32;RPS8;RPS5;RPS6;RPL13A;RPL35A;RPSA;RPS3A;RPS17;RPS19;RPS20;RPL39;TPT1
Gentamicin-2900_uM_in_DMSO-Rat-Primary_rat_hepatocytes-0.67d-dn	13/365	0,002805879	0,027486446	0	0	2,447865179	14,38374987	RPL32;RPS8;RPS5;RPS6;RPL35A;RPSA;RPL10A;SEPP1;TOMM20;RPS17;ARL6IP5;RPL39;TPT1
Azathioprine-20_mg/kg_in_Corn_Oil-Rat-Spleen-1d-dn	11/281	0,002855125	0,027934116	0	0	2,690440376	15,76232085	EEF1B2;RPS19;RPS8;RPS5;RPL13A;RPL35A;RPS20;RPSA;RPL10A;TOMM20;TPT1
Pentobarbital-70_mg/kg_in_Water-Rat-Liver-3d-dn	11/281	0,002855125	0,027899458	0	0	2,690440376	15,76232085	EEF1B2;C1QA;RPS17;RPL32;ARL2BP;RPS6;RPS20;RPL10A;RPL39;SEPP1;TPT1
Valeric_Acid-23000_uM_in_DMSO-Rat-Primary_rat_hepatocytes-1d-up	11/281	0,002855125	0,027864886	0	0	2,690440376	15,76232085	EEF1B2;RPS17;RPL32;RPS5;RPL35A;RPS20;RPSA;RPS3A;RPL10A;TOMM20;PDRG1

Dexamethasone-456_uM_in_DMSO-Rat-Primary_rat_hepatocytes-0.67d-dn	12/323	0,002870268	0,027978005	0	0	2,553381635	14,94583641	RPS17;CXCL11;RPS19;RPS8;ARL6IP5;RPSA;RPS3A;SEPP1;RPL39;GOLGA7;TPT1;PDRG1
Ethanol-6000_mg/kg_in_Saline-Rat-Liver-3d-up	12/323	0,002870268	0,027943421	0	0	2,553381635	14,94583641	EEF1B2;RPS17;RPL32;RPS19;RPS8;RPS5;RPS6;RPL35A;RPS20;RPSA;RPS3A;RPL10A
Carvedilol-50_uM_in_DMSO-Rat-Primary_rat_hepatocytes-1d-up	11/282	0,002933626	0,028524982	0	0	2,680899807	15,6337107	EEF1B2;RPS17;RPL32;ARL2BP;RPS5;RPS6;RPS20;RPSA;RPS3A;RPL10A;PDRG1
Insulin-0.56_mg/kg_in_Saline-Rat-Liver-5d-dn	11/282	0,002933626	0,028489809	0	0	2,680899807	15,6337107	RPS17;RPL32;RPS19;CLP1;RPS8;RPS5;RPL35A;RPSA;RPS3A;SEPP1;TPT1
Cisplatin-2_mg/kg_in_Saline-Rat-Kidney-1d-up	13/367	0,002939914	0,028515716	0	0	2,434525314	14,19176103	RPL32;RPS8;RPS6;RPL13A;RPL35A;RPSA;RPS3A;RPS17;RPS19;FUNDC1;ARL6IP5;RPS20;RPL39
Secobarbital-20_mg/kg_in_Water-Rat-Liver-1d-up	12/324	0,002942962	0,028510173	0	0	2,545500827	14,83604083	EEF1B2;RPS17;RPL32;RPS19;RPS5;RPS6;RPL13A;RPL35A;RPS20;RPSA;RPS3A;RPL39
Carboplatin-6_mg/kg_in_Saline-Rat-Spleen-3d-dn	11/283	0,003013857	0,0291611	0	0	2,671426663	15,50638879	EEF1B2;C1QA;RPS17;RPL32;ARL2BP;RPS8;FUNDC1;RPS6;ARL6IP5;RPSA;RPS3A
Fluorouracil-29_mg/kg_in_CMC-Rat-Spleen-5d-up	11/283	0,003013857	0,02912532	0	0	2,671426663	15,50638879	EEF1B2;RPL32;RPS19;RPS5;FUNDC1;RPS6;RPS20;RPSA;RPL10A;RPL39;SEPP1
Leflunomide-30_mg/kg_in_Corn_Oil-Rat-Kidney-3d-up	11/283	0,003013857	0,029089627	0	0	2,671426663	15,50638879	RPS17;RPL32;ARL2BP;RPS8;RPS5;FUNDC1;RPL35A;RPS20;RAPGEF5;GOLGA7;TPT1
Procarbazine-27_mg/kg_in_Water-Rat-Bone_marrow-3d-dn	11/283	0,003013857	0,029054022	0	0	2,671426663	15,50638879	RPS17;RPL32;ARL2BP;RPS8;FEM1B;FUNDC1;RPS6;RPS20;RPSA;RPS3A;TPT1
Acemetacin-2_mg/kg_in_Corn_Oil-Rat-Liver-5d-up	12/325	0,003017145	0,02905016	0	0	2,537668517	14,72721811	EEF1B2;C1QA;RPS17;RPL32;RPS8;RPS5;RPL35A;RPSA;RPS3A;SEPP1;RPL39;TPT1
Bromisovalum-250_mg/kg_in_Corn_Oil-Rat-Kidney-3d-dn	12/325	0,003017145	0,02901469	0	0	2,537668517	14,72721811	EEF1B2;RPS17;RPL32;RPS19;RPS8;RPS6;RPS20;RPSA;RPS3A;SEPP1;RPL39;TPT1
Cadmium_Acetate-167_mg/kg_in_Water-Rat-Kidney-3d-up	12/325	0,003017145	0,028979306	0	0	2,537668517	14,72721811	RPS17;RPL32;RPS19;RPS8;RPS5;RPS6;RPL13A;RPL35A;RPS20;RPS3A;RPL39;TPT1
Cisplatin-2_mg/kg_in_Saline-Rat-Spleen-1d-dn	12/325	0,003017145	0,028944009	0	0	2,537668517	14,72721811	EEF1B2;C1QA;RPS17;RPL32;RPS8;RPS5;FUNDC1;RPL13A;RPL35A;RPSA;RPS3A;SEPP1
Isoprenaline-4.2_mg/kg_in_Saline-Rat-Heart-3d-up	12/325	0,003017145	0,028908797	0	0	2,537668517	14,72721811	EEF1B2;C1QA;RPS19;RPS8;RPS5;RPS6;RPS20;RPSA;RPS3A;RPL10A;SEPP1;RPL39
Clotrimazole-52_mg/kg_in_Corn_Oil-Rat-Liver-3d-dn	12/326	0,003092837	0,029598036	0	0	2,529884258	14,61935741	EEF1B2;C1QA;RPL32;RPS19;RPS8;RPS6;RPL35A;RPS20;RPL10A;SEPP1;RPL39;TPT1
Methotrexate-0.3_mg/kg_in_Corn_Oil-Rat-Kidney-0.25d-up	12/326	0,003092837	0,029562116	0	0	2,529884258	14,61935741	EEF1B2;RPS17;RPS19;FUNDC1;RPS6;RPS20;RPS3A;RAPGEF5;RPL10A;SEPP1;RPL39;GOLGA7
NN-Dimethylformamide-700_mg/kg_in_Saline-Rat-Liver-3d-up	12/326	0,003092837	0,029526284	0	0	2,529884258	14,61935741	EEF1B2;RPS17;RPL32;RPS19;RPS8;RPS6;RPS20;RPS3A;RPL10A;SEPP1;RPL39;TPT1
Sulfadimethoxine-1100_mg/kg_in_CMC-Rat-Kidney-5d-up	12/326	0,003092837	0,029490537	0	0	2,529884258	14,61935741	EEF1B2;RPS17;CXCL11;RPL32;ARL2BP;ARL6IP5;RPL13A;RPL35A;RPSA;SEPP1;RPL39;TPT1
Cyclopropane_Carboxylic_Acid-375_mg/kg_in_Water-Rat-Liver-5d-up	11/284	0,003095846	0,029483532	0	0	2,662020231	15,38033877	EEF1B2;RPL32;RPS19;RPS8;RPS5;RPS6;RPS20;RPS3A;RPL10A;RPL39;TPT1
Losartan-1000_mg/kg_in_Water-Rat-Kidney-3d-dn	11/284	0,003095846	0,029447923	0	0	2,662020231	15,38033877	RPS17;RPL32;RPS19;RPS8;RPS6;ARL6IP5;RPL35A;RPSA;RPL10A;RPL39;SEPP1
Zidovudine-1540_mg/kg_in_Corn_Oil-Rat-Liver-1d-up	11/284	0,003095846	0,029412401	0	0	2,662020231	15,38033877	RPL32;RPS19;ARL2BP;RPS8;FEM1B;RPS6;RPS20;RPS3A;RPL10A;RPL39;SEPP1
Stanozolol-150_mg/kg_in_Water-Rat-Liver-1d-up	10/244	0,003162982	0,030014034	0	0	2,816742719	16,21384692	RPL32;RPS19;RPS5;RPS6;RPL35A;RPS20;RPSA;RPS3A;RPL10A;RPL39
Bithionol-333_mg/kg_in_Corn_Oil-Rat-Bone_marrow-3d-up	12/327	0,003170062	0,030045013	0	0	2,522147609	14,51244804	EEF1B2;C1QA;RPS17;RPL32;RPS8;RPS5;NKG7;RPL35A;RPS20;RPS3A;RPL39;TPT1
Clofibrate-130_mg/kg_in_Corn_Oil-Rat-Kidney-3d-dn	12/327	0,003170062	0,030008901	0	0	2,522147609	14,51244804	EEF1B2;RPS17;RPL32;RPS19;RPS6;ARL6IP5;RPL35A;RPS20;RPSA;RPL10A;RPL39;TPT1
Fludrocortisone_Acetate-125_mg/kg_in_CMC-Rat-Liver-1d-dn	12/327	0,003170062	0,029972876	0	0	2,522147609	14,51244804	EEF1B2;C1QA;RPS17;RPL32;RPS19;RPS8;RPS5;RPS6;RPL35A;RPS3A;RPL10A;SEPP1
Foscarnet-350_mg/kg_in_Saline-Rat-Kidney-3d-dn	12/327	0,003170062	0,029936937	0	0	2,522147609	14,51244804	RPS17;RPL32;ARL2BP;RPS5;RPS6;RPS20;RPSA;RPS3A;RPL10A;SEPP1;RPL39;TPT1
Ibuprofen-54_mg/kg_in_Corn_Oil-Rat-Kidney-3d-up	12/327	0,003170062	0,029901084	0	0	2,522147609	14,51244804	C1QA;RPS17;RPL32;ARL2BP;RPS8;RPS5;RPS6;RPL13A;RPL35A;RPS20;RPSA;RPS3A
Naproxen-10_mg/kg_in_Corn_Oil-Rat-Kidney-3d-up	12/327	0,003170062	0,029865318	0	0	2,522147609	14,51244804	EEF1B2;RPS17;RPS8;RPS5;RPL13A;RPL35A;RPS20;RPSA;RPS3A;RPL39;TOMM20;TPT1
Stannous_Fluoride-90_mg/kg_in_Water-Rat-Kidney-3d-up	12/327	0,003170062	0,029829636	0	0	2,522147609	14,51244804	EEF1B2;C1QA;RPS17;RPL32;RPS19;RPS6;RPS20;RPS3A;RAPGEF5;SEPP1;RPL39;TPT1
Vancomycin-160_mg/kg_in_Saline-Rat-Kidney-1d-up	12/327	0,003170062	0,02979404	0	0	2,522147609	14,51244804	EEF1B2;RPS17;RPL32;RPS19;RPS8;RPS6;ARL6IP5;RPL13A;RPL35A;RPSA;RPS3A;RPL39
Aspirin-375_mg/kg_in_Corn_Oil-Rat-Liver-0.25d-up	11/285	0,00317962	0,029848257	0	0	2,652679809	15,25554453	EEF1B2;RPS17;RPL32;RPS19;RPS8;RPS6;RPL35A;RPS20;RPSA;RPL10A;SEPP1
Desloratadine-57_uM_in_DMSO-Rat-Primary_rat_hepatocytes-0.67d-up	11/285	0,00317962	0,029812723	0	0	2,652679809	15,25554453	EEF1B2;CXCL11;RPS8;FEM1B;RPS6;ARL6IP5;RPSA;RPL10A;RPL39;GOLGA7;TPT1
Famciclovir-1200_mg/kg_in_Saline-Rat-Bone_marrow-5d-up	11/285	0,00317962	0,029777274	0	0	2,652679809	15,25554453	C1QA;RPS17;RPS19;ARL2BP;RPS8;FUNDC1;RPS6;RPS20;RPSA;RPS3A;TPT1
Megestrol_Acetate-132_mg/kg_in_CMC-Rat-Liver-1d-dn	11/285	0,00317962	0,029741909	0	0	2,652679809	15,25554453	C1QA;RPS17;RPL32;RPS19;RPS8;FEM1B;RPS6;RPL35A;RPS20;RPS3A;SEPP1
Oxcarbazepine-1600_mg/kg_in_CMC-Rat-Heart-1d-dn	11/285	0,00317962	0,029706628	0	0	2,652679809	15,25554453	RPL32;RPS19;ARL2BP;COL5A3;RPS6;ARL6IP5;RPSA;RPS3A;RPL10A;RPL39;PDRG1
Rapamycin-60_mg/kg_in_CMC-Rat-Heart-3d-up	11/285	0,00317962	0,029671431	0	0	2,652679809	15,25554453	EEF1B2;CXCL11;RPL32;ARL2BP;RPS8;FUNDC1;RPS6;RPS20;RPSA;RPS3A;TPT1
Doxapram-2_mg/kg_in_Saline-Rat-Liver-5d-up	12/328	0,003248841	0,030281507	0	0	2,514458134	14,40647948	C1QA;RPS17;RPL32;RPS8;RPS5;ARL6IP5;RPL13A;RPL35A;RPS20;RPSA;RPS3A;RPL39
Fluvastatin-5_mg/kg_in_Corn_Oil-Rat-Kidney-0.25d-dn	12/328	0,003248841	0,030245713	0	0	2,514458134	14,40647948	EEF1B2;RPS17;RPL32;RPS8;ARL6IP5;RPL35A;RPS20;RPSA;RPS3A;RPL39;TOMM20;TPT1
Glimepiride-2500_mg/kg_in_CMC-Rat-Liver-0.25d-up	12/328	0,003248841	0,030210004	0	0	2,514458134	14,40647948	C1QA;RPS17;RPL32;RPS19;RPS8;RPS6;RPS20;RPSA;RPS3A;RPL10A;RPL39;TPT1
Lead(IV)_Acetate-600_mg/kg_in_Saline-Rat-Bone_marrow-3d-up	12/328	0,003248841	0,030174379	0	0	2,514458134	14,40647948	EEF1B2;RPS17;RPL32;RPS19;RPS8;RPS6;RPL35A;RPS20;RPS3A;RPL10A;RPL39;TPT1
N-Nitrosodiethylamine-1.67_mg/kg_in_Saline-Rat-Spleen-1d-up	12/328	0,003248841	0,030138838	0	0	2,514458134	14,40647948	EEF1B2;C1QA;RPS17;RPS19;RPS5;RPL35A;RPS20;RPSA;RPS3A;RPL10A;SEPP1;RPL39
Sodium_Nitroprusside-6_mg/kg_in_Saline-Rat-Heart-5d-up	12/328	0,003248841	0,030103381	0	0	2,514458134	14,40647948	C1QA;RPS17;RPL32;RPS19;RPS8;RPS5;FUNDC1;RPS6;ARL6IP5;RPL35A;RPSA;RPS3A
TGF_Beta-1_Human_Recombinant-5_ng/ml_in_DMSO-Rat-Primary_rat_hepatocytes-0.67d-dn	10/245	0,00325635	0,030137503	0	0	2,80524581	16,06605861	RPS17;RPS19;RPS8;RPS5;RPS20;RPS3A;RPL10A;RPL39;SEPP1;PDRG1
Acetaminophen-1950_uM_in_DMSO-Rat-Primary_rat_hepatocytes-0.67d-up	11/286	0,003265208	0,030184009	0	0	2,643404705	15,13199023	C1QA;RPL32;RPS19;ARL2BP;CLP1;RPS8;FEM1B;RPS6;RPS20;RPSA;TPT1
Azithromycin-225_mg/kg_in_Water-Rat-Kidney-3d-dn	11/286	0,003265208	0,030148624	0	0	2,643404705	15,13199023	RPS17;RPL32;RPS19;ARL6IP5;RPL35A;RPS20;RPSA;RPL10A;RPL39;SEPP1;TPT1
Clopidogrel-400_mg/kg_in_Corn_Oil-Rat-Bone_marrow-5d-up	11/286	0,003265208	0,030113321	0	0	2,643404705	15,13199023	EEF1B2;C1QA;RPS17;RPL32;RPS8;FEM1B;RPS6;RPL35A;RPS3A;RPL39;SLC16A3
Lead(IV)_Acetate-200_mg/kg_in_Saline-Rat-Spleen-0.25d-dn	11/286	0,003265208	0,0300781	0	0	2,643404705	15,13199023	EEF1B2;CXCL11;RPL32;RPS19;RPS8;FUNDC1;RPL35A;RPS20;RPSA;RPL39;TPT1
Tacrolimus-134_mg/kg_in_CMC-Rat-Heart-3d-up	11/286	0,003265208	0,030042962	0	0	2,643404705	15,13199023	RPS17;CXCL11;RPL32;RPS19;ARL2BP;RPS6;RPS20;RPS3A;RAPGEF5;SEPP1;TPT1
Venlafaxine-320_mg/kg_in_Water-Rat-Kidney-5d-dn	11/286	0,003265208	0,030007906	0	0	2,643404705	15,13199023	RPS17;RPL32;RPS19;RPS6;RPL35A;RPS20;RPSA;RPS3A;RPL10A;RPL39;TPT1
Neomycin-877_mg/kg_in_Corn_Oil-Rat-Kidney-5d-up	12/329	0,003329198	0,030560334	0	0	2,506815404	14,30144133	EEF1B2;RPS17;RPL32;RPS19;RPS8;RPS5;RPS6;RPL35A;RPS20;RPS3A;RPL10A;RPL39
Streptomycin-120_mg/kg_in_Saline-Rat-Bone_marrow-5d-up	12/329	0,003329198	0,030524758	0	0	2,506815404	14,30144133	EEF1B2;RPL32;RPS19;ARL2BP;RPS8;FEM1B;RPS6;RPS20;RPSA;RPS3A;RPL10A;TPT1
Dichlorvos-17_mg/kg_in_Water-Rat-Heart-1d-up	11/287	0,003352637	0,030703915	0	0	2,634194236	15,00966026	RPS17;RPL32;ARL2BP;CLP1;RPS8;FUNDC1;RPS6;RPS20;RPS3A;RPL39;TPT1
Ifosfamide-17_mg/kg_in_Saline-Rat-Spleen-1d-dn	11/287	0,003352637	0,030668254	0	0	2,634194236	15,00966026	EEF1B2;RPS17;RPL32;ARL2BP;RPS8;RPS5;FUNDC1;RPL13A;RPL35A;RPS3A;TPT1
Leucovorin-1500_mg/kg_in_CMC-Rat-Bone_marrow-5d-up	11/287	0,003352637	0,030632676	0	0	2,634194236	15,00966026	C1QA;RPS17;RPL32;RPS8;RPS5;RPS6;RPS20;RPSA;RPS3A;RPL10A;SEPP1
Methotrexate-27_mg/kg_in_Corn_Oil-Rat-Spleen-3d-up	11/287	0,003352637	0,030597181	0	0	2,634194236	15,00966026	EEF1B2;RPL32;RPS8;RPS5;RPS6;RPL35A;RPS20;RPSA;RPS3A;RAPGEF5;SEPP1
Progesterone-148_uM_in_DMSO-Rat-Primary_rat_hepatocytes-1d-up	11/287	0,003352637	0,030561767	0	0	2,634194236	15,00966026	EEF1B2;RPS17;RPL32;CLP1;RPL35A;RPS20;RPS3A;RPL10A;RPL39;TOMM20;TPT1
Thioguanine-24_mg/kg_in_Corn_Oil-Rat-Spleen-1d-up	11/287	0,003352637	0,030526436	0	0	2,634194236	15,00966026	C1QA;CXCL11;RPS8;FUNDC1;RPS6;RPS20;RPSA;RPS3A;RPL10A;SEPP1;TPT1
Amantadine-1500_uM_in_DMSO-Rat-Primary_rat_hepatocytes-0.67d-up	13/373	0,003373729	0,030683015	0	0	2,395364049	13,63378116	RPL32;RPS8;RPS5;RPS6;RPL35A;RPSA;SEPP1;PDRG1;EEF1B2;RPS19;RPS20;RPL39;TPT1
Leflunomide-60_mg/kg_in_Corn_Oil-Rat-Liver-1d-dn	13/373	0,003373729	0,030647625	0	0	2,395364049	13,63378116	C1QA;RPL32;RPS8;RPS6;RPSA;RPS3A;RPL10A;SEPP1;EEF1B2;RPS17;RPS20;RPL39;TPT1
Propylene_Glycol-2000_mg/kg_in_Water-Rat-Kidney-1d-dn	9/207	0,003407086	0,030914985	0	0	2,988196623	16,97862838	RPS17;RPL32;RPS19;RPS6;RPL35A;RPS20;RPSA;RPS3A;RPL39
Aspirin-500_mg/kg_in_Corn_Oil-Rat-Liver-3d-dn	12/330	0,003411156	0,030916301	0	0	2,499218994	14,19732334	EEF1B2;RPL32;RPS19;RPS8;RPS5;RPS6;RPL35A;RPS20;RPSA;RPL10A;RPL39;TPT1
Carboplatin-6_mg/kg_in_Saline-Rat-Liver-1d-dn	12/330	0,003411156	0,030880765	0	0	2,499218994	14,19732334	EEF1B2;RPS17;RPL32;RPS19;RPS8;RPS5;RPS6;RPL35A;RPSA;RPS3A;RPL10A;SEPP1
Cisplatin-2_mg/kg_in_Saline-Rat-Spleen-3d-dn	12/330	0,003411156	0,030845311	0	0	2,499218994	14,19732334	EEF1B2;C1QA;RPS17;RPL32;RPS6;ARL6IP5;RPL35A;RPSA;RPS3A;RPL10A;SEPP1;TPT1
Enrofloxacin-2000_mg/kg_in_CMC-Rat-Kidney-1d-up	12/330	0,003411156	0,030809938	0	0	2,499218994	14,19732334	EEF1B2;RPS17;RPL32;RPS19;RPS6;ARL6IP5;RPL13A;RPL35A;RPS20;RPSA;RPL10A;RPL39
Epirubicin-2.7_mg/kg_in_Saline-Rat-Kidney-1d-up	12/330	0,003411156	0,030774646	0	0	2,499218994	14,19732334	EEF1B2;RPS17;RPL32;RPS19;RPS8;ARL6IP5;RPL13A;RPL35A;RPS20;RPS3A;RPL10A;SEPP1
Fluoxetine-52_mg/kg_in_CMC-Rat-Liver-1d-up	12/330	0,003411156	0,030739435	0	0	2,499218994	14,19732334	EEF1B2;RPS17;RPL32;RPS8;RPS5;RPL13A;RPL35A;RPS20;RPSA;RPS3A;SEPP1;TPT1
Lead(IV)_Acetate-200_mg/kg_in_Saline-Rat-Kidney-1d-up	12/330	0,003411156	0,030704304	0	0	2,499218994	14,19732334	RPS17;CXCL11;RPS19;RPS8;RPS6;ARL6IP5;RPL35A;RPS20;RPSA;RPL10A;RPL39;GOLGA7
Valproic_Acid-850_mg/kg_in_Water-Rat-Kidney-5d-up	12/330	0,003411156	0,030669253	0	0	2,499218994	14,19732334	EEF1B2;C1QA;RPS17;RPL32;RPS8;RPS6;RPL35A;RPS20;RPSA;RPS3A;RPL39;TPT1
Amitraz-75_mg/kg_in_CMC-Rat-Heart-5d-up	11/288	0,003441935	0,030910699	0	0	2,625047728	14,88853927	RPS17;RPL32;RPS19;RPS8;RPS5;RPS6;RPS20;RPSA;RPS3A;SEPP1;TOMM20
Gabapentin-500_uM_in_DMSO-Rat-Primary_rat_hepatocytes-0.67d-dn	11/288	0,003441935	0,030875493	0	0	2,625047728	14,88853927	CXCL11;RPL32;RPS19;ARL2BP;RPS8;RPS5;ARL6IP5;RPSA;RPS3A;GOLGA7;SEPP1
Leflunomide-60_mg/kg_in_Corn_Oil-Rat-Bone_marrow-3d-dn	11/288	0,003441935	0,030840367	0	0	2,625047728	14,88853927	RPS17;RPL32;RPS19;RPS8;FEM1B;RPS6;ARL6IP5;RPS20;RPSA;RPS3A;RPL10A
Mitomycin_C-0.5_mg/kg_in_Saline-Rat-Bone_marrow-1d-dn	11/288	0,003441935	0,030805322	0	0	2,625047728	14,88853927	EEF1B2;RPL32;RPS19;ARL2BP;RPS8;RPS6;RPS20;RPSA;RPS3A;RPL39;TPT1
Urethane-453_mg/kg_in_Water-Rat-Bone_marrow-3d-up	11/288	0,003441935	0,030770355	0	0	2,625047728	14,88853927	C1QA;RPS17;RPL32;RPS19;RPS8;RPS5;RPS6;RPL35A;RPSA;RPL10A;SEPP1
Cisplatin-1.17_mg/kg_in_Saline-Rat-Liver-1d-dn	13/374	0,003450852	0,030815092	0	0	2,388959333	13,54333081	RPL32;RPS8;RPS5;RPS6;RPL35A;RPSA;RPL10A;SEPP1;EEF1B2;RPS17;RPS19;RPS20;RPL39
Promazine-100_mg/kg_in_Corn_Oil-Rat-Liver-0.25d-up	13/375	0,003529403	0,031480835	0	0	2,382588774	13,45358906	C1QA;RPL32;RPS8;RPS5;RPS6;RPSA;RPS3A;RPL10A;RPS17;RPS19;RPS20;RPL39;TPT1
Altretamine-40_mg/kg_in_Corn_Oil-Rat-Liver-3d-dn	11/289	0,003533132	0,031478449	0	0	2,615964518	14,76861214	EEF1B2;RPS17;RPL32;RPS19;ARL2BP;RPS8;RPS6;RPL35A;RPSA;RPL10A;SEPP1
BW-723C86-50_mg/kg_in_Saline-Rat-Heart-1d-up	11/289	0,003533132	0,03144288	0	0	2,615964518	14,76861214	EEF1B2;C1QA;RPS19;RPS8;RPS6;RPS20;RPSA;RPS3A;RAPGEF5;RPL10A;RPL39
Lead_(II)_Acetate-200_mg/kg_in_Saline-Rat-Kidney-3d-up	11/289	0,003533132	0,031407391	0	0	2,615964518	14,76861214	RPS17;CXCL11;RPS8;RPS5;ARL6IP5;RPL13A;RPL35A;RPS20;RPSA;RPL39;TOMM20
Lipopolysaccharide_E._Coli_O55:B5-1.25_mg/kg_in_Saline-Rat-Kidney-5d-up	11/289	0,003533132	0,031371983	0	0	2,615964518	14,76861214	C1QA;RPS19;RPS6;RPS20;RPSA;RAPGEF5;RPL10A;RPL39;GOLGA7;SEPP1;TPT1
Ampicillin-2000_mg/kg_in_CMC-Rat-Kidney-5d-up	12/332	0,003579965	0,031752036	0	0	2,484163458	13,99180754	EEF1B2;C1QA;CXCL11;CXADR;RPL32;RPS19;RPS8;RPS6;ARL6IP5;RPS20;RPSA;RPL10A
Isoprenaline-15_mg/kg_in_Saline-Rat-Liver-3d-up	12/332	0,003579965	0,031716319	0	0	2,484163458	13,99180754	EEF1B2;C1QA;RPS17;RPL32;RPS19;RPS6;RPS20;RPSA;RPS3A;RPL10A;SEPP1;TPT1
Naloxone-76_mg/kg_in_Saline-Rat-Liver-5d-up	12/332	0,003579965	0,031680683	0	0	2,484163458	13,99180754	EEF1B2;C1QA;RPS17;RPL32;RPS19;RPS8;RPL35A;RPS20;RPSA;RPS3A;RPL10A;RPL39
Beta-Estradiol_3-Benzoate-0.25_mg/kg_in_Corn_Oil-Rat-Liver-1d-up	11/290	0,003626256	0,03205431	0	0	2,606943951	14,64986399	RPS17;RPL32;RPS5;RPS6;RPL35A;RPS20;RPSA;RPS3A;RPL39;TOMM20;TPT1
Bisphenol_A-100_mg/kg_in_Corn_Oil-Rat-Bone_marrow-3d-up	11/290	0,003626256	0,032018374	0	0	2,606943951	14,64986399	C1QA;RPS17;ARL2BP;CLP1;RPS8;RPS5;RPS6;RPL35A;RPS20;RPS3A;TPT1
Bromhexine-1200_mg/kg_in_CMC-Rat-Liver-5d-up	11/290	0,003626256	0,03198252	0	0	2,606943951	14,64986399	RPS17;RPL32;RPS19;RPS8;RPS6;RPS20;RPSA;RPS3A;RPL10A;RPL39;SEPP1
Catechol-195_mg/kg_in_Saline-Rat-Liver-0.25d-up	11/290	0,003626256	0,031946745	0	0	2,606943951	14,64986399	EEF1B2;RPS17;RPL32;RPS8;RPS5;RPS6;RPSA;RPS3A;RPL10A;RPL39;TPT1
Closantel-22_mg/kg_in_CMC-Rat-Liver-5d-dn	11/290	0,003626256	0,03191105	0	0	2,606943951	14,64986399	EEF1B2;C1QA;RPS17;RPL32;RPS19;RPS8;RPSA;RPS3A;RPL10A;RPL39;TPT1
Ethylestrenol-390_mg/kg_in_CMC-Rat-Liver-1d-up	11/290	0,003626256	0,031875435	0	0	2,606943951	14,64986399	RPS17;RPL32;RPS19;RPS8;RPS5;ARL6IP5;RPSA;RPS3A;RPL10A;RPL39;SEPP1
Leflunomide-30_mg/kg_in_Corn_Oil-Rat-Spleen-1d-dn	11/290	0,003626256	0,0318399	0	0	2,606943951	14,64986399	EEF1B2;RPS17;RPL32;RPS19;RPS8;RPS5;RPS6;RPL35A;RPS20;RPL10A;RPL39

Procarbazine-54_mg/kg_in_Water-Rat-Bone_marrow-3d-dn	11/290	0,003626256	0,031804443	0	0	2,606943951	14,64986399	RPS17;RPL32;RPS8;FEM1B;FUNDC1;RPS6;ARL6IP5;RPS20;RPS3A;RPL39;TPT1
Sildenafil-14.6_mg/kg_in_Saline-Rat-Liver-0.25d-up	11/290	0,003626256	0,031769066	0	0	2,606943951	14,64986399	EEF1B2;C1QA;RPS17;RPL32;RPS6;RPSA;RPS3A;RPL10A;RPL39;SEPP1;TPT1
Torsemide-3_mg/kg_in_Corn_Oil-Rat-Liver-5d-up	11/290	0,003626256	0,031733767	0	0	2,606943951	14,64986399	EEF1B2;RPS17;RPL32;RPS19;RPS8;RPS5;RPL35A;RPS20;RPSA;RPL39;TPT1
Amitriptyline-160_mg/kg_in_Water-Rat-Kidney-1d-dn	10/249	0,003652001	0,031923592	0	0	2,76018162	15,49146457	RPS17;RPL32;RPS19;RPS6;RPL35A;RPS20;RPSA;RPL10A;RPL39;GOLGA7
Streptozotocin-3000_uM_in_DMSO-Rat-Primary_rat_hepatocytes-0.67d-dn	10/249	0,003652001	0,0318882	0	0	2,76018162	15,49146457	CXCL11;RPS19;RPS5;ARL6IP5;RPS20;RPSA;RPL10A;RPL39;GOLGA7;SEPP1
3-Acetamidophenol-700_mg/kg_in_Corn_Oil-Rat-Kidney-5d-dn	12/333	0,003666864	0,031982523	0	0	2,476703508	13,89038993	EEF1B2;RPS17;RPL32;RPS19;RPS8;RPS6;RPS20;RPSA;RPS3A;SEPP1;RPL39;TPT1
Bromisovalum-250_mg/kg_in_Corn_Oil-Rat-Kidney-5d-dn	12/333	0,003666864	0,031947144	0	0	2,476703508	13,89038993	EEF1B2;RPS17;RPL32;RPS19;RPS8;RPS6;RPS20;RPSA;RPS3A;SEPP1;RPL39;TPT1
Lovastatin-450_mg/kg_in_Corn_Oil-Rat-Kidney-0.25d-up	12/333	0,003666864	0,031911843	0	0	2,476703508	13,89038993	EEF1B2;RPS17;RPL32;RPS5;RPS6;RPL13A;RPS20;RPSA;RPS3A;SEPP1;RPL39;TPT1
Acrolein-20_mg/kg_in_Water-Rat-Heart-5d-up	11/291	0,003721335	0,032350148	0	0	2,59798538	14,53228018	EEF1B2;RPS17;RPL32;RPS8;ARL6IP5;RPS20;RPS3A;RPL10A;RPL39;SEPP1;TPT1
Allyl_Alcohol-32_mg/kg_in_Saline-Rat-Liver-1d-up	11/291	0,003721335	0,032314481	0	0	2,59798538	14,53228018	EEF1B2;RPS17;RPL32;RPS19;RPS8;RPS6;RPS20;RPSA;RPS3A;RPL10A;RPL39
Bleomycin_A2-12_mg/kg_in_Saline-Rat-Bone_marrow-0.25d-up	11/291	0,003721335	0,032278892	0	0	2,59798538	14,53228018	RPL32;RPS19;RPS8;FEM1B;RPS5;FUNDC1;RPS6;RPS20;RPSA;RPL10A;TPT1
Dyphylline-3855_mg/kg_in_CMC-Rat-Heart-3d-up	11/291	0,003721335	0,032243382	0	0	2,59798538	14,53228018	RPS17;RPL32;RPS19;RPS5;RPS6;RPL35A;RPS20;RPSA;RPS3A;TOMM20;TPT1
Sildenafil-300_mg/kg_in_Saline-Rat-Liver-5d-dn	11/291	0,003721335	0,03220795	0	0	2,59798538	14,53228018	EEF1B2;C1QA;RPS17;RPL32;RPS8;RPS5;RPS6;RPSA;RPS3A;RPL10A;RPL39
Lead(IV)_Acetate-200_mg/kg_in_Saline-Rat-Bone_marrow-3d-dn	10/250	0,003756638	0,032477809	0	0	2,749140893	15,35183714	EEF1B2;RPS17;RPL32;RPS8;RPS5;RPS6;NKG7;RPSA;RPS3A;TPT1
Carboplatin-14_mg/kg_in_Saline-Rat-Bone_marrow-1d-up	13/378	0,003773817	0,032590549	0	0	2,36367934	13,1885469	C1QA;RPS8;RPS5;RPS6;NKG7;RPL35A;RPSA;RPS3A;RPL10A;SEPP1;RPS17;RPS20;TPT1
Bisacodyl-1750_mg/kg_in_CMC-Rat-Intestine-3d-dn	11/292	0,003818399	0,032939442	0	0	2,58908817	14,41584627	EEF1B2;C1QA;RPS17;RPS19;ARL2BP;RPS8;RPS6;ARL6IP5;RPSA;RPS3A;SEPP1
Cholecalciferol-8_mg/kg_in_CMC-Rat-Heart-5d-up	11/292	0,003818399	0,032903404	0	0	2,58908817	14,41584627	EEF1B2;C1QA;RPS17;CXCL11;RPS19;RPS8;RPS20;RPSA;RPL10A;SEPP1;RPL39
Famciclovir-112_mg/kg_in_Saline-Rat-Liver-3d-up	11/292	0,003818399	0,032867444	0	0	2,58908817	14,41584627	EEF1B2;RPS17;RPS19;CLP1;RPS8;RPS6;RPL35A;RPS20;RPSA;RPS3A;RPL10A
Pralidoxime_Chloride-48_mg/kg_in_Saline-Rat-Heart-5d-up	11/292	0,003818399	0,032831562	0	0	2,58908817	14,41584627	C1QA;RPS17;RPL32;RPS19;NKG7;RPS20;RPS3A;RPL10A;SEPP1;RPL39;TPT1
Pravastatin-1200_mg/kg_in_Corn_Oil-Rat-Liver-3d-up	11/292	0,003818399	0,032795759	0	0	2,58908817	14,41584627	CXADR;RPS19;RPS8;FEM1B;RPS6;RPS20;RPSA;RPS3A;RPL10A;SEPP1;TPT1
Valproic_Acid-1500_mg/kg_in_Water-Rat-Liver-1d-up	11/292	0,003818399	0,032760034	0	0	2,58908817	14,41584627	RPS17;RPL32;RPS19;RPS8;RPS6;RPS20;RPSA;RPS3A;RPL10A;RPL39;TPT1
D-Tubocurarine_Chloride-0.0033_mg/kg_in_Saline-Rat-Heart-3d-up	12/335	0,003845765	0,032958917	0	0	2,461917218	13,69018679	RPS17;RPL32;RPS19;ARL2BP;RPS8;RPS6;RPS20;RPSA;RPS3A;RPL10A;RPL39;TPT1
Irinotecan-5_mg/kg_in_Saline-Rat-Bone_marrow-1d-dn	12/335	0,003845765	0,032923092	0	0	2,461917218	13,69018679	EEF1B2;RPS17;RPL32;RPS8;FUNDC1;RPS6;RPL35A;RPS20;RPSA;RPS3A;RPL39;TPT1
Mannitol-1000_mg/kg_in_Saline-Rat-Kidney-3d-up	12/335	0,003845765	0,032887345	0	0	2,461917218	13,69018679	C1QA;RPS17;CXCL11;RPL32;RPS8;RPS5;ARL6IP5;RPL13A;RPL35A;RPSA;RPS3A;SEPP1
Carbon_Tetrachloride-775_uM_in_DMSO-Rat-Primary_rat_hepatocytes-1d-dn	11/293	0,003917477	0,033464263	0	0	2,580251692	14,30054808	EEF1B2;RPL32;RPS8;RPS5;RPS6;RPS20;RPS3A;RPL10A;SEPP1;RPL39;TPT1
Iproniazid-46_mg/kg_in_Water-Rat-Brain-5d-up	11/293	0,003917477	0,033428007	0	0	2,580251692	14,30054808	EEF1B2;RPS19;ARL2BP;CLP1;RPS6;ARL6IP5;RPS20;RPL10A;SEPP1;RPL39;GOLGA7
Lomustine-8.75_mg/kg_in_Corn_Oil-Rat-Bone_marrow-5d-dn	11/293	0,003917477	0,033391829	0	0	2,580251692	14,30054808	EEF1B2;RPS17;RPL32;RPS19;RPS8;FUNDC1;RPS6;ARL6IP5;RPS20;RPSA;RPS3A
Mitoxantrone-2_mg/kg_in_Saline-Rat-Heart-5d-up	11/293	0,003917477	0,03335573	0	0	2,580251692	14,30054808	RPS17;RPL32;RPS19;CLP1;RPS8;FUNDC1;RPS6;RPS20;RPS3A;RPL10A;TPT1
Atorvastatin-300_mg/kg_in_Corn_Oil-Rat-Kidney-1d-dn	11/294	0,004018599	0,034179789	0	0	2,571475326	14,18637161	EEF1B2;RPS17;RPL32;RPS19;ARL2BP;RPS8;RPS6;RPS3A;RPL10A;RPL39;TPT1
CerIVastatin-7_mg/kg_in_Corn_Oil-Rat-Kidney-5d-dn	11/294	0,004018599	0,034142917	0	0	2,571475326	14,18637161	EEF1B2;RPS17;RPL32;RPS19;ARL2BP;RPS6;RPS20;RPSA;RPS3A;SEPP1;RPL39
Cholic_Acid-1402_mg/kg_in_Water-Rat-Liver-1d-up	11/294	0,004018599	0,034106125	0	0	2,571475326	14,18637161	EEF1B2;RPS17;RPL32;RPS8;RPS5;RPS6;RPL13A;RPL35A;RPS20;RPSA;RPL39
Clobetasol_Propionate-17_mg/kg_in_Corn_Oil-Rat-Bone_marrow-3d-dn	11/294	0,004018599	0,034069413	0	0	2,571475326	14,18637161	RPS17;RPL32;RPS19;FEM1B;RPS6;RPS20;RPSA;RPS3A;RPL10A;SEPP1;RPL39
Ethanol-3000_mg/kg_in_Saline-Rat-Kidney-3d-up	11/294	0,004018599	0,034032779	0	0	2,571475326	14,18637161	RPS17;ARL2BP;RPS5;ARL6IP5;RPL13A;RPL35A;RPS20;RPSA;RPL39;TOMM20;TPT1
Lidocaine-71_mg/kg_in_Water-Rat-Heart-3d-dn	11/294	0,004018599	0,033996224	0	0	2,571475326	14,18637161	EEF1B2;RPS17;RPL32;RPS8;RPS20;RPSA;RPS3A;RPL10A;SEPP1;RPL39;TPT1
Propylene_Glycol-2000_mg/kg_in_Water-Rat-Liver-3d-up	11/294	0,004018599	0,033959747	0	0	2,571475326	14,18637161	EEF1B2;RPS17;RPL32;RPS8;RPS5;RPS6;ARL6IP5;RPS20;RPSA;RPS3A;SEPP1
Tamoxifen-37.5_uM_in_DMSO-Rat-Primary_rat_hepatocytes-0.67d-up	11/294	0,004018599	0,033923349	0	0	2,571475326	14,18637161	EEF1B2;RPS17;RPS19;ARL2BP;RPS8;RPS5;RPS20;RPS3A;RPL10A;RPL39;PDRG1
4-Methylpyrazole-155_mg/kg_in_Saline-Rat-Kidney-3d-dn	12/337	0,004031632	0,033996927	0	0	2,447306433	13,49342991	EEF1B2;RPS17;RPL32;ARL2BP;RPS8;RPS6;RPS20;RPSA;RPS3A;SEPP1;RPL39;TPT1
Mannitol-1000_mg/kg_in_Saline-Rat-Kidney-1d-up	12/337	0,004031632	0,033960567	0	0	2,447306433	13,49342991	RPS17;RPL32;RPS19;RPS8;RPS6;ARL6IP5;RPL35A;RPS20;RPSA;RPS3A;SEPP1;TOMM20
Clotrimazole-60_uM_in_DMSO-Rat-Primary_rat_hepatocytes-0.67d-dn	13/381	0,004031785	0,033925574	0	0	2,345067691	12,92963883	RPL32;RPS8;RPS5;RPS6;RPSA;RPS3A;SEPP1;RPS17;CXCL11;CLP1;ARL6IP5;RPS20;TPT1
Buflomedil-615_mg/kg_in_CMC-Rat-Heart-3d-up	11/295	0,004121794	0,034645942	0	0	2,56275846	14,07330311	EEF1B2;RPS17;RPL32;RPS19;RPS8;RPS5;FUNDC1;RPS6;RPL35A;RPS20;RPS3A
Clonidine-6_mg/kg_in_Water-Rat-Heart-1d-up	11/295	0,004121794	0,034609006	0	0	2,56275846	14,07330311	C1QA;RPS17;RPL32;RPS19;RPS8;RPS6;RPS20;RPS3A;RAPGEF5;SEPP1;TPT1
Lipopolysaccharide_E._Coli_O55:B5-1.25_mg/kg_in_Saline-Rat-Kidney-1d-up	11/295	0,004121794	0,034572148	0	0	2,56275846	14,07330311	C1QA;CXCL11;RPS19;RPS6;RPS20;RPSA;RAPGEF5;RPL10A;SEPP1;RPL39;TPT1
N-Nitrosodiethylamine-1.67_mg/kg_in_Saline-Rat-Bone_marrow-1d-up	11/295	0,004121794	0,034535369	0	0	2,56275846	14,07330311	C1QA;RPS17;RPL32;RPS19;RPS8;RPS5;RPS6;ARL6IP5;RPL35A;RPS3A;TPT1
Oxyquinoline-68_mg/kg_in_Corn_Oil-Rat-Liver-3d-up	11/295	0,004121794	0,034498669	0	0	2,56275846	14,07330311	EEF1B2;C1QA;RPS17;RPL32;RPS8;RPS6;RPS20;RPS3A;RAPGEF5;SEPP1;TPT1
Paraquat_Dichloride-650_uM_in_DMSO-Rat-Primary_rat_hepatocytes-0.67d-dn	11/295	0,004121794	0,034462046	0	0	2,56275846	14,07330311	RPS17;CXCL11;RPL32;RPS8;RPS5;ARL6IP5;RPL35A;RPSA;RPS3A;TOMM20;TPT1
Acyclovir-980_mg/kg_in_Saline-Rat-Liver-1d-dn	12/338	0,004127237	0,034470964	0	0	2,440065882	13,39632061	EEF1B2;RPS17;RPL32;RPS19;RPS6;RPL35A;RPS20;RPS3A;RPL10A;SEPP1;RPL39;TPT1
Celecoxib-400_mg/kg_in_Corn_Oil-Rat-Liver-3d-dn	12/338	0,004127237	0,034434448	0	0	2,440065882	13,39632061	EEF1B2;RPL32;RPS19;RPS8;RPS5;RPS6;RPL35A;RPS20;RPSA;RPL10A;RPL39;TPT1
Penicillin_G-243_mg/kg_in_Saline-Rat-Kidney-1d-up	12/338	0,004127237	0,034398009	0	0	2,440065882	13,39632061	RPS17;RPL32;RPS19;RPS8;RPS5;RPS6;ARL6IP5;RPL35A;RPS20;RPSA;RPL10A;SEPP1
Daunorubicin-3.25_mg/kg_in_Saline-Rat-Kidney-1d-dn	10/254	0,004199194	0,034960731	0	0	2,705847336	14,80873093	RPS17;RPL32;RPS8;RPS5;ARL6IP5;RPL35A;RPSA;RPS3A;TOMM20;TPT1
44'-Methylenedianiline-81_mg/kg_in_Corn_Oil-Rat-Kidney-5d-dn	12/339	0,004224656	0,035135579	0	0	2,432868047	13,30004528	EEF1B2;RPS17;RPL32;RPS19;ARL2BP;RPS8;RPS6;RPS20;RPSA;RPS3A;SEPP1;RPL39
Dexfenfluramine-29_mg/kg_in_Water-Rat-Heart-5d-dn	12/339	0,004224656	0,035098516	0	0	2,432868047	13,30004528	RPS17;RPL32;RPS19;RPS8;COL5A3;RPS6;RPL35A;RPS20;RPSA;RPS3A;RPL39;TPT1
Rofecoxib-250_mg/kg_in_Corn_Oil-Rat-Liver-3d-dn	12/339	0,004224656	0,035061531	0	0	2,432868047	13,30004528	EEF1B2;RPL32;RPS19;RPS8;RPS5;RPS6;RPL35A;RPS20;RPSA;RPL10A;RPL39;TPT1
Acetaminophen-972_mg/kg_in_Corn_Oil-Rat-Liver-1d-up	11/296	0,004227092	0,035044815	0	0	2,554100492	13,96132901	EEF1B2;RPS17;RPL32;RPS19;RPS8;RPS6;RPS20;RPSA;RPS3A;RPL10A;RPL39
Amineptine-1667_uM_in_DMSO-Rat-Primary_rat_hepatocytes-0.67d-up	11/296	0,004227092	0,035007964	0	0	2,554100492	13,96132901	EEF1B2;RPS19;ARL2BP;CLP1;RPS8;FEM1B;RPS6;RPS20;RPL10A;RPL39;PDRG1
Aztreonam-1100_mg/kg_in_CMC-Rat-Spleen-5d-up	11/296	0,004227092	0,034971191	0	0	2,554100492	13,96132901	EEF1B2;C1QA;RPS19;RPS5;FUNDC1;RPS6;RPS20;RPSA;RPL10A;SEPP1;RPL39
Fluconazole-394_mg/kg_in_Corn_Oil-Rat-Liver-1d-up	11/296	0,004227092	0,034934495	0	0	2,554100492	13,96132901	RPS17;RPL32;RPS19;RPS8;RPS6;RPS20;RPSA;RPS3A;RPL10A;RPL39;TPT1
Flunarizine-126_mg/kg_in_CMC-Rat-Heart-3d-up	11/296	0,004227092	0,034897876	0	0	2,554100492	13,96132901	EEF1B2;RPS17;RPL32;RPS19;RPS8;RPS5;RPS6;RPL35A;RPSA;RPS3A;RPL39
Guanethidine-132_mg/kg_in_CMC-Rat-Heart-1d-dn	11/296	0,004227092	0,034861334	0	0	2,554100492	13,96132901	EEF1B2;RPS17;RPL32;RPS19;RPS8;COL5A3;RPS6;RPSA;RPS3A;RPL10A;RPL39
Lead_(II)_Acetate-200_mg/kg_in_Saline-Rat-Bone_marrow-0.25d-up	11/296	0,004227092	0,034824868	0	0	2,554100492	13,96132901	RPS17;RPL32;RPS8;RPS5;FUNDC1;RPL13A;RPL35A;RPS20;RPSA;RPS3A;TPT1
Clomipramine-115_mg/kg_in_Water-Rat-Heart-1d-dn	12/340	0,004323914	0,035585315	0	0	2,425712553	13,20459497	EEF1B2;RPS17;RPL32;RPS19;FUNDC1;RPS6;RPSA;RPS3A;RPL10A;SEPP1;RPL39;TPT1
17-Methyltestosterone-2000_mg/kg_in_CMC-Rat-Liver-1d-up	11/297	0,004334523	0,035635388	0	0	2,545500827	13,85043595	RPS17;RPL32;RPS19;RPS8;RPS6;RPS20;RPSA;RPS3A;RPL10A;RPL39;TPT1
Carbimazole-400_mg/kg_in_Water-Rat-Liver-3d-up	11/297	0,004334523	0,035598229	0	0	2,545500827	13,85043595	RPS17;RPL32;RPS19;RPS8;RPS5;RPS6;RPSA;RPS3A;RPL10A;SEPP1;RPL39
Cyclosporin_A-350_mg/kg_in_Corn_Oil-Rat-Heart-1d-dn	11/297	0,004334523	0,035561147	0	0	2,545500827	13,85043595	EEF1B2;RPS17;RPL32;RPS19;ARL2BP;RPS8;RPS6;RPL35A;RPS20;RPS3A;RPL10A
Doxepin-147_mg/kg_in_CMC-Rat-Heart-5d-up	11/297	0,004334523	0,035524143	0	0	2,545500827	13,85043595	EEF1B2;RPS17;CXCL11;RPL32;RPS8;RPS5;RPS6;RPL35A;RPS3A;RPL39;TPT1
Norethindrone_Acetate-125_mg/kg_in_Corn_Oil-Rat-Liver-3d-up	11/297	0,004334523	0,035487215	0	0	2,545500827	13,85043595	RPS17;RPS19;RPS8;FEM1B;RPS6;RPS20;RPSA;RPS3A;RPL10A;SEPP1;RPL39
Sulindac-64_mg/kg_in_Corn_Oil-Rat-Liver-5d-up	11/297	0,004334523	0,035450365	0	0	2,545500827	13,85043595	EEF1B2;RPS17;RPL32;RPS19;RPS6;RPS20;RPSA;RPS3A;RPL10A;RPL39;TPT1
Cycloheximide-0.25_mg/kg_in_Water-Rat-Liver-3d-up	13/385	0,00439776	0,035930249	0	0	2,320703352	12,59366781	RPL32;RPS8;RPS5;RPS6;RPL35A;RPSA;RPS3A;RPL10A;EEF1B2;RPS17;RPS19;RPS20;RPL39
Rofecoxib-800_mg/kg_in_Corn_Oil-Rat-Liver-3d-dn	12/341	0,004425035	0,036115619	0	0	2,418599027	13,10996085	EEF1B2;RPL32;RPS19;RPS8;RPS5;RPS6;RPL35A;RPS20;RPSA;RPL10A;RPL39;TPT1
Carmustine-4_mg/kg_in_Corn_Oil-Rat-Bone_marrow-3d-dn	11/298	0,004444117	0,036233817	0	0	2,536958878	13,7406108	RPS19;RPS8;FEM1B;RPS6;RPS20;RPSA;RPS3A;RPL10A;SEPP1;RPL39;GOLGA7
Clozapine-69_uM_in_DMSO-Rat-Primary_rat_hepatocytes-0.67d-up	11/298	0,004444117	0,036196347	0	0	2,536958878	13,7406108	EEF1B2;RPS17;RPL32;RPS19;RPS5;RPS6;RPL35A;RPS20;RPS3A;RPL39;PDRG1
Diethylstilbestrol-2.8_mg/kg_in_Corn_Oil-Rat-Liver-0.25d-up	11/298	0,004444117	0,036158954	0	0	2,536958878	13,7406108	EEF1B2;RPS17;RPS19;RPS8;FEM1B;RPS5;RPS6;RPS20;RPSA;RPS3A;RPL10A
Ketoconazole-114_mg/kg_in_Corn_Oil-Rat-Liver-1d-dn	11/298	0,004444117	0,036121638	0	0	2,536958878	13,7406108	EEF1B2;C1QA;RPS17;RPL32;RPS8;RPS6;RPSA;RPS3A;RPL10A;SEPP1;TPT1
Methyldopa-325_mg/kg_in_Water-Rat-Heart-3d-up	11/298	0,004444117	0,036084399	0	0	2,536958878	13,7406108	RPS17;RPL32;ARL2BP;RPS8;RPS5;RPS6;RPL35A;RPS20;RPSA;RPS3A;TPT1
Miconazole-920_mg/kg_in_Corn_Oil-Rat-Liver-3d-up	11/298	0,004444117	0,036047237	0	0	2,536958878	13,7406108	RPS17;RPL32;RPS19;RPS8;RPS5;RPS6;RPS20;RPSA;RPS3A;RPL10A;TPT1
Bisphenol_A-100_mg/kg_in_Corn_Oil-Rat-Liver-3d-dn	12/342	0,004528043	0,036690191	0	0	2,4115271	13,01613422	EEF1B2;C1QA;RPS17;RPL32;RPS19;RPS6;RPSA;RPS3A;RPL10A;RPL39;GOLGA7;TPT1
Cephalexin-2500_mg/kg_in_CMC-Rat-Kidney-5d-up	12/342	0,004528043	0,036652483	0	0	2,4115271	13,01613422	CXCL11;RPL32;RPS19;ARL2BP;RPS8;RPS6;RPS20;RPSA;RAPGEF5;RPL10A;SEPP1;TPT1
Ibuprofen-90_mg/kg_in_Corn_Oil-Rat-Liver-3d-dn	12/342	0,004528043	0,036614852	0	0	2,4115271	13,01613422	EEF1B2;RPL32;RPS19;RPS8;RPS5;RPS6;RPL35A;RPS20;RPSA;RPL10A;RPL39;TPT1
Naproxen-134_mg/kg_in_Corn_Oil-Rat-Liver-0.25d-up	12/342	0,004528043	0,036577299	0	0	2,4115271	13,01613422	EEF1B2;RPL32;RPS19;ARL2BP;RPS6;RPS20;RPS3A;RPL10A;SEPP1;RPL39;TPT1;PDRG1
6-Mercaptopurine-25_mg/kg_in_Corn_Oil-Rat-Liver-0.25d-up	11/299	0,004555905	0,036764663	0	0	2,528474066	13,63184061	RPS17;RPL32;RPS19;RPS8;RPS6;RPS20;RPSA;RPS3A;RPL10A;SEPP1;TPT1
Antipyrine-1500_mg/kg_in_CMC-Rat-Kidney-5d-up	11/299	0,004555905	0,036727033	0	0	2,528474066	13,63184061	EEF1B2;RPS17;RPL32;RPS8;RPS5;ARL6IP5;RPL13A;RPL35A;RPSA;SEPP1;TOMM20
Fludrocortisone_Acetate-125_mg/kg_in_CMC-Rat-Kidney-3d-up	11/299	0,004555905	0,03668948	0	0	2,528474066	13,63184061	EEF1B2;CXCL11;RPS19;RPS6;ARL6IP5;RPS20;RPSA;RPL10A;SEPP1;RPL39;TPT1
Praziquantel-1200_mg/kg_in_CMC-Rat-Liver-3d-dn	11/299	0,004555905	0,036652003	0	0	2,528474066	13,63184061	C1QA;RPS17;RPL32;RPS8;ARL6IP5;RPL35A;RPS20;RPS3A;SEPP1;RPL39;TPT1
Flurbiprofen-10_mg/kg_in_CMC-Rat-Kidney-5d-up	12/343	0,004632964	0,037233902	0	0	2,404496408	12,92310648	EEF1B2;C1QA;RPS17;RPL32;RPS19;RPS8;RPS6;RPSA;RPS3A;RPL10A;RPL39;PDRG1
Insulin-0.56_mg/kg_in_Saline-Rat-Liver-3d-up	12/343	0,004632964	0,037195947	0	0	2,404496408	12,92310648	EEF1B2;C1QA;RPS17;RPL32;RPS19;RPS6;RPS20;RPS3A;RPL10A;SEPP1;RPL39;TPT1
Sparteine-2222_uM_in_DMSO-Rat-Primary_rat_hepatocytes-1d-dn	12/343	0,004632964	0,03715807	0	0	2,404496408	12,92310648	EEF1B2;RPS17;RPS19;RPS6;ARL6IP5;RPS20;RPSA;RPS3A;SEPP1;RPL39;SLC16A3;TPT1
Sulfamethoxazole-1000_mg/kg_in_Water-Rat-Kidney-3d-up	12/343	0,004632964	0,037120269	0	0	2,404496408	12,92310648	RPS17;RPL32;RPS19;ARL2BP;RPS8;FEM1B;RPS6;RPS20;RPSA;RPS3A;RAPGEF5;TPT1
Allyl_Alcohol-16_mg/kg_in_Saline-Rat-Kidney-0.25d-up	11/300	0,004669918	0,037378327	0	0	2,520045819	13,5241126	EEF1B2;RPS17;RPL32;RPS8;RPS5;RPL35A;RPSA;RPS3A;RPL39;TOMM20;TPT1
Benzoic_Acid-1700_mg/kg_in_CMC-Rat-Liver-5d-up	11/300	0,004669918	0,03734038	0	0	2,520045819	13,5241126	EEF1B2;RPS17;RPL32;RPS19;RPS8;RPS6;RPS20;RPSA;RPL10A;RPL39;TPT1
Carbon_Tetrachloride-400_mg/kg_in_Corn_Oil-Rat-Liver-1d-up	11/300	0,004669918	0,037302509	0	0	2,520045819	13,5241126	EEF1B2;RPS17;RPL32;RPS19;RPS8;RPS6;RPS20;RPSA;RPS3A;RPL10A;SEPP1
Chloroform-600_mg/kg_in_Corn_Oil-Rat-Liver-1d-up	11/300	0,004669918	0,037264715	0	0	2,520045819	13,5241126	EEF1B2;RPS19;ARL2BP;RPS8;RPS6;RPS20;RPSA;RPS3A;RPL10A;RPL39;TPT1

Ciprofloxacin-72_mg/kg_in_Corn_Oil-Rat-Liver-0.25d-up	11/300	0,004669918	0,037226998	0	0	2,520045819	13,5241126	RPS17;RPL32;RPS19;RPS8;RPS5;RPL35A;RPSA;RPS3A;RPL39;TOMM20;TPT1
Crotamiton-750_mg/kg_in_Corn_Oil-Rat-Liver-1d-up	11/300	0,004669918	0,037189357	0	0	2,520045819	13,5241126	RPS17;RPL32;RPS8;RPS5;RPS6;RPL13A;RPL35A;RPS20;RPSA;RPS3A;RPL10A
Daunorubicin-1.5_uM_in_DMSO-Rat-Primary_rat_hepatocytes-0.67d-up	11/300	0,004669918	0,037151792	0	0	2,520045819	13,5241126	EEF1B2;RPS17;CXCL11;RPL32;CLP1;RPS8;RPS6;ARL6IP5;RPL35A;RPS3A;GOLGA7
Harringtonine-23_mg/kg_in_Saline-Rat-Bone_marrow-5d-up	11/300	0,004669918	0,037114303	0	0	2,520045819	13,5241126	EEF1B2;RPL32;RPS19;ARL2BP;RPS8;RPS6;ARL6IP5;RPS20;RPSA;RPL10A;RPL39
Lead(IV)_Acetate-600_mg/kg_in_Saline-Rat-Liver-1d-dn	11/300	0,004669918	0,037076889	0	0	2,520045819	13,5241126	EEF1B2;RPL32;RPS8;RPS5;RPS6;RPL35A;RPSA;RPS3A;RPL10A;SEPP1;TPT1
Lovastatin-1500_mg/kg_in_Corn_Oil-Rat-Skeletal_muscle-5d-up	11/300	0,004669918	0,037039551	0	0	2,520045819	13,5241126	RPS17;RPL32;RPS19;RPS8;RPS5;RPS6;RPS20;RPS3A;RPL10A;RPL39;TPT1
Mitotane-5000_mg/kg_in_Water-Rat-Bone_marrow-1d-dn	11/300	0,004669918	0,037002288	0	0	2,520045819	13,5241126	EEF1B2;C1QA;RPS17;RPL32;RPS8;RPS5;ARL6IP5;RPS20;RPS3A;RPL39;DUSP14
Nimodipine-1100_mg/kg_in_Corn_Oil-Rat-Heart-3d-up	11/300	0,004669918	0,036965099	0	0	2,520045819	13,5241126	C1QA;RPS17;RPL32;RPS19;RPS6;RPS20;RPS3A;RPL10A;SEPP1;RPL39;TPT1
Penicillamine-852_mg/kg_in_Water-Rat-Liver-1d-up	11/300	0,004669918	0,036927986	0	0	2,520045819	13,5241126	EEF1B2;C1QA;RPL32;RPS19;RPS8;RPS6;RPL35A;RPS20;RPS3A;RPL39;TPT1
Roflumilast-17_mg/kg_in_CMC-Rat-Liver-3d-up	11/300	0,004669918	0,036890947	0	0	2,520045819	13,5241126	EEF1B2;RPS17;RPS8;RPS5;RPS6;RPS20;RPSA;RPS3A;RPL10A;RPL39;TPT1
Carboplatin-6_mg/kg_in_Saline-Rat-Kidney-3d-dn	11/301	0,004786186	0,037771541	0	0	2,511673574	13,41741424	EEF1B2;RPS17;RPS19;ARL2BP;CLP1;RPS5;FUNDC1;RPS3A;RPL10A;SEPP1;RPL39
Diethylstilbestrol-280_mg/kg_in_Corn_Oil-Rat-Bone_marrow-5d-up	11/301	0,004786186	0,037733732	0	0	2,511673574	13,41741424	EEF1B2;C1QA;RPL32;RPS5;RPS6;NKG7;RPL35A;RPS20;RPL10A;SEPP1;RPL39
Disulfiram-500_mg/kg_in_Water-Rat-Liver-1d-up	11/301	0,004786186	0,037695998	0	0	2,511673574	13,41741424	EEF1B2;RPS17;RPL32;RPS19;RPS6;RPL35A;RPS20;RPSA;RPS3A;RPL10A;RPL39
Finasteride-25_mg/kg_in_Corn_Oil-Rat-Liver-1d-dn	11/301	0,004786186	0,03765834	0	0	2,511673574	13,41741424	EEF1B2;RPS17;RPL32;RPS6;RPL35A;RPSA;RPS3A;RPL10A;SEPP1;RPL39;TPT1
Lead(IV)_Acetate-600_mg/kg_in_Saline-Rat-Bone_marrow-5d-up	11/301	0,004786186	0,037620757	0	0	2,511673574	13,41741424	RPL32;RPS8;FEM1B;RPS5;FUNDC1;RPS6;RPL35A;RPS20;RPSA;RPS3A;TPT1
Leucovorin-1500_mg/kg_in_CMC-Rat-Bone_marrow-3d-dn	11/301	0,004786186	0,037583248	0	0	2,511673574	13,41741424	EEF1B2;RPS17;RPS8;RPS5;RPS6;NKG7;RPL13A;RPL35A;RPS3A;RPL39;TPT1
Nevirapine-29_mg/kg_in_Saline-Rat-Liver-5d-dn	11/301	0,004786186	0,037545815	0	0	2,511673574	13,41741424	EEF1B2;C1QA;RPS17;RPL32;RPS19;RPS6;RPS3A;RPL10A;SEPP1;RPL39;TPT1
Paroxetine-104_mg/kg_in_Water-Rat-Brain-3d-up	11/301	0,004786186	0,037508456	0	0	2,511673574	13,41741424	EEF1B2;RPS17;RPL32;RPS19;RPS8;RPL35A;RPS20;RPSA;RPS3A;RPL10A;TPT1
Sodium_Arsenite-30_mg/kg_in_Saline-Rat-Liver-3d-up	11/301	0,004786186	0,037471171	0	0	2,511673574	13,41741424	EEF1B2;RPS17;RPL32;RPS19;RPS8;RPS5;RPL35A;RPS20;RPSA;RPS3A;RPL10A
Nimesulide-162_mg/kg_in_Corn_Oil-Rat-Liver-3d-up	10/259	0,004809218	0,037614102	0	0	2,653610901	14,16290729	EEF1B2;RPS19;RPS8;RPS5;RPS6;RPS20;RPSA;RPS3A;RPL10A;RPL39
Tranexamic_Acid-2000_mg/kg_in_CMC-Rat-Heart-1d-dn	10/259	0,004809218	0,037576786	0	0	2,653610901	14,16290729	EEF1B2;RPS17;RPL32;RPS8;RPS5;RPS6;RPL35A;RPSA;RPS3A;TOMM20
Carbon_Tetrachloride-1175_mg/kg_in_Corn_Oil-Rat-Liver-3d-up	12/345	0,004848645	0,0378473	0	0	2,390557299	12,73941387	EEF1B2;RPL32;RPS19;RPS8;RPS5;RPS6;RPL35A;RPS20;RPSA;RPL10A;RPL39;TPT1
Aldosterone-0.065_mg/kg_in_Saline-Rat-Heart-1d-dn	11/302	0,004904739	0,038247255	0	0	2,503356774	13,31173313	EEF1B2;RPS17;RPL32;RPS19;RPS6;RPS20;RPSA;RPS3A;RPL10A;SEPP1;RPL39
Betamethasone-79_mg/kg_in_Corn_Oil-Rat-Bone_marrow-3d-dn	11/302	0,004904739	0,038209424	0	0	2,503356774	13,31173313	RPS17;RPL32;RPS19;FEM1B;RPS6;NKG7;RPSA;RPS3A;RPL10A;RPL39;GOLGA7
Bithionol-59_mg/kg_in_Corn_Oil-Rat-Kidney-3d-up	11/302	0,004904739	0,038171667	0	0	2,503356774	13,31173313	RPS17;RPL32;ARL2BP;RPS8;RPS5;ARL6IP5;RPL13A;RPL35A;RPS20;RPSA;RPS3A
Busulfan-3_mg/kg_in_Corn_Oil-Rat-Bone_marrow-0.25d-up	11/302	0,004904739	0,038133985	0	0	2,503356774	13,31173313	EEF1B2;C1QA;RPS17;RPL32;RPS5;RPL13A;RPL35A;RPS20;RPS3A;RPL39;DUSP14
Metronidazole-1500_mg/kg_in_Water-Rat-Liver-3d-up	11/302	0,004904739	0,038096378	0	0	2,503356774	13,31173313	EEF1B2;RPS17;RPL32;RPS19;RPS8;RPS5;RPS6;RPL35A;RPSA;RPS3A;TPT1
NN'-Diphenyl-P-Phenylenediamine-1000_mg/kg_in_CMC-Rat-Liver-5d-up	11/302	0,004904739	0,038058845	0	0	2,503356774	13,31173313	EEF1B2;RPS17;RPL32;RPS19;RPS8;FEM1B;RPS6;RPS3A;RPL10A;SEPP1;RPL39
Naproxen-10_mg/kg_in_Corn_Oil-Rat-Liver-5d-up	11/302	0,004904739	0,038021385	0	0	2,503356774	13,31173313	EEF1B2;RPS17;RPS19;RPS8;RPS6;RPL35A;RPS20;RPS3A;SEPP1;RPL39;GOLGA7
Phenobarbital-54_mg/kg_in_Water-Rat-Liver-5d-dn	11/302	0,004904739	0,037983999	0	0	2,503356774	13,31173313	EEF1B2;RPL32;RPS8;RPS6;RPS20;RPSA;RPS3A;RPL10A;SEPP1;RPL39;TPT1
Troxipide-1200_mg/kg_in_CMC-Rat-Heart-1d-dn	11/302	0,004904739	0,037946687	0	0	2,503356774	13,31173313	RPS17;RPL32;RPS8;FEM1B;COL5A3;RPS6;ARL6IP5;RPL35A;RPSA;RPL39;TPT1
Carmustine-16_mg/kg_in_Corn_Oil-Rat-Bone_marrow-5d-up	11/303	0,00502561	0,038843677	0	0	2,49509487	13,20705709	EEF1B2;C1QA;RPS17;RPL32;RPS19;RPS8;RPS5;RPL35A;RPS20;RPSA;SEPP1
Cimetidine-2000_mg/kg_in_CMC-Rat-Heart-5d-up	11/303	0,00502561	0,038805595	0	0	2,49509487	13,20705709	C1QA;RPS17;RPL32;RPS6;RPL35A;RPS20;RPSA;RPS3A;RPL39;TOMM20;TPT1
Lead(IV)_Acetate-200_mg/kg_in_Saline-Rat-Liver-0.25d-up	11/303	0,00502561	0,038767587	0	0	2,49509487	13,20705709	RPS17;RPL32;RPS8;RPS6;RPL35A;RPS20;RPSA;RPS3A;RPL10A;SEPP1;RPL39
Mitomycin_C-0.5_mg/kg_in_Saline-Rat-Liver-3d-up	11/303	0,00502561	0,038729654	0	0	2,49509487	13,20705709	EEF1B2;RPS17;RPL32;RPS19;RPS8;RPS5;RPS6;RPL35A;RPS20;RPS3A;RPL10A
Modafinil-17.5_mg/kg_in_Saline-Rat-Liver-1d-dn	11/303	0,00502561	0,038691795	0	0	2,49509487	13,20705709	EEF1B2;RPL32;RPS8;RPS5;RPS6;RPL35A;RPS3A;RPL10A;SEPP1;RPL39;TPT1
NN-Dimethylformamide-2100_mg/kg_in_Saline-Rat-Liver-1d-dn	11/303	0,00502561	0,03865401	0	0	2,49509487	13,20705709	EEF1B2;RPS17;RPL32;RPS19;RPS8;RPS6;RPL35A;RPS20;RPS3A;SEPP1;TPT1
Ascorbic_Acid-2000_mg/kg_in_Water-Rat-Kidney-5d-dn	10/261	0,00507189	0,03897191	0	0	2,633276718	13,91434401	EEF1B2;RPL32;RPS19;CLP1;RPS8;ARL6IP5;RPL35A;RPS20;RPL39;TPT1
Neostigmine_Bromide-40_mg/kg_in_Water-Rat-Liver-1d-up	12/347	0,005072279	0,03893691	0	0	2,37677887	12,55881649	EEF1B2;RPS17;RPL32;RPS19;RPS5;RPS6;RPL35A;RPS20;RPSA;RPS3A;SEPP1;TPT1
44'-Methylenedianiline-296_uM_in_DMSO-Rat-Primary_rat_hepatocytes-0.67d-up	11/304	0,00514883	0,039486058	0	0	2,486887321	13,10337412	EEF1B2;RPS17;RPL32;RPS8;RPS5;ARL6IP5;RPS3A;RPL10A;RPL39;TPT1;PDRG1
Beta-Naphthoflavone-1500_mg/kg_in_CMC-Rat-Liver-5d-up	11/304	0,00514883	0,039447648	0	0	2,486887321	13,10337412	C1QA;RPS17;RPL32;RPS19;CLP1;RPS8;RPS5;RPSA;RPS3A;RPL10A;SEPP1
Bis(2-Ethylhexyl)Phthalate-1000_mg/kg_in_Corn_Oil-Rat-Liver-3d-dn	11/304	0,00514883	0,039409312	0	0	2,486887321	13,10337412	EEF1B2;C1QA;RPS17;RPL32;CLP1;RPS8;RPS6;RPS3A;RPL10A;SEPP1;TPT1
Cetraxate-1503_mg/kg_in_CMC-Rat-Liver-3d-up	11/304	0,00514883	0,03937105	0	0	2,486887321	13,10337412	EEF1B2;RPS17;RPL32;RPS19;RPS6;RPS20;RPSA;RPS3A;RPL10A;SEPP1;RPL39
Colchicine-1000_uM_in_DMSO-Rat-Primary_rat_hepatocytes-1d-dn	11/304	0,00514883	0,039332863	0	0	2,486887321	13,10337412	EEF1B2;CXCL11;RPL32;RPS19;RPS6;RPS20;RPSA;RPS3A;RPL10A;SEPP1;RPL39
Etoposide-100_mg/kg_in_Corn_Oil-Rat-Spleen-1d-dn	11/304	0,00514883	0,03929475	0	0	2,486887321	13,10337412	EEF1B2;RPS17;RPL32;RPS8;RPS5;RPL13A;RPL35A;RPS20;RPSA;RPL39;TPT1
Isoprenaline-4.2_mg/kg_in_Saline-Rat-Heart-5d-up	11/304	0,00514883	0,03925671	0	0	2,486887321	13,10337412	EEF1B2;C1QA;RPS17;RPS19;RPS8;RPS6;RPS20;RPSA;RPL10A;SEPP1;RPL39
Pioglitazone-300_mg/kg_in_Corn_Oil-Rat-Liver-1d-dn	11/304	0,00514883	0,039218745	0	0	2,486887321	13,10337412	C1QA;RPS17;RPS19;RPS8;FUNDC1;RPS6;RPS20;RPS3A;RPL10A;RPL39;TPT1
Promethazine-2.3_mg/kg_in_Saline-Rat-Liver-3d-up	11/304	0,00514883	0,039180852	0	0	2,486887321	13,10337412	EEF1B2;C1QA;RPS17;RPL32;RPS19;RPS6;RPL35A;RPSA;RPS3A;RPL39;TPT1
Roflumilast-17_mg/kg_in_CMC-Rat-Liver-4d-up	11/304	0,00514883	0,039143033	0	0	2,486887321	13,10337412	EEF1B2;RPS17;RPL32;RPS19;RPS8;RPS5;RPS6;RPS20;RPS3A;RPL39;TPT1
Azathioprine-160_mg/kg_in_Corn_Oil-Rat-Liver-3d-dn	12/348	0,005187143	0,039396274	0	0	2,369949046	12,4696582	EEF1B2;C1QA;RPS17;RPL32;RPS19;RPS6;RPSA;RPS3A;RPL10A;RPL39;GOLGA7;TPT1
1-Naphthyl_Isothiocyanate-30_mg/kg_in_Corn_Oil-Rat-Kidney-3d-up	11/305	0,005274429	0,040020618	0	0	2,478733592	13,00067237	EEF1B2;RPS17;RPL32;RPS19;RPS8;RPS5;RPS6;RPL13A;RPL35A;RPS20;RPS3A
Artemether-74_mg/kg_in_Corn_Oil-Rat-Liver-1d-up	11/305	0,005274429	0,039982099	0	0	2,478733592	13,00067237	C1QA;RPS17;RPL32;RPS5;RPL35A;RPSA;RPS3A;SEPP1;RPL39;GOLGA7;TPT1
Benoxaprofen-89_mg/kg_in_CMC-Rat-Liver-5d-up	11/305	0,005274429	0,039943655	0	0	2,478733592	13,00067237	EEF1B2;RPS19;RPS8;RPS5;RPS6;RPS20;RPSA;RPS3A;RPL10A;RPL39;TPT1
Catechol-195_mg/kg_in_Saline-Rat-Bone_marrow-1d-dn	11/305	0,005274429	0,039905284	0	0	2,478733592	13,00067237	RPS17;RPL32;RPS19;RPS8;RPS6;RPS20;RPSA;RPS3A;RPL10A;RPL39;TPT1
Doxorubicin-3_mg/kg_in_Saline-Rat-Spleen-3d-up	11/305	0,005274429	0,039866988	0	0	2,478733592	13,00067237	CXCL11;RPS19;RPS8;RPS5;RPL35A;RPS20;RAPGEF5;RPL10A;SEPP1;GOLGA7;TPT1
Etoposide-100_mg/kg_in_Corn_Oil-Rat-Spleen-0.25d-up	11/305	0,005274429	0,039828764	0	0	2,478733592	13,00067237	EEF1B2;C1QA;RPL32;RPS19;RPS8;RPS5;RPS6;RPL35A;RPS3A;RPL10A;SEPP1
Famciclovir-112_mg/kg_in_Saline-Rat-Bone_marrow-3d-dn	11/305	0,005274429	0,039790614	0	0	2,478733592	13,00067237	C1QA;RPL32;RPS19;NKG7;RPS20;RPSA;RPS3A;RPL10A;SEPP1;RPL39;TPT1
Foscarnet-350_mg/kg_in_Saline-Rat-Bone_marrow-3d-dn	11/305	0,005274429	0,039752537	0	0	2,478733592	13,00067237	RPS17;RPL32;RPS19;RPS8;RPS6;NKG7;RPS20;RPSA;SEPP1;RPL39;TPT1
Losartan-1000_mg/kg_in_Water-Rat-Heart-3d-up	11/305	0,005274429	0,039714533	0	0	2,478733592	13,00067237	RPS17;RPL32;RPS19;ARL2BP;RPS8;RPS5;RPS6;RPSA;RPS3A;RAPGEF5;RPL10A
Leflunomide-30_mg/kg_in_Corn_Oil-Rat-Liver-0.25d-up	12/349	0,005304072	0,039899588	0	0	2,363158361	12,38124956	EEF1B2;RPS17;RPL32;RPS19;RPS8;RPS6;RPL35A;RPS20;RPSA;RPS3A;RPL10A;SEPP1
Pyrogallol-1000_mg/kg_in_Water-Rat-Liver-3d-up	12/349	0,005304072	0,039861516	0	0	2,363158361	12,38124956	RPS17;CXCL11;RPL32;RPS19;ARL2BP;RPS8;RPS6;RPS20;RPSA;RPS3A;RPL10A;TPT1
Leflunomide-30_mg/kg_in_Corn_Oil-Rat-Kidney-1d-up	9/222	0,005368555	0,040307663	0	0	2,786291446	14,56449294	RPS17;RPL32;RPS8;RPS5;FUNDC1;RPL35A;RPS20;RPS3A;TPT1
1-Naphthyl_Isothiocyanate-60_mg/kg_in_Corn_Oil-Rat-Bone_marrow-3d-up	11/306	0,005402439	0,040523441	0	0	2,470633156	12,8989402	EEF1B2;C1QA;RPL32;RPS5;RPS6;RPL35A;RPS20;RPS3A;RPL39;SLC16A3;TPT1
Citalopram-40_mg/kg_in_Corn_Oil-Rat-Liver-3d-dn	11/306	0,005402439	0,040484884	0	0	2,470633156	12,8989402	EEF1B2;RPS17;RPL32;RPS19;RPS8;RPS6;RPS20;RPSA;RPS3A;RPL10A;TPT1
Manganese_(II)_Chloride-700_mg/kg_in_Water-Rat-Liver-5d-dn	11/306	0,005402439	0,0404464	0	0	2,470633156	12,8989402	RPS17;RPL32;RPS19;RPS8;RPS6;RPL35A;RPS20;RPSA;SEPP1;RPL39;TPT1
Rofecoxib-1550_mg/kg_in_Corn_Oil-Rat-Liver-5d-dn	11/306	0,005402439	0,04040799	0	0	2,470633156	12,8989402	C1QA;RPS17;RPL32;RPS19;RPS8;FEM1B;RPS6;RPS3A;RPL10A;RPL39;TPT1
Zomepirac-2.8_mg/kg_in_Saline-Rat-Kidney-3d-up	11/306	0,005402439	0,040369652	0	0	2,470633156	12,8989402	EEF1B2;C1QA;RPS17;RPL32;RPS5;ARL6IP5;RPL35A;RPS20;RPSA;RPS3A;RPL39
Penicillamine-100_mg/kg_in_Water-Rat-Liver-3d-dn	12/350	0,005423092	0,040485565	0	0	2,35640648	12,29358273	EEF1B2;C1QA;RPS17;RPL32;RPS19;RPS6;RPL35A;RPS20;RPSA;RPL10A;RPL39;TPT1
Prochlorperazine-85_mg/kg_in_CMC-Rat-Heart-3d-up	10/264	0,005486869	0,040922901	0	0	2,603353119	13,55148761	RPS17;RPL32;RPS19;RPS8;FUNDC1;RPS6;RPS3A;RAPGEF5;SEPP1;TPT1
Naproxen-10_mg/kg_in_Corn_Oil-Rat-Kidney-1d-dn	9/223	0,005525116	0,041169173	0	0	2,773796866	14,41944704	EEF1B2;RPS17;RPL32;ARL2BP;RPS8;RPS6;RPL35A;RPS3A;TPT1
Cholecalciferol-8_mg/kg_in_CMC-Rat-Kidney-5d-up	11/307	0,005532893	0,041188155	0	0	2,462585491	12,79816612	EEF1B2;C1QA;RPS17;CXCL11;RPS19;CLP1;RPS8;RPS6;RPS20;RPSA;TPT1
Cytarabine-23_mg/kg_in_Saline-Rat-Liver-5d-dn	11/307	0,005532893	0,041149261	0	0	2,462585491	12,79816612	EEF1B2;C1QA;RPS17;RPL32;RPS19;RPS8;RPS6;RPS20;RPS3A;SEPP1;GOLGA7
Cytarabine-487_mg/kg_in_Saline-Rat-Bone_marrow-5d-up	11/307	0,005532893	0,041110441	0	0	2,462585491	12,79816612	RPS17;RPL32;RPS19;ARL2BP;RPS8;RPS6;RPS20;RPSA;RPS3A;RPL10A;TPT1
Gentian_Violet-18_mg/kg_in_Corn_Oil-Rat-Liver-5d-dn	11/307	0,005532893	0,041071694	0	0	2,462585491	12,79816612	RPS17;RPL32;RPS19;RPS8;RPS6;ARL6IP5;RPL35A;RPS3A;RPL10A;RPL39;TPT1
Melphalan-12_mg/kg_in_CMC-Rat-Spleen-5d-up	11/307	0,005532893	0,041033021	0	0	2,462585491	12,79816612	EEF1B2;RPL32;RPS19;RPS5;FUNDC1;RPS6;RPS20;RPSA;RPL10A;SEPP1;RPL39
Tobramycin-40_mg/kg_in_Saline-Rat-Kidney-14d-up	11/307	0,005532893	0,040994419	0	0	2,462585491	12,79816612	EEF1B2;RPS17;CXCL11;RPL32;RPS19;RPS8;RPS6;RPS3A;RPL10A;RPL39;TPT1
Troglitazone-100_mg/kg_in_Corn_Oil-Rat-Liver-3d-dn	13/397	0,005659246	0,041891184	0	0	2,250556147	11,64542324	RPL32;RPS5;RPS6;RPL35A;RPSA;RPS3A;RPL10A;SEPP1;EEF1B2;RPS17;RPS19;RPL39;TPT1
Atorvastatin-300_mg/kg_in_Corn_Oil-Rat-Kidney-5d-up	11/308	0,005665822	0,041900485	0	0	2,454590083	12,69833882	RPS17;RPL32;RPS19;ARL2BP;RPS8;RPS5;RPS6;RPL35A;RPS20;RPS3A;TPT1
Bezafibrate-617_mg/kg_in_Corn_Oil-Rat-Liver-3d-up	11/308	0,005665822	0,041861179	0	0	2,454590083	12,69833882	EEF1B2;RPL32;RPS19;RPS8;RPS5;RPS6;RPL35A;RPS20;RPSA;RPL10A;TPT1
Ethylene_Glycol-352_mg/kg_in_Water-Rat-Bone_marrow-3d-dn	11/308	0,005665822	0,041821946	0	0	2,454590083	12,69833882	RPS19;ARL2BP;RPS8;FEM1B;FUNDC1;RPS6;RPS20;RPSA;RPS3A;RPL39;TPT1
Fluconazole-10_mg/kg_in_Corn_Oil-Rat-Liver-0.25d-up	11/308	0,005665822	0,041782787	0	0	2,454590083	12,69833882	EEF1B2;RPS17;RPL32;RPS19;RPS8;RPS6;RPS20;RPS3A;RPL10A;SEPP1;TPT1
Phenelzine-27_mg/kg_in_Water-Rat-Brain-5d-up	11/308	0,005665822	0,041743701	0	0	2,454590083	12,69833882	RPS19;ARL2BP;FUNDC1;RPS6;ARL6IP5;RPSA;RPL10A;SEPP1;RPL39;GOLGA7;TPT1
Sulfanilamide-636_mg/kg_in_Corn_Oil-Rat-Kidney-1d-up	11/308	0,005665822	0,041704688	0	0	2,454590083	12,69833882	EEF1B2;RPS17;RPL32;RPS19;ARL2BP;RPS8;RPS6;RPL35A;RPS20;RPS3A;RPL39
Tacrolimus-134_mg/kg_in_CMC-Rat-Kidney-5d-up	11/308	0,005665822	0,041665748	0	0	2,454590083	12,69833882	RPL32;RPS19;ARL2BP;RPS8;RPS5;RPL35A;RPS20;RPSA;RAPGEF5;TOMM20;TPT1
Sulindac-750_uM_in_DMSO-Rat-Primary_rat_hepatocytes-1d-dn	12/352	0,00566751	0,041639283	0	0	2,343017807	12,12044367	EEF1B2;CXCL11;RPS19;RPS8;RPS5;FUNDC1;RPS6;ARL6IP5;RPS3A;SEPP1;GOLGA7;TPT1
Lead(IV)_Acetate-600_mg/kg_in_Saline-Rat-Spleen-1d-dn	10/266	0,005777965	0,04241123	0	0	2,583779035	13,31603182	EEF1B2;ARL2BP;RPS8;RPS5;FUNDC1;RPL13A;RPL35A;RPSA;RPS3A;TPT1
Venlafaxine-320_mg/kg_in_Water-Rat-Kidney-3d-dn	10/266	0,005777965	0,042371741	0	0	2,583779035	13,31603182	RPS17;RPL32;RPS19;RPS6;ARL6IP5;RPL35A;RPS20;RPSA;RPL10A;RPL39
Podophyllotoxin-4_mg/kg_in_Corn_Oil-Rat-Bone_marrow-5d-up	12/353	0,005792961	0,042442196	0	0	2,336380363	12,03495625	EEF1B2;C1QA;RPS17;RPL32;RPS19;FEM1B;RPS6;NKG7;RPS20;RPL10A;SEPP1;GOLGA7
Betamethasone-79_mg/kg_in_Corn_Oil-Rat-Liver-1d-dn	11/309	0,005801259	0,042463487	0	0	2,446646426	12,59944715	EEF1B2;C1QA;RPS17;RPL32;RPS19;RPS8;RPS6;RPSA;RPS3A;RPL10A;SEPP1
Bisacodyl-1750_mg/kg_in_CMC-Rat-Intestine-5d-dn	11/309	0,005801259	0,042424059	0	0	2,446646426	12,59944715	EEF1B2;RPS17;RPS19;RPS8;RPS6;ARL6IP5;RPL13A;RPL35A;RPS3A;SEPP1;RPL39

Ciprofloxacin-450_mg/kg_in_Corn_Oil-Rat-Liver-0.25d-up	11/309	0,005801259	0,042384705	0	0	2,446646426	12,59944715	EEF1B2;C1QA;RPL32;RPS19;RPS8;FEM1B;RPS6;RPS20;RPS3A;SEPP1;RPL39
Citalopram-90_mg/kg_in_Corn_Oil-Rat-Liver-0.25d-up	11/309	0,005801259	0,042345424	0	0	2,446646426	12,59944715	EEF1B2;RPS17;RPL32;RPS8;RPS5;RPS6;RPS20;RPSA;RPS3A;RPL10A;SEPP1
Doxazosin-5_mg/kg_in_Water-Rat-Heart-0.25d-up	11/309	0,005801259	0,042306215	0	0	2,446646426	12,59944715	RPS17;RPL32;RPS19;RPS8;RPS5;RPS6;ARL6IP5;RPL35A;RPSA;RPS3A;TPT1
Erythromycin-1500_mg/kg_in_CMC-Rat-Kidney-5d-up	11/309	0,005801259	0,042267079	0	0	2,446646426	12,59944715	EEF1B2;RPS17;RPS19;CLP1;RPS8;RPS6;RPS20;RPS3A;SEPP1;RPL39;TPT1
Fludrocortisone_Acetate-125_mg/kg_in_CMC-Rat-Heart-1d-dn	11/309	0,005801259	0,042228015	0	0	2,446646426	12,59944715	RPS17;RPL32;RPS19;ARL2BP;RPS8;RPS6;RPS20;RPSA;RPS3A;RPL10A;RPL39
Fluocinolone_Acetonide-2.5_mg/kg_in_Corn_Oil-Rat-Heart-5d-dn	11/309	0,005801259	0,042189023	0	0	2,446646426	12,59944715	EEF1B2;RPS17;RPL32;RPS19;ARL2BP;RPS8;COL5A3;RPSA;RPS3A;RPL10A;RPL39
Hydralazine-280_mg/kg_in_Water-Rat-Liver-0.25d-up	11/309	0,005801259	0,042150103	0	0	2,446646426	12,59944715	RPL32;RPS19;ARL2BP;CLP1;RPS6;RPS20;RPSA;RPS3A;RPL10A;SEPP1;TPT1
Ibuprofen-263_mg/kg_in_Corn_Oil-Rat-Liver-5d-up	11/309	0,005801259	0,042111255	0	0	2,446646426	12,59944715	EEF1B2;C1QA;RPS19;RPS5;RPS6;RPS20;RPSA;RPS3A;RPL10A;RPL39;TPT1
Imatinib-150_mg/kg_in_Water-Rat-Liver-5d-dn	11/309	0,005801259	0,042072479	0	0	2,446646426	12,59944715	EEF1B2;RPS17;RPL32;RPS19;RPS6;RPL35A;RPS20;RPSA;RPS3A;SEPP1;RPL39
Lansoprazole-240_uM_in_DMSO-Rat-Primary_rat_hepatocytes-0.67d-dn	11/309	0,005801259	0,042033774	0	0	2,446646426	12,59944715	CXCL11;RPL32;RPS19;RPS8;ARL6IP5;RPS20;RPSA;RPS3A;RPL10A;SEPP1;TPT1
Noscapine-570_mg/kg_in_CMC-Rat-Spleen-5d-up	11/309	0,005801259	0,04199514	0	0	2,446646426	12,59944715	EEF1B2;RPL32;RPS19;RPS5;FUNDC1;RPS6;RPS20;RPSA;RPL10A;SEPP1;RPL39
Ticrynafen-570_mg/kg_in_CMC-Rat-Liver-1d-up	11/309	0,005801259	0,041956577	0	0	2,446646426	12,59944715	EEF1B2;RPL32;RPS8;RPS5;RPS6;RPS20;RPSA;RPS3A;RPL10A;RPL39;TPT1
Tolazamide-1500_mg/kg_in_CMC-Rat-Kidney-5d-up	11/309	0,005801259	0,041918084	0	0	2,446646426	12,59944715	EEF1B2;RPS17;RPS8;RPS5;ARL6IP5;RPSA;RPS3A;RAPGEF5;RPL10A;SEPP1;TPT1
Trichloroethylene-1230_mg/kg_in_CMC-Rat-Kidney-5d-up	11/309	0,005801259	0,041879663	0	0	2,446646426	12,59944715	EEF1B2;RPS17;CXCL11;RPS8;ARL6IP5;RPSA;RPS3A;RAPGEF5;RPL10A;TOMM20;TPT1
Zopiclone-414_mg/kg_in_Water-Rat-Liver-5d-up	11/309	0,005801259	0,041841311	0	0	2,446646426	12,59944715	RPS17;RPL32;RPS8;RPS5;RPS6;RPL35A;RPS20;RPSA;RPS3A;RPL10A;RPL39
Ethinylestradiol-10_mg/kg_in_Corn_Oil-Rat-Liver-3d-up	10/267	0,005927965	0,042716058	0	0	2,57410196	13,20018616	EEF1B2;RPL32;RPS19;RPS8;RPS6;RPS20;RPSA;RPL10A;RPL39;TPT1
Leflunomide-60_mg/kg_in_Corn_Oil-Rat-Spleen-3d-up	10/267	0,005927965	0,042677012	0	0	2,57410196	13,20018616	EEF1B2;RPS17;RPL32;RPS8;RPS5;RPS6;RPS20;RPSA;RPS3A;RAPGEF5
2-Nitroanisole-740_mg/kg_in_CMC-Rat-Kidney-5d-up	11/310	0,005939234	0,042719094	0	0	2,438754018	12,50148012	EEF1B2;RPS17;CLP1;ARL6IP5;RPSA;RPS3A;RAPGEF5;SEPP1;RPL39;TOMM20;TPT1
Amlodipine-19_mg/kg_in_Corn_Oil-Rat-Liver-3d-up	11/310	0,005939234	0,042680116	0	0	2,438754018	12,50148012	EEF1B2;C1QA;RPS17;RPL32;RPS5;RPL13A;RPL35A;RPS20;RPSA;RPS3A;TPT1
Ethanol-3000_mg/kg_in_Saline-Rat-Bone_marrow-3d-dn	11/310	0,005939234	0,04264121	0	0	2,438754018	12,50148012	EEF1B2;C1QA;RPS17;RPL32;RPS8;RPS6;NKG7;RPSA;RPS3A;RPL10A;TPT1
Methimazole-100_mg/kg_in_Water-Rat-Liver-1d-up	11/310	0,005939234	0,042602375	0	0	2,438754018	12,50148012	EEF1B2;RPS17;RPL32;RPS19;RPS8;RPS5;RPS20;RPSA;RPS3A;RPL10A;RPL39
Procarbazine-27_mg/kg_in_Water-Rat-Bone_marrow-1d-dn	11/310	0,005939234	0,04256361	0	0	2,438754018	12,50148012	EEF1B2;RPS17;RPL32;RPS19;RPS8;RPS6;ARL6IP5;RPS20;RPSA;RPL39;DUSP14
Sodium_Arsenite-10_mg/kg_in_Saline-Rat-Liver-1d-dn	11/310	0,005939234	0,042524916	0	0	2,438754018	12,50148012	C1QA;RPL32;RPS19;CLP1;RPS6;RPS20;RPS3A;RPL10A;SEPP1;RPL39;TPT1
Thioguanine-12_mg/kg_in_Corn_Oil-Rat-Liver-5d-up	11/310	0,005939234	0,042486292	0	0	2,438754018	12,50148012	EEF1B2;RPS17;CLP1;RPS8;RPS6;RPS20;RPSA;RPS3A;SEPP1;RPL39;TPT1
Trichloroethane-3000_mg/kg_in_Water-Rat-Liver-5d-up	11/310	0,005939234	0,042447738	0	0	2,438754018	12,50148012	EEF1B2;C1QA;RPS17;RPL32;RPS8;RPS5;RPS6;ARL6IP5;RPL35A;RPS20;RPS3A
Allyl_Alcohol-25_mg/kg_in_Saline-Rat-Bone_marrow-3d-dn	9/226	0,006016067	0,042957881	0	0	2,736976553	13,99504128	RPL32;RPS19;ARL2BP;RPS8;FUNDC1;RPS6;RPS20;RPSA;RPL10A
Camptothecin-5_mg/kg_in_Saline-Rat-Kidney-3d-dn	12/355	0,006050478	0,043164458	0	0	2,323217656	11,86610847	EEF1B2;RPS17;RPL32;RPS19;ARL2BP;RPS6;RPL35A;RPS20;RPSA;RPS3A;RPL10A;RPL39
Gatifloxacin-770_mg/kg_in_Corn_Oil-Rat-Kidney-5d-up	12/355	0,006050478	0,043125395	0	0	2,323217656	11,86610847	EEF1B2;RPS17;RPL32;RPS19;RPS8;RPS6;RPL35A;RPS20;RPSA;RPS3A;RPL10A;RPL39
Betamethasone-79_mg/kg_in_Corn_Oil-Rat-Bone_marrow-1d-up	11/311	0,006079781	0,043295079	0	0	2,430912366	12,40442691	C1QA;RPS17;RPL32;RPS19;RPS8;RPS5;RPS6;RPL13A;RPL35A;RPS3A;SEPP1
Cisplatin-0.5_mg/kg_in_Saline-Rat-Kidney-14d-up	11/311	0,006079781	0,043255969	0	0	2,430912366	12,40442691	CXCL11;RPL32;RPS19;RPS8;RPS6;RPS20;RPSA;RPS3A;RPL10A;RPL39;TPT1
Ethisterone-1500_mg/kg_in_CMC-Rat-Liver-1d-up	11/311	0,006079781	0,04321693	0	0	2,430912366	12,40442691	C1QA;RPS17;RPL32;RPS6;ARL6IP5;RPL35A;RPS20;RPSA;RPS3A;RPL10A;RPL39
Flurbiprofen-10_mg/kg_in_CMC-Rat-Kidney-3d-up	11/311	0,006079781	0,04317796	0	0	2,430912366	12,40442691	EEF1B2;C1QA;RPL32;RPS5;RPS6;RPS20;RPS3A;RPL10A;RPL39;TPT1;PDRG1
Leucovorin-1500_mg/kg_in_CMC-Rat-Bone_marrow-1d-dn	11/311	0,006079781	0,043139061	0	0	2,430912366	12,40442691	EEF1B2;C1QA;RPS17;RPL32;RPS19;ARL2BP;RPS8;RPS6;RPL35A;RPS3A;RPL39
Mitomycin_C-0.5_mg/kg_in_Saline-Rat-Bone_marrow-0.25d-dn	11/311	0,006079781	0,043100232	0	0	2,430912366	12,40442691	EEF1B2;RPS19;RPS8;RPS6;NKG7;RPS20;RPSA;RPS3A;RPL10A;RPL39;TPT1
Nadolol-1500_mg/kg_in_CMC-Rat-Heart-3d-up	11/311	0,006079781	0,043061473	0	0	2,430912366	12,40442691	EEF1B2;RPS17;RPL32;RPS8;RPS5;RPS6;RPSA;RPS3A;RPL10A;RPL39;PDRG1
Neomycin-877_mg/kg_in_Corn_Oil-Rat-Kidney-3d-up	11/311	0,006079781	0,043022783	0	0	2,430912366	12,40442691	RPS17;RPL32;ARL2BP;RPS8;RPS6;ARL6IP5;RPL35A;RPS20;RPS3A;RPL39;GOLGA7
Stavudine-1400_mg/kg_in_Water-Rat-Heart-3d-up	11/311	0,006079781	0,042984163	0	0	2,430912366	12,40442691	RPS17;RPS8;RPS6;ARL6IP5;RPSA;RAPGEF5;RPL10A;SEPP1;RPL39;GOLGA7;TPT1
Streptomycin-120_mg/kg_in_Saline-Rat-Kidney-1d-dn	11/311	0,006079781	0,042945612	0	0	2,430912366	12,40442691	EEF1B2;RPS17;RPL32;RPS8;RPS6;RPL35A;RPS20;RPS3A;SEPP1;RPL39;TPT1
Triacetin-1200_mg/kg_in_CMC-Rat-Liver-3d-up	11/311	0,006079781	0,042907131	0	0	2,430912366	12,40442691	EEF1B2;C1QA;RPS17;RPL32;RPS19;RPS8;RPS6;RPS20;RPSA;RPS3A;RPL39
Finasteride-25_mg/kg_in_Corn_Oil-Rat-Liver-5d-dn	10/268	0,006080987	0,04287722	0	0	2,564497102	13,08557277	EEF1B2;C1QA;RPS17;RPL32;RPS8;RPS6;ARL6IP5;RPL35A;RPS3A;SEPP1
Indomethacin-9.6_mg/kg_in_Water-Rat-Kidney-3d-up	12/356	0,006182597	0,04355468	0	0	2,316691764	11,7827335	RPS17;RPL32;RPS19;RPS8;RPS5;RPS6;RPL35A;RPS20;RPS3A;RPL10A;RPL39;TPT1
Valproic_Acid-850_mg/kg_in_Water-Rat-Kidney-3d-up	12/356	0,006182597	0,043515757	0	0	2,316691764	11,7827335	EEF1B2;RPS17;RPL32;RPS5;RPL13A;RPL35A;RPS20;RPSA;SEPP1;RPL39;TOMM20;TPT1
Aspirin-35_mg/kg_in_Corn_Oil-Rat-Liver-3d-up	11/312	0,006222933	0,043760553	0	0	2,42312098	12,30827685	RPS17;RPL32;RPS19;RPS8;RPS5;RPS6;RPS20;RPSA;RPS3A;SEPP1;GOLGA7
Bisphenol_A-610_mg/kg_in_Corn_Oil-Rat-Bone_marrow-3d-up	11/312	0,006222933	0,043721516	0	0	2,42312098	12,30827685	EEF1B2;C1QA;RPL32;ARL2BP;RPS8;RPS5;FUNDC1;RPS6;RPL35A;RPS3A;TPT1
Camptothecin-5_mg/kg_in_Saline-Rat-Kidney-5d-dn	11/312	0,006222933	0,043682548	0	0	2,42312098	12,30827685	EEF1B2;RPS19;ARL2BP;RPS8;RPS6;ARL6IP5;RPS20;RPS3A;RAPGEF5;RPL10A;TPT1
Captopril-1750_mg/kg_in_Water-Rat-Kidney-3d-up	11/312	0,006222933	0,04364365	0	0	2,42312098	12,30827685	RPS17;RPL32;ARL2BP;RPS5;RPL35A;RPS20;RPSA;RPS3A;RPL10A;RPL39;TPT1
Flubendazole-1200_mg/kg_in_Corn_Oil-Rat-Liver-5d-up	11/312	0,006222933	0,043604821	0	0	2,42312098	12,30827685	C1QA;RPS17;RPL32;RPS19;RPS8;RPS20;RPSA;RPS3A;RPL10A;RPL39;TPT1
Hydrocortisone-56_mg/kg_in_Corn_Oil-Rat-Bone_marrow-1d-dn	11/312	0,006222933	0,043566061	0	0	2,42312098	12,30827685	RPL32;RPS8;FEM1B;RPS6;RPL35A;RPS20;RPSA;RPS3A;RPL10A;RPL39;TPT1
Hydrocortisone-56_mg/kg_in_Corn_Oil-Rat-Kidney-3d-up	11/312	0,006222933	0,04352737	0	0	2,42312098	12,30827685	EEF1B2;RPS17;CXCL11;RPL32;RPS19;RPS6;ARL6IP5;RPL35A;RPS20;RPSA;SEPP1
Nimesulide-30_mg/kg_in_Corn_Oil-Rat-Kidney-1d-dn	11/312	0,006222933	0,043488748	0	0	2,42312098	12,30827685	EEF1B2;RPS17;RPL32;RPS19;RPS6;RPL35A;RPS20;RPS3A;RPL10A;SEPP1;RPL39
Famciclovir-112_mg/kg_in_Saline-Rat-Spleen-1d-dn	10/269	0,006237072	0,043548916	0	0	2,554963656	12,97217508	EEF1B2;C1QA;RPS17;ARL2BP;RPS8;RPS5;FUNDC1;RPL13A;RPL35A;TPT1
Pravastatin-1200_mg/kg_in_Corn_Oil-Rat-Liver-1d-dn	12/357	0,006316992	0,044067874	0	0	2,310202431	11,70004823	EEF1B2;RPS17;RPL32;CLP1;RPS8;FEM1B;RPS20;RPSA;RPS3A;RPL10A;RPL39;TPT1
Acetazolamide-250_mg/kg_in_Corn_Oil-Rat-Liver-0.25d-up	11/313	0,006368721	0,044389423	0	0	2,415379379	12,21301943	EEF1B2;RPL32;RPS19;RPS8;FEM1B;RPS6;RPS20;RPSA;RPS3A;RPL10A;SEPP1
Acetazolamide-250_mg/kg_in_Corn_Oil-Rat-Liver-3d-up	11/313	0,006368721	0,044350175	0	0	2,415379379	12,21301943	EEF1B2;RPS17;RPL32;RPS19;RPS8;RPS5;RPS20;RPSA;RPS3A;RPL10A;RPL39
Clofibric_Acid-448_mg/kg_in_Corn_Oil-Rat-Liver-5d-up	11/313	0,006368721	0,044310997	0	0	2,415379379	12,21301943	RPS17;RPL32;RPS5;RPS6;RPL35A;RPS20;RPSA;RPS3A;RPL10A;RPL39;TPT1
Doxorubicin-4_mg/kg_in_Saline-Rat-Kidney-14d-up	11/313	0,006368721	0,044271887	0	0	2,415379379	12,21301943	EEF1B2;CXCL11;RPL32;RPS19;RPS8;RPS6;RPS20;RPSA;RPL10A;RPL39;GOLGA7
Galantamine-110_mg/kg_in_CMC-Rat-Heart-3d-dn	11/313	0,006368721	0,044232847	0	0	2,415379379	12,21301943	EEF1B2;RPS19;ARL2BP;RPS8;COL5A3;FUNDC1;ARL6IP5;RPS20;RPSA;RPL39;TPT1
Ifosfamide-17_mg/kg_in_Saline-Rat-Spleen-0.25d-up	11/313	0,006368721	0,044193875	0	0	2,415379379	12,21301943	EEF1B2;C1QA;RPS17;RPL32;RPS8;RPS5;RPL13A;RPL35A;RPSA;RPL10A;SEPP1
Clotrimazole-52_mg/kg_in_Corn_Oil-Rat-Liver-5d-up	10/270	0,00639626	0,044345899	0	0	2,545500827	12,85997679	RPS17;RPL32;RPS19;RPS8;RPS5;RPL35A;RPS20;RPSA;RPS3A;RPL39
Diclofenac-3.5_mg/kg_in_Corn_Oil-Rat-Liver-3d-dn	10/270	0,00639626	0,044306897	0	0	2,545500827	12,85997679	EEF1B2;C1QA;RPS17;RPL32;RPS8;RPS6;RPS20;RPSA;RPS3A;TPT1
Esmolol-27_mg/kg_in_Saline-Rat-Heart-3d-dn	10/270	0,00639626	0,044267963	0	0	2,545500827	12,85997679	EEF1B2;RPS17;RPL32;RPS19;RPS8;RPS6;ARL6IP5;RPS3A;RPL10A;RPL39
Etoposide-100_mg/kg_in_Corn_Oil-Rat-Liver-1d-dn	10/270	0,00639626	0,044229097	0	0	2,545500827	12,85997679	EEF1B2;RPS17;RPL32;RPS8;RPS6;RPL35A;RPS3A;RPL10A;RPL39;TPT1
Etoposide-188_mg/kg_in_Corn_Oil-Rat-Heart-3d-dn	10/270	0,00639626	0,044190299	0	0	2,545500827	12,85997679	EEF1B2;RPS17;RPL32;RPS19;RPS8;RPS6;RPS20;RPS3A;RPL39;TPT1
Tacrolimus-50_uM_in_DMSO-Rat-Primary_rat_hepatocytes-1d-up	10/270	0,00639626	0,04415157	0	0	2,545500827	12,85997679	EEF1B2;RPS17;ARL2BP;RPS8;RPS5;RPS20;RPSA;RPS3A;RPL10A;PDRG1
Benzocaine-1100_mg/kg_in_Corn_Oil-Rat-Liver-3d-up	11/314	0,006517179	0,04494685	0	0	2,407687088	12,11864428	RPL32;RPS19;RPS8;RPS5;RPS6;RPL35A;RPS20;RPSA;RPL10A;SEPP1;RPL39
Carmustine-4_mg/kg_in_Corn_Oil-Rat-Liver-0.25d-dn	11/314	0,006517179	0,044907526	0	0	2,407687088	12,11864428	EEF1B2;RPS17;RPL32;RPS19;CLP1;RPS8;RPS20;RPSA;RPS3A;RPL39;TPT1
Cetylpyridinium_Bromide-16.5_mg/kg_in_Water-Rat-Liver-1d-up	11/314	0,006517179	0,044868272	0	0	2,407687088	12,11864428	EEF1B2;RPS17;RPL32;RPS19;RPS6;RPL35A;RPSA;RPS3A;RPL10A;RPL39;TPT1
Isotretinoin-125_mg/kg_in_Corn_Oil-Rat-Liver-5d-up	11/314	0,006517179	0,044829085	0	0	2,407687088	12,11864428	EEF1B2;C1QA;RPS17;RPL32;RPS19;CLP1;RPS8;RPL35A;RPS20;RPS3A;TPT1
NN-Dimethylformamide-65000_uM_in_DMSO-Rat-Primary_rat_hepatocytes-1d-up	11/314	0,006517179	0,044789967	0	0	2,407687088	12,11864428	RPS17;CXCL11;RPL32;RPS8;RPS5;RPS6;RPL35A;RPSA;RPL10A;SEPP1;PDRG1
Valacyclovir-2000_mg/kg_in_CMC-Rat-Kidney-1d-up	11/314	0,006517179	0,044750918	0	0	2,407687088	12,11864428	C1QA;RPS17;CXCL11;RPL32;RPS19;RPS8;RPS6;ARL6IP5;RPS20;RPL10A;RPL39
Zomepirac-2.8_mg/kg_in_Saline-Rat-Liver-5d-up	11/314	0,006517179	0,044711936	0	0	2,407687088	12,11864428	EEF1B2;C1QA;RPS17;RPL32;ARL6IP5;RPL35A;RPSA;RPS3A;SEPP1;RPL39;TPT1
Carboplatin-6_mg/kg_in_Saline-Rat-Bone_marrow-0.25d-up	10/271	0,006558591	0,04495689	0	0	2,536107835	12,74896189	RPL32;RPS19;RPS8;RPS5;FUNDC1;RPL13A;RPL35A;RPS20;RPSA;TPT1
Fenofibrate-215_mg/kg_in_Corn_Oil-Rat-Kidney-1d-up	12/359	0,006592719	0,045151526	0	0	2,297332223	11,53671864	RPS17;RPS19;RPS8;RPS5;RPS6;RPS20;RPSA;RPS3A;RAPGEF5;RPL39;GOLGA7;TPT1
Bisphenol_A-610_mg/kg_in_Corn_Oil-Rat-Bone_marrow-5d-dn	11/315	0,00666834	0,04562975	0	0	2,400043637	12,02514119	EEF1B2;RPS17;RPL32;RPS8;RPS5;ARL6IP5;RPL13A;RPL35A;RPSA;RPS3A;TPT1
Cobalt(II)_Acetylacetonate-100_mg/kg_in_Corn_Oil-Rat-Heart-5d-dn	11/315	0,00666834	0,045590141	0	0	2,400043637	12,02514119	EEF1B2;RPS17;RPL32;RPS19;RPS8;RPS6;RPS20;RPSA;RPS3A;RPL10A;RPL39
Lovastatin-1500_mg/kg_in_Corn_Oil-Rat-Liver-3d-up	11/315	0,00666834	0,0455506	0	0	2,400043637	12,02514119	EEF1B2;RPL32;RPS8;RPS5;RPS6;RPL35A;RPS20;RPSA;RPL10A;RPL39;TPT1
Netilmicin-40_mg/kg_in_Saline-Rat-Kidney-5d-dn	9/230	0,00672222	0,045878859	0	0	2,689376961	13,4531695	RPS17;RPS19;ARL2BP;RPS8;FUNDC1;RPSA;RPS3A;RPL39;SEPP1
2378-Tetrachlorodibenzo-P-Dioxin-0.02_mg/kg_in_CMC-Rat-Liver-1d-dn	10/272	0,006724108	0,045852012	0	0	2,526783909	12,63911464	EEF1B2;RPS17;RPL32;RPS19;CLP1;RPS8;RPS6;RPS20;RPL39;TPT1
Nevirapine-29_mg/kg_in_Saline-Rat-Liver-1d-dn	10/272	0,006724108	0,045812348	0	0	2,526783909	12,63911464	EEF1B2;RPL32;RPS19;RPS8;RPS5;RPS6;RPSA;RPL10A;SEPP1;TPT1
Nizatidine-1653_mg/kg_in_Corn_Oil-Rat-Heart-5d-dn	10/272	0,006724108	0,045772752	0	0	2,526783909	12,63911464	EEF1B2;RPS17;RPL32;ARL2BP;RPS8;RPS6;RPL35A;RPSA;RPS3A;RPL39
Aflatoxin_B1-0.3_mg/kg_in_CMC-Rat-Liver-1d-up	11/316	0,006822235	0,046400627	0	0	2,392448562	11,9325001	EEF1B2;RPS17;RPL32;RPS19;RPS8;RPS20;RPSA;RPS3A;SEPP1;RPL39;TPT1
Diethylstilbestrol-280_mg/kg_in_Corn_Oil-Rat-Bone_marrow-3d-up	11/316	0,006822235	0,046360592	0	0	2,392448562	11,9325001	EEF1B2;C1QA;RPS17;RPL32;RPS5;NKG7;RPL35A;RPS3A;SEPP1;RPL39;TPT1
Ifosfamide-17_mg/kg_in_Saline-Rat-Liver-0.25d-dn	11/316	0,006822235	0,046320626	0	0	2,392448562	11,9325001	EEF1B2;RPS17;RPL32;RPS19;RPS6;RPL35A;RPS20;RPSA;RPL10A;RPL39;TPT1
Irinotecan-5_mg/kg_in_Saline-Rat-Bone_marrow-3d-dn	11/316	0,006822235	0,046280729	0	0	2,392448562	11,9325001	CLP1;RPS8;FEM1B;FUNDC1;RPS6;NKG7;RPS20;RPSA;RPS3A;GOLGA7;TPT1
Nimesulide-162_mg/kg_in_Corn_Oil-Rat-Kidney-1d-up	11/316	0,006822235	0,0462409	0	0	2,392448562	11,9325001	RPS17;RPS19;RPS8;RPS6;RPS20;RPSA;RPS3A;RAPGEF5;RPL10A;GOLGA7;TPT1
Paroxetine-104_mg/kg_in_Water-Rat-Brain-5d-up	11/316	0,006822235	0,04620114	0	0	2,392448562	11,9325001	EEF1B2;RPS19;CLP1;RPS6;RPS20;RPSA;RPS3A;RAPGEF5;RPL10A;RPL39;TPT1
Quetiapine-500_mg/kg_in_CMC-Rat-Liver-1d-dn	11/316	0,006822235	0,046161449	0	0	2,392448562	11,9325001	EEF1B2;C1QA;RPS17;RPL32;RPS19;RPS8;RPS6;RPL35A;RPS20;RPL10A;SEPP1
1-Naphthyl_Isothiocyanate-60_mg/kg_in_Corn_Oil-Rat-Liver-1d-up	10/273	0,00689285	0,046599218	0	0	2,517528291	12,53041952	EEF1B2;RPS17;RPS19;ARL2BP;RPS6;RPS20;RPSA;RPS3A;RPL10A;RPL39
Busulfan-9_mg/kg_in_Corn_Oil-Rat-Spleen-5d-up	10/273	0,00689285	0,046559253	0	0	2,517528291	12,53041952	EEF1B2;CXCL11;RPS19;CLP1;RPS8;FUNDC1;RPS20;RPSA;GOLGA7;SEPP1
Mebendazole-714_mg/kg_in_Corn_Oil-Rat-Liver-3d-up	10/273	0,00689285	0,046519357	0	0	2,517528291	12,53041952	EEF1B2;RPL32;RPS19;RPS8;RPS5;RPL35A;RPS20;RPSA;RPL39;SEPP1

Dichlorvos-17_mg/kg_in_Water-Rat-Heart-5d-dn	11/317	0,0069789	0,047059774	0	0	2,384901406	11,84071108	RPS17;RPL32;RPS19;RPS6;RPS20;RPSA;RPS3A;RPL10A;RPL39;TPT1;PDRG1
Ethanol-3000_mg/kg_in_Saline-Rat-Bone_marrow-0.25d-dn	11/317	0,0069789	0,047019517	0	0	2,384901406	11,84071108	EEF1B2;C1QA;RPS17;RPL32;RPS8;RPL35A;RPSA;RPS3A;RPL10A;RPL39;TPT1
Lidocaine-71_mg/kg_in_Water-Rat-Heart-1d-up	11/317	0,0069789	0,04697933	0	0	2,384901406	11,84071108	C1QA;RPS17;RPL32;RPS19;ARL2BP;RPS8;RPS6;RPSA;RPS3A;RAPGEF5;TPT1
Mitomycin_C-1.7_mg/kg_in_Saline-Rat-Bone_marrow-5d-dn	11/317	0,0069789	0,046939211	0	0	2,384901406	11,84071108	EEF1B2;RPS17;RPL32;FEM1B;RPS5;RPL35A;RPSA;RPL10A;RPL39;TPT1;DUSP14
Nitrofurantoin-76_mg/kg_in_CMC-Rat-Liver-3d-up	11/317	0,0069789	0,04689916	0	0	2,384901406	11,84071108	RPS17;RPS19;RPS8;RPS6;RPS20;RPSA;RPS3A;RAPGEF5;RPL10A;SEPP1;RPL39
Allyl_Alcohol-25_mg/kg_in_Saline-Rat-Liver-0.25d-up	10/274	0,00706486	0,047436347	0	0	2,508340231	12,42286132	EEF1B2;RPS17;RPL32;RPS8;RPS5;RPS6;RPSA;RPS3A;RPL39;SEPP1
Atropine-2.3_mg/kg_in_Water-Rat-Liver-1d-dn	10/274	0,00706486	0,047395941	0	0	2,508340231	12,42286132	EEF1B2;RPS17;RPL32;RPS19;RPS8;RPS6;RPS20;RPSA;RPS3A;SEPP1
Bromhexine-1200_mg/kg_in_CMC-Rat-Liver-1d-up	11/318	0,007138366	0,047848318	0	0	2,377401716	11,74976436	C1QA;RPS17;RPL32;RPS19;RPS8;RPS5;RPS6;RPSA;RPS3A;TPT1;PDRG1
Chlorambucil-0.6_mg/kg_in_Corn_Oil-Rat-Bone_marrow-3d-up	11/318	0,007138366	0,04780763	0	0	2,377401716	11,74976436	EEF1B2;C1QA;RPL32;RPS8;RPS5;RPL35A;RPS20;RPL10A;SEPP1;RPL39;TPT1
Cisplatin-1.17_mg/kg_in_Saline-Rat-Kidney-1d-up	11/318	0,007138366	0,047767012	0	0	2,377401716	11,74976436	EEF1B2;RPS17;RPS19;RPS8;RPS6;RPL13A;RPL35A;RPS20;RPSA;RPS3A;RPL39
Deferiprone-500_mg/kg_in_Water-Rat-Liver-5d-up	11/318	0,007138366	0,047726463	0	0	2,377401716	11,74976436	EEF1B2;RPS17;RPL32;RPS19;RPS5;RPS6;RPL35A;RPSA;RPS3A;RAPGEF5;RPL10A
Gadopentetate_Dimeglumine-125_mg/kg_in_Saline-Rat-Kidney-1d-up	11/318	0,007138366	0,047685982	0	0	2,377401716	11,74976436	EEF1B2;RPS17;RPL32;RPS19;RPS8;RPS6;ARL6IP5;RPL35A;RPS20;RPSA;RPS3A
Isotretinoin-500_uM_in_DMSO-Rat-Primary_rat_hepatocytes-0.67d-up	11/318	0,007138366	0,04764557	0	0	2,377401716	11,74976436	EEF1B2;RPS19;RPS8;RPS6;ARL6IP5;RPSA;RPL10A;SEPP1;RPL39;GOLGA7;TPT1
NN-Dimethylformamide-1400_mg/kg_in_Saline-Rat-Spleen-1d-dn	11/318	0,007138366	0,047605227	0	0	2,377401716	11,74976436	EEF1B2;RPS17;RPL32;RPS8;RPS5;ARL6IP5;RPL35A;RPS20;RPSA;RPS3A;TPT1
Nateglinide-625_mg/kg_in_CMC-Rat-Kidney-1d-dn	11/318	0,007138366	0,047564952	0	0	2,377401716	11,74976436	RPS19;ARL2BP;RPS8;RPS6;ARL6IP5;RPS20;RPSA;RPL10A;SEPP1;RPL39;PDRG1
Perhexiline-12_uM_in_DMSO-Rat-Primary_rat_hepatocytes-1d-dn	11/318	0,007138366	0,047524745	0	0	2,377401716	11,74976436	RPS17;CXCL11;RPL32;RPS19;ARL2BP;RPS8;RPS5;ARL6IP5;RPL35A;SEPP1;TPT1
Sparfloxacin-29_mg/kg_in_Corn_Oil-Rat-Liver-1d-up	11/318	0,007138366	0,047484606	0	0	2,377401716	11,74976436	EEF1B2;RPL32;RPS19;RPS8;FEM1B;RPS6;RPS20;RPS3A;SEPP1;GOLGA7;TPT1
Valdecoxib-404_mg/kg_in_CMC-Rat-Kidney-5d-up	11/318	0,007138366	0,047444534	0	0	2,377401716	11,74976436	C1QA;RPL32;RPS5;RPS6;ARL6IP5;RPL13A;RPL35A;RPS20;RPSA;RPL39;TOMM20
Acrolein-20_mg/kg_in_Water-Rat-Kidney-1d-up	12/363	0,007172679	0,047632394	0	0	2,272017267	11,21803087	EEF1B2;RPS19;ARL2BP;CLP1;RPS8;RPS6;RPS20;RPS3A;SEPP1;RPL39;TOMM20;TPT1
Ramipril-1500_mg/kg_in_CMC-Rat-Kidney-3d-dn	13/409	0,007195092	0,047740983	0	0	2,18452516	10,77922508	RPL32;RPS8;RPS6;RPS3A;RPL10A;SEPP1;EEF1B2;RPS17;RPS19;CLP1;RPS20;RPL39;TPT1
Venlafaxine-320_mg/kg_in_Water-Rat-Kidney-1d-dn	8/192	0,007229517	0,04792902	0	0	2,863688431	14,11678997	RPS17;RPL32;RPS19;RPS6;RPSA;RPS20;RPL10A;RPL39
Beta-Estradiol-150_mg/kg_in_Corn_Oil-Rat-Kidney-5d-up	10/275	0,007240177	0,047959325	0	0	2,499218994	12,31642504	EEF1B2;RPL32;RPS19;ARL2BP;RPS8;RPS6;RPS20;RPSA;RPS3A;RPL10A
Carbon_Tetrachloride-400_mg/kg_in_Corn_Oil-Rat-Kidney-1d-dn	10/275	0,007240177	0,047919023	0	0	2,499218994	12,31642504	EEF1B2;RPS17;RPL32;ARL2BP;RPS8;ARL6IP5;RPL35A;RPSA;RPS3A;RPL39
Cephalexin-2500_mg/kg_in_CMC-Rat-Kidney-3d-dn	10/275	0,007240177	0,047878789	0	0	2,499218994	12,31642504	EEF1B2;RPL32;RPS19;ARL2BP;CLP1;RPS8;RPS6;RPL35A;RPS20;RPL10A
Lamotrigine-26_mg/kg_in_CMC-Rat-Kidney-5d-dn	10/275	0,007240177	0,047838622	0	0	2,499218994	12,31642504	RPL32;RPS19;ARL2BP;RPS8;RPS6;RPS20;RPSA;RPL10A;SEPP1;TPT1
Norethindrone-375_mg/kg_in_Corn_Oil-Rat-Liver-3d-up	10/275	0,007240177	0,047798522	0	0	2,499218994	12,31642504	EEF1B2;RPS17;RPL32;RPS19;RPS8;RPS5;RPS6;RPS20;RPSA;RPL10A
Piracetam-2500_mg/kg_in_CMC-Rat-Brain-5d-dn	10/275	0,007240177	0,04775849	0	0	2,499218994	12,31642504	EEF1B2;RPS17;RPL32;RPS19;ARL2BP;RPS8;ARL6IP5;RPS20;RPS3A;GOLGA7
Ascorbic_Acid-2000_mg/kg_in_Water-Rat-Kidney-3d-dn	9/233	0,007292325	0,048062219	0	0	2,65474979	13,06384544	EEF1B2;RPL32;RPS19;CLP1;RPS8;ARL6IP5;RPL35A;RPL10A;RPL39
Neomycin-56_mg/kg_in_Corn_Oil-Rat-Kidney-3d-dn	9/233	0,007292325	0,048022034	0	0	2,65474979	13,06384544	EEF1B2;RPL32;RPS19;ARL2BP;CLP1;RPS3A;RPL10A;SEPP1;TPT1
3-Methylcholanthrene-12.5_mg/kg_in_CMC-Rat-Liver-91d-dn	11/319	0,007300668	0,048036809	0	0	2,369949046	11,65965028	EEF1B2;RPS17;RPL32;RPS19;RPS8;RPS6;RPS20;RPSA;RPS3A;RPL10A;TPT1
Cytarabine-23_mg/kg_in_Saline-Rat-Bone_marrow-1d-dn	11/319	0,007300668	0,047996712	0	0	2,369949046	11,65965028	RPS17;RPL32;RPS8;FEM1B;RPS5;RPL13A;RPL35A;RPSA;RPS3A;RPL10A;TPT1
Fenofibrate-215_mg/kg_in_Corn_Oil-Rat-Heart-3d-up	11/319	0,007300668	0,047956681	0	0	2,369949046	11,65965028	RPS19;ARL2BP;RPS6;ARL6IP5;RPS20;RPSA;RAPGEF5;RPL10A;SEPP1;RPL39;TPT1
Finasteride-800_mg/kg_in_Corn_Oil-Rat-Liver-0.25d-up	12/364	0,007323767	0,048068326	0	0	2,265775462	11,13998057	EEF1B2;C1QA;RPS17;RPL32;RPS19;RPS8;RPS5;RPL35A;RPS20;RPSA;RPL10A;RPL39
Aceclofenac-9_mg/kg_in_Corn_Oil-Rat-Kidney-1d-dn	10/276	0,007418845	0,048651809	0	0	2,490163853	12,21109596	EEF1B2;RPS17;RPL32;ARL2BP;RPS8;RPS6;RPL13A;RPL35A;RPS3A;TOMM20
Benoxaprofen-89_mg/kg_in_CMC-Rat-Liver-1d-up	10/276	0,007418845	0,048611333	0	0	2,490163853	12,21109596	EEF1B2;RPS17;RPL32;RPS8;RPS6;RPL35A;RPS20;RPS3A;RPL10A;SEPP1
Hydrazine-45_mg/kg_in_Water-Rat-Kidney-3d-dn	10/276	0,007418845	0,048570924	0	0	2,490163853	12,21109596	RPS17;RPL32;RPS19;FEM1B;RPS6;ARL6IP5;RPL35A;RPSA;RPS3A;RPL10A
Ofloxacin-3000_mg/kg_in_Water-Rat-Kidney-3d-dn	10/276	0,007418845	0,048530583	0	0	2,490163853	12,21109596	RPS17;RPL32;RPS19;RPS6;RPL35A;RPSA;RPS3A;RPL10A;RPL39;TPT1
Dexamethasone-300_mg/kg_in_Corn_Oil-Rat-Kidney-3d-up	11/320	0,007465838	0,048797463	0	0	2,362542955	11,57035935	C1QA;RPS19;RPS8;RPS6;ARL6IP5;RPS20;RPSA;RPL10A;SEPP1;GOLGA7;TPT1
Dobutamine-43_mg/kg_in_Saline-Rat-Heart-1d-up	11/320	0,007465838	0,048757	0	0	2,362542955	11,57035935	EEF1B2;RPS17;RPL32;RPS19;ARL6IP5;RPL35A;RPS20;RPSA;RPL10A;RPL39;TPT1
Butenafine-150_mg/kg_in_Corn_Oil-Rat-Liver-3d-up	10/277	0,007600904	0,049597943	0	0	2,481174092	12,10685955	EEF1B2;RPS17;RPL32;RPS19;CLP1;RPS8;RPS6;RPS20;RPL10A;SEPP1
Esmolol-27_mg/kg_in_Saline-Rat-Heart-1d-dn	10/277	0,007600904	0,049556885	0	0	2,481174092	12,10685955	EEF1B2;RPL32;ARL2BP;RPS8;RPS5;FUNDC1;RPS20;RPS3A;SEPP1;TPT1
Fluphenazine-2.5_mg/kg_in_Corn_Oil-Rat-Liver-5d-dn	10/277	0,007600904	0,049515895	0	0	2,481174092	12,10685955	C1QA;RPS17;RPL32;RPS19;RPS8;RPS6;RPL35A;RPSA;RPS3A;RPL10A
Acrolein-20_mg/kg_in_Water-Rat-Heart-3d-dn	11/321	0,007633911	0,04968982	0	0	2,355183008	11,4818822	EEF1B2;RPS17;CXCL11;RPL32;RPS8;COL5A3;RPS6;RPSA;RPS3A;RPL39;TPT1
Aldosterone-0.065_mg/kg_in_Saline-Rat-Heart-3d-dn	11/321	0,007633911	0,049648788	0	0	2,355183008	11,4818822	EEF1B2;RPS17;RPL32;RPS19;RPS5;RPS6;RPS20;RPS3A;RPL10A;SEPP1;RPL39
Artemisinin-2000_mg/kg_in_CMC-Rat-Liver-1d-up	11/321	0,007633911	0,049607824	0	0	2,355183008	11,4818822	RPS17;RPS19;RPS8;RPS5;RPS6;RPL35A;RPSA;RPS3A;RPL10A;RPL39;TPT1
Chlorambucil-0.6_mg/kg_in_Corn_Oil-Rat-Liver-3d-dn	11/321	0,007633911	0,049566927	0	0	2,355183008	11,4818822	C1QA;RPS17;RPL32;RPS19;RPS6;RPSA;RPS3A;RPL10A;RPL39;GOLGA7;TPT1
Oxymetholone-1170_mg/kg_in_CMC-Rat-Liver-1d-up	11/321	0,007633911	0,049526098	0	0	2,355183008	11,4818822	RPS17;RPL32;RPS5;ARL6IP5;RPL35A;RPS20;RPSA;RPS3A;RPL10A;RPL39;TPT1
Oxytetracycline-1500_mg/kg_in_Water-Rat-Liver-3d-up	11/321	0,007633911	0,049485335	0	0	2,355183008	11,4818822	RPS17;RPL32;RPS19;ARL2BP;RPS8;RPS6;ARL6IP5;RPS20;RPSA;RPS3A;RPL10A
Procaine-2500_mg/kg_in_Water-Rat-Heart-1d-dn	11/321	0,007633911	0,04944464	0	0	2,355183008	11,4818822	EEF1B2;RPS17;RPL32;RPS8;RPS5;RPL35A;RPS20;RPS3A;RPL39;TOMM20;TPT1
Rofecoxib-775_mg/kg_in_Corn_Oil-Rat-Kidney-5d-up	11/321	0,007633911	0,049404012	0	0	2,355183008	11,4818822	RPS17;RPS19;ARL2BP;CLP1;RPS8;RPS5;RPS6;RPL35A;RPS20;GOLGA7;TPT1
Valproic_Acid-850_mg/kg_in_Water-Rat-Bone_marrow-3d-up	11/321	0,007633911	0,04936345	0	0	2,355183008	11,4818822	C1QA;RPS17;RPL32;RPS19;RPS8;RPS5;RPS6;NKG7;RPL35A;RPSA;RPL10A
Bisacodyl-1750_mg/kg_in_CMC-Rat-Intestine-1d-dn	10/278	0,007786395	0,050308162	0	0	2,472249005	12,00370158	EEF1B2;C1QA;RPS19;ARL2BP;RPS6;RPS20;RPSA;RPS3A;RPL39;SEPP1
Carvedilol-2000_mg/kg_in_Corn_Oil-Rat-Heart-3d-up	10/278	0,007786395	0,050266926	0	0	2,472249005	12,00370158	RPS17;RPL32;RPS19;RPS6;RPL35A;RPS20;RAPGEF5;RPL10A;RPL39;SEPP1
Chloroquine-21.95_uM_in_DMSO-Rat-Primary_rat_hepatocytes-1d-up	10/278	0,007786395	0,050225758	0	0	2,472249005	12,00370158	EEF1B2;RPS17;CXCL11;RPL32;RPL35A;RPSA;RPL10A;GOLGA7;SEPP1;SLC16A3
Clozapine-95_mg/kg_in_CMC-Rat-Heart-5d-up	10/278	0,007786395	0,050184656	0	0	2,472249005	12,00370158	RPS17;RPL32;ARL2BP;RPS8;FUNDC1;RPS6;RPL35A;RPS3A;RAPGEF5;TOMM20
Doxorubicin-1.5_uM_in_DMSO-Rat-Primary_rat_hepatocytes-1d-up	10/278	0,007786395	0,050143622	0	0	2,472249005	12,00370158	EEF1B2;RPS17;CXCL11;RPL32;FEM1B;RPS5;RPS20;RPSA;SEPP1;TOMM20
Isoeugenol-1560_mg/kg_in_CMC-Rat-Liver-1d-up	10/278	0,007786395	0,050102655	0	0	2,472249005	12,00370158	EEF1B2;RPS19;RPS8;RPS5;RPS6;RPSA;RPS3A;RPL10A;RPL39;TPT1
Atorvastatin-2.5_mg/kg_in_Corn_Oil-Rat-Kidney-3d-dn	11/322	0,00780492	0,050180854	0	0	2,347868775	11,39420959	EEF1B2;RPS17;RPL32;RPS19;RPS8;RPS6;RPSA;RPL10A;SEPP1;RPL39;TPT1
Azathioprine-20_mg/kg_in_Corn_Oil-Rat-Liver-0.25d-up	11/322	0,00780492	0,050139924	0	0	2,347868775	11,39420959	EEF1B2;RPS19;RPS8;RPS6;RPS20;RPSA;RPS3A;RPL10A;SEPP1;RPL39;TPT1
Capsaicin-35_mg/kg_in_CMC-Rat-Liver-3d-dn	11/322	0,00780492	0,05009906	0	0	2,347868775	11,39420959	C1QA;RPL32;RPS19;CLP1;FEM1B;RPS5;RPS6;RPS20;RPSA;RPL10A;TPT1
Ofloxacin-3000_mg/kg_in_Water-Rat-Kidney-5d-dn	9/236	0,007898716	0,050659844	0	0	2,62100297	12,68841966	RPS17;RPL32;RPS19;RPS6;RPS20;RPSA;RPS3A;RPL10A;RPL39
44'-Diethylaminoethoxyhexestrol-20_uM_in_DMSO-Rat-Primary_rat_hepatocytes-1d-up	10/279	0,007975362	0,051109805	0	0	2,463387897	11,90160799	EEF1B2;RPL32;RPS8;RPS5;RPL35A;RPS20;RPSA;RPS3A;RPL10A;PDRG1
Carmustine-16_mg/kg_in_Corn_Oil-Rat-Liver-0.25d-dn	10/279	0,007975362	0,051068252	0	0	2,463387897	11,90160799	EEF1B2;RPS17;RPL32;RPS19;RPS8;RPL35A;RPS20;RPS3A;RPL39;TPT1
Lead(IV)_Acetate-200_mg/kg_in_Saline-Rat-Liver-1d-dn	10/279	0,007975362	0,051026767	0	0	2,463387897	11,90160799	EEF1B2;RPL32;RPS19;RPS8;RPS5;RPS6;RPSA;RPS3A;RPL10A;TPT1
Rabeprazole-1024_mg/kg_in_Water-Rat-Liver-1d-up	10/279	0,007975362	0,050985349	0	0	2,463387897	11,90160799	EEF1B2;RPS17;RPL32;RPS19;RPS6;RPS20;RPSA;RPS3A;RPL10A;RPL39
Sulfaphenazole-1695_mg/kg_in_Water-Rat-Liver-5d-dn	10/279	0,007975362	0,050943998	0	0	2,463387897	11,90160799	EEF1B2;RPS17;RPL32;RPS19;CLP1;RPS8;RPS6;RPS20;RPL39;SEPP1
Vinblastine-0.3_mg/kg_in_Saline-Rat-Bone_marrow-1d-dn	10/279	0,007975362	0,050902715	0	0	2,463387897	11,90160799	EEF1B2;RPS17;RPS8;RPS6;NKG7;RPS20;RPSA;RPS3A;RPL10A;RPL39
Buspirone-196_mg/kg_in_CMC-Rat-Heart-1d-dn	11/323	0,007978898	0,050884049	0	0	2,340599832	11,30733241	RPS17;RPL32;RPS19;RPS8;RPS20;RPSA;RPS3A;RPL10A;SEPP1;RPL39;TPT1
Digoxin-11_mg/kg_in_Corn_Oil-Rat-Liver-1d-dn	11/323	0,007978898	0,050842881	0	0	2,340599832	11,30733241	EEF1B2;C1QA;RPS17;RPL32;RPS8;RPS5;RPS6;RPS3A;RPL10A;GOLGA7;TPT1
Hydroxyurea-59_mg/kg_in_Saline-Rat-Bone_marrow-1d-dn	11/323	0,007978898	0,050801779	0	0	2,340599832	11,30733241	RPS17;RPL32;RPS19;RPS8;RPS5;RPL35A;RPS20;RPSA;RPL10A;SEPP1;TPT1
Proglumide-1125_mg/kg_in_CMC-Rat-Liver-3d-dn	11/323	0,007978898	0,050760743	0	0	2,340599832	11,30733241	EEF1B2;RPL32;RPS19;CLP1;RPS8;FEM1B;RPS5;RPS20;RPSA;RPL10A;SEPP1
Beta-Estradiol-150_mg/kg_in_Corn_Oil-Rat-Liver-0.25d-up	12/369	0,008117183	0,051598815	0	0	2,235073897	10,75913643	EEF1B2;RPS17;RPL32;RPS19;FEM1B;RPS6;RPSA;RPS3A;RPL10A;SEPP1;RPL39;PDRG1
Isotretinoin-13_mg/kg_in_Corn_Oil-Rat-Liver-0.25d-dn	11/324	0,00815588	0,051802993	0	0	2,333375758	11,22124167	EEF1B2;RPS17;RPL32;RPS8;RPS5;RPL35A;RPS20;RPSA;RPS3A;RPL39;TPT1
Lomustine-4.2_mg/kg_in_Corn_Oil-Rat-Liver-0.25d-up	11/324	0,00815588	0,05176125	0	0	2,333375758	11,22124167	EEF1B2;RPS17;RPL32;RPS19;RPS6;RPSA;RPS3A;RPL10A;SEPP1;RPL39;TPT1
Nortriptyline-63_mg/kg_in_Water-Rat-Heart-0.25d-up	11/324	0,00815588	0,051719574	0	0	2,333375758	11,22124167	RPL32;RPS19;ARL2BP;RPS8;RPS6;ARL6IP5;RPL35A;RPS20;RPSA;RPL39;TPT1
Carboplatin-14_mg/kg_in_Saline-Rat-Liver-0.25d-up	10/280	0,008167845	0,051753779	0	0	2,454590083	11,80056499	EEF1B2;C1QA;RPS17;RPS8;RPS6;RPL35A;RPS20;RPS3A;RPL39;TPT1
Chlorpromazine-18_mg/kg_in_Water-Rat-Liver-5d-dn	10/280	0,008167845	0,051712176	0	0	2,454590083	11,80056499	EEF1B2;RPS17;RPL32;RPS8;RPS5;RPL35A;RPS20;RPSA;RPS3A;TPT1
Letrozole-250_mg/kg_in_Corn_Oil-Rat-Liver-3d-up	10/280	0,008167845	0,05167064	0	0	2,454590083	11,80056499	RPL32;RPS19;RPS8;RPS5;RPL35A;RPS20;RPSA;RPL10A;RPL39;SEPP1
Rofecoxib-3_mg/kg_in_Corn_Oil-Rat-Liver-0.25d-dn	10/280	0,008167845	0,051629171	0	0	2,454590083	11,80056499	EEF1B2;RPS17;RPL32;RPS8;RPS5;RPS20;RPSA;RPS3A;RPL39;TPT1
Sulconazole-40_uM_in_DMSO-Rat-Primary_rat_hepatocytes-1d-up	10/280	0,008167845	0,051587768	0	0	2,454590083	11,80056499	EEF1B2;RPS17;RPL32;RPS8;RPS5;RPL35A;RPS20;RPSA;RPS3A;RPL10A
Pentamidine-18_mg/kg_in_Saline-Rat-Bone_marrow-3d-dn	13/416	0,008233176	0,05195873	0	0	2,147766323	10,30838362	C1QA;RPL32;RPS8;FEM1B;RPS5;RPSA;RPL10A;EEF1B2;RPS17;RPS19;ARL6IP5;RPS20;RPL39
Clarithromycin-56_mg/kg_in_Water-Rat-Liver-0.25d-up	12/370	0,008283656	0,052235449	0	0	2,229033157	10,68480545	EEF1B2;RPS17;RPL32;RPS6;RPL35A;RPS20;RPSA;RPS3A;RPL10A;SEPP1;RPL39;TPT1
D-Tubocurarine_Chloride-0.033_mg/kg_in_Saline-Rat-Heart-3d-up	12/370	0,008283656	0,052193661	0	0	2,229033157	10,68480545	RPL32;RPS19;ARL2BP;RPS8;FEM1B;RPS6;RPS20;RPSA;RPS3A;RPL10A;RPL39;TPT1
Gemfibrozil-700_mg/kg_in_Corn_Oil-Rat-Liver-3d-dn	11/325	0,0083359	0,052480852	0	0	2,326196141	11,13592853	EEF1B2;C1QA;RPS17;RPL32;RPS8;RPS6;RPL35A;RPSA;RPL10A;SEPP1;RPL39
Hydralazine-280_mg/kg_in_Water-Rat-Heart-5d-dn	11/325	0,0083359	0,052438935	0	0	2,326196141	11,13592853	C1QA;RPS17;RPL32;RPS19;RPS8;COL5A3;RPS6;RPS20;RPSA;TOMM20;TPT1
Olanzapine-23_mg/kg_in_CMC-Rat-Liver-5d-up	11/325	0,0083359	0,052397084	0	0	2,326196141	11,13592853	EEF1B2;RPS17;RPL32;RPS19;RPS6;RPL35A;RPS20;RPL10A;SEPP1;RPL39;TPT1
Oxcarbazepine-1600_mg/kg_in_CMC-Rat-Heart-5d-dn	11/325	0,0083359	0,0523553	0	0	2,326196141	11,13592853	C1QA;RPS17;RPS19;RPS6;ARL6IP5;RPS3A;RPL10A;SEPP1;GOLGA7;TPT1;PDRG1
Ethinylestradiol-1480_mg/kg_in_Corn_Oil-Rat-Liver-0.25d-up	10/281	0,008363887	0,05248922	0	0	2,445854887	11,700559	EEF1B2;RPS17;RPS19;RPS8;RPS6;RPS20;RPSA;RPS3A;RPL10A;RPL39
Mesna-755_mg/kg_in_Saline-Rat-Kidney-5d-up	10/281	0,008363887	0,052447429	0	0	2,445854887	11,700559	C1QA;RPS17;CXCL11;RPL32;RPS19;RPS5;RPS20;RPSA;RAPGEF5;SEPP1
Progesterone-164_mg/kg_in_Corn_Oil-Rat-Liver-1d-up	10/281	0,008363887	0,052405705	0	0	2,445854887	11,700559	RPL32;RPS19;RPS8;RPS5;RPS6;RPS20;RPSA;RPS3A;RPL10A;TPT1

3-Chloroaniline-200_mg/kg_in_CMC-Rat-Kidney-1d-dn	11/326	0,008518991	0,053335114	0	0	2,31906057	11,05138426	EEF1B2;RPL32;ARL2BP;RPS8;RPS6;RPS20;RPSA;RPL10A;RPL39;TOMM20;TPT1
4-Chloroaniline-75_mg/kg_in_CMC-Rat-Kidney-3d-up	11/326	0,008518991	0,053292751	0	0	2,31906057	11,05138426	EEF1B2;RPS17;CXCL11;RPS19;RPS8;ARL6IP5;RPS20;RPS3A;RAPGEF5;GOLGA7;TPT1
Azathioprine-160_mg/kg_in_Corn_Oil-Rat-Liver-1d-up	11/326	0,008518991	0,053250456	0	0	2,31906057	11,05138426	EEF1B2;RPS17;RPL32;RPS19;RPS8;RPS6;RPS20;RPSA;RPL10A;SEPP1;RPL39
Cortisone-206_mg/kg_in_CMC-Rat-Liver-1d-dn	11/326	0,008518991	0,053208227	0	0	2,31906057	11,05138426	EEF1B2;C1QA;RPL32;RPS19;RPS8;RPS6;ARL6IP5;RPS20;RPS3A;RPL10A;TPT1
Digitonin-75_mg/kg_in_Corn_Oil-Rat-Heart-1d-up	11/326	0,008518991	0,053166065	0	0	2,31906057	11,05138426	EEF1B2;RPS17;RPL32;RPS19;RPS6;RPS20;RPSA;RPS3A;RPL39;TPT1;PDRG1
Dimenhydrinate-165_mg/kg_in_CMC-Rat-Liver-1d-up	11/326	0,008518991	0,05312397	0	0	2,31906057	11,05138426	C1QA;RPS17;RPL32;RPS19;RPS8;RPS6;RPSA;RPS3A;RPL39;TPT1;PDRG1
Itraconazole-1093_mg/kg_in_Corn_Oil-Rat-Liver-5d-dn	10/282	0,008563529	0,053359457	0	0	2,437181643	11,60157665	EEF1B2;RPS17;RPL32;RPS8;ARL6IP5;RPL35A;RPSA;RPS3A;SEPP1;TPT1
Meropenem-1500_mg/kg_in_CMC-Rat-Spleen-5d-up	10/282	0,008563529	0,053317276	0	0	2,437181643	11,60157665	EEF1B2;RPL32;RPS19;RPS5;RPS6;RPS20;RPSA;RPL10A;RPL39;SEPP1
Naproxen-134_mg/kg_in_Corn_Oil-Rat-Kidney-1d-dn	10/282	0,008563529	0,053275161	0	0	2,437181643	11,60157665	EEF1B2;RPS17;RPL32;ARL2BP;RPS8;ARL6IP5;RPL35A;RPSA;RPS3A;RPL39
Sulfaphenazole-1695_mg/kg_in_Water-Rat-Liver-1d-up	10/282	0,008563529	0,053233113	0	0	2,437181643	11,60157665	RPS17;RPL32;RPS8;RPS5;RPL35A;RPSA;RPS3A;RPL10A;RPL39;TPT1
Valproic_Acid-850_mg/kg_in_Water-Rat-Bone_marrow-1d-up	10/282	0,008563529	0,053191131	0	0	2,437181643	11,60157665	EEF1B2;RPS17;RPL32;RPS8;RPS5;NKG7;RPL13A;RPL35A;RPS20;TOMM20
Allyl_Alcohol-16_mg/kg_in_Saline-Rat-Liver-0.25d-up	11/327	0,008705188	0,054028419	0	0	2,311968641	10,96760024	EEF1B2;C1QA;RPL32;RPS19;RPS8;RPS5;RPS20;RPSA;RPS3A;SEPP1;RPL39
Benzethonium_Chloride-5_mg/kg_in_Water-Rat-Heart-3d-up	11/327	0,008705188	0,053985877	0	0	2,311968641	10,96760024	RPS17;RPL32;RPS19;ARL2BP;RPS8;RPS6;RPL35A;RPS20;RPSA;GOLGA7;TPT1
Famotidine-1200_mg/kg_in_CMC-Rat-Heart-5d-up	11/327	0,008705188	0,053943401	0	0	2,311968641	10,96760024	EEF1B2;RPS17;RPL32;ARL6IP5;RPS20;RPSA;RAPGEF5;RPL10A;SEPP1;RPL39;PDRG1
Indomethacin-4.5_mg/kg_in_Water-Rat-Kidney-1d-up	11/327	0,008705188	0,053900993	0	0	2,311968641	10,96760024	EEF1B2;RPS17;RPL32;RPS8;RPS5;RPS6;RPL13A;RPL35A;RPS3A;SEPP1;TOMM20
Streptomycin-120_mg/kg_in_Saline-Rat-Kidney-5d-dn	11/327	0,008705188	0,053858651	0	0	2,311968641	10,96760024	EEF1B2;ARL2BP;RPS8;FUNDC1;RPS6;RPS20;RPS3A;RPL10A;SEPP1;RPL39;TPT1
Acyclovir-330_mg/kg_in_Saline-Rat-Spleen-1d-dn	10/283	0,008766814	0,054197352	0	0	2,428569694	11,50360479	EEF1B2;RPS17;CXCL11;RPS8;RPS5;FUNDC1;RPL13A;RPL35A;RPSA;TPT1
Lomustine-4.2_mg/kg_in_Corn_Oil-Rat-Bone_marrow-0.25d-up	10/283	0,008766814	0,054154845	0	0	2,428569694	11,50360479	EEF1B2;C1QA;RPS17;RPL32;RPL35A;RPS20;RPS3A;RPL10A;RPL39;TPT1
N-Nitrosodiethylamine-34_mg/kg_in_Saline-Rat-Spleen-1d-dn	10/283	0,008766814	0,054112404	0	0	2,428569694	11,50360479	RPS17;RPL32;RPS8;RPS5;FUNDC1;RPS6;RPL13A;RPL35A;RPS3A;TPT1
Tamoxifen-32_mg/kg_in_Corn_Oil-Rat-Liver-1d-up	10/283	0,008766814	0,054070029	0	0	2,428569694	11,50360479	EEF1B2;RPS17;RPL32;RPS19;RPS8;RPS6;ARL6IP5;RPSA;RPS3A;RPL10A
Thioguanine-24_mg/kg_in_Corn_Oil-Rat-Kidney-5d-dn	10/283	0,008766814	0,054027721	0	0	2,428569694	11,50360479	EEF1B2;RPS17;RPL32;RPS19;RPS6;RPSA;RPS3A;RPL10A;RPL39;TPT1
Omeprazole-415_mg/kg_in_Corn_Oil-Rat-Liver-1d-up	11/328	0,008894526	0,054771918	0	0	2,304919956	10,88456798	EEF1B2;RPS17;RPL32;RPS19;RPS8;RPS20;RPSA;RPS3A;SEPP1;RPL39;TPT1
Troglitazone-1200_mg/kg_in_Corn_Oil-Rat-Liver-5d-dn	11/328	0,008894526	0,054729128	0	0	2,304919956	10,88456798	RPS17;RPL32;RPS19;RPS6;RPL35A;RPS20;RPSA;RPS3A;RPL10A;RPL39;TPT1
Famciclovir-1200_mg/kg_in_Saline-Rat-Liver-0.25d-up	10/284	0,008973784	0,05517371	0	0	2,420018392	11,40663047	C1QA;RPS17;RPL32;RPS8;RPS6;RPL35A;RPS20;RPSA;RPS3A;SEPP1
Pralidoxime_Chloride-48_mg/kg_in_Saline-Rat-Heart-3d-dn	10/284	0,008973784	0,055130673	0	0	2,420018392	11,40663047	EEF1B2;RPS17;RPL32;ARL2BP;RPS8;RPS5;FUNDC1;RPSA;RPL39;TOMM20
Sulfadiazine-1170_mg/kg_in_Water-Rat-Kidney-3d-dn	10/284	0,008973784	0,055087703	0	0	2,420018392	11,40663047	RPS17;RPL32;RPS19;RPS6;RPL35A;RPS20;RPSA;RPS3A;RPL10A;RPL39
Zomepirac-2.8_mg/kg_in_Saline-Rat-Liver-0.25d-up	10/284	0,008973784	0,0550448	0	0	2,420018392	11,40663047	C1QA;RPS17;RPL32;RPS8;RPS5;RPL35A;RPSA;RPS3A;TOMM20;TPT1
Trimethadione-535_mg/kg_in_Water-Rat-Kidney-5d-dn	9/241	0,008993894	0,055125223	0	0	2,566625315	12,09190914	RPS17;RPL32;RPS19;RPS6;RPL35A;RPS20;RPSA;RPS3A;RPL39
Bithionol-333_mg/kg_in_Corn_Oil-Rat-Kidney-5d-up	11/329	0,009087037	0,055652803	0	0	2,297914121	10,80227911	EEF1B2;RPS17;RPL32;RPS19;ARL2BP;RPS5;RPS6;ARL6IP5;RPL35A;RPS20;RPS3A
Bithionol-59_mg/kg_in_Corn_Oil-Rat-Liver-0.25d-up	11/329	0,009087037	0,055609561	0	0	2,297914121	10,80227911	EEF1B2;RPS17;RPL32;RPS19;RPS6;RPL35A;RPS20;RPS3A;SEPP1;RPL39;TPT1
Idarubicin-4_mg/kg_in_Saline-Rat-Heart-5d-dn	11/329	0,009087037	0,055566386	0	0	2,297914121	10,80227911	EEF1B2;RPS17;CXCL11;RPL32;RPS8;RPS6;ARL6IP5;RPS20;RPS3A;RPL39;TPT1
Lomustine-8.75_mg/kg_in_Corn_Oil-Rat-Liver-5d-up	11/329	0,009087037	0,055523278	0	0	2,297914121	10,80227911	EEF1B2;RPS17;RPL32;RPS19;RPS20;RPSA;RPS3A;RPL10A;SEPP1;RPL39;TPT1
Lomustine-8.75_mg/kg_in_Corn_Oil-Rat-Spleen-5d-dn	11/329	0,009087037	0,055480237	0	0	2,297914121	10,80227911	EEF1B2;RPS17;RPL32;RPS8;RPS5;RPL13A;RPL35A;RPSA;RPS3A;RPL39;TPT1
Methocarbamol-1125_mg/kg_in_CMC-Rat-Liver-5d-up	11/329	0,009087037	0,055437262	0	0	2,297914121	10,80227911	C1QA;RPS17;RPS19;FEM1B;RPS6;RPS20;RPSA;RPL10A;RPL39;GOLGA7;TPT1
Zaleplon-100_mg/kg_in_Water-Rat-Liver-3d-dn	11/329	0,009087037	0,055394354	0	0	2,297914121	10,80227911	EEF1B2;RPL32;RPS19;RPS8;RPL35A;RPS20;RPS3A;RPL10A;SEPP1;RPL39;TPT1
Zileuton-450_mg/kg_in_Corn_Oil-Rat-Kidney-5d-up	11/329	0,009087037	0,055351512	0	0	2,297914121	10,80227911	RPS17;RPL32;RPS19;RPS8;RPS5;RPS6;RPL35A;RPS20;RPSA;RPS3A;RPL39
Levofloxacin-739_mg/kg_in_Water-Rat-Kidney-1d-dn	8/200	0,009137289	0,055614594	0	0	2,749140893	12,90829304	RPS17;RPL32;RPS19;RPS6;RPSA;RPS3A;RPL10A;RPL39
123-Trichloropropane-108_mg/kg_in_CMC-Rat-Kidney-3d-up	10/285	0,009184481	0,055858667	0	0	2,4115271	11,31064097	EEF1B2;RPS17;RPL32;RPS19;RPS8;RPS3A;RPL10A;RPL39;SEPP1;TPT1
Diethylstilbestrol-280_mg/kg_in_Corn_Oil-Rat-Liver-0.25d-up	10/285	0,009184481	0,055815566	0	0	2,4115271	11,31064097	EEF1B2;RPS17;RPL32;RPS19;RPS8;RPS6;RPSA;RPS3A;RPL10A;RPL39
Sulfathiazole-2629_mg/kg_in_Water-Rat-Kidney-3d-dn	10/285	0,009184481	0,055772532	0	0	2,4115271	11,31064097	RPS17;RPL32;RPS19;RPS6;ARL6IP5;RPL35A;RPS20;RPSA;RPL10A;RPL39
Diclofenac-10_mg/kg_in_Corn_Oil-Rat-Liver-0.25d-up	11/330	0,009282758	0,056325885	0	0	2,290950745	10,72072537	CXADR;RPS19;RPS8;FEM1B;RPS5;RPS6;ARL6IP5;RPL35A;RPS20;RPL10A;TOMM20
Neomycin-56_mg/kg_in_Corn_Oil-Rat-Kidney-5d-up	11/330	0,009282758	0,056282524	0	0	2,290950745	10,72072537	RPS17;CXCL11;RPL32;RPS19;ARL2BP;RPS8;R3HCC1;RPS6;RPS20;RPS3A;TPT1
Ifosfamide-17_mg/kg_in_Saline-Rat-Liver-3d-up	12/376	0,009339396	0,056582371	0	0	2,193463479	10,25118164	C1QA;RPS17;RPL32;RPS19;RPS8;RPS5;RPS6;RPL35A;RPS20;RPSA;RPS3A;TPT1
Amiodarone-147_mg/kg_in_CMC-Rat-Liver-3d-dn	10/286	0,009398948	0,056899396	0	0	2,403095187	11,21562375	RPS17;RPL32;RPS19;RPS8;RPS6;RPL35A;RPS20;RPL39;SEPP1;TPT1
Diethylstilbestrol-2.8_mg/kg_in_Corn_Oil-Rat-Bone_marrow-1d-dn	10/286	0,009398948	0,056855695	0	0	2,403095187	11,21562375	RPL32;RPS19;RPS8;FEM1B;RPS6;ARL6IP5;RPL35A;RPS20;RPSA;RPS3A
Fenoprofen-52_mg/kg_in_Corn_Oil-Rat-Kidney-5d-up	10/286	0,009398948	0,05681206	0	0	2,403095187	11,21562375	RPS17;RPL32;RPS8;RPS5;RPS6;RPL35A;RPSA;RPS3A;RAPGEF5;TPT1
Indomethacin-9.6_mg/kg_in_Water-Rat-Liver-1d-dn	10/286	0,009398948	0,056768493	0	0	2,403095187	11,21562375	EEF1B2;RPS17;RPL32;RPS8;RPS5;RPL35A;RPS20;RPSA;RPS3A;TPT1
Isoniazid-79_mg/kg_in_Water-Rat-Liver-5d-dn	10/286	0,009398948	0,056724992	0	0	2,403095187	11,21562375	EEF1B2;RPL32;RPS19;RPS8;RPS6;RPS3A;RPL10A;RPL39;SEPP1;TPT1
NN-Dimethylformamide-700_mg/kg_in_Saline-Rat-Liver-0.25d-up	10/286	0,009398948	0,056681558	0	0	2,403095187	11,21562375	RPL32;RPS19;RPS8;RPS6;RPSA;RPS3A;RAPGEF5;RPL10A;SEPP1;TPT1
Sulfisoxazole-2500_mg/kg_in_Corn_Oil-Rat-Liver-3d-up	10/286	0,009398948	0,05663819	0	0	2,403095187	11,21562375	EEF1B2;RPL32;RPS19;RPS8;RPS5;RPS6;RPL35A;RPSA;SEPP1;TOMM20
2-Amino-4-Nitrophenol-625_mg/kg_in_CMC-Rat-Heart-3d-dn	11/331	0,009481721	0,057093299	0	0	2,284029443	10,63989862	RPS17;RPL32;RPS19;ARL2BP;COL5A3;RPS6;RPS20;RPSA;RPS3A;RPL39;TPT1
6-Mercaptopurine-25_mg/kg_in_Corn_Oil-Rat-Kidney-5d-dn	11/331	0,009481721	0,057049683	0	0	2,284029443	10,63989862	EEF1B2;RPS17;RPL32;RPS8;RPS6;RPS20;RPSA;RPS3A;SEPP1;RPL39;TPT1
Beta-Estradiol-0.3_mg/kg_in_Corn_Oil-Rat-Liver-3d-up	11/331	0,009481721	0,057006134	0	0	2,284029443	10,63989862	EEF1B2;C1QA;RPL32;RPS19;RPS6;RPL35A;RPS20;RPL10A;SEPP1;RPL39;TPT1
Coumarin-111_mg/kg_in_Corn_Oil-Rat-Bone_marrow-0.25d-dn	11/331	0,009481721	0,056962651	0	0	2,284029443	10,63989862	RPS17;RPL32;RPS19;RPS8;FEM1B;RPS5;RPS6;RPL35A;RPS20;RPSA;RPL10A
Gemcitabine-11_mg/kg_in_Saline-Rat-Bone_marrow-1d-up	11/331	0,009481721	0,056919234	0	0	2,284029443	10,63989862	EEF1B2;C1QA;RPS17;RPL32;ARL2BP;RPS8;RPS6;ARL6IP5;NKG7;RPL35A;RPS3A
Carboplatin-6_mg/kg_in_Saline-Rat-Bone_marrow-3d-up	12/377	0,009525101	0,057136099	0	0	2,187645273	10,18091767	EEF1B2;RPS17;RPL32;RPS8;RPS5;RPS6;RPL13A;RPS20;RPSA;RPS3A;RPL10A;SEPP1
Benzocaine-1100_mg/kg_in_Corn_Oil-Rat-Liver-1d-dn	10/287	0,009617227	0,057644808	0	0	2,394722033	11,12156648	EEF1B2;C1QA;RPS17;RPL32;RPS8;RPS6;RPS3A;RPL10A;RPL39;TPT1
Cisplatin-1.17_mg/kg_in_Saline-Rat-Liver-5d-dn	10/287	0,009617227	0,057600972	0	0	2,394722033	11,12156648	EEF1B2;C1QA;RPS17;RPL32;CLP1;RPS20;RPSA;RPS3A;RPL39;TPT1
Ethacrynic_Acid-125_mg/kg_in_CMC-Rat-Kidney-1d-up	10/287	0,009617227	0,057557202	0	0	2,394722033	11,12156648	EEF1B2;RPL32;RPS19;ARL2BP;RPS8;RPS5;RPS6;RPS20;RPL10A;TPT1
Mitomycin_C-1.7_mg/kg_in_Saline-Rat-Spleen-3d-dn	10/287	0,009617227	0,057513499	0	0	2,394722033	11,12156648	EEF1B2;C1QA;RPS17;RPL32;RPS8;RPS5;RPS6;ARL6IP5;RPSA;RPL10A
1-Naphthyl_Isothiocyanate-60_mg/kg_in_Corn_Oil-Rat-Kidney-1d-up	11/332	0,009683962	0,057868654	0	0	2,277149836	10,55979082	EEF1B2;RPL32;RPS19;RPS5;RPS6;RPS20;RPSA;RPS3A;RAPGEF5;RPL10A;SEPP1
Clozapine-95_mg/kg_in_CMC-Rat-Heart-1d-dn	11/332	0,009683962	0,057824781	0	0	2,277149836	10,55979082	RPS17;RPL32;RPS19;RPS8;COL5A3;RPS6;RPS20;RPSA;RPS3A;RPL10A;RPL39
Hydroxyurea-59_mg/kg_in_Saline-Rat-Spleen-1d-dn	11/332	0,009683962	0,057780975	0	0	2,277149836	10,55979082	EEF1B2;RPS17;RPL32;RPS8;RPS5;RPS6;RPS20;RPSA;RPS3A;RPL10A;TPT1
Ibuprofen-275_mg/kg_in_Corn_Oil-Rat-Liver-3d-dn	11/332	0,009683962	0,057737234	0	0	2,277149836	10,55979082	EEF1B2;RPL32;RPS19;RPS8;RPS6;RPL35A;RPS20;RPSA;RPL10A;RPL39;TPT1
Sulindac-64_mg/kg_in_Corn_Oil-Rat-Kidney-5d-up	11/332	0,009683962	0,05769356	0	0	2,277149836	10,55979082	EEF1B2;RPS17;RPL32;RPS19;RPS8;RPS5;RPS6;RPL35A;RPS20;RPL10A;SEPP1
Troglitazone-100_mg/kg_in_Corn_Oil-Rat-Liver-1d-dn	11/332	0,009683962	0,057649952	0	0	2,277149836	10,55979082	EEF1B2;RPS17;RPL32;RPS8;RPS6;RPS20;RPSA;RPS3A;RPL10A;SEPP1;RPL39
Carboplatin-14_mg/kg_in_Saline-Rat-Bone_marrow-5d-up	9/244	0,009704064	0,057725987	0	0	2,535068447	11,75057594	C1QA;RPS19;ARL2BP;RPS8;RPS6;NKG7;RPS20;RPSA;TPT1
1-Naphthyl_Isothiocyanate-30_mg/kg_in_Corn_Oil-Rat-Kidney-1d-dn	10/288	0,00983936	0,05848664	0	0	2,386407026	11,02845701	EEF1B2;RPS17;RPL32;ARL2BP;CLP1;RPS8;RPS6;RPL35A;RPSA;RPS3A
Altretamine-40_mg/kg_in_Corn_Oil-Rat-Spleen-1d-dn	10/288	0,00983936	0,058442533	0	0	2,386407026	11,02845701	EEF1B2;C1QA;RPL32;RPS19;RPS5;RPS6;RPL13A;RPL35A;RPL39;TOMM20
Amitraz-75_mg/kg_in_CMC-Rat-Liver-3d-dn	10/288	0,00983936	0,058398492	0	0	2,386407026	11,02845701	C1QA;RPS17;RPL32;RPS19;CLP1;RPS8;ARL6IP5;RPL35A;RPS20;SEPP1
Amlodipine-19_mg/kg_in_Corn_Oil-Rat-Liver-1d-dn	10/288	0,00983936	0,058354517	0	0	2,386407026	11,02845701	RPS17;CLP1;RPS8;RPS6;RPS20;RPS3A;RPL10A;RPL39;SEPP1;TPT1
Loratadine-2000_mg/kg_in_Water-Rat-Heart-5d-up	10/288	0,00983936	0,058310608	0	0	2,386407026	11,02845701	C1QA;RPS17;RPL32;RPS19;RPS8;RPS6;RPS20;RPS3A;RPL39;TPT1
Nimesulide-30_mg/kg_in_Corn_Oil-Rat-Liver-1d-up	10/288	0,00983936	0,058266766	0	0	2,386407026	11,02845701	EEF1B2;RPS17;RPL32;RPS19;RPS5;RPS6;RPS20;RPSA;RPS3A;RPL10A
Procarbazine-54_mg/kg_in_Water-Rat-Liver-0.25d-dn	10/288	0,00983936	0,058222989	0	0	2,386407026	11,02845701	EEF1B2;RPS17;RPL32;RPS8;RPS5;RPL35A;RPSA;RPS3A;RPL39;TPT1
Sulbactam-671_mg/kg_in_Saline-Rat-Kidney-5d-up	10/288	0,00983936	0,058179278	0	0	2,386407026	11,02845701	EEF1B2;RPS17;RPL32;RPS19;ARL6IP5;RPL35A;RPSA;RPS3A;RAPGEF5;RPL39
Chlorpyrifos-82_mg/kg_in_CMC-Rat-Kidney-5d-dn	11/333	0,009889516	0,058431977	0	0	2,270311549	10,48039405	EEF1B2;RPS17;RPL32;RPS19;ARL2BP;RPS6;RPL35A;RPS20;RPSA;SEPP1;RPL39
Harringtonine-23_mg/kg_in_Saline-Rat-Bone_marrow-1d-up	11/333	0,009889516	0,058388175	0	0	2,270311549	10,48039405	EEF1B2;RPS17;RPL32;RPS19;RPS5;RPS6;RPL35A;RPSA;RPL10A;SEPP1;RPL39
1-Naphthyl_Isothiocyanate-30_mg/kg_in_Corn_Oil-Rat-Liver-0.25d-up	10/289	0,01006539	0,059382031	0	0	2,378149562	10,93628339	EEF1B2;RPS17;CXCL11;RPS19;RPS8;RPS6;RPS3A;RPL10A;RPL39;SEPP1
Acyclovir-980_mg/kg_in_Saline-Rat-Liver-0.25d-up	10/289	0,01006539	0,059337583	0	0	2,378149562	10,93628339	C1QA;RPS17;RPL32;RPS8;RPS6;RPL35A;RPS20;RPSA;RPS3A;RPL39
Carboplatin-14_mg/kg_in_Saline-Rat-Liver-1d-dn	10/289	0,01006539	0,059293202	0	0	2,378149562	10,93628339	EEF1B2;RPL32;RPS19;RPS8;RPS5;RPS6;RPSA;RPS3A;RPL10A;TPT1
Diethylstilbestrol-2.8_mg/kg_in_Corn_Oil-Rat-Bone_marrow-3d-dn	10/289	0,01006539	0,059248887	0	0	2,378149562	10,93628339	EEF1B2;RPS17;RPL32;RPS8;FEM1B;RPS6;ARL6IP5;RPS20;RPSA;RPL10A
Fludrocortisone_Acetate-125_mg/kg_in_CMC-Rat-Kidney-5d-up	10/289	0,01006539	0,059204638	0	0	2,378149562	10,93628339	EEF1B2;RPS19;RPS6;RPS20;RPSA;RPL10A;RPL39;GOLGA7;SEPP1;TPT1
Leflunomide-30_mg/kg_in_Corn_Oil-Rat-Bone_marrow-1d-dn	10/289	0,01006539	0,059160456	0	0	2,378149562	10,93628339	EEF1B2;RPL32;RPS8;FEM1B;RPS6;ARL6IP5;RPL35A;RPS20;RPSA;RPS3A
Promethazine-113_mg/kg_in_Saline-Rat-Liver-0.25d-up	10/289	0,01006539	0,059116339	0	0	2,378149562	10,93628339	RPL32;RPS19;RPS8;RPS5;RPS6;RPSA;RPS3A;RPL10A;SEPP1;TPT1
Tazobactam-1500_mg/kg_in_CMC-Rat-Spleen-5d-up	10/289	0,01006539	0,059072288	0	0	2,378149562	10,93628339	CXCL11;RPS19;RPS5;RPS6;RPS20;RPSA;RAPGEF5;RPL10A;RPL39;SEPP1
Cisplatin-1.17_mg/kg_in_Saline-Rat-Spleen-3d-dn	11/334	0,010098416	0,059221983	0	0	2,263514209	10,4017005	EEF1B2;C1QA;RPS17;RPL32;RPS8;RPS6;ARL6IP5;RPSA;RPS3A;RPL10A;TPT1
Sulindac-132_mg/kg_in_Corn_Oil-Rat-Heart-3d-up	11/334	0,010098416	0,059177919	0	0	2,263514209	10,4017005	EEF1B2;C1QA;RPS19;RPS8;RPS6;ARL6IP5;RPSA;RPS3A;RPL10A;RPL39;PDRG1
Cycloheximide-0.25_mg/kg_in_Water-Rat-Liver-5d-up	12/380	0,010099502	0,059140281	0	0	2,17037439	9,973454497	EEF1B2;RPS17;RPL32;RPS19;ARL2BP;RPS8;RPS5;RPS6;RPS20;RPSA;RPS3A;RPL10A
Buspirone-196_mg/kg_in_CMC-Rat-Heart-3d-up	10/290	0,010295359	0,060242385	0	0	2,369949046	10,84503385	RPS17;RPL32;RPS19;RPS5;RPS6;ARL6IP5;RPL35A;RPSA;RPS3A;TPT1
Cortisone-206_mg/kg_in_CMC-Rat-Kidney-5d-up	10/290	0,010295359	0,060197662	0	0	2,369949046	10,84503385	EEF1B2;RPS17;RPS19;ARL2BP;CLP1;RPS8;RPS20;RPSA;RPS3A;TPT1

Doxazosin-500_mg/kg_in_Water-Rat-Heart-3d-dn	10/290	0,010295359	0,060153005	0	0	2,369949046	10,84503385	EEF1B2;RPS17;RPL32;RPS19;RPS8;COL5A3;ARL6IP5;RPSA;RPL39;TPT1
Lamotrigine-26_mg/kg_in_CMC-Rat-Kidney-3d-dn	10/290	0,010295359	0,060108414	0	0	2,369949046	10,84503385	RPL32;RPS19;ARL2BP;RPS6;RPS20;RPSA;RPL10A;RPL39;SEPP1;PDRG1
Lead(IV)_Acetate-200_mg/kg_in_Saline-Rat-Spleen-1d-dn	10/290	0,010295359	0,060063889	0	0	2,369949046	10,84503385	EEF1B2;CXCL11;ARL2BP;RPS8;RPS5;FUNDC1;RPL13A;RPL35A;RPSA;TPT1
Nystatin-134_mg/kg_in_Corn_Oil-Rat-Liver-5d-up	10/290	0,010295359	0,06001943	0	0	2,369949046	10,84503385	EEF1B2;C1QA;RPL32;RPS19;RPS8;RPS5;RPS3A;RPL10A;SEPP1;TPT1
Amitraz-75_mg/kg_in_CMC-Rat-Heart-3d-dn	11/335	0,010310697	0,060064389	0	0	2,25675745	10,32370245	C1QA;RPS17;RPL32;RPS8;COL5A3;ARL6IP5;RPS20;RPSA;RPS3A;RPL39;TPT1
Azathioprine-20_mg/kg_in_Corn_Oil-Rat-Spleen-0.25d-up	11/335	0,010310697	0,060019995	0	0	2,25675745	10,32370245	EEF1B2;RPL32;RPS19;RPS5;RPS6;RPL13A;RPL35A;RPSA;RPL10A;SEPP1;RPL39
Valacyclovir-88_mg/kg_in_CMC-Rat-Kidney-5d-up	11/335	0,010310697	0,059975667	0	0	2,25675745	10,32370245	RPS17;RPL32;RPS19;FEM1B;ARL6IP5;RPS20;RPSA;SEPP1;RPL39;GOLGA7;TPT1
Hydroxyurea-59_mg/kg_in_Saline-Rat-Liver-0.25d-up	9/247	0,010455795	0,060774793	0	0	2,504278142	11,42100805	EEF1B2;RPS17;RPS19;RPS8;RPS6;ARL6IP5;RPS20;RPSA;RPS3A
Busulfan-500_uM_in_DMSO-Rat-Primary_rat_hepatocytes-1d-up	11/336	0,010526395	0,061140037	0	0	2,25004091	10,2463923	EEF1B2;RPS17;CXCL11;RPL32;RPS19;ARL2BP;RPS6;ARL6IP5;RPS3A;RPL39;SLC16A3
Doxorubicin-4_mg/kg_in_Saline-Rat-Heart-28d-up	11/336	0,010526395	0,061094981	0	0	2,25004091	10,2463923	EEF1B2;RPS17;RPL32;RPS19;RPS6;RPL35A;RPS20;RPSA;RPS3A;RPL10A;RPL39
Clopidogrel-400_mg/kg_in_Corn_Oil-Rat-Bone_marrow-3d-dn	10/291	0,010529311	0,061066901	0	0	2,361804891	10,75469683	EEF1B2;RPS17;RPS19;FEM1B;RPSA;RPS3A;RPL10A;RPL39;SEPP1;TPT1
Daunorubicin-3.25_mg/kg_in_Saline-Rat-Liver-3d-dn	10/291	0,010529311	0,061021966	0	0	2,361804891	10,75469683	EEF1B2;RPS17;RPL32;RPS19;RPS6;RPL35A;RPSA;RPS3A;RPL10A;RPL39
Famciclovir-112_mg/kg_in_Saline-Rat-Bone_marrow-1d-dn	10/291	0,010529311	0,060977097	0	0	2,361804891	10,75469683	C1QA;RPS17;RPL32;RPS8;FEM1B;RPS20;RPS3A;RPL10A;RPL39;TPT1
Fluvastatin-94_mg/kg_in_Corn_Oil-Rat-Kidney-1d-dn	10/291	0,010529311	0,060932294	0	0	2,361804891	10,75469683	EEF1B2;RPS17;RPL32;RPS19;RPS8;RPL35A;RPSA;RPS3A;RPL10A;RPL39
Iproniazid-46_mg/kg_in_Water-Rat-Heart-3d-dn	10/291	0,010529311	0,060887556	0	0	2,361804891	10,75469683	RPS17;CXCL11;RPL32;RPS19;RPS8;RPS6;RPSA;RPS3A;RPL39;PDRG1
Mitotane-5000_mg/kg_in_Water-Rat-Bone_marrow-3d-dn	10/291	0,010529311	0,060842884	0	0	2,361804891	10,75469683	EEF1B2;RPS17;RPL32;ARL2BP;RPS8;RPS5;RPS20;RPSA;RPS3A;TPT1
Niacinamide-750_mg/kg_in_Water-Rat-Liver-3d-dn	10/291	0,010529311	0,060798278	0	0	2,361804891	10,75469683	EEF1B2;RPS17;RPL32;RPS19;CLP1;RPS8;RPS20;RPL10A;RPL39;SEPP1
Phenylbutazone-368_mg/kg_in_CMC-Rat-Kidney-1d-up	10/291	0,010529311	0,060753737	0	0	2,361804891	10,75469683	RPS17;RPL32;RPS19;RPS8;FEM1B;RPS6;RPSA;RPL10A;GOLGA7;SEPP1
Tamoxifen-2.5_mg/kg_in_Corn_Oil-Rat-Liver-5d-up	10/291	0,010529311	0,060709262	0	0	2,361804891	10,75469683	RPS17;RPL32;RPS19;RPS8;RPS6;RPL35A;RPS20;RPS3A;RPL39;TPT1
Thioguanine-12_mg/kg_in_Corn_Oil-Rat-Bone_marrow-1d-dn	10/291	0,010529311	0,060664851	0	0	2,361804891	10,75469683	EEF1B2;RPL32;RPS19;RPS8;FUNDC1;RPS6;ARL6IP5;RPSA;RPS3A;RPL39
Diethanolamine-620_mg/kg_in_Water-Rat-Kidney-5d-dn	9/248	0,010715858	0,061694518	0	0	2,494180246	11,31367776	RPS17;RPL32;ARL2BP;RPS8;RPS6;RPL35A;RPS20;RPSA;RPS3A
Lithocholic_Acid-74_uM_in_DMSO-Rat-Primary_rat_hepatocytes-1d-up	9/248	0,010715858	0,061649453	0	0	2,494180246	11,31367776	EEF1B2;RPS17;RPL32;CLP1;RPS8;RPS5;ARL6IP5;RPS20;PDRG1
Sulfadiazine-1170_mg/kg_in_Water-Rat-Kidney-1d-dn	9/248	0,010715858	0,061604453	0	0	2,494180246	11,31367776	RPS17;RPL32;RPS19;RPS6;RPL35A;RPS20;RPSA;RPS3A;RPL39
Valproic_Acid-1500_mg/kg_in_Water-Rat-Liver-5d-dn	9/248	0,010715858	0,061559519	0	0	2,494180246	11,31367776	EEF1B2;C1QA;RPS17;RPL32;RPS19;RPL35A;RPS20;RPL39;SEPP1
Catechol-195_mg/kg_in_Saline-Rat-Bone_marrow-3d-up	11/337	0,010745544	0,061685063	0	0	2,243364231	10,16976255	C1QA;RPS17;RPL32;RPS19;RPS8;FEM1B;RPS6;RPL35A;RPSA;RPS3A;RPL39
Chlorpropamide-269_mg/kg_in_CMC-Rat-Heart-1d-dn	11/337	0,010745544	0,061640135	0	0	2,243364231	10,16976255	EEF1B2;RPS17;RPL32;RPS19;RPS8;RPS5;RPS6;RPS20;RPSA;RPL10A;RPL39
Choline_Chloride-2550_mg/kg_in_Water-Rat-Liver-0.25d-up	11/337	0,010745544	0,061595274	0	0	2,243364231	10,16976255	RPS17;RPL32;RPS19;RPS8;RPS6;ARL6IP5;RPS20;RPSA;RPS3A;RPL10A;SEPP1
Cisplatin-2_mg/kg_in_Saline-Rat-Kidney-3d-up	11/337	0,010745544	0,061550477	0	0	2,243364231	10,16976255	RPS17;CXCL11;RPS19;ARL2BP;CLP1;RPS8;RPS6;RPS20;RPSA;RPS3A;TPT1
Streptozotocin-3000_uM_in_DMSO-Rat-Primary_rat_hepatocytes-1d-up	6/127	0,010760208	0,061589679	0	0	3,247016803	14,71515672	EEF1B2;CLP1;RPS6;RPS20;GOLGA7;PDRG1
Cephalexin-2500_mg/kg_in_CMC-Rat-Kidney-1d-dn	10/292	0,010767287	0,06158544	0	0	2,353716518	10,66526091	RPL32;ARL2BP;CLP1;RPS8;RPS6;RPL35A;RPS20;RPS3A;SEPP1;TPT1
Diclofenac-10_mg/kg_in_Corn_Oil-Rat-Liver-1d-dn	10/292	0,010767287	0,061540748	0	0	2,353716518	10,66526091	EEF1B2;RPS17;RPL32;RPS19;RPS8;RPS5;RPSA;RPS3A;SEPP1;TPT1
Diclofenac-10_mg/kg_in_Corn_Oil-Rat-Liver-3d-dn	10/292	0,010767287	0,061496121	0	0	2,353716518	10,66526091	RPS17;RPL32;RPS19;CLP1;RPS8;RPS5;RPL35A;RPSA;SEPP1;TPT1
Diethylstilbestrol-2.8_mg/kg_in_Corn_Oil-Rat-Bone_marrow-0.25d-dn	10/292	0,010767287	0,061451558	0	0	2,353716518	10,66526091	RPS17;RPL32;RPS19;CLP1;RPS8;FEM1B;RPS6;NKG7;RPS3A;TPT1
Digoxin-11_mg/kg_in_Corn_Oil-Rat-Heart-1d-dn	10/292	0,010767287	0,06140706	0	0	2,353716518	10,66526091	EEF1B2;RPL32;RPS8;RPS6;RPL35A;RPS20;RPL10A;RPL39;TPT1;PDRG1
Gentamicin-267_mg/kg_in_Corn_Oil-Rat-Liver-3d-dn	10/292	0,010767287	0,061362627	0	0	2,353716518	10,66526091	EEF1B2;RPS17;RPL32;RPS19;RPS8;RPS6;RPS20;RPS3A;RPL39;TPT1
Ibuprofen-263_mg/kg_in_Corn_Oil-Rat-Kidney-3d-up	10/292	0,010767287	0,061318258	0	0	2,353716518	10,66526091	C1QA;CXCL11;RPL32;RPS19;RPS8;RPS5;RPS6;RPS20;RPS3A;RPL10A
Mitomycin_C-0.5_mg/kg_in_Saline-Rat-Spleen-1d-dn	10/292	0,010767287	0,061273952	0	0	2,353716518	10,66526091	EEF1B2;RPS17;RPS8;RPS5;FUNDC1;RPL13A;RPL35A;RPSA;RPS3A;TPT1
Sulpiride-1000_mg/kg_in_CMC-Rat-Heart-5d-up	10/292	0,010767287	0,061229711	0	0	2,353716518	10,66526091	RPS17;RPL32;COL5A3;RPS6;RPSA;RPS3A;RAPGEF5;RPL10A;RPL39;TPT1
Busulfan-9_mg/kg_in_Corn_Oil-Rat-Spleen-1d-up	11/338	0,010968179	0,062327111	0	0	2,236727058	10,09380579	C1QA;RPL32;ARL2BP;CLP1;RPS8;RPS6;NKG7;RPS20;RPSA;RPL39;TPT1
Ethylene_Glycol-3525_mg/kg_in_Water-Rat-Liver-3d-up	11/338	0,010968179	0,062282174	0	0	2,236727058	10,09380579	RPS17;CXCL11;RPS19;RPS8;FUNDC1;RPS6;RPS20;RPS3A;RPL10A;RPL39;TPT1
Sulindac-64_mg/kg_in_Corn_Oil-Rat-Kidney-1d-up	11/338	0,010968179	0,062237302	0	0	2,236727058	10,09380579	EEF1B2;RPS17;RPL32;RPS19;RPS5;RPS6;RPS20;RPS3A;RPL10A;RPL39;TPT1
Cyclophosphamide-25_mg/kg_in_Water-Rat-Heart-5d-up	10/293	0,01100933	0,062425833	0	0	2,345683356	10,57671487	EEF1B2;RPS17;CXCL11;RPL32;RPS19;ARL2BP;RPS6;RPS3A;RPL10A;SEPP1
Hydrocortisone-56_mg/kg_in_Corn_Oil-Rat-Liver-3d-dn	10/293	0,01100933	0,062380922	0	0	2,345683356	10,57671487	C1QA;RPS17;RPL32;RPS19;RPS8;FEM1B;RPS6;RPS3A;RPL10A;RPL39
Idarubicin-4_mg/kg_in_Saline-Rat-Heart-28d-up	10/293	0,01100933	0,062336076	0	0	2,345683356	10,57671487	EEF1B2;C1QA;RPS19;RPS6;RPS20;RPSA;RPL10A;SEPP1;RPL39;GOLGA7
Rapamycin-60_mg/kg_in_CMC-Rat-Kidney-5d-up	13/432	0,011049872	0,062520685	0	0	2,068219422	9,31802427	RPL32;RPS8;RPS6;RPL35A;RPSA;RPS3A;EEF1B2;RPS17;RPS19;ARL2BP;RPS20;RPL39;TPT1
Cisplatin-2_mg/kg_in_Saline-Rat-Spleen-5d-dn	11/339	0,011194334	0,063292586	0	0	2,230129043	10,01851474	EEF1B2;C1QA;RPS17;RPL32;RPS19;RPS8;RPS6;RPS20;RPSA;RPS3A;RPL10A
Indomethacin-5_mg/kg_in_Corn_Oil-Rat-Liver-1d-dn	11/339	0,011194334	0,063247183	0	0	2,230129043	10,01851474	EEF1B2;RPS17;RPL32;RPS6;RPL35A;RPS20;RPS3A;RPL10A;RPL39;GOLGA7;TPT1
BW-723C86-10_mg/kg_in_Saline-Rat-Heart-5d-dn	10/294	0,011255483	0,063547084	0	0	2,337704841	10,48904766	RPS17;RPL32;RPS8;COL5A3;RPS5;RPS6;ARL6IP5;RPL35A;RPS3A;TPT1
Beta-Estradiol-0.3_mg/kg_in_Corn_Oil-Rat-Liver-5d-up	10/294	0,011255483	0,063501563	0	0	2,337704841	10,48904766	EEF1B2;RPS17;RPL32;RPS19;RPS5;RPS6;RPL35A;RPSA;RPL10A;RPL39
Carmustine-4_mg/kg_in_Corn_Oil-Rat-Spleen-1d-dn	10/294	0,011255483	0,063456107	0	0	2,337704841	10,48904766	EEF1B2;RPS17;RPS19;RPS8;RPS5;RPL13A;RPL35A;RPS20;RPSA;TOMM20
Digoxin-11_mg/kg_in_Corn_Oil-Rat-Liver-0.25d-dn	10/294	0,011255483	0,063410717	0	0	2,337704841	10,48904766	EEF1B2;RPS17;RPL32;RPS19;RPS8;RPS6;RPL35A;RPS20;RPS3A;TPT1
Methotrexate-0.3_mg/kg_in_Corn_Oil-Rat-Bone_marrow-1d-dn	10/294	0,011255483	0,063365391	0	0	2,337704841	10,48904766	EEF1B2;RPL32;RPS19;RPS8;FEM1B;RPS6;ARL6IP5;RPL35A;RPS20;RPSA
Mitomycin_C-1.7_mg/kg_in_Saline-Rat-Spleen-1d-dn	10/294	0,011255483	0,06332013	0	0	2,337704841	10,48904766	EEF1B2;RPS17;RPS8;RPS5;FUNDC1;RPL13A;RPL35A;RPSA;GOLGA7;TPT1
Noscapine-570_mg/kg_in_CMC-Rat-Bone_marrow-5d-dn	10/294	0,011255483	0,063274934	0	0	2,337704841	10,48904766	RPS17;RPS19;RPS8;FEM1B;RPS5;RPSA;RPL10A;SEPP1;GOLGA7;TPT1
Progesterone-11.3_mg/kg_in_Corn_Oil-Rat-Liver-3d-up	10/294	0,011255483	0,063229802	0	0	2,337704841	10,48904766	RPL32;RPS19;RPS8;RPS6;RPS20;RPS3A;RPL10A;SEPP1;RPL39;TPT1
Warfarin-0.25_mg/kg_in_CMC-Rat-Liver-5d-up	10/294	0,011255483	0,063184734	0	0	2,337704841	10,48904766	EEF1B2;RPS17;RPL32;RPS19;ARL6IP5;RPS20;RPSA;RAPGEF5;SEPP1;RPL39
Acyclovir-330_mg/kg_in_Saline-Rat-Bone_marrow-1d-up	12/386	0,011328681	0,063550349	0	0	2,136638	9,573030588	EEF1B2;C1QA;RPL32;RPS8;FEM1B;RPS5;RPS6;RPL13A;RPL35A;RPSA;TOMM20;TPT1
Cytarabine-487_mg/kg_in_Saline-Rat-Liver-3d-up	12/386	0,011328681	0,063505118	0	0	2,136638	9,573030588	EEF1B2;C1QA;RPS17;RPL32;RPS19;RPS8;RPS5;RPS6;RPS3A;RPL10A;RPL39;TPT1
Amikacin-82_mg/kg_in_Saline-Rat-Kidney-1d-up	11/340	0,011424044	0,063994148	0	0	2,22356984	9,943882171	EEF1B2;RPS17;RPL32;ARL2BP;RPS8;RPS6;RPSA;RPS3A;RPL10A;SEPP1;TPT1
Interleukin_1_Beta-0.01_mg/kg_in_RSA/Saline-Rat-Liver-0.25d-up	11/340	0,011424044	0,063948665	0	0	2,22356984	9,943882171	C1QA;RPS17;RPS19;RPS8;RPS6;RPS20;RPS3A;RPL10A;RPL39;GOLGA7;TPT1
Altretamine-13_mg/kg_in_Corn_Oil-Rat-Bone_marrow-3d-dn	10/295	0,011505788	0,064360502	0	0	2,329780418	10,40224839	EEF1B2;RPS17;RPL32;RPS8;ARL6IP5;NKG7;RPS20;RPSA;RPS3A;TPT1
Aspirin-375_mg/kg_in_Corn_Oil-Rat-Liver-1d-up	10/295	0,011505788	0,064314823	0	0	2,329780418	10,40224839	EEF1B2;RPS17;RPL32;RPS19;RPS8;RPS6;RPS20;RPSA;RPS3A;SEPP1
Atorvastatin-2.5_mg/kg_in_Corn_Oil-Rat-Kidney-1d-dn	10/295	0,011505788	0,06426921	0	0	2,329780418	10,40224839	EEF1B2;RPS17;RPL32;RPS8;RPSA;RPS3A;RPL10A;RPL39;TOMM20;TPT1
Carboplatin-6_mg/kg_in_Saline-Rat-Bone_marrow-1d-up	10/295	0,011505788	0,064223661	0	0	2,329780418	10,40224839	C1QA;RPS17;RPS19;ARL2BP;RPS8;FEM1B;RPS5;RPL35A;RPS20;TPT1
Cholecalciferol-8_mg/kg_in_CMC-Rat-Kidney-1d-dn	10/295	0,011505788	0,064178177	0	0	2,329780418	10,40224839	RPS19;ARL2BP;RPS8;FUNDC1;RPS6;RPS20;RPS3A;RPL10A;SEPP1;RPL39
Cisplatin-1.17_mg/kg_in_Saline-Rat-Liver-3d-up	10/295	0,011505788	0,064132757	0	0	2,329780418	10,40224839	EEF1B2;RPS17;RPS19;RPS8;RPS6;RPS20;RPSA;RPL10A;RPL39;TPT1
Diazepam-710_mg/kg_in_CMC-Rat-Liver-3d-dn	10/295	0,011505788	0,064087402	0	0	2,329780418	10,40224839	EEF1B2;C1QA;RPS17;RPL32;CLP1;ARL6IP5;RPL35A;RPS3A;SEPP1;TPT1
NN-Dimethylformamide-1400_mg/kg_in_Saline-Rat-Spleen-5d-up	10/295	0,011505788	0,06404211	0	0	2,329780418	10,40224839	RPS17;CXCL11;RPL32;RPS8;RPS5;FUNDC1;RPL35A;RPS3A;RPL39;TPT1
Ofloxacin-30_mg/kg_in_Water-Rat-Heart-5d-up	10/295	0,011505788	0,063996883	0	0	2,329780418	10,40224839	RPS17;RPL32;ARL2BP;RPS6;ARL6IP5;RPS20;RPS3A;RAPGEF5;GOLGA7;TPT1
Vincristine-0.05_mg/kg_in_Saline-Rat-Bone_marrow-5d-up	10/295	0,011505788	0,063951719	0	0	2,329780418	10,40224839	C1QA;RPL32;RPS19;ARL2BP;RPS8;RPS6;NKG7;RPS20;RPSA;RPS3A
Sulfaphenazole-1695_mg/kg_in_Water-Rat-Kidney-5d-dn	9/251	0,011525254	0,064014739	0	0	2,464369327	10,99900931	RPS17;RPL32;RPS19;RPS6;RPL35A;RPS20;RPSA;RPS3A;RPL39
Azlocillin-800_mg/kg_in_Saline-Rat-Kidney-1d-up	12/387	0,011544301	0,064075347	0	0	2,131116972	9,508113392	RPS17;RPL32;RPS8;RPS5;RPS6;RPL13A;RPL35A;RPS20;RPSA;RPS3A;SEPP1;RPL39
Trimethadione-535_mg/kg_in_Water-Rat-Kidney-1d-dn	8/209	0,011707237	0,064933942	0	0	2,630756836	11,70041755	RPS17;RPL32;RPS19;RPS6;RPS20;RPSA;RPS3A;RPL39
Acetaminophen-486_mg/kg_in_Corn_Oil-Rat-Liver-0.25d-up	10/296	0,011760288	0,065182287	0	0	2,321909538	10,31630637	EEF1B2;RPS17;RPL32;RPS19;RPS8;RPS6;RPS3A;RPL10A;SEPP1;PDRG1
Baclofen-6.6_mg/kg_in_CMC-Rat-Heart-3d-dn	10/296	0,011760288	0,065136449	0	0	2,321909538	10,31630637	EEF1B2;RPS17;RPS8;ARL6IP5;RPS20;RPSA;RPS3A;RPL10A;SEPP1;TOMM20
Chlorpyrifos-82_mg/kg_in_CMC-Rat-Heart-3d-dn	10/296	0,011760288	0,065090675	0	0	2,321909538	10,31630637	RPS17;RPL32;RPS19;ARL2BP;RPS6;RPS20;RPS3A;RPL10A;RPL39;TPT1
Diisopropyl_Fluorophosphate-3.75_mg/kg_in_Water-Rat-Heart-1d-dn	10/296	0,011760288	0,065044965	0	0	2,321909538	10,31630637	EEF1B2;RPS17;RPL32;RPS19;FUNDC1;RPS6;RPS20;RPSA;RPL10A;RPL39
Epalrestat-2500_mg/kg_in_CMC-Rat-Liver-3d-up	10/296	0,011760288	0,064999319	0	0	2,321909538	10,31630637	RPL32;RPS19;RPS8;RPS5;RPS6;RPS20;RPSA;RPS3A;RPL10A;RPL39
Epirubicin-2.7_mg/kg_in_Saline-Rat-Liver-5d-dn	10/296	0,011760288	0,064953738	0	0	2,321909538	10,31630637	EEF1B2;RPS17;RPL32;RPS19;RPS8;RPS6;RPS20;RPS3A;RPL10A;RPL39
Etoposide-188_mg/kg_in_Corn_Oil-Rat-Liver-5d-dn	10/296	0,011760288	0,06490822	0	0	2,321909538	10,31630637	RPS17;RPL32;RPS8;RPS5;RPL35A;RPSA;RPS3A;RPL10A;RPL39;TPT1
Fenbendazole-375_mg/kg_in_Corn_Oil-Rat-Liver-3d-dn	10/296	0,011760288	0,064862766	0	0	2,321909538	10,31630637	EEF1B2;RPS17;RPL32;RPS8;RPL35A;RPS20;RPS3A;SEPP1;RPL39;TPT1
Levamisole-120_mg/kg_in_Water-Rat-Liver-1d-up	10/296	0,011760288	0,064817376	0	0	2,321909538	10,31630637	RPS17;RPL32;RPS8;RPS5;RPL35A;RPSA;RPS3A;RPL10A;RPL39;TPT1
Levofloxacin-739_mg/kg_in_Water-Rat-Heart-5d-up	10/296	0,011760288	0,064772049	0	0	2,321909538	10,31630637	EEF1B2;RPS17;RPL32;ARL2BP;RPL35A;RPS3A;RAPGEF5;SEPP1;GOLGA7;TPT1
Mifepristone-3_mg/kg_in_Corn_Oil-Rat-Liver-1d-dn	10/296	0,011760288	0,064726786	0	0	2,321909538	10,31630637	EEF1B2;C1QA;RPS17;RPL32;RPS8;RPS20;RPSA;RPS3A;SEPP1;TPT1
Penicillamine-852_mg/kg_in_Water-Rat-Liver-5d-up	10/296	0,011760288	0,064681585	0	0	2,321909538	10,31630637	EEF1B2;RPS17;CLP1;RPS8;RPS5;RPS6;RPL35A;RPS3A;SEPP1;GOLGA7
Phenacetin-619_mg/kg_in_Corn_Oil-Rat-Liver-0.25d-up	10/296	0,011760288	0,064636448	0	0	2,321909538	10,31630637	RPS17;RPL32;RPS19;CLP1;RPS8;RPS6;ARL6IP5;RPL35A;RPSA;SEPP1
Sertraline-210_mg/kg_in_Corn_Oil-Rat-Liver-5d-dn	10/296	0,011760288	0,064591374	0	0	2,321909538	10,31630637	EEF1B2;RPS17;RPL32;RPS19;CLP1;RPS6;RPL35A;RPS3A;RPL10A;TPT1
Simvastatin-1200_mg/kg_in_Corn_Oil-Rat-Kidney-3d-up	10/296	0,011760288	0,064546362	0	0	2,321909538	10,31630637	RPS17;RPL32;RPS8;RPS5;ARL6IP5;RPL35A;RPS20;RPSA;RPS3A;SEPP1
Sparteine-33_mg/kg_in_Water-Rat-Heart-0.25d-up	10/296	0,011760288	0,064501414	0	0	2,321909538	10,31630637	EEF1B2;RPS17;RPL32;RPS19;RPS6;RPL35A;RPS20;RPS3A;RPL39;TPT1
Sulindac-132_mg/kg_in_Corn_Oil-Rat-Liver-1d-up	10/296	0,011760288	0,064456528	0	0	2,321909538	10,31630637	EEF1B2;RPS17;RPL32;RPS19;RPS6;RPS20;RPSA;RPS3A;RPL10A;RPL39

Warfarin-0.25_mg/kg_in_CMC-Rat-Liver-1d-up	10/296	0,011760288	0,064411704	0	0	2,321909538	10,31630637	EEF1B2;RPS17;RPL32;RPS6;ARL6IP5;RPS20;RPSA;RPS3A;SEPP1;TPT1
Carboplatin-6_mg/kg_in_Saline-Rat-Kidney-1d-dn	9/252	0,011804966	0,064611474	0	0	2,454590083	10,8965022	EEF1B2;RPS17;RPL32;RPS19;RPS5;RPL35A;RPS3A;TPT1;PDRG1
Acetazolamide-47_mg/kg_in_Corn_Oil-Rat-Liver-5d-dn	10/297	0,012019026	0,065737396	0	0	2,314091661	10,23121102	EEF1B2;RPS17;RPL32;RPS19;RPS6;RPL35A;RPS3A;RPL10A;SEPP1;RPL39
Azathioprine-20_mg/kg_in_Corn_Oil-Rat-Bone_marrow-0.25d-dn	10/297	0,012019026	0,065691776	0	0	2,314091661	10,23121102	RPS17;RPL32;RPS19;RPS8;FEM1B;RPS5;RPS6;ARL6IP5;RPS20;RPSA
Benzothiazyl_Disulfide-2600_mg/kg_in_Corn_Oil-Rat-Kidney-3d-up	10/297	0,012019026	0,06564622	0	0	2,314091661	10,23121102	RPS17;CXCL11;RPL32;ARL2BP;CLP1;RPS8;RPS6;RPS20;RPS3A;TPT1
Busulfan-500_uM_in_DMSO-Rat-Primary_rat_hepatocytes-0.67d-up	10/297	0,012019026	0,065600727	0	0	2,314091661	10,23121102	EEF1B2;RPS17;CXCL11;RPL32;ARL2BP;FEM1B;RPS6;ARL6IP5;RPL39;GOLGA7
Cyclophosphamide-1320_uM_in_DMSO-Rat-Primary_rat_hepatocytes-0.67d-up	10/297	0,012019026	0,065555297	0	0	2,314091661	10,23121102	EEF1B2;RPL32;RPS19;ARL2BP;CLP1;RPS8;FEM1B;RPS5;RPS6;PDRG1
Diethylstilbestrol-2.8_mg/kg_in_Corn_Oil-Rat-Kidney-0.25d-dn	10/297	0,012019026	0,065509931	0	0	2,314091661	10,23121102	EEF1B2;RPS17;RPL32;RPS19;RPS8;RPS6;ARL6IP5;RPS20;RPSA;RPS3A
Ethambutol-998_mg/kg_in_CMC-Rat-Kidney-1d-up	10/297	0,012019026	0,065464626	0	0	2,314091661	10,23121102	EEF1B2;RPS17;RPL32;RPS19;RPS8;ARL6IP5;RPL13A;RPL35A;RPS20;RPS3A
Irinotecan-5_mg/kg_in_Saline-Rat-Bone_marrow-5d-up	10/297	0,012019026	0,065419385	0	0	2,314091661	10,23121102	C1QA;RPL32;RPS19;RPS8;RPS6;NKG7;RPS20;RPSA;RPL10A;TPT1
Lacidipine-1_mg/kg_in_Corn_Oil-Rat-Heart-0.25d-dn	10/297	0,012019026	0,065374206	0	0	2,314091661	10,23121102	RPS17;RPL32;ARL2BP;RPS8;FUNDC1;RPS6;ARL6IP5;RPL13A;RPS3A;SEPP1
Progesterone-148_uM_in_DMSO-Rat-Primary_rat_hepatocytes-0.67d-up	10/297	0,012019026	0,065329089	0	0	2,314091661	10,23121102	EEF1B2;RPL32;RPS19;RPS6;RPL35A;RPSA;RPS3A;RPL10A;RPL39;TOMM20
Tegafur-175_mg/kg_in_Water-Rat-Bone_marrow-5d-up	10/297	0,012019026	0,065284034	0	0	2,314091661	10,23121102	EEF1B2;C1QA;RPL32;ARL2BP;RPS8;RPS6;ARL6IP5;RPS3A;RPL39;TPT1
Vancomycin-160_mg/kg_in_Saline-Rat-Kidney-3d-up	10/297	0,012019026	0,065239042	0	0	2,314091661	10,23121102	RPS17;RPL32;RPS19;RPS8;RPS5;RPS6;RPL13A;RPL35A;RPSA;SEPP1
Verapamil-108_mg/kg_in_Water-Rat-Heart-5d-up	10/297	0,012019026	0,065194111	0	0	2,314091661	10,23121102	RPS17;RPL32;RPS19;RPS5;RPS6;RPSA;RAPGEF5;RPL10A;RPL39;TPT1
Vinorelbine-1.5_mg/kg_in_Saline-Rat-Liver-3d-up	10/297	0,012019026	0,065149243	0	0	2,314091661	10,23121102	EEF1B2;RPS17;RPL32;RPS19;ARL6IP5;RPS20;RPSA;RPS3A;RPL10A;SEPP1
Zaleplon-100_mg/kg_in_Water-Rat-Liver-5d-dn	10/297	0,012019026	0,065104436	0	0	2,314091661	10,23121102	EEF1B2;RPL32;RPS19;CLP1;RPS8;RPS6;RPL35A;RPS3A;RPL10A;TPT1
Famciclovir-1200_mg/kg_in_Saline-Rat-Spleen-3d-dn	9/253	0,012089724	0,06544238	0	0	2,444888146	10,79515783	EEF1B2;C1QA;RPL32;ARL2BP;RPS8;FUNDC1;RPS6;ARL6IP5;RPSA
Doxorubicin-3_mg/kg_in_Saline-Rat-Kidney-1d-up	11/343	0,012134856	0,065641572	0	0	2,204121708	9,72386483	EEF1B2;RPS17;RPL32;RPS19;RPS8;RPS6;RPSA;RPS3A;RPL10A;RPL39;TPT1
Atorvastatin-2.5_mg/kg_in_Corn_Oil-Rat-Liver-5d-up	10/298	0,012282044	0,066392162	0	0	2,306326253	10,14695198	EEF1B2;RPS17;RPL32;RPS19;RPS20;RPSA;RPS3A;RPL10A;RPL39;TPT1
Azathioprine-20_mg/kg_in_Corn_Oil-Rat-Bone_marrow-3d-up	10/298	0,012282044	0,066346625	0	0	2,306326253	10,14695198	EEF1B2;C1QA;CLP1;RPS5;NKG7;RPL35A;RPS20;SEPP1;RPL39;GOLGA7
Bezafibrate-617_mg/kg_in_Corn_Oil-Rat-Liver-0.25d-up	10/298	0,012282044	0,066301151	0	0	2,306326253	10,14695198	EEF1B2;C1QA;RPS17;RPL32;RPS19;RPS6;RPS3A;RPL10A;RPL39;TPT1
Bromhexine-1200_mg/kg_in_CMC-Rat-Liver-3d-up	10/298	0,012282044	0,06625574	0	0	2,306326253	10,14695198	RPS17;RPL32;RPS19;RPS8;RPS5;RPS6;RPS20;RPS3A;RPL10A;SEPP1
Carmustine-16_mg/kg_in_Corn_Oil-Rat-Bone_marrow-1d-up	10/298	0,012282044	0,06621039	0	0	2,306326253	10,14695198	EEF1B2;C1QA;RPS17;COL5A3;RPS5;NKG7;RPL35A;RPL10A;RPL39;DUSP14
Ethylene_Glycol-3525_mg/kg_in_Water-Rat-Liver-1d-up	10/298	0,012282044	0,066165102	0	0	2,306326253	10,14695198	C1QA;RPS17;CXCL11;RPS8;FUNDC1;RPL35A;RPS20;RPSA;RPS3A;TPT1
Etodolac-24_mg/kg_in_CMC-Rat-Liver-5d-up	10/298	0,012282044	0,066119877	0	0	2,306326253	10,14695198	EEF1B2;C1QA;RPS17;RPS19;ARL2BP;RPS6;RPSA;RPS3A;RPL10A;RPL39
Fenoprofen-52_mg/kg_in_Corn_Oil-Rat-Kidney-1d-up	10/298	0,012282044	0,066074713	0	0	2,306326253	10,14695198	RPL32;RPS19;RPS8;RPS6;RPS20;RPSA;RPS3A;RPL10A;RPL39;PDRG1
Gentamicin-267_mg/kg_in_Corn_Oil-Rat-Liver-5d-dn	10/298	0,012282044	0,066029611	0	0	2,306326253	10,14695198	EEF1B2;RPS17;RPL32;RPS19;RPS8;RPS6;RPS20;RPS3A;RPL39;TPT1
Oxaprozin-1500_mg/kg_in_CMC-Rat-Kidney-3d-up	10/298	0,012282044	0,06598457	0	0	2,306326253	10,14695198	RPS17;RPL32;RPS19;RPS8;RPS6;RPS20;RPS3A;RAPGEF5;RPL10A;RPL39
Diclofenac-3.5_mg/kg_in_Corn_Oil-Rat-Kidney-3d-up	11/344	0,012379137	0,066460862	0	0	2,197714377	9,6517961	C1QA;RPL32;ARL2BP;RPS8;RPS5;RPL35A;RPS20;RPSA;RPS3A;TOMM20;TPT1
Gentamicin-2900_uM_in_DMSO-Rat-Primary_rat_hepatocytes-1d-dn	11/344	0,012379137	0,066415589	0	0	2,197714377	9,6517961	RPS17;CXCL11;RPL32;ARL2BP;RPS8;RPS5;RPS6;ARL6IP5;RPSA;RPL10A;SEPP1
Isoprenaline-15_mg/kg_in_Saline-Rat-Heart-1d-dn	11/344	0,012379137	0,066370377	0	0	2,197714377	9,6517961	RPS17;RPL32;RPS8;FUNDC1;RPS6;RPS20;RPS3A;RPL10A;SEPP1;RPL39;TPT1
Loratadine-2000_mg/kg_in_Water-Rat-Heart-3d-up	11/344	0,012379137	0,066325227	0	0	2,197714377	9,6517961	RPS17;RPL32;RPS19;RPS5;RPS6;RPL35A;RPS20;RPSA;RPS3A;RPL10A;GOLGA7
Rifapentine-75_mg/kg_in_Corn_Oil-Rat-Kidney-5d-up	11/344	0,012379137	0,066280139	0	0	2,197714377	9,6517961	EEF1B2;RPS17;RPL32;RPS19;RPS6;RPL35A;RPS20;RPSA;RPS3A;RPL10A;RPL39
Nystatin-134_mg/kg_in_Corn_Oil-Rat-Kidney-5d-dn	9/254	0,01237958	0,066237483	0	0	2,435262602	10,69495961	EEF1B2;RPS17;RPL32;CLP1;RPS8;RPS5;RPL35A;RPS3A;TOMM20
Chlormadinone_Acetate-2000_mg/kg_in_CMC-Rat-Kidney-3d-dn	10/299	0,012549385	0,067100446	0	0	2,298612787	10,06351899	EEF1B2;RPL32;RPS8;FEM1B;RPS6;ARL6IP5;RPL35A;RPSA;RPL10A;SEPP1
Chloroform-60_mg/kg_in_Water-Rat-Liver-1d-up	10/299	0,012549385	0,067054923	0	0	2,298612787	10,06351899	EEF1B2;RPS17;RPL32;RPS5;RPS6;RPL35A;RPS3A;RPL10A;RPL39;TPT1
Clomipramine-115_mg/kg_in_Water-Rat-Liver-1d-up	10/299	0,012549385	0,067009462	0	0	2,298612787	10,06351899	RPS17;RPL32;RPS8;RPS5;RPS6;RPL35A;RPSA;RPS3A;RPL39;GOLGA7
Fluocinolone_Acetonide-2.5_mg/kg_in_Corn_Oil-Rat-Liver-1d-dn	10/299	0,012549385	0,066964063	0	0	2,298612787	10,06351899	C1QA;RPS17;RPL32;RPS19;RPS6;RPSA;RPS3A;RPL10A;SEPP1;RPL39
Galantamine-110_mg/kg_in_CMC-Rat-Heart-5d-up	10/299	0,012549385	0,066918725	0	0	2,298612787	10,06351899	RPS17;CXCL11;RPL32;RPS19;RPS6;RPSA;RPS3A;RPL10A;RPL39;TPT1
Itraconazole-1093_mg/kg_in_Corn_Oil-Rat-Liver-1d-up	10/299	0,012549385	0,066873448	0	0	2,298612787	10,06351899	EEF1B2;RPL32;RPS19;RPS8;RPS6;RPS20;RPS3A;RPL10A;RPL39;TPT1
Promethazine-113_mg/kg_in_Saline-Rat-Liver-5d-dn	10/299	0,012549385	0,066828233	0	0	2,298612787	10,06351899	EEF1B2;C1QA;RPS17;RPL32;RPS8;RPS6;RPS3A;SEPP1;RPL39;TPT1
Staurosporine-1.3_uM_in_DMSO-Rat-Primary_rat_hepatocytes-0.67d-dn	10/299	0,012549385	0,066783079	0	0	2,298612787	10,06351899	RPS17;RPL32;RPS19;RPS8;RPS5;RPL35A;RPS20;RPS3A;TPT1;PDRG1
Sulfisoxazole-250_mg/kg_in_Corn_Oil-Rat-Liver-1d-up	10/299	0,012549385	0,066737985	0	0	2,298612787	10,06351899	EEF1B2;C1QA;RPS17;RPL32;RPS19;RPS8;RPL35A;RPS20;RPS3A;TPT1
Terazosin-657_mg/kg_in_Water-Rat-Heart-5d-dn	10/299	0,012549385	0,066692953	0	0	2,298612787	10,06351899	EEF1B2;RPS17;RPL32;ARL2BP;RPS8;RPS20;RPSA;RPS3A;TOMM20;TPT1
Valdecoxib-21_mg/kg_in_CMC-Rat-Heart-5d-dn	10/299	0,012549385	0,066647981	0	0	2,298612787	10,06351899	C1QA;RPS17;RPS19;ARL2BP;COL5A3;FUNDC1;RPS6;RPL10A;SEPP1;GOLGA7
Clotrimazole-89_mg/kg_in_Corn_Oil-Rat-Liver-1d-dn	11/345	0,012627148	0,067015779	0	0	2,19134419	9,580351258	EEF1B2;C1QA;RPS17;RPL32;RPS6;RPSA;RPS3A;RPL10A;SEPP1;RPL39;TPT1
Valproic_Acid-850_mg/kg_in_Water-Rat-Liver-3d-up	11/345	0,012627148	0,06697065	0	0	2,19134419	9,580351258	EEF1B2;C1QA;RPS17;RPL32;RPS5;RPS6;RPL35A;RPSA;RPS3A;RPL10A;RPL39
Sulfathiazole-2629_mg/kg_in_Water-Rat-Kidney-1d-dn	8/212	0,012671918	0,067162869	0	0	2,593529145	11,32948696	RPS17;RPL32;RPS19;RPS6;ARL6IP5;RPS20;RPSA;RPL39
Amantadine-58_mg/kg_in_Water-Rat-Liver-0.25d-dn	9/255	0,012674589	0,067131851	0	0	2,425712553	10,59589122	EEF1B2;RPS17;RPL32;RPS8;RPL35A;RPS20;RPS3A;RPL39;TPT1
1-Naphthyl_Isothiocyanate-60_mg/kg_in_Corn_Oil-Rat-Kidney-3d-up	10/300	0,012821091	0,067862173	0	0	2,290950745	9,980901987	EEF1B2;RPS17;RPL32;RPS8;RPS5;RPS6;RPL13A;RPL35A;RPS20;RPS3A
Aflatoxin_B1-0.3_mg/kg_in_CMC-Rat-Liver-5d-dn	10/300	0,012821091	0,067816598	0	0	2,290950745	9,980901987	C1QA;RPL32;CLP1;RPS8;RPS5;RPS6;RPS20;RPSA;RPS3A;RPL10A
Amikacin-20_mg/kg_in_Saline-Rat-Kidney-1d-dn	10/300	0,012821091	0,067771083	0	0	2,290950745	9,980901987	EEF1B2;RPS17;RPL32;CLP1;RPS5;FUNDC1;RPL35A;RPS20;RPS3A;SEPP1
Bezafibrate-100_mg/kg_in_Corn_Oil-Rat-Kidney-5d-up	10/300	0,012821091	0,06772563	0	0	2,290950745	9,980901987	RPS17;RPL32;RPS19;ARL2BP;RPS8;ARL6IP5;RPS20;RPS3A;RPL10A;TPT1
Bromisovalum-250_mg/kg_in_Corn_Oil-Rat-Liver-5d-up	10/300	0,012821091	0,067680237	0	0	2,290950745	9,980901987	RPS17;RPS19;RPS8;RPS6;RPS20;RPSA;RPS3A;SEPP1;RPL39;TPT1
Carbon_Tetrachloride-1175_mg/kg_in_Corn_Oil-Rat-Kidney-1d-dn	10/300	0,012821091	0,067634906	0	0	2,290950745	9,980901987	EEF1B2;RPS17;RPL32;ARL2BP;ARL6IP5;RPL35A;RPSA;RPS3A;RPL39;TOMM20
Carbon_Tetrachloride-1175_mg/kg_in_Corn_Oil-Rat-Kidney-1d-up	10/300	0,012821091	0,067589635	0	0	2,290950745	9,980901987	C1QA;RPS19;RPS8;RPS5;RPS6;RPS20;RAPGEF5;SEPP1;GOLGA7;TPT1
Cinnarizine-750_mg/kg_in_CMC-Rat-Liver-1d-up	10/300	0,012821091	0,067544424	0	0	2,290950745	9,980901987	RPS17;RPL32;RPS19;RPS8;RPS5;RPS6;RPSA;RPS3A;RPL10A;TPT1
Econazole-334_mg/kg_in_Corn_Oil-Rat-Liver-5d-up	10/300	0,012821091	0,067499274	0	0	2,290950745	9,980901987	EEF1B2;RPS17;RPL32;RPS8;RPS6;RPL35A;RPS20;RPS3A;RPL39;TPT1
Ethanol-3000_mg/kg_in_Saline-Rat-Liver-3d-up	10/300	0,012821091	0,067454184	0	0	2,290950745	9,980901987	EEF1B2;RPS17;RPL32;RPS19;RPS8;RPS6;RPL35A;RPS20;RPS3A;RPL10A
Etoposide-188_mg/kg_in_Corn_Oil-Rat-Bone_marrow-1d-up	10/300	0,012821091	0,067409155	0	0	2,290950745	9,980901987	EEF1B2;C1QA;RPS17;RPS19;NKG7;RPL35A;RPS20;RPS3A;SEPP1;RPL39
Hydroxyurea-400_mg/kg_in_Saline-Rat-Spleen-1d-dn	10/300	0,012821091	0,067364185	0	0	2,290950745	9,980901987	EEF1B2;RPS17;RPL32;RPS8;RPS5;RPL13A;RPL35A;RPS20;RPS3A;TPT1
Lovastatin-450_mg/kg_in_Corn_Oil-Rat-Liver-5d-up	10/300	0,012821091	0,067319276	0	0	2,290950745	9,980901987	RPS17;CXADR;RPL32;RPS19;RPS6;RPS20;RPSA;RPL10A;RPL39;TPT1
Methylparaben-1500_mg/kg_in_CMC-Rat-Liver-1d-up	10/300	0,012821091	0,067274426	0	0	2,290950745	9,980901987	RPS17;RPS19;RPS8;RPS5;RPS6;RPSA;RPS3A;RPL10A;RPL39;TPT1
N-Nitrosodiethylamine-100_mg/kg_in_Saline-Rat-Liver-1d-up	10/300	0,012821091	0,067229636	0	0	2,290950745	9,980901987	EEF1B2;CXCL11;RPS19;RPS8;RPS6;RPS20;SEPP1;RPL39;TPT1;PDRG1
NN-Dimethylformamide-140_mg/kg_in_Saline-Rat-Liver-0.25d-up	10/300	0,012821091	0,067184906	0	0	2,290950745	9,980901987	EEF1B2;C1QA;RPS17;RPL32;RPS5;RPL35A;RPSA;RPS3A;RPL39;TPT1
Nitrazepam-310_mg/kg_in_CMC-Rat-Liver-3d-dn	10/300	0,012821091	0,067140235	0	0	2,290950745	9,980901987	RPL32;CLP1;RPS8;ARL6IP5;RPL35A;RPS20;RPSA;RPL10A;SEPP1;TPT1
Sildenafil-420_mg/kg_in_Water-Rat-Liver-1d-up	10/300	0,012821091	0,067095624	0	0	2,290950745	9,980901987	EEF1B2;RPS17;RPL32;RPS8;RPL13A;RPL35A;RPS20;RPS3A;RPL39;TPT1
Tetracycline-1500_mg/kg_in_Water-Rat-Liver-5d-up	10/300	0,012821091	0,067051072	0	0	2,290950745	9,980901987	RPS17;RPL32;RPS5;RPS6;ARL6IP5;RPS20;RPSA;RAPGEF5;SEPP1;RPL39
Tranexamic_Acid-2000_mg/kg_in_CMC-Rat-Heart-3d-dn	10/300	0,012821091	0,067006579	0	0	2,290950745	9,980901987	EEF1B2;RPS17;RPL32;RPS8;RPS6;ARL6IP5;RPL35A;RPS20;RPS3A;RPL39
Methotrexate-27_mg/kg_in_Corn_Oil-Rat-Kidney-1d-up	12/393	0,012905218	0,067401521	0	0	2,098580835	9,129085985	RPS17;RPL32;RPS8;RPS5;RPS6;RPL35A;RPS20;RPSA;RPS3A;RPL39;TOMM20;TPT1
Cloxacillin-1200_mg/kg_in_Water-Rat-Kidney-1d-up	9/256	0,012974803	0,067720045	0	0	2,416237113	10,49793663	EEF1B2;RPS17;RPL32;RPS19;RPS8;RPS6;RPS20;RPL10A;RPL39
Ofloxacin-3000_mg/kg_in_Water-Rat-Kidney-1d-dn	8/213	0,013006078	0,067838323	0	0	2,581352952	11,20910835	RPS17;RPL32;RPS19;RPS6;RPL35A;RPS20;RPSA;RPL39
Sodium_Selenate-1.6_mg/kg_in_Water-Rat-Kidney-1d-dn	8/213	0,013006078	0,067793427	0	0	2,581352952	11,20910835	EEF1B2;RPS17;RPL32;RPS19;RPS6;RPSA;RPS3A;RPL39
Allyl_Alcohol-32_mg/kg_in_Saline-Rat-Liver-3d-up	10/301	0,013097205	0,068223272	0	0	2,283339613	9,899091045	EEF1B2;RPS17;RPS19;RPS8;RPS5;RPS6;RPS20;RPSA;RPS3A;RPL10A
Azauridine-1500_mg/kg_in_CMC-Rat-Bone_marrow-5d-dn	10/301	0,013097205	0,068178181	0	0	2,283339613	9,899091045	RPS17;RPL32;RPS8;RPS5;RPS6;ARL6IP5;RPL13A;RPL35A;RPS3A;TPT1
Carvedilol-20_mg/kg_in_Corn_Oil-Rat-Heart-1d-dn	10/301	0,013097205	0,068133149	0	0	2,283339613	9,899091045	RPL32;RPS19;ARL2BP;RPS8;FEM1B;FUNDC1;RPS6;ARL6IP5;RPS20;RPSA
Dexamethasone-1_mg/kg_in_Corn_Oil-Rat-Liver-0.25d-up	10/301	0,013097205	0,068088177	0	0	2,283339613	9,899091045	EEF1B2;RPS17;RPL32;RPS19;RPS6;ARL6IP5;RPSA;RPS3A;RPL10A;TPT1
Digoxin-0.26_mg/kg_in_Corn_Oil-Rat-Liver-3d-dn	10/301	0,013097205	0,068043264	0	0	2,283339613	9,899091045	EEF1B2;C1QA;RPS17;RPL32;RPS8;RPS5;RPS6;RPSA;RPL10A;TPT1
Fluocinolone_Acetonide-2.5_mg/kg_in_Corn_Oil-Rat-Heart-3d-dn	10/301	0,013097205	0,06799841	0	0	2,283339613	9,899091045	RPS17;RPL32;RPS19;ARL2BP;RPS8;COL5A3;RPS6;RPSA;RPS3A;TPT1
Ketamine-30_mg/kg_in_Saline-Rat-Heart-5d-up	10/301	0,013097205	0,067953615	0	0	2,283339613	9,899091045	C1QA;RPS17;RPL32;RPS19;RPL35A;RPS20;RPSA;RPS3A;SEPP1;RPL39
Pravastatin-1200_mg/kg_in_Corn_Oil-Rat-Skeletal_muscle-3d-dn	10/301	0,013097205	0,067908879	0	0	2,283339613	9,899091045	RPS8;FEM1B;COL5A3;ARL6IP5;RPS20;RPL10A;SEPP1;RPL39;SLC16A3;TPT1
Tranilast-300_mg/kg_in_CMC-Rat-Heart-1d-dn	10/301	0,013097205	0,067864202	0	0	2,283339613	9,899091045	RPL32;RPS19;RPS8;COL5A3;RPS6;ARL6IP5;RPSA;RPL10A;SEPP1;GOLGA7
Amitriptyline-72_uM_in_DMSO-Rat-Primary_rat_hepatocytes-1d-up	11/347	0,013134498	0,068012693	0	0	2,178713965	9,439306728	RPS17;RPL32;RPS19;ARL2BP;RPS8;RPS5;RPS3A;RPL10A;RPL39;TPT1;PDRG1
Clarithromycin-56_mg/kg_in_Water-Rat-Liver-3d-up	11/347	0,013134498	0,067968007	0	0	2,178713965	9,439306728	EEF1B2;RPS17;RPL32;RPS19;RPS8;RPS5;RPS6;RPL35A;RPS20;RPS3A;RPL10A
Fluocinolone_Acetonide-2.5_mg/kg_in_Corn_Oil-Rat-Liver-3d-up	11/347	0,013134498	0,067923379	0	0	2,178713965	9,439306728	EEF1B2;RPS17;RPL32;RPS19;RPS8;RPS5;RPS6;RPL35A;RPS3A;RPL10A;TPT1
Ubenimex-1300_mg/kg_in_Water-Rat-Kidney-5d-up	11/347	0,013134498	0,06787881	0	0	2,178713965	9,439306728	C1QA;RPS17;RPL32;RPS19;RPS6;RPS20;RPSA;RPS3A;RPL10A;RPL39;PDRG1
Fluoxetine-39.6_uM_in_DMSO-Rat-Primary_rat_hepatocytes-1d-up	9/257	0,013280275	0,068587177	0	0	2,406835413	10,40108011	EEF1B2;RPS17;RPL32;RPS8;RPS5;RPS6;RPSA;RPS3A;RPL10A
123-Trichloropropane-108_mg/kg_in_CMC-Rat-Kidney-5d-up	10/302	0,013377769	0,069045421	0	0	2,275778885	9,818076388	EEF1B2;RPS17;RPS19;CLP1;RPS8;RPSA;RPS3A;RPL10A;RPL39;TPT1
Acetazolamide-47_mg/kg_in_Corn_Oil-Rat-Liver-0.25d-dn	10/302	0,013377769	0,069000204	0	0	2,275778885	9,818076388	EEF1B2;C1QA;RPS17;RPL32;RPL35A;RPS20;RPS3A;SEPP1;RPL39;TPT1

Aspirin-375_mg/kg_in_Corn_Oil-Rat-Liver-3d-dn	10/302	0,013377769	0,068955047	0	0	2,275778885	9,818076388	C1QA;RPS17;RPL32;CLP1;RPS8;RPS6;RPL35A;RPS20;RPL39;TPT1
Azathioprine-20_mg/kg_in_Corn_Oil-Rat-Bone_marrow-3d-dn	10/302	0,013377769	0,068909949	0	0	2,275778885	9,818076388	RPS17;RPL32;RPS8;FEM1B;RPS6;ARL6IP5;RPSA;RPS3A;RPL10A;TPT1
Carmustine-16_mg/kg_in_Corn_Oil-Rat-Bone_marrow-1d-dn	10/302	0,013377769	0,06886491	0	0	2,275778885	9,818076388	RPL32;RPS19;RPS8;FEM1B;RPS6;ARL6IP5;RPS20;RPSA;RPS3A;TPT1
Chloroform-600_mg/kg_in_Water-Rat-Liver-1d-up	10/302	0,013377769	0,06881993	0	0	2,275778885	9,818076388	RPS17;RPL32;RPS8;RPS5;RPS6;RPL35A;RPS20;RPSA;RPL39;TPT1
Cimetidine-2000_mg/kg_in_CMC-Rat-Heart-3d-dn	10/302	0,013377769	0,068775008	0	0	2,275778885	9,818076388	EEF1B2;RPS17;ARL2BP;RPS8;FUNDC1;RPS6;ARL6IP5;RPS20;RPSA;RPS3A
Cloxacillin-1200_mg/kg_in_Water-Rat-Kidney-5d-up	10/302	0,013377769	0,068730145	0	0	2,275778885	9,818076388	EEF1B2;RPS17;RPL32;RPS19;RPS5;ARL6IP5;RPL13A;RPL35A;RPS20;RPS3A
Daunorubicin-1_mg/kg_in_Saline-Rat-Kidney-5d-dn	10/302	0,013377769	0,06868534	0	0	2,275778885	9,818076388	RPS17;RPL32;RPS19;ARL2BP;ARL6IP5;RPS20;RPS3A;RPL10A;SEPP1;TPT1
Ethisterone-1500_mg/kg_in_CMC-Rat-Liver-3d-up	10/302	0,013377769	0,068640594	0	0	2,275778885	9,818076388	C1QA;RPS17;RPL32;RPS6;ARL6IP5;RPL35A;RPS20;RPSA;RPS3A;RPL10A
Granisetron-175_mg/kg_in_Water-Rat-Liver-5d-up	10/302	0,013377769	0,068595906	0	0	2,275778885	9,818076388	RPS17;RPL32;RPS19;RPS8;RPS5;RPS6;RPS20;RPL10A;SEPP1;RPL39
Lead(IV)_Acetate-200_mg/kg_in_Saline-Rat-Bone_marrow-0.25d-up	10/302	0,013377769	0,068551277	0	0	2,275778885	9,818076388	RPS17;RPL32;RPS19;RPS8;RPS5;RPL13A;RPL35A;RPS20;RPSA;TPT1
N-Nitrosodiethylamine-1.67_mg/kg_in_Saline-Rat-Bone_marrow-3d-up	10/302	0,013377769	0,068506705	0	0	2,275778885	9,818076388	RPL32;RPS19;RPS8;RPS5;RPL35A;RPSA;RPS3A;RPL10A;SEPP1;TPT1
Flutamide-15_mg/kg_in_Corn_Oil-Rat-Kidney-0.25d-up	11/348	0,013393908	0,06854478	0	0	2,172453292	9,369694003	RPS17;RPL32;RPS19;RPS6;RPS20;RPSA;RPS3A;RPL10A;SEPP1;RPL39;TPT1
Penciclovir-1200_mg/kg_in_Corn_Oil-Rat-Bone_marrow-1d-up	11/348	0,013393908	0,068500271	0	0	2,172453292	9,369694003	EEF1B2;C1QA;RPS17;RPL32;RPS19;RPS8;NKG7;RPS20;RPS3A;RPL39;TPT1
Zopiclone-414_mg/kg_in_Water-Rat-Liver-0.25d-up	11/349	0,013657187	0,069801429	0	0	2,166228498	9,300679089	C1QA;RPS17;RPL32;RPS19;RPS8;RPS6;RPL35A;RPS20;RPSA;RPS3A;RPL10A
Acrolein-20_mg/kg_in_Water-Rat-Kidney-3d-dn	10/303	0,013662826	0,069784968	0	0	2,268268064	9,737848387	EEF1B2;RPS17;RPL32;RPS19;ARL2BP;RPS8;RPS6;RPL35A;RPSA;RPL39
Amiodarone-147_mg/kg_in_CMC-Rat-Kidney-5d-up	10/303	0,013662826	0,069739742	0	0	2,268268064	9,737848387	RPS19;FEM1B;RPS6;RPS20;RPSA;RPS3A;RPL10A;SEPP1;RPL39;TPT1
Amlodipine-0.2_mg/kg_in_Corn_Oil-Rat-Liver-1d-up	10/303	0,013662826	0,069694573	0	0	2,268268064	9,737848387	EEF1B2;RPS17;RPL32;RPS19;RPS8;COL5A3;RPS5;RPL35A;RPS3A;SEPP1
Amoxapine-313_mg/kg_in_CMC-Rat-Liver-1d-up	10/303	0,013662826	0,069649464	0	0	2,268268064	9,737848387	EEF1B2;RPS17;RPL32;RPS5;RPS6;RPL35A;RPSA;RPS3A;RPL39;TPT1
Artemisinin-2000_mg/kg_in_CMC-Rat-Liver-5d-up	10/303	0,013662826	0,069604412	0	0	2,268268064	9,737848387	RPS17;RPS19;RPS8;FEM1B;RPS6;RPS20;RPSA;RPS3A;SEPP1;RPL39
Carvedilol-50_uM_in_DMSO-Rat-Primary_rat_hepatocytes-0.67d-up	10/303	0,013662826	0,069559419	0	0	2,268268064	9,737848387	RPS17;RPL32;RPS19;ARL2BP;RPS6;RPS20;RPS3A;RPL10A;RPL39;PDRG1
Clonazepam-2500_mg/kg_in_CMC-Rat-Heart-3d-dn	10/303	0,013662826	0,069514484	0	0	2,268268064	9,737848387	RPS17;RPL32;RPS19;RPS6;ARL6IP5;RPSA;RPS3A;RPL10A;SEPP1;PDRG1
Cortisone-206_mg/kg_in_CMC-Rat-Liver-5d-dn	10/303	0,013662826	0,069469607	0	0	2,268268064	9,737848387	EEF1B2;RPS17;RPL32;RPS19;RPS6;ARL6IP5;RPS20;RPS3A;SEPP1;RPL39
Erythromycin-1500_mg/kg_in_CMC-Rat-Liver-1d-dn	10/303	0,013662826	0,069424788	0	0	2,268268064	9,737848387	EEF1B2;RPL32;RPS19;CLP1;RPS6;RPS20;RPS3A;RPL10A;SEPP1;RPL39
Lacidipine-1_mg/kg_in_Corn_Oil-Rat-Heart-1d-up	10/303	0,013662826	0,069380027	0	0	2,268268064	9,737848387	C1QA;RPS17;RPL32;RPS19;RPS20;RPS3A;RAPGEF5;RPL39;TPT1;PDRG1
Mianserin-116_mg/kg_in_Corn_Oil-Rat-Heart-1d-up	10/303	0,013662826	0,069335323	0	0	2,268268064	9,737848387	C1QA;RPS17;RPL32;RPS19;RPS6;ARL6IP5;RPS20;RPS3A;RPL39;TPT1
Nimetazepam-122_mg/kg_in_CMC-Rat-Liver-5d-dn	10/303	0,013662826	0,069290677	0	0	2,268268064	9,737848387	EEF1B2;RPS17;RPL32;RPS8;RPS6;ARL6IP5;RPL35A;RPS20;SEPP1;TPT1
Stavudine-58_mg/kg_in_Water-Rat-Liver-1d-up	10/303	0,013662826	0,069246088	0	0	2,268268064	9,737848387	EEF1B2;RPS17;RPL32;RPS19;RPS6;RPS20;RPSA;RPS3A;SEPP1;RPL39
Sulpiride-667_uM_in_DMSO-Rat-Primary_rat_hepatocytes-1d-dn	10/303	0,013662826	0,069201557	0	0	2,268268064	9,737848387	CXCL11;RPS19;RPS8;RPS5;ARL6IP5;RPL35A;RPSA;SEPP1;GOLGA7;TPT1
Isotretinoin-500_uM_in_DMSO-Rat-Primary_rat_hepatocytes-1d-up	9/259	0,013907205	0,070394051	0	0	2,388249811	10,21059966	EEF1B2;ARL2BP;RPS6;ARL6IP5;RPS20;RPSA;RPS3A;RPL10A;PDRG1
Loxoprofen-18_mg/kg_in_Corn_Oil-Rat-Kidney-0.25d-dn	9/259	0,013907205	0,07034884	0	0	2,388249811	10,21059966	EEF1B2;RPS17;RPL32;CLP1;RPS8;FUNDC1;ARL6IP5;RPL35A;SEPP1
Acetaminophen-486_mg/kg_in_Corn_Oil-Rat-Liver-1d-up	10/304	0,013952419	0,070532252	0	0	2,260806656	9,658397557	RPS17;RPL32;RPS19;RPS8;RPS5;RPS6;RPS20;RPSA;RPS3A;RPL10A
Allyl_Alcohol-16_mg/kg_in_Saline-Rat-Kidney-1d-up	10/304	0,013952419	0,07048701	0	0	2,260806656	9,658397557	RPS17;RPL32;RPS6;RPL35A;RPS20;RPSA;RPS3A;SEPP1;RPL39;TPT1
Aspirin-35_mg/kg_in_Corn_Oil-Rat-Kidney-3d-up	10/304	0,013952419	0,070441826	0	0	2,260806656	9,658397557	C1QA;RPS17;RPL32;ARL2BP;RPL35A;RPS20;RPSA;RPS3A;RAPGEF5;RPL39
Daunorubicin-3.25_mg/kg_in_Saline-Rat-Heart-5d-up	10/304	0,013952419	0,0703967	0	0	2,260806656	9,658397557	EEF1B2;CXCL11;RPL32;RPS19;FUNDC1;RPS6;RPS20;RPL10A;SEPP1;RPL39
Doxifluridine-1230_mg/kg_in_CMC-Rat-Bone_marrow-3d-dn	10/304	0,013952419	0,070351632	0	0	2,260806656	9,658397557	EEF1B2;RPS17;FEM1B;RPS5;RPS6;NKG7;RPSA;RPS3A;RPL10A;RPL39
Idarubicin-0.625_mg/kg_in_Saline-Rat-Heart-5d-dn	10/304	0,013952419	0,070306621	0	0	2,260806656	9,658397557	EEF1B2;RPS19;RPS8;RPS6;ARL6IP5;RPS20;RAPGEF5;RPL10A;SEPP1;RPL39
Lead_(II)_Acetate-2_mg/kg_in_Saline-Rat-Kidney-28d-up	10/304	0,013952419	0,070261668	0	0	2,260806656	9,658397557	EEF1B2;RPS17;CXCL11;RPL32;RPS19;RPS8;RPS5;RPS20;RPS3A;RPL39
Nafenopin-338_mg/kg_in_Corn_Oil-Rat-Liver-3d-up	10/304	0,013952419	0,070216773	0	0	2,260806656	9,658397557	EEF1B2;RPS17;RPL32;RPS19;RPS8;RPS6;RPSA;RPS3A;RPL10A;TPT1
Nisoldipine-15_mg/kg_in_Water-Rat-Liver-5d-dn	10/304	0,013952419	0,070171934	0	0	2,260806656	9,658397557	EEF1B2;C1QA;RPS17;RPL32;RPS8;RPS6;RPSA;RPS3A;RPL10A;TPT1
Penciclovir-1200_mg/kg_in_Corn_Oil-Rat-Bone_marrow-3d-up	10/304	0,013952419	0,070127153	0	0	2,260806656	9,658397557	EEF1B2;C1QA;RPL32;RPS8;ARL6IP5;NKG7;RPL35A;RPS20;RPS3A;SLC16A3
Phenytoin-46_mg/kg_in_Water-Rat-Heart-5d-dn	10/304	0,013952419	0,070082429	0	0	2,260806656	9,658397557	RPS17;RPL32;RPS19;ARL2BP;RPS8;RPS6;RPS20;RPSA;RPS3A;TPT1
Clonazepam-2500_mg/kg_in_CMC-Rat-Liver-0.25d-up	10/305	0,014246588	0,071514422	0	0	2,253394175	9,579714555	RPS17;RPL32;RPS19;RPS8;RPS6;RPS20;RPSA;RPS3A;RPL10A;RPL39
Cyclosporin_A-350_mg/kg_in_Corn_Oil-Rat-Liver-5d-up	10/305	0,014246588	0,071468872	0	0	2,253394175	9,579714555	EEF1B2;RPS17;RPL32;RPS19;RPS5;RPS6;RPS20;RPSA;RPL39;TPT1
Cyproheptadine-37_mg/kg_in_CMC-Rat-Heart-3d-dn	10/305	0,014246588	0,071423379	0	0	2,253394175	9,579714555	EEF1B2;RPS17;RPL32;RPS8;RPS6;RPL35A;RPS20;RPS3A;RPL10A;RPL39
Gabapentin-100_mg/kg_in_Water-Rat-Heart-3d-up	10/305	0,014246588	0,071377944	0	0	2,253394175	9,579714555	RPS19;ARL2BP;FEM1B;FUNDC1;RPS6;RPSA;RAPGEF5;RPL10A;GOLGA7;TPT1
Gallamine_Triethiodide-2.5_mg/kg_in_Saline-Rat-Heart-5d-up	10/305	0,014246588	0,071332567	0	0	2,253394175	9,579714555	C1QA;RPS17;RPL32;RPS8;RPS6;RPL35A;RPS20;RPS3A;RPL39;TPT1
Hydrocortisone-56_mg/kg_in_Corn_Oil-Rat-Bone_marrow-5d-dn	10/305	0,014246588	0,071287248	0	0	2,253394175	9,579714555	EEF1B2;C1QA;RPS17;CLP1;RPS8;FUNDC1;RPS3A;RPL10A;RPL39;TPT1
Lacidipine-14_mg/kg_in_Corn_Oil-Rat-Heart-1d-up	10/305	0,014246588	0,071241986	0	0	2,253394175	9,579714555	EEF1B2;C1QA;RPS17;CXCL11;RPL32;RPS19;RPS6;RPS3A;RPL10A;RPL39
Lamivudine-35_mg/kg_in_Water-Rat-Liver-1d-up	10/305	0,014246588	0,071196782	0	0	2,253394175	9,579714555	EEF1B2;RPS17;RPL32;RPS6;RPL35A;RPS20;RPS3A;RPL10A;RPL39;TPT1
Mefenamic_Acid-93_mg/kg_in_Corn_Oil-Rat-Liver-5d-up	10/305	0,014246588	0,071151635	0	0	2,253394175	9,579714555	C1QA;RPS17;RPS8;RPS5;RPL35A;RPS20;RPSA;SEPP1;RPL39;TPT1
Norepinephrine-1.75_mg/kg_in_Saline-Rat-Heart-3d-up	10/305	0,014246588	0,071106545	0	0	2,253394175	9,579714555	C1QA;RPS19;RPS8;RPSA;RPS3A;RAPGEF5;RPL10A;SEPP1;RPL39;TPT1
Sulbactam-671_mg/kg_in_Saline-Rat-Kidney-3d-up	10/305	0,014246588	0,071061513	0	0	2,253394175	9,579714555	EEF1B2;RPS17;CXCL11;RPL32;RPS19;ARL6IP5;RPS20;RPSA;RAPGEF5;SEPP1
Sulindac-64_mg/kg_in_Corn_Oil-Rat-Liver-1d-up	10/305	0,014246588	0,071016537	0	0	2,253394175	9,579714555	EEF1B2;RPS17;RPL32;RPS19;RPS8;RPS20;RPS3A;RPL10A;RPL39;TPT1
Terbinafine-2000_mg/kg_in_CMC-Rat-Liver-5d-dn	10/305	0,014246588	0,070971618	0	0	2,253394175	9,579714555	EEF1B2;C1QA;RPL32;RPS19;RPS6;ARL6IP5;RPL35A;RPS3A;SEPP1;TPT1
Vinblastine-0.08_mg/kg_in_Saline-Rat-Bone_marrow-0.25d-up	10/305	0,014246588	0,070926756	0	0	2,253394175	9,579714555	RPL32;RPS19;RPS8;RPS5;FUNDC1;RPS6;RPL13A;RPL35A;RPS20;RPSA
Prednisolone-184_mg/kg_in_Water-Rat-Kidney-1d-dn	8/217	0,014407527	0,071682678	0	0	2,533770409	10,74319802	RPS17;RPL32;RPS19;RPL35A;RPSA;RPS3A;RPL39;SEPP1
Benzyl_Acetate-1868_mg/kg_in_CMC-Rat-Kidney-1d-up	10/306	0,014545377	0,072322849	0	0	2,246030142	9,501790175	RPS17;CXCL11;CLP1;RPS8;RPS6;RPS20;RPSA;RPS3A;TOMM20;TPT1
Carbamazepine-490_mg/kg_in_CMC-Rat-Liver-3d-up	10/306	0,014545377	0,072277219	0	0	2,246030142	9,501790175	RPS17;RPL32;RPS19;RPS8;RPS6;RPS20;RPSA;RPS3A;RPL10A;RPL39
Catechol-40_mg/kg_in_Saline-Rat-Bone_marrow-0.25d-dn	10/306	0,014545377	0,072231647	0	0	2,246030142	9,501790175	RPL32;RPS19;RPS8;RPS6;RPS20;RPSA;RPS3A;RPL10A;RPL39;TPT1
Chlorambucil-4.5_mg/kg_in_Corn_Oil-Rat-Bone_marrow-3d-up	10/306	0,014545377	0,072186133	0	0	2,246030142	9,501790175	EEF1B2;C1QA;RPS8;RPS5;RPL35A;RPS20;SEPP1;RPL39;SLC16A3;TPT1
Clomiphene-250_mg/kg_in_CMC-Rat-Liver-3d-up	10/306	0,014545377	0,072140675	0	0	2,246030142	9,501790175	C1QA;RPS17;RPS19;RPS6;RPS20;RPSA;RPS3A;RPL10A;RPL39;TPT1
Diethylstilbestrol-280_mg/kg_in_Corn_Oil-Rat-Kidney-3d-up	10/306	0,014545377	0,072095275	0	0	2,246030142	9,501790175	CXCL11;RPS19;ARL2BP;RPS6;RPS20;RPS3A;RPL10A;RPL39;GOLGA7;TPT1
Indomethacin-4.5_mg/kg_in_Water-Rat-Liver-1d-up	10/306	0,014545377	0,072049932	0	0	2,246030142	9,501790175	EEF1B2;RPS17;RPL32;RPS5;RPL35A;RPS20;RPS3A;RPL10A;RPL39;TPT1
Oxaprozin-1500_mg/kg_in_CMC-Rat-Kidney-5d-up	10/306	0,014545377	0,072004646	0	0	2,246030142	9,501790175	RPS17;RPL32;RPS19;RPS8;RPS6;RPS20;RAPGEF5;RPL10A;RPL39;TPT1
Vinorelbine-1.5_mg/kg_in_Saline-Rat-Heart-5d-dn	10/306	0,014545377	0,071959417	0	0	2,246030142	9,501790175	RPL32;RPS8;FUNDC1;RPS6;ARL6IP5;RPSA;RPS3A;SEPP1;RPL39;TPT1
Lead_(II)_Acetate-600_mg/kg_in_Saline-Rat-Spleen-3d-dn	9/261	0,014555799	0,071965773	0	0	2,369949046	10,02432937	EEF1B2;RPS17;RPL32;RPS8;RPS5;RPL35A;RPSA;RPS3A;TPT1
Tenidap-41_uM_in_DMSO-Rat-Primary_rat_hepatocytes-1d-dn	11/353	0,014749695	0,072878667	0	0	2,141681999	9,030472617	CXCL11;RPS19;RPS8;RPS6;ARL6IP5;RPS20;RPSA;RPL10A;SEPP1;RPL39;TPT1
Sodium_Selenite-2_mg/kg_in_Water-Rat-Kidney-1d-dn	8/218	0,014774428	0,072955104	0	0	2,522147609	10,63049266	RPS17;RPL32;RPS19;RPS6;RPL35A;RPS20;RPSA;RPL39
Azathioprine-54_mg/kg_in_Water-Rat-Liver-1d-up	10/307	0,014848828	0,07327655	0	0	2,238714083	9,42461535	EEF1B2;RPS17;RPL32;RPS19;RPS6;RPS20;RPS3A;RPL10A;RPL39;TPT1
Beta-Estradiol-150_mg/kg_in_Corn_Oil-Rat-Kidney-1d-dn	10/307	0,014848828	0,073230666	0	0	2,238714083	9,42461535	RPS17;RPL32;RPS19;RPS6;RPSA;RPL10A;SEPP1;RPL39;GOLGA7;TPT1
Doxifluridine-1230_mg/kg_in_CMC-Rat-Bone_marrow-0.25d-up	10/307	0,014848828	0,073184839	0	0	2,238714083	9,42461535	RPS17;RPL32;RPS19;RPS8;FUNDC1;RPS6;RPL35A;RPS20;RPL39;TPT1
Furosemide-375_mg/kg_in_CMC-Rat-Kidney-5d-up	10/307	0,014848828	0,07313907	0	0	2,238714083	9,42461535	EEF1B2;RPL32;RPS8;ARL6IP5;RPL13A;RPL35A;RPS20;RPSA;RPL39;TPT1
Ifosfamide-143_mg/kg_in_Saline-Rat-Liver-5d-up	10/307	0,014848828	0,073093358	0	0	2,238714083	9,42461535	RPS17;RPL32;ARL2BP;RPS8;RPS6;RPL35A;RPS20;RPS3A;RPL39;TPT1
Lipopolysaccharide_E._Coli_O55:B5-1.25_mg/kg_in_Saline-Rat-Liver-1d-up	10/307	0,014848828	0,073047703	0	0	2,238714083	9,42461535	RPS17;RPL32;RPS19;RPS8;RPS20;RPSA;RPL10A;SEPP1;RPL39;TPT1
Sildenafil-2.5_mg/kg_in_Water-Rat-Liver-5d-up	10/307	0,014848828	0,073002106	0	0	2,238714083	9,42461535	RPS17;RPL32;RPS19;RPS8;FEM1B;RPS6;RPS20;RPS3A;RPL39;TPT1
Tetracaine-24_mg/kg_in_Corn_Oil-Rat-Heart-1d-up	10/307	0,014848828	0,072956565	0	0	2,238714083	9,42461535	EEF1B2;RPS17;RPL32;RPS8;RPS5;ARL6IP5;RPL35A;RPS20;RPS3A;TPT1
Thioacetamide-200_mg/kg_in_Saline-Rat-Liver-1d-up	10/307	0,014848828	0,07291108	0	0	2,238714083	9,42461535	EEF1B2;RPS19;RPS8;RPS6;RPS20;RPSA;RPS3A;RPL10A;RPL39;TPT1
Chloroxylenol-1915_mg/kg_in_Corn_Oil-Rat-Liver-1d-up	9/262	0,014888352	0,073059603	0	0	2,360903439	9,932736506	EEF1B2;C1QA;RPS17;ARL2BP;RPS8;RPS5;RPS20;RPSA;SEPP1
Ramipril-620_uM_in_DMSO-Rat-Primary_rat_hepatocytes-1d-up	9/262	0,014888352	0,073014112	0	0	2,360903439	9,932736506	EEF1B2;RPS17;RPL32;RPS8;RPS5;RPL35A;RPS20;SEPP1;PDRG1
Azathioprine-0.5_uM_in_DMSO-Rat-Primary_rat_hepatocytes-1d-dn	11/354	0,015032844	0,073676836	0	0	2,13563205	8,964353767	RPS17;RPL32;RPS19;ARL2BP;CLP1;RPS8;RPS5;RPS20;RPS3A;RPL39;TPT1
Chlorambucil-4.5_mg/kg_in_Corn_Oil-Rat-Liver-3d-dn	11/354	0,015032844	0,073631017	0	0	2,13563205	8,964353767	EEF1B2;C1QA;RPS17;RPL32;RPS19;RPS6;RPSA;RPS3A;RPL10A;RPL39;TPT1
Phenothiazine-36_mg/kg_in_Corn_Oil-Rat-Liver-3d-up	11/354	0,015032844	0,073585255	0	0	2,13563205	8,964353767	EEF1B2;C1QA;RPL32;RPS8;RPS5;RPS6;RPL35A;RPS20;RPSA;RPL39;TPT1
Epinephrine-0.0375_mg/kg_in_Saline-Rat-Heart-3d-dn	8/219	0,015148078	0,074103267	0	0	2,510630953	10,51924654	RPS19;ARL2BP;RPS8;FUNDC1;ARL6IP5;RPS20;SEPP1;TPT1
Acetaminophen-486_mg/kg_in_Corn_Oil-Rat-Liver-5d-dn	10/308	0,015156983	0,074100808	0	0	2,23144553	9,348181146	EEF1B2;C1QA;RPS17;RPL32;RPS19;RPS6;RPS3A;SEPP1;RPL39;TPT1
Azauridine-1500_mg/kg_in_CMC-Rat-Bone_marrow-1d-dn	10/308	0,015156983	0,07405484	0	0	2,23144553	9,348181146	EEF1B2;C1QA;RPS17;RPL32;RPS8;FEM1B;RPS6;RPS3A;RPL39;TPT1
Caffeine-93_mg/kg_in_Water-Rat-Brain-5d-up	10/308	0,015156983	0,074008929	0	0	2,23144553	9,348181146	EEF1B2;RPS17;RPS19;ARL2BP;CLP1;RPS8;ARL6IP5;RPSA;RPS3A;TPT1
Carvedilol-2000_mg/kg_in_Corn_Oil-Rat-Liver-5d-up	10/308	0,015156983	0,073963074	0	0	2,23144553	9,348181146	EEF1B2;RPL32;RPS19;ARL2BP;RPS6;RPS20;RPL10A;SEPP1;RPL39;TPT1
Cyproterone_Acetate-2500_mg/kg_in_CMC-Rat-Liver-0.25d-up	10/308	0,015156983	0,073917277	0	0	2,23144553	9,348181146	RPS17;RPL32;RPS19;RPS6;ARL6IP5;RPS20;RPSA;RPL10A;RPL39;GOLGA7
Erythromycin-1500_mg/kg_in_CMC-Rat-Kidney-1d-dn	10/308	0,015156983	0,073871536	0	0	2,23144553	9,348181146	RPL32;ARL2BP;RPS6;ARL6IP5;RPS20;RPSA;RPL10A;SEPP1;RPL39;TOMM20
Hydrocortisone-56_mg/kg_in_Corn_Oil-Rat-Kidney-5d-dn	10/308	0,015156983	0,073825851	0	0	2,23144553	9,348181146	C1QA;RPS17;RPL32;RPS8;RPSA;RPS3A;RAPGEF5;RPL10A;SEPP1;RPL39

Ritonavir-1200_mg/kg_in_CMC-Rat-Kidney-5d-up	10/308	0,015156983	0,073780224	0	0	2,23144553	9,348181146	C1QA;CXCL11;RPS19;RPS6;RPSA;RPS3A;RAPGEF5;RPL10A;RPL39;TPT1
Thalidomide-113_mg/kg_in_CMC-Rat-Liver-1d-up	10/308	0,015156983	0,073734652	0	0	2,23144553	9,348181146	EEF1B2;RPS17;RPL32;RPS8;RPS5;RPL35A;RPSA;RPS3A;RPL10A;TPT1
Diclofenac-3.5_mg/kg_in_Corn_Oil-Rat-Kidney-1d-dn	9/263	0,015226479	0,074027003	0	0	2,35192662	9,842152856	EEF1B2;RPS17;RPL32;CLP1;RPS8;RPL35A;RPS3A;SEPP1;TOMM20
Leflunomide-60_mg/kg_in_Corn_Oil-Rat-Kidney-3d-up	9/263	0,015226479	0,073981336	0	0	2,35192662	9,842152856	RPS17;RPL32;ARL2BP;RPS8;FUNDC1;RPL35A;RPS20;RPS3A;RAPGEF5
Modafinil-325_mg/kg_in_Saline-Rat-Liver-0.25d-up	9/263	0,015226479	0,073935724	0	0	2,35192662	9,842152856	RPS17;RPL32;RPS19;RPS8;RPS6;RPSA;RPS3A;RPL10A;SEPP1
N-Nitrosodiethylamine-34_mg/kg_in_Saline-Rat-Bone_marrow-1d-dn	9/263	0,015226479	0,073890169	0	0	2,35192662	9,842152856	EEF1B2;RPS17;RPL32;RPS8;RPS5;RPL35A;RPL10A;RPL39;TPT1
Bisphenol_A-50_mg/kg_in_Corn_Oil-Rat-Liver-0.25d-up	10/309	0,015469884	0,075025129	0	0	2,224224024	9,27247876	RPS17;RPL32;RPS19;RPL35A;RPS20;RPSA;RPS3A;RPL10A;SEPP1;RPL39
Cadmium_Chloride-2_mg/kg_in_Saline-Rat-Kidney-14d-up	10/309	0,015469884	0,07497896	0	0	2,224224024	9,27247876	RPL32;ARL2BP;RPS8;RPS6;RPS20;RPSA;RPS3A;RPL10A;RPL39;TPT1
Clopidogrel-400_mg/kg_in_Corn_Oil-Rat-Bone_marrow-3d-up	10/309	0,015469884	0,074932847	0	0	2,224224024	9,27247876	C1QA;RPL32;ARL2BP;CLP1;RPS8;RPS5;RPS6;RPL35A;RPS20;SLC16A3
Closantel-22_mg/kg_in_CMC-Rat-Liver-1d-up	10/309	0,015469884	0,074886791	0	0	2,224224024	9,27247876	EEF1B2;RPS17;CXCL11;RPL32;RPS6;RPS20;RPSA;RPS3A;RPL10A;TPT1
Etoposide-188_mg/kg_in_Corn_Oil-Rat-Kidney-5d-up	10/309	0,015469884	0,074840792	0	0	2,224224024	9,27247876	ARL2BP;CLP1;RPS8;RPS6;RPS20;RPSA;RPS3A;RPL10A;RPL39;TPT1
Hexachloro-13-Butadiene-50_mg/kg_in_Corn_Oil-Rat-Kidney-1d-up	10/309	0,015469884	0,074794849	0	0	2,224224024	9,27247876	RPS17;RPL32;RPS8;RPS5;ARL6IP5;RPL35A;RPSA;RPS3A;TOMM20;TPT1
Irinotecan-5_mg/kg_in_Saline-Rat-Heart-3d-dn	10/309	0,015469884	0,074748963	0	0	2,224224024	9,27247876	EEF1B2;RPS17;RPL32;RPS19;ARL2BP;RPS8;RPS6;ARL6IP5;RPS3A;TPT1
Meloxicam-33_mg/kg_in_Corn_Oil-Rat-Liver-0.25d-up	10/309	0,015469884	0,074703133	0	0	2,224224024	9,27247876	EEF1B2;RPS17;RPL32;RPS19;RPS6;ARL6IP5;RPL35A;RPS20;RPSA;RPL39
Microcystin-LR-0.07_uM_in_DMSO-Rat-Primary_rat_hepatocytes-0.67d-up	10/309	0,015469884	0,074657359	0	0	2,224224024	9,27247876	EEF1B2;RPS19;ARL2BP;CLP1;RPS6;ARL6IP5;RPS20;RPSA;RPL39;PDRG1
Sulconazole-40_uM_in_DMSO-Rat-Primary_rat_hepatocytes-0.67d-up	10/309	0,015469884	0,074611641	0	0	2,224224024	9,27247876	EEF1B2;RPS17;RPL32;RPS19;RPS5;RPS6;RPS20;RPSA;RPL10A;RPL39
Tranilast-300_mg/kg_in_CMC-Rat-Liver-5d-dn	10/309	0,015469884	0,074565979	0	0	2,224224024	9,27247876	RPS17;RPL32;RPS19;RPS8;RPS5;RPS6;RPL35A;RPS20;RPSA;RPL10A
Lead(IV)_Acetate-600_mg/kg_in_Saline-Rat-Liver-5d-dn	9/264	0,01557023	0,07500375	0	0	2,343017807	9,752564495	EEF1B2;RPS17;RPL32;RPS19;RPS8;RPS6;RPSA;RPS3A;SEPP1
Secobarbital-20_mg/kg_in_Water-Rat-Liver-0.25d-up	11/356	0,015611411	0,075156156	0	0	2,123634117	8,833793749	EEF1B2;RPS17;RPL32;RPS19;RPS8;RPS6;RPL35A;RPS20;RPSA;SEPP1;GOLGA7
4-Nitrobenzoic_Acid-1470_mg/kg_in_CMC-Rat-Kidney-1d-up	10/310	0,015787573	0,075957805	0	0	2,217049108	9,197499518	RPS17;RPS19;RPS8;RPS6;RPS20;RPSA;RPS3A;RAPGEF5;RPL10A;RPL39
Acyclovir-980_mg/kg_in_Saline-Rat-Liver-5d-dn	10/310	0,015787573	0,075911432	0	0	2,217049108	9,197499518	EEF1B2;RPL32;RPS19;RPS5;RPS6;RPSA;RPS3A;RPL10A;SEPP1;RPL39
Bromobenzene-439_mg/kg_in_CMC-Rat-Kidney-5d-up	10/310	0,015787573	0,075865117	0	0	2,217049108	9,197499518	RPL32;RPS8;RPS5;RPS6;RPSA;RPS3A;RAPGEF5;RPL10A;SEPP1;TPT1
Gemfibrozil-700_mg/kg_in_Corn_Oil-Rat-Skeletal_muscle-3d-up	10/310	0,015787573	0,075818857	0	0	2,217049108	9,197499518	RPS17;RPL32;ARL2BP;RPS8;RPS5;FUNDC1;RPS6;RPL35A;RPS20;RPS3A
Hydralazine-280_mg/kg_in_Water-Rat-Heart-1d-dn	10/310	0,015787573	0,075772655	0	0	2,217049108	9,197499518	EEF1B2;RPS17;RPL32;RPS19;RPS5;RPL35A;RPS20;RPS3A;RPL10A;RPL39
Hydrocortisone-56_mg/kg_in_Corn_Oil-Rat-Heart-5d-dn	10/310	0,015787573	0,075726508	0	0	2,217049108	9,197499518	RPS17;RPL32;RPS19;ARL2BP;RPS8;COL5A3;RPS6;RPSA;RPS3A;RPL39
Neomycin-877_mg/kg_in_Corn_Oil-Rat-Liver-3d-up	10/310	0,015787573	0,075680418	0	0	2,217049108	9,197499518	RPS17;RPL32;RPS19;RPS8;RPL35A;RPS20;RPS3A;RPL39;GOLGA7;TPT1
Nystatin-134_mg/kg_in_Corn_Oil-Rat-Kidney-1d-up	10/310	0,015787573	0,075634383	0	0	2,217049108	9,197499518	C1QA;RPS19;ARL2BP;FEM1B;RPS6;ARL6IP5;RPS20;RPSA;SEPP1;GOLGA7
Testosterone-375_mg/kg_in_CMC-Rat-Liver-3d-up	10/310	0,015787573	0,075588405	0	0	2,217049108	9,197499518	EEF1B2;RPS17;RPL32;RPS8;RPS5;RPSA;RPS3A;RPL10A;SEPP1;TPT1
Thioguanine-24_mg/kg_in_Corn_Oil-Rat-Liver-5d-up	10/310	0,015787573	0,075542482	0	0	2,217049108	9,197499518	EEF1B2;RPS17;RPL32;RPS19;RPS8;RPS20;RPSA;RPS3A;RPL39;TPT1
Tolazamide-1500_mg/kg_in_CMC-Rat-Liver-5d-up	10/310	0,015787573	0,075496616	0	0	2,217049108	9,197499518	EEF1B2;RPS17;RPS19;RPS8;RPS6;RPS20;RPSA;RPS3A;RPL10A;SEPP1
Vinblastine-0.3_mg/kg_in_Saline-Rat-Liver-3d-up	10/310	0,015787573	0,075450805	0	0	2,217049108	9,197499518	RPS17;RPL32;RPS19;RPS8;RPS6;RPS20;RPSA;RPS3A;RPL10A;TPT1
Dexchlorpheniramine-47_mg/kg_in_Water-Rat-Heart-3d-up	11/357	0,015906899	0,075974974	0	0	2,117685562	8,769340986	RPS19;ARL2BP;RPS8;FUNDC1;RPS6;ARL6IP5;RPS20;RPSA;RAPGEF5;RPL39;GOLGA7
Doxorubicin-0.65_mg/kg_in_Saline-Rat-Bone_marrow-3d-dn	8/221	0,01591589	0,075971848	0	0	2,487910311	10,30103667	RPS17;RPL32;RPS8;FEM1B;RPSA;RPS3A;RPL39;TPT1
Mannitol-1000_mg/kg_in_Saline-Rat-Kidney-3d-dn	9/265	0,015919658	0,075943809	0	0	2,33417623	9,663957734	EEF1B2;RPS19;CLP1;FUNDC1;RPS6;RPS20;RPL10A;RPL39;TPT1
1-Naphthyl_Isothiocyanate-15_mg/kg_in_Corn_Oil-Rat-Liver-5d-up	10/311	0,016110092	0,076805739	0	0	2,209920332	9,123234875	EEF1B2;C1QA;RPS17;RPL32;RPS19;RPS20;RPSA;RPS3A;RPL10A;SEPP1
2378-Tetrachlorodibenzo-P-Dioxin-0.0007_mg/kg_in_CMC-Rat-Liver-91d-dn	10/311	0,016110092	0,076759275	0	0	2,209920332	9,123234875	EEF1B2;RPS17;RPL32;RPS19;RPS8;RPS6;RPS20;RPS3A;RPL10A;RPL39
Azathioprine-54_mg/kg_in_Water-Rat-Liver-0.25d-up	10/311	0,016110092	0,076712867	0	0	2,209920332	9,123234875	EEF1B2;RPS17;RPL32;RPS19;RPS8;FEM1B;RPS20;RPS3A;RPL10A;TPT1
Danazol-95_uM_in_DMSO-Rat-Primary_rat_hepatocytes-0.67d-dn	10/311	0,016110092	0,076666514	0	0	2,209920332	9,123234875	EEF1B2;CXCL11;RPL32;RPS19;RPS8;ARL6IP5;RPSA;SEPP1;RPL39;SLC16A3
Gallamine_Triethiodide-2.5_mg/kg_in_Saline-Rat-Heart-1d-dn	10/311	0,016110092	0,076620218	0	0	2,209920332	9,123234875	EEF1B2;RPS17;RPL32;RPS8;RPS20;RPSA;RPS3A;SEPP1;RPL39;TPT1
Indomethacin-10_mg/kg_in_Corn_Oil-Rat-Liver-3d-up	10/311	0,016110092	0,076573978	0	0	2,209920332	9,123234875	EEF1B2;C1QA;RPS19;ARL2BP;RPS8;RPS6;RPS20;RPSA;RPL10A;RPL39
Lidocaine-71_mg/kg_in_Water-Rat-Heart-5d-up	10/311	0,016110092	0,076527793	0	0	2,209920332	9,123234875	RPS17;CXCL11;RPL32;RPS19;ARL2BP;RPS8;RPS6;RPS3A;RAPGEF5;RPL10A
Penicillamine-100_mg/kg_in_Water-Rat-Liver-5d-up	10/311	0,016110092	0,076481664	0	0	2,209920332	9,123234875	EEF1B2;RPS17;RPS19;RPS8;RPS5;RPS6;RPL35A;RPS20;SEPP1;GOLGA7
Sodium_Orthovanadate-83_mg/kg_in_CMC-Rat-Kidney-3d-up	10/311	0,016110092	0,076435591	0	0	2,209920332	9,123234875	EEF1B2;RPS17;CXCL11;RPS8;RPS5;ARL6IP5;RPSA;RPS3A;RPL39;TPT1
Doxorubicin-3_mg/kg_in_Saline-Rat-Heart-3d-up	11/358	0,016206568	0,076847039	0	0	2,111770239	8,705432116	RPS17;CXCL11;RPL32;RPS19;RPS6;RPSA;RPS3A;RAPGEF5;RPL10A;SEPP1;TPT1
Meloxicam-0.6_mg/kg_in_Corn_Oil-Rat-Kidney-3d-up	11/358	0,016206568	0,076800801	0	0	2,111770239	8,705432116	C1QA;RPS17;ARL2BP;RPS8;RPL13A;RPL35A;RPS20;RPS3A;RAPGEF5;RPL39;TPT1
Ouabain-144_mg/kg_in_CMC-Rat-Heart-1d-up	11/358	0,016206568	0,076754619	0	0	2,111770239	8,705432116	EEF1B2;RPS17;RPL32;RPS19;RPS6;RPL35A;RPSA;RPS3A;RPL10A;RPL39;PDRG1
Enalapril-1500_mg/kg_in_CMC-Rat-Kidney-3d-up	9/266	0,016274815	0,077031518	0	0	2,325401132	9,576319118	RPS17;ARL2BP;DOK4;RPS8;RPS6;RPL35A;RPSA;RPS3A;RPL39
Nimodipine-10_mg/kg_in_Corn_Oil-Rat-Heart-1d-up	8/222	0,016310183	0,077152555	0	0	2,476703508	10,19402649	RPS19;COL5A3;RPS6;RPS20;RPS3A;RPL10A;RPL39;TPT1
2378-Tetrachlorodibenzo-P-Dioxin-0.0007_mg/kg_in_CMC-Rat-Liver-28d-dn	10/312	0,016437481	0,077708045	0	0	2,202837254	9,049676408	C1QA;RPS17;RPL32;RPS19;RPS8;RPS6;RPS3A;RPL10A;RPL39;TPT1
Alpha-Naphthoflavone-80_mg/kg_in_CMC-Rat-Liver-5d-dn	10/312	0,016437481	0,07766143	0	0	2,202837254	9,049676408	C1QA;RPS17;RPL32;RPS19;RPS8;RPS6;RPS20;RPL10A;RPL39;TPT1
Cadmium_Acetate-167_mg/kg_in_Water-Rat-Kidney-1d-up	10/312	0,016437481	0,07761487	0	0	2,202837254	9,049676408	EEF1B2;RPS17;RPL32;RPS19;RPS8;RPS5;RPS3A;RPL10A;RPL39;TPT1
Carbon_Tetrachloride-1175_mg/kg_in_Corn_Oil-Rat-Bone_marrow-3d-up	10/312	0,016437481	0,077568366	0	0	2,202837254	9,049676408	C1QA;RPS8;FEM1B;RPS5;FUNDC1;RPS6;NKG7;RPS20;RPL39;TPT1
Cisplatin-1.17_mg/kg_in_Saline-Rat-Spleen-1d-dn	10/312	0,016437481	0,077521918	0	0	2,202837254	9,049676408	EEF1B2;C1QA;CXCL11;RPL32;RPS8;RPS5;RPL13A;RPL35A;RPSA;TPT1
Daunorubicin-3.25_mg/kg_in_Saline-Rat-Heart-1d-dn	10/312	0,016437481	0,077475526	0	0	2,202837254	9,049676408	EEF1B2;RPS17;RPL32;RPS8;FUNDC1;RPS20;RPSA;RPS3A;RPL39;TPT1
Diazepam-710_mg/kg_in_CMC-Rat-Kidney-1d-dn	10/312	0,016437481	0,077429189	0	0	2,202837254	9,049676408	RPS17;RPL32;RPS19;ARL2BP;RPS6;RPL35A;RPS20;RPL39;GOLGA7;TPT1
Diflunisal-207_mg/kg_in_Corn_Oil-Rat-Kidney-0.25d-up	10/312	0,016437481	0,077382907	0	0	2,202837254	9,049676408	RPS17;RPL32;RPS19;RPS6;RPS20;RPSA;RPS3A;SEPP1;RPL39;TPT1
Epalrestat-2500_mg/kg_in_CMC-Rat-Liver-0.25d-up	10/312	0,016437481	0,077336681	0	0	2,202837254	9,049676408	C1QA;RPS17;RPL32;RPS19;RPS8;RPS6;RPSA;RPS3A;RPL10A;RPL39
Ethylestrenol-390_mg/kg_in_CMC-Rat-Liver-5d-dn	10/312	0,016437481	0,077290509	0	0	2,202837254	9,049676408	EEF1B2;C1QA;RPS17;RPL32;RPS19;RPS8;RPS6;RPS20;RPL10A;TPT1
Mevastatin-1200_mg/kg_in_CMC-Rat-Skeletal_muscle-5d-up	10/312	0,016437481	0,077244393	0	0	2,202837254	9,049676408	EEF1B2;RPL32;RPS8;FUNDC1;ARL6IP5;RPL35A;RPS20;RPSA;SEPP1;TOMM20
NN-Dimethylformamide-140_mg/kg_in_Saline-Rat-Spleen-0.25d-dn	10/312	0,016437481	0,077198332	0	0	2,202837254	9,049676408	EEF1B2;RPL32;RPS19;RPS8;FEM1B;RPS6;RPL35A;RPS20;RPL10A;RPL39
Zomepirac-2.8_mg/kg_in_Saline-Rat-Kidney-0.25d-dn	10/312	0,016437481	0,077152326	0	0	2,202837254	9,049676408	RPS19;ARL2BP;RPS8;FUNDC1;RPS6;RPS20;RPSA;RPL10A;SEPP1;TPT1
Famciclovir-112_mg/kg_in_Saline-Rat-Bone_marrow-0.25d-up	12/407	0,016557286	0,077668367	0	0	2,026393779	8,310097031	EEF1B2;C1QA;RPS17;RPL32;RPS5;NKG7;RPL13A;RPL35A;RPS20;RPS3A;RPL39;TPT1
Methyl_Salicylate-444_mg/kg_in_Corn_Oil-Rat-Kidney-5d-dn	9/267	0,016635753	0,077989992	0	0	2,316691764	9,489635422	EEF1B2;RPS17;RPL32;RPS8;ARL6IP5;RPS20;RPSA;SEPP1;TOMM20
Nortriptyline-70_uM_in_DMSO-Rat-Primary_rat_hepatocytes-1d-up	9/267	0,016635753	0,077943597	0	0	2,316691764	9,489635422	EEF1B2;RPS17;RPL32;RPS5;RPS20;RPSA;RPS3A;RPL10A;PDRG1
Pyridine-223_mg/kg_in_Water-Rat-Kidney-5d-dn	9/267	0,016635753	0,077897258	0	0	2,316691764	9,489635422	RPS17;RPL32;RPS19;RPS6;ARL6IP5;RPL35A;RPSA;RPS3A;RPL39
Aceclofenac-1_mg/kg_in_Corn_Oil-Rat-Kidney-0.25d-dn	8/223	0,016711489	0,078205398	0	0	2,465597214	10,08838266	RPS17;CLP1;RPS8;RPS6;ARL6IP5;RPL35A;RPS20;TOMM20
Tacrine-24_mg/kg_in_Water-Rat-Heart-3d-dn	8/223	0,016711489	0,078158958	0	0	2,465597214	10,08838266	RPS17;RPL32;RPS19;COL5A3;RPS6;RPSA;RPL10A;RPL39
Amiodarone-147_mg/kg_in_CMC-Rat-Heart-3d-up	10/313	0,016769784	0,078385056	0	0	2,195799436	8,976815818	C1QA;RPS17;RPL32;RPS19;RPS5;RPL35A;RPS20;RPS3A;TOMM20;TPT1
Flavoxate-1255_mg/kg_in_CMC-Rat-Heart-1d-dn	10/313	0,016769784	0,078338565	0	0	2,195799436	8,976815818	EEF1B2;RPL32;RPS19;RPS8;FUNDC1;RPS6;RPS3A;SEPP1;RPL39;TPT1
Ifosfamide-143_mg/kg_in_Saline-Rat-Heart-3d-up	10/313	0,016769784	0,078292128	0	0	2,195799436	8,976815818	CXCL11;RPS19;RPS8;RPS6;RPSA;RAPGEF5;RPL10A;SEPP1;RPL39;TPT1
Ifosfamide-17_mg/kg_in_Saline-Rat-Spleen-3d-dn	10/313	0,016769784	0,078245746	0	0	2,195799436	8,976815818	EEF1B2;RPS17;RPL32;RPS8;RPS6;ARL6IP5;RPS3A;RPL10A;RPL39;TPT1
Methocarbamol-1125_mg/kg_in_CMC-Rat-Liver-3d-up	10/313	0,016769784	0,07819942	0	0	2,195799436	8,976815818	RPS17;CXCL11;RPL32;RPS19;RPS6;RPSA;RPS3A;RPL10A;RPL39;TPT1
Nortriptyline-63_mg/kg_in_Water-Rat-Heart-5d-dn	10/313	0,016769784	0,078153148	0	0	2,195799436	8,976815818	EEF1B2;RPS17;RPS19;RPS8;COL5A3;ARL6IP5;RPS20;RPSA;SEPP1;TPT1
Ouabain-144_mg/kg_in_CMC-Rat-Heart-3d-up	10/313	0,016769784	0,078106931	0	0	2,195799436	8,976815818	EEF1B2;RPS17;RPL32;RPS19;RPS6;RPL35A;RPS20;RPSA;RPS3A;RPL10A
Proglumide-1125_mg/kg_in_CMC-Rat-Liver-1d-dn	10/313	0,016769784	0,078060768	0	0	2,195799436	8,976815818	EEF1B2;C1QA;RPL32;RPS19;RPS8;FEM1B;RPS6;RPL35A;RPS3A;SEPP1
Foscarnet-350_mg/kg_in_Saline-Rat-Bone_marrow-1d-dn	9/268	0,017002521	0,079097375	0	0	2,308047392	9,403893643	C1QA;RPL32;RPS19;RPS8;FUNDC1;ARL6IP5;NKG7;RPS20;RPL39
Altretamine-40_mg/kg_in_Corn_Oil-Rat-Bone_marrow-3d-dn	10/314	0,017107041	0,079536634	0	0	2,188806444	8,904644927	RPS17;RPS19;FEM1B;RPS6;ARL6IP5;RPSA;RPS3A;RPL10A;RPL39;TPT1
Amlodipine-0.2_mg/kg_in_Corn_Oil-Rat-Liver-5d-up	10/314	0,017107041	0,079489709	0	0	2,188806444	8,904644927	C1QA;RPS17;RPL32;RPS8;RPS5;RPL35A;RPS20;RPS3A;RPL39;TPT1
Azathioprine-54_mg/kg_in_Water-Rat-Bone_marrow-3d-up	10/314	0,017107041	0,07944284	0	0	2,188806444	8,904644927	C1QA;RPS17;RPL32;RPS5;NKG7;RPS20;RPSA;RPS3A;RPL10A;SEPP1
Benzothiazyl_Disulfide-2600_mg/kg_in_Corn_Oil-Rat-Liver-1d-up	10/314	0,017107041	0,079396027	0	0	2,188806444	8,904644927	C1QA;RPS17;RPL32;RPS19;RPS8;RPS6;RPSA;RPL10A;RPL39;TPT1
Bithionol-59_mg/kg_in_Corn_Oil-Rat-Kidney-1d-dn	10/314	0,017107041	0,079349268	0	0	2,188806444	8,904644927	RPS17;RPL32;RPS8;FUNDC1;RPS6;RPSA;RPS3A;RPL10A;RPL39;TPT1
Clarithromycin-476_mg/kg_in_Water-Rat-Liver-0.25d-up	10/314	0,017107041	0,079302565	0	0	2,188806444	8,904644927	EEF1B2;RPS17;RPL32;RPS19;RPS8;RPS6;RPS20;RPS3A;RPL39;TPT1
Digitoxin-2.75_mg/kg_in_Corn_Oil-Rat-Heart-3d-up	10/314	0,017107041	0,079255916	0	0	2,188806444	8,904644927	EEF1B2;RPS17;RPL32;COL5A3;RPS6;RPS3A;RPL10A;SEPP1;RPL39;TPT1
Etidronate-1005_mg/kg_in_Water-Rat-Heart-5d-dn	10/314	0,017107041	0,079209322	0	0	2,188806444	8,904644927	EEF1B2;C1QA;RPS19;RPS8;FEM1B;RPS5;RPS20;RPSA;SEPP1;TOMM20
Finasteride-800_mg/kg_in_Corn_Oil-Rat-Liver-1d-dn	10/314	0,017107041	0,079162783	0	0	2,188806444	8,904644927	RPS17;RPL32;CLP1;RPS8;ARL6IP5;RPS20;RPSA;SEPP1;RPL39;TPT1
Furosemide-375_mg/kg_in_CMC-Rat-Kidney-1d-up	10/314	0,017107041	0,079116299	0	0	2,188806444	8,904644927	EEF1B2;RPS17;RPL32;RPS8;RPS6;ARL6IP5;RPL13A;RPL35A;RPS20;RPS3A
Phenothiazine-386_mg/kg_in_Corn_Oil-Rat-Liver-0.25d-up	10/314	0,017107041	0,079069869	0	0	2,188806444	8,904644927	RPS17;RPL32;RPS19;RPS8;RPS6;RPS20;RPS3A;RPL10A;SEPP1;RPL39
Risperidone-400_uM_in_DMSO-Rat-Primary_rat_hepatocytes-0.67d-dn	10/314	0,017107041	0,079023494	0	0	2,188806444	8,904644927	RPS17;CXCL11;RPL32;RPS19;RPS8;ARL6IP5;RPSA;SEPP1;SLC16A3;TPT1
Valsartan-30_mg/kg_in_Corn_Oil-Rat-Heart-1d-up	10/314	0,017107041	0,078977173	0	0	2,188806444	8,904644927	C1QA;RPS17;RPL32;RPS19;RPS6;RPL35A;RPS3A;RAPGEF5;RPL39;PDRG1
Indomethacin-9.6_mg/kg_in_Water-Rat-Liver-3d-up	11/361	0,017131015	0,07904152	0	0	2,094220902	8,516913077	EEF1B2;RPS17;RPS19;RPS6;RPL35A;RPS20;RPS3A;SEPP1;RPL39;GOLGA7;TPT1

Pyrogallol-1000_mg/kg_in_Water-Rat-Liver-1d-up	12/409	0,017136906	0,079022406	0	0	2,016484763	8,200077453	RPS17;RPL32;RPS19;RPS8;RPS5;RPS6;RPL35A;RPS20;RPSA;RPS3A;RPL10A;RPL39
Enoxacin-100_mg/kg_in_Corn_Oil-Rat-Liver-3d-dn	12/410	0,01743239	0,080337921	0	0	2,011566507	8,145688276	EEF1B2;C1QA;RPS17;RPL32;RPS19;RPS6;RPL35A;RPSA;RPS3A;RPL10A;RPL39;TPT1
Doxifluridine-1230_mg/kg_in_CMC-Rat-Bone_marrow-1d-dn	10/315	0,017449294	0,080368796	0	0	2,181857852	8,833155675	EEF1B2;RPS17;RPL32;RPS19;RPS8;RPS6;RPS20;RPS3A;RPL10A;RPL39
Furan-24_mg/kg_in_Corn_Oil-Rat-Heart-3d-dn	10/315	0,017449294	0,080321824	0	0	2,181857852	8,833155675	EEF1B2;RPS17;RPL32;RPS19;RPS8;RPS6;RPS20;RPS3A;RPL39;TPT1
Furan-24_mg/kg_in_Corn_Oil-Rat-Heart-5d-up	10/315	0,017449294	0,080274907	0	0	2,181857852	8,833155675	EEF1B2;RPL32;ARL2BP;RPS8;RPS5;ARL6IP5;RPS20;RPS3A;RAPGEF5;TOMM20
Miconazole-200_mg/kg_in_Corn_Oil-Rat-Liver-3d-dn	10/315	0,017449294	0,080228045	0	0	2,181857852	8,833155675	C1QA;RPS17;RPS19;CLP1;RPS3A;RPL10A;SEPP1;RPL39;GOLGA7;TPT1
Phenobarbital-80_mg/kg_in_Water-Rat-Liver-1d-up	10/315	0,017449294	0,080181238	0	0	2,181857852	8,833155675	EEF1B2;RPS17;RPL32;RPS8;RPS6;RPL35A;RPSA;RPS3A;RPL10A;SEPP1
Chlorpropamide-269_mg/kg_in_CMC-Rat-Heart-3d-up	9/270	0,017753755	0,081532698	0	0	2,290950745	9,23518492	RPS17;RPL32;ARL2BP;FUNDC1;RPS6;RPL35A;RPS3A;GOLGA7;TPT1
Clofibrate-500_mg/kg_in_Corn_Oil-Rat-Liver-0.25d-up	9/270	0,017753755	0,081485185	0	0	2,290950745	9,23518492	EEF1B2;RPS17;RPL32;RPS6;RPS20;RPL10A;RPL39;SEPP1;TPT1
Kanamycin-6584_uM_in_DMSO-Rat-Primary_rat_hepatocytes-1d-up	9/270	0,017753755	0,081437727	0	0	2,290950745	9,23518492	EEF1B2;RPS17;RPL32;CLP1;RPS6;RPL35A;RPS3A;RPL39;TPT1
Rifampin-99_mg/kg_in_CMC-Rat-Liver-1d-dn	9/270	0,017753755	0,081390324	0	0	2,290950745	9,23518492	EEF1B2;RPS17;RPL32;RPS8;RPL35A;RPS20;RPS3A;RPL39;TPT1
Tamoxifen-2.5_mg/kg_in_Corn_Oil-Rat-Liver-3d-dn	9/270	0,017753755	0,081342977	0	0	2,290950745	9,23518492	EEF1B2;RPS17;RPL32;RPS8;RPS20;RPSA;RPS3A;RPL10A;TPT1
Omeprazole-400_uM_in_DMSO-Rat-Primary_rat_hepatocytes-1d-dn	11/363	0,017768854	0,081364824	0	0	2,082682495	8,393852144	EEF1B2;CXCL11;RPL32;RPS19;RPS6;ARL6IP5;RPS20;RPL10A;SEPP1;RPL39;TPT1
Altretamine-13_mg/kg_in_Corn_Oil-Rat-Liver-1d-up	10/316	0,017796584	0,081444449	0	0	2,174953239	8,762340116	EEF1B2;RPS17;RPL32;RPS19;COL5A3;RPS5;RPL35A;RPSA;RPS3A;TPT1
Beta-Naphthoflavone-80_mg/kg_in_CMC-Rat-Liver-91d-dn	10/316	0,017796584	0,081397153	0	0	2,174953239	8,762340116	RPS17;CXCL11;RPL32;RPS19;CLP1;RPS6;RPS20;RPS3A;RPL10A;RPL39
Busulfan-9_mg/kg_in_Corn_Oil-Rat-Bone_marrow-5d-up	10/316	0,017796584	0,081349911	0	0	2,174953239	8,762340116	EEF1B2;C1QA;RPS17;RPL32;RPL35A;RPS20;RPS3A;SEPP1;RPL39;TPT1
Doxycycline-14_mg/kg_in_Water-Rat-Liver-0.25d-dn	10/316	0,017796584	0,081302725	0	0	2,174953239	8,762340116	EEF1B2;RPS17;RPL32;RPS19;RPS8;RPL35A;RPS20;RPS3A;RPL39;TPT1
Etoposide-100_mg/kg_in_Corn_Oil-Rat-Bone_marrow-3d-up	10/316	0,017796584	0,081255593	0	0	2,174953239	8,762340116	EEF1B2;C1QA;RPL32;RPS8;RPS5;NKG7;RPL35A;RPS20;SEPP1;SLC16A3
Hydrocortisone-56_mg/kg_in_Corn_Oil-Rat-Liver-5d-dn	10/316	0,017796584	0,081208515	0	0	2,174953239	8,762340116	C1QA;RPS17;RPL32;RPS19;RPS8;RPS6;RPS3A;RPL10A;RPL39;TPT1
Paroxetine-30_uM_in_DMSO-Rat-Primary_rat_hepatocytes-0.67d-up	10/316	0,017796584	0,081161492	0	0	2,174953239	8,762340116	EEF1B2;RPS17;RPL32;RPS5;RPS6;RPS20;RPS3A;RPL39;TPT1;PDRG1
Progesterone-11.3_mg/kg_in_Corn_Oil-Rat-Liver-5d-up	10/316	0,017796584	0,081114524	0	0	2,174953239	8,762340116	RPS17;RPL32;RPS19;RPS8;RPS6;RPS20;RPSA;RPL10A;SEPP1;GOLGA7
Rosiglitazone-1800_mg/kg_in_Corn_Oil-Rat-Heart-3d-up	10/316	0,017796584	0,08106761	0	0	2,174953239	8,762340116	ARL2BP;RPS8;FUNDC1;RPS6;ARL6IP5;RPS20;RPSA;RAPGEF5;RPL10A;TPT1
Tetracycline-1500_mg/kg_in_Water-Rat-Liver-3d-up	10/316	0,017796584	0,08102075	0	0	2,174953239	8,762340116	RPS17;RPL32;ARL2BP;RPS8;RPS5;RPS6;RPSA;RPS3A;SEPP1;TPT1
Thalidomide-113_mg/kg_in_CMC-Rat-Kidney-5d-dn	10/316	0,017796584	0,080973944	0	0	2,174953239	8,762340116	EEF1B2;RPS17;RPL32;RPS8;RPS6;RPS20;RPSA;RPS3A;RPL39;TPT1
Thimerosal-10_mg/kg_in_Water-Rat-Brain-3d-dn	10/316	0,017796584	0,080927192	0	0	2,174953239	8,762340116	RPS17;RPL32;RPS19;ARL2BP;RPS8;RPS20;RPS3A;RAPGEF5;RPL39;TOMM20
Carbon_Tetrachloride-1175_mg/kg_in_Corn_Oil-Rat-Kidney-3d-up	9/271	0,018138323	0,0824336	0	0	2,282497052	9,15219305	EEF1B2;RPS17;RPL32;ARL2BP;RPS8;RPL13A;RPL35A;RPS20;RPS3A
Lomustine-8.75_mg/kg_in_Corn_Oil-Rat-Spleen-5d-up	9/271	0,018138323	0,08238606	0	0	2,282497052	9,15219305	C1QA;CXCL11;RPS19;ARL2BP;CLP1;RPS6;RPS20;RPL10A;SEPP1
Microcystin-LR-0.07_uM_in_DMSO-Rat-Primary_rat_hepatocytes-1d-up	9/271	0,018138323	0,082338575	0	0	2,282497052	9,15219305	EEF1B2;RPS17;CXCL11;RPS6;ARL6IP5;RPL35A;RPL10A;GOLGA7;SLC16A3
Amantadine-58_mg/kg_in_Water-Rat-Liver-1d-up	10/317	0,018148952	0,082339371	0	0	2,168092187	8,692190421	EEF1B2;RPS17;RPL32;RPS19;RPS6;RPS20;RPSA;RPL10A;RPL39;TPT1
Chlorambucil-4.5_mg/kg_in_Corn_Oil-Rat-Spleen-1d-up	10/317	0,018148952	0,082291968	0	0	2,168092187	8,692190421	EEF1B2;C1QA;RPL32;RPS6;NKG7;RPS3A;RPL10A;SEPP1;RPL39;TPT1
Famciclovir-1200_mg/kg_in_Saline-Rat-Spleen-1d-up	10/317	0,018148952	0,082244619	0	0	2,168092187	8,692190421	RPS17;RPL32;RPS19;RPS6;RPS20;RPS3A;RPL10A;SEPP1;RPL39;TPT1
Granisetron-175_mg/kg_in_Water-Rat-Liver-0.25d-up	10/317	0,018148952	0,082197325	0	0	2,168092187	8,692190421	RPS17;RPL32;RPS19;ARL2BP;RPS8;RPS6;ARL6IP5;RPS20;RPSA;RPL10A
N-Nitrosodiethylamine-34_mg/kg_in_Saline-Rat-Spleen-1d-up	10/317	0,018148952	0,082150085	0	0	2,168092187	8,692190421	EEF1B2;CXCL11;RPS19;RPS20;RPSA;RAPGEF5;RPL10A;SEPP1;RPL39;PDRG1
Netilmicin-41_mg/kg_in_Saline-Rat-Kidney-3d-up	10/317	0,018148952	0,082102899	0	0	2,168092187	8,692190421	RPS17;RPL32;RPS19;RPS5;ARL6IP5;RPL35A;RPS20;RPSA;TOMM20;TPT1
Paroxetine-30_uM_in_DMSO-Rat-Primary_rat_hepatocytes-1d-dn	10/317	0,018148952	0,082055768	0	0	2,168092187	8,692190421	EEF1B2;CXCL11;RPS19;RPS8;RPS6;ARL6IP5;RPS20;SEPP1;RPL39;TPT1
Sertraline-210_mg/kg_in_Corn_Oil-Rat-Heart-5d-dn	10/317	0,018148952	0,082008691	0	0	2,168092187	8,692190421	RPS17;RPL32;RPS19;ARL2BP;RPS6;RPS20;RPSA;RPS3A;RPL10A;GOLGA7
Beta-Estradiol-250_uM_in_DMSO-Rat-Primary_rat_hepatocytes-1d-up	8/227	0,018388119	0,083041757	0	0	2,422150567	9,679036063	EEF1B2;CXCL11;RPS6;ARL6IP5;RPSA;RPL10A;GOLGA7;SLC16A3
Bisphenol_A-100_mg/kg_in_Corn_Oil-Rat-Liver-1d-dn	11/365	0,018424246	0,083157227	0	0	2,071270536	8,272836314	EEF1B2;C1QA;RPS17;RPL32;RPS6;RPS20;RPS3A;RPL10A;SEPP1;GOLGA7;TPT1
Anastrozole-400_mg/kg_in_CMC-Rat-Liver-3d-up	10/318	0,018506439	0,083480364	0	0	2,161274287	8,622698873	RPS17;RPL32;RPS19;RPS5;RPS6;RPS20;RPSA;RPS3A;RPL10A;RPL39
Bisphenol_A-610_mg/kg_in_Corn_Oil-Rat-Liver-5d-up	10/318	0,018506439	0,083432579	0	0	2,161274287	8,622698873	RPS17;RPL32;RPS19;RPL35A;RPS20;RPSA;RPS3A;SEPP1;RPL39;TPT1
Ethanol-6000_mg/kg_in_Saline-Rat-Kidney-3d-up	10/318	0,018506439	0,083384849	0	0	2,161274287	8,622698873	RPS17;ARL2BP;RPS5;ARL6IP5;RPL13A;RPL35A;RPS20;RPSA;SEPP1;TOMM20
Lamotrigine-26_mg/kg_in_CMC-Rat-Heart-1d-dn	10/318	0,018506439	0,083337173	0	0	2,161274287	8,622698873	EEF1B2;RPL32;RPS19;ARL2BP;RPS8;FUNDC1;RPS20;RPS3A;SEPP1;GOLGA7
Naproxen-10_mg/kg_in_Corn_Oil-Rat-Liver-0.25d-up	10/318	0,018506439	0,083289552	0	0	2,161274287	8,622698873	EEF1B2;RPS17;RPS8;RPS6;ARL6IP5;RPS20;RPSA;RPS3A;SEPP1;GOLGA7
Naproxen-10_mg/kg_in_Corn_Oil-Rat-Liver-1d-up	10/318	0,018506439	0,083241985	0	0	2,161274287	8,622698873	EEF1B2;RPS17;RPL32;RPS19;RPS8;RPS6;RPS3A;RPL10A;RPL39;TPT1
Sulfathiazole-2629_mg/kg_in_Water-Rat-Liver-1d-up	10/318	0,018506439	0,083194472	0	0	2,161274287	8,622698873	RPL32;RPS8;RPS5;RPL35A;RPS20;RPSA;RPS3A;RPL10A;RPL39;TPT1
Teicoplanin-41_mg/kg_in_Saline-Rat-Bone_marrow-1d-up	10/318	0,018506439	0,083147014	0	0	2,161274287	8,622698873	EEF1B2;RPS17;RPL32;RPS8;RPS5;RPL35A;RPS20;RPSA;RPS3A;TPT1
1-Naphthyl_Isothiocyanate-60_mg/kg_in_Corn_Oil-Rat-Bone_marrow-1d-dn	9/272	0,018528924	0,083200571	0	0	2,274105518	9,070093237	RPS17;RPL32;RPS19;RPS8;RPS5;ARL6IP5;RPL35A;RPS20;RPSA
Nevirapine-200_mg/kg_in_Saline-Rat-Liver-1d-dn	9/272	0,018528924	0,083153163	0	0	2,274105518	9,070093237	EEF1B2;C1QA;RPS17;RPL32;RPS8;RPS5;RPL10A;SEPP1;TPT1
Penicillamine-100_mg/kg_in_Water-Rat-Bone_marrow-3d-dn	9/272	0,018528924	0,08310581	0	0	2,274105518	9,070093237	ARL2BP;CLP1;RPS8;FUNDC1;RPS6;ARL6IP5;RPS20;RPSA;TPT1
Zomepirac-2.8_mg/kg_in_Saline-Rat-Liver-1d-dn	9/272	0,018528924	0,08305851	0	0	2,274105518	9,070093237	EEF1B2;RPL32;RPS8;RPS6;RPS20;RPL10A;SEPP1;TPT1;PDRG1
Hydroxyurea-400_mg/kg_in_Saline-Rat-Liver-0.25d-up	11/366	0,018758609	0,084040275	0	0	2,065611327	8,213082347	EEF1B2;RPS17;RPL32;RPS8;COL5A3;RPS5;RPSA;RPS3A;RPL10A;RPL39;TPT1
Allyl_Alcohol-16_mg/kg_in_Saline-Rat-Kidney-3d-up	10/319	0,018869086	0,084487164	0	0	2,154499133	8,553857863	RPS17;RPL32;ARL2BP;RPS8;RPS5;RPL35A;RPSA;RPL39;TOMM20;TPT1
Catechol-195_mg/kg_in_Saline-Rat-Kidney-5d-up	10/319	0,018869086	0,08443916	0	0	2,154499133	8,553857863	RPS17;RPL32;RPS8;RPS5;RPL13A;RPL35A;RPS20;RPSA;SEPP1;RPL39
Gentamicin-40_mg/kg_in_Saline-Rat-Kidney-5d-up	10/319	0,018869086	0,08439121	0	0	2,154499133	8,553857863	CXCL11;CLP1;RPS6;RPS20;RPSA;RPS3A;RPL10A;SEPP1;RPL39;TPT1
Hydroxyurea-59_mg/kg_in_Saline-Rat-Liver-5d-dn	10/319	0,018869086	0,084343315	0	0	2,154499133	8,553857863	C1QA;RPS19;RPS8;RPS6;RPS20;RPS3A;RPL10A;SEPP1;GOLGA7;TOMM20
NN-Dimethylformamide-140_mg/kg_in_Saline-Rat-Liver-5d-up	10/319	0,018869086	0,084295474	0	0	2,154499133	8,553857863	EEF1B2;RPS17;RPS6;RPL35A;RPSA;RPS3A;RPL10A;SEPP1;RPL39;TPT1
Ciprofloxacin-450_mg/kg_in_Corn_Oil-Rat-Liver-1d-dn	9/273	0,018925608	0,084500052	0	0	2,265775462	8,988873532	EEF1B2;RPS17;RPL32;CLP1;RPS8;RPS6;RPS20;RPS3A;RPL10A
Etoposide-188_mg/kg_in_Corn_Oil-Rat-Kidney-1d-dn	9/273	0,018925608	0,084452177	0	0	2,265775462	8,988873532	EEF1B2;RPS17;RPL32;RPS19;RPS8;RPS6;RPS20;RPSA;RPL39
Nimodipine-1100_mg/kg_in_Corn_Oil-Rat-Heart-1d-dn	9/273	0,018925608	0,084404355	0	0	2,265775462	8,988873532	EEF1B2;RPL32;RPS19;ARL2BP;RPS8;FUNDC1;RPS6;ARL6IP5;RPSA
3-Acetamidophenol-700_mg/kg_in_Corn_Oil-Rat-Kidney-3d-dn	10/320	0,019236933	0,085744248	0	0	2,147766323	8,485659894	EEF1B2;RPS17;RPL32;RPS19;RPS6;RPS20;RPS3A;SEPP1;RPL39;TPT1
Busulfan-3_mg/kg_in_Corn_Oil-Rat-Bone_marrow-1d-up	10/320	0,019236933	0,08569575	0	0	2,147766323	8,485659894	C1QA;RPS17;RPS8;RPS5;NKG7;RPL13A;RPL35A;RPS20;TPT1;DUSP14
Clonazepam-2500_mg/kg_in_CMC-Rat-Heart-5d-dn	10/320	0,019236933	0,085647307	0	0	2,147766323	8,485659894	RPS17;COL5A3;RPS6;ARL6IP5;RPS20;RPSA;RAPGEF5;RPL10A;SEPP1;PDRG1
Digitoxin-1_mg/kg_in_Corn_Oil-Rat-Heart-1d-dn	10/320	0,019236933	0,085598918	0	0	2,147766323	8,485659894	EEF1B2;RPS17;RPL32;ARL2BP;RPS8;FUNDC1;RPSA;RPS3A;TOMM20;TPT1
Doxapram-2_mg/kg_in_Saline-Rat-Liver-1d-up	10/320	0,019236933	0,085550585	0	0	2,147766323	8,485659894	C1QA;RPS17;RPL32;RPS19;RPS8;RPS6;RPSA;RPS3A;RPL10A;RPL39
Lead_(II)_Acetate-600_mg/kg_in_Saline-Rat-Spleen-1d-up	10/320	0,019236933	0,085502306	0	0	2,147766323	8,485659894	C1QA;RPS17;RPL32;RPS19;RPS6;RPS20;RPS3A;RPL10A;SEPP1;RPL39
Losartan-1000_mg/kg_in_Water-Rat-Heart-1d-up	10/320	0,019236933	0,085454081	0	0	2,147766323	8,485659894	RPS17;RPL32;FUNDC1;RPS6;ARL6IP5;RPS20;RPSA;RAPGEF5;SEPP1;TPT1
Racepinephrine-0.77_mg/kg_in_Saline-Rat-Heart-3d-dn	10/320	0,019236933	0,085405911	0	0	2,147766323	8,485659894	RPS17;RPL32;RPS19;RPS6;RPS20;RPSA;RPS3A;RPL10A;SEPP1;TPT1
Disulfiram-100_mg/kg_in_Water-Rat-Liver-0.25d-dn	9/274	0,019328427	0,085763769	0	0	2,257506208	8,908522184	EEF1B2;RPS17;RPL32;RPS8;RPL35A;RPSA;RPS3A;RPL39;TPT1
Oxaliplatin-1.31_mg/kg_in_Saline-Rat-Bone_marrow-1d-dn	9/274	0,019328427	0,085715478	0	0	2,257506208	8,908522184	EEF1B2;RPL32;CLP1;RPS8;FUNDC1;RPS6;RPL35A;RPS3A;RPL39
Urethane-453_mg/kg_in_Water-Rat-Bone_marrow-1d-up	9/274	0,019328427	0,085667242	0	0	2,257506208	8,908522184	EEF1B2;RPL32;RPS19;RPS8;RPS5;FUNDC1;RPL35A;RPSA;RPL10A
2-Amino-4-Nitrophenol-625_mg/kg_in_CMC-Rat-Heart-1d-dn	10/321	0,019610021	0,086866438	0	0	2,141075462	8,418097572	RPL32;RPS19;ARL2BP;RPS8;RPS6;RPSA;RPS3A;RPL10A;RPL39;TPT1
Bezafibrate-100_mg/kg_in_Corn_Oil-Rat-Kidney-1d-dn	10/321	0,019610021	0,086817609	0	0	2,141075462	8,418097572	EEF1B2;RPS17;RPL32;RPL35A;RPSA;RPL10A;SEPP1;RPL39;TOMM20;TPT1
Capsaicin-35_mg/kg_in_CMC-Rat-Liver-1d-dn	10/321	0,019610021	0,086768835	0	0	2,141075462	8,418097572	EEF1B2;C1QA;RPL32;RPS19;FEM1B;RPS6;RPL35A;RPS3A;SEPP1;TPT1
Chlorambucil-1540_uM_in_DMSO-Rat-Primary_rat_hepatocytes-0.67d-dn	10/321	0,019610021	0,086720116	0	0	2,141075462	8,418097572	RPS17;CXCL11;RPS19;RPS8;ARL6IP5;RPSA;RPL10A;SEPP1;RPL39;GOLGA7
Daunorubicin-4_mg/kg_in_Saline-Rat-Kidney-14d-up	10/321	0,019610021	0,086671451	0	0	2,141075462	8,418097572	EEF1B2;RPS17;RPL32;RPS19;RPS6;RPL35A;RPS20;RPS3A;RPL10A;RPL39
Doxorubicin-4_mg/kg_in_Saline-Rat-Heart-14d-dn	10/321	0,019610021	0,086622841	0	0	2,141075462	8,418097572	EEF1B2;RPS17;RPL32;FUNDC1;RPS6;ARL6IP5;RPL35A;RPS20;RPL10A;TOMM20
Ergocalciferol-15_mg/kg_in_CMC-Rat-Heart-5d-dn	10/321	0,019610021	0,086574286	0	0	2,141075462	8,418097572	RPS17;RPL32;COL5A3;FUNDC1;RPS6;RPS20;RPS3A;RPL10A;SEPP1;TPT1
Triacetin-1200_mg/kg_in_CMC-Rat-Liver-1d-up	10/321	0,019610021	0,086525785	0	0	2,141075462	8,418097572	C1QA;RPL32;RPS19;RPS8;RPS5;RPS6;RPSA;RPS3A;RPL39;TPT1
Carbachol-15_mg/kg_in_Water-Rat-Brain-5d-up	9/275	0,019737428	0,087039185	0	0	2,249297095	8,829027636	RPS17;RPS19;RPS6;RPS3A;RPL10A;RPL39;SEPP1;TPT1;PDRG1
Ethanol-6000_mg/kg_in_Saline-Rat-Liver-0.25d-up	9/275	0,019737428	0,086990478	0	0	2,249297095	8,829027636	EEF1B2;RPS17;RPL32;RPS8;RPS6;RPS20;RPS3A;RPL10A;RPL39
Nevirapine-250_uM_in_DMSO-Rat-Primary_rat_hepatocytes-0.67d-dn	9/275	0,019737428	0,086941826	0	0	2,249297095	8,829027636	RPS17;RPL32;RPS19;RPS8;RPS5;RPS20;RPSA;RPS3A;PDRG1
Pramoxine-526_mg/kg_in_Corn_Oil-Rat-Liver-3d-up	9/275	0,019737428	0,086893228	0	0	2,249297095	8,829027636	EEF1B2;C1QA;RPS17;RPL32;RPS20;RPSA;RPS3A;RAPGEF5;SEPP1
Sotalol-2000_mg/kg_in_Water-Rat-Liver-3d-dn	9/275	0,019737428	0,086844684	0	0	2,249297095	8,829027636	EEF1B2;RPS17;RPL32;RPS19;RPS6;RPS20;RPS3A;RPL39;TPT1
Venlafaxine-320_mg/kg_in_Water-Rat-Liver-3d-up	9/275	0,019737428	0,086796195	0	0	2,249297095	8,829027636	EEF1B2;RPS17;RPL32;RPS19;RPS6;RPS20;RPSA;RAPGEF5;SEPP1
Busulfan-9_mg/kg_in_Corn_Oil-Rat-Liver-0.25d-up	11/369	0,019788772	0,086973421	0	0	2,048817739	8,036775561	EEF1B2;RPS17;RPS19;RPS6;RPS20;RPS3A;RPL10A;SEPP1;RPL39;TPT1;PDRG1
Bis(2-Ethylhexyl)Phthalate-100_mg/kg_in_Corn_Oil-Rat-Liver-1d-up	10/322	0,01998839	0,087801763	0	0	2,13442616	8,35116361	RPS17;RPL32;RPS19;RPS6;RPS20;RPSA;RPL10A;SEPP1;RPL39;TPT1
Carbon_Tetrachloride-400_mg/kg_in_Corn_Oil-Rat-Kidney-3d-up	10/322	0,01998839	0,087752821	0	0	2,13442616	8,35116361	EEF1B2;C1QA;RPL32;RPS19;RPS8;RPS5;RPS20;RPSA;RPS3A;SEPP1
Chloroxylenol-1915_mg/kg_in_Corn_Oil-Rat-Liver-3d-dn	10/322	0,01998839	0,087703934	0	0	2,13442616	8,35116361	RPL32;RPS19;RPS6;ARL6IP5;RPL35A;RPS20;RPS3A;RPL10A;RPL39;TPT1
Fenofibrate-215_mg/kg_in_Corn_Oil-Rat-Skeletal_muscle-5d-dn	10/322	0,01998839	0,087655101	0	0	2,13442616	8,35116361	EEF1B2;RPS17;COL5A3;ARL6IP5;RPSA;RPS3A;RPL10A;SEPP1;RPL39;SLC16A3
Hydroxyurea-59_mg/kg_in_Saline-Rat-Bone_marrow-0.25d-up	10/322	0,01998839	0,087606322	0	0	2,13442616	8,35116361	EEF1B2;C1QA;RPS17;RPL32;FEM1B;RPS6;RPL35A;RPS20;RPS3A;PDRG1

Sparfloxacin-450_mg/kg_in_Corn_Oil-Rat-Liver-1d-up	10/322	0,01998839	0,087557598	0	0	2,13442616	8,35116361	EEF1B2;C1QA;RPS17;RPS19;RPS6;RPS20;RPS3A;SEPP1;RPL39;TPT1
Zopiclone-414_mg/kg_in_Water-Rat-Liver-3d-up	10/322	0,01998839	0,087508928	0	0	2,13442616	8,35116361	RPS17;RPL32;RPS19;RPS8;RPS6;RPS20;RPSA;RPS3A;RPL10A;RPL39
Antipyrine-1500_mg/kg_in_CMC-Rat-Kidney-3d-dn	9/276	0,020152662	0,088179092	0	0	2,241147468	8,750378521	EEF1B2;RPL32;RPS19;CLP1;RPS8;RPS6;RPS3A;RPL10A;RPL39
Clomipramine-115_mg/kg_in_Water-Rat-Liver-3d-up	9/276	0,020152662	0,08813013	0	0	2,241147468	8,750378521	C1QA;RPS17;RPS8;RPS5;ARL6IP5;RPSA;RPS3A;RAPGEF5;TPT1
Gliclazide-1500_mg/kg_in_CMC-Rat-Kidney-3d-dn	9/276	0,020152662	0,088081224	0	0	2,241147468	8,750378521	EEF1B2;RPS19;ARL2BP;CLP1;RPS8;RPS6;RPSA;RPS3A;SEPP1
Leflunomide-30_mg/kg_in_Corn_Oil-Rat-Spleen-3d-up	9/276	0,020152662	0,088032371	0	0	2,241147468	8,750378521	EEF1B2;RPL32;RPS8;RPS5;RPS6;RPL35A;RPS20;RPS3A;RAPGEF5
N-Nitrosodiethylamine-1.67_mg/kg_in_Saline-Rat-Liver-3d-up	9/276	0,020152662	0,087983573	0	0	2,241147468	8,750378521	EEF1B2;RPS19;RPS6;RPS20;RPSA;RPS3A;RPL10A;SEPP1;PDRG1
Niacin-2625_mg/kg_in_Water-Rat-Liver-0.25d-up	9/276	0,020152662	0,087934828	0	0	2,241147468	8,750378521	C1QA;RPL32;RPS19;ARL2BP;RPS8;RPS6;RPS20;RPSA;RPL10A
Tamoxifen-37.5_uM_in_DMSO-Rat-Primary_rat_hepatocytes-1d-up	9/276	0,020152662	0,087886138	0	0	2,241147468	8,750378521	EEF1B2;RPS17;RPL32;RPS19;RPS8;RPS6;RPS3A;RPL10A;PDRG1
(R)-Bicalutamide-1400_mg/kg_in_CMC-Rat-Kidney-3d-up	10/323	0,020372081	0,088793861	0	0	2,127818029	8,284850823	RPS17;CXCL11;RPL32;RPS19;RPS6;RPSA;RAPGEF5;RPL39;GOLGA7;TPT1
Acyclovir-330_mg/kg_in_Saline-Rat-Spleen-3d-up	10/323	0,020372081	0,088744749	0	0	2,127818029	8,284850823	EEF1B2;CXCL11;RPL32;RPS19;FEM1B;R3HCC1;RPS6;ARL6IP5;RPS20;RPS3A
Bisphenol_A-100_mg/kg_in_Corn_Oil-Rat-Kidney-0.25d-up	10/323	0,020372081	0,088695692	0	0	2,127818029	8,284850823	CLP1;RPS8;RPS5;FUNDC1;RPL35A;RPS20;RPS3A;SEPP1;GOLGA7;TOMM20
Famciclovir-1200_mg/kg_in_Saline-Rat-Liver-5d-up	10/323	0,020372081	0,088646689	0	0	2,127818029	8,284850823	RPS17;RPL32;RPS19;RPS8;RPS5;RPS6;RPL35A;RPS20;RPS3A;RPL10A
Neomycin-877_mg/kg_in_Corn_Oil-Rat-Liver-5d-up	10/323	0,020372081	0,08859774	0	0	2,127818029	8,284850823	EEF1B2;C1QA;RPS17;RPL32;RPS8;RPL35A;RPS20;RPS3A;RPL39;TPT1
Ticlopidine-223_mg/kg_in_CMC-Rat-Liver-1d-up	10/323	0,020372081	0,088548845	0	0	2,127818029	8,284850823	RPL32;RPS19;RPS8;RPS5;RPS6;RPS20;RPS3A;RPL10A;RPL39;TPT1
Tretinoin-7_mg/kg_in_Corn_Oil-Rat-Kidney-1d-up	10/323	0,020372081	0,088500003	0	0	2,127818029	8,284850823	RPS17;RPL32;ARL2BP;CLP1;RPS8;ARL6IP5;RPSA;SEPP1;TOMM20;TPT1
Tretinoin-7_mg/kg_in_Corn_Oil-Rat-Liver-3d-up	10/323	0,020372081	0,088451216	0	0	2,127818029	8,284850823	EEF1B2;C1QA;RPS17;RPL32;RPS8;RPS20;RPSA;RPS3A;SEPP1;TPT1
Trichloroacetic_Acid-474_mg/kg_in_Corn_Oil-Rat-Liver-5d-up	10/323	0,020372081	0,088402483	0	0	2,127818029	8,284850823	EEF1B2;C1QA;RPS17;RPS19;RPS8;RPS20;RPSA;RAPGEF5;RPL10A;TPT1
Vecuronium_Bromide-0.05_mg/kg_in_Saline-Rat-Heart-5d-up	10/323	0,020372081	0,088353803	0	0	2,127818029	8,284850823	C1QA;RPL32;RPS19;ARL2BP;RPS6;RPS20;RPSA;RPL10A;SEPP1;RPL39
24-Diaminophenol-60_mg/kg_in_Water-Rat-Kidney-3d-dn	9/277	0,020574177	0,089181186	0	0	2,233056682	8,672563658	EEF1B2;RPS17;RPL32;RPS19;RPS6;RPL35A;RPSA;RPL10A;RPL39
Acyclovir-980_mg/kg_in_Saline-Rat-Spleen-1d-dn	9/277	0,020574177	0,089132132	0	0	2,233056682	8,672563658	EEF1B2;RPS17;RPL32;RPS8;FUNDC1;RPL35A;RPS20;RPSA;TPT1
Bisphenol_A-100_mg/kg_in_Corn_Oil-Rat-Spleen-3d-up	9/277	0,020574177	0,089083131	0	0	2,233056682	8,672563658	EEF1B2;RPS17;RPL32;RPS19;RPS8;RPS5;RPS6;RPS20;RPS3A
Cyclosporin_A-350_mg/kg_in_Corn_Oil-Rat-Bone_marrow-1d-dn	9/277	0,020574177	0,089034184	0	0	2,233056682	8,672563658	EEF1B2;RPL32;RPS8;FEM1B;RPS6;ARL6IP5;RPL35A;RPSA;RPS3A
Ethacrynic_Acid-125_mg/kg_in_CMC-Rat-Kidney-3d-up	9/277	0,020574177	0,088985291	0	0	2,233056682	8,672563658	EEF1B2;RPS17;RPS19;ARL2BP;RPS8;RPSA;RPS3A;RPL10A;TPT1
Indomethacin-12_mg/kg_in_Water-Rat-Liver-1d-up	9/277	0,020574177	0,088936452	0	0	2,233056682	8,672563658	EEF1B2;RPS17;RPS19;RPS8;RPS6;RPL35A;RPS20;RPSA;RPL39
Omeprazole-415_mg/kg_in_Corn_Oil-Rat-Liver-0.25d-up	9/277	0,020574177	0,088887666	0	0	2,233056682	8,672563658	EEF1B2;RPL32;RPS19;RPS8;RPS6;RPS20;RPS3A;RPL10A;TPT1
Oxfendazole-1500_mg/kg_in_CMC-Rat-Kidney-3d-dn	9/277	0,020574177	0,088838934	0	0	2,233056682	8,672563658	RPL32;RPS19;ARL2BP;RPS8;FEM1B;RPS6;RPS20;GOLGA7;TPT1
Alfacalcidol-0.043_mg/kg_in_CMC-Rat-Heart-5d-dn	10/324	0,020761132	0,089597082	0	0	2,121250689	8,219152128	RPS17;RPL32;RPS8;FUNDC1;RPS6;ARL6IP5;RPSA;RPS3A;SEPP1;TPT1
Auranofin-33_mg/kg_in_CMC-Rat-Kidney-1d-dn	10/324	0,020761132	0,089548015	0	0	2,121250689	8,219152128	RPS19;ARL2BP;CLP1;FEM1B;RPS6;RPS20;RPSA;RPL10A;SEPP1;RPL39
Mevastatin-1200_mg/kg_in_CMC-Rat-Kidney-5d-up	10/324	0,020761132	0,089499001	0	0	2,121250689	8,219152128	EEF1B2;RPS17;CLP1;RPS8;FUNDC1;RPSA;RPS3A;RAPGEF5;SEPP1;RPL39
Pyrilamine-688_mg/kg_in_Water-Rat-Heart-1d-up	10/324	0,020761132	0,089450041	0	0	2,121250689	8,219152128	RPS17;RPL32;RPS19;RPS8;RPS6;ARL6IP5;RPS20;RPS3A;RAPGEF5;TPT1
Ritonavir-1200_mg/kg_in_CMC-Rat-Kidney-3d-up	10/324	0,020761132	0,089401135	0	0	2,121250689	8,219152128	C1QA;RPS17;RPS19;RPS6;RPSA;RPS3A;RAPGEF5;RPL10A;RPL39;TPT1
Sulpiride-1000_mg/kg_in_CMC-Rat-Heart-3d-up	10/324	0,020761132	0,089352282	0	0	2,121250689	8,219152128	RPS17;RPL32;RPS6;ARL6IP5;RPL35A;RPS20;RPSA;RPS3A;RAPGEF5;SEPP1
Flunarizine-126_mg/kg_in_CMC-Rat-Heart-1d-up	11/372	0,020860253	0,089729849	0	0	2,032295015	7,864798182	EEF1B2;RPS17;CXCL11;RPL32;RPS8;FUNDC1;RPL35A;RPS20;RPS3A;RAPGEF5;TOMM20
Bisphenol_A-100_mg/kg_in_Corn_Oil-Rat-Kidney-3d-up	9/278	0,021002021	0,090290349	0	0	2,225024104	8,595572051	RPS17;RPS8;RPS5;RPL35A;RPS20;RPSA;RPS3A;TOMM20;TPT1
Gliclazide-1500_mg/kg_in_CMC-Rat-Kidney-5d-dn	9/278	0,021002021	0,09024109	0	0	2,225024104	8,595572051	EEF1B2;RPS17;RPS19;ARL2BP;CLP1;RPS8;RPS6;RPS3A;RPL10A
Meropenem-1500_mg/kg_in_CMC-Rat-Spleen-3d-up	9/278	0,021002021	0,090191886	0	0	2,225024104	8,595572051	C1QA;RPL32;RPS8;RPS5;RPS6;RPSA;RAPGEF5;RPL10A;SEPP1
Quinapril-2500_mg/kg_in_CMC-Rat-Kidney-1d-up	9/278	0,021002021	0,090142735	0	0	2,225024104	8,595572051	RPS17;RPL32;RPS19;RPS8;RPS5;RPL35A;RPS20;RPS3A;TPT1
Levosulpiride-1200_mg/kg_in_CMC-Rat-Heart-1d-dn	8/233	0,021124238	0,09061792	0	0	2,359777591	9,102450727	RPS19;ARL2BP;RPS6;RPL35A;RPS20;RPS3A;RPL39;TPT1
Alfacalcidol-0.043_mg/kg_in_CMC-Rat-Liver-1d-dn	10/325	0,021155584	0,090702984	0	0	2,114723764	8,15406054	EEF1B2;RPS17;RPL32;RPS19;RPS6;RPL35A;RPS20;RPS3A;RPL10A;SEPP1
Gabapentin-2500_mg/kg_in_Water-Rat-Heart-5d-up	10/325	0,021155584	0,090653635	0	0	2,114723764	8,15406054	EEF1B2;C1QA;RPL32;RPS6;RPSA;RPS3A;RPL10A;SEPP1;RPL39;TPT1
Methotrexate-27_mg/kg_in_Corn_Oil-Rat-Liver-1d-dn	10/325	0,021155584	0,09060434	0	0	2,114723764	8,15406054	EEF1B2;RPS17;RPL32;RPS8;RPS6;RPSA;RPS3A;RPL10A;SEPP1;TPT1
Myrtecaine-140_mg/kg_in_Corn_Oil-Rat-Heart-5d-dn	10/325	0,021155584	0,090555099	0	0	2,114723764	8,15406054	EEF1B2;RPL32;COL5A3;ARL6IP5;RPL35A;RPS20;RPSA;RPL39;TOMM20;TPT1
Pantoprazole-1100_mg/kg_in_Water-Rat-Liver-3d-up	10/325	0,021155584	0,090505911	0	0	2,114723764	8,15406054	RPS17;RPL32;RPS19;RPS8;RPS5;RPS6;ARL6IP5;RPL35A;RPSA;RPS3A
Troglitazone-1200_mg/kg_in_Corn_Oil-Rat-Liver-1d-dn	10/325	0,021155584	0,090456776	0	0	2,114723764	8,15406054	EEF1B2;RPS17;RPL32;RPS19;RPS6;ARL6IP5;RPS20;RPSA;RPL10A;RPL39
Trichloroethane-300_mg/kg_in_Water-Rat-Liver-0.25d-up	11/373	0,021226751	0,090711823	0	0	2,026846503	7,808412096	EEF1B2;C1QA;RPS17;RPL32;RPS19;RPS8;RPS5;RPS6;RPSA;SEPP1;TPT1
Aminoglutethimide-350_mg/kg_in_CMC-Rat-Liver-3d-dn	9/279	0,021436244	0,091557405	0	0	2,217049108	8,519392881	EEF1B2;C1QA;RPL32;RPS8;RPS6;RPL35A;RPS3A;SEPP1;TPT1
Enalapril-1500_mg/kg_in_CMC-Rat-Kidney-5d-up	9/279	0,021436244	0,091507781	0	0	2,217049108	8,519392881	RPL32;RPS19;RPS8;RPS6;ARL6IP5;RPL13A;RPL35A;RPSA;RPL39
Sulindac-23_mg/kg_in_Corn_Oil-Rat-Liver-5d-up	9/279	0,021436244	0,09145821	0	0	2,217049108	8,519392881	EEF1B2;RPS17;RPL32;RPS19;RPS6;RPS20;RPS3A;RPL39;TPT1
Chlordiazepoxide-196_mg/kg_in_Water-Rat-Heart-1d-up	10/326	0,021555477	0,091917128	0	0	2,108236881	8,089569174	RPS17;RPL32;ARL2BP;RPS8;RPS5;RPS6;RPS20;RPSA;RPS3A;TPT1
Chlorpromazine-73_mg/kg_in_Water-Rat-Liver-5d-dn	10/326	0,021555477	0,09186739	0	0	2,108236881	8,089569174	C1QA;RPS17;RPL32;RPS19;RPS8;RPL35A;RPSA;RPL10A;RPL39;TPT1
Clotrimazole-60_uM_in_DMSO-Rat-Primary_rat_hepatocytes-1d-dn	10/326	0,021555477	0,091817705	0	0	2,108236881	8,089569174	RPS17;RPL32;RPS19;RPS8;RPS6;ARL6IP5;RPSA;SEPP1;RPL39;TPT1
Fenofibrate-250_uM_in_DMSO-Rat-Primary_rat_hepatocytes-0.67d-dn	10/326	0,021555477	0,091768074	0	0	2,108236881	8,089569174	EEF1B2;CXCL11;RPS19;RPS5;ARL6IP5;RPS20;RPSA;RPL10A;SEPP1;RPL39
Gentamicin-40_mg/kg_in_Saline-Rat-Kidney-28d-up	10/326	0,021555477	0,091718496	0	0	2,108236881	8,089569174	EEF1B2;C1QA;RPS19;CLP1;RPS8;FUNDC1;RPS20;RPSA;RPL10A;RPL39
Hydroxyurea-400_mg/kg_in_Saline-Rat-Spleen-5d-dn	10/326	0,021555477	0,091668972	0	0	2,108236881	8,089569174	EEF1B2;RPS17;RPL32;RPS19;RPS8;RPS5;RPL35A;RPS20;RPS3A;RPL10A
Mycophenolate_Mofetil-63_mg/kg_in_CMC-Rat-Bone_marrow-5d-up	10/326	0,021555477	0,091619501	0	0	2,108236881	8,089569174	C1QA;RPL32;RPS8;ARL6IP5;NKG7;RPS20;RPSA;RPS3A;RPL10A;SEPP1
Phenylbutazone-368_mg/kg_in_CMC-Rat-Kidney-5d-up	10/326	0,021555477	0,091570084	0	0	2,108236881	8,089569174	CXCL11;RPL32;FEM1B;RPS6;RPSA;RPL10A;SEPP1;RPL39;GOLGA7;TPT1
Rofecoxib-3_mg/kg_in_Corn_Oil-Rat-Liver-3d-up	10/326	0,021555477	0,09152072	0	0	2,108236881	8,089569174	C1QA;RPL32;RPS19;RPS6;RPL35A;RPS20;RPL10A;SEPP1;RPL39;GOLGA7
Zileuton-450_mg/kg_in_Corn_Oil-Rat-Liver-0.25d-dn	10/326	0,021555477	0,09147141	0	0	2,108236881	8,089569174	EEF1B2;C1QA;RPS17;RPL32;RPS19;RPS20;RPSA;RPL10A;RPL39;TPT1
33'4'5-Tetrachlorosalicylanilide-5_mg/kg_in_Corn_Oil-Rat-Liver-1d-up	9/280	0,021876892	0,092785354	0	0	2,209131075	8,444015505	C1QA;RPL32;RPS8;RPS5;RPS6;RPL35A;RPSA;RPL10A;TPT1
Azithromycin-225_mg/kg_in_Water-Rat-Kidney-1d-dn	9/280	0,021876892	0,092735416	0	0	2,209131075	8,444015505	RPS17;RPL32;RPS19;RPS8;FUNDC1;RPL35A;RPS20;RPSA;SEPP1
Benzothiazyl_Disulfide-2600_mg/kg_in_Corn_Oil-Rat-Kidney-5d-dn	9/280	0,021876892	0,092685531	0	0	2,209131075	8,444015505	EEF1B2;RPL32;RPS8;RPS6;RPS20;RPS3A;RPL10A;RPL39;PDRG1
Ceramide_C2-44_uM_in_DMSO-Rat-Primary_rat_hepatocytes-1d-up	9/280	0,021876892	0,0926357	0	0	2,209131075	8,444015505	RPS17;RPS5;ARL6IP5;RPL35A;RPS20;RPSA;RPL10A;GOLGA7;PDRG1
Chlorzoxazone-763_mg/kg_in_CMC-Rat-Liver-5d-up	9/280	0,021876892	0,092585923	0	0	2,209131075	8,444015505	RPS17;ARL2BP;CLP1;RPS8;RPS5;RPS6;RPS20;RPSA;RPS3A
Ethylene_Glycol-352_mg/kg_in_Water-Rat-Liver-1d-dn	9/280	0,021876892	0,092536199	0	0	2,209131075	8,444015505	RPS19;RPS8;FUNDC1;RPS6;RPSA;RPL10A;RPL39;SEPP1;TPT1
Oxybutynin-230_mg/kg_in_Water-Rat-Heart-5d-up	9/280	0,021876892	0,092486529	0	0	2,209131075	8,444015505	C1QA;RPS17;RPL32;RPS19;RPSA;RPS3A;RPL39;SEPP1;TPT1
Zidovudine-1540_mg/kg_in_Corn_Oil-Rat-Liver-0.25d-up	9/280	0,021876892	0,092436911	0	0	2,209131075	8,444015505	C1QA;RPS17;RPL32;RPS19;RPS8;RPSA;RPL10A;SEPP1;TPT1
Bromisovalum-250_mg/kg_in_Corn_Oil-Rat-Heart-5d-dn	10/327	0,021960849	0,092741903	0	0	2,101789674	8,02567124	RPS17;ARL2BP;RPS8;FUNDC1;RPS6;RPS20;RPSA;RPS3A;SEPP1;TPT1
Cytarabine-487_mg/kg_in_Saline-Rat-Kidney-3d-dn	10/327	0,021960849	0,092692202	0	0	2,101789674	8,02567124	EEF1B2;RPS17;RPL32;RPS6;RPL35A;RPS20;RPL10A;RPL39;GOLGA7;TPT1
Doxepin-132_uM_in_DMSO-Rat-Primary_rat_hepatocytes-1d-dn	10/327	0,021960849	0,092642554	0	0	2,101789674	8,02567124	C1QA;CXCL11;RPL32;RPS19;RPS6;ARL6IP5;SEPP1;RPL39;SLC16A3;TPT1
Fluvastatin-94_mg/kg_in_Corn_Oil-Rat-Liver-5d-dn	10/327	0,021960849	0,09259296	0	0	2,101789674	8,02567124	EEF1B2;RPS17;RPL32;RPS19;RPS8;RPSA;RPS3A;RPL10A;SEPP1;TPT1
Glipizide-2500_mg/kg_in_CMC-Rat-Liver-0.25d-up	10/327	0,021960849	0,092543418	0	0	2,101789674	8,02567124	C1QA;RPS17;RPL32;RPS19;RPS8;RPS6;RPS20;RPSA;RPS3A;RPL39
Lisinopril-2000_mg/kg_in_CMC-Rat-Kidney-5d-up	10/327	0,021960849	0,09249393	0	0	2,101789674	8,02567124	C1QA;RPS8;FEM1B;RPS20;RPSA;RAPGEF5;SEPP1;RPL39;TOMM20;TPT1
Methyldopa-325_mg/kg_in_Water-Rat-Liver-3d-dn	10/327	0,021960849	0,092444494	0	0	2,101789674	8,02567124	C1QA;RPS17;RPL32;RPS19;FEM1B;RPS6;RPS3A;RPL10A;RPL39;TPT1
Allyl_Alcohol-30_uM_in_DMSO-Rat-Primary_rat_hepatocytes-1d-up	9/281	0,022324014	0,093923043	0	0	2,201269399	8,369429453	RPS17;CXADR;RPS8;ARL6IP5;RPS20;RPSA;RPS3A;RPL10A;RPL39
Alprazolam-115_mg/kg_in_Corn_Oil-Rat-Liver-5d-dn	9/281	0,022324014	0,093872897	0	0	2,201269399	8,369429453	EEF1B2;RPS19;RPS8;RPS6;RPS20;RPS3A;RPL10A;RPL39;TPT1
Clofibrate-500_mg/kg_in_Corn_Oil-Rat-Kidney-3d-up	9/281	0,022324014	0,093822805	0	0	2,201269399	8,369429453	RPS17;RPS19;ARL2BP;CLP1;RPL35A;RPS20;RPS3A;RPL39;GOLGA7
Doxorubicin-3_mg/kg_in_Saline-Rat-Bone_marrow-1d-dn	9/281	0,022324014	0,093772766	0	0	2,201269399	8,369429453	RPS17;RPL32;RPS8;FEM1B;RPSA;RPS3A;RPL10A;RPL39;TPT1
Marimastat-1000_uM_in_DMSO-Rat-Primary_rat_hepatocytes-1d-dn	10/328	0,02237174	0,093923149	0	0	2,095381779	7,962360042	CXCL11;RPL32;RPS19;ARL2BP;RPS5;RPS6;ARL6IP5;RPL10A;SEPP1;GOLGA7
Penicillamine-100_mg/kg_in_Water-Rat-Bone_marrow-3d-up	10/328	0,02237174	0,09387311	0	0	2,095381779	7,962360042	EEF1B2;C1QA;RPS17;RPL32;RPS19;NKG7;RPS3A;RPL10A;SEPP1;RPL39
Phenylhydrazine-78_mg/kg_in_Water-Rat-Liver-0.25d-up	10/328	0,02237174	0,093823124	0	0	2,095381779	7,962360042	RPL32;RPS19;RPS8;RPS6;ARL6IP5;RPS20;RPSA;RPL10A;SEPP1;GOLGA7
Theophylline-225_mg/kg_in_Water-Rat-Heart-5d-dn	10/328	0,02237174	0,093773192	0	0	2,095381779	7,962360042	EEF1B2;RPL32;RPS19;ARL2BP;RPS6;ARL6IP5;RPSA;RPS3A;RPL10A;RPL39
Sulfathiazole-2629_mg/kg_in_Water-Rat-Kidney-5d-dn	8/236	0,022595424	0,094660403	0	0	2,329780418	8,829886134	RPS17;RPL32;RPS19;RPS6;ARL6IP5;RPS20;RPSA;RPL39
Cyclosporin_A-70_mg/kg_in_Corn_Oil-Rat-Kidney-1d-up	11/377	0,022740308	0,09521673	0	0	2,005341501	7,587442651	C1QA;RPS17;RPL32;RPS19;RPS8;RPS5;RPS6;RPSA;RPS3A;RPL39;TPT1
Roflumilast-17_mg/kg_in_CMC-Rat-Heart-1d-dn	9/282	0,022777657	0,095322438	0	0	2,193463479	8,295624424	RPL32;RPS19;RPS6;RPS20;RPSA;RPL10A;RPL39;GOLGA7;SEPP1
Ampicillin-2000_mg/kg_in_CMC-Rat-Kidney-3d-up	10/329	0,022788189	0,095315867	0	0	2,089012837	7,899628977	EEF1B2;RPS17;RPL32;RPS6;ARL6IP5;RPL13A;RPL35A;RPS3A;RAPGEF5;RPL10A
Atorvastatin-2.5_mg/kg_in_Corn_Oil-Rat-Liver-1d-up	10/329	0,022788189	0,095265275	0	0	2,089012837	7,899628977	EEF1B2;RPS17;RPL32;RPS19;RPS20;RPSA;RPS3A;RPL10A;RPL39;TPT1
Diethylstilbestrol-2.8_mg/kg_in_Corn_Oil-Rat-Spleen-1d-up	10/329	0,022788189	0,095214737	0	0	2,089012837	7,899628977	EEF1B2;C1QA;RPS17;RPL32;FUNDC1;RPS20;RPSA;RPL10A;SEPP1;TPT1
Cytarabine-487_mg/kg_in_Saline-Rat-Liver-1d-dn	8/237	0,023101495	0,096472627	0	0	2,319950121	8,741242499	EEF1B2;RPL32;RPS19;RPS8;RPS6;RPS20;RPS3A;SEPP1
Artemisinin-2000_mg/kg_in_CMC-Rat-Liver-3d-up	10/330	0,023210234	0,096875361	0	0	2,082682495	7,837471535	RPS17;RPL32;RPS19;RPS8;RPS6;RPL35A;RPS20;RPSA;RPL10A;RPL39

Beta-Estradiol-150_mg/kg_in_Corn_Oil-Rat-Skeletal_muscle-3d-dn	10/330	0,023210234	0,09682405	0	0	2,082682495	7,837471535	RPS17;RPL32;RPS8;FEM1B;RPS5;ARL6IP5;RPSA;RPS3A;SEPP1;SLC16A3
Fluvastatin-5_mg/kg_in_Corn_Oil-Rat-Liver-3d-up	10/330	0,023210234	0,096772793	0	0	2,082682495	7,837471535	EEF1B2;RPL32;RPS19;RPS8;RPS6;RPL35A;RPS20;RPSA;RPL39;TPT1
Phenylhydrazine-78_mg/kg_in_Water-Rat-Liver-1d-up	10/330	0,023210234	0,096721591	0	0	2,082682495	7,837471535	EEF1B2;RPS17;RPL32;RPS5;RPS6;RPS20;RPSA;RPS3A;RPL39;TPT1
Amlodipine-0.2_mg/kg_in_Corn_Oil-Rat-Liver-0.25d-up	9/283	0,023237868	0,096785535	0	0	2,185712724	8,222590282	EEF1B2;RPS17;RPS19;FEM1B;RPS20;RPS3A;RPL10A;RPL39;TPT1
Doxorubicin-3_mg/kg_in_Saline-Rat-Spleen-5d-up	9/283	0,023237868	0,09673438	0	0	2,185712724	8,222590282	CXCL11;RPL32;RPS19;CLP1;RPS8;FUNDC1;RPS3A;SEPP1;TPT1
Famciclovir-1200_mg/kg_in_Saline-Rat-Spleen-1d-dn	9/283	0,023237868	0,096683279	0	0	2,185712724	8,222590282	EEF1B2;CXCL11;ARL2BP;RPS8;RPS5;FUNDC1;RPL13A;RPL35A;RPSA
Isotretinoin-13_mg/kg_in_Corn_Oil-Rat-Liver-5d-up	9/283	0,023237868	0,096632232	0	0	2,185712724	8,222590282	EEF1B2;RPS17;RPL32;RPS8;RPL35A;RPS20;RPS3A;RPL39;TPT1
Leflunomide-60_mg/kg_in_Corn_Oil-Rat-Spleen-1d-dn	9/283	0,023237868	0,096581239	0	0	2,185712724	8,222590282	EEF1B2;RPS17;RPS19;RPS5;RPS6;RPL13A;RPL35A;RPL10A;RPL39
NN-Dimethylformamide-140_mg/kg_in_Saline-Rat-Kidney-3d-up	11/379	0,023526085	0,097727554	0	0	1,994759223	7,47963992	RPS17;ARL2BP;RPL35A;RPS20;RPSA;RPS3A;SEPP1;RPL39;GOLGA7;TOMM20;TPT1
Azathioprine-54_mg/kg_in_Water-Rat-Liver-3d-up	10/331	0,023637914	0,098140334	0	0	2,076390403	7,775881294	EEF1B2;RPS17;RPL32;RPS19;RPS6;RPL35A;RPS20;RPS3A;SEPP1;TPT1
Cisapride-250_mg/kg_in_Corn_Oil-Rat-Heart-5d-dn	10/331	0,023637914	0,098088627	0	0	2,076390403	7,775881294	EEF1B2;ARL2BP;RPS8;RPS6;RPL35A;RPSA;RPS3A;RPL39;TOMM20;TPT1
Epirubicin-2.7_mg/kg_in_Saline-Rat-Heart-3d-up	10/331	0,023637914	0,098036974	0	0	2,076390403	7,775881294	CXCL11;RPS19;RPS6;RPS20;RAPGEF5;RPL10A;SEPP1;RPL39;GOLGA7;TPT1
Finasteride-25_mg/kg_in_Corn_Oil-Rat-Liver-0.25d-up	10/331	0,023637914	0,097985376	0	0	2,076390403	7,775881294	EEF1B2;RPL32;RPS19;RPS8;RPS6;RPS20;RPSA;RPS3A;RPL10A;TPT1
Gatifloxacin-770_mg/kg_in_Corn_Oil-Rat-Heart-5d-dn	10/331	0,023637914	0,097933832	0	0	2,076390403	7,775881294	EEF1B2;RPS17;RPL32;ARL2BP;RPS8;RPS6;RPSA;RPS3A;SEPP1;TPT1
Nadolol-1500_mg/kg_in_CMC-Rat-Heart-1d-up	10/331	0,023637914	0,097882342	0	0	2,076390403	7,775881294	EEF1B2;C1QA;RPS17;CXCL11;FUNDC1;ARL6IP5;RPSA;RPS3A;RPL10A;PDRG1
Thioridazine-63_mg/kg_in_Corn_Oil-Rat-Heart-1d-up	10/331	0,023637914	0,097830906	0	0	2,076390403	7,775881294	RPS19;ARL2BP;FUNDC1;RPS6;RPS20;RPSA;RAPGEF5;RPL10A;RPL39;TOMM20
Cisplatin-2_mg/kg_in_Saline-Rat-Liver-0.25d-up	9/284	0,023704693	0,098055757	0	0	2,178016553	8,150317054	C1QA;RPS17;RPL32;RPS8;RPS5;RPL35A;RPSA;RPS3A;TPT1
Epirubicin-2.7_mg/kg_in_Saline-Rat-Liver-1d-dn	9/284	0,023704693	0,098004284	0	0	2,178016553	8,150317054	RPS17;RPL32;RPS19;RPS8;RPS5;RPS6;RPL10A;RPL39;TPT1
Ifosfamide-11000_uM_in_DMSO-Rat-Primary_rat_hepatocytes-0.67d-dn	9/284	0,023704693	0,097952865	0	0	2,178016553	8,150317054	RPS17;RPL32;RPS8;RPS5;RPS6;RPL35A;RPS3A;TPT1;PDRG1
Irinotecan-5_mg/kg_in_Saline-Rat-Heart-5d-dn	9/284	0,023704693	0,097901501	0	0	2,178016553	8,150317054	EEF1B2;RPS17;RPL32;RPS19;RPS8;RPS5;RPS6;RPSA;RAPGEF5
Isoeugenol-1560_mg/kg_in_CMC-Rat-Liver-3d-up	9/284	0,023704693	0,097850189	0	0	2,178016553	8,150317054	RPS17;RPS19;RPS6;RPSA;RPS3A;RAPGEF5;RPL10A;RPL39;TPT1
Isoprenaline-4.2_mg/kg_in_Saline-Rat-Heart-1d-up	9/284	0,023704693	0,097798932	0	0	2,178016553	8,150317054	EEF1B2;RPS19;RPS8;RPS5;RPS6;RPS20;RPSA;RPL10A;RPL39
Mebendazole-50_mg/kg_in_Corn_Oil-Rat-Liver-1d-dn	9/284	0,023704693	0,097747729	0	0	2,178016553	8,150317054	EEF1B2;RPL32;RPS19;ARL2BP;RPS6;RPS3A;RPL10A;RPL39;TPT1
Nevirapine-200_mg/kg_in_Saline-Rat-Liver-0.25d-up	9/284	0,023704693	0,097696578	0	0	2,178016553	8,150317054	RPS17;RPL32;RPS19;RPS6;RPSA;RPS3A;RPL10A;RPL39;SEPP1
Sulfadiazine-1170_mg/kg_in_Water-Rat-Kidney-5d-dn	9/284	0,023704693	0,097645482	0	0	2,178016553	8,150317054	RPS17;RPL32;RPS19;RPS6;RPS20;RPSA;RPL10A;RPL39;SEPP1
Torsemide-110_mg/kg_in_Corn_Oil-Rat-Liver-1d-dn	9/284	0,023704693	0,097594439	0	0	2,178016553	8,150317054	EEF1B2;RPS17;RPL32;RPS19;RPS6;RPL35A;RPS20;RPS3A;RPL10A
Venlafaxine-1481_uM_in_DMSO-Rat-Primary_rat_hepatocytes-1d-up	9/284	0,023704693	0,097543449	0	0	2,178016553	8,150317054	EEF1B2;RPS17;RPS19;FEM1B;RPS6;RPS3A;RPL10A;TPT1;PDRG1
Dexamethasone-456_uM_in_DMSO-Rat-Primary_rat_hepatocytes-1d-dn	10/332	0,024071268	0,099000158	0	0	2,070136215	7,714851922	CXCL11;RPS19;ARL6IP5;RPSA;RPS3A;SEPP1;RPL39;GOLGA7;TPT1;PDRG1
N-Nitrosodiethylamine-34_mg/kg_in_Saline-Rat-Spleen-3d-up	10/332	0,024071268	0,098948488	0	0	2,070136215	7,714851922	CXCL11;RPS19;RPS5;RPL35A;RPS20;RPS3A;RPL10A;SEPP1;GOLGA7;TPT1
Pantoprazole-1100_mg/kg_in_Water-Rat-Liver-5d-up	10/332	0,024071268	0,098896872	0	0	2,070136215	7,714851922	RPS17;RPL32;RPS8;RPS5;RPS6;RPL35A;RPSA;RPS3A;SEPP1;GOLGA7
Torsemide-110_mg/kg_in_Corn_Oil-Rat-Liver-5d-up	10/332	0,024071268	0,098845309	0	0	2,070136215	7,714851922	EEF1B2;RPS17;RPL32;RPS19;CLP1;RPS8;RPL35A;RPS20;RPL39;TPT1
Zidovudine-1540_mg/kg_in_Corn_Oil-Rat-Liver-5d-up	10/332	0,024071268	0,0987938	0	0	2,070136215	7,714851922	RPS17;RPL32;RPS19;RPS8;RPS5;RPL35A;RPS20;SEPP1;RPL39;TPT1
D-Galactosamine-470_uM_in_DMSO-Rat-Primary_rat_hepatocytes-1d-up	8/239	0,024137499	0,099014032	0	0	2,300536313	8,567171169	EEF1B2;RPL32;ARL2BP;CLP1;RPS8;RPS20;RPS3A;TPT1
Bisphenol_A-100_mg/kg_in_Corn_Oil-Rat-Spleen-0.25d-up	9/285	0,024178179	0,099129274	0	0	2,17037439	8,078794925	EEF1B2;CXCL11;RPS19;RPS8;RPS5;FUNDC1;RPS20;RPSA;RPL10A
Bleomycin_A2-12_mg/kg_in_Saline-Rat-Bone_marrow-5d-up	9/285	0,024178179	0,099077698	0	0	2,17037439	8,078794925	RPL32;RPS19;ARL2BP;RPS8;FUNDC1;RPS6;RPS20;RPSA;RPS3A
Chlorambucil-4.5_mg/kg_in_Corn_Oil-Rat-Bone_marrow-1d-dn	9/285	0,024178179	0,099026175	0	0	2,17037439	8,078794925	RPS17;RPL32;RPS19;RPS8;FEM1B;RPS6;ARL6IP5;RPSA;RPS3A
Chlorobenzene-500_mg/kg_in_CMC-Rat-Kidney-1d-dn	9/285	0,024178179	0,098974706	0	0	2,17037439	8,078794925	EEF1B2;RPS19;ARL2BP;FEM1B;FUNDC1;RPS6;RPSA;RPL10A;RPL39
Ciprofloxacin-72_mg/kg_in_Corn_Oil-Rat-Liver-1d-dn	9/285	0,024178179	0,098923291	0	0	2,17037439	8,078794925	EEF1B2;RPS17;RPL32;CLP1;RPS6;ARL6IP5;RPL10A;SEPP1;TPT1
Enoxacin-100_mg/kg_in_Corn_Oil-Rat-Liver-1d-up	9/285	0,024178179	0,098871929	0	0	2,17037439	8,078794925	RPS17;RPS19;RPS8;FEM1B;RPS6;RPS20;RPSA;RPL39;GOLGA7
NN-Dimethylformamide-1400_mg/kg_in_Saline-Rat-Kidney-5d-up	9/285	0,024178179	0,09882062	0	0	2,17037439	8,078794925	RPS17;RPL32;RPS19;RPS6;RPL35A;RPS20;RPSA;RPS3A;RPL39
Terbutaline-130_mg/kg_in_Corn_Oil-Rat-Heart-5d-up	9/285	0,024178179	0,098769365	0	0	2,17037439	8,078794925	C1QA;RPS20;RPSA;RAPGEF5;RPL10A;RPL39;SEPP1;TPT1;PDRG1
Thioguanine-12_mg/kg_in_Corn_Oil-Rat-Bone_marrow-0.25d-dn	9/285	0,024178179	0,098718162	0	0	2,17037439	8,078794925	RPS17;RPL32;RPS19;RPS8;FEM1B;RPS6;RPSA;RPS3A;TPT1
Hydroxyurea-59_mg/kg_in_Saline-Rat-Liver-3d-up	11/381	0,024331538	0,099292845	0	0	1,984288046	7,373578519	EEF1B2;RPS17;RPL32;RPS19;RPS6;RPS20;RPSA;RPS3A;RPL10A;RPL39;TPT1
4-Nonylphenol-200_mg/kg_in_Corn_Oil-Rat-Liver-5d-dn	10/333	0,024510332	0,099970674	0	0	2,06391959	7,654377173	EEF1B2;C1QA;RPS17;RPL32;FEM1B;RPL35A;RPS20;RPS3A;SEPP1;RPL39
44'-Diethylaminoethoxyhexestrol-20_uM_in_DMSO-Rat-Primary_rat_hepatocytes-0.67d-up	10/333	0,024510332	0,099918929	0	0	2,06391959	7,654377173	EEF1B2;RPS19;RPS6;ARL6IP5;RPSA;RPL10A;SEPP1;RPL39;GOLGA7;TPT1
Amoxicillin-1100_mg/kg_in_Water-Rat-Liver-3d-up	10/333	0,024510332	0,099867238	0	0	2,06391959	7,654377173	EEF1B2;C1QA;RPS17;RPL32;RPS8;RPS5;RPL35A;SEPP1;RPL39;TPT1
Sotalol-6666_uM_in_DMSO-Rat-Primary_rat_hepatocytes-1d-dn	10/333	0,024510332	0,0998156	0	0	2,06391959	7,654377173	CXCL11;RPL32;RPS19;ARL6IP5;RPS20;RPSA;RPS3A;SEPP1;RPL39;TPT1
5-Fluoro-2'-Deoxyuridine-54_mg/kg_in_Saline-Rat-Bone_marrow-5d-up	9/286	0,024658371	0,100366578	0	0	2,162785668	8,008014238	C1QA;RPL32;ARL2BP;CLP1;RPS8;FUNDC1;RPS6;RPS3A;TPT1
Allyl_Alcohol-16_mg/kg_in_Saline-Rat-Bone_marrow-0.25d-dn	9/286	0,024658371	0,100314736	0	0	2,162785668	8,008014238	EEF1B2;RPS17;RPL32;RPS8;RPS3A;RPL10A;RPL39;SEPP1;TPT1
Altretamine-40_mg/kg_in_Corn_Oil-Rat-Bone_marrow-3d-up	9/286	0,024658371	0,100262947	0	0	2,162785668	8,008014238	C1QA;RPL32;RPS8;RPS5;NKG7;RPL35A;RPS20;GOLGA7;SEPP1
Ampiroxicam-94_mg/kg_in_CMC-Rat-Kidney-3d-up	9/286	0,024658371	0,100211212	0	0	2,162785668	8,008014238	C1QA;CXCL11;RPL32;RPS6;RPS20;RPS3A;RPL10A;SEPP1;TPT1
Cytarabine-23_mg/kg_in_Saline-Rat-Spleen-1d-dn	9/286	0,024658371	0,10015953	0	0	2,162785668	8,008014238	C1QA;RPS17;RPL32;RPS8;RPS5;FUNDC1;RPL35A;RPS3A;TPT1
Hydralazine-280_mg/kg_in_Water-Rat-Liver-3d-dn	9/286	0,024658371	0,100107901	0	0	2,162785668	8,008014238	EEF1B2;RPS17;RPL32;RPS19;RPS6;RPS20;RPL10A;RPL39;SEPP1
Lead_(II)_Acetate-200_mg/kg_in_Saline-Rat-Liver-0.25d-up	9/286	0,024658371	0,100056326	0	0	2,162785668	8,008014238	EEF1B2;RPS17;RPL32;RPS19;RPS5;RPS6;RPL35A;RPSA;RPS3A
Sparfloxacin-29_mg/kg_in_Corn_Oil-Rat-Liver-0.25d-up	9/286	0,024658371	0,100004804	0	0	2,162785668	8,008014238	EEF1B2;RPS17;RPL32;RPS19;RPS6;RPS20;RPS3A;RPL10A;SEPP1
Torsemide-3_mg/kg_in_Corn_Oil-Rat-Liver-3d-dn	9/286	0,024658371	0,099953334	0	0	2,162785668	8,008014238	EEF1B2;C1QA;RPS17;RPL32;RPS19;CLP1;RPS6;RPS3A;RPL10A
Azathioprine-20_mg/kg_in_Corn_Oil-Rat-Kidney-1d-dn	8/240	0,024667546	0,09993909	0	0	2,290950745	8,481710952	EEF1B2;RPS19;ARL2BP;CLP1;RPS8;RPL35A;RPSA;TOMM20
Anisindione-75_mg/kg_in_CMC-Rat-Liver-3d-up	10/334	0,024955144	0,101052296	0	0	2,05774019	7,594450887	EEF1B2;C1QA;RPS17;RPS19;RPS8;RPS6;RPS20;RPS3A;RPL39;TPT1
Atenolol-1000_mg/kg_in_CMC-Rat-Heart-1d-dn	10/334	0,024955144	0,101000368	0	0	2,05774019	7,594450887	EEF1B2;RPS17;RPL32;RPS19;RPS8;RPS20;RPSA;RPL10A;SEPP1;RPL39
Cyproterone_Acetate-2500_mg/kg_in_CMC-Rat-Liver-5d-dn	10/334	0,024955144	0,100948493	0	0	2,05774019	7,594450887	EEF1B2;C1QA;RPL32;RPS19;CLP1;RPS6;RPSA;RPS3A;RPL10A;SEPP1
Doxorubicin-3_mg/kg_in_Saline-Rat-Liver-5d-up	10/334	0,024955144	0,100896671	0	0	2,05774019	7,594450887	EEF1B2;RPS19;RPS8;RPS6;RPS20;RPSA;RPS3A;RPL10A;SEPP1;RPL39
Sparfloxacin-450_mg/kg_in_Corn_Oil-Rat-Liver-5d-up	10/334	0,024955144	0,100844903	0	0	2,05774019	7,594450887	EEF1B2;RPS17;RPS19;RPS20;RPSA;RPS3A;RPL10A;SEPP1;RPL39;TPT1
Stannous_Fluoride-90_mg/kg_in_Water-Rat-Kidney-5d-up	10/334	0,024955144	0,100793188	0	0	2,05774019	7,594450887	EEF1B2;RPS17;RPL32;ARL2BP;RPS6;RPS3A;RAPGEF5;RPL10A;RPL39;TPT1
Albendazole-62_mg/kg_in_Corn_Oil-Rat-Liver-3d-dn	9/287	0,025145315	0,101509226	0	0	2,155249829	7,937965489	EEF1B2;RPS17;RPL32;RPS19;CLP1;RPS6;RPS20;RPL39;SEPP1
Daunorubicin-3.25_mg/kg_in_Saline-Rat-Kidney-5d-dn	9/287	0,025145315	0,101457223	0	0	2,155249829	7,937965489	C1QA;RPS17;RPL32;RPS8;RPL35A;RPSA;RPS3A;SEPP1;TPT1
Itraconazole-10_uM_in_DMSO-Rat-Primary_rat_hepatocytes-0.67d-dn	9/287	0,025145315	0,101405274	0	0	2,155249829	7,937965489	RPS17;RPL32;RPS8;RPS5;RPL35A;RPS20;RPS3A;TPT1;PDRG1
Ketorolac-48_mg/kg_in_Water-Rat-Liver-5d-up	9/287	0,025145315	0,101353378	0	0	2,155249829	7,937965489	EEF1B2;RPS19;RPS8;RPS6;RPS20;RPSA;RPS3A;RPL10A;RPL39
Olanzapine-23_mg/kg_in_CMC-Rat-Heart-1d-dn	9/287	0,025145315	0,101301534	0	0	2,155249829	7,937965489	RPS17;RPL32;ARL2BP;RPS5;COL5A3;RPS6;RPL35A;RPSA;RPS3A
Prazosin-2000_mg/kg_in_CMC-Rat-Heart-5d-up	9/287	0,025145315	0,101249744	0	0	2,155249829	7,937965489	RPL32;RPS19;FEM1B;FUNDC1;RPS6;RPSA;RAPGEF5;GOLGA7;SEPP1
Rolipram-75_mg/kg_in_CMC-Rat-Heart-1d-dn	9/287	0,025145315	0,101198007	0	0	2,155249829	7,937965489	RPS17;RPL32;RPS19;COL5A3;RPS6;RPSA;RPL10A;RPL39;SEPP1
Phenylhydrazine-78_mg/kg_in_Water-Rat-Liver-3d-up	10/335	0,025405743	0,102193888	0	0	2,051597682	7,535066987	C1QA;RPS17;RPS19;RPS8;RPS5;RPS6;RPSA;RPS3A;RAPGEF5;TPT1
Rolipram-75_mg/kg_in_CMC-Rat-Liver-1d-dn	10/335	0,025405743	0,102141721	0	0	2,051597682	7,535066987	EEF1B2;C1QA;RPS17;RPL32;RPS19;RPS6;RPS20;RPSA;RPL10A;RPL39
Zomepirac-11_mg/kg_in_Saline-Rat-Liver-0.25d-dn	10/335	0,025405743	0,102089608	0	0	2,051597682	7,535066987	EEF1B2;RPS17;RPL32;RPS19;RPS6;ARL6IP5;RPS20;RPL10A;RPL39;TPT1
Acyclovir-980_mg/kg_in_Saline-Rat-Liver-3d-up	9/288	0,025639056	0,102974607	0	0	2,147766323	7,868639324	EEF1B2;C1QA;RPS17;RPS19;CLP1;RPS6;RPS20;RPSA;RPS3A
Chlorambucil-1540_uM_in_DMSO-Rat-Primary_rat_hepatocytes-1d-up	9/288	0,025639056	0,102922122	0	0	2,147766323	7,868639324	RPS17;CXCL11;RPL32;RPS6;ARL6IP5;RPL35A;RPS3A;RPL10A;PDRG1
Lovastatin-450_mg/kg_in_Corn_Oil-Rat-Liver-0.25d-up	9/288	0,025639056	0,102869691	0	0	2,147766323	7,868639324	EEF1B2;RPS17;RPL32;RPS19;RPS8;RPS6;RPS20;RPL10A;SEPP1
Cytarabine-23_mg/kg_in_Saline-Rat-Liver-1d-up	10/336	0,025862165	0,103712023	0	0	2,045491736	7,476219481	EEF1B2;RPS17;RPL32;CLP1;RPS8;RPL13A;RPL35A;RPS20;RPL39;TPT1
Pralidoxime_Chloride-48_mg/kg_in_Saline-Rat-Liver-3d-dn	10/336	0,025862165	0,103659243	0	0	2,045491736	7,476219481	EEF1B2;RPS17;RPL32;RPS19;RPL35A;RPS20;RPSA;RPS3A;SEPP1;RPL39
Tosufloxacin-2000_mg/kg_in_CMC-Rat-Liver-1d-dn	10/336	0,025862165	0,103606518	0	0	2,045491736	7,476219481	EEF1B2;C1QA;RPS17;RPL32;RPS5;ARL6IP5;RPL35A;RPS3A;RPL10A;SEPP1
TGF_Beta-1_Human_Recombinant-5_ng/ml_in_DMSO-Rat-Primary_rat_hepatocytes-1d-dn	11/385	0,026002492	0,104115723	0	0	1,963672067	7,166544718	RPS17;RPS19;ARL2BP;RPS8;FUNDC1;RPS6;RPS20;RPSA;RPL10A;RPL39;TPT1
Acetaminophen-100_mg/kg_in_Corn_Oil-Rat-Liver-5d-dn	9/289	0,026139639	0,104611684	0	0	2,140334606	7,800026535	C1QA;RPS17;CLP1;RPS8;RPS6;RPS20;RPS3A;RPL39;TPT1
Altretamine-13_mg/kg_in_Corn_Oil-Rat-Bone_marrow-0.25d-dn	9/289	0,026139639	0,104558554	0	0	2,140334606	7,800026535	RPS17;RPL32;RPS19;RPS8;RPS6;ARL6IP5;RPSA;RPS3A;TPT1
Aspirin-167_mg/kg_in_Corn_Oil-Rat-Liver-3d-dn	9/289	0,026139639	0,104505479	0	0	2,140334606	7,800026535	RPL32;RPS8;RPS6;RPS20;RPSA;RPS3A;RPL10A;RPL39;GOLGA7
Didanosine-500_uM_in_DMSO-Rat-Primary_rat_hepatocytes-0.67d-dn	9/289	0,026139639	0,104452457	0	0	2,140334606	7,800026535	RPS17;RPS19;ARL2BP;RPS8;FUNDC1;RPS6;RPS20;RPSA;SEPP1
Epalrestat-2500_mg/kg_in_CMC-Rat-Liver-1d-up	9/289	0,026139639	0,10439949	0	0	2,140334606	7,800026535	RPS17;RPS19;RPS8;RPS5;RPS6;RPSA;RPL10A;RPL39;TPT1
Fenofibrate-215_mg/kg_in_Corn_Oil-Rat-Liver-0.25d-dn	9/289	0,026139639	0,104346575	0	0	2,140334606	7,800026535	EEF1B2;RPS17;CLP1;RPS8;RPS20;RPSA;RPS3A;RPL39;TPT1
Ivermectin-7.5_mg/kg_in_CMC-Rat-Liver-5d-dn	9/289	0,026139639	0,104293715	0	0	2,140334606	7,800026535	EEF1B2;RPL32;ARL2BP;RPS8;FUNDC1;RPS6;RPS3A;RPL10A;TPT1
Lansoprazole-600_mg/kg_in_CMC-Rat-Liver-1d-up	9/289	0,026139639	0,104240908	0	0	2,140334606	7,800026535	RPS17;RPS8;RPS5;ARL6IP5;RPL35A;RPSA;RPS3A;RPL10A;RPL39
Nateglinide-625_mg/kg_in_CMC-Rat-Kidney-3d-dn	9/289	0,026139639	0,104188155	0	0	2,140334606	7,800026535	ARL2BP;RPS6;ARL6IP5;RPS20;RPSA;RAPGEF5;RPL10A;RPL39;PDRG1
Spironolactone-300_mg/kg_in_CMC-Rat-Heart-3d-up	9/289	0,026139639	0,104135454	0	0	2,140334606	7,800026535	EEF1B2;RPS17;RPL32;RPS19;RPS5;RPS20;RPS3A;RAPGEF5;RPL39

Minoxidil-83_mg/kg_in_CMC-Rat-Heart-1d-dn	8/243	0,026306537	0,104747363	0	0	2,262667402	8,231443319	EEF1B2;RPS17;RPL32;FUNDC1;ARL6IP5;RPL35A;RPS3A;TOMM20
Azasetron-580_mg/kg_in_CMC-Rat-Intestine-1d-dn	10/337	0,026324448	0,104765715	0	0	2,039422028	7,417902456	RPS17;RPL32;RPS19;RPS6;RPS20;RPSA;RPS3A;SEPP1;RPL39;TPT1
Lead(IV)_Acetate-200_mg/kg_in_Saline-Rat-Bone_marrow-1d-up	10/337	0,026324448	0,104712803	0	0	2,039422028	7,417902456	C1QA;RPS17;ARL2BP;RPS8;FUNDC1;RPS6;RPS20;RPL39;GOLGA7;TPT1
Quetiapine-150_uM_in_DMSO-Rat-Primary_rat_hepatocytes-1d-dn	10/337	0,026324448	0,104659945	0	0	2,039422028	7,417902456	CXCL11;RPS19;ARL2BP;CLP1;RPS8;ARL6IP5;SEPP1;RPL39;SLC16A3;TPT1
Clarithromycin-476_mg/kg_in_Water-Rat-Liver-5d-up	9/290	0,026647107	0,105889312	0	0	2,132954141	7,732118061	EEF1B2;RPS17;RPL32;RPS6;RPL35A;RPS20;RPL10A;RPL39;TPT1
Diflunisal-207_mg/kg_in_Corn_Oil-Rat-Kidney-5d-up	9/290	0,026647107	0,105835913	0	0	2,132954141	7,732118061	EEF1B2;RPL32;RPS19;RPS6;RPS20;RPSA;RAPGEF5;RPL10A;RPL39
Ethylene_Glycol-352_mg/kg_in_Water-Rat-Bone_marrow-1d-up	9/290	0,026647107	0,105782568	0	0	2,132954141	7,732118061	RPL32;RPS8;RPS5;RPS6;NKG7;RPL35A;RPS3A;RPL10A;TPT1
Fenofibrate-43_mg/kg_in_Corn_Oil-Rat-Kidney-0.25d-dn	9/290	0,026647107	0,105729278	0	0	2,132954141	7,732118061	EEF1B2;RPS17;RPL32;RPS19;RPS6;RPS20;RPSA;RPS3A;RPL10A
Fenofibrate-43_mg/kg_in_Corn_Oil-Rat-Kidney-1d-dn	9/290	0,026647107	0,10567604	0	0	2,132954141	7,732118061	EEF1B2;RPS17;RPL32;FEM1B;RPL35A;RPS3A;RPL39;SEPP1;TOMM20
Lamotrigine-26_mg/kg_in_CMC-Rat-Heart-5d-up	9/290	0,026647107	0,105622857	0	0	2,132954141	7,732118061	RPS17;RPL32;RPS8;RPS5;RPL35A;RPS20;RPSA;TOMM20;TPT1
Methyl_Salicylate-444_mg/kg_in_Corn_Oil-Rat-Kidney-3d-up	9/290	0,026647107	0,105569726	0	0	2,132954141	7,732118061	EEF1B2;RPS17;RPL32;RPS19;RPS8;RPS6;RPS20;RPS3A;TPT1
Myrtecaine-140_mg/kg_in_Corn_Oil-Rat-Heart-1d-dn	9/290	0,026647107	0,10551665	0	0	2,132954141	7,732118061	EEF1B2;RPS17;ARL2BP;RPS8;RPS5;RPL35A;RPS20;SEPP1;TOMM20
Saquinavir-1500_mg/kg_in_CMC-Rat-Kidney-5d-up	9/290	0,026647107	0,105463626	0	0	2,132954141	7,732118061	EEF1B2;RPS17;RPL32;RPS19;RPS8;RPL35A;RPS20;RPS3A;RPL39
6-Mercaptopurine-25_mg/kg_in_Corn_Oil-Rat-Kidney-1d-dn	10/338	0,026792627	0,105986304	0	0	2,033388235	7,36011008	EEF1B2;RPS19;ARL2BP;RPS8;RPS6;RPS20;RPSA;RPS3A;SEPP1;RPL39
Mexiletine-350_mg/kg_in_CMC-Rat-Heart-1d-up	10/338	0,026792627	0,105933098	0	0	2,033388235	7,36011008	EEF1B2;RPS17;RPL32;RPS6;RPS20;RPSA;RPS3A;RAPGEF5;RPL10A;PDRG1
Nitrobenzene-225_mg/kg_in_CMC-Rat-Kidney-1d-dn	10/338	0,026792627	0,105879945	0	0	2,033388235	7,36011008	EEF1B2;RPL32;RPS19;RPS8;RPS5;RPS6;RPSA;SEPP1;RPL39;TOMM20
Simvastatin-160_uM_in_DMSO-Rat-Primary_rat_hepatocytes-1d-dn	10/338	0,026792627	0,105826846	0	0	2,033388235	7,36011008	RPS17;RPL32;RPS19;ARL2BP;CLP1;RPS8;RPS5;RPS20;RPS3A;TPT1
Diisopropyl_Fluorophosphate-3.75_mg/kg_in_Water-Rat-Liver-3d-dn	8/244	0,026869333	0,106076626	0	0	2,253394175	8,150007713	C1QA;RPS17;RPL32;CLP1;RPS6;RPS20;RPL39;SEPP1
Ifosfamide-17_mg/kg_in_Saline-Rat-Liver-1d-dn	8/244	0,026869333	0,106023481	0	0	2,253394175	8,150007713	EEF1B2;RPL32;RPS19;RPS8;RPS6;RPS3A;RPL10A;SEPP1
Acemetacin-2_mg/kg_in_Corn_Oil-Rat-Liver-1d-up	9/291	0,027161505	0,107122693	0	0	2,125624402	7,664904981	C1QA;RPS17;RPL32;RPS19;RPS8;RPS6;RPS3A;SEPP1;TPT1
Alprazolam-115_mg/kg_in_Corn_Oil-Rat-Liver-1d-up	9/291	0,027161505	0,107069078	0	0	2,125624402	7,664904981	EEF1B2;RPS17;RPL32;RPS19;RPS6;RPS20;RPL39;SEPP1;TPT1
Balsalazide-1100_mg/kg_in_Water-Rat-Liver-1d-dn	9/291	0,027161505	0,107015516	0	0	2,125624402	7,664904981	EEF1B2;RPS17;RPL32;CLP1;RPS8;RPS5;RPS20;RPS3A;TPT1
Benzethonium_Chloride-30_mg/kg_in_Corn_Oil-Rat-Liver-1d-up	9/291	0,027161505	0,106962009	0	0	2,125624402	7,664904981	EEF1B2;C1QA;RPS17;RPL32;RPS19;ARL2BP;RPS8;RPS20;RPSA
Cytarabine-487_mg/kg_in_Saline-Rat-Liver-5d-dn	9/291	0,027161505	0,106908554	0	0	2,125624402	7,664904981	C1QA;RPS17;RPS19;CLP1;RPS8;RPS6;RPS20;RPSA;RPL10A
Itraconazole-1093_mg/kg_in_Corn_Oil-Rat-Liver-3d-up	9/291	0,027161505	0,106855153	0	0	2,125624402	7,664904981	EEF1B2;BMP3;RPS19;RPS20;RPSA;RPS3A;RPL10A;RPL39;SEPP1
Methapyrilene-300_uM_in_DMSO-Rat-Primary_rat_hepatocytes-0.67d-up	9/291	0,027161505	0,106801806	0	0	2,125624402	7,664904981	EEF1B2;RPL32;RPS19;RPS6;RPS20;RPSA;RPL39;GOLGA7;PDRG1
Piclamilast-17_mg/kg_in_CMC-Rat-Heart-1d-dn	9/291	0,027161505	0,106748511	0	0	2,125624402	7,664904981	RPS17;RPL32;RPS19;RPS6;RPSA;RPL10A;RPL39;GOLGA7;SEPP1
Rabeprazole-333_uM_in_DMSO-Rat-Primary_rat_hepatocytes-0.67d-dn	9/291	0,027161505	0,10669527	0	0	2,125624402	7,664904981	RPS17;CXCL11;RPL32;RPS8;ARL6IP5;RPS20;SEPP1;SLC16A3;TPT1
Raloxifene-650_mg/kg_in_Corn_Oil-Rat-Liver-0.25d-dn	9/291	0,027161505	0,106642082	0	0	2,125624402	7,664904981	RPS17;RPL32;CLP1;RPS8;RPL35A;RPS20;RPS3A;RPL39;SEPP1
Simvastatin-15_mg/kg_in_Corn_Oil-Rat-Liver-5d-up	9/291	0,027161505	0,106588947	0	0	2,125624402	7,664904981	EEF1B2;RPL32;RPS19;RPS8;RPS6;RPS20;RPL10A;RPL39;TPT1
Sulpiride-667_uM_in_DMSO-Rat-Primary_rat_hepatocytes-0.67d-dn	9/291	0,027161505	0,106535865	0	0	2,125624402	7,664904981	CXCL11;RPL32;RPS19;RPS8;RPS5;FUNDC1;ARL6IP5;RPL35A;RPS20
Vecuronium_Bromide-0.005_mg/kg_in_Saline-Rat-Heart-5d-dn	9/291	0,027161505	0,106482836	0	0	2,125624402	7,664904981	EEF1B2;RPS17;ARL2BP;RPS8;ARL6IP5;RPS3A;RAPGEF5;SEPP1;TPT1
Metoclopramide-185_mg/kg_in_CMC-Rat-Heart-1d-up	10/339	0,02726674	0,106842211	0	0	2,027390039	7,302836599	EEF1B2;RPS17;RPS8;FUNDC1;ARL6IP5;RPS20;RPSA;SEPP1;RPL39;TPT1
Vincristine-0.05_mg/kg_in_Saline-Rat-Heart-3d-up	10/339	0,02726674	0,106789082	0	0	2,027390039	7,302836599	EEF1B2;RPL32;RPS8;COL5A3;RPS6;RPL35A;RPS20;RPSA;RPL10A;TPT1
Amikacin-160_mg/kg_in_Saline-Rat-Kidney-28d-dn	8/245	0,027440452	0,107416005	0	0	2,244196648	8,069540914	EEF1B2;RPS17;RPL32;FUNDC1;RPS6;RPS20;RPS3A;RPL39
Lead_(II)_Acetate-200_mg/kg_in_Saline-Rat-Bone_marrow-3d-dn	8/245	0,027440452	0,107362644	0	0	2,244196648	8,069540914	EEF1B2;RPL32;RPS19;RPS6;NKG7;RPS3A;RPL10A;RPL39
Cimetidine-100_mg/kg_in_CMC-Rat-Heart-3d-dn	7/200	0,027442483	0,107317276	0	0	2,405498282	8,64936117	RPS19;ARL2BP;RPS8;FUNDC1;RPS6;ARL6IP5;RPSA
2378-Tetrachlorodibenzo-P-Dioxin-0.02_mg/kg_in_CMC-Rat-Liver-5d-up	9/292	0,027682877	0,108203642	0	0	2,118344867	7,598378516	EEF1B2;RPS17;RPL32;RPS8;RPS20;RPSA;RPS3A;RPL10A;SEPP1
Angiotensin_II_Human-0.72_mg/kg_in_Saline-Rat-Heart-1d-up	9/292	0,027682877	0,10814997	0	0	2,118344867	7,598378516	RPS19;RPS8;RPS6;ARL6IP5;RPS20;RPSA;RAPGEF5;RPL10A;RPL39
Cisplatin-2_mg/kg_in_Saline-Rat-Bone_marrow-5d-up	9/292	0,027682877	0,10809635	0	0	2,118344867	7,598378516	C1QA;RPS19;ARL2BP;RPS8;RPS6;NKG7;RPL35A;RPS20;TPT1
Fenofibrate-43_mg/kg_in_Corn_Oil-Rat-Liver-0.25d-dn	9/292	0,027682877	0,108042784	0	0	2,118344867	7,598378516	EEF1B2;RPS17;RPS19;CLP1;RPS8;RPL35A;RPS20;RPS3A;SEPP1
Fluvastatin-5_mg/kg_in_Corn_Oil-Rat-Liver-5d-up	9/292	0,027682877	0,107989271	0	0	2,118344867	7,598378516	EEF1B2;RPS17;RPS19;RPS6;RPS20;RPS3A;SEPP1;RPL39;TPT1
Hexachloroethane-750_mg/kg_in_CMC-Rat-Kidney-1d-up	9/292	0,027682877	0,107935811	0	0	2,118344867	7,598378516	RPS17;ARL2BP;CLP1;FEM1B;RPSA;RPS3A;RPL10A;SEPP1;GOLGA7
Nafenopin-338_mg/kg_in_Corn_Oil-Rat-Liver-5d-up	9/292	0,027682877	0,107882404	0	0	2,118344867	7,598378516	EEF1B2;RPS17;RPL32;RPS19;RPS8;RPSA;RPS3A;RPL10A;TPT1
Nitrendipine-10_mg/kg_in_Corn_Oil-Rat-Heart-0.25d-dn	9/292	0,027682877	0,10782905	0	0	2,118344867	7,598378516	RPL32;RPS19;ARL2BP;RPS8;RPS5;FUNDC1;RPS6;RPSA;TPT1
Pemoline-70_mg/kg_in_CMC-Rat-Liver-3d-dn	9/292	0,027682877	0,107775748	0	0	2,118344867	7,598378516	EEF1B2;RPS17;RPL32;RPS19;RPS8;RPL35A;RPS20;SEPP1;TPT1
Perhexiline-12_uM_in_DMSO-Rat-Primary_rat_hepatocytes-0.67d-dn	9/292	0,027682877	0,107722499	0	0	2,118344867	7,598378516	RPS17;RPL32;ARL2BP;RPS8;RPS5;ARL6IP5;RPSA;RPS3A;SEPP1
Tetracycline-520_uM_in_DMSO-Rat-Primary_rat_hepatocytes-0.67d-up	9/292	0,027682877	0,107669303	0	0	2,118344867	7,598378516	EEF1B2;RPS17;ARL2BP;RPS6;RPS20;RPS3A;RPL10A;RPL39;PDRG1
Phenacetin-619_mg/kg_in_Corn_Oil-Rat-Kidney-5d-dn	10/340	0,027746822	0,107864744	0	0	2,021427128	7,24607634	RPS17;RPL32;RPS8;RPS6;RPS20;RPSA;RPS3A;SEPP1;RPL39;TPT1
Prinomastat-500_uM_in_DMSO-Rat-Primary_rat_hepatocytes-1d-dn	10/340	0,027746822	0,10781153	0	0	2,021427128	7,24607634	CXCL11;RPS19;RPS6;ARL6IP5;RPSA;RPL10A;SEPP1;RPL39;SLC16A3;TPT1
Azathioprine-160_mg/kg_in_Corn_Oil-Rat-Liver-0.25d-up	9/293	0,028211265	0,109562091	0	0	2,111115021	7,532530021	EEF1B2;RPS17;RPL32;RPS19;RPS6;RPSA;RPS3A;RPL10A;RPL39
Benoxaprofen-89_mg/kg_in_CMC-Rat-Liver-3d-up	9/293	0,028211265	0,109508092	0	0	2,111115021	7,532530021	EEF1B2;RPS8;RPS5;RPS6;RPS20;RPS3A;RPL10A;RPL39;TPT1
Cerivastatin-7_mg/kg_in_Corn_Oil-Rat-Liver-5d-dn	9/293	0,028211265	0,109454148	0	0	2,111115021	7,532530021	EEF1B2;RPS17;RPL32;RPS8;RPL35A;RPS3A;SEPP1;RPL39;TPT1
Gemfibrozil-100_mg/kg_in_Corn_Oil-Rat-Liver-1d-up	9/293	0,028211265	0,109400256	0	0	2,111115021	7,532530021	EEF1B2;RPS17;RPL32;RPS19;RPS6;RPS20;RPSA;RPL39;TPT1
Valsartan-30_mg/kg_in_Corn_Oil-Rat-Heart-0.25d-dn	9/293	0,028211265	0,109346417	0	0	2,111115021	7,532530021	RPS17;RPS19;ARL2BP;RPS8;RPS6;ARL6IP5;RPL13A;RPSA;RPS3A
Vincristine-0.05_mg/kg_in_Saline-Rat-Bone_marrow-3d-up	9/293	0,028211265	0,109292631	0	0	2,111115021	7,532530021	RPL32;RPS8;RPS5;NKG7;RPL35A;RPS20;RPL10A;SEPP1;RPL39
Cobalt(II)_Acetylacetonate-100_mg/kg_in_Corn_Oil-Rat-Kidney-1d-up	10/341	0,028232909	0,109322711	0	0	2,015499189	7,1898237	EEF1B2;RPS17;RPL32;RPS6;ARL6IP5;RPL35A;RPS20;RPSA;RPS3A;RPL39
Fluphenazine-45_uM_in_DMSO-Rat-Primary_rat_hepatocytes-1d-up	8/247	0,028607869	0,110720184	0	0	2,226025015	7,911456448	EEF1B2;RPS17;RPL32;RPS5;RPS6;RPS3A;RPL10A;PDRG1
3-Methylcholanthrene-23_uM_in_DMSO-Rat-Primary_rat_hepatocytes-1d-dn	10/342	0,028725038	0,111119055	0	0	2,009605916	7,134073158	EEF1B2;RPL32;RPS8;RPS6;RPSA;RPS3A;RPL10A;SEPP1;RPL39;SLC16A3
Crotamiton-750_mg/kg_in_Corn_Oil-Rat-Liver-3d-dn	10/342	0,028725038	0,111064505	0	0	2,009605916	7,134073158	EEF1B2;RPS17;RPL32;RPS19;RPS8;RPS6;RPS20;RPS3A;GOLGA7;TPT1
Fluoxetine-52_mg/kg_in_CMC-Rat-Heart-1d-up	10/342	0,028725038	0,111010008	0	0	2,009605916	7,134073158	EEF1B2;CXCL11;FUNDC1;RPS6;ARL6IP5;RPS20;RPSA;SEPP1;RPL39;TPT1
Flutamide-197_mg/kg_in_Corn_Oil-Rat-Kidney-1d-up	10/342	0,028725038	0,110955565	0	0	2,009605916	7,134073158	C1QA;RPS17;RPL32;RPS19;RPS5;RPS6;RPS3A;SEPP1;RPL39;TPT1
Imatinib-150_mg/kg_in_Water-Rat-Liver-3d-dn	10/342	0,028725038	0,110901175	0	0	2,009605916	7,134073158	C1QA;RPS17;RPL32;RPS19;RPS6;RPS20;RPSA;RPL10A;SEPP1;RPL39
Naproxen-10_mg/kg_in_Corn_Oil-Rat-Liver-3d-up	10/342	0,028725038	0,110846838	0	0	2,009605916	7,134073158	EEF1B2;RPL32;RPS19;RPS8;RPS6;RPL35A;RPS20;RPSA;RPL39;TPT1
6-Mercaptopurine-5_uM_in_DMSO-Rat-Primary_rat_hepatocytes-0.67d-dn	9/294	0,028746711	0,110876148	0	0	2,103934357	7,467350985	EEF1B2;RPS17;RPL32;RPS19;ARL2BP;CLP1;RPS6;RPS20;SEPP1
Acetaminophen-486_mg/kg_in_Corn_Oil-Rat-Kidney-5d-up	9/294	0,028746711	0,110821877	0	0	2,103934357	7,467350985	RPS17;RPL32;ARL2BP;RPS5;ARL6IP5;RPL35A;RPS20;RAPGEF5;RPL39
Betamethasone-79_mg/kg_in_Corn_Oil-Rat-Bone_marrow-5d-dn	9/294	0,028746711	0,110767659	0	0	2,103934357	7,467350985	EEF1B2;RPS17;RPS8;FUNDC1;RPS20;RPSA;RPS3A;RPL39;TPT1
Catechol-40_mg/kg_in_Saline-Rat-Kidney-3d-dn	9/294	0,028746711	0,110713494	0	0	2,103934357	7,467350985	EEF1B2;RPS17;RPL32;RPS19;RPS8;R3HCC1;RPS6;RPS3A;TPT1
Econazole-43_mg/kg_in_Corn_Oil-Rat-Liver-5d-up	9/294	0,028746711	0,110659382	0	0	2,103934357	7,467350985	EEF1B2;RPS17;RPL32;RPS8;RPS6;RPS3A;RPL10A;RPL39;TPT1
Fenofibrate-215_mg/kg_in_Corn_Oil-Rat-Heart-5d-dn	9/294	0,028746711	0,110605322	0	0	2,103934357	7,467350985	RPS17;RPL32;RPS8;COL5A3;RPL35A;RPS3A;SEPP1;RPL39;GOLGA7
Hydroquinone-800_mg/kg_in_CMC-Rat-Kidney-3d-up	9/294	0,028746711	0,110551316	0	0	2,103934357	7,467350985	EEF1B2;RPS17;RPS19;RPS8;RPS5;RPS3A;SEPP1;GOLGA7;TPT1
Ibuprofen-54_mg/kg_in_Corn_Oil-Rat-Kidney-1d-up	9/294	0,028746711	0,110497362	0	0	2,103934357	7,467350985	RPS19;ARL2BP;CLP1;RPS8;RPS5;RPSA;RPS3A;RAPGEF5;TPT1
Irinotecan-5_mg/kg_in_Saline-Rat-Kidney-5d-dn	9/294	0,028746711	0,110443461	0	0	2,103934357	7,467350985	EEF1B2;RPS17;RPL32;ARL2BP;RPS8;RPS6;RPS20;RPS3A;TPT1
Thalidomide-113_mg/kg_in_CMC-Rat-Liver-3d-dn	9/294	0,028746711	0,110389612	0	0	2,103934357	7,467350985	EEF1B2;RPS19;CLP1;RPS6;ARL6IP5;RPS20;RPS3A;SEPP1;TPT1
Zileuton-450_mg/kg_in_Corn_Oil-Rat-Liver-3d-up	9/294	0,028746711	0,110335816	0	0	2,103934357	7,467350985	RPL32;RPS19;CLP1;RPS8;RPS5;RPS6;RPSA;RPS3A;RPL10A
Ibufenac-500_mg/kg_in_Corn_Oil-Rat-Liver-5d-dn	10/343	0,029223242	0,112110206	0	0	2,003747007	7,07881926	EEF1B2;C1QA;RPS17;RPL32;RPS19;RPS6;RPL35A;RPSA;RPL10A;RPL39
Lead_(II)_Acetate-600_mg/kg_in_Saline-Rat-Kidney-1d-up	10/343	0,029223242	0,112055624	0	0	2,003747007	7,07881926	RPS17;CXCL11;RPS19;RPS6;RPS20;RPSA;RPL10A;SEPP1;RPL39;GOLGA7
Benzethonium_Chloride-30_mg/kg_in_Corn_Oil-Rat-Heart-5d-up	9/295	0,029289258	0,11225411	0	0	2,096802376	7,402833031	C1QA;RPL32;RPS19;CLP1;RPS8;FUNDC1;RPS6;RPS3A;SEPP1
Bithionol-333_mg/kg_in_Corn_Oil-Rat-Kidney-3d-dn	9/295	0,029289258	0,112199511	0	0	2,096802376	7,402833031	EEF1B2;RPS17;RPL32;RPS8;RPL35A;RPSA;RPL10A;SEPP1;TPT1
Buspirone-196_mg/kg_in_CMC-Rat-Heart-5d-up	9/295	0,029289258	0,112144966	0	0	2,096802376	7,402833031	RPS17;CXCL11;RPL32;RPS19;RPS8;RPS5;RPS6;RPL35A;RPSA
Chlorambucil-4.5_mg/kg_in_Corn_Oil-Rat-Spleen-3d-up	9/295	0,029289258	0,112090474	0	0	2,096802376	7,402833031	EEF1B2;C1QA;RPL32;RPS5;RPS6;RPL35A;RPS3A;RAPGEF5;SEPP1
Citalopram-40_mg/kg_in_Corn_Oil-Rat-Liver-0.25d-dn	9/295	0,029289258	0,112036035	0	0	2,096802376	7,402833031	EEF1B2;RPS17;RPL32;RPS8;RPS5;RPL35A;RPSA;RPS3A;TPT1
Daunorubicin-3.25_mg/kg_in_Saline-Rat-Bone_marrow-0.25d-dn	9/295	0,029289258	0,111981648	0	0	2,096802376	7,402833031	EEF1B2;RPS17;RPL32;RPS8;RPS20;RPS3A;SEPP1;RPL39;TPT1
Gemfibrozil-100_mg/kg_in_Corn_Oil-Rat-Kidney-0.25d-dn	9/295	0,029289258	0,111927315	0	0	2,096802376	7,402833031	EEF1B2;RPS17;RPL32;RPS8;RPS6;ARL6IP5;RPL35A;RPSA;TOMM20
Ketoconazole-227_mg/kg_in_Corn_Oil-Rat-Liver-3d-up	9/295	0,029289258	0,111873034	0	0	2,096802376	7,402833031	RPL32;RPS19;RPS8;RPS6;RPS20;RPSA;SEPP1;RPL39;GOLGA7
Labetalol-2500_mg/kg_in_Water-Rat-Heart-1d-up	9/295	0,029289258	0,111818805	0	0	2,096802376	7,402833031	RPS17;RPL32;RPS19;RPS6;ARL6IP5;RPS20;RPSA;RAPGEF5;RPL39
Miconazole-200_mg/kg_in_Corn_Oil-Rat-Liver-5d-up	9/295	0,029289258	0,11176463	0	0	2,096802376	7,402833031	RPS17;RPL32;RPS5;RPS6;RPL35A;RPS20;RPSA;RPL10A;RPL39
Mifepristone-300_mg/kg_in_Corn_Oil-Rat-Liver-0.25d-dn	9/295	0,029289258	0,111710506	0	0	2,096802376	7,402833031	RPS17;RPS19;CLP1;RPS8;FEM1B;RPL35A;RPSA;SEPP1;RPL39
Papaverine-69_mg/kg_in_CMC-Rat-Heart-5d-up	9/295	0,029289258	0,111656435	0	0	2,096802376	7,402833031	RPS17;CXCL11;RPL32;ARL2BP;RPS8;FUNDC1;RPL35A;RPS20;HK2
Atorvastatin-300_mg/kg_in_Corn_Oil-Rat-Skeletal_muscle-5d-dn	10/344	0,029727557	0,11327249	0	0	1,997922161	7,02405663	EEF1B2;RPS19;COL5A3;ARL6IP5;RPSA;RPS3A;RPL10A;RPL39;SLC16A3;TPT1

Carmustine-16_mg/kg_in_Corn_Oil-Rat-Liver-1d-up	10/344	0,029727557	0,113217716	0	0	1,997922161	7,02405663	CXCL11;RPL32;RPS6;RPS20;RPS3A;RPL10A;SEPP1;RPL39;GOLGA7;TPT1
Albendazole-62_mg/kg_in_Corn_Oil-Rat-Liver-1d-up	9/296	0,029838947	0,113587023	0	0	2,089718585	7,33896791	C1QA;RPS19;RPS8;FEM1B;RPS6;RPS20;RPSA;RPL10A;TPT1
Amikacin-20_mg/kg_in_Saline-Rat-Bone_marrow-1d-up	9/296	0,029838947	0,11353215	0	0	2,089718585	7,33896791	C1QA;RPL32;RPS19;FEM1B;RPS6;RPS3A;RPL10A;SEPP1;GOLGA7
Carbamazepine-490_mg/kg_in_CMC-Rat-Liver-5d-up	9/296	0,029838947	0,11347733	0	0	2,089718585	7,33896791	RPS17;RPL32;RPS19;RPS8;RPS6;RPS20;RPS3A;RPL39;TPT1
Choline_Chloride-2550_mg/kg_in_Water-Rat-Liver-3d-up	9/296	0,029838947	0,113422563	0	0	2,089718585	7,33896791	RPS17;RPL32;RPS8;RPS5;RPS6;RPSA;RPS3A;RAPGEF5;TPT1
Methyl_Salicylate-444_mg/kg_in_Corn_Oil-Rat-Liver-1d-dn	9/296	0,029838947	0,113367849	0	0	2,089718585	7,33896791	EEF1B2;C1QA;RPS17;RPL32;RPS3A;RPL10A;SEPP1;RPL39;TPT1
N-Nitrosodiethylamine-34_mg/kg_in_Saline-Rat-Spleen-5d-up	9/296	0,029838947	0,113313187	0	0	2,089718585	7,33896791	EEF1B2;C1QA;RPS17;RPL32;RPS19;RPS6;RPS20;RPS3A;RPL39
Oxcarbazepine-1600_mg/kg_in_CMC-Rat-Kidney-1d-dn	9/296	0,029838947	0,113258578	0	0	2,089718585	7,33896791	RPS19;ARL2BP;RPS6;ARL6IP5;RPS20;RPSA;RPL10A;SEPP1;PDRG1
Oxfendazole-1500_mg/kg_in_CMC-Rat-Kidney-1d-dn	9/296	0,029838947	0,113204022	0	0	2,089718585	7,33896791	RPS17;RPL32;RPS19;RPS6;ARL6IP5;RPL35A;RPS20;GOLGA7;TPT1
Amiodarone-37.04_uM_in_DMSO-Rat-Primary_rat_hepatocytes-1d-dn	10/345	0,030238017	0,114662794	0	0	1,992131082	6,969779961	RPS17;CXCL11;RPL32;RPS19;CLP1;ARL6IP5;RPL10A;SEPP1;RPL39;TPT1
Leflunomide-30_mg/kg_in_Corn_Oil-Rat-Kidney-0.25d-up	10/345	0,030238017	0,114607615	0	0	1,992131082	6,969779961	EEF1B2;RPS17;RPL32;RPS8;FUNDC1;RPL13A;RPL35A;RPSA;RPL39;TPT1
D-Tubocurarine_Chloride-0.0033_mg/kg_in_Saline-Rat-Heart-5d-dn	9/297	0,030395821	0,115150304	0	0	2,082682495	7,275747498	EEF1B2;RPS17;RPL32;RPS8;FUNDC1;ARL6IP5;RPS3A;RAPGEF5;TPT1
Lovastatin-450_mg/kg_in_Corn_Oil-Rat-Kidney-3d-up	9/297	0,030395821	0,115094944	0	0	2,082682495	7,275747498	RPS17;RPL32;RPS8;RPS5;RPS6;RPL13A;RPL35A;RPS20;RPS3A
Methyl_Salicylate-444_mg/kg_in_Corn_Oil-Rat-Kidney-1d-up	9/297	0,030395821	0,115039636	0	0	2,082682495	7,275747498	EEF1B2;RPS17;RPL32;RPS19;RPS8;RPS6;RPS20;RPS3A;TPT1
Nimetazepam-122_mg/kg_in_CMC-Rat-Liver-3d-dn	9/297	0,030395821	0,114984382	0	0	2,082682495	7,275747498	EEF1B2;RPS17;RPL32;RPS8;ARL6IP5;RPS20;SEPP1;GOLGA7;TPT1
Promethazine-113_mg/kg_in_Saline-Rat-Liver-3d-up	9/297	0,030395821	0,11492918	0	0	2,082682495	7,275747498	RPS17;RPL32;RPS19;RPS5;RPS6;RPL35A;RPS20;RPS3A;RPL39
SCH-351591-4.6_mg/kg_in_CMC-Rat-Liver-1d-dn	9/297	0,030395821	0,114874032	0	0	2,082682495	7,275747498	EEF1B2;RPL32;RPS19;RPS6;RPS20;RPS3A;RPL10A;RPL39;TPT1
Timolol-10_mg/kg_in_Water-Rat-Heart-1d-up	9/297	0,030395821	0,114818937	0	0	2,082682495	7,275747498	RPS17;RPL32;RPS19;COL5A3;RPS5;ARL6IP5;RPS20;RPS3A;RPL39
Troglitazone-1200_mg/kg_in_Corn_Oil-Rat-Liver-3d-dn	9/297	0,030395821	0,114763894	0	0	2,082682495	7,275747498	C1QA;RPS17;RPL32;RPS19;RPS6;RPS20;RPSA;RPL10A;RPL39
Podophyllotoxin-4_mg/kg_in_Corn_Oil-Rat-Bone_marrow-3d-up	10/346	0,030754657	0,116063097	0	0	1,986373478	6,915984015	EEF1B2;C1QA;RPL32;RPS5;RPS6;ARL6IP5;NKG7;RPL10A;SEPP1;RPL39
Bezafibrate-617_mg/kg_in_Corn_Oil-Rat-Liver-1d-up	9/298	0,030959918	0,11678176	0	0	2,075693628	7,213163798	EEF1B2;RPS17;RPL32;RPS19;RPS8;RPS5;RPS6;RPS20;RPS3A
Cimetidine-100_mg/kg_in_CMC-Rat-Heart-5d-up	9/298	0,030959918	0,116725856	0	0	2,075693628	7,213163798	RPS17;RPL32;ARL2BP;RPS6;RPS3A;RPL10A;RPL39;TOMM20;TPT1
Cobalt_(II)_Chloride-10_mg/kg_in_Saline-Rat-Kidney-28d-up	9/298	0,030959918	0,116670007	0	0	2,075693628	7,213163798	EEF1B2;RPS19;RPS8;RPS20;RPSA;RPS3A;SEPP1;RPL39;TPT1
Ketoconazole-25_mg/kg_in_Corn_Oil-Rat-Liver-3d-dn	9/298	0,030959918	0,11661421	0	0	2,075693628	7,213163798	C1QA;RPL32;RPS19;RPS8;RPS5;RPS6;RPS3A;RPL10A;SEPP1
Methotrexate-0.3_mg/kg_in_Corn_Oil-Rat-Bone_marrow-3d-dn	9/298	0,030959918	0,116558468	0	0	2,075693628	7,213163798	RPS17;RPL32;RPS19;FEM1B;RPS6;RPS3A;RPL10A;RPL39;TPT1
Mitomycin_C-0.5_mg/kg_in_Saline-Rat-Spleen-0.25d-dn	9/298	0,030959918	0,116502778	0	0	2,075693628	7,213163798	CXCL11;RPS19;CLP1;RPS8;FUNDC1;RPL35A;RPS20;RPSA;TPT1
N-Nitrosodiethylamine-1.67_mg/kg_in_Saline-Rat-Bone_marrow-3d-dn	9/298	0,030959918	0,116447141	0	0	2,075693628	7,213163798	EEF1B2;ARL2BP;CLP1;FUNDC1;RPS6;NKG7;RPS20;RPL39;GOLGA7
Pentobarbital-70_mg/kg_in_Water-Rat-Liver-1d-up	9/298	0,030959918	0,116391558	0	0	2,075693628	7,213163798	EEF1B2;RPS17;RPL32;RPS19;RPS8;RPS20;RPSA;RPS3A;RPL39
Salicylic_Acid-223_mg/kg_in_Corn_Oil-Rat-Liver-3d-dn	9/298	0,030959918	0,116336028	0	0	2,075693628	7,213163798	EEF1B2;RPS17;RPL32;RPS8;RPSA;RPS3A;RPL10A;RPL39;TPT1
Sodium_Arsenite-10_mg/kg_in_Saline-Rat-Liver-3d-dn	9/298	0,030959918	0,11628055	0	0	2,075693628	7,213163798	C1QA;RPS17;CLP1;RPS8;RPSA;RPS3A;SEPP1;RPL39;TPT1
Valsartan-1000_mg/kg_in_Corn_Oil-Rat-Heart-3d-dn	9/298	0,030959918	0,116225126	0	0	2,075693628	7,213163798	EEF1B2;RPS17;RPL32;RPS8;ARL6IP5;RPSA;RPS3A;TOMM20;TPT1
Vinblastine-45_uM_in_DMSO-Rat-Primary_rat_hepatocytes-0.67d-up	9/298	0,030959918	0,116169754	0	0	2,075693628	7,213163798	EEF1B2;RPS19;ARL2BP;RPS6;RPS20;RPS3A;RPL39;GOLGA7;PDRG1
Zirconium(IV)_Chloride-633_mg/kg_in_Corn_Oil-Rat-Liver-1d-up	9/298	0,030959918	0,116114435	0	0	2,075693628	7,213163798	EEF1B2;RPS17;RPL32;RPS19;RPS8;RPS5;RPSA;RPL10A;TPT1
Dideoxycytidine-1200_mg/kg_in_CMC-Rat-Liver-5d-dn	10/347	0,031277511	0,117249729	0	0	1,980649059	6,862663627	C1QA;RPS17;RPL32;RPS19;RPS8;RPS6;ARL6IP5;RPS20;RPSA;RPL10A
Azauridine-1500_mg/kg_in_CMC-Rat-Bone_marrow-5d-up	9/299	0,03153128	0,118144796	0	0	2,068751508	7,151208935	C1QA;RPS19;CLP1;NKG7;RPS20;RPSA;RPL10A;SEPP1;RPL39
Bisphenol_A-100_mg/kg_in_Corn_Oil-Rat-Bone_marrow-1d-dn	9/299	0,03153128	0,118088617	0	0	2,068751508	7,151208935	EEF1B2;RPS17;RPL32;RPS8;ARL6IP5;RPL35A;RPSA;RPS3A;TPT1
Caffeine-93_mg/kg_in_Water-Rat-Heart-5d-up	9/299	0,03153128	0,118032491	0	0	2,068751508	7,151208935	EEF1B2;RPS17;RPL32;ARL2BP;CLP1;RPS8;RPS20;RPS3A;TPT1
Carvedilol-2000_mg/kg_in_Corn_Oil-Rat-Heart-5d-dn	9/299	0,03153128	0,117976418	0	0	2,068751508	7,151208935	RPS17;RPL32;RPS19;ARL2BP;COL5A3;RPL35A;RPSA;RPS3A;RPL39
Clobetasol_Propionate-17_mg/kg_in_Corn_Oil-Rat-Bone_marrow-1d-dn	9/299	0,03153128	0,117920399	0	0	2,068751508	7,151208935	RPL32;RPS19;RPS8;FEM1B;RPS6;RPS20;RPSA;RPL10A;RPL39
Diethylstilbestrol-280_mg/kg_in_Corn_Oil-Rat-Kidney-5d-up	9/299	0,03153128	0,117864433	0	0	2,068751508	7,151208935	RPS17;RPS19;RPS8;RPS5;RPS20;RPS3A;RPL10A;RPL39;TPT1
Econazole-334_mg/kg_in_Corn_Oil-Rat-Liver-1d-dn	9/299	0,03153128	0,11780852	0	0	2,068751508	7,151208935	EEF1B2;C1QA;RPS17;RPL32;RPS6;RPSA;RPS3A;SEPP1;RPL39
Epirubicin-2.7_mg/kg_in_Saline-Rat-Kidney-3d-dn	9/299	0,03153128	0,11775266	0	0	2,068751508	7,151208935	EEF1B2;RPS17;RPS19;ARL2BP;CLP1;RPS20;RPSA;SEPP1;RPL39
Finasteride-800_mg/kg_in_Corn_Oil-Rat-Kidney-5d-dn	9/299	0,03153128	0,117696853	0	0	2,068751508	7,151208935	RPS17;RPL32;RPL35A;RPS3A;RAPGEF5;RPL10A;RPL39;GOLGA7;TPT1
Finasteride-800_mg/kg_in_Corn_Oil-Rat-Liver-3d-dn	9/299	0,03153128	0,117641099	0	0	2,068751508	7,151208935	EEF1B2;C1QA;RPS17;RPS6;RPS3A;RPL10A;RPL39;GOLGA7;TPT1
Haloperidol-21_mg/kg_in_Corn_Oil-Rat-Heart-3d-up	9/299	0,03153128	0,117585398	0	0	2,068751508	7,151208935	RPL32;ARL2BP;RPS8;ARL6IP5;RPS20;RPS3A;RPL10A;SEPP1;RPL39
Leflunomide-30_mg/kg_in_Corn_Oil-Rat-Liver-3d-dn	9/299	0,03153128	0,11752975	0	0	2,068751508	7,151208935	C1QA;RPS17;RPL32;RPS19;RPS6;RPS20;RPL10A;SEPP1;RPL39
Lovastatin-1500_mg/kg_in_Corn_Oil-Rat-Liver-1d-up	9/299	0,03153128	0,117474154	0	0	2,068751508	7,151208935	C1QA;RPS19;RPS5;RPS20;RPSA;RPS3A;RPL10A;RPL39;TPT1
Mesna-755_mg/kg_in_Saline-Rat-Kidney-3d-up	9/299	0,03153128	0,11741861	0	0	2,068751508	7,151208935	C1QA;CXCL11;RPL32;ARL2BP;RPS8;RPS6;RPL35A;RPSA;SEPP1
Tranilast-300_mg/kg_in_CMC-Rat-Spleen-5d-up	9/299	0,03153128	0,117363119	0	0	2,068751508	7,151208935	EEF1B2;RPS19;RPS5;RPS6;RPS20;RPSA;RPL10A;SEPP1;RPL39
Zomepirac-11_mg/kg_in_Saline-Rat-Kidney-3d-up	9/299	0,03153128	0,117307681	0	0	2,068751508	7,151208935	RPS17;RPS19;ARL2BP;RPS5;RPS20;RPSA;RPS3A;SEPP1;RPL39
D-Galactosamine-470_uM_in_DMSO-Rat-Primary_rat_hepatocytes-0.67d-dn	8/252	0,031675666	0,117789211	0	0	2,181857852	7,532223895	EEF1B2;RPS17;RPS8;RPS20;RPS3A;RPL39;TPT1;PDRG1
Haloperidol-77_uM_in_DMSO-Rat-Primary_rat_hepatocytes-1d-up	8/252	0,031675666	0,117733623	0	0	2,181857852	7,532223895	EEF1B2;RPS17;RPL32;RPS20;RPSA;RPS3A;RPL10A;PDRG1
Hydrochlorothiazide-1500_mg/kg_in_CMC-Rat-Kidney-5d-dn	8/252	0,031675666	0,117678089	0	0	2,181857852	7,532223895	EEF1B2;RPS17;RPS19;ARL2BP;CLP1;RPS8;RPS6;RPS3A
Simvastatin-15_mg/kg_in_Corn_Oil-Rat-Kidney-3d-dn	10/348	0,031806613	0,118108855	0	0	1,974957538	6,809813697	EEF1B2;RPS17;RPL32;RPS19;ARL2BP;RPS6;RPS20;SEPP1;RPL39;TPT1
Cadmium_Chloride-2_mg/kg_in_Saline-Rat-Kidney-5d-up	9/300	0,032109946	0,119179046	0	0	2,06185567	7,089875151	CLP1;RPS8;RPS6;RPS20;RPSA;RPS3A;RPL10A;RPL39;TPT1
Fluconazole-394_mg/kg_in_Corn_Oil-Rat-Liver-3d-up	9/300	0,032109946	0,119122909	0	0	2,06185567	7,089875151	RPS19;RPS8;RPS5;RPS6;RPS20;RPSA;RPS3A;RPL10A;RPL39
N-Nitrosodiethylamine-34_mg/kg_in_Saline-Rat-Kidney-3d-up	9/300	0,032109946	0,119066825	0	0	2,06185567	7,089875151	RPS17;RPL32;RPS5;RPS6;RPL35A;RPS20;RPS3A;SEPP1;RPL39
Naloxone-76_mg/kg_in_Saline-Rat-Liver-3d-up	9/300	0,032109946	0,119010793	0	0	2,06185567	7,089875151	EEF1B2;C1QA;RPS17;RPL32;RPL35A;RPS20;RPSA;RPS3A;SEPP1
Penciclovir-1200_mg/kg_in_Corn_Oil-Rat-Bone_marrow-5d-dn	9/300	0,032109946	0,118954815	0	0	2,06185567	7,089875151	RPS17;RPS19;RPS8;RPS6;RPSA;RPS3A;RPL10A;RPL39;TPT1
Sertraline-23_uM_in_DMSO-Rat-Primary_rat_hepatocytes-0.67d-up	9/300	0,032109946	0,118898888	0	0	2,06185567	7,089875151	ARL2BP;RPS8;RPS5;RPS6;RPS20;RPS3A;RPL10A;GOLGA7;PDRG1
Spironolactone-300_mg/kg_in_CMC-Rat-Heart-1d-dn	9/300	0,032109946	0,118843015	0	0	2,06185567	7,089875151	EEF1B2;RPS17;RPL32;ARL2BP;RPS8;COL5A3;ARL6IP5;RPSA;TPT1
Sulconazole-1200_mg/kg_in_Corn_Oil-Rat-Liver-5d-dn	9/300	0,032109946	0,118787194	0	0	2,06185567	7,089875151	RPL32;RPS19;RPS8;FEM1B;RPS6;RPS3A;RPL10A;RPL39;TPT1
Tocainide-67_mg/kg_in_Corn_Oil-Rat-Liver-1d-up	9/300	0,032109946	0,118731425	0	0	2,06185567	7,089875151	RPS19;RPS8;RPS6;RPL35A;RPS20;RPSA;RPL39;GOLGA7;TPT1
Tranilast-300_mg/kg_in_CMC-Rat-Liver-1d-dn	9/300	0,032109946	0,118675709	0	0	2,06185567	7,089875151	RPS17;RPS19;RPS6;ARL6IP5;RPS20;RPSA;RPS3A;SEPP1;RPL39
Fluoxetine-39.6_uM_in_DMSO-Rat-Primary_rat_hepatocytes-0.67d-dn	6/163	0,032256465	0,119161313	0	0	2,529884258	8,687715606	RPS17;CXADR;CLP1;ARL6IP5;RPSA;RPL10A
Azithromycin-50_mg/kg_in_Water-Rat-Liver-3d-dn	8/253	0,032315208	0,119322353	0	0	2,173233908	7,459011068	RPS17;RPL32;RPS19;RPS8;RPS20;RPSA;RPL39;TPT1
Desloratadine-57_uM_in_DMSO-Rat-Primary_rat_hepatocytes-1d-dn	8/253	0,032315208	0,119266438	0	0	2,173233908	7,459011068	EEF1B2;CXCL11;RPS19;RPS6;ARL6IP5;RPS20;RPL39;SEPP1
Lomustine-4.2_mg/kg_in_Corn_Oil-Rat-Spleen-3d-up	8/253	0,032315208	0,119210575	0	0	2,173233908	7,459011068	RPL32;CLP1;RPS8;RPS5;RPS6;RPL35A;RPS20;RAPGEF5
Cobalt(II)_Acetylacetonate-100_mg/kg_in_Corn_Oil-Rat-Kidney-3d-up	10/349	0,032341994	0,119253534	0	0	1,969298634	6,757429193	EEF1B2;RPS17;RPL32;RPS6;ARL6IP5;RPL35A;RPS20;RPSA;RPS3A;RPL39
Rapamycin-20_uM_in_DMSO-Rat-Primary_rat_hepatocytes-1d-dn	10/349	0,032341994	0,119197729	0	0	1,969298634	6,757429193	C1QA;CXCL11;RPS19;RPS6;ARL6IP5;RPS3A;SEPP1;RPL39;SLC16A3;TPT1
Atorvastatin-250_uM_in_DMSO-Rat-Primary_rat_hepatocytes-0.67d-dn	9/301	0,032695956	0,120445907	0	0	2,055005651	7,02915481	EEF1B2;CXCL11;RPS8;RPS6;ARL6IP5;RPS20;RPSA;SEPP1;SLC16A3
Bithionol-59_mg/kg_in_Corn_Oil-Rat-Bone_marrow-0.25d-dn	9/301	0,032695956	0,120389597	0	0	2,055005651	7,02915481	RPL32;RPS19;RPS8;RPS6;ARL6IP5;NKG7;RPSA;RPL10A;TPT1
Bupropion-895_mg/kg_in_Water-Rat-Liver-1d-up	9/301	0,032695956	0,12033334	0	0	2,055005651	7,02915481	EEF1B2;RPS17;RPS19;ARL2BP;RPS6;RPS20;RPS3A;RPL10A;RPL39
Busulfan-9_mg/kg_in_Corn_Oil-Rat-Liver-3d-up	9/301	0,032695956	0,120277136	0	0	2,055005651	7,02915481	EEF1B2;RPS17;RPL32;RPS19;RPS8;RPS20;RPS3A;SEPP1;TPT1
Caffeine-93_mg/kg_in_Water-Rat-Heart-5d-dn	9/301	0,032695956	0,120220984	0	0	2,055005651	7,02915481	RPS19;COL5A3;FUNDC1;RPS6;RPSA;RPL10A;SEPP1;RPL39;PDRG1
Carboplatin-6_mg/kg_in_Saline-Rat-Spleen-0.25d-dn	9/301	0,032695956	0,120164885	0	0	2,055005651	7,02915481	EEF1B2;CXCL11;RPL32;RPS19;RPS8;FEM1B;FUNDC1;RPS20;RPS3A
Dichlorvos-17_mg/kg_in_Water-Rat-Liver-1d-up	9/301	0,032695956	0,120108838	0	0	2,055005651	7,02915481	EEF1B2;RPS17;RPL32;RPS19;RPS6;RPS20;RPSA;RPS3A;RPL10A
Etoposide-125_uM_in_DMSO-Rat-Primary_rat_hepatocytes-0.67d-dn	9/301	0,032695956	0,120052843	0	0	2,055005651	7,02915481	RPS17;RPL32;RPS8;RPS5;RPL35A;RPS20;RPS3A;TPT1;PDRG1
Fenofibrate-100_mg/kg_in_Corn_Oil-Rat-Liver-1d-up	9/301	0,032695956	0,1199969	0	0	2,055005651	7,02915481	EEF1B2;RPS17;RPL32;RPS19;RPS8;RPS6;RPS20;RPS3A;RPL10A
Fenofibrate-43_mg/kg_in_Corn_Oil-Rat-Liver-1d-dn	9/301	0,032695956	0,11994101	0	0	2,055005651	7,02915481	EEF1B2;C1QA;RPS17;RPL32;RPS8;RPL35A;RPS3A;RPL39;TPT1
Lisinopril-2000_mg/kg_in_CMC-Rat-Kidney-3d-dn	9/301	0,032695956	0,119885171	0	0	2,055005651	7,02915481	EEF1B2;RPL32;RPS19;ARL2BP;CLP1;RPS8;RPS6;RPL10A;SEPP1
Mosapride-2000_mg/kg_in_CMC-Rat-Intestine-5d-dn	9/301	0,032695956	0,119829385	0	0	2,055005651	7,02915481	EEF1B2;RPS17;RPS19;RPS6;RPL13A;RPL35A;RPS3A;SEPP1;RPL39
Nateglinide-625_mg/kg_in_CMC-Rat-Liver-5d-up	9/301	0,032695956	0,11977365	0	0	2,055005651	7,02915481	RPS17;RPL32;RPS19;RPS8;RPS6;RPL35A;RPSA;RPS3A;SEPP1
Nystatin-134_mg/kg_in_Corn_Oil-Rat-Kidney-3d-up	9/301	0,032695956	0,119717968	0	0	2,055005651	7,02915481	C1QA;ARL2BP;RPSA;RAPGEF5;RPL10A;SEPP1;RPL39;GOLGA7;TPT1
Olanzapine-23_mg/kg_in_CMC-Rat-Heart-5d-dn	9/301	0,032695956	0,119662337	0	0	2,055005651	7,02915481	RPS17;RPS19;ARL2BP;COL5A3;RPS6;RPL35A;RPS20;RPS3A;SEPP1
Pirinixic_Acid-364_mg/kg_in_CMC-Rat-Liver-3d-up	9/301	0,032695956	0,119606757	0	0	2,055005651	7,02915481	RPL32;RPS19;RPS8;RPS6;RPSA;RPS3A;RPL10A;RPL39;TPT1
Rapamycin-20_uM_in_DMSO-Rat-Primary_rat_hepatocytes-0.67d-up	9/301	0,032695956	0,119551229	0	0	2,055005651	7,02915481	EEF1B2;RPS17;RPL32;RPS19;ARL2BP;RPS20;RPL10A;RPL39;GOLGA7
Sisomicin-34_mg/kg_in_Saline-Rat-Kidney-5d-up	9/301	0,032695956	0,119495753	0	0	2,055005651	7,02915481	RPS17;CXCL11;RPS19;RPS6;ARL6IP5;RPL35A;RPS20;SEPP1;GOLGA7
Sodium_Arsenite-10_mg/kg_in_Saline-Rat-Bone_marrow-0.25d-up	9/301	0,032695956	0,119440329	0	0	2,055005651	7,02915481	EEF1B2;RPS19;RPS8;RPS5;FUNDC1;RPS6;RPL35A;RPS20;SLC16A3
Ticlopidine-223_mg/kg_in_CMC-Rat-Liver-3d-up	9/301	0,032695956	0,119384955	0	0	2,055005651	7,02915481	RPL32;RPS19;RPS8;RPS5;RPS6;RPSA;RPS3A;RPL10A;RPL39

Tretinoin-7_mg/kg_in_Corn_Oil-Rat-Liver-5d-dn	9/301	0,032695956	0,119329633	0	0	2,055005651	7,02915481	EEF1B2;RPS17;RPL32;RPS19;RPS8;RPS6;RPS3A;RPL10A;RPL39
Troxipide-1200_mg/kg_in_CMC-Rat-Heart-3d-up	9/301	0,032695956	0,119274362	0	0	2,055005651	7,02915481	RPS17;RPL32;FUNDC1;RPSA;RAPGEF5;RPL10A;SEPP1;TPT1;PDRG1
Atorvastatin-300_mg/kg_in_Corn_Oil-Rat-Liver-1d-dn	10/350	0,03288369	0,119903676	0	0	1,963672067	6,705505148	EEF1B2;RPL32;RPS19;CLP1;RPS8;RPS6;RPS20;RPS3A;SEPP1;TPT1
Carbon_Tetrachloride-400_mg/kg_in_Corn_Oil-Rat-Kidney-0.25d-up	10/350	0,03288369	0,119848191	0	0	1,963672067	6,705505148	EEF1B2;RPS17;RPL32;RPS19;FUNDC1;RPS6;RPS20;RPL10A;SEPP1;RPL39
Bromisovalum-250_mg/kg_in_Corn_Oil-Rat-Heart-3d-dn	8/254	0,032963522	0,120083579	0	0	2,164677869	7,386646601	EEF1B2;RPS17;RPS8;FUNDC1;RPS6;RPS20;RPL39;SEPP1
Manganese_(II)_Chloride-700_mg/kg_in_Water-Rat-Liver-3d-dn	8/254	0,032963522	0,120028062	0	0	2,164677869	7,386646601	RPS17;RPL32;RPS19;RPS8;RPL35A;RPS3A;SEPP1;TPT1
1-Naphthyl_Isothiocyanate-30_mg/kg_in_Corn_Oil-Rat-Bone_marrow-0.25d-dn	9/302	0,033289348	0,121158458	0	0	2,048200997	6,969040388	RPS17;RPL32;RPS8;RPS6;NKG7;RPS20;RPSA;RPS3A;SEPP1
Alfacalcidol-0.043_mg/kg_in_CMC-Rat-Liver-3d-dn	9/302	0,033289348	0,121102496	0	0	2,048200997	6,969040388	RPS17;RPL32;RPS19;CLP1;RPS8;RPS6;RPS20;RPL10A;SEPP1
Choline_Chloride-2550_mg/kg_in_Water-Rat-Liver-1d-up	9/302	0,033289348	0,121046585	0	0	2,048200997	6,969040388	RPL32;RPS8;RPS5;RPS6;RPL35A;RPS20;RPSA;RPS3A;TPT1
Geraniol-1500_mg/kg_in_CMC-Rat-Liver-5d-up	9/302	0,033289348	0,120990726	0	0	2,048200997	6,969040388	C1QA;RPS17;RPL32;RPS19;RPS6;RPL35A;RPS3A;RPL10A;RPL39
Gold_Sodium_Thiomalate-8_mg/kg_in_Saline-Rat-Kidney-28d-up	9/302	0,033289348	0,120934919	0	0	2,048200997	6,969040388	EEF1B2;RPS17;RPL32;RPS19;RPS8;RPS20;RPS3A;RPL39;TPT1
Norepinephrine-1.75_mg/kg_in_Saline-Rat-Heart-5d-up	9/302	0,033289348	0,120879162	0	0	2,048200997	6,969040388	C1QA;RPS19;CLP1;RPS8;FUNDC1;RPS20;RPSA;RPL10A;RPL39
Oxfendazole-1500_mg/kg_in_CMC-Rat-Kidney-5d-up	9/302	0,033289348	0,120823458	0	0	2,048200997	6,969040388	EEF1B2;RPS17;CXCL11;RPL32;ARL6IP5;RPS20;RPSA;RPS3A;RAPGEF5
Rosiglitazone-1800_mg/kg_in_Corn_Oil-Rat-Liver-5d-dn	9/302	0,033289348	0,120767804	0	0	2,048200997	6,969040388	EEF1B2;C1QA;CLP1;RPS6;RPS3A;RPL10A;SEPP1;RPL39;TPT1
Testosterone-375_mg/kg_in_CMC-Rat-Liver-5d-up	9/302	0,033289348	0,120712202	0	0	2,048200997	6,969040388	EEF1B2;RPS17;RPS8;RPS20;RPSA;RPS3A;RPL10A;SEPP1;TPT1
Valproic_Acid-1500_mg/kg_in_Water-Rat-Heart-1d-dn	9/302	0,033289348	0,120656651	0	0	2,048200997	6,969040388	EEF1B2;RPS17;COL5A3;RPS6;RPS20;RPSA;RPL10A;SEPP1;RPL39
(R)-Bicalutamide-1400_mg/kg_in_CMC-Rat-Kidney-5d-up	10/351	0,033431731	0,12111698	0	0	1,958077559	6,654036662	RPL32;RPS19;RPS8;RPS5;RPS6;RPL13A;RPL35A;RPSA;SEPP1;TPT1
Cyclophosphamide-1320_uM_in_DMSO-Rat-Primary_rat_hepatocytes-1d-up	8/255	0,033620654	0,121745411	0	0	2,156188936	7,315118288	EEF1B2;CXCL11;ARL6IP5;RPL10A;RPL39;GOLGA7;SEPP1;SLC16A3
Aspirin-375_mg/kg_in_Corn_Oil-Rat-Liver-5d-up	9/303	0,03389016	0,122664937	0	0	2,041441258	6,909524476	EEF1B2;RPS17;RPL32;RPS19;RPS6;RPS20;RPS3A;RPL10A;RPL39
Buflomedil-615_mg/kg_in_CMC-Rat-Heart-5d-dn	9/303	0,03389016	0,122608591	0	0	2,041441258	6,909524476	RPS19;ARL2BP;FUNDC1;RPL35A;RPS20;RPSA;RPS3A;SEPP1;RPL39
Busulfan-3_mg/kg_in_Corn_Oil-Rat-Bone_marrow-3d-dn	9/303	0,03389016	0,122552297	0	0	2,041441258	6,909524476	RPS19;FEM1B;RPS6;ARL6IP5;NKG7;RPSA;RPL10A;SEPP1;GOLGA7
Carmustine-16_mg/kg_in_Corn_Oil-Rat-Spleen-1d-up	9/303	0,03389016	0,122496054	0	0	2,041441258	6,909524476	C1QA;RPL32;RPS8;FUNDC1;RPSA;RPL10A;SEPP1;RPL39;TPT1
Daunorubicin-4_mg/kg_in_Saline-Rat-Heart-14d-up	9/303	0,03389016	0,122439863	0	0	2,041441258	6,909524476	EEF1B2;RPS17;RPS8;RPS6;RPS20;RPS3A;RPL10A;RPL39;GOLGA7
Etidronate-1005_mg/kg_in_Water-Rat-Heart-3d-up	9/303	0,03389016	0,122383724	0	0	2,041441258	6,909524476	EEF1B2;RPS19;RPS8;FUNDC1;RPS6;RPSA;RAPGEF5;RPL10A;TOMM20
Indinavir-1000_mg/kg_in_CMC-Rat-Kidney-1d-up	9/303	0,03389016	0,122327636	0	0	2,041441258	6,909524476	EEF1B2;RPS17;RPL32;RPS19;ARL2BP;RPS8;ARL6IP5;RPS20;RPS3A
Metoclopramide-185_mg/kg_in_CMC-Rat-Heart-5d-up	9/303	0,03389016	0,1222716	0	0	2,041441258	6,909524476	RPS17;RPL32;RPS8;ARL6IP5;RPSA;RAPGEF5;SEPP1;RPL39;TPT1
Mitotane-900_mg/kg_in_Water-Rat-Bone_marrow-1d-up	9/303	0,03389016	0,122215615	0	0	2,041441258	6,909524476	EEF1B2;RPL32;RPS19;RPS6;NKG7;RPL35A;RPSA;RPL10A;SEPP1
Pemoline-833_uM_in_DMSO-Rat-Primary_rat_hepatocytes-0.67d-dn	9/303	0,03389016	0,122159681	0	0	2,041441258	6,909524476	RPS17;RPL32;RPS8;RPS5;RPL35A;RPS20;RPS3A;TPT1;PDRG1
Phenacemide-400_mg/kg_in_CMC-Rat-Heart-5d-up	9/303	0,03389016	0,122103798	0	0	2,041441258	6,909524476	EEF1B2;RPS17;RPL32;CLP1;RPS8;RPS3A;RAPGEF5;RPL39;TPT1
Prednisone-68_mg/kg_in_CMC-Rat-Heart-5d-up	9/303	0,03389016	0,122047966	0	0	2,041441258	6,909524476	EEF1B2;RPS17;RPL32;ARL6IP5;RPL35A;RPS20;RPSA;RAPGEF5;TPT1
Prednisone-68_mg/kg_in_CMC-Rat-Kidney-1d-dn	9/303	0,03389016	0,121992186	0	0	2,041441258	6,909524476	EEF1B2;RPS17;RPL32;RPS19;RPS8;RPSA;RPS3A;RPL10A;SEPP1
Prochlorperazine-85_mg/kg_in_CMC-Rat-Heart-1d-dn	9/303	0,03389016	0,121936456	0	0	2,041441258	6,909524476	RPS17;RPL32;ARL2BP;RPS8;RPS20;RPSA;RPS3A;RPL39;TPT1
Quetiapine-500_mg/kg_in_CMC-Rat-Liver-5d-dn	9/303	0,03389016	0,121880777	0	0	2,041441258	6,909524476	EEF1B2;C1QA;RPS17;RPL32;RPS19;RPS8;RPL35A;RPS3A;RPL10A
Ticrynafen-570_mg/kg_in_CMC-Rat-Liver-5d-up	9/303	0,03389016	0,121825149	0	0	2,041441258	6,909524476	EEF1B2;CXCL11;RPL32;RPS6;RPSA;RPS3A;RPL10A;RPL39;TPT1
Gold_Sodium_Thiomalate-8_mg/kg_in_Saline-Rat-Kidney-14d-up	10/352	0,033986151	0,122114473	0	0	1,952514839	6,603018897	CXCL11;RPL32;RPS19;RPS8;RPS6;RPS3A;RPL10A;RPL39;GOLGA7;TPT1
Rofecoxib-3_mg/kg_in_Corn_Oil-Rat-Liver-1d-up	10/352	0,033986151	0,12205879	0	0	1,952514839	6,603018897	C1QA;RPS19;RPS6;RPS20;RPSA;RPS3A;RPL10A;SEPP1;RPL39;GOLGA7
Gentamicin-2900_uM_in_DMSO-Rat-Primary_rat_hepatocytes-1d-up	8/256	0,03428665	0,123081884	0	0	2,147766323	7,244414137	EEF1B2;RPS19;CLP1;RPS20;RPS3A;RPL39;SLC16A3;PDRG1
Lomustine-8.75_mg/kg_in_Corn_Oil-Rat-Spleen-3d-up	8/256	0,03428665	0,12302581	0	0	2,147766323	7,244414137	RPL32;CLP1;RPS8;RPS5;RPS6;RPL35A;RPS20;RAPGEF5
Colchicine-1000_uM_in_DMSO-Rat-Primary_rat_hepatocytes-0.67d-up	3/49	0,034363312	0,123244737	0	0	4,207868714	14,18373996	EEF1B2;RPS6;RPS20
4-Methylpyrazole-155_mg/kg_in_Saline-Rat-Kidney-1d-dn	9/304	0,034498431	0,123673028	0	0	2,03472599	6,850599775	EEF1B2;RPS17;RPL32;RPS19;ARL2BP;RPS6;RPS20;RPS3A;RPL39
Acetazolamide-250_mg/kg_in_Corn_Oil-Rat-Liver-1d-dn	9/304	0,034498431	0,123616762	0	0	2,03472599	6,850599775	EEF1B2;RPS17;RPL32;RPL35A;RPS3A;RPL10A;SEPP1;RPL39;TPT1
Celecoxib-400_mg/kg_in_Corn_Oil-Rat-Liver-5d-dn	9/304	0,034498431	0,123560547	0	0	2,03472599	6,850599775	EEF1B2;RPS17;RPS19;FEM1B;RPS6;RPS20;RPL10A;RPL39;TPT1
Chlorobenzene-500_mg/kg_in_CMC-Rat-Heart-1d-dn	9/304	0,034498431	0,123504383	0	0	2,03472599	6,850599775	RPS17;RPL32;RPS19;COL5A3;RPS6;RPSA;RPL10A;SEPP1;RPL39
Ferulic_Acid-1500_mg/kg_in_CMC-Rat-Liver-5d-up	9/304	0,034498431	0,12344827	0	0	2,03472599	6,850599775	RPS17;RPL32;FEM1B;RPS6;NKG7;RPS20;RPSA;RPL10A;SEPP1
Gemfibrozil-700_mg/kg_in_Corn_Oil-Rat-Liver-1d-up	9/304	0,034498431	0,123392208	0	0	2,03472599	6,850599775	EEF1B2;RPL32;RPS19;RPS8;RPS6;RPS20;RPS3A;GOLGA7;TPT1
Methylparaben-1500_mg/kg_in_CMC-Rat-Liver-5d-up	9/304	0,034498431	0,123336197	0	0	2,03472599	6,850599775	RPS17;RPL32;RPS8;RPS5;ARL6IP5;RPL35A;RPSA;RPS3A;RPL39
Metronidazole-1500_mg/kg_in_Water-Rat-Liver-1d-up	9/304	0,034498431	0,123280237	0	0	2,03472599	6,850599775	EEF1B2;RPS17;RPL32;RPS8;RPS5;RPL35A;RPSA;RPS3A;RPL39
N-Nitrosodiethylamine-1.67_mg/kg_in_Saline-Rat-Bone_marrow-0.25d-up	9/304	0,034498431	0,123224327	0	0	2,03472599	6,850599775	C1QA;CLP1;RPS8;RPS5;FUNDC1;RPS6;NKG7;RPS20;RPSA
Nisoldipine-15_mg/kg_in_Water-Rat-Liver-3d-up	9/304	0,034498431	0,123168469	0	0	2,03472599	6,850599775	C1QA;RPS17;RPS19;RPS8;RPS5;RPS6;RPS20;RPSA;RPS3A
Quetiapine-150_uM_in_DMSO-Rat-Primary_rat_hepatocytes-0.67d-up	9/304	0,034498431	0,123112661	0	0	2,03472599	6,850599775	EEF1B2;RPS17;RPS5;RPS6;RPS3A;RPL10A;RPL39;TPT1;PDRG1
Salicylamide-1300_mg/kg_in_CMC-Rat-Liver-3d-up	9/304	0,034498431	0,123056903	0	0	2,03472599	6,850599775	C1QA;RPS17;RPS19;RPS6;RPSA;RPS3A;RAPGEF5;RPL10A;RPL39
Sulconazole-1200_mg/kg_in_Corn_Oil-Rat-Liver-3d-dn	9/304	0,034498431	0,123001196	0	0	2,03472599	6,850599775	RPS17;RPS19;RPS8;RPS6;RPSA;RPS3A;RPL10A;RPL39;TPT1
Tocainide-67_mg/kg_in_Corn_Oil-Rat-Liver-3d-up	9/304	0,034498431	0,122945539	0	0	2,03472599	6,850599775	RPS19;CLP1;RPS8;RPS6;RPS20;RPSA;RPS3A;SEPP1;TPT1
Trichloroethane-300_mg/kg_in_Water-Rat-Liver-1d-dn	9/304	0,034498431	0,122889933	0	0	2,03472599	6,850599775	EEF1B2;RPS17;RPS19;CLP1;RPS8;RPS20;RPS3A;SEPP1;TPT1
Ebastine-2_mg/kg_in_Water-Rat-Heart-3d-up	10/353	0,034546981	0,123007244	0	0	1,946983636	6,55244708	RPS19;ARL2BP;RPS8;FUNDC1;RPS6;ARL6IP5;RPS20;RPSA;RPL10A;GOLGA7
Acetazolamide-250_mg/kg_in_Corn_Oil-Rat-Liver-5d-dn	9/305	0,035114197	0,124970364	0	0	2,028054757	6,792259099	EEF1B2;C1QA;RPS17;RPL32;RPS6;RPS3A;RPL10A;SEPP1;RPL39
Altretamine-40_mg/kg_in_Corn_Oil-Rat-Liver-1d-up	9/305	0,035114197	0,124913918	0	0	2,028054757	6,792259099	EEF1B2;RPS17;RPL32;RPS19;RPS8;RPS20;RPL10A;RPL39;TPT1
Bithionol-59_mg/kg_in_Corn_Oil-Rat-Liver-1d-up	9/305	0,035114197	0,124857524	0	0	2,028054757	6,792259099	C1QA;RPS17;RPL32;RPS19;RPS6;RPL35A;RPS20;RPSA;RPS3A
Bromobenzene-439_mg/kg_in_CMC-Rat-Kidney-1d-up	9/305	0,035114197	0,12480118	0	0	2,028054757	6,792259099	RPS17;RPL32;RPS8;RPS6;RPSA;RPS3A;SEPP1;RPL39;TPT1
Chlorambucil-4.5_mg/kg_in_Corn_Oil-Rat-Spleen-3d-dn	9/305	0,035114197	0,124744887	0	0	2,028054757	6,792259099	RPS17;RPS19;RPS8;FEM1B;FUNDC1;RPL13A;RPSA;RPL10A;TPT1
Cyclosporin_A-70_mg/kg_in_Corn_Oil-Rat-Bone_marrow-0.25d-up	9/305	0,035114197	0,124688645	0	0	2,028054757	6,792259099	EEF1B2;C1QA;RPS19;NKG7;RPS20;RPS3A;RPL10A;RPL39;TPT1
Dexamethasone-300_mg/kg_in_Corn_Oil-Rat-Kidney-1d-up	9/305	0,035114197	0,124632454	0	0	2,028054757	6,792259099	RPL32;RPS8;RPS6;ARL6IP5;RPS20;RPSA;SEPP1;RPL39;TPT1
Diethylstilbestrol-280_mg/kg_in_Corn_Oil-Rat-Spleen-1d-dn	9/305	0,035114197	0,124576313	0	0	2,028054757	6,792259099	RPS17;RPS19;FEM1B;RPS5;RPS6;RPL35A;RPL10A;RPL39;TPT1
Fluoxetine-52_mg/kg_in_CMC-Rat-Kidney-5d-up	9/305	0,035114197	0,124520223	0	0	2,028054757	6,792259099	RPS17;RPS19;FEM1B;RPS6;RPSA;RPS3A;RPL10A;SEPP1;TPT1
Genistein-375_mg/kg_in_Corn_Oil-Rat-Liver-1d-up	9/305	0,035114197	0,124464183	0	0	2,028054757	6,792259099	EEF1B2;RPL32;RPS19;RPS8;RPS5;RPS6;RPS20;RPSA;RPL10A
Ifosfamide-143_mg/kg_in_Saline-Rat-Spleen-1d-dn	9/305	0,035114197	0,124408194	0	0	2,028054757	6,792259099	RPS17;RPL32;RPS8;RPS5;FUNDC1;RPL13A;RPL35A;RPS3A;TPT1
Indomethacin-9.6_mg/kg_in_Water-Rat-Liver-5d-up	9/305	0,035114197	0,124352255	0	0	2,028054757	6,792259099	EEF1B2;C1QA;RPS6;RPS20;RPS3A;SEPP1;RPL39;GOLGA7;TPT1
Leflunomide-30_mg/kg_in_Corn_Oil-Rat-Bone_marrow-0.25d-dn	9/305	0,035114197	0,124296367	0	0	2,028054757	6,792259099	RPS17;RPL32;RPS8;FEM1B;RPS6;ARL6IP5;RPSA;RPS3A;TPT1
Manganese_(II)_Chloride-700_mg/kg_in_Water-Rat-Liver-1d-dn	9/305	0,035114197	0,124240528	0	0	2,028054757	6,792259099	EEF1B2;RPS17;RPL32;RPS19;RPS6;RPL35A;RPS20;RPS3A;SEPP1
Ondansetron-84_mg/kg_in_Water-Rat-Intestine-0.25d-dn	9/305	0,035114197	0,12418474	0	0	2,028054757	6,792259099	RPS17;RPS19;RPS5;RPL35A;RPS20;RPSA;RPL10A;SEPP1;TPT1
Pantoprazole-650_uM_in_DMSO-Rat-Primary_rat_hepatocytes-1d-dn	9/305	0,035114197	0,124129002	0	0	2,028054757	6,792259099	RPS19;ARL6IP5;RPS20;RPSA;RPL10A;SEPP1;RPL39;SLC16A3;TPT1
Procarbazine-54_mg/kg_in_Water-Rat-Liver-1d-dn	9/305	0,035114197	0,124073313	0	0	2,028054757	6,792259099	EEF1B2;RPL32;RPS19;RPS8;RPS6;RPS20;RPS3A;RPL10A;SEPP1
Trichloroacetic_Acid-474_mg/kg_in_Corn_Oil-Rat-Liver-1d-up	9/305	0,035114197	0,124017675	0	0	2,028054757	6,792259099	EEF1B2;C1QA;RPS17;RPL32;ARL6IP5;RPL35A;RPSA;RPS3A;RPL39
Celecoxib-100_uM_in_DMSO-Rat-Primary_rat_hepatocytes-1d-dn	10/354	0,035114253	0,123962285	0	0	1,941483682	6,502316496	EEF1B2;CXCL11;RPS19;ARL2BP;CLP1;RPS8;FUNDC1;RPS20;SEPP1;TPT1
Cytochalasin_B-167_uM_in_DMSO-Rat-Primary_rat_hepatocytes-0.67d-dn	10/354	0,035114253	0,123906746	0	0	1,941483682	6,502316496	EEF1B2;ARL2BP;RPS8;RPS6;ARL6IP5;RPSA;RPL10A;SEPP1;RPL39;SLC16A3
Fluvastatin-5_mg/kg_in_Corn_Oil-Rat-Kidney-3d-dn	10/354	0,035114253	0,123851258	0	0	1,941483682	6,502316496	EEF1B2;RPS17;RPL32;RPS19;RPS6;RPSA;RPL10A;SEPP1;RPL39;TPT1
Gentamicin-267_mg/kg_in_Corn_Oil-Rat-Kidney-3d-up	10/354	0,035114253	0,123795818	0	0	1,941483682	6,502316496	C1QA;RPS17;RPL32;RPS19;RPS8;RPS6;RPS20;RPSA;RPS3A;PDRG1
Fenoprofen-52_mg/kg_in_Corn_Oil-Rat-Kidney-0.25d-dn	8/258	0,035645411	0,125612194	0	0	2,131116972	7,105431423	EEF1B2;RPS17;RPS19;ARL2BP;RPS8;ARL6IP5;RPSA;RPL39
Idarubicin-1.5_uM_in_DMSO-Rat-Primary_rat_hepatocytes-1d-up	8/258	0,035645411	0,125556017	0	0	2,131116972	7,105431423	EEF1B2;RPS17;CXCL11;RPL32;RPS3A;GOLGA7;SEPP1;PDRG1
Modafinil-325_mg/kg_in_Saline-Rat-Liver-5d-up	10/355	0,035687998	0,125649832	0	0	1,936014714	6,452622495	C1QA;RPL32;RPS19;RPS8;RPS6;RPL35A;RPS20;RPS3A;RPL10A;GOLGA7
2-Acetylaminofluorene-30_mg/kg_in_CMC-Rat-Liver-5d-up	9/306	0,035737495	0,125767878	0	0	2,021427128	6,734495366	RPS17;RPL32;RPS19;RPS6;RPS20;RPSA;SEPP1;RPL39;TPT1
Acetaminophen-100_mg/kg_in_Corn_Oil-Rat-Kidney-3d-up	9/306	0,035737495	0,125711707	0	0	2,021427128	6,734495366	EEF1B2;RPS17;RPL32;RPS8;RPS5;ARL6IP5;RPL13A;RPL35A;RPS20
Carvedilol-2000_mg/kg_in_Corn_Oil-Rat-Liver-3d-up	9/306	0,035737495	0,125655586	0	0	2,021427128	6,734495366	RPS19;ARL2BP;RPS6;RPS20;RPSA;RPS3A;RPL10A;RPL39;TPT1
Daunorubicin-4_mg/kg_in_Saline-Rat-Heart-28d-up	9/306	0,035737495	0,125599514	0	0	2,021427128	6,734495366	EEF1B2;RPS17;CXCL11;RPS19;RPS6;RPS20;RPL10A;SEPP1;RPL39
Deferiprone-500_mg/kg_in_Water-Rat-Liver-1d-up	9/306	0,035737495	0,125543493	0	0	2,021427128	6,734495366	RPS17;RPL32;RPS8;RPS5;RPS6;RPL35A;RPSA;RPS3A;TPT1
Lead(IV)_Acetate-200_mg/kg_in_Saline-Rat-Kidney-3d-up	9/306	0,035737495	0,125487522	0	0	2,021427128	6,734495366	RPS17;CXCL11;RPS19;RPS5;RPL13A;RPL35A;RPS20;SEPP1;GOLGA7
Omeprazole-415_mg/kg_in_Corn_Oil-Rat-Liver-5d-up	9/306	0,035737495	0,125431601	0	0	2,021427128	6,734495366	EEF1B2;RPS17;RPL32;RPS19;RPS8;RPS20;RPSA;RPS3A;RPL10A
Primidone-750_mg/kg_in_CMC-Rat-Liver-1d-dn	9/306	0,035737495	0,125375729	0	0	2,021427128	6,734495366	EEF1B2;RPS17;RPS19;RPS8;RPS6;ARL6IP5;RPL35A;RPS20;SEPP1
Mercuric_Chloride-2.5_mg/kg_in_Water-Rat-Kidney-1d-dn	7/212	0,03595492	0,126082346	0	0	2,269338002	7,546659354	RPS17;RPL32;RPS19;RPS6;ARL6IP5;RPSA;RPL39
Etoposide-188_mg/kg_in_Corn_Oil-Rat-Spleen-3d-up	8/259	0,036338262	0,127369895	0	0	2,122888721	7,037129924	EEF1B2;CXCL11;RPL32;RPS8;RPS5;RPS6;RPL35A;RPS20

Ifosfamide-143_mg/kg_in_Saline-Rat-Liver-1d-dn	8/259	0,036338262	0,127313236	0	0	2,122888721	7,037129924	RPS17;RPL32;RPS19;RPS8;RPS6;RPS20;RPS3A;SEPP1
Neomycin-56_mg/kg_in_Corn_Oil-Rat-Liver-1d-dn	8/259	0,036338262	0,127256627	0	0	2,122888721	7,037129924	RPS17;RPL32;RPS8;RPS6;RPSA;RPS3A;RPL10A;TPT1
Tretinoin-7_mg/kg_in_Corn_Oil-Rat-Liver-1d-up	8/259	0,036338262	0,127200069	0	0	2,122888721	7,037129924	EEF1B2;C1QA;RPL32;RPS5;RPL35A;RPS20;RPSA;SEPP1
4-Octylphenol-700_mg/kg_in_Corn_Oil-Rat-Kidney-5d-dn	9/307	0,036368361	0,127248874	0	0	2,014842674	6,677301599	C1QA;RPS17;RPL32;RPS8;FEM1B;RPS6;RPL35A;RAPGEF5;SEPP1
Cetraxate-1503_mg/kg_in_CMC-Rat-Liver-1d-up	9/307	0,036368361	0,127192369	0	0	2,014842674	6,677301599	EEF1B2;RPS17;RPL32;RPS8;RPL35A;RPS20;RPSA;RPS3A;TPT1
Colchicine-0.6_mg/kg_in_Saline-Rat-Kidney-1d-up	9/307	0,036368361	0,127135914	0	0	2,014842674	6,677301599	C1QA;RPS17;RPS19;RPS8;RPS6;RPS20;RPS3A;RPL10A;SEPP1
Dichlorvos-17_mg/kg_in_Water-Rat-Heart-3d-dn	9/307	0,036368361	0,12707951	0	0	2,014842674	6,677301599	RPL32;RPS19;RPS6;ARL6IP5;RPSA;RPL10A;SEPP1;RPL39;PDRG1
Ergocalciferol-15_mg/kg_in_CMC-Rat-Liver-1d-dn	9/307	0,036368361	0,127023155	0	0	2,014842674	6,677301599	RPS17;RPL32;RPS19;RPS6;ARL6IP5;RPS20;RPSA;SEPP1;RPL39
Foscarnet-350_mg/kg_in_Saline-Rat-Bone_marrow-5d-dn	9/307	0,036368361	0,126966851	0	0	2,014842674	6,677301599	RPS17;RPL32;RPS5;RPL35A;RPS20;RPL10A;SEPP1;RPL39;DUSP14
Loxoprofen-18_mg/kg_in_Corn_Oil-Rat-Kidney-1d-up	9/307	0,036368361	0,126910596	0	0	2,014842674	6,677301599	RPL32;RPS19;FEM1B;RPS6;ARL6IP5;RPSA;RAPGEF5;SEPP1;TPT1
Primidone-750_mg/kg_in_CMC-Rat-Liver-3d-dn	9/307	0,036368361	0,126854391	0	0	2,014842674	6,677301599	EEF1B2;C1QA;RPS19;CLP1;RPS8;RPS5;RPS20;SEPP1;TPT1
Tretinoin-156_uM_in_DMSO-Rat-Primary_rat_hepatocytes-0.67d-up	9/307	0,036368361	0,126798236	0	0	2,014842674	6,677301599	EEF1B2;RPS17;ARL2BP;RPS8;RPS5;RPS20;RPS3A;RPL10A;PDRG1
Papaverine-69_mg/kg_in_CMC-Rat-Heart-3d-dn	10/357	0,03685503	0,128438148	0	0	1,925168693	6,354525934	RPS19;ARL2BP;RPS8;COL5A3;ARL6IP5;RPS20;RPSA;RPS3A;RPL10A;RPL39
Amoxicillin-1100_mg/kg_in_Water-Rat-Liver-5d-dn	9/308	0,037006832	0,128910131	0	0	2,008300977	6,620670928	EEF1B2;RPS17;RPL32;RPL35A;RPS20;RPSA;RPS3A;SEPP1;RPL39
Bisphenol_A-610_mg/kg_in_Corn_Oil-Rat-Kidney-1d-dn	9/308	0,037006832	0,128853142	0	0	2,008300977	6,620670928	EEF1B2;RPS17;RPL32;RPS19;RPS6;RPL35A;RPS3A;RPL10A;RPL39
Closantel-22_mg/kg_in_CMC-Rat-Liver-3d-up	9/308	0,037006832	0,128796202	0	0	2,008300977	6,620670928	C1QA;RPS17;RPL32;RPL35A;RPS20;RPSA;RPL10A;RPL39;TPT1
Hexachloroethane-750_mg/kg_in_CMC-Rat-Kidney-1d-dn	9/308	0,037006832	0,128739314	0	0	2,008300977	6,620670928	EEF1B2;RPL32;RPS19;RPS8;RPS6;RPS20;RPL39;TOMM20;TPT1
Irinotecan-5_mg/kg_in_Saline-Rat-Kidney-3d-dn	9/308	0,037006832	0,128682475	0	0	2,008300977	6,620670928	EEF1B2;RPS17;RPS8;FUNDC1;RPS6;RPS20;RPS3A;RPL39;TPT1
Leflunomide-60_mg/kg_in_Corn_Oil-Rat-Bone_marrow-1d-up	9/308	0,037006832	0,128625687	0	0	2,008300977	6,620670928	C1QA;RPS17;RPS19;CLP1;RPS5;RPS20;SEPP1;RPL39;TPT1
Lomustine-8.75_mg/kg_in_Corn_Oil-Rat-Bone_marrow-1d-up	9/308	0,037006832	0,128568948	0	0	2,008300977	6,620670928	C1QA;RPS17;RPS19;NKG7;RPS20;RPS3A;RPL10A;RPL39;TPT1
Methotrexate-0.3_mg/kg_in_Corn_Oil-Rat-Liver-1d-up	9/308	0,037006832	0,12851226	0	0	2,008300977	6,620670928	EEF1B2;C1QA;RPL32;RPS19;RPS8;RPS6;RPS20;RPSA;RPS3A
Moxonidine-17_mg/kg_in_CMC-Rat-Heart-1d-up	9/308	0,037006832	0,128455622	0	0	2,008300977	6,620670928	RPL32;RPS19;RPS6;RPL35A;RPS20;RAPGEF5;RPL10A;RPL39;PDRG1
Niacinamide-750_mg/kg_in_Water-Rat-Liver-5d-dn	9/308	0,037006832	0,128399034	0	0	2,008300977	6,620670928	EEF1B2;RPL32;RPS19;FUNDC1;RPS6;RPS20;RPL10A;RPL39;TPT1
Nimesulide-61.7_uM_in_DMSO-Rat-Primary_rat_hepatocytes-0.67d-dn	9/308	0,037006832	0,128342495	0	0	2,008300977	6,620670928	RPS17;CXCL11;RPL32;ARL6IP5;RPS20;RPS3A;SEPP1;RPL39;TPT1
Pravastatin-1200_mg/kg_in_Corn_Oil-Rat-Kidney-3d-dn	9/308	0,037006832	0,128286006	0	0	2,008300977	6,620670928	RPS17;RPL32;RPS19;RPS6;RPSA;RPS3A;RPL10A;RPL39;TPT1
Rosiglitazone-1800_mg/kg_in_Corn_Oil-Rat-Skeletal_muscle-3d-dn	9/308	0,037006832	0,128229567	0	0	2,008300977	6,620670928	EEF1B2;C1QA;RPS17;FEM1B;COL5A3;ARL6IP5;RPSA;RPL39;SLC16A3
Stannous_Fluoride-90_mg/kg_in_Water-Rat-Kidney-1d-up	9/308	0,037006832	0,128173178	0	0	2,008300977	6,620670928	EEF1B2;RPS17;RPL32;RPS19;RPS8;RPS6;RPS20;RPS3A;RPL10A
Tinidazole-1200_mg/kg_in_CMC-Rat-Liver-1d-dn	9/308	0,037006832	0,128116838	0	0	2,008300977	6,620670928	EEF1B2;C1QA;RPL32;RPS19;RPS8;RPS6;RPS20;SEPP1;TPT1
Tolazamide-1500_mg/kg_in_CMC-Rat-Liver-3d-up	9/308	0,037006832	0,128060547	0	0	2,008300977	6,620670928	RPS17;RPS19;RPS20;RPSA;RPS3A;RPL10A;SEPP1;GOLGA7;TPT1
Acetaminophen-100_mg/kg_in_Corn_Oil-Rat-Liver-1d-dn	8/260	0,037040152	0,128119559	0	0	2,114723764	6,969606707	C1QA;RPS17;CLP1;RPS6;RPS3A;RPL10A;RPL39;TPT1
4-Nonylphenol-200_mg/kg_in_Corn_Oil-Rat-Kidney-5d-dn	9/309	0,037652941	0,130181984	0	0	2,001801621	6,564596583	EEF1B2;RPS17;RPL32;RPS8;RPS5;RPL35A;RPS3A;SEPP1;RPL39
Benzethonium_Chloride-30_mg/kg_in_Corn_Oil-Rat-Heart-3d-dn	9/309	0,037652941	0,130124862	0	0	2,001801621	6,564596583	EEF1B2;RPS17;RPL32;RPS8;ARL6IP5;RPS20;RPSA;RPS3A;TPT1
Carmustine-4_mg/kg_in_Corn_Oil-Rat-Bone_marrow-1d-up	9/309	0,037652941	0,13006779	0	0	2,001801621	6,564596583	C1QA;RPS19;RPS8;RPS5;NKG7;RPL35A;SEPP1;RPL39;DUSP14
Clopidogrel-400_mg/kg_in_Corn_Oil-Rat-Bone_marrow-0.25d-dn	9/309	0,037652941	0,130010767	0	0	2,001801621	6,564596583	RPS17;RPS19;RPS5;NKG7;RPS20;RPSA;RPL10A;SEPP1;TPT1
Diazepam-710_mg/kg_in_CMC-Rat-Heart-1d-dn	9/309	0,037652941	0,129953795	0	0	2,001801621	6,564596583	RPS19;COL5A3;RPS6;RPL10A;SEPP1;RPL39;GOLGA7;TPT1;PDRG1
Isoniazid-79_mg/kg_in_Water-Rat-Liver-3d-up	9/309	0,037652941	0,129896873	0	0	2,001801621	6,564596583	RPS17;RPL32;RPS8;RPS5;RPS6;RPL35A;RPSA;RPS3A;TPT1
Lovastatin-1500_mg/kg_in_Corn_Oil-Rat-Liver-0.25d-up	9/309	0,037652941	0,12984	0	0	2,001801621	6,564596583	C1QA;RPS19;RPS8;RPS6;ARL6IP5;RPSA;SEPP1;GOLGA7;TPT1
NN'-Diphenyl-P-Phenylenediamine-1000_mg/kg_in_CMC-Rat-Kidney-5d-dn	9/309	0,037652941	0,129783177	0	0	2,001801621	6,564596583	RPS19;ARL2BP;RPS8;RPS6;RPS20;RPL10A;SEPP1;RPL39;PDRG1
Neomycin-56_mg/kg_in_Corn_Oil-Rat-Liver-3d-dn	9/309	0,037652941	0,129726404	0	0	2,001801621	6,564596583	EEF1B2;RPS17;RPL32;RPS8;RPS6;RPSA;RPS3A;RPL10A;TPT1
Sulpiride-667_uM_in_DMSO-Rat-Primary_rat_hepatocytes-0.67d-up	9/309	0,037652941	0,129669681	0	0	2,001801621	6,564596583	RPS17;FEM1B;RPSA;RPS3A;RPL10A;RPL39;SLC16A3;TPT1;PDRG1
Timolol-900_mg/kg_in_Water-Rat-Heart-3d-up	9/309	0,037652941	0,129613007	0	0	2,001801621	6,564596583	RPS17;RPL32;RPS19;RPS5;RPSA;RPS3A;RPL10A;SEPP1;RPL39
Tolazamide-1500_mg/kg_in_CMC-Rat-Kidney-1d-up	9/309	0,037652941	0,129556383	0	0	2,001801621	6,564596583	EEF1B2;RPS17;CLP1;RPS8;RPS5;HOXA1;RPS3A;SEPP1;TPT1
Acetazolamide-250_mg/kg_in_Corn_Oil-Rat-Kidney-3d-up	8/261	0,03775112	0,129837477	0	0	2,106621374	6,902850799	EEF1B2;RPS17;RPL32;ARL2BP;RPL13A;RPL35A;RPSA;RPS3A
Cisplatin-2_mg/kg_in_Saline-Rat-Spleen-5d-up	8/261	0,03775112	0,129780804	0	0	2,106621374	6,902850799	CXCL11;ARL2BP;FUNDC1;NKG7;RAPGEF5;RPL39;GOLGA7;TPT1
Clofibrate-130_mg/kg_in_Corn_Oil-Rat-Liver-0.25d-up	8/261	0,03775112	0,129724181	0	0	2,106621374	6,902850799	RPS17;RPL32;RPS19;RPS6;RPS20;RPS3A;RPL10A;SEPP1
Sertraline-23_uM_in_DMSO-Rat-Primary_rat_hepatocytes-1d-up	8/261	0,03775112	0,129667607	0	0	2,106621374	6,902850799	EEF1B2;RPS17;RPS6;RPS20;RPSA;RPS3A;RPL10A;PDRG1
Sildenafil-420_mg/kg_in_Water-Rat-Liver-3d-up	10/359	0,038048318	0,130631452	0	0	1,914443519	6,258121369	EEF1B2;RPS17;RPS19;RPS8;RPS6;RPL35A;RPS20;RPSA;RPS3A;RPL39
Carboplatin-5_mg/kg_in_Saline-Rat-Kidney-5d-up	9/310	0,038306723	0,131461329	0	0	1,995344197	6,509071892	CXCL11;RPL32;CLP1;RPS8;RPS6;RPS20;RPSA;RPL39;TPT1
Ethylene_Glycol-352_mg/kg_in_Water-Rat-Bone_marrow-1d-dn	9/310	0,038306723	0,131404072	0	0	1,995344197	6,509071892	C1QA;RPS17;RPS19;FEM1B;ARL6IP5;RPSA;SEPP1;RPL39;DUSP14
Etoposide-100_mg/kg_in_Corn_Oil-Rat-Bone_marrow-0.25d-dn	9/310	0,038306723	0,131346866	0	0	1,995344197	6,509071892	RPS17;RPL32;RPS8;RPS5;RPS6;RPSA;RPS3A;RPL10A;TPT1
Glipizide-2500_mg/kg_in_CMC-Rat-Liver-1d-dn	9/310	0,038306723	0,131289709	0	0	1,995344197	6,509071892	EEF1B2;C1QA;RPS17;RPL32;RPS6;RPL35A;RPS20;SEPP1;RPL39
Lincomycin-1200_mg/kg_in_CMC-Rat-Kidney-5d-up	9/310	0,038306723	0,131232601	0	0	1,995344197	6,509071892	RPS17;RPS19;FEM1B;RPS6;RPSA;RAPGEF5;SEPP1;RPL39;TPT1
Mebendazole-50_mg/kg_in_Corn_Oil-Rat-Liver-0.25d-up	9/310	0,038306723	0,131175544	0	0	1,995344197	6,509071892	C1QA;RPL32;RPS19;RPS8;RPS6;RPSA;RPL10A;SEPP1;GOLGA7
Nateglinide-625_mg/kg_in_CMC-Rat-Kidney-5d-dn	9/310	0,038306723	0,131118536	0	0	1,995344197	6,509071892	RPS19;ARL2BP;RPS8;RPS6;RPSA;RPL10A;RPL39;TPT1;PDRG1
Nizatidine-1653_mg/kg_in_Corn_Oil-Rat-Heart-1d-up	9/310	0,038306723	0,131061577	0	0	1,995344197	6,509071892	RPS19;RPS6;RPSA;RAPGEF5;RPL10A;SEPP1;RPL39;GOLGA7;TPT1
Noscapine-570_mg/kg_in_CMC-Rat-Spleen-1d-up	9/310	0,038306723	0,131004668	0	0	1,995344197	6,509071892	RPL32;RPS19;RPS8;RPS5;RPL35A;RPSA;RPL10A;SEPP1;TPT1
Pergolide-1.1_mg/kg_in_CMC-Rat-Liver-1d-dn	9/310	0,038306723	0,130947808	0	0	1,995344197	6,509071892	C1QA;RPS17;RPL32;RPS19;FEM1B;RPL35A;RPS20;RPL10A;RPL39
Streptozotocin-138_mg/kg_in_Saline-Rat-Liver-5d-dn	9/310	0,038306723	0,130890998	0	0	1,995344197	6,509071892	EEF1B2;RPS17;RPL32;RPS19;CLP1;RPS8;RPSA;RPL10A;TPT1
Trichloroacetic_Acid-474_mg/kg_in_Corn_Oil-Rat-Liver-3d-dn	9/310	0,038306723	0,130834237	0	0	1,995344197	6,509071892	RPS17;RPL32;RPS19;RPS8;RPS6;RPSA;RPS3A;SEPP1;TPT1
Isoniazid-50_mg/kg_in_Saline-Rat-Liver-1d-dn	8/262	0,038471208	0,13133907	0	0	2,098580835	6,83685142	RPS19;RPS8;RPS6;RPS20;RPS3A;RPL10A;RPL39;TPT1
Mitomycin_C-0.5_mg/kg_in_Saline-Rat-Liver-0.25d-up	8/262	0,038471208	0,131282164	0	0	2,098580835	6,83685142	RPS17;RPL32;RPS8;RPL35A;RPSA;RPS3A;SEPP1;TPT1
Nevirapine-29_mg/kg_in_Saline-Rat-Liver-3d-up	8/262	0,038471208	0,131225307	0	0	2,098580835	6,83685142	RPS17;RPL32;RPS6;RPL35A;RPS20;RPSA;RPL10A;RPL39
Nimesulide-61.7_uM_in_DMSO-Rat-Primary_rat_hepatocytes-1d-up	8/262	0,038471208	0,1311685	0	0	2,098580835	6,83685142	EEF1B2;C1QA;RPS8;RPS6;RPS3A;RPL10A;SLC16A3;PDRG1
Amiloride-22_mg/kg_in_CMC-Rat-Heart-1d-dn	10/360	0,038654882	0,131737711	0	0	1,90912562	6,210542577	RPS17;RPL32;RPS8;RPS6;ARL6IP5;RPS20;RPSA;RPS3A;RPL10A;RPL39
Procaine-2500_mg/kg_in_Water-Rat-Heart-3d-up	10/360	0,038654882	0,131680731	0	0	1,90912562	6,210542577	RPS17;RPL32;RPS19;ARL2BP;RPS8;FUNDC1;RPS6;ARL6IP5;RPS20;RPSA
Benzoic_Acid-1700_mg/kg_in_CMC-Rat-Liver-1d-up	9/311	0,038968213	0,132690723	0	0	1,988928299	6,454090285	CXCL11;RPS19;RPS6;RPSA;RPS3A;RPL10A;RPL39;GOLGA7;TPT1
Cinnarizine-750_mg/kg_in_CMC-Rat-Liver-3d-up	9/311	0,038968213	0,13263338	0	0	1,988928299	6,454090285	RPL32;RPS19;RPS8;RPS6;RPS20;RPSA;RPS3A;RPL10A;RPL39
Fenbendazole-375_mg/kg_in_Corn_Oil-Rat-Liver-1d-up	9/311	0,038968213	0,132576087	0	0	1,988928299	6,454090285	RPS19;RPS6;RPS20;RPSA;RPS3A;RPL10A;SEPP1;RPL39;TPT1
Glimepiride-2500_mg/kg_in_CMC-Rat-Liver-5d-dn	9/311	0,038968213	0,132518844	0	0	1,988928299	6,454090285	EEF1B2;RPL32;RPS19;RPS6;RPS3A;RPL10A;SEPP1;RPL39;TPT1
Hydroxyurea-400_mg/kg_in_Saline-Rat-Bone_marrow-1d-up	9/311	0,038968213	0,13246165	0	0	1,988928299	6,454090285	C1QA;RPS19;ARL2BP;RPS8;RPS6;NKG7;RPS20;RPL10A;SEPP1
Proglumide-1125_mg/kg_in_CMC-Rat-Liver-5d-dn	9/311	0,038968213	0,132404505	0	0	1,988928299	6,454090285	EEF1B2;RPL32;RPS19;CLP1;RPS8;FEM1B;RPS6;RPS3A;TPT1
SCH-351591-4.6_mg/kg_in_CMC-Rat-Liver-6d-dn	9/311	0,038968213	0,132347409	0	0	1,988928299	6,454090285	C1QA;RPS17;RPL32;RPS19;RPS8;ARL6IP5;RPL35A;RPS3A;TPT1
Valdecoxib-404_mg/kg_in_CMC-Rat-Kidney-3d-up	9/311	0,038968213	0,132290363	0	0	1,988928299	6,454090285	EEF1B2;C1QA;RPS17;RPS8;RPS5;RPS6;ARL6IP5;RPL35A;PDRG1
Altretamine-40_mg/kg_in_Corn_Oil-Rat-Spleen-5d-up	8/263	0,039200455	0,133021449	0	0	2,09060144	6,771597974	C1QA;CXCL11;RPL32;RPS19;CLP1;RPS8;RPS20;RPL10A
Acetazolamide-47_mg/kg_in_Corn_Oil-Rat-Kidney-1d-up	9/312	0,039637443	0,134446382	0	0	1,982553529	6,399645285	RPS17;RPL32;RPS19;RPS5;ARL6IP5;RPL13A;RPL35A;RPSA;RPS3A
Acyclovir-980_mg/kg_in_Saline-Rat-Bone_marrow-5d-dn	9/312	0,039637443	0,134388506	0	0	1,982553529	6,399645285	EEF1B2;RPS17;RPL32;COL5A3;RPL35A;RPL10A;SEPP1;RPL39;DUSP14
Allyl_Alcohol-25_mg/kg_in_Saline-Rat-Bone_marrow-1d-dn	9/312	0,039637443	0,13433068	0	0	1,982553529	6,399645285	EEF1B2;RPL32;RPS19;CLP1;RPS8;RPS6;RPS20;IL12A;RPL10A
Allyl_Alcohol-25_mg/kg_in_Saline-Rat-Liver-3d-up	9/312	0,039637443	0,134272903	0	0	1,982553529	6,399645285	EEF1B2;RPS17;RPL32;RPS8;FEM1B;RPS5;RPS6;RPS20;RPSA
Benazepril-1750_mg/kg_in_CMC-Rat-Kidney-5d-up	9/312	0,039637443	0,134215176	0	0	1,982553529	6,399645285	EEF1B2;RPL32;RPS19;RPS6;RPL35A;RPS20;RPSA;RPL39;TPT1
Bisphenol_A-100_mg/kg_in_Corn_Oil-Rat-Bone_marrow-0.25d-dn	9/312	0,039637443	0,134157499	0	0	1,982553529	6,399645285	EEF1B2;RPS17;RPL32;RPS8;RPS5;ARL6IP5;RPSA;RPS3A;RPL10A
Capsaicin-35_mg/kg_in_CMC-Rat-Liver-5d-dn	9/312	0,039637443	0,134099871	0	0	1,982553529	6,399645285	EEF1B2;RPL32;RPS19;RPS8;FEM1B;RPS6;RPS3A;RPL10A;TPT1
Captopril-1750_mg/kg_in_Water-Rat-Kidney-1d-dn	9/312	0,039637443	0,134042293	0	0	1,982553529	6,399645285	EEF1B2;RPS17;RPS19;RPS6;ARL6IP5;RPL35A;RPS20;RPSA;SEPP1
Ethosuximide-1200_mg/kg_in_Water-Rat-Heart-1d-dn	9/312	0,039637443	0,133984764	0	0	1,982553529	6,399645285	RPS17;RPL32;RPS19;FUNDC1;RPSA;RPS3A;RPL10A;SEPP1;TPT1
Griseofulvin-75_uM_in_DMSO-Rat-Primary_rat_hepatocytes-0.67d-dn	9/312	0,039637443	0,133927284	0	0	1,982553529	6,399645285	EEF1B2;CXCL11;RPL32;RPS8;RPS5;ARL6IP5;RPSA;RPL10A;SEPP1
Mitomycin_C-1.7_mg/kg_in_Saline-Rat-Liver-1d-dn	9/312	0,039637443	0,133869854	0	0	1,982553529	6,399645285	EEF1B2;RPS19;RPS8;RPS6;ARL6IP5;RPS20;RPSA;RPL10A;SEPP1
Prazosin-2000_mg/kg_in_CMC-Rat-Heart-1d-dn	9/312	0,039637443	0,133812473	0	0	1,982553529	6,399645285	EEF1B2;RPS17;RPL32;ARL2BP;RPS8;ARL6IP5;RPS20;RPS3A;RPL39
Sparfloxacin-29_mg/kg_in_Corn_Oil-Rat-Liver-3d-dn	9/312	0,039637443	0,133755141	0	0	1,982553529	6,399645285	EEF1B2;RPS17;RPL32;RPS19;RPS8;RPS6;RPS3A;RPL10A;TPT1
Sulbactam-671_mg/kg_in_Saline-Rat-Kidney-5d-dn	9/312	0,039637443	0,133697859	0	0	1,982553529	6,399645285	ARL2BP;RPS8;FEM1B;FUNDC1;RPS6;RPS20;RPL10A;SEPP1;TPT1
Acetaminophen-100_mg/kg_in_Corn_Oil-Rat-Kidney-1d-up	10/362	0,039887995	0,134485379	0	0	1,898577965	6,116610449	RPS17;RPL32;RPS19;RPS8;RPS5;RPS6;RPS20;RPSA;RPS3A;SEPP1
Lead_(II)_Acetate-200_mg/kg_in_Saline-Rat-Bone_marrow-1d-dn	7/217	0,03996534	0,134688498	0	0	2,217049108	7,13832767	EEF1B2;RPS19;RPS6;RPS3A;RPL10A;RPL39;SEPP1

4-Octylphenol-450_mg/kg_in_Corn_Oil-Rat-Liver-3d-dn	9/313	0,040314446	0,135806921	0	0	1,976219492	6,345730512	RPS17;RPL32;RPS19;RPS8;RPS6;RPS3A;RPL10A;RPL39;TPT1
Acyclovir-980_mg/kg_in_Saline-Rat-Spleen-5d-up	9/313	0,040314446	0,135748859	0	0	1,976219492	6,345730512	C1QA;RPS17;RPL32;RPS6;NKG7;RPS20;RPS3A;RPL39;TPT1
Albendazole-62_mg/kg_in_Corn_Oil-Rat-Liver-5d-up	9/313	0,040314446	0,135690846	0	0	1,976219492	6,345730512	C1QA;RPS17;RPS20;RPSA;RPS3A;RAPGEF5;SEPP1;RPL39;TPT1
Benzethonium_Chloride-138_mg/kg_in_Water-Rat-Heart-5d-up	9/313	0,040314446	0,135632883	0	0	1,976219492	6,345730512	RPS17;RPL32;RPS8;RPS6;RPL35A;RPS3A;RPL39;GOLGA7;TPT1
Hydralazine-280_mg/kg_in_Water-Rat-Heart-3d-dn	9/313	0,040314446	0,13557497	0	0	1,976219492	6,345730512	EEF1B2;RPS17;RPL32;RPS19;RPS6;RPSA;RPS3A;RPL10A;RPL39
Kanamycin-225_mg/kg_in_Saline-Rat-Kidney-5d-up	9/313	0,040314446	0,135517106	0	0	1,976219492	6,345730512	C1QA;RPS17;RPL32;ARL6IP5;RPL35A;RPSA;RPL39;GOLGA7;TPT1
Methyl_Salicylate-444_mg/kg_in_Corn_Oil-Rat-Liver-3d-dn	9/313	0,040314446	0,135459292	0	0	1,976219492	6,345730512	EEF1B2;RPS17;RPL32;RPS19;RPS6;RPS20;RPL10A;RPL39;TPT1
Methyldopa-325_mg/kg_in_Water-Rat-Liver-5d-dn	9/313	0,040314446	0,135401527	0	0	1,976219492	6,345730512	RPS17;RPL32;RPS19;RPS6;RPL35A;RPS20;RPSA;RPL10A;RPL39
Ranitidine-1500_mg/kg_in_CMC-Rat-Heart-3d-up	9/313	0,040314446	0,135343811	0	0	1,976219492	6,345730512	RPS17;RPL32;RPS6;ARL6IP5;RPSA;RPS3A;RAPGEF5;TOMM20;TPT1
Sotalol-2000_mg/kg_in_Water-Rat-Heart-5d-up	9/313	0,040314446	0,135286144	0	0	1,976219492	6,345730512	RPS17;ARL2BP;FUNDC1;RPS20;RPSA;RAPGEF5;RPL10A;SEPP1;GOLGA7
Valsartan-1000_mg/kg_in_Corn_Oil-Rat-Heart-1d-dn	9/313	0,040314446	0,135228527	0	0	1,976219492	6,345730512	EEF1B2;RPL32;RPS19;ARL2BP;RPS8;FUNDC1;RPS6;ARL6IP5;RPSA
Zidovudine-1540_mg/kg_in_Corn_Oil-Rat-Liver-3d-up	9/313	0,040314446	0,135170958	0	0	1,976219492	6,345730512	RPL32;RPS8;RPS6;RPS20;RPS3A;RPL10A;SEPP1;RPL39;TPT1
Clobetasol_Propionate-17_mg/kg_in_Corn_Oil-Rat-Bone_marrow-5d-dn	8/265	0,040686582	0,136360646	0	0	2,074823316	6,643287413	RPS17;RPS19;RPS8;FUNDC1;RPS6;RPSA;RPL10A;RPL39
Diethylstilbestrol-280_mg/kg_in_Corn_Oil-Rat-Spleen-3d-up	8/265	0,040686582	0,136302644	0	0	2,074823316	6,643287413	EEF1B2;RPL32;RPS8;RPS5;RPS6;RPL35A;RPS20;RPS3A
Dipyridamole-750_mg/kg_in_CMC-Rat-Liver-1d-dn	8/265	0,040686582	0,136244693	0	0	2,074823316	6,643287413	EEF1B2;RPL32;RPS19;CLP1;RPS6;RPSA;RPS3A;RPL10A
Streptomycin-120_mg/kg_in_Saline-Rat-Bone_marrow-3d-dn	8/265	0,040686582	0,13618679	0	0	2,074823316	6,643287413	C1QA;RPL32;RPS8;RPS6;RPL35A;RPS3A;RPL39;TPT1
Losartan-1000_mg/kg_in_Water-Rat-Kidney-5d-dn	7/218	0,040801058	0,136511953	0	0	2,206879158	7,05991071	RPS17;RPL32;RPL35A;RPS20;RPSA;RPL39;SEPP1
Chlorpromazine-18_mg/kg_in_Water-Rat-Liver-3d-up	9/314	0,040999256	0,137116831	0	0	1,969925799	6,292339679	EEF1B2;C1QA;CXCL11;CLP1;ARL6IP5;RPS3A;SEPP1;RPL39;TPT1
Clopidogrel-400_mg/kg_in_Corn_Oil-Rat-Bone_marrow-5d-dn	9/314	0,040999256	0,137058632	0	0	1,969925799	6,292339679	RPS19;RPS5;ARL6IP5;NKG7;RPS20;RPSA;RPL10A;SEPP1;TPT1
Clotrimazole-178_mg/kg_in_Corn_Oil-Rat-Liver-3d-dn	9/314	0,040999256	0,137000483	0	0	1,969925799	6,292339679	EEF1B2;C1QA;RPS17;RPL32;CLP1;RPS3A;SEPP1;RPL39;GOLGA7
Cortisone-206_mg/kg_in_CMC-Rat-Kidney-3d-dn	9/314	0,040999256	0,136942383	0	0	1,969925799	6,292339679	C1QA;RPS17;RPL32;RPS6;RPL35A;RPS20;RPL10A;GOLGA7;TPT1
Diphenhydramine-100_mg/kg_in_Water-Rat-Heart-5d-up	9/314	0,040999256	0,136884332	0	0	1,969925799	6,292339679	RPS17;RPL32;RPS8;RPS6;RPSA;RPL10A;SEPP1;RPL39;GOLGA7
Doxorubicin-0.65_mg/kg_in_Saline-Rat-Bone_marrow-0.25d-up	9/314	0,040999256	0,13682633	0	0	1,969925799	6,292339679	RPS19;ARL2BP;RPS8;RPS5;FUNDC1;RPS6;RPS20;RPSA;TPT1
Ondansetron-84_mg/kg_in_Water-Rat-Intestine-1d-dn	9/314	0,040999256	0,136768377	0	0	1,969925799	6,292339679	RPS17;RPL32;RPS8;RPS5;RPS6;RPL35A;RPS3A;RPL10A;TPT1
Paroxetine-104_mg/kg_in_Water-Rat-Heart-3d-up	9/314	0,040999256	0,136710473	0	0	1,969925799	6,292339679	ARL2BP;RPS8;FUNDC1;ARL6IP5;RPSA;RAPGEF5;RPL10A;GOLGA7;TPT1
Allylamine-75_mg/kg_in_Water-Rat-Heart-3d-dn	10/364	0,041147941	0,137148194	0	0	1,888146218	6,024284194	EEF1B2;RPS17;RPL32;RPS19;RPS8;RPS6;RPS20;RPSA;RPS3A;TPT1
Aspirin-35_mg/kg_in_Corn_Oil-Rat-Liver-5d-dn	10/364	0,041147941	0,137090178	0	0	1,888146218	6,024284194	EEF1B2;C1QA;RPS17;RPL32;RPS19;RPS6;RPS3A;RPL10A;RPL39;TPT1
Propranolol-175_mg/kg_in_Water-Rat-Heart-3d-up	10/364	0,041147941	0,137032212	0	0	1,888146218	6,024284194	RPS17;RPL32;RPS19;FUNDC1;RPS6;ARL6IP5;RPL35A;RPSA;RPS3A;RPL10A
Daunorubicin-3.25_mg/kg_in_Saline-Rat-Heart-3d-dn	8/266	0,041443536	0,137958281	0	0	2,067023228	6,580210008	EEF1B2;RPS17;RPL32;ARL2BP;RPS8;FUNDC1;RPS6;RPS20
Etoposide-100_mg/kg_in_Corn_Oil-Rat-Spleen-3d-up	8/266	0,041443536	0,137899997	0	0	2,067023228	6,580210008	EEF1B2;RPL32;RPS5;RPS6;RPL35A;RPS20;RPS3A;RAPGEF5
Clotrimazole-178_mg/kg_in_Corn_Oil-Rat-Liver-0.25d-dn	9/315	0,041691902	0,13866783	0	0	1,963672067	6,239466588	EEF1B2;RPL32;RPS8;RPS6;RPS20;RPS3A;SEPP1;RPL39;TPT1
Eperisone-501_mg/kg_in_CMC-Rat-Liver-5d-dn	9/315	0,041691902	0,138609296	0	0	1,963672067	6,239466588	EEF1B2;RPS17;RPL32;RPS8;RPS6;RPL35A;RPS20;RPL10A;TPT1
Ifosfamide-17_mg/kg_in_Saline-Rat-Bone_marrow-0.25d-dn	9/315	0,041691902	0,138550811	0	0	1,963672067	6,239466588	EEF1B2;RPS17;RPL32;RPS6;NKG7;RPS3A;RPL39;GOLGA7;TPT1
Itraconazole-1093_mg/kg_in_Corn_Oil-Rat-Liver-0.25d-up	9/315	0,041691902	0,138492375	0	0	1,963672067	6,239466588	CXADR;RPS19;RPS6;RPSA;RPS3A;RPL10A;SEPP1;RPL39;TPT1
Zidovudine-1540_mg/kg_in_Corn_Oil-Rat-Heart-3d-up	10/365	0,041788045	0,138753223	0	0	1,882973215	5,978712943	RPS8;COL5A3;FUNDC1;RPS6;ARL6IP5;RPS20;RPSA;RPL10A;RPL39;TPT1
Carmustine-4_mg/kg_in_Corn_Oil-Rat-Spleen-3d-up	8/267	0,042209801	0,140094559	0	0	2,059281568	6,517837947	EEF1B2;RPL32;RPS8;RPS5;RPS6;RPL35A;RPS20;RPS3A
Foscarnet-350_mg/kg_in_Saline-Rat-Kidney-1d-dn	8/267	0,042209801	0,140035547	0	0	2,059281568	6,517837947	RPL32;RPS19;RPS6;RPS20;RPL10A;RPL39;SEPP1;TPT1
Gentian_Violet-18_mg/kg_in_Corn_Oil-Rat-Liver-1d-up	8/267	0,042209801	0,139976585	0	0	2,059281568	6,517837947	C1QA;RPL32;RPS8;RPS5;RPS20;RPSA;SEPP1;TPT1
Acetaminophen-100_mg/kg_in_Corn_Oil-Rat-Liver-3d-dn	9/316	0,042392417	0,140523012	0	0	1,957457915	6,187105134	EEF1B2;RPS17;RPL32;RPS8;RPS6;RPS3A;RPL10A;RPL39;TPT1
Amoxapine-313_mg/kg_in_CMC-Rat-Liver-5d-up	9/316	0,042392417	0,140463894	0	0	1,957457915	6,187105134	RPS17;RPL32;RPS19;RPS8;RPS5;RPS6;RPSA;RPS3A;TPT1
Diisopropyl_Fluorophosphate-3.75_mg/kg_in_Water-Rat-Liver-5d-dn	9/316	0,042392417	0,140404826	0	0	1,957457915	6,187105134	EEF1B2;RPL32;RPS19;RPS6;RPS20;RPS3A;RPL10A;SEPP1;TPT1
Penciclovir-1200_mg/kg_in_Corn_Oil-Rat-Kidney-3d-dn	9/316	0,042392417	0,140345808	0	0	1,957457915	6,187105134	EEF1B2;RPS17;RPL32;RPS6;RPS20;RPSA;RPS3A;RPL39;TPT1
Temafloxacin-1000_mg/kg_in_CMC-Rat-Kidney-3d-up	9/316	0,042392417	0,140286839	0	0	1,957457915	6,187105134	EEF1B2;RPS17;RPS8;RPS6;ARL6IP5;RPS3A;RPL10A;RPL39;TPT1
Thioguanine-24_mg/kg_in_Corn_Oil-Rat-Bone_marrow-5d-up	9/316	0,042392417	0,14022792	0	0	1,957457915	6,187105134	EEF1B2;C1QA;RPL32;NKG7;RPL35A;RPS20;RPS3A;SEPP1;RPL39
Dexamethasone-1_mg/kg_in_Corn_Oil-Rat-Heart-3d-dn	8/268	0,04298541	0,14212976	0	0	2,051597682	6,456161515	RPS17;RPL32;RPS19;COL5A3;RPS6;RPS3A;GOLGA7;TPT1
N-Nitrosodiethylamine-34_mg/kg_in_Saline-Rat-Spleen-3d-dn	8/268	0,04298541	0,142070117	0	0	2,051597682	6,456161515	EEF1B2;C1QA;RPL32;ARL2BP;RPS8;RPS6;ARL6IP5;RPSA
Ubenimex-1300_mg/kg_in_Water-Rat-Kidney-3d-up	10/367	0,043088652	0,142351602	0	0	1,87271178	5,888733993	RPL32;RPS19;RPS6;RPS20;RPSA;RPS3A;RAPGEF5;RPL10A;SEPP1;RPL39
Acyclovir-330_mg/kg_in_Saline-Rat-Spleen-1d-up	9/317	0,043100831	0,142332135	0	0	1,951282969	6,135249299	C1QA;RPL32;RPS19;RPS6;NKG7;RPS20;RPL10A;SEPP1;RPL39
Beta-Naphthoflavone-1500_mg/kg_in_CMC-Rat-Liver-3d-up	9/317	0,043100831	0,142272482	0	0	1,951282969	6,135249299	EEF1B2;RPS17;RPL32;RPS8;RPL35A;RPS20;RPS3A;RPL10A;SEPP1
Digoxin-11_mg/kg_in_Corn_Oil-Rat-Liver-3d-dn	9/317	0,043100831	0,142212879	0	0	1,951282969	6,135249299	EEF1B2;RPS17;RPL32;RPS8;RPS6;RPSA;RPS3A;RPL10A;TPT1
Doxorubicin-3_mg/kg_in_Saline-Rat-Liver-3d-up	9/317	0,043100831	0,142153326	0	0	1,951282969	6,135249299	EEF1B2;RPS17;RPS19;RPS8;RPS5;RPS6;RPS20;RPSA;SEPP1
N-Nitrosodiethylamine-100_mg/kg_in_Saline-Rat-Liver-5d-dn	9/317	0,043100831	0,142093822	0	0	1,951282969	6,135249299	EEF1B2;RPS17;RPL32;CLP1;RPS8;RPS6;RPL35A;RPS20;RPL39
Norfloxacin-1500_mg/kg_in_CMC-Rat-Kidney-1d-up	9/317	0,043100831	0,142034369	0	0	1,951282969	6,135249299	C1QA;RPS17;RPL32;RPS19;ARL2BP;RPS6;RPS20;RAPGEF5;RPL39
Penicillamine-100_mg/kg_in_Water-Rat-Liver-0.25d-up	9/317	0,043100831	0,141974965	0	0	1,951282969	6,135249299	EEF1B2;C1QA;RPS17;RPS8;RPS6;RPS20;RPS3A;SEPP1;RPL39
Pioglitazone-1500_mg/kg_in_Corn_Oil-Rat-Liver-1d-up	9/317	0,043100831	0,141915611	0	0	1,951282969	6,135249299	EEF1B2;C1QA;RPL32;RPS19;RPS6;RPS20;RPL10A;RPL39;GOLGA7
Probenecid-600_mg/kg_in_Corn_Oil-Rat-Kidney-0.25d-up	9/317	0,043100831	0,141856306	0	0	1,951282969	6,135249299	RPS17;RPL32;RPS19;RPSA;RPS3A;RPL10A;SEPP1;RPL39;TPT1
Tacrine-24_mg/kg_in_Water-Rat-Heart-5d-up	9/317	0,043100831	0,141797052	0	0	1,951282969	6,135249299	RPS17;RPL32;ARL2BP;RPS8;RPS5;FUNDC1;ARL6IP5;RPL35A;RPS3A
Lansoprazole-240_uM_in_DMSO-Rat-Primary_rat_hepatocytes-1d-dn	10/368	0,043749206	0,143870039	0	0	1,86762289	5,844318365	CXCL11;RPL32;RPS19;RPS8;RPS6;ARL6IP5;RPS20;SEPP1;RPL39;TPT1
Meloxicam-33_mg/kg_in_Corn_Oil-Rat-Liver-5d-up	8/269	0,043770399	0,143879658	0	0	2,043970925	6,395171158	RPS17;RPL32;RPS19;CLP1;RPS8;RPL35A;RPS20;TPT1
Aldosterone-0.065_mg/kg_in_Saline-Rat-Kidney-3d-dn	9/318	0,043817173	0,143973322	0	0	1,945146859	6,083893152	EEF1B2;RPS17;RPL32;RPS6;RPS20;RPS3A;RPL10A;RPL39;TPT1
Fenofibrate-215_mg/kg_in_Corn_Oil-Rat-Liver-3d-up	9/318	0,043817173	0,143913283	0	0	1,945146859	6,083893152	RPS17;RPL32;RPS19;RPS8;RPS6;RPL35A;RPS20;RPSA;RPL39
Fluvastatin-5_mg/kg_in_Corn_Oil-Rat-Liver-0.25d-up	9/318	0,043817173	0,143853294	0	0	1,945146859	6,083893152	EEF1B2;RPL32;RPS19;RPS6;RPS20;RPS3A;RPL10A;SEPP1;TPT1
Hexachlorophene-8_mg/kg_in_Corn_Oil-Rat-Liver-5d-up	9/318	0,043817173	0,143793355	0	0	1,945146859	6,083893152	RPS17;RPL32;RPS19;RPS8;RPS5;RPS20;SEPP1;RPL39;TPT1
Nadolol-1500_mg/kg_in_CMC-Rat-Heart-5d-dn	9/318	0,043817173	0,143733466	0	0	1,945146859	6,083893152	C1QA;RPS17;RPS19;ARL2BP;RPS6;RPS20;SEPP1;GOLGA7;TPT1
Streptomycin-120_mg/kg_in_Saline-Rat-Kidney-3d-dn	9/318	0,043817173	0,143673627	0	0	1,945146859	6,083893152	RPS17;RPL32;FUNDC1;RPS6;RPS20;RPS3A;RPL10A;RPL39;TPT1
Cetylpyridinium_Bromide-119_mg/kg_in_Water-Rat-Liver-3d-up	9/319	0,044541473	0,145987781	0	0	1,93904922	6,033030845	RPS17;RPL32;RPS5;RPS6;RPSA;RPS3A;RAPGEF5;RPL39;TPT1
Citalopram-90_mg/kg_in_Corn_Oil-Rat-Heart-5d-up	9/319	0,044541473	0,145927054	0	0	1,93904922	6,033030845	CXCL11;RPS8;FUNDC1;ARL6IP5;RPS20;RAPGEF5;RPL10A;RPL39;TPT1
Dyphylline-3855_mg/kg_in_CMC-Rat-Heart-1d-up	9/319	0,044541473	0,145866377	0	0	1,93904922	6,033030845	C1QA;RPS17;RPL32;RPS19;RPS6;RPS3A;RPL10A;RPL39;PDRG1
Fluphenazine-22_mg/kg_in_Corn_Oil-Rat-Liver-0.25d-up	9/319	0,044541473	0,145805751	0	0	1,93904922	6,033030845	RPL32;RPS19;RPS6;RPS20;RPSA;RPS3A;RPL10A;SEPP1;RPL39
Glyburide-1500_mg/kg_in_CMC-Rat-Heart-1d-dn	9/319	0,044541473	0,145745176	0	0	1,93904922	6,033030845	EEF1B2;RPS17;RPL32;RPS8;RPS6;RPSA;RPS3A;RPL10A;RPL39
Letrozole-250_mg/kg_in_Corn_Oil-Rat-Liver-5d-up	9/319	0,044541473	0,14568465	0	0	1,93904922	6,033030845	C1QA;RPL32;RPS6;RPS20;RPS3A;RAPGEF5;RPL10A;RPL39;TPT1
Lovastatin-1500_mg/kg_in_Corn_Oil-Rat-Kidney-3d-dn	9/319	0,044541473	0,145624175	0	0	1,93904922	6,033030845	EEF1B2;RPS17;RPS19;ARL2BP;FEM1B;RPSA;RPL10A;SEPP1;TPT1
Penicillamine-852_mg/kg_in_Water-Rat-Bone_marrow-3d-up	9/319	0,044541473	0,14556375	0	0	1,93904922	6,033030845	C1QA;RPS17;RPL32;RPS19;NKG7;RPS3A;RPL10A;SEPP1;RPL39
Prednisolone-184_mg/kg_in_Water-Rat-Heart-1d-dn	9/319	0,044541473	0,145503375	0	0	1,93904922	6,033030845	EEF1B2;RPS17;RPL32;RPS19;RPS8;FUNDC1;RPS20;RPS3A;SEPP1
Hydrochlorothiazide-1500_mg/kg_in_CMC-Rat-Kidney-3d-dn	8/270	0,044564801	0,145519225	0	0	2,036400662	6,334857485	EEF1B2;ARL2BP;RPS8;RPS6;RPL10A;RPL39;SEPP1;TPT1
Lead_(II)_Acetate-200_mg/kg_in_Saline-Rat-Spleen-0.25d-dn	8/270	0,044564801	0,145458918	0	0	2,036400662	6,334857485	CLP1;RPS8;FUNDC1;RPS6;RPL35A;RPS20;RPS3A;TPT1
Moxonidine-17_mg/kg_in_CMC-Rat-Heart-5d-up	8/270	0,044564801	0,145398662	0	0	2,036400662	6,334857485	RPL32;RPS19;ARL2BP;RPS6;RPS20;RPSA;RPL10A;RPL39
Nizatidine-1653_mg/kg_in_Corn_Oil-Rat-Heart-3d-dn	8/270	0,044564801	0,145338456	0	0	2,036400662	6,334857485	RPS17;RPL32;RPS19;RPS8;RPS6;RPS3A;SEPP1;PDRG1
Sodium_Selenite-2_mg/kg_in_Water-Rat-Kidney-3d-dn	8/270	0,044564801	0,145278299	0	0	2,036400662	6,334857485	RPS17;RPL32;RPS19;RPS6;RPL35A;RPS20;RPSA;RPL39
Trihexyphenidyl-588_mg/kg_in_CMC-Rat-Heart-3d-up	8/270	0,044564801	0,145218192	0	0	2,036400662	6,334857485	RPS17;RPL32;RPS19;COL5A3;RPS6;RPS3A;RAPGEF5;TPT1
Streptozotocin-3000_uM_in_DMSO-Rat-Primary_rat_hepatocytes-1d-dn	12/473	0,044896274	0,146237821	0	0	1,743641159	5,411216806	RPL32;RPS19;RPS8;RPS5;FUNDC1;RPL35A;RPSA;RPS3A;RPL10A;SEPP1;RPL39;TPT1
Cetylpyridinium_Bromide-16.5_mg/kg_in_Water-Rat-Liver-0.25d-dn	7/223	0,045150185	0,147004076	0	0	2,157397562	6,6831018	RPS17;RPL32;RPS8;RPL35A;RPS3A;RPL39;TPT1
Danazol-95_uM_in_DMSO-Rat-Primary_rat_hepatocytes-1d-dn	7/223	0,045150185	0,146943331	0	0	2,157397562	6,6831018	EEF1B2;CXCL11;RPS8;RPS20;GOLGA7;SEPP1;TOMM20
Cyproheptadine-37_mg/kg_in_CMC-Rat-Heart-1d-dn	6/177	0,045204318	0,147058738	0	0	2,329780418	7,214311088	RPS8;RPS6;ARL6IP5;RPL35A;RPS3A;RPL39
Carboplatin-14_mg/kg_in_Saline-Rat-Liver-3d-dn	9/320	0,045273759	0,147223833	0	0	1,932989691	5,982656618	EEF1B2;RPS17;RPL32;RPS8;RPS5;RPL35A;RPS3A;RPL39;TPT1
Catechol-195_mg/kg_in_Saline-Rat-Spleen-1d-up	9/320	0,045273759	0,147163072	0	0	1,932989691	5,982656618	C1QA;RPS17;RPS19;RPS6;RPS20;RPSA;RPL10A;SEPP1;RPL39
Ceramide_C2-44_uM_in_DMSO-Rat-Primary_rat_hepatocytes-1d-dn	9/320	0,045273759	0,147102362	0	0	1,932989691	5,982656618	EEF1B2;CXCL11;RPL32;RPS19;RPS6;SEPP1;RPL39;SLC16A3;TPT1
Chlorambucil-0.6_mg/kg_in_Corn_Oil-Rat-Bone_marrow-0.25d-up	9/320	0,045273759	0,147041701	0	0	1,932989691	5,982656618	EEF1B2;C1QA;RPL32;RPS19;RPS5;RPS20;RPS3A;SEPP1;RPL39
Clonazepam-2500_mg/kg_in_CMC-Rat-Liver-1d-up	9/320	0,045273759	0,14698109	0	0	1,932989691	5,982656618	RPL32;RPS19;RPS6;RPSA;RPS3A;RPL10A;SEPP1;RPL39;TPT1
Etoposide-100_mg/kg_in_Corn_Oil-Rat-Bone_marrow-1d-up	9/320	0,045273759	0,146920529	0	0	1,932989691	5,982656618	EEF1B2;C1QA;RPS17;RPS19;NKG7;RPS20;RPL10A;SEPP1;RPL39

Piclamilast-17_mg/kg_in_CMC-Rat-Liver-1d-dn	9/320	0,045273759	0,146860018	0	0	1,932989691	5,982656618	EEF1B2;RPS17;RPL32;RPS19;RPS6;RPSA;RPS3A;RPL10A;SEPP1
Aceclofenac-9_mg/kg_in_Corn_Oil-Rat-Kidney-3d-dn	8/271	0,045368649	0,147107236	0	0	2,028886268	6,275211264	EEF1B2;ARL2BP;CLP1;RPS6;RPL13A;SEPP1;TPT1;PDRG1
Hydrochlorothiazide-1500_mg/kg_in_CMC-Rat-Kidney-1d-dn	8/271	0,045368649	0,147046698	0	0	2,028886268	6,275211264	EEF1B2;RPS17;RPS8;RPS6;RPS3A;RPL10A;RPL39;SEPP1
Lovastatin-450_mg/kg_in_Corn_Oil-Rat-Liver-3d-up	8/271	0,045368649	0,14698621	0	0	2,028886268	6,275211264	RPS19;ARL2BP;RPS6;RPS20;RPSA;RPL10A;RPL39;TPT1
Lead(IV)_Acetate-600_mg/kg_in_Saline-Rat-Bone_marrow-1d-up	10/371	0,045772176	0,14823259	0	0	1,852520818	5,713320336	RPS17;RPL32;RPS8;RPS5;FUNDC1;RPL13A;RPL35A;RPS20;RPSA;TPT1
Camptothecin-2.5_mg/kg_in_Saline-Rat-Heart-5d-dn	9/321	0,04601406	0,148954678	0	0	1,926967916	5,932764789	EEF1B2;RPS17;RPL32;ARL2BP;RPS6;RPS20;RPS3A;RAPGEF5;RPL39
Droperidol-94_mg/kg_in_CMC-Rat-Heart-5d-dn	9/321	0,04601406	0,148893481	0	0	1,926967916	5,932764789	EEF1B2;RPS19;RPS8;COL5A3;ARL6IP5;RPS3A;SEPP1;GOLGA7;TPT1
Erythromycin-1500_mg/kg_in_CMC-Rat-Kidney-3d-dn	9/321	0,04601406	0,148832334	0	0	1,926967916	5,932764789	RPS19;ARL2BP;RPS6;ARL6IP5;RPS20;RPSA;RPL10A;RPL39;PDRG1
Leflunomide-30_mg/kg_in_Corn_Oil-Rat-Liver-1d-up	9/321	0,04601406	0,148771237	0	0	1,926967916	5,932764789	EEF1B2;RPS17;RPL32;RPS19;RPL35A;RPSA;RPS3A;RPL39;TPT1
Procaine-2500_mg/kg_in_Water-Rat-Heart-5d-dn	9/321	0,04601406	0,14871019	0	0	1,926967916	5,932764789	EEF1B2;RPS17;RPL32;CLP1;RPS8;RPL35A;RPS20;RPS3A;TOMM20
Sodium_Nitroprusside-6_mg/kg_in_Saline-Rat-Heart-1d-up	9/321	0,04601406	0,148649193	0	0	1,926967916	5,932764789	EEF1B2;RPS17;RPL32;RPS19;RPS6;ARL6IP5;RPL35A;RPL10A;SEPP1
Tegafur-175_mg/kg_in_Water-Rat-Bone_marrow-3d-up	9/321	0,04601406	0,148588246	0	0	1,926967916	5,932764789	C1QA;RPL32;RPS19;FEM1B;RPS6;RPSA;RPS3A;RPL10A;SEPP1
Amineptine-1667_uM_in_DMSO-Rat-Primary_rat_hepatocytes-1d-up	8/272	0,046181974	0,149069356	0	0	2,021427128	6,216223416	EEF1B2;RPS5;RPS6;ARL6IP5;RPL35A;RPS3A;SEPP1;SLC16A3
Azauridine-1500_mg/kg_in_CMC-Rat-Kidney-3d-dn	8/272	0,046181974	0,149008287	0	0	2,021427128	6,216223416	C1QA;RPL32;ARL2BP;RPS8;RPS6;RPL35A;RPS20;GOLGA7
Glycine-2000_mg/kg_in_CMC-Rat-Kidney-3d-dn	8/272	0,046181974	0,148947268	0	0	2,021427128	6,216223416	RPL32;ARL2BP;RPS8;RPS6;RPS20;RPS3A;RPL10A;TPT1
Promethazine-113_mg/kg_in_Saline-Rat-Liver-1d-up	10/372	0,046460351	0,14978376	0	0	1,847540923	5,670391298	C1QA;RPS17;RPL32;RPS19;RPS20;RPSA;RPS3A;RPL10A;RPL39;TPT1
Atorvastatin-2.5_mg/kg_in_Corn_Oil-Rat-Kidney-0.25d-up	9/322	0,046762402	0,150695859	0	0	1,920983544	5,883349762	EEF1B2;RPS17;RPS19;FUNDC1;RPS6;ARL6IP5;RPL35A;RPS3A;RPL39
Catechol-40_mg/kg_in_Saline-Rat-Liver-5d-up	9/322	0,046762402	0,150634224	0	0	1,920983544	5,883349762	EEF1B2;RPS17;RPL32;RPS19;RPS8;RPS6;RPS20;RPS3A;RPL10A
Levamisole-120_mg/kg_in_Water-Rat-Liver-0.25d-up	9/322	0,046762402	0,15057264	0	0	1,920983544	5,883349762	C1QA;RPL32;RPS8;RPS6;RPSA;RPL10A;SEPP1;GOLGA7;TPT1
Pravastatin-1200_mg/kg_in_Corn_Oil-Rat-Kidney-1d-up	9/322	0,046762402	0,150511107	0	0	1,920983544	5,883349762	RPS17;ARL2BP;CLP1;RPS8;ARL6IP5;RPS3A;SEPP1;TOMM20;TPT1
Diphenhydramine-263_uM_in_DMSO-Rat-Primary_rat_hepatocytes-1d-up	7/225	0,046970138	0,151117977	0	0	2,138220695	6,539198977	EEF1B2;RPS19;RPS5;RPS20;RPS3A;RPL10A;PDRG1
Bisphenol_A-610_mg/kg_in_Corn_Oil-Rat-Liver-1d-up	8/273	0,047004809	0,151167772	0	0	2,014022633	6,157885016	EEF1B2;RPL32;RPS19;RPS8;FEM1B;RPL39;SEPP1;TPT1
Droperidol-94_mg/kg_in_CMC-Rat-Heart-1d-dn	8/273	0,047004809	0,151106071	0	0	2,014022633	6,157885016	EEF1B2;RPS19;RPS8;COL5A3;ARL6IP5;RPS20;GOLGA7;SEPP1
Lisinopril-2000_mg/kg_in_CMC-Rat-Kidney-5d-dn	8/273	0,047004809	0,15104442	0	0	2,014022633	6,157885016	EEF1B2;RPS17;RPS19;ARL2BP;CLP1;RPS6;RPS3A;RPL10A
Naloxone-76_mg/kg_in_Saline-Rat-Liver-1d-dn	8/273	0,047004809	0,150982819	0	0	2,014022633	6,157885016	EEF1B2;RPS19;RPS8;RPS6;RPS3A;RPL10A;RPL39;TPT1
Rifabutin-1500_mg/kg_in_CMC-Rat-Kidney-5d-dn	8/273	0,047004809	0,150921269	0	0	2,014022633	6,157885016	EEF1B2;RPS17;RPL32;RPS19;ARL2BP;RPS8;RPL35A;RPS3A
Valacyclovir-88_mg/kg_in_CMC-Rat-Kidney-3d-dn	8/273	0,047004809	0,150859769	0	0	2,014022633	6,157885016	EEF1B2;RPL32;ARL2BP;CLP1;RPS8;FUNDC1;RPL35A;RPL10A
Venlafaxine-1481_uM_in_DMSO-Rat-Primary_rat_hepatocytes-0.67d-dn	8/273	0,047004809	0,150798319	0	0	2,014022633	6,157885016	RPS17;RPL32;RPS8;RPS5;RPS20;RPS3A;SLC16A3;PDRG1
Dideoxycytidine-1200_mg/kg_in_CMC-Rat-Liver-3d-dn	9/323	0,047518813	0,152385249	0	0	1,915036226	5,834406015	RPS17;RPL32;RPS19;RPS8;RPS6;RPL35A;RPS20;RPSA;RPL10A
Etoposide-188_mg/kg_in_Corn_Oil-Rat-Heart-5d-dn	9/323	0,047518813	0,152323228	0	0	1,915036226	5,834406015	EEF1B2;RPS17;RPS19;COL5A3;RPS6;RPSA;RPS3A;RPL10A;RPL39
L-Phenylephrine-0.44_mg/kg_in_Saline-Rat-Heart-3d-up	9/323	0,047518813	0,152261258	0	0	1,915036226	5,834406015	ARL2BP;CLP1;RPS8;FUNDC1;ARL6IP5;RPS20;RAPGEF5;RPL10A;RPL39
Propylene_Glycol-2000_mg/kg_in_Water-Rat-Liver-5d-up	9/323	0,047518813	0,152199338	0	0	1,915036226	5,834406015	C1QA;RPS17;RPS19;RPS6;RPL35A;RPS3A;SEPP1;RPL39;GOLGA7
Valdecoxib-450_uM_in_DMSO-Rat-Primary_rat_hepatocytes-1d-dn	9/323	0,047518813	0,152137468	0	0	1,915036226	5,834406015	RPS17;CXCL11;RPL32;RPS19;ARL6IP5;SEPP1;RPL39;SLC16A3;TPT1
4-Octylphenol-450_mg/kg_in_Corn_Oil-Rat-Liver-1d-up	8/274	0,047837182	0,153094532	0	0	2,006672185	6,100187286	EEF1B2;C1QA;RPS17;RPL32;RPS19;RPS20;RPSA;RPL39
Benoxaprofen-20_mg/kg_in_CMC-Rat-Liver-3d-dn	8/274	0,047837182	0,153032349	0	0	2,006672185	6,100187286	RPS17;RPL32;RPS8;RPS6;RPL35A;RPS20;RPS3A;RPL39
Cisplatin-2_mg/kg_in_Saline-Rat-Bone_marrow-3d-dn	8/274	0,047837182	0,152970217	0	0	2,006672185	6,100187286	RPS17;ARL2BP;FEM1B;RPS6;ARL6IP5;RPSA;RPS3A;RPL10A
N-Nitrosodiethylamine-34_mg/kg_in_Saline-Rat-Bone_marrow-5d-up	8/274	0,047837182	0,152908134	0	0	2,006672185	6,100187286	C1QA;RPS19;CLP1;RPS8;FUNDC1;RPS6;RPS20;RPS3A
NN-Dimethylformamide-140_mg/kg_in_Saline-Rat-Kidney-0.25d-dn	8/274	0,047837182	0,152846103	0	0	2,006672185	6,100187286	RPS17;RPL32;RPS19;ARL2BP;RPS6;RPSA;RPS3A;RPL10A
Nevirapine-200_mg/kg_in_Saline-Rat-Liver-5d-dn	8/274	0,047837182	0,152784121	0	0	2,006672185	6,100187286	EEF1B2;C1QA;RPL32;RPS19;RPS6;RPS3A;SEPP1;TPT1
Sulindac-132_mg/kg_in_Corn_Oil-Rat-Liver-3d-up	8/274	0,047837182	0,15272219	0	0	2,006672185	6,100187286	EEF1B2;RPS17;RPS19;RPS20;RPSA;RPS3A;RPL10A;TPT1
(R)-Bicalutamide-1400_mg/kg_in_CMC-Rat-Kidney-1d-up	9/324	0,048283319	0,154084044	0	0	1,90912562	5,785928108	RPS17;RPL32;RPS19;RPS8;RPS6;RPL35A;RPSA;GOLGA7;TPT1
Ampiroxicam-94_mg/kg_in_CMC-Rat-Kidney-1d-up	9/324	0,048283319	0,154021636	0	0	1,90912562	5,785928108	C1QA;RPS17;RPL32;RPS19;RPS8;RPS3A;RPL10A;SEPP1;TPT1
Daunorubicin-1.5_uM_in_DMSO-Rat-Primary_rat_hepatocytes-1d-up	9/324	0,048283319	0,153959279	0	0	1,90912562	5,785928108	RPS17;RPL32;RPS5;RPS6;ARL6IP5;RPS3A;RPL10A;RPL39;TPT1
Ferrocene-330_mg/kg_in_CMC-Rat-Kidney-1d-dn	9/324	0,048283319	0,153896973	0	0	1,90912562	5,785928108	EEF1B2;RPL32;RPS19;RPS6;RPSA;RPL10A;SEPP1;RPL39;TPT1
Flubendazole-1200_mg/kg_in_Corn_Oil-Rat-Liver-3d-dn	9/324	0,048283319	0,153834717	0	0	1,90912562	5,785928108	EEF1B2;RPS17;RPS19;RPS8;RPL35A;RPSA;RPS3A;SEPP1;TPT1
Sildenafil-300_mg/kg_in_Saline-Rat-Liver-0.25d-dn	9/324	0,048283319	0,153772511	0	0	1,90912562	5,785928108	EEF1B2;C1QA;RPS17;RPL32;RPS19;RPS6;RPS20;RPL39;TPT1
Zidovudine-1473_uM_in_DMSO-Rat-Primary_rat_hepatocytes-0.67d-up	9/324	0,048283319	0,153710355	0	0	1,90912562	5,785928108	EEF1B2;RPL32;CLP1;RPS8;RPS5;RPS6;RPS20;RPSA;TPT1
Beta-Estradiol-150_mg/kg_in_Corn_Oil-Rat-Kidney-3d-up	8/275	0,048679123	0,154907786	0	0	1,999375195	6,043121595	RPS17;RPL32;ARL2BP;RPS8;RPS5;RPS20;RPS3A;TPT1
Alendronic_Acid-138_mg/kg_in_CMC-Rat-Heart-1d-dn	9/325	0,049055945	0,15604387	0	0	1,903251388	5,737910676	RPS17;RPS8;FEM1B;RPS6;RPSA;RPS3A;RPL10A;RPL39;TPT1
Ciprofloxacin-72_mg/kg_in_Corn_Oil-Rat-Liver-3d-up	9/325	0,049055945	0,155980873	0	0	1,903251388	5,737910676	RPS19;RPS8;RPS20;RPSA;RPS3A;SEPP1;RPL39;GOLGA7;TPT1
Dideoxycytidine-1200_mg/kg_in_CMC-Rat-Liver-1d-dn	9/325	0,049055945	0,155917926	0	0	1,903251388	5,737910676	RPS17;RPL32;RPS19;RPS6;ARL6IP5;RPS20;RPSA;RPL10A;RPL39
Lomustine-4.2_mg/kg_in_Corn_Oil-Rat-Liver-5d-up	9/325	0,049055945	0,155855031	0	0	1,903251388	5,737910676	EEF1B2;RPS17;RPS19;RPS6;RPS20;RPS3A;SEPP1;RPL39;TPT1
Tacrine-24_mg/kg_in_Water-Rat-Liver-5d-dn	9/325	0,049055945	0,155792186	0	0	1,903251388	5,737910676	C1QA;RPS17;RPL32;RPS19;RPS8;RPSA;RPS3A;RPL39;TPT1
Oxymetazoline-0.5_mg/kg_in_Water-Rat-Liver-0.25d-up	10/376	0,049282941	0,156449998	0	0	1,827886232	5,502261603	RPS19;ARL2BP;RPS8;RPS6;RPS20;RPSA;RPL10A;SEPP1;GOLGA7;TPT1
Allyl_Alcohol-32_mg/kg_in_Saline-Rat-Liver-5d-dn	8/276	0,049530659	0,157173035	0	0	1,992131082	5,986679455	EEF1B2;C1QA;RPS17;RPL32;CLP1;RPS6;RPS20;SEPP1
Aminocaproic_Acid-2000_mg/kg_in_Water-Rat-Kidney-1d-up	8/276	0,049530659	0,157109736	0	0	1,992131082	5,986679455	EEF1B2;RPS17;CXCL11;RPL32;RPS19;RPS6;ARL6IP5;RPL10A
Bisphenol_A-610_mg/kg_in_Corn_Oil-Rat-Spleen-3d-up	8/276	0,049530659	0,157046487	0	0	1,992131082	5,986679455	EEF1B2;RPS17;RPL32;RPS5;RPS6;RPS20;RPSA;RPS3A
Methotrexate-0.3_mg/kg_in_Corn_Oil-Rat-Bone_marrow-0.25d-dn	8/276	0,049530659	0,156983289	0	0	1,992131082	5,986679455	RPS17;RPL32;RPS19;RPS8;FEM1B;RPS6;ARL6IP5;RPSA
NN-Dimethylformamide-1400_mg/kg_in_Saline-Rat-Kidney-3d-up	8/276	0,049530659	0,156920142	0	0	1,992131082	5,986679455	C1QA;RPS17;ARL2BP;RPL35A;RPS20;RPS3A;RPL39;TPT1
Phenothiazine-386_mg/kg_in_Corn_Oil-Rat-Liver-3d-dn	8/276	0,049530659	0,156857046	0	0	1,992131082	5,986679455	EEF1B2;RPS17;RPS8;RPS6;RPSA;RPS3A;RPL10A;RPL39
Rofecoxib-111_uM_in_DMSO-Rat-Primary_rat_hepatocytes-1d-up	8/276	0,049530659	0,156794001	0	0	1,992131082	5,986679455	EEF1B2;CXADR;RPS6;ARL6IP5;RPS20;RPSA;RPS3A;RPL10A
Azathioprine-20_mg/kg_in_Corn_Oil-Rat-Kidney-3d-dn	9/326	0,049836716	0,157699467	0	0	1,897413193	5,690348431	RPS17;RPL32;RPS19;RPS6;RPL35A;RPS20;RPSA;SEPP1;TPT1
Clofibric_Acid-448_mg/kg_in_Corn_Oil-Rat-Heart-1d-dn	9/326	0,049836716	0,157636134	0	0	1,897413193	5,690348431	RPS17;RPL32;RPS8;FUNDC1;RPS6;RPS20;RPS3A;SEPP1;RPL39
Lamivudine-1300_mg/kg_in_Water-Rat-Liver-1d-up	9/326	0,049836716	0,157572852	0	0	1,897413193	5,690348431	C1QA;RPS17;RPL32;RPS6;RPS20;RPS3A;SEPP1;RPL39;TPT1
N-Nitrosodiethylamine-34_mg/kg_in_Saline-Rat-Bone_marrow-5d-dn	9/326	0,049836716	0,15750962	0	0	1,897413193	5,690348431	EEF1B2;RPS17;RPL32;RPS5;RPL35A;RPSA;RPL10A;RPL39;DUSP14
Netilmicin-41_mg/kg_in_Saline-Rat-Kidney-1d-up	9/326	0,049836716	0,157446439	0	0	1,897413193	5,690348431	RPS17;RPL32;RPS8;RPS5;RPS6;ARL6IP5;RPL35A;RPS20;SEPP1
Valproic_Acid-1340_mg/kg_in_Water-Rat-Liver-0.25d-up	9/326	0,049836716	0,157383309	0	0	1,897413193	5,690348431	EEF1B2;RPS17;RPS19;RPS20;RPSA;RPS3A;RPL10A;SEPP1;RPL39
Bisphenol_A-100_mg/kg_in_Corn_Oil-Rat-Kidney-0.25d-dn	8/277	0,050391819	0,159072533	0	0	1,984939273	5,930852517	RPS17;RPL32;RPS19;ARL2BP;RPS6;ARL6IP5;RPL39;TPT1
Chlormadinone_Acetate-2000_mg/kg_in_CMC-Rat-Kidney-5d-dn	8/277	0,050391819	0,159008802	0	0	1,984939273	5,930852517	EEF1B2;RPS17;RPS8;RPS6;RPSA;RPS3A;RPL10A;TPT1
Diphenidol-300_mg/kg_in_Water-Rat-Heart-5d-up	8/277	0,050391819	0,158945122	0	0	1,984939273	5,930852517	RPS19;RPS6;ARL6IP5;RPS3A;RAPGEF5;RPL10A;SEPP1;PDRG1
Fluphenazine-22_mg/kg_in_Corn_Oil-Rat-Heart-5d-dn	8/277	0,050391819	0,158881493	0	0	1,984939273	5,930852517	RPS17;RPL32;RPS19;ARL2BP;RPS6;RPL35A;RPSA;RPS3A
Ivermectin-7.5_mg/kg_in_CMC-Rat-Liver-1d-dn	8/277	0,050391819	0,158817915	0	0	1,984939273	5,930852517	RPL32;RPS19;ARL2BP;RPS8;RPS6;RPS3A;RPL10A;SEPP1
Miconazole-200_mg/kg_in_Corn_Oil-Rat-Liver-0.25d-up	8/277	0,050391819	0,158754388	0	0	1,984939273	5,930852517	C1QA;RPS17;RPL32;RPS19;RPS6;RPS3A;RPL10A;RPL39
Piclamilast-17_mg/kg_in_CMC-Rat-Liver-3d-up	8/277	0,050391819	0,158690912	0	0	1,984939273	5,930852517	EEF1B2;RPS8;RPS6;RPS20;RPS3A;RPL10A;RPL39;TPT1
17-Methyltestosterone-2000_mg/kg_in_CMC-Rat-Liver-3d-up	9/327	0,050625656	0,159363576	0	0	1,891610707	5,643236159	RPS17;RPS19;FEM1B;RPS6;RPS20;RPSA;RPS3A;RPL10A;RPL39
2-Acetylaminofluorene-30_mg/kg_in_CMC-Rat-Kidney-3d-dn	9/327	0,050625656	0,159299907	0	0	1,891610707	5,643236159	ARL2BP;RPS8;RPS6;RPS20;RPSA;RPL10A;SEPP1;RPL39;TPT1
4-Nonylphenol-20_mg/kg_in_Corn_Oil-Rat-Liver-1d-up	9/327	0,050625656	0,159236289	0	0	1,891610707	5,643236159	RPL32;RPS8;RPS5;RPS6;RPL35A;RPSA;RPL10A;SEPP1;TOMM20
Atropine-94_mg/kg_in_Water-Rat-Liver-0.25d-up	9/327	0,050625656	0,159172721	0	0	1,891610707	5,643236159	EEF1B2;RPL32;RPS19;RPS8;RPS20;RPSA;RPS3A;RPL10A;RPL39
Cerivastatin-7_mg/kg_in_Corn_Oil-Rat-Liver-1d-dn	9/327	0,050625656	0,159109205	0	0	1,891610707	5,643236159	EEF1B2;RPS19;CLP1;RPS8;RPL35A;RPL10A;SEPP1;TPT1;PDRG1
Clarithromycin-56_mg/kg_in_Water-Rat-Liver-1d-up	9/327	0,050625656	0,159045739	0	0	1,891610707	5,643236159	EEF1B2;RPS17;RPL32;RPS8;RPS5;RPS6;RPL35A;RPS20;RPS3A
Colistin-121_mg/kg_in_Water-Rat-Liver-5d-dn	9/327	0,050625656	0,158982323	0	0	1,891610707	5,643236159	EEF1B2;RPS17;RPL32;RPS19;RPS8;RPL35A;RPS20;RPL39;TPT1
Dipyrone-1636_mg/kg_in_Water-Rat-Liver-1d-up	9/327	0,050625656	0,158918958	0	0	1,891610707	5,643236159	RPS17;RPL32;RPS19;RPS6;RPS20;RPSA;RPS3A;RPL10A;RPL39
Mannitol-1000_mg/kg_in_Saline-Rat-Kidney-5d-up	9/327	0,050625656	0,158855644	0	0	1,891610707	5,643236159	RPS17;CXCL11;RPL32;ARL6IP5;RPS20;RPSA;RAPGEF5;SEPP1;TOMM20
Oxymetazoline-0.5_mg/kg_in_Water-Rat-Liver-1d-up	9/327	0,050625656	0,15879238	0	0	1,891610707	5,643236159	EEF1B2;C1QA;RPS17;RPL32;RPS8;RPS5;RPL35A;RPS20;RPS3A
Sparfloxacin-450_mg/kg_in_Corn_Oil-Rat-Liver-0.25d-up	9/327	0,050625656	0,158729167	0	0	1,891610707	5,643236159	EEF1B2;RPS19;RPS20;RPSA;RPS3A;RPL10A;SEPP1;RPL39;TPT1
Tocainide-224_mg/kg_in_Corn_Oil-Rat-Liver-0.25d-up	9/327	0,050625656	0,158666003	0	0	1,891610707	5,643236159	EEF1B2;RPS17;RPL32;RPS19;RPS6;RPS20;RPS3A;RPL10A;SEPP1
Sulindac-23_mg/kg_in_Corn_Oil-Rat-Kidney-0.25d-dn	7/229	0,050749214	0,158989981	0	0	2,100871862	6,26240309	RPS17;RPS8;RPS5;RPL35A;RPS20;RPS3A;TOMM20
Amiodarone-147_mg/kg_in_CMC-Rat-Kidney-1d-up	8/278	0,051262629	0,160534579	0	0	1,977799204	5,875632571	RPS17;RPS19;CLP1;RPS6;RPSA;RPS3A;RPL10A;TPT1
Aspirin-35_mg/kg_in_Corn_Oil-Rat-Kidney-0.25d-dn	8/278	0,051262629	0,160470773	0	0	1,977799204	5,875632571	EEF1B2;RPL32;RPS19;ARL2BP;CLP1;RPS8;RPS6;RPL10A
Aspirin-375_mg/kg_in_Corn_Oil-Rat-Kidney-3d-up	8/278	0,051262629	0,160407018	0	0	1,977799204	5,875632571	RPS17;RPS19;RPL13A;RPL35A;RPSA;RPS3A;RAPGEF5;RPL39

Pioglitazone-1500_mg/kg_in_Corn_Oil-Rat-Heart-5d-dn	8/278	0,051262629	0,160343314	0	0	1,977799204	5,875632571	RPS17;RPL32;RPS19;ARL2BP;COL5A3;RPS6;RPL35A;RPS3A
44'-Methylenedianiline-81_mg/kg_in_Corn_Oil-Rat-Kidney-3d-dn	9/328	0,051422789	0,160780423	0	0	1,885843601	5,596568718	EEF1B2;RPS19;RPS8;RPS6;RPS20;RPSA;RPS3A;SEPP1;RPL39
Acetazolamide-47_mg/kg_in_Corn_Oil-Rat-Liver-3d-up	9/328	0,051422789	0,160716622	0	0	1,885843601	5,596568718	RPS17;RPL32;RPS19;RPS8;RPS5;RPS20;RPSA;RPS3A;RPL10A
Chlorambucil-0.6_mg/kg_in_Corn_Oil-Rat-Liver-5d-dn	9/328	0,051422789	0,16065287	0	0	1,885843601	5,596568718	RPS17;RPL32;RPS8;RPS5;RPL35A;RPS20;RPSA;RPS3A;TPT1
Dexchlorpheniramine-47_mg/kg_in_Water-Rat-Heart-5d-dn	9/328	0,051422789	0,16058917	0	0	1,885843601	5,596568718	RPS17;RPL32;ARL2BP;RPS8;ARL6IP5;RPS20;RPS3A;SEPP1;TPT1
Enoxacin-750_mg/kg_in_Corn_Oil-Rat-Liver-3d-dn	9/328	0,051422789	0,16052552	0	0	1,885843601	5,596568718	EEF1B2;RPS17;RPL32;RPS19;RPS6;RPS3A;RPL10A;RPL39;TPT1
Rosiglitazone-1800_mg/kg_in_Corn_Oil-Rat-Skeletal_muscle-5d-up	7/230	0,051723153	0,161399188	0	0	2,091737636	6,195412635	RPL32;RPS19;RPS8;RPS6;RPS20;RPL10A;SEPP1
Famciclovir-112_mg/kg_in_Saline-Rat-Spleen-0.25d-dn	8/279	0,052143113	0,162645212	0	0	1,970710318	5,821011539	EEF1B2;CXCL11;RPS19;CLP1;RPS8;FUNDC1;RPS6;RPS20
Fluorouracil-29_mg/kg_in_CMC-Rat-Spleen-3d-up	8/279	0,052143113	0,162580824	0	0	1,970710318	5,821011539	RPL32;RPS8;RPS5;RPS6;RPSA;RAPGEF5;RPL10A;SEPP1
Meloxicam-0.6_mg/kg_in_Corn_Oil-Rat-Kidney-1d-dn	8/279	0,052143113	0,162516486	0	0	1,970710318	5,821011539	EEF1B2;RPS17;RPL32;ARL2BP;RPL35A;RPS20;RPS3A;SEPP1
Sulindac-132_mg/kg_in_Corn_Oil-Rat-Liver-0.25d-up	8/279	0,052143113	0,1624522	0	0	1,970710318	5,821011539	EEF1B2;RPS19;RPS6;RPS20;RPSA;RPS3A;RPL10A;RPL39
Colchicine-2000_uM_in_DMSO-Rat-Primary_rat_hepatocytes-0.67d-up	9/329	0,052228138	0,162652753	0	0	1,880111553	5,550341039	EEF1B2;RPS19;RPS6;ARL6IP5;RPL10A;SEPP1;RPL39;GOLGA7;TPT1
Zirconium(IV)_Chloride-633_mg/kg_in_Corn_Oil-Rat-Liver-3d-dn	9/329	0,052228138	0,162588463	0	0	1,880111553	5,550341039	EEF1B2;RPS17;RPS8;RPS20;RPSA;RPS3A;SEPP1;RPL39;TPT1
6-Mercaptopurine-25_mg/kg_in_Corn_Oil-Rat-Liver-3d-dn	8/280	0,053033297	0,16502973	0	0	1,963672067	5,766981479	EEF1B2;C1QA;RPS17;RPL32;RPS8;RPSA;RPS3A;TPT1
Carbon_Tetrachloride-400_mg/kg_in_Corn_Oil-Rat-Liver-0.25d-up	8/280	0,053033297	0,164964552	0	0	1,963672067	5,766981479	RPS19;RPS6;RPS3A;RPL10A;RPL39;GOLGA7;SEPP1;PDRG1
Piroxicam-14_mg/kg_in_CMC-Rat-Kidney-3d-dn	8/280	0,053033297	0,164899426	0	0	1,963672067	5,766981479	EEF1B2;RPL32;RPS19;RPS8;RPS6;RPL10A;SEPP1;TPT1
Acyclovir-330_mg/kg_in_Saline-Rat-Liver-0.25d-dn	9/330	0,053041724	0,164860545	0	0	1,874414246	5,504548122	EEF1B2;RPS17;RPL32;RPS19;RPS5;RPS20;RPL10A;SEPP1;TPT1
Clofibrate-500_mg/kg_in_Corn_Oil-Rat-Kidney-1d-dn	9/330	0,053041724	0,164795511	0	0	1,874414246	5,504548122	EEF1B2;RPS17;RPL32;ARL2BP;RPS8;RPL35A;RPSA;SEPP1;TOMM20
Clotrimazole-52_mg/kg_in_Corn_Oil-Rat-Liver-0.25d-up	9/330	0,053041724	0,164730529	0	0	1,874414246	5,504548122	C1QA;RPS17;RPL32;RPS19;RPS6;RPSA;RPS3A;RPL10A;TPT1
Doxapram-20_mg/kg_in_Saline-Rat-Heart-5d-up	9/330	0,053041724	0,164665598	0	0	1,874414246	5,504548122	RPS8;COL5A3;RPS6;RPS20;RPSA;RPL10A;SEPP1;RPL39;TPT1
Lead_(II)_Acetate-200_mg/kg_in_Saline-Rat-Spleen-0.25d-up	9/330	0,053041724	0,164600718	0	0	1,874414246	5,504548122	EEF1B2;C1QA;RPS17;RPL32;NKG7;RPSA;RPL10A;SEPP1;RPL39
Aspirin-35_mg/kg_in_Corn_Oil-Rat-Heart-3d-up	10/382	0,05372892	0,166667576	0	0	1,799175977	5,260437684	RPS19;RPS8;COL5A3;RPS5;ARL6IP5;RPSA;RPL10A;RPL39;TPT1;PDRG1
2-Amino-4-Nitrophenol-625_mg/kg_in_CMC-Rat-Heart-5d-up	9/331	0,053863571	0,167019481	0	0	1,868751363	5,459185038	EEF1B2;RPL32;ARL2BP;RPS8;ARL6IP5;RPS20;RPSA;RPL10A;RPL39
Cytarabine-487_mg/kg_in_Saline-Rat-Spleen-3d-up	9/331	0,053863571	0,166953751	0	0	1,868751363	5,459185038	EEF1B2;RPS17;RPS19;RPS5;RPL13A;RPL35A;RPS20;RPL10A;SEPP1
Dipyridamole-750_mg/kg_in_CMC-Rat-Liver-3d-dn	9/331	0,053863571	0,166888073	0	0	1,868751363	5,459185038	EEF1B2;RPS17;RPS5;RPSA;RPS3A;RPL10A;SEPP1;GOLGA7;TPT1
Hydroxyurea-400_mg/kg_in_Saline-Rat-Liver-3d-up	9/331	0,053863571	0,166822447	0	0	1,868751363	5,459185038	EEF1B2;RPS17;RPL32;RPS19;RPS6;RPL35A;RPS20;RPS3A;RPL10A
Sparfloxacin-450_mg/kg_in_Corn_Oil-Rat-Liver-3d-up	9/331	0,053863571	0,166756872	0	0	1,868751363	5,459185038	C1QA;RPL32;RPS19;RPL35A;RPS20;RPSA;SEPP1;RPL39;TPT1
Bisphenol_A-100_mg/kg_in_Corn_Oil-Rat-Liver-0.25d-up	8/281	0,053933203	0,166906841	0	0	1,95668391	5,713534574	EEF1B2;RPL32;RPS19;RPS8;RPSA;RPL10A;TPT1;PDRG1
Chlorambucil-0.6_mg/kg_in_Corn_Oil-Rat-Liver-1d-up	8/281	0,053933203	0,166841284	0	0	1,95668391	5,713534574	EEF1B2;RPL32;RPS19;RPS6;RPS20;RPS3A;RPL10A;SEPP1
Glycine-2000_mg/kg_in_CMC-Rat-Kidney-5d-up	8/281	0,053933203	0,166775779	0	0	1,95668391	5,713534574	RPL32;ARL6IP5;RPL13A;RPL35A;RPS20;RPSA;RPL39;TPT1
Penicillamine-852_mg/kg_in_Water-Rat-Bone_marrow-3d-dn	8/281	0,053933203	0,166710326	0	0	1,95668391	5,713534574	ARL2BP;CLP1;RPS8;FUNDC1;RPS6;RPS20;RPSA;TPT1
Promethazine-2.3_mg/kg_in_Saline-Rat-Liver-0.25d-up	8/281	0,053933203	0,166644923	0	0	1,95668391	5,713534574	RPL32;RPS8;RPS6;RPSA;RPS3A;RPL10A;SEPP1;TPT1
Valproic_Acid-235_mg/kg_in_Water-Rat-Liver-5d-dn	8/281	0,053933203	0,166579572	0	0	1,95668391	5,713534574	C1QA;RPS19;RPS8;RPS6;RPS20;RPSA;RPS3A;RPL10A
Anisindione-75_mg/kg_in_CMC-Rat-Liver-1d-up	9/332	0,054693697	0,168862234	0	0	1,863122593	5,414246926	RPL32;RPS19;RPS8;RPS6;RPS20;RPS3A;RPL10A;RPL39;TPT1
Dichlorvos-17_mg/kg_in_Water-Rat-Liver-3d-up	9/332	0,054693697	0,168796066	0	0	1,863122593	5,414246926	RPL32;RPS5;RPS6;RPSA;RPS3A;RAPGEF5;RPL10A;SEPP1;TPT1
Prednisone-68_mg/kg_in_CMC-Rat-Heart-3d-up	9/332	0,054693697	0,168729949	0	0	1,863122593	5,414246926	EEF1B2;C1QA;RPS17;RPL32;ARL6IP5;RPL35A;RPSA;RPS3A;TPT1
Tamoxifen-64_mg/kg_in_Corn_Oil-Rat-Heart-5d-dn	9/332	0,054693697	0,168663884	0	0	1,863122593	5,414246926	EEF1B2;RPS17;RPL32;RPS19;ARL2BP;RPS6;RPS20;RPSA;RPL39
Benzocaine-1100_mg/kg_in_Corn_Oil-Rat-Liver-5d-up	8/282	0,054842855	0,169057662	0	0	1,949745315	5,660663137	RPS17;ARL2BP;RPS6;RPS20;RPS3A;RPL39;SEPP1;TPT1
Cisplatin-1.17_mg/kg_in_Saline-Rat-Bone_marrow-0.25d-up	8/282	0,054842855	0,16899152	0	0	1,949745315	5,660663137	RPL32;RPS19;ARL2BP;RPS8;RPS5;RPL35A;RPS20;RPSA
Fluoxetine-52_mg/kg_in_CMC-Rat-Heart-5d-dn	8/282	0,054842855	0,168925431	0	0	1,949745315	5,660663137	RPS19;ARL2BP;COL5A3;FUNDC1;RPL35A;RPS20;RPS3A;SEPP1
Lead_(II)_Acetate-600_mg/kg_in_Saline-Rat-Kidney-3d-dn	8/282	0,054842855	0,168859393	0	0	1,949745315	5,660663137	EEF1B2;RPS17;RPL32;RPS6;RPS20;RPL39;SEPP1;TPT1
Lorazepam-2000_mg/kg_in_CMC-Rat-Liver-5d-dn	8/282	0,054842855	0,168793406	0	0	1,949745315	5,660663137	EEF1B2;C1QA;RPS17;RPL32;RPS8;RPS6;RPS3A;TPT1
Raloxifene-650_mg/kg_in_Corn_Oil-Rat-Liver-5d-up	8/282	0,054842855	0,168727471	0	0	1,949745315	5,660663137	C1QA;RPS19;RPS8;RPS20;RPSA;RPL10A;RPL39;TPT1
Teicoplanin-41_mg/kg_in_Saline-Rat-Bone_marrow-1d-dn	8/282	0,054842855	0,168661588	0	0	1,949745315	5,660663137	C1QA;RPS19;FUNDC1;RPS6;ARL6IP5;NKG7;RPL39;SEPP1
Celecoxib-400_mg/kg_in_Corn_Oil-Rat-Heart-3d-up	9/333	0,055532125	0,170714681	0	0	1,857527631	5,369728992	RPS19;RPS8;ARL6IP5;RPSA;RPS3A;RPL10A;RPL39;TPT1;PDRG1
Etoposide-125_uM_in_DMSO-Rat-Primary_rat_hepatocytes-1d-dn	9/333	0,055532125	0,170648074	0	0	1,857527631	5,369728992	EEF1B2;RPS17;RPL32;RPS8;RPS5;RPS6;ARL6IP5;RPL10A;SEPP1
Thiamphenicol-1500_mg/kg_in_Water-Rat-Kidney-5d-up	9/333	0,055532125	0,170581519	0	0	1,857527631	5,369728992	EEF1B2;C1QA;RPS17;RPL32;RPS19;RPS6;RPS3A;RPL39;PDRG1
Clofibrate-500_mg/kg_in_Corn_Oil-Rat-Skeletal_muscle-3d-dn	8/283	0,055762274	0,171221702	0	0	1,942855755	5,608359604	EEF1B2;COL5A3;ARL6IP5;RPSA;RPL39;SEPP1;SLC16A3;TPT1
Colchicine-2000_uM_in_DMSO-Rat-Primary_rat_hepatocytes-1d-up	8/283	0,055762274	0,171154975	0	0	1,942855755	5,608359604	EEF1B2;ARL2BP;CLP1;RPS8;RPS20;RPS3A;GOLGA7;PDRG1
Losartan-1000_mg/kg_in_Water-Rat-Heart-5d-up	8/283	0,055762274	0,1710883	0	0	1,942855755	5,608359604	EEF1B2;RPS17;RPL32;ARL2BP;RPS6;RPS3A;RAPGEF5;RPL10A
Neostigmine_Bromide-40_mg/kg_in_Water-Rat-Liver-3d-up	8/283	0,055762274	0,171021677	0	0	1,942855755	5,608359604	RPS17;RPL32;RPS8;RPS5;RPS6;RPSA;RPS3A;TPT1
Nifedipine-1022_mg/kg_in_CMC-Rat-Heart-3d-up	8/283	0,055762274	0,170955106	0	0	1,942855755	5,608359604	C1QA;RPS17;RPL32;RPS19;RPS20;RPS3A;RPL39;SEPP1
Phalloidin-1_uM_in_DMSO-Rat-Primary_rat_hepatocytes-0.67d-up	8/283	0,055762274	0,170888586	0	0	1,942855755	5,608359604	RPS19;ARL2BP;RPS6;ARL6IP5;RPS20;RPSA;RPL10A;PDRG1
Prazosin-2000_mg/kg_in_CMC-Rat-Heart-3d-dn	8/283	0,055762274	0,170822118	0	0	1,942855755	5,608359604	RPS17;ARL2BP;RPS8;COL5A3;ARL6IP5;RPS20;RPS3A;TPT1
Streptozotocin-138_mg/kg_in_Saline-Rat-Liver-1d-dn	8/283	0,055762274	0,170755702	0	0	1,942855755	5,608359604	RPS19;CLP1;RPS8;RPS6;RPS3A;RPL10A;SEPP1;TPT1
Thiabendazole-10_mg/kg_in_Corn_Oil-Rat-Liver-1d-up	8/283	0,055762274	0,170689338	0	0	1,942855755	5,608359604	EEF1B2;RPS17;RPS19;RPS6;RPS20;RPS3A;RPL10A;RPL39
Triacetin-1200_mg/kg_in_CMC-Rat-Liver-5d-dn	8/283	0,055762274	0,170623025	0	0	1,942855755	5,608359604	EEF1B2;C1QA;RPL32;RPS19;RPS6;RPS3A;RPL10A;SEPP1
Stavudine-58_mg/kg_in_Water-Rat-Liver-0.25d-up	10/385	0,056048546	0,171432369	0	0	1,785156424	5,143994407	EEF1B2;C1QA;RPS17;RPL32;RPS6;RPL35A;RPS20;RPSA;RPS3A;RPL10A
Ketamine-30_mg/kg_in_Saline-Rat-Heart-3d-dn	9/334	0,056378872	0,172375776	0	0	1,851966171	5,325626508	EEF1B2;RPS17;RPL32;RPS19;RPS8;RPS6;RPSA;RPS3A;RPL39
Phalloidin-1_uM_in_DMSO-Rat-Primary_rat_hepatocytes-1d-dn	9/334	0,056378872	0,172308886	0	0	1,851966171	5,325626508	RPS17;RPL32;RPS19;RPS6;RPSA;RPL10A;SEPP1;RPL39;TPT1
Tenidap-41_uM_in_DMSO-Rat-Primary_rat_hepatocytes-0.67d-dn	9/334	0,056378872	0,172242048	0	0	1,851966171	5,325626508	EEF1B2;CXCL11;RPL32;RPS19;RPS8;ARL6IP5;RPS20;RPSA;SEPP1
Acarbose-2000_mg/kg_in_Water-Rat-Kidney-5d-up	8/284	0,056691479	0,173129931	0	0	1,936014714	5,556616533	RPS17;CXCL11;RPL32;ARL6IP5;RPSA;RAPGEF5;RPL39;TPT1
Atorvastatin-2.5_mg/kg_in_Corn_Oil-Rat-Liver-0.25d-up	8/284	0,056691479	0,173062826	0	0	1,936014714	5,556616533	EEF1B2;RPS17;RPS19;RPS6;RPS20;RPSA;RPL10A;RPL39
Ketoconazole-90_uM_in_DMSO-Rat-Primary_rat_hepatocytes-1d-up	8/284	0,056691479	0,172995774	0	0	1,936014714	5,556616533	EEF1B2;RPL32;RPS5;RPS20;RPSA;RPS3A;RPL10A;PDRG1
L-Phenylephrine-0.44_mg/kg_in_Saline-Rat-Heart-5d-dn	8/284	0,056691479	0,172928773	0	0	1,936014714	5,556616533	RPS17;RPL32;RPS5;COL5A3;RPS6;RPS3A;SEPP1;TPT1
Mexiletine-350_mg/kg_in_CMC-Rat-Heart-5d-dn	8/284	0,056691479	0,172861824	0	0	1,936014714	5,556616533	C1QA;RPS17;RPS8;COL5A3;R3HCC1;RPS3A;RPL39;TPT1
Nitrazepam-310_mg/kg_in_CMC-Rat-Liver-5d-dn	8/284	0,056691479	0,172794927	0	0	1,936014714	5,556616533	EEF1B2;RPL32;RPS19;RPS6;RPS3A;RPL10A;SEPP1;TPT1
Gentamicin-2900_uM_in_DMSO-Rat-Primary_rat_hepatocytes-0.67d-up	7/235	0,056769148	0,172964723	0	0	2,04723258	5,873023585	EEF1B2;RPS19;CLP1;FEM1B;RPS20;RPS3A;SLC16A3
Prednisolone-184_mg/kg_in_Water-Rat-Kidney-5d-dn	7/235	0,056769148	0,172897838	0	0	2,04723258	5,873023585	RPS17;RPL32;RPS19;CLP1;RPS20;RPSA;RPS3A
Cisapride-250_mg/kg_in_Corn_Oil-Rat-Intestine-0.25d-dn	9/335	0,057233959	0,174246101	0	0	1,846437914	5,281934811	C1QA;RPL32;RPS19;RPSA;RPL10A;SEPP1;RPL39;SLC16A3;TPT1
Mitomycin_C-0.5_mg/kg_in_Saline-Rat-Liver-1d-dn	9/335	0,057233959	0,174178773	0	0	1,846437914	5,281934811	EEF1B2;RPS17;RPL32;RPS19;RPS6;RPS20;RPL10A;SEPP1;RPL39
Neostigmine_Bromide-11_mg/kg_in_Water-Rat-Liver-0.25d-up	9/335	0,057233959	0,174111496	0	0	1,846437914	5,281934811	C1QA;RPS19;RPS8;RPS6;RPS20;RPSA;RPL10A;SEPP1;GOLGA7
Nisoldipine-1125_mg/kg_in_Water-Rat-Liver-5d-up	9/335	0,057233959	0,174044272	0	0	1,846437914	5,281934811	RPS17;RPL32;RPS19;RPS8;RPS6;RPL35A;RPS20;RPS3A;TPT1
Balsalazide-1100_mg/kg_in_Water-Rat-Liver-3d-dn	8/285	0,057630492	0,175182462	0	0	1,92922168	5,505426601	C1QA;RPS17;CLP1;RPS8;RPS3A;RPL39;SEPP1;TPT1
Calcitriol-0.04_mg/kg_in_CMC-Rat-Heart-1d-dn	8/285	0,057630492	0,175114876	0	0	1,92922168	5,505426601	RPS19;RPS6;ARL6IP5;RPS20;RPL10A;GOLGA7;SEPP1;PDRG1
Carbon_Tetrachloride-1175_mg/kg_in_Corn_Oil-Rat-Bone_marrow-1d-dn	8/285	0,057630492	0,175047342	0	0	1,92922168	5,505426601	EEF1B2;RPL32;RPS19;RPS6;ARL6IP5;NKG7;RPS3A;SEPP1
Chlorambucil-0.6_mg/kg_in_Corn_Oil-Rat-Spleen-1d-dn	8/285	0,057630492	0,17497986	0	0	1,92922168	5,505426601	EEF1B2;RPS19;RPS8;RPS5;RPL13A;RPL35A;RPS20;RPSA
Cyclosporin_A-350_mg/kg_in_Corn_Oil-Rat-Bone_marrow-5d-dn	8/285	0,057630492	0,174912431	0	0	1,92922168	5,505426601	RPS17;RPL32;RPS19;RPS8;RPS6;RPS20;RPS3A;TPT1
Flavoxate-1255_mg/kg_in_CMC-Rat-Heart-3d-up	8/285	0,057630492	0,174845053	0	0	1,92922168	5,505426601	RPS17;RPL32;RPS19;RPS6;RPL35A;RPS3A;SEPP1;TPT1
Roxithromycin-312_mg/kg_in_CMC-Rat-Kidney-1d-up	8/285	0,057630492	0,174777727	0	0	1,92922168	5,505426601	EEF1B2;RPS17;CLP1;RPS8;RPSA;RPS3A;RPL39;TPT1
Chlorpromazine-43.9_uM_in_DMSO-Rat-Primary_rat_hepatocytes-1d-up	7/236	0,05781376	0,175266042	0	0	2,038557866	5,810967219	EEF1B2;CXCL11;RPL32;RPS6;RPL35A;RPS3A;SEPP1
Methapyrilene-300_uM_in_DMSO-Rat-Primary_rat_hepatocytes-1d-up	7/236	0,05781376	0,175198606	0	0	2,038557866	5,810967219	EEF1B2;RPS17;RPL32;RPS5;RPS3A;GOLGA7;PDRG1
Stavudine-1120_uM_in_DMSO-Rat-Primary_rat_hepatocytes-1d-up	7/236	0,05781376	0,175131222	0	0	2,038557866	5,810967219	EEF1B2;RPL32;RPS6;ARL6IP5;RPL35A;GOLGA7;PDRG1
24-Diaminophenol-60_mg/kg_in_Water-Rat-Kidney-1d-up	9/336	0,058097404	0,175922781	0	0	1,840942563	5,238649302	CXCL11;CLP1;RPS8;FEM1B;RPS6;RPS3A;RPL10A;SEPP1;TPT1
Indomethacin-4.5_mg/kg_in_Water-Rat-Liver-0.25d-up	9/336	0,058097404	0,17585517	0	0	1,840942563	5,238649302	EEF1B2;RPS17;RPL32;RPS19;RPS20;RPSA;RPL10A;RPL39;TPT1
Indomethacin-4.5_mg/kg_in_Water-Rat-Liver-3d-up	9/336	0,058097404	0,175787611	0	0	1,840942563	5,238649302	EEF1B2;RPS17;RPL32;RPS8;RPS5;RPS6;RPL35A;RPS20;TPT1
Ketorolac-48_mg/kg_in_Water-Rat-Liver-3d-up	9/336	0,058097404	0,175720105	0	0	1,840942563	5,238649302	EEF1B2;RPL32;RPS8;RPS5;RPS6;RPSA;RPS3A;RPL10A;TPT1
Modafinil-325_mg/kg_in_Saline-Rat-Liver-1d-up	9/336	0,058097404	0,17565265	0	0	1,840942563	5,238649302	RPS17;RPS19;ARL6IP5;RPS20;RPSA;RPS3A;RPL10A;SEPP1;RPL39
Sildenafil-2.5_mg/kg_in_Water-Rat-Liver-0.25d-up	9/336	0,058097404	0,175585247	0	0	1,840942563	5,238649302	EEF1B2;C1QA;RPS17;RPS19;RPS6;RPS20;RPL10A;SEPP1;RPL39
Sporidesmin_A-0.15_uM_in_DMSO-Rat-Primary_rat_hepatocytes-1d-dn	10/388	0,058433262	0,176532555	0	0	1,771353668	5,030414135	RPS17;RPL32;RPS19;ARL2BP;RPS8;FUNDC1;RPS20;RPS3A;RPL39;TPT1

Baclofen-19_mg/kg_in_CMC-Rat-Heart-5d-up	8/286	0,058579331	0,176905986	0	0	1,922476149	5,454782605	RPS17;RPS19;ARL2BP;RPS8;RPS6;RPS20;RPSA;RPL10A
Cloxacillin-1200_mg/kg_in_Water-Rat-Kidney-3d-dn	8/286	0,058579331	0,17683818	0	0	1,922476149	5,454782605	EEF1B2;RPL32;RPS19;RPS8;RPS6;RPSA;RPL10A;TPT1
D-Limonene-1650_mg/kg_in_Corn_Oil-Rat-Kidney-1d-dn	8/286	0,058579331	0,176770426	0	0	1,922476149	5,454782605	EEF1B2;RPL32;RPS19;RPS6;RPS20;RPL10A;RPL39;SEPP1
Phenytoin-572_mg/kg_in_Water-Rat-Heart-5d-up	8/286	0,058579331	0,176702724	0	0	1,922476149	5,454782605	RPS17;RPL32;RPS19;RPS6;RPS3A;RPL10A;RPL39;SEPP1
Risperidone-400_uM_in_DMSO-Rat-Primary_rat_hepatocytes-0.67d-up	8/286	0,058579331	0,176635073	0	0	1,922476149	5,454782605	EEF1B2;FEM1B;RPS6;RPS20;RPS3A;RPL10A;RPL39;PDRG1
Tosufloxacin-2000_mg/kg_in_CMC-Rat-Liver-3d-up	8/286	0,058579331	0,176567475	0	0	1,922476149	5,454782605	RPS17;RPS19;RPS6;RPS20;RPSA;RPS3A;RPL10A;RPL39
Valsartan-30_mg/kg_in_Corn_Oil-Rat-Heart-1d-dn	8/286	0,058579331	0,176499928	0	0	1,922476149	5,454782605	ARL2BP;RPS8;FUNDC1;ARL6IP5;RPS20;RPSA;SEPP1;TPT1
Ethylene_Glycol-3525_mg/kg_in_Water-Rat-Liver-0.25d-dn	7/237	0,058870221	0,177308552	0	0	2,029956356	5,749688767	RPS17;RPL32;RPS8;RPS5;RPL35A;RPL39;TPT1
Cromolyn-1500_mg/kg_in_Water-Rat-Kidney-1d-up	9/337	0,058969223	0,177538839	0	0	1,835479825	5,195765443	EEF1B2;RPS17;RPL32;RPS19;RPS8;RPS5;RPS6;RPSA;RPL10A
Digoxin-11_mg/kg_in_Corn_Oil-Rat-Liver-5d-dn	9/337	0,058969223	0,177470998	0	0	1,835479825	5,195765443	EEF1B2;RPL32;RPS19;RPS8;RPS6;RPSA;RPL10A;SEPP1;GOLGA7
Penicillamine-852_mg/kg_in_Water-Rat-Liver-0.25d-up	9/337	0,058969223	0,17740321	0	0	1,835479825	5,195765443	EEF1B2;RPL32;RPS19;RPS6;RPS20;RPS3A;RPL10A;SEPP1;TPT1
Primaquine-45_mg/kg_in_CMC-Rat-Liver-1d-up	9/337	0,058969223	0,177335473	0	0	1,835479825	5,195765443	C1QA;RPS19;RPS8;RPS6;RPS20;RPL10A;SEPP1;RPL39;TPT1
Vinblastine-0.08_mg/kg_in_Saline-Rat-Bone_marrow-1d-up	9/337	0,058969223	0,177267787	0	0	1,835479825	5,195765443	C1QA;RPL32;RPS19;FEM1B;RPL35A;RPL10A;SEPP1;RPL39;TPT1
2-Acetylaminofluorene-30_mg/kg_in_CMC-Rat-Liver-3d-dn	8/287	0,059538013	0,178909345	0	0	1,915777626	5,404677453	EEF1B2;RPS17;CLP1;RPS8;ARL6IP5;RPS20;RPS3A;RPL10A
Bisphenol_A-610_mg/kg_in_Corn_Oil-Rat-Bone_marrow-1d-dn	8/287	0,059538013	0,178841111	0	0	1,915777626	5,404677453	EEF1B2;RPL32;RPS8;RPS5;ARL6IP5;RPL35A;RPSA;TPT1
Calcitriol-0.04_mg/kg_in_CMC-Rat-Kidney-1d-up	8/287	0,059538013	0,178772929	0	0	1,915777626	5,404677453	EEF1B2;RPS17;RPL32;RPS19;RPS6;RPS20;RPS3A;RPL39
Chlorambucil-0.6_mg/kg_in_Corn_Oil-Rat-Spleen-3d-up	8/287	0,059538013	0,178704799	0	0	1,915777626	5,404677453	EEF1B2;RPL32;RPS5;RPS6;RPS20;RPSA;RPS3A;RPL39
Chlorambucil-4.5_mg/kg_in_Corn_Oil-Rat-Liver-1d-up	8/287	0,059538013	0,178636721	0	0	1,915777626	5,404677453	EEF1B2;RPS19;RPS8;FEM1B;RPS20;RPS3A;RPL39;TPT1
Gemfibrozil-700_mg/kg_in_Corn_Oil-Rat-Liver-0.25d-up	8/287	0,059538013	0,178568695	0	0	1,915777626	5,404677453	EEF1B2;RPL32;RPS19;RPS6;RPS20;RPS3A;RPL10A;SEPP1
Ifosfamide-17_mg/kg_in_Saline-Rat-Liver-5d-up	8/287	0,059538013	0,17850072	0	0	1,915777626	5,404677453	C1QA;RPS17;RPS8;FUNDC1;RPS6;RPS20;RPS3A;RPL39
Zomepirac-11_mg/kg_in_Saline-Rat-Kidney-5d-dn	8/287	0,059538013	0,178432798	0	0	1,915777626	5,404677453	ARL2BP;RPS8;R3HCC1;RPS6;RPSA;RPL10A;SEPP1;TPT1
Methyl_Salicylate-444_mg/kg_in_Corn_Oil-Rat-Liver-5d-up	9/338	0,059849435	0,179297888	0	0	1,830049411	5,153278762	C1QA;RPL32;RPS8;RPS20;RPSA;RPS3A;RAPGEF5;RPL39;TPT1
Nortriptyline-63_mg/kg_in_Water-Rat-Heart-3d-dn	9/338	0,059849435	0,179229714	0	0	1,830049411	5,153278762	EEF1B2;RPS17;FUNDC1;RPL35A;RPS20;RPS3A;SEPP1;RPL39;TPT1
Sulfadoxine-380_mg/kg_in_Corn_Oil-Rat-Liver-3d-dn	9/338	0,059849435	0,179161592	0	0	1,830049411	5,153278762	EEF1B2;RPS17;RPL32;RPS19;RPS8;RPS6;RPS3A;RPL10A;TPT1
5-Fluoro-2'-Deoxyuridine-54_mg/kg_in_Saline-Rat-Bone_marrow-3d-dn	8/288	0,060506556	0,18105989	0	0	1,90912562	5,35510417	RPS17;RPL32;RPS8;FEM1B;NKG7;RPL13A;RPS3A;TPT1
Bithionol-59_mg/kg_in_Corn_Oil-Rat-Bone_marrow-3d-dn	8/288	0,060506556	0,180991125	0	0	1,90912562	5,35510417	RPS19;FEM1B;ARL6IP5;NKG7;RPSA;RPL10A;SEPP1;PDRG1
Cisplatin-1.17_mg/kg_in_Saline-Rat-Spleen-1d-up	8/288	0,060506556	0,180922411	0	0	1,90912562	5,35510417	RPS17;RPS19;RPS6;RPS20;RPS3A;RPL10A;RPL39;SEPP1
Indinavir-1000_mg/kg_in_CMC-Rat-Kidney-3d-dn	8/288	0,060506556	0,18085375	0	0	1,90912562	5,35510417	RPL32;RPS19;CLP1;RPS8;RPS6;RPL10A;RPL39;TPT1
L-Phenylephrine-0.44_mg/kg_in_Saline-Rat-Heart-1d-dn	8/288	0,060506556	0,180785141	0	0	1,90912562	5,35510417	EEF1B2;RPS17;RPL32;COL5A3;RPS6;RPS3A;SEPP1;TPT1
Mefenamic_Acid-93_mg/kg_in_Corn_Oil-Rat-Kidney-1d-up	8/288	0,060506556	0,180716584	0	0	1,90912562	5,35510417	EEF1B2;RPS17;CLP1;RPS8;RPS20;RPS3A;TOMM20;TPT1
Nortriptyline-70_uM_in_DMSO-Rat-Primary_rat_hepatocytes-0.67d-dn	8/288	0,060506556	0,180648079	0	0	1,90912562	5,35510417	RPS17;RPL32;RPS8;RPS5;RPL35A;RPS3A;TPT1;PDRG1
Progesterone-164_mg/kg_in_Corn_Oil-Rat-Liver-5d-dn	8/288	0,060506556	0,180579625	0	0	1,90912562	5,35510417	EEF1B2;C1QA;RPL32;RPS6;RPL35A;RPS3A;RPL39;TPT1
Rofecoxib-775_mg/kg_in_Corn_Oil-Rat-Heart-5d-dn	8/288	0,060506556	0,180511224	0	0	1,90912562	5,35510417	RPS17;RPS8;FUNDC1;RPS6;RPL35A;RPSA;RPS3A;SEPP1
Rofecoxib-800_mg/kg_in_Corn_Oil-Rat-Liver-1d-up	8/288	0,060506556	0,180442874	0	0	1,90912562	5,35510417	C1QA;RPS17;RPS8;RPS6;NKG7;RPSA;SEPP1;TPT1
Temafloxacin-1000_mg/kg_in_CMC-Rat-Liver-5d-up	8/288	0,060506556	0,180374577	0	0	1,90912562	5,35510417	EEF1B2;RPS6;RPS20;RPSA;RPS3A;RPL10A;RPL39;TPT1
Cyclosporin_A-8.5_uM_in_DMSO-Rat-Primary_rat_hepatocytes-1d-dn	9/339	0,060738054	0,180996184	0	0	1,824651035	5,111184843	CXCL11;RPS19;RPS8;ARL6IP5;RPSA;RPL10A;SEPP1;RPL39;TPT1
Meloxicam-6_mg/kg_in_Corn_Oil-Rat-Liver-0.25d-up	9/339	0,060738054	0,180927729	0	0	1,824651035	5,111184843	EEF1B2;RPS17;RPL32;RPS8;RPS6;ARL6IP5;RPS20;RPL10A;SEPP1
Sertraline-23_uM_in_DMSO-Rat-Primary_rat_hepatocytes-1d-dn	9/339	0,060738054	0,180859325	0	0	1,824651035	5,111184843	C1QA;CXCL11;RPL32;RPS19;RPS8;SEPP1;RPL39;SLC16A3;TPT1
Sotalol-2000_mg/kg_in_Water-Rat-Liver-5d-dn	9/339	0,060738054	0,180790973	0	0	1,824651035	5,111184843	EEF1B2;RPS17;RPL32;RPS19;RPL35A;RPS20;RPSA;RPL39;TPT1
Benzoic_Acid-1700_mg/kg_in_CMC-Rat-Liver-1d-dn	8/289	0,061484974	0,18294509	0	0	1,902519649	5,306055888	EEF1B2;C1QA;RPS17;RPL32;CLP1;RPS8;RPL35A;SEPP1
Cortisone-206_mg/kg_in_CMC-Rat-Kidney-1d-up	8/289	0,061484974	0,182876002	0	0	1,902519649	5,306055888	EEF1B2;ARL2BP;CLP1;RPS8;RPS20;RPSA;RPS3A;SEPP1
Cyclosporin_A-350_mg/kg_in_Corn_Oil-Rat-Liver-3d-up	8/289	0,061484974	0,182806966	0	0	1,902519649	5,306055888	RPL32;ARL2BP;RPS8;RPS5;RPS20;RPSA;SEPP1;TPT1
Dexfenfluramine-29_mg/kg_in_Water-Rat-Heart-3d-dn	8/289	0,061484974	0,182737982	0	0	1,902519649	5,306055888	RPL32;RPS8;COL5A3;RPS6;RPS20;RPSA;RPL39;TPT1
Gliclazide-1500_mg/kg_in_CMC-Rat-Kidney-1d-dn	8/289	0,061484974	0,182669051	0	0	1,902519649	5,306055888	EEF1B2;RPS19;ARL2BP;CLP1;RPS8;RPS6;RPS3A;SEPP1
Mefenamic_Acid-93_mg/kg_in_Corn_Oil-Rat-Liver-1d-up	8/289	0,061484974	0,182600171	0	0	1,902519649	5,306055888	C1QA;RPS17;CLP1;RPS8;RPS5;RPS6;RPS3A;RPL39
Procarbazine-27_mg/kg_in_Water-Rat-Liver-5d-up	8/289	0,061484974	0,182531343	0	0	1,902519649	5,306055888	RPS17;RPS8;RPS6;RPL35A;RPS20;RPS3A;RPL39;GOLGA7
Propranolol-175_mg/kg_in_Water-Rat-Heart-5d-dn	8/289	0,061484974	0,182462567	0	0	1,902519649	5,306055888	EEF1B2;RPS19;ARL2BP;RPS8;RPS20;GOLGA7;SEPP1;TPT1
Salicylamide-1300_mg/kg_in_CMC-Rat-Liver-1d-up	8/289	0,061484974	0,182393843	0	0	1,902519649	5,306055888	CXCL11;RPS19;RPS6;RPSA;RPS3A;RPL10A;RPL39;TPT1
Ibufenac-500_mg/kg_in_Corn_Oil-Rat-Liver-1d-dn	9/340	0,061635097	0,182770342	0	0	1,819284415	5,069479334	C1QA;RPS17;RPL32;RPS19;CLP1;RPS20;RPSA;RPL10A;RPL39
Ibufenac-500_mg/kg_in_Corn_Oil-Rat-Liver-3d-dn	9/340	0,061635097	0,182701553	0	0	1,819284415	5,069479334	RPS17;RPL32;RPS19;RPS6;RPS20;RPSA;RPL10A;SEPP1;RPL39
Modafinil-17.5_mg/kg_in_Saline-Rat-Liver-5d-up	9/340	0,061635097	0,182632817	0	0	1,819284415	5,069479334	EEF1B2;RPS17;RPL32;RPS19;RPS8;RPS6;RPL35A;RPS20;RPS3A
Rofecoxib-775_mg/kg_in_Corn_Oil-Rat-Kidney-3d-up	9/340	0,061635097	0,182564132	0	0	1,819284415	5,069479334	C1QA;RPS17;RPL32;RPS8;RPL35A;RPS20;RPSA;RPS3A;TPT1
Auranofin-33_mg/kg_in_CMC-Rat-Kidney-3d-up	8/290	0,062473283	0,184977284	0	0	1,895959237	5,25752585	RPS17;RPL32;RPS8;RPS20;RPSA;RPS3A;RAPGEF5;SEPP1
Dexamethasone-1_mg/kg_in_Corn_Oil-Rat-Liver-3d-up	8/290	0,062473283	0,184907769	0	0	1,895959237	5,25752585	EEF1B2;RPL32;RPS19;RPS8;ARL6IP5;RPS20;RPSA;RPL39
Diisopropyl_Fluorophosphate-3.75_mg/kg_in_Water-Rat-Heart-3d-dn	8/290	0,062473283	0,184838307	0	0	1,895959237	5,25752585	RPL32;RPS8;COL5A3;RPS6;RPS20;RPL10A;RPL39;TPT1
Doxofylline-483_mg/kg_in_Water-Rat-Heart-3d-up	8/290	0,062473283	0,184768898	0	0	1,895959237	5,25752585	RPS19;ARL2BP;RPS6;RPSA;RAPGEF5;RPL10A;GOLGA7;SEPP1
Enrofloxacin-2000_mg/kg_in_CMC-Rat-Kidney-3d-up	8/290	0,062473283	0,18469954	0	0	1,895959237	5,25752585	EEF1B2;RPS17;RPS6;RPL13A;RPL35A;RPS3A;RPL39;TPT1
Famciclovir-1200_mg/kg_in_Saline-Rat-Bone_marrow-3d-dn	8/290	0,062473283	0,184630234	0	0	1,895959237	5,25752585	EEF1B2;C1QA;RPL32;RPS19;NKG7;RPS20;RPS3A;RPL39
Gemfibrozil-700_mg/kg_in_Corn_Oil-Rat-Skeletal_muscle-3d-dn	8/290	0,062473283	0,184560981	0	0	1,895959237	5,25752585	FEM1B;COL5A3;ARL6IP5;RPSA;RPL10A;RPL39;SLC16A3;TPT1
Methimazole-100_mg/kg_in_Water-Rat-Liver-3d-up	8/290	0,062473283	0,184491779	0	0	1,895959237	5,25752585	EEF1B2;RPS19;RPS6;RPS20;RPSA;RPL10A;RPL39;SEPP1
Nizatidine-1653_mg/kg_in_Corn_Oil-Rat-Heart-1d-dn	8/290	0,062473283	0,184422629	0	0	1,895959237	5,25752585	EEF1B2;RPS17;RPL32;RPS8;RPL35A;RPS20;RPS3A;TOMM20
Secobarbital-70_mg/kg_in_Water-Rat-Liver-3d-up	8/290	0,062473283	0,184353531	0	0	1,895959237	5,25752585	RPS17;RPL32;RPS19;RPS8;RPS6;RPSA;RPL39;TPT1
Sparteine-225_mg/kg_in_Water-Rat-Heart-3d-dn	8/290	0,062473283	0,184284485	0	0	1,895959237	5,25752585	RPS19;ARL2BP;RPS8;FUNDC1;ARL6IP5;RPSA;RPL10A;TPT1
Cisapride-250_mg/kg_in_Corn_Oil-Rat-Intestine-1d-dn	9/341	0,062540579	0,184413927	0	0	1,81394927	5,028157938	EEF1B2;C1QA;RPS17;RPS19;RPL35A;RPS20;RPSA;RPL10A;RPL39
Dexibuprofen-239_mg/kg_in_CMC-Rat-Kidney-5d-up	9/341	0,062540579	0,18434491	0	0	1,81394927	5,028157938	C1QA;RPL32;RPS19;RPS8;RPS5;RPSA;SEPP1;RPL39;TPT1
Fluphenazine-2.5_mg/kg_in_Corn_Oil-Rat-Liver-1d-up	9/341	0,062540579	0,184275945	0	0	1,81394927	5,028157938	EEF1B2;RPL32;RPS19;RPS8;FEM1B;RPS20;RPSA;SEPP1;TPT1
Valproic_Acid-1340_mg/kg_in_Water-Rat-Liver-3d-up	9/341	0,062540579	0,184207031	0	0	1,81394927	5,028157938	RPS17;RPS19;RPS8;FEM1B;RPS6;RPS20;RPSA;RPS3A;RPL10A
Piroxicam-14_mg/kg_in_CMC-Rat-Kidney-5d-up	9/342	0,063454513	0,186829064	0	0	1,808645325	4,98721642	RPL32;RPS19;ARL2BP;ARL6IP5;RPL13A;RPS20;RPSA;SEPP1;RPL39
Acarbose-2000_mg/kg_in_Water-Rat-Kidney-3d-dn	8/291	0,063471496	0,186809232	0	0	1,889443913	5,209507405	RPL32;RPS19;RPS8;FEM1B;RPS6;RPSA;RPS3A;RPL10A
Amiodarone-37.04_uM_in_DMSO-Rat-Primary_rat_hepatocytes-0.67d-dn	8/291	0,063471496	0,186739448	0	0	1,889443913	5,209507405	EEF1B2;RPS17;CXCL11;RPL32;ARL6IP5;RPL10A;SEPP1;TPT1
Carvedilol-2000_mg/kg_in_Corn_Oil-Rat-Heart-1d-dn	8/291	0,063471496	0,186669718	0	0	1,889443913	5,209507405	EEF1B2;RPS19;ARL2BP;RPS8;FUNDC1;RPS6;ARL6IP5;RPS20
Cisplatin-1.17_mg/kg_in_Saline-Rat-Liver-0.25d-up	8/291	0,063471496	0,186600039	0	0	1,889443913	5,209507405	EEF1B2;C1QA;RPS17;RPL32;RPS6;RPS3A;RPL39;SEPP1
Clofibrate-500_uM_in_DMSO-Rat-Primary_rat_hepatocytes-0.67d-dn	8/291	0,063471496	0,186530412	0	0	1,889443913	5,209507405	EEF1B2;ARL2BP;ARL6IP5;RPS20;RPSA;RPL10A;SEPP1;SLC16A3
Epinephrine-0.0375_mg/kg_in_Saline-Rat-Heart-5d-up	8/291	0,063471496	0,186460837	0	0	1,889443913	5,209507405	EEF1B2;RPS17;RPL32;RPS19;RPS6;RPSA;RPS3A;RPL39
Gentamicin-656.6_uM_in_DMSO-Rat-Primary_rat_hepatocytes-0.67d-dn	8/291	0,063471496	0,186391314	0	0	1,889443913	5,209507405	RPS17;RPS8;RPS5;RPS6;ARL6IP5;RPS20;RPL39;SLC16A3
Oxaprozin-1500_mg/kg_in_CMC-Rat-Kidney-1d-up	8/291	0,063471496	0,186321843	0	0	1,889443913	5,209507405	RPS17;RPS19;ARL2BP;RPS6;RPS20;RPL10A;RPL39;TPT1
Pergolide-1.1_mg/kg_in_CMC-Rat-Liver-3d-dn	8/291	0,063471496	0,186252423	0	0	1,889443913	5,209507405	EEF1B2;RPS19;CLP1;FEM1B;RPS6;RPS20;SEPP1;TPT1
Pramoxine-526_mg/kg_in_Corn_Oil-Rat-Heart-5d-up	8/291	0,063471496	0,186183055	0	0	1,889443913	5,209507405	EEF1B2;RPS17;RPS19;CLP1;RPS8;RPS20;RAPGEF5;TPT1
Praziquantel-1200_mg/kg_in_CMC-Rat-Liver-1d-dn	8/291	0,063471496	0,186113739	0	0	1,889443913	5,209507405	EEF1B2;C1QA;RPS17;RPL32;RPSA;RPL39;SEPP1;TPT1
Doxepin-147_mg/kg_in_CMC-Rat-Heart-3d-dn	9/343	0,064376913	0,188698388	0	0	1,803372306	4,946650597	EEF1B2;RPS19;RPS8;COL5A3;RPS6;RPS20;RPSA;RPL10A;RPL39
Tretinoin-156_uM_in_DMSO-Rat-Primary_rat_hepatocytes-1d-dn	9/343	0,064376913	0,188628188	0	0	1,803372306	4,946650597	RPS19;RPS8;RPS6;ARL6IP5;RPSA;RPS3A;SEPP1;RPL39;SLC16A3
Azathioprine-20_mg/kg_in_Corn_Oil-Rat-Bone_marrow-1d-dn	8/292	0,064479627	0,188858885	0	0	1,882973215	5,161994006	EEF1B2;RPL32;RPS19;RPS8;RPS6;RPS20;RPSA;RPL10A
Ethanol-3000_mg/kg_in_Saline-Rat-Bone_marrow-1d-dn	8/292	0,064479627	0,188788677	0	0	1,882973215	5,161994006	EEF1B2;RPS17;RPL32;RPSA;RPS3A;RPL10A;RPL39;TPT1
Gatifloxacin-770_mg/kg_in_Corn_Oil-Rat-Kidney-1d-up	8/292	0,064479627	0,188718522	0	0	1,882973215	5,161994006	RPS17;RPS19;RPS6;ARL6IP5;RPS20;RPSA;RPS3A;RAPGEF5
Leflunomide-60_mg/kg_in_Corn_Oil-Rat-Bone_marrow-1d-dn	8/292	0,064479627	0,188648418	0	0	1,882973215	5,161994006	EEF1B2;RPL32;RPS8;FEM1B;RPS6;ARL6IP5;RPSA;RPS3A
Lomustine-8.75_mg/kg_in_Corn_Oil-Rat-Bone_marrow-1d-dn	8/292	0,064479627	0,188578367	0	0	1,882973215	5,161994006	RPL32;RPS8;FEM1B;RPS5;RPS6;ARL6IP5;RPL35A;RPSA
Lovastatin-1500_mg/kg_in_Corn_Oil-Rat-Liver-5d-up	8/292	0,064479627	0,188508367	0	0	1,882973215	5,161994006	CXADR;RPS8;RPS6;RPS20;RPSA;RPL10A;RPL39;TPT1
Methotrexate-0.3_mg/kg_in_Corn_Oil-Rat-Spleen-1d-up	8/292	0,064479627	0,18843842	0	0	1,882973215	5,161994006	RPS17;ARL2BP;RPS8;RPS5;ARL6IP5;RPS20;RPS3A;TPT1
Moxonidine-17_mg/kg_in_CMC-Rat-Heart-1d-dn	8/292	0,064479627	0,188368524	0	0	1,882973215	5,161994006	RPS17;ARL2BP;RPS8;COL5A3;ARL6IP5;RPSA;SEPP1;TPT1
Nisoldipine-15_mg/kg_in_Water-Rat-Liver-1d-up	8/292	0,064479627	0,188298681	0	0	1,882973215	5,161994006	RPS17;RPS8;RPL35A;RPS20;RPSA;RPS3A;RPL39;TPT1

Omeprazole-30_mg/kg_in_Corn_Oil-Rat-Liver-0.25d-dn	8/292	0,064479627	0,188228889	0	0	1,882973215	5,161994006	C1QA;RPS17;RPL32;RPS8;RPS5;RPL35A;RPSA;TPT1
Procarbazine-27_mg/kg_in_Water-Rat-Liver-0.25d-dn	8/292	0,064479627	0,188159148	0	0	1,882973215	5,161994006	RPS17;RPL32;RPL35A;RPSA;RPS3A;RPL10A;RPL39;TPT1
Thiabendazole-92_mg/kg_in_Corn_Oil-Rat-Liver-1d-dn	8/292	0,064479627	0,18808946	0	0	1,882973215	5,161994006	EEF1B2;C1QA;RPS17;RPL32;RPS8;FEM1B;RPS20;RPS3A
Azathioprine-54_mg/kg_in_Water-Rat-Kidney-5d-up	9/344	0,065307792	0,190434717	0	0	1,798129945	4,906456347	EEF1B2;RPS17;RPS8;RPS6;RPL13A;RPL35A;RPS20;RPS3A;RPL39
Betahistine-1500_mg/kg_in_Water-Rat-Kidney-3d-up	9/344	0,065307792	0,190364238	0	0	1,798129945	4,906456347	EEF1B2;RPS17;RPL32;RPS19;ARL6IP5;RPS20;RPSA;RPS3A;SEPP1
Cromolyn-1500_mg/kg_in_Water-Rat-Kidney-3d-up	9/344	0,065307792	0,190293811	0	0	1,798129945	4,906456347	EEF1B2;CXCL11;RPL32;RPS19;RPS8;RPS5;RPS20;RPS3A;TPT1
Cyclosporin_A-70_mg/kg_in_Corn_Oil-Rat-Kidney-0.25d-up	9/344	0,065307792	0,190223436	0	0	1,798129945	4,906456347	RPL32;RPS19;FUNDC1;RPS6;RPS3A;RPL10A;SEPP1;RPL39;PDRG1
Aldosterone-0.065_mg/kg_in_Saline-Rat-Kidney-5d-dn	8/293	0,065497687	0,190706019	0	0	1,876546685	5,114979209	EEF1B2;RPS17;RPL32;RPS6;RPS20;RAPGEF5;SEPP1;TPT1
Aminoglutethimide-350_mg/kg_in_CMC-Rat-Liver-1d-dn	8/293	0,065497687	0,190635544	0	0	1,876546685	5,114979209	EEF1B2;RPS17;RPL32;CLP1;RPS8;RPS6;SEPP1;RPL39
Azauridine-1500_mg/kg_in_CMC-Rat-Kidney-1d-dn	8/293	0,065497687	0,190565121	0	0	1,876546685	5,114979209	RPS17;RPL32;RPS5;RPS6;RPS20;RPSA;RPL10A;GOLGA7
Bisphenol_A-100_mg/kg_in_Corn_Oil-Rat-Liver-5d-up	8/293	0,065497687	0,19049475	0	0	1,876546685	5,114979209	RPS17;RPL32;RPL35A;RPS20;RPSA;SEPP1;RPL39;TPT1
Chloroform-600_mg/kg_in_Water-Rat-Liver-3d-dn	8/293	0,065497687	0,19042443	0	0	1,876546685	5,114979209	C1QA;RPS17;RPL32;RPL35A;RPS20;RPS3A;RPL39;TPT1
Chloroxylenol-1915_mg/kg_in_Corn_Oil-Rat-Liver-5d-dn	8/293	0,065497687	0,190354163	0	0	1,876546685	5,114979209	RPS17;RPL32;RPS19;RPS8;RPS6;RPS3A;RPL10A;TPT1
Dicumarol-13_mg/kg_in_Corn_Oil-Rat-Liver-3d-up	8/293	0,065497687	0,190283948	0	0	1,876546685	5,114979209	EEF1B2;RPL32;RPS8;RPS5;RPL35A;RPS20;SEPP1;TPT1
Digitonin-75_mg/kg_in_Corn_Oil-Rat-Heart-5d-dn	8/293	0,065497687	0,190213784	0	0	1,876546685	5,114979209	EEF1B2;RPS19;ARL2BP;RPS8;FUNDC1;RPS6;RPSA;RPL10A
Lovastatin-1500_mg/kg_in_Corn_Oil-Rat-Kidney-1d-up	8/293	0,065497687	0,190143672	0	0	1,876546685	5,114979209	RPS17;RPL32;RPS8;RPS5;RPS6;RPS20;RPS3A;RPL39
Methyl_Methanesulfonate-113_mg/kg_in_Water-Rat-Bone_marrow-3d-dn	8/293	0,065497687	0,190073612	0	0	1,876546685	5,114979209	EEF1B2;ARL2BP;RPS8;FUNDC1;RPS20;RPS3A;RPL39;TPT1
Oxytetracycline-1500_mg/kg_in_Water-Rat-Liver-1d-dn	8/293	0,065497687	0,190003603	0	0	1,876546685	5,114979209	EEF1B2;RPS19;CLP1;RPS8;RPSA;RPL10A;SEPP1;TPT1
Procarbazine-54_mg/kg_in_Water-Rat-Bone_marrow-1d-dn	8/293	0,065497687	0,189933646	0	0	1,876546685	5,114979209	RPL32;RPS19;FEM1B;RPS6;ARL6IP5;RPS3A;RPL39;DUSP14
Sulfadiazine-1170_mg/kg_in_Water-Rat-Liver-5d-up	8/293	0,065497687	0,18986374	0	0	1,876546685	5,114979209	C1QA;RPS17;RPS8;RPS6;RPSA;RPS3A;RPL10A;SEPP1
Valeric_Acid-23000_uM_in_DMSO-Rat-Primary_rat_hepatocytes-0.67d-up	8/293	0,065497687	0,189793886	0	0	1,876546685	5,114979209	RPS17;RPL32;RPS8;RPS5;RPSA;RPS3A;RPL10A;PDRG1
Azathioprine-54_mg/kg_in_Water-Rat-Liver-5d-up	8/294	0,066525687	0,192701842	0	0	1,870163873	5,068456671	RPS17;RPS19;CLP1;RPS6;RPS20;SEPP1;RPL39;TPT1
Chlorambucil-4.5_mg/kg_in_Corn_Oil-Rat-Bone_marrow-5d-dn	8/294	0,066525687	0,192630996	0	0	1,870163873	5,068456671	RPS17;RPS19;CLP1;RPS8;FUNDC1;RPS6;RPSA;RPS3A
Chlorpromazine-73_mg/kg_in_Water-Rat-Liver-1d-dn	8/294	0,066525687	0,192560202	0	0	1,870163873	5,068456671	EEF1B2;C1QA;RPS19;RPS6;RPSA;RPL10A;SEPP1;RPL39
Clotrimazole-89_mg/kg_in_Corn_Oil-Rat-Liver-5d-up	8/294	0,066525687	0,19248946	0	0	1,870163873	5,068456671	RPS17;RPL32;RPS19;RPS8;RPS20;RPS3A;RPL39;TPT1
Digoxin-0.26_mg/kg_in_Corn_Oil-Rat-Liver-0.25d-dn	8/294	0,066525687	0,19241877	0	0	1,870163873	5,068456671	EEF1B2;C1QA;RPS17;RPL32;RPS8;RPSA;RPS3A;TPT1
Diisopropyl_Fluorophosphate-3.75_mg/kg_in_Water-Rat-Heart-5d-dn	8/294	0,066525687	0,192348131	0	0	1,870163873	5,068456671	EEF1B2;RPS19;RPS6;RPS20;RAPGEF5;RPL10A;RPL39;GOLGA7
Mevastatin-1200_mg/kg_in_CMC-Rat-Skeletal_muscle-3d-dn	8/294	0,066525687	0,192277545	0	0	1,870163873	5,068456671	RPS17;RPL32;RPS19;ARL6IP5;RPSA;RPS3A;SLC16A3;TPT1
Norethindrone-0.08_mg/kg_in_Corn_Oil-Rat-Liver-0.25d-up	8/294	0,066525687	0,19220701	0	0	1,870163873	5,068456671	EEF1B2;RPL32;RPS19;RPS6;RPS20;RPS3A;RPL10A;SEPP1
Noscapine-570_mg/kg_in_CMC-Rat-Bone_marrow-3d-dn	8/294	0,066525687	0,192136527	0	0	1,870163873	5,068456671	EEF1B2;RPS19;RPS8;RPS5;NKG7;RPSA;RPL10A;SEPP1
Procarbazine-54_mg/kg_in_Water-Rat-Liver-5d-up	8/294	0,066525687	0,192066096	0	0	1,870163873	5,068456671	RPS17;RPS19;CLP1;RPS8;RPS6;RPS3A;SEPP1;GOLGA7
Risperidone-22_mg/kg_in_CMC-Rat-Heart-1d-dn	8/294	0,066525687	0,191995716	0	0	1,870163873	5,068456671	RPS17;RPS19;RPS8;FEM1B;COL5A3;RPSA;SEPP1;RPL39
Tacrine-24_mg/kg_in_Water-Rat-Heart-1d-dn	8/294	0,066525687	0,191925388	0	0	1,870163873	5,068456671	EEF1B2;RPS17;RPL32;RPS19;COL5A3;RPSA;RPL10A;RPL39
Theophylline-225_mg/kg_in_Water-Rat-Heart-3d-up	8/294	0,066525687	0,191855112	0	0	1,870163873	5,068456671	EEF1B2;C1QA;RPS8;RPSA;RAPGEF5;SEPP1;GOLGA7;TPT1
Tocainide-224_mg/kg_in_Corn_Oil-Rat-Liver-1d-up	8/294	0,066525687	0,191784886	0	0	1,870163873	5,068456671	EEF1B2;RPL32;RPS19;RPS8;RPS6;RPS20;SEPP1;TPT1
Ubenimex-1300_mg/kg_in_Water-Rat-Kidney-1d-dn	8/294	0,066525687	0,191714713	0	0	1,870163873	5,068456671	RPL32;ARL2BP;CLP1;RPS8;RPSA;RPS3A;SEPP1;TPT1
Rofecoxib-111_uM_in_DMSO-Rat-Primary_rat_hepatocytes-0.67d-up	10/398	0,066857874	0,192601543	0	0	1,726847295	4,671443455	EEF1B2;RPL32;RPS8;RPS5;RPS6;RPL35A;RPS3A;RPL39;TPT1;PDRG1
Carboplatin-6_mg/kg_in_Saline-Rat-Kidney-5d-dn	8/295	0,067563637	0,194563511	0	0	1,863824335	5,022420146	EEF1B2;RPS17;RPL32;ARL2BP;CLP1;RPL35A;RPS3A;SEPP1
Cyclosporin_A-70_mg/kg_in_Corn_Oil-Rat-Bone_marrow-0.25d-dn	8/295	0,067563637	0,194492399	0	0	1,863824335	5,022420146	RPS17;RPL32;RPS8;RPS5;RPS6;ARL6IP5;RPL35A;RPSA
Econazole-43_mg/kg_in_Corn_Oil-Rat-Liver-1d-up	8/295	0,067563637	0,194421338	0	0	1,863824335	5,022420146	RPS17;RPL32;RPS19;RPS6;RPS20;RPS3A;RPL39;TPT1
Enoxacin-750_mg/kg_in_Corn_Oil-Rat-Liver-1d-dn	8/295	0,067563637	0,19435033	0	0	1,863824335	5,022420146	EEF1B2;RPS17;RPL32;RPS6;RPSA;RPS3A;RPL10A;RPL39
Fluvastatin-94_mg/kg_in_Corn_Oil-Rat-Skeletal_muscle-3d-dn	8/295	0,067563637	0,194279373	0	0	1,863824335	5,022420146	FEM1B;COL5A3;RPS6;ARL6IP5;RPSA;RPL10A;RPL39;SLC16A3
Labetalol-60_mg/kg_in_Water-Rat-Heart-5d-up	8/295	0,067563637	0,194208468	0	0	1,863824335	5,022420146	RPS17;RPL32;RPS19;RPS6;RPSA;RAPGEF5;RPL10A;SEPP1
N-Nitrosodimethylamine-10_mg/kg_in_Saline-Rat-Liver-5d-up	8/295	0,067563637	0,194137615	0	0	1,863824335	5,022420146	C1QA;RPS19;RPS20;RPSA;RPL10A;RPL39;GOLGA7;TPT1
Pioglitazone-300_mg/kg_in_Corn_Oil-Rat-Liver-3d-up	8/295	0,067563637	0,194066814	0	0	1,863824335	5,022420146	RPS17;RPL32;RPL35A;RPS20;RPSA;RAPGEF5;RPL39;TPT1
Piroxicam-14_mg/kg_in_CMC-Rat-Kidney-1d-up	8/295	0,067563637	0,193996064	0	0	1,863824335	5,022420146	RPS17;RPS19;ARL6IP5;RPL13A;RPL35A;RPS20;RPSA;RPL39
Prednisone-68_mg/kg_in_CMC-Rat-Kidney-5d-up	8/295	0,067563637	0,193925366	0	0	1,863824335	5,022420146	EEF1B2;RPS17;RPL32;RPS8;RPS6;RPS20;RPSA;RPS3A
Pyrazinamide-1500_mg/kg_in_CMC-Rat-Liver-3d-up	8/295	0,067563637	0,193854719	0	0	1,863824335	5,022420146	EEF1B2;RPS19;RPS6;RPSA;RPS3A;RPL10A;SEPP1;RPL39
Risperidone-22_mg/kg_in_CMC-Rat-Heart-5d-up	8/295	0,067563637	0,193784123	0	0	1,863824335	5,022420146	EEF1B2;RPS17;CXCL11;RPL32;RPS8;ARL6IP5;RPS3A;TPT1
Safrole-488_mg/kg_in_Corn_Oil-Rat-Liver-5d-up	8/295	0,067563637	0,19371358	0	0	1,863824335	5,022420146	RPS17;RPL32;RPS8;RPS6;RPS20;RPS3A;RPL39;TPT1
Torsemide-3_mg/kg_in_Corn_Oil-Rat-Liver-0.25d-dn	8/295	0,067563637	0,193643087	0	0	1,863824335	5,022420146	RPS17;RPS19;FEM1B;FUNDC1;RPS6;RPS20;RPL10A;SEPP1
Bisphenol_A-100_mg/kg_in_Corn_Oil-Rat-Kidney-1d-up	7/245	0,067750076	0,194106801	0	0	1,963672067	5,286067158	RPS17;RPS8;RPS5;FUNDC1;RPL35A;RPS20;TPT1
Enoxacin-100_mg/kg_in_Corn_Oil-Rat-Liver-5d-up	7/245	0,067750076	0,194036217	0	0	1,963672067	5,286067158	RPS17;RPS19;RPS20;RPSA;RPL39;SEPP1;TPT1
Haloperidol-77_uM_in_DMSO-Rat-Primary_rat_hepatocytes-0.67d-dn	7/245	0,067750076	0,193965684	0	0	1,963672067	5,286067158	RPS17;RPL32;RPS8;RPS5;RPS20;RPS3A;PDRG1
Cisplatin-1.17_mg/kg_in_Saline-Rat-Kidney-0.25d-up	9/347	0,068151417	0,19504381	0	0	1,782584153	4,788062608	EEF1B2;RPS17;RPS19;CLP1;ARL6IP5;RPL35A;RPS20;RPSA;RPS3A
Famciclovir-1200_mg/kg_in_Saline-Rat-Spleen-3d-up	9/347	0,068151417	0,194972962	0	0	1,782584153	4,788062608	RPS17;RPS19;RPS5;RPS20;RPS3A;RPL10A;SEPP1;RPL39;TPT1
Acarbose-2000_mg/kg_in_Water-Rat-Kidney-1d-up	8/296	0,068611545	0,196218058	0	0	1,857527631	4,976863486	C1QA;RPS17;RPL32;RPS19;RPS6;RPS20;RAPGEF5;RPL10A
Acetazolamide-250_mg/kg_in_Corn_Oil-Rat-Liver-1d-up	8/296	0,068611545	0,196146836	0	0	1,857527631	4,976863486	RPS19;CLP1;RPS8;FEM1B;RPS6;RPS20;RPSA;GOLGA7
Allylamine-75_mg/kg_in_Water-Rat-Heart-1d-up	8/296	0,068611545	0,196075665	0	0	1,857527631	4,976863486	C1QA;RPS17;RPL32;RPS8;RPS6;ARL6IP5;RPS3A;TPT1
Bis(2-Ethylhexyl)Phthalate-100_mg/kg_in_Corn_Oil-Rat-Liver-0.25d-up	8/296	0,068611545	0,196004545	0	0	1,857527631	4,976863486	RPL32;RPS19;RPS5;RPS6;RPSA;RPL10A;SEPP1;RPL39
Chlordiazepoxide-196_mg/kg_in_Water-Rat-Heart-5d-up	8/296	0,068611545	0,195933478	0	0	1,857527631	4,976863486	EEF1B2;RPS17;RPL32;RPS19;ARL6IP5;RPS3A;RPL39;GOLGA7
Chlorpyrifos-82_mg/kg_in_CMC-Rat-Kidney-1d-up	8/296	0,068611545	0,195862462	0	0	1,857527631	4,976863486	RPS17;RPS19;CLP1;RPS3A;SEPP1;RPL39;GOLGA7;TPT1
Cisplatin-0.5_mg/kg_in_Saline-Rat-Kidney-5d-dn	8/296	0,068611545	0,195791497	0	0	1,857527631	4,976863486	EEF1B2;RPS17;RPL32;ARL2BP;FEM1B;RAPGEF5;SEPP1;TOMM20
Ergocalciferol-15_mg/kg_in_CMC-Rat-Kidney-3d-up	8/296	0,068611545	0,195720584	0	0	1,857527631	4,976863486	EEF1B2;CXCL11;RPL32;RPS19;RPS8;RPS6;RPS20;RPS3A
Ketoconazole-227_mg/kg_in_Corn_Oil-Rat-Liver-0.25d-up	8/296	0,068611545	0,195649722	0	0	1,857527631	4,976863486	EEF1B2;RPS19;RPS6;RPS20;RPL10A;SEPP1;RPL39;GOLGA7
Methyldopa-325_mg/kg_in_Water-Rat-Liver-1d-dn	8/296	0,068611545	0,195578911	0	0	1,857527631	4,976863486	RPS17;RPL32;RPS19;RPS8;RPS6;RPL10A;SEPP1;RPL39
Quetiapine-150_uM_in_DMSO-Rat-Primary_rat_hepatocytes-0.67d-dn	8/296	0,068611545	0,195508152	0	0	1,857527631	4,976863486	CXCL11;RPS19;RPS8;ARL6IP5;RPS20;RPSA;SEPP1;SLC16A3
Rofecoxib-3_mg/kg_in_Corn_Oil-Rat-Kidney-3d-dn	8/296	0,068611545	0,195437444	0	0	1,857527631	4,976863486	EEF1B2;RPS17;RPS19;ARL6IP5;RPSA;RPL10A;SEPP1;RPL39
Thioguanine-12_mg/kg_in_Corn_Oil-Rat-Liver-0.25d-up	8/296	0,068611545	0,195366787	0	0	1,857527631	4,976863486	EEF1B2;RPL32;RPS19;RPS6;RPS20;RPSA;RPS3A;RPL10A
Allyl_Alcohol-30_uM_in_DMSO-Rat-Primary_rat_hepatocytes-0.67d-up	10/400	0,068631402	0,195352699	0	0	1,718213058	4,603101548	RPL32;RPS8;RPS5;RPS6;RPL35A;RPSA;RPS3A;RPL39;TPT1;PDRG1
Troglitazone-100_mg/kg_in_Corn_Oil-Rat-Liver-5d-dn	10/400	0,068631402	0,195282124	0	0	1,718213058	4,603101548	EEF1B2;RPS17;RPL32;RPS19;RPS6;RPSA;RPL10A;SEPP1;RPL39;GOLGA7
Sodium_Arsenite-10_mg/kg_in_Saline-Rat-Bone_marrow-1d-dn	7/246	0,068913721	0,196014613	0	0	1,95568966	5,23127424	RPS17;RPL32;NKG7;RPSA;RPS3A;RPL10A;TPT1
Busulfan-9_mg/kg_in_Corn_Oil-Rat-Spleen-3d-dn	9/348	0,069116324	0,196519917	0	0	1,777461785	4,749314493	C1QA;RPS17;RPL32;RPS19;RPS8;RPSA;RPL10A;SEPP1;TPT1
Amiodarone-147_mg/kg_in_CMC-Rat-Kidney-5d-dn	8/297	0,069669421	0,19802106	0	0	1,851273329	4,931780639	EEF1B2;RPL32;ARL2BP;CLP1;RPS8;RPS5;RPL35A;TOMM20
Amlodipine-0.2_mg/kg_in_Corn_Oil-Rat-Liver-3d-dn	8/297	0,069669421	0,197949624	0	0	1,851273329	4,931780639	EEF1B2;RPS17;RPL32;RPS19;CLP1;RPS6;RPS3A;RPL10A
Bithionol-59_mg/kg_in_Corn_Oil-Rat-Liver-3d-up	8/297	0,069669421	0,19787824	0	0	1,851273329	4,931780639	EEF1B2;RPS17;RPL32;RPS19;RPS6;ARL6IP5;RPS20;RPS3A
Colchicine-0.6_mg/kg_in_Saline-Rat-Kidney-5d-up	8/297	0,069669421	0,197806906	0	0	1,851273329	4,931780639	CXCL11;RPS19;ARL2BP;RPS8;RPS6;RPS20;RPS3A;RPL10A
Diethylstilbestrol-120_uM_in_DMSO-Rat-Primary_rat_hepatocytes-0.67d-dn	8/297	0,069669421	0,197735625	0	0	1,851273329	4,931780639	CXCL11;RPS19;RPS8;RPS20;RPSA;RPL10A;SEPP1;SLC16A3
NN-Dimethylformamide-140_mg/kg_in_Saline-Rat-Bone_marrow-0.25d-dn	8/297	0,069669421	0,197664394	0	0	1,851273329	4,931780639	EEF1B2;RPL32;RPS8;RPL35A;RPS20;RPSA;RPL10A;TPT1
Oxcarbazepine-1600_mg/kg_in_CMC-Rat-Kidney-5d-up	8/297	0,069669421	0,197593215	0	0	1,851273329	4,931780639	RPS17;RPL32;CLP1;RPS8;RPS20;RPS3A;RAPGEF5;TPT1
Prinomastat-500_uM_in_DMSO-Rat-Primary_rat_hepatocytes-0.67d-dn	8/297	0,069669421	0,197522087	0	0	1,851273329	4,931780639	RPS17;CXCL11;RPL32;ARL2BP;RPS8;ARL6IP5;RPS3A;TPT1
Prochlorperazine-85_mg/kg_in_CMC-Rat-Heart-1d-up	8/297	0,069669421	0,197451011	0	0	1,851273329	4,931780639	C1QA;CXCL11;RPS19;FUNDC1;RPS6;NKG7;RAPGEF5;SEPP1
Sertraline-210_mg/kg_in_Corn_Oil-Rat-Heart-1d-dn	8/297	0,069669421	0,197379985	0	0	1,851273329	4,931780639	RPS17;RPL32;RPS19;ARL2BP;RPS6;RPL35A;RPS20;TPT1
Sulconazole-1200_mg/kg_in_Corn_Oil-Rat-Liver-1d-up	8/297	0,069669421	0,197309011	0	0	1,851273329	4,931780639	EEF1B2;C1QA;RPS17;RPL32;ARL6IP5;RPL35A;RPS20;RPSA
Sulpiride-667_uM_in_DMSO-Rat-Primary_rat_hepatocytes-1d-up	8/297	0,069669421	0,197238087	0	0	1,851273329	4,931780639	RPS17;ARL2BP;FEM1B;RPS6;RPS20;RPS3A;RPL10A;RPL39
Griseofulvin-75_uM_in_DMSO-Rat-Primary_rat_hepatocytes-1d-dn	9/349	0,070089763	0,1983568	0	0	1,772368771	4,710918138	EEF1B2;C1QA;RPS17;CXCL11;RPS19;FUNDC1;ARL6IP5;SEPP1;RPL39
33'5-Triiodo-L-Thyronine-0.1_mg/kg_in_PEG400-Rat-Heart-1d-up	8/298	0,07073727	0,200117362	0	0	1,845061002	4,887165646	EEF1B2;RPS17;RPL32;RPS19;ARL2BP;RPS20;RPSA;RAPGEF5
Anastrozole-400_mg/kg_in_CMC-Rat-Liver-1d-up	8/298	0,07073727	0,200045507	0	0	1,845061002	4,887165646	RPL32;RPS8;RPS5;RPL35A;RPSA;RPS3A;RPL10A;TPT1
Azaribine-750_mg/kg_in_CMC-Rat-Heart-1d-dn	8/298	0,07073727	0,199973703	0	0	1,845061002	4,887165646	RPS19;RPS8;ARL6IP5;RPSA;RPS3A;RPL10A;SEPP1;TPT1
Betamethasone-79_mg/kg_in_Corn_Oil-Rat-Bone_marrow-3d-up	8/298	0,07073727	0,199901951	0	0	1,845061002	4,887165646	EEF1B2;C1QA;RPS8;RPS5;RPL13A;RPL35A;RPS20;TPT1

Bisphenol_A-50_mg/kg_in_Corn_Oil-Rat-Liver-1d-up	8/298	0,07073727	0,19983025	0	0	1,845061002	4,887165646	EEF1B2;RPS17;RPL32;RPL35A;RPSA;RPS3A;SEPP1;RPL39
Diisopropyl_Fluorophosphate-3.75_mg/kg_in_Water-Rat-Liver-1d-dn	8/298	0,07073727	0,199758601	0	0	1,845061002	4,887165646	EEF1B2;RPL32;RPS19;RPS8;RPS6;RPS3A;SEPP1;RPL39
Fludrocortisone_Acetate-125_mg/kg_in_CMC-Rat-Heart-5d-dn	8/298	0,07073727	0,199687003	0	0	1,845061002	4,887165646	RPS17;RPS19;ARL2BP;COL5A3;RPS20;RPSA;RPS3A;TPT1
Lovastatin-1500_mg/kg_in_Corn_Oil-Rat-Kidney-5d-up	8/298	0,07073727	0,199615456	0	0	1,845061002	4,887165646	RPL32;RPS19;RPS5;RPS6;HOXA1;RPS20;RPS3A;RPL39
NN-Dimethylformamide-1400_mg/kg_in_Saline-Rat-Spleen-3d-dn	8/298	0,07073727	0,19954396	0	0	1,845061002	4,887165646	EEF1B2;C1QA;RPL32;RPS8;RPS6;ARL6IP5;RPSA;RPL10A
Pentoxifylline-1170_mg/kg_in_CMC-Rat-Heart-1d-up	8/298	0,07073727	0,199472516	0	0	1,845061002	4,887165646	C1QA;RPS17;RPL32;RPS6;ARL6IP5;RPS3A;RPL10A;TPT1
Sulindac-23_mg/kg_in_Corn_Oil-Rat-Kidney-1d-dn	8/298	0,07073727	0,199401123	0	0	1,845061002	4,887165646	EEF1B2;RPS17;RPL32;RPS8;RPS6;RPS3A;SEPP1;TOMM20
Tobramycin-40_mg/kg_in_Saline-Rat-Kidney-5d-dn	8/298	0,07073727	0,199329781	0	0	1,845061002	4,887165646	EEF1B2;RPS17;RPS19;ARL2BP;RPS8;RPS3A;RAPGEF5;SEPP1
Tranexamic_Acid-2000_mg/kg_in_CMC-Rat-Heart-5d-up	8/298	0,07073727	0,19925849	0	0	1,845061002	4,887165646	RPS17;RPS19;RPS6;RPS20;RPSA;RAPGEF5;RPL39;TPT1
Atropine-94_mg/kg_in_Water-Rat-Heart-3d-dn	9/350	0,071071742	0,200129081	0	0	1,76730486	4,672869738	EEF1B2;RPS8;FUNDC1;RPS6;RPS20;RPSA;RPS3A;SEPP1;TPT1
Atropine-94_mg/kg_in_Water-Rat-Liver-3d-up	9/350	0,071071742	0,200057556	0	0	1,76730486	4,672869738	RPS17;RPL32;RPS19;RPS6;RPS20;RPSA;RPL10A;SEPP1;RPL39
Glycine-2000_mg/kg_in_CMC-Rat-Kidney-1d-up	9/350	0,071071742	0,199986081	0	0	1,76730486	4,672869738	EEF1B2;RPL32;RPS19;RPS8;RPS6;RPL13A;RPS20;RPSA;SEPP1
Ethanol-6000_mg/kg_in_Saline-Rat-Bone_marrow-1d-dn	7/248	0,071276842	0,200491573	0	0	1,939917969	5,123679925	EEF1B2;RPS17;RPL32;RPS8;NKG7;RPS3A;TPT1
Pioglitazone-3_mg/kg_in_Corn_Oil-Rat-Liver-1d-dn	7/248	0,071276842	0,200419995	0	0	1,939917969	5,123679925	C1QA;RPS8;RPS6;RPSA;RPS3A;RPL39;GOLGA7
Acetazolamide-47_mg/kg_in_Corn_Oil-Rat-Liver-1d-dn	8/299	0,071815098	0,201861426	0	0	1,83889023	4,843012638	EEF1B2;C1QA;RPS17;RPL32;RPS6;RPL35A;RPS3A;SEPP1
Altretamine-40_mg/kg_in_Corn_Oil-Rat-Bone_marrow-5d-dn	8/299	0,071815098	0,201789409	0	0	1,83889023	4,843012638	RPS19;ARL2BP;CLP1;RPS8;FUNDC1;RPS6;ARL6IP5;RPSA
Altretamine-40_mg/kg_in_Corn_Oil-Rat-Liver-0.25d-up	8/299	0,071815098	0,201717445	0	0	1,83889023	4,843012638	RPS17;RPL32;RPS19;RPS6;RPS20;RPS3A;RPL10A;SEPP1
Antipyrine-1500_mg/kg_in_CMC-Rat-Kidney-1d-dn	8/299	0,071815098	0,201645531	0	0	1,83889023	4,843012638	EEF1B2;RPS19;RPS6;RPSA;RPS3A;RPL10A;SEPP1;RPL39
Azathioprine-20_mg/kg_in_Corn_Oil-Rat-Liver-1d-dn	8/299	0,071815098	0,201573669	0	0	1,83889023	4,843012638	EEF1B2;RPS17;RPL32;RPS8;RPS6;RPS3A;SEPP1;TPT1
Baclofen-6.6_mg/kg_in_CMC-Rat-Heart-5d-dn	8/299	0,071815098	0,201501858	0	0	1,83889023	4,843012638	EEF1B2;RPS17;RPL32;RPS8;ARL6IP5;RPL35A;RPS20;TPT1
Bezafibrate-617_mg/kg_in_Corn_Oil-Rat-Heart-1d-dn	8/299	0,071815098	0,201430098	0	0	1,83889023	4,843012638	RPS17;RPL32;RPS19;RPS5;FUNDC1;RPL35A;RPS20;RPS3A
Bisphenol_A-610_mg/kg_in_Corn_Oil-Rat-Liver-0.25d-up	8/299	0,071815098	0,201358389	0	0	1,83889023	4,843012638	RPS17;RPL32;RPS19;RPSA;RPS3A;RPL10A;SEPP1;TPT1
Diethylstilbestrol-280_mg/kg_in_Corn_Oil-Rat-Kidney-1d-dn	8/299	0,071815098	0,201286731	0	0	1,83889023	4,843012638	EEF1B2;RPS17;RPL32;RPS6;RPL35A;RPSA;SEPP1;RPL39
Flunarizine-126_mg/kg_in_CMC-Rat-Heart-5d-up	8/299	0,071815098	0,201215124	0	0	1,83889023	4,843012638	RPS17;RPL32;RPS8;RPS5;ARL6IP5;RPL35A;RPSA;TOMM20
Ibuprofen-54_mg/kg_in_Corn_Oil-Rat-Liver-1d-dn	8/299	0,071815098	0,201143569	0	0	1,83889023	4,843012638	EEF1B2;RPS17;RPL32;RPS8;ARL6IP5;RPL35A;RPS3A;SEPP1
Paraquat_Dichloride-650_uM_in_DMSO-Rat-Primary_rat_hepatocytes-1d-up	8/299	0,071815098	0,201072064	0	0	1,83889023	4,843012638	EEF1B2;RPS6;RPS20;RPSA;RPS3A;RPL10A;SLC16A3;PDRG1
Rosiglitazone-10_mg/kg_in_Corn_Oil-Rat-Liver-0.25d-up	8/299	0,071815098	0,201000609	0	0	1,83889023	4,843012638	EEF1B2;RPL32;RPS19;RPS5;RPL35A;RPS3A;SEPP1;PDRG1
Thioguanine-12_mg/kg_in_Corn_Oil-Rat-Spleen-1d-dn	8/299	0,071815098	0,200929206	0	0	1,83889023	4,843012638	RPS19;RPS8;RPS5;ARL6IP5;RPSA;RPL10A;SEPP1;RPL39
Tranilast-300_mg/kg_in_CMC-Rat-Liver-3d-dn	8/299	0,071815098	0,200857853	0	0	1,83889023	4,843012638	EEF1B2;RPS17;RPL32;RPS19;CLP1;RPS6;RPL35A;RPS20
Cytochalasin_B-167_uM_in_DMSO-Rat-Primary_rat_hepatocytes-1d-dn	9/351	0,072062269	0,201477612	0	0	1,762269804	4,635165535	EEF1B2;RPS6;ARL6IP5;RPSA;RPS3A;RPL10A;SEPP1;RPL39;SLC16A3
Loxoprofen-18_mg/kg_in_Corn_Oil-Rat-Kidney-5d-up	9/351	0,072062269	0,201406115	0	0	1,762269804	4,635165535	RPL32;RPS19;RPS8;RPS6;RPS3A;RAPGEF5;RPL10A;RPL39;TPT1
Melatonin-2000_mg/kg_in_Corn_Oil-Rat-Liver-0.25d-up	9/351	0,072062269	0,201334669	0	0	1,762269804	4,635165535	RPS17;RPS19;ARL2BP;RPS6;RPS20;RPSA;RPL10A;SEPP1;RPL39
Niacin-2625_mg/kg_in_Water-Rat-Liver-5d-up	9/351	0,072062269	0,201263274	0	0	1,762269804	4,635165535	RPS17;RPL32;RPS19;RPS8;RPS6;RPL35A;RPSA;RPS3A;SEPP1
3-Methylcholanthrene-23_uM_in_DMSO-Rat-Primary_rat_hepatocytes-0.67d-dn	7/249	0,07247632	0,202347924	0	0	1,932127134	5,070858768	RPS17;RPS19;ARL6IP5;RPS20;RPSA;RPL10A;SEPP1
Acyclovir-330_mg/kg_in_Saline-Rat-Bone_marrow-3d-dn	7/249	0,07247632	0,20227622	0	0	1,932127134	5,070858768	EEF1B2;C1QA;RPS19;RPS6;NKG7;RPS20;RPL39
Clofibrate-500_uM_in_DMSO-Rat-Primary_rat_hepatocytes-1d-up	7/249	0,07247632	0,202204567	0	0	1,932127134	5,070858768	EEF1B2;RPS17;CXCL11;RPS6;ARL6IP5;RPL10A;SEPP1
Acetaminophen-972_mg/kg_in_Corn_Oil-Rat-Liver-3d-up	8/300	0,072902911	0,203322709	0	0	1,832760596	4,799315838	EEF1B2;RPS19;RPS8;RPS6;RPSA;RPS3A;RPL10A;RPL39
Acyclovir-330_mg/kg_in_Saline-Rat-Liver-5d-dn	8/300	0,072902911	0,203250736	0	0	1,832760596	4,799315838	EEF1B2;RPS17;RPL32;RPS6;RPSA;RPS3A;RPL10A;RPL39
Amiodarone-147_mg/kg_in_CMC-Rat-Kidney-3d-up	8/300	0,072902911	0,203178815	0	0	1,832760596	4,799315838	C1QA;RPS6;RPS20;RPSA;RPS3A;RPL10A;RPL39;TPT1
Benzethonium_Chloride-30_mg/kg_in_Corn_Oil-Rat-Liver-3d-dn	8/300	0,072902911	0,203106944	0	0	1,832760596	4,799315838	RPL32;RPS19;RPS6;RPS20;RPSA;RPL10A;RPL39;TPT1
Bupropion-895_mg/kg_in_Water-Rat-Liver-3d-dn	8/300	0,072902911	0,203035124	0	0	1,832760596	4,799315838	C1QA;RPS17;RPL32;CLP1;RPL35A;RPL39;GOLGA7;TPT1
Cobalt_(II)_Chloride-10_mg/kg_in_Saline-Rat-Heart-14d-up	8/300	0,072902911	0,202963355	0	0	1,832760596	4,799315838	C1QA;ARL2BP;ARL6IP5;RPS3A;RPL10A;GOLGA7;TPT1;PDRG1
Diethylstilbestrol-280_mg/kg_in_Corn_Oil-Rat-Bone_marrow-1d-up	8/300	0,072902911	0,202891636	0	0	1,832760596	4,799315838	C1QA;NKG7;RPS20;RPL10A;SEPP1;RPL39;GOLGA7;TPT1
Doxorubicin-4_mg/kg_in_Saline-Rat-Kidney-5d-dn	8/300	0,072902911	0,202819968	0	0	1,832760596	4,799315838	RPS17;RPS8;RPS6;RPL35A;RPS3A;SEPP1;RPL39;TPT1
Fenofibrate-215_mg/kg_in_Corn_Oil-Rat-Liver-5d-up	8/300	0,072902911	0,202748351	0	0	1,832760596	4,799315838	RPS17;RPL32;RPS8;RPS6;RPS3A;RPL10A;RPL39;TPT1
Glimepiride-2500_mg/kg_in_CMC-Rat-Liver-3d-up	8/300	0,072902911	0,202676784	0	0	1,832760596	4,799315838	RPS19;RPS6;RPS20;RPSA;RPS3A;RAPGEF5;RPL10A;RPL39
Isoeugenol-1560_mg/kg_in_CMC-Rat-Kidney-5d-up	8/300	0,072902911	0,202605268	0	0	1,832760596	4,799315838	EEF1B2;RPS17;RPL32;CLP1;RPS8;RPS3A;SEPP1;TPT1
Minoxidil-83_mg/kg_in_CMC-Rat-Heart-3d-up	8/300	0,072902911	0,202533803	0	0	1,832760596	4,799315838	C1QA;RPS17;RPL32;RPS19;RPS8;RPS6;RPS20;RPL39
Mosapride-30_mg/kg_in_CMC-Rat-Intestine-5d-dn	8/300	0,072902911	0,202462387	0	0	1,832760596	4,799315838	EEF1B2;RPS17;RPS6;ARL6IP5;RPL13A;RPL35A;RPS3A;RPL39
Praziquantel-1200_mg/kg_in_CMC-Rat-Liver-5d-dn	8/300	0,072902911	0,202391022	0	0	1,832760596	4,799315838	EEF1B2;C1QA;RPS17;RPL32;RPS5;RPS3A;RPL39;TPT1
Ifosfamide-143_mg/kg_in_Saline-Rat-Spleen-3d-up	9/352	0,073061352	0,20275941	0	0	1,757263355	4,597801823	EEF1B2;C1QA;CXCL11;RPS19;RPS20;RAPGEF5;RPL10A;SEPP1;GOLGA7
Pramoxine-526_mg/kg_in_Corn_Oil-Rat-Heart-3d-up	9/352	0,073061352	0,202687991	0	0	1,757263355	4,597801823	C1QA;RPL32;RPS19;ARL6IP5;RPSA;RPS3A;RAPGEF5;RPL10A;RPL39
Epinephrine-0.0375_mg/kg_in_Saline-Rat-Heart-1d-dn	7/250	0,073687741	0,20435375	0	0	1,924398625	5,018675406	ARL2BP;RPS8;COL5A3;ARL6IP5;RPS20;RPS3A;TPT1
Olanzapine-250_uM_in_DMSO-Rat-Primary_rat_hepatocytes-1d-up	7/250	0,073687741	0,20428182	0	0	1,924398625	5,018675406	EEF1B2;RPS17;RPS20;RPSA;RPS3A;RPL10A;PDRG1
3-Methylcholanthrene-300_mg/kg_in_CMC-Rat-Liver-1d-dn	8/301	0,074000713	0,205077275	0	0	1,82667169	4,756069555	EEF1B2;RPS19;RPS8;RPS6;RPS20;SEPP1;RPL39;TPT1
Amikacin-20_mg/kg_in_Saline-Rat-Bone_marrow-0.25d-dn	8/301	0,074000713	0,205005141	0	0	1,82667169	4,756069555	EEF1B2;RPL32;RPS5;RPS6;RPL35A;RPS3A;RPL10A;RPL39
Antipyrine-1500_mg/kg_in_CMC-Rat-Kidney-1d-up	8/301	0,074000713	0,204933057	0	0	1,82667169	4,756069555	RPS17;RPL32;ARL2BP;RPS8;FUNDC1;ARL6IP5;RPS20;TPT1
Busulfan-3_mg/kg_in_Corn_Oil-Rat-Liver-0.25d-dn	8/301	0,074000713	0,204861024	0	0	1,82667169	4,756069555	RPS17;RPL32;RPS8;RPL35A;RPS20;RPSA;RPS3A;TPT1
Etoposide-100_mg/kg_in_Corn_Oil-Rat-Liver-3d-up	8/301	0,074000713	0,204789042	0	0	1,82667169	4,756069555	EEF1B2;RPL32;RPS19;RPS8;RPL35A;RPS20;RPSA;TPT1
Glipizide-2500_mg/kg_in_CMC-Rat-Liver-3d-dn	8/301	0,074000713	0,204717111	0	0	1,82667169	4,756069555	EEF1B2;RPS17;CLP1;RPS8;RPS5;RPS3A;SEPP1;TPT1
Ibuprofen-263_mg/kg_in_Corn_Oil-Rat-Liver-3d-up	8/301	0,074000713	0,20464523	0	0	1,82667169	4,756069555	EEF1B2;C1QA;RPS19;RPS5;RPS20;SEPP1;RPL39;TPT1
Ibuprofen-54_mg/kg_in_Corn_Oil-Rat-Liver-1d-up	8/301	0,074000713	0,204573399	0	0	1,82667169	4,756069555	C1QA;RPS19;FEM1B;RPS6;RPS20;RPSA;RPL39;TPT1
Idebenone-2500_mg/kg_in_CMC-Rat-Liver-1d-dn	8/301	0,074000713	0,204501619	0	0	1,82667169	4,756069555	EEF1B2;C1QA;RPS17;RPL32;RPS19;RPS6;RPS3A;SEPP1
Ipriflavone-1500_mg/kg_in_CMC-Rat-Liver-1d-up	8/301	0,074000713	0,204429889	0	0	1,82667169	4,756069555	RPS17;RPL32;FEM1B;RPS5;RPSA;RPS3A;RPL39;TPT1
Isoeugenol-1560_mg/kg_in_CMC-Rat-Kidney-3d-dn	8/301	0,074000713	0,20435821	0	0	1,82667169	4,756069555	RPS19;ARL2BP;CLP1;RPS8;RPS6;RPS20;RPSA;RPL39
Mitomycin_C-1.7_mg/kg_in_Saline-Rat-Spleen-5d-up	8/301	0,074000713	0,204286581	0	0	1,82667169	4,756069555	C1QA;RPL32;RPS19;RPS6;RPS3A;RAPGEF5;SEPP1;RPL39
Niacin-2625_mg/kg_in_Water-Rat-Liver-3d-up	8/301	0,074000713	0,204215002	0	0	1,82667169	4,756069555	C1QA;RPS17;RPL32;RPS8;RPS6;RPL35A;RPSA;RPS3A
Terazosin-657_mg/kg_in_Water-Rat-Heart-5d-up	8/301	0,074000713	0,204143473	0	0	1,82667169	4,756069555	RPS19;FUNDC1;RPS6;ARL6IP5;RPL10A;SEPP1;RPL39;GOLGA7
Fluphenazine-45_uM_in_DMSO-Rat-Primary_rat_hepatocytes-1d-dn	9/353	0,074068996	0,204260297	0	0	1,752285272	4,560774943	RPS19;ARL2BP;CLP1;RPS8;ARL6IP5;RPS20;SEPP1;RPL39;TPT1
Griseofulvin-75_uM_in_DMSO-Rat-Primary_rat_hepatocytes-1d-up	7/251	0,074911104	0,206510274	0	0	1,916731699	4,967120406	RPL32;CLP1;RPS8;RPL35A;RPS20;RPS3A;TPT1
Rapamycin-20_uM_in_DMSO-Rat-Primary_rat_hepatocytes-1d-up	7/251	0,074911104	0,206438017	0	0	1,916731699	4,967120406	EEF1B2;RPL32;ARL2BP;RPS5;RPS20;RPSA;RPL10A
Diethanolamine-620_mg/kg_in_Water-Rat-Kidney-3d-up	10/407	0,075073456	0,206813059	0	0	1,688661482	4,37243131	EEF1B2;C1QA;RPS19;CLP1;FUNDC1;RPS3A;SEPP1;RPL39;GOLGA7;TPT1
Sodium_Arsenite-10_mg/kg_in_Saline-Rat-Bone_marrow-1d-up	9/354	0,075085207	0,206773108	0	0	1,747335314	4,524081285	EEF1B2;RPS19;RPS8;RPS5;FUNDC1;RPS6;RPL35A;RPS20;RPL39
4-Nonylphenol-200_mg/kg_in_Corn_Oil-Rat-Kidney-1d-dn	8/302	0,075108506	0,206764974	0	0	1,820623108	4,713268187	EEF1B2;RPS17;RPL32;RPS8;RPS6;RPL35A;RPS3A;RPL39
Azathioprine-54_mg/kg_in_Water-Rat-Bone_marrow-5d-dn	8/302	0,075108506	0,206692729	0	0	1,820623108	4,713268187	EEF1B2;RPS17;RPL32;RPS19;RPS6;RPSA;RPL10A;RPL39
Carboplatin-6_mg/kg_in_Saline-Rat-Liver-5d-dn	8/302	0,075108506	0,206620535	0	0	1,820623108	4,713268187	EEF1B2;RPS17;RPL32;RPS6;RPSA;RPS3A;RPL10A;RPL39
Chlorpromazine-73_mg/kg_in_Water-Rat-Liver-3d-up	8/302	0,075108506	0,206548391	0	0	1,820623108	4,713268187	RPS19;RPS8;RPS6;RPS20;RPSA;RPS3A;RPL10A;SEPP1
Doxorubicin-3_mg/kg_in_Saline-Rat-Liver-0.25d-up	8/302	0,075108506	0,206476297	0	0	1,820623108	4,713268187	RPL32;RPS19;ARL2BP;RPS8;RPS20;RPSA;SEPP1;RPL39
Flurbiprofen-10_mg/kg_in_CMC-Rat-Kidney-1d-dn	8/302	0,075108506	0,206404254	0	0	1,820623108	4,713268187	RPS17;RPS20;RPSA;RPS3A;SEPP1;RPL39;GOLGA7;TPT1
Ibuprofen-263_mg/kg_in_Corn_Oil-Rat-Kidney-1d-up	8/302	0,075108506	0,206332261	0	0	1,820623108	4,713268187	RPS19;RPS5;RPS6;RPS20;RAPGEF5;RPL10A;RPL39;TPT1
Ibuprofen-263_mg/kg_in_Corn_Oil-Rat-Liver-0.25d-up	8/302	0,075108506	0,206260318	0	0	1,820623108	4,713268187	EEF1B2;C1QA;RPS17;RPS19;RPS6;RPS3A;RPL10A;SEPP1
Indomethacin-12_mg/kg_in_Water-Rat-Liver-0.25d-up	8/302	0,075108506	0,206188425	0	0	1,820623108	4,713268187	RPS19;ARL2BP;RPS8;RPS6;RPS20;RPSA;SEPP1;GOLGA7
Isotretinoin-125_mg/kg_in_Corn_Oil-Rat-Liver-1d-dn	8/302	0,075108506	0,206116582	0	0	1,820623108	4,713268187	EEF1B2;C1QA;RPS17;RPL32;RPS8;RPL10A;SEPP1;RPL39
Metronidazole-50_mg/kg_in_Water-Rat-Liver-0.25d-up	8/302	0,075108506	0,20604479	0	0	1,820623108	4,713268187	C1QA;ARL2BP;RPS8;FEM1B;RPS6;RPSA;RPS3A;SEPP1
Norethindrone-0.08_mg/kg_in_Corn_Oil-Rat-Liver-1d-up	8/302	0,075108506	0,205973047	0	0	1,820623108	4,713268187	EEF1B2;RPL32;RPS19;RPS8;RPS6;RPS20;RPSA;SEPP1
Phenobarbital-54_mg/kg_in_Water-Rat-Liver-1d-up	8/302	0,075108506	0,205901354	0	0	1,820623108	4,713268187	RPS17;RPS19;RPS6;RPS20;RPSA;RPS3A;RPL10A;RPL39
Sildenafil-300_mg/kg_in_Saline-Rat-Liver-3d-dn	8/302	0,075108506	0,205829712	0	0	1,820623108	4,713268187	C1QA;CLP1;RPS8;RPS6;RPS3A;SEPP1;RPL39;TPT1
Torsemide-110_mg/kg_in_Corn_Oil-Rat-Liver-0.25d-up	8/302	0,075108506	0,205758119	0	0	1,820623108	4,713268187	EEF1B2;C1QA;RPS19;RPS6;RPL10A;SEPP1;RPL39;TPT1
Bisphenol_A-100_mg/kg_in_Corn_Oil-Rat-Kidney-1d-dn	9/355	0,07610999	0,208429167	0	0	1,742413242	4,487717284	EEF1B2;RPS19;ARL2BP;CLP1;RPS6;RPSA;RPL10A;SEPP1;RPL39
Benzethonium_Chloride-30_mg/kg_in_Corn_Oil-Rat-Heart-1d-up	7/252	0,076146403	0,208456401	0	0	1,90912562	4,916184502	C1QA;RPS19;FEM1B;RPS6;RPS3A;RAPGEF5;TPT1

Citalopram-150_uM_in_DMSO-Rat-Primary_rat_hepatocytes-1d-up	7/252	0,076146403	0,20838397	0	0	1,90912562	4,916184502	EEF1B2;RPS17;RPL32;RPS8;RPS5;RPL35A;RPS3A
Acetaminophen-486_mg/kg_in_Corn_Oil-Rat-Kidney-3d-up	8/303	0,076226292	0,208530141	0	0	1,814614451	4,670906216	RPS17;RPL32;RPS8;RPS5;RPS6;RPL35A;RPS20;TOMM20
Aspirin-35_mg/kg_in_Corn_Oil-Rat-Heart-5d-up	8/303	0,076226292	0,208457735	0	0	1,814614451	4,670906216	EEF1B2;RPS8;ARL6IP5;RPS20;RAPGEF5;RPL10A;TPT1;PDRG1
Bezafibrate-100_mg/kg_in_Corn_Oil-Rat-Liver-1d-up	8/303	0,076226292	0,208385379	0	0	1,814614451	4,670906216	RPS17;RPL32;RPS19;RPS8;RPS6;RPS20;RPS3A;TPT1
Bezafibrate-617_mg/kg_in_Corn_Oil-Rat-Kidney-3d-up	8/303	0,076226292	0,208313073	0	0	1,814614451	4,670906216	RPS19;RPS8;RPS5;RPS6;RPS20;RPS3A;RPL39;TPT1
Choline_Chloride-2550_mg/kg_in_Water-Rat-Liver-5d-up	8/303	0,076226292	0,208240817	0	0	1,814614451	4,670906216	C1QA;RPS17;RPL32;RPS19;RPS6;RPSA;RPS3A;SEPP1
Clomipramine-115_mg/kg_in_Water-Rat-Liver-5d-up	8/303	0,076226292	0,208168612	0	0	1,814614451	4,670906216	C1QA;RPS19;RPS8;RPS6;RPSA;RPS3A;RPL10A;TPT1
Clotrimazole-178_mg/kg_in_Corn_Oil-Rat-Liver-1d-dn	8/303	0,076226292	0,208096456	0	0	1,814614451	4,670906216	EEF1B2;RPL32;RPS8;RPS6;RPS3A;RPL10A;SEPP1;TPT1
Doxorubicin-4_mg/kg_in_Saline-Rat-Heart-5d-dn	8/303	0,076226292	0,208024351	0	0	1,814614451	4,670906216	RPS17;RPL32;RPS8;RPS6;RPS3A;RAPGEF5;RPL39;TPT1
Eperisone-501_mg/kg_in_CMC-Rat-Liver-3d-up	8/303	0,076226292	0,207952295	0	0	1,814614451	4,670906216	EEF1B2;RPS17;RPL32;RPS8;RPL35A;RPS20;RPSA;RPS3A
Etoposide-188_mg/kg_in_Corn_Oil-Rat-Bone_marrow-5d-up	8/303	0,076226292	0,207880289	0	0	1,814614451	4,670906216	C1QA;RPL32;RPS8;RPS5;NKG7;RPL35A;RPS20;SEPP1
Fluphenazine-45_uM_in_DMSO-Rat-Primary_rat_hepatocytes-0.67d-dn	8/303	0,076226292	0,207808334	0	0	1,814614451	4,670906216	CXCL11;CLP1;RPS8;ARL6IP5;RPS20;SEPP1;SLC16A3;TPT1
Hydralazine-280_mg/kg_in_Water-Rat-Liver-5d-up	8/303	0,076226292	0,207736428	0	0	1,814614451	4,670906216	RPS17;RPL32;RPS8;RPS5;RPS20;RPS3A;SEPP1;GOLGA7
Levosulpiride-1200_mg/kg_in_CMC-Rat-Heart-5d-dn	8/303	0,076226292	0,207664571	0	0	1,814614451	4,670906216	EEF1B2;RPS19;ARL2BP;COL5A3;RPS6;RPSA;RPL10A;RPL39
Mitomycin_C-1.7_mg/kg_in_Saline-Rat-Liver-5d-up	8/303	0,076226292	0,207592765	0	0	1,814614451	4,670906216	EEF1B2;RPS17;RPS19;RPS8;RPS20;RPS3A;GOLGA7;TPT1
NN-Dimethylformamide-140_mg/kg_in_Saline-Rat-Bone_marrow-0.25d-up	8/303	0,076226292	0,207521008	0	0	1,814614451	4,670906216	C1QA;RPS17;RPS19;RPS6;NKG7;RPS3A;SEPP1;RPL39
Norethindrone-125_uM_in_DMSO-Rat-Primary_rat_hepatocytes-0.67d-up	8/303	0,076226292	0,207449301	0	0	1,814614451	4,670906216	EEF1B2;RPS17;RPL32;RPS6;RPS20;RPSA;RPL10A;RPL39
Oxcarbazepine-1600_mg/kg_in_CMC-Rat-Kidney-5d-dn	8/303	0,076226292	0,207377643	0	0	1,814614451	4,670906216	RPS19;ARL2BP;RPS6;ARL6IP5;RPL10A;SEPP1;RPL39;PDRG1
Phenacemide-400_mg/kg_in_CMC-Rat-Kidney-1d-up	8/303	0,076226292	0,207306035	0	0	1,814614451	4,670906216	RPS17;RPS8;RPS6;RPSA;RPS3A;RPL10A;SEPP1;TPT1
Sulconazole-1200_mg/kg_in_Corn_Oil-Rat-Liver-1d-dn	8/303	0,076226292	0,207234476	0	0	1,814614451	4,670906216	RPS8;RPS6;RPS3A;RPL10A;SEPP1;RPL39;GOLGA7;TPT1
Amikacin-20_mg/kg_in_Saline-Rat-Bone_marrow-1d-dn	8/304	0,077354073	0,210227977	0	0	1,808645325	4,628978207	EEF1B2;RPS17;RPS8;FUNDC1;NKG7;RPS20;RPL39;TPT1
Antimycin_A-0.03_uM_in_DMSO-Rat-Primary_rat_hepatocytes-0.67d-dn	8/304	0,077354073	0,210155459	0	0	1,808645325	4,628978207	C1QA;RPS17;RPL32;RPS6;RPS3A;SEPP1;RPL39;SLC16A3
Azasetron-580_mg/kg_in_CMC-Rat-Intestine-5d-dn	8/304	0,077354073	0,210082992	0	0	1,808645325	4,628978207	EEF1B2;RPS19;RPS6;RPL13A;RPL35A;RPS3A;SEPP1;RPL39
Azithromycin-225_mg/kg_in_Water-Rat-Liver-5d-up	8/304	0,077354073	0,210010574	0	0	1,808645325	4,628978207	EEF1B2;C1QA;RPS17;RPL32;RPS20;RPS3A;SEPP1;TPT1
Bisphenol_A-610_mg/kg_in_Corn_Oil-Rat-Spleen-1d-up	8/304	0,077354073	0,209938207	0	0	1,808645325	4,628978207	C1QA;RPS17;RPL32;ARL2BP;RPS8;RPS3A;SEPP1;TPT1
Carmofur-201_mg/kg_in_CMC-Rat-Bone_marrow-3d-up	8/304	0,077354073	0,209865889	0	0	1,808645325	4,628978207	EEF1B2;RPL32;RPS8;RPS5;RPS20;RPSA;SEPP1;TPT1
Cisplatin-0.5_mg/kg_in_Saline-Rat-Kidney-5d-up	8/304	0,077354073	0,209793621	0	0	1,808645325	4,628978207	CXCL11;CLP1;RPS6;RPSA;RPS3A;RPL10A;RPL39;TPT1
Diazepam-710_mg/kg_in_CMC-Rat-Heart-5d-dn	8/304	0,077354073	0,209721403	0	0	1,808645325	4,628978207	RPS17;COL5A3;FUNDC1;RPS6;ARL6IP5;RPL10A;SEPP1;PDRG1
Diphenidol-300_mg/kg_in_Water-Rat-Heart-1d-up	8/304	0,077354073	0,209649235	0	0	1,808645325	4,628978207	RPS17;RPL32;RPS8;RPS6;ARL6IP5;RPSA;RPS3A;RAPGEF5
Famciclovir-1200_mg/kg_in_Saline-Rat-Spleen-5d-dn	8/304	0,077354073	0,209577116	0	0	1,808645325	4,628978207	EEF1B2;C1QA;RPS17;RPL32;RPS8;RPS5;RPL13A;RPL35A
Mianserin-116_mg/kg_in_Corn_Oil-Rat-Heart-5d-dn	8/304	0,077354073	0,209505047	0	0	1,808645325	4,628978207	EEF1B2;RPL32;RPS19;ARL2BP;RPS8;RPSA;RPS3A;SEPP1
Prednisolone-184_mg/kg_in_Water-Rat-Kidney-3d-dn	8/304	0,077354073	0,209433027	0	0	1,808645325	4,628978207	RPS17;RPL32;RPS19;RPS8;RPS5;RPL35A;RPSA;RPS3A
Sulfanilamide-636_mg/kg_in_Corn_Oil-Rat-Kidney-3d-up	8/304	0,077354073	0,209361057	0	0	1,808645325	4,628978207	RPS17;RPL32;RPS19;ARL2BP;RPS6;RPSA;RPS3A;RPL10A
Tacrine-24_mg/kg_in_Water-Rat-Liver-3d-dn	8/304	0,077354073	0,209289137	0	0	1,808645325	4,628978207	RPS17;RPL32;RPS19;RPS8;RPL35A;RPS20;RPL39;TPT1
Tamoxifen-64_mg/kg_in_Corn_Oil-Rat-Liver-0.25d-dn	8/304	0,077354073	0,209217265	0	0	1,808645325	4,628978207	C1QA;RPS17;RPL32;CLP1;RPL35A;RPS20;RPL39;TPT1
Thioguanine-24_mg/kg_in_Corn_Oil-Rat-Liver-3d-up	8/304	0,077354073	0,209145443	0	0	1,808645325	4,628978207	RPL32;RPS19;RPL35A;RPS20;RPSA;SEPP1;RPL39;TPT1
Tretinoin-7_mg/kg_in_Corn_Oil-Rat-Kidney-5d-dn	8/304	0,077354073	0,209073671	0	0	1,808645325	4,628978207	EEF1B2;RPS17;RPL32;RPS19;RPS8;RPS3A;RPL10A;GOLGA7
Simvastatin-1200_mg/kg_in_Corn_Oil-Rat-Liver-3d-up	7/253	0,077393632	0,209108833	0	0	1,901579669	4,865858602	RPS17;RPS6;RPS20;RPSA;RPS3A;RPL10A;RPL39
Allopurinol-175_mg/kg_in_Corn_Oil-Rat-Kidney-5d-up	8/305	0,078491848	0,212003358	0	0	1,80271534	4,587478808	C1QA;RPS17;RPS19;RPS6;RPS20;RPS3A;RPL10A;RPL39
Alprazolam-115_mg/kg_in_Corn_Oil-Rat-Liver-3d-dn	8/305	0,078491848	0,21193068	0	0	1,80271534	4,587478808	EEF1B2;RPS17;RPS19;RPS20;RPSA;RPL10A;RPL39;TPT1
Amikacin-20_mg/kg_in_Saline-Rat-Kidney-5d-up	8/305	0,078491848	0,211858051	0	0	1,80271534	4,587478808	C1QA;RPS19;RPS6;ARL6IP5;RPS20;RPSA;SEPP1;GOLGA7
Benoxaprofen-20_mg/kg_in_CMC-Rat-Liver-1d-up	8/305	0,078491848	0,211785472	0	0	1,80271534	4,587478808	EEF1B2;RPS17;RPL32;RPS19;RPS8;RPS20;RPSA;TPT1
Bisphenol_A-150_mg/kg_in_Corn_Oil-Rat-Liver-3d-dn	8/305	0,078491848	0,211712943	0	0	1,80271534	4,587478808	EEF1B2;RPL32;RPS19;RPS8;RPS6;RPS3A;RPL10A;TPT1
Carbimazole-400_mg/kg_in_Water-Rat-Liver-1d-up	8/305	0,078491848	0,211640463	0	0	1,80271534	4,587478808	C1QA;RPS17;FEM1B;RPS6;RPSA;RPL10A;SEPP1;RPL39
Carboplatin-6_mg/kg_in_Saline-Rat-Kidney-5d-up	8/305	0,078491848	0,211568033	0	0	1,80271534	4,587478808	RPS19;RPS6;ARL6IP5;RPS20;RPSA;RPL10A;RPL39;GOLGA7
Catechol-40_mg/kg_in_Saline-Rat-Kidney-1d-up	8/305	0,078491848	0,211495653	0	0	1,80271534	4,587478808	RPS17;RPS19;FEM1B;RPS6;RPL35A;RPS20;RPS3A;SEPP1
Chloramphenicol-1500_mg/kg_in_CMC-Rat-Kidney-5d-dn	8/305	0,078491848	0,211423322	0	0	1,80271534	4,587478808	EEF1B2;ARL2BP;RPS20;RPSA;RPS3A;RPL10A;RPL39;TPT1
Cyproterone_Acetate-2500_mg/kg_in_CMC-Rat-Liver-3d-up	8/305	0,078491848	0,21135104	0	0	1,80271534	4,587478808	RPS17;RPS19;RPS6;RPS20;RPSA;RPL10A;SEPP1;RPL39
Ethacrynic_Acid-125_mg/kg_in_CMC-Rat-Kidney-5d-up	8/305	0,078491848	0,211278808	0	0	1,80271534	4,587478808	EEF1B2;RPL32;RPS19;ARL2BP;RPS8;RPS5;RPSA;TPT1
Melphalan-12_mg/kg_in_CMC-Rat-Spleen-3d-up	8/305	0,078491848	0,211206625	0	0	1,80271534	4,587478808	RPS19;RPS8;RPS5;RPSA;RAPGEF5;RPL10A;SEPP1;TPT1
Methyl_Methanesulfonate-113_mg/kg_in_Water-Rat-Bone_marrow-5d-up	8/305	0,078491848	0,211134492	0	0	1,80271534	4,587478808	EEF1B2;C1QA;RPS19;RPS8;FUNDC1;RPS6;RPSA;RPL10A
Metoprolol-1200_mg/kg_in_Water-Rat-Heart-3d-dn	8/305	0,078491848	0,211062408	0	0	1,80271534	4,587478808	CXCL11;RPS19;ARL2BP;RPS8;RPS20;SEPP1;RPL39;TPT1
NN-Dimethylformamide-1400_mg/kg_in_Saline-Rat-Kidney-1d-up	8/305	0,078491848	0,210990373	0	0	1,80271534	4,587478808	RPS17;RPL32;RPS8;RPL35A;RPS20;RPSA;RPL39;TPT1
Pyrazinamide-1500_mg/kg_in_CMC-Rat-Liver-3d-dn	8/305	0,078491848	0,210918387	0	0	1,80271534	4,587478808	C1QA;RPS17;RPL32;CLP1;RPS8;FEM1B;RPL35A;TPT1
Rapamycin-60_mg/kg_in_CMC-Rat-Kidney-1d-dn	8/305	0,078491848	0,21084645	0	0	1,80271534	4,587478808	EEF1B2;RPL32;RPS8;RPL35A;RPS3A;RPL10A;TOMM20;TPT1
Stavudine-1400_mg/kg_in_Water-Rat-Heart-5d-up	8/305	0,078491848	0,210774563	0	0	1,80271534	4,587478808	ARL2BP;RPS6;ARL6IP5;RPSA;RPL10A;SEPP1;RPL39;TPT1
Tamoxifen-64_mg/kg_in_Corn_Oil-Rat-Liver-1d-up	8/305	0,078491848	0,210702724	0	0	1,80271534	4,587478808	RPS19;RPS8;RPS6;RPS20;RPSA;RPS3A;RPL39;GOLGA7
Amiloride-22_mg/kg_in_CMC-Rat-Heart-3d-up	7/254	0,078652786	0,211062809	0	0	1,894093135	4,816133773	C1QA;RPS19;RPS8;RPS6;RPS3A;GOLGA7;TPT1
Stanozolol-39_mg/kg_in_Water-Rat-Liver-0.25d-dn	7/254	0,078652786	0,210990921	0	0	1,894093135	4,816133773	EEF1B2;RPS17;RPL32;RPS8;RPL35A;RPL39;TPT1
Nisoldipine-1125_mg/kg_in_Water-Rat-Liver-0.25d-up	9/358	0,079235813	0,212482554	0	0	1,727812014	4,38056828	C1QA;RPS19;ARL2BP;RPS8;RPS6;RPS20;RPSA;RPS3A;RPL10A
4-Nonylphenol-20_mg/kg_in_Corn_Oil-Rat-Liver-0.25d-up	8/306	0,079639616	0,213492722	0	0	1,796824113	4,546402747	RPL32;RPS6;RPL35A;RPS20;RPSA;RPL10A;SEPP1;RPL39
Aspirin-500_mg/kg_in_Corn_Oil-Rat-Liver-1d-dn	8/306	0,079639616	0,213420081	0	0	1,796824113	4,546402747	EEF1B2;RPS19;RPS8;RPS20;RPSA;RPS3A;RPL10A;GOLGA7
Bithionol-59_mg/kg_in_Corn_Oil-Rat-Bone_marrow-1d-dn	8/306	0,079639616	0,213347489	0	0	1,796824113	4,546402747	RPL32;RPS19;RPS8;FEM1B;RPS6;ARL6IP5;RPS20;RPSA
Butenafine-150_mg/kg_in_Corn_Oil-Rat-Liver-1d-dn	8/306	0,079639616	0,213274946	0	0	1,796824113	4,546402747	EEF1B2;RPS19;RPS8;RPS5;RPL10A;SEPP1;RPL39;TPT1
Diisopropyl_Fluorophosphate-3.75_mg/kg_in_Water-Rat-Heart-5d-up	8/306	0,079639616	0,213202453	0	0	1,796824113	4,546402747	RPS17;RPL32;ARL2BP;RPS8;RPS5;RPL35A;RPS3A;TPT1
Dobutamine-43_mg/kg_in_Saline-Rat-Heart-3d-up	8/306	0,079639616	0,213130009	0	0	1,796824113	4,546402747	RPL32;RPS19;RPS6;RPS20;RPS3A;SEPP1;RPL39;TPT1
Ferulic_Acid-1500_mg/kg_in_CMC-Rat-Liver-3d-up	8/306	0,079639616	0,213057615	0	0	1,796824113	4,546402747	EEF1B2;RPS17;RPL32;RPS19;RPS6;RPS20;RPL10A;SEPP1
Fluvastatin-94_mg/kg_in_Corn_Oil-Rat-Liver-0.25d-dn	8/306	0,079639616	0,212985269	0	0	1,796824113	4,546402747	RPS17;RPL32;RPS8;RPS20;RPSA;RPS3A;RPL39;TPT1
Isotretinoin-125_mg/kg_in_Corn_Oil-Rat-Liver-3d-up	8/306	0,079639616	0,212912973	0	0	1,796824113	4,546402747	C1QA;RPS19;RPS8;RPS5;RPL35A;RPS20;RPSA;TPT1
Nimesulide-30_mg/kg_in_Corn_Oil-Rat-Liver-3d-up	8/306	0,079639616	0,212840725	0	0	1,796824113	4,546402747	EEF1B2;RPL32;RPS19;RPS8;RPS5;RPL35A;RPS20;RPSA
Noscapine-570_mg/kg_in_CMC-Rat-Bone_marrow-5d-up	8/306	0,079639616	0,212768527	0	0	1,796824113	4,546402747	EEF1B2;C1QA;RPL32;RPS6;ARL6IP5;RPS20;RPS3A;RPL39
Pemoline-70_mg/kg_in_CMC-Rat-Liver-1d-up	8/306	0,079639616	0,212696378	0	0	1,796824113	4,546402747	EEF1B2;RPL32;RPS8;RPS6;RPS20;SEPP1;RPL39;TPT1
Prednisone-68_mg/kg_in_CMC-Rat-Heart-1d-dn	8/306	0,079639616	0,212624277	0	0	1,796824113	4,546402747	ARL2BP;RPS8;COL5A3;RPS3A;SEPP1;RPL39;GOLGA7;TPT1
Theophylline-225_mg/kg_in_Water-Rat-Brain-5d-dn	8/306	0,079639616	0,212552225	0	0	1,796824113	4,546402747	RPS17;RPL32;RPS8;FEM1B;RPL35A;RPS3A;RPL39;TOMM20
Theophylline-225_mg/kg_in_Water-Rat-Heart-3d-dn	8/306	0,079639616	0,212480223	0	0	1,796824113	4,546402747	RPS17;RPL32;ARL2BP;COL5A3;RPS6;ARL6IP5;RPS3A;RPL39
Thioguanine-12_mg/kg_in_Corn_Oil-Rat-Liver-3d-dn	8/306	0,079639616	0,212408269	0	0	1,796824113	4,546402747	C1QA;RPS17;RPL32;RPS19;RPS6;RPL35A;RPL10A;SEPP1
44'-Methylenedianiline-296_uM_in_DMSO-Rat-Primary_rat_hepatocytes-1d-dn	10/412	0,079899502	0,213029275	0	0	1,668168018	4,215436659	RPS17;RPL32;RPS19;RPS8;ARL6IP5;RPSA;RPS3A;SEPP1;GOLGA7;TPT1
Rifampin-125_uM_in_DMSO-Rat-Primary_rat_hepatocytes-0.67d-up	9/359	0,08029492	0,214011097	0	0	1,722999167	4,345488188	RPS19;RPS8;RPS6;RPS20;RPSA;RPL10A;RPL39;TPT1;PDRG1
S(-)-Carbidopa-1200_mg/kg_in_CMC-Rat-Brain-1d-dn	9/359	0,08029492	0,213938698	0	0	1,722999167	4,345488188	RPS19;RPS6;ARL6IP5;RPS20;RPSA;SEPP1;RPL39;GOLGA7;TPT1
Dicumarol-13_mg/kg_in_Corn_Oil-Rat-Liver-3d-dn	8/307	0,080797376	0,215204645	0	0	1,790971266	4,50574483	C1QA;RPS17;RPS19;RPS6;RPSA;RPS3A;RPL10A;GOLGA7
Fluconazole-10_mg/kg_in_Corn_Oil-Rat-Liver-3d-dn	8/307	0,080797376	0,215131891	0	0	1,790971266	4,50574483	C1QA;RPL32;RPS19;RPS6;RPL35A;RPL10A;RPL39;TPT1
Glipizide-2500_mg/kg_in_CMC-Rat-Liver-5d-up	8/307	0,080797376	0,215059187	0	0	1,790971266	4,50574483	C1QA;RPS17;RPS19;RPS8;RPS20;RPSA;RPL39;GOLGA7
Isoeugenol-1560_mg/kg_in_CMC-Rat-Kidney-1d-dn	8/307	0,080797376	0,214986532	0	0	1,790971266	4,50574483	RPL32;RPS19;ARL2BP;RPS6;RPS20;RPSA;RPL10A;RPL39
Roxarsone-11_mg/kg_in_Corn_Oil-Rat-Kidney-0.25d-up	8/307	0,080797376	0,214913926	0	0	1,790971266	4,50574483	RPS17;RPS19;RPS6;ARL6IP5;RPS20;RPL10A;SEPP1;RPL39
Sulfadimethoxine-1100_mg/kg_in_CMC-Rat-Kidney-1d-up	8/307	0,080797376	0,214841369	0	0	1,790971266	4,50574483	EEF1B2;RPS17;RPL32;ARL2BP;RPS8;ARL6IP5;RPL35A;RPS20
Suxamethonium_Chloride-0.125_mg/kg_in_Saline-Rat-Heart-1d-up	8/307	0,080797376	0,214768861	0	0	1,790971266	4,50574483	EEF1B2;RPS17;RPL32;RPS19;ARL2BP;RPL10A;RPL39;TPT1
Tiapride-1200_mg/kg_in_Water-Rat-Heart-1d-up	8/307	0,080797376	0,214696402	0	0	1,790971266	4,50574483	RPS17;RPL32;RPS6;ARL6IP5;RPS3A;SEPP1;TPT1;PDRG1
Tinidazole-1200_mg/kg_in_CMC-Rat-Liver-5d-up	8/307	0,080797376	0,214623992	0	0	1,790971266	4,50574483	RPS17;RPL32;RPS19;RPS6;RPS20;RPSA;RPS3A;RPL10A
Trichloroethylene-1230_mg/kg_in_CMC-Rat-Kidney-3d-up	8/307	0,080797376	0,21455163	0	0	1,790971266	4,50574483	EEF1B2;RPS19;RPS8;ARL6IP5;RPSA;RPS3A;RPL10A;TOMM20
Valproic_Acid-1500_mg/kg_in_Water-Rat-Liver-3d-dn	8/307	0,080797376	0,214479317	0	0	1,790971266	4,50574483	EEF1B2;C1QA;RPS17;RPS19;CLP1;RPS20;SEPP1;GOLGA7

Cytarabine-23_mg/kg_in_Saline-Rat-Spleen-0.25d-dn	7/256	0,081206829	0,215493595	0	0	1,879295533	4,718452401	CXCL11;RPS8;FEM1B;FUNDC1;RPS20;RPS3A;TPT1
Methotrexate-0.3_mg/kg_in_Corn_Oil-Rat-Spleen-3d-up	7/256	0,081206829	0,215421013	0	0	1,879295533	4,718452401	EEF1B2;RPL32;RPS8;RPS5;RPS6;RPS20;RPS3A
Lamivudine-35_mg/kg_in_Water-Rat-Liver-0.25d-up	9/360	0,081362614	0,215761599	0	0	1,718213058	4,310720615	C1QA;RPS17;RPL32;RPS6;RPL13A;RPL35A;RPS20;RPSA;SEPP1
Amikacin-82_mg/kg_in_Saline-Rat-Kidney-3d-dn	8/308	0,081965125	0,217286207	0	0	1,785156424	4,465499944	RPS17;RPL32;RPS19;RPS20;RPSA;RPL39;GOLGA7;TPT1
Bithionol-333_mg/kg_in_Corn_Oil-Rat-Liver-1d-up	8/308	0,081965125	0,217213096	0	0	1,785156424	4,465499944	EEF1B2;CXCL11;RPS19;RPS20;SEPP1;RPL39;GOLGA7;TPT1
Carbon_Tetrachloride-775_uM_in_DMSO-Rat-Primary_rat_hepatocytes-0.67d-up	8/308	0,081965125	0,217140034	0	0	1,785156424	4,465499944	EEF1B2;RPS19;RPS6;RPS20;RPS3A;RPL39;TPT1;PDRG1
Citalopram-90_mg/kg_in_Corn_Oil-Rat-Liver-1d-dn	8/308	0,081965125	0,217067021	0	0	1,785156424	4,465499944	EEF1B2;RPS17;RPL32;RPS19;RPS6;RPS20;RPL10A;RPL39
Digitoxin-2.75_mg/kg_in_Corn_Oil-Rat-Heart-1d-up	8/308	0,081965125	0,216994058	0	0	1,785156424	4,465499944	RPS17;RPL32;RPS6;RPS3A;RPL10A;RPL39;TPT1;PDRG1
Furan-24_mg/kg_in_Corn_Oil-Rat-Heart-1d-dn	8/308	0,081965125	0,216921143	0	0	1,785156424	4,465499944	RPL32;RPS19;ARL2BP;RPS8;FUNDC1;RPS6;RPS20;TPT1
Gatifloxacin-770_mg/kg_in_Corn_Oil-Rat-Kidney-1d-dn	8/308	0,081965125	0,216848277	0	0	1,785156424	4,465499944	EEF1B2;RPL32;RPS8;RPL35A;RPL10A;SEPP1;RPL39;TPT1
Genistein-375_mg/kg_in_Corn_Oil-Rat-Liver-0.25d-up	8/308	0,081965125	0,216775461	0	0	1,785156424	4,465499944	EEF1B2;C1QA;RPS19;RPS6;RPS20;RPL10A;SEPP1;GOLGA7
Ifosfamide-143_mg/kg_in_Saline-Rat-Liver-0.25d-dn	8/308	0,081965125	0,216702693	0	0	1,785156424	4,465499944	EEF1B2;RPS19;RPS8;RPS20;RPL10A;SEPP1;GOLGA7;TPT1
Omeprazole-30_mg/kg_in_Corn_Oil-Rat-Liver-1d-dn	8/308	0,081965125	0,216629974	0	0	1,785156424	4,465499944	EEF1B2;C1QA;RPL32;CLP1;RPS8;RPS6;RPL10A;TPT1
Safrole-488_mg/kg_in_Corn_Oil-Rat-Liver-3d-dn	8/308	0,081965125	0,216557304	0	0	1,785156424	4,465499944	EEF1B2;C1QA;RPS17;RPS6;RPS3A;RPL10A;SEPP1;RPL39
Moclobemide-266_mg/kg_in_Water-Rat-Brain-3d-dn	9/361	0,082438895	0,217735995	0	0	1,713453465	4,276262264	EEF1B2;RPL32;RPS19;RPS8;RPS6;RPS20;RPS3A;RPL39;TOMM20
Amikacin-82_mg/kg_in_Saline-Rat-Bone_marrow-1d-up	8/309	0,083142858	0,219521672	0	0	1,779379219	4,425663049	C1QA;RPS17;RPS19;FEM1B;RPS6;RPS20;RPL10A;TPT1
Beta-Estradiol_3-Benzoate-2_mg/kg_in_Corn_Oil-Rat-Liver-1d-up	8/309	0,083142858	0,219448106	0	0	1,779379219	4,425663049	RPS19;RPS6;RPS20;RPSA;RPS3A;RPL10A;SEPP1;RPL39
Chlorambucil-4.5_mg/kg_in_Corn_Oil-Rat-Liver-0.25d-up	8/309	0,083142858	0,219374589	0	0	1,779379219	4,425663049	EEF1B2;RPS17;RPS19;RPS6;RPSA;RPL10A;SEPP1;RPL39
Chlorobenzene-500_mg/kg_in_CMC-Rat-Kidney-5d-up	8/309	0,083142858	0,219301121	0	0	1,779379219	4,425663049	EEF1B2;RPS17;RPS8;ARL6IP5;RPS20;RPSA;RPS3A;TPT1
Cisplatin-2_mg/kg_in_Saline-Rat-Liver-1d-up	8/309	0,083142858	0,219227703	0	0	1,779379219	4,425663049	EEF1B2;C1QA;RPS17;RPS5;RPSA;RPL10A;SEPP1;RPL39
Clotrimazole-89_mg/kg_in_Corn_Oil-Rat-Liver-0.25d-up	8/309	0,083142858	0,219154333	0	0	1,779379219	4,425663049	RPS17;RPL32;RPS19;RPS8;RPS6;RPS20;RPSA;RPS3A
Cyproheptadine-37_mg/kg_in_CMC-Rat-Heart-5d-up	8/309	0,083142858	0,219081013	0	0	1,779379219	4,425663049	RPS17;RPL32;RPS19;FUNDC1;RPS20;RPSA;RPL10A;TPT1
Etodolac-24_mg/kg_in_CMC-Rat-Liver-1d-dn	8/309	0,083142858	0,219007742	0	0	1,779379219	4,425663049	EEF1B2;RPS17;RPS8;ARL6IP5;RPL35A;RPS20;SEPP1;TPT1
Isoprenaline-15_mg/kg_in_Saline-Rat-Heart-3d-dn	8/309	0,083142858	0,218934519	0	0	1,779379219	4,425663049	RPS17;RPL32;RPS19;RPS8;COL5A3;RPS6;RPS3A;TPT1
Ketoconazole-114_mg/kg_in_Corn_Oil-Rat-Liver-5d-dn	8/309	0,083142858	0,218861346	0	0	1,779379219	4,425663049	EEF1B2;C1QA;RPS17;RPS19;RPS6;RPSA;RPL10A;RPL39
Mitoxantrone-2_mg/kg_in_Saline-Rat-Heart-3d-dn	8/309	0,083142858	0,218788222	0	0	1,779379219	4,425663049	EEF1B2;RPL32;RPS5;FUNDC1;RPS6;RPSA;RPS3A;SEPP1
Myrtecaine-140_mg/kg_in_Corn_Oil-Rat-Heart-3d-up	8/309	0,083142858	0,218715146	0	0	1,779379219	4,425663049	RPS19;FUNDC1;RPS6;RPS20;RAPGEF5;RPL10A;SEPP1;RPL39
Pergolide-1.1_mg/kg_in_CMC-Rat-Liver-3d-up	8/309	0,083142858	0,218642119	0	0	1,779379219	4,425663049	C1QA;RPS17;RPL32;RPS5;RPL35A;RPSA;RPS3A;RPL39
Piclamilast-17_mg/kg_in_CMC-Rat-Heart-1d-up	8/309	0,083142858	0,218569141	0	0	1,779379219	4,425663049	EEF1B2;ARL2BP;RPS8;FUNDC1;ARL6IP5;RPS20;TOMM20;TPT1
Thioguanine-12_mg/kg_in_Corn_Oil-Rat-Bone_marrow-3d-up	8/309	0,083142858	0,218496212	0	0	1,779379219	4,425663049	EEF1B2;C1QA;RPS17;RPL32;RPS8;RPS5;RPL35A;SEPP1
Venlafaxine-320_mg/kg_in_Water-Rat-Liver-5d-up	8/309	0,083142858	0,218423331	0	0	1,779379219	4,425663049	RPS17;RPS19;RPS6;RPS20;RPSA;RAPGEF5;RPL10A;RPL39
Chlorobenzene-500_mg/kg_in_CMC-Rat-Heart-3d-up	9/362	0,083523763	0,219350835	0	0	1,708720169	4,242109881	EEF1B2;RPS17;RPL32;RPS8;RPS5;RPL35A;RPS3A;RAPGEF5;TOMM20
3-Methylcholanthrene-23_uM_in_DMSO-Rat-Primary_rat_hepatocytes-1d-up	7/258	0,083808448	0,220025111	0	0	1,86472735	4,623072081	RPS17;CXCL11;RPS19;RPS20;GOLGA7;TPT1;PDRG1
Piroxicam-14_mg/kg_in_CMC-Rat-Kidney-5d-dn	7/258	0,083808448	0,219951794	0	0	1,86472735	4,623072081	RPS17;RPS8;RPS6;RPS3A;RPL10A;TOMM20;TPT1
Neomycin-56_mg/kg_in_Corn_Oil-Rat-Kidney-1d-dn	6/208	0,084056207	0,220528543	0	0	1,982553529	4,909336984	EEF1B2;ARL2BP;CLP1;RPL10A;SEPP1;TPT1
2-Acetylaminofluorene-30_mg/kg_in_CMC-Rat-Kidney-1d-dn	8/310	0,084330571	0,221174683	0	0	1,773639286	4,386229181	EEF1B2;RPS19;RPS6;RPS20;RPSA;RPL10A;RPL39;GOLGA7
4-Nonylphenol-200_mg/kg_in_Corn_Oil-Rat-Liver-3d-up	8/310	0,084330571	0,221101056	0	0	1,773639286	4,386229181	RPS17;RPL32;RPS19;RPS5;RPS6;RPS20;RPSA;SEPP1
Catechol-195_mg/kg_in_Saline-Rat-Bone_marrow-5d-dn	8/310	0,084330571	0,221027479	0	0	1,773639286	4,386229181	EEF1B2;RPS6;RPS20;RPSA;RPS3A;RPL10A;RPL39;DUSP14
Clofibrate-500_mg/kg_in_Corn_Oil-Rat-Liver-1d-dn	8/310	0,084330571	0,22095395	0	0	1,773639286	4,386229181	EEF1B2;C1QA;RPS17;RPS19;RPS8;RPSA;RPL10A;SEPP1
Dexibuprofen-239_mg/kg_in_CMC-Rat-Kidney-1d-up	8/310	0,084330571	0,22088047	0	0	1,773639286	4,386229181	RPS17;CXCL11;RPS19;ARL2BP;FUNDC1;RPSA;RPL10A;RPL39
Doxofylline-483_mg/kg_in_Water-Rat-Heart-3d-dn	8/310	0,084330571	0,220807039	0	0	1,773639286	4,386229181	EEF1B2;RPS17;RPL32;RPS8;RPL35A;RPS3A;RPL39;TPT1
Methimazole-100_mg/kg_in_Water-Rat-Liver-3d-dn	8/310	0,084330571	0,220733657	0	0	1,773639286	4,386229181	C1QA;RPS17;RPL32;RPS8;FEM1B;RPL35A;RPS3A;TPT1
NN'-Diphenyl-P-Phenylenediamine-1000_mg/kg_in_CMC-Rat-Liver-1d-up	8/310	0,084330571	0,220660323	0	0	1,773639286	4,386229181	EEF1B2;RPL32;RPS8;RPSA;RPL10A;SEPP1;GOLGA7;TPT1
NN-Dimethylformamide-700_mg/kg_in_Saline-Rat-Liver-1d-dn	8/310	0,084330571	0,220587039	0	0	1,773639286	4,386229181	EEF1B2;RPS17;RPL32;RPS8;RPSA;RPL10A;SEPP1;RPL39
Sparteine-225_mg/kg_in_Water-Rat-Heart-3d-up	8/310	0,084330571	0,220513803	0	0	1,773639286	4,386229181	RPS17;RPL32;RPS6;RPL35A;RPS3A;SEPP1;RPL39;GOLGA7
Ifosfamide-11000_uM_in_DMSO-Rat-Primary_rat_hepatocytes-1d-dn	9/363	0,084617216	0,221189909	0	0	1,70401295	4,208260253	RPS17;RPL32;RPS19;ARL2BP;RPS8;RPS20;RPL39;TOMM20;TPT1
Hydroxyurea-59_mg/kg_in_Saline-Rat-Bone_marrow-3d-dn	7/259	0,085127065	0,222448826	0	0	1,857527631	4,576224131	RPS19;ARL2BP;RPS8;FUNDC1;RPS6;RPSA;TPT1
4-Nonylphenol-200_mg/kg_in_Corn_Oil-Rat-Liver-1d-up	8/311	0,085528257	0,223423069	0	0	1,767936266	4,347193449	RPS19;RPS8;RPS5;RPS6;RPSA;RPS3A;SEPP1;RPL39
Altretamine-13_mg/kg_in_Corn_Oil-Rat-Bone_marrow-0.25d-up	8/311	0,085528257	0,22334899	0	0	1,767936266	4,347193449	EEF1B2;C1QA;NKG7;RPL35A;RPS20;RPL10A;SEPP1;RPL39
Chlorpyrifos-82_mg/kg_in_CMC-Rat-Heart-1d-dn	8/311	0,085528257	0,22327496	0	0	1,767936266	4,347193449	RPL32;RPS19;COL5A3;ARL6IP5;RPS20;RPL10A;RPL39;TPT1
Ciprofloxacin-450_mg/kg_in_Corn_Oil-Rat-Liver-3d-dn	8/311	0,085528257	0,223200979	0	0	1,767936266	4,347193449	EEF1B2;RPS17;RPL32;RPS19;RPS8;RPS6;RPS3A;RPL10A
Fluphenazine-22_mg/kg_in_Corn_Oil-Rat-Heart-3d-up	8/311	0,085528257	0,223127046	0	0	1,767936266	4,347193449	RPS8;RPS6;ARL6IP5;RPSA;RPS3A;RPL10A;SEPP1;TPT1
Idarubicin-4_mg/kg_in_Saline-Rat-Kidney-5d-up	8/311	0,085528257	0,223053163	0	0	1,767936266	4,347193449	EEF1B2;CLP1;RPS8;RPS20;RPSA;RPL10A;SEPP1;TPT1
Lansoprazole-600_mg/kg_in_CMC-Rat-Liver-1d-dn	8/311	0,085528257	0,222979329	0	0	1,767936266	4,347193449	EEF1B2;C1QA;RPS19;CLP1;RPS6;RPS20;SEPP1;TPT1
Mevastatin-1200_mg/kg_in_CMC-Rat-Kidney-1d-dn	8/311	0,085528257	0,222905544	0	0	1,767936266	4,347193449	RPL32;ARL2BP;RPS8;RPS6;RPL10A;RPL39;TOMM20;TPT1
Mianserin-116_mg/kg_in_Corn_Oil-Rat-Heart-3d-dn	8/311	0,085528257	0,222831807	0	0	1,767936266	4,347193449	RPS19;ARL2BP;RPS6;RPS3A;RPL10A;SEPP1;RPL39;GOLGA7
Oxyquinoline-68_mg/kg_in_Corn_Oil-Rat-Liver-1d-up	8/311	0,085528257	0,222758119	0	0	1,767936266	4,347193449	C1QA;RPS17;RPS5;RPS6;RPSA;RPS3A;RPL39;TPT1
Promazine-100_mg/kg_in_Corn_Oil-Rat-Liver-5d-dn	8/311	0,085528257	0,22268448	0	0	1,767936266	4,347193449	EEF1B2;RPL32;RPS19;CLP1;RPS6;RPSA;RPS3A;RPL10A
Roxithromycin-312_mg/kg_in_CMC-Rat-Liver-3d-up	8/311	0,085528257	0,22261089	0	0	1,767936266	4,347193449	C1QA;RPS17;RPL32;RPSA;RPS3A;RPL10A;RPL39;TPT1
Tamoxifen-2.5_mg/kg_in_Corn_Oil-Rat-Liver-1d-up	8/311	0,085528257	0,222537348	0	0	1,767936266	4,347193449	EEF1B2;RPL32;RPS8;RPS6;RPS20;RPS3A;RPL10A;SEPP1
Dimethyl_Methylphosphonate-1500_mg/kg_in_Water-Rat-Kidney-1d-dn	6/209	0,085545116	0,222507705	0	0	1,973067627	4,851203814	RPS17;RPL32;RPS19;RPS6;RPL10A;RPL39
Methapyrilene-300_uM_in_DMSO-Rat-Primary_rat_hepatocytes-1d-dn	9/364	0,085719255	0,222887042	0	0	1,699331596	4,174710208	CXCL11;RPS19;ARL2BP;FUNDC1;RPS6;RPS20;SEPP1;RPL39;TPT1
Stavudine-1120_uM_in_DMSO-Rat-Primary_rat_hepatocytes-1d-dn	9/364	0,085719255	0,222813482	0	0	1,699331596	4,174710208	CXCL11;RPS19;RPS20;RPSA;RPS3A;RPL10A;SEPP1;RPL39;TPT1
6-Mercaptopurine-25_mg/kg_in_Corn_Oil-Rat-Liver-1d-dn	7/260	0,086457533	0,224658374	0	0	1,850383294	4,529926913	EEF1B2;C1QA;RPL32;RPS8;RPS6;ARL6IP5;RPS3A
Amantadine-1500_uM_in_DMSO-Rat-Primary_rat_hepatocytes-1d-up	7/260	0,086457533	0,224584278	0	0	1,850383294	4,529926913	EEF1B2;ARL2BP;RPS8;RPS5;RPS20;RPS3A;PDRG1
Cisplatin-1.17_mg/kg_in_Saline-Rat-Bone_marrow-1d-dn	7/260	0,086457533	0,224510231	0	0	1,850383294	4,529926913	RPL32;RPS5;RPS6;RPSA;RPS3A;RPL10A;RPL39
Enoxacin-750_mg/kg_in_Corn_Oil-Rat-Liver-0.25d-dn	7/260	0,086457533	0,224436233	0	0	1,850383294	4,529926913	RPS17;RPL32;RPS8;RPSA;RPS3A;RPL39;TPT1
Fluocinolone_Acetonide-2.5_mg/kg_in_Corn_Oil-Rat-Kidney-5d-dn	7/260	0,086457533	0,224362284	0	0	1,850383294	4,529926913	RPS17;RPL32;RPS19;ARL2BP;RPSA;RPS3A;RPL39
D-Tubocurarine_Chloride-0.033_mg/kg_in_Saline-Rat-Heart-5d-up	8/312	0,086735911	0,225010551	0	0	1,762269804	4,308551036	RPS17;RPL32;RPS19;RPS6;RPS20;RPSA;RPL10A;RPL39
Indinavir-1000_mg/kg_in_CMC-Rat-Kidney-3d-up	8/312	0,086735911	0,224936461	0	0	1,762269804	4,308551036	EEF1B2;RPS17;CXCL11;ARL6IP5;RPS20;RPSA;RAPGEF5;SEPP1
Indomethacin-4.5_mg/kg_in_Water-Rat-Liver-5d-up	8/312	0,086735911	0,22486242	0	0	1,762269804	4,308551036	EEF1B2;RPS17;RPL32;RPS20;RPS3A;RPL10A;RPL39;TPT1
Ketoconazole-90_uM_in_DMSO-Rat-Primary_rat_hepatocytes-0.67d-up	8/312	0,086735911	0,224788428	0	0	1,762269804	4,308551036	RPL32;RPS19;RPS6;RPS20;RPSA;RPS3A;RPL10A;PDRG1
Mefenamic_Acid-93_mg/kg_in_Corn_Oil-Rat-Kidney-1d-dn	8/312	0,086735911	0,224714485	0	0	1,762269804	4,308551036	RPS19;RPS6;ARL6IP5;RPSA;RPL10A;SEPP1;RPL39;PDRG1
Minoxidil-83_mg/kg_in_CMC-Rat-Heart-5d-up	8/312	0,086735911	0,22464059	0	0	1,762269804	4,308551036	RPL32;RPS19;RPS6;RPS20;RPSA;RPL10A;SEPP1;RPL39
Propylthiouracil-625_mg/kg_in_CMC-Rat-Liver-5d-dn	8/312	0,086735911	0,224566743	0	0	1,762269804	4,308551036	EEF1B2;C1QA;RPL32;RPS19;RPS6;RPS3A;RPL39;TPT1
Rofecoxib-800_mg/kg_in_Corn_Oil-Rat-Liver-1d-dn	8/312	0,086735911	0,224492946	0	0	1,762269804	4,308551036	EEF1B2;RPS19;ARL6IP5;RPS20;RPS3A;RPL10A;RPL39;GOLGA7
Valproic_Acid-850_mg/kg_in_Water-Rat-Liver-1d-up	8/312	0,086735911	0,224419196	0	0	1,762269804	4,308551036	RPS17;RPL32;RPS19;RPS6;RPL35A;RPSA;RPS3A;RPL10A
Sodium_Arsenite-30_mg/kg_in_Saline-Rat-Bone_marrow-1d-dn	7/261	0,087799837	0,227097377	0	0	1,843293703	4,48417255	RPS17;RPSA;RPS3A;RPL10A;RPL39;SEPP1;TPT1
Probenecid-600_mg/kg_in_Corn_Oil-Rat-Kidney-1d-up	9/366	0,087949076	0,227408709	0	0	1,690045631	4,10849638	RPS17;RPL32;RPS19;RPS5;RPS6;RPL35A;RPSA;RPL10A;RPL39
Albendazole-62_mg/kg_in_Corn_Oil-Rat-Liver-3d-up	8/313	0,087953524	0,22734557	0	0	1,756639549	4,270297194	C1QA;RPS8;RPS5;RPSA;RPS3A;RAPGEF5;TOMM20;TPT1
Bisphenol_A-610_mg/kg_in_Corn_Oil-Rat-Bone_marrow-1d-up	8/313	0,087953524	0,227270981	0	0	1,756639549	4,270297194	C1QA;RPS17;RPS19;RPS6;RPS20;RPS3A;SEPP1;RPL39
Citalopram-150_uM_in_DMSO-Rat-Primary_rat_hepatocytes-0.67d-dn	8/313	0,087953524	0,227196442	0	0	1,756639549	4,270297194	EEF1B2;RPS17;CXCL11;RPL32;RPS8;ARL6IP5;SEPP1;SLC16A3
Cobalt_(II)_Chloride-10_mg/kg_in_Saline-Rat-Kidney-5d-up	8/313	0,087953524	0,227121951	0	0	1,756639549	4,270297194	CXCL11;RPS8;FUNDC1;RPS6;RPS3A;RPL10A;SEPP1;TPT1
Cyclosporin_A-350_mg/kg_in_Corn_Oil-Rat-Heart-3d-up	8/313	0,087953524	0,227047509	0	0	1,756639549	4,270297194	C1QA;CXCL11;RPS8;FUNDC1;RPS20;SEPP1;RPL39;TPT1
Fluvastatin-94_mg/kg_in_Corn_Oil-Rat-Liver-1d-dn	8/313	0,087953524	0,226973116	0	0	1,756639549	4,270297194	EEF1B2;RPS17;RPL32;RPS8;RPS6;RPSA;RPL39;TPT1
Gallamine_Triethiodide-2.5_mg/kg_in_Saline-Rat-Heart-3d-up	8/313	0,087953524	0,226898772	0	0	1,756639549	4,270297194	EEF1B2;RPS17;RPL32;RPS19;RPS8;FUNDC1;RPL35A;RPS20
Olanzapine-23_mg/kg_in_CMC-Rat-Heart-1d-up	8/313	0,087953524	0,226824477	0	0	1,756639549	4,270297194	EEF1B2;CXCL11;RPS8;FUNDC1;ARL6IP5;RPL10A;SEPP1;TPT1
Tacrine-24_mg/kg_in_Water-Rat-Liver-1d-dn	8/313	0,087953524	0,22675023	0	0	1,756639549	4,270297194	RPS17;RPL32;RPS8;RPSA;RPS3A;RPL10A;RPL39;TPT1
Thioguanine-24_mg/kg_in_Corn_Oil-Rat-Bone_marrow-1d-up	8/313	0,087953524	0,226676031	0	0	1,756639549	4,270297194	C1QA;RPS17;NKG7;RPS20;RPL10A;SEPP1;RPL39;TPT1
Ethinylestradiol-190_uM_in_DMSO-Rat-Primary_rat_hepatocytes-0.67d-dn	6/211	0,088566476	0,228181081	0	0	1,954365564	4,737385778	RPS17;RPL32;ARL6IP5;RPSA;RPS3A;SLC16A3

Fenofibrate-215_mg/kg_in_Corn_Oil-Rat-Kidney-3d-up	7/262	0,089153957	0,229619543	0	0	1,83625823	4,438953301	RPS8;RPS6;RPL35A;RPS20;RPS3A;GOLGA7;TPT1
Camptothecin-2.5_mg/kg_in_Saline-Rat-Heart-3d-dn	8/314	0,089181087	0,229614331	0	0	1,751045155	4,232427245	EEF1B2;RPS17;RPL32;RPS19;RPS6;ARL6IP5;RPS20;RPSA
Carboplatin-14_mg/kg_in_Saline-Rat-Spleen-5d-dn	8/314	0,089181087	0,229539294	0	0	1,751045155	4,232427245	EEF1B2;C1QA;RPS17;RPS8;RPS5;RPS20;RPSA;TPT1
Fluphenazine-22_mg/kg_in_Corn_Oil-Rat-Liver-3d-up	8/314	0,089181087	0,229464305	0	0	1,751045155	4,232427245	EEF1B2;RPL32;RPS19;RPS8;RPS5;RPS20;RPS3A;SEPP1
Terbinafine-2000_mg/kg_in_CMC-Rat-Liver-1d-dn	8/314	0,089181087	0,229389366	0	0	1,751045155	4,232427245	EEF1B2;C1QA;RPS19;CLP1;ARL6IP5;RPS20;RPL10A;SEPP1
Theophylline-225_mg/kg_in_Water-Rat-Brain-3d-dn	8/314	0,089181087	0,229314476	0	0	1,751045155	4,232427245	RPL32;FEM1B;RPS6;RPS20;RPSA;RPL10A;RPL39;TOMM20
Sporidesmin_A-0.15_uM_in_DMSO-Rat-Primary_rat_hepatocytes-1d-up	6/212	0,090098852	0,231598747	0	0	1,945146859	4,681672541	EEF1B2;CXCL11;COL5A3;ARL6IP5;GOLGA7;SEPP1
Acetaminophen-100_mg/kg_in_Corn_Oil-Rat-Liver-0.25d-up	8/315	0,09041859	0,232344802	0	0	1,745486282	4,194936581	EEF1B2;RPL32;RPS19;RPS6;RPS20;RPS3A;RPL10A;SEPP1
Azauridine-1500_mg/kg_in_CMC-Rat-Kidney-5d-up	8/315	0,09041859	0,232269021	0	0	1,745486282	4,194936581	EEF1B2;RPS17;CLP1;RPS8;ARL6IP5;RPSA;RPS3A;TPT1
Chlorambucil-4.5_mg/kg_in_Corn_Oil-Rat-Heart-3d-up	8/315	0,09041859	0,232193289	0	0	1,745486282	4,194936581	RPS19;ARL6IP5;HOXA1;RPSA;RAPGEF5;RPL10A;RPL39;PDRG1
Doxorubicin-0.65_mg/kg_in_Saline-Rat-Spleen-1d-dn	8/315	0,09041859	0,232117607	0	0	1,745486282	4,194936581	EEF1B2;RPL32;RPS8;RPS5;FUNDC1;RPSA;RPS3A;TPT1
Flavoxate-1255_mg/kg_in_CMC-Rat-Heart-3d-dn	8/315	0,09041859	0,232041974	0	0	1,745486282	4,194936581	EEF1B2;ARL2BP;RPS8;COL5A3;FUNDC1;RPS20;RPL10A;RPL39
Fluoxetine-52_mg/kg_in_CMC-Rat-Kidney-1d-dn	8/315	0,09041859	0,23196639	0	0	1,745486282	4,194936581	EEF1B2;RPS17;RPL32;ARL2BP;RPS8;RPS20;RPL39;TOMM20
Melphalan-12_mg/kg_in_CMC-Rat-Spleen-1d-up	8/315	0,09041859	0,231890856	0	0	1,745486282	4,194936581	RPS19;RPS8;RPS5;RPL35A;RPSA;RPL10A;SEPP1;RPL39
Mosapride-30_mg/kg_in_CMC-Rat-Intestine-3d-dn	8/315	0,09041859	0,231815371	0	0	1,745486282	4,194936581	EEF1B2;RPS19;RPS8;ARL6IP5;RPSA;RPL10A;SEPP1;SLC16A3
N-Nitrosodiethylamine-34_mg/kg_in_Saline-Rat-Liver-5d-up	8/315	0,09041859	0,231739934	0	0	1,745486282	4,194936581	EEF1B2;C1QA;RPS17;RPS19;RPS6;RPS3A;RPL39;TPT1
Rifabutin-1500_mg/kg_in_CMC-Rat-Kidney-3d-up	8/315	0,09041859	0,231664547	0	0	1,745486282	4,194936581	RPS17;CXCL11;RPS8;RPS6;RPS20;RPS3A;RAPGEF5;TPT1
Sulfinpyrazone-269_mg/kg_in_CMC-Rat-Kidney-3d-up	8/315	0,09041859	0,231589209	0	0	1,745486282	4,194936581	CXCL11;RPS19;RPS6;RPSA;RAPGEF5;RPL10A;GOLGA7;TPT1
Terbutaline-130_mg/kg_in_Corn_Oil-Rat-Heart-5d-dn	8/315	0,09041859	0,23151392	0	0	1,745486282	4,194936581	EEF1B2;RPS17;RPL32;ARL2BP;RPS8;COL5A3;RPS6;RPL35A
Zidovudine-1540_mg/kg_in_Corn_Oil-Rat-Heart-5d-up	8/315	0,09041859	0,23143868	0	0	1,745486282	4,194936581	CLP1;RPS8;FUNDC1;RPS6;RPS20;SEPP1;TOMM20;TPT1
24-Diaminophenol-60_mg/kg_in_Water-Rat-Kidney-5d-dn	7/263	0,090519873	0,231622652	0	0	1,82927626	4,39426156	RPS17;RPL32;RPS6;RPS20;RPSA;RPL39;SEPP1
Abamectin-10_mg/kg_in_Water-Rat-Liver-3d-up	7/263	0,090519873	0,231547425	0	0	1,82927626	4,39426156	RPS17;RPS19;RPS8;RPS5;ARL6IP5;RPS3A;SEPP1
Pentamidine-18_mg/kg_in_Saline-Rat-Bone_marrow-1d-up	7/263	0,090519873	0,231472248	0	0	1,82927626	4,39426156	RPS19;RPS8;NKG7;IL12A;RPL10A;SEPP1;TPT1
4-Chloro-2-Nitroaniline-1200_mg/kg_in_CMC-Rat-Kidney-3d-up	8/316	0,091666024	0,234327039	0	0	1,739962591	4,157820661	EEF1B2;RPS17;CXCL11;RPL32;RPS8;RPS20;RPS3A;RAPGEF5
Altretamine-13_mg/kg_in_Corn_Oil-Rat-Liver-5d-up	8/316	0,091666024	0,234251008	0	0	1,739962591	4,157820661	RPL32;RPS8;RPL35A;RPS20;RPL10A;RPL39;GOLGA7;TPT1
Carboplatin-6_mg/kg_in_Saline-Rat-Liver-3d-dn	8/316	0,091666024	0,234175026	0	0	1,739962591	4,157820661	EEF1B2;RPS17;RPL32;RPS8;RPSA;RPS3A;SEPP1;TPT1
Carmofur-201_mg/kg_in_CMC-Rat-Bone_marrow-1d-dn	8/316	0,091666024	0,234099094	0	0	1,739962591	4,157820661	EEF1B2;C1QA;RPS17;RPL32;RPS8;RPS20;RPS3A;RPL39
Catechol-40_mg/kg_in_Saline-Rat-Bone_marrow-3d-dn	8/316	0,091666024	0,234023211	0	0	1,739962591	4,157820661	EEF1B2;RPL32;ARL2BP;RPS6;NKG7;RPS20;RPL39;TPT1
Daunorubicin-4_mg/kg_in_Saline-Rat-Kidney-5d-up	8/316	0,091666024	0,233947377	0	0	1,739962591	4,157820661	EEF1B2;RPS19;ARL2BP;CLP1;RPS8;RPS20;RPL10A;TPT1
Dipyridamole-750_mg/kg_in_CMC-Rat-Liver-5d-up	8/316	0,091666024	0,233871593	0	0	1,739962591	4,157820661	EEF1B2;RPS17;RPL32;RPS8;RPL35A;RPS20;RPSA;SEPP1
Ethinylestradiol-1480_mg/kg_in_Corn_Oil-Rat-Liver-3d-dn	8/316	0,091666024	0,233795857	0	0	1,739962591	4,157820661	EEF1B2;C1QA;RPL32;RPL35A;RPL10A;SEPP1;RPL39;TPT1
Ethylene_Glycol-3525_mg/kg_in_Water-Rat-Liver-5d-dn	8/316	0,091666024	0,23372017	0	0	1,739962591	4,157820661	C1QA;RPL32;RPS6;RPSA;RPS3A;RPL10A;SEPP1;TPT1
Itraconazole-10_uM_in_DMSO-Rat-Primary_rat_hepatocytes-1d-dn	8/316	0,091666024	0,233644533	0	0	1,739962591	4,157820661	C1QA;CXCL11;RPS19;ARL6IP5;SEPP1;RPL39;SLC16A3;TPT1
Mebendazole-50_mg/kg_in_Corn_Oil-Rat-Liver-1d-up	8/316	0,091666024	0,233568944	0	0	1,739962591	4,157820661	C1QA;RPS17;RPS8;RPL35A;RPS20;RPSA;SEPP1;TOMM20
Metronidazole-50_mg/kg_in_Water-Rat-Liver-1d-up	8/316	0,091666024	0,233493404	0	0	1,739962591	4,157820661	CXCL11;RPL32;RPS19;RPS8;RPS5;RPS6;RPSA;TPT1
Mexiletine-350_mg/kg_in_CMC-Rat-Heart-5d-up	8/316	0,091666024	0,233417913	0	0	1,739962591	4,157820661	RPL32;RPS19;ARL2BP;RPS6;RPS20;RPSA;RPL10A;SEPP1
Nevirapine-200_mg/kg_in_Saline-Rat-Liver-3d-dn	8/316	0,091666024	0,233342471	0	0	1,739962591	4,157820661	EEF1B2;C1QA;RPL32;RPS19;RPS8;RPS6;SEPP1;TPT1
Omeprazole-30_mg/kg_in_Corn_Oil-Rat-Liver-5d-up	8/316	0,091666024	0,233267078	0	0	1,739962591	4,157820661	EEF1B2;RPS17;RPL32;RPS19;RPS8;RPS20;RPS3A;TPT1
Simvastatin-15_mg/kg_in_Corn_Oil-Rat-Kidney-1d-up	8/316	0,091666024	0,233191733	0	0	1,739962591	4,157820661	RPS17;RPS8;RPS5;RPL35A;RPS20;RPS3A;RAPGEF5;TPT1
Simvastatin-15_mg/kg_in_Corn_Oil-Rat-Liver-1d-up	8/316	0,091666024	0,233116437	0	0	1,739962591	4,157820661	EEF1B2;RPS17;RPL32;RPS6;RPS20;RPS3A;RPL10A;RPL39
Tropisetron-100_mg/kg_in_CMC-Rat-Liver-5d-up	8/316	0,091666024	0,23304119	0	0	1,739962591	4,157820661	EEF1B2;RPL32;RPS19;RPS6;RPS20;RPSA;RPL10A;RPL39
Thioguanine-12_mg/kg_in_Corn_Oil-Rat-Spleen-3d-up	7/264	0,091897566	0,233554446	0	0	1,822347183	4,350089855	EEF1B2;RPL32;RPS8;RPS5;RPS6;RAPGEF5;SEPP1
5-Fluoro-2'-Deoxyuridine-54_mg/kg_in_Saline-Rat-Bone_marrow-1d-up	8/317	0,092923376	0,236085326	0	0	1,73447375	4,121075008	C1QA;RPS17;RPS19;ARL2BP;RPS6;RPS20;RPSA;SEPP1
Alfacalcidol-0.043_mg/kg_in_CMC-Rat-Heart-1d-dn	8/317	0,092923376	0,236009194	0	0	1,73447375	4,121075008	RPL32;RPS19;RPS8;FEM1B;RPS6;RPS3A;SEPP1;TPT1
Azasetron-580_mg/kg_in_CMC-Rat-Intestine-3d-dn	8/317	0,092923376	0,235933111	0	0	1,73447375	4,121075008	C1QA;RPS17;RPS8;RPSA;RPS3A;SEPP1;RPL39;TPT1
Bithionol-59_mg/kg_in_Corn_Oil-Rat-Kidney-0.25d-up	8/317	0,092923376	0,235857077	0	0	1,73447375	4,121075008	EEF1B2;RPS5;RPL35A;RPS20;RPS3A;SEPP1;TOMM20;TPT1
Carboplatin-6_mg/kg_in_Saline-Rat-Spleen-1d-dn	8/317	0,092923376	0,235781092	0	0	1,73447375	4,121075008	EEF1B2;RPS17;RPL32;RPS8;RPS5;RPL35A;RPSA;RPS3A
Doxepin-132_uM_in_DMSO-Rat-Primary_rat_hepatocytes-0.67d-dn	8/317	0,092923376	0,235705156	0	0	1,73447375	4,121075008	EEF1B2;CXCL11;RPL32;ARL6IP5;RPL35A;SEPP1;SLC16A3;TPT1
Gold_Sodium_Thiomalate-8_mg/kg_in_Saline-Rat-Kidney-5d-up	8/317	0,092923376	0,235629269	0	0	1,73447375	4,121075008	C1QA;CXCL11;RPS6;RPS20;RPSA;RPL10A;RPL39;TPT1
Lomustine-4.2_mg/kg_in_Corn_Oil-Rat-Bone_marrow-0.25d-dn	8/317	0,092923376	0,235553431	0	0	1,73447375	4,121075008	RPS19;RPS8;RPS5;RPS6;ARL6IP5;NKG7;RPSA;SEPP1
Monocrotaline-333_uM_in_DMSO-Rat-Primary_rat_hepatocytes-1d-dn	8/317	0,092923376	0,235477641	0	0	1,73447375	4,121075008	RPS19;RPS8;ARL6IP5;RPS20;RPL10A;SEPP1;RPL39;TPT1
Nimesulide-30_mg/kg_in_Corn_Oil-Rat-Kidney-0.25d-up	8/317	0,092923376	0,235401901	0	0	1,73447375	4,121075008	EEF1B2;RPS17;RPS19;RPS20;RPS3A;SEPP1;RPL39;GOLGA7
Prazosin-2000_mg/kg_in_CMC-Rat-Heart-3d-up	8/317	0,092923376	0,235326209	0	0	1,73447375	4,121075008	EEF1B2;RPS19;RPS6;RPSA;RAPGEF5;RPL10A;SEPP1;RPL39
Valproic_Acid-235_mg/kg_in_Water-Rat-Liver-3d-dn	6/214	0,093206794	0,235968083	0	0	1,926967916	4,572568967	RPS17;RPL32;RPL35A;RPS3A;RPL39;TPT1
Aspirin-167_mg/kg_in_Corn_Oil-Rat-Liver-1d-up	7/265	0,093287012	0,236095278	0	0	1,815470401	4,306430841	C1QA;RPS17;RPS8;RPS6;RPL35A;SEPP1;TPT1
Balsalazide-1100_mg/kg_in_Water-Rat-Liver-0.25d-dn	7/265	0,093287012	0,236019437	0	0	1,815470401	4,306430841	EEF1B2;RPS17;RPS8;ARL6IP5;RPL35A;RPL39;TPT1
Meloxicam-33_mg/kg_in_Corn_Oil-Rat-Kidney-3d-up	7/265	0,093287012	0,235943644	0	0	1,815470401	4,306430841	C1QA;CXCL11;RPS8;RPL35A;RPS3A;RAPGEF5;TPT1
Olanzapine-23_mg/kg_in_CMC-Rat-Liver-3d-dn	7/265	0,093287012	0,235867899	0	0	1,815470401	4,306430841	C1QA;ARL2BP;CLP1;RPS8;RPS6;RPS20;SEPP1
Trihexyphenidyl-588_mg/kg_in_CMC-Rat-Heart-5d-up	7/265	0,093287012	0,235792204	0	0	1,815470401	4,306430841	RPL32;ARL2BP;RPS8;FUNDC1;RPS6;RPS20;RAPGEF5
Valacyclovir-88_mg/kg_in_CMC-Rat-Kidney-5d-dn	7/265	0,093287012	0,235716556	0	0	1,815470401	4,306430841	EEF1B2;ARL2BP;RPS8;RPS5;RPL35A;RPS3A;RPL10A
Thiamphenicol-1500_mg/kg_in_Water-Rat-Kidney-1d-up	9/371	0,093673622	0,236617526	0	0	1,667268736	3,94799007	EEF1B2;RPS17;RPL32;RPS19;RPS6;RPS3A;RPL10A;TPT1;PDRG1
Amikacin-20_mg/kg_in_Saline-Rat-Kidney-3d-dn	8/318	0,094190633	0,237847204	0	0	1,72901943	4,084695212	EEF1B2;RPS17;RPS19;CLP1;RPSA;RPS3A;RPL10A;SEPP1
Amitriptyline-160_mg/kg_in_Water-Rat-Heart-5d-up	8/318	0,094190633	0,237770971	0	0	1,72901943	4,084695212	C1QA;RPS17;RPL32;FEM1B;RPS6;RPS3A;RPL39;GOLGA7
Baclofen-19_mg/kg_in_CMC-Rat-Heart-3d-dn	8/318	0,094190633	0,237694787	0	0	1,72901943	4,084695212	EEF1B2;ARL2BP;RPS8;COL5A3;ARL6IP5;RPS20;RPS3A;SEPP1
Busulfan-3_mg/kg_in_Corn_Oil-Rat-Liver-3d-dn	8/318	0,094190633	0,237618651	0	0	1,72901943	4,084695212	EEF1B2;RPS17;RPL32;RPS19;RPS8;RPS6;RPS3A;RPL10A
Carbachol-15_mg/kg_in_Water-Rat-Brain-3d-up	8/318	0,094190633	0,237542565	0	0	1,72901943	4,084695212	EEF1B2;RPS17;RPL32;RPS8;RPS5;RPL35A;RPS3A;TPT1
Cisplatin-1.17_mg/kg_in_Saline-Rat-Bone_marrow-0.25d-dn	8/318	0,094190633	0,237466527	0	0	1,72901943	4,084695212	RPS17;RPS6;NKG7;RPS3A;RPL10A;SEPP1;RPL39;TPT1
Cyclophosphamide-25_mg/kg_in_Water-Rat-Bone_marrow-1d-dn	8/318	0,094190633	0,237390537	0	0	1,72901943	4,084695212	EEF1B2;RPS17;RPS6;ARL6IP5;RPS20;RPS3A;RPL39;DUSP14
NN-Dimethylformamide-1400_mg/kg_in_Saline-Rat-Liver-0.25d-up	8/318	0,094190633	0,237314597	0	0	1,72901943	4,084695212	EEF1B2;RPL32;RPS8;RPS5;RPSA;RPS3A;RPL10A;TPT1
Phenacetin-619_mg/kg_in_Corn_Oil-Rat-Liver-3d-up	8/318	0,094190633	0,237238705	0	0	1,72901943	4,084695212	RPL32;RPS19;RPS8;RPS5;RPS20;RPS3A;RPL10A;SEPP1
Tocainide-224_mg/kg_in_Corn_Oil-Rat-Liver-5d-up	8/318	0,094190633	0,237162861	0	0	1,72901943	4,084695212	RPL32;RPS19;RPS8;RPS20;RPSA;SEPP1;RPL39;TPT1
Valproic_Acid-850_mg/kg_in_Water-Rat-Bone_marrow-1d-dn	8/318	0,094190633	0,237087066	0	0	1,72901943	4,084695212	FEM1B;RPS6;RPS3A;RPL10A;SEPP1;RPL39;TPT1;DUSP14
Cisplatin-1.17_mg/kg_in_Saline-Rat-Spleen-3d-up	7/266	0,094688188	0,238263312	0	0	1,808645325	4,263277303	CXCL11;RPS19;RPS5;RPS20;RAPGEF5;RPL39;SEPP1
Cytarabine-23_mg/kg_in_Saline-Rat-Bone_marrow-0.25d-dn	6/215	0,094782272	0,238423881	0	0	1,918005275	4,51915204	RPS8;FEM1B;RPSA;RPS3A;RPL39;SEPP1
Pentobarbital-20_mg/kg_in_Water-Rat-Liver-0.25d-up	9/372	0,0948442	0,238503485	0	0	1,662786831	3,9167272	RPS19;RPS8;RPS6;RPS20;RPSA;RPS3A;RPL10A;SEPP1;GOLGA7
Antimycin_A-0.03_uM_in_DMSO-Rat-Primary_rat_hepatocytes-1d-up	8/319	0,095467783	0,239994975	0	0	1,723599306	4,048676927	RPS17;CXCL11;RPL32;RPS8;RPS5;RPL35A;RPSA;SEPP1
Aspirin-35_mg/kg_in_Corn_Oil-Rat-Liver-0.25d-up	8/319	0,095467783	0,239918398	0	0	1,723599306	4,048676927	RPL32;RPS19;RPS8;RPS5;RPS6;RPS20;RPSA;SEPP1
Bleomycin_A2-12_mg/kg_in_Saline-Rat-Bone_marrow-1d-up	8/319	0,095467783	0,239841869	0	0	1,723599306	4,048676927	RPL32;RPS19;RPS8;RPS5;RPS6;RPL35A;RPSA;RPL10A
Finasteride-800_mg/kg_in_Corn_Oil-Rat-Kidney-1d-up	8/319	0,095467783	0,239765388	0	0	1,723599306	4,048676927	EEF1B2;ARL2BP;CLP1;FUNDC1;RPS20;RPSA;SEPP1;TOMM20
Isoprenaline-15_mg/kg_in_Saline-Rat-Heart-5d-dn	8/319	0,095467783	0,239688957	0	0	1,723599306	4,048676927	RPS17;ARL2BP;RPS8;FUNDC1;RPS6;RPS3A;RAPGEF5;TPT1
Isoprenaline-15_mg/kg_in_Saline-Rat-Liver-1d-up	8/319	0,095467783	0,239612574	0	0	1,723599306	4,048676927	EEF1B2;RPS17;RPL32;FUNDC1;RPS6;RPS20;RPSA;SEPP1
Nevirapine-250_uM_in_DMSO-Rat-Primary_rat_hepatocytes-1d-dn	8/319	0,095467783	0,23953624	0	0	1,723599306	4,048676927	EEF1B2;RPS17;RPS19;RPS8;RPS20;RPSA;RPL39;TPT1
Pralidoxime_Chloride-48_mg/kg_in_Saline-Rat-Liver-5d-dn	8/319	0,095467783	0,239459955	0	0	1,723599306	4,048676927	EEF1B2;RPS17;RPL32;RPS19;CLP1;RPS20;RPS3A;SEPP1
Probenecid-600_mg/kg_in_Corn_Oil-Rat-Kidney-5d-up	8/319	0,095467783	0,239383718	0	0	1,723599306	4,048676927	RPS17;RPL32;ARL2BP;RPL35A;RPS20;RPSA;RPS3A;RPL39
Sodium_Selenate-1.6_mg/kg_in_Water-Rat-Heart-1d-dn	8/319	0,095467783	0,23930753	0	0	1,723599306	4,048676927	EEF1B2;RPL32;RPS19;FUNDC1;RPS6;RPS20;RPL10A;RPL39
Sulfaphenazole-1695_mg/kg_in_Water-Rat-Liver-0.25d-up	8/319	0,095467783	0,23923139	0	0	1,723599306	4,048676927	RPS17;RPL32;RPS8;RPS20;RPSA;RPS3A;RPL10A;TPT1
Tolazamide-1500_mg/kg_in_CMC-Rat-Heart-3d-dn	8/319	0,095467783	0,239155298	0	0	1,723599306	4,048676927	ARL2BP;FUNDC1;RPS6;RPS20;RPSA;SEPP1;RPL39;TPT1
Beta-Estradiol-250_uM_in_DMSO-Rat-Primary_rat_hepatocytes-1d-dn	9/373	0,096023316	0,240470474	0	0	1,658328957	3,885737106	RPS17;RPS19;ARL2BP;RPS8;RPS5;RPS20;RPS3A;RPL39;TPT1
Diethylstilbestrol-2.8_mg/kg_in_Corn_Oil-Rat-Spleen-3d-up	7/267	0,096101068	0,240588688	0	0	1,801871372	4,220622147	CXCL11;RPL32;RPS8;RPS5;FUNDC1;RPS6;RPS20
Temafloxacin-1000_mg/kg_in_CMC-Rat-Kidney-5d-dn	7/267	0,096101068	0,240512238	0	0	1,801871372	4,220622147	RPS17;RPS19;ARL2BP;RPS8;RPL35A;RPS20;SEPP1

Genistein-375_mg/kg_in_Corn_Oil-Rat-Liver-3d-up	8/320	0,09675481	0,242071436	0	0	1,718213058	4,013015868	EEF1B2;RPS17;RPS8;RPS5;RPS6;RPS20;RPS3A;GOLGA7
Nitrendipine-1100_mg/kg_in_Corn_Oil-Rat-Heart-1d-up	8/320	0,09675481	0,241994564	0	0	1,718213058	4,013015868	C1QA;RPS17;RPL32;COL5A3;RPSA;RPS3A;RPL39;TPT1
Phenobarbital-80_mg/kg_in_Water-Rat-Liver-5d-dn	8/320	0,09675481	0,24191774	0	0	1,718213058	4,013015868	EEF1B2;C1QA;RPS17;RPS19;RPS6;RPS20;RPL39;TPT1
Procainamide-755_mg/kg_in_Water-Rat-Heart-5d-dn	8/320	0,09675481	0,241840965	0	0	1,718213058	4,013015868	EEF1B2;CXCL11;RPS19;RPS8;RPS6;RPS20;RPSA;TPT1
Acetaminophen-100_mg/kg_in_Corn_Oil-Rat-Kidney-0.25d-dn	7/268	0,097525627	0,243690303	0	0	1,795147971	4,178458403	RPS19;RPS8;RPS5;RPSA;SEPP1;TOMM20;TPT1
Lead_(II)_Acetate-200_mg/kg_in_Saline-Rat-Liver-3d-up	7/268	0,097525627	0,243613014	0	0	1,795147971	4,178458403	C1QA;RPS17;RPS19;RPS6;RPS20;RPL10A;TPT1
Lomustine-4.2_mg/kg_in_Corn_Oil-Rat-Liver-1d-dn	7/268	0,097525627	0,243535775	0	0	1,795147971	4,178458403	C1QA;RPS17;RPL32;CLP1;RPS8;RPS6;RPL10A
Prednisone-68_mg/kg_in_CMC-Rat-Heart-3d-dn	7/268	0,097525627	0,243458585	0	0	1,795147971	4,178458403	RPS19;ARL2BP;COL5A3;FUNDC1;RPS6;RPS20;SEPP1
Tamoxifen-2.5_mg/kg_in_Corn_Oil-Rat-Liver-0.25d-dn	7/268	0,097525627	0,243381443	0	0	1,795147971	4,178458403	RPS17;RPS8;RPL35A;RPSA;RPL10A;RPL39;TPT1
Busulfan-9_mg/kg_in_Corn_Oil-Rat-Liver-1d-up	8/321	0,098051698	0,244616781	0	0	1,71286037	3,977707814	EEF1B2;RPS19;RPS8;RPS20;RPSA;SEPP1;RPL39;TPT1
Cisplatin-1.17_mg/kg_in_Saline-Rat-Spleen-0.25d-dn	8/321	0,098051698	0,244539321	0	0	1,71286037	3,977707814	EEF1B2;RPL32;RPS19;FUNDC1;RPS6;RPS20;RPL39;TPT1
Danazol-2000_mg/kg_in_CMC-Rat-Liver-1d-up	8/321	0,098051698	0,244461911	0	0	1,71286037	3,977707814	RPL32;RPS19;RPS5;RPS20;RPSA;RPL10A;RPL39;TPT1
Famciclovir-1200_mg/kg_in_Saline-Rat-Bone_marrow-1d-up	8/321	0,098051698	0,24438455	0	0	1,71286037	3,977707814	RPS17;RPS19;ARL2BP;RPS8;RPS5;RPS6;RPL35A;TPT1
Iproniazid-46_mg/kg_in_Water-Rat-Brain-3d-dn	8/321	0,098051698	0,244307237	0	0	1,71286037	3,977707814	RPL32;RPS19;RPS8;RPS6;RPS20;SEPP1;RPL39;PDRG1
Rosiglitazone-10_mg/kg_in_Corn_Oil-Rat-Liver-3d-up	8/321	0,098051698	0,244229974	0	0	1,71286037	3,977707814	C1QA;RPS19;RPL35A;RPS20;RPSA;SEPP1;RPL39;TPT1
Diphenhydramine-263_uM_in_DMSO-Rat-Primary_rat_hepatocytes-1d-dn	9/375	0,098407134	0,245037807	0	0	1,649484536	3,824564094	RPS17;CXCL11;ARL2BP;RPS8;ARL6IP5;SEPP1;RPL39;SLC16A3;TPT1
2-Amino-4-Nitrophenol-625_mg/kg_in_CMC-Rat-Heart-5d-dn	7/269	0,098961836	0,246341156	0	0	1,788474559	4,136779217	RPS17;RPS19;COL5A3;FUNDC1;RPS6;RPS3A;TPT1
Cytarabine-487_mg/kg_in_Saline-Rat-Spleen-3d-dn	7/269	0,098961836	0,246263323	0	0	1,788474559	4,136779217	C1QA;ARL2BP;RPS8;FUNDC1;RPS6;RPSA;TPT1
Doxorubicin-0.65_mg/kg_in_Saline-Rat-Liver-5d-dn	7/269	0,098961836	0,246185539	0	0	1,788474559	4,136779217	C1QA;RPS17;CLP1;RPS6;RPSA;RPS3A;RPL10A
Gemcitabine-11_mg/kg_in_Saline-Rat-Bone_marrow-1d-dn	7/269	0,098961836	0,246107805	0	0	1,788474559	4,136779217	RPS19;RPS20;RPSA;RPL10A;RPL39;SEPP1;TPT1
Methyldopa-325_mg/kg_in_Water-Rat-Heart-1d-up	7/269	0,098961836	0,246030119	0	0	1,788474559	4,136779217	RPS17;RPL32;RPS8;RPS6;ARL6IP5;RPL35A;RPS3A
Sulfaphenazole-1695_mg/kg_in_Water-Rat-Kidney-3d-dn	7/269	0,098961836	0,245952482	0	0	1,788474559	4,136779217	RPS17;RPL32;RPS6;ARL6IP5;RPL35A;RPS20;RPSA
Thioridazine-63_mg/kg_in_Corn_Oil-Rat-Heart-1d-dn	7/269	0,098961836	0,245874895	0	0	1,788474559	4,136779217	RPL32;RPS8;R3HCC1;RPS3A;SEPP1;TPT1;PDRG1
Aspirin-375_mg/kg_in_Corn_Oil-Rat-Kidney-3d-dn	8/322	0,099358433	0,246782408	0	0	1,707540928	3,942748603	EEF1B2;ARL2BP;CLP1;RPS8;FUNDC1;RPS6;SEPP1;TPT1
Ciprofloxacin-450_mg/kg_in_Corn_Oil-Rat-Liver-5d-up	8/322	0,099358433	0,246704608	0	0	1,707540928	3,942748603	EEF1B2;RPS19;RPS8;RPS20;RPSA;SEPP1;RPL39;TPT1
Fenofibrate-43_mg/kg_in_Corn_Oil-Rat-Liver-3d-dn	8/322	0,099358433	0,246626857	0	0	1,707540928	3,942748603	C1QA;RPS19;RPS8;RPS6;RPSA;RPL10A;SEPP1;TPT1
Nimesulide-162_mg/kg_in_Corn_Oil-Rat-Kidney-3d-dn	8/322	0,099358433	0,246549154	0	0	1,707540928	3,942748603	EEF1B2;RPS17;RPL32;RPS19;RPS6;SEPP1;RPL39;TPT1
Promazine-100_mg/kg_in_Corn_Oil-Rat-Liver-3d-dn	8/322	0,099358433	0,246471501	0	0	1,707540928	3,942748603	C1QA;RPS17;RPS19;RPS6;RPS3A;RPL10A;GOLGA7;TPT1
Temafloxacin-1000_mg/kg_in_CMC-Rat-Liver-1d-dn	8/322	0,099358433	0,246393897	0	0	1,707540928	3,942748603	EEF1B2;RPS17;RPL32;RPS19;RPS6;RPL35A;RPS3A;SEPP1
Ticrynafen-570_mg/kg_in_CMC-Rat-Liver-3d-up	8/322	0,099358433	0,246316341	0	0	1,707540928	3,942748603	EEF1B2;RPS6;RPS20;RPSA;RPL10A;SEPP1;RPL39;TPT1
Valproic_Acid-235_mg/kg_in_Water-Rat-Bone_marrow-1d-up	8/322	0,099358433	0,246238835	0	0	1,707540928	3,942748603	EEF1B2;RPS19;NKG7;RPS20;RPSA;RPL10A;SEPP1;TPT1
Carboplatin-14_mg/kg_in_Saline-Rat-Bone_marrow-3d-up	9/376	0,099611815	0,246789132	0	0	1,645097609	3,794375689	RPS17;RPS8;RPS5;NKG7;RPL35A;RPSA;RPL10A;TOMM20;TPT1
Cisplatin-2_mg/kg_in_Saline-Rat-Spleen-3d-up	7/270	0,100409666	0,248687588	0	0	1,781850579	4,095577856	CXCL11;RPS19;RPS8;NKG7;RPS20;RAPGEF5;RPL39
Clofibrate-500_mg/kg_in_Corn_Oil-Rat-Kidney-1d-up	7/270	0,100409666	0,248609409	0	0	1,781850579	4,095577856	RPS19;RPS5;RPS20;RPS3A;RAPGEF5;RPL39;TPT1
Emetine-1_mg/kg_in_Saline-Rat-Heart-5d-dn	7/270	0,100409666	0,248531279	0	0	1,781850579	4,095577856	RPS17;RPS6;RPS3A;RPL10A;RPL39;SEPP1;TPT1
Lead_(II)_Acetate-600_mg/kg_in_Saline-Rat-Bone_marrow-3d-dn	7/270	0,100409666	0,248453198	0	0	1,781850579	4,095577856	EEF1B2;RPL32;RPS19;FEM1B;RPS6;NKG7;RPL39
Sodium_Arsenite-30_mg/kg_in_Saline-Rat-Bone_marrow-3d-dn	7/270	0,100409666	0,248375166	0	0	1,781850579	4,095577856	C1QA;RPS17;RPL32;FEM1B;ARL6IP5;RPS3A;TPT1
Cyclosporin_A-350_mg/kg_in_Corn_Oil-Rat-Bone_marrow-1d-up	8/323	0,100674995	0,2489533	0	0	1,702254423	3,908134132	C1QA;RPS17;RPS19;RPS20;RPL10A;SEPP1;RPL39;TPT1
Mitomycin_C-0.5_mg/kg_in_Saline-Rat-Spleen-3d-up	8/323	0,100674995	0,24887516	0	0	1,702254423	3,908134132	RPS19;NKG7;RPS20;RPS3A;RAPGEF5;SEPP1;RPL39;TPT1
Tacrolimus-134_mg/kg_in_CMC-Rat-Kidney-1d-up	8/323	0,100674995	0,24879707	0	0	1,702254423	3,908134132	EEF1B2;RPS17;RPL32;RPS8;RPS6;RPL13A;RPL35A;RPS20
Danazol-95_uM_in_DMSO-Rat-Primary_rat_hepatocytes-1d-up	9/377	0,100824996	0,249089608	0	0	1,640733955	3,76444908	RPS17;RPL32;RPS19;RPS5;RPSA;RPS3A;RPL10A;RPL39;TPT1
Famciclovir-1200_mg/kg_in_Saline-Rat-Liver-3d-dn	9/377	0,100824996	0,249011499	0	0	1,640733955	3,76444908	C1QA;RPS17;RPL32;RPS8;RPSA;RPS3A;SEPP1;RPL39;TPT1
Loratadine-62.25_uM_in_DMSO-Rat-Primary_rat_hepatocytes-1d-up	9/377	0,100824996	0,248933439	0	0	1,640733955	3,76444908	RPS17;RPL32;RPS19;ARL2BP;RPS8;RPS5;RPS6;RPS3A;TPT1
Sildenafil-90_uM_in_DMSO-Rat-Primary_rat_hepatocytes-1d-up	9/377	0,100824996	0,248855428	0	0	1,640733955	3,76444908	EEF1B2;RPS17;RPL32;ARL2BP;RPS8;RPS5;RPS3A;TPT1;PDRG1
Diphenhydramine-263_uM_in_DMSO-Rat-Primary_rat_hepatocytes-0.67d-up	6/219	0,101226453	0,249768027	0	0	1,882973215	4,312752744	EEF1B2;RPS19;RPS8;RPS6;RPL10A;SEPP1
N-Nitrosodiethylamine-34_mg/kg_in_Saline-Rat-Liver-0.25d-dn	7/271	0,101869088	0,251274956	0	0	1,775275485	4,054847697	EEF1B2;RPL32;RPS19;RPS6;RPS20;RPL10A;RPL39
Antipyrine-1500_mg/kg_in_CMC-Rat-Kidney-3d-up	8/324	0,102001367	0,25152247	0	0	1,697000552	3,873860358	RPS17;ARL2BP;ARL6IP5;RPS20;RPSA;GOLGA7;TOMM20;TPT1
Calcitriol-0.04_mg/kg_in_CMC-Rat-Heart-3d-dn	8/324	0,102001367	0,251443746	0	0	1,697000552	3,873860358	RPS17;RPL32;ARL2BP;FUNDC1;RPS6;ARL6IP5;RPSA;SEPP1
Cilostazol-1500_mg/kg_in_CMC-Rat-Heart-5d-up	8/324	0,102001367	0,251365071	0	0	1,697000552	3,873860358	RPS17;CXCL11;RPL32;RPS6;RPS3A;RPL10A;RPL39;TPT1
Clomipramine-115_mg/kg_in_Water-Rat-Liver-3d-dn	8/324	0,102001367	0,251286446	0	0	1,697000552	3,873860358	EEF1B2;RPS19;CLP1;RPS6;RPS20;RPL10A;SEPP1;RPL39
Lead(IV)_Acetate-200_mg/kg_in_Saline-Rat-Liver-5d-up	8/324	0,102001367	0,25120787	0	0	1,697000552	3,873860358	C1QA;RPS17;RPS8;RPS20;RPSA;RPS3A;RPL39;TPT1
Rifabutin-1500_mg/kg_in_CMC-Rat-Kidney-1d-up	8/324	0,102001367	0,251129343	0	0	1,697000552	3,873860358	RPS17;RPL32;RPS8;RPS6;ARL6IP5;RPS3A;RAPGEF5;RPL39
Rofecoxib-111_uM_in_DMSO-Rat-Primary_rat_hepatocytes-1d-dn	8/324	0,102001367	0,251050865	0	0	1,697000552	3,873860358	RPS17;CXCL11;RPS19;RPS8;RPS5;SEPP1;RPL39;TPT1
Aldosterone-0.065_mg/kg_in_Saline-Rat-Heart-5d-up	8/325	0,10333753	0,254260039	0	0	1,691779011	3,839923295	ARL2BP;CLP1;RPS8;FUNDC1;ARL6IP5;RPS20;RPSA;RPL10A
Amikacin-82_mg/kg_in_Saline-Rat-Kidney-5d-dn	8/325	0,10333753	0,254180632	0	0	1,691779011	3,839923295	EEF1B2;RPS17;RPL32;RPS19;RPL35A;RPS20;RPS3A;SEPP1
Benzethonium_Chloride-30_mg/kg_in_Corn_Oil-Rat-Liver-5d-dn	8/325	0,10333753	0,254101275	0	0	1,691779011	3,839923295	RPS17;RPL32;RPS19;RPS6;RPS3A;RPL10A;RPL39;TPT1
Benzoic_Acid-1700_mg/kg_in_CMC-Rat-Liver-3d-up	8/325	0,10333753	0,254021968	0	0	1,691779011	3,839923295	CXADR;RPS19;RPS6;RPS20;RPSA;RAPGEF5;RPL39;GOLGA7
Chlorpyrifos-82_mg/kg_in_CMC-Rat-Heart-5d-dn	8/325	0,10333753	0,25394271	0	0	1,691779011	3,839923295	RPS17;RPL32;RPS19;COL5A3;FUNDC1;RPS6;RPSA;RPL39
Diclofenac-3.5_mg/kg_in_Corn_Oil-Rat-Kidney-5d-dn	8/325	0,10333753	0,253863501	0	0	1,691779011	3,839923295	EEF1B2;RPS17;RPS19;ARL2BP;RPS6;RPS3A;RPL10A;RPL39
Ifosfamide-143_mg/kg_in_Saline-Rat-Heart-5d-up	8/325	0,10333753	0,253784342	0	0	1,691779011	3,839923295	CXCL11;RPS19;RPS8;RPS6;RPL10A;SEPP1;RPL39;TPT1
Norfloxacin-1500_mg/kg_in_CMC-Rat-Kidney-5d-up	8/325	0,10333753	0,253705232	0	0	1,691779011	3,839923295	RPL32;ARL2BP;RPS8;RPSA;RAPGEF5;SEPP1;RPL39;TPT1
Oxybutynin-230_mg/kg_in_Water-Rat-Heart-1d-dn	8/325	0,10333753	0,253626172	0	0	1,691779011	3,839923295	EEF1B2;RPS17;RPL32;RPS20;RPS3A;RPL39;TPT1;PDRG1
Racepinephrine-0.77_mg/kg_in_Saline-Rat-Heart-5d-up	8/325	0,10333753	0,25354716	0	0	1,691779011	3,839923295	EEF1B2;RPS8;RPS20;RPL10A;SEPP1;RPL39;TPT1;PDRG1
Benazepril-1750_mg/kg_in_CMC-Rat-Kidney-3d-dn	7/272	0,103340069	0,253474426	0	0	1,768748737	4,014582233	ARL2BP;CLP1;RPS8;FUNDC1;RPS6;RPS3A;TPT1
Valproic_Acid-235_mg/kg_in_Water-Rat-Liver-0.25d-dn	7/272	0,103340069	0,253395511	0	0	1,768748737	4,014582233	C1QA;RPS17;RPS6;RPL35A;RPL10A;RPL39;SEPP1
Hydroxyurea-400_mg/kg_in_Saline-Rat-Liver-1d-dn	6/221	0,104533177	0,256241303	0	0	1,865932733	4,213744033	RPS17;CLP1;RPS8;RPS6;RPS3A;SEPP1
Methotrexate-0.3_mg/kg_in_Corn_Oil-Rat-Kidney-1d-dn	6/221	0,104533177	0,256161576	0	0	1,865932733	4,213744033	RPS19;ARL2BP;RPS8;RPSA;RPL10A;TPT1
BW-723C86-10_mg/kg_in_Saline-Rat-Heart-1d-up	8/326	0,104683463	0,256450063	0	0	1,686589505	3,806319014	C1QA;RPS6;ARL6IP5;RPS20;RPSA;RPS3A;RPL10A;RPL39
Diphenhydramine-10.9_mg/kg_in_Water-Rat-Heart-5d-up	8/326	0,104683463	0,256370321	0	0	1,686589505	3,806319014	RPS17;RPL32;FEM1B;RPS6;RPL35A;RPSA;RPS3A;RPL39
Hydrocortisone-56_mg/kg_in_Corn_Oil-Rat-Kidney-1d-up	8/326	0,104683463	0,256290629	0	0	1,686589505	3,806319014	EEF1B2;C1QA;RPS17;RPL32;RPS20;RPSA;SEPP1;TPT1
Melatonin-2000_mg/kg_in_Corn_Oil-Rat-Liver-3d-dn	8/326	0,104683463	0,256210986	0	0	1,686589505	3,806319014	EEF1B2;C1QA;RPS17;RPL35A;RPS20;SEPP1;RPL39;TPT1
Oxaliplatin-1.31_mg/kg_in_Saline-Rat-Bone_marrow-1d-up	8/326	0,104683463	0,256131393	0	0	1,686589505	3,806319014	C1QA;RPS17;RPS19;FEM1B;NKG7;RPSA;RPL10A;SEPP1
Probenecid-600_mg/kg_in_Corn_Oil-Rat-Kidney-3d-up	8/326	0,104683463	0,256051849	0	0	1,686589505	3,806319014	RPL32;RPS19;RPS8;RPS6;RPSA;RPS3A;RPL10A;RPL39
Urethane-453_mg/kg_in_Water-Rat-Bone_marrow-1d-dn	8/326	0,104683463	0,255972354	0	0	1,686589505	3,806319014	C1QA;RPS17;RPS6;NKG7;RPS20;RPS3A;RPL39;TPT1
Acyclovir-330_mg/kg_in_Saline-Rat-Bone_marrow-0.25d-dn	7/273	0,104822576	0,256232964	0	0	1,762269804	3,974775063	RPS8;NKG7;RPS20;RPS3A;RPL10A;RPL39;SEPP1
Atropine-2.3_mg/kg_in_Water-Rat-Liver-5d-dn	7/273	0,104822576	0,256153462	0	0	1,762269804	3,974775063	EEF1B2;C1QA;RPS17;RPL32;RPS19;RPS6;SEPP1
Mannitol-1000_mg/kg_in_Saline-Rat-Kidney-5d-dn	7/273	0,104822576	0,25607401	0	0	1,762269804	3,974775063	EEF1B2;RPS19;ARL2BP;RPS6;RPS3A;RPL10A;RPL39
Tramadol-114_mg/kg_in_Water-Rat-Brain-3d-up	7/273	0,104822576	0,255994608	0	0	1,762269804	3,974775063	EEF1B2;RPS17;CLP1;FUNDC1;RPSA;RPS3A;TPT1
Zileuton-450_mg/kg_in_Corn_Oil-Rat-Liver-1d-up	7/273	0,104822576	0,255915254	0	0	1,762269804	3,974775063	RPS6;RPS20;RPSA;RPS3A;RPL10A;GOLGA7;SEPP1
Diphenhydramine-263_uM_in_DMSO-Rat-Primary_rat_hepatocytes-0.67d-dn	9/381	0,105762496	0,258129971	0	0	1,623508402	3,647307905	RPS17;RPL32;RPL35A;RPS20;RPSA;RPS3A;RPL39;TPT1;PDRG1
Cerivastatin-0.05_mg/kg_in_Corn_Oil-Rat-Liver-0.25d-up	8/327	0,106039145	0,258725002	0	0	1,681431739	3,773043641	EEF1B2;C1QA;RPS17;RPL32;RPS19;RPS6;RPL10A;SEPP1
Clomipramine-115_mg/kg_in_Water-Rat-Heart-5d-dn	8/327	0,106039145	0,258644876	0	0	1,681431739	3,773043641	RPL32;RPS19;FUNDC1;RPS6;RPS20;RPSA;RPS3A;TPT1
Droperidol-94_mg/kg_in_CMC-Rat-Heart-1d-up	8/327	0,106039145	0,258564801	0	0	1,681431739	3,773043641	RPS17;RPL32;RPSA;RPS3A;RPL10A;RPL39;TPT1;PDRG1
Ethinylestradiol-190_uM_in_DMSO-Rat-Primary_rat_hepatocytes-1d-dn	8/327	0,106039145	0,258484774	0	0	1,681431739	3,773043641	CXCL11;RPS19;ARL6IP5;RPS20;SEPP1;RPL39;SLC16A3;TPT1
Valacyclovir-88_mg/kg_in_CMC-Rat-Kidney-3d-up	8/327	0,106039145	0,258404798	0	0	1,681431739	3,773043641	RPS17;RPS19;RPS6;ARL6IP5;RPS20;RPSA;SEPP1;RPL39
Venlafaxine-1481_uM_in_DMSO-Rat-Primary_rat_hepatocytes-0.67d-up	8/327	0,106039145	0,25832487	0	0	1,681431739	3,773043641	EEF1B2;RPS19;RPS6;RPL10A;SEPP1;RPL39;GOLGA7;TPT1
Aminoglutethimide-350_mg/kg_in_CMC-Rat-Liver-0.25d-dn	7/274	0,106316576	0,25892064	0	0	1,755838162	3,935419896	EEF1B2;RPL32;RPS8;RPS6;RPL35A;SEPP1;TPT1
Cerivastatin-7_mg/kg_in_Corn_Oil-Rat-Skeletal_muscle-5d-up	7/274	0,106316576	0,258840603	0	0	1,755838162	3,935419896	RPL32;RPS19;RPS8;RPS6;RPS20;RPL10A;TPT1
Stanozolol-150_mg/kg_in_Water-Rat-Liver-3d-dn	7/274	0,106316576	0,258760615	0	0	1,755838162	3,935419896	RPS17;RPL32;RPS8;RPL35A;RPS20;RPS3A;TPT1
Hydrazine-45_mg/kg_in_Water-Rat-Kidney-1d-dn	5/172	0,106823015	0,259912902	0	0	1,997922161	4,468516509	RPS17;RPSA;RPL10A;RPL39;SEPP1

Irinotecan-5_mg/kg_in_Saline-Rat-Heart-1d-dn	8/328	0,107404554	0,26124715	0	0	1,676305423	3,740093356	RPS17;RPL32;RPS8;RPS6;ARL6IP5;RPS20;SEPP1;TPT1
Phenytoin-572_mg/kg_in_Water-Rat-Heart-3d-dn	8/328	0,107404554	0,261166493	0	0	1,676305423	3,740093356	EEF1B2;RPS17;RPS8;RPS20;RPSA;RPS3A;RPL39;TPT1
Aconitine-0.9_mg/kg_in_CMC-Rat-Heart-5d-up	7/275	0,107822032	0,262100718	0	0	1,749453296	3,896510545	RPS17;RPS6;RPSA;RPS3A;RPL39;SEPP1;TPT1
Aldosterone-0.065_mg/kg_in_Saline-Rat-Heart-5d-dn	7/275	0,107822032	0,262019848	0	0	1,749453296	3,896510545	EEF1B2;RPS17;RPL32;RPS6;RPS3A;SEPP1;TPT1
Benzethonium_Chloride-5_mg/kg_in_Water-Rat-Heart-5d-dn	7/275	0,107822032	0,261939027	0	0	1,749453296	3,896510545	EEF1B2;RPS8;FUNDC1;R3HCC1;ARL6IP5;SEPP1;TPT1
Lead(IV)_Acetate-600_mg/kg_in_Saline-Rat-Liver-3d-up	7/275	0,107822032	0,261858257	0	0	1,749453296	3,896510545	EEF1B2;RPS17;RPL32;RPS8;RPS5;RPL35A;RPS20
Pantoprazole-1100_mg/kg_in_Water-Rat-Liver-3d-dn	7/275	0,107822032	0,261777536	0	0	1,749453296	3,896510545	EEF1B2;C1QA;CLP1;RPS20;RPL39;SEPP1;TPT1
Loratadine-62.25_uM_in_DMSO-Rat-Primary_rat_hepatocytes-1d-dn	6/223	0,107895724	0,261875724	0	0	1,84919791	4,117405637	EEF1B2;CXCL11;ARL6IP5;RPS20;RPL10A;SEPP1
Colchicine-1000_uM_in_DMSO-Rat-Primary_rat_hepatocytes-0.67d-dn	12/551	0,108531543	0,263337778	0	0	1,496809924	3,323987403	RPS17;RPL32;ARL2BP;CLP1;ARL6IP5;RPSA;RPS3A;RPL10A;SEPP1;RPL39;GOLGA7;TPT1
Aceclofenac-9_mg/kg_in_Corn_Oil-Rat-Kidney-3d-up	8/329	0,108779668	0,263858536	0	0	1,67121027	3,707464395	C1QA;RPS17;RPL32;RPS19;RPS20;RPSA;RPS3A;RPL39
Cisapride-250_mg/kg_in_Corn_Oil-Rat-Heart-3d-up	8/329	0,108779668	0,263777299	0	0	1,67121027	3,707464395	ARL6IP5;RPS20;RPSA;RPS3A;RPL10A;RPL39;TPT1;PDRG1
Cortisone-206_mg/kg_in_CMC-Rat-Heart-1d-up	8/329	0,108779668	0,263696112	0	0	1,67121027	3,707464395	EEF1B2;ARL2BP;CLP1;RPS8;FUNDC1;RPS20;RPL10A;RPL39
Labetalol-250_uM_in_DMSO-Rat-Primary_rat_hepatocytes-1d-dn	8/329	0,108779668	0,263614974	0	0	1,67121027	3,707464395	CXCL11;RPS19;ARL6IP5;RPSA;SEPP1;RPL39;SLC16A3;TPT1
Microcystin-LR-0.07_uM_in_DMSO-Rat-Primary_rat_hepatocytes-1d-dn	8/329	0,108779668	0,263533887	0	0	1,67121027	3,707464395	RPL32;RPS19;ARL2BP;RPS8;RPS20;RPS3A;RPL39;TPT1
Cilostazol-1500_mg/kg_in_CMC-Rat-Heart-5d-dn	7/276	0,109338909	0,264807271	0	0	1,743114697	3,858040927	EEF1B2;RPS19;ARL2BP;RPS8;FUNDC1;GOLGA7;SEPP1
Doxorubicin-0.65_mg/kg_in_Saline-Rat-Bone_marrow-1d-dn	7/276	0,109338909	0,264725867	0	0	1,743114697	3,858040927	EEF1B2;RPL32;RPSA;RPS3A;RPL10A;RPL39;TPT1
Rifabutin-1500_mg/kg_in_CMC-Rat-Kidney-1d-dn	7/276	0,109338909	0,264644513	0	0	1,743114697	3,858040927	EEF1B2;CLP1;RPS5;RPSA;RPL10A;SEPP1;TOMM20
Spironolactone-300_mg/kg_in_CMC-Rat-Kidney-5d-up	7/276	0,109338909	0,264563209	0	0	1,743114697	3,858040927	RPS17;FEM1B;RPS6;ARL6IP5;RPS20;RPSA;RPS3A
Zidovudine-1473_uM_in_DMSO-Rat-Primary_rat_hepatocytes-0.67d-dn	7/276	0,109338909	0,264481955	0	0	1,743114697	3,858040927	RPS17;RPS19;ARL6IP5;RPS3A;RPL10A;RPL39;SEPP1
Altretamine-40_mg/kg_in_Corn_Oil-Rat-Liver-5d-up	8/330	0,110164462	0,266397085	0	0	1,666145996	3,675153044	EEF1B2;C1QA;RPS17;RPL32;RPL35A;RPS20;RPL39;TPT1
Fenofibrate-215_mg/kg_in_Corn_Oil-Rat-Liver-1d-up	8/330	0,110164462	0,266315318	0	0	1,666145996	3,675153044	RPS17;RPL32;RPS19;RPS8;RPS6;RPS20;RPS3A;RPL10A
Maprotiline-380_mg/kg_in_CMC-Rat-Heart-5d-dn	8/330	0,110164462	0,266233601	0	0	1,666145996	3,675153044	EEF1B2;RPS17;RPS19;COL5A3;ARL6IP5;SEPP1;RPL39;GOLGA7
Podophyllotoxin-4_mg/kg_in_Corn_Oil-Rat-Bone_marrow-1d-up	8/330	0,110164462	0,266151934	0	0	1,666145996	3,675153044	EEF1B2;C1QA;RPS17;RPL32;RPS8;NKG7;RPS3A;TPT1
Procarbazine-54_mg/kg_in_Water-Rat-Liver-3d-dn	8/330	0,110164462	0,266070317	0	0	1,666145996	3,675153044	C1QA;RPL32;RPS20;RPSA;RPS3A;SEPP1;RPL39;TPT1
Sodium_Arsenite-30_mg/kg_in_Saline-Rat-Bone_marrow-3d-up	8/330	0,110164462	0,265988751	0	0	1,666145996	3,675153044	RPS19;RPS8;RPS5;RPS6;NKG7;RPS20;RPL10A;RPL39
Tacrolimus-50_uM_in_DMSO-Rat-Primary_rat_hepatocytes-1d-dn	8/330	0,110164462	0,265907234	0	0	1,666145996	3,675153044	CXCL11;RPS19;RPS6;ARL6IP5;SEPP1;RPL39;SLC16A3;TPT1
Testosterone-375_mg/kg_in_CMC-Rat-Liver-1d-up	8/330	0,110164462	0,265825768	0	0	1,666145996	3,675153044	EEF1B2;RPS8;RPS5;RPSA;RPS3A;RPL10A;SEPP1;TPT1
Hydrazine-45_mg/kg_in_Water-Rat-Kidney-5d-dn	5/174	0,110745351	0,267145601	0	0	1,974957538	4,345937206	RPS17;RPL32;RPS6;RPSA;RPS20
Cytarabine-23_mg/kg_in_Saline-Rat-Bone_marrow-0.25d-up	9/385	0,110834977	0,267279937	0	0	1,606640782	3,534148425	RPS17;RPL32;RPS19;RPS5;RPS6;NKG7;RPS20;TPT1;PDRG1
Amiodarone-147_mg/kg_in_CMC-Rat-Heart-5d-up	7/277	0,110867167	0,26727573	0	0	1,736821864	3,82000506	RPS17;RPL32;ARL2BP;RPS8;RPS20;RPSA;TOMM20
L-Phenylephrine-0.44_mg/kg_in_Saline-Rat-Heart-3d-dn	7/277	0,110867167	0,267193944	0	0	1,736821864	3,82000506	EEF1B2;RPS17;RPL32;RPS6;RPS3A;SEPP1;TPT1
Lamivudine-1300_mg/kg_in_Water-Rat-Liver-3d-dn	7/277	0,110867167	0,267112209	0	0	1,736821864	3,82000506	RPL32;RPS8;RPL35A;RPS3A;RPL10A;RPL39;TPT1
Lead_(II)_Acetate-200_mg/kg_in_Saline-Rat-Spleen-1d-dn	7/277	0,110867167	0,267030523	0	0	1,736821864	3,82000506	EEF1B2;RPS8;RPS5;FUNDC1;RPL13A;RPL35A;RPS3A
NN-Dimethylformamide-1400_mg/kg_in_Saline-Rat-Bone_marrow-5d-up	7/277	0,110867167	0,266948887	0	0	1,736821864	3,82000506	C1QA;ARL2BP;RPS8;RPS6;RPS3A;SEPP1;TPT1
Vecuronium_Bromide-0.05_mg/kg_in_Saline-Rat-Heart-5d-dn	7/277	0,110867167	0,266867301	0	0	1,736821864	3,82000506	EEF1B2;RPS17;RPS8;ARL6IP5;RPS3A;RAPGEF5;TPT1
Lansoprazole-600_mg/kg_in_CMC-Rat-Liver-3d-up	8/331	0,111558912	0,268450349	0	0	1,661112322	3,643155644	EEF1B2;RPS17;RPL32;RPS5;RPS6;RPL35A;RPS20;RPSA
Cisapride-250_mg/kg_in_Corn_Oil-Rat-Intestine-5d-up	7/278	0,112406769	0,270407975	0	0	1,730574303	3,782397061	RPL32;RPS19;RPS8;RPS6;RPS3A;SLC16A3;TPT1
Dexamethasone-150_mg/kg_in_Corn_Oil-Rat-Liver-1d-dn	7/278	0,112406769	0,270325407	0	0	1,730574303	3,782397061	EEF1B2;C1QA;RPS17;RPS6;RPL10A;RPL39;SEPP1
Promazine-100_mg/kg_in_Corn_Oil-Rat-Liver-3d-up	7/278	0,112406769	0,270242891	0	0	1,730574303	3,782397061	RPL32;RPS8;RPS5;RPL35A;RPS20;RPSA;SEPP1
Sulindac-23_mg/kg_in_Corn_Oil-Rat-Kidney-5d-up	7/278	0,112406769	0,270160424	0	0	1,730574303	3,782397061	RPS19;RPS8;RPS6;ARL6IP5;RPS3A;RPL10A;TPT1
CerIVastatin-7_mg/kg_in_Corn_Oil-Rat-Kidney-1d-dn	8/332	0,112962992	0,271414437	0	0	1,656108972	3,611468584	EEF1B2;RPS19;RPS8;RPS6;RPS20;RPSA;RPS3A;SEPP1
Dexamethasone-1_mg/kg_in_Corn_Oil-Rat-Heart-3d-up	8/332	0,112962992	0,271331664	0	0	1,656108972	3,611468584	EEF1B2;RPS8;FUNDC1;ARL6IP5;RPS20;RAPGEF5;SEPP1;RPL39
Ethosuximide-1200_mg/kg_in_Water-Rat-Heart-5d-dn	8/332	0,112962992	0,271248941	0	0	1,656108972	3,611468584	RPS19;COL5A3;FUNDC1;RPS6;RPSA;RPS3A;SEPP1;TPT1
Loperamide-47_mg/kg_in_CMC-Rat-Intestine-1d-dn	8/332	0,112962992	0,271166268	0	0	1,656108972	3,611468584	EEF1B2;RPS19;RPS20;RPSA;RPS3A;RPL10A;RPL39;TOMM20
Phenobarbital-54_mg/kg_in_Water-Rat-Liver-3d-up	8/332	0,112962992	0,271083646	0	0	1,656108972	3,611468584	RPS17;RPL32;RPS19;RPS8;RPS5;RPSA;RPS3A;SEPP1
Acrolein-20_mg/kg_in_Water-Rat-Heart-3d-up	7/279	0,113957672	0,273387336	0	0	1,724371528	3,745211144	C1QA;ARL2BP;FUNDC1;RPS20;RAPGEF5;GOLGA7;SEPP1
Busulfan-9_mg/kg_in_Corn_Oil-Rat-Liver-1d-dn	7/279	0,113957672	0,273304088	0	0	1,724371528	3,745211144	C1QA;RPS17;RPL32;RPS6;RPL35A;RPS3A;RPL10A
Colistin-121_mg/kg_in_Water-Rat-Liver-3d-dn	7/279	0,113957672	0,273220891	0	0	1,724371528	3,745211144	EEF1B2;RPL32;RPS8;RPL35A;RPS20;RPL39;TPT1
Danazol-2000_mg/kg_in_CMC-Rat-Liver-1d-dn	7/279	0,113957672	0,273137744	0	0	1,724371528	3,745211144	EEF1B2;C1QA;RPS17;CLP1;RPS8;RPS6;SEPP1
Doxorubicin-3_mg/kg_in_Saline-Rat-Kidney-3d-dn	7/279	0,113957672	0,273054647	0	0	1,724371528	3,745211144	RPS17;RPS19;RPS20;RPSA;RPL39;SEPP1;TPT1
Eperisone-501_mg/kg_in_CMC-Rat-Liver-1d-up	7/279	0,113957672	0,272971601	0	0	1,724371528	3,745211144	EEF1B2;RPS17;RPS19;RPSA;RPS3A;RPL10A;SEPP1
Mitomycin_C-1.7_mg/kg_in_Saline-Rat-Liver-0.25d-up	7/279	0,113957672	0,272888606	0	0	1,724371528	3,745211144	C1QA;RPS17;RPS19;RPS6;RPS3A;RPL39;TPT1
Nifedipine-1022_mg/kg_in_CMC-Rat-Heart-1d-dn	7/279	0,113957672	0,272805661	0	0	1,724371528	3,745211144	RPS17;RPL32;ARL2BP;RPS8;ARL6IP5;RPS3A;TPT1
Procaine-2500_mg/kg_in_Water-Rat-Heart-1d-up	7/279	0,113957672	0,272722767	0	0	1,724371528	3,745211144	CXCL11;RPS19;ARL2BP;RPS6;RAPGEF5;GOLGA7;SEPP1
Tegafur-175_mg/kg_in_Water-Rat-Bone_marrow-3d-dn	7/279	0,113957672	0,272639923	0	0	1,724371528	3,745211144	EEF1B2;RPS17;ARL2BP;RPS8;FUNDC1;RPS20;TPT1
Carmustine-4_mg/kg_in_Corn_Oil-Rat-Spleen-3d-dn	8/333	0,114376675	0,273559276	0	0	1,651135672	3,580088305	RPS17;ARL6IP5;RPL13A;RPSA;RPL10A;SEPP1;RPL39;GOLGA7
Diethylstilbestrol-2.8_mg/kg_in_Corn_Oil-Rat-Spleen-3d-dn	8/333	0,114376675	0,273476228	0	0	1,651135672	3,580088305	C1QA;RPS17;RPS19;RPS3A;RPL10A;SEPP1;RPL39;TPT1
Econazole-43_mg/kg_in_Corn_Oil-Rat-Liver-0.25d-up	8/333	0,114376675	0,273393231	0	0	1,651135672	3,580088305	EEF1B2;RPS17;RPL32;RPS6;RPL35A;RPS3A;RPL10A;RPL39
Nortriptyline-70_uM_in_DMSO-Rat-Primary_rat_hepatocytes-1d-dn	8/333	0,114376675	0,273310284	0	0	1,651135672	3,580088305	C1QA;CXCL11;RPS19;ARL6IP5;SEPP1;RPL39;SLC16A3;TPT1
Atropine-2.3_mg/kg_in_Water-Rat-Liver-3d-dn	6/227	0,114786309	0,274205935	0	0	1,816612925	3,932391232	EEF1B2;RPS17;RPS20;RPL10A;SEPP1;TPT1
Azathioprine-54_mg/kg_in_Water-Rat-Kidney-3d-dn	6/227	0,114786309	0,274122792	0	0	1,816612925	3,932391232	EEF1B2;RPL32;FEM1B;RPS6;RPL10A;RPL39
Aflatoxin_B1-0.3_mg/kg_in_CMC-Rat-Liver-3d-dn	7/280	0,115519836	0,275790915	0	0	1,718213058	3,708441619	C1QA;CLP1;RPS8;RPL35A;RPS20;RPL10A;SEPP1
Busulfan-3_mg/kg_in_Corn_Oil-Rat-Bone_marrow-1d-dn	7/280	0,115519836	0,275707342	0	0	1,718213058	3,708441619	RPL32;FEM1B;RPS6;ARL6IP5;RPSA;RPS3A;RPL10A
Carmustine-4_mg/kg_in_Corn_Oil-Rat-Liver-3d-dn	7/280	0,115519836	0,27562382	0	0	1,718213058	3,708441619	EEF1B2;C1QA;RPS17;RPS19;RPSA;RPL39;TPT1
Catechol-195_mg/kg_in_Saline-Rat-Spleen-1d-dn	7/280	0,115519836	0,275540348	0	0	1,718213058	3,708441619	EEF1B2;RPS8;RPS5;FUNDC1;RPL35A;RPS3A;TPT1
Cisplatin-1.17_mg/kg_in_Saline-Rat-Kidney-5d-up	7/280	0,115519836	0,275456927	0	0	1,718213058	3,708441619	RPS19;RPS6;RPS20;RPSA;RPL10A;RPL39;TPT1
Ibuprofen-90_mg/kg_in_Corn_Oil-Rat-Liver-1d-up	7/280	0,115519836	0,275373556	0	0	1,718213058	3,708441619	C1QA;RPS17;RPS8;RPS6;RPL39;SEPP1;TPT1
Miconazole-920_mg/kg_in_Corn_Oil-Rat-Liver-0.25d-up	7/280	0,115519836	0,275290236	0	0	1,718213058	3,708441619	RPS17;RPL32;RPS19;RPS6;RPS20;RPS3A;RPL10A
Salicylamide-1300_mg/kg_in_CMC-Rat-Liver-5d-up	7/280	0,115519836	0,275206966	0	0	1,718213058	3,708441619	RPS19;RPS8;RPS6;RPS20;RPS3A;RAPGEF5;RPL39
Etoposide-100_mg/kg_in_Corn_Oil-Rat-Spleen-3d-dn	8/334	0,115799935	0,275790834	0	0	1,646192152	3,549011298	C1QA;RPS17;RPS19;RPSA;RPL10A;SEPP1;RPL39;TPT1
Zidovudine-14800_uM_in_DMSO-Rat-Primary_rat_hepatocytes-1d-dn	8/334	0,115799935	0,275707463	0	0	1,646192152	3,549011298	CXCL11;RPS19;ARL6IP5;RPL10A;SEPP1;RPL39;SLC16A3;TPT1
Bithionol-333_mg/kg_in_Corn_Oil-Rat-Liver-0.25d-up	9/389	0,116041425	0,27619893	0	0	1,590120054	3,424813362	EEF1B2;RPS17;RPL32;RPS19;RPS6;RPS3A;SEPP1;RPL39;TPT1
Ethinylestradiol-190_uM_in_DMSO-Rat-Primary_rat_hepatocytes-0.67d-up	9/389	0,116041425	0,276115486	0	0	1,590120054	3,424813362	EEF1B2;RPS19;RPS6;RPL10A;SEPP1;RPL39;GOLGA7;TPT1;PDRG1
Flunarizine-126_mg/kg_in_CMC-Rat-Heart-1d-dn	6/228	0,116543048	0,277225324	0	0	1,808645325	3,887673287	RPS19;RPSA;RPL10A;RPL39;GOLGA7;SEPP1
Pyridine-223_mg/kg_in_Water-Rat-Kidney-1d-dn	6/228	0,116543048	0,277141621	0	0	1,808645325	3,887673287	EEF1B2;RPS17;RPL32;RPS19;RPS6;RPSA
Antimycin_A-0.03_uM_in_DMSO-Rat-Primary_rat_hepatocytes-1d-dn	7/281	0,117093218	0,278365887	0	0	1,712098421	3,672082888	RPS19;RPS6;RPS20;RPS3A;RPL10A;RPL39;TPT1
Bithionol-333_mg/kg_in_Corn_Oil-Rat-Liver-3d-dn	7/281	0,117093218	0,27828189	0	0	1,712098421	3,672082888	RPS17;RPL32;RPS19;RPS6;RPS3A;RPL10A;TPT1
Bleomycin_A2-12_mg/kg_in_Saline-Rat-Bone_marrow-1d-dn	7/281	0,117093218	0,278197944	0	0	1,712098421	3,672082888	EEF1B2;RPS17;NKG7;RPS20;RPS3A;RPL39;GOLGA7
Cholecalciferol-8_mg/kg_in_CMC-Rat-Heart-1d-dn	7/281	0,117093218	0,278114048	0	0	1,712098421	3,672082888	RPS17;RPL32;RPS8;RPS6;RPSA;RPS3A;TPT1
Citalopram-90_mg/kg_in_Corn_Oil-Rat-Heart-5d-dn	7/281	0,117093218	0,278030203	0	0	1,712098421	3,672082888	RPS17;RPL32;RPS19;ARL2BP;RPL35A;RPS3A;GOLGA7
Cytarabine-487_mg/kg_in_Saline-Rat-Kidney-5d-up	7/281	0,117093218	0,277946409	0	0	1,712098421	3,672082888	EEF1B2;RPS19;ARL2BP;CLP1;RPS3A;SEPP1;TPT1
Ferrocene-330_mg/kg_in_CMC-Rat-Kidney-5d-up	7/281	0,117093218	0,277862665	0	0	1,712098421	3,672082888	ARL2BP;CLP1;RPS8;RPS20;RPSA;RPS3A;SEPP1
Lincomycin-1200_mg/kg_in_CMC-Rat-Kidney-1d-dn	7/281	0,117093218	0,277778971	0	0	1,712098421	3,672082888	EEF1B2;RPL35A;RPSA;RPS3A;RPL10A;TOMM20;TPT1
Aspirin-167_mg/kg_in_Corn_Oil-Rat-Liver-1d-dn	8/335	0,117232742	0,27802622	0	0	1,641278145	3,5182341	EEF1B2;RPS19;CLP1;RPS20;RPSA;RPS3A;RPL10A;GOLGA7
Clozapine-69_uM_in_DMSO-Rat-Primary_rat_hepatocytes-1d-dn	8/335	0,117232742	0,277942528	0	0	1,641278145	3,5182341	CXCL11;RPS19;RPS8;ARL6IP5;SEPP1;RPL39;SLC16A3;TPT1
Olanzapine-23_mg/kg_in_CMC-Rat-Liver-3d-up	8/335	0,117232742	0,277858886	0	0	1,641278145	3,5182341	RPS17;RPL32;RPS19;RPL35A;RPSA;RPS3A;RPL39;TPT1
Penicillamine-852_mg/kg_in_Water-Rat-Liver-3d-up	8/335	0,117232742	0,277775294	0	0	1,641278145	3,5182341	EEF1B2;RPS19;CLP1;RPS6;RPS20;RPL10A;SEPP1;GOLGA7
Lead(IV)_Acetate-600_mg/kg_in_Saline-Rat-Bone_marrow-1d-dn	6/229	0,118313287	0,280251262	0	0	1,80074731	3,843549634	RPS19;RPS6;NKG7;RPS3A;RPL10A;RPL39
Thioguanine-12_mg/kg_in_Corn_Oil-Rat-Spleen-3d-dn	8/336	0,118675068	0,281023703	0	0	1,636393389	3,487753299	RPS17;RPS19;NKG7;RPSA;RPS3A;RPL10A;RPL39;TPT1
Venlafaxine-320_mg/kg_in_Water-Rat-Liver-1d-up	8/336	0,118675068	0,280939235	0	0	1,636393389	3,487753299	C1QA;RPS17;RPL32;RPS6;RPL35A;RPS20;SEPP1;GOLGA7

Amitraz-75_mg/kg_in_CMC-Rat-Liver-5d-up	7/282	0,118677773	0,28086122	0	0	1,70602715	3,636129446	EEF1B2;RPS6;RPS20;RPSA;RPS3A;RPL39;TPT1
Chlorambucil-0.6_mg/kg_in_Corn_Oil-Rat-Bone_marrow-3d-dn	7/282	0,118677773	0,280776852	0	0	1,70602715	3,636129446	RPS19;FEM1B;RPS6;ARL6IP5;NKG7;RPSA;RPS3A
Citalopram-40_mg/kg_in_Corn_Oil-Rat-Liver-1d-dn	7/282	0,118677773	0,280692535	0	0	1,70602715	3,636129446	EEF1B2;RPL32;RPS8;RPS5;RPSA;RPS3A;SEPP1
Colistin-121_mg/kg_in_Water-Rat-Liver-1d-dn	7/282	0,118677773	0,280608268	0	0	1,70602715	3,636129446	EEF1B2;RPS19;RPS20;RPL10A;RPL39;SEPP1;TPT1
Idebenone-2500_mg/kg_in_CMC-Rat-Liver-3d-dn	7/282	0,118677773	0,280524052	0	0	1,70602715	3,636129446	EEF1B2;C1QA;RPS17;RPL32;CLP1;SEPP1;TPT1
Nadolol-1500_mg/kg_in_CMC-Rat-Heart-5d-up	7/282	0,118677773	0,280439886	0	0	1,70602715	3,636129446	EEF1B2;RPL32;RPSA;RPS3A;RAPGEF5;RPL39;PDRG1
Neomycin-56_mg/kg_in_Corn_Oil-Rat-Liver-0.25d-dn	7/282	0,118677773	0,280355771	0	0	1,70602715	3,636129446	RPS17;RPL32;RPS8;RPS5;RPL35A;RPSA;TPT1
Pentoxifylline-1170_mg/kg_in_CMC-Rat-Heart-5d-up	7/282	0,118677773	0,280271707	0	0	1,70602715	3,636129446	RPS17;CXCL11;RPL32;RPS20;RPSA;RPL10A;TPT1
Rofecoxib-775_mg/kg_in_Corn_Oil-Rat-Liver-0.25d-dn	7/282	0,118677773	0,280187692	0	0	1,70602715	3,636129446	C1QA;RPS17;RPL35A;RPS20;RPS3A;RPL39;TPT1
Simvastatin-15_mg/kg_in_Corn_Oil-Rat-Liver-0.25d-dn	7/282	0,118677773	0,280103728	0	0	1,70602715	3,636129446	RPL32;RPS19;CLP1;RPS8;RPS20;SEPP1;TPT1
Fenofibrate-430_mg/kg_in_Corn_Oil-Rat-Liver-5d-dn	8/337	0,120126882	0,283438983	0	0	1,631537622	3,457565528	C1QA;RPS17;RPL32;RPS19;RPL35A;RPSA;RPL10A;SEPP1
Leflunomide-60_mg/kg_in_Corn_Oil-Rat-Kidney-3d-dn	8/337	0,120126882	0,283354096	0	0	1,631537622	3,457565528	RPS19;RPS6;RPSA;RPL10A;SEPP1;RPL39;GOLGA7;TPT1
Rabeprazole-333_uM_in_DMSO-Rat-Primary_rat_hepatocytes-1d-dn	8/337	0,120126882	0,283269259	0	0	1,631537622	3,457565528	CXCL11;RPS19;RPS8;ARL6IP5;SEPP1;RPL39;SLC16A3;TPT1
(+)-Pulegone-200_mg/kg_in_CMC-Rat-Kidney-1d-up	7/283	0,120273457	0,283530004	0	0	1,699998786	3,600575877	RPS17;RPS8;RPS20;RPS3A;RPL10A;SEPP1;TPT1
5-Fluoro-2'-Deoxyuridine-54_mg/kg_in_Saline-Rat-Bone_marrow-1d-dn	7/283	0,120273457	0,283445166	0	0	1,699998786	3,600575877	EEF1B2;FUNDC1;NKG7;RPL13A;RPS3A;RPL39;TPT1
Alfacalcidol-0.043_mg/kg_in_CMC-Rat-Liver-5d-up	7/283	0,120273457	0,283360378	0	0	1,699998786	3,600575877	C1QA;CXCL11;RPS6;RPSA;RPS3A;RPL39;TPT1
Bisphenol_A-610_mg/kg_in_Corn_Oil-Rat-Liver-3d-dn	7/283	0,120273457	0,283275641	0	0	1,699998786	3,600575877	EEF1B2;C1QA;RPL32;CLP1;RPS5;RPS6;RPL10A
Bithionol-59_mg/kg_in_Corn_Oil-Rat-Kidney-0.25d-dn	7/283	0,120273457	0,283190955	0	0	1,699998786	3,600575877	RPS19;ARL2BP;RPS8;RPS6;RPSA;RPL10A;RPL39
Catechol-195_mg/kg_in_Saline-Rat-Liver-3d-up	7/283	0,120273457	0,283106319	0	0	1,699998786	3,600575877	EEF1B2;C1QA;RPS17;RPS19;RPL35A;RPS20;RPL39
Cetylpyridinium_Bromide-119_mg/kg_in_Water-Rat-Liver-1d-dn	7/283	0,120273457	0,283021734	0	0	1,699998786	3,600575877	EEF1B2;RPS17;RPS19;RPS8;RPS3A;SEPP1;TPT1
Clofibric_Acid-448_mg/kg_in_Corn_Oil-Rat-Liver-3d-dn	7/283	0,120273457	0,2829372	0	0	1,699998786	3,600575877	EEF1B2;RPL32;RPS8;RPS5;RPL35A;SEPP1;TPT1
Danazol-2000_mg/kg_in_CMC-Rat-Liver-3d-dn	7/283	0,120273457	0,282852716	0	0	1,699998786	3,600575877	EEF1B2;C1QA;RPS8;RPS6;RPL35A;SEPP1;TPT1
Doxycycline-14_mg/kg_in_Water-Rat-Liver-1d-dn	7/283	0,120273457	0,282768282	0	0	1,699998786	3,600575877	EEF1B2;RPS19;RPS8;RPS3A;RPL10A;SEPP1;TPT1
Norfloxacin-1500_mg/kg_in_CMC-Rat-Kidney-1d-dn	7/283	0,120273457	0,282683899	0	0	1,699998786	3,600575877	EEF1B2;RPS8;RPL35A;RPSA;RPS3A;RPL10A;TOMM20
Prochlorperazine-85_mg/kg_in_CMC-Rat-Heart-5d-up	7/283	0,120273457	0,282599566	0	0	1,699998786	3,600575877	RPS17;RPS19;RPS6;RPSA;RPL39;GOLGA7;SEPP1
Ranitidine-1500_mg/kg_in_CMC-Rat-Heart-5d-up	7/283	0,120273457	0,282515283	0	0	1,699998786	3,600575877	RPS17;RPS19;RPS6;RPSA;RPS3A;RAPGEF5;SEPP1
Tacrine-24_mg/kg_in_Water-Rat-Heart-5d-dn	7/283	0,120273457	0,282431051	0	0	1,699998786	3,600575877	C1QA;RPS19;COL5A3;RPSA;RPL10A;RPL39;SEPP1
Busulfan-36_mg/kg_in_Corn_Oil-Rat-Liver-0.25d-up	8/338	0,121588154	0,285433175	0	0	1,626710588	3,427667467	RPL32;RPS8;RPS6;ARL6IP5;RPS20;RPSA;SEPP1;RPL39
Genistein-20_mg/kg_in_Corn_Oil-Rat-Liver-5d-dn	8/338	0,121588154	0,285348124	0	0	1,626710588	3,427667467	EEF1B2;RPS17;RPL32;RPS8;RPL35A;RPS3A;SEPP1;TPT1
1-Naphthyl_Isothiocyanate-15_mg/kg_in_Corn_Oil-Rat-Liver-1d-dn	7/284	0,121880222	0,285948355	0	0	1,694012874	3,565416854	RPL32;RPS19;RPS6;RPS20;RPL10A;RPL39;TPT1
Altretamine-13_mg/kg_in_Corn_Oil-Rat-Liver-1d-dn	7/284	0,121880222	0,285863201	0	0	1,694012874	3,565416854	C1QA;RPS8;RPS6;RPS20;RPL10A;RPL39;SEPP1
Azathioprine-20_mg/kg_in_Corn_Oil-Rat-Liver-3d-dn	7/284	0,121880222	0,285778097	0	0	1,694012874	3,565416854	RPS17;RPS19;RPS6;RPS20;RPSA;RPL10A;TPT1
Betahistine-1500_mg/kg_in_Water-Rat-Kidney-1d-dn	7/284	0,121880222	0,285693044	0	0	1,694012874	3,565416854	EEF1B2;RPL32;ARL2BP;RPS8;FUNDC1;R3HCC1;RPS3A
Carboplatin-5_mg/kg_in_Saline-Rat-Kidney-28d-up	7/284	0,121880222	0,285608042	0	0	1,694012874	3,565416854	EEF1B2;RPL32;RPS19;ARL2BP;RPS8;RPS20;RPL39
Catechol-40_mg/kg_in_Saline-Rat-Bone_marrow-3d-up	7/284	0,121880222	0,28552309	0	0	1,694012874	3,565416854	RPS17;RPS19;RPS8;FEM1B;RPL35A;RPS3A;RPL10A
Cerivastatin-7_mg/kg_in_Corn_Oil-Rat-Liver-3d-up	7/284	0,121880222	0,285438189	0	0	1,694012874	3,565416854	CXADR;RPS19;RPS8;RPS6;RPS20;RPSA;RPL10A
Cyclosporin_A-70_mg/kg_in_Corn_Oil-Rat-Spleen-3d-up	7/284	0,121880222	0,285353338	0	0	1,694012874	3,565416854	EEF1B2;RPL32;RPS6;RPS20;RPSA;RPS3A;RPL10A
Dipyrone-1636_mg/kg_in_Water-Rat-Liver-3d-dn	7/284	0,121880222	0,285268538	0	0	1,694012874	3,565416854	EEF1B2;C1QA;RPS17;CLP1;RPS8;RPL35A;TPT1
Ethinylestradiol-1480_mg/kg_in_Corn_Oil-Rat-Liver-3d-up	7/284	0,121880222	0,285183787	0	0	1,694012874	3,565416854	RPS19;RPS8;FEM1B;RPS6;RPS20;RPSA;RPS3A
Gemfibrozil-100_mg/kg_in_Corn_Oil-Rat-Liver-3d-dn	7/284	0,121880222	0,285099088	0	0	1,694012874	3,565416854	C1QA;RPS17;CLP1;RPS3A;RPL10A;RPL39;SEPP1
Glyburide-1500_mg/kg_in_CMC-Rat-Heart-5d-up	7/284	0,121880222	0,285014438	0	0	1,694012874	3,565416854	RPS17;RPL32;RPS8;RPS6;RPL35A;RPS3A;TPT1
Metronidazole-50_mg/kg_in_Water-Rat-Liver-1d-dn	7/284	0,121880222	0,284929839	0	0	1,694012874	3,565416854	C1QA;RPS17;FEM1B;RPS20;RPL10A;RPL39;SEPP1
Oxymetazoline-0.5_mg/kg_in_Water-Rat-Liver-3d-up	7/284	0,121880222	0,28484529	0	0	1,694012874	3,565416854	RPL32;RPS5;RPS6;RPS3A;RPL10A;RPL39;TPT1
Sulfisoxazole-250_mg/kg_in_Corn_Oil-Rat-Liver-0.25d-up	7/284	0,121880222	0,284760792	0	0	1,694012874	3,565416854	C1QA;RPS19;RPS6;RPL35A;RPS3A;RPL10A;SEPP1
Tetracycline-1500_mg/kg_in_Water-Rat-Liver-3d-dn	7/284	0,121880222	0,284676343	0	0	1,694012874	3,565416854	EEF1B2;C1QA;RPS19;CLP1;RPS20;RPL10A;RPL39
Tranilast-300_mg/kg_in_CMC-Rat-Heart-5d-up	7/284	0,121880222	0,284591944	0	0	1,694012874	3,565416854	EEF1B2;CLP1;RPS8;FUNDC1;RAPGEF5;RPL39;TPT1
Clomipramine-7_uM_in_DMSO-Rat-Primary_rat_hepatocytes-0.67d-up	9/394	0,122736052	0,286505379	0	0	1,569940866	3,293295012	EEF1B2;RPS19;RPS8;RPS5;RPS6;RPL39;GOLGA7;TPT1;PDRG1
Acetazolamide-250_mg/kg_in_Corn_Oil-Rat-Kidney-3d-dn	8/339	0,123058852	0,287173783	0	0	1,621912032	3,398055839	RPS19;CLP1;RPS8;FEM1B;RPS6;RPL10A;SEPP1;TPT1
Hydroxyurea-400_mg/kg_in_Saline-Rat-Bone_marrow-3d-dn	8/339	0,123058852	0,28708872	0	0	1,621912032	3,398055839	EEF1B2;RPS17;RPS19;RPS6;RPSA;RPS3A;RPL10A;RPL39
Lincomycin-1200_mg/kg_in_CMC-Rat-Kidney-3d-up	8/339	0,123058852	0,287003707	0	0	1,621912032	3,398055839	RPS17;RPS8;RPS6;RPS20;RPSA;SEPP1;RPL39;TPT1
Pramoxine-526_mg/kg_in_Corn_Oil-Rat-Heart-1d-dn	8/339	0,123058852	0,286918745	0	0	1,621912032	3,398055839	RPL32;RPS8;RPS6;RPS20;RPSA;RPL10A;SEPP1;TPT1
Sodium_Arsenite-30_mg/kg_in_Saline-Rat-Bone_marrow-1d-up	8/339	0,123058852	0,286833832	0	0	1,621912032	3,398055839	EEF1B2;C1QA;RPL32;RPS19;RPS5;RPS6;RPL35A;RPS20
4-Nitrotoluene-750_mg/kg_in_CMC-Rat-Kidney-1d-up	7/285	0,123498022	0,287772313	0	0	1,68806897	3,530647134	RPS17;CLP1;RPS8;ARL6IP5;RPS3A;SEPP1;TPT1
Carvedilol-20_mg/kg_in_Corn_Oil-Rat-Heart-0.25d-up	7/285	0,123498022	0,287687198	0	0	1,68806897	3,530647134	RPS17;RPL32;RPS8;RPS20;RPL39;SEPP1;TPT1
Chlorambucil-4.5_mg/kg_in_Corn_Oil-Rat-Heart-3d-dn	7/285	0,123498022	0,287602134	0	0	1,68806897	3,530647134	RPS17;RPL32;RPS8;FUNDC1;RPS20;RPS3A;TPT1
Doxorubicin-0.65_mg/kg_in_Saline-Rat-Spleen-1d-up	7/285	0,123498022	0,28751712	0	0	1,68806897	3,530647134	C1QA;RPS17;RPS19;RPS6;RPS20;RPL10A;RPL39
Indinavir-1000_mg/kg_in_CMC-Rat-Kidney-5d-dn	7/285	0,123498022	0,287432156	0	0	1,68806897	3,530647134	RPS19;ARL2BP;RPS3A;RPL10A;RPL39;SEPP1;TPT1
Melphalan-12_mg/kg_in_CMC-Rat-Spleen-1d-dn	7/285	0,123498022	0,287347243	0	0	1,68806897	3,530647134	EEF1B2;C1QA;RPS17;RPL32;RPS6;NKG7;RPS3A
Miconazole-200_mg/kg_in_Corn_Oil-Rat-Liver-3d-up	7/285	0,123498022	0,28726238	0	0	1,68806897	3,530647134	EEF1B2;RPL32;RPS8;RPS6;RPL35A;RPS20;RPSA
Sulfinpyrazone-269_mg/kg_in_CMC-Rat-Kidney-3d-dn	7/285	0,123498022	0,287177566	0	0	1,68806897	3,530647134	EEF1B2;RPL32;CLP1;RPS8;ARL6IP5;RPL35A;SEPP1
Tosufloxacin-2000_mg/kg_in_CMC-Rat-Liver-5d-up	7/285	0,123498022	0,287092803	0	0	1,68806897	3,530647134	RPS17;RPS19;RPS6;RPS20;RPSA;RPL39;SEPP1
Trans-Platinum(II)Diammine_Dichloride-0.5_mg/kg_in_Saline-Rat-Kidney-5d-dn	7/285	0,123498022	0,28700809	0	0	1,68806897	3,530647134	RPS17;RPS19;ARL2BP;RPS8;RPS20;RPSA;SEPP1
Amantadine-1500_uM_in_DMSO-Rat-Primary_rat_hepatocytes-1d-dn	8/340	0,124538943	0,289341804	0	0	1,617141702	3,368727417	CXCL11;RPL32;RPS19;ARL6IP5;SEPP1;RPL39;SLC16A3;TPT1
Ergocalciferol-15_mg/kg_in_CMC-Rat-Liver-3d-dn	8/340	0,124538943	0,289256478	0	0	1,617141702	3,368727417	RPS17;RPL32;RPS19;RPL35A;RPS20;RPSA;RPL10A;SEPP1
(+)-Pulegone-200_mg/kg_in_CMC-Rat-Kidney-5d-up	7/286	0,125126806	0,290536181	0	0	1,682166631	3,49626156	EEF1B2;RPS17;RPS8;ARL6IP5;RPS20;RPSA;RAPGEF5
Amoxicillin-1100_mg/kg_in_Water-Rat-Liver-1d-dn	7/286	0,125126806	0,290450553	0	0	1,682166631	3,49626156	EEF1B2;RPL32;RPS19;RPS6;RPS20;RPS3A;SEPP1
Anastrozole-400_mg/kg_in_CMC-Rat-Liver-5d-dn	7/286	0,125126806	0,290364976	0	0	1,682166631	3,49626156	EEF1B2;C1QA;RPS17;RPL32;RPS8;RPS3A;SEPP1
Azathioprine-54_mg/kg_in_Water-Rat-Bone_marrow-3d-dn	7/286	0,125126806	0,290279448	0	0	1,682166631	3,49626156	ARL2BP;CLP1;RPS8;FEM1B;RPS6;RPL39;TPT1
Benzothiazyl_Disulfide-2600_mg/kg_in_Corn_Oil-Rat-Kidney-1d-dn	7/286	0,125126806	0,290193972	0	0	1,682166631	3,49626156	RPS19;RPS8;RPS6;RPL10A;RPL39;SEPP1;PDRG1
Carboplatin-14_mg/kg_in_Saline-Rat-Spleen-5d-up	7/286	0,125126806	0,290108545	0	0	1,682166631	3,49626156	CXCL11;RPL32;RPS19;RPS6;RAPGEF5;RPL39;SEPP1
Cerivastatin-0.05_mg/kg_in_Corn_Oil-Rat-Liver-1d-dn	7/286	0,125126806	0,290023169	0	0	1,682166631	3,49626156	EEF1B2;RPS17;RPS8;RPL35A;RPS20;RPSA;RPS3A
Chlorpromazine-18_mg/kg_in_Water-Rat-Liver-3d-dn	7/286	0,125126806	0,289937843	0	0	1,682166631	3,49626156	RPS17;RPL32;RPS19;RPS6;RPS20;RPSA;RPL10A
Clotrimazole-178_mg/kg_in_Corn_Oil-Rat-Liver-5d-up	7/286	0,125126806	0,289852567	0	0	1,682166631	3,49626156	RPS17;RPL32;RPS19;RPS6;RPS20;RPS3A;TPT1
Deferiprone-500_mg/kg_in_Water-Rat-Liver-3d-up	7/286	0,125126806	0,289767341	0	0	1,682166631	3,49626156	C1QA;RPS17;RPS19;ARL2BP;RPSA;RPL10A;SEPP1
Diazepam-710_mg/kg_in_CMC-Rat-Kidney-3d-dn	7/286	0,125126806	0,289682166	0	0	1,682166631	3,49626156	RPS19;ARL2BP;RPS8;RPS6;RPS20;GOLGA7;TPT1
Doxorubicin-0.65_mg/kg_in_Saline-Rat-Bone_marrow-0.25d-dn	7/286	0,125126806	0,28959704	0	0	1,682166631	3,49626156	EEF1B2;C1QA;ARL6IP5;NKG7;RPL10A;RPL39;SEPP1
Emetine-1_mg/kg_in_Saline-Rat-Heart-3d-up	7/286	0,125126806	0,289511965	0	0	1,682166631	3,49626156	ARL2BP;CLP1;RPS8;RPS20;RAPGEF5;RPL10A;RPL39
Etidronate-1005_mg/kg_in_Water-Rat-Heart-5d-up	7/286	0,125126806	0,289426939	0	0	1,682166631	3,49626156	RPS17;RPL32;RPS6;RPS3A;RAPGEF5;RPL39;TPT1
Fluphenazine-22_mg/kg_in_Corn_Oil-Rat-Liver-3d-dn	7/286	0,125126806	0,289341963	0	0	1,682166631	3,49626156	C1QA;RPS17;CLP1;RPS6;RPL35A;RPL39;TPT1
Paroxetine-104_mg/kg_in_Water-Rat-Heart-3d-dn	7/286	0,125126806	0,289257038	0	0	1,682166631	3,49626156	RPS17;RPL32;RPS19;COL5A3;RPS3A;RPL39;SEPP1
Perhexiline-320_mg/kg_in_CMC-Rat-Liver-1d-up	7/286	0,125126806	0,289172162	0	0	1,682166631	3,49626156	EEF1B2;RPS17;RPS20;RPSA;RPS3A;RPL10A;SEPP1
Rofecoxib-3_mg/kg_in_Corn_Oil-Rat-Heart-5d-dn	7/286	0,125126806	0,289087336	0	0	1,682166631	3,49626156	RPS17;RPL32;FUNDC1;RPS6;RPSA;RPS3A;SEPP1
Rolipram-75_mg/kg_in_CMC-Rat-Liver-5d-up	7/286	0,125126806	0,289002559	0	0	1,682166631	3,49626156	EEF1B2;RPS17;RPS8;RPSA;RPS3A;RPL39;TPT1
Sulfathiazole-31_mg/kg_in_Water-Rat-Liver-0.25d-dn	7/286	0,125126806	0,288917833	0	0	1,682166631	3,49626156	RPS17;RPL32;RPS8;RPL35A;RPS3A;RPL39;TPT1
Beta-Naphthoflavone-80_mg/kg_in_CMC-Rat-Liver-28d-dn	8/341	0,126028394	0,290914311	0	0	1,612399351	3,339679012	RPS17;RPS19;RPS8;RPS20;RPSA;RPL10A;RPL39;TPT1
Etoposide-188_mg/kg_in_Corn_Oil-Rat-Spleen-3d-dn	8/341	0,126028394	0,290829074	0	0	1,612399351	3,339679012	C1QA;RPS17;RPSA;RPS3A;RPL10A;SEPP1;RPL39;TPT1
Hydroxyurea-59_mg/kg_in_Saline-Rat-Bone_marrow-3d-up	8/341	0,126028394	0,290743887	0	0	1,612399351	3,339679012	EEF1B2;RPS17;RPL32;NKG7;RPS20;RPS3A;RPL10A;RPL39
Bromfenac-5_mg/kg_in_CMC-Rat-Liver-5d-dn	7/287	0,126766527	0,292361103	0	0	1,676305423	3,46225506	C1QA;RPS17;RPL32;RPS19;CLP1;RPS6;TPT1
Citalopram-150_uM_in_DMSO-Rat-Primary_rat_hepatocytes-0.67d-up	7/287	0,126766527	0,292275517	0	0	1,676305423	3,46225506	RPS6;RPSA;RPS3A;RPL10A;RPL39;TPT1;PDRG1
Ifosfamide-143_mg/kg_in_Saline-Rat-Heart-3d-dn	7/287	0,126766527	0,292189981	0	0	1,676305423	3,46225506	EEF1B2;RPS17;RPL32;ARL2BP;COL5A3;RPL35A;RPS3A

Phenothiazine-386_mg/kg_in_Corn_Oil-Rat-Liver-1d-up	7/287	0,126766527	0,292104495	0	0	1,676305423	3,46225506	RPL32;RPS19;RPS8;RPS6;RPS20;RPSA;RPL10A
Ranitidine-1500_mg/kg_in_CMC-Rat-Heart-3d-dn	7/287	0,126766527	0,29201906	0	0	1,676305423	3,46225506	C1QA;RPS19;ARL2BP;FUNDC1;RPS20;RPL10A;RPL39
Saquinavir-1500_mg/kg_in_CMC-Rat-Kidney-3d-up	7/287	0,126766527	0,291933674	0	0	1,676305423	3,46225506	EEF1B2;RPS17;RPL32;RPS19;RPS8;RPS20;RPL39
Terbinafine-2000_mg/kg_in_CMC-Rat-Liver-3d-dn	7/287	0,126766527	0,291848338	0	0	1,676305423	3,46225506	EEF1B2;C1QA;RPS19;RPS8;ARL6IP5;SEPP1;TPT1
Thioguanine-24_mg/kg_in_Corn_Oil-Rat-Bone_marrow-1d-dn	7/287	0,126766527	0,291763052	0	0	1,676305423	3,46225506	RPL32;RPS19;RPS8;RPS6;ARL6IP5;RPSA;RPS3A
Zileuton-450_mg/kg_in_Corn_Oil-Rat-Liver-5d-up	7/287	0,126766527	0,291677816	0	0	1,676305423	3,46225506	RPL32;RPS19;CLP1;RPS8;RPS20;RPSA;SEPP1
Amitriptyline-160_mg/kg_in_Water-Rat-Heart-3d-up	8/342	0,12752717	0,293342287	0	0	1,607684733	3,310907483	RPS19;RPS6;ARL6IP5;RPS20;RAPGEF5;RPL10A;RPL39;GOLGA7
Carboplatin-5_mg/kg_in_Saline-Rat-Kidney-14d-up	8/342	0,12752717	0,293256639	0	0	1,607684733	3,310907483	EEF1B2;RPL32;RPS19;ARL6IP5;RPS3A;RPL10A;RPL39;TPT1
Cobalt(II)_Acetylacetonate-100_mg/kg_in_Corn_Oil-Rat-Heart-1d-up	8/342	0,12752717	0,293171042	0	0	1,607684733	3,310907483	EEF1B2;ARL2BP;RPS8;ARL6IP5;RPSA;RPL10A;RPL39;PDRG1
Doxazosin-500_mg/kg_in_Water-Rat-Heart-1d-up	8/342	0,12752717	0,293085494	0	0	1,607684733	3,310907483	RPS17;RPL32;RPS8;FUNDC1;RPSA;RPS3A;RPL39;TPT1
Idarubicin-1.5_uM_in_DMSO-Rat-Primary_rat_hepatocytes-1d-dn	8/342	0,12752717	0,292999997	0	0	1,607684733	3,310907483	RPS19;ARL2BP;RPS8;FUNDC1;RPS20;RPSA;RPL39;TPT1
Hydroxyurea-59_mg/kg_in_Saline-Rat-Liver-1d-up	9/398	0,128239329	0,294550294	0	0	1,554162565	3,192027666	C1QA;RPS17;RPS19;RPS6;RPS20;RPSA;RPL10A;RPL39;TPT1
(+)-Pulegone-200_mg/kg_in_CMC-Rat-Kidney-3d-up	7/288	0,128417132	0,294872691	0	0	1,670484918	3,428622639	RPS17;RPS8;RPSA;RPS3A;GOLGA7;SEPP1;TPT1
Acetazolamide-47_mg/kg_in_Corn_Oil-Rat-Kidney-1d-dn	7/288	0,128417132	0,294786747	0	0	1,670484918	3,428622639	ARL2BP;CLP1;RPS8;RPL10A;RPL39;SEPP1;TPT1
Acyclovir-980_mg/kg_in_Saline-Rat-Bone_marrow-5d-up	7/288	0,128417132	0,294700853	0	0	1,670484918	3,428622639	C1QA;RPS19;ARL2BP;CLP1;RPS8;FUNDC1;TPT1
Amitraz-75_mg/kg_in_CMC-Rat-Liver-1d-up	7/288	0,128417132	0,29461501	0	0	1,670484918	3,428622639	CXCL11;RPS8;RPS6;RPSA;RPS3A;RPL10A;TPT1
Bromisovalum-250_mg/kg_in_Corn_Oil-Rat-Liver-3d-dn	7/288	0,128417132	0,294529216	0	0	1,670484918	3,428622639	EEF1B2;C1QA;RPL32;RPS6;RPL35A;SEPP1;TPT1
Clotrimazole-89_mg/kg_in_Corn_Oil-Rat-Liver-3d-up	7/288	0,128417132	0,294443473	0	0	1,670484918	3,428622639	RPS17;RPS19;RPS8;RPS20;RPSA;RPS3A;RPL39
Emetine-1_mg/kg_in_Saline-Rat-Heart-1d-dn	7/288	0,128417132	0,294357779	0	0	1,670484918	3,428622639	RPS17;RPL32;RPS19;RPS6;RPS3A;SEPP1;TPT1
Epirubicin-2.7_mg/kg_in_Saline-Rat-Heart-5d-up	7/288	0,128417132	0,294272135	0	0	1,670484918	3,428622639	C1QA;CXCL11;RPS19;RPS6;RPS20;RPL10A;RPL39
Fluvastatin-5_mg/kg_in_Corn_Oil-Rat-Kidney-1d-up	7/288	0,128417132	0,294186541	0	0	1,670484918	3,428622639	RPS19;RPS8;RPS5;RPS6;RPS20;SEPP1;TPT1
Lead_(II)_Acetate-600_mg/kg_in_Saline-Rat-Liver-5d-dn	7/288	0,128417132	0,294100997	0	0	1,670484918	3,428622639	EEF1B2;RPS17;RPL32;RPS19;CLP1;RPL39;SEPP1
Mevastatin-1200_mg/kg_in_CMC-Rat-Skeletal_muscle-5d-dn	7/288	0,128417132	0,294015503	0	0	1,670484918	3,428622639	RPS19;CLP1;FEM1B;RPS3A;RPL10A;RPL39;TPT1
N-Nitrosodiethylamine-34_mg/kg_in_Saline-Rat-Liver-3d-dn	7/288	0,128417132	0,293930058	0	0	1,670484918	3,428622639	RPS17;RPL32;RPS8;RPS20;RPS3A;RPL39;TPT1
Phentolamine-44_mg/kg_in_Saline-Rat-Heart-1d-up	7/288	0,128417132	0,293844663	0	0	1,670484918	3,428622639	RPS17;RPS6;RPS3A;RPL10A;RPL39;SEPP1;TPT1
Pravastatin-1200_mg/kg_in_Corn_Oil-Rat-Liver-5d-up	7/288	0,128417132	0,293759317	0	0	1,670484918	3,428622639	CXADR;RPS19;RPS6;RPS20;RPSA;RPL10A;RPL39
Valproic_Acid-235_mg/kg_in_Water-Rat-Kidney-1d-dn	7/288	0,128417132	0,293674021	0	0	1,670484918	3,428622639	RPL32;ARL2BP;RPS8;FEM1B;ARL6IP5;RPS20;RPS3A
Azithromycin-50_mg/kg_in_Water-Rat-Liver-0.25d-up	8/343	0,129035236	0,295001892	0	0	1,602997606	3,282409729	RPS17;RPS19;RPS20;RPS3A;RPL10A;SEPP1;RPL39;TPT1
Cyclosporin_A-8.5_uM_in_DMSO-Rat-Primary_rat_hepatocytes-0.67d-up	8/343	0,129035236	0,294916285	0	0	1,602997606	3,282409729	EEF1B2;RPS19;RPS8;RPS6;RPL10A;RPL39;TPT1;PDRG1
Fluoxetine-39.6_uM_in_DMSO-Rat-Primary_rat_hepatocytes-1d-dn	8/343	0,129035236	0,294830728	0	0	1,602997606	3,282409729	CXCL11;RPS19;CLP1;ARL6IP5;RPS20;SEPP1;RPL39;TPT1
Oxiconazole-1500_mg/kg_in_Corn_Oil-Rat-Liver-3d-dn	8/343	0,129035236	0,29474522	0	0	1,602997606	3,282409729	RPS17;RPL32;RPS19;RPS6;RPS20;RPS3A;RPL10A;GOLGA7
17-Methyltestosterone-2000_mg/kg_in_CMC-Rat-Liver-5d-dn	7/289	0,130078569	0,297042275	0	0	1,664704693	3,395359387	EEF1B2;RPS17;RPL32;RPS8;RPL35A;SEPP1;TPT1
Amitriptyline-72_uM_in_DMSO-Rat-Primary_rat_hepatocytes-0.67d-up	7/289	0,130078569	0,296956176	0	0	1,664704693	3,395359387	RPS17;RPL32;RPS5;RPS20;RPS3A;RPL10A;PDRG1
Carmustine-4_mg/kg_in_Corn_Oil-Rat-Liver-5d-dn	7/289	0,130078569	0,296870127	0	0	1,664704693	3,395359387	EEF1B2;C1QA;RPS17;RPL32;RPS19;RPL35A;SEPP1
Celecoxib-400_mg/kg_in_Corn_Oil-Rat-Heart-5d-up	7/289	0,130078569	0,296784127	0	0	1,664704693	3,395359387	C1QA;RPS19;RPS8;ARL6IP5;RPS20;RPL39;TPT1
Dexamethasone-1_mg/kg_in_Corn_Oil-Rat-Heart-5d-up	7/289	0,130078569	0,296698178	0	0	1,664704693	3,395359387	EEF1B2;FUNDC1;ARL6IP5;RPS20;RAPGEF5;SEPP1;PDRG1
Doxorubicin-3_mg/kg_in_Saline-Rat-Kidney-5d-up	7/289	0,130078569	0,296612278	0	0	1,664704693	3,395359387	RPS8;RPS6;RPS20;RPS3A;RPL10A;RPL39;TPT1
Dyphylline-3855_mg/kg_in_CMC-Rat-Heart-5d-up	7/289	0,130078569	0,296526428	0	0	1,664704693	3,395359387	RPS17;RPL32;RPS19;RPS6;RPL35A;RPSA;RPS3A
Glimepiride-2500_mg/kg_in_CMC-Rat-Liver-5d-up	7/289	0,130078569	0,296440627	0	0	1,664704693	3,395359387	C1QA;RPS17;ARL2BP;CLP1;FEM1B;RPS20;RPSA
Hydroxyurea-400_mg/kg_in_Saline-Rat-Bone_marrow-1d-dn	7/289	0,130078569	0,296354876	0	0	1,664704693	3,395359387	RPS17;RPL32;FEM1B;RPSA;RPS3A;RPL39;TPT1
Ifosfamide-143_mg/kg_in_Saline-Rat-Bone_marrow-3d-dn	7/289	0,130078569	0,296269175	0	0	1,664704693	3,395359387	RPS6;NKG7;RPSA;RPS3A;RPL10A;RPL39;TPT1
Nevirapine-200_mg/kg_in_Saline-Rat-Heart-3d-dn	7/289	0,130078569	0,296183523	0	0	1,664704693	3,395359387	EEF1B2;RPS17;RPL32;RPS19;ARL2BP;RPL35A;RPS3A
Thiabendazole-10_mg/kg_in_Corn_Oil-Rat-Liver-0.25d-dn	7/289	0,130078569	0,296097921	0	0	1,664704693	3,395359387	RPS17;RPS8;RPS20;RPSA;RPS3A;RPL39;TPT1
Cytarabine-23_mg/kg_in_Saline-Rat-Spleen-0.25d-up	8/344	0,130552557	0,297090996	0	0	1,598337729	3,254182691	C1QA;RPS17;RPL32;RPS6;ARL6IP5;RPSA;RPL10A;SEPP1
Busulfan-36_mg/kg_in_Corn_Oil-Rat-Liver-1d-dn	7/290	0,131750785	0,299731133	0	0	1,658964332	3,362460468	EEF1B2;RPS17;RPS19;RPS8;RPS3A;RPL10A;TPT1
Cilostazol-1500_mg/kg_in_CMC-Rat-Heart-3d-dn	7/290	0,131750785	0,29964458	0	0	1,658964332	3,362460468	EEF1B2;RPS19;ARL2BP;RPS8;ARL6IP5;RPS20;RPS3A
Clofibrate-130_mg/kg_in_Corn_Oil-Rat-Liver-3d-dn	7/290	0,131750785	0,299558078	0	0	1,658964332	3,362460468	C1QA;RPS19;RPS8;RPS6;RPSA;RPL10A;SEPP1
Clopidogrel-400_mg/kg_in_Corn_Oil-Rat-Bone_marrow-1d-dn	7/290	0,131750785	0,299471625	0	0	1,658964332	3,362460468	RPS19;FEM1B;NKG7;RPL35A;RPSA;RPS3A;TPT1
Daunorubicin-4_mg/kg_in_Saline-Rat-Heart-5d-dn	7/290	0,131750785	0,299385222	0	0	1,658964332	3,362460468	RPS17;RPL32;RPS6;RPS3A;RAPGEF5;GOLGA7;TPT1
Dexchlorpheniramine-3_mg/kg_in_Water-Rat-Heart-5d-dn	7/290	0,131750785	0,29929887	0	0	1,658964332	3,362460468	RPS17;RPL32;RPS19;ARL2BP;ARL6IP5;RPS20;RPS3A
Diclofenac-10_mg/kg_in_Corn_Oil-Rat-Kidney-1d-dn	7/290	0,131750785	0,299212567	0	0	1,658964332	3,362460468	EEF1B2;RPS17;RPL32;RPS8;RPL35A;RPS20;RPS3A
Gadopentetate_Dimeglumine-125_mg/kg_in_Saline-Rat-Kidney-5d-up	7/290	0,131750785	0,299126313	0	0	1,658964332	3,362460468	EEF1B2;RPS17;RPL32;ARL6IP5;RPL35A;RPSA;RPL39
Glipizide-2500_mg/kg_in_CMC-Rat-Liver-1d-up	7/290	0,131750785	0,29904011	0	0	1,658964332	3,362460468	CXCL11;RPS19;RPS8;RPS5;RPSA;RPL10A;TPT1
Noscapine-570_mg/kg_in_CMC-Rat-Spleen-3d-up	7/290	0,131750785	0,298953956	0	0	1,658964332	3,362460468	RPS8;RPS5;FUNDC1;RPSA;RPL10A;SEPP1;TPT1
Nystatin-134_mg/kg_in_Corn_Oil-Rat-Kidney-1d-dn	7/290	0,131750785	0,298867852	0	0	1,658964332	3,362460468	EEF1B2;RPS17;RPL32;CLP1;RPS8;RPL35A;RPS3A
Pantoprazole-650_uM_in_DMSO-Rat-Primary_rat_hepatocytes-0.67d-dn	7/290	0,131750785	0,298781797	0	0	1,658964332	3,362460468	CXCL11;ARL6IP5;RPS20;RPSA;RPL10A;SEPP1;SLC16A3
Roxithromycin-312_mg/kg_in_CMC-Rat-Liver-1d-dn	7/290	0,131750785	0,298695792	0	0	1,658964332	3,362460468	EEF1B2;RPS19;RPS6;RPS20;RPS3A;RPL10A;SEPP1
Sodium_Arsenite-10_mg/kg_in_Saline-Rat-Liver-0.25d-up	7/290	0,131750785	0,298609836	0	0	1,658964332	3,362460468	C1QA;RPS17;RPS19;RPS20;RPSA;RPS3A;SEPP1
Cyclophosphamide-1320_uM_in_DMSO-Rat-Primary_rat_hepatocytes-1d-dn	8/345	0,132079095	0,299267822	0	0	1,593704866	3,226223352	RPS17;RPL32;ARL2BP;RPS8;RPS5;RPS20;RPS3A;TPT1
Cyclosporin_A-350_mg/kg_in_Corn_Oil-Rat-Liver-0.25d-dn	6/237	0,132952142	0,301159352	0	0	1,739962591	3,51083745	C1QA;RPS17;RPL32;RPS3A;RPL39;TPT1
N-Nitrosodiethylamine-1.67_mg/kg_in_Saline-Rat-Kidney-0.25d-dn	6/237	0,132952142	0,301072762	0	0	1,739962591	3,51083745	ARL2BP;RPS8;RPL35A;RPS3A;RPL39;TPT1
Roxarsone-11_mg/kg_in_Corn_Oil-Rat-Kidney-5d-up	6/237	0,132952142	0,300986222	0	0	1,739962591	3,51083745	RPS17;RPL32;RPS19;RPS6;ARL6IP5;RPS3A
Aconitine-0.9_mg/kg_in_CMC-Rat-Heart-1d-dn	7/291	0,133433726	0,301989662	0	0	1,653263424	3,329921124	RPL32;RPS19;ARL2BP;RPS8;RPSA;RPL10A;GOLGA7
Amikacin-82_mg/kg_in_Saline-Rat-Bone_marrow-1d-dn	7/291	0,133433726	0,301902908	0	0	1,653263424	3,329921124	EEF1B2;RPS8;FUNDC1;NKG7;RPSA;RPS3A;RPL39
Clonazepam-2500_mg/kg_in_CMC-Rat-Liver-5d-up	7/291	0,133433726	0,301816204	0	0	1,653263424	3,329921124	RPS17;RPS8;FEM1B;RPS20;RPSA;RPL39;TPT1
Clopidogrel-400_mg/kg_in_Corn_Oil-Rat-Bone_marrow-0.25d-up	7/291	0,133433726	0,30172955	0	0	1,653263424	3,329921124	C1QA;RPL32;ARL2BP;RPS8;FUNDC1;RPS6;RPS3A
Fluphenazine-2.5_mg/kg_in_Corn_Oil-Rat-Liver-3d-dn	7/291	0,133433726	0,301642946	0	0	1,653263424	3,329921124	C1QA;RPS17;RPS6;RPL35A;RPSA;RPL10A;SEPP1
Isoniazid-79_mg/kg_in_Water-Rat-Liver-3d-dn	7/291	0,133433726	0,301556391	0	0	1,653263424	3,329921124	EEF1B2;C1QA;RPS19;CLP1;RPS20;RPL39;SEPP1
Myrtecaine-140_mg/kg_in_Corn_Oil-Rat-Heart-3d-dn	7/291	0,133433726	0,301469886	0	0	1,653263424	3,329921124	EEF1B2;RPS17;RPL32;RPS8;RPSA;RPS3A;TPT1
Norfloxacin-1500_mg/kg_in_CMC-Rat-Kidney-3d-up	7/291	0,133433726	0,301383431	0	0	1,653263424	3,329921124	RPS17;RPL32;ARL2BP;RPS6;RPS20;RPL39;TPT1
Nystatin-134_mg/kg_in_Corn_Oil-Rat-Liver-3d-up	7/291	0,133433726	0,301297025	0	0	1,653263424	3,329921124	EEF1B2;C1QA;RPS17;RPS3A;RAPGEF5;RPL39;SEPP1
Tamoxifen-32_mg/kg_in_Corn_Oil-Rat-Liver-5d-up	7/291	0,133433726	0,301210668	0	0	1,653263424	3,329921124	EEF1B2;RPS17;CXCL11;RPL32;RPS19;RPSA;RPS3A
Thioguanine-12_mg/kg_in_Corn_Oil-Rat-Bone_marrow-3d-dn	7/291	0,133433726	0,301124362	0	0	1,653263424	3,329921124	RPS19;FEM1B;RPS6;RPSA;RPS3A;RPL10A;RPL39
Nystatin-134_mg/kg_in_Corn_Oil-Rat-Kidney-5d-up	8/346	0,133614813	0,301446652	0	0	1,589098782	3,198528736	C1QA;ARL2BP;RPS6;RPS20;RAPGEF5;SEPP1;RPL39;TPT1
Stanozolol-39_mg/kg_in_Water-Rat-Liver-0.25d-up	8/346	0,133614813	0,301360327	0	0	1,589098782	3,198528736	C1QA;RPS19;RPS6;RPS20;RPSA;RPS3A;RPL10A;SEPP1
Bisphenol_A-150_mg/kg_in_Corn_Oil-Rat-Liver-5d-dn	6/238	0,134840425	0,304037557	0	0	1,732651824	3,471650764	RPS17;RPL32;RPS8;RPS20;RPS3A;TPT1
Chlorobenzene-500_mg/kg_in_CMC-Rat-Heart-3d-dn	6/238	0,134840425	0,30395054	0	0	1,732651824	3,471650764	RPS19;COL5A3;RPS6;RPS20;SEPP1;TPT1
4-Chloroaniline-75_mg/kg_in_CMC-Rat-Kidney-1d-dn	7/292	0,135127335	0,304510127	0	0	1,647601563	3,297736674	RPL32;RPS19;FEM1B;RPS6;RPL10A;SEPP1;RPL39
Amikacin-82_mg/kg_in_Saline-Rat-Kidney-3d-up	7/292	0,135127335	0,304423025	0	0	1,647601563	3,297736674	EEF1B2;ARL2BP;CLP1;RPS8;RPS6;RPS3A;SEPP1
Beta-Estradiol-0.3_mg/kg_in_Corn_Oil-Rat-Liver-0.25d-up	7/292	0,135127335	0,304335972	0	0	1,647601563	3,297736674	EEF1B2;RPL32;RPS6;RPL10A;SEPP1;RPL39;PDRG1
Bithionol-333_mg/kg_in_Corn_Oil-Rat-Liver-1d-dn	7/292	0,135127335	0,304248969	0	0	1,647601563	3,297736674	RPS17;RPL32;CLP1;RPS8;RPSA;RPS3A;RPL10A
Chloramphenicol-1500_mg/kg_in_CMC-Rat-Kidney-1d-up	7/292	0,135127335	0,304162016	0	0	1,647601563	3,297736674	EEF1B2;RPL32;RPS8;RPS5;RPS6;RPL35A;RPS20
Clozapine-95_mg/kg_in_CMC-Rat-Heart-3d-up	7/292	0,135127335	0,304075113	0	0	1,647601563	3,297736674	RPS17;CXCL11;RPL32;RPS19;RPS3A;RAPGEF5;SEPP1
Diethylstilbestrol-2.8_mg/kg_in_Corn_Oil-Rat-Kidney-1d-up	7/292	0,135127335	0,303988259	0	0	1,647601563	3,297736674	RPS17;RPS19;ARL2BP;RPS5;RPS6;RPS20;RPS3A
Digitoxin-2.75_mg/kg_in_Corn_Oil-Rat-Heart-1d-dn	7/292	0,135127335	0,303901455	0	0	1,647601563	3,297736674	RPS19;ARL2BP;RPS8;FUNDC1;ARL6IP5;RPS20;RPSA
Ethisterone-1500_mg/kg_in_CMC-Rat-Liver-5d-dn	7/292	0,135127335	0,3038147	0	0	1,647601563	3,297736674	EEF1B2;RPL32;RPS19;RPS8;RPS6;RPS3A;TPT1
Idebenone-2500_mg/kg_in_CMC-Rat-Liver-5d-up	7/292	0,135127335	0,303727995	0	0	1,647601563	3,297736674	RPS17;RPS8;RPL35A;RPS20;RPSA;RPL39;TPT1
Niacinamide-750_mg/kg_in_Water-Rat-Liver-5d-up	7/292	0,135127335	0,303641339	0	0	1,647601563	3,297736674	C1QA;RPS17;RPS8;ARL6IP5;RPSA;RPS3A;SEPP1
Tretinoin-7_mg/kg_in_Corn_Oil-Rat-Kidney-3d-dn	7/292	0,135127335	0,303554733	0	0	1,647601563	3,297736674	RPS17;RPL32;RPS19;RPS6;RPS20;RPL39;GOLGA7
Lomustine-4.2_mg/kg_in_Corn_Oil-Rat-Spleen-3d-dn	8/347	0,135159672	0,303540797	0	0	1,584519247	3,171095905	C1QA;RPS17;RPSA;RPL10A;SEPP1;RPL39;GOLGA7;TPT1

Mexiletine-350_mg/kg_in_CMC-Rat-Heart-3d-dn	8/347	0,135159672	0,303454269	0	0	1,584519247	3,171095905	EEF1B2;ARL2BP;FUNDC1;R3HCC1;ARL6IP5;RPSA;RPL39;TPT1
Phentolamine-44_mg/kg_in_Saline-Rat-Heart-3d-up	8/347	0,135159672	0,30336779	0	0	1,584519247	3,171095905	RPS17;RPL32;RPS19;RPS6;ARL6IP5;RPS3A;RPL39;PDRG1
Olanzapine-250_uM_in_DMSO-Rat-Primary_rat_hepatocytes-0.67d-dn	5/186	0,135694339	0,304481086	0	0	1,847540923	3,690186659	RPS17;RPL32;ARL6IP5;RPSA;RPS3A
Citalopram-150_uM_in_DMSO-Rat-Primary_rat_hepatocytes-1d-dn	8/348	0,136713633	0,306680882	0	0	1,579966031	3,143921962	C1QA;CXCL11;RPS19;ARL6IP5;SEPP1;RPL39;SLC16A3;TPT1
Haloperidol-77_uM_in_DMSO-Rat-Primary_rat_hepatocytes-1d-dn	8/348	0,136713633	0,306593558	0	0	1,579966031	3,143921962	CXCL11;RPS19;RPS8;RPS6;SEPP1;RPL39;SLC16A3;TPT1
Harringtonine-23_mg/kg_in_Saline-Rat-Bone_marrow-3d-up	8/348	0,136713633	0,306506284	0	0	1,579966031	3,143921962	RPS17;RPL32;RPS19;RPS5;RPSA;RPL10A;SEPP1;TPT1
Isotretinoin-13_mg/kg_in_Corn_Oil-Rat-Liver-1d-dn	8/348	0,136713633	0,30641906	0	0	1,579966031	3,143921962	EEF1B2;RPS17;RPL32;RPS19;RPS6;RPSA;RPL10A;SEPP1
Rosiglitazone-1800_mg/kg_in_Corn_Oil-Rat-Liver-3d-up	8/348	0,136713633	0,306331885	0	0	1,579966031	3,143921962	RPL32;RPS19;RPS6;RPL35A;RPS20;RPSA;RPL10A;RPL39
4-Octylphenol-700_mg/kg_in_Corn_Oil-Rat-Kidney-5d-up	7/293	0,136831558	0,306508917	0	0	1,641978349	3,26590251	EEF1B2;RPS19;RPS5;RPS20;RPSA;RPL10A;TPT1
Clofibrate-500_mg/kg_in_Corn_Oil-Rat-Liver-3d-up	7/293	0,136831558	0,306421766	0	0	1,641978349	3,26590251	RPL32;RPS19;RPS8;RPS6;RPS20;RPS3A;TPT1
Ergocalciferol-15_mg/kg_in_CMC-Rat-Kidney-1d-dn	7/293	0,136831558	0,306334665	0	0	1,641978349	3,26590251	RPS17;RPL32;RPS19;ARL2BP;RPS6;ARL6IP5;RPL39
Fenofibrate-100_mg/kg_in_Corn_Oil-Rat-Liver-0.25d-dn	7/293	0,136831558	0,306247614	0	0	1,641978349	3,26590251	EEF1B2;RPS19;CLP1;RPSA;RPL10A;SEPP1;RPL39
Flavoxate-1255_mg/kg_in_CMC-Rat-Heart-5d-up	7/293	0,136831558	0,306160611	0	0	1,641978349	3,26590251	RPS17;RPL32;RPS8;RPS6;RPS3A;SEPP1;TPT1
Melatonin-2000_mg/kg_in_Corn_Oil-Rat-Liver-1d-up	7/293	0,136831558	0,306073659	0	0	1,641978349	3,26590251	RPS19;RPS8;RPS6;RPS20;RPSA;RPL10A;RPL39
Nevirapine-29_mg/kg_in_Saline-Rat-Liver-0.25d-up	7/293	0,136831558	0,305986755	0	0	1,641978349	3,26590251	EEF1B2;RPS17;RPL32;RPS19;RPS6;RPS3A;SEPP1
Nisoldipine-15_mg/kg_in_Water-Rat-Liver-0.25d-dn	7/293	0,136831558	0,305899901	0	0	1,641978349	3,26590251	EEF1B2;RPS17;RPL32;RPS19;RPS5;RPL35A;RPSA
Primidone-750_mg/kg_in_CMC-Rat-Liver-3d-up	7/293	0,136831558	0,305813097	0	0	1,641978349	3,26590251	RPS17;RPL32;RPS6;RPSA;RPS3A;RPL10A;RPL39
Sildenafil-2.5_mg/kg_in_Water-Rat-Liver-1d-up	7/293	0,136831558	0,305726341	0	0	1,641978349	3,26590251	EEF1B2;C1QA;RPS17;RPS19;RPS20;RPS3A;RPL39
Tiapride-1200_mg/kg_in_Water-Rat-Heart-5d-dn	7/293	0,136831558	0,305639635	0	0	1,641978349	3,26590251	RPS19;ARL2BP;RPS8;ARL6IP5;RPS20;RPSA;TPT1
Tretinoin-156_uM_in_DMSO-Rat-Primary_rat_hepatocytes-0.67d-dn	7/293	0,136831558	0,305552978	0	0	1,641978349	3,26590251	CXCL11;RPS19;RPSA;SEPP1;RPL39;SLC16A3;TPT1
Valsartan-1000_mg/kg_in_Corn_Oil-Rat-Kidney-1d-up	7/293	0,136831558	0,30546637	0	0	1,641978349	3,26590251	ARL2BP;CLP1;RPS8;RPS6;RPS20;RPS3A;TPT1
1-Naphthyl_Isothiocyanate-30_mg/kg_in_Corn_Oil-Rat-Kidney-0.25d-up	8/349	0,138276657	0,308604974	0	0	1,575438907	3,117004049	RPS17;FUNDC1;RPS6;RPL35A;RPS3A;SEPP1;RPL39;TPT1
Bisphenol_A-610_mg/kg_in_Corn_Oil-Rat-Kidney-3d-dn	8/349	0,138276657	0,30851755	0	0	1,575438907	3,117004049	EEF1B2;RPS17;RPL32;RPS19;RPS6;RPS3A;RPL39;TPT1
Camptothecin-2.5_mg/kg_in_Saline-Rat-Bone_marrow-3d-up	8/349	0,138276657	0,308430176	0	0	1,575438907	3,117004049	EEF1B2;RPL32;RPS19;RPS6;RPS20;RPL10A;SEPP1;RPL39
Rabeprazole-1024_mg/kg_in_Water-Rat-Liver-5d-dn	8/349	0,138276657	0,308342852	0	0	1,575438907	3,117004049	EEF1B2;C1QA;RPS17;RPL32;RPS19;CLP1;RPS8;RPL39
2378-Tetrachlorodibenzo-P-Dioxin-0.02_mg/kg_in_CMC-Rat-Liver-3d-dn	7/294	0,138546336	0,308856763	0	0	1,636393389	3,234414095	RPS19;CLP1;RPS8;RPS6;RPS20;RPL39;TPT1
4-Chloro-2-Nitroaniline-1200_mg/kg_in_CMC-Rat-Kidney-1d-dn	7/294	0,138546336	0,308769367	0	0	1,636393389	3,234414095	RPL32;RPS19;FEM1B;RPS6;RPSA;RPL10A;RPL39
Artemether-74_mg/kg_in_Corn_Oil-Rat-Liver-3d-up	7/294	0,138546336	0,308682021	0	0	1,636393389	3,234414095	EEF1B2;RPS19;RPSA;RPS3A;SEPP1;RPL39;TPT1
Aspirin-500_mg/kg_in_Corn_Oil-Rat-Liver-1d-up	7/294	0,138546336	0,308594724	0	0	1,636393389	3,234414095	C1QA;RPS17;RPS6;RPL35A;SEPP1;RPL39;TPT1
Benzethonium_Chloride-138_mg/kg_in_Water-Rat-Heart-3d-dn	7/294	0,138546336	0,308507476	0	0	1,636393389	3,234414095	EEF1B2;RPS17;ARL6IP5;RPS20;RPS3A;SEPP1;TPT1
Bithionol-59_mg/kg_in_Corn_Oil-Rat-Bone_marrow-1d-up	7/294	0,138546336	0,308420278	0	0	1,636393389	3,234414095	C1QA;RPS17;RPS5;RPS3A;SEPP1;RPL39;TPT1
Carvedilol-2000_mg/kg_in_Corn_Oil-Rat-Liver-3d-dn	7/294	0,138546336	0,308333129	0	0	1,636393389	3,234414095	EEF1B2;RPS17;RPL32;CLP1;RPS8;RPL35A;SEPP1
Cisplatin-2_mg/kg_in_Saline-Rat-Liver-5d-dn	7/294	0,138546336	0,308246029	0	0	1,636393389	3,234414095	EEF1B2;RPS17;RPL32;CLP1;RPS3A;RPL10A;SEPP1
Hydroxyurea-400_mg/kg_in_Saline-Rat-Liver-5d-dn	7/294	0,138546336	0,308158978	0	0	1,636393389	3,234414095	EEF1B2;C1QA;RPS17;RPL32;RPS20;RPS3A;RPL10A
Lovastatin-450_mg/kg_in_Corn_Oil-Rat-Kidney-1d-up	7/294	0,138546336	0,308071977	0	0	1,636393389	3,234414095	RPS17;RPS19;RPS6;RPL35A;RPS20;RPS3A;RPL39
NN-Dimethylformamide-1400_mg/kg_in_Saline-Rat-Liver-1d-dn	7/294	0,138546336	0,307985025	0	0	1,636393389	3,234414095	RPL32;RPS19;RPS8;RPS6;RPS20;RPS3A;SEPP1
Niacin-2625_mg/kg_in_Water-Rat-Liver-1d-up	7/294	0,138546336	0,307898121	0	0	1,636393389	3,234414095	RPL32;RPS19;RPS8;RPS6;RPS20;RPS3A;TPT1
Progesterone-164_mg/kg_in_Corn_Oil-Rat-Liver-3d-dn	7/294	0,138546336	0,307811267	0	0	1,636393389	3,234414095	EEF1B2;C1QA;RPL32;RPS6;RPL35A;RPL39;TPT1
Tetracycline-1500_mg/kg_in_Water-Rat-Liver-1d-dn	7/294	0,138546336	0,307724462	0	0	1,636393389	3,234414095	RPS19;RPS8;RPS20;RPS3A;SEPP1;RPL39;TPT1
Tinidazole-1200_mg/kg_in_CMC-Rat-Liver-3d-dn	7/294	0,138546336	0,307637706	0	0	1,636393389	3,234414095	EEF1B2;C1QA;RPL32;RPS8;RPL35A;SEPP1;TPT1
Simvastatin-1200_mg/kg_in_Corn_Oil-Rat-Liver-1d-dn	8/350	0,139848702	0,310442046	0	0	1,570937653	3,090339343	C1QA;CLP1;RPS6;RPS3A;RPL10A;SEPP1;RPL39;TPT1
Allylamine-75_mg/kg_in_Water-Rat-Heart-5d-up	7/295	0,14027161	0,311293098	0	0	1,630846293	3,203266964	EEF1B2;RPS17;RPL32;ARL6IP5;RPS3A;RPL39;TPT1
Amikacin-20_mg/kg_in_Saline-Rat-Kidney-5d-dn	7/295	0,14027161	0,31120541	0	0	1,630846293	3,203266964	EEF1B2;RPS17;RPL32;CLP1;RPS8;RPS3A;TPT1
Artemether-74_mg/kg_in_Corn_Oil-Rat-Liver-1d-dn	7/295	0,14027161	0,311117771	0	0	1,630846293	3,203266964	EEF1B2;CLP1;RPS8;FEM1B;RPS6;RPS20;RPL10A
Balsalazide-1100_mg/kg_in_Water-Rat-Liver-5d-dn	7/295	0,14027161	0,311030181	0	0	1,630846293	3,203266964	EEF1B2;C1QA;RPL32;CLP1;RPS8;RPS3A;SEPP1
Bisphenol_A-150_mg/kg_in_Corn_Oil-Rat-Liver-3d-up	7/295	0,14027161	0,310942641	0	0	1,630846293	3,203266964	C1QA;RPS17;CXADR;RPL35A;RPSA;SEPP1;RPL39
Catechol-40_mg/kg_in_Saline-Rat-Kidney-1d-dn	7/295	0,14027161	0,31085515	0	0	1,630846293	3,203266964	EEF1B2;ARL2BP;RPS8;R3HCC1;ARL6IP5;RPSA;RPL10A
Celecoxib-100_uM_in_DMSO-Rat-Primary_rat_hepatocytes-0.67d-dn	7/295	0,14027161	0,310767709	0	0	1,630846293	3,203266964	RPS17;CXCL11;RPL32;RPS19;RPS8;SEPP1;TPT1
Cobalt(II)_Acetylacetonate-100_mg/kg_in_Corn_Oil-Rat-Heart-3d-dn	7/295	0,14027161	0,310680316	0	0	1,630846293	3,203266964	RPS17;RPL32;RPS19;ARL2BP;RPS8;RPS6;RPSA
Diethylstilbestrol-280_mg/kg_in_Corn_Oil-Rat-Spleen-1d-up	7/295	0,14027161	0,310592973	0	0	1,630846293	3,203266964	EEF1B2;C1QA;RPS8;ARL6IP5;RPSA;RPS3A;SEPP1
Doxorubicin-3_mg/kg_in_Saline-Rat-Spleen-1d-up	7/295	0,14027161	0,310505678	0	0	1,630846293	3,203266964	RPS19;RPS6;RPSA;RPL10A;SEPP1;RPL39;TPT1
Droperidol-94_mg/kg_in_CMC-Rat-Heart-3d-dn	7/295	0,14027161	0,310418433	0	0	1,630846293	3,203266964	EEF1B2;RPS8;COL5A3;ARL6IP5;RPS20;RPSA;SEPP1
Levosulpiride-1200_mg/kg_in_CMC-Rat-Heart-3d-up	7/295	0,14027161	0,310331237	0	0	1,630846293	3,203266964	RPS17;RPL32;RPS8;RPS5;FUNDC1;RPS20;TPT1
Stavudine-1400_mg/kg_in_Water-Rat-Heart-5d-dn	7/295	0,14027161	0,31024409	0	0	1,630846293	3,203266964	RPS17;RPL32;RPS19;RPS8;RPL35A;RPS20;RPS3A
Trichloroacetic_Acid-474_mg/kg_in_Corn_Oil-Rat-Liver-1d-dn	7/295	0,14027161	0,310156992	0	0	1,630846293	3,203266964	RPS19;CLP1;RPS8;RPS6;RPL10A;SEPP1;TPT1
Venlafaxine-320_mg/kg_in_Water-Rat-Heart-5d-up	7/295	0,14027161	0,310069942	0	0	1,630846293	3,203266964	EEF1B2;RPS8;RPS6;ARL6IP5;RAPGEF5;RPL39;TPT1
Vinblastine-0.08_mg/kg_in_Saline-Rat-Bone_marrow-0.25d-dn	7/295	0,14027161	0,309982942	0	0	1,630846293	3,203266964	EEF1B2;NKG7;RPS3A;RPL10A;SEPP1;RPL39;TPT1
Raloxifene-650_mg/kg_in_Corn_Oil-Rat-Liver-1d-up	6/241	0,14058115	0,310579842	0	0	1,711083544	3,357095225	C1QA;RPS19;RPS6;RPSA;RPS3A;TPT1
Acyclovir-330_mg/kg_in_Saline-Rat-Bone_marrow-3d-up	8/351	0,141429727	0,312366947	0	0	1,566462048	3,063925062	RPS17;RPL32;ARL2BP;RPS8;RPS5;RPL35A;RPSA;TPT1
Clofibrate-500_uM_in_DMSO-Rat-Primary_rat_hepatocytes-1d-dn	8/351	0,141429727	0,312279376	0	0	1,566462048	3,063925062	RPS19;ARL2BP;RPS8;RPS5;RPS20;RPSA;RPL39;TPT1
Tranilast-1100_mg/kg_in_CMC-Rat-Heart-1d-up	8/351	0,141429727	0,312191854	0	0	1,566462048	3,063925062	EEF1B2;RPL32;ARL2BP;RPS8;FUNDC1;RAPGEF5;RPL10A;TPT1
1-Naphthyl_Isothiocyanate-166.67_uM_in_DMSO-Rat-Primary_rat_hepatocytes-0.67d-up	7/296	0,142007322	0,313379004	0	0	1,625336677	3,172456725	EEF1B2;RPS6;RPS3A;RPL10A;RPL39;GOLGA7;PDRG1
3-Chloroaniline-200_mg/kg_in_CMC-Rat-Kidney-5d-up	7/296	0,142007322	0,313291223	0	0	1,625336677	3,172456725	RPS17;RPSA;RPS3A;RPL10A;SEPP1;GOLGA7;TPT1
Acyclovir-330_mg/kg_in_Saline-Rat-Spleen-0.25d-dn	7/296	0,142007322	0,313203491	0	0	1,625336677	3,172456725	CXCL11;RPS8;FUNDC1;RPS20;RPSA;RPL10A;TPT1
Diazepam-710_mg/kg_in_CMC-Rat-Heart-5d-up	7/296	0,142007322	0,313115808	0	0	1,625336677	3,172456725	EEF1B2;ARL2BP;CLP1;RPS8;RPS20;RPS3A;TPT1
Dicumarol-5_mg/kg_in_Corn_Oil-Rat-Liver-1d-up	7/296	0,142007322	0,313028174	0	0	1,625336677	3,172456725	RPS17;CLP1;FEM1B;RPS20;RPS3A;RPL39;TPT1
Ketoconazole-25_mg/kg_in_Corn_Oil-Rat-Liver-5d-up	7/296	0,142007322	0,31294059	0	0	1,625336677	3,172456725	EEF1B2;RPS17;RPL32;RPS20;RPSA;RPL39;TPT1
Letrozole-250_mg/kg_in_Corn_Oil-Rat-Liver-0.25d-up	7/296	0,142007322	0,312853054	0	0	1,625336677	3,172456725	C1QA;RPS19;RPS6;RPSA;RPL10A;SEPP1;RPL39
Methylparaben-1500_mg/kg_in_CMC-Rat-Liver-5d-dn	7/296	0,142007322	0,312765567	0	0	1,625336677	3,172456725	EEF1B2;C1QA;RPS19;RPS20;RPL10A;SEPP1;TPT1
Metronidazole-1500_mg/kg_in_Water-Rat-Liver-1d-dn	7/296	0,142007322	0,312678129	0	0	1,625336677	3,172456725	RPS19;FUNDC1;RPS6;ARL6IP5;RPS20;RPL10A;SEPP1
Mifepristone-3_mg/kg_in_Corn_Oil-Rat-Liver-0.25d-dn	7/296	0,142007322	0,31259074	0	0	1,625336677	3,172456725	RPS17;RPL32;RPS8;RPL35A;RPS20;RPL39;TPT1
Oxymetholone-1170_mg/kg_in_CMC-Rat-Liver-5d-up	7/296	0,142007322	0,3125034	0	0	1,625336677	3,172456725	C1QA;RPS17;RPS6;ARL6IP5;RPSA;RPL10A;RPL39
Perhexiline-320_mg/kg_in_CMC-Rat-Liver-3d-dn	7/296	0,142007322	0,312416108	0	0	1,625336677	3,172456725	C1QA;RPS17;RPS19;RPS6;RPS20;RPSA;TPT1
Sulfadiazine-1170_mg/kg_in_Water-Rat-Liver-3d-dn	7/296	0,142007322	0,312328865	0	0	1,625336677	3,172456725	EEF1B2;RPL32;RPS8;RPL35A;RPS3A;RPL39;TPT1
Sulfadoxine-12_mg/kg_in_Corn_Oil-Rat-Liver-1d-up	7/296	0,142007322	0,312241671	0	0	1,625336677	3,172456725	C1QA;RPS19;RPS6;RPS20;RPL39;GOLGA7;TPT1
Thimerosal-10_mg/kg_in_Water-Rat-Brain-5d-dn	7/296	0,142007322	0,312154526	0	0	1,625336677	3,172456725	RPS17;RPL32;RPS8;FEM1B;RPL35A;RPS3A;TOMM20
Tranilast-300_mg/kg_in_CMC-Rat-Spleen-3d-up	7/296	0,142007322	0,312067429	0	0	1,625336677	3,172456725	RPS19;RPS8;RPS5;RPSA;RAPGEF5;RPL10A;SEPP1
Beta-Estradiol-250_uM_in_DMSO-Rat-Primary_rat_hepatocytes-0.67d-dn	5/189	0,142293588	0,312609289	0	0	1,818214877	3,545269608	RPS17;CXADR;RPSA;RPS3A;SLC16A3
Nisoldipine-1125_mg/kg_in_Water-Rat-Liver-0.25d-dn	6/242	0,14251972	0,313018771	0	0	1,70401295	3,31988567	EEF1B2;RPS17;RPL32;RPL35A;RPL39;SEPP1
Lithocholic_Acid-74_uM_in_DMSO-Rat-Primary_rat_hepatocytes-1d-dn	8/352	0,14301969	0,314029295	0	0	1,562011871	3,037758458	RPS19;RPS6;RPSA;RPS3A;RPL10A;RPL39;SLC16A3;TPT1
Pioglitazone-3_mg/kg_in_Corn_Oil-Rat-Liver-1d-up	8/352	0,14301969	0,313941772	0	0	1,562011871	3,037758458	EEF1B2;RPS17;RPL32;RPS19;RPS20;RPL10A;SEPP1;TPT1
Acemetacin-2_mg/kg_in_Corn_Oil-Rat-Liver-3d-dn	7/297	0,14375341	0,315464434	0	0	1,619864163	3,141979049	RPL32;RPS8;FEM1B;RPS6;RPS20;RPL10A;TPT1
Aconitine-0.9_mg/kg_in_CMC-Rat-Heart-3d-dn	7/297	0,14375341	0,315376561	0	0	1,619864163	3,141979049	RPS19;ARL2BP;RPS8;RPS20;RPSA;RPL10A;RPL39
Altretamine-13_mg/kg_in_Corn_Oil-Rat-Liver-3d-dn	7/297	0,14375341	0,315288737	0	0	1,619864163	3,141979049	C1QA;RPS17;RPS19;CLP1;RPS6;RPS3A;RPL39
Amlodipine-0.2_mg/kg_in_Corn_Oil-Rat-Liver-1d-dn	7/297	0,14375341	0,315200962	0	0	1,619864163	3,141979049	C1QA;CLP1;RPS6;RPS20;RPSA;RPL10A;RPL39
Doxorubicin-4_mg/kg_in_Saline-Rat-Heart-5d-up	7/297	0,14375341	0,315113235	0	0	1,619864163	3,141979049	RPS19;COL5A3;ARL6IP5;RPS20;RPSA;RPL10A;SEPP1
Ethylene_Glycol-3525_mg/kg_in_Water-Rat-Kidney-5d-dn	7/297	0,14375341	0,315025558	0	0	1,619864163	3,141979049	RPS19;ARL2BP;CLP1;RPS6;RPS3A;RPL10A;RPL39
Etidronate-1005_mg/kg_in_Water-Rat-Heart-3d-dn	7/297	0,14375341	0,314937929	0	0	1,619864163	3,141979049	C1QA;ARL6IP5;RPS20;RPS3A;SEPP1;RPL39;TPT1
Lacidipine-1_mg/kg_in_Corn_Oil-Rat-Heart-1d-dn	7/297	0,14375341	0,314850349	0	0	1,619864163	3,141979049	EEF1B2;RPS8;FUNDC1;RPS6;ARL6IP5;RPL13A;TOMM20
Levosulpiride-1200_mg/kg_in_CMC-Rat-Heart-5d-up	7/297	0,14375341	0,314762817	0	0	1,619864163	3,141979049	RPS17;CXCL11;RPL32;RPS8;RPS3A;SEPP1;TPT1

Methocarbamol-1125_mg/kg_in_CMC-Rat-Liver-1d-dn	7/297	0,14375341	0,314675335	0	0	1,619864163	3,141979049	EEF1B2;RPL32;RPS19;CLP1;RPS20;RPS3A;SEPP1
Metoclopramide-185_mg/kg_in_CMC-Rat-Heart-5d-dn	7/297	0,14375341	0,314587901	0	0	1,619864163	3,141979049	RPS19;ARL2BP;COL5A3;FUNDC1;RPS20;RPS3A;GOLGA7
Mitotane-900_mg/kg_in_Water-Rat-Bone_marrow-1d-dn	7/297	0,14375341	0,314500515	0	0	1,619864163	3,141979049	C1QA;ARL6IP5;RPS20;RPS3A;RPL39;TPT1;DUSP14
Phenacemide-400_mg/kg_in_CMC-Rat-Heart-5d-dn	7/297	0,14375341	0,314413178	0	0	1,619864163	3,141979049	ARL2BP;COL5A3;RPS6;ARL6IP5;RPSA;SEPP1;PDRG1
Piracetam-2500_mg/kg_in_CMC-Rat-Brain-1d-dn	7/297	0,14375341	0,31432589	0	0	1,619864163	3,141979049	EEF1B2;RPS19;RPS6;SEPP1;RPL39;GOLGA7;TPT1
Sodium_Arsenite-10_mg/kg_in_Saline-Rat-Bone_marrow-3d-up	7/297	0,14375341	0,314238649	0	0	1,619864163	3,141979049	RPS19;RPS5;RPS6;RPL13A;RPL35A;RPS20;RPL39
Thioguanine-24_mg/kg_in_Corn_Oil-Rat-Kidney-1d-up	7/297	0,14375341	0,314151458	0	0	1,619864163	3,141979049	RPS17;RPS8;FUNDC1;RPS20;SEPP1;TOMM20;TPT1
Trichloroethylene-1230_mg/kg_in_CMC-Rat-Kidney-1d-up	7/297	0,14375341	0,314064315	0	0	1,619864163	3,141979049	RPS17;CLP1;RPS8;ARL6IP5;RPSA;RPL10A;TOMM20
Atropine-94_mg/kg_in_Water-Rat-Heart-5d-dn	8/353	0,144618547	0,315866799	0	0	1,557586908	3,011836822	RPL32;RPS19;RPS8;RPS6;RPSA;RPS3A;SEPP1;TPT1
Altretamine-13_mg/kg_in_Corn_Oil-Rat-Bone_marrow-1d-dn	7/298	0,145509812	0,317725334	0	0	1,614428377	3,111829679	RPL32;RPS19;RPS8;RPS5;RPS6;ARL6IP5;RPSA
Ampicillin-2000_mg/kg_in_CMC-Rat-Kidney-1d-up	7/298	0,145509812	0,317637273	0	0	1,614428377	3,111829679	EEF1B2;RPS19;RPS8;RPS6;RPSA;RPL10A;RPL39
Aspirin-35_mg/kg_in_Corn_Oil-Rat-Liver-1d-dn	7/298	0,145509812	0,31754926	0	0	1,614428377	3,111829679	EEF1B2;RPS17;RPL32;RPS6;RPS3A;RPL39;TPT1
Bromobenzene-439_mg/kg_in_CMC-Rat-Kidney-3d-up	7/298	0,145509812	0,317461296	0	0	1,614428377	3,111829679	RPS17;RPL32;RPS6;RPS3A;RAPGEF5;RPL10A;SEPP1
Busulfan-3_mg/kg_in_Corn_Oil-Rat-Bone_marrow-0.25d-dn	7/298	0,145509812	0,317373381	0	0	1,614428377	3,111829679	RPS19;RPS6;ARL6IP5;RPSA;RPL10A;SEPP1;TPT1
Cloxacillin-1200_mg/kg_in_Water-Rat-Kidney-5d-dn	7/298	0,145509812	0,317285515	0	0	1,614428377	3,111829679	ARL2BP;CLP1;RPS6;RPL10A;SEPP1;RPL39;TPT1
Ebastine-2000_mg/kg_in_Water-Rat-Heart-3d-dn	7/298	0,145509812	0,317197697	0	0	1,614428377	3,111829679	EEF1B2;RPS19;COL5A3;RPS20;RPS3A;RPL10A;RPL39
Ebastine-2_mg/kg_in_Water-Rat-Heart-5d-dn	7/298	0,145509812	0,317109928	0	0	1,614428377	3,111829679	EEF1B2;RPS17;ARL2BP;RPS8;RPS20;RPS3A;TPT1
Ethisterone-1500_mg/kg_in_CMC-Rat-Liver-3d-dn	7/298	0,145509812	0,317022207	0	0	1,614428377	3,111829679	RPS19;CLP1;RPS8;SEPP1;RPL39;GOLGA7;TPT1
Ibuprofen-263_mg/kg_in_Corn_Oil-Rat-Kidney-1d-dn	7/298	0,145509812	0,316934535	0	0	1,614428377	3,111829679	EEF1B2;RPS17;RPL32;RPL35A;RPS3A;SEPP1;TOMM20
Ibuprofen-263_mg/kg_in_Corn_Oil-Rat-Liver-1d-up	7/298	0,145509812	0,316846912	0	0	1,614428377	3,111829679	EEF1B2;RPS19;RPS20;RPSA;SEPP1;RPL39;TPT1
Isotretinoin-125_mg/kg_in_Corn_Oil-Rat-Liver-0.25d-up	7/298	0,145509812	0,316759337	0	0	1,614428377	3,111829679	RPS17;RPL32;RPS19;RPS8;RPS6;RPS20;RPSA
Isotretinoin-125_mg/kg_in_Corn_Oil-Rat-Liver-1d-up	7/298	0,145509812	0,31667181	0	0	1,614428377	3,111829679	RPS19;RPS6;RPL35A;RPS20;RPSA;RPS3A;TPT1
Norepinephrine-1.75_mg/kg_in_Saline-Rat-Heart-5d-dn	7/298	0,145509812	0,316584331	0	0	1,614428377	3,111829679	EEF1B2;RPS17;RPL32;RPS6;RPS3A;SEPP1;TPT1
Fenofibrate-43_mg/kg_in_Corn_Oil-Rat-Kidney-3d-dn	8/354	0,146226255	0,318055229	0	0	1,553186945	2,986157479	EEF1B2;RPS17;RPL32;RPS19;RPS3A;RPL10A;SEPP1;RPL39
Meloxicam-0.6_mg/kg_in_Corn_Oil-Rat-Kidney-0.25d-up	8/354	0,146226255	0,317967417	0	0	1,553186945	2,986157479	RPS19;FUNDC1;RPS6;RPS20;SEPP1;RPL39;GOLGA7;TPT1
Beta-Estradiol-250_uM_in_DMSO-Rat-Primary_rat_hepatocytes-0.67d-up	9/411	0,147010105	0,319583657	0	0	1,505004139	2,885475131	EEF1B2;RPS19;RPS8;RPS6;RPS20;SEPP1;RPL39;TPT1;PDRG1
Amikacin-20_mg/kg_in_Saline-Rat-Bone_marrow-0.25d-up	7/299	0,147276467	0,320074352	0	0	1,609028951	3,082004421	C1QA;RPS17;RPS19;NKG7;RPSA;SLC16A3;TPT1
Amprenavir-600_mg/kg_in_CMC-Rat-Kidney-5d-dn	7/299	0,147276467	0,319986056	0	0	1,609028951	3,082004421	RPS19;CLP1;RPS6;RPL10A;SEPP1;GOLGA7;TPT1
Atorvastatin-250_uM_in_DMSO-Rat-Primary_rat_hepatocytes-0.67d-up	7/299	0,147276467	0,319897808	0	0	1,609028951	3,082004421	RPS17;RPL32;RPL35A;RPS3A;RPL39;TPT1;PDRG1
Carboplatin-6_mg/kg_in_Saline-Rat-Spleen-0.25d-up	7/299	0,147276467	0,319809609	0	0	1,609028951	3,082004421	RPS17;RPS5;RPSA;RPL10A;SEPP1;RPL39;TPT1
Carmustine-16_mg/kg_in_Corn_Oil-Rat-Liver-3d-up	7/299	0,147276467	0,319721458	0	0	1,609028951	3,082004421	CXADR;ARL2BP;RPS6;RPS20;RPS3A;RPL10A;GOLGA7
Cefotaxime-1000_mg/kg_in_Saline-Rat-Kidney-3d-up	7/299	0,147276467	0,319633357	0	0	1,609028951	3,082004421	RPS17;ARL2BP;RPS5;ARL6IP5;RPSA;SEPP1;TPT1
Etoposide-100_mg/kg_in_Corn_Oil-Rat-Liver-3d-dn	7/299	0,147276467	0,319545303	0	0	1,609028951	3,082004421	C1QA;RPS17;CLP1;RPS3A;RPL10A;SEPP1;GOLGA7
Idebenone-2500_mg/kg_in_CMC-Rat-Liver-1d-up	7/299	0,147276467	0,319457299	0	0	1,609028951	3,082004421	CXCL11;RPS8;RPS20;RPSA;RPL10A;RPL39;TPT1
Indomethacin-9.6_mg/kg_in_Water-Rat-Heart-3d-up	7/299	0,147276467	0,319369342	0	0	1,609028951	3,082004421	RPS17;RPS19;ARL6IP5;RPS20;SEPP1;GOLGA7;TPT1
Ipriflavone-1500_mg/kg_in_CMC-Rat-Liver-1d-dn	7/299	0,147276467	0,319281434	0	0	1,609028951	3,082004421	EEF1B2;RPS19;CLP1;RPS8;RPS6;RPL10A;SEPP1
Methyl_Salicylate-444_mg/kg_in_Corn_Oil-Rat-Liver-0.25d-up	7/299	0,147276467	0,319193575	0	0	1,609028951	3,082004421	RPL32;RPS19;ARL2BP;RPS8;RPS6;RPSA;SEPP1
Metoprolol-1200_mg/kg_in_Water-Rat-Heart-5d-dn	7/299	0,147276467	0,319105764	0	0	1,609028951	3,082004421	RPS19;ARL2BP;RPS8;RPS20;RPSA;SEPP1;TPT1
Mitomycin_C-1.7_mg/kg_in_Saline-Rat-Spleen-5d-dn	7/299	0,147276467	0,319018001	0	0	1,609028951	3,082004421	RPS17;RPS8;RPL35A;RPS20;RPSA;RPL10A;TPT1
Mosapride-2000_mg/kg_in_CMC-Rat-Intestine-5d-up	7/299	0,147276467	0,318930286	0	0	1,609028951	3,082004421	RPL32;RPS8;FEM1B;RPS20;RPL10A;GOLGA7;TPT1
NN-Dimethylformamide-140_mg/kg_in_Saline-Rat-Liver-3d-up	7/299	0,147276467	0,31884262	0	0	1,609028951	3,082004421	EEF1B2;RPS17;RPS19;RPS6;RPS3A;RPL10A;SEPP1
Nystatin-134_mg/kg_in_Corn_Oil-Rat-Kidney-3d-dn	7/299	0,147276467	0,318755002	0	0	1,609028951	3,082004421	EEF1B2;RPS17;RPL32;CLP1;RPS8;RPL35A;RPS3A
Pioglitazone-3_mg/kg_in_Corn_Oil-Rat-Liver-0.25d-up	7/299	0,147276467	0,318667432	0	0	1,609028951	3,082004421	RPL32;RPS19;RPS6;RPS3A;RPL10A;SEPP1;TPT1
Pramoxine-526_mg/kg_in_Corn_Oil-Rat-Liver-5d-dn	7/299	0,147276467	0,31857991	0	0	1,609028951	3,082004421	RPL32;RPS19;RPS8;RPS6;RPS3A;RPL39;TPT1
Prednisolone-184_mg/kg_in_Water-Rat-Liver-1d-up	7/299	0,147276467	0,318492436	0	0	1,609028951	3,082004421	RPS17;CXCL11;RPL32;RPS20;RPSA;RPL39;TPT1
Rofecoxib-1550_mg/kg_in_Corn_Oil-Rat-Liver-1d-up	7/299	0,147276467	0,31840501	0	0	1,609028951	3,082004421	EEF1B2;RPS17;RPL32;RPS19;RPS20;RPL10A;SEPP1
Stavudine-1120_uM_in_DMSO-Rat-Primary_rat_hepatocytes-0.67d-dn	7/299	0,147276467	0,318317632	0	0	1,609028951	3,082004421	RPS17;CXCL11;RPS8;RPS20;RPS3A;RPL10A;SEPP1
Thioguanine-24_mg/kg_in_Corn_Oil-Rat-Kidney-3d-dn	7/299	0,147276467	0,318230302	0	0	1,609028951	3,082004421	EEF1B2;RPS19;ARL2BP;CLP1;RPS6;RPS20;RPL39
Troxipide-1200_mg/kg_in_CMC-Rat-Heart-3d-dn	7/299	0,147276467	0,31814302	0	0	1,609028951	3,082004421	RPS19;RPS8;FEM1B;COL5A3;RPS6;RPS20;RPL39
Valproic_Acid-850_mg/kg_in_Water-Rat-Kidney-1d-dn	7/299	0,147276467	0,318055786	0	0	1,609028951	3,082004421	RPS17;ARL2BP;RPS8;FUNDC1;ARL6IP5;RPS20;RPSA
Haloperidol-77_uM_in_DMSO-Rat-Primary_rat_hepatocytes-0.67d-up	8/355	0,147842769	0,319191241	0	0	1,548811771	2,960717788	EEF1B2;RPS19;RPS6;RPL10A;SEPP1;RPL39;GOLGA7;TPT1
Tiapride-1200_mg/kg_in_Water-Rat-Heart-3d-up	8/355	0,147842769	0,319103767	0	0	1,548811771	2,960717788	C1QA;RPS17;RPL32;RPS6;ARL6IP5;RPS3A;RPL10A;TPT1
Diclofenac-3.5_mg/kg_in_Corn_Oil-Rat-Kidney-0.25d-dn	6/245	0,148409126	0,320238431	0	0	1,683147486	3,211079245	RPS17;RPL32;RPS19;ARL2BP;ARL6IP5;RPS3A
Azlocillin-800_mg/kg_in_Saline-Rat-Kidney-0.25d-up	7/300	0,14905331	0,321540364	0	0	1,603665521	3,052499147	EEF1B2;RPS17;RPS8;RPS5;RPSA;SEPP1;TPT1
Bupropion-895_mg/kg_in_Water-Rat-Liver-3d-up	7/300	0,14905331	0,321452319	0	0	1,603665521	3,052499147	RPS19;RPS8;RPS6;RPSA;RPS3A;RPL10A;SEPP1
Celecoxib-35_mg/kg_in_Corn_Oil-Rat-Heart-5d-up	7/300	0,14905331	0,321364322	0	0	1,603665521	3,052499147	RPS17;RPS8;ARL6IP5;RPS20;RPL39;TPT1;PDRG1
Clomiphene-250_mg/kg_in_CMC-Rat-Liver-5d-up	7/300	0,14905331	0,321276373	0	0	1,603665521	3,052499147	C1QA;RPS17;RPL32;RPS6;RPSA;RPS3A;RPL39
Cyproterone_Acetate-2500_mg/kg_in_CMC-Rat-Liver-1d-dn	7/300	0,14905331	0,321188473	0	0	1,603665521	3,052499147	C1QA;RPS17;RPL32;CLP1;RPS8;RPS20;SEPP1
Hydroxyurea-400_mg/kg_in_Saline-Rat-Spleen-1d-up	7/300	0,14905331	0,32110062	0	0	1,603665521	3,052499147	C1QA;RPS19;RPS6;RPSA;RPL10A;SEPP1;RPL39
Lomustine-4.2_mg/kg_in_Corn_Oil-Rat-Bone_marrow-1d-dn	7/300	0,14905331	0,321012816	0	0	1,603665521	3,052499147	RPL32;RPS19;RPS8;FEM1B;RPS6;ARL6IP5;RPSA
Methotrexate-27_mg/kg_in_Corn_Oil-Rat-Bone_marrow-1d-up	7/300	0,14905331	0,320925059	0	0	1,603665521	3,052499147	C1QA;RPS19;ARL2BP;CLP1;NKG7;SEPP1;TPT1
Methylparaben-1500_mg/kg_in_CMC-Rat-Liver-1d-dn	7/300	0,14905331	0,320837351	0	0	1,603665521	3,052499147	EEF1B2;C1QA;RPL32;CLP1;RPL35A;RPS20;SEPP1
Mifepristone-3_mg/kg_in_Corn_Oil-Rat-Liver-5d-dn	7/300	0,14905331	0,32074969	0	0	1,603665521	3,052499147	C1QA;RPL32;RPS19;RPS6;ARL6IP5;RPS3A;SEPP1
Mifepristone-3_mg/kg_in_Corn_Oil-Rat-Liver-5d-up	7/300	0,14905331	0,320662078	0	0	1,603665521	3,052499147	EEF1B2;RPS8;RPS20;RPSA;RPL39;GOLGA7;TPT1
Mitotane-5000_mg/kg_in_Water-Rat-Bone_marrow-1d-up	7/300	0,14905331	0,320574513	0	0	1,603665521	3,052499147	RPS19;FEM1B;RPS6;NKG7;RPL10A;SEPP1;TPT1
Nitrendipine-10_mg/kg_in_Corn_Oil-Rat-Heart-1d-dn	7/300	0,14905331	0,320486996	0	0	1,603665521	3,052499147	RPS19;ARL2BP;CLP1;RPS8;RPS6;ARL6IP5;RPS20
Nitrendipine-10_mg/kg_in_Corn_Oil-Rat-Heart-1d-up	7/300	0,14905331	0,320399527	0	0	1,603665521	3,052499147	RPS17;RPL32;COL5A3;RPS3A;SEPP1;RPL39;TPT1
Penicillamine-100_mg/kg_in_Water-Rat-Liver-1d-dn	7/300	0,14905331	0,320312106	0	0	1,603665521	3,052499147	EEF1B2;RPS17;RPL32;RPS6;RPL10A;SEPP1;RPL39
Penicillamine-100_mg/kg_in_Water-Rat-Liver-1d-up	7/300	0,14905331	0,320224732	0	0	1,603665521	3,052499147	CXCL11;RPS19;CLP1;RPS8;RPS20;RPSA;TPT1
Roflumilast-17_mg/kg_in_CMC-Rat-Liver-1d-dn	7/300	0,14905331	0,320137406	0	0	1,603665521	3,052499147	RPS17;RPL32;RPS19;RPS6;RPS20;RPL10A;SEPP1
Secobarbital-70_mg/kg_in_Water-Rat-Liver-5d-dn	7/300	0,14905331	0,320050127	0	0	1,603665521	3,052499147	C1QA;RPS17;RPL32;RPS8;RPS3A;SEPP1;TPT1
Spironolactone-300_mg/kg_in_CMC-Rat-Heart-1d-up	7/300	0,14905331	0,319962896	0	0	1,603665521	3,052499147	RPS19;RPS6;RPS20;RPS3A;RAPGEF5;SEPP1;RPL39
Suxamethonium_Chloride-0.125_mg/kg_in_Saline-Rat-Heart-3d-dn	7/300	0,14905331	0,319875713	0	0	1,603665521	3,052499147	RPS19;RPS8;FEM1B;FUNDC1;RPS6;ARL6IP5;RPSA
Tenidap-75_mg/kg_in_Corn_Oil-Rat-Kidney-3d-up	7/300	0,14905331	0,319788577	0	0	1,603665521	3,052499147	CXCL11;RPS19;RPS6;RPS20;RPSA;RPS3A;RPL39
Tetracaine-24_mg/kg_in_Corn_Oil-Rat-Heart-5d-up	7/300	0,14905331	0,319701489	0	0	1,603665521	3,052499147	RPS17;RPS8;RPS5;ARL6IP5;RPS20;RPS3A;RPL10A
Altretamine-40_mg/kg_in_Corn_Oil-Rat-Spleen-3d-up	6/246	0,150396511	0,32249467	0	0	1,676305423	3,175727205	RPL32;CLP1;RPS8;RPS5;RPS6;RPS3A
Azathioprine-0.5_uM_in_DMSO-Rat-Primary_rat_hepatocytes-1d-up	6/246	0,150396511	0,322406892	0	0	1,676305423	3,175727205	EEF1B2;CXCL11;ARL6IP5;RPL10A;GOLGA7;SEPP1
Amikacin-160_mg/kg_in_Saline-Rat-Kidney-5d-up	7/301	0,150840277	0,323270209	0	0	1,598337729	3,023309793	CXCL11;CLP1;RPS6;RPS3A;RPL10A;RPL39;TPT1
Bisphenol_A-100_mg/kg_in_Corn_Oil-Rat-Bone_marrow-1d-up	7/301	0,150840277	0,323182269	0	0	1,598337729	3,023309793	C1QA;RPS19;RPS6;NKG7;RPS20;SEPP1;RPL39
Bromfenac-5_mg/kg_in_CMC-Rat-Kidney-3d-dn	7/301	0,150840277	0,323094376	0	0	1,598337729	3,023309793	ARL2BP;RPS8;FEM1B;RPS6;RPSA;RPL39;TPT1
Bupropion-895_mg/kg_in_Water-Rat-Liver-5d-up	7/301	0,150840277	0,323006531	0	0	1,598337729	3,023309793	RPS19;RPS8;RPS6;RPS20;RPS3A;RPL10A;TPT1
Carmustine-16_mg/kg_in_Corn_Oil-Rat-Liver-3d-dn	7/301	0,150840277	0,322918733	0	0	1,598337729	3,023309793	EEF1B2;RPS17;RPL32;RPS19;RPS8;RPL35A;TPT1
Carvedilol-2000_mg/kg_in_Corn_Oil-Rat-Heart-5d-up	7/301	0,150840277	0,322830984	0	0	1,598337729	3,023309793	FUNDC1;ARL6IP5;RPS20;RAPGEF5;SEPP1;TPT1;PDRG1
Cyclopropane_Carboxylic_Acid-375_mg/kg_in_Water-Rat-Liver-3d-dn	7/301	0,150840277	0,322743282	0	0	1,598337729	3,023309793	C1QA;RPS17;RPL32;RPS19;RPSA;SEPP1;RPL39
Ethambutol-998_mg/kg_in_CMC-Rat-Kidney-3d-dn	7/301	0,150840277	0,322655627	0	0	1,598337729	3,023309793	RPL32;RPS19;RPS6;RPS20;RPL10A;SEPP1;TPT1
Finasteride-800_mg/kg_in_Corn_Oil-Rat-Kidney-5d-up	7/301	0,150840277	0,322568021	0	0	1,598337729	3,023309793	ARL2BP;CLP1;FUNDC1;RPS6;RPS20;RPSA;SEPP1
Lomefloxacin-2000_mg/kg_in_CMC-Rat-Liver-3d-dn	7/301	0,150840277	0,322480461	0	0	1,598337729	3,023309793	RPS17;RPL32;RPS8;ARL6IP5;RPS3A;SEPP1;TPT1
Meloxicam-33_mg/kg_in_Corn_Oil-Rat-Liver-1d-up	7/301	0,150840277	0,32239295	0	0	1,598337729	3,023309793	EEF1B2;C1QA;RPS17;RPL32;RPS19;RPS20;RPS3A
Methyl_Salicylate-444_mg/kg_in_Corn_Oil-Rat-Liver-0.25d-dn	7/301	0,150840277	0,322305485	0	0	1,598337729	3,023309793	EEF1B2;RPS17;RPL35A;RPS20;RPS3A;RPL39;TPT1
Nimetazepam-122_mg/kg_in_CMC-Rat-Liver-1d-dn	7/301	0,150840277	0,322218069	0	0	1,598337729	3,023309793	EEF1B2;RPS17;ARL6IP5;RPL35A;RPS20;SEPP1;RPL39

Safrole-488_mg/kg_in_Corn_Oil-Rat-Liver-1d-dn	7/301	0,150840277	0,322130699	0	0	1,598337729	3,023309793	EEF1B2;C1QA;RPL32;RPS6;RPL10A;SEPP1;RPL39
Stavudine-1120_uM_in_DMSO-Rat-Primary_rat_hepatocytes-0.67d-up	7/301	0,150840277	0,322043377	0	0	1,598337729	3,023309793	EEF1B2;CLP1;RPS6;ARL6IP5;RPL39;TOMM20;PDRG1
Temafloxacin-1000_mg/kg_in_CMC-Rat-Liver-3d-dn	7/301	0,150840277	0,321956103	0	0	1,598337729	3,023309793	RPS17;RPL32;RPS19;CLP1;RPL35A;RPS3A;SEPP1
Valproic_Acid-850_mg/kg_in_Water-Rat-Kidney-1d-up	7/301	0,150840277	0,321868876	0	0	1,598337729	3,023309793	RPS19;RPS6;RPS3A;RPL10A;SEPP1;RPL39;TPT1
Tenidap-41_uM_in_DMSO-Rat-Primary_rat_hepatocytes-1d-up	6/247	0,152395861	0,325100163	0	0	1,669518761	3,140821893	RPS17;RPL32;RPS5;RPS3A;SLC16A3;PDRG1
2-Nitroanisole-740_mg/kg_in_CMC-Rat-Kidney-1d-dn	7/302	0,152637302	0,325527049	0	0	1,59304522	2,994432357	RPL32;RPS19;ARL2BP;FUNDC1;RPS6;RPS20;SEPP1
Alpha-Naphthoflavone-80_mg/kg_in_CMC-Rat-Liver-91d-dn	7/302	0,152637302	0,325438926	0	0	1,59304522	2,994432357	EEF1B2;RPS17;RPS19;RPS6;RPS20;RPS3A;RPL10A
Aspirin-35_mg/kg_in_Corn_Oil-Rat-Liver-1d-up	7/302	0,152637302	0,32535085	0	0	1,59304522	2,994432357	C1QA;RPS19;RPS8;RPS20;RPSA;RPL10A;SEPP1
Candesartan-1300_mg/kg_in_CMC-Rat-Heart-3d-dn	7/302	0,152637302	0,325262823	0	0	1,59304522	2,994432357	RPS19;FUNDC1;RPS6;ARL6IP5;RPSA;RPL10A;SEPP1
Clozapine-69_uM_in_DMSO-Rat-Primary_rat_hepatocytes-0.67d-dn	7/302	0,152637302	0,325174842	0	0	1,59304522	2,994432357	CXCL11;CLP1;RPS8;RPSA;SEPP1;SLC16A3;TPT1
Cyclosporin_A-350_mg/kg_in_Corn_Oil-Rat-Liver-1d-up	7/302	0,152637302	0,32508691	0	0	1,59304522	2,994432357	RPS19;ARL2BP;RPS8;RPS5;RPS20;RPSA;SEPP1
Finasteride-25_mg/kg_in_Corn_Oil-Rat-Liver-3d-dn	7/302	0,152637302	0,324999025	0	0	1,59304522	2,994432357	EEF1B2;RPS17;RPL32;RPS8;RPSA;RPS3A;TPT1
Ibuprofen-54_mg/kg_in_Corn_Oil-Rat-Liver-0.25d-dn	7/302	0,152637302	0,324911187	0	0	1,59304522	2,994432357	RPS17;RPL32;RPS19;RPS8;RPS20;RPS3A;RPL39
Lovastatin-1500_mg/kg_in_Corn_Oil-Rat-Kidney-5d-dn	7/302	0,152637302	0,324823397	0	0	1,59304522	2,994432357	RPS17;ARL2BP;CLP1;RPS8;RPSA;SEPP1;TPT1
Methotrexate-0.3_mg/kg_in_Corn_Oil-Rat-Bone_marrow-3d-up	7/302	0,152637302	0,324735654	0	0	1,59304522	2,994432357	C1QA;CLP1;RPS8;RPS5;RPL35A;RPS20;RPSA
Mitomycin_C-0.5_mg/kg_in_Saline-Rat-Spleen-0.25d-up	7/302	0,152637302	0,324647959	0	0	1,59304522	2,994432357	C1QA;RPS17;RPS5;RPS6;RPL10A;SEPP1;RPL39
Ritonavir-1200_mg/kg_in_CMC-Rat-Kidney-1d-dn	7/302	0,152637302	0,324560311	0	0	1,59304522	2,994432357	RPS17;RPS20;RPSA;RPS3A;SEPP1;GOLGA7;TPT1
Spironolactone-300_mg/kg_in_CMC-Rat-Kidney-3d-dn	7/302	0,152637302	0,324472711	0	0	1,59304522	2,994432357	RPS17;RPL32;RPS19;CLP1;RPS8;RPS3A;TPT1
Sulindac-23_mg/kg_in_Corn_Oil-Rat-Kidney-1d-up	7/302	0,152637302	0,324385157	0	0	1,59304522	2,994432357	RPS19;ARL2BP;CLP1;RPS20;RPSA;RPL39;GOLGA7
Tranilast-1100_mg/kg_in_CMC-Rat-Liver-1d-up	7/302	0,152637302	0,324297651	0	0	1,59304522	2,994432357	RPS8;FEM1B;RPS6;RPSA;RPS3A;RPL10A;TPT1
Lomustine-8.75_mg/kg_in_Corn_Oil-Rat-Liver-0.25d-up	8/358	0,152744689	0,324438287	0	0	1,535832901	2,88581075	EEF1B2;C1QA;RPS19;RPS20;RPS3A;SEPP1;RPL39;TPT1
Raloxifene-650_mg/kg_in_Corn_Oil-Rat-Liver-1d-dn	8/359	0,154395966	0,327857274	0	0	1,531554815	2,861303957	RPS17;RPL32;RPS8;RPL35A;RPS20;RPL10A;SEPP1;RPL39
Ifosfamide-143_mg/kg_in_Saline-Rat-Spleen-3d-dn	6/248	0,154407077	0,32779249	0	0	1,662786831	3,106356512	RPS17;RPL32;RPS8;RPS6;RPS3A;TPT1
Rifapentine-75_mg/kg_in_Corn_Oil-Rat-Kidney-3d-dn	6/248	0,154407077	0,32770416	0	0	1,662786831	3,106356512	RPS17;RPS19;CLP1;RPS6;GOLGA7;SEPP1
4-Nonylphenol-200_mg/kg_in_Corn_Oil-Rat-Kidney-3d-up	7/303	0,15444432	0,327694898	0	0	1,587787645	2,965862897	RPS17;RPS19;ARL2BP;RPS20;RPSA;RPS3A;TPT1
Acemetacin-2_mg/kg_in_Corn_Oil-Rat-Liver-3d-up	7/303	0,15444432	0,327606642	0	0	1,587787645	2,965862897	RPS17;RPS19;RPL35A;RPSA;RPS3A;SEPP1;RPL39
Amitriptyline-20_mg/kg_in_Water-Rat-Heart-3d-dn	7/303	0,15444432	0,327518434	0	0	1,587787645	2,965862897	EEF1B2;RPS17;RPL32;RPS8;RPS20;RPS3A;SEPP1
Busulfan-500_uM_in_DMSO-Rat-Primary_rat_hepatocytes-0.67d-dn	7/303	0,15444432	0,327430273	0	0	1,587787645	2,965862897	C1QA;RPS19;RPS8;RPS5;RPS20;SEPP1;TPT1
Chlorpromazine-43.9_uM_in_DMSO-Rat-Primary_rat_hepatocytes-0.67d-up	7/303	0,15444432	0,327342159	0	0	1,587787645	2,965862897	EEF1B2;RPS8;RPS6;RPS20;RPS3A;RPL10A;RPL39
D-Tubocurarine_Chloride-0.0033_mg/kg_in_Saline-Rat-Heart-5d-up	7/303	0,15444432	0,327254093	0	0	1,587787645	2,965862897	RPS19;RPS6;RPS20;RPSA;RPL10A;SEPP1;RPL39
Danazol-2000_mg/kg_in_CMC-Rat-Liver-5d-up	7/303	0,15444432	0,327166074	0	0	1,587787645	2,965862897	RPS17;RPL32;FEM1B;RPS20;RPSA;RPL10A;RPL39
Etoposide-188_mg/kg_in_Corn_Oil-Rat-Kidney-3d-up	7/303	0,15444432	0,327078102	0	0	1,587787645	2,965862897	CXCL11;RPL32;RPS20;RPS3A;RPL10A;SEPP1;TPT1
Hydrocortisone-56_mg/kg_in_Corn_Oil-Rat-Liver-1d-up	7/303	0,15444432	0,326990178	0	0	1,587787645	2,965862897	RPL32;RPS19;CLP1;RPS6;RPS20;RPS3A;SEPP1
Interleukin_6-0.125_mg/kg_in_RSA/Saline-Rat-Liver-0.25d-up	7/303	0,15444432	0,326902301	0	0	1,587787645	2,965862897	RPS8;RPS6;RPS20;RPS3A;RPL10A;GOLGA7;TPT1
Levofloxacin-739_mg/kg_in_Water-Rat-Heart-3d-dn	7/303	0,15444432	0,326814471	0	0	1,587787645	2,965862897	RPS19;RPS8;FEM1B;RPS20;SEPP1;RPL39;TPT1
Nimetazepam-122_mg/kg_in_CMC-Rat-Liver-3d-up	7/303	0,15444432	0,326726689	0	0	1,587787645	2,965862897	C1QA;RPS19;RPS6;RPSA;RPS3A;RPL10A;RPL39
Prinomastat-500_uM_in_DMSO-Rat-Primary_rat_hepatocytes-0.67d-up	7/303	0,15444432	0,326638953	0	0	1,587787645	2,965862897	EEF1B2;RPS19;RPS6;RPS20;RPSA;RPL39;PDRG1
Progesterone-148_uM_in_DMSO-Rat-Primary_rat_hepatocytes-0.67d-dn	7/303	0,15444432	0,326551265	0	0	1,587787645	2,965862897	CXCL11;FUNDC1;ARL6IP5;RPS20;SEPP1;SLC16A3;TPT1
Sodium_Arsenite-10_mg/kg_in_Saline-Rat-Bone_marrow-3d-dn	7/303	0,15444432	0,326463624	0	0	1,587787645	2,965862897	C1QA;RPS17;RPL32;NKG7;RPS3A;RPL10A;SEPP1
Valdecoxib-404_mg/kg_in_CMC-Rat-Heart-5d-up	7/303	0,15444432	0,32637603	0	0	1,587787645	2,965862897	EEF1B2;RPL32;ARL2BP;RPS8;RPS20;RPSA;RPL39
Vecuronium_Bromide-0.05_mg/kg_in_Saline-Rat-Liver-5d-dn	7/303	0,15444432	0,326288482	0	0	1,587787645	2,965862897	RPS17;RPL32;RPS19;CLP1;RPSA;RPS3A;SEPP1
Azathioprine-20_mg/kg_in_Corn_Oil-Rat-Kidney-1d-up	8/360	0,156055815	0,329604613	0	0	1,527300496	2,837024125	RPS17;RPL32;RPS5;RPS6;RAPGEF5;SEPP1;RPL39;TPT1
Abamectin-10_mg/kg_in_Water-Rat-Liver-5d-dn	7/304	0,156261262	0,329950054	0	0	1,582564659	2,937597533	EEF1B2;C1QA;RPS8;RPS6;RPS20;RPL10A;TPT1
Aztreonam-1100_mg/kg_in_CMC-Rat-Spleen-5d-dn	7/304	0,156261262	0,329861619	0	0	1,582564659	2,937597533	RPS17;RPL32;ARL2BP;RPS8;RPL35A;RPS3A;TPT1
Carboplatin-14_mg/kg_in_Saline-Rat-Kidney-5d-dn	7/304	0,156261262	0,329773232	0	0	1,582564659	2,937597533	EEF1B2;RPS17;RPL32;CLP1;RPS8;RPS3A;SEPP1
Clarithromycin-476_mg/kg_in_Water-Rat-Liver-1d-up	7/304	0,156261262	0,329684892	0	0	1,582564659	2,937597533	EEF1B2;RPS17;RPS8;RPS20;RPS3A;RPL39;TPT1
Hexachlorophene-8_mg/kg_in_Corn_Oil-Rat-Liver-3d-up	7/304	0,156261262	0,329596599	0	0	1,582564659	2,937597533	RPS17;RPS19;RPS8;RPS20;RPSA;SEPP1;RPL39
Idarubicin-1_mg/kg_in_Saline-Rat-Kidney-5d-dn	7/304	0,156261262	0,329508353	0	0	1,582564659	2,937597533	RPS17;RPL32;RPS19;RPS6;RPL35A;SEPP1;TPT1
Ketoconazole-227_mg/kg_in_Corn_Oil-Rat-Liver-0.25d-dn	7/304	0,156261262	0,329420155	0	0	1,582564659	2,937597533	RPS17;RPL32;RPS8;RPL35A;RPSA;RPS3A;TPT1
Lead_(II)_Acetate-200_mg/kg_in_Saline-Rat-Bone_marrow-0.25d-dn	7/304	0,156261262	0,329332004	0	0	1,582564659	2,937597533	EEF1B2;RPS19;RPS6;NKG7;RPL10A;SEPP1;RPL39
Lorazepam-2000_mg/kg_in_CMC-Rat-Liver-1d-dn	7/304	0,156261262	0,329243901	0	0	1,582564659	2,937597533	EEF1B2;C1QA;RPS20;RPSA;RPL10A;SEPP1;RPL39
Meloxicam-6_mg/kg_in_Corn_Oil-Rat-Liver-3d-dn	6/249	0,156430059	0,329511406	0	0	1,656108972	3,072324389	EEF1B2;RPS17;RPL32;RPS8;RPS3A;TPT1
Nicotine-75_mg/kg_in_Water-Rat-Brain-3d-up	6/249	0,156430059	0,329423301	0	0	1,656108972	3,072324389	RPS17;RPL32;RPL35A;RPS3A;RPL10A;SEPP1
Amoxapine-313_mg/kg_in_CMC-Rat-Heart-5d-up	7/305	0,158088061	0,332825867	0	0	1,577375922	2,909632444	EEF1B2;RPL32;ARL2BP;RPS8;RPS6;SEPP1;RPL39
Bisphenol_A-150_mg/kg_in_Corn_Oil-Rat-Liver-1d-up	7/305	0,158088061	0,332736923	0	0	1,577375922	2,909632444	C1QA;RPS17;RPS19;RPL35A;RPS20;RPSA;RPL39
Buflomedil-615_mg/kg_in_CMC-Rat-Heart-3d-dn	7/305	0,158088061	0,332648027	0	0	1,577375922	2,909632444	C1QA;COL5A3;RPSA;RPL10A;SEPP1;GOLGA7;TPT1
Dexibuprofen-239_mg/kg_in_CMC-Rat-Kidney-3d-dn	7/305	0,158088061	0,332559179	0	0	1,577375922	2,909632444	EEF1B2;RPS19;CLP1;RPS6;ARL6IP5;RPS20;SEPP1
Diclofenac-10_mg/kg_in_Corn_Oil-Rat-Liver-5d-up	7/305	0,158088061	0,332470378	0	0	1,577375922	2,909632444	EEF1B2;C1QA;RPS6;RPS20;RPS3A;RPL10A;RPL39
Doxorubicin-3_mg/kg_in_Saline-Rat-Spleen-1d-dn	7/305	0,158088061	0,332381625	0	0	1,577375922	2,909632444	EEF1B2;C1QA;RPL32;RPS8;RPS5;RPL35A;RPS20
Droperidol-94_mg/kg_in_CMC-Rat-Heart-3d-up	7/305	0,158088061	0,332292919	0	0	1,577375922	2,909632444	RPS17;RPL32;RPS6;RPS3A;RPL39;TPT1;PDRG1
Enoxacin-750_mg/kg_in_Corn_Oil-Rat-Liver-1d-up	7/305	0,158088061	0,33220426	0	0	1,577375922	2,909632444	C1QA;RPS19;RPS8;FEM1B;RPS20;SEPP1;GOLGA7
Gadopentetate_Dimeglumine-125_mg/kg_in_Saline-Rat-Kidney-3d-up	7/305	0,158088061	0,332115649	0	0	1,577375922	2,909632444	EEF1B2;RPS17;RPL32;RPS19;RPS20;RPSA;RAPGEF5
Glimepiride-2500_mg/kg_in_CMC-Rat-Liver-1d-up	7/305	0,158088061	0,332027085	0	0	1,577375922	2,909632444	RPS6;RPS20;RPSA;RPL10A;SEPP1;RPL39;TPT1
Mefenamic_Acid-93_mg/kg_in_Corn_Oil-Rat-Kidney-3d-dn	7/305	0,158088061	0,331938568	0	0	1,577375922	2,909632444	EEF1B2;RPS19;RPS6;RPSA;RPL10A;SEPP1;RPL39
Penciclovir-1200_mg/kg_in_Corn_Oil-Rat-Kidney-5d-dn	7/305	0,158088061	0,331850098	0	0	1,577375922	2,909632444	RPS17;RPL32;RPS8;RPS6;RPS3A;SEPP1;TPT1
Phenothiazine-36_mg/kg_in_Corn_Oil-Rat-Liver-0.25d-dn	7/305	0,158088061	0,331761675	0	0	1,577375922	2,909632444	RPS17;RPS19;CLP1;RPS3A;SEPP1;RPL39;TPT1
Promazine-100_mg/kg_in_Corn_Oil-Rat-Liver-1d-up	7/305	0,158088061	0,3316733	0	0	1,577375922	2,909632444	RPL32;RPS19;RPS6;RPS3A;RPL10A;SEPP1;GOLGA7
Promethazine-2.3_mg/kg_in_Saline-Rat-Liver-5d-up	7/305	0,158088061	0,331584971	0	0	1,577375922	2,909632444	EEF1B2;RPS17;RPL32;RPL35A;RPSA;RPL39;TPT1
Sodium_Arsenite-30_mg/kg_in_Saline-Rat-Kidney-3d-up	7/305	0,158088061	0,33149669	0	0	1,577375922	2,909632444	RPS17;RPS19;ARL2BP;RPL35A;RPS20;RPS3A;TPT1
Torsemide-3_mg/kg_in_Corn_Oil-Rat-Liver-0.25d-up	7/305	0,158088061	0,331408455	0	0	1,577375922	2,909632444	EEF1B2;C1QA;RPL32;RPS8;RPS5;RPSA;TPT1
Venlafaxine-320_mg/kg_in_Water-Rat-Heart-5d-dn	7/305	0,158088061	0,331320268	0	0	1,577375922	2,909632444	RPS17;RPL32;RPS19;ARL2BP;RPL35A;RPS20;RPSA
Vincristine-0.05_mg/kg_in_Saline-Rat-Bone_marrow-1d-up	7/305	0,158088061	0,331232127	0	0	1,577375922	2,909632444	EEF1B2;RPS17;ARL2BP;RPS8;RPL35A;RPS3A;TPT1
Bisphenol_A-150_mg/kg_in_Corn_Oil-Rat-Liver-5d-up	8/362	0,159401034	0,333894293	0	0	1,518862372	2,789135618	EEF1B2;C1QA;RPS19;RPS6;RPSA;RPL10A;SEPP1;RPL39
Doxorubicin-0.65_mg/kg_in_Saline-Rat-Liver-1d-up	8/362	0,159401034	0,333805515	0	0	1,518862372	2,789135618	EEF1B2;RPS17;RPL32;RPS20;RPL10A;SEPP1;RPL39;TPT1
Alpha-Naphthoflavone-80_mg/kg_in_CMC-Rat-Liver-28d-dn	7/306	0,159924647	0,334813003	0	0	1,572221099	2,881963866	C1QA;RPS17;RPL32;RPS19;RPS8;RPS3A;TPT1
Betamethasone-79_mg/kg_in_Corn_Oil-Rat-Bone_marrow-5d-up	7/306	0,159924647	0,334724028	0	0	1,572221099	2,881963866	C1QA;RPL32;RPS19;RPS6;NKG7;RPL10A;SEPP1
Bithionol-59_mg/kg_in_Corn_Oil-Rat-Liver-5d-up	7/306	0,159924647	0,334635101	0	0	1,572221099	2,881963866	EEF1B2;RPS17;RPS19;RPS6;RPS20;RPL39;TPT1
Catechol-40_mg/kg_in_Saline-Rat-Kidney-3d-up	7/306	0,159924647	0,33454622	0	0	1,572221099	2,881963866	RPS5;RPL13A;RPL35A;RPS20;SEPP1;RPL39;GOLGA7
Cetraxate-1503_mg/kg_in_CMC-Rat-Liver-5d-up	7/306	0,159924647	0,334457387	0	0	1,572221099	2,881963866	EEF1B2;RPS17;RPL32;RPS19;RPS8;RPS3A;RPL39
Chlorambucil-4.5_mg/kg_in_Corn_Oil-Rat-Bone_marrow-5d-up	7/306	0,159924647	0,334368601	0	0	1,572221099	2,881963866	C1QA;RPS5;RPS20;RPL10A;SEPP1;RPL39;TPT1
Chloroquine-21.95_uM_in_DMSO-Rat-Primary_rat_hepatocytes-0.67d-up	7/306	0,159924647	0,334279862	0	0	1,572221099	2,881963866	EEF1B2;RPL32;RPS5;RPS6;RPS3A;RPL39;TPT1
Clemastine-5.4_mg/kg_in_CMC-Rat-Heart-3d-up	7/306	0,159924647	0,33419117	0	0	1,572221099	2,881963866	RPS17;RPS19;FEM1B;RPS6;RPSA;RPL10A;RPL39
Ibuprofen-54_mg/kg_in_Corn_Oil-Rat-Kidney-1d-dn	7/306	0,159924647	0,334102525	0	0	1,572221099	2,881963866	EEF1B2;RPS17;RPL32;RPS6;RPL35A;SEPP1;RPL39
Lovastatin-450_mg/kg_in_Corn_Oil-Rat-Kidney-1d-dn	7/306	0,159924647	0,334013927	0	0	1,572221099	2,881963866	EEF1B2;RPS8;FEM1B;RPSA;RPL10A;SEPP1;TPT1
Methotrexate-0.3_mg/kg_in_Corn_Oil-Rat-Bone_marrow-1d-up	7/306	0,159924647	0,333925376	0	0	1,572221099	2,881963866	C1QA;RPS17;RPS3A;RPL10A;SEPP1;RPL39;TPT1
Mitomycin_C-1.7_mg/kg_in_Saline-Rat-Spleen-1d-up	7/306	0,159924647	0,333836872	0	0	1,572221099	2,881963866	RPL32;RPS19;RPS6;RPS3A;RPL10A;SEPP1;RPL39
Niacin-2625_mg/kg_in_Water-Rat-Liver-1d-dn	7/306	0,159924647	0,333748415	0	0	1,572221099	2,881963866	EEF1B2;C1QA;RPS17;CLP1;RPSA;RPL10A;SEPP1
Norethindrone-0.08_mg/kg_in_Corn_Oil-Rat-Liver-3d-up	7/306	0,159924647	0,333660005	0	0	1,572221099	2,881963866	RPL32;RPS8;RPL35A;RPS20;RPSA;SEPP1;RPL39
Noscapine-570_mg/kg_in_CMC-Rat-Bone_marrow-3d-up	7/306	0,159924647	0,333571642	0	0	1,572221099	2,881963866	C1QA;RPL32;RPS6;ARL6IP5;RPS20;RPS3A;RPL39
Progesterone-164_mg/kg_in_Corn_Oil-Rat-Liver-3d-up	7/306	0,159924647	0,333483325	0	0	1,572221099	2,881963866	RPS17;RPS19;RPS8;FEM1B;RPS20;RPSA;RPS3A

Sertraline-210_mg/kg_in_Corn_Oil-Rat-Liver-1d-dn	7/306	0,159924647	0,333395055	0	0	1,572221099	2,881963866	EEF1B2;RPS19;CLP1;RPL10A;SEPP1;RPL39;TPT1
Streptozotocin-138_mg/kg_in_Saline-Rat-Liver-3d-dn	7/306	0,159924647	0,333306832	0	0	1,572221099	2,881963866	RPS8;FEM1B;RPS6;RPS20;RPS3A;SEPP1;RPL39
Rabeprazole-1024_mg/kg_in_Water-Rat-Liver-5d-up	6/251	0,160510918	0,334440209	0	0	1,642912885	3,005533845	RPS6;RPSA;RAPGEF5;RPL10A;SEPP1;TPT1
Roxarsone-11_mg/kg_in_Corn_Oil-Rat-Kidney-5d-dn	8/363	0,161086303	0,335550311	0	0	1,514678178	2,765522157	C1QA;CLP1;RPSA;RAPGEF5;RPL10A;SEPP1;RPL39;GOLGA7
Clofibrate-130_mg/kg_in_Corn_Oil-Rat-Kidney-1d-dn	7/307	0,16177095	0,336887362	0	0	1,567099858	2,854588092	RPS17;RPL32;ARL2BP;RPS8;RPL35A;SEPP1;RPL39
Cobalt_(II)_Chloride-10_mg/kg_in_Saline-Rat-Heart-5d-dn	7/307	0,16177095	0,336798309	0	0	1,567099858	2,854588092	RPS17;RPL32;RPS8;RPS6;RPSA;RAPGEF5;TPT1
Lovastatin-1500_mg/kg_in_Corn_Oil-Rat-Kidney-1d-dn	7/307	0,16177095	0,336709303	0	0	1,567099858	2,854588092	RPS19;ARL2BP;FEM1B;RPSA;RPL10A;SEPP1;GOLGA7
Methyl_Methanesulfonate-113_mg/kg_in_Water-Rat-Bone_marrow-3d-up	7/307	0,16177095	0,336620345	0	0	1,567099858	2,854588092	C1QA;RPL32;RPS19;NKG7;RPSA;RPL10A;SEPP1
Procarbazine-54_mg/kg_in_Water-Rat-Bone_marrow-1d-up	7/307	0,16177095	0,336531433	0	0	1,567099858	2,854588092	EEF1B2;C1QA;RPS8;NKG7;RPL10A;SEPP1;TPT1
Sotalol-6666_uM_in_DMSO-Rat-Primary_rat_hepatocytes-0.67d-dn	7/307	0,16177095	0,336442568	0	0	1,567099858	2,854588092	CXCL11;RPL32;RPS20;RPSA;SEPP1;SLC16A3;TPT1
Thioguanine-12_mg/kg_in_Corn_Oil-Rat-Liver-1d-up	7/307	0,16177095	0,33635375	0	0	1,567099858	2,854588092	RPS19;FEM1B;RPS20;RPSA;RPS3A;RPL39;TPT1
Tiapride-1200_mg/kg_in_Water-Rat-Heart-5d-up	7/307	0,16177095	0,336264979	0	0	1,567099858	2,854588092	EEF1B2;RPS17;RPL32;FUNDC1;RPS6;SEPP1;RPL39
Trichloroethane-3000_mg/kg_in_Water-Rat-Liver-1d-up	7/307	0,16177095	0,336176254	0	0	1,567099858	2,854588092	EEF1B2;RPS17;RPS6;RPSA;RPL10A;SEPP1;RPL39
Azithromycin-225_mg/kg_in_Water-Rat-Kidney-5d-dn	6/252	0,16256859	0,337744715	0	0	1,636393389	2,972762683	C1QA;RPS17;ARL6IP5;RPSA;RPL39;SEPP1
Diethylstilbestrol-2.8_mg/kg_in_Corn_Oil-Rat-Kidney-3d-up	6/252	0,16256859	0,337655647	0	0	1,636393389	2,972762683	RPS19;ARL2BP;RPS20;RPS3A;GOLGA7;TPT1
Harringtonine-23_mg/kg_in_Saline-Rat-Bone_marrow-3d-dn	6/252	0,16256859	0,337566626	0	0	1,636393389	2,972762683	EEF1B2;RPS8;RPS6;NKG7;RPS3A;SLC16A3
Rosiglitazone-1800_mg/kg_in_Corn_Oil-Rat-Liver-3d-dn	6/252	0,16256859	0,337477652	0	0	1,636393389	2,972762683	RPS17;CLP1;RPS8;RPS3A;SEPP1;TPT1
Chlorpromazine-43.9_uM_in_DMSO-Rat-Primary_rat_hepatocytes-1d-dn	8/364	0,162779945	0,337827364	0	0	1,510516974	2,742126083	RPS17;RPS19;ARL2BP;RPS8;FUNDC1;RPS20;RPL39;TPT1
Chloramphenicol-1500_mg/kg_in_CMC-Rat-Kidney-1d-dn	7/308	0,1636269	0,339495644	0	0	1,562011871	2,827501472	RPS17;RPSA;RPS3A;RPL10A;SEPP1;RPL39;TPT1
Clozapine-95_mg/kg_in_CMC-Rat-Heart-3d-dn	7/308	0,1636269	0,339406233	0	0	1,562011871	2,827501472	ARL2BP;RPS8;COL5A3;RPS20;RPSA;RPL10A;RPL39
Fludrocortisone_Acetate-125_mg/kg_in_CMC-Rat-Heart-3d-up	7/308	0,1636269	0,339316868	0	0	1,562011871	2,827501472	C1QA;RPL32;RPS19;RPS6;ARL6IP5;RPS3A;SEPP1
Ketoconazole-25_mg/kg_in_Corn_Oil-Rat-Liver-1d-up	7/308	0,1636269	0,339227551	0	0	1,562011871	2,827501472	EEF1B2;RPS19;RPS8;RPS6;RPS20;RPL10A;TPT1
Lomustine-8.75_mg/kg_in_Corn_Oil-Rat-Liver-3d-dn	7/308	0,1636269	0,33913828	0	0	1,562011871	2,827501472	EEF1B2;RPS17;RPL32;RPS8;RPS6;RPS3A;RPL10A
Methotrexate-0.3_mg/kg_in_Corn_Oil-Rat-Spleen-1d-dn	7/308	0,1636269	0,339049057	0	0	1,562011871	2,827501472	EEF1B2;RPS19;RPS6;RPSA;RPL10A;SEPP1;RPL39
Nafenopin-338_mg/kg_in_Corn_Oil-Rat-Liver-1d-dn	7/308	0,1636269	0,33895988	0	0	1,562011871	2,827501472	C1QA;RPS17;RPL32;RPS6;SEPP1;RPL39;TPT1
Omeprazole-30_mg/kg_in_Corn_Oil-Rat-Liver-0.25d-up	7/308	0,1636269	0,338870751	0	0	1,562011871	2,827501472	RPS19;RPS6;RPS20;RPS3A;SEPP1;RPL39;GOLGA7
Pralidoxime_Chloride-48_mg/kg_in_Saline-Rat-Heart-5d-dn	7/308	0,1636269	0,338781668	0	0	1,562011871	2,827501472	EEF1B2;ARL2BP;RPS8;FUNDC1;RPSA;RAPGEF5;TOMM20
Rofecoxib-1550_mg/kg_in_Corn_Oil-Rat-Liver-3d-up	7/308	0,1636269	0,338692632	0	0	1,562011871	2,827501472	EEF1B2;C1QA;RPS19;CLP1;RPS8;RPSA;SEPP1
Tranilast-1100_mg/kg_in_CMC-Rat-Liver-3d-up	7/308	0,1636269	0,338603643	0	0	1,562011871	2,827501472	RPS19;RPS8;RPS6;RPSA;RPS3A;RPL10A;TPT1
Cisplatin-1.17_mg/kg_in_Saline-Rat-Kidney-0.25d-dn	6/253	0,164637618	0,340605694	0	0	1,629925431	2,940399296	RPL32;ARL2BP;RPS6;RPL10A;SEPP1;TPT1
Dideoxycytidine-1200_mg/kg_in_CMC-Rat-Liver-5d-up	6/253	0,164637618	0,340516249	0	0	1,629925431	2,940399296	EEF1B2;RPS3A;RAPGEF5;RPL39;SEPP1;TPT1
Mexiletine-350_mg/kg_in_CMC-Rat-Heart-3d-up	6/253	0,164637618	0,340426851	0	0	1,629925431	2,940399296	RPS17;RPL32;RPS6;RPS20;RPS3A;SEPP1
Phentolamine-44_mg/kg_in_Saline-Rat-Heart-3d-dn	6/253	0,164637618	0,3403375	0	0	1,629925431	2,940399296	EEF1B2;RPS8;FUNDC1;RPS20;RPSA;TPT1
Troxipide-1200_mg/kg_in_CMC-Rat-Heart-5d-up	6/253	0,164637618	0,340248196	0	0	1,629925431	2,940399296	RPS19;RPS8;RPSA;RAPGEF5;RPL10A;TPT1
Alendronic_Acid-138_mg/kg_in_CMC-Rat-Heart-3d-up	7/309	0,165492425	0,341925062	0	0	1,556956817	2,800700409	RPS17;RPL32;ARL2BP;RPS8;RPL35A;RPS3A;TPT1
Clofibrate-500_uM_in_DMSO-Rat-Primary_rat_hepatocytes-0.67d-up	7/309	0,165492425	0,341835388	0	0	1,556956817	2,800700409	C1QA;RPS17;RPL32;RPS19;RPS6;RPL13A;RPL39
Clonazepam-2500_mg/kg_in_CMC-Rat-Liver-5d-dn	7/309	0,165492425	0,341745762	0	0	1,556956817	2,800700409	EEF1B2;C1QA;RPL32;RPS19;RPS6;RPS3A;SEPP1
Cyclosporin_A-70_mg/kg_in_Corn_Oil-Rat-Liver-5d-up	7/309	0,165492425	0,341656183	0	0	1,556956817	2,800700409	RPS19;ARL2BP;RPS8;RPS5;RPS20;RPSA;SEPP1
Epinephrine-0.0375_mg/kg_in_Saline-Rat-Heart-5d-dn	7/309	0,165492425	0,34156665	0	0	1,556956817	2,800700409	ARL2BP;RPS8;ARL6IP5;RPS20;RAPGEF5;SEPP1;TPT1
Lomustine-8.75_mg/kg_in_Corn_Oil-Rat-Liver-1d-up	7/309	0,165492425	0,341477164	0	0	1,556956817	2,800700409	EEF1B2;RPS19;RPS8;RPS6;RPS20;RPS3A;RPL39
Nystatin-134_mg/kg_in_Corn_Oil-Rat-Liver-3d-dn	7/309	0,165492425	0,341387726	0	0	1,556956817	2,800700409	RPL32;RPS19;RPS8;RPS6;RPS20;RPSA;TPT1
Oxiconazole-1500_mg/kg_in_Corn_Oil-Rat-Liver-5d-dn	7/309	0,165492425	0,341298334	0	0	1,556956817	2,800700409	RPS17;RPL32;RPS19;RPS6;RPSA;RPL10A;RPL39
Roxithromycin-312_mg/kg_in_CMC-Rat-Kidney-5d-dn	7/309	0,165492425	0,341208989	0	0	1,556956817	2,800700409	RPS17;RPL32;RPS19;RPS6;RPSA;RPL10A;SEPP1
Sulfadoxine-380_mg/kg_in_Corn_Oil-Rat-Liver-0.25d-up	7/309	0,165492425	0,34111969	0	0	1,556956817	2,800700409	RPS19;RPS8;RPSA;RPS3A;RPL10A;RPL39;TPT1
Temafloxacin-1000_mg/kg_in_CMC-Rat-Kidney-1d-up	7/309	0,165492425	0,341030439	0	0	1,556956817	2,800700409	EEF1B2;RPS17;RPS20;RPS3A;RPL10A;RPL39;TPT1
Tropisetron-100_mg/kg_in_CMC-Rat-Liver-1d-up	7/309	0,165492425	0,340941234	0	0	1,556956817	2,800700409	EEF1B2;RPS19;RPS6;RPS3A;RPL10A;RPL39;TPT1
Vecuronium_Bromide-0.005_mg/kg_in_Saline-Rat-Heart-5d-up	7/309	0,165492425	0,340852075	0	0	1,556956817	2,800700409	RPL32;RPS19;COL5A3;RPS6;RPSA;RPL10A;RPL39
Nimodipine-10_mg/kg_in_Corn_Oil-Rat-Heart-0.25d-dn	8/366	0,166192139	0,342203735	0	0	1,502262783	2,695976859	EEF1B2;RPS17;RPS19;ARL2BP;RPS6;RPL13A;RPL35A;RPS3A
11-Dichloroethene-600_mg/kg_in_Water-Rat-Liver-1d-up	7/310	0,167367451	0,344533729	0	0	1,551934375	2,774181362	C1QA;RPS17;RPS19;RPS6;RPSA;RPL39;PDRG1
Amprenavir-600_mg/kg_in_CMC-Rat-Kidney-3d-up	7/310	0,167367451	0,344443702	0	0	1,551934375	2,774181362	EEF1B2;C1QA;RPS17;RPS3A;RPL10A;RPL39;PDRG1
Auranofin-33_mg/kg_in_CMC-Rat-Kidney-5d-up	7/310	0,167367451	0,344353722	0	0	1,551934375	2,774181362	EEF1B2;RPS17;RPS19;RPS6;RPSA;RPS3A;RPL10A
Clopidogrel-400_mg/kg_in_Corn_Oil-Rat-Bone_marrow-1d-up	7/310	0,167367451	0,344263789	0	0	1,551934375	2,774181362	C1QA;RPS17;ARL2BP;RPS8;RPS6;RPS20;RPL39
Diazepam-710_mg/kg_in_CMC-Rat-Liver-1d-dn	7/310	0,167367451	0,344173903	0	0	1,551934375	2,774181362	EEF1B2;C1QA;RPL32;RPS19;RPS6;RPL10A;SEPP1
Fenofibrate-100_mg/kg_in_Corn_Oil-Rat-Liver-3d-dn	7/310	0,167367451	0,344084063	0	0	1,551934375	2,774181362	C1QA;RPS17;RPS3A;RPL10A;SEPP1;RPL39;TPT1
Fenofibrate-43_mg/kg_in_Corn_Oil-Rat-Kidney-1d-up	7/310	0,167367451	0,343994271	0	0	1,551934375	2,774181362	RPS19;RPS5;RPS6;RPS20;RAPGEF5;GOLGA7;TPT1
Glyburide-1500_mg/kg_in_CMC-Rat-Heart-3d-up	7/310	0,167367451	0,343904526	0	0	1,551934375	2,774181362	RPS17;RPL32;RPS8;RPS6;RPL35A;RPS20;RPS3A
Lead(IV)_Acetate-200_mg/kg_in_Saline-Rat-Spleen-1d-up	7/310	0,167367451	0,343814827	0	0	1,551934375	2,774181362	RPS19;RPS6;RPS20;RPS3A;RPL10A;SEPP1;RPL39
Lead(IV)_Acetate-200_mg/kg_in_Saline-Rat-Spleen-3d-dn	7/310	0,167367451	0,343725175	0	0	1,551934375	2,774181362	EEF1B2;C1QA;RPS17;ARL2BP;ARL6IP5;RPSA;TPT1
Leflunomide-60_mg/kg_in_Corn_Oil-Rat-Kidney-5d-up	7/310	0,167367451	0,34363557	0	0	1,551934375	2,774181362	CXCL11;RPS19;RPS6;RPS20;SEPP1;RPL39;GOLGA7
Methotrexate-27_mg/kg_in_Corn_Oil-Rat-Spleen-1d-up	7/310	0,167367451	0,343546012	0	0	1,551934375	2,774181362	C1QA;RPS8;FUNDC1;RPS20;RPS3A;SEPP1;TPT1
Myrtecaine-140_mg/kg_in_Corn_Oil-Rat-Heart-1d-up	7/310	0,167367451	0,3434565	0	0	1,551934375	2,774181362	RPS19;RPS6;RPS3A;RAPGEF5;RPL10A;RPL39;TPT1
Perhexiline-320_mg/kg_in_CMC-Rat-Liver-5d-dn	7/310	0,167367451	0,343367035	0	0	1,551934375	2,774181362	RPS17;RPL32;RPS19;RPS6;RPS20;RPL10A;TPT1
Roxithromycin-312_mg/kg_in_CMC-Rat-Liver-1d-up	7/310	0,167367451	0,343277616	0	0	1,551934375	2,774181362	C1QA;RPS17;RPL32;RPL35A;RPSA;RPL39;TPT1
Tenidap-75_mg/kg_in_Corn_Oil-Rat-Kidney-1d-up	7/310	0,167367451	0,343188244	0	0	1,551934375	2,774181362	RPL32;RPS5;RPSA;RPS3A;RAPGEF5;SEPP1;TPT1
Allyl_Alcohol-30_uM_in_DMSO-Rat-Primary_rat_hepatocytes-0.67d-dn	5/200	0,167630974	0,343639133	0	0	1,718213058	3,068711857	RPS17;CXCL11;ARL6IP5;RPL10A;SEPP1
Amiodarone-37.04_uM_in_DMSO-Rat-Primary_rat_hepatocytes-1d-up	6/255	0,168809319	0,345964663	0	0	1,617141702	2,876871622	EEF1B2;RPS8;RPS6;RPS20;RPSA;RPS3A
17-Methyltestosterone-2000_mg/kg_in_CMC-Rat-Liver-5d-up	7/311	0,169251907	0,346781482	0	0	1,546944233	2,747940842	RPS19;FEM1B;RPS6;RPSA;RPL10A;RPL39;GOLGA7
Amitriptyline-72_uM_in_DMSO-Rat-Primary_rat_hepatocytes-0.67d-dn	7/311	0,169251907	0,346691292	0	0	1,546944233	2,747940842	CXCL11;RPS8;ARL6IP5;RPSA;SEPP1;RPL39;TPT1
Celecoxib-400_mg/kg_in_Corn_Oil-Rat-Heart-5d-dn	7/311	0,169251907	0,346601149	0	0	1,546944233	2,747940842	RPS17;RPL32;ARL2BP;RPS6;RPL35A;RPSA;RPS3A
Cisapride-250_mg/kg_in_Corn_Oil-Rat-Intestine-3d-dn	7/311	0,169251907	0,346511052	0	0	1,546944233	2,747940842	C1QA;RPS17;RPL32;RPL35A;RPSA;SEPP1;SLC16A3
Citalopram-90_mg/kg_in_Corn_Oil-Rat-Liver-5d-dn	7/311	0,169251907	0,346421003	0	0	1,546944233	2,747940842	C1QA;RPS17;RPL32;RPS6;RPS3A;RPL10A;SEPP1
Cortisone-206_mg/kg_in_CMC-Rat-Kidney-1d-dn	7/311	0,169251907	0,346331	0	0	1,546944233	2,747940842	RPS17;RPL32;RPS19;RPL35A;RPL39;GOLGA7;TPT1
Diethylstilbestrol-2.8_mg/kg_in_Corn_Oil-Rat-Bone_marrow-3d-up	7/311	0,169251907	0,346241044	0	0	1,546944233	2,747940842	C1QA;RPS5;NKG7;SEPP1;RPL39;GOLGA7;TPT1
Ifosfamide-143_mg/kg_in_Saline-Rat-Bone_marrow-3d-up	7/311	0,169251907	0,346151134	0	0	1,546944233	2,747940842	EEF1B2;C1QA;ARL2BP;RPS8;RPL35A;RPS20;SEPP1
Leflunomide-30_mg/kg_in_Corn_Oil-Rat-Bone_marrow-1d-up	7/311	0,169251907	0,346061272	0	0	1,546944233	2,747940842	C1QA;RPS17;RPS19;CLP1;SEPP1;RPL39;TPT1
Phentolamine-44_mg/kg_in_Saline-Rat-Heart-5d-dn	7/311	0,169251907	0,345971456	0	0	1,546944233	2,747940842	EEF1B2;RPS19;RPS8;COL5A3;RAPGEF5;SEPP1;GOLGA7
Propylthiouracil-625_mg/kg_in_CMC-Rat-Liver-3d-up	7/311	0,169251907	0,345881686	0	0	1,546944233	2,747940842	RPS19;RPS8;RPS6;RPS20;RPSA;RPL10A;RPL39
Salicylamide-1300_mg/kg_in_CMC-Rat-Liver-1d-dn	7/311	0,169251907	0,345791963	0	0	1,546944233	2,747940842	EEF1B2;RPS17;RPL32;RPS8;RPL35A;RPS20;SEPP1
Thiabendazole-10_mg/kg_in_Corn_Oil-Rat-Liver-0.25d-up	7/311	0,169251907	0,345702287	0	0	1,546944233	2,747940842	C1QA;RPL32;RPS19;RPS6;RPL10A;SEPP1;GOLGA7
Cytarabine-23_mg/kg_in_Saline-Rat-Bone_marrow-3d-up	8/368	0,169637195	0,346399415	0	0	1,494098312	2,650669762	RPS19;RPS5;RPS6;RPL35A;RPS20;RPSA;RPL10A;SEPP1
N-Nitrosodiethylamine-34_mg/kg_in_Saline-Rat-Liver-1d-dn	6/256	0,170911779	0,348911658	0	0	1,610824742	2,845695498	EEF1B2;RPL32;RPS19;RPS6;RPS3A;RPL10A
Neomycin-56_mg/kg_in_Corn_Oil-Rat-Kidney-0.25d-dn	6/256	0,170911779	0,348821243	0	0	1,610824742	2,845695498	EEF1B2;RPS19;ARL2BP;CLP1;RPS8;RPSA
Rifapentine-75_mg/kg_in_Corn_Oil-Rat-Kidney-5d-dn	6/256	0,170911779	0,348730874	0	0	1,610824742	2,845695498	CLP1;RPS8;FEM1B;GOLGA7;SEPP1;TPT1
33'4'5-Tetrachlorosalicylanilide-5_mg/kg_in_Corn_Oil-Rat-Liver-0.25d-up	7/312	0,171145717	0,349117759	0	0	1,541986078	2,721975411	RPS19;ARL2BP;CLP1;FEM1B;RPS6;RPSA;SEPP1
Altretamine-40_mg/kg_in_Corn_Oil-Rat-Spleen-1d-up	7/312	0,171145717	0,349027361	0	0	1,541986078	2,721975411	ARL2BP;CLP1;RPS8;FUNDC1;RPS20;RPSA;TPT1
Amlodipine-19_mg/kg_in_Corn_Oil-Rat-Liver-1d-up	7/312	0,171145717	0,348937009	0	0	1,541986078	2,721975411	EEF1B2;C1QA;RPL32;RPS19;RPS5;RPL35A;RPSA
Azithromycin-50_mg/kg_in_Water-Rat-Liver-5d-up	7/312	0,171145717	0,348846705	0	0	1,541986078	2,721975411	EEF1B2;RPL32;RPS3A;RPL10A;SEPP1;RPL39;TPT1
Bromisovalum-250_mg/kg_in_Corn_Oil-Rat-Liver-3d-up	7/312	0,171145717	0,348756447	0	0	1,541986078	2,721975411	RPS17;RPS19;RPSA;RPS3A;RAPGEF5;RPL10A;RPL39
Chlorambucil-1540_uM_in_DMSO-Rat-Primary_rat_hepatocytes-1d-dn	7/312	0,171145717	0,348666235	0	0	1,541986078	2,721975411	RPS19;RPS8;FUNDC1;RPS20;RPSA;SEPP1;TPT1
Cyclosporin_A-70_mg/kg_in_Corn_Oil-Rat-Bone_marrow-1d-up	7/312	0,171145717	0,348576071	0	0	1,541986078	2,721975411	C1QA;RPS17;NKG7;RPS20;RPL10A;RPL39;TPT1

Emetine-1_mg/kg_in_Saline-Rat-Heart-1d-up	7/312	0,171145717	0,348485953	0	0	1,541986078	2,721975411	ARL2BP;RPS8;FUNDC1;ARL6IP5;RPL35A;RPSA;TOMM20
Griseofulvin-2500_mg/kg_in_Corn_Oil-Rat-Liver-5d-dn	7/312	0,171145717	0,348395882	0	0	1,541986078	2,721975411	EEF1B2;C1QA;CLP1;RPS8;RPS20;SEPP1;RPL39
Lead_(II)_Acetate-200_mg/kg_in_Saline-Rat-Kidney-5d-dn	7/312	0,171145717	0,348305857	0	0	1,541986078	2,721975411	RPS17;RPL32;RPS8;RPSA;RPS3A;SEPP1;TPT1
Moclobemide-266_mg/kg_in_Water-Rat-Brain-5d-dn	7/312	0,171145717	0,348215879	0	0	1,541986078	2,721975411	RPL32;RPS8;FEM1B;RPL35A;RPS3A;TOMM20;TPT1
Progesterone-164_mg/kg_in_Corn_Oil-Rat-Liver-5d-up	7/312	0,171145717	0,348125947	0	0	1,541986078	2,721975411	RPS17;RPS19;RPS8;RPS5;RPS20;RPSA;SEPP1
Rabeprazole-1024_mg/kg_in_Water-Rat-Liver-3d-up	7/312	0,171145717	0,348036062	0	0	1,541986078	2,721975411	RPS17;RPS6;RPS20;RPSA;RPS3A;RPL10A;SEPP1
Rosiglitazone-1800_mg/kg_in_Corn_Oil-Rat-Heart-5d-dn	7/312	0,171145717	0,347946223	0	0	1,541986078	2,721975411	RPS17;RPL32;ARL2BP;COL5A3;RPL35A;RPSA;RPS3A
Stanozolol-39_mg/kg_in_Water-Rat-Liver-1d-dn	7/312	0,171145717	0,34785643	0	0	1,541986078	2,721975411	RPS17;CLP1;FEM1B;FUNDC1;RPS20;RPS3A;RPL10A
Sulfadimethoxine-1100_mg/kg_in_CMC-Rat-Kidney-3d-up	7/312	0,171145717	0,347766684	0	0	1,541986078	2,721975411	EEF1B2;RPS17;ARL2BP;RPS3A;RAPGEF5;SEPP1;TPT1
Sulfanilamide-636_mg/kg_in_Corn_Oil-Rat-Kidney-5d-up	7/312	0,171145717	0,347676984	0	0	1,541986078	2,721975411	RPS17;RPL32;RPS19;RPS6;RPS20;RPL10A;RPL39
Valproic_Acid-235_mg/kg_in_Water-Rat-Kidney-1d-up	7/312	0,171145717	0,34758733	0	0	1,541986078	2,721975411	RPS17;RPSA;RPL10A;SEPP1;RPL39;TOMM20;TPT1
Rofecoxib-111_uM_in_DMSO-Rat-Primary_rat_hepatocytes-0.67d-dn	5/202	0,172419149	0,350083325	0	0	1,701201048	2,990416884	RPS17;CXCL11;ARL6IP5;RPS20;SEPP1
Doxofylline-13500_uM_in_DMSO-Rat-Primary_rat_hepatocytes-1d-up	6/257	0,173025168	0,351223254	0	0	1,604556942	2,814903468	EEF1B2;RPS17;RPL32;RPSA;RPL10A;PDRG1
Trans-Platinum(II)Diammine_Dichloride-0.5_mg/kg_in_Saline-Rat-Kidney-28d-up	6/257	0,173025168	0,351132756	0	0	1,604556942	2,814903468	ARL2BP;CLP1;RPS8;FUNDC1;RPS20;RPS3A
123-Trichloropropane-108_mg/kg_in_CMC-Rat-Heart-3d-dn	7/313	0,173048807	0,351090263	0	0	1,537059605	2,696281685	RPS19;ARL2BP;COL5A3;FUNDC1;RPS6;RPS20;RPSA
2-Acetylaminofluorene-30_mg/kg_in_CMC-Rat-Liver-3d-up	7/313	0,173048807	0,350999846	0	0	1,537059605	2,696281685	RPL32;RPS19;RPS6;RPSA;SEPP1;RPL39;TPT1
Epirubicin-2.7_mg/kg_in_Saline-Rat-Liver-3d-dn	7/313	0,173048807	0,350909475	0	0	1,537059605	2,696281685	EEF1B2;C1QA;RPS17;RPS19;RPS6;RPS20;RPL39
Gatifloxacin-770_mg/kg_in_Corn_Oil-Rat-Kidney-3d-up	7/313	0,173048807	0,350819151	0	0	1,537059605	2,696281685	RPS17;CXCL11;RPS8;RPS6;RPSA;RPS3A;RAPGEF5
Genistein-375_mg/kg_in_Corn_Oil-Rat-Liver-5d-up	7/313	0,173048807	0,350728873	0	0	1,537059605	2,696281685	EEF1B2;RPS19;RPS6;RPS20;RPL10A;GOLGA7;TPT1
Ifosfamide-17_mg/kg_in_Saline-Rat-Spleen-1d-up	7/313	0,173048807	0,350638642	0	0	1,537059605	2,696281685	C1QA;RPS19;RPS6;RPSA;RPL10A;SEPP1;RPL39
Pemoline-833_uM_in_DMSO-Rat-Primary_rat_hepatocytes-1d-dn	7/313	0,173048807	0,350548457	0	0	1,537059605	2,696281685	EEF1B2;RPS19;RPS5;RPS20;SEPP1;SLC16A3;TPT1
Phenacetin-619_mg/kg_in_Corn_Oil-Rat-Liver-1d-dn	7/313	0,173048807	0,350458319	0	0	1,537059605	2,696281685	C1QA;RPS17;RPL32;RPS6;SEPP1;RPL39;TPT1
Rolipram-75_mg/kg_in_CMC-Rat-Heart-1d-up	7/313	0,173048807	0,350368227	0	0	1,537059605	2,696281685	EEF1B2;CLP1;RPS8;FUNDC1;ARL6IP5;RPS20;RPS3A
Terbinafine-2000_mg/kg_in_CMC-Rat-Liver-3d-up	7/313	0,173048807	0,350278181	0	0	1,537059605	2,696281685	RPS17;RPL32;RPS6;RPS20;RPSA;RPL10A;RPL39
Thioguanine-24_mg/kg_in_Corn_Oil-Rat-Spleen-1d-dn	7/313	0,173048807	0,350188181	0	0	1,537059605	2,696281685	EEF1B2;RPS17;RPL32;RPS19;FEM1B;RPL13A;RPL35A
Zileuton-450_mg/kg_in_Corn_Oil-Rat-Liver-5d-dn	7/313	0,173048807	0,350098228	0	0	1,537059605	2,696281685	EEF1B2;C1QA;RPS6;RPS3A;RPL10A;RPL39;TPT1
1-Naphthyl_Isothiocyanate-166.67_uM_in_DMSO-Rat-Primary_rat_hepatocytes-1d-up	8/371	0,174865449	0,353682659	0	0	1,482016654	2,584249447	RPS17;RPL32;ARL2BP;RPS8;RPS5;RPS3A;TPT1;PDRG1
Ethylene_Glycol-3525_mg/kg_in_Water-Rat-Kidney-1d-up	8/371	0,174865449	0,353591855	0	0	1,482016654	2,584249447	EEF1B2;RPS17;RPS8;RPS5;RPL10A;SEPP1;TOMM20;TPT1
3-Acetamidophenol-700_mg/kg_in_Corn_Oil-Rat-Liver-5d-dn	7/314	0,1749611	0,353694462	0	0	1,532164511	2,670856326	EEF1B2;RPS17;CLP1;RPS8;RPS20;RPS3A;SEPP1
Atorvastatin-300_mg/kg_in_Corn_Oil-Rat-Kidney-3d-dn	7/314	0,1749611	0,353603702	0	0	1,532164511	2,670856326	EEF1B2;RPS19;ARL2BP;FUNDC1;RPS6;RPL10A;RPL39
Baclofen-19_mg/kg_in_CMC-Rat-Heart-5d-dn	7/314	0,1749611	0,353512988	0	0	1,532164511	2,670856326	EEF1B2;COL5A3;FUNDC1;ARL6IP5;RPL13A;SEPP1;TPT1
Benzothiazyl_Disulfide-2600_mg/kg_in_Corn_Oil-Rat-Kidney-1d-up	7/314	0,1749611	0,35342232	0	0	1,532164511	2,670856326	RPS17;ARL2BP;CLP1;RPS20;RPSA;TOMM20;TPT1
Carmustine-4_mg/kg_in_Corn_Oil-Rat-Liver-1d-up	7/314	0,1749611	0,353331699	0	0	1,532164511	2,670856326	C1QA;RPL32;RPS19;RPS20;RPSA;RPL10A;TPT1
Cloxacillin-1200_mg/kg_in_Water-Rat-Kidney-3d-up	7/314	0,1749611	0,353241124	0	0	1,532164511	2,670856326	RPS17;ARL2BP;CLP1;RPS20;RPS3A;SEPP1;RPL39
Cytarabine-23_mg/kg_in_Saline-Rat-Spleen-1d-up	7/314	0,1749611	0,353150596	0	0	1,532164511	2,670856326	RPS19;RPS6;RPS20;RPSA;RPL10A;SEPP1;RPL39
Deferiprone-500_mg/kg_in_Water-Rat-Liver-3d-dn	7/314	0,1749611	0,353060114	0	0	1,532164511	2,670856326	EEF1B2;RPL32;CLP1;RPS8;RPS6;RPS3A;RPL39
Fluconazole-10_mg/kg_in_Corn_Oil-Rat-Liver-1d-up	7/314	0,1749611	0,352969679	0	0	1,532164511	2,670856326	EEF1B2;RPS17;RPL32;RPS19;RPS8;RPS6;RPL10A
Melatonin-2000_mg/kg_in_Corn_Oil-Rat-Liver-5d-dn	7/314	0,1749611	0,35287929	0	0	1,532164511	2,670856326	EEF1B2;C1QA;RPS17;RPS8;RPS6;RPS3A;RPL39
NN'-Diphenyl-P-Phenylenediamine-1000_mg/kg_in_CMC-Rat-Liver-3d-up	7/314	0,1749611	0,352788947	0	0	1,532164511	2,670856326	RPS19;RPS6;RPS20;RPS3A;RPL10A;SEPP1;RPL39
Paroxetine-104_mg/kg_in_Water-Rat-Heart-5d-dn	7/314	0,1749611	0,35269865	0	0	1,532164511	2,670856326	RPS17;RPL32;RPS19;ARL2BP;RPL35A;RPS20;RPS3A
Vinblastine-0.3_mg/kg_in_Saline-Rat-Liver-1d-up	7/314	0,1749611	0,3526084	0	0	1,532164511	2,670856326	EEF1B2;C1QA;RPS6;RPL10A;SEPP1;RPL39;TPT1
Cobalt(II)_Acetylacetonate-100_mg/kg_in_Corn_Oil-Rat-Heart-1d-dn	6/258	0,175149376	0,352897541	0	0	1,598337729	2,784489875	RPS17;RPL32;RPS6;RPS20;RAPGEF5;TPT1
2-Acetylaminofluorene-30_mg/kg_in_CMC-Rat-Kidney-5d-dn	7/315	0,176882521	0,356298398	0	0	1,527300496	2,645696049	ARL2BP;RPS6;RPS3A;RPL10A;SEPP1;RPL39;TPT1
Carbon_Tetrachloride-1175_mg/kg_in_Corn_Oil-Rat-Bone_marrow-1d-up	7/315	0,176882521	0,356207297	0	0	1,527300496	2,645696049	C1QA;RPS17;RPS8;RPS5;RPS20;RPL39;TPT1
Carvedilol-2000_mg/kg_in_Corn_Oil-Rat-Liver-1d-up	7/315	0,176882521	0,356116242	0	0	1,527300496	2,645696049	EEF1B2;RPS6;RPS20;RPL10A;SEPP1;RPL39;TPT1
Chlorambucil-0.6_mg/kg_in_Corn_Oil-Rat-Spleen-1d-up	7/315	0,176882521	0,356025233	0	0	1,527300496	2,645696049	C1QA;RPL32;NKG7;RPS3A;SEPP1;RPL39;TPT1
Chlorobenzene-500_mg/kg_in_CMC-Rat-Kidney-1d-up	7/315	0,176882521	0,355934271	0	0	1,527300496	2,645696049	C1QA;RPS17;CXCL11;RPS8;RPS3A;SEPP1;TPT1
Ciprofloxacin-72_mg/kg_in_Corn_Oil-Rat-Liver-1d-up	7/315	0,176882521	0,355843356	0	0	1,527300496	2,645696049	C1QA;RPS19;RPL35A;RPS20;RPSA;RPL39;GOLGA7
Clofibrate-130_mg/kg_in_Corn_Oil-Rat-Liver-1d-up	7/315	0,176882521	0,355752487	0	0	1,527300496	2,645696049	RPS17;RPL32;RPS19;RPS8;FEM1B;RPS20;TPT1
Cyclosporin_A-350_mg/kg_in_Corn_Oil-Rat-Bone_marrow-5d-up	7/315	0,176882521	0,355661664	0	0	1,527300496	2,645696049	EEF1B2;C1QA;NKG7;RPSA;RPL10A;SEPP1;RPL39
Omeprazole-400_uM_in_DMSO-Rat-Primary_rat_hepatocytes-0.67d-dn	7/315	0,176882521	0,355570888	0	0	1,527300496	2,645696049	CXCL11;RPL32;RPS19;RPS8;SEPP1;SLC16A3;TPT1
Rofecoxib-3_mg/kg_in_Corn_Oil-Rat-Liver-5d-dn	7/315	0,176882521	0,355480158	0	0	1,527300496	2,645696049	RPS17;RPL32;RPS8;RPS6;RPS3A;RPL10A;SEPP1
Sulindac-64_mg/kg_in_Corn_Oil-Rat-Liver-0.25d-up	7/315	0,176882521	0,355389474	0	0	1,527300496	2,645696049	EEF1B2;RPL32;RPS19;RPS6;RPS20;RPL10A;SEPP1
Sulpiride-1000_mg/kg_in_CMC-Rat-Heart-1d-up	7/315	0,176882521	0,355298836	0	0	1,527300496	2,645696049	RPS17;RPL32;RPS6;RPS3A;RAPGEF5;RPL39;TPT1
Thioguanine-12_mg/kg_in_Corn_Oil-Rat-Bone_marrow-0.25d-up	7/315	0,176882521	0,355208245	0	0	1,527300496	2,645696049	EEF1B2;C1QA;RPL35A;RPS20;RPL10A;SEPP1;RPL39
Tramadol-114_mg/kg_in_Water-Rat-Brain-5d-dn	7/315	0,176882521	0,3551177	0	0	1,527300496	2,645696049	RPS17;RPL32;RPS8;RPS3A;RAPGEF5;TOMM20;TPT1
Trovafloxacin-600_mg/kg_in_CMC-Rat-Liver-3d-dn	7/315	0,176882521	0,355027201	0	0	1,527300496	2,645696049	C1QA;RPS17;RPL32;RPS19;RPL35A;RPSA;SEPP1
Rofecoxib-800_mg/kg_in_Corn_Oil-Rat-Liver-3d-up	6/259	0,177284293	0,355742954	0	0	1,592166541	2,754449164	C1QA;RPS17;CLP1;RPS3A;GOLGA7;SEPP1
3-Acetamidophenol-700_mg/kg_in_Corn_Oil-Rat-Liver-1d-up	8/373	0,178390752	0,357872023	0	0	1,474070184	2,540971081	C1QA;RPS6;RPS20;RPSA;RPS3A;SEPP1;RPL39;TPT1
Dipyrone-1636_mg/kg_in_Water-Rat-Liver-3d-up	7/316	0,178812992	0,358627738	0	0	1,522467267	2,620797616	RPL32;RPS19;RPS6;RPS3A;RPL10A;SEPP1;RPL39
Fluocinolone_Acetonide-2.5_mg/kg_in_Corn_Oil-Rat-Kidney-1d-up	7/316	0,178812992	0,358536438	0	0	1,522467267	2,620797616	RPS17;CLP1;RPS8;RPS20;RPS3A;SEPP1;TPT1
Gemfibrozil-100_mg/kg_in_Corn_Oil-Rat-Liver-3d-up	7/316	0,178812992	0,358445184	0	0	1,522467267	2,620797616	EEF1B2;RPL32;RPS19;RPS8;RPS6;RPS20;RPSA
Ipriflavone-1500_mg/kg_in_CMC-Rat-Liver-5d-up	7/316	0,178812992	0,358353977	0	0	1,522467267	2,620797616	RPS19;RPSA;RAPGEF5;RPL10A;SEPP1;RPL39;TPT1
Ketoconazole-90_uM_in_DMSO-Rat-Primary_rat_hepatocytes-1d-dn	7/316	0,178812992	0,358262816	0	0	1,522467267	2,620797616	RPS19;RPS6;ARL6IP5;SEPP1;RPL39;SLC16A3;TPT1
Phenacemide-400_mg/kg_in_CMC-Rat-Kidney-3d-up	7/316	0,178812992	0,358171701	0	0	1,522467267	2,620797616	RPS17;RPL32;RPS6;RPSA;RPS3A;RPL10A;SEPP1
Roxithromycin-312_mg/kg_in_CMC-Rat-Liver-5d-dn	7/316	0,178812992	0,358080633	0	0	1,522467267	2,620797616	EEF1B2;RPL32;RPS19;RPS8;RPS6;RPS20;SEPP1
Sulfadiazine-1170_mg/kg_in_Water-Rat-Kidney-5d-up	7/316	0,178812992	0,357989611	0	0	1,522467267	2,620797616	EEF1B2;ARL2BP;CLP1;RPS8;FUNDC1;GOLGA7;TPT1
Ibufenac-500_mg/kg_in_Corn_Oil-Rat-Liver-1d-up	6/260	0,179429808	0,359133206	0	0	1,586042823	2,724775879	RPS8;RPS6;RPS3A;GOLGA7;SEPP1;TPT1
Allyl_Alcohol-25_mg/kg_in_Saline-Rat-Bone_marrow-3d-up	8/374	0,180165174	0,360513442	0	0	1,47012882	2,519626173	EEF1B2;C1QA;RPS17;NKG7;RPL35A;RPS3A;RPL39;TPT1
Cisplatin-1.17_mg/kg_in_Saline-Rat-Kidney-3d-up	8/374	0,180165174	0,360421871	0	0	1,47012882	2,519626173	RPS17;RPS19;ARL2BP;RPS8;R3HCC1;RPS20;RPSA;TPT1
Alfacalcidol-0.043_mg/kg_in_CMC-Rat-Liver-5d-dn	7/317	0,180752436	0,361504871	0	0	1,517664531	2,596157837	RPS17;RPL32;RPS19;RPS8;RPS20;RPL10A;SEPP1
Amlodipine-0.2_mg/kg_in_Corn_Oil-Rat-Liver-0.25d-dn	7/317	0,180752436	0,361413096	0	0	1,517664531	2,596157837	C1QA;CLP1;RPS8;RPS5;RPS6;RPL35A;RPSA
Bisphenol_A-610_mg/kg_in_Corn_Oil-Rat-Liver-3d-up	7/317	0,180752436	0,361321366	0	0	1,517664531	2,596157837	RPS17;RPS20;RPSA;RPS3A;SEPP1;RPL39;TPT1
Clofibrate-500_mg/kg_in_Corn_Oil-Rat-Skeletal_muscle-3d-up	7/317	0,180752436	0,361229684	0	0	1,517664531	2,596157837	RPS19;ARL2BP;RPS8;RPS6;RPS20;RPS3A;PDRG1
Clofibric_Acid-448_mg/kg_in_Corn_Oil-Rat-Heart-3d-up	7/317	0,180752436	0,361138048	0	0	1,517664531	2,596157837	RPS19;RPSA;RPS3A;RPL10A;RPL39;TPT1;PDRG1
Cortisone-206_mg/kg_in_CMC-Rat-Heart-5d-up	7/317	0,180752436	0,361046458	0	0	1,517664531	2,596157837	EEF1B2;RPL32;RPS19;ARL2BP;RPS8;FUNDC1;RPS20
Fluoxetine-52_mg/kg_in_CMC-Rat-Liver-3d-up	7/317	0,180752436	0,360954915	0	0	1,517664531	2,596157837	RPS17;RPL32;RPS19;RPS8;RPS6;RPS20;RPS3A
Leflunomide-30_mg/kg_in_Corn_Oil-Rat-Kidney-3d-dn	7/317	0,180752436	0,360863418	0	0	1,517664531	2,596157837	EEF1B2;RPS19;RPS6;RPSA;RPL10A;SEPP1;RPL39
Leflunomide-60_mg/kg_in_Corn_Oil-Rat-Spleen-1d-up	7/317	0,180752436	0,360771967	0	0	1,517664531	2,596157837	C1QA;CXCL11;CLP1;FUNDC1;RPS3A;SEPP1;TPT1
Mifepristone-300_mg/kg_in_Corn_Oil-Rat-Liver-3d-dn	7/317	0,180752436	0,360680563	0	0	1,517664531	2,596157837	C1QA;RPS17;RPL32;RPS8;RPL35A;SEPP1;RPL39
Neostigmine_Bromide-40_mg/kg_in_Water-Rat-Liver-3d-dn	7/317	0,180752436	0,360589206	0	0	1,517664531	2,596157837	EEF1B2;RPS19;CLP1;RPS20;RPL10A;SEPP1;RPL39
Triacetin-1200_mg/kg_in_CMC-Rat-Liver-5d-up	7/317	0,180752436	0,360497894	0	0	1,517664531	2,596157837	RPS17;RPS8;RPS5;RPS20;RPSA;RPL39;TPT1
Didanosine-500_uM_in_DMSO-Rat-Primary_rat_hepatocytes-1d-dn	6/261	0,181585807	0,362068308	0	0	1,579966031	2,695464659	CXCL11;RPS8;ARL6IP5;RPS20;RPS3A;SEPP1
Furan-24_mg/kg_in_Corn_Oil-Rat-Kidney-3d-up	6/261	0,181585807	0,361976669	0	0	1,579966031	2,695464659	RPS17;RPS19;RPS8;RPS6;RPS3A;TPT1
Trovafloxacin-600_mg/kg_in_CMC-Rat-Liver-5d-up	6/261	0,181585807	0,361885075	0	0	1,579966031	2,695464659	EEF1B2;RPS6;RPS20;RPS3A;RPL39;SEPP1
33'4'5-Tetrachlorosalicylanilide-50_mg/kg_in_Corn_Oil-Rat-Liver-3d-up	7/318	0,182700772	0,364014997	0	0	1,512892001	2,571773567	RPL32;RPS19;RPS8;RPS6;RPS3A;SEPP1;RPL39
Carvedilol-50_uM_in_DMSO-Rat-Primary_rat_hepatocytes-1d-dn	7/318	0,182700772	0,363922935	0	0	1,512892001	2,571773567	C1QA;RPS19;ARL6IP5;SEPP1;RPL39;SLC16A3;TPT1
Colistin-121_mg/kg_in_Water-Rat-Liver-1d-up	7/318	0,182700772	0,363830919	0	0	1,512892001	2,571773567	C1QA;RPS17;RPL32;RPS6;ARL6IP5;RPSA;RPS3A
Fluoxetine-52_mg/kg_in_CMC-Rat-Heart-5d-up	7/318	0,182700772	0,363738949	0	0	1,512892001	2,571773567	RPS8;RPS6;ARL6IP5;RPSA;RPL10A;RPL39;TPT1
Noscapine-570_mg/kg_in_CMC-Rat-Bone_marrow-1d-up	7/318	0,182700772	0,363647026	0	0	1,512892001	2,571773567	C1QA;ARL6IP5;NKG7;RPSA;RPL10A;SEPP1;TPT1

Roflumilast-17_mg/kg_in_CMC-Rat-Heart-1d-up	7/318	0,182700772	0,36355515	0	0	1,512892001	2,571773567	EEF1B2;ARL2BP;CLP1;RPS8;FUNDC1;ARL6IP5;RPS3A
SCH-351591-4.6_mg/kg_in_CMC-Rat-Heart-1d-up	7/318	0,182700772	0,36346332	0	0	1,512892001	2,571773567	EEF1B2;RPS17;RPS8;ARL6IP5;RPL35A;RPSA;RPS3A
Thioacetamide-200_mg/kg_in_Saline-Rat-Liver-5d-up	7/318	0,182700772	0,363371536	0	0	1,512892001	2,571773567	EEF1B2;RPS19;RPS8;RPSA;SEPP1;GOLGA7;TPT1
Tolazamide-1500_mg/kg_in_CMC-Rat-Heart-1d-dn	7/318	0,182700772	0,363279799	0	0	1,512892001	2,571773567	RPS19;COL5A3;RPS6;RPS3A;SEPP1;RPL39;TPT1
Valproic_Acid-235_mg/kg_in_Water-Rat-Liver-1d-dn	7/318	0,182700772	0,363188108	0	0	1,512892001	2,571773567	C1QA;RPS17;RPL32;CLP1;RPS8;RPSA;SEPP1
Busulfan-9_mg/kg_in_Corn_Oil-Rat-Spleen-1d-dn	6/262	0,183752179	0,365186011	0	0	1,573935626	2,666510241	RPS17;RPS19;RPS5;RPL13A;RPL35A;TOMM20
Spironolactone-300_mg/kg_in_CMC-Rat-Kidney-1d-up	6/262	0,183752179	0,365093886	0	0	1,573935626	2,666510241	C1QA;RPS6;RPS3A;RPL10A;RPL39;TPT1
Triamterene-200_mg/kg_in_CMC-Rat-Kidney-5d-dn	6/262	0,183752179	0,365001807	0	0	1,573935626	2,666510241	RPS17;RPS6;RPS3A;RPL10A;RPL39;SEPP1
Acetazolamide-250_mg/kg_in_Corn_Oil-Rat-Kidney-1d-up	7/319	0,184657923	0,366708473	0	0	1,508149393	2,547641709	EEF1B2;RPL32;RPS19;RPS5;ARL6IP5;RPSA;RPL39
Atropine-94_mg/kg_in_Water-Rat-Liver-1d-up	7/319	0,184657923	0,366616033	0	0	1,508149393	2,547641709	EEF1B2;RPS17;RPL32;RPS6;RPSA;RPS3A;RPL39
Doxycycline-1000_mg/kg_in_Water-Rat-Liver-1d-dn	7/319	0,184657923	0,36652364	0	0	1,508149393	2,547641709	EEF1B2;RPL32;RPS19;RPS8;RPL10A;SEPP1;TPT1
Lincomycin-1200_mg/kg_in_CMC-Rat-Kidney-1d-up	7/319	0,184657923	0,366431293	0	0	1,508149393	2,547641709	RPS17;RPL32;RPS19;RPS6;ARL6IP5;RPS20;RPL39
Pemoline-70_mg/kg_in_CMC-Rat-Liver-5d-up	7/319	0,184657923	0,366338993	0	0	1,508149393	2,547641709	RPS17;CXADR;RPS19;RPS6;RPS20;SEPP1;RPL39
Phenacetin-619_mg/kg_in_Corn_Oil-Rat-Kidney-3d-dn	7/319	0,184657923	0,366246739	0	0	1,508149393	2,547641709	RPL32;RPS19;RPS6;RPS20;RPS3A;SEPP1;RPL39
Valproic_Acid-235_mg/kg_in_Water-Rat-Liver-5d-up	7/319	0,184657923	0,366154532	0	0	1,508149393	2,547641709	EEF1B2;RPS17;RPL32;COL5A3;SEPP1;RPL39;TPT1
Beta-Estradiol-150_mg/kg_in_Corn_Oil-Rat-Skeletal_muscle-5d-up	6/263	0,185928809	0,368581752	0	0	1,56795108	2,637907451	RPS19;CLP1;RPS8;RPS6;RPS20;TPT1
Lead(IV)_Acetate-200_mg/kg_in_Saline-Rat-Bone_marrow-1d-dn	6/263	0,185928809	0,368489004	0	0	1,56795108	2,637907451	EEF1B2;RPL32;NKG7;RPS3A;RPL10A;SEPP1
Rabeprazole-333_uM_in_DMSO-Rat-Primary_rat_hepatocytes-1d-up	6/263	0,185928809	0,368396302	0	0	1,56795108	2,637907451	RPS17;RPS6;RPSA;RPS3A;RPL10A;PDRG1
Vinblastine-0.08_mg/kg_in_Saline-Rat-Bone_marrow-1d-dn	6/263	0,185928809	0,368303647	0	0	1,56795108	2,637907451	EEF1B2;CLP1;RPS8;NKG7;RPS20;RPSA
3-Methylcholanthrene-300_mg/kg_in_CMC-Rat-Liver-5d-dn	7/320	0,186623808	0,369587405	0	0	1,503436426	2,523759211	EEF1B2;RPS19;RPS8;RPS6;RPL10A;RPL39;TPT1
Aflatoxin_B1-0.3_mg/kg_in_CMC-Rat-Liver-3d-up	7/320	0,186623808	0,369494497	0	0	1,503436426	2,523759211	EEF1B2;RPS17;RPL32;RPS19;RPS6;RPL39;TPT1
Angiotensin_II_Human-0.72_mg/kg_in_Saline-Rat-Heart-5d-up	7/320	0,186623808	0,369401636	0	0	1,503436426	2,523759211	EEF1B2;RPS19;FUNDC1;ARL6IP5;RPS20;RPSA;RPL39
Carmustine-16_mg/kg_in_Corn_Oil-Rat-Liver-5d-up	7/320	0,186623808	0,369308821	0	0	1,503436426	2,523759211	CXADR;RPS6;RPS20;RPL10A;SEPP1;RPL39;TPT1
Econazole-43_mg/kg_in_Corn_Oil-Rat-Liver-3d-dn	7/320	0,186623808	0,369216054	0	0	1,503436426	2,523759211	RPS17;RPS19;RPS8;RPS6;RPSA;RPS3A;TPT1
Ibuprofen-90_mg/kg_in_Corn_Oil-Rat-Liver-1d-dn	7/320	0,186623808	0,369123332	0	0	1,503436426	2,523759211	RPS19;CLP1;RPS20;RPSA;RPS3A;RPL10A;GOLGA7
Maprotiline-380_mg/kg_in_CMC-Rat-Heart-3d-dn	7/320	0,186623808	0,369030658	0	0	1,503436426	2,523759211	EEF1B2;RPS19;ARL2BP;COL5A3;RPS6;ARL6IP5;RPL39
Salicylamide-1300_mg/kg_in_CMC-Rat-Liver-5d-dn	7/320	0,186623808	0,368938029	0	0	1,503436426	2,523759211	EEF1B2;RPS17;RPL32;RPL35A;RPSA;RPL10A;SEPP1
Sulconazole-40_uM_in_DMSO-Rat-Primary_rat_hepatocytes-1d-dn	7/320	0,186623808	0,368845448	0	0	1,503436426	2,523759211	CXCL11;RPS19;ARL6IP5;SEPP1;RPL39;SLC16A3;TPT1
Ticlopidine-223_mg/kg_in_CMC-Rat-Liver-5d-dn	7/320	0,186623808	0,368752913	0	0	1,503436426	2,523759211	EEF1B2;C1QA;RPS17;RPL32;RPS8;RPS3A;TPT1
Leflunomide-30_mg/kg_in_Corn_Oil-Rat-Kidney-1d-dn	8/378	0,187339975	0,370075155	0	0	1,454571901	2,436161043	EEF1B2;RPS19;CLP1;RPS6;RPSA;RPL10A;SEPP1;RPL39
Fluphenazine-2.5_mg/kg_in_Corn_Oil-Rat-Liver-0.25d-dn	6/264	0,188115583	0,371514125	0	0	1,562011871	2,609651209	C1QA;RPS17;RPS8;RPL35A;RPS20;RPL39
Sildenafil-2.5_mg/kg_in_Water-Rat-Liver-0.25d-dn	6/264	0,188115583	0,37142099	0	0	1,562011871	2,609651209	RPL32;RPS8;RPS5;RPL35A;RPS3A;TPT1
44'-Diethylaminoethoxyhexestrol-20_uM_in_DMSO-Rat-Primary_rat_hepatocytes-1d-dn	7/321	0,188598345	0,372280844	0	0	1,498752824	2,500123063	CXCL11;RPS19;ARL6IP5;SEPP1;RPL39;SLC16A3;TPT1
Clarithromycin-56_mg/kg_in_Water-Rat-Liver-5d-up	7/321	0,188598345	0,372187564	0	0	1,498752824	2,500123063	EEF1B2;RPS17;RPL32;RPS5;RPL35A;RPS20;RPL10A
Griseofulvin-2500_mg/kg_in_Corn_Oil-Rat-Liver-3d-up	7/321	0,188598345	0,372094331	0	0	1,498752824	2,500123063	RPS19;FEM1B;RPS20;RPSA;RPS3A;SEPP1;RPL39
Miconazole-920_mg/kg_in_Corn_Oil-Rat-Liver-1d-dn	7/321	0,188598345	0,372001144	0	0	1,498752824	2,500123063	EEF1B2;C1QA;RPS6;RPSA;RPL10A;SEPP1;TPT1
Neomycin-56_mg/kg_in_Corn_Oil-Rat-Liver-5d-dn	7/321	0,188598345	0,371908004	0	0	1,498752824	2,500123063	EEF1B2;RPS17;RPS6;RPSA;RPS3A;RPL10A;SEPP1
Oxcarbazepine-1600_mg/kg_in_CMC-Rat-Brain-1d-dn	7/321	0,188598345	0,371814911	0	0	1,498752824	2,500123063	EEF1B2;CXADR;FUNDC1;RPS6;RPSA;SEPP1;TPT1
Tropisetron-100_mg/kg_in_CMC-Rat-Liver-3d-up	7/321	0,188598345	0,371721864	0	0	1,498752824	2,500123063	C1QA;RPS19;RPS6;RPS3A;RPL10A;RPL39;PDRG1
Hydroxyurea-400_mg/kg_in_Saline-Rat-Liver-1d-up	8/379	0,189152658	0,372721124	0	0	1,450733981	2,415763497	EEF1B2;RPL32;RPS19;COL5A3;RPS20;RPL10A;RPL39;TPT1
Sildenafil-420_mg/kg_in_Water-Rat-Liver-0.25d-up	8/379	0,189152658	0,372627897	0	0	1,450733981	2,415763497	EEF1B2;C1QA;RPS19;RPS6;RPS20;RPL10A;SEPP1;TPT1
Clozapine-69_uM_in_DMSO-Rat-Primary_rat_hepatocytes-1d-up	6/265	0,190312384	0,374818788	0	0	1,556117487	2,58173652	EEF1B2;RPS17;RPS5;RPS6;RPS3A;PDRG1
Digoxin-11_mg/kg_in_Corn_Oil-Rat-Heart-5d-up	6/265	0,190312384	0,374725084	0	0	1,556117487	2,58173652	RPS17;RPS19;ARL2BP;RPS20;RPS3A;TPT1
Ethylene_Glycol-3525_mg/kg_in_Water-Rat-Kidney-3d-dn	6/265	0,190312384	0,374631426	0	0	1,556117487	2,58173652	RPS17;ARL2BP;RPS8;ARL6IP5;RPSA;TPT1
Irinotecan-5_mg/kg_in_Saline-Rat-Bone_marrow-1d-up	6/265	0,190312384	0,374537815	0	0	1,556117487	2,58173652	C1QA;RPS19;NKG7;RPL10A;GOLGA7;SEPP1
Neostigmine_Bromide-11_mg/kg_in_Water-Rat-Liver-0.25d-dn	6/265	0,190312384	0,374444251	0	0	1,556117487	2,58173652	EEF1B2;RPS17;RPL32;RPL35A;RPS3A;RPL39
Bisacodyl-1750_mg/kg_in_CMC-Rat-Intestine-1d-up	7/322	0,190581455	0,374880005	0	0	1,494098312	2,476730302	RPS17;RPL32;RPS5;RPL35A;RPL10A;SLC16A3;TPT1
Chlorambucil-0.6_mg/kg_in_Corn_Oil-Rat-Bone_marrow-1d-up	7/322	0,190581455	0,374786402	0	0	1,494098312	2,476730302	C1QA;RPS17;RPS19;RPS20;RPL10A;SEPP1;RPL39
Chloroquine-21.95_uM_in_DMSO-Rat-Primary_rat_hepatocytes-1d-dn	7/322	0,190581455	0,374692845	0	0	1,494098312	2,476730302	RPS19;ARL2BP;RPS8;RPS20;RPS3A;RPL39;TPT1
Sulindac-23_mg/kg_in_Corn_Oil-Rat-Kidney-5d-dn	7/322	0,190581455	0,374599336	0	0	1,494098312	2,476730302	C1QA;RPS17;RPL32;RPS20;RAPGEF5;RPL39;GOLGA7
Iproniazid-46_mg/kg_in_Water-Rat-Heart-5d-up	6/266	0,192519096	0,378313474	0	0	1,550267421	2,554158481	EEF1B2;RPS17;CLP1;RPS8;RPS3A;TPT1
Stannous_Fluoride-90_mg/kg_in_Water-Rat-Kidney-5d-dn	6/266	0,192519096	0,378219108	0	0	1,550267421	2,554158481	RPS19;RPS8;FEM1B;RPS20;RPSA;SEPP1
Valproic_Acid-235_mg/kg_in_Water-Rat-Bone_marrow-3d-dn	6/266	0,192519096	0,378124789	0	0	1,550267421	2,554158481	ARL2BP;CLP1;RPS8;FUNDC1;RPS6;TPT1
Acyclovir-980_mg/kg_in_Saline-Rat-Spleen-1d-up	7/323	0,192573054	0,378136468	0	0	1,48947262	2,453578006	C1QA;RPS19;RPS6;NKG7;RPL10A;SEPP1;RPL39
Dipyrone-1636_mg/kg_in_Water-Rat-Liver-5d-dn	7/323	0,192573054	0,378042216	0	0	1,48947262	2,453578006	EEF1B2;C1QA;RPS17;CLP1;RPL35A;SEPP1;TPT1
Etodolac-24_mg/kg_in_CMC-Rat-Liver-3d-up	7/323	0,192573054	0,377948012	0	0	1,48947262	2,453578006	C1QA;RPS19;ARL2BP;RPSA;RPS3A;RPL10A;RPL39
Lead(IV)_Acetate-600_mg/kg_in_Saline-Rat-Spleen-5d-up	7/323	0,192573054	0,377853855	0	0	1,48947262	2,453578006	C1QA;RPL32;NKG7;RPL10A;SEPP1;RPL39;TPT1
NN-Dimethylformamide-1400_mg/kg_in_Saline-Rat-Bone_marrow-5d-dn	7/323	0,192573054	0,377759744	0	0	1,48947262	2,453578006	RPS19;FEM1B;RPS20;RPSA;RPL10A;RPL39;DUSP14
Omeprazole-30_mg/kg_in_Corn_Oil-Rat-Liver-3d-up	7/323	0,192573054	0,37766568	0	0	1,48947262	2,453578006	RPS19;RPS8;RPS20;RPS3A;SEPP1;GOLGA7;TPT1
Simvastatin-15_mg/kg_in_Corn_Oil-Rat-Kidney-0.25d-up	7/323	0,192573054	0,377571664	0	0	1,48947262	2,453578006	RPS17;RPS8;FUNDC1;RPL35A;RPS20;RPS3A;TPT1
Sulfisoxazole-2500_mg/kg_in_Corn_Oil-Rat-Liver-1d-dn	7/323	0,192573054	0,377477694	0	0	1,48947262	2,453578006	EEF1B2;RPS17;RPL32;RPS20;RPSA;RPL10A;TPT1
Sodium_Selenite-2_mg/kg_in_Water-Rat-Kidney-5d-up	8/381	0,192800381	0,377829261	0	0	1,443118579	2,375517381	C1QA;RPS19;RPS8;RPS3A;RPL39;GOLGA7;TOMM20;TPT1
Allyl_Alcohol-32_mg/kg_in_Saline-Rat-Liver-5d-up	7/324	0,19457306	0,381208313	0	0	1,484875483	2,430663294	RPS19;RPS8;RPS3A;RPL10A;RPL39;GOLGA7;TPT1
Chlorpromazine-18_mg/kg_in_Water-Rat-Liver-1d-dn	7/324	0,19457306	0,381113509	0	0	1,484875483	2,430663294	RPS17;RPS19;RPS8;RPS6;RPS20;RPSA;RPL10A
Cholecalciferol-8_mg/kg_in_CMC-Rat-Kidney-3d-up	7/324	0,19457306	0,381018752	0	0	1,484875483	2,430663294	EEF1B2;RPS17;CXCL11;RPS19;RPSA;RPL10A;TPT1
Marimastat-1000_uM_in_DMSO-Rat-Primary_rat_hepatocytes-0.67d-dn	7/324	0,19457306	0,380924042	0	0	1,484875483	2,430663294	EEF1B2;CXCL11;RPL32;RPS19;ARL2BP;RPS8;RPS20
Ofloxacin-3000_mg/kg_in_Water-Rat-Kidney-3d-up	7/324	0,19457306	0,380829379	0	0	1,484875483	2,430663294	EEF1B2;CXCL11;ARL2BP;CLP1;RPS20;RAPGEF5;SEPP1
S(-)-Carbidopa-1200_mg/kg_in_CMC-Rat-Brain-5d-up	7/324	0,19457306	0,380734763	0	0	1,484875483	2,430663294	FUNDC1;RPS6;RPSA;RPS3A;RPL10A;RPL39;GOLGA7
Thalidomide-113_mg/kg_in_CMC-Rat-Kidney-3d-dn	7/324	0,19457306	0,380640194	0	0	1,484875483	2,430663294	RPL32;RPS6;RPS20;RPL10A;SEPP1;RPL39;TPT1
Thioguanine-24_mg/kg_in_Corn_Oil-Rat-Liver-0.25d-up	7/324	0,19457306	0,380545672	0	0	1,484875483	2,430663294	RPS19;RPS8;RPS6;RPS3A;RPL10A;RPL39;TPT1
Losartan-1000_mg/kg_in_Water-Rat-Kidney-5d-up	8/382	0,194635298	0,380572892	0	0	1,439340782	2,355665047	EEF1B2;ARL2BP;RPS8;RPS6;RPS3A;RAPGEF5;GOLGA7;TPT1
N-Nitrosodiethylamine-1.67_mg/kg_in_Saline-Rat-Liver-5d-dn	6/267	0,194735603	0,380674512	0	0	1,544461176	2,52691227	EEF1B2;RPL32;RPS8;RPS5;RPSA;TPT1
Naproxen-10_mg/kg_in_Corn_Oil-Rat-Kidney-0.25d-dn	6/267	0,194735603	0,380580052	0	0	1,544461176	2,52691227	RPS19;ARL2BP;RPS8;RPS6;RPSA;RPL39
Lead_(II)_Acetate-200_mg/kg_in_Saline-Rat-Bone_marrow-1d-up	8/383	0,196477504	0,383889065	0	0	1,435582712	2,335990724	RPS17;RPS8;RPS5;ARL6IP5;RPL35A;RPS20;RPSA;TPT1
Cinnarizine-750_mg/kg_in_CMC-Rat-Liver-5d-dn	7/325	0,196581389	0,383996781	0	0	1,480306635	2,407983329	EEF1B2;C1QA;RPS19;RPS6;RPSA;RPL10A;SEPP1
Cyclosporin_A-70_mg/kg_in_Corn_Oil-Rat-Spleen-1d-up	7/325	0,196581389	0,383901568	0	0	1,480306635	2,407983329	C1QA;RPS17;RPL32;RPS3A;RPL10A;SEPP1;TPT1
Etoposide-188_mg/kg_in_Corn_Oil-Rat-Heart-1d-up	7/325	0,196581389	0,383806401	0	0	1,480306635	2,407983329	ARL6IP5;RPSA;RAPGEF5;RPL10A;SEPP1;RPL39;PDRG1
Loratadine-10_mg/kg_in_Water-Rat-Heart-5d-up	7/325	0,196581389	0,383711282	0	0	1,480306635	2,407983329	RPS17;RPL32;RPS6;RPL35A;RPS3A;RPL39;TPT1
Nevirapine-250_uM_in_DMSO-Rat-Primary_rat_hepatocytes-0.67d-up	7/325	0,196581389	0,38361621	0	0	1,480306635	2,407983329	EEF1B2;RPS6;RPL10A;SEPP1;RPL39;GOLGA7;TPT1
Dichlorvos-17_mg/kg_in_Water-Rat-Liver-3d-dn	6/268	0,196961786	0,384263321	0	0	1,538698261	2,499993151	RPS17;RPS19;CLP1;FEM1B;RPS20;RPL39
Pantoprazole-1100_mg/kg_in_Water-Rat-Liver-5d-dn	6/268	0,196961786	0,384168159	0	0	1,538698261	2,499993151	EEF1B2;C1QA;CLP1;RPS20;RPL39;TPT1
Phenobarbital-54_mg/kg_in_Water-Rat-Liver-3d-dn	6/268	0,196961786	0,384073044	0	0	1,538698261	2,499993151	EEF1B2;RPS6;RPS20;RPL10A;RPL39;TPT1
123-Trichloropropane-108_mg/kg_in_CMC-Rat-Kidney-1d-dn	7/326	0,198597959	0,387167703	0	0	1,475765817	2,385535312	EEF1B2;RPL32;ARL2BP;RPS8;RPS6;RPS20;RPL39
Aminoglutethimide-350_mg/kg_in_CMC-Rat-Liver-0.25d-up	7/326	0,198597959	0,387071893	0	0	1,475765817	2,385535312	C1QA;RPS17;RPS19;RPS20;RPSA;RPL10A;RPL39
Doxapram-20_mg/kg_in_Saline-Rat-Heart-1d-up	7/326	0,198597959	0,386976131	0	0	1,475765817	2,385535312	RPS19;COL5A3;RPS6;RPSA;SEPP1;RPL39;TPT1
Fluocinolone_Acetonide-2.5_mg/kg_in_Corn_Oil-Rat-Kidney-3d-up	7/326	0,198597959	0,386880416	0	0	1,475765817	2,385535312	CXCL11;RPS8;RPS5;RPS20;RPS3A;SEPP1;TPT1
Rifampin-99_mg/kg_in_CMC-Rat-Kidney-3d-dn	7/326	0,198597959	0,386784748	0	0	1,475765817	2,385535312	RPS8;RPS6;RPS20;RPSA;SEPP1;RPL39;TPT1
Enoxacin-100_mg/kg_in_Corn_Oil-Rat-Liver-0.25d-up	6/269	0,199197527	0,387856544	0	0	1,532978193	2,473396467	EEF1B2;RPS19;RPS8;RPS6;RPS20;RPS3A
Epirubicin-2.7_mg/kg_in_Saline-Rat-Heart-3d-dn	6/269	0,199197527	0,387760682	0	0	1,532978193	2,473396467	EEF1B2;RPS17;RPS8;FEM1B;ARL6IP5;RPSA
Furan-24_mg/kg_in_Corn_Oil-Rat-Kidney-1d-up	6/269	0,199197527	0,387664868	0	0	1,532978193	2,473396467	C1QA;RPS19;ARL2BP;RPS8;RPSA;SEPP1

3-Acetamidophenol-700_mg/kg_in_Corn_Oil-Rat-Kidney-1d-dn	7/327	0,200622683	0,390341959	0	0	1,471252772	2,363316486	EEF1B2;RPS6;RPS20;RPS3A;SEPP1;RPL39;TPT1
Acyclovir-330_mg/kg_in_Saline-Rat-Bone_marrow-0.25d-up	7/327	0,200622683	0,390245554	0	0	1,471252772	2,363316486	C1QA;RPS17;RPS19;RPS5;RPL35A;RPSA;TPT1
Genistein-20_mg/kg_in_Corn_Oil-Rat-Liver-1d-up	7/327	0,200622683	0,390149197	0	0	1,471252772	2,363316486	C1QA;RPS19;RPS5;RPS6;RPS20;RPSA;GOLGA7
Meloxicam-33_mg/kg_in_Corn_Oil-Rat-Liver-3d-up	7/327	0,200622683	0,390052888	0	0	1,471252772	2,363316486	EEF1B2;C1QA;RPS19;RPS8;RPS20;RPS3A;SEPP1
Moxonidine-17_mg/kg_in_CMC-Rat-Heart-3d-dn	7/327	0,200622683	0,389956626	0	0	1,471252772	2,363316486	RPS8;COL5A3;ARL6IP5;RPSA;SEPP1;TOMM20;TPT1
Sulindac-23_mg/kg_in_Corn_Oil-Rat-Liver-0.25d-dn	7/327	0,200622683	0,389860412	0	0	1,471252772	2,363316486	RPL32;RPS19;RPS8;RPS5;RPL35A;RPSA;SEPP1
Vinblastine-0.3_mg/kg_in_Saline-Rat-Heart-3d-up	7/327	0,200622683	0,389764245	0	0	1,471252772	2,363316486	EEF1B2;RPL32;RPS8;RPS6;RPL35A;RPS20;RPSA
Altretamine-40_mg/kg_in_Corn_Oil-Rat-Liver-5d-dn	6/270	0,201442706	0,391260851	0	0	1,527300496	2,447117645	RPS19;CLP1;RPS6;RPSA;RPL10A;SEPP1
Aspirin-500_mg/kg_in_Corn_Oil-Rat-Liver-3d-up	6/270	0,201442706	0,391164386	0	0	1,527300496	2,447117645	C1QA;RPS17;CLP1;RPS3A;GOLGA7;SEPP1
Cromolyn-1500_mg/kg_in_Water-Rat-Kidney-5d-dn	6/270	0,201442706	0,391067969	0	0	1,527300496	2,447117645	RPS19;CLP1;RPS8;RPS20;RPS3A;TPT1
Diclofenac-10_mg/kg_in_Corn_Oil-Rat-Liver-0.25d-dn	6/270	0,201442706	0,390971599	0	0	1,527300496	2,447117645	EEF1B2;RPS17;RPS3A;RPL39;SEPP1;TPT1
Doxapram-20_mg/kg_in_Saline-Rat-Heart-5d-dn	6/270	0,201442706	0,390875277	0	0	1,527300496	2,447117645	RPS17;RPL32;ARL2BP;RPL35A;RPS3A;RAPGEF5
Hydroquinone-800_mg/kg_in_CMC-Rat-Kidney-1d-up	6/270	0,201442706	0,390779002	0	0	1,527300496	2,447117645	RPS17;RPL32;RPS8;RPSA;SEPP1;TPT1
Neomycin-56_mg/kg_in_Corn_Oil-Rat-Kidney-5d-dn	6/270	0,201442706	0,390682775	0	0	1,527300496	2,447117645	C1QA;RAPGEF5;RPL10A;RPL39;GOLGA7;SEPP1
Podophyllotoxin-4_mg/kg_in_Corn_Oil-Rat-Bone_marrow-1d-dn	6/270	0,201442706	0,390586595	0	0	1,527300496	2,447117645	RPS19;FEM1B;RPS20;RPSA;RPL10A;SEPP1
Sodium_Orthovanadate-83_mg/kg_in_CMC-Rat-Kidney-1d-up	6/270	0,201442706	0,390490462	0	0	1,527300496	2,447117645	RPS17;RPL32;CLP1;RPS8;RPS3A;TPT1
Testosterone-375_mg/kg_in_CMC-Rat-Liver-1d-dn	6/270	0,201442706	0,390394377	0	0	1,527300496	2,447117645	C1QA;RPS17;RPS19;RPS6;RPS20;RPL39
Amineptine-1667_uM_in_DMSO-Rat-Primary_rat_hepatocytes-1d-dn	7/328	0,202655477	0,392648103	0	0	1,466767245	2,341324133	RPS17;RPL32;RPS19;RPS20;RPL39;TOMM20;TPT1
Dexamethasone-1_mg/kg_in_Corn_Oil-Rat-Liver-5d-dn	7/328	0,202655477	0,392551534	0	0	1,466767245	2,341324133	C1QA;RPS17;RPL32;RPS8;RPS6;RPSA;SEPP1
Griseofulvin-2500_mg/kg_in_Corn_Oil-Rat-Liver-1d-up	7/328	0,202655477	0,392455013	0	0	1,466767245	2,341324133	RPS19;RPS8;RPS20;RPSA;RPL10A;SEPP1;TPT1
Staurosporine-1.3_uM_in_DMSO-Rat-Primary_rat_hepatocytes-1d-dn	7/328	0,202655477	0,392358539	0	0	1,466767245	2,341324133	RPS19;CLP1;RPS8;ARL6IP5;RPL39;SLC16A3;TPT1
Valproic_Acid-235_mg/kg_in_Water-Rat-Liver-0.25d-up	7/328	0,202655477	0,392262113	0	0	1,466767245	2,341324133	EEF1B2;RPS19;RPS8;RPS20;RPSA;RPS3A;GOLGA7
Cortisone-206_mg/kg_in_CMC-Rat-Heart-1d-dn	6/271	0,203697203	0,394181614	0	0	1,521664701	2,421152186	RPS17;COL5A3;RPS6;RPSA;RPS3A;TPT1
Mefenamic_Acid-93_mg/kg_in_Corn_Oil-Rat-Liver-3d-dn	6/271	0,203697203	0,394084787	0	0	1,521664701	2,421152186	RPS17;RPL32;RPS8;RPL35A;RPS3A;TPT1
Atorvastatin-300_mg/kg_in_Corn_Oil-Rat-Liver-0.25d-up	8/387	0,203917948	0,39441497	0	0	1,420744648	2,259037384	C1QA;RPS17;RPS19;RPS8;RPS6;RPSA;RPS3A;RPL10A
Carboplatin-6_mg/kg_in_Saline-Rat-Bone_marrow-0.25d-dn	7/329	0,204696256	0,395823155	0	0	1,462308986	2,319555572	EEF1B2;RPS17;RPS6;NKG7;RPS3A;RPL10A;RPL39
Genistein-20_mg/kg_in_Corn_Oil-Rat-Liver-3d-up	7/329	0,204696256	0,395725997	0	0	1,462308986	2,319555572	RPL32;RPS8;RPS6;RPL35A;RPS20;RPSA;SEPP1
Ibuprofen-54_mg/kg_in_Corn_Oil-Rat-Liver-5d-dn	7/329	0,204696256	0,395628886	0	0	1,462308986	2,319555572	RPS19;RPS6;RPSA;RPS3A;RPL10A;SEPP1;TPT1
Mefenamic_Acid-93_mg/kg_in_Corn_Oil-Rat-Liver-3d-up	7/329	0,204696256	0,395531823	0	0	1,462308986	2,319555572	RPS19;RPS20;RPSA;RAPGEF5;RPL10A;SEPP1;RPL39
Tamoxifen-64_mg/kg_in_Corn_Oil-Rat-Heart-3d-up	7/329	0,204696256	0,395434808	0	0	1,462308986	2,319555572	ARL2BP;RPS8;RAPGEF5;RPL10A;RPL39;TPT1;PDRG1
Dexchlorpheniramine-47_mg/kg_in_Water-Rat-Heart-5d-up	6/272	0,205960898	0,397780292	0	0	1,516070346	2,395495671	EEF1B2;RPS19;RPS6;RPSA;RPL10A;RPL39
Ebastine-2000_mg/kg_in_Water-Rat-Heart-5d-up	6/272	0,205960898	0,397682773	0	0	1,516070346	2,395495671	C1QA;RPL32;RPS6;RPS3A;RPL10A;SEPP1
Marimastat-1000_uM_in_DMSO-Rat-Primary_rat_hepatocytes-1d-up	6/272	0,205960898	0,397585302	0	0	1,516070346	2,395495671	RPS20;RPSA;RPS3A;SLC16A3;TPT1;PDRG1
Neomycin-877_mg/kg_in_Corn_Oil-Rat-Liver-1d-dn	6/272	0,205960898	0,397487878	0	0	1,516070346	2,395495671	EEF1B2;RPS17;RPS8;RPSA;RPL10A;SEPP1
Phenylhydrazine-78_mg/kg_in_Water-Rat-Liver-0.25d-dn	6/272	0,205960898	0,397390502	0	0	1,516070346	2,395495671	C1QA;RPS17;RPL35A;RPS3A;RPL39;TPT1
Altretamine-13_mg/kg_in_Corn_Oil-Rat-Liver-0.25d-up	7/330	0,206744933	0,398805557	0	0	1,457877747	2,298008161	RPS17;RPL32;RPS19;RPL35A;RPS3A;SEPP1;TPT1
CerIVastatin-7_mg/kg_in_Corn_Oil-Rat-Kidney-3d-dn	7/330	0,206744933	0,398707906	0	0	1,457877747	2,298008161	EEF1B2;RPS19;RPS8;RPS6;RPS20;RPS3A;RPL39
Emetine-1_mg/kg_in_Saline-Rat-Heart-5d-up	7/330	0,206744933	0,398610303	0	0	1,457877747	2,298008161	EEF1B2;ARL2BP;RPS8;RPL35A;RPS20;RAPGEF5;PDRG1
Etoposide-188_mg/kg_in_Corn_Oil-Rat-Heart-3d-up	7/330	0,206744933	0,398512748	0	0	1,457877747	2,298008161	CXCL11;FUNDC1;ARL6IP5;RAPGEF5;RPL10A;SEPP1;PDRG1
Fluphenazine-22_mg/kg_in_Corn_Oil-Rat-Liver-1d-up	7/330	0,206744933	0,398415241	0	0	1,457877747	2,298008161	EEF1B2;RPL32;RPS19;RPS8;RPS6;RPS20;RPL10A
Hydrochlorothiazide-1500_mg/kg_in_CMC-Rat-Kidney-3d-up	7/330	0,206744933	0,398317781	0	0	1,457877747	2,298008161	RPS17;RPL32;RPS19;RPL13A;RPL35A;RPS20;RPSA
Moxonidine-17_mg/kg_in_CMC-Rat-Heart-5d-dn	7/330	0,206744933	0,398220369	0	0	1,457877747	2,298008161	EEF1B2;RPS17;COL5A3;ARL6IP5;RPL35A;SEPP1;TPT1
Oxymetazoline-0.5_mg/kg_in_Water-Rat-Heart-3d-dn	7/330	0,206744933	0,398123005	0	0	1,457877747	2,298008161	RPL32;RPS8;FUNDC1;RPS6;RPS3A;SEPP1;TPT1
Cerivastatin-7_mg/kg_in_Corn_Oil-Rat-Liver-1d-up	6/273	0,208233669	0,400891806	0	0	1,510516974	2,370143755	C1QA;RPS17;RPS6;RPS3A;RPL39;GOLGA7
Clarithromycin-56_mg/kg_in_Water-Rat-Liver-1d-dn	6/273	0,208233669	0,400793837	0	0	1,510516974	2,370143755	RPS19;RPSA;RPL10A;RPL39;SEPP1;TPT1
Colistin-121_mg/kg_in_Water-Rat-Liver-5d-up	6/273	0,208233669	0,400695915	0	0	1,510516974	2,370143755	CLP1;RPS6;RPSA;RPS3A;RPL10A;SEPP1
Ethinylestradiol-190_uM_in_DMSO-Rat-Primary_rat_hepatocytes-1d-up	6/273	0,208233669	0,400598041	0	0	1,510516974	2,370143755	EEF1B2;RPS6;RPSA;RPS3A;RPL10A;PDRG1
Fluvastatin-5_mg/kg_in_Corn_Oil-Rat-Liver-1d-dn	6/273	0,208233669	0,400500215	0	0	1,510516974	2,370143755	RPL32;RPS6;RPS20;RPSA;RPS3A;RPL10A
Cyclosporin_A-350_mg/kg_in_Corn_Oil-Rat-Spleen-1d-up	7/331	0,208801421	0,401494138	0	0	1,453473282	2,276679297	C1QA;RPS17;CXCL11;RPL32;ARL6IP5;RPS3A;TPT1
Famciclovir-112_mg/kg_in_Saline-Rat-Spleen-1d-up	7/331	0,208801421	0,401396141	0	0	1,453473282	2,276679297	RPS19;NKG7;RPS20;RPSA;RPL10A;SEPP1;RPL39
Furan-24_mg/kg_in_Corn_Oil-Rat-Kidney-1d-dn	7/331	0,208801421	0,401298192	0	0	1,453473282	2,276679297	EEF1B2;RPS17;RPL32;RPS6;RPS3A;RPL39;TPT1
Etoposide-188_mg/kg_in_Corn_Oil-Rat-Liver-0.25d-dn	5/217	0,209906388	0,403323424	0	0	1,583606505	2,472158009	RPS17;RPL32;RPS19;RPS3A;RPL39
Azathioprine-20_mg/kg_in_Corn_Oil-Rat-Kidney-0.25d-dn	6/274	0,210515396	0,40439494	0	0	1,505004139	2,345092168	CLP1;RPS8;RPSA;RPL39;SEPP1;TPT1
BW-723C86-10_mg/kg_in_Saline-Rat-Heart-1d-dn	6/274	0,210515396	0,404296331	0	0	1,505004139	2,345092168	EEF1B2;RPS17;RPL32;ARL2BP;SEPP1;TPT1
Cytarabine-487_mg/kg_in_Saline-Rat-Spleen-5d-dn	6/274	0,210515396	0,404197771	0	0	1,505004139	2,345092168	C1QA;RPS6;RPS3A;RPL10A;RPL39;TPT1
Doxapram-20_mg/kg_in_Saline-Rat-Heart-1d-dn	6/274	0,210515396	0,404099258	0	0	1,505004139	2,345092168	EEF1B2;RPS17;RPL32;ARL2BP;RPS3A;RAPGEF5
Hydroxyurea-400_mg/kg_in_Saline-Rat-Spleen-5d-up	6/274	0,210515396	0,404000793	0	0	1,505004139	2,345092168	CXCL11;FUNDC1;RPS6;RPL39;SEPP1;TPT1
Melatonin-2000_mg/kg_in_Corn_Oil-Rat-Liver-3d-up	6/274	0,210515396	0,403902376	0	0	1,505004139	2,345092168	RPS19;RPS8;RPS6;RPSA;RPS3A;RPL10A
Rofecoxib-775_mg/kg_in_Corn_Oil-Rat-Liver-3d-dn	6/274	0,210515396	0,403804008	0	0	1,505004139	2,345092168	RPS17;RPS19;RPS6;RPL35A;RPL39;SEPP1
Chlorambucil-0.6_mg/kg_in_Corn_Oil-Rat-Liver-0.25d-up	7/332	0,210865633	0,404377338	0	0	1,44909535	2,25556641	RPS17;RPS19;RPS6;RPS3A;RPL10A;SEPP1;RPL39
Lomustine-4.2_mg/kg_in_Corn_Oil-Rat-Liver-1d-up	7/332	0,210865633	0,404278902	0	0	1,44909535	2,25556641	EEF1B2;RPS19;RPSA;RPS3A;SEPP1;RPL39;TPT1
Methimazole-28_mg/kg_in_Water-Rat-Liver-0.25d-up	7/332	0,210865633	0,404180513	0	0	1,44909535	2,25556641	RPS17;RPS5;RPS6;RPS20;RPSA;RPL10A;SEPP1
Amikacin-82_mg/kg_in_Saline-Rat-Kidney-5d-up	6/275	0,212805954	0,407800412	0	0	1,499531396	2,320336709	ARL2BP;RPS8;RPS6;RPSA;RPL39;TPT1
Angiotensin_II_Human-0.72_mg/kg_in_Saline-Rat-Heart-3d-dn	6/275	0,212805954	0,407701215	0	0	1,499531396	2,320336709	EEF1B2;RPS17;RPL32;RPS6;SEPP1;TPT1
Cinnarizine-750_mg/kg_in_CMC-Rat-Liver-5d-up	6/275	0,212805954	0,407602066	0	0	1,499531396	2,320336709	RPS8;FEM1B;RPS20;RPS3A;RPL39;TPT1
Citalopram-90_mg/kg_in_Corn_Oil-Rat-Liver-3d-dn	6/275	0,212805954	0,407502965	0	0	1,499531396	2,320336709	EEF1B2;RPS17;RPL32;RPS8;RPS3A;TPT1
Diclofenac-3.5_mg/kg_in_Corn_Oil-Rat-Kidney-5d-up	6/275	0,212805954	0,407403912	0	0	1,499531396	2,320336709	CLP1;RPS8;RPS5;RPS20;SEPP1;TOMM20
Myrtecaine-140_mg/kg_in_Corn_Oil-Rat-Heart-5d-up	6/275	0,212805954	0,407304907	0	0	1,499531396	2,320336709	RPS17;RPS19;RPS8;RPS6;RPS3A;RAPGEF5
Phenothiazine-36_mg/kg_in_Corn_Oil-Rat-Liver-1d-dn	6/275	0,212805954	0,407205951	0	0	1,499531396	2,320336709	RPS17;RPL32;RPS6;RPS20;RPS3A;RPL10A
Racepinephrine-0.77_mg/kg_in_Saline-Rat-Heart-5d-dn	6/275	0,212805954	0,407107042	0	0	1,499531396	2,320336709	CXCL11;RPS19;COL5A3;RPS6;RPS3A;RAPGEF5
Rifampin-99_mg/kg_in_CMC-Rat-Kidney-1d-up	6/275	0,212805954	0,407008182	0	0	1,499531396	2,320336709	RPS17;RPL32;RPS19;RPS8;RPS3A;TPT1
Salicylamide-1300_mg/kg_in_CMC-Rat-Kidney-3d-dn	6/275	0,212805954	0,40690937	0	0	1,499531396	2,320336709	RPS19;ARL2BP;RPS6;RPS20;RPL10A;RPL39
Salicylic_Acid-223_mg/kg_in_Corn_Oil-Rat-Liver-1d-dn	6/275	0,212805954	0,406810605	0	0	1,499531396	2,320336709	RPL32;RPS19;RPL35A;RPS3A;SEPP1;TPT1
Busulfan-3_mg/kg_in_Corn_Oil-Rat-Liver-5d-up	7/333	0,212937482	0,406963264	0	0	1,444743713	2,234666969	RPS17;RPS19;RPS8;RPS5;RPS20;RPSA;TPT1
Hexachloro-13-Butadiene-50_mg/kg_in_Corn_Oil-Rat-Kidney-5d-up	7/333	0,212937482	0,406864534	0	0	1,444743713	2,234666969	RPS17;RPS19;RPS8;RPSA;RPS3A;RPL10A;TPT1
Meloxicam-6_mg/kg_in_Corn_Oil-Rat-Liver-5d-dn	7/333	0,212937482	0,406765853	0	0	1,444743713	2,234666969	EEF1B2;RPS17;RPL32;RPS20;RPSA;RPL10A;SEPP1
Rofecoxib-3_mg/kg_in_Corn_Oil-Rat-Kidney-0.25d-up	7/333	0,212937482	0,406667219	0	0	1,444743713	2,234666969	RPS17;RPL13A;RPS20;RPSA;SEPP1;RPL39;TPT1
Temafloxacin-1000_mg/kg_in_CMC-Rat-Kidney-5d-up	7/333	0,212937482	0,406568633	0	0	1,444743713	2,234666969	RPS6;RPSA;RPS3A;RAPGEF5;RPL10A;RPL39;TPT1
Vincristine-0.05_mg/kg_in_Saline-Rat-Bone_marrow-0.25d-dn	7/333	0,212937482	0,406470095	0	0	1,444743713	2,234666969	RPS19;RPS20;RPSA;RPL10A;SEPP1;RPL39;TPT1
Neomycin-56_mg/kg_in_Corn_Oil-Rat-Kidney-1d-up	8/392	0,213374836	0,407206254	0	0	1,402622905	2,166638427	RPS17;RPL32;RPS19;RPS8;RPS6;RPS20;RPS3A;RPL39
Amikacin-160_mg/kg_in_Saline-Rat-Kidney-14d-up	7/334	0,21501688	0,410240539	0	0	1,440418133	2,213978479	CXCL11;RPL32;RPS6;RPSA;RPS3A;RPL10A;RPL39
Bezafibrate-100_mg/kg_in_Corn_Oil-Rat-Kidney-0.25d-up	7/334	0,21501688	0,410141183	0	0	1,440418133	2,213978479	EEF1B2;FUNDC1;RPS20;RPS3A;RPL39;GOLGA7;PDRG1
Bis(2-Ethylhexyl)Phthalate-1000_mg/kg_in_Corn_Oil-Rat-Liver-1d-up	7/334	0,21501688	0,410041875	0	0	1,440418133	2,213978479	RPS17;RPS19;RPS20;RPSA;RPL10A;SEPP1;RPL39
Genistein-20_mg/kg_in_Corn_Oil-Rat-Liver-0.25d-up	7/334	0,21501688	0,409942615	0	0	1,440418133	2,213978479	RPS17;RPS8;RPS5;RPS6;RPS20;RPSA;GOLGA7
Iproniazid-46_mg/kg_in_Water-Rat-Heart-5d-dn	7/334	0,21501688	0,409843403	0	0	1,440418133	2,213978479	RPS19;RPS6;RPSA;RPL10A;SEPP1;RPL39;PDRG1
Methyldopa-325_mg/kg_in_Water-Rat-Heart-5d-dn	7/334	0,21501688	0,40974424	0	0	1,440418133	2,213978479	EEF1B2;RPL32;RPS19;RPS8;RPS6;RPSA;RPS3A
Sodium_Selenite-2_mg/kg_in_Water-Rat-Kidney-5d-dn	5/219	0,21509563	0,409795158	0	0	1,569144346	2,411261054	RPS17;RPL32;RPS20;RPSA;SEPP1
Azithromycin-225_mg/kg_in_Water-Rat-Liver-3d-dn	6/276	0,215105222	0,409714323	0	0	1,494098312	2,29587325	RPS19;RPS20;RPSA;RPL39;SEPP1;TPT1
Calcitriol-0.04_mg/kg_in_CMC-Rat-Heart-3d-up	6/276	0,215105222	0,409615263	0	0	1,494098312	2,29587325	EEF1B2;C1QA;CXCL11;RPS19;RPS8;RPL39
Ketorolac-48_mg/kg_in_Water-Rat-Liver-1d-up	6/276	0,215105222	0,40951625	0	0	1,494098312	2,29587325	RPL32;RPS8;RPS6;RPS3A;RPL39;TPT1

Leflunomide-60_mg/kg_in_Corn_Oil-Rat-Liver-0.25d-dn	6/276	0,215105222	0,409417286	0	0	1,494098312	2,29587325	RPS17;RPS19;RPS8;RPS5;RPL39;TPT1
Pyrilamine-688_mg/kg_in_Water-Rat-Heart-1d-dn	6/276	0,215105222	0,409318369	0	0	1,494098312	2,29587325	EEF1B2;FUNDC1;RPSA;RPL10A;RPL39;SEPP1
S(-)-Carbidopa-1200_mg/kg_in_CMC-Rat-Brain-5d-dn	6/276	0,215105222	0,409219499	0	0	1,494098312	2,29587325	RPS19;ARL2BP;RPS8;FEM1B;ARL6IP5;TPT1
Glycidol-210_mg/kg_in_Water-Rat-Kidney-1d-up	8/393	0,215286404	0,409465278	0	0	1,39905389	2,148647416	RPS17;ARL2BP;RPS8;ARL6IP5;RPS3A;SEPP1;GOLGA7;TPT1
Amiodarone-147_mg/kg_in_CMC-Rat-Heart-1d-dn	7/335	0,217103736	0,412822073	0	0	1,436118377	2,193498476	RPS17;RPL32;RPS8;FEM1B;RPSA;RPL10A;RPL39
Clobetasol_Propionate-17_mg/kg_in_Corn_Oil-Rat-Bone_marrow-5d-up	7/335	0,217103736	0,41272243	0	0	1,436118377	2,193498476	EEF1B2;C1QA;ARL6IP5;NKG7;RPL35A;SLC16A3;TPT1
Dipyridamole-750_mg/kg_in_CMC-Rat-Liver-1d-up	7/335	0,217103736	0,412622835	0	0	1,436118377	2,193498476	C1QA;RPS17;RPS8;RPS20;SEPP1;RPL39;GOLGA7
Fenbendazole-375_mg/kg_in_Corn_Oil-Rat-Liver-5d-up	7/335	0,217103736	0,412523288	0	0	1,436118377	2,193498476	C1QA;RPS17;RPL32;RPS8;RPS5;RPSA;TPT1
Ibuprofen-54_mg/kg_in_Corn_Oil-Rat-Kidney-0.25d-up	7/335	0,217103736	0,412423788	0	0	1,436118377	2,193498476	RPS17;RPS19;CLP1;RPS20;RPSA;SEPP1;GOLGA7
Acyclovir-330_mg/kg_in_Saline-Rat-Spleen-3d-dn	6/277	0,217413076	0,412911836	0	0	1,488704455	2,271697734	C1QA;RPS17;RPL10A;RPL39;SEPP1;TPT1
Etodolac-24_mg/kg_in_CMC-Rat-Liver-3d-dn	6/277	0,217413076	0,412812291	0	0	1,488704455	2,271697734	EEF1B2;RPL32;RPS8;ARL6IP5;RPS20;SEPP1
Ifosfamide-143_mg/kg_in_Saline-Rat-Spleen-5d-up	6/277	0,217413076	0,412712794	0	0	1,488704455	2,271697734	C1QA;CXCL11;RPS19;ARL2BP;RPS3A;SEPP1
Omeprazole-30_mg/kg_in_Corn_Oil-Rat-Liver-3d-dn	6/277	0,217413076	0,412613346	0	0	1,488704455	2,271697734	C1QA;RPS17;RPS6;RPL35A;RPSA;RPL10A
Tacrolimus-134_mg/kg_in_CMC-Rat-Kidney-1d-dn	6/277	0,217413076	0,412513945	0	0	1,488704455	2,271697734	RPS19;RPSA;RPL10A;RPL39;SEPP1;TPT1
Valdecoxib-450_uM_in_DMSO-Rat-Primary_rat_hepatocytes-1d-up	6/277	0,217413076	0,412414592	0	0	1,488704455	2,271697734	FEM1B;RPS20;RPSA;RPL10A;GOLGA7;PDRG1
Isoprenaline-15_mg/kg_in_Saline-Rat-Liver-5d-up	7/336	0,219197963	0,415700255	0	0	1,431844215	2,173224535	C1QA;RPS17;RPS19;RPS8;RPSA;RPS3A;RPL39
Leflunomide-60_mg/kg_in_Corn_Oil-Rat-Kidney-1d-up	7/336	0,219197963	0,415600183	0	0	1,431844215	2,173224535	RPS19;RPS8;RPS5;RPS6;RPS20;RPL10A;TPT1
Trans-Platinum(II)Diammine_Dichloride-0.5_mg/kg_in_Saline-Rat-Kidney-14d-up	7/336	0,219197963	0,415500159	0	0	1,431844215	2,173224535	RPL32;RPS19;ARL6IP5;RPS3A;SEPP1;RPL39;TPT1
Chlorambucil-0.6_mg/kg_in_Corn_Oil-Rat-Bone_marrow-1d-dn	6/278	0,219729391	0,416407288	0	0	1,483349403	2,247806169	RPS8;RPS6;ARL6IP5;NKG7;RPSA;TPT1
Hydroxyurea-59_mg/kg_in_Saline-Rat-Bone_marrow-0.25d-dn	6/278	0,219729391	0,416307117	0	0	1,483349403	2,247806169	RPS8;RPSA;RPL10A;GOLGA7;SEPP1;TPT1
Levamisole-120_mg/kg_in_Water-Rat-Liver-0.25d-dn	6/278	0,219729391	0,416206995	0	0	1,483349403	2,247806169	EEF1B2;RPS17;RPL35A;RPS20;RPS3A;RPL39
Roxithromycin-312_mg/kg_in_CMC-Rat-Kidney-3d-up	6/278	0,219729391	0,416106922	0	0	1,483349403	2,247806169	EEF1B2;RPS17;RPS19;RPS8;RPS3A;TPT1
Sildenafil-420_mg/kg_in_Water-Rat-Liver-0.25d-dn	5/221	0,220325193	0,417134908	0	0	1,554943944	2,352087007	RPS17;RPL32;RPS8;RPL35A;RPS3A
Beta-Estradiol-150_mg/kg_in_Corn_Oil-Rat-Skeletal_muscle-5d-dn	7/337	0,221299471	0,418878788	0	0	1,427595419	2,153154261	COL5A3;ARL6IP5;RPSA;RPS3A;SEPP1;RPL39;SLC16A3
Diclofenac-3.5_mg/kg_in_Corn_Oil-Rat-Kidney-1d-up	7/337	0,221299471	0,418778144	0	0	1,427595419	2,153154261	RPS6;RPSA;RAPGEF5;RPL10A;RPL39;GOLGA7;TPT1
N-Nitrosodiethylamine-34_mg/kg_in_Saline-Rat-Bone_marrow-1d-up	7/337	0,221299471	0,418677549	0	0	1,427595419	2,153154261	C1QA;RPS19;R3HCC1;RPS6;RPS20;RPSA;RPS3A
Pentamidine-18_mg/kg_in_Saline-Rat-Bone_marrow-1d-dn	7/337	0,221299471	0,418577002	0	0	1,427595419	2,153154261	RPL32;FEM1B;RPS5;RPS6;RPL35A;RPS3A;RPL39
Rofecoxib-775_mg/kg_in_Corn_Oil-Rat-Liver-1d-up	7/337	0,221299471	0,418476503	0	0	1,427595419	2,153154261	RPL32;RPS19;RPS8;RPS5;RPS20;RPSA;RPL10A
Droperidol-94_mg/kg_in_CMC-Rat-Heart-5d-up	6/279	0,222054045	0,419802606	0	0	1,478032738	2,22419463	RPS17;RPL32;FUNDC1;RPS20;RPL10A;RPL39
Epirubicin-2.7_mg/kg_in_Saline-Rat-Kidney-5d-up	6/279	0,222054045	0,419701861	0	0	1,478032738	2,22419463	EEF1B2;RPS17;CXCL11;RPL10A;RPL39;SEPP1
Griseofulvin-2500_mg/kg_in_Corn_Oil-Rat-Liver-3d-dn	6/279	0,222054045	0,419601165	0	0	1,478032738	2,22419463	EEF1B2;RPL32;RPS8;RPS5;RPL35A;RPL10A
Neomycin-56_mg/kg_in_Corn_Oil-Rat-Liver-5d-up	6/279	0,222054045	0,419500517	0	0	1,478032738	2,22419463	RPL32;RPS19;RPS8;RPS20;RPL39;TPT1
Quinapril-2500_mg/kg_in_CMC-Rat-Kidney-3d-up	6/279	0,222054045	0,419399918	0	0	1,478032738	2,22419463	CXCL11;RPS19;RPS6;RPS20;RPSA;RPL39
Sulfaphenazole-1695_mg/kg_in_Water-Rat-Liver-3d-dn	6/279	0,222054045	0,419299366	0	0	1,478032738	2,22419463	EEF1B2;RPS19;CLP1;RPS20;RPL39;SEPP1
Tropisetron-100_mg/kg_in_CMC-Rat-Liver-3d-dn	6/279	0,222054045	0,419198863	0	0	1,478032738	2,22419463	RPS17;RPL32;RPS8;RPS20;SEPP1;TPT1
Chloroxylenol-1915_mg/kg_in_Corn_Oil-Rat-Liver-1d-dn	7/338	0,22340817	0,421654145	0	0	1,423371764	2,133285295	RPL32;RPS6;RPL35A;RPS3A;RPL10A;RPL39;TPT1
Nimesulide-61.7_uM_in_DMSO-Rat-Primary_rat_hepatocytes-1d-dn	7/338	0,22340817	0,421553126	0	0	1,423371764	2,133285295	CXCL11;RPL32;RPS19;ARL6IP5;SEPP1;RPL39;TPT1
Pioglitazone-1500_mg/kg_in_Corn_Oil-Rat-Liver-3d-up	7/338	0,22340817	0,421452155	0	0	1,423371764	2,133285295	EEF1B2;RPL32;RPS8;RPS5;RPL35A;RPS20;TPT1
Sodium_Arsenite-30_mg/kg_in_Saline-Rat-Liver-1d-up	7/338	0,22340817	0,421351232	0	0	1,423371764	2,133285295	RPS17;RPS8;RPS6;RPS20;RPSA;RPS3A;RPL39
Spironolactone-300_mg/kg_in_CMC-Rat-Kidney-1d-dn	7/338	0,22340817	0,421250358	0	0	1,423371764	2,133285295	RPL32;RPS19;ARL2BP;CLP1;RPS8;RPSA;SEPP1
Triamterene-200_mg/kg_in_CMC-Rat-Kidney-5d-up	7/338	0,22340817	0,421149532	0	0	1,423371764	2,133285295	EEF1B2;C1QA;RPL32;RPS19;RPS8;ARL6IP5;RPSA
Clotrimazole-52_mg/kg_in_Corn_Oil-Rat-Liver-1d-dn	6/280	0,224386911	0,42289335	0	0	1,47275405	2,200859259	EEF1B2;RPS17;RPL32;RPS6;RPS3A;SEPP1
Doxofylline-483_mg/kg_in_Water-Rat-Heart-5d-up	6/280	0,224386911	0,422792179	0	0	1,47275405	2,200859259	CXCL11;RPS19;FEM1B;RPL10A;RPL39;GOLGA7
Etoposide-100_mg/kg_in_Corn_Oil-Rat-Bone_marrow-1d-dn	6/280	0,224386911	0,422691057	0	0	1,47275405	2,200859259	RPS8;FEM1B;RPS6;RPSA;RPS3A;TPT1
Ketamine-30_mg/kg_in_Saline-Rat-Heart-1d-dn	6/280	0,224386911	0,422589983	0	0	1,47275405	2,200859259	RPS19;RPS6;RPS3A;RPL39;SEPP1;TPT1
Nitrendipine-1100_mg/kg_in_Corn_Oil-Rat-Heart-1d-dn	6/280	0,224386911	0,422488958	0	0	1,47275405	2,200859259	ARL2BP;CLP1;RPS8;FUNDC1;RPS6;SEPP1
Sulindac-750_uM_in_DMSO-Rat-Primary_rat_hepatocytes-0.67d-up	6/280	0,224386911	0,42238798	0	0	1,47275405	2,200859259	EEF1B2;RPS17;CLP1;FEM1B;RPS6;RPL39
Doxepin-147_mg/kg_in_CMC-Rat-Heart-1d-dn	7/339	0,225523969	0,424426949	0	0	1,419173028	2,113615309	EEF1B2;RPL32;RPS8;RPS5;RPS20;RPSA;RPL10A
Trovafloxacin-600_mg/kg_in_CMC-Rat-Liver-5d-dn	7/339	0,225523969	0,424325556	0	0	1,419173028	2,113615309	C1QA;RPS17;RPL32;RPS19;RPS8;RPSA;TPT1
Carboplatin-6_mg/kg_in_Saline-Rat-Bone_marrow-3d-dn	5/223	0,225593599	0,424355191	0	0	1,540998259	2,294577433	RPS19;FUNDC1;RPL35A;RPL39;TPT1
Acetazolamide-250_mg/kg_in_Corn_Oil-Rat-Kidney-1d-dn	6/281	0,226727864	0,426386977	0	0	1,467512932	2,17779626	RPS17;CLP1;RPS6;RPS3A;GOLGA7;SEPP1
Allyl_Alcohol-16_mg/kg_in_Saline-Rat-Kidney-3d-dn	6/281	0,226727864	0,426285189	0	0	1,467512932	2,17779626	EEF1B2;RPS19;RPS6;RPS20;RPL10A;SEPP1
Beta-Naphthoflavone-1500_mg/kg_in_CMC-Rat-Liver-1d-dn	6/281	0,226727864	0,426183451	0	0	1,467512932	2,17779626	EEF1B2;CLP1;RPS6;RPS20;RPL39;SEPP1
Econazole-334_mg/kg_in_Corn_Oil-Rat-Liver-0.25d-up	6/281	0,226727864	0,426081761	0	0	1,467512932	2,17779626	RPS17;RPS19;RPS6;RPS3A;RPL10A;RPL39
Hydroxyurea-59_mg/kg_in_Saline-Rat-Liver-5d-up	6/281	0,226727864	0,425980119	0	0	1,467512932	2,17779626	EEF1B2;RPS17;RPL32;RPSA;RPL39;TPT1
Nevirapine-200_mg/kg_in_Saline-Rat-Heart-5d-dn	6/281	0,226727864	0,425878526	0	0	1,467512932	2,17779626	RPS17;RPL32;ARL2BP;RPL35A;RPS3A;RAPGEF5
Nimesulide-30_mg/kg_in_Corn_Oil-Rat-Liver-5d-up	6/281	0,226727864	0,425776981	0	0	1,467512932	2,17779626	EEF1B2;RPS17;RPL32;RPS3A;RPL39;TPT1
Quinapril-2500_mg/kg_in_CMC-Rat-Kidney-0.25d-dn	6/281	0,226727864	0,425675485	0	0	1,467512932	2,17779626	RPS19;ARL2BP;CLP1;RPS8;RPS6;RPL10A
Tocainide-67_mg/kg_in_Corn_Oil-Rat-Liver-5d-up	6/281	0,226727864	0,425574037	0	0	1,467512932	2,17779626	RPS17;RPS19;CLP1;RPS8;RPS20;TPT1
6-Mercaptopurine-25_mg/kg_in_Corn_Oil-Rat-Liver-1d-up	7/340	0,227646777	0,42719705	0	0	1,414998989	2,094142008	RPS17;RPS19;RPS20;RPSA;SEPP1;RPL39;TPT1
Emetine-1_mg/kg_in_Saline-Rat-Kidney-3d-dn	7/340	0,227646777	0,427095288	0	0	1,414998989	2,094142008	EEF1B2;RPS19;FUNDC1;RPS6;RPL10A;SEPP1;RPL39
Sparteine-225_mg/kg_in_Water-Rat-Heart-1d-up	7/340	0,227646777	0,426993574	0	0	1,414998989	2,094142008	RPS17;COL5A3;RPS6;RPS3A;SEPP1;RPL39;TPT1
Carboplatin-14_mg/kg_in_Saline-Rat-Bone_marrow-3d-dn	5/224	0,228241905	0,428007916	0	0	1,534118802	2,266429222	RPL32;RPS19;RPS6;RPS3A;RPL39
Aldosterone-0.065_mg/kg_in_Saline-Rat-Kidney-3d-up	6/282	0,229076779	0,429471248	0	0	1,462308986	2,155001902	C1QA;ARL2BP;CLP1;RPS8;RPSA;SEPP1
Baclofen-19_mg/kg_in_CMC-Rat-Heart-3d-up	6/282	0,229076779	0,429369042	0	0	1,462308986	2,155001902	RPL32;RPS19;RPS6;RPSA;RPL39;TPT1
Cyclophosphamide-25_mg/kg_in_Water-Rat-Bone_marrow-1d-up	6/282	0,229076779	0,429266884	0	0	1,462308986	2,155001902	RPS8;FUNDC1;NKG7;RPSA;RPL10A;SEPP1
Cytarabine-487_mg/kg_in_Saline-Rat-Kidney-1d-up	6/282	0,229076779	0,429164775	0	0	1,462308986	2,155001902	ARL2BP;CLP1;RPS8;RPSA;SEPP1;TPT1
Dicyclomine-645_mg/kg_in_Water-Rat-Heart-5d-up	6/282	0,229076779	0,429062714	0	0	1,462308986	2,155001902	RPS17;RPS6;RPS3A;RPL10A;SEPP1;TPT1
Mebendazole-714_mg/kg_in_Corn_Oil-Rat-Liver-1d-dn	6/282	0,229076779	0,428960702	0	0	1,462308986	2,155001902	EEF1B2;RPS17;RPS8;RPS3A;RPL10A;SEPP1
NN-Dimethylformamide-1400_mg/kg_in_Saline-Rat-Spleen-1d-up	6/282	0,229076779	0,428858739	0	0	1,462308986	2,155001902	C1QA;CXCL11;RPS19;RPS6;RPL39;SEPP1
Nitrofurantoin-76_mg/kg_in_CMC-Rat-Liver-5d-dn	6/282	0,229076779	0,428756824	0	0	1,462308986	2,155001902	RPS17;RPL32;RPS8;RPL35A;RPS3A;TPT1
Phenacetin-619_mg/kg_in_Corn_Oil-Rat-Liver-3d-dn	6/282	0,229076779	0,428654957	0	0	1,462308986	2,155001902	EEF1B2;RPS17;RPS6;RPL35A;RPL39;TPT1
Rofecoxib-775_mg/kg_in_Corn_Oil-Rat-Liver-5d-up	6/282	0,229076779	0,428553139	0	0	1,462308986	2,155001902	EEF1B2;C1QA;RPS20;RPSA;RPL10A;RPL39
Valsartan-1000_mg/kg_in_Corn_Oil-Rat-Kidney-3d-up	6/282	0,229076779	0,428451369	0	0	1,462308986	2,155001902	RPS17;ARL2BP;RPS8;RPS20;RPS3A;TPT1
Loxoprofen-18_mg/kg_in_Corn_Oil-Rat-Kidney-0.25d-up	7/341	0,229776504	0,429658059	0	0	1,410849432	2,074863127	C1QA;RPS19;RPS6;RPSA;RPL10A;RPL39;TPT1
Nabumetone-2500_mg/kg_in_Corn_Oil-Rat-Kidney-1d-up	7/341	0,229776504	0,429556076	0	0	1,410849432	2,074863127	C1QA;RPS19;RPS5;RPS6;RPS3A;RPL10A;RPL39
Tretinoin-7_mg/kg_in_Corn_Oil-Rat-Liver-1d-dn	7/341	0,229776504	0,42945414	0	0	1,410849432	2,074863127	RPS17;RPS19;RPS8;RPS6;RPL10A;RPL39;TPT1
Azithromycin-225_mg/kg_in_Water-Rat-Liver-0.25d-dn	5/225	0,230899367	0,431450395	0	0	1,527300496	2,238676296	RPS17;RPS19;RPS8;RPL35A;RPSA
Valproic_Acid-235_mg/kg_in_Water-Rat-Kidney-3d-dn	5/225	0,230899367	0,431348058	0	0	1,527300496	2,238676296	ARL2BP;CLP1;RPL10A;SEPP1;TPT1
1-Naphthyl_Isothiocyanate-30_mg/kg_in_Corn_Oil-Rat-Liver-1d-up	6/283	0,23143353	0,432243416	0	0	1,457141816	2,132472512	EEF1B2;RPS17;RPS19;RPS6;RPSA;RPS3A
Catechol-195_mg/kg_in_Saline-Rat-Kidney-1d-dn	6/283	0,23143353	0,43214094	0	0	1,457141816	2,132472512	RPS19;ARL2BP;CLP1;R3HCC1;RPL10A;TPT1
Chlorambucil-4.5_mg/kg_in_Corn_Oil-Rat-Spleen-1d-dn	6/283	0,23143353	0,432038512	0	0	1,457141816	2,132472512	RPS19;RPS8;RPL13A;RPL35A;RPS20;RPSA
Doxepin-132_uM_in_DMSO-Rat-Primary_rat_hepatocytes-0.67d-up	6/283	0,23143353	0,431936134	0	0	1,457141816	2,132472512	RPS19;RPS6;RPS20;RPS3A;RPL10A;PDRG1
Mifepristone-300_mg/kg_in_Corn_Oil-Rat-Liver-3d-up	6/283	0,23143353	0,431833803	0	0	1,457141816	2,132472512	EEF1B2;RPS19;RPS6;RPS20;RPSA;RPL10A
Monocrotaline-333_uM_in_DMSO-Rat-Primary_rat_hepatocytes-1d-up	6/283	0,23143353	0,431731521	0	0	1,457141816	2,132472512	EEF1B2;RPS17;RPL32;CLP1;RPS3A;PDRG1
Sulfadiazine-1170_mg/kg_in_Water-Rat-Liver-1d-up	6/283	0,23143353	0,431629288	0	0	1,457141816	2,132472512	RPS17;CLP1;RPS8;RPS6;RPSA;RPL10A
3-Acetamidophenol-700_mg/kg_in_Corn_Oil-Rat-Liver-3d-dn	6/284	0,23379799	0,435935836	0	0	1,452011035	2,110204481	EEF1B2;RPL32;RPS8;RPS5;RPS6;RPL35A
Altretamine-13_mg/kg_in_Corn_Oil-Rat-Liver-5d-dn	6/284	0,23379799	0,435832656	0	0	1,452011035	2,110204481	RPS17;RPS19;RPS6;RPSA;RPS3A;SEPP1
Daunorubicin-4_mg/kg_in_Saline-Rat-Kidney-5d-dn	6/284	0,23379799	0,435729525	0	0	1,452011035	2,110204481	C1QA;RPS17;RPS6;ARL6IP5;RPL35A;RPL39
Etoposide-100_mg/kg_in_Corn_Oil-Rat-Bone_marrow-3d-dn	6/284	0,23379799	0,435626442	0	0	1,452011035	2,110204481	RPS19;FEM1B;RPS3A;RPL10A;RPL39;TPT1

Ipriflavone-1500_mg/kg_in_CMC-Rat-Liver-5d-dn	6/284	0,23379799	0,435523408	0	0	1,452011035	2,110204481	EEF1B2;RPS17;CLP1;RPS8;RPL35A;RPS3A
Irinotecan-5_mg/kg_in_Saline-Rat-Bone_marrow-3d-up	6/284	0,23379799	0,435420423	0	0	1,452011035	2,110204481	EEF1B2;RPS17;RPS19;RPL10A;RPL39;SEPP1
Itraconazole-10_uM_in_DMSO-Rat-Primary_rat_hepatocytes-1d-up	6/284	0,23379799	0,435317487	0	0	1,452011035	2,110204481	EEF1B2;RPS17;RPL32;RPS8;RPS5;RPS3A
N-Nitrosodiethylamine-7000_uM_in_DMSO-Rat-Primary_rat_hepatocytes-0.67d-dn	6/284	0,23379799	0,4352146	0	0	1,452011035	2,110204481	RPS17;RPS19;FUNDC1;RPL10A;SLC16A3;TPT1
Nimesulide-162_mg/kg_in_Corn_Oil-Rat-Kidney-1d-dn	6/284	0,23379799	0,435111761	0	0	1,452011035	2,110204481	EEF1B2;FUNDC1;RPL35A;RPL39;SEPP1;TOMM20
Oxymetholone-1170_mg/kg_in_CMC-Rat-Liver-3d-dn	6/284	0,23379799	0,43500897	0	0	1,452011035	2,110204481	RPL32;RPS19;CLP1;RPS8;SEPP1;TPT1
Phenothiazine-36_mg/kg_in_Corn_Oil-Rat-Liver-5d-dn	6/284	0,23379799	0,434906228	0	0	1,452011035	2,110204481	C1QA;RPS17;RPS6;RPS20;RPS3A;RPL10A
Piclamilast-17_mg/kg_in_CMC-Rat-Liver-5d-up	6/284	0,23379799	0,434803535	0	0	1,452011035	2,110204481	RPS8;RPS3A;RAPGEF5;RPL10A;RPL39;TPT1
Roxithromycin-312_mg/kg_in_CMC-Rat-Liver-5d-up	6/284	0,23379799	0,43470089	0	0	1,452011035	2,110204481	C1QA;RPS17;RPSA;RPS3A;RPL39;TPT1
Simvastatin-15_mg/kg_in_Corn_Oil-Rat-Kidney-1d-dn	6/284	0,23379799	0,434598294	0	0	1,452011035	2,110204481	EEF1B2;RPL32;RPS19;RPL10A;RPL39;SEPP1
Tacrolimus-50_uM_in_DMSO-Rat-Primary_rat_hepatocytes-0.67d-up	6/284	0,23379799	0,434495746	0	0	1,452011035	2,110204481	EEF1B2;RPS19;RPS5;RPS6;RPS3A;PDRG1
Temafloxacin-1000_mg/kg_in_CMC-Rat-Kidney-3d-dn	6/284	0,23379799	0,434393246	0	0	1,452011035	2,110204481	RPL32;RPS19;RPL35A;RPS20;RPSA;SEPP1
N-Nitrosodiethylamine-1.67_mg/kg_in_Saline-Rat-Liver-1d-up	7/343	0,234056345	0,434770702	0	0	1,402622905	2,036879725	C1QA;RPS17;RPS19;RPS8;RPS20;RPSA;TPT1
Atropine-2.3_mg/kg_in_Water-Rat-Liver-0.25d-dn	6/285	0,236170033	0,438593534	0	0	1,44691626	2,088194258	C1QA;RPS17;RPS19;RPS6;RPSA;SEPP1
Bisphenol_A-610_mg/kg_in_Corn_Oil-Rat-Bone_marrow-5d-up	6/285	0,236170033	0,438490141	0	0	1,44691626	2,088194258	C1QA;RPS19;FEM1B;RPS6;RPL10A;RPL39
Dexamethasone-150_mg/kg_in_Corn_Oil-Rat-Liver-0.25d-dn	6/285	0,236170033	0,438386796	0	0	1,44691626	2,088194258	EEF1B2;C1QA;RPL32;RPS8;SEPP1;TPT1
Ethanol-3000_mg/kg_in_Saline-Rat-Liver-0.25d-dn	6/285	0,236170033	0,438283501	0	0	1,44691626	2,088194258	RPS19;ARL2BP;CLP1;RPS8;RPS20;RPL39
Guanethidine-132_mg/kg_in_CMC-Rat-Heart-5d-dn	6/285	0,236170033	0,438180254	0	0	1,44691626	2,088194258	RPS8;COL5A3;RPS6;RPL39;GOLGA7;SEPP1
Leflunomide-60_mg/kg_in_Corn_Oil-Rat-Liver-3d-up	6/285	0,236170033	0,438077055	0	0	1,44691626	2,088194258	EEF1B2;RPS17;RPL32;RPSA;RPL39;TPT1
Lovastatin-1500_mg/kg_in_Corn_Oil-Rat-Liver-3d-dn	6/285	0,236170033	0,437973905	0	0	1,44691626	2,088194258	C1QA;RPS17;CLP1;RPS3A;GOLGA7;SEPP1
Methotrexate-27_mg/kg_in_Corn_Oil-Rat-Liver-3d-up	6/285	0,236170033	0,437870804	0	0	1,44691626	2,088194258	EEF1B2;RPS17;RPS8;RPL35A;RPS20;SEPP1
Oxcarbazepine-1600_mg/kg_in_CMC-Rat-Brain-5d-dn	6/285	0,236170033	0,437767752	0	0	1,44691626	2,088194258	EEF1B2;RPS19;ARL2BP;CLP1;R3HCC1;PDRG1
Trovafloxacin-600_mg/kg_in_CMC-Rat-Liver-3d-up	6/285	0,236170033	0,437664747	0	0	1,44691626	2,088194258	EEF1B2;RPS6;RPS3A;RPL10A;RPL39;TPT1
Zidovudine-1540_mg/kg_in_Corn_Oil-Rat-Heart-5d-dn	6/285	0,236170033	0,437561792	0	0	1,44691626	2,088194258	RPS17;RPL32;RPS19;RPL35A;RPS3A;GOLGA7
Acetaminophen-1950_uM_in_DMSO-Rat-Primary_rat_hepatocytes-1d-dn	7/344	0,236206276	0,437526018	0	0	1,398545513	2,01817083	RPS17;RPS19;RPSA;RPL10A;SEPP1;RPL39;SLC16A3
Lomustine-8.75_mg/kg_in_Corn_Oil-Rat-Spleen-3d-dn	7/344	0,236206276	0,437423143	0	0	1,398545513	2,01817083	C1QA;RPS17;RPS19;RPSA;RPL10A;RPL39;TPT1
Trichloroethane-300_mg/kg_in_Water-Rat-Liver-0.25d-dn	5/227	0,236241011	0,437384628	0	0	1,513844104	2,184329837	CLP1;RPL35A;RPS20;RPS3A;RPL39
Gemfibrozil-100_mg/kg_in_Corn_Oil-Rat-Kidney-3d-dn	7/345	0,238362756	0,441209181	0	0	1,394491758	1,999647605	EEF1B2;RPS17;RPL32;RPS19;CLP1;RPS6;TPT1
Bis(2-Ethylhexyl)Phthalate-1000_mg/kg_in_Corn_Oil-Rat-Liver-5d-dn	6/286	0,23854953	0,441451152	0	0	1,441857112	2,066438347	EEF1B2;RPS6;RPS20;RPSA;RPS3A;SEPP1
Cortisone-206_mg/kg_in_CMC-Rat-Kidney-3d-up	6/286	0,23854953	0,441347452	0	0	1,441857112	2,066438347	EEF1B2;ARL2BP;CLP1;RPS8;RPS3A;SEPP1
Digitoxin-2.75_mg/kg_in_Corn_Oil-Rat-Heart-3d-dn	6/286	0,23854953	0,4412438	0	0	1,441857112	2,066438347	ARL2BP;RPS8;FUNDC1;ARL6IP5;RPS20;RPSA
Lead(IV)_Acetate-600_mg/kg_in_Saline-Rat-Liver-0.25d-up	6/286	0,23854953	0,441140198	0	0	1,441857112	2,066438347	RPS17;RPS6;RPSA;RPS3A;RPL39;SEPP1
Ondansetron-84_mg/kg_in_Water-Rat-Intestine-1d-up	6/286	0,23854953	0,441036644	0	0	1,441857112	2,066438347	C1QA;ARL2BP;RPS20;RPSA;RPL39;SEPP1
Vinblastine-0.3_mg/kg_in_Saline-Rat-Heart-5d-dn	6/286	0,23854953	0,440933138	0	0	1,441857112	2,066438347	RPS17;CXCL11;FEM1B;ARL6IP5;GOLGA7;SEPP1
Pentobarbital-20_mg/kg_in_Water-Rat-Liver-0.25d-dn	5/228	0,238924822	0,441523205	0	0	1,507204437	2,157723409	EEF1B2;RPS17;RPL32;RPL35A;TPT1
Manganese_(II)_Chloride-700_mg/kg_in_Water-Rat-Liver-3d-up	7/346	0,240525693	0,444377283	0	0	1,390461435	1,981307937	EEF1B2;C1QA;RPS6;RPS20;RPSA;RPL10A;RPL39
123-Trichloropropane-108_mg/kg_in_CMC-Rat-Heart-1d-dn	6/287	0,240936356	0,4450316	0	0	1,43683322	2,044933313	RPS19;RPS8;RPS6;RPS3A;SEPP1;TPT1
Benzethonium_Chloride-138_mg/kg_in_Water-Rat-Heart-5d-dn	6/287	0,240936356	0,444927255	0	0	1,43683322	2,044933313	EEF1B2;RPS19;FUNDC1;ARL6IP5;RPSA;SEPP1
Catechol-40_mg/kg_in_Saline-Rat-Liver-3d-up	6/287	0,240936356	0,444822958	0	0	1,43683322	2,044933313	EEF1B2;RPS19;RPS6;RPS3A;RPL10A;TPT1
Cobalt_(II)_Chloride-10_mg/kg_in_Saline-Rat-Kidney-5d-dn	6/287	0,240936356	0,444718711	0	0	1,43683322	2,044933313	EEF1B2;RPS17;RPS19;RPSA;RPL39;TOMM20
Econazole-334_mg/kg_in_Corn_Oil-Rat-Liver-3d-up	6/287	0,240936356	0,444614513	0	0	1,43683322	2,044933313	RPS17;RPS6;RPS20;RPS3A;RPL10A;RPL39
Epirubicin-2.7_mg/kg_in_Saline-Rat-Heart-1d-up	6/287	0,240936356	0,444510363	0	0	1,43683322	2,044933313	CXCL11;RPS19;CLP1;FUNDC1;RAPGEF5;SEPP1
Ifosfamide-17_mg/kg_in_Saline-Rat-Spleen-3d-up	6/287	0,240936356	0,444406263	0	0	1,43683322	2,044933313	C1QA;RPS19;NKG7;RPL35A;RPS20;SEPP1
Pemoline-833_uM_in_DMSO-Rat-Primary_rat_hepatocytes-1d-up	6/287	0,240936356	0,44430221	0	0	1,43683322	2,044933313	RPS17;RPL32;ARL2BP;CLP1;RPS6;RPS3A
Pyrazinamide-1500_mg/kg_in_CMC-Rat-Liver-5d-up	6/287	0,240936356	0,444198207	0	0	1,43683322	2,044933313	EEF1B2;RPS19;RPS8;RPS6;RPS20;RPS3A
Sotalol-2000_mg/kg_in_Water-Rat-Heart-5d-dn	6/287	0,240936356	0,444094252	0	0	1,43683322	2,044933313	EEF1B2;RPS8;RPS6;RPL13A;RPS3A;TPT1
Ethylene_Glycol-3525_mg/kg_in_Water-Rat-Kidney-1d-dn	5/229	0,241617044	0,445244698	0	0	1,500622758	2,131486469	RPS19;ARL2BP;RPS6;ARL6IP5;RPS20
Catechol-195_mg/kg_in_Saline-Rat-Liver-1d-up	7/347	0,242694993	0,447126496	0	0	1,386454341	1,96314974	C1QA;RPS17;RPL35A;RPSA;RPL10A;RPL39;TPT1
33'4'5-Tetrachlorosalicylanilide-5_mg/kg_in_Corn_Oil-Rat-Liver-0.25d-dn	6/288	0,243330382	0,448192257	0	0	1,431844215	2,023675775	EEF1B2;RPS17;RPL32;RPS3A;RPL39;TPT1
Captopril-1750_mg/kg_in_Water-Rat-Kidney-1d-up	6/288	0,243330382	0,448087466	0	0	1,431844215	2,023675775	RPS8;RPS3A;RAPGEF5;RPL10A;RPL39;TPT1
Chlorambucil-4.5_mg/kg_in_Corn_Oil-Rat-Heart-5d-up	6/288	0,243330382	0,447982723	0	0	1,431844215	2,023675775	RPS19;RPS8;ARL6IP5;RPS20;RPL10A;RPL39
Chlorzoxazone-763_mg/kg_in_CMC-Rat-Liver-3d-up	6/288	0,243330382	0,44787803	0	0	1,431844215	2,023675775	EEF1B2;RPS17;RPS5;RPS6;RPS20;RPSA
Cyclosporin_A-70_mg/kg_in_Corn_Oil-Rat-Bone_marrow-1d-dn	6/288	0,243330382	0,447773386	0	0	1,431844215	2,023675775	RPL32;RPS8;RPS6;ARL6IP5;RPL35A;RPSA
Diazepam-710_mg/kg_in_CMC-Rat-Kidney-1d-up	6/288	0,243330382	0,44766879	0	0	1,431844215	2,023675775	EEF1B2;CLP1;ARL6IP5;RPS3A;RAPGEF5;SEPP1
Ketoconazole-90_uM_in_DMSO-Rat-Primary_rat_hepatocytes-0.67d-dn	6/288	0,243330382	0,447564243	0	0	1,431844215	2,023675775	RPS17;CXCL11;ARL6IP5;SEPP1;SLC16A3;TPT1
Lead_(II)_Acetate-200_mg/kg_in_Saline-Rat-Kidney-5d-up	6/288	0,243330382	0,447459746	0	0	1,431844215	2,023675775	C1QA;CXCL11;RPS19;RPS6;RPL39;GOLGA7
Loratadine-62.25_uM_in_DMSO-Rat-Primary_rat_hepatocytes-0.67d-dn	6/288	0,243330382	0,447355297	0	0	1,431844215	2,023675775	CXCL11;CLP1;ARL6IP5;RPL10A;GOLGA7;SEPP1
Moclobemide-266_mg/kg_in_Water-Rat-Brain-5d-up	6/288	0,243330382	0,447250896	0	0	1,431844215	2,023675775	EEF1B2;RPS19;ARL2BP;RPS6;RPS20;RAPGEF5
N-Nitrosodiethylamine-1.67_mg/kg_in_Saline-Rat-Kidney-1d-up	6/288	0,243330382	0,447146545	0	0	1,431844215	2,023675775	RPS17;RPS20;RPS3A;RPL39;GOLGA7;SEPP1
Oxcarbazepine-1600_mg/kg_in_CMC-Rat-Heart-3d-dn	6/288	0,243330382	0,447042242	0	0	1,431844215	2,023675775	COL5A3;RPS6;ARL6IP5;RPSA;SEPP1;PDRG1
Pentoxifylline-1170_mg/kg_in_CMC-Rat-Heart-3d-up	6/288	0,243330382	0,446937988	0	0	1,431844215	2,023675775	RPS17;RPL32;RPSA;RPS3A;RPL39;TPT1
Prazosin-2000_mg/kg_in_CMC-Rat-Heart-1d-up	6/288	0,243330382	0,446833782	0	0	1,431844215	2,023675775	CXCL11;RPS19;FUNDC1;RAPGEF5;SEPP1;TPT1
Rabeprazole-1024_mg/kg_in_Water-Rat-Liver-3d-dn	6/288	0,243330382	0,446729625	0	0	1,431844215	2,023675775	C1QA;RPS19;CLP1;RPS8;RPL39;TPT1
Sulfanilamide-636_mg/kg_in_Corn_Oil-Rat-Kidney-5d-dn	6/288	0,243330382	0,446625516	0	0	1,431844215	2,023675775	CLP1;RPS8;RPSA;SEPP1;TOMM20;TPT1
Zopiclone-414_mg/kg_in_Water-Rat-Liver-1d-up	6/288	0,243330382	0,446521456	0	0	1,431844215	2,023675775	RPS8;RPS6;RPS20;RPSA;RPL39;TPT1
Aceclofenac-1_mg/kg_in_Corn_Oil-Rat-Kidney-1d-up	7/348	0,244870564	0,449243084	0	0	1,382470277	1,945170958	RPS17;RPL32;RPS5;RPS6;SEPP1;RPL39;TPT1
Azithromycin-225_mg/kg_in_Water-Rat-Kidney-5d-up	7/348	0,244870564	0,449138463	0	0	1,382470277	1,945170958	EEF1B2;ARL2BP;RPS8;RPS6;RPS20;RPS3A;TPT1
Famciclovir-112_mg/kg_in_Saline-Rat-Liver-5d-up	7/348	0,244870564	0,449033891	0	0	1,382470277	1,945170958	C1QA;RPS17;RPS19;RPS8;RPS20;RPSA;TPT1
Hydrochlorothiazide-1500_mg/kg_in_CMC-Rat-Kidney-5d-up	7/348	0,244870564	0,448929367	0	0	1,382470277	1,945170958	RPL32;RPS5;RPL35A;RPSA;SEPP1;TOMM20;TPT1
123-Trichloropropane-108_mg/kg_in_CMC-Rat-Heart-5d-dn	6/289	0,245731481	0,450402872	0	0	1,426889737	2,002662406	CXCL11;RPS19;COL5A3;RPS6;SEPP1;TPT1
4-Nonylphenol-200_mg/kg_in_Corn_Oil-Rat-Liver-1d-dn	6/289	0,245731481	0,450298079	0	0	1,426889737	2,002662406	EEF1B2;C1QA;RPS17;RPL32;RPS20;TPT1
Cadmium_Chloride-2_mg/kg_in_Saline-Rat-Kidney-28d-up	6/289	0,245731481	0,450193334	0	0	1,426889737	2,002662406	RPS19;FEM1B;RPS20;RPSA;RPS3A;RPL10A
Ceramide_C2-44_uM_in_DMSO-Rat-Primary_rat_hepatocytes-0.67d-dn	6/289	0,245731481	0,450088638	0	0	1,426889737	2,002662406	CXCL11;RPS19;RPS20;SEPP1;SLC16A3;TPT1
Chlorpyrifos-82_mg/kg_in_CMC-Rat-Heart-1d-up	6/289	0,245731481	0,44998399	0	0	1,426889737	2,002662406	RPS17;RPS8;FUNDC1;RPS6;RPS3A;SEPP1
Citalopram-90_mg/kg_in_Corn_Oil-Rat-Liver-5d-up	6/289	0,245731481	0,449879392	0	0	1,426889737	2,002662406	RPS19;RPS8;RPS20;RPSA;RPL39;TPT1
Ethisterone-1500_mg/kg_in_CMC-Rat-Liver-1d-dn	6/289	0,245731481	0,449774841	0	0	1,426889737	2,002662406	EEF1B2;RPS19;CLP1;RPS8;SEPP1;TPT1
Lidocaine-71_mg/kg_in_Water-Rat-Heart-5d-dn	6/289	0,245731481	0,44967034	0	0	1,426889737	2,002662406	EEF1B2;COL5A3;FUNDC1;ARL6IP5;RPS20;TPT1
Metoprolol-1200_mg/kg_in_Water-Rat-Heart-1d-up	6/289	0,245731481	0,449565887	0	0	1,426889737	2,002662406	RPS17;RPL32;RPS6;ARL6IP5;RPS3A;SEPP1
Neomycin-877_mg/kg_in_Corn_Oil-Rat-Kidney-3d-dn	6/289	0,245731481	0,449461482	0	0	1,426889737	2,002662406	RPS19;CLP1;RPSA;RPL10A;SEPP1;TPT1
Oxaliplatin-8_mg/kg_in_Saline-Rat-Kidney-5d-up	6/289	0,245731481	0,449357126	0	0	1,426889737	2,002662406	RPS17;RPS19;ARL2BP;CLP1;RPS20;RAPGEF5
Phentolamine-44_mg/kg_in_Saline-Rat-Heart-5d-up	6/289	0,245731481	0,449252819	0	0	1,426889737	2,002662406	C1QA;RPL32;RPS6;RPS20;RPL10A;TPT1
Proglumide-1125_mg/kg_in_CMC-Rat-Liver-5d-up	6/289	0,245731481	0,44914856	0	0	1,426889737	2,002662406	RPS17;RPS20;RPSA;RPL10A;RPL39;SEPP1
Promazine-100_mg/kg_in_Corn_Oil-Rat-Liver-5d-up	6/289	0,245731481	0,449044349	0	0	1,426889737	2,002662406	RPS17;RPS20;RAPGEF5;RPL39;SEPP1;TPT1
Propylthiouracil-625_mg/kg_in_CMC-Rat-Liver-3d-dn	6/289	0,245731481	0,448940186	0	0	1,426889737	2,002662406	EEF1B2;C1QA;RPL32;RPS3A;SEPP1;TPT1
Roxithromycin-312_mg/kg_in_CMC-Rat-Liver-3d-dn	6/289	0,245731481	0,448836072	0	0	1,426889737	2,002662406	EEF1B2;RPS19;RPS8;ARL6IP5;RPS20;SEPP1
Valdecoxib-404_mg/kg_in_CMC-Rat-Kidney-3d-dn	6/289	0,245731481	0,448732006	0	0	1,426889737	2,002662406	RPS19;RPS3A;RPL39;GOLGA7;SEPP1;TPT1
11-Dichloroethene-600_mg/kg_in_Water-Rat-Liver-1d-dn	6/290	0,248139524	0,453024314	0	0	1,421969428	1,981889933	CLP1;RPS8;RPS20;RPS3A;SEPP1;TPT1
Atorvastatin-2.5_mg/kg_in_Corn_Oil-Rat-Liver-3d-up	6/290	0,248139524	0,452919325	0	0	1,421969428	1,981889933	RPS19;RPS8;RPS20;RPSA;SEPP1;TPT1
Bromobenzene-439_mg/kg_in_CMC-Rat-Kidney-5d-dn	6/290	0,248139524	0,452814386	0	0	1,421969428	1,981889933	RPS17;RPS19;ARL2BP;CLP1;RPS20;RPL39
Busulfan-3_mg/kg_in_Corn_Oil-Rat-Liver-1d-dn	6/290	0,248139524	0,452709495	0	0	1,421969428	1,981889933	RPS17;RPS8;RPS6;RPS20;RPL10A;TPT1

Carboplatin-5_mg/kg_in_Saline-Rat-Kidney-5d-dn	6/290	0,248139524	0,452604652	0	0	1,421969428	1,981889933	EEF1B2;RPS17;RPS19;ARL2BP;RAPGEF5;SEPP1
Hydroquinone-800_mg/kg_in_CMC-Rat-Kidney-5d-dn	6/290	0,248139524	0,452499858	0	0	1,421969428	1,981889933	RPS17;RPL32;RPS19;RPS6;RPS20;SEPP1
Ketoconazole-114_mg/kg_in_Corn_Oil-Rat-Liver-0.25d-up	6/290	0,248139524	0,452395113	0	0	1,421969428	1,981889933	RPS19;RPS8;RPS6;RPSA;RPL10A;SEPP1
Lead_(II)_Acetate-2_mg/kg_in_Saline-Rat-Kidney-5d-dn	6/290	0,248139524	0,452290416	0	0	1,421969428	1,981889933	EEF1B2;RPS17;RPS19;ARL2BP;RPS20;RPS3A
Leflunomide-60_mg/kg_in_Corn_Oil-Rat-Kidney-5d-dn	6/290	0,248139524	0,452185768	0	0	1,421969428	1,981889933	EEF1B2;RPS17;RPL32;RPS8;RPSA;TPT1
NN'-Diphenyl-P-Phenylenediamine-1000_mg/kg_in_CMC-Rat-Liver-1d-dn	6/290	0,248139524	0,452081168	0	0	1,421969428	1,981889933	C1QA;RPS19;CLP1;RPS6;RPS20;RPS3A
NN-Dimethylformamide-700_mg/kg_in_Saline-Rat-Liver-1d-up	6/290	0,248139524	0,451976616	0	0	1,421969428	1,981889933	C1QA;CXCL11;RPS19;RPS6;RPS3A;TPT1
Nateglinide-625_mg/kg_in_CMC-Rat-Kidney-5d-up	6/290	0,248139524	0,451872113	0	0	1,421969428	1,981889933	EEF1B2;RPS17;CXCL11;RPS3A;RAPGEF5;SEPP1
Pergolide-1.1_mg/kg_in_CMC-Rat-Liver-1d-up	6/290	0,248139524	0,451767658	0	0	1,421969428	1,981889933	EEF1B2;ARL2BP;RPS8;RPSA;RPS3A;TPT1
Procarbazine-27_mg/kg_in_Water-Rat-Bone_marrow-1d-up	6/290	0,248139524	0,451663251	0	0	1,421969428	1,981889933	C1QA;NKG7;RPS3A;GOLGA7;SEPP1;TPT1
Epinephrine-0.0375_mg/kg_in_Saline-Rat-Heart-1d-up	7/350	0,249240139	0,453561769	0	0	1,374570447	1,90974355	RPS17;RPS19;RPS6;RPSA;RAPGEF5;RPL10A;SEPP1
Simvastatin-1200_mg/kg_in_Corn_Oil-Rat-Kidney-1d-up	7/350	0,249240139	0,453456996	0	0	1,374570447	1,90974355	RPS17;RPS19;RPS8;RPS5;RPS6;RPL35A;RPS20
Sulindac-23_mg/kg_in_Corn_Oil-Rat-Liver-1d-dn	7/350	0,249240139	0,453352272	0	0	1,374570447	1,90974355	RPS17;RPL32;RPS8;RPSA;RPS3A;RPL10A;SEPP1
Cytarabine-23_mg/kg_in_Saline-Rat-Bone_marrow-3d-dn	5/232	0,249742313	0,454160808	0	0	1,481218154	2,054931923	RPS17;RPL32;FEM1B;NKG7;TPT1
Alendronic_Acid-138_mg/kg_in_CMC-Rat-Heart-3d-dn	6/291	0,250554383	0,455532391	0	0	1,417082935	1,961355136	FUNDC1;RPS6;ARL6IP5;RPS20;RPL10A;SEPP1
Benzethonium_Chloride-30_mg/kg_in_Corn_Oil-Rat-Heart-3d-up	6/291	0,250554383	0,45542726	0	0	1,417082935	1,961355136	C1QA;RPS19;RPS6;RPL10A;GOLGA7;SEPP1
Carmustine-4_mg/kg_in_Corn_Oil-Rat-Bone_marrow-1d-dn	6/291	0,250554383	0,455322178	0	0	1,417082935	1,961355136	RPL32;FEM1B;RPS6;ARL6IP5;RPSA;TPT1
Chlorambucil-4.5_mg/kg_in_Corn_Oil-Rat-Liver-0.25d-dn	6/291	0,250554383	0,455217144	0	0	1,417082935	1,961355136	C1QA;RPL32;RPS8;RPS20;RPS3A;TPT1
Cisplatin-2_mg/kg_in_Saline-Rat-Liver-1d-dn	6/291	0,250554383	0,455112158	0	0	1,417082935	1,961355136	RPS19;RPS8;FEM1B;RPS20;RPS3A;TPT1
Clonazepam-2500_mg/kg_in_CMC-Rat-Liver-3d-dn	6/291	0,250554383	0,455007221	0	0	1,417082935	1,961355136	EEF1B2;RPS17;RPS19;RPS8;ARL6IP5;SEPP1
Closantel-22_mg/kg_in_CMC-Rat-Liver-1d-dn	6/291	0,250554383	0,454902332	0	0	1,417082935	1,961355136	C1QA;RPS19;CLP1;RPL35A;RPL39;SEPP1
Etodolac-24_mg/kg_in_CMC-Rat-Liver-1d-up	6/291	0,250554383	0,454797492	0	0	1,417082935	1,961355136	C1QA;RPS19;RPSA;RPS3A;RPL10A;RPL39
Lovastatin-1500_mg/kg_in_Corn_Oil-Rat-Liver-0.25d-dn	6/291	0,250554383	0,4546927	0	0	1,417082935	1,961355136	EEF1B2;RPS17;RPL35A;RPS20;RPS3A;RPL39
Methylparaben-1500_mg/kg_in_CMC-Rat-Liver-3d-dn	6/291	0,250554383	0,454587956	0	0	1,417082935	1,961355136	C1QA;RPS17;CLP1;RPS8;ARL6IP5;TPT1
Mitoxantrone-2_mg/kg_in_Saline-Rat-Heart-3d-up	6/291	0,250554383	0,454483261	0	0	1,417082935	1,961355136	RPS19;RPS8;RPS20;RAPGEF5;RPL10A;TPT1
Oxiconazole-1500_mg/kg_in_Corn_Oil-Rat-Liver-5d-up	6/291	0,250554383	0,454378613	0	0	1,417082935	1,961355136	EEF1B2;C1QA;CLP1;RPS20;SEPP1;TPT1
Oxyquinoline-68_mg/kg_in_Corn_Oil-Rat-Liver-5d-up	6/291	0,250554383	0,454274014	0	0	1,417082935	1,961355136	EEF1B2;RPL32;RPS19;RPS20;SEPP1;TPT1
Progesterone-164_mg/kg_in_Corn_Oil-Rat-Liver-0.25d-up	6/291	0,250554383	0,454169463	0	0	1,417082935	1,961355136	EEF1B2;RPS19;RPS6;RPS20;RPSA;RPL10A
Roxarsone-11_mg/kg_in_Corn_Oil-Rat-Kidney-3d-up	6/291	0,250554383	0,45406496	0	0	1,417082935	1,961355136	RPL32;RPS8;RPS6;ARL6IP5;RPSA;RPS3A
Roxithromycin-312_mg/kg_in_CMC-Rat-Kidney-5d-up	6/291	0,250554383	0,453960506	0	0	1,417082935	1,961355136	EEF1B2;CLP1;RPS8;RPS3A;RPL39;TPT1
Sildenafil-14.6_mg/kg_in_Saline-Rat-Liver-3d-up	6/291	0,250554383	0,453856099	0	0	1,417082935	1,961355136	RPS17;RPS8;RPS20;RPSA;RPS3A;RPL39
Sulconazole-40_uM_in_DMSO-Rat-Primary_rat_hepatocytes-0.67d-dn	6/291	0,250554383	0,45375174	0	0	1,417082935	1,961355136	CXCL11;CLP1;ARL6IP5;SEPP1;SLC16A3;TPT1
Tolazamide-1500_mg/kg_in_CMC-Rat-Kidney-1d-dn	6/291	0,250554383	0,453647429	0	0	1,417082935	1,961355136	RPS19;ARL2BP;FUNDC1;RPS6;RPS20;RPL39
Venlafaxine-320_mg/kg_in_Water-Rat-Liver-5d-dn	6/291	0,250554383	0,453543167	0	0	1,417082935	1,961355136	EEF1B2;C1QA;RPL32;RPS8;ARL6IP5;TPT1
Zopiclone-414_mg/kg_in_Water-Rat-Liver-5d-dn	6/291	0,250554383	0,453438952	0	0	1,417082935	1,961355136	EEF1B2;C1QA;RPS19;CLP1;SEPP1;TPT1
3-Methylcholanthrene-23_uM_in_DMSO-Rat-Primary_rat_hepatocytes-0.67d-up	7/351	0,251433956	0,45492622	0	0	1,370654292	1,892290946	RPL32;RPS8;RPS5;RPS6;RPL35A;TPT1;PDRG1
Amitriptyline-160_mg/kg_in_Water-Rat-Kidney-1d-up	7/351	0,251433956	0,454821735	0	0	1,370654292	1,892290946	EEF1B2;ARL2BP;CLP1;RPS8;RAPGEF5;SEPP1;TPT1
N-Nitrosodimethylamine-10_mg/kg_in_Saline-Rat-Liver-1d-up	7/351	0,251433956	0,454717299	0	0	1,370654292	1,892290946	RPS19;RPS8;RPSA;RPS3A;SEPP1;GOLGA7;TPT1
Nicotine-75_mg/kg_in_Water-Rat-Brain-3d-dn	7/351	0,251433956	0,45461291	0	0	1,370654292	1,892290946	ARL2BP;RPS8;RPS6;RPSA;RPL39;TOMM20;TPT1
Amoxicillin-1100_mg/kg_in_Water-Rat-Liver-5d-up	6/292	0,252975929	0,457295942	0	0	1,412229911	1,941054846	RPS19;ARL2BP;RPS8;RPS5;RPS6;RPL10A
Bezafibrate-617_mg/kg_in_Corn_Oil-Rat-Liver-3d-dn	6/292	0,252975929	0,457191009	0	0	1,412229911	1,941054846	C1QA;RPS17;CLP1;RPS3A;SEPP1;RPL39
Carbon_Tetrachloride-775_uM_in_DMSO-Rat-Primary_rat_hepatocytes-0.67d-dn	6/292	0,252975929	0,457086125	0	0	1,412229911	1,941054846	RPS17;RPS8;RPS5;ARL6IP5;RPL10A;SEPP1
Ciprofloxacin-72_mg/kg_in_Corn_Oil-Rat-Liver-5d-dn	6/292	0,252975929	0,456981288	0	0	1,412229911	1,941054846	EEF1B2;C1QA;RPS17;RPL32;RPS6;RPL10A
Cyproterone_Acetate-2500_mg/kg_in_CMC-Rat-Liver-0.25d-dn	6/292	0,252975929	0,4568765	0	0	1,412229911	1,941054846	EEF1B2;CLP1;RPS8;RPS3A;SEPP1;TPT1
Ketoconazole-25_mg/kg_in_Corn_Oil-Rat-Liver-1d-dn	6/292	0,252975929	0,45677176	0	0	1,412229911	1,941054846	RPS17;RPL32;RPSA;RPS3A;SEPP1;RPL39
Lomefloxacin-2000_mg/kg_in_CMC-Rat-Liver-1d-up	6/292	0,252975929	0,456667068	0	0	1,412229911	1,941054846	RPS17;RPS20;RPSA;SEPP1;RPL39;TPT1
Nafenopin-338_mg/kg_in_Corn_Oil-Rat-Liver-1d-up	6/292	0,252975929	0,456562424	0	0	1,412229911	1,941054846	EEF1B2;RPS19;RPS20;RPSA;RPS3A;RPL10A
Nimesulide-162_mg/kg_in_Corn_Oil-Rat-Kidney-5d-up	6/292	0,252975929	0,456457828	0	0	1,412229911	1,941054846	RPS19;RPS6;RPS20;RPL10A;RPL39;GOLGA7
Omeprazole-30_mg/kg_in_Corn_Oil-Rat-Liver-1d-up	6/292	0,252975929	0,456353279	0	0	1,412229911	1,941054846	RPS19;RPS20;RPSA;RPS3A;SEPP1;RPL39
Sildenafil-420_mg/kg_in_Water-Rat-Liver-5d-dn	6/292	0,252975929	0,456248779	0	0	1,412229911	1,941054846	C1QA;RPS17;RPL32;RPS6;RPL10A;SEPP1
Stannous_Fluoride-90_mg/kg_in_Water-Rat-Kidney-1d-dn	6/292	0,252975929	0,456144326	0	0	1,412229911	1,941054846	FEM1B;RPL35A;RPSA;SEPP1;GOLGA7;TPT1
Sulfadoxine-380_mg/kg_in_Corn_Oil-Rat-Liver-5d-dn	6/292	0,252975929	0,456039922	0	0	1,412229911	1,941054846	EEF1B2;RPS19;RPS8;RPS6;SEPP1;TPT1
Thalidomide-113_mg/kg_in_CMC-Rat-Liver-1d-dn	6/292	0,252975929	0,455935565	0	0	1,412229911	1,941054846	C1QA;CLP1;RPS6;RPS20;SEPP1;RPL39
Diethanolamine-620_mg/kg_in_Water-Rat-Kidney-5d-up	7/352	0,253633666	0,457016415	0	0	1,366760387	1,875009802	EEF1B2;RPS19;FUNDC1;RPL10A;SEPP1;RPL39;TPT1
N-Nitrosodiethylamine-7000_uM_in_DMSO-Rat-Primary_rat_hepatocytes-1d-dn	7/352	0,253633666	0,456911882	0	0	1,366760387	1,875009802	RPS19;RPS8;RPS5;RPS20;RPS3A;RPL39;TPT1
Thiamphenicol-1500_mg/kg_in_Water-Rat-Kidney-3d-dn	5/234	0,2551978	0,459624485	0	0	1,46855817	2,005633901	RPS8;RPL10A;GOLGA7;SEPP1;TPT1
33'5-Triiodo-L-Thyronine-0.1_mg/kg_in_PEG400-Rat-Heart-3d-dn	6/293	0,255404034	0,459890758	0	0	1,407410014	1,920985946	RPS17;ARL2BP;RPS8;FUNDC1;RPS6;RPS3A
Doxifluridine-1230_mg/kg_in_CMC-Rat-Bone_marrow-0.25d-dn	6/293	0,255404034	0,45978564	0	0	1,407410014	1,920985946	EEF1B2;RPS5;RPSA;RPS3A;RPL10A;SEPP1
Fluconazole-10_mg/kg_in_Corn_Oil-Rat-Liver-3d-up	6/293	0,255404034	0,45968057	0	0	1,407410014	1,920985946	EEF1B2;RPS17;RPS20;RPSA;RPS3A;SEPP1
Foscarnet-350_mg/kg_in_Saline-Rat-Bone_marrow-5d-up	6/293	0,255404034	0,459575548	0	0	1,407410014	1,920985946	RPS19;ARL2BP;RPS8;FEM1B;RPS6;RPS3A
Furan-24_mg/kg_in_Corn_Oil-Rat-Kidney-5d-up	6/293	0,255404034	0,459470574	0	0	1,407410014	1,920985946	RPS19;RPS8;RPS20;RPSA;SEPP1;RPL39
Glipizide-2500_mg/kg_in_CMC-Rat-Liver-5d-dn	6/293	0,255404034	0,459365649	0	0	1,407410014	1,920985946	EEF1B2;RPL32;RPS3A;RPL10A;SEPP1;TPT1
Hydrocortisone-56_mg/kg_in_Corn_Oil-Rat-Bone_marrow-3d-up	6/293	0,255404034	0,459260771	0	0	1,407410014	1,920985946	EEF1B2;C1QA;RPS8;RPS5;RPL35A;SLC16A3
Leflunomide-60_mg/kg_in_Corn_Oil-Rat-Bone_marrow-5d-dn	6/293	0,255404034	0,45915594	0	0	1,407410014	1,920985946	RPS17;RPS8;FEM1B;RPS6;RPS3A;TPT1
Loxoprofen-18_mg/kg_in_Corn_Oil-Rat-Kidney-1d-dn	6/293	0,255404034	0,459051158	0	0	1,407410014	1,920985946	EEF1B2;RPS17;CLP1;RPS8;RPL35A;RPS20
Melphalan-12_mg/kg_in_CMC-Rat-Spleen-5d-dn	6/293	0,255404034	0,458946424	0	0	1,407410014	1,920985946	RPS17;ARL2BP;RPS8;ARL6IP5;RPS3A;TPT1
Simvastatin-15_mg/kg_in_Corn_Oil-Rat-Liver-3d-up	6/293	0,255404034	0,458841737	0	0	1,407410014	1,920985946	EEF1B2;RPS17;RPS19;RPS6;RPS3A;RPL10A
Sotalol-6666_uM_in_DMSO-Rat-Primary_rat_hepatocytes-0.67d-up	6/293	0,255404034	0,458737098	0	0	1,407410014	1,920985946	EEF1B2;RPS17;RPS8;RPS6;RPS3A;RPL39
Tetracaine-24_mg/kg_in_Corn_Oil-Rat-Heart-1d-dn	6/293	0,255404034	0,458632507	0	0	1,407410014	1,920985946	RPS6;RPSA;RPL10A;SEPP1;RPL39;GOLGA7
Tolazamide-1500_mg/kg_in_CMC-Rat-Kidney-3d-dn	6/293	0,255404034	0,458527963	0	0	1,407410014	1,920985946	RPL32;RPS19;RPS8;FEM1B;RPS6;TPT1
Trichloroethane-3000_mg/kg_in_Water-Rat-Liver-1d-dn	6/293	0,255404034	0,458423467	0	0	1,407410014	1,920985946	RPL32;RPS8;FUNDC1;RPS20;RPS3A;TPT1
Trichloroethylene-1230_mg/kg_in_CMC-Rat-Kidney-3d-dn	6/293	0,255404034	0,458319019	0	0	1,407410014	1,920985946	RPL32;ARL2BP;RPS6;RPS20;SEPP1;TPT1
Olanzapine-250_uM_in_DMSO-Rat-Primary_rat_hepatocytes-0.67d-up	8/414	0,256841662	0,460793834	0	0	1,328087388	1,80526319	EEF1B2;RPS19;RPS6;RPL10A;SEPP1;RPL39;TPT1;PDRG1
Acetaminophen-100_mg/kg_in_Corn_Oil-Rat-Kidney-3d-dn	6/294	0,25783857	0,462477016	0	0	1,402622905	1,901145366	CLP1;RPS6;RPL10A;SEPP1;RPL39;TPT1
Azathioprine-54_mg/kg_in_Water-Rat-Bone_marrow-1d-up	6/294	0,25783857	0,462371716	0	0	1,402622905	1,901145366	C1QA;RPS19;NKG7;RPL10A;SEPP1;GOLGA7
Carbamazepine-490_mg/kg_in_CMC-Rat-Liver-1d-dn	6/294	0,25783857	0,462266464	0	0	1,402622905	1,901145366	EEF1B2;RPS17;RPL32;RPL35A;RPS20;SEPP1
Chlorambucil-4.5_mg/kg_in_Corn_Oil-Rat-Bone_marrow-3d-dn	6/294	0,25783857	0,46216126	0	0	1,402622905	1,901145366	RPS17;FEM1B;RPS6;RPSA;RPS3A;RPL10A
Chloroquine-21.95_uM_in_DMSO-Rat-Primary_rat_hepatocytes-0.67d-dn	6/294	0,25783857	0,462056104	0	0	1,402622905	1,901145366	RPS8;ARL6IP5;RPS20;RPSA;RPL10A;SEPP1
Clemastine-5.4_mg/kg_in_CMC-Rat-Heart-3d-dn	6/294	0,25783857	0,461950995	0	0	1,402622905	1,901145366	EEF1B2;ARL2BP;RPS8;RPS20;SEPP1;TPT1
Diethylstilbestrol-280_mg/kg_in_Corn_Oil-Rat-Kidney-3d-dn	6/294	0,25783857	0,461845935	0	0	1,402622905	1,901145366	EEF1B2;RPS17;RPL32;CLP1;RPSA;SEPP1
Ferulic_Acid-1500_mg/kg_in_CMC-Rat-Liver-3d-dn	6/294	0,25783857	0,461740922	0	0	1,402622905	1,901145366	C1QA;CLP1;RPS8;RPS3A;RPL39;TPT1
Lead(IV)_Acetate-200_mg/kg_in_Saline-Rat-Kidney-3d-dn	6/294	0,25783857	0,461635957	0	0	1,402622905	1,901145366	EEF1B2;RPL32;RPS6;RPSA;RPL39;TPT1
Nimesulide-30_mg/kg_in_Corn_Oil-Rat-Liver-3d-dn	6/294	0,25783857	0,46153104	0	0	1,402622905	1,901145366	RPS17;CLP1;RPS3A;RPL10A;RPL39;TPT1
Prednisolone-184_mg/kg_in_Water-Rat-Liver-5d-dn	6/294	0,25783857	0,46142617	0	0	1,402622905	1,901145366	EEF1B2;C1QA;RPS19;RPS8;RPS6;RPS3A
Prednisone-68_mg/kg_in_CMC-Rat-Heart-1d-up	6/294	0,25783857	0,461321348	0	0	1,402622905	1,901145366	RPS17;RPL32;FUNDC1;RPS6;ARL6IP5;RPS20
Primidone-750_mg/kg_in_CMC-Rat-Liver-1d-up	6/294	0,25783857	0,461216574	0	0	1,402622905	1,901145366	C1QA;RPSA;RPS3A;RPL10A;RPL39;TPT1
Rifabutin-1500_mg/kg_in_CMC-Rat-Liver-5d-up	6/294	0,25783857	0,461111847	0	0	1,402622905	1,901145366	RPS8;RPS20;RPSA;RPL10A;SEPP1;TPT1
Safrole-488_mg/kg_in_Corn_Oil-Rat-Liver-0.25d-up	6/294	0,25783857	0,461007168	0	0	1,402622905	1,901145366	RPS19;RPS8;RPS6;ARL6IP5;RPSA;SEPP1
Sertraline-210_mg/kg_in_Corn_Oil-Rat-Liver-1d-up	6/294	0,25783857	0,460902536	0	0	1,402622905	1,901145366	RPS17;RPL32;RPS8;RPS6;RPS20;RPS3A
Sildenafil-90_uM_in_DMSO-Rat-Primary_rat_hepatocytes-0.67d-dn	6/294	0,25783857	0,460797952	0	0	1,402622905	1,901145366	CXCL11;RPS8;ARL6IP5;RPSA;SEPP1;SLC16A3

Tazobactam-1500_mg/kg_in_CMC-Rat-Spleen-3d-up	6/294	0,25783857	0,460693415	0	0	1,402622905	1,901145366	RPS19;RPS8;RPS5;RPSA;RPL10A;SEPP1
Altretamine-40_mg/kg_in_Corn_Oil-Rat-Spleen-3d-dn	7/354	0,258050385	0,460967302	0	0	1,359038577	1,840954229	C1QA;RPS19;RPS20;RPSA;RPL10A;SEPP1;RPL39
Levofloxacin-739_mg/kg_in_Water-Rat-Kidney-5d-up	8/415	0,258881773	0,462347584	0	0	1,324887178	1,790431063	EEF1B2;CXCL11;RPS8;RPS3A;RAPGEF5;SEPP1;GOLGA7;TPT1
Diclofenac-3.5_mg/kg_in_Corn_Oil-Rat-Kidney-0.25d-up	7/355	0,260267205	0,464716506	0	0	1,3552103	1,824176031	RPS8;FUNDC1;RPS6;RPS20;RAPGEF5;RPL10A;TPT1
Lead_(II)_Acetate-200_mg/kg_in_Saline-Rat-Bone_marrow-3d-up	7/355	0,260267205	0,464611176	0	0	1,3552103	1,824176031	RPS17;RPS8;RPS5;RPL35A;RPS20;RPSA;TPT1
Acetazolamide-250_mg/kg_in_Corn_Oil-Rat-Liver-5d-up	6/295	0,260279406	0,464527669	0	0	1,397868251	1,881530086	RPS19;RPS8;FEM1B;RPS20;RPSA;TPT1
Amprenavir-600_mg/kg_in_CMC-Rat-Kidney-1d-dn	6/295	0,260279406	0,464422429	0	0	1,397868251	1,881530086	RPS20;RPSA;SEPP1;RPL39;GOLGA7;TPT1
Beta-Naphthoflavone-80_mg/kg_in_CMC-Rat-Liver-5d-up	6/295	0,260279406	0,464317238	0	0	1,397868251	1,881530086	EEF1B2;RPS17;RPS8;RPSA;RPL10A;SEPP1
Carbimazole-400_mg/kg_in_Water-Rat-Liver-1d-dn	6/295	0,260279406	0,464212093	0	0	1,397868251	1,881530086	EEF1B2;RPL32;RPS8;RPS20;RPS3A;TPT1
Catechol-40_mg/kg_in_Saline-Rat-Liver-0.25d-dn	6/295	0,260279406	0,464106997	0	0	1,397868251	1,881530086	ARL2BP;CLP1;RPS8;RPS6;RPS20;RPSA
Dexibuprofen-239_mg/kg_in_CMC-Rat-Kidney-3d-up	6/295	0,260279406	0,464001947	0	0	1,397868251	1,881530086	C1QA;RPS17;RPS8;RPL35A;RPS3A;TPT1
Diclofenac-10_mg/kg_in_Corn_Oil-Rat-Liver-5d-dn	6/295	0,260279406	0,463896946	0	0	1,397868251	1,881530086	RPS17;RPL32;RPS19;RPS8;RPL35A;SEPP1
Doxorubicin-3_mg/kg_in_Saline-Rat-Spleen-3d-dn	6/295	0,260279406	0,463791992	0	0	1,397868251	1,881530086	EEF1B2;C1QA;RPL32;RPS6;ARL6IP5;RPL39
Ethacrynic_Acid-125_mg/kg_in_CMC-Rat-Kidney-5d-dn	6/295	0,260279406	0,463687085	0	0	1,397868251	1,881530086	RPS17;FEM1B;RPS6;RPS20;RPS3A;SEPP1
Fenofibrate-43_mg/kg_in_Corn_Oil-Rat-Liver-5d-dn	6/295	0,260279406	0,463582226	0	0	1,397868251	1,881530086	EEF1B2;C1QA;CLP1;RPS6;RPSA;SEPP1
Gentamicin-267_mg/kg_in_Corn_Oil-Rat-Liver-1d-up	6/295	0,260279406	0,463477414	0	0	1,397868251	1,881530086	C1QA;RPS19;RPS6;RPL10A;SEPP1;TPT1
Hydroxyurea-400_mg/kg_in_Saline-Rat-Spleen-3d-up	6/295	0,260279406	0,46337265	0	0	1,397868251	1,881530086	CXCL11;RPS19;RPL10A;SEPP1;RPL39;GOLGA7
Ifosfamide-143_mg/kg_in_Saline-Rat-Spleen-1d-up	6/295	0,260279406	0,463267933	0	0	1,397868251	1,881530086	C1QA;RPS19;RPS6;RPS20;RPL10A;SEPP1
Leflunomide-30_mg/kg_in_Corn_Oil-Rat-Bone_marrow-0.25d-up	6/295	0,260279406	0,463163263	0	0	1,397868251	1,881530086	EEF1B2;C1QA;CLP1;NKG7;RPL10A;RPL39
Melphalan-12_mg/kg_in_CMC-Rat-Spleen-3d-dn	6/295	0,260279406	0,463058641	0	0	1,397868251	1,881530086	C1QA;RPS6;NKG7;RPS20;RPS3A;RPL39
Metoprolol-1200_mg/kg_in_Water-Rat-Heart-3d-up	6/295	0,260279406	0,462954066	0	0	1,397868251	1,881530086	FUNDC1;RPS6;ARL6IP5;RPSA;RPS3A;RPL10A
Oxaliplatin-1.31_mg/kg_in_Saline-Rat-Bone_marrow-0.25d-up	6/295	0,260279406	0,462849538	0	0	1,397868251	1,881530086	RPL32;RPS19;FEM1B;RPS6;RPSA;TPT1
Phenothiazine-36_mg/kg_in_Corn_Oil-Rat-Liver-0.25d-up	6/295	0,260279406	0,462745057	0	0	1,397868251	1,881530086	RPS8;RPS6;RPS20;RPSA;RAPGEF5;PDRG1
Promethazine-2.3_mg/kg_in_Saline-Rat-Liver-5d-dn	6/295	0,260279406	0,462640624	0	0	1,397868251	1,881530086	C1QA;RPS19;CLP1;RPS8;SEPP1;PDRG1
Rapamycin-60_mg/kg_in_CMC-Rat-Kidney-1d-up	6/295	0,260279406	0,462536237	0	0	1,397868251	1,881530086	RPS19;ARL2BP;RPS6;RPS20;SEPP1;GOLGA7
Sodium_Arsenite-30_mg/kg_in_Saline-Rat-Kidney-3d-dn	6/295	0,260279406	0,462431898	0	0	1,397868251	1,881530086	EEF1B2;RPL32;RPS6;RPSA;RPL10A;SEPP1
Sulfadoxine-380_mg/kg_in_Corn_Oil-Rat-Liver-1d-up	6/295	0,260279406	0,462327606	0	0	1,397868251	1,881530086	RPS19;RPS6;RPL35A;RPS20;RPL10A;RPL39
TNF-alpha-0.25_mg/kg_in_RSA/Saline-Rat-Liver-0.25d-dn	6/295	0,260279406	0,46222336	0	0	1,397868251	1,881530086	C1QA;RPS17;RPL32;RPS19;RPSA;SEPP1
Trimethadione-535_mg/kg_in_Water-Rat-Heart-5d-up	6/295	0,260279406	0,462119162	0	0	1,397868251	1,881530086	EEF1B2;RPS17;RPL32;RPS3A;RPL10A;SEPP1
Valproic_Acid-850_mg/kg_in_Water-Rat-Liver-0.25d-dn	6/295	0,260279406	0,462015011	0	0	1,397868251	1,881530086	EEF1B2;RPS8;RPL35A;RPSA;SEPP1;TPT1
Valsartan-1000_mg/kg_in_Corn_Oil-Rat-Kidney-5d-dn	6/295	0,260279406	0,461910907	0	0	1,397868251	1,881530086	RPS17;RPL32;RPSA;RPS3A;SEPP1;TPT1
Vincristine-0.05_mg/kg_in_Saline-Rat-Bone_marrow-1d-dn	6/295	0,260279406	0,461806849	0	0	1,397868251	1,881530086	RPL32;RPS19;RPS20;RPSA;RPL10A;RPL39
Aspirin-35_mg/kg_in_Corn_Oil-Rat-Liver-5d-up	5/236	0,260682463	0,462417811	0	0	1,456112761	1,957674046	RPS8;RPS20;RPSA;GOLGA7;SEPP1
Carboplatin-14_mg/kg_in_Saline-Rat-Bone_marrow-5d-dn	7/356	0,262489538	0,465518487	0	0	1,351403529	1,807561754	EEF1B2;RPS17;FEM1B;RPS3A;RPL10A;RPL39;DUSP14
Antimycin_A-0.03_uM_in_DMSO-Rat-Primary_rat_hepatocytes-0.67d-up	6/296	0,262726415	0,465833688	0	0	1,393145723	1,862137135	RPS19;RPS8;RPS5;RPS20;RPSA;RPL10A
Aspirin-35_mg/kg_in_Corn_Oil-Rat-Kidney-3d-dn	6/296	0,262726415	0,465728842	0	0	1,393145723	1,862137135	CLP1;FEM1B;RPS6;RPL10A;SEPP1;TPT1
Azasetron-580_mg/kg_in_CMC-Rat-Intestine-5d-up	6/296	0,262726415	0,465624042	0	0	1,393145723	1,862137135	RPS17;RPL32;RPS8;RPS20;RPSA;TPT1
Azathioprine-0.5_uM_in_DMSO-Rat-Primary_rat_hepatocytes-0.67d-dn	6/296	0,262726415	0,46551929	0	0	1,393145723	1,862137135	ARL2BP;CLP1;RPS8;RPS20;RPSA;RPL10A
Bisphenol_A-610_mg/kg_in_Corn_Oil-Rat-Spleen-1d-dn	6/296	0,262726415	0,465414585	0	0	1,393145723	1,862137135	EEF1B2;RPS19;RPL35A;RPS20;RPL10A;RPL39
Bleomycin_A2-12_mg/kg_in_Saline-Rat-Bone_marrow-3d-dn	6/296	0,262726415	0,465309927	0	0	1,393145723	1,862137135	C1QA;RPS17;RPS19;FEM1B;RPS3A;GOLGA7
Chlorobenzene-500_mg/kg_in_CMC-Rat-Heart-1d-up	6/296	0,262726415	0,465205316	0	0	1,393145723	1,862137135	EEF1B2;ARL2BP;RPS8;RPS20;RPS3A;SLC16A3
Famciclovir-1200_mg/kg_in_Saline-Rat-Spleen-5d-up	6/296	0,262726415	0,465100752	0	0	1,393145723	1,862137135	RPS19;FUNDC1;RPS6;RPSA;SEPP1;RPL39
Ferulic_Acid-1500_mg/kg_in_CMC-Rat-Liver-5d-dn	6/296	0,262726415	0,464996235	0	0	1,393145723	1,862137135	EEF1B2;RPS19;CLP1;RPS8;RPS3A;RPL39
Fluocinolone_Acetonide-2.5_mg/kg_in_Corn_Oil-Rat-Heart-1d-dn	6/296	0,262726415	0,464891764	0	0	1,393145723	1,862137135	RPS19;ARL2BP;RPS8;FUNDC1;RPS20;RPSA
Fluphenazine-22_mg/kg_in_Corn_Oil-Rat-Liver-5d-up	6/296	0,262726415	0,464787341	0	0	1,393145723	1,862137135	EEF1B2;RPL32;RPS19;RPS20;RPSA;RPS3A
Leflunomide-60_mg/kg_in_Corn_Oil-Rat-Liver-5d-dn	6/296	0,262726415	0,464682965	0	0	1,393145723	1,862137135	RPS17;RPS19;RPS8;RPS6;RPL10A;SEPP1
Lorazepam-2000_mg/kg_in_CMC-Rat-Liver-1d-up	6/296	0,262726415	0,464578636	0	0	1,393145723	1,862137135	RPL32;RPS19;FEM1B;RPS6;RPS3A;TPT1
N-Nitrosodiethylamine-1.67_mg/kg_in_Saline-Rat-Bone_marrow-0.25d-dn	6/296	0,262726415	0,464474353	0	0	1,393145723	1,862137135	EEF1B2;RPL32;RPS3A;RPL10A;RPL39;TPT1
Neomycin-877_mg/kg_in_Corn_Oil-Rat-Liver-0.25d-dn	6/296	0,262726415	0,464370118	0	0	1,393145723	1,862137135	EEF1B2;RPL32;RPSA;SEPP1;RPL39;TPT1
Penciclovir-1200_mg/kg_in_Corn_Oil-Rat-Bone_marrow-3d-dn	6/296	0,262726415	0,464265929	0	0	1,393145723	1,862137135	RPS17;RPS19;RPSA;RPL10A;SEPP1;TPT1
Salicylamide-1300_mg/kg_in_CMC-Rat-Liver-3d-dn	6/296	0,262726415	0,464161786	0	0	1,393145723	1,862137135	EEF1B2;RPL32;RPL35A;RPS20;SEPP1;TPT1
Sulconazole-1200_mg/kg_in_Corn_Oil-Rat-Liver-3d-up	6/296	0,262726415	0,464057691	0	0	1,393145723	1,862137135	EEF1B2;C1QA;RPL32;RPL35A;RPS20;SEPP1
Sulfanilamide-636_mg/kg_in_Corn_Oil-Rat-Kidney-3d-dn	6/296	0,262726415	0,463953642	0	0	1,393145723	1,862137135	EEF1B2;CLP1;RPS20;SEPP1;RPL39;TPT1
Tretinoin-7_mg/kg_in_Corn_Oil-Rat-Kidney-5d-up	6/296	0,262726415	0,46384964	0	0	1,393145723	1,862137135	ARL2BP;RPS5;ARL6IP5;RPSA;SEPP1;TPT1
Ifosfamide-11000_uM_in_DMSO-Rat-Primary_rat_hepatocytes-1d-up	5/237	0,263435272	0,464996908	0	0	1,449968826	1,934182418	EEF1B2;RPSA;RPS3A;SEPP1;PDRG1
Lead_(II)_Acetate-200_mg/kg_in_Saline-Rat-Liver-5d-dn	7/357	0,264717288	0,467155133	0	0	1,347618085	1,791109576	EEF1B2;RPS19;RPS20;RPL10A;SEPP1;RPL39;TPT1
Amitriptyline-20_mg/kg_in_Water-Rat-Heart-3d-up	6/297	0,265179466	0,467865922	0	0	1,388454997	1,842963588	FUNDC1;RPS6;RPSA;RAPGEF5;GOLGA7;TPT1
Aspirin-35_mg/kg_in_Corn_Oil-Rat-Heart-5d-dn	6/297	0,265179466	0,467761137	0	0	1,388454997	1,842963588	RPS17;RPL32;RPS19;RPS6;RPS3A;SEPP1
Chlorpromazine-43.9_uM_in_DMSO-Rat-Primary_rat_hepatocytes-0.67d-dn	6/297	0,265179466	0,467656399	0	0	1,388454997	1,842963588	RPS17;CXCL11;RPL32;ARL6IP5;SEPP1;TPT1
Clomipramine-115_mg/kg_in_Water-Rat-Liver-5d-dn	6/297	0,265179466	0,467551708	0	0	1,388454997	1,842963588	EEF1B2;RPS17;RPL32;RPS20;SEPP1;RPL39
Danazol-2000_mg/kg_in_CMC-Rat-Liver-5d-dn	6/297	0,265179466	0,467447063	0	0	1,388454997	1,842963588	EEF1B2;C1QA;RPS8;RPS6;SEPP1;TPT1
Eperisone-501_mg/kg_in_CMC-Rat-Liver-3d-dn	6/297	0,265179466	0,467342465	0	0	1,388454997	1,842963588	C1QA;RPS19;CLP1;SEPP1;RPL39;TPT1
Esmolol-27_mg/kg_in_Saline-Rat-Heart-5d-up	6/297	0,265179466	0,467237915	0	0	1,388454997	1,842963588	RPS17;RPS19;RPS6;RPL39;GOLGA7;TPT1
Etoposide-188_mg/kg_in_Corn_Oil-Rat-Bone_marrow-5d-dn	6/297	0,265179466	0,46713341	0	0	1,388454997	1,842963588	RPS19;RPS6;RPSA;RPS3A;RPL10A;TPT1
Fluphenazine-45_uM_in_DMSO-Rat-Primary_rat_hepatocytes-0.67d-up	6/297	0,265179466	0,467028953	0	0	1,388454997	1,842963588	EEF1B2;RPS17;RPS3A;RPL10A;RPL39;PDRG1
Hydrocortisone-56_mg/kg_in_Corn_Oil-Rat-Liver-1d-dn	6/297	0,265179466	0,466924542	0	0	1,388454997	1,842963588	RPS17;RPS8;RPSA;RPL10A;RPL39;TPT1
Interleukin_6-0.125_mg/kg_in_RSA/Saline-Rat-Liver-0.25d-dn	6/297	0,265179466	0,466820178	0	0	1,388454997	1,842963588	RPS17;RPS19;CLP1;RPSA;SEPP1;RPL39
Levofloxacin-739_mg/kg_in_Water-Rat-Heart-3d-up	6/297	0,265179466	0,466715861	0	0	1,388454997	1,842963588	ARL2BP;RPS6;RPSA;RPS3A;RAPGEF5;GOLGA7
Norethindrone-125_uM_in_DMSO-Rat-Primary_rat_hepatocytes-0.67d-dn	6/297	0,265179466	0,46661159	0	0	1,388454997	1,842963588	CXCL11;RPS8;RPS3A;SEPP1;SLC16A3;TPT1
Oxaliplatin-8_mg/kg_in_Saline-Rat-Kidney-3d-dn	6/297	0,265179466	0,466507366	0	0	1,388454997	1,842963588	RPS6;RPS20;RPSA;RPS3A;RPL10A;RPL39
Prednisone-68_mg/kg_in_CMC-Rat-Kidney-1d-up	6/297	0,265179466	0,466403188	0	0	1,388454997	1,842963588	C1QA;CXCL11;RPS6;RPS20;RPL39;TPT1
Propylthiouracil-625_mg/kg_in_CMC-Rat-Liver-1d-dn	6/297	0,265179466	0,466299057	0	0	1,388454997	1,842963588	EEF1B2;C1QA;RPS17;RPL32;SEPP1;TPT1
Quetiapine-500_mg/kg_in_CMC-Rat-Liver-5d-up	6/297	0,265179466	0,466194973	0	0	1,388454997	1,842963588	RPS6;RPS20;RPSA;SEPP1;RPL39;GOLGA7
Rosiglitazone-10_mg/kg_in_Corn_Oil-Rat-Liver-5d-dn	6/297	0,265179466	0,466090935	0	0	1,388454997	1,842963588	C1QA;RPL32;RPS6;RPS3A;SEPP1;RPL39
Simvastatin-1200_mg/kg_in_Corn_Oil-Rat-Liver-0.25d-up	6/297	0,265179466	0,465986943	0	0	1,388454997	1,842963588	EEF1B2;RPS17;RPL32;RPS6;RPS3A;RPL10A
Doxorubicin-0.65_mg/kg_in_Saline-Rat-Liver-1d-dn	5/238	0,266194818	0,467666827	0	0	1,44387652	1,911009327	RPS19;CLP1;RPS6;RPSA;RPS3A
2-Nitroanisole-740_mg/kg_in_CMC-Rat-Kidney-1d-up	6/298	0,267638432	0,470098191	0	0	1,383795752	1,824006565	RPS17;RPS8;RPS3A;RAPGEF5;TOMM20;TPT1
4-Nonylphenol-200_mg/kg_in_Corn_Oil-Rat-Kidney-1d-up	6/298	0,267638432	0,469993376	0	0	1,383795752	1,824006565	RPS19;ARL2BP;RPSA;SEPP1;GOLGA7;TPT1
Acetazolamide-47_mg/kg_in_Corn_Oil-Rat-Liver-0.25d-up	6/298	0,267638432	0,469888607	0	0	1,383795752	1,824006565	RPS19;RPS8;FEM1B;RPS6;RPSA;RPL10A
Aldosterone-0.065_mg/kg_in_Saline-Rat-Heart-1d-up	6/298	0,267638432	0,469783884	0	0	1,383795752	1,824006565	ARL2BP;RPS8;FUNDC1;ARL6IP5;RAPGEF5;TPT1
Bithionol-59_mg/kg_in_Corn_Oil-Rat-Kidney-3d-dn	6/298	0,267638432	0,469679209	0	0	1,383795752	1,824006565	CLP1;RPS6;RPL10A;SEPP1;RPL39;TPT1
Candesartan-1300_mg/kg_in_CMC-Rat-Heart-3d-up	6/298	0,267638432	0,46957458	0	0	1,383795752	1,824006565	EEF1B2;ARL2BP;CLP1;RPS8;RPS3A;RPL39
Chlorobenzene-500_mg/kg_in_CMC-Rat-Kidney-3d-dn	6/298	0,267638432	0,469469998	0	0	1,383795752	1,824006565	RPS17;RPS19;ARL2BP;RPS6;RPL39;TPT1
Colchicine-0.6_mg/kg_in_Saline-Rat-Kidney-3d-up	6/298	0,267638432	0,469365462	0	0	1,383795752	1,824006565	RPL32;RPS8;RPS6;RPS20;RPS3A;SEPP1
Finasteride-25_mg/kg_in_Corn_Oil-Rat-Liver-3d-up	6/298	0,267638432	0,469260973	0	0	1,383795752	1,824006565	C1QA;RPS19;RPS20;SEPP1;RPL39;GOLGA7
Finasteride-800_mg/kg_in_Corn_Oil-Rat-Kidney-3d-up	6/298	0,267638432	0,46915653	0	0	1,383795752	1,824006565	ARL2BP;RPS8;FUNDC1;RPS20;RPSA;RAPGEF5
Indomethacin-12_mg/kg_in_Water-Rat-Liver-0.25d-dn	6/298	0,267638432	0,469052134	0	0	1,383795752	1,824006565	RPS17;RPL32;RPL35A;RPS3A;RPL39;TPT1
Lead(IV)_Acetate-200_mg/kg_in_Saline-Rat-Bone_marrow-0.25d-dn	6/298	0,267638432	0,468947784	0	0	1,383795752	1,824006565	EEF1B2;RPS6;NKG7;RPS3A;SEPP1;RPL39
Metronidazole-50_mg/kg_in_Water-Rat-Liver-0.25d-dn	6/298	0,267638432	0,468843481	0	0	1,383795752	1,824006565	EEF1B2;RPS17;RPL32;RPS19;RPL35A;RPL39
Sulfadoxine-12_mg/kg_in_Corn_Oil-Rat-Liver-3d-dn	6/298	0,267638432	0,468739224	0	0	1,383795752	1,824006565	C1QA;RPS6;RPSA;RPL10A;SEPP1;TPT1

Troxipide-1200_mg/kg_in_CMC-Rat-Heart-1d-up	6/298	0,267638432	0,468635013	0	0	1,383795752	1,824006565	EEF1B2;FUNDC1;RPS20;RAPGEF5;RPL10A;SEPP1
Allyl_Alcohol-25_mg/kg_in_Saline-Rat-Liver-1d-dn	5/239	0,268960916	0,470846005	0	0	1,437835195	1,888149653	RPL32;RPS19;RPSA;RPL10A;SEPP1
Netilmicin-41_mg/kg_in_Saline-Rat-Kidney-5d-dn	5/239	0,268960916	0,470741373	0	0	1,437835195	1,888149653	EEF1B2;ARL2BP;RPS20;RPL10A;RPL39
Doxycycline-1000_mg/kg_in_Water-Rat-Liver-3d-up	7/359	0,269188657	0,471035295	0	0	1,340110463	1,758684341	EEF1B2;C1QA;RPL32;RPS6;RPSA;RPS3A;RAPGEF5
Benzothiazyl_Disulfide-2600_mg/kg_in_Corn_Oil-Rat-Liver-5d-up	6/299	0,270103182	0,472530578	0	0	1,379167672	1,805263231	C1QA;ARL2BP;RPS6;RPSA;RPL10A;RPL39
Bithionol-59_mg/kg_in_Corn_Oil-Rat-Bone_marrow-0.25d-up	6/299	0,270103182	0,472425641	0	0	1,379167672	1,805263231	EEF1B2;C1QA;RPS17;RPL35A;RPS3A;RPL39
Chloroform-600_mg/kg_in_Water-Rat-Liver-5d-up	6/299	0,270103182	0,472320751	0	0	1,379167672	1,805263231	C1QA;RPS19;RPS8;RPS5;ARL6IP5;RPSA
Cytarabine-23_mg/kg_in_Saline-Rat-Spleen-3d-up	6/299	0,270103182	0,472215907	0	0	1,379167672	1,805263231	CXCL11;RPS19;RPS20;RPL10A;SEPP1;GOLGA7
Emetine-1_mg/kg_in_Saline-Rat-Kidney-5d-dn	6/299	0,270103182	0,47211111	0	0	1,379167672	1,805263231	RPL32;RPS19;FUNDC1;RPS6;RPS20;RPL39
Ethambutol-998_mg/kg_in_CMC-Rat-Kidney-3d-up	6/299	0,270103182	0,472006359	0	0	1,379167672	1,805263231	RPS17;ARL6IP5;RPL13A;RPL35A;RPS3A;RPL39
Ethylestrenol-390_mg/kg_in_CMC-Rat-Liver-3d-up	6/299	0,270103182	0,471901655	0	0	1,379167672	1,805263231	EEF1B2;RPS19;RPS20;RPSA;RPL10A;RPL39
Etoposide-125_uM_in_DMSO-Rat-Primary_rat_hepatocytes-0.67d-up	6/299	0,270103182	0,471796997	0	0	1,379167672	1,805263231	EEF1B2;CXCL11;RPS19;ARL6IP5;SEPP1;GOLGA7
Glipizide-2500_mg/kg_in_CMC-Rat-Liver-3d-up	6/299	0,270103182	0,471692386	0	0	1,379167672	1,805263231	RPL32;RPS19;RPS6;RPSA;RPL10A;RPL39
Lead(IV)_Acetate-600_mg/kg_in_Saline-Rat-Bone_marrow-5d-dn	6/299	0,270103182	0,471587821	0	0	1,379167672	1,805263231	EEF1B2;NKG7;RPL10A;SEPP1;RPL39;DUSP14
Lisinopril-2000_mg/kg_in_CMC-Rat-Kidney-3d-up	6/299	0,270103182	0,471483303	0	0	1,379167672	1,805263231	RPS17;ARL6IP5;RPS20;RPSA;RPL39;TPT1
Niacin-2625_mg/kg_in_Water-Rat-Liver-3d-dn	6/299	0,270103182	0,47137883	0	0	1,379167672	1,805263231	RPS19;CLP1;FEM1B;RPS20;SEPP1;RPL39
Nimetazepam-122_mg/kg_in_CMC-Rat-Liver-1d-up	6/299	0,270103182	0,471274404	0	0	1,379167672	1,805263231	RPS19;RPS6;RPSA;RPS3A;RPL10A;TPT1
Safrole-488_mg/kg_in_Corn_Oil-Rat-Liver-1d-up	6/299	0,270103182	0,471170025	0	0	1,379167672	1,805263231	RPS17;RPS8;FEM1B;RPS20;RPSA;RPS3A
Sisomicin-34_mg/kg_in_Saline-Rat-Kidney-5d-dn	6/299	0,270103182	0,471065691	0	0	1,379167672	1,805263231	EEF1B2;ARL2BP;RPS3A;RPL10A;RPL39;TPT1
Sodium_Arsenite-10_mg/kg_in_Saline-Rat-Bone_marrow-0.25d-dn	6/299	0,270103182	0,470961404	0	0	1,379167672	1,805263231	RPS17;NKG7;RPS3A;RPL10A;SEPP1;TPT1
Sulfamethoxazole-1000_mg/kg_in_Water-Rat-Kidney-1d-dn	6/299	0,270103182	0,470857163	0	0	1,379167672	1,805263231	EEF1B2;RPL32;ARL6IP5;RPL35A;RPS20;RPL39
Temafloxacin-1000_mg/kg_in_CMC-Rat-Liver-3d-up	6/299	0,270103182	0,470752968	0	0	1,379167672	1,805263231	RPS8;FEM1B;RPS6;RPL10A;RPL39;TPT1
Bithionol-333_mg/kg_in_Corn_Oil-Rat-Liver-5d-up	7/360	0,271432085	0,472964402	0	0	1,336387934	1,742707759	C1QA;RPS17;RPL32;RPS20;SEPP1;RPL39;TPT1
Bithionol-333_mg/kg_in_Corn_Oil-Rat-Liver-5d-dn	5/240	0,271733382	0,473384676	0	0	1,431844215	1,865598376	RPS19;RPS8;RPS6;RPSA;RPL10A
Lamivudine-35_mg/kg_in_Water-Rat-Liver-0.25d-dn	5/240	0,271733382	0,473279991	0	0	1,431844215	1,865598376	RPS19;RPS8;RPL10A;RPL39;TPT1
1-Naphthyl_Isothiocyanate-60_mg/kg_in_Corn_Oil-Rat-Kidney-3d-dn	6/300	0,272573587	0,47463842	0	0	1,374570447	1,786730798	RPS19;ARL2BP;CLP1;SEPP1;RPL39;TPT1
Acyclovir-330_mg/kg_in_Saline-Rat-Liver-5d-up	6/300	0,272573587	0,474533505	0	0	1,374570447	1,786730798	RPS19;RPS8;RPS20;SEPP1;GOLGA7;TPT1
Aflatoxin_B1-0.3_mg/kg_in_CMC-Rat-Liver-5d-up	6/300	0,272573587	0,474428635	0	0	1,374570447	1,786730798	EEF1B2;RPS17;RPS19;SEPP1;RPL39;TPT1
Azlocillin-800_mg/kg_in_Saline-Rat-Kidney-0.25d-dn	6/300	0,272573587	0,474323812	0	0	1,374570447	1,786730798	RPL32;RPS19;RPS6;RPS20;RPL10A;RPL39
Benzethonium_Chloride-30_mg/kg_in_Corn_Oil-Rat-Liver-3d-up	6/300	0,272573587	0,474219036	0	0	1,374570447	1,786730798	EEF1B2;C1QA;RPS17;RPS3A;SEPP1;GOLGA7
Cadmium_Chloride-2_mg/kg_in_Saline-Rat-Kidney-5d-dn	6/300	0,272573587	0,474114305	0	0	1,374570447	1,786730798	EEF1B2;RPS17;RPL32;RPS19;ARL2BP;SEPP1
Cisplatin-2_mg/kg_in_Saline-Rat-Bone_marrow-1d-dn	6/300	0,272573587	0,474009621	0	0	1,374570447	1,786730798	RPS17;RPS6;RPS3A;RPL10A;SEPP1;RPL39
Cisplatin-2_mg/kg_in_Saline-Rat-Bone_marrow-1d-up	6/300	0,272573587	0,473904983	0	0	1,374570447	1,786730798	RPS8;FEM1B;FUNDC1;RPL35A;RPS20;TPT1
Clomiphene-250_mg/kg_in_CMC-Rat-Liver-5d-dn	6/300	0,272573587	0,473800392	0	0	1,374570447	1,786730798	EEF1B2;RPS19;RPS8;RPS20;SEPP1;TPT1
Cyproterone_Acetate-2500_mg/kg_in_CMC-Rat-Liver-1d-up	6/300	0,272573587	0,473695846	0	0	1,374570447	1,786730798	RPS19;RPSA;RPS3A;RPL10A;RPL39;TPT1
Daunorubicin-1.5_uM_in_DMSO-Rat-Primary_rat_hepatocytes-0.67d-dn	6/300	0,272573587	0,473591347	0	0	1,374570447	1,786730798	RPS19;RPS20;RPSA;RPL10A;RPL39;TPT1
Etoposide-188_mg/kg_in_Corn_Oil-Rat-Bone_marrow-1d-dn	6/300	0,272573587	0,473486893	0	0	1,374570447	1,786730798	RPS8;FEM1B;RPS6;ARL6IP5;RPSA;TPT1
Glimepiride-2500_mg/kg_in_CMC-Rat-Liver-3d-dn	6/300	0,272573587	0,473382486	0	0	1,374570447	1,786730798	EEF1B2;RPL32;RPS8;RPS5;ARL6IP5;SEPP1
Isoeugenol-1560_mg/kg_in_CMC-Rat-Kidney-5d-dn	6/300	0,272573587	0,473278125	0	0	1,374570447	1,786730798	RPS19;RPS6;RPS20;RPSA;RPL10A;RPL39
Lithocholic_Acid-74_uM_in_DMSO-Rat-Primary_rat_hepatocytes-0.67d-up	6/300	0,272573587	0,47317381	0	0	1,374570447	1,786730798	ARL2BP;CLP1;RPS6;ARL6IP5;RPL10A;PDRG1
Lomustine-4.2_mg/kg_in_Corn_Oil-Rat-Bone_marrow-1d-up	6/300	0,272573587	0,47306954	0	0	1,374570447	1,786730798	EEF1B2;C1QA;RPS17;RPS3A;RPL39;TPT1
Minoxidil-83_mg/kg_in_CMC-Rat-Heart-3d-dn	6/300	0,272573587	0,472965317	0	0	1,374570447	1,786730798	EEF1B2;FUNDC1;RPL10A;SEPP1;TOMM20;TPT1
Mosapride-30_mg/kg_in_CMC-Rat-Intestine-5d-up	6/300	0,272573587	0,47286114	0	0	1,374570447	1,786730798	RPL32;RPS19;RPS20;RPSA;RPL10A;TPT1
NN-Dimethylformamide-140_mg/kg_in_Saline-Rat-Spleen-1d-up	6/300	0,272573587	0,472757008	0	0	1,374570447	1,786730798	C1QA;CXCL11;RPS19;RPL10A;SEPP1;RPL39
Nisoldipine-1125_mg/kg_in_Water-Rat-Liver-1d-dn	6/300	0,272573587	0,472652923	0	0	1,374570447	1,786730798	EEF1B2;RPL32;RPL10A;SEPP1;RPL39;TPT1
Nisoldipine-1125_mg/kg_in_Water-Rat-Liver-1d-up	6/300	0,272573587	0,472548883	0	0	1,374570447	1,786730798	RPS17;RPS8;RPL35A;RPS20;RPSA;RPS3A
Racepinephrine-0.77_mg/kg_in_Saline-Rat-Heart-1d-dn	6/300	0,272573587	0,472444889	0	0	1,374570447	1,786730798	EEF1B2;RPS17;RPL32;RPS6;SEPP1;TPT1
Rifampin-99_mg/kg_in_CMC-Rat-Liver-5d-dn	6/300	0,272573587	0,472340941	0	0	1,374570447	1,786730798	RPL32;RPS6;RPS20;RPS3A;RPL10A;TPT1
Secobarbital-70_mg/kg_in_Water-Rat-Liver-5d-up	6/300	0,272573587	0,472237038	0	0	1,374570447	1,786730798	EEF1B2;RPS19;RPS6;RPS20;RPSA;RPL10A
Sertraline-23_uM_in_DMSO-Rat-Primary_rat_hepatocytes-0.67d-dn	6/300	0,272573587	0,472133181	0	0	1,374570447	1,786730798	EEF1B2;CXCL11;RPS19;RPSA;SEPP1;RPL39
Suxamethonium_Chloride-0.125_mg/kg_in_Saline-Rat-Heart-3d-up	6/300	0,272573587	0,47202937	0	0	1,374570447	1,786730798	RPS17;RPL32;RPS20;RPS3A;SEPP1;TPT1
Tetracycline-1500_mg/kg_in_Water-Rat-Liver-5d-dn	6/300	0,272573587	0,471925604	0	0	1,374570447	1,786730798	C1QA;RPS19;CLP1;RPS8;RPS3A;TPT1
Allyl_Alcohol-25_mg/kg_in_Saline-Rat-Liver-1d-up	7/361	0,273680546	0,473738017	0	0	1,332686029	1,726886219	EEF1B2;C1QA;RPS8;RPS6;RPS20;RPS3A;RPL39
D-Galactosamine-470_uM_in_DMSO-Rat-Primary_rat_hepatocytes-1d-dn	7/361	0,273680546	0,473633922	0	0	1,332686029	1,726886219	RPS19;RPS5;RPS6;RPSA;SEPP1;RPL39;SLC16A3
Sildenafil-420_mg/kg_in_Water-Rat-Liver-3d-dn	5/241	0,274512031	0,474968532	0	0	1,425902953	1,843350572	C1QA;RPL32;CLP1;SEPP1;TPT1
Acetazolamide-47_mg/kg_in_Corn_Oil-Rat-Liver-1d-up	6/301	0,275049519	0,475793985	0	0	1,370003768	1,768406519	RPS19;RPS8;RPS20;RPSA;RPL39;TPT1
Amprenavir-600_mg/kg_in_CMC-Rat-Kidney-5d-up	6/301	0,275049519	0,475689506	0	0	1,370003768	1,768406519	C1QA;RPS17;RPL32;RPL35A;RPS20;RPL39
Antipyrine-1500_mg/kg_in_CMC-Rat-Kidney-5d-dn	6/301	0,275049519	0,475585074	0	0	1,370003768	1,768406519	RPS19;ARL2BP;RPS6;RPS3A;RPL10A;RPL39
Cefotaxime-1000_mg/kg_in_Saline-Rat-Kidney-3d-dn	6/301	0,275049519	0,475480688	0	0	1,370003768	1,768406519	EEF1B2;RPS19;FUNDC1;RPS6;RPS3A;RPL39
Chloroform-600_mg/kg_in_Water-Rat-Liver-5d-dn	6/301	0,275049519	0,475376347	0	0	1,370003768	1,768406519	RPS17;CLP1;RPS3A;SEPP1;RPL39;TPT1
Digoxin-0.26_mg/kg_in_Corn_Oil-Rat-Liver-1d-dn	6/301	0,275049519	0,475272052	0	0	1,370003768	1,768406519	RPL32;RPS8;RPSA;RPL10A;SEPP1;TPT1
Emetine-1_mg/kg_in_Saline-Rat-Kidney-5d-up	6/301	0,275049519	0,475167803	0	0	1,370003768	1,768406519	CXCL11;RPS8;RPS5;RAPGEF5;SEPP1;TPT1
Metoprolol-1200_mg/kg_in_Water-Rat-Heart-5d-up	6/301	0,275049519	0,475063599	0	0	1,370003768	1,768406519	EEF1B2;RPS17;RPL32;RPS6;RPS3A;RPL39
Paraquat_Dichloride-650_uM_in_DMSO-Rat-Primary_rat_hepatocytes-1d-dn	6/301	0,275049519	0,474959441	0	0	1,370003768	1,768406519	RPS17;RPL32;RPS19;RPS8;SEPP1;TPT1
Pramoxine-526_mg/kg_in_Corn_Oil-Rat-Liver-5d-up	6/301	0,275049519	0,474855329	0	0	1,370003768	1,768406519	EEF1B2;C1QA;RPS17;RPS20;RPL10A;SEPP1
Prednisolone-184_mg/kg_in_Water-Rat-Liver-1d-dn	6/301	0,275049519	0,474751263	0	0	1,370003768	1,768406519	EEF1B2;RPS8;RPS6;RPS3A;RPL10A;SEPP1
Sulfamethoxazole-1000_mg/kg_in_Water-Rat-Kidney-1d-up	6/301	0,275049519	0,474647242	0	0	1,370003768	1,768406519	RPS17;ARL2BP;RPS8;RPS3A;SEPP1;TPT1
Trovafloxacin-600_mg/kg_in_CMC-Rat-Liver-1d-dn	6/301	0,275049519	0,474543267	0	0	1,370003768	1,768406519	RPS17;RPL32;RPS19;RPL10A;SEPP1;RPL39
Chlorpromazine-18_mg/kg_in_Water-Rat-Liver-0.25d-up	7/362	0,275933944	0,475964903	0	0	1,329004576	1,711218017	EEF1B2;RPL32;RPS19;RPS6;RPS20;RPS3A;RPL10A
Meloxicam-0.6_mg/kg_in_Corn_Oil-Rat-Kidney-3d-dn	5/242	0,277296679	0,478210782	0	0	1,420010792	1,821401413	RPS19;CLP1;RPS6;RPL10A;SEPP1
14-Dichlorobenzene-735_mg/kg_in_CMC-Rat-Kidney-1d-up	6/302	0,277530848	0,478509842	0	0	1,365467331	1,750287689	RPS17;RPL32;ARL6IP5;RPS3A;RPL10A;TPT1
Anastrozole-400_mg/kg_in_CMC-Rat-Liver-1d-dn	6/302	0,277530848	0,478405112	0	0	1,365467331	1,750287689	EEF1B2;C1QA;RPS17;CLP1;SEPP1;RPL39
Atenolol-1000_mg/kg_in_CMC-Rat-Heart-3d-dn	6/302	0,277530848	0,478300428	0	0	1,365467331	1,750287689	RPS6;RPS20;RPL10A;SEPP1;RPL39;TPT1
Aztreonam-1100_mg/kg_in_CMC-Rat-Spleen-3d-up	6/302	0,277530848	0,47819579	0	0	1,365467331	1,750287689	RPS19;RPS8;RPS5;RPSA;RPL10A;SEPP1
Bromobenzene-439_mg/kg_in_CMC-Rat-Kidney-3d-dn	6/302	0,277530848	0,478091198	0	0	1,365467331	1,750287689	EEF1B2;ARL2BP;CLP1;RPS8;RPS20;RPL39
Ethylene_Glycol-3525_mg/kg_in_Water-Rat-Liver-1d-dn	6/302	0,277530848	0,477986651	0	0	1,365467331	1,750287689	EEF1B2;RPL32;RPS19;RPS6;RPL10A;SEPP1
Lead_(II)_Acetate-600_mg/kg_in_Saline-Rat-Bone_marrow-5d-dn	6/302	0,277530848	0,477882151	0	0	1,365467331	1,750287689	EEF1B2;NKG7;RPL10A;SEPP1;RPL39;DUSP14
Pentoxifylline-1170_mg/kg_in_CMC-Rat-Heart-1d-dn	6/302	0,277530848	0,477777695	0	0	1,365467331	1,750287689	EEF1B2;RPS19;ARL2BP;RPS8;SEPP1;GOLGA7
Sulfadoxine-12_mg/kg_in_Corn_Oil-Rat-Liver-3d-up	6/302	0,277530848	0,477673286	0	0	1,365467331	1,750287689	RPS17;RPS19;RPS8;RPS20;RPS3A;GOLGA7
Zirconium(IV)_Chloride-633_mg/kg_in_Corn_Oil-Rat-Liver-1d-dn	6/302	0,277530848	0,477568922	0	0	1,365467331	1,750287689	C1QA;RPS6;RPS20;SEPP1;RPL39;GOLGA7
Cyclosporin_A-350_mg/kg_in_Corn_Oil-Rat-Liver-0.25d-up	7/363	0,278192184	0,478602369	0	0	1,325343406	1,695701467	RPS19;ARL2BP;RPS8;RPS5;RPS20;RPSA;SEPP1
Cytarabine-487_mg/kg_in_Saline-Rat-Liver-1d-up	7/363	0,278192184	0,478497848	0	0	1,325343406	1,695701467	C1QA;RPS17;RPL35A;RPSA;RPL10A;RPL39;TPT1
N-Nitrosodiethylamine-1.67_mg/kg_in_Saline-Rat-Kidney-0.25d-up	7/363	0,278192184	0,478393372	0	0	1,325343406	1,695701467	RPS17;CLP1;RPS6;RPS20;RPSA;SEPP1;GOLGA7
Benzothiazyl_Disulfide-2600_mg/kg_in_Corn_Oil-Rat-Kidney-3d-dn	6/303	0,280017444	0,481427066	0	0	1,360960838	1,732371648	RPS19;ARL6IP5;RPSA;RPL10A;SEPP1;RPL39
Cyclophosphamide-1320_uM_in_DMSO-Rat-Primary_rat_hepatocytes-0.67d-dn	6/303	0,280017444	0,481321997	0	0	1,360960838	1,732371648	CXCL11;ARL6IP5;RPSA;RPL10A;SEPP1;TPT1
Diethylstilbestrol-120_uM_in_DMSO-Rat-Primary_rat_hepatocytes-0.67d-up	6/303	0,280017444	0,481216973	0	0	1,360960838	1,732371648	EEF1B2;ARL2BP;CLP1;RPS3A;RPL39;PDRG1
Ethambutol-998_mg/kg_in_CMC-Rat-Kidney-1d-dn	6/303	0,280017444	0,481111996	0	0	1,360960838	1,732371648	ARL2BP;RPSA;RPL10A;SEPP1;RPL39;TPT1
Lead_(II)_Acetate-200_mg/kg_in_Saline-Rat-Kidney-0.25d-dn	6/303	0,280017444	0,481007064	0	0	1,360960838	1,732371648	RPS19;ARL2BP;RPS6;RPL10A;SEPP1;RPL39
Miconazole-200_mg/kg_in_Corn_Oil-Rat-Liver-1d-up	6/303	0,280017444	0,480902178	0	0	1,360960838	1,732371648	EEF1B2;RPS17;RPL32;RPS3A;RPL10A;RPL39
Oxaliplatin-8_mg/kg_in_Saline-Rat-Kidney-3d-up	6/303	0,280017444	0,480797338	0	0	1,360960838	1,732371648	RPS17;RPL32;RPS19;ARL2BP;RPS8;SEPP1

Rosiglitazone-10_mg/kg_in_Corn_Oil-Rat-Liver-5d-up	6/303	0,280017444	0,480692543	0	0	1,360960838	1,732371648	RPS17;RPS19;RPS8;RPS5;RPS20;RPSA
Sertraline-210_mg/kg_in_Corn_Oil-Rat-Heart-1d-up	6/303	0,280017444	0,480587795	0	0	1,360960838	1,732371648	EEF1B2;CXCL11;ARL6IP5;RAPGEF5;SEPP1;RPL39
Thioguanine-24_mg/kg_in_Corn_Oil-Rat-Kidney-1d-dn	6/303	0,280017444	0,480483091	0	0	1,360960838	1,732371648	RPL32;RPS19;RPS6;RPSA;RPL10A;RPL39
Trichloroethylene-1230_mg/kg_in_CMC-Rat-Kidney-1d-dn	6/303	0,280017444	0,480378434	0	0	1,360960838	1,732371648	RPS19;FEM1B;RPS6;RPS20;SEPP1;TPT1
Verapamil-108_mg/kg_in_Water-Rat-Heart-5d-dn	6/303	0,280017444	0,480273822	0	0	1,360960838	1,732371648	EEF1B2;ARL2BP;CLP1;COL5A3;RPS20;SEPP1
Hydrazine-45_mg/kg_in_Water-Rat-Kidney-5d-up	8/426	0,281637801	0,482947817	0	0	1,290676476	1,635459304	C1QA;RPS8;RPS3A;RAPGEF5;RPL10A;SEPP1;RPL39;TPT1
3-Chloroaniline-200_mg/kg_in_CMC-Rat-Kidney-5d-dn	6/304	0,282509179	0,48433659	0	0	1,356483993	1,714655774	EEF1B2;C1QA;RPL32;ARL2BP;RPS6;RPS20
Acyclovir-330_mg/kg_in_Saline-Rat-Spleen-0.25d-up	6/304	0,282509179	0,484231185	0	0	1,356483993	1,714655774	C1QA;RPS17;RPL32;RPS6;NKG7;RPS3A
Allylamine-75_mg/kg_in_Water-Rat-Heart-1d-dn	6/304	0,282509179	0,484125826	0	0	1,356483993	1,714655774	EEF1B2;RPS19;FUNDC1;RPS20;RPL10A;SEPP1
Baclofen-6.6_mg/kg_in_CMC-Rat-Heart-3d-up	6/304	0,282509179	0,484020512	0	0	1,356483993	1,714655774	RPS19;FEM1B;RPS6;RPL39;GOLGA7;TPT1
Carmustine-16_mg/kg_in_Corn_Oil-Rat-Bone_marrow-3d-dn	6/304	0,282509179	0,483915245	0	0	1,356483993	1,714655774	RPS19;FEM1B;RPS6;RPSA;RPS3A;RPL10A
Chlordiazepoxide-196_mg/kg_in_Water-Rat-Heart-5d-dn	6/304	0,282509179	0,483810023	0	0	1,356483993	1,714655774	ARL2BP;RPS8;RPS20;RPSA;TOMM20;TPT1
Chlorpyrifos-82_mg/kg_in_CMC-Rat-Kidney-1d-dn	6/304	0,282509179	0,483704847	0	0	1,356483993	1,714655774	EEF1B2;ARL2BP;RPS8;RPS20;RPSA;TOMM20
Cholic_Acid-1402_mg/kg_in_Water-Rat-Liver-3d-dn	6/304	0,282509179	0,483599716	0	0	1,356483993	1,714655774	EEF1B2;RPS17;CLP1;RPS20;SEPP1;TPT1
Cyclosporin_A-70_mg/kg_in_Corn_Oil-Rat-Liver-1d-up	6/304	0,282509179	0,483494632	0	0	1,356483993	1,714655774	RPL32;RPS19;RPS8;RPS5;RPS20;RPSA
Dicumarol-5_mg/kg_in_Corn_Oil-Rat-Liver-1d-dn	6/304	0,282509179	0,483389593	0	0	1,356483993	1,714655774	EEF1B2;RPL32;RPS6;ARL6IP5;RPL10A;SEPP1
Fluoxetine-52_mg/kg_in_CMC-Rat-Liver-5d-up	6/304	0,282509179	0,483284599	0	0	1,356483993	1,714655774	C1QA;RPS17;ARL2BP;RPS8;RPSA;RPS3A
Fluphenazine-22_mg/kg_in_Corn_Oil-Rat-Liver-5d-dn	6/304	0,282509179	0,483179651	0	0	1,356483993	1,714655774	C1QA;CLP1;RPL10A;SEPP1;RPL39;TPT1
Mefenamic_Acid-93_mg/kg_in_Corn_Oil-Rat-Liver-0.25d-dn	6/304	0,282509179	0,483074749	0	0	1,356483993	1,714655774	EEF1B2;RPS17;RPS20;RPSA;RPL39;TPT1
Mifepristone-3_mg/kg_in_Corn_Oil-Rat-Liver-0.25d-up	6/304	0,282509179	0,482969893	0	0	1,356483993	1,714655774	EEF1B2;C1QA;RPS19;RPS6;SEPP1;GOLGA7
NN-Dimethylformamide-1400_mg/kg_in_Saline-Rat-Liver-5d-up	6/304	0,282509179	0,482865081	0	0	1,356483993	1,714655774	RPS17;RPS6;RPL35A;RPS20;RPSA;RPL39
Niacinamide-750_mg/kg_in_Water-Rat-Liver-1d-dn	6/304	0,282509179	0,482760316	0	0	1,356483993	1,714655774	RPS19;RPS8;RPS20;RPSA;SEPP1;TPT1
Oxfendazole-1500_mg/kg_in_CMC-Rat-Kidney-1d-up	6/304	0,282509179	0,482655595	0	0	1,356483993	1,714655774	CLP1;RPSA;RPS3A;RAPGEF5;SEPP1;RPL39
Rofecoxib-3_mg/kg_in_Corn_Oil-Rat-Kidney-3d-up	6/304	0,282509179	0,482550921	0	0	1,356483993	1,714655774	CXCL11;RPS8;RPS20;RPS3A;GOLGA7;TPT1
Simvastatin-1200_mg/kg_in_Corn_Oil-Rat-Kidney-3d-dn	6/304	0,282509179	0,482446291	0	0	1,356483993	1,714655774	RPS19;ARL2BP;RPS6;RPL10A;RPL39;TPT1
Sulfadoxine-12_mg/kg_in_Corn_Oil-Rat-Liver-1d-dn	6/304	0,282509179	0,482341707	0	0	1,356483993	1,714655774	EEF1B2;RPS17;RPL32;RPS8;RPS3A;SEPP1
Thimerosal-10_mg/kg_in_Water-Rat-Brain-5d-up	6/304	0,282509179	0,482237168	0	0	1,356483993	1,714655774	RPS19;CLP1;RPL10A;SEPP1;RPL39;GOLGA7
Tranilast-300_mg/kg_in_CMC-Rat-Spleen-3d-dn	6/304	0,282509179	0,482132675	0	0	1,356483993	1,714655774	C1QA;RPS17;RPL32;NKG7;RPS3A;PDRG1
Levofloxacin-739_mg/kg_in_Water-Rat-Kidney-5d-dn	4/185	0,283250176	0,483292545	0	0	1,486022105	1,874505072	RPS17;RPL32;RPS19;RPS6
Thiamphenicol-1500_mg/kg_in_Water-Rat-Kidney-3d-up	7/366	0,284994988	0,48616429	0	0	1,314479935	1,650045215	EEF1B2;RPS17;RPL32;RPS6;RPS20;RPS3A;RPL39
Allylamine-75_mg/kg_in_Water-Rat-Heart-5d-dn	6/305	0,285005924	0,486077665	0	0	1,352036505	1,697137487	RPS19;ARL2BP;COL5A3;FUNDC1;RPS6;RPS20
Atorvastatin-2.5_mg/kg_in_Corn_Oil-Rat-Kidney-1d-up	6/305	0,285005924	0,48597243	0	0	1,352036505	1,697137487	RPS19;ARL2BP;CLP1;RPS20;SEPP1;GOLGA7
Balsalazide-1100_mg/kg_in_Water-Rat-Liver-5d-up	6/305	0,285005924	0,485867242	0	0	1,352036505	1,697137487	RPS17;RPS19;RPS6;RAPGEF5;RPL10A;RPL39
Buspirone-196_mg/kg_in_CMC-Rat-Heart-5d-dn	6/305	0,285005924	0,485762098	0	0	1,352036505	1,697137487	FEM1B;FUNDC1;RPS20;RPL10A;SEPP1;RPL39
Catechol-40_mg/kg_in_Saline-Rat-Liver-0.25d-up	6/305	0,285005924	0,485657	0	0	1,352036505	1,697137487	RPS17;RPL35A;RPS3A;RPL10A;SEPP1;RPL39
Celecoxib-100_uM_in_DMSO-Rat-Primary_rat_hepatocytes-0.67d-up	6/305	0,285005924	0,485551948	0	0	1,352036505	1,697137487	EEF1B2;ARL2BP;RPS6;RPS20;RPS3A;RPL39
Daunorubicin-3.25_mg/kg_in_Saline-Rat-Bone_marrow-0.25d-up	6/305	0,285005924	0,485446941	0	0	1,352036505	1,697137487	RPS19;FEM1B;RPS6;NKG7;RPSA;RPL10A
Diazepam-710_mg/kg_in_CMC-Rat-Liver-3d-up	6/305	0,285005924	0,48534198	0	0	1,352036505	1,697137487	RPS19;RPS8;RPS6;RPSA;RPL10A;RPL39
Econazole-43_mg/kg_in_Corn_Oil-Rat-Liver-1d-dn	6/305	0,285005924	0,485237064	0	0	1,352036505	1,697137487	C1QA;RPS8;RPS5;RPL35A;RPSA;SEPP1
Ethanol-6000_mg/kg_in_Saline-Rat-Liver-1d-dn	6/305	0,285005924	0,485132193	0	0	1,352036505	1,697137487	RPL32;RPS6;RPS20;RPS3A;RPL10A;SEPP1
Ethylene_Glycol-352_mg/kg_in_Water-Rat-Kidney-1d-up	6/305	0,285005924	0,485027367	0	0	1,352036505	1,697137487	RPS17;FUNDC1;RPL35A;RPS20;SEPP1;RPL39
Fenofibrate-43_mg/kg_in_Corn_Oil-Rat-Liver-5d-up	6/305	0,285005924	0,484922587	0	0	1,352036505	1,697137487	RPL32;RPS19;RPL35A;RPS20;RPL39;TPT1
Gemfibrozil-100_mg/kg_in_Corn_Oil-Rat-Kidney-0.25d-up	6/305	0,285005924	0,484817852	0	0	1,352036505	1,697137487	RPS19;CLP1;FUNDC1;RPS20;SEPP1;GOLGA7
Levosulpiride-1200_mg/kg_in_CMC-Rat-Heart-3d-dn	6/305	0,285005924	0,484713163	0	0	1,352036505	1,697137487	EEF1B2;RPS19;COL5A3;RPS6;RPL10A;RPL39
Miconazole-200_mg/kg_in_Corn_Oil-Rat-Liver-5d-dn	6/305	0,285005924	0,484608518	0	0	1,352036505	1,697137487	C1QA;RPS19;CLP1;RPS8;SEPP1;TPT1
Oxaliplatin-1.31_mg/kg_in_Saline-Rat-Bone_marrow-0.25d-dn	6/305	0,285005924	0,484503919	0	0	1,352036505	1,697137487	EEF1B2;RPL35A;RPS20;RPS3A;RPL10A;RPL39
Paraquat_Dichloride-650_uM_in_DMSO-Rat-Primary_rat_hepatocytes-0.67d-up	6/305	0,285005924	0,484399365	0	0	1,352036505	1,697137487	RPS19;RPS20;RPL10A;RPL39;SLC16A3;PDRG1
Pioglitazone-1500_mg/kg_in_Corn_Oil-Rat-Heart-1d-up	6/305	0,285005924	0,484294856	0	0	1,352036505	1,697137487	EEF1B2;RPS8;COL5A3;RPSA;RPL39;TPT1
Pioglitazone-300_mg/kg_in_Corn_Oil-Rat-Liver-3d-dn	6/305	0,285005924	0,484190392	0	0	1,352036505	1,697137487	RPS19;CLP1;RPS8;RPS3A;RPL10A;SEPP1
Piroxicam-14_mg/kg_in_CMC-Rat-Kidney-1d-dn	6/305	0,285005924	0,484085973	0	0	1,352036505	1,697137487	CLP1;RPS5;RPS6;RPS3A;SEPP1;TPT1
Trimethadione-535_mg/kg_in_Water-Rat-Heart-5d-dn	6/305	0,285005924	0,483981599	0	0	1,352036505	1,697137487	RPS8;FUNDC1;RPS20;RPSA;RPL39;TPT1
Valproic_Acid-850_mg/kg_in_Water-Rat-Liver-0.25d-up	6/305	0,285005924	0,48387727	0	0	1,352036505	1,697137487	RPL32;RPS19;RPS6;RPS20;RPS3A;RPL10A
Modafinil-325_mg/kg_in_Saline-Rat-Liver-5d-dn	5/245	0,285684781	0,484925287	0	0	1,402622905	1,757298887	CLP1;RPSA;RPL39;SEPP1;TPT1
Hydrazine-45_mg/kg_in_Water-Rat-Kidney-1d-up	8/428	0,285832744	0,4850719	0	0	1,284645277	1,608823525	EEF1B2;RPL32;RPS19;ARL2BP;RPS8;RPS3A;GOLGA7;TPT1
Neomycin-56_mg/kg_in_Corn_Oil-Rat-Kidney-3d-up	7/367	0,287271629	0,487408736	0	0	1,310898246	1,635118865	RPS17;RPS6;RPS20;RPSA;RAPGEF5;RPL39;GOLGA7
6-Mercaptopurine-5_uM_in_DMSO-Rat-Primary_rat_hepatocytes-0.67d-up	6/306	0,287507549	0,487703954	0	0	1,347618085	1,679814246	RPS8;RPSA;RPS3A;RPL10A;RPL39;TPT1
Artemether-74_mg/kg_in_Corn_Oil-Rat-Liver-3d-dn	6/306	0,287507549	0,487598936	0	0	1,347618085	1,679814246	RPS17;RPL32;RPS8;FEM1B;RPL35A;RPS20
Azaribine-750_mg/kg_in_CMC-Rat-Heart-5d-dn	6/306	0,287507549	0,487493963	0	0	1,347618085	1,679814246	RPS19;FUNDC1;RPS6;ARL6IP5;SEPP1;TPT1
BW-723C86-10_mg/kg_in_Saline-Rat-Heart-5d-up	6/306	0,287507549	0,487389035	0	0	1,347618085	1,679814246	RPS19;ARL2BP;FUNDC1;RPS20;RAPGEF5;RPL39
Bisphenol_A-100_mg/kg_in_Corn_Oil-Rat-Spleen-1d-dn	6/306	0,287507549	0,487284153	0	0	1,347618085	1,679814246	EEF1B2;RPS19;RPS6;RPSA;RPL10A;RPL39
Candesartan-1300_mg/kg_in_CMC-Rat-Heart-5d-up	6/306	0,287507549	0,487179315	0	0	1,347618085	1,679814246	EEF1B2;RPL32;CLP1;RPS20;RAPGEF5;RPL39
Carbamazepine-490_mg/kg_in_CMC-Rat-Liver-1d-up	6/306	0,287507549	0,487074523	0	0	1,347618085	1,679814246	RPS19;RPS6;RPSA;RPS3A;RPL10A;TPT1
Carmustine-4_mg/kg_in_Corn_Oil-Rat-Spleen-1d-up	6/306	0,287507549	0,486969776	0	0	1,347618085	1,679814246	C1QA;RPS6;RPS3A;RPL10A;SEPP1;TPT1
Chlorpromazine-73_mg/kg_in_Water-Rat-Liver-1d-up	6/306	0,287507549	0,486865074	0	0	1,347618085	1,679814246	RPS17;CXCL11;RPL32;RPS20;RPS3A;TPT1
Clofibric_Acid-448_mg/kg_in_Corn_Oil-Rat-Liver-1d-dn	6/306	0,287507549	0,486760417	0	0	1,347618085	1,679814246	EEF1B2;C1QA;RPS20;SEPP1;RPL39;TPT1
Econazole-43_mg/kg_in_Corn_Oil-Rat-Liver-5d-dn	6/306	0,287507549	0,486655804	0	0	1,347618085	1,679814246	C1QA;RPS19;CLP1;RPS20;RPSA;SEPP1
Ethanol-3000_mg/kg_in_Saline-Rat-Kidney-3d-dn	6/306	0,287507549	0,486551237	0	0	1,347618085	1,679814246	EEF1B2;RPL32;RPS6;RPS3A;RPL10A;SEPP1
Fenofibrate-215_mg/kg_in_Corn_Oil-Rat-Heart-5d-up	6/306	0,287507549	0,486446715	0	0	1,347618085	1,679814246	RPS19;RPSA;RAPGEF5;TOMM20;TPT1;PDRG1
Lidocaine-71_mg/kg_in_Water-Rat-Heart-3d-up	6/306	0,287507549	0,486342237	0	0	1,347618085	1,679814246	RPS19;ARL2BP;FUNDC1;RPS6;RAPGEF5;GOLGA7
Rifabutin-1500_mg/kg_in_CMC-Rat-Liver-5d-dn	6/306	0,287507549	0,486237805	0	0	1,347618085	1,679814246	C1QA;RPS17;RPL32;RPS6;RPS3A;RPL39
Sildenafil-90_uM_in_DMSO-Rat-Primary_rat_hepatocytes-0.67d-up	6/306	0,287507549	0,486133417	0	0	1,347618085	1,679814246	RPS6;RPS3A;RPL10A;RPL39;TPT1;PDRG1
Sparteine-2222_uM_in_DMSO-Rat-Primary_rat_hepatocytes-0.67d-dn	6/306	0,287507549	0,486029074	0	0	1,347618085	1,679814246	EEF1B2;RPS20;RPSA;RPL39;SLC16A3;TPT1
Tacrine-24_mg/kg_in_Water-Rat-Heart-1d-up	6/306	0,287507549	0,485924776	0	0	1,347618085	1,679814246	C1QA;ARL2BP;RPS8;RPS6;RPS3A;TPT1
Tinidazole-1200_mg/kg_in_CMC-Rat-Liver-3d-up	6/306	0,287507549	0,485820523	0	0	1,347618085	1,679814246	RPS19;RPS6;RPS20;RPSA;RPL10A;RPL39
Ubenimex-1300_mg/kg_in_Water-Rat-Kidney-1d-up	6/306	0,287507549	0,485716314	0	0	1,347618085	1,679814246	RPS17;RPS19;RPS6;RPS20;RPL10A;RPL39
Celecoxib-100_uM_in_DMSO-Rat-Primary_rat_hepatocytes-1d-up	5/246	0,288491591	0,487274238	0	0	1,396921186	1,736497836	RPS17;RPS6;RPS3A;RPL10A;PDRG1
Fenofibrate-43_mg/kg_in_Corn_Oil-Rat-Kidney-3d-up	5/246	0,288491591	0,487169762	0	0	1,396921186	1,736497836	RPS8;RPS6;RPS20;RPSA;GOLGA7
33'5-Triiodo-L-Thyronine-0.1_mg/kg_in_PEG400-Rat-Heart-3d-up	6/307	0,290013926	0,489635517	0	0	1,34322845	1,662683549	EEF1B2;RPS19;RPSA;RAPGEF5;RPL10A;TPT1
Azauridine-1500_mg/kg_in_CMC-Rat-Kidney-1d-up	6/307	0,290013926	0,48953058	0	0	1,34322845	1,662683549	EEF1B2;CLP1;RPS8;RPS3A;SEPP1;TPT1
Digitonin-75_mg/kg_in_Corn_Oil-Rat-Heart-5d-up	6/307	0,290013926	0,489425688	0	0	1,34322845	1,662683549	C1QA;RPS17;RPL32;RPS3A;RPL39;PDRG1
Ergocalciferol-15_mg/kg_in_CMC-Rat-Kidney-1d-up	6/307	0,290013926	0,489320841	0	0	1,34322845	1,662683549	EEF1B2;RPS8;RPS20;RPS3A;SEPP1;TPT1
Flavoxate-1255_mg/kg_in_CMC-Rat-Heart-5d-dn	6/307	0,290013926	0,489216039	0	0	1,34322845	1,662683549	EEF1B2;COL5A3;ARL6IP5;RPSA;RPL10A;RPL39
Iproniazid-46_mg/kg_in_Water-Rat-Brain-5d-dn	6/307	0,290013926	0,489111282	0	0	1,34322845	1,662683549	RPL32;RPS8;FEM1B;RPS3A;TOMM20;TPT1
Lead(IV)_Acetate-200_mg/kg_in_Saline-Rat-Kidney-0.25d-up	6/307	0,290013926	0,489006569	0	0	1,34322845	1,662683549	CXCL11;RPS19;RPS8;RPS20;RPSA;TOMM20
Mitoxantrone-2_mg/kg_in_Saline-Rat-Heart-5d-dn	6/307	0,290013926	0,488901902	0	0	1,34322845	1,662683549	EEF1B2;ARL6IP5;RPSA;RAPGEF5;SEPP1;RPL39
Nisoldipine-15_mg/kg_in_Water-Rat-Liver-0.25d-up	6/307	0,290013926	0,488797279	0	0	1,34322845	1,662683549	C1QA;RPS6;RPS20;SEPP1;RPL39;TPT1
Oxytetracycline-1500_mg/kg_in_Water-Rat-Liver-1d-up	6/307	0,290013926	0,488692701	0	0	1,34322845	1,662683549	C1QA;RPS17;RPS6;RPS20;RPS3A;RPL39
Pentobarbital-70_mg/kg_in_Water-Rat-Liver-5d-dn	6/307	0,290013926	0,488588168	0	0	1,34322845	1,662683549	RPS17;RPS20;RPSA;SEPP1;RPL39;TPT1
Simvastatin-15_mg/kg_in_Corn_Oil-Rat-Liver-3d-dn	6/307	0,290013926	0,488483679	0	0	1,34322845	1,662683549	C1QA;RPS20;RPSA;SEPP1;RPL39;TPT1
Tolazamide-1500_mg/kg_in_CMC-Rat-Kidney-3d-up	6/307	0,290013926	0,488379236	0	0	1,34322845	1,662683549	RPS17;RPS20;RPS3A;RAPGEF5;RPL10A;SEPP1

Valeric_Acid-23000_uM_in_DMSO-Rat-Primary_rat_hepatocytes-0.67d-dn	6/307	0,290013926	0,488274836	0	0	1,34322845	1,662683549	ARL2BP;RPS6;ARL6IP5;RPS20;SEPP1;SLC16A3
Valproic_Acid-3168_uM_in_DMSO-Rat-Primary_rat_hepatocytes-1d-dn	6/307	0,290013926	0,488170482	0	0	1,34322845	1,662683549	ARL2BP;RPS8;RPS5;RPS20;RPS3A;TPT1
Valsartan-30_mg/kg_in_Corn_Oil-Rat-Heart-0.25d-up	6/307	0,290013926	0,488066172	0	0	1,34322845	1,662683549	RPS5;RPS20;RAPGEF5;SEPP1;RPL39;TPT1
Vincristine-0.05_mg/kg_in_Saline-Rat-Bone_marrow-3d-dn	6/307	0,290013926	0,487961907	0	0	1,34322845	1,662683549	EEF1B2;RPS17;RPS19;FEM1B;RPS6;TPT1
Zidovudine-14800_uM_in_DMSO-Rat-Primary_rat_hepatocytes-0.67d-dn	6/307	0,290013926	0,487857686	0	0	1,34322845	1,662683549	CXCL11;ARL6IP5;RPSA;RPL10A;SEPP1;SLC16A3
Allyl_Alcohol-16_mg/kg_in_Saline-Rat-Liver-1d-dn	6/308	0,292524927	0,49197658	0	0	1,338867318	1,645742932	RPS17;RPS6;RPS3A;RPL10A;RPL39;TPT1
Azathioprine-20_mg/kg_in_Corn_Oil-Rat-Bone_marrow-1d-up	6/308	0,292524927	0,491871546	0	0	1,338867318	1,645742932	C1QA;RPS17;NKG7;SEPP1;RPL39;TPT1
Beta-Estradiol-0.3_mg/kg_in_Corn_Oil-Rat-Liver-0.25d-dn	6/308	0,292524927	0,491766558	0	0	1,338867318	1,645742932	C1QA;RPS19;CLP1;RPS8;RPS20;TPT1
Ciprofloxacin-72_mg/kg_in_Corn_Oil-Rat-Liver-5d-up	6/308	0,292524927	0,491661614	0	0	1,338867318	1,645742932	RPS8;RPS20;RPSA;SEPP1;RPL39;TPT1
Dichlorvos-17_mg/kg_in_Water-Rat-Liver-5d-dn	6/308	0,292524927	0,491556715	0	0	1,338867318	1,645742932	EEF1B2;RPS19;RPS20;RPSA;RPL39;TPT1
Diclofenac-10_mg/kg_in_Corn_Oil-Rat-Liver-3d-up	6/308	0,292524927	0,491451861	0	0	1,338867318	1,645742932	C1QA;RPS6;RPS20;RPS3A;RPL10A;RPL39
Diethylstilbestrol-2.8_mg/kg_in_Corn_Oil-Rat-Bone_marrow-0.25d-up	6/308	0,292524927	0,491347051	0	0	1,338867318	1,645742932	EEF1B2;C1QA;RPSA;RPL10A;SEPP1;RPL39
Ibuprofen-263_mg/kg_in_Corn_Oil-Rat-Kidney-3d-dn	6/308	0,292524927	0,491242286	0	0	1,338867318	1,645742932	EEF1B2;RPS17;ARL2BP;SEPP1;RPL39;TPT1
Mevastatin-1200_mg/kg_in_CMC-Rat-Liver-3d-dn	6/308	0,292524927	0,491137566	0	0	1,338867318	1,645742932	EEF1B2;RPL32;RPS19;RPS8;RPS3A;RPL10A
NN-Dimethylformamide-140_mg/kg_in_Saline-Rat-Liver-1d-up	6/308	0,292524927	0,491032891	0	0	1,338867318	1,645742932	RPS17;RPS19;RPSA;RPS3A;RPL10A;RPL39
Nimesulide-30_mg/kg_in_Corn_Oil-Rat-Kidney-3d-dn	6/308	0,292524927	0,49092826	0	0	1,338867318	1,645742932	RPS19;RPS6;RPS20;RPL10A;SEPP1;RPL39
Nitrendipine-10_mg/kg_in_Corn_Oil-Rat-Heart-0.25d-up	6/308	0,292524927	0,490823673	0	0	1,338867318	1,645742932	RPS17;COL5A3;RPS20;RPL10A;SEPP1;RPL39
Procarbazine-27_mg/kg_in_Water-Rat-Liver-0.25d-up	6/308	0,292524927	0,490719132	0	0	1,338867318	1,645742932	EEF1B2;RPS19;CLP1;RPS6;RPS20;SEPP1
Thiabendazole-92_mg/kg_in_Corn_Oil-Rat-Liver-1d-up	6/308	0,292524927	0,490614634	0	0	1,338867318	1,645742932	RPS19;RPS6;RPSA;RPL10A;SEPP1;RPL39
Tretinoin-7_mg/kg_in_Corn_Oil-Rat-Kidney-3d-up	6/308	0,292524927	0,490510182	0	0	1,338867318	1,645742932	ARL2BP;RPS8;RPSA;RAPGEF5;SEPP1;TPT1
44'-Methylenedianiline-296_uM_in_DMSO-Rat-Primary_rat_hepatocytes-1d-up	4/188	0,292995113	0,491194022	0	0	1,462308986	1,795129557	EEF1B2;RPS6;RPL39;PDRG1
Gold_Sodium_Thiomalate-8_mg/kg_in_Saline-Rat-Kidney-14d-dn	5/248	0,294120284	0,49297539	0	0	1,385655692	1,695718968	EEF1B2;RPS17;ARL2BP;RPS20;SEPP1
N-Nitrosodiethylamine-7000_uM_in_DMSO-Rat-Primary_rat_hepatocytes-1d-up	5/248	0,294120284	0,492870502	0	0	1,385655692	1,695718968	EEF1B2;CXCL11;ARL6IP5;SEPP1;PDRG1
Pramoxine-526_mg/kg_in_Corn_Oil-Rat-Heart-3d-dn	5/248	0,294120284	0,492765658	0	0	1,385655692	1,695718968	RPS8;RPS20;SEPP1;TPT1;PDRG1
Rofecoxib-3_mg/kg_in_Corn_Oil-Rat-Liver-1d-dn	5/248	0,294120284	0,492660859	0	0	1,385655692	1,695718968	EEF1B2;RPS17;RPL32;RPS8;TPT1
4-Octylphenol-450_mg/kg_in_Corn_Oil-Rat-Liver-5d-up	6/309	0,295040422	0,494097036	0	0	1,334534414	1,62898997	C1QA;RPS19;ARL2BP;RPS6;RPS3A;TPT1
Acemetacin-2_mg/kg_in_Corn_Oil-Rat-Liver-1d-dn	6/309	0,295040422	0,493991999	0	0	1,334534414	1,62898997	EEF1B2;FEM1B;RPS20;RPSA;RPL10A;RPL39
Aconitine-0.9_mg/kg_in_CMC-Rat-Heart-1d-up	6/309	0,295040422	0,493887006	0	0	1,334534414	1,62898997	RPS17;RPS6;ARL6IP5;SEPP1;RPL39;TPT1
Amiodarone-37.04_uM_in_DMSO-Rat-Primary_rat_hepatocytes-0.67d-up	6/309	0,295040422	0,493782057	0	0	1,334534414	1,62898997	RPS6;RPS20;RPSA;RPS3A;RPL39;SLC16A3
Balsalazide-1100_mg/kg_in_Water-Rat-Liver-1d-up	6/309	0,295040422	0,493677153	0	0	1,334534414	1,62898997	RPS19;RPS6;RPSA;RPL10A;SEPP1;RPL39
Bleomycin_A2-12_mg/kg_in_Saline-Rat-Bone_marrow-0.25d-dn	6/309	0,295040422	0,493572294	0	0	1,334534414	1,62898997	EEF1B2;RPS17;NKG7;RPS3A;SEPP1;RPL39
Carvedilol-2000_mg/kg_in_Corn_Oil-Rat-Heart-1d-up	6/309	0,295040422	0,49346748	0	0	1,334534414	1,62898997	C1QA;RPS17;RPS3A;SEPP1;RPL39;TPT1
Clonazepam-2500_mg/kg_in_CMC-Rat-Liver-3d-up	6/309	0,295040422	0,493362709	0	0	1,334534414	1,62898997	RPS6;RPS20;RPSA;RPL10A;RPL39;TPT1
Dyphylline-3855_mg/kg_in_CMC-Rat-Heart-3d-dn	6/309	0,295040422	0,493257984	0	0	1,334534414	1,62898997	EEF1B2;RPS8;ARL6IP5;RPL10A;SEPP1;RPL39
Fluphenazine-2.5_mg/kg_in_Corn_Oil-Rat-Liver-3d-up	6/309	0,295040422	0,493153302	0	0	1,334534414	1,62898997	EEF1B2;RPL32;RPS19;RPS3A;RPL39;TPT1
Labetalol-250_uM_in_DMSO-Rat-Primary_rat_hepatocytes-0.67d-dn	6/309	0,295040422	0,493048666	0	0	1,334534414	1,62898997	CXCL11;CLP1;ARL6IP5;SEPP1;SLC16A3;TPT1
Methapyrilene-300_uM_in_DMSO-Rat-Primary_rat_hepatocytes-0.67d-dn	6/309	0,295040422	0,492944073	0	0	1,334534414	1,62898997	CXCL11;FUNDC1;RPL10A;SEPP1;SLC16A3;TPT1
Norfloxacin-1500_mg/kg_in_CMC-Rat-Kidney-3d-dn	6/309	0,295040422	0,492839525	0	0	1,334534414	1,62898997	EEF1B2;RPS19;CLP1;RPSA;SEPP1;TOMM20
Oxaprozin-1500_mg/kg_in_CMC-Rat-Kidney-1d-dn	6/309	0,295040422	0,492735021	0	0	1,334534414	1,62898997	EEF1B2;RPS8;RPL35A;RPSA;SEPP1;TOMM20
Oxyquinoline-68_mg/kg_in_Corn_Oil-Rat-Liver-5d-dn	6/309	0,295040422	0,492630562	0	0	1,334534414	1,62898997	RPS17;RPS6;RPSA;RPS3A;RPL10A;RPL39
Pramoxine-526_mg/kg_in_Corn_Oil-Rat-Heart-5d-dn	6/309	0,295040422	0,492526147	0	0	1,334534414	1,62898997	RPS6;RPSA;SEPP1;GOLGA7;TOMM20;PDRG1
Roxarsone-11_mg/kg_in_Corn_Oil-Rat-Kidney-3d-dn	6/309	0,295040422	0,492421776	0	0	1,334534414	1,62898997	EEF1B2;RPS19;RPL10A;SEPP1;RPL39;TPT1
Sildenafil-14.6_mg/kg_in_Saline-Rat-Liver-3d-dn	6/309	0,295040422	0,492317449	0	0	1,334534414	1,62898997	EEF1B2;C1QA;RPL32;RPS19;CLP1;SEPP1
Zomepirac-11_mg/kg_in_Saline-Rat-Liver-1d-dn	6/309	0,295040422	0,492213167	0	0	1,334534414	1,62898997	RPL32;RPS19;RPS6;RPL10A;SEPP1;TPT1
Sulindac-23_mg/kg_in_Corn_Oil-Rat-Kidney-0.25d-up	7/371	0,296420817	0,494411342	0	0	1,296764572	1,576833502	EEF1B2;RPS19;FUNDC1;RPS6;RPSA;RPL39;TPT1
N-Nitrosodimethylamine-10_mg/kg_in_Saline-Rat-Liver-1d-dn	5/249	0,296941805	0,495175452	0	0	1,38009081	1,675732623	RPS17;RPL32;RPS6;RPL10A;RPL39
Atorvastatin-2.5_mg/kg_in_Corn_Oil-Rat-Liver-3d-dn	6/310	0,297560283	0,496101775	0	0	1,330229465	1,612422273	C1QA;RPS17;RPL32;RPS6;RPS3A;GOLGA7
Bisphenol_A-100_mg/kg_in_Corn_Oil-Rat-Bone_marrow-3d-dn	6/310	0,297560283	0,49599678	0	0	1,330229465	1,612422273	RPS19;ARL6IP5;NKG7;RPSA;RPL10A;RPL39
Busulfan-3_mg/kg_in_Corn_Oil-Rat-Liver-1d-up	6/310	0,297560283	0,49589183	0	0	1,330229465	1,612422273	EEF1B2;RPL32;RPS19;RPS3A;SEPP1;RPL39
Daunorubicin-4_mg/kg_in_Saline-Rat-Heart-5d-up	6/310	0,297560283	0,495786923	0	0	1,330229465	1,612422273	RPS19;ARL2BP;COL5A3;RPS20;IL12A;SEPP1
Diclofenac-10_mg/kg_in_Corn_Oil-Rat-Kidney-1d-up	6/310	0,297560283	0,495682061	0	0	1,330229465	1,612422273	RPS19;RPS5;RPSA;RAPGEF5;GOLGA7;TPT1
Etoposide-100_mg/kg_in_Corn_Oil-Rat-Liver-0.25d-up	6/310	0,297560283	0,495577244	0	0	1,330229465	1,612422273	EEF1B2;RPS19;RPS8;RPS6;RPS20;SEPP1
Fenofibrate-43_mg/kg_in_Corn_Oil-Rat-Kidney-0.25d-up	6/310	0,297560283	0,495472471	0	0	1,330229465	1,612422273	RPS8;FUNDC1;RAPGEF5;SEPP1;RPL39;TPT1
Noscapine-570_mg/kg_in_CMC-Rat-Spleen-3d-dn	6/310	0,297560283	0,495367742	0	0	1,330229465	1,612422273	C1QA;RPS17;RPS6;NKG7;RPS20;RPS3A
Phenothiazine-386_mg/kg_in_Corn_Oil-Rat-Liver-5d-dn	6/310	0,297560283	0,495263057	0	0	1,330229465	1,612422273	C1QA;RPS17;RPS19;RPS8;RPS6;RPSA
Procarbazine-54_mg/kg_in_Water-Rat-Bone_marrow-3d-up	6/310	0,297560283	0,495158417	0	0	1,330229465	1,612422273	C1QA;RPS19;NKG7;RPSA;RPL10A;SEPP1
Thioguanine-24_mg/kg_in_Corn_Oil-Rat-Liver-1d-dn	6/310	0,297560283	0,495053821	0	0	1,330229465	1,612422273	EEF1B2;RPS6;RPS20;RPSA;RPL10A;SEPP1
Simvastatin-1200_mg/kg_in_Corn_Oil-Rat-Kidney-1d-dn	5/250	0,299767868	0,498621273	0	0	1,374570447	1,656009452	EEF1B2;RPL10A;RPL39;SEPP1;TPT1
Simvastatin-1200_mg/kg_in_Corn_Oil-Rat-Liver-1d-up	5/250	0,299767868	0,49851599	0	0	1,374570447	1,656009452	RPS17;RPS19;ARL2BP;RPS8;RPSA
Carmustine-4_mg/kg_in_Corn_Oil-Rat-Liver-5d-up	6/311	0,300084382	0,498937004	0	0	1,325952199	1,596037487	RPS8;RPS20;RPSA;RPL39;GOLGA7;TPT1
Doxorubicin-3_mg/kg_in_Saline-Rat-Kidney-5d-dn	6/311	0,300084382	0,498831699	0	0	1,325952199	1,596037487	RPS17;RPL32;RPS19;ARL2BP;RPSA;SEPP1
Dyphylline-3855_mg/kg_in_CMC-Rat-Heart-5d-dn	6/311	0,300084382	0,498726438	0	0	1,325952199	1,596037487	EEF1B2;RPS8;FUNDC1;SEPP1;RPL39;TPT1
Famotidine-1200_mg/kg_in_CMC-Rat-Heart-3d-up	6/311	0,300084382	0,498621222	0	0	1,325952199	1,596037487	C1QA;RPS17;RPL32;RPS6;RPS3A;RAPGEF5
Fludrocortisone_Acetate-125_mg/kg_in_CMC-Rat-Kidney-5d-dn	6/311	0,300084382	0,498516049	0	0	1,325952199	1,596037487	RPS17;RPL32;ARL2BP;CLP1;RPL35A;RPS3A
Propranolol-175_mg/kg_in_Water-Rat-Heart-5d-up	6/311	0,300084382	0,498410922	0	0	1,325952199	1,596037487	C1QA;RPS17;RPL32;RPSA;RPL10A;RPL39
Tazobactam-1500_mg/kg_in_CMC-Rat-Spleen-5d-dn	6/311	0,300084382	0,498305838	0	0	1,325952199	1,596037487	C1QA;RPS17;RPL32;RPS8;RPS3A;TPT1
Azathioprine-54_mg/kg_in_Water-Rat-Kidney-3d-up	7/373	0,301019633	0,499753506	0	0	1,289811411	1,548521513	RPS19;CLP1;ARL6IP5;RPS20;RPSA;SEPP1;GOLGA7
Bisphenol_A-610_mg/kg_in_Corn_Oil-Rat-Kidney-3d-up	5/251	0,302598294	0,502268527	0	0	1,36909407	1,636545387	ARL2BP;RPS8;RPS20;RPSA;SEPP1
Cobalt(II)_Acetylacetonate-100_mg/kg_in_Corn_Oil-Rat-Kidney-3d-dn	5/251	0,302598294	0,502162697	0	0	1,36909407	1,636545387	RPS19;CLP1;GOLGA7;SEPP1;TPT1
5-Fluoro-2'-Deoxyuridine-54_mg/kg_in_Saline-Rat-Bone_marrow-3d-up	6/312	0,302612591	0,502080633	0	0	1,321702353	1,579833294	C1QA;RPS19;RPS6;RPSA;RPL10A;SEPP1
Amitraz-75_mg/kg_in_CMC-Rat-Liver-1d-dn	6/312	0,302612591	0,501974887	0	0	1,321702353	1,579833294	C1QA;RPS17;RPL32;RPL35A;RPS20;SEPP1
Amitraz-75_mg/kg_in_CMC-Rat-Liver-3d-up	6/312	0,302612591	0,501869186	0	0	1,321702353	1,579833294	CXCL11;RPS6;RPSA;RPL10A;RPL39;TPT1
Chlorambucil-4.5_mg/kg_in_Corn_Oil-Rat-Heart-5d-dn	6/312	0,302612591	0,501763529	0	0	1,321702353	1,579833294	RPS17;RPL32;FUNDC1;RPS6;RPS3A;TPT1
Chlorzoxazone-763_mg/kg_in_CMC-Rat-Liver-3d-dn	6/312	0,302612591	0,501657917	0	0	1,321702353	1,579833294	C1QA;RPL32;RPS19;RPS8;SEPP1;TPT1
Epirubicin-2.7_mg/kg_in_Saline-Rat-Heart-5d-dn	6/312	0,302612591	0,501552349	0	0	1,321702353	1,579833294	EEF1B2;RPS17;ARL6IP5;RPL35A;RAPGEF5;TOMM20
Fluvastatin-5_mg/kg_in_Corn_Oil-Rat-Kidney-1d-dn	6/312	0,302612591	0,501446826	0	0	1,321702353	1,579833294	RPS17;RPL32;RPSA;RPS3A;RPL10A;RPL39
L-Phenylephrine-0.44_mg/kg_in_Saline-Rat-Heart-1d-up	6/312	0,302612591	0,501341347	0	0	1,321702353	1,579833294	RPS19;ARL2BP;RPS8;FUNDC1;ARL6IP5;RAPGEF5
Leflunomide-60_mg/kg_in_Corn_Oil-Rat-Bone_marrow-3d-up	6/312	0,302612591	0,501235912	0	0	1,321702353	1,579833294	C1QA;NKG7;SEPP1;RPL39;GOLGA7;TPT1
Loratadine-62.25_uM_in_DMSO-Rat-Primary_rat_hepatocytes-0.67d-up	6/312	0,302612591	0,501130522	0	0	1,321702353	1,579833294	RPL32;RPS6;RPS20;RPS3A;RPL39;PDRG1
N-Nitrosodiethylamine-1.67_mg/kg_in_Saline-Rat-Kidney-1d-dn	6/312	0,302612591	0,501025176	0	0	1,321702353	1,579833294	EEF1B2;RPL32;RPS19;RPSA;RPL10A;TPT1
Pentoxifylline-1170_mg/kg_in_CMC-Rat-Heart-3d-dn	6/312	0,302612591	0,500919875	0	0	1,321702353	1,579833294	RPS19;ARL2BP;RPS8;FUNDC1;RPS6;SEPP1
Phentolamine-44_mg/kg_in_Saline-Rat-Heart-1d-dn	6/312	0,302612591	0,500814617	0	0	1,321702353	1,579833294	EEF1B2;ARL2BP;RPS8;FUNDC1;ARL6IP5;RPS20
Rofecoxib-775_mg/kg_in_Corn_Oil-Rat-Heart-5d-up	6/312	0,302612591	0,500709404	0	0	1,321702353	1,579833294	CXCL11;ARL2BP;ARL6IP5;RPS20;TPT1;PDRG1
Rolipram-75_mg/kg_in_CMC-Rat-Liver-3d-dn	6/312	0,302612591	0,500604235	0	0	1,321702353	1,579833294	C1QA;RPS17;RPL32;RPS19;RPL35A;SEPP1
Zopiclone-414_mg/kg_in_Water-Rat-Liver-1d-dn	6/312	0,302612591	0,50049911	0	0	1,321702353	1,579833294	EEF1B2;C1QA;RPS17;RPS19;RPS3A;SEPP1
Fluoxetine-39.6_uM_in_DMSO-Rat-Primary_rat_hepatocytes-0.67d-up	8/437	0,304901544	0,504178997	0	0	1,258188052	1,494433443	EEF1B2;RPS8;RPS5;RPS6;RPL35A;RPL39;TPT1;PDRG1
Chlorambucil-0.6_mg/kg_in_Corn_Oil-Rat-Spleen-3d-dn	6/313	0,305144782	0,504475294	0	0	1,317479661	1,563807413	C1QA;RPS19;ARL2BP;RPS8;FEM1B;SEPP1
Daunorubicin-3.25_mg/kg_in_Saline-Rat-Kidney-5d-up	6/313	0,305144782	0,504369423	0	0	1,317479661	1,563807413	EEF1B2;RPS19;ARL2BP;RPS20;RPL10A;RPL39
Dichlorvos-17_mg/kg_in_Water-Rat-Heart-1d-dn	6/313	0,305144782	0,504263596	0	0	1,317479661	1,563807413	RPS19;RPS5;RPSA;RPL10A;SEPP1;PDRG1
Econazole-334_mg/kg_in_Corn_Oil-Rat-Liver-3d-dn	6/313	0,305144782	0,504157814	0	0	1,317479661	1,563807413	EEF1B2;C1QA;RPL32;RPS19;RPSA;SEPP1

Ketorolac-48_mg/kg_in_Water-Rat-Liver-5d-dn	6/313	0,305144782	0,504052076	0	0	1,317479661	1,563807413	C1QA;RPS17;RPL32;RPL35A;SEPP1;TPT1
Lamotrigine-26_mg/kg_in_CMC-Rat-Heart-3d-dn	6/313	0,305144782	0,503946383	0	0	1,317479661	1,563807413	RPS19;ARL2BP;ARL6IP5;SEPP1;RPL39;GOLGA7
N-Nitrosodiethylamine-34_mg/kg_in_Saline-Rat-Bone_marrow-3d-up	6/313	0,305144782	0,503840734	0	0	1,317479661	1,563807413	C1QA;RPS19;CLP1;RPS6;RPS20;SEPP1
Oxymetazoline-0.5_mg/kg_in_Water-Rat-Liver-5d-up	6/313	0,305144782	0,503735129	0	0	1,317479661	1,563807413	RPS8;RPS5;RPS6;RPSA;SEPP1;TPT1
Progesterone-148_uM_in_DMSO-Rat-Primary_rat_hepatocytes-1d-dn	6/313	0,305144782	0,503629568	0	0	1,317479661	1,563807413	C1QA;CXCL11;RPS8;RPS6;ARL6IP5;GOLGA7
Zomepirac-11_mg/kg_in_Saline-Rat-Kidney-5d-up	6/313	0,305144782	0,503524052	0	0	1,317479661	1,563807413	RPS17;RPS19;FUNDC1;RPS20;RPL39;GOLGA7
Cilostazol-1500_mg/kg_in_CMC-Rat-Heart-1d-dn	5/252	0,305432903	0,503893913	0	0	1,363661157	1,617336436	EEF1B2;RPS19;ARL2BP;RPS8;TPT1
Famciclovir-112_mg/kg_in_Saline-Rat-Liver-5d-dn	5/252	0,305432903	0,503788386	0	0	1,363661157	1,617336436	EEF1B2;RPL32;RPS6;RPL10A;RPL39
Flutamide-15_mg/kg_in_Corn_Oil-Rat-Kidney-0.25d-dn	5/252	0,305432903	0,503682903	0	0	1,363661157	1,617336436	ARL2BP;CLP1;RPS8;RPL35A;TOMM20
3-Methylcholanthrene-300_mg/kg_in_CMC-Rat-Liver-3d-up	6/314	0,307680827	0,507283691	0	0	1,313283866	1,547957593	RPS17;RPSA;RPS3A;RPL10A;SEPP1;TPT1
5-Fluoro-2'-Deoxyuridine-54_mg/kg_in_Saline-Rat-Bone_marrow-5d-dn	6/314	0,307680827	0,507177521	0	0	1,313283866	1,547957593	RPS17;NKG7;RPL10A;SEPP1;RPL39;DUSP14
Anastrozole-400_mg/kg_in_CMC-Rat-Liver-5d-up	6/314	0,307680827	0,507071394	0	0	1,313283866	1,547957593	RPS19;RPS5;RPS20;RPSA;RPL10A;TPT1
Azithromycin-225_mg/kg_in_Water-Rat-Kidney-3d-up	6/314	0,307680827	0,506965313	0	0	1,313283866	1,547957593	EEF1B2;ARL2BP;CLP1;RPS8;RPS6;RPS3A
Cerivastatin-0.05_mg/kg_in_Corn_Oil-Rat-Liver-1d-up	6/314	0,307680827	0,506859275	0	0	1,313283866	1,547957593	C1QA;RPS19;RPS6;SEPP1;RPL39;TPT1
D-Limonene-1650_mg/kg_in_Corn_Oil-Rat-Kidney-1d-up	6/314	0,307680827	0,506753282	0	0	1,313283866	1,547957593	ARL2BP;RPS8;ARL6IP5;RPSA;RPS3A;TPT1
Diethylstilbestrol-2.8_mg/kg_in_Corn_Oil-Rat-Bone_marrow-1d-up	6/314	0,307680827	0,506647333	0	0	1,313283866	1,547957593	C1QA;RPS17;RPL10A;SEPP1;RPL39;TPT1
Etoposide-100_mg/kg_in_Corn_Oil-Rat-Liver-5d-dn	6/314	0,307680827	0,506541428	0	0	1,313283866	1,547957593	RPL32;RPS8;RPSA;RPS3A;SEPP1;TPT1
Isoniazid-79_mg/kg_in_Water-Rat-Liver-5d-up	6/314	0,307680827	0,506435568	0	0	1,313283866	1,547957593	C1QA;RPS17;ARL6IP5;RPL35A;RPS20;RPSA
Ketoconazole-25_mg/kg_in_Corn_Oil-Rat-Liver-0.25d-up	6/314	0,307680827	0,506329752	0	0	1,313283866	1,547957593	C1QA;RPL32;RPS19;RPS3A;RPL10A;TPT1
Levodopa-1000_mg/kg_in_Water-Rat-Brain-3d-dn	6/314	0,307680827	0,50622398	0	0	1,313283866	1,547957593	RPL32;RPS19;ARL2BP;RPS6;ARL6IP5;RPL39
Rofecoxib-3_mg/kg_in_Corn_Oil-Rat-Heart-5d-up	6/314	0,307680827	0,506118253	0	0	1,313283866	1,547957593	EEF1B2;RPS8;ARL6IP5;RPS20;RAPGEF5;PDRG1
Torsemide-3_mg/kg_in_Corn_Oil-Rat-Liver-3d-up	6/314	0,307680827	0,506012569	0	0	1,313283866	1,547957593	RPS8;RPS20;RPSA;SEPP1;RPL39;TPT1
Tosufloxacin-2000_mg/kg_in_CMC-Rat-Liver-3d-dn	6/314	0,307680827	0,50590693	0	0	1,313283866	1,547957593	EEF1B2;RPL32;RPS8;ARL6IP5;SEPP1;TPT1
Zaleplon-100_mg/kg_in_Water-Rat-Liver-1d-up	6/314	0,307680827	0,505801335	0	0	1,313283866	1,547957593	RPL32;FEM1B;RPS6;RPS20;RPS3A;RPL39
Amitriptyline-72_uM_in_DMSO-Rat-Primary_rat_hepatocytes-1d-dn	5/253	0,308271517	0,506666625	0	0	1,358271192	1,598378679	EEF1B2;CXCL11;ARL6IP5;RPS20;SEPP1
Catechol-195_mg/kg_in_Saline-Rat-Liver-1d-dn	5/253	0,308271517	0,506560916	0	0	1,358271192	1,598378679	EEF1B2;RPL32;RPS8;RPS6;SEPP1
Atropine-2.3_mg/kg_in_Water-Rat-Liver-0.25d-up	6/315	0,3102206	0,509657372	0	0	1,309114711	1,53228162	EEF1B2;RPL32;RPS8;RPS20;RPS3A;RPL10A
Azaribine-750_mg/kg_in_CMC-Rat-Heart-3d-dn	6/315	0,3102206	0,509551083	0	0	1,309114711	1,53228162	RPS19;FUNDC1;RPS6;ARL6IP5;RPS20;RPSA
Bleomycin_A2-12_mg/kg_in_Saline-Rat-Bone_marrow-5d-dn	6/315	0,3102206	0,509444838	0	0	1,309114711	1,53228162	EEF1B2;RPS17;RPL10A;SEPP1;RPL39;DUSP14
Calcitriol-0.04_mg/kg_in_CMC-Rat-Heart-1d-up	6/315	0,3102206	0,509338637	0	0	1,309114711	1,53228162	EEF1B2;RPS17;RPL32;RPS3A;RPL39;TPT1
Chlorambucil-4.5_mg/kg_in_Corn_Oil-Rat-Bone_marrow-1d-up	6/315	0,3102206	0,509232481	0	0	1,309114711	1,53228162	C1QA;RPS5;RPL10A;SEPP1;RPL39;TPT1
Chlormadinone_Acetate-2000_mg/kg_in_CMC-Rat-Kidney-1d-up	6/315	0,3102206	0,509126369	0	0	1,309114711	1,53228162	EEF1B2;RPL32;RPS19;CLP1;RPS6;RPS20
Desloratadine-57_uM_in_DMSO-Rat-Primary_rat_hepatocytes-0.67d-dn	6/315	0,3102206	0,509020301	0	0	1,309114711	1,53228162	RPS17;RPL32;RPS19;RPS5;RPS3A;PDRG1
Dexamethasone-150_mg/kg_in_Corn_Oil-Rat-Liver-0.25d-up	6/315	0,3102206	0,508914277	0	0	1,309114711	1,53228162	RPS19;RPS6;RPS20;RPSA;RPS3A;RPL10A
Ethanol-3000_mg/kg_in_Saline-Rat-Liver-0.25d-up	6/315	0,3102206	0,508808297	0	0	1,309114711	1,53228162	RPS17;RPL32;RPL35A;RPS3A;RPL10A;TPT1
Famciclovir-1200_mg/kg_in_Saline-Rat-Bone_marrow-5d-dn	6/315	0,3102206	0,508702362	0	0	1,309114711	1,53228162	EEF1B2;RPL32;RPL10A;SEPP1;RPL39;DUSP14
Indinavir-1000_mg/kg_in_CMC-Rat-Kidney-5d-up	6/315	0,3102206	0,50859647	0	0	1,309114711	1,53228162	EEF1B2;RPL32;RPS6;RPL35A;RPS20;RPSA
Leflunomide-60_mg/kg_in_Corn_Oil-Rat-Liver-3d-dn	6/315	0,3102206	0,508490623	0	0	1,309114711	1,53228162	C1QA;RPS19;RPS8;RPS6;RPS20;RPL10A
Aceclofenac-1_mg/kg_in_Corn_Oil-Rat-Kidney-0.25d-up	7/377	0,31026185	0,508452419	0	0	1,276126409	1,493500074	C1QA;RPL32;RPS19;RPL10A;SEPP1;RPL39;TPT1
Labetalol-60_mg/kg_in_Water-Rat-Heart-3d-dn	5/254	0,311113959	0,50974278	0	0	1,352923668	1,579668271	EEF1B2;RPL32;RPS8;RPS3A;TPT1
3-Acetamidophenol-700_mg/kg_in_Corn_Oil-Rat-Liver-3d-up	6/316	0,312763972	0,512339652	0	0	1,304971943	1,516777312	RPS19;RPS20;RPSA;RPS3A;SEPP1;RPL39
Acarbose-2000_mg/kg_in_Water-Rat-Kidney-5d-dn	6/316	0,312763972	0,512233114	0	0	1,304971943	1,516777312	RPS19;CLP1;FEM1B;RPS6;RPS20;SEPP1
Betahistine-1500_mg/kg_in_Water-Rat-Kidney-1d-up	6/316	0,312763972	0,512126621	0	0	1,304971943	1,516777312	RPS17;RPS19;RPS6;RPS20;RPL10A;RPL39
Bithionol-333_mg/kg_in_Corn_Oil-Rat-Kidney-1d-dn	6/316	0,312763972	0,512020172	0	0	1,304971943	1,516777312	RPS17;RPL32;RPSA;RPL10A;SEPP1;RPL39
Cerivastatin-7_mg/kg_in_Corn_Oil-Rat-Liver-3d-dn	6/316	0,312763972	0,511913767	0	0	1,304971943	1,516777312	C1QA;RPS17;CLP1;RPS3A;SEPP1;TPT1
Glyburide-1500_mg/kg_in_CMC-Rat-Heart-5d-dn	6/316	0,312763972	0,511807406	0	0	1,304971943	1,516777312	RPS19;COL5A3;RPSA;RPL10A;SEPP1;RPL39
Irinotecan-5_mg/kg_in_Saline-Rat-Heart-5d-up	6/316	0,312763972	0,51170109	0	0	1,304971943	1,516777312	C1QA;CLP1;RPS20;RPS3A;RPL39;TPT1
Losartan-1000_mg/kg_in_Water-Rat-Kidney-3d-up	6/316	0,312763972	0,511594818	0	0	1,304971943	1,516777312	CXCL11;ARL2BP;CLP1;RPS3A;RAPGEF5;TPT1
Pralidoxime_Chloride-48_mg/kg_in_Saline-Rat-Heart-3d-up	6/316	0,312763972	0,51148859	0	0	1,304971943	1,516777312	COL5A3;RPS6;ARL6IP5;RPS3A;RPL10A;TPT1
Sulfadiazine-1170_mg/kg_in_Water-Rat-Kidney-3d-up	6/316	0,312763972	0,511382406	0	0	1,304971943	1,516777312	ARL2BP;CLP1;RPS8;SEPP1;GOLGA7;TPT1
Sulfisoxazole-250_mg/kg_in_Corn_Oil-Rat-Liver-0.25d-dn	6/316	0,312763972	0,511276266	0	0	1,304971943	1,516777312	RPL32;RPS8;RPS20;RPSA;RPL39;TPT1
Tacrolimus-50_uM_in_DMSO-Rat-Primary_rat_hepatocytes-0.67d-dn	6/316	0,312763972	0,51117017	0	0	1,304971943	1,516777312	RPS20;RPSA;SEPP1;RPL39;SLC16A3;TPT1
Tranilast-300_mg/kg_in_CMC-Rat-Heart-5d-dn	6/316	0,312763972	0,511064118	0	0	1,304971943	1,516777312	RPS17;RPS19;RPS6;ARL6IP5;RPSA;RPS3A
Vecuronium_Bromide-0.005_mg/kg_in_Saline-Rat-Heart-3d-dn	6/316	0,312763972	0,51095811	0	0	1,304971943	1,516777312	EEF1B2;RPS17;ARL6IP5;RPSA;RPS3A;SEPP1
Zomepirac-2.8_mg/kg_in_Saline-Rat-Liver-0.25d-dn	6/316	0,312763972	0,510852146	0	0	1,304971943	1,516777312	RPS19;RPS6;RPS20;RPL10A;SEPP1;RPL39
33'5-Triiodo-L-Thyronine-0.001_mg/kg_in_PEG400-Rat-Heart-3d-up	6/317	0,315310819	0,514905247	0	0	1,300855312	1,50144252	CXCL11;RPS19;ARL2BP;RPS8;ARL6IP5;RPS3A
Carboplatin-6_mg/kg_in_Saline-Rat-Spleen-3d-up	6/317	0,315310819	0,514798509	0	0	1,300855312	1,50144252	RPS19;NKG7;RPL10A;SEPP1;RPL39;TPT1
Losartan-1000_mg/kg_in_Water-Rat-Heart-5d-dn	6/317	0,315310819	0,514691815	0	0	1,300855312	1,50144252	RPS19;RPS8;ARL6IP5;RPS20;RPSA;SEPP1
Oxcarbazepine-1600_mg/kg_in_CMC-Rat-Kidney-3d-dn	6/317	0,315310819	0,514585165	0	0	1,300855312	1,50144252	RPS19;ARL2BP;RPS8;RPS6;RPL39;TPT1
Ranitidine-1500_mg/kg_in_CMC-Rat-Heart-5d-dn	6/317	0,315310819	0,51447856	0	0	1,300855312	1,50144252	EEF1B2;C1QA;RPS8;ARL6IP5;RPS20;TPT1
Rifampin-99_mg/kg_in_CMC-Rat-Kidney-5d-dn	6/317	0,315310819	0,514371998	0	0	1,300855312	1,50144252	RPS17;RPS19;RPS8;RPS6;RPS3A;TPT1
Sulfadiazine-1170_mg/kg_in_Water-Rat-Liver-1d-dn	6/317	0,315310819	0,514265481	0	0	1,300855312	1,50144252	EEF1B2;C1QA;RPS19;RPS20;SEPP1;TPT1
Betahistine-1500_mg/kg_in_Water-Rat-Kidney-3d-dn	5/256	0,316809618	0,516603013	0	0	1,342353952	1,542974466	RPS8;FEM1B;RPS6;RPL39;TPT1
Cromolyn-1500_mg/kg_in_Water-Rat-Kidney-3d-dn	5/256	0,316809618	0,516496078	0	0	1,342353952	1,542974466	RPS17;FEM1B;RPS6;RPL10A;RPL39
Cyclosporin_A-70_mg/kg_in_Corn_Oil-Rat-Kidney-0.25d-dn	5/256	0,316809618	0,516389188	0	0	1,342353952	1,542974466	ARL2BP;ARL6IP5;RPS20;RPSA;GOLGA7
Diclofenac-3.5_mg/kg_in_Corn_Oil-Rat-Kidney-3d-dn	5/256	0,316809618	0,516282341	0	0	1,342353952	1,542974466	EEF1B2;RPS19;RPL10A;RPL39;SEPP1
Benzothiazyl_Disulfide-2600_mg/kg_in_Corn_Oil-Rat-Liver-3d-dn	6/318	0,317861011	0,517888566	0	0	1,296764572	1,486275125	EEF1B2;RPL32;RPL35A;RPS20;SEPP1;TPT1
DActinomycin-0.06_mg/kg_in_Saline-Rat-Bone_marrow-3d-dn	6/318	0,317861011	0,517781453	0	0	1,296764572	1,486275125	CLP1;RPS8;FEM1B;RPS6;RPS20;TPT1
Idebenone-2500_mg/kg_in_CMC-Rat-Liver-3d-up	6/318	0,317861011	0,517674385	0	0	1,296764572	1,486275125	RPS19;RPS8;RPS6;RPSA;RPL10A;RPL39
Insulin-0.56_mg/kg_in_Saline-Rat-Liver-5d-up	6/318	0,317861011	0,517567361	0	0	1,296764572	1,486275125	EEF1B2;C1QA;RPS6;RPL10A;RPL39;GOLGA7
Ipriflavone-1500_mg/kg_in_CMC-Rat-Liver-3d-up	6/318	0,317861011	0,517460382	0	0	1,296764572	1,486275125	RPS17;RPS19;RPSA;RPS3A;RPL10A;TPT1
Mebendazole-714_mg/kg_in_Corn_Oil-Rat-Liver-1d-up	6/318	0,317861011	0,517353446	0	0	1,296764572	1,486275125	C1QA;RPL32;CLP1;RPSA;RPL39;TPT1
Rofecoxib-775_mg/kg_in_Corn_Oil-Rat-Liver-0.25d-up	6/318	0,317861011	0,517246555	0	0	1,296764572	1,486275125	RPS19;RPS8;RPS6;RPL10A;SEPP1;GOLGA7
Simvastatin-15_mg/kg_in_Corn_Oil-Rat-Liver-0.25d-up	6/318	0,317861011	0,517139708	0	0	1,296764572	1,486275125	C1QA;RPS17;RPS6;RPS3A;RPL10A;RPL39
Valproic_Acid-1340_mg/kg_in_Water-Rat-Liver-5d-up	6/318	0,317861011	0,517032905	0	0	1,296764572	1,486275125	EEF1B2;RPL32;RPS3A;RPL10A;RPL39;TPT1
Epinephrine-0.0375_mg/kg_in_Saline-Rat-Heart-3d-up	7/381	0,319558376	0,51968651	0	0	1,262728757	1,4405403	C1QA;RPS17;RPL32;RPS6;RPL35A;RPS3A;RAPGEF5
Doxepin-147_mg/kg_in_CMC-Rat-Heart-3d-up	5/257	0,319662484	0,519748498	0	0	1,337130785	1,524983723	ARL2BP;FUNDC1;RPS3A;RAPGEF5;TPT1
Oxiconazole-1500_mg/kg_in_Corn_Oil-Rat-Liver-3d-up	5/257	0,319662484	0,519641223	0	0	1,337130785	1,524983723	EEF1B2;C1QA;RPSA;RPL39;SEPP1
Acrolein-20_mg/kg_in_Water-Rat-Heart-1d-dn	6/319	0,320414425	0,52075609	0	0	1,29269948	1,471273042	EEF1B2;RPS17;RPL32;RPS19;RPS3A;RPL39
Allyl_Alcohol-30_uM_in_DMSO-Rat-Primary_rat_hepatocytes-1d-dn	6/319	0,320414425	0,520648651	0	0	1,29269948	1,471273042	CXCL11;RPL32;RPS19;RPS5;RPL35A;TPT1
Azathioprine-20_mg/kg_in_Corn_Oil-Rat-Spleen-1d-up	6/319	0,320414425	0,520541256	0	0	1,29269948	1,471273042	C1QA;RPS17;RPL32;RPS6;RPS3A;SEPP1
Azlocillin-800_mg/kg_in_Saline-Rat-Kidney-3d-up	6/319	0,320414425	0,520433906	0	0	1,29269948	1,471273042	RPS17;RPL32;RPS8;RPS5;RPSA;RPL39
Bithionol-333_mg/kg_in_Corn_Oil-Rat-Liver-3d-up	6/319	0,320414425	0,5203266	0	0	1,29269948	1,471273042	EEF1B2;RPS8;RPS20;RPSA;SEPP1;RPL39
Clofibrate-500_mg/kg_in_Corn_Oil-Rat-Kidney-3d-dn	6/319	0,320414425	0,520219338	0	0	1,29269948	1,471273042	EEF1B2;RPL32;RPS6;RPL10A;SEPP1;TPT1
Doxorubicin-3_mg/kg_in_Saline-Rat-Bone_marrow-1d-up	6/319	0,320414425	0,520112121	0	0	1,29269948	1,471273042	C1QA;RPS19;NKG7;RPL35A;RPS20;SEPP1
Glycine-2000_mg/kg_in_CMC-Rat-Kidney-5d-dn	6/319	0,320414425	0,520004948	0	0	1,29269948	1,471273042	RPS17;RPS19;RPS6;RPS3A;RPL10A;SEPP1
Nevirapine-200_mg/kg_in_Saline-Rat-Heart-5d-up	6/319	0,320414425	0,519897818	0	0	1,29269948	1,471273042	RPS6;ARL6IP5;RPSA;RPL10A;RPL39;TPT1
Valeric_Acid-23000_uM_in_DMSO-Rat-Primary_rat_hepatocytes-1d-dn	6/319	0,320414425	0,519790733	0	0	1,29269948	1,471273042	RPS19;ARL2BP;RPS6;ARL6IP5;SEPP1;SLC16A3
Doxazosin-500_mg/kg_in_Water-Rat-Heart-1d-dn	5/258	0,322518474	0,523096273	0	0	1,331948107	1,507225633	EEF1B2;RPS19;RPS6;RPS20;RPL10A
Fluoxetine-52_mg/kg_in_CMC-Rat-Heart-1d-dn	5/258	0,322518474	0,522988573	0	0	1,331948107	1,507225633	RPS17;RPS19;ARL2BP;FEM1B;RPS3A

Flutamide-197_mg/kg_in_Corn_Oil-Rat-Kidney-1d-dn	5/258	0,322518474	0,522880918	0	0	1,331948107	1,507225633	EEF1B2;CLP1;RPS20;RPSA;RPL10A
Ibuprofen-90_mg/kg_in_Corn_Oil-Rat-Liver-3d-up	5/258	0,322518474	0,522773308	0	0	1,331948107	1,507225633	C1QA;RPS17;RPS3A;GOLGA7;SEPP1
Tocainide-67_mg/kg_in_Corn_Oil-Rat-Liver-0.25d-up	5/258	0,322518474	0,522665741	0	0	1,331948107	1,507225633	RPS8;RPSA;SEPP1;TPT1;PDRG1
6-Mercaptopurine-25_mg/kg_in_Corn_Oil-Rat-Liver-3d-up	6/320	0,322970933	0,523291313	0	0	1,288659794	1,456434214	RPS19;RPS6;RPS20;SEPP1;RPL39;GOLGA7
Carmustine-4_mg/kg_in_Corn_Oil-Rat-Liver-3d-up	6/320	0,322970933	0,523183684	0	0	1,288659794	1,456434214	RPS6;RPS20;RPS3A;RPL10A;SEPP1;GOLGA7
Doxofylline-483_mg/kg_in_Water-Rat-Heart-5d-dn	6/320	0,322970933	0,523076099	0	0	1,288659794	1,456434214	EEF1B2;RPL32;RPS8;RPL35A;RPS3A;TOMM20
Letrozole-250_mg/kg_in_Corn_Oil-Rat-Liver-3d-dn	6/320	0,322970933	0,522968559	0	0	1,288659794	1,456434214	EEF1B2;C1QA;RPS17;RPS6;RPS3A;GOLGA7
Miconazole-920_mg/kg_in_Corn_Oil-Rat-Liver-0.25d-dn	6/320	0,322970933	0,522861063	0	0	1,288659794	1,456434214	EEF1B2;C1QA;RPSA;SEPP1;RPL39;TPT1
Piroxicam-14_mg/kg_in_CMC-Rat-Kidney-3d-up	6/320	0,322970933	0,522753611	0	0	1,288659794	1,456434214	RPS17;ARL6IP5;RPL13A;RPS20;RPSA;RPL39
Rofecoxib-3_mg/kg_in_Corn_Oil-Rat-Liver-0.25d-up	6/320	0,322970933	0,522646203	0	0	1,288659794	1,456434214	CXCL11;RPS19;RPS6;RPL10A;SEPP1;GOLGA7
Cisapride-250_mg/kg_in_Corn_Oil-Rat-Intestine-1d-up	5/259	0,325377414	0,526432316	0	0	1,326805451	1,489696687	RPL32;RPS8;RPS6;SEPP1;SLC16A3
Dexibuprofen-239_mg/kg_in_CMC-Rat-Kidney-5d-dn	5/259	0,325377414	0,526324197	0	0	1,326805451	1,489696687	EEF1B2;RPS17;RPS6;RPS20;RPS3A
Fluphenazine-2.5_mg/kg_in_Corn_Oil-Rat-Liver-1d-dn	5/259	0,325377414	0,526216122	0	0	1,326805451	1,489696687	C1QA;RPS17;RPS6;RPS3A;RPL39
Nabumetone-2500_mg/kg_in_Corn_Oil-Rat-Kidney-1d-dn	5/259	0,325377414	0,526108092	0	0	1,326805451	1,489696687	EEF1B2;RPS8;GOLGA7;SEPP1;TPT1
Valproic_Acid-1340_mg/kg_in_Water-Rat-Liver-3d-dn	5/259	0,325377414	0,526000106	0	0	1,326805451	1,489696687	EEF1B2;RPL32;RPL39;SEPP1;TPT1
Aminoglutethimide-350_mg/kg_in_CMC-Rat-Liver-3d-up	6/321	0,325530411	0,526139445	0	0	1,284645277	1,441756616	RPS17;RPS19;RPS20;RPSA;RPL10A;RPL39
Benazepril-1750_mg/kg_in_CMC-Rat-Kidney-1d-up	6/321	0,325530411	0,526031497	0	0	1,284645277	1,441756616	RPS17;RPL32;RPS6;RPS20;RPL39;TPT1
Cisplatin-63_uM_in_DMSO-Rat-Primary_rat_hepatocytes-1d-dn	6/321	0,325530411	0,525923593	0	0	1,284645277	1,441756616	RPS19;RPS3A;SEPP1;RPL39;SLC16A3;TPT1
Colistin-121_mg/kg_in_Water-Rat-Liver-3d-up	6/321	0,325530411	0,525815733	0	0	1,284645277	1,441756616	C1QA;RPS6;RPSA;RPS3A;RPL10A;SEPP1
Doxorubicin-3_mg/kg_in_Saline-Rat-Kidney-3d-up	6/321	0,325530411	0,525707918	0	0	1,284645277	1,441756616	EEF1B2;RPL32;RPS8;RPS6;RPS3A;RPL10A
Meloxicam-0.6_mg/kg_in_Corn_Oil-Rat-Kidney-1d-up	6/321	0,325530411	0,525600147	0	0	1,284645277	1,441756616	RPS19;RPS8;RPS5;RPS6;RPL10A;TPT1
Quinapril-2500_mg/kg_in_CMC-Rat-Kidney-3d-dn	6/321	0,325530411	0,52549242	0	0	1,284645277	1,441756616	EEF1B2;CLP1;RPS8;RPS3A;SEPP1;TPT1
Sulfaphenazole-1695_mg/kg_in_Water-Rat-Liver-3d-up	6/321	0,325530411	0,525384737	0	0	1,284645277	1,441756616	RPS17;RPL32;RPS8;RPS5;RPS3A;TPT1
3-Chloroaniline-200_mg/kg_in_CMC-Rat-Kidney-3d-dn	6/322	0,328092732	0,52941167	0	0	1,280655696	1,427238253	EEF1B2;RPS19;RPS8;RPS6;RPSA;TOMM20
Amiloride-22_mg/kg_in_CMC-Rat-Heart-5d-dn	6/322	0,328092732	0,529303228	0	0	1,280655696	1,427238253	RPS19;COL5A3;RPS20;RPL10A;SEPP1;RPL39
Amiodarone-147_mg/kg_in_CMC-Rat-Kidney-1d-dn	6/322	0,328092732	0,529194831	0	0	1,280655696	1,427238253	RPL32;ARL2BP;RPS8;RPS20;SEPP1;PDRG1
Cisapride-250_mg/kg_in_Corn_Oil-Rat-Intestine-5d-dn	6/322	0,328092732	0,529086478	0	0	1,280655696	1,427238253	EEF1B2;ARL6IP5;RPL13A;RPSA;SEPP1;RPL39
Clozapine-95_mg/kg_in_CMC-Rat-Heart-5d-dn	6/322	0,328092732	0,52897817	0	0	1,280655696	1,427238253	COL5A3;RPS20;RPSA;RPL10A;RPL39;TPT1
Etoposide-188_mg/kg_in_Corn_Oil-Rat-Liver-1d-up	6/322	0,328092732	0,528869906	0	0	1,280655696	1,427238253	C1QA;RPS17;RPL32;RPS6;RPS20;RPL39
Quinapril-2500_mg/kg_in_CMC-Rat-Kidney-1d-dn	6/322	0,328092732	0,528761686	0	0	1,280655696	1,427238253	EEF1B2;RPS6;RPSA;SEPP1;RPL39;GOLGA7
Ergocalciferol-15_mg/kg_in_CMC-Rat-Liver-3d-up	5/260	0,328239132	0,528889403	0	0	1,321702353	1,47239344	CLP1;RPS8;RPS3A;RPL39;TPT1
Modafinil-17.5_mg/kg_in_Saline-Rat-Liver-5d-dn	5/260	0,328239132	0,528781223	0	0	1,321702353	1,47239344	C1QA;RPSA;RPL39;SEPP1;TPT1
Rofecoxib-775_mg/kg_in_Corn_Oil-Rat-Kidney-3d-dn	5/260	0,328239132	0,528673088	0	0	1,321702353	1,47239344	RPS19;RPS6;RPL10A;RPL39;SEPP1
Sparteine-225_mg/kg_in_Water-Rat-Heart-1d-dn	5/260	0,328239132	0,528564997	0	0	1,321702353	1,47239344	EEF1B2;ARL2BP;RPS8;FUNDC1;RPSA
24-Diaminophenol-60_mg/kg_in_Water-Rat-Kidney-3d-up	6/323	0,330657773	0,532350904	0	0	1,276690817	1,412877158	CLP1;RPS8;FUNDC1;RPS20;SEPP1;TPT1
Amiodarone-147_mg/kg_in_CMC-Rat-Heart-5d-dn	6/323	0,330657773	0,532242105	0	0	1,276690817	1,412877158	COL5A3;RPS6;RPS3A;SEPP1;RPL39;TPT1
Bisphenol_A-100_mg/kg_in_Corn_Oil-Rat-Spleen-3d-dn	6/323	0,330657773	0,532133351	0	0	1,276690817	1,412877158	C1QA;RPSA;RPL10A;SEPP1;RPL39;TPT1
Chlormadinone_Acetate-2000_mg/kg_in_CMC-Rat-Kidney-5d-up	6/323	0,330657773	0,532024642	0	0	1,276690817	1,412877158	RPL32;RPS19;CLP1;RPS20;SEPP1;RPL39
Digoxin-11_mg/kg_in_Corn_Oil-Rat-Heart-3d-up	6/323	0,330657773	0,531915977	0	0	1,276690817	1,412877158	EEF1B2;RPS19;ARL6IP5;RPS20;RPL10A;TPT1
Diphenidol-300_mg/kg_in_Water-Rat-Heart-5d-dn	6/323	0,330657773	0,531807356	0	0	1,276690817	1,412877158	EEF1B2;C1QA;RPS8;RPS20;RPSA;TPT1
Fluphenazine-22_mg/kg_in_Corn_Oil-Rat-Heart-5d-up	6/323	0,330657773	0,531698779	0	0	1,276690817	1,412877158	EEF1B2;RPS8;FUNDC1;RPS20;SEPP1;TPT1
Ifosfamide-143_mg/kg_in_Saline-Rat-Spleen-5d-dn	6/323	0,330657773	0,531590247	0	0	1,276690817	1,412877158	EEF1B2;RPS17;RPS5;RPS20;RPSA;RPL10A
Isotretinoin-13_mg/kg_in_Corn_Oil-Rat-Liver-3d-up	6/323	0,330657773	0,53148176	0	0	1,276690817	1,412877158	RPS19;RPS6;RPS20;RPS3A;RPL39;TPT1
Ivermectin-7.5_mg/kg_in_CMC-Rat-Liver-1d-up	6/323	0,330657773	0,531373316	0	0	1,276690817	1,412877158	RPS17;RPL35A;RPS20;RPSA;RPL39;TPT1
N-Nitrosodiethylamine-1.67_mg/kg_in_Saline-Rat-Kidney-3d-dn	6/323	0,330657773	0,531264917	0	0	1,276690817	1,412877158	EEF1B2;RPS8;RPSA;RPL10A;RPL39;TPT1
Oxfendazole-1500_mg/kg_in_CMC-Rat-Kidney-3d-up	6/323	0,330657773	0,531156562	0	0	1,276690817	1,412877158	EEF1B2;RPS17;CLP1;RPS3A;SEPP1;RPL39
Sulfinpyrazone-269_mg/kg_in_CMC-Rat-Kidney-1d-dn	6/323	0,330657773	0,531048251	0	0	1,276690817	1,412877158	EEF1B2;RPL32;RPL35A;RPS20;SEPP1;TPT1
Catechol-40_mg/kg_in_Saline-Rat-Spleen-0.25d-dn	5/261	0,331103454	0,531655617	0	0	1,316638359	1,455312508	CXCL11;CLP1;FUNDC1;RPS20;TPT1
Cyclosporin_A-8.5_uM_in_DMSO-Rat-Primary_rat_hepatocytes-1d-up	5/261	0,331103454	0,531547249	0	0	1,316638359	1,455312508	EEF1B2;RPS17;RPS20;RPS3A;PDRG1
Lincomycin-1200_mg/kg_in_CMC-Rat-Kidney-3d-dn	5/261	0,331103454	0,531438925	0	0	1,316638359	1,455312508	EEF1B2;RPL32;RPS19;CLP1;RPS3A
Metoclopramide-185_mg/kg_in_CMC-Rat-Heart-1d-dn	5/261	0,331103454	0,531330645	0	0	1,316638359	1,455312508	RPL32;RPS19;COL5A3;RPS3A;GOLGA7
Rofecoxib-250_mg/kg_in_Corn_Oil-Rat-Liver-3d-up	5/261	0,331103454	0,531222409	0	0	1,316638359	1,455312508	RPS17;CLP1;RPS3A;GOLGA7;SEPP1
Valproic_Acid-235_mg/kg_in_Water-Rat-Liver-3d-up	7/386	0,331246055	0,531342959	0	0	1,246372167	1,377108894	C1QA;RPS19;CLP1;RPS6;RPSA;RPL10A;SEPP1
Aminocaproic_Acid-2000_mg/kg_in_Water-Rat-Kidney-1d-dn	6/324	0,333225409	0,534409147	0	0	1,272750414	1,398671394	ARL2BP;RPS20;RPS3A;SEPP1;RPL39;TPT1
Azithromycin-225_mg/kg_in_Water-Rat-Liver-3d-up	6/324	0,333225409	0,53430035	0	0	1,272750414	1,398671394	EEF1B2;RPS17;RPL32;RPS8;RPS6;RPS3A
Cyclosporin_A-70_mg/kg_in_Corn_Oil-Rat-Liver-0.25d-up	6/324	0,333225409	0,534191598	0	0	1,272750414	1,398671394	RPL32;RPS6;RPSA;RPL10A;RPL39;TPT1
Ibuprofen-275_mg/kg_in_Corn_Oil-Rat-Liver-1d-dn	6/324	0,333225409	0,53408289	0	0	1,272750414	1,398671394	RPS17;RPS6;RPS20;RPS3A;RPL10A;GOLGA7
Ketorolac-48_mg/kg_in_Water-Rat-Liver-1d-dn	6/324	0,333225409	0,533974226	0	0	1,272750414	1,398671394	C1QA;RPS17;RPSA;RPL10A;SEPP1;GOLGA7
Lipopolysaccharide_E._Coli_O55:B5-1.25_mg/kg_in_Saline-Rat-Liver-3d-dn	6/324	0,333225409	0,533865606	0	0	1,272750414	1,398671394	EEF1B2;RPS17;RPS20;RPS3A;RPL39;GOLGA7
Spironolactone-300_mg/kg_in_CMC-Rat-Kidney-5d-dn	6/324	0,333225409	0,533757031	0	0	1,272750414	1,398671394	RPL32;RPS19;RPS8;RPS5;SEPP1;TPT1
Tamoxifen-64_mg/kg_in_Corn_Oil-Rat-Liver-3d-up	6/324	0,333225409	0,5336485	0	0	1,272750414	1,398671394	RPS19;RPS6;RPS20;SEPP1;RPL39;GOLGA7
Cobalt_(II)_Chloride-10_mg/kg_in_Saline-Rat-Kidney-14d-up	5/262	0,33397021	0,534732542	0	0	1,311613022	1,438450568	RPL32;ARL2BP;RPS20;RPS3A;RPL39
Mexiletine-350_mg/kg_in_CMC-Rat-Heart-1d-dn	5/262	0,33397021	0,534623856	0	0	1,311613022	1,438450568	RPS19;RPS8;FEM1B;COL5A3;TPT1
Nitrobenzene-225_mg/kg_in_CMC-Rat-Kidney-1d-up	5/262	0,33397021	0,534515215	0	0	1,311613022	1,438450568	CLP1;RPS20;RPS3A;RPL10A;TPT1
Pioglitazone-1500_mg/kg_in_Corn_Oil-Rat-Liver-3d-dn	5/262	0,33397021	0,534406618	0	0	1,311613022	1,438450568	RPS17;CLP1;RPS3A;RPL39;SEPP1
Salicylic_Acid-223_mg/kg_in_Corn_Oil-Rat-Liver-5d-up	5/262	0,33397021	0,534298065	0	0	1,311613022	1,438450568	RPS19;CLP1;RPS6;GOLGA7;SEPP1
Aconitine-0.9_mg/kg_in_CMC-Rat-Heart-5d-dn	6/325	0,335795516	0,537109155	0	0	1,268834259	1,38461905	EEF1B2;RPS19;ARL2BP;RPS8;RPS20;RAPGEF5
Amikacin-82_mg/kg_in_Saline-Rat-Bone_marrow-3d-up	6/325	0,335795516	0,537000098	0	0	1,268834259	1,38461905	C1QA;RPL32;NKG7;RPS3A;SLC16A3;TPT1
Benzethonium_Chloride-5_mg/kg_in_Water-Rat-Heart-5d-up	6/325	0,335795516	0,536891084	0	0	1,268834259	1,38461905	C1QA;ARL2BP;FEM1B;RPS6;RPL10A;GOLGA7
Cephalexin-2500_mg/kg_in_CMC-Rat-Kidney-3d-up	6/325	0,335795516	0,536782115	0	0	1,268834259	1,38461905	RPS17;FEM1B;RAPGEF5;SEPP1;RPL39;TPT1
Citalopram-90_mg/kg_in_Corn_Oil-Rat-Liver-3d-up	6/325	0,335795516	0,53667319	0	0	1,268834259	1,38461905	RPS19;RPS6;RPS20;SEPP1;RPL39;GOLGA7
Lead(IV)_Acetate-600_mg/kg_in_Saline-Rat-Liver-3d-dn	6/325	0,335795516	0,536564309	0	0	1,268834259	1,38461905	RPS19;RPS6;RPS3A;SEPP1;RPL39;TPT1
Oxymetazoline-0.5_mg/kg_in_Water-Rat-Heart-5d-dn	6/325	0,335795516	0,536455473	0	0	1,268834259	1,38461905	RPS8;FUNDC1;RPS6;RPS20;RPS3A;TPT1
Salicylic_Acid-223_mg/kg_in_Corn_Oil-Rat-Liver-1d-up	6/325	0,335795516	0,53634668	0	0	1,268834259	1,38461905	EEF1B2;RPS17;RPS6;RPS20;RPSA;RPL39
Venlafaxine-320_mg/kg_in_Water-Rat-Liver-3d-dn	6/325	0,335795516	0,536237932	0	0	1,268834259	1,38461905	CLP1;FEM1B;RPS3A;RPL10A;RPL39;TPT1
Mercuric_Chloride-2.5_mg/kg_in_Water-Rat-Kidney-1d-up	7/388	0,335939529	0,536359158	0	0	1,239947568	1,352564702	ARL2BP;CLP1;RPS8;RPS3A;SEPP1;TOMM20;TPT1
Catechol-195_mg/kg_in_Saline-Rat-Bone_marrow-3d-dn	5/263	0,336839229	0,537686617	0	0	1,3066259	1,421804357	EEF1B2;ARL2BP;RPS20;RPL10A;TPT1
Cromolyn-1500_mg/kg_in_Water-Rat-Kidney-1d-dn	5/263	0,336839229	0,537577663	0	0	1,3066259	1,421804357	ARL2BP;RPS20;RPL39;GOLGA7;SEPP1
Mannitol-1000_mg/kg_in_Saline-Rat-Kidney-1d-dn	5/263	0,336839229	0,537468753	0	0	1,3066259	1,421804357	EEF1B2;ARL2BP;CLP1;RPL39;TPT1
Primaquine-45_mg/kg_in_CMC-Rat-Liver-1d-dn	5/263	0,336839229	0,537359888	0	0	1,3066259	1,421804357	EEF1B2;RPS17;RPL32;RPSA;RPS3A
Quetiapine-150_uM_in_DMSO-Rat-Primary_rat_hepatocytes-1d-up	5/263	0,336839229	0,537251066	0	0	1,3066259	1,421804357	EEF1B2;RPS17;RPS6;RPS3A;RPL10A
Azathioprine-20_mg/kg_in_Corn_Oil-Rat-Kidney-0.25d-up	6/326	0,338367969	0,539580103	0	0	1,264942129	1,370718246	RPS17;RPL32;RPS6;RPL35A;RPS20;RPS3A
Celecoxib-400_mg/kg_in_Corn_Oil-Rat-Liver-1d-dn	6/326	0,338367969	0,539470876	0	0	1,264942129	1,370718246	EEF1B2;RPL32;RPS19;RPS6;RPL39;TPT1
Cisplatin-2_mg/kg_in_Saline-Rat-Bone_marrow-3d-up	6/326	0,338367969	0,539361693	0	0	1,264942129	1,370718246	EEF1B2;RPS19;RPS8;FUNDC1;RPS20;TPT1
NN-Dimethylformamide-140_mg/kg_in_Saline-Rat-Kidney-0.25d-up	6/326	0,338367969	0,539252555	0	0	1,264942129	1,370718246	RPS5;RPL35A;RPS20;SEPP1;TOMM20;TPT1
Busulfan-500_uM_in_DMSO-Rat-Primary_rat_hepatocytes-1d-dn	5/264	0,339710341	0,541282348	0	0	1,301676559	1,405370668	RPS8;RPS5;RPS20;RPSA;SEPP1
Cytarabine-23_mg/kg_in_Saline-Rat-Liver-1d-dn	5/264	0,339710341	0,541172866	0	0	1,301676559	1,405370668	RPS19;RPSA;RPS3A;RPL10A;SEPP1
Indomethacin-4.5_mg/kg_in_Water-Rat-Liver-0.25d-dn	5/264	0,339710341	0,541063427	0	0	1,301676559	1,405370668	C1QA;RPS8;RPS6;RPS3A;SEPP1
Leflunomide-60_mg/kg_in_Corn_Oil-Rat-Kidney-1d-dn	5/264	0,339710341	0,540954033	0	0	1,301676559	1,405370668	EEF1B2;RPL32;RPSA;RPL39;SEPP1
Chlorpyrifos-82_mg/kg_in_CMC-Rat-Kidney-3d-dn	6/327	0,340942647	0,542806608	0	0	1,261073804	1,356967125	EEF1B2;RPS19;ARL2BP;RPS6;RPS20;RPL39

Ciprofloxacin-450_mg/kg_in_Corn_Oil-Rat-Liver-1d-up	6/327	0,340942647	0,542696906	0	0	1,261073804	1,356967125	C1QA;RPSA;SEPP1;RPL39;GOLGA7;TPT1
Fluvastatin-5_mg/kg_in_Corn_Oil-Rat-Liver-1d-up	6/327	0,340942647	0,542587248	0	0	1,261073804	1,356967125	EEF1B2;RPS19;SEPP1;RPL39;GOLGA7;TPT1
Lomustine-4.2_mg/kg_in_Corn_Oil-Rat-Liver-3d-up	6/327	0,340942647	0,542477634	0	0	1,261073804	1,356967125	RPS20;RPS3A;RAPGEF5;SEPP1;RPL39;TPT1
Betahistine-1500_mg/kg_in_Water-Rat-Kidney-5d-dn	5/265	0,342583378	0,544978123	0	0	1,296764572	1,389146353	CLP1;RPS8;FEM1B;R3HCC1;SEPP1
Busulfan-9_mg/kg_in_Corn_Oil-Rat-Bone_marrow-3d-dn	5/265	0,342583378	0,544868071	0	0	1,296764572	1,389146353	RPS19;FEM1B;RPS6;ARL6IP5;NKG7
Cisapride-250_mg/kg_in_Corn_Oil-Rat-Intestine-0.25d-up	5/265	0,342583378	0,544758063	0	0	1,296764572	1,389146353	RPS8;FEM1B;RPS6;RPS20;RPS3A
Fenbendazole-375_mg/kg_in_Corn_Oil-Rat-Liver-5d-dn	5/265	0,342583378	0,5446481	0	0	1,296764572	1,389146353	EEF1B2;RPS6;RPS3A;RPL39;SEPP1
Ibuprofen-54_mg/kg_in_Corn_Oil-Rat-Kidney-0.25d-dn	5/265	0,342583378	0,544538181	0	0	1,296764572	1,389146353	RPL32;RPS8;RPS5;RPL35A;TOMM20
Nisoldipine-1125_mg/kg_in_Water-Rat-Liver-5d-dn	5/265	0,342583378	0,544428307	0	0	1,296764572	1,389146353	C1QA;RPSA;RPL10A;RPL39;SEPP1
Penicillamine-852_mg/kg_in_Water-Rat-Liver-3d-dn	5/265	0,342583378	0,544318477	0	0	1,296764572	1,389146353	RPS17;RPL32;RPSA;RPL39;TPT1
1-Naphthyl_Isothiocyanate-60_mg/kg_in_Corn_Oil-Rat-Bone_marrow-1d-up	6/328	0,343519426	0,545695644	0	0	1,257229067	1,343363861	C1QA;RPS6;NKG7;RPS3A;RPL10A;RPL39
Azauridine-1500_mg/kg_in_CMC-Rat-Kidney-3d-up	6/328	0,343519426	0,545585602	0	0	1,257229067	1,343363861	EEF1B2;RPS17;RPS19;RPSA;RPS3A;SEPP1
Ebastine-2000_mg/kg_in_Water-Rat-Heart-5d-dn	6/328	0,343519426	0,545475605	0	0	1,257229067	1,343363861	EEF1B2;COL5A3;ARL6IP5;RPS20;RPSA;TPT1
Glycine-2000_mg/kg_in_CMC-Rat-Kidney-3d-up	6/328	0,343519426	0,545365652	0	0	1,257229067	1,343363861	EEF1B2;RPS17;ARL6IP5;RPL13A;RPSA;RPL39
Lead_(II)_Acetate-600_mg/kg_in_Saline-Rat-Bone_marrow-1d-up	6/328	0,343519426	0,545255744	0	0	1,257229067	1,343363861	ARL2BP;CLP1;RPS8;FUNDC1;ARL6IP5;RPS20
Neomycin-877_mg/kg_in_Corn_Oil-Rat-Liver-1d-up	6/328	0,343519426	0,54514588	0	0	1,257229067	1,343363861	RPS19;RPS6;RPS20;RPS3A;RPL39;GOLGA7
Nizatidine-1653_mg/kg_in_Corn_Oil-Rat-Heart-5d-up	6/328	0,343519426	0,54503606	0	0	1,257229067	1,343363861	CXCL11;FUNDC1;RPS20;RAPGEF5;SEPP1;TPT1
Ofloxacin-3000_mg/kg_in_Water-Rat-Heart-5d-dn	6/328	0,343519426	0,544926284	0	0	1,257229067	1,343363861	EEF1B2;RPS8;COL5A3;RPSA;RPS3A;TPT1
Tolazamide-1500_mg/kg_in_CMC-Rat-Heart-5d-dn	6/328	0,343519426	0,544816553	0	0	1,257229067	1,343363861	ARL2BP;FUNDC1;RPS6;RPS3A;SEPP1;TPT1
Atenolol-1000_mg/kg_in_CMC-Rat-Heart-1d-up	5/266	0,345458173	0,547781068	0	0	1,291889518	1,373128319	CXCL11;FUNDC1;ARL6IP5;RPS3A;TPT1
Genistein-20_mg/kg_in_Corn_Oil-Rat-Liver-0.25d-dn	5/266	0,345458173	0,547670807	0	0	1,291889518	1,373128319	RPL32;RPS19;RPL39;SEPP1;TPT1
Ketamine-30_mg/kg_in_Saline-Rat-Heart-3d-up	5/266	0,345458173	0,547560589	0	0	1,291889518	1,373128319	CLP1;FUNDC1;RPS20;SEPP1;TPT1
Carmustine-16_mg/kg_in_Corn_Oil-Rat-Spleen-5d-dn	6/329	0,346098184	0,548464648	0	0	1,253407702	1,329906651	EEF1B2;RPS17;RPS6;RPL35A;RPL10A;RPL39
Levodopa-1000_mg/kg_in_Water-Rat-Brain-5d-dn	6/329	0,346098184	0,548354315	0	0	1,253407702	1,329906651	RPL32;FEM1B;RPS6;SEPP1;RPL39;PDRG1
N-Nitrosodiethylamine-34_mg/kg_in_Saline-Rat-Liver-0.25d-up	6/329	0,346098184	0,548244027	0	0	1,253407702	1,329906651	C1QA;CLP1;RPSA;RPS3A;SEPP1;TPT1
Amoxicillin-1100_mg/kg_in_Water-Rat-Liver-3d-dn	5/267	0,348334557	0,551675643	0	0	1,28705098	1,357313526	RPS19;CLP1;RPS6;RPS20;RPL10A
Etoposide-125_uM_in_DMSO-Rat-Primary_rat_hepatocytes-1d-up	5/267	0,348334557	0,551564731	0	0	1,28705098	1,357313526	CXCL11;ARL2BP;CLP1;FEM1B;TPT1
Rofecoxib-3_mg/kg_in_Corn_Oil-Rat-Kidney-0.25d-dn	5/267	0,348334557	0,551453864	0	0	1,28705098	1,357313526	CLP1;RPS8;RPS6;RPS3A;RPL10A
Sotalol-6666_uM_in_DMSO-Rat-Primary_rat_hepatocytes-1d-up	5/267	0,348334557	0,551343041	0	0	1,28705098	1,357313526	RPS17;RPS8;RPS6;RPL10A;SLC16A3
Thiamphenicol-1500_mg/kg_in_Water-Rat-Kidney-5d-dn	5/267	0,348334557	0,551232263	0	0	1,28705098	1,357313526	CLP1;RPS20;GOLGA7;SEPP1;TPT1
Vincristine-0.05_mg/kg_in_Saline-Rat-Bone_marrow-0.25d-up	5/267	0,348334557	0,551121529	0	0	1,28705098	1,357313526	RPS17;RPL32;RPS8;RPS6;NKG7
Bezafibrate-100_mg/kg_in_Corn_Oil-Rat-Liver-0.25d-dn	6/330	0,3486788	0,551555378	0	0	1,249609497	1,316593721	RPSA;RPS3A;RPL10A;SEPP1;RPL39;TPT1
Chlorpropamide-269_mg/kg_in_CMC-Rat-Heart-3d-dn	6/330	0,3486788	0,551444624	0	0	1,249609497	1,316593721	RPS19;RPS8;RPS20;RPSA;RPL10A;RPL39
Kanamycin-6584_uM_in_DMSO-Rat-Primary_rat_hepatocytes-1d-dn	6/330	0,3486788	0,551333914	0	0	1,249609497	1,316593721	C1QA;RPS19;RPS8;RPS5;RPL10A;GOLGA7
Lead_(II)_Acetate-600_mg/kg_in_Saline-Rat-Bone_marrow-3d-up	6/330	0,3486788	0,551223249	0	0	1,249609497	1,316593721	C1QA;RPS8;RPS20;RPS3A;RPL10A;TPT1
Sodium_Orthovanadate-83_mg/kg_in_CMC-Rat-Kidney-1d-dn	6/330	0,3486788	0,551112628	0	0	1,249609497	1,316593721	RPS19;RPS6;RPL35A;RPSA;RPL10A;TOMM20
Sodium_Selenite-2_mg/kg_in_Water-Rat-Kidney-3d-up	6/330	0,3486788	0,551002052	0	0	1,249609497	1,316593721	ARL2BP;CLP1;RPS8;RPS3A;SEPP1;TPT1
Dexamethasone-456_uM_in_DMSO-Rat-Primary_rat_hepatocytes-1d-up	5/268	0,351212366	0,554894402	0	0	1,282248551	1,341698989	RPS17;RPL32;RPS8;RPS6;RPS20
Ibuprofen-275_mg/kg_in_Corn_Oil-Rat-Liver-3d-up	5/268	0,351212366	0,554783111	0	0	1,282248551	1,341698989	C1QA;RPS17;RPS3A;GOLGA7;SEPP1
Loperamide-47_mg/kg_in_CMC-Rat-Intestine-1d-up	5/268	0,351212366	0,554671865	0	0	1,282248551	1,341698989	RPL32;RPS8;FEM1B;SLC16A3;TPT1
Torsemide-110_mg/kg_in_Corn_Oil-Rat-Liver-5d-dn	5/268	0,351212366	0,554560664	0	0	1,282248551	1,341698989	C1QA;RPS6;RPSA;RPL10A;SEPP1
Meloxicam-33_mg/kg_in_Corn_Oil-Rat-Liver-5d-dn	6/331	0,351261151	0,554526523	0	0	1,245834242	1,303423319	EEF1B2;RPSA;RPS3A;RPL10A;SEPP1;RPL39
Sulfaphenazole-1695_mg/kg_in_Water-Rat-Kidney-3d-up	6/331	0,351261151	0,554415396	0	0	1,245834242	1,303423319	EEF1B2;CLP1;RPS8;RPS3A;SEPP1;TPT1
Zomepirac-11_mg/kg_in_Saline-Rat-Kidney-1d-up	6/331	0,351261151	0,554304313	0	0	1,245834242	1,303423319	RPS17;RPL32;RPS19;RPL35A;RPS20;RPL39
Clomipramine-7_uM_in_DMSO-Rat-Primary_rat_hepatocytes-0.67d-dn	4/206	0,352131547	0,55556652	0	0	1,334534414	1,392920907	RPS17;ARL6IP5;RPS20;RPS3A
Finasteride-25_mg/kg_in_Corn_Oil-Rat-Liver-5d-up	6/332	0,353845117	0,55815825	0	0	1,242081729	1,29039372	RPS19;RPS20;RPSA;RPL39;GOLGA7;TPT1
Foscarnet-350_mg/kg_in_Saline-Rat-Bone_marrow-1d-up	6/332	0,353845117	0,558046484	0	0	1,242081729	1,29039372	RPS17;FEM1B;RPS3A;RPL10A;GOLGA7;TPT1
Cyclosporin_A-350_mg/kg_in_Corn_Oil-Rat-Spleen-1d-dn	5/269	0,354091434	0,55832315	0	0	1,277481828	1,326281775	EEF1B2;RPS19;RPS8;RPS6;RPL13A
Hydroxyurea-400_mg/kg_in_Saline-Rat-Liver-3d-dn	5/269	0,354091434	0,558211396	0	0	1,277481828	1,326281775	C1QA;CLP1;RPS8;SEPP1;TPT1
Lansoprazole-600_mg/kg_in_CMC-Rat-Liver-3d-dn	5/269	0,354091434	0,558099687	0	0	1,277481828	1,326281775	C1QA;CLP1;RPL10A;SEPP1;TPT1
Lorazepam-2000_mg/kg_in_CMC-Rat-Liver-3d-dn	5/269	0,354091434	0,557988022	0	0	1,277481828	1,326281775	EEF1B2;C1QA;RPS17;RPS3A;SEPP1
Nadolol-1500_mg/kg_in_CMC-Rat-Heart-1d-dn	5/269	0,354091434	0,557876402	0	0	1,277481828	1,326281775	RPS19;ARL2BP;RPS8;FEM1B;RPS20
Zomepirac-11_mg/kg_in_Saline-Rat-Kidney-1d-dn	5/269	0,354091434	0,557764827	0	0	1,277481828	1,326281775	RPS8;RPS6;RPL10A;SEPP1;TPT1
Glycidol-210_mg/kg_in_Water-Rat-Kidney-1d-dn	4/207	0,355432343	0,559765074	0	0	1,328087388	1,373800638	RPL32;RPS19;RPS20;RPSA
Ethosuximide-1200_mg/kg_in_Water-Rat-Brain-3d-dn	6/333	0,356430578	0,561224956	0	0	1,238351754	1,277503224	RPL32;RPS19;RPS6;RPS20;SEPP1;RPL39
N-Nitrosodiethylamine-1.67_mg/kg_in_Saline-Rat-Liver-5d-up	6/333	0,356430578	0,561112778	0	0	1,238351754	1,277503224	C1QA;RPS17;RPS6;RPS20;RPS3A;RPL39
Naproxen-10_mg/kg_in_Corn_Oil-Rat-Kidney-0.25d-up	6/333	0,356430578	0,561000646	0	0	1,238351754	1,277503224	FUNDC1;RPS20;RPS3A;RAPGEF5;SEPP1;TPT1
Oxaliplatin-8_mg/kg_in_Saline-Rat-Bone_marrow-3d-up	6/333	0,356430578	0,560888558	0	0	1,238351754	1,277503224	C1QA;NKG7;RPSA;RPL10A;SEPP1;SLC16A3
Artemisinin-2000_mg/kg_in_CMC-Rat-Liver-3d-dn	5/270	0,356971598	0,561627708	0	0	1,272750414	1,311059001	EEF1B2;C1QA;RPS3A;SEPP1;TPT1
Fluvastatin-5_mg/kg_in_Corn_Oil-Rat-Liver-3d-dn	5/270	0,356971598	0,56151554	0	0	1,272750414	1,311059001	C1QA;RPS17;CLP1;RPS3A;SEPP1
Enalapril-1500_mg/kg_in_CMC-Rat-Kidney-3d-dn	6/334	0,359017413	0,564620836	0	0	1,234644114	1,264750153	RPL32;RPS19;RPS20;RPL10A;SEPP1;TPT1
Venlafaxine-320_mg/kg_in_Water-Rat-Kidney-3d-up	6/334	0,359017413	0,564508115	0	0	1,234644114	1,264750153	EEF1B2;CXCL11;ARL2BP;CLP1;RPS8;SEPP1
Ampicillin-2000_mg/kg_in_CMC-Rat-Kidney-3d-dn	5/271	0,359852695	0,565708549	0	0	1,268053918	1,296027834	RPS19;ARL2BP;RPSA;SEPP1;TPT1
Cisapride-250_mg/kg_in_Corn_Oil-Rat-Heart-3d-dn	5/271	0,359852695	0,565595655	0	0	1,268053918	1,296027834	RPS17;RPL32;RPS19;RPS8;RPS6
Ibuprofen-54_mg/kg_in_Corn_Oil-Rat-Liver-5d-up	5/271	0,359852695	0,565482807	0	0	1,268053918	1,296027834	EEF1B2;RPS17;RPL32;RPS20;RPL39
Methocarbamol-1125_mg/kg_in_CMC-Rat-Liver-5d-dn	5/271	0,359852695	0,565370004	0	0	1,268053918	1,296027834	EEF1B2;RPL32;CLP1;RPS8;SEPP1
Penciclovir-1200_mg/kg_in_Corn_Oil-Rat-Kidney-1d-up	5/271	0,359852695	0,565257246	0	0	1,268053918	1,296027834	C1QA;RPS17;RPS19;RPS8;RPSA
Tamoxifen-64_mg/kg_in_Corn_Oil-Rat-Heart-3d-dn	5/271	0,359852695	0,565144532	0	0	1,268053918	1,296027834	EEF1B2;RPS17;RPL32;RPS19;RPS6
Zaleplon-100_mg/kg_in_Water-Rat-Liver-3d-up	5/271	0,359852695	0,565031864	0	0	1,268053918	1,296027834	C1QA;RPS17;CXCL11;RPS6;RPSA
Balsalazide-1100_mg/kg_in_Water-Rat-Liver-0.25d-up	6/335	0,361605504	0,567670909	0	0	1,230958609	1,252132856	C1QA;RPS19;RPS6;RPSA;RPL10A;SEPP1
Ethylene_Glycol-3525_mg/kg_in_Water-Rat-Kidney-3d-up	6/335	0,361605504	0,567557782	0	0	1,230958609	1,252132856	EEF1B2;RPS19;RPS6;RPS20;SEPP1;RPL39
Streptomycin-120_mg/kg_in_Saline-Rat-Bone_marrow-3d-up	6/335	0,361605504	0,5674447	0	0	1,230958609	1,252132856	EEF1B2;RPS19;FEM1B;RPSA;RPL10A;SEPP1
Griseofulvin-2500_mg/kg_in_Corn_Oil-Rat-Liver-1d-dn	5/272	0,362734564	0,569103073	0	0	1,263391955	1,281185493	EEF1B2;RPL32;CLP1;RPS6;RPL39
Phenacetin-619_mg/kg_in_Corn_Oil-Rat-Kidney-1d-up	5/272	0,362734564	0,568989728	0	0	1,263391955	1,281185493	EEF1B2;RPS17;CLP1;RPS8;RPS20
Sodium_Nitroprusside-6_mg/kg_in_Saline-Rat-Heart-5d-dn	5/272	0,362734564	0,568876429	0	0	1,263391955	1,281185493	CXCL11;RPS20;RAPGEF5;SEPP1;TPT1
Daunorubicin-3.25_mg/kg_in_Saline-Rat-Bone_marrow-3d-up	6/336	0,364194732	0,571052699	0	0	1,227295042	1,239649702	C1QA;RPS19;RPL10A;SEPP1;SLC16A3;TPT1
Fluphenazine-2.5_mg/kg_in_Corn_Oil-Rat-Liver-0.25d-up	6/336	0,364194732	0,570939034	0	0	1,227295042	1,239649702	EEF1B2;RPL32;RPS19;RPSA;RPS3A;SEPP1
Amlodipine-19_mg/kg_in_Corn_Oil-Rat-Liver-0.25d-dn	5/273	0,365617043	0,573054692	0	0	1,258764145	1,266529241	CLP1;RPS6;RPS20;RPL10A;RPL39
Atropine-94_mg/kg_in_Water-Rat-Liver-5d-dn	5/273	0,365617043	0,572940674	0	0	1,258764145	1,266529241	EEF1B2;C1QA;RPS8;RPS20;TPT1
Cerivastatin-0.05_mg/kg_in_Corn_Oil-Rat-Liver-0.25d-dn	5/273	0,365617043	0,572826701	0	0	1,258764145	1,266529241	RPS8;RPS20;RPS3A;RPL39;TPT1
Chloramphenicol-1500_mg/kg_in_CMC-Rat-Kidney-3d-dn	5/273	0,365617043	0,572712774	0	0	1,258764145	1,266529241	RPL32;CLP1;RPS8;SEPP1;TPT1
Chlorpyrifos-82_mg/kg_in_CMC-Rat-Kidney-3d-up	5/273	0,365617043	0,572598892	0	0	1,258764145	1,266529241	RPS17;RPL32;RPS3A;SEPP1;TPT1
Doxepin-132_uM_in_DMSO-Rat-Primary_rat_hepatocytes-1d-up	5/273	0,365617043	0,572485055	0	0	1,258764145	1,266529241	EEF1B2;RPS17;RPS20;RPS3A;PDRG1
Famotidine-1200_mg/kg_in_CMC-Rat-Heart-5d-dn	5/273	0,365617043	0,572371264	0	0	1,258764145	1,266529241	RPS19;ARL2BP;RPS8;RPS6;RPS3A
Genistein-20_mg/kg_in_Corn_Oil-Rat-Liver-1d-dn	5/273	0,365617043	0,572257517	0	0	1,258764145	1,266529241	RPS17;RPS3A;RPL39;SEPP1;TPT1
Lomustine-4.2_mg/kg_in_Corn_Oil-Rat-Liver-3d-dn	5/273	0,365617043	0,572143816	0	0	1,258764145	1,266529241	RPS17;RPL32;CLP1;RPS6;RPL10A
Meloxicam-33_mg/kg_in_Corn_Oil-Rat-Liver-3d-dn	5/273	0,365617043	0,57203016	0	0	1,258764145	1,266529241	RPS17;RPS6;RPSA;RPL39;TPT1
Methyldopa-325_mg/kg_in_Water-Rat-Liver-3d-up	5/273	0,365617043	0,57191655	0	0	1,258764145	1,266529241	EEF1B2;RPS8;RPS20;RAPGEF5;SEPP1
Moxonidine-17_mg/kg_in_CMC-Rat-Heart-3d-up	5/273	0,365617043	0,571802984	0	0	1,258764145	1,266529241	RPL32;RPS19;RPS6;RPS20;RPS3A
Procainamide-755_mg/kg_in_Water-Rat-Heart-3d-dn	5/273	0,365617043	0,571689463	0	0	1,258764145	1,266529241	RPS19;RPS3A;RPL39;GOLGA7;TPT1

Sulindac-23_mg/kg_in_Corn_Oil-Rat-Liver-0.25d-up	5/273	0,365617043	0,571575988	0	0	1,258764145	1,266529241	RPS6;RPS20;RPS3A;RPL39;TPT1
24-Diaminophenol-60_mg/kg_in_Water-Rat-Kidney-5d-up	6/337	0,366784978	0,57328805	0	0	1,223653217	1,227299085	CLP1;RPS8;RPS3A;RPL10A;GOLGA7;TPT1
Abamectin-10_mg/kg_in_Water-Rat-Liver-3d-dn	6/337	0,366784978	0,573174302	0	0	1,223653217	1,227299085	RPS6;RPL35A;RPS20;RPL10A;RPL39;TPT1
Fluoxetine-52_mg/kg_in_CMC-Rat-Kidney-3d-dn	6/337	0,366784978	0,5730606	0	0	1,223653217	1,227299085	CLP1;RPS8;RPS20;RPSA;SEPP1;RPL39
Celecoxib-400_mg/kg_in_Corn_Oil-Rat-Liver-1d-up	5/274	0,368499972	0,575625899	0	0	1,254170116	1,252056391	RPS17;RPS20;RPSA;RPS3A;SEPP1
Diphenhydramine-10.9_mg/kg_in_Water-Rat-Heart-5d-dn	5/274	0,368499972	0,575511755	0	0	1,254170116	1,252056391	EEF1B2;RPS19;FUNDC1;RPS20;TPT1
Gentamicin-40_mg/kg_in_Saline-Rat-Kidney-28d-dn	5/274	0,368499972	0,575397657	0	0	1,254170116	1,252056391	RPS17;RPL32;RPS6;RPS3A;TPT1
Hydrocortisone-56_mg/kg_in_Corn_Oil-Rat-Kidney-1d-dn	5/274	0,368499972	0,575283604	0	0	1,254170116	1,252056391	ARL2BP;RPS8;RPS6;RPS3A;RPL39
Netilmicin-41_mg/kg_in_Saline-Rat-Kidney-1d-dn	5/274	0,368499972	0,575169596	0	0	1,254170116	1,252056391	EEF1B2;ARL2BP;RPSA;RPL10A;RPL39
Sulfadimethoxine-1100_mg/kg_in_CMC-Rat-Kidney-5d-dn	5/274	0,368499972	0,575055633	0	0	1,254170116	1,252056391	RPS19;CLP1;RPS6;RPS3A;RPL10A
Fenofibrate-215_mg/kg_in_Corn_Oil-Rat-Kidney-3d-dn	6/338	0,369376124	0,576308707	0	0	1,220032941	1,215079421	RPL32;RPS19;ARL6IP5;RPL10A;SEPP1;RPL39
Ramipril-620_uM_in_DMSO-Rat-Primary_rat_hepatocytes-1d-dn	6/338	0,369376124	0,576194564	0	0	1,220032941	1,215079421	RPS19;RPSA;RPS3A;RPL39;SLC16A3;TPT1
Salicylic_Acid-223_mg/kg_in_Corn_Oil-Rat-Liver-5d-dn	6/338	0,369376124	0,576080466	0	0	1,220032941	1,215079421	RPS17;RPS8;RPSA;RPS3A;RPL39;TPT1
Alfacalcidol-0.043_mg/kg_in_CMC-Rat-Liver-1d-up	5/275	0,371383194	0,579096028	0	0	1,249609497	1,2377643	RPS8;RPSA;RPL39;GOLGA7;TPT1
Cyclosporin_A-70_mg/kg_in_Corn_Oil-Rat-Spleen-1d-dn	5/275	0,371383194	0,578981401	0	0	1,249609497	1,2377643	RPS19;RPS5;RPS6;RPL13A;RPL35A
Dideoxycytidine-1200_mg/kg_in_CMC-Rat-Liver-1d-up	5/275	0,371383194	0,578866819	0	0	1,249609497	1,2377643	EEF1B2;C1QA;CLP1;RPS8;TPT1
Etoposide-188_mg/kg_in_Corn_Oil-Rat-Heart-1d-dn	5/275	0,371383194	0,578752283	0	0	1,249609497	1,2377643	RPS17;RPL32;RPS6;RPS20;TPT1
Gabapentin-2500_mg/kg_in_Water-Rat-Heart-5d-dn	5/275	0,371383194	0,578637792	0	0	1,249609497	1,2377643	RPS19;ARL2BP;CLP1;RPS8;RPS20
Gemfibrozil-700_mg/kg_in_Corn_Oil-Rat-Liver-3d-up	5/275	0,371383194	0,578523346	0	0	1,249609497	1,2377643	RPS19;ARL2BP;RPS20;RPS3A;TPT1
Hydralazine-280_mg/kg_in_Water-Rat-Heart-5d-up	5/275	0,371383194	0,578408945	0	0	1,249609497	1,2377643	EEF1B2;ARL2BP;CLP1;RAPGEF5;SEPP1
Ifosfamide-143_mg/kg_in_Saline-Rat-Heart-5d-dn	5/275	0,371383194	0,57829459	0	0	1,249609497	1,2377643	RPS17;RPL32;RPL35A;RPSA;RPS3A
Loratadine-10_mg/kg_in_Water-Rat-Heart-5d-dn	5/275	0,371383194	0,57818028	0	0	1,249609497	1,2377643	C1QA;RPS8;ARL6IP5;RPL10A;SEPP1
Norfloxacin-1500_mg/kg_in_CMC-Rat-Kidney-5d-dn	5/275	0,371383194	0,578066015	0	0	1,249609497	1,2377643	EEF1B2;RPS17;RPS19;RPS20;RPS3A
Rosiglitazone-1800_mg/kg_in_Corn_Oil-Rat-Liver-0.25d-dn	5/275	0,371383194	0,577951796	0	0	1,249609497	1,2377643	EEF1B2;RPL32;RPL10A;RPL39;SEPP1
Valdecoxib-404_mg/kg_in_CMC-Rat-Heart-3d-up	5/275	0,371383194	0,577837621	0	0	1,249609497	1,2377643	EEF1B2;RPL32;RPS8;ARL6IP5;TPT1
Valdecoxib-404_mg/kg_in_CMC-Rat-Kidney-1d-dn	5/275	0,371383194	0,577723491	0	0	1,249609497	1,2377643	ARL2BP;RPL39;GOLGA7;SEPP1;TPT1
Chlorambucil-4.5_mg/kg_in_Corn_Oil-Rat-Spleen-5d-up	5/276	0,374266551	0,58209387	0	0	1,245081926	1,223650372	C1QA;CXCL11;NKG7;SEPP1;PDRG1
Chlorpromazine-18_mg/kg_in_Water-Rat-Liver-1d-up	5/276	0,374266551	0,581978945	0	0	1,245081926	1,223650372	EEF1B2;RPL32;RPS3A;RPL39;SEPP1
Cholecalciferol-8_mg/kg_in_CMC-Rat-Kidney-3d-dn	5/276	0,374266551	0,581864065	0	0	1,245081926	1,223650372	ARL2BP;RPS8;FUNDC1;RPS3A;SEPP1
Daunorubicin-1.5_uM_in_DMSO-Rat-Primary_rat_hepatocytes-1d-dn	5/276	0,374266551	0,581749231	0	0	1,245081926	1,223650372	RPS8;RPS20;RPSA;SEPP1;TOMM20
Ferrocene-330_mg/kg_in_CMC-Rat-Kidney-1d-up	5/276	0,374266551	0,581634443	0	0	1,245081926	1,223650372	RPS17;ARL2BP;RPS8;RPS20;RPS3A
Ritonavir-1200_mg/kg_in_CMC-Rat-Kidney-3d-dn	5/276	0,374266551	0,581519699	0	0	1,245081926	1,223650372	EEF1B2;RPL32;ARL2BP;CLP1;SEPP1
Tamoxifen-64_mg/kg_in_Corn_Oil-Rat-Liver-3d-dn	5/276	0,374266551	0,581405001	0	0	1,245081926	1,223650372	RPS17;RPS8;RPS3A;RPL10A;TPT1
Thioguanine-24_mg/kg_in_Corn_Oil-Rat-Liver-0.25d-dn	5/276	0,374266551	0,581290348	0	0	1,245081926	1,223650372	EEF1B2;RPS17;RPL32;RPSA;SEPP1
Cisplatin-1.17_mg/kg_in_Saline-Rat-Bone_marrow-1d-up	6/340	0,374560652	0,581632431	0	0	1,212856277	1,191026726	C1QA;RPS17;RPS19;RPS8;RPS20;TPT1
Enoxacin-750_mg/kg_in_Corn_Oil-Rat-Liver-0.25d-up	6/340	0,374560652	0,581517779	0	0	1,212856277	1,191026726	RPS19;RPS6;RPS20;RPL10A;SEPP1;GOLGA7
(R)-Bicalutamide-1400_mg/kg_in_CMC-Rat-Kidney-3d-dn	5/277	0,377149886	0,585422252	0	0	1,240587046	1,209712053	EEF1B2;ARL2BP;RPS8;RPS20;SEPP1
Dexamethasone-456_uM_in_DMSO-Rat-Primary_rat_hepatocytes-0.67d-up	5/277	0,377149886	0,585306897	0	0	1,240587046	1,209712053	RPL32;FEM1B;RPS6;RPS20;RPL10A
Digoxin-11_mg/kg_in_Corn_Oil-Rat-Heart-3d-dn	5/277	0,377149886	0,585191589	0	0	1,240587046	1,209712053	RPL32;RPS8;RPS6;RPL39;PDRG1
Isotretinoin-13_mg/kg_in_Corn_Oil-Rat-Liver-3d-dn	5/277	0,377149886	0,585076325	0	0	1,240587046	1,209712053	EEF1B2;RPS17;RPL32;RPL10A;SEPP1
Proglumide-1125_mg/kg_in_CMC-Rat-Liver-3d-up	5/277	0,377149886	0,584961108	0	0	1,240587046	1,209712053	C1QA;RPS17;RPS3A;RPL39;TPT1
Propylene_Glycol-2000_mg/kg_in_Water-Rat-Liver-5d-dn	5/277	0,377149886	0,584845935	0	0	1,240587046	1,209712053	EEF1B2;RPL32;RPS8;RPS20;TPT1
Sulfinpyrazone-269_mg/kg_in_CMC-Rat-Kidney-1d-up	5/277	0,377149886	0,584730808	0	0	1,240587046	1,209712053	RPS17;RPS19;FUNDC1;RPS6;RPL39
Tretinoin-7_mg/kg_in_Corn_Oil-Rat-Kidney-1d-dn	5/277	0,377149886	0,584615726	0	0	1,240587046	1,209712053	RPS6;RPS20;RPS3A;RPL39;GOLGA7
Aminocaproic_Acid-2000_mg/kg_in_Water-Rat-Kidney-3d-up	6/341	0,3771538	0,584506755	0	0	1,209299513	1,179190637	EEF1B2;RPS19;ARL2BP;RPS6;RPS20;RPS3A
Isotretinoin-500_uM_in_DMSO-Rat-Primary_rat_hepatocytes-1d-dn	6/341	0,3771538	0,584391762	0	0	1,209299513	1,179190637	CXCL11;RPS19;RPS8;SEPP1;RPL39;TPT1
Acyclovir-330_mg/kg_in_Saline-Rat-Bone_marrow-1d-dn	4/214	0,378524184	0,586399778	0	0	1,284645277	1,248001176	RPS19;RPS3A;RPL10A;RPL39
Phenelzine-27_mg/kg_in_Water-Rat-Heart-3d-up	6/342	0,379747384	0,588179036	0	0	1,20576355	1,167479383	ARL2BP;RPS8;FUNDC1;ARL6IP5;GOLGA7;TPT1
3-Chloroaniline-200_mg/kg_in_CMC-Rat-Kidney-3d-up	5/278	0,380033045	0,588505753	0	0	1,236124502	1,195946834	ARL6IP5;RPS3A;RPL10A;SEPP1;TPT1
Acrolein-20_mg/kg_in_Water-Rat-Kidney-5d-dn	5/278	0,380033045	0,588390065	0	0	1,236124502	1,195946834	RPS17;RPL32;RPL35A;RPSA;RPL39
Atorvastatin-2.5_mg/kg_in_Corn_Oil-Rat-Kidney-0.25d-dn	5/278	0,380033045	0,588274422	0	0	1,236124502	1,195946834	CLP1;RPS8;RPS20;SEPP1;TPT1
Bis(2-Ethylhexyl)Phthalate-100_mg/kg_in_Corn_Oil-Rat-Liver-1d-dn	5/278	0,380033045	0,588158825	0	0	1,236124502	1,195946834	EEF1B2;ARL2BP;CLP1;RPS8;RPS3A
Candesartan-1300_mg/kg_in_CMC-Rat-Kidney-5d-dn	5/278	0,380033045	0,588043273	0	0	1,236124502	1,195946834	EEF1B2;RPS17;CLP1;RPS8;RPS3A
Chloroxylenol-1915_mg/kg_in_Corn_Oil-Rat-Liver-3d-up	5/278	0,380033045	0,587927767	0	0	1,236124502	1,195946834	C1QA;RPS17;RPS8;RPSA;SEPP1
Fenofibrate-215_mg/kg_in_Corn_Oil-Rat-Skeletal_muscle-5d-up	5/278	0,380033045	0,587812306	0	0	1,236124502	1,195946834	RPS19;RPS8;RPS6;RPS20;TPT1
Nimesulide-162_mg/kg_in_Corn_Oil-Rat-Kidney-3d-up	5/278	0,380033045	0,58769689	0	0	1,236124502	1,195946834	C1QA;CXCL11;RPS8;RPS20;RPS3A
Ticrynafen-570_mg/kg_in_CMC-Rat-Liver-3d-dn	5/278	0,380033045	0,58758152	0	0	1,236124502	1,195946834	C1QA;RPS17;CLP1;RPS8;RPL35A
Valproic_Acid-235_mg/kg_in_Water-Rat-Bone_marrow-1d-dn	5/278	0,380033045	0,587466195	0	0	1,236124502	1,195946834	RPL32;RPS6;ARL6IP5;RPL39;DUSP14
Losartan-1000_mg/kg_in_Water-Rat-Kidney-1d-up	7/407	0,380889665	0,588674844	0	0	1,182063038	1,140981073	EEF1B2;RPS19;RPS8;RPS3A;SEPP1;TOMM20;TPT1
Cycloheximide-0.25_mg/kg_in_Water-Rat-Liver-3d-dn	4/215	0,381818311	0,589994313	0	0	1,278670183	1,231116963	CLP1;GOLGA7;SEPP1;TPT1
Stavudine-58_mg/kg_in_Water-Rat-Liver-0.25d-dn	4/215	0,381818311	0,589878583	0	0	1,278670183	1,231116963	RPS8;RPL39;SEPP1;TPT1
Doxofylline-13500_uM_in_DMSO-Rat-Primary_rat_hepatocytes-1d-dn	6/343	0,382341289	0,590570699	0	0	1,202248204	1,155891488	CXCL11;RPS19;ARL6IP5;RPS20;RPL39;SLC16A3
Ifosfamide-17_mg/kg_in_Saline-Rat-Bone_marrow-3d-up	6/343	0,382341289	0,590454901	0	0	1,202248204	1,155891488	C1QA;CLP1;RPS8;NKG7;RPL10A;SEPP1
Trans-Platinum(II)Diammine_Dichloride-0.5_mg/kg_in_Saline-Rat-Kidney-28d-dn	6/343	0,382341289	0,590339148	0	0	1,202248204	1,155891488	RPS6;RPSA;RPL10A;SEPP1;RPL39;TPT1
Artemisinin-2000_mg/kg_in_CMC-Rat-Liver-1d-dn	5/279	0,382915873	0,59111043	0	0	1,231693949	1,182352249	EEF1B2;C1QA;CLP1;RPS20;SEPP1
Bezafibrate-100_mg/kg_in_Corn_Oil-Rat-Kidney-1d-up	5/279	0,382915873	0,590994594	0	0	1,231693949	1,182352249	RPS19;RPS5;RPS6;RPS20;RAPGEF5
Bromfenac-5_mg/kg_in_CMC-Rat-Kidney-1d-up	5/279	0,382915873	0,590878803	0	0	1,231693949	1,182352249	RPS17;RPL32;RPS3A;SEPP1;TPT1
Chlorambucil-1540_uM_in_DMSO-Rat-Primary_rat_hepatocytes-0.67d-up	5/279	0,382915873	0,590763058	0	0	1,231693949	1,182352249	EEF1B2;RPL32;RPS5;RPS6;PDRG1
Cisplatin-1.17_mg/kg_in_Saline-Rat-Spleen-0.25d-up	5/279	0,382915873	0,590647359	0	0	1,231693949	1,182352249	C1QA;RPS17;RPS5;RPS3A;RPL10A
Diflunisal-207_mg/kg_in_Corn_Oil-Rat-Kidney-1d-dn	5/279	0,382915873	0,590531704	0	0	1,231693949	1,182352249	EEF1B2;RPS17;ARL2BP;RPL35A;RPL39
Doxorubicin-4_mg/kg_in_Saline-Rat-Heart-14d-up	5/279	0,382915873	0,590416095	0	0	1,231693949	1,182352249	RPS8;RPS3A;RAPGEF5;GOLGA7;TPT1
Erythromycin-1500_mg/kg_in_CMC-Rat-Kidney-3d-up	5/279	0,382915873	0,590300531	0	0	1,231693949	1,182352249	EEF1B2;RPS17;RPS3A;SEPP1;TPT1
Ethylene_Glycol-352_mg/kg_in_Water-Rat-Liver-0.25d-dn	5/279	0,382915873	0,590185012	0	0	1,231693949	1,182352249	RPS8;RPS6;RPSA;SEPP1;TPT1
Famciclovir-1200_mg/kg_in_Saline-Rat-Bone_marrow-1d-dn	5/279	0,382915873	0,590069539	0	0	1,231693949	1,182352249	NKG7;RPS20;RPL10A;RPL39;SEPP1
Oxcarbazepine-1600_mg/kg_in_CMC-Rat-Brain-1d-up	5/279	0,382915873	0,589954111	0	0	1,231693949	1,182352249	RPS17;RPS8;ARL6IP5;RPS3A;RPL10A
Sodium_Nitroprusside-6_mg/kg_in_Saline-Rat-Heart-1d-dn	5/279	0,382915873	0,589838727	0	0	1,231693949	1,182352249	CXCL11;FEM1B;RPS20;RPS3A;TPT1
Sporidesmin_A-0.15_uM_in_DMSO-Rat-Primary_rat_hepatocytes-0.67d-up	5/279	0,382915873	0,589723389	0	0	1,231693949	1,182352249	RPS19;CLP1;ARL6IP5;RPS20;TPT1
Carboplatin-14_mg/kg_in_Saline-Rat-Kidney-1d-up	6/344	0,384935401	0,592717736	0	0	1,198753297	1,144425495	RPS17;RPS8;RPS6;RPS20;RPSA;RPS3A
Neomycin-56_mg/kg_in_Corn_Oil-Rat-Kidney-0.25d-up	6/344	0,384935401	0,59260188	0	0	1,198753297	1,144425495	C1QA;RPS17;RPS20;RPS3A;RPL39;GOLGA7
Angiotensin_II_Human-0.72_mg/kg_in_Saline-Rat-Heart-5d-dn	5/280	0,385798217	0,593814101	0	0	1,227295042	1,168925872	RPS17;RPL32;RPS6;RPS3A;TPT1
Chlorambucil-0.6_mg/kg_in_Corn_Oil-Rat-Bone_marrow-0.25d-dn	5/280	0,385798217	0,593698076	0	0	1,227295042	1,168925872	RPS17;RPS8;FEM1B;RPSA;TPT1
Clonazepam-2500_mg/kg_in_CMC-Rat-Heart-5d-up	5/280	0,385798217	0,593582097	0	0	1,227295042	1,168925872	RPL32;CLP1;RPS8;RPS3A;TPT1
Clonazepam-2500_mg/kg_in_CMC-Rat-Liver-1d-dn	5/280	0,385798217	0,593466163	0	0	1,227295042	1,168925872	EEF1B2;C1QA;RPS17;CLP1;RPS20
Cobalt(II)_Acetylacetonate-100_mg/kg_in_Corn_Oil-Rat-Kidney-5d-dn	5/280	0,385798217	0,593350274	0	0	1,227295042	1,168925872	ARL2BP;CLP1;GOLGA7;SEPP1;TPT1
Dobutamine-43_mg/kg_in_Saline-Rat-Heart-1d-dn	5/280	0,385798217	0,593234431	0	0	1,227295042	1,168925872	C1QA;ARL2BP;RPS8;FUNDC1;SEPP1
Genistein-375_mg/kg_in_Corn_Oil-Rat-Liver-3d-dn	5/280	0,385798217	0,593118633	0	0	1,227295042	1,168925872	C1QA;RPL32;RPS19;RPSA;SEPP1
Lead_(II)_Acetate-600_mg/kg_in_Saline-Rat-Spleen-1d-dn	5/280	0,385798217	0,59300288	0	0	1,227295042	1,168925872	EEF1B2;RPS8;FUNDC1;RPL13A;RPL35A
Maprotiline-380_mg/kg_in_CMC-Rat-Heart-3d-up	5/280	0,385798217	0,592887172	0	0	1,227295042	1,168925872	RPS17;RPL32;RPS3A;SEPP1;TPT1
Ticlopidine-223_mg/kg_in_CMC-Rat-Liver-5d-up	5/280	0,385798217	0,592771509	0	0	1,227295042	1,168925872	RPS19;RPS6;RPSA;RPL10A;SEPP1
Amantadine-58_mg/kg_in_Water-Rat-Liver-0.25d-up	6/345	0,387529607	0,59531562	0	0	1,195278649	1,133079968	C1QA;RPS19;RPS6;RPSA;RPL10A;SEPP1

Doxycycline-1000_mg/kg_in_Water-Rat-Liver-1d-up	5/281	0,388679925	0,596966282	0	0	1,222927444	1,155665319	C1QA;RPS17;RPS6;RPS20;RPL39
Dyphylline-3855_mg/kg_in_CMC-Rat-Heart-1d-dn	5/281	0,388679925	0,596849891	0	0	1,222927444	1,155665319	RPS8;RPSA;GOLGA7;SEPP1;TPT1
Fenofibrate-215_mg/kg_in_Corn_Oil-Rat-Heart-3d-dn	5/281	0,388679925	0,596733546	0	0	1,222927444	1,155665319	RPS17;RPL32;COL5A3;RPL35A;RPS3A
Ferrocene-330_mg/kg_in_CMC-Rat-Kidney-3d-up	5/281	0,388679925	0,596617247	0	0	1,222927444	1,155665319	EEF1B2;RPS17;RPS8;ARL6IP5;RPS3A
Fludrocortisone_Acetate-125_mg/kg_in_CMC-Rat-Kidney-1d-up	5/281	0,388679925	0,596500992	0	0	1,222927444	1,155665319	EEF1B2;CXCL11;RPS6;RPL10A;RPL39
Pemoline-70_mg/kg_in_CMC-Rat-Liver-5d-dn	5/281	0,388679925	0,596384783	0	0	1,222927444	1,155665319	EEF1B2;RPS8;RPSA;RPS3A;TPT1
Pralidoxime_Chloride-48_mg/kg_in_Saline-Rat-Liver-5d-up	5/281	0,388679925	0,596268619	0	0	1,222927444	1,155665319	C1QA;RPS8;RPL39;GOLGA7;TPT1
Probenecid-600_mg/kg_in_Corn_Oil-Rat-Kidney-5d-dn	5/281	0,388679925	0,596152501	0	0	1,222927444	1,155665319	EEF1B2;RPS19;CLP1;RPS8;TPT1
Trihexyphenidyl-588_mg/kg_in_CMC-Rat-Heart-1d-up	5/281	0,388679925	0,596036428	0	0	1,222927444	1,155665319	CXCL11;RPS19;FUNDC1;RPS6;RAPGEF5
Amiloride-22_mg/kg_in_CMC-Rat-Heart-3d-dn	6/346	0,390123793	0,598134124	0	0	1,191824087	1,121853492	EEF1B2;RPS20;RPSA;RPL10A;SEPP1;RPL39
Bromisovalum-250_mg/kg_in_Corn_Oil-Rat-Heart-3d-up	6/346	0,390123793	0,59801771	0	0	1,191824087	1,121853492	COL5A3;ARL6IP5;RPSA;RPL10A;TPT1;PDRG1
Labetalol-60_mg/kg_in_Water-Rat-Heart-3d-up	6/346	0,390123793	0,597901341	0	0	1,191824087	1,121853492	RPS19;RPS6;RPS20;RPSA;RPL10A;GOLGA7
Amikacin-20_mg/kg_in_Saline-Rat-Kidney-3d-up	5/282	0,391560848	0,599987011	0	0	1,218590822	1,142568245	ARL2BP;RPS8;RPS6;GOLGA7;TPT1
Anastrozole-400_mg/kg_in_CMC-Rat-Liver-3d-dn	5/282	0,391560848	0,599870305	0	0	1,218590822	1,142568245	EEF1B2;C1QA;CLP1;ARL6IP5;SEPP1
Benzothiazyl_Disulfide-2600_mg/kg_in_Corn_Oil-Rat-Liver-3d-up	5/282	0,391560848	0,599753644	0	0	1,218590822	1,142568245	RPS8;RPS6;RPS3A;RAPGEF5;RPL10A
DActinomycin-0.06_mg/kg_in_Saline-Rat-Bone_marrow-1d-dn	5/282	0,391560848	0,599637028	0	0	1,218590822	1,142568245	EEF1B2;RPS8;FUNDC1;NKG7;RPS20
Deferiprone-500_mg/kg_in_Water-Rat-Liver-5d-dn	5/282	0,391560848	0,599520458	0	0	1,218590822	1,142568245	C1QA;RPS20;RPL39;SEPP1;TPT1
Ethanol-6000_mg/kg_in_Saline-Rat-Kidney-3d-dn	5/282	0,391560848	0,599403933	0	0	1,218590822	1,142568245	EEF1B2;RPS6;RPL10A;RPL39;TPT1
Flubendazole-1200_mg/kg_in_Corn_Oil-Rat-Liver-1d-dn	5/282	0,391560848	0,599287454	0	0	1,218590822	1,142568245	RPS8;RPS6;RPS20;RPL10A;SEPP1
Kanamycin-225_mg/kg_in_Saline-Rat-Kidney-3d-dn	5/282	0,391560848	0,59917102	0	0	1,218590822	1,142568245	EEF1B2;CLP1;RPS6;RPL10A;SEPP1
Perhexiline-12_uM_in_DMSO-Rat-Primary_rat_hepatocytes-1d-up	5/282	0,391560848	0,59905463	0	0	1,218590822	1,142568245	EEF1B2;RPS20;RPS3A;RPL10A;RPL39
SCH-351591-4.6_mg/kg_in_CMC-Rat-Heart-1d-dn	5/282	0,391560848	0,598938287	0	0	1,218590822	1,142568245	RPS19;RPS6;RPS20;RAPGEF5;TPT1
Valdecoxib-404_mg/kg_in_CMC-Rat-Kidney-5d-dn	5/282	0,391560848	0,598821988	0	0	1,218590822	1,142568245	RPS17;RPS19;RPS3A;RPL10A;SEPP1
Valproic_Acid-235_mg/kg_in_Water-Rat-Liver-1d-up	5/282	0,391560848	0,598705734	0	0	1,218590822	1,142568245	RPS19;RPS6;RPS3A;RPL39;TPT1
33'5-Triiodo-L-Thyronine-0.001_mg/kg_in_PEG400-Rat-Heart-3d-dn	5/283	0,394440835	0,602992239	0	0	1,214284847	1,129632345	RPS17;FUNDC1;RPS20;RPSA;RPL39
Chlorpromazine-73_mg/kg_in_Water-Rat-Liver-0.25d-dn	5/283	0,394440835	0,602875221	0	0	1,214284847	1,129632345	C1QA;RPS8;RPSA;RPL39;SEPP1
Cortisone-206_mg/kg_in_CMC-Rat-Heart-5d-dn	5/283	0,394440835	0,602758249	0	0	1,214284847	1,129632345	RPS17;COL5A3;RPS6;SEPP1;TPT1
Ethylene_Glycol-3525_mg/kg_in_Water-Rat-Bone_marrow-5d-dn	5/283	0,394440835	0,602641322	0	0	1,214284847	1,129632345	RPS19;RPS8;FEM1B;RPS6;ARL6IP5
Gold_Sodium_Thiomalate-8_mg/kg_in_Saline-Rat-Kidney-5d-dn	5/283	0,394440835	0,60252444	0	0	1,214284847	1,129632345	EEF1B2;RPS17;RPS19;ARL2BP;SEPP1
Granisetron-175_mg/kg_in_Water-Rat-Liver-0.25d-dn	5/283	0,394440835	0,602407604	0	0	1,214284847	1,129632345	EEF1B2;RPL35A;RPL39;SEPP1;TPT1
Insulin-0.56_mg/kg_in_Saline-Rat-Liver-1d-dn	5/283	0,394440835	0,602290813	0	0	1,214284847	1,129632345	RPL32;RPS19;RPL10A;RPL39;TPT1
Lidocaine-71_mg/kg_in_Water-Rat-Heart-1d-dn	5/283	0,394440835	0,602174068	0	0	1,214284847	1,129632345	EEF1B2;CLP1;FUNDC1;SEPP1;TOMM20
N-Nitrosodiethylamine-100_mg/kg_in_Saline-Rat-Liver-5d-up	5/283	0,394440835	0,602057367	0	0	1,214284847	1,129632345	C1QA;RPS19;GOLGA7;SEPP1;TPT1
Netilmicin-41_mg/kg_in_Saline-Rat-Kidney-3d-dn	5/283	0,394440835	0,601940712	0	0	1,214284847	1,129632345	EEF1B2;RPS8;RPS6;RPS3A;SEPP1
Nitrofurantoin-76_mg/kg_in_CMC-Rat-Liver-3d-dn	5/283	0,394440835	0,601824102	0	0	1,214284847	1,129632345	C1QA;CLP1;FEM1B;RPL35A;TPT1
Ondansetron-84_mg/kg_in_Water-Rat-Intestine-3d-up	5/283	0,394440835	0,601707537	0	0	1,214284847	1,129632345	RPS19;ARL6IP5;GOLGA7;SEPP1;SLC16A3
Paroxetine-30_uM_in_DMSO-Rat-Primary_rat_hepatocytes-1d-up	5/283	0,394440835	0,601591018	0	0	1,214284847	1,129632345	RPL32;RPS5;RPSA;RPS3A;RPL10A
Pioglitazone-1500_mg/kg_in_Corn_Oil-Rat-Liver-1d-dn	5/283	0,394440835	0,601474543	0	0	1,214284847	1,129632345	CLP1;RPS8;ARL6IP5;RPSA;SEPP1
Sertraline-210_mg/kg_in_Corn_Oil-Rat-Heart-5d-up	5/283	0,394440835	0,601358114	0	0	1,214284847	1,129632345	RPS8;FUNDC1;RAPGEF5;SEPP1;TPT1
Clotrimazole-60_uM_in_DMSO-Rat-Primary_rat_hepatocytes-0.67d-up	4/219	0,394974206	0,60205474	0	0	1,255315476	1,166106254	FEM1B;RPL10A;RPL39;PDRG1
Benzethonium_Chloride-30_mg/kg_in_Corn_Oil-Rat-Heart-1d-dn	6/348	0,395311659	0,60245252	0	0	1,184974523	1,099752122	EEF1B2;RPL32;RPS8;ARL6IP5;SEPP1;RPL39
Diethylstilbestrol-2.8_mg/kg_in_Corn_Oil-Rat-Kidney-3d-dn	6/348	0,395311659	0,602335969	0	0	1,184974523	1,099752122	EEF1B2;RPS17;RPL32;RPS8;RPSA;RPL39
Nimesulide-162_mg/kg_in_Corn_Oil-Rat-Liver-0.25d-dn	6/348	0,395311659	0,602219463	0	0	1,184974523	1,099752122	RPS19;RPS20;RPSA;SEPP1;RPL39;TPT1
4-Chloro-2-Nitroaniline-1200_mg/kg_in_CMC-Rat-Kidney-3d-dn	5/284	0,397319738	0,605161527	0	0	1,210009196	1,116855352	FEM1B;RPS6;RPSA;RPL39;SEPP1
Bithionol-333_mg/kg_in_Corn_Oil-Rat-Bone_marrow-1d-dn	5/284	0,397319738	0,60504452	0	0	1,210009196	1,116855352	RPS8;FEM1B;RPS6;ARL6IP5;RPS3A
Bithionol-333_mg/kg_in_Corn_Oil-Rat-Kidney-1d-up	5/284	0,397319738	0,604927558	0	0	1,210009196	1,116855352	RPS19;RPS5;RPS6;RPL35A;RPS20
Bromisovalum-250_mg/kg_in_Corn_Oil-Rat-Liver-1d-up	5/284	0,397319738	0,604810641	0	0	1,210009196	1,116855352	RPS19;RPS8;RPS3A;RPL10A;SEPP1
Busulfan-9_mg/kg_in_Corn_Oil-Rat-Bone_marrow-5d-dn	5/284	0,397319738	0,60469377	0	0	1,210009196	1,116855352	RPS8;RPS6;NKG7;RPSA;RPL10A
Carboplatin-6_mg/kg_in_Saline-Rat-Liver-3d-up	5/284	0,397319738	0,604576943	0	0	1,210009196	1,116855352	RPS19;RPS6;RPS20;RPL10A;GOLGA7
Carmofur-201_mg/kg_in_CMC-Rat-Bone_marrow-1d-up	5/284	0,397319738	0,604460162	0	0	1,210009196	1,116855352	RPS19;RPS6;ARL6IP5;NKG7;RPL10A
Diethylstilbestrol-280_mg/kg_in_Corn_Oil-Rat-Bone_marrow-3d-dn	5/284	0,397319738	0,604343426	0	0	1,210009196	1,116855352	RPS19;RPS6;ARL6IP5;RPSA;RPL10A
Dipyridamole-750_mg/kg_in_CMC-Rat-Liver-5d-dn	5/284	0,397319738	0,604226734	0	0	1,210009196	1,116855352	C1QA;RPS3A;RPL10A;RPL39;TPT1
Ethylene_Glycol-3525_mg/kg_in_Water-Rat-Liver-5d-up	5/284	0,397319738	0,604110088	0	0	1,210009196	1,116855352	EEF1B2;RPS17;RPS8;RPL35A;RPS20
Fluocinolone_Acetonide-2.5_mg/kg_in_Corn_Oil-Rat-Kidney-1d-dn	5/284	0,397319738	0,603993487	0	0	1,210009196	1,116855352	RPS19;RPS6;RPSA;RPL10A;RPL39
Indomethacin-9.6_mg/kg_in_Water-Rat-Kidney-1d-dn	5/284	0,397319738	0,603876931	0	0	1,210009196	1,116855352	EEF1B2;ARL2BP;FUNDC1;ARL6IP5;RPSA
Paroxetine-30_uM_in_DMSO-Rat-Primary_rat_hepatocytes-0.67d-dn	5/284	0,397319738	0,60376042	0	0	1,210009196	1,116855352	CXCL11;RPS19;ARL6IP5;RPSA;SEPP1
Rosiglitazone-1800_mg/kg_in_Corn_Oil-Rat-Heart-3d-dn	5/284	0,397319738	0,603643954	0	0	1,210009196	1,116855352	RPS17;RPL32;RPS19;COL5A3;RPS3A
Thioguanine-24_mg/kg_in_Corn_Oil-Rat-Bone_marrow-5d-dn	5/284	0,397319738	0,603527533	0	0	1,210009196	1,116855352	RPS19;RPS8;RPS6;RPSA;TPT1
Vecuronium_Bromide-0.005_mg/kg_in_Saline-Rat-Heart-3d-up	5/284	0,397319738	0,603411156	0	0	1,210009196	1,116855352	RPS19;RPS8;RPS20;RPL39;TPT1
Acetaminophen-100_mg/kg_in_Corn_Oil-Rat-Liver-0.25d-dn	5/285	0,400197411	0,607664316	0	0	1,20576355	1,104235036	C1QA;RPS17;RPS8;RPL39;TPT1
Azauridine-1500_mg/kg_in_CMC-Rat-Kidney-5d-dn	5/285	0,400197411	0,607547187	0	0	1,20576355	1,104235036	RPS20;RPL10A;RPL39;GOLGA7;SEPP1
Clofibrate-130_mg/kg_in_Corn_Oil-Rat-Liver-1d-dn	5/285	0,400197411	0,607430103	0	0	1,20576355	1,104235036	C1QA;RPSA;RPS3A;RPL39;SEPP1
Fluoxetine-52_mg/kg_in_CMC-Rat-Kidney-1d-up	5/285	0,400197411	0,607313065	0	0	1,20576355	1,104235036	RPS19;RPS6;RPL10A;SEPP1;TPT1
Itraconazole-1093_mg/kg_in_Corn_Oil-Rat-Liver-0.25d-dn	5/285	0,400197411	0,607196071	0	0	1,20576355	1,104235036	C1QA;RPL32;ARL2BP;RPS8;RPS20
N-Nitrosodiethylamine-34_mg/kg_in_Saline-Rat-Liver-5d-dn	5/285	0,400197411	0,607079123	0	0	1,20576355	1,104235036	CLP1;RPS8;RPS20;RPSA;SEPP1
Rifabutin-1500_mg/kg_in_CMC-Rat-Kidney-3d-dn	5/285	0,400197411	0,60696222	0	0	1,20576355	1,104235036	EEF1B2;CLP1;RPL35A;RPL39;SEPP1
Rofecoxib-3_mg/kg_in_Corn_Oil-Rat-Liver-5d-up	5/285	0,400197411	0,606845361	0	0	1,20576355	1,104235036	EEF1B2;RPS19;RPS20;RPSA;RPL39
Sulindac-64_mg/kg_in_Corn_Oil-Rat-Liver-0.25d-dn	5/285	0,400197411	0,606728548	0	0	1,20576355	1,104235036	C1QA;RPS17;RPS8;RPS5;RPSA
Sulpiride-1000_mg/kg_in_CMC-Rat-Heart-1d-dn	5/285	0,400197411	0,60661178	0	0	1,20576355	1,104235036	RPS19;CLP1;RPS8;COL5A3;SEPP1
Olanzapine-250_uM_in_DMSO-Rat-Primary_rat_hepatocytes-1d-dn	6/350	0,400498109	0,606950762	0	0	1,17820324	1,078110437	CXCL11;RPS19;ARL6IP5;SEPP1;RPL39;TPT1
NN-Dimethylformamide-140_mg/kg_in_Saline-Rat-Kidney-3d-dn	4/221	0,401536807	0,608407828	0	0	1,243955155	1,135054441	EEF1B2;RPL32;RPS8;RPL10A
3-Acetamidophenol-700_mg/kg_in_Corn_Oil-Rat-Liver-5d-up	5/286	0,403073707	0,610619064	0	0	1,201547593	1,091769205	RPL32;RPS19;RPS6;RPSA;RPL39
3-Methylcholanthrene-12.5_mg/kg_in_CMC-Rat-Liver-5d-up	5/286	0,403073707	0,610501637	0	0	1,201547593	1,091769205	EEF1B2;CLP1;RPSA;RPL10A;SEPP1
3-Methylcholanthrene-300_mg/kg_in_CMC-Rat-Liver-3d-dn	5/286	0,403073707	0,610384256	0	0	1,201547593	1,091769205	RPS19;CLP1;RPS8;RPS6;RPL39
Amlodipine-0.2_mg/kg_in_Corn_Oil-Rat-Liver-5d-dn	5/286	0,403073707	0,610266919	0	0	1,201547593	1,091769205	RPS19;RPS6;RPSA;RPL10A;SEPP1
Atorvastatin-300_mg/kg_in_Corn_Oil-Rat-Kidney-3d-up	5/286	0,403073707	0,610149628	0	0	1,201547593	1,091769205	RPS5;RPL35A;RPSA;RPS3A;SEPP1
Azithromycin-225_mg/kg_in_Water-Rat-Liver-1d-dn	5/286	0,403073707	0,610032382	0	0	1,201547593	1,091769205	RPS19;CLP1;RPS20;RPSA;SEPP1
Camptothecin-2.5_mg/kg_in_Saline-Rat-Heart-3d-up	5/286	0,403073707	0,60991518	0	0	1,201547593	1,091769205	RPS8;HOXA1;RPS3A;SEPP1;TPT1
Clotrimazole-178_mg/kg_in_Corn_Oil-Rat-Liver-3d-up	5/286	0,403073707	0,609798024	0	0	1,201547593	1,091769205	RPS19;RPS8;RPS6;RPS20;RPSA
Etoposide-100_mg/kg_in_Corn_Oil-Rat-Liver-5d-up	5/286	0,403073707	0,609680913	0	0	1,201547593	1,091769205	RPS17;RPS19;RPS6;RPS20;RPL39
Paroxetine-104_mg/kg_in_Water-Rat-Heart-5d-up	5/286	0,403073707	0,609563847	0	0	1,201547593	1,091769205	RPS8;ARL6IP5;RPL10A;RPL39;TPT1
Terbinafine-2000_mg/kg_in_CMC-Rat-Liver-1d-up	5/286	0,403073707	0,609446826	0	0	1,201547593	1,091769205	RPS17;RPS6;RPS3A;RPL39;TPT1
Vinblastine-0.3_mg/kg_in_Saline-Rat-Liver-1d-dn	5/286	0,403073707	0,609329849	0	0	1,201547593	1,091769205	RPS17;RPS8;FEM1B;RPS20;RPS3A
Meloxicam-6_mg/kg_in_Corn_Oil-Rat-Liver-3d-up	6/351	0,403090529	0,609238343	0	0	1,174846536	1,067458637	C1QA;RPS19;RPS20;RPSA;SEPP1;RPL39
Streptozotocin-3000_uM_in_DMSO-Rat-Primary_rat_hepatocytes-0.67d-up	6/351	0,403090529	0,609121451	0	0	1,174846536	1,067458637	RPS17;RPL32;RPS3A;SLC16A3;TPT1;PDRG1
Ramipril-1500_mg/kg_in_CMC-Rat-Kidney-5d-dn	4/222	0,404813543	0,6116078	0	0	1,238351754	1,119877039	RPS19;CLP1;RPL10A;TPT1
Ethanol-6000_mg/kg_in_Saline-Rat-Bone_marrow-1d-up	6/352	0,405682265	0,612802747	0	0	1,171508903	1,056917787	RPS19;RPS6;RPS20;SEPP1;RPL39;GOLGA7
Rifapentine-75_mg/kg_in_Corn_Oil-Rat-Kidney-3d-up	6/352	0,405682265	0,612685239	0	0	1,171508903	1,056917787	C1QA;RPL32;R3HCC1;RPSA;RPL39;TPT1
123-Trichloropropane-108_mg/kg_in_CMC-Rat-Heart-3d-up	5/287	0,405948482	0,612969755	0	0	1,197361016	1,079455704	EEF1B2;C1QA;RPS17;RPS8;TPT1
Acyclovir-980_mg/kg_in_Saline-Rat-Spleen-5d-dn	5/287	0,405948482	0,61285226	0	0	1,197361016	1,079455704	EEF1B2;RPS19;RPS8;RPL10A;SEPP1

Albendazole-62_mg/kg_in_Corn_Oil-Rat-Liver-5d-dn	5/287	0,405948482	0,612734811	0	0	1,197361016	1,079455704	EEF1B2;RPS19;CLP1;RPS6;RPL10A
Epirubicin-2.7_mg/kg_in_Saline-Rat-Liver-3d-up	5/287	0,405948482	0,612617406	0	0	1,197361016	1,079455704	RPS8;RPS3A;RAPGEF5;SEPP1;TPT1
Gallamine_Triethiodide-2.5_mg/kg_in_Saline-Rat-Heart-3d-dn	5/287	0,405948482	0,612500046	0	0	1,197361016	1,079455704	RPS6;RPSA;RPL10A;RPL39;SEPP1
Genistein-375_mg/kg_in_Corn_Oil-Rat-Liver-5d-dn	5/287	0,405948482	0,612382732	0	0	1,197361016	1,079455704	C1QA;RPS17;RPS3A;RPL39;SEPP1
Kanamycin-225_mg/kg_in_Saline-Rat-Kidney-5d-dn	5/287	0,405948482	0,612265462	0	0	1,197361016	1,079455704	EEF1B2;RPS8;RPS20;RPS3A;SEPP1
Methocarbamol-1125_mg/kg_in_CMC-Rat-Liver-3d-dn	5/287	0,405948482	0,612148237	0	0	1,197361016	1,079455704	EEF1B2;C1QA;RPS8;ARL6IP5;SEPP1
Methyldopa-325_mg/kg_in_Water-Rat-Liver-5d-up	5/287	0,405948482	0,612031057	0	0	1,197361016	1,079455704	C1QA;CLP1;RPS8;SEPP1;TPT1
NN-Dimethylformamide-2100_mg/kg_in_Saline-Rat-Liver-0.25d-up	5/287	0,405948482	0,611913922	0	0	1,197361016	1,079455704	RPS17;RPL32;RPS8;RPS3A;SEPP1
Nitrobenzene-225_mg/kg_in_CMC-Rat-Kidney-3d-up	5/287	0,405948482	0,611796832	0	0	1,197361016	1,079455704	RPL32;RPS3A;RPL10A;SEPP1;TPT1
Oxymetazoline-0.5_mg/kg_in_Water-Rat-Liver-5d-dn	5/287	0,405948482	0,611679786	0	0	1,197361016	1,079455704	EEF1B2;RPS17;RPL32;RPS19;RPS20
Phenacetin-619_mg/kg_in_Corn_Oil-Rat-Liver-1d-up	5/287	0,405948482	0,611562785	0	0	1,197361016	1,079455704	RPS19;RPS8;RPS20;RPS3A;RPL10A
Piclamilast-17_mg/kg_in_CMC-Rat-Heart-3d-dn	5/287	0,405948482	0,611445829	0	0	1,197361016	1,079455704	RPS19;COL5A3;RPS6;SEPP1;TPT1
Prednisone-68_mg/kg_in_CMC-Rat-Kidney-3d-up	5/287	0,405948482	0,611328918	0	0	1,197361016	1,079455704	RPS6;ARL6IP5;RPL10A;RPL39;TPT1
Proglumide-1125_mg/kg_in_CMC-Rat-Liver-1d-up	5/287	0,405948482	0,611212052	0	0	1,197361016	1,079455704	RPS17;RPS20;RPSA;RPL10A;TPT1
Valproic_Acid-1340_mg/kg_in_Water-Rat-Liver-1d-up	5/287	0,405948482	0,61109523	0	0	1,197361016	1,079455704	EEF1B2;RPS17;RPL32;RPS3A;RPL39
Valsartan-1000_mg/kg_in_Corn_Oil-Rat-Heart-1d-up	5/287	0,405948482	0,610978452	0	0	1,197361016	1,079455704	RAPGEF5;RPL39;SEPP1;TPT1;PDRG1
Sildenafil-90_uM_in_DMSO-Rat-Primary_rat_hepatocytes-1d-dn	4/223	0,408086965	0,61407966	0	0	1,232798607	1,104926543	COL5A3;ARL6IP5;RPS20;SEPP1
Allyl_Alcohol-25_mg/kg_in_Saline-Rat-Bone_marrow-1d-up	5/288	0,408821592	0,615067595	0	0	1,193203513	1,06729241	RPS17;NKG7;RPS3A;SLC16A3;TPT1
Bisphenol_A-100_mg/kg_in_Corn_Oil-Rat-Bone_marrow-0.25d-up	5/288	0,408821592	0,614950126	0	0	1,193203513	1,06729241	C1QA;RPS19;RPS6;RPS20;RPL39
Bisphenol_A-610_mg/kg_in_Corn_Oil-Rat-Bone_marrow-3d-dn	5/288	0,408821592	0,614832702	0	0	1,193203513	1,06729241	RPS19;ARL6IP5;NKG7;RPSA;RPL10A
Cadmium_Acetate-167_mg/kg_in_Water-Rat-Kidney-1d-dn	5/288	0,408821592	0,614715323	0	0	1,193203513	1,06729241	ARL2BP;FUNDC1;RPS6;RPS20;SEPP1
Capsaicin-35_mg/kg_in_CMC-Rat-Liver-5d-up	5/288	0,408821592	0,614597988	0	0	1,193203513	1,06729241	RPS17;RPS20;RPSA;RPL39;SEPP1
Citric_Acid-3000_mg/kg_in_Water-Rat-Liver-1d-dn	5/288	0,408821592	0,614480698	0	0	1,193203513	1,06729241	CLP1;RPL39;GOLGA7;SEPP1;TPT1
Gemfibrozil-100_mg/kg_in_Corn_Oil-Rat-Kidney-1d-up	5/288	0,408821592	0,614363454	0	0	1,193203513	1,06729241	CLP1;RPS8;RPS5;RAPGEF5;TPT1
Hydrocortisone-56_mg/kg_in_Corn_Oil-Rat-Bone_marrow-1d-up	5/288	0,408821592	0,614246253	0	0	1,193203513	1,06729241	C1QA;RPS17;RPS19;NKG7;SEPP1
Itraconazole-30_mg/kg_in_Corn_Oil-Rat-Liver-3d-up	5/288	0,408821592	0,614129098	0	0	1,193203513	1,06729241	RPS19;RPS8;RPS20;RPSA;SEPP1
Mevastatin-1200_mg/kg_in_CMC-Rat-Kidney-3d-up	5/288	0,408821592	0,614011987	0	0	1,193203513	1,06729241	EEF1B2;RPS17;CXCL11;HOXA1;SEPP1
Mitomycin_C-1.7_mg/kg_in_Saline-Rat-Liver-1d-up	5/288	0,408821592	0,613894921	0	0	1,193203513	1,06729241	C1QA;RPS17;RPS3A;RPL39;TPT1
NN-Dimethylformamide-140_mg/kg_in_Saline-Rat-Spleen-0.25d-up	5/288	0,408821592	0,613777899	0	0	1,193203513	1,06729241	C1QA;RPS17;CXCL11;NKG7;RPSA
Rolipram-75_mg/kg_in_CMC-Rat-Liver-3d-up	5/288	0,408821592	0,613660922	0	0	1,193203513	1,06729241	EEF1B2;RPS8;RPS3A;RPL39;TPT1
Rosiglitazone-1800_mg/kg_in_Corn_Oil-Rat-Heart-5d-up	5/288	0,408821592	0,61354399	0	0	1,193203513	1,06729241	RPS8;RPS6;RPS20;SEPP1;TPT1
Betamethasone-79_mg/kg_in_Corn_Oil-Rat-Bone_marrow-1d-dn	5/289	0,411692896	0,617735426	0	0	1,189074781	1,055277239	FEM1B;NKG7;RPS20;RPL39;TPT1
Bromfenac-5_mg/kg_in_CMC-Rat-Kidney-5d-up	5/289	0,411692896	0,617617762	0	0	1,189074781	1,055277239	RPS17;CLP1;RPS20;RPSA;RPS3A
Chloroform-60_mg/kg_in_Water-Rat-Liver-0.25d-dn	5/289	0,411692896	0,617500143	0	0	1,189074781	1,055277239	RPL32;RPL35A;RPS3A;RPL39;TPT1
Ciprofloxacin-450_mg/kg_in_Corn_Oil-Rat-Liver-3d-up	5/289	0,411692896	0,617382569	0	0	1,189074781	1,055277239	C1QA;RPL39;GOLGA7;SEPP1;TPT1
Cisapride-250_mg/kg_in_Corn_Oil-Rat-Intestine-3d-up	5/289	0,411692896	0,617265039	0	0	1,189074781	1,055277239	RPS19;RPS6;RPS3A;RPL10A;TPT1
Famotidine-1200_mg/kg_in_CMC-Rat-Heart-3d-dn	5/289	0,411692896	0,617147554	0	0	1,189074781	1,055277239	ARL2BP;RPS8;FUNDC1;RPSA;TPT1
Indomethacin-10_mg/kg_in_Corn_Oil-Rat-Liver-3d-dn	5/289	0,411692896	0,617030114	0	0	1,189074781	1,055277239	RPS17;RPL32;RPL35A;GOLGA7;SEPP1
Leucovorin-1500_mg/kg_in_CMC-Rat-Bone_marrow-1d-up	5/289	0,411692896	0,616912719	0	0	1,189074781	1,055277239	NKG7;RPSA;RPL10A;SEPP1;TPT1
Mevastatin-1200_mg/kg_in_CMC-Rat-Kidney-1d-up	5/289	0,411692896	0,616795368	0	0	1,189074781	1,055277239	RPS17;RPS19;HOXA1;RPS20;SEPP1
Mianserin-116_mg/kg_in_Corn_Oil-Rat-Heart-3d-up	5/289	0,411692896	0,616678062	0	0	1,189074781	1,055277239	RPS17;RPL32;RPL35A;RPS20;TPT1
Nadolol-1500_mg/kg_in_CMC-Rat-Heart-3d-dn	5/289	0,411692896	0,616560801	0	0	1,189074781	1,055277239	ARL2BP;FUNDC1;RPS20;SEPP1;TPT1
Penicillamine-100_mg/kg_in_Water-Rat-Liver-5d-dn	5/289	0,411692896	0,616443584	0	0	1,189074781	1,055277239	C1QA;RPS3A;RPL10A;RPL39;TPT1
Risperidone-22_mg/kg_in_CMC-Rat-Heart-3d-dn	5/289	0,411692896	0,616326411	0	0	1,189074781	1,055277239	FEM1B;COL5A3;RPS6;RPL10A;RPL39
SCH-351591-4.6_mg/kg_in_CMC-Rat-Liver-6d-up	5/289	0,411692896	0,616209284	0	0	1,189074781	1,055277239	CXCL11;RPS6;RPL10A;RPL39;SEPP1
Sodium_Orthovanadate-83_mg/kg_in_CMC-Rat-Kidney-3d-dn	5/289	0,411692896	0,6160922	0	0	1,189074781	1,055277239	ARL2BP;RPS6;RPS20;RPL10A;SEPP1
Stavudine-1400_mg/kg_in_Water-Rat-Heart-3d-dn	5/289	0,411692896	0,615975161	0	0	1,189074781	1,055277239	RPL32;COL5A3;RPL35A;RPS20;RPS3A
Amikacin-160_mg/kg_in_Saline-Rat-Kidney-28d-up	6/355	0,413452303	0,618490092	0	0	1,161608828	1,025948159	RPS19;FEM1B;ARL6IP5;RPSA;RPL10A;SEPP1
Allyl_Alcohol-16_mg/kg_in_Saline-Rat-Liver-3d-up	5/290	0,414562253	0,62003272	0	0	1,184974523	1,043408139	C1QA;RPS19;RPS20;RPSA;SEPP1
Amlodipine-19_mg/kg_in_Corn_Oil-Rat-Liver-3d-dn	5/290	0,414562253	0,619915	0	0	1,184974523	1,043408139	CLP1;RPS6;RPL10A;RPL39;SEPP1
Amprenavir-600_mg/kg_in_CMC-Rat-Kidney-3d-dn	5/290	0,414562253	0,619797324	0	0	1,184974523	1,043408139	RPL32;RPS19;RPS20;GOLGA7;SEPP1
Auranofin-33_mg/kg_in_CMC-Rat-Kidney-5d-dn	5/290	0,414562253	0,619679693	0	0	1,184974523	1,043408139	ARL2BP;CLP1;RPL39;SEPP1;TPT1
Dexibuprofen-239_mg/kg_in_CMC-Rat-Kidney-1d-dn	5/290	0,414562253	0,619562107	0	0	1,184974523	1,043408139	EEF1B2;RPL32;RPS20;RPS3A;SEPP1
Ethylene_Glycol-3525_mg/kg_in_Water-Rat-Bone_marrow-3d-up	5/290	0,414562253	0,619444565	0	0	1,184974523	1,043408139	RPS17;NKG7;RPL35A;RPL10A;DUSP14
Fenofibrate-100_mg/kg_in_Corn_Oil-Rat-Liver-3d-up	5/290	0,414562253	0,619327068	0	0	1,184974523	1,043408139	RPL32;RPS19;RPS8;RPS5;RPS20
Hydrocortisone-56_mg/kg_in_Corn_Oil-Rat-Heart-5d-up	5/290	0,414562253	0,619209616	0	0	1,184974523	1,043408139	CLP1;ARL6IP5;RPS20;SEPP1;PDRG1
Lead(IV)_Acetate-200_mg/kg_in_Saline-Rat-Spleen-3d-up	5/290	0,414562253	0,619092208	0	0	1,184974523	1,043408139	RPL32;RPS19;RPS6;RPL10A;SEPP1
Lincomycin-1200_mg/kg_in_CMC-Rat-Kidney-5d-dn	5/290	0,414562253	0,618974844	0	0	1,184974523	1,043408139	EEF1B2;RPL32;RPL35A;RPS20;RPS3A
Methimazole-100_mg/kg_in_Water-Rat-Liver-1d-dn	5/290	0,414562253	0,618857525	0	0	1,184974523	1,043408139	C1QA;CLP1;RPS6;SEPP1;TPT1
Methotrexate-27_mg/kg_in_Corn_Oil-Rat-Spleen-1d-dn	5/290	0,414562253	0,618740251	0	0	1,184974523	1,043408139	RPS19;RPS6;RPSA;RPL10A;RPL39
NN'-Diphenyl-P-Phenylenediamine-1000_mg/kg_in_CMC-Rat-Kidney-1d-up	5/290	0,414562253	0,618623021	0	0	1,184974523	1,043408139	RPS17;CLP1;RPS3A;GOLGA7;TPT1
Tenidap-75_mg/kg_in_Corn_Oil-Rat-Kidney-1d-dn	5/290	0,414562253	0,618505835	0	0	1,184974523	1,043408139	EEF1B2;RPS17;RPS19;RPS6;RPL35A
Thioguanine-24_mg/kg_in_Corn_Oil-Rat-Liver-1d-up	5/290	0,414562253	0,618388694	0	0	1,184974523	1,043408139	RPL32;RPS8;RPS3A;RPL39;TPT1
Trichloroethane-3000_mg/kg_in_Water-Rat-Liver-5d-dn	5/290	0,414562253	0,618271597	0	0	1,184974523	1,043408139	RPS19;CLP1;RPL39;SEPP1;TPT1
4-Nonylphenol-200_mg/kg_in_Corn_Oil-Rat-Kidney-5d-up	5/291	0,417429522	0,622429935	0	0	1,180902446	1,03168309	RPS19;ARL2BP;RPS6;RPS20;RPSA
Beta-Estradiol_3-Benzoate-2_mg/kg_in_Corn_Oil-Rat-Liver-1d-dn	5/291	0,417429522	0,622312118	0	0	1,180902446	1,03168309	RPS17;RPL32;RPS8;RPL35A;TPT1
Cyclosporin_A-70_mg/kg_in_Corn_Oil-Rat-Liver-5d-dn	5/291	0,417429522	0,622194345	0	0	1,180902446	1,03168309	EEF1B2;RPS17;RPL32;RPL39;TPT1
Doxorubicin-0.65_mg/kg_in_Saline-Rat-Liver-3d-dn	5/291	0,417429522	0,622076617	0	0	1,180902446	1,03168309	C1QA;CLP1;RPS8;RPSA;TPT1
Ethanol-3000_mg/kg_in_Saline-Rat-Liver-5d-up	5/291	0,417429522	0,621958933	0	0	1,180902446	1,03168309	C1QA;HOXA1;RAPGEF5;GOLGA7;SEPP1
Hydralazine-280_mg/kg_in_Water-Rat-Liver-0.25d-dn	5/291	0,417429522	0,621841293	0	0	1,180902446	1,03168309	EEF1B2;RPS17;RPS8;RPL35A;RPL39
Isoprenaline-15_mg/kg_in_Saline-Rat-Heart-3d-up	5/291	0,417429522	0,621723699	0	0	1,180902446	1,03168309	RPS20;RPSA;RPL10A;RPL39;SEPP1
Ketoconazole-114_mg/kg_in_Corn_Oil-Rat-Liver-5d-up	5/291	0,417429522	0,621606148	0	0	1,180902446	1,03168309	RPL32;RPS8;RPS20;RPS3A;TPT1
Leflunomide-30_mg/kg_in_Corn_Oil-Rat-Bone_marrow-3d-dn	5/291	0,417429522	0,621488642	0	0	1,180902446	1,03168309	RPS19;FEM1B;RPS6;RPSA;RPL10A
NN'-Diphenyl-P-Phenylenediamine-1000_mg/kg_in_CMC-Rat-Kidney-5d-up	5/291	0,417429522	0,621371181	0	0	1,180902446	1,03168309	EEF1B2;RPS17;CLP1;RPSA;RPS3A
Salicylamide-1300_mg/kg_in_CMC-Rat-Kidney-1d-dn	5/291	0,417429522	0,621253764	0	0	1,180902446	1,03168309	RPL32;RPS19;ARL2BP;RPS6;RPS20
Temafloxacin-1000_mg/kg_in_CMC-Rat-Kidney-1d-dn	5/291	0,417429522	0,621136391	0	0	1,180902446	1,03168309	RPS19;RPS6;RPL35A;RPSA;SEPP1
Timolol-900_mg/kg_in_Water-Rat-Heart-3d-dn	5/291	0,417429522	0,621019063	0	0	1,180902446	1,03168309	ARL2BP;RPS8;FUNDC1;RPS6;RPS20
Tropisetron-100_mg/kg_in_CMC-Rat-Liver-1d-dn	5/291	0,417429522	0,620901779	0	0	1,180902446	1,03168309	RPS17;ARL6IP5;RPL35A;RPS20;SEPP1
Zomepirac-11_mg/kg_in_Saline-Rat-Liver-1d-up	5/291	0,417429522	0,620784539	0	0	1,180902446	1,03168309	C1QA;RPS17;RPL35A;RPS20;RPL39
Minoxidil-83_mg/kg_in_CMC-Rat-Heart-1d-up	6/357	0,418626951	0,622447776	0	0	1,155101216	1,005833361	C1QA;RPS19;RPS8;RAPGEF5;SEPP1;RPL39
Azauridine-1500_mg/kg_in_CMC-Rat-Bone_marrow-1d-up	5/292	0,420294567	0,624809363	0	0	1,176858259	1,020100109	RPS19;NKG7;RPS20;RPSA;RPL10A
Bisphenol_A-610_mg/kg_in_Corn_Oil-Rat-Kidney-1d-up	5/292	0,420294567	0,624691453	0	0	1,176858259	1,020100109	CLP1;RPS5;SEPP1;GOLGA7;TPT1
Closantel-22_mg/kg_in_CMC-Rat-Liver-3d-dn	5/292	0,420294567	0,624573586	0	0	1,176858259	1,020100109	EEF1B2;CLP1;RPS6;RPS3A;SEPP1
Dichlorvos-17_mg/kg_in_Water-Rat-Liver-5d-up	5/292	0,420294567	0,624455764	0	0	1,176858259	1,020100109	RPS17;RPS3A;RAPGEF5;SEPP1;GOLGA7
Ethylene_Glycol-352_mg/kg_in_Water-Rat-Bone_marrow-3d-up	5/292	0,420294567	0,624337987	0	0	1,176858259	1,020100109	RPS17;NKG7;RPL35A;RPL10A;DUSP14
Genistein-375_mg/kg_in_Corn_Oil-Rat-Liver-0.25d-dn	5/292	0,420294567	0,624220254	0	0	1,176858259	1,020100109	RPS17;RPS8;RPSA;RPL39;TPT1
Ifosfamide-143_mg/kg_in_Saline-Rat-Liver-0.25d-up	5/292	0,420294567	0,624102565	0	0	1,176858259	1,020100109	C1QA;RPS17;RPS6;RPSA;RPS3A
Nimesulide-61.7_uM_in_DMSO-Rat-Primary_rat_hepatocytes-0.67d-up	5/292	0,420294567	0,623984921	0	0	1,176858259	1,020100109	C1QA;RPS6;RPL10A;SLC16A3;PDRG1
Ritonavir-1200_mg/kg_in_CMC-Rat-Kidney-5d-dn	5/292	0,420294567	0,623867321	0	0	1,176858259	1,020100109	EEF1B2;RPS17;RPL32;RPS8;SEPP1
Rofecoxib-1550_mg/kg_in_Corn_Oil-Rat-Liver-3d-dn	5/292	0,420294567	0,623749766	0	0	1,176858259	1,020100109	RPS17;RPS6;RPS20;RPL39;TPT1

Sulfanilamide-636_mg/kg_in_Corn_Oil-Rat-Kidney-1d-dn	5/292	0,420294567	0,623632255	0	0	1,176858259	1,020100109	CLP1;RPSA;SEPP1;GOLGA7;TPT1
Thalidomide-113_mg/kg_in_CMC-Rat-Liver-5d-up	5/292	0,420294567	0,623514788	0	0	1,176858259	1,020100109	RPS17;RPL32;RPSA;RPL10A;DUSP14
Tolazamide-1500_mg/kg_in_CMC-Rat-Liver-3d-dn	5/292	0,420294567	0,623397365	0	0	1,176858259	1,020100109	RPL32;CLP1;RPS8;RPS6;RPL35A
3-Acetamidophenol-700_mg/kg_in_Corn_Oil-Rat-Liver-1d-dn	4/227	0,421143559	0,624539008	0	0	1,211075283	1,04731551	EEF1B2;RPL32;RPS8;RPL10A
4-Methylpyrazole-155_mg/kg_in_Saline-Rat-Liver-3d-dn	5/293	0,423157249	0,627407095	0	0	1,172841678	1,00865724	CLP1;RPS8;RPS3A;SEPP1;TPT1
Beta-Estradiol-150_mg/kg_in_Corn_Oil-Rat-Kidney-1d-up	5/293	0,423157249	0,627289006	0	0	1,172841678	1,00865724	ARL2BP;CLP1;RPS8;RPS20;RPS3A
Clofibrate-130_mg/kg_in_Corn_Oil-Rat-Kidney-1d-up	5/293	0,423157249	0,627170962	0	0	1,172841678	1,00865724	RPS19;RPS5;RPS6;RAPGEF5;RPL10A
Colchicine-0.6_mg/kg_in_Saline-Rat-Kidney-1d-dn	5/293	0,423157249	0,627052962	0	0	1,172841678	1,00865724	EEF1B2;CLP1;ARL6IP5;RPSA;TPT1
Furosemide-375_mg/kg_in_CMC-Rat-Kidney-5d-dn	5/293	0,423157249	0,626935006	0	0	1,172841678	1,00865724	RPS17;CLP1;RPS6;RPS3A;RPL10A
Lead(IV)_Acetate-200_mg/kg_in_Saline-Rat-Kidney-0.25d-dn	5/293	0,423157249	0,626817094	0	0	1,172841678	1,00865724	RPL32;ARL2BP;RPS6;RPS3A;SEPP1
Methotrexate-27_mg/kg_in_Corn_Oil-Rat-Liver-0.25d-up	5/293	0,423157249	0,626699227	0	0	1,172841678	1,00865724	RPS19;RPS6;RPS3A;RPL10A;RPL39
Oxfendazole-1500_mg/kg_in_CMC-Rat-Liver-3d-dn	5/293	0,423157249	0,626581405	0	0	1,172841678	1,00865724	CLP1;ARL6IP5;RPS3A;SEPP1;TPT1
Pentobarbital-70_mg/kg_in_Water-Rat-Liver-5d-up	5/293	0,423157249	0,626463626	0	0	1,172841678	1,00865724	EEF1B2;RPS19;RPS6;RPL10A;PDRG1
Sulfadimethoxine-1100_mg/kg_in_CMC-Rat-Kidney-1d-dn	5/293	0,423157249	0,626345892	0	0	1,172841678	1,00865724	RPS6;RPL10A;SEPP1;RPL39;TPT1
Tiapride-1200_mg/kg_in_Water-Rat-Heart-1d-dn	5/293	0,423157249	0,626228202	0	0	1,172841678	1,00865724	EEF1B2;RPS19;ARL2BP;RPS8;RPS20
Tinidazole-1200_mg/kg_in_CMC-Rat-Liver-5d-dn	5/293	0,423157249	0,626110556	0	0	1,172841678	1,00865724	EEF1B2;C1QA;SEPP1;RPL39;TPT1
Valproic_Acid-3168_uM_in_DMSO-Rat-Primary_rat_hepatocytes-1d-up	5/293	0,423157249	0,625992955	0	0	1,172841678	1,00865724	CXCL11;RPS6;RPSA;RPL39;SLC16A3
Bithionol-59_mg/kg_in_Corn_Oil-Rat-Liver-5d-dn	5/294	0,426017433	0,630105784	0	0	1,168852421	0,997352563	C1QA;RPS8;ARL6IP5;RPL10A;SEPP1
Candesartan-1300_mg/kg_in_CMC-Rat-Heart-5d-dn	5/294	0,426017433	0,629987476	0	0	1,168852421	0,997352563	RPS17;RPS8;RPSA;SEPP1;TPT1
Cetraxate-1503_mg/kg_in_CMC-Rat-Liver-5d-dn	5/294	0,426017433	0,629869213	0	0	1,168852421	0,997352563	C1QA;RPS6;RPL10A;SEPP1;TPT1
Clofibric_Acid-448_mg/kg_in_Corn_Oil-Rat-Liver-1d-up	5/294	0,426017433	0,629750995	0	0	1,168852421	0,997352563	RPS17;RPL32;RPS19;RPS8;RPSA
Coumarin-111_mg/kg_in_Corn_Oil-Rat-Bone_marrow-3d-dn	5/294	0,426017433	0,62963282	0	0	1,168852421	0,997352563	RPS17;ARL6IP5;RPSA;SEPP1;TPT1
Isotretinoin-125_mg/kg_in_Corn_Oil-Rat-Liver-3d-dn	5/294	0,426017433	0,62951469	0	0	1,168852421	0,997352563	RPS17;RPS3A;RPL10A;SEPP1;RPL39
Norethindrone-0.08_mg/kg_in_Corn_Oil-Rat-Liver-3d-dn	5/294	0,426017433	0,629396605	0	0	1,168852421	0,997352563	RPS17;CLP1;RPS3A;RPL10A;TPT1
Pemoline-70_mg/kg_in_CMC-Rat-Liver-1d-dn	5/294	0,426017433	0,629278563	0	0	1,168852421	0,997352563	C1QA;RPS17;RPS19;FEM1B;RPL10A
Phenelzine-27_mg/kg_in_Water-Rat-Heart-5d-dn	5/294	0,426017433	0,629160566	0	0	1,168852421	0,997352563	C1QA;RPS6;RPSA;TOMM20;PDRG1
Raloxifene-5_mg/kg_in_Corn_Oil-Rat-Liver-3d-dn	5/294	0,426017433	0,629042613	0	0	1,168852421	0,997352563	RPS17;CLP1;RPSA;RPS3A;SEPP1
Zomepirac-11_mg/kg_in_Saline-Rat-Liver-3d-up	5/294	0,426017433	0,628924705	0	0	1,168852421	0,997352563	EEF1B2;RPS17;RPS20;RPS3A;SEPP1
1-Naphthyl_Isothiocyanate-166.67_uM_in_DMSO-Rat-Primary_rat_hepatocytes-1d-dn	4/229	0,427646893	0,631211943	0	0	1,200498207	1,019772135	ARL6IP5;RPS20;RPL39;SEPP1
Catechol-40_mg/kg_in_Saline-Rat-Liver-1d-dn	4/229	0,427646893	0,631093672	0	0	1,200498207	1,019772135	RPS8;RPS6;RPL39;SEPP1
Sulindac-64_mg/kg_in_Corn_Oil-Rat-Kidney-3d-dn	4/229	0,427646893	0,630975445	0	0	1,200498207	1,019772135	ARL2BP;RPL39;SEPP1;TPT1
2-Nitroanisole-740_mg/kg_in_CMC-Rat-Kidney-3d-up	5/295	0,428874984	0,632668922	0	0	1,164890209	0,986184187	RPS17;CLP1;RPS8;RPSA;RPS3A
Alprazolam-115_mg/kg_in_Corn_Oil-Rat-Liver-3d-up	5/295	0,428874984	0,632550444	0	0	1,164890209	0,986184187	RPL32;RPS8;RPS6;RPS3A;SEPP1
Benoxaprofen-20_mg/kg_in_CMC-Rat-Liver-5d-dn	5/295	0,428874984	0,632432011	0	0	1,164890209	0,986184187	RPS6;RPS20;RPL10A;SEPP1;RPL39
Bisphenol_A-150_mg/kg_in_Corn_Oil-Rat-Liver-1d-dn	5/295	0,428874984	0,632313623	0	0	1,164890209	0,986184187	RPS8;RPS6;RPL10A;SEPP1;TPT1
Cyproterone_Acetate-2500_mg/kg_in_CMC-Rat-Liver-3d-dn	5/295	0,428874984	0,632195278	0	0	1,164890209	0,986184187	EEF1B2;C1QA;RPS8;ARL6IP5;RPL35A
Ethanol-6000_mg/kg_in_Saline-Rat-Liver-1d-up	5/295	0,428874984	0,632076978	0	0	1,164890209	0,986184187	RPS17;RPS8;RPSA;RPL39;TPT1
Ethylene_Glycol-352_mg/kg_in_Water-Rat-Kidney-1d-dn	5/295	0,428874984	0,631958723	0	0	1,164890209	0,986184187	RPL32;RPS19;RPS8;RPSA;RPL10A
Glimepiride-2500_mg/kg_in_CMC-Rat-Liver-1d-dn	5/295	0,428874984	0,631840511	0	0	1,164890209	0,986184187	EEF1B2;C1QA;RPS17;RPS19;RPL35A
Lacidipine-14_mg/kg_in_Corn_Oil-Rat-Heart-1d-dn	5/295	0,428874984	0,631722344	0	0	1,164890209	0,986184187	ARL2BP;RPS8;FUNDC1;ARL6IP5;SEPP1
Losartan-1000_mg/kg_in_Water-Rat-Heart-3d-dn	5/295	0,428874984	0,631604221	0	0	1,164890209	0,986184187	EEF1B2;RPS20;SEPP1;RPL39;TPT1
Mefenamic_Acid-93_mg/kg_in_Corn_Oil-Rat-Kidney-3d-up	5/295	0,428874984	0,631486142	0	0	1,164890209	0,986184187	C1QA;RPS8;RPS3A;RAPGEF5;TPT1
Mefenamic_Acid-93_mg/kg_in_Corn_Oil-Rat-Liver-5d-dn	5/295	0,428874984	0,631368107	0	0	1,164890209	0,986184187	EEF1B2;RPL32;RPS6;ARL6IP5;RPL10A
Methyl_Methanesulfonate-113_mg/kg_in_Water-Rat-Bone_marrow-5d-dn	5/295	0,428874984	0,631250116	0	0	1,164890209	0,986184187	RPS17;RPS20;RPS3A;RPL39;TPT1
NN-Dimethylformamide-1400_mg/kg_in_Saline-Rat-Kidney-1d-dn	5/295	0,428874984	0,63113217	0	0	1,164890209	0,986184187	EEF1B2;ARL2BP;CLP1;RPL10A;SEPP1
Nicotine-75_mg/kg_in_Water-Rat-Brain-5d-up	5/295	0,428874984	0,631014267	0	0	1,164890209	0,986184187	RPL32;RPS19;RPS6;RPL10A;SEPP1
Pantoprazole-1100_mg/kg_in_Water-Rat-Liver-1d-dn	5/295	0,428874984	0,630896409	0	0	1,164890209	0,986184187	C1QA;CLP1;RPL10A;SEPP1;TPT1
Pantoprazole-650_uM_in_DMSO-Rat-Primary_rat_hepatocytes-1d-up	5/295	0,428874984	0,630778594	0	0	1,164890209	0,986184187	EEF1B2;RPS17;RPS8;RPS3A;PDRG1
Pioglitazone-1500_mg/kg_in_Corn_Oil-Rat-Heart-1d-dn	5/295	0,428874984	0,630660824	0	0	1,164890209	0,986184187	RPL32;ARL2BP;RPL35A;RPS20;GOLGA7
Procarbazine-54_mg/kg_in_Water-Rat-Liver-1d-up	5/295	0,428874984	0,630543097	0	0	1,164890209	0,986184187	C1QA;RPS17;RPSA;RPL39;TPT1
Promazine-100_mg/kg_in_Corn_Oil-Rat-Liver-1d-dn	5/295	0,428874984	0,630425415	0	0	1,164890209	0,986184187	EEF1B2;RPS17;RPS8;RPSA;RPL39
Sulbactam-671_mg/kg_in_Saline-Rat-Kidney-3d-dn	5/295	0,428874984	0,630307776	0	0	1,164890209	0,986184187	RPS8;RPS6;RPS3A;RPL39;TPT1
Sulindac-64_mg/kg_in_Corn_Oil-Rat-Liver-3d-dn	5/295	0,428874984	0,630190182	0	0	1,164890209	0,986184187	RPS17;RPS8;RPSA;RPS3A;TPT1
Tamoxifen-64_mg/kg_in_Corn_Oil-Rat-Liver-1d-dn	5/295	0,428874984	0,630072631	0	0	1,164890209	0,986184187	RPS17;RPL32;RPL10A;SEPP1;TPT1
Abamectin-10_mg/kg_in_Water-Rat-Liver-5d-up	5/296	0,431729769	0,634148389	0	0	1,160954769	0,975150251	RPS17;RPS5;SEPP1;RPL39;GOLGA7
Acetaminophen-486_mg/kg_in_Corn_Oil-Rat-Liver-1d-dn	5/296	0,431729769	0,634030144	0	0	1,160954769	0,975150251	C1QA;CLP1;SEPP1;RPL39;TPT1
Carboplatin-14_mg/kg_in_Saline-Rat-Kidney-5d-up	5/296	0,431729769	0,633911943	0	0	1,160954769	0,975150251	RPS19;RPS6;RPSA;RPL10A;RPL39
Cholic_Acid-1402_mg/kg_in_Water-Rat-Liver-3d-up	5/296	0,431729769	0,633793786	0	0	1,160954769	0,975150251	RPS19;RPS8;RPS6;RPSA;GOLGA7
Cyclosporin_A-70_mg/kg_in_Corn_Oil-Rat-Liver-1d-dn	5/296	0,431729769	0,633675673	0	0	1,160954769	0,975150251	EEF1B2;RPS17;SEPP1;RPL39;TPT1
Daunorubicin-3.25_mg/kg_in_Saline-Rat-Heart-5d-dn	5/296	0,431729769	0,633557605	0	0	1,160954769	0,975150251	ARL6IP5;NKG7;RPS3A;RAPGEF5;TPT1
Fluoxetine-52_mg/kg_in_CMC-Rat-Liver-5d-dn	5/296	0,431729769	0,63343958	0	0	1,160954769	0,975150251	EEF1B2;RPS6;RPS20;RPL39;TPT1
Hexachlorophene-8_mg/kg_in_Corn_Oil-Rat-Liver-3d-dn	5/296	0,431729769	0,633321599	0	0	1,160954769	0,975150251	FEM1B;RPS6;ARL6IP5;RPL10A;TPT1
Lomustine-4.2_mg/kg_in_Corn_Oil-Rat-Spleen-0.25d-dn	5/296	0,431729769	0,633203662	0	0	1,160954769	0,975150251	C1QA;RPS17;RPL32;RPS5;RPL35A
Mefenamic_Acid-93_mg/kg_in_Corn_Oil-Rat-Liver-0.25d-up	5/296	0,431729769	0,633085769	0	0	1,160954769	0,975150251	CXCL11;RPS19;RPS8;RPS6;SEPP1
Mianserin-116_mg/kg_in_Corn_Oil-Rat-Heart-5d-up	5/296	0,431729769	0,63296792	0	0	1,160954769	0,975150251	C1QA;COL5A3;RPS20;RAPGEF5;TPT1
NN-Dimethylformamide-1400_mg/kg_in_Saline-Rat-Liver-5d-dn	5/296	0,431729769	0,632850114	0	0	1,160954769	0,975150251	C1QA;RPL32;RPS19;SEPP1;TPT1
Pioglitazone-1500_mg/kg_in_Corn_Oil-Rat-Liver-5d-up	5/296	0,431729769	0,632732353	0	0	1,160954769	0,975150251	EEF1B2;RPS19;RPS6;RPS20;RPL10A
Tamoxifen-64_mg/kg_in_Corn_Oil-Rat-Liver-0.25d-up	5/296	0,431729769	0,632614635	0	0	1,160954769	0,975150251	RPS19;RPS8;RPS6;RPL10A;SEPP1
Thioguanine-24_mg/kg_in_Corn_Oil-Rat-Liver-3d-dn	5/296	0,431729769	0,632496961	0	0	1,160954769	0,975150251	EEF1B2;RPS17;RPS6;RPS3A;RPL10A
Tocainide-67_mg/kg_in_Corn_Oil-Rat-Liver-3d-dn	5/296	0,431729769	0,632379331	0	0	1,160954769	0,975150251	EEF1B2;C1QA;RPS17;RPL10A;RPL39
Ethylene_Glycol-3525_mg/kg_in_Water-Rat-Liver-0.25d-up	6/363	0,43411591	0,635756212	0	0	1,136008634	0,947935254	EEF1B2;RPS19;RPS6;RPS20;RPL10A;SEPP1
Acetaminophen-486_mg/kg_in_Corn_Oil-Rat-Kidney-3d-dn	5/297	0,434581657	0,636319973	0	0	1,157045831	0,964248925	CLP1;ARL6IP5;RPL10A;RPL39;TPT1
Acetazolamide-47_mg/kg_in_Corn_Oil-Rat-Kidney-0.25d-up	5/297	0,434581657	0,636201697	0	0	1,157045831	0,964248925	RPS17;FUNDC1;RPS6;RPS3A;RPL39
Acrolein-20_mg/kg_in_Water-Rat-Kidney-3d-up	5/297	0,434581657	0,636083466	0	0	1,157045831	0,964248925	CLP1;RPS3A;RPL10A;SEPP1;TPT1
Bezafibrate-100_mg/kg_in_Corn_Oil-Rat-Liver-1d-dn	5/297	0,434581657	0,635965279	0	0	1,157045831	0,964248925	C1QA;RPSA;RPL10A;SEPP1;RPL39
Cadmium_Chloride-66_mg/kg_in_Corn_Oil-Rat-Kidney-3d-dn	5/297	0,434581657	0,635847136	0	0	1,157045831	0,964248925	EEF1B2;CLP1;ARL6IP5;SEPP1;RPL39
Carbon_Tetrachloride-400_mg/kg_in_Corn_Oil-Rat-Bone_marrow-1d-dn	5/297	0,434581657	0,635729036	0	0	1,157045831	0,964248925	EEF1B2;RPS19;ARL6IP5;RPSA;SEPP1
Cimetidine-2000_mg/kg_in_CMC-Rat-Heart-5d-dn	5/297	0,434581657	0,635610981	0	0	1,157045831	0,964248925	EEF1B2;RPS19;RPS8;ARL6IP5;SEPP1
Clonazepam-2500_mg/kg_in_CMC-Rat-Heart-3d-up	5/297	0,434581657	0,635492969	0	0	1,157045831	0,964248925	EEF1B2;C1QA;CLP1;FUNDC1;TPT1
Erythromycin-1500_mg/kg_in_CMC-Rat-Liver-1d-up	5/297	0,434581657	0,635375001	0	0	1,157045831	0,964248925	RPS17;RPS8;RPL35A;RPSA;TPT1
Hydralazine-280_mg/kg_in_Water-Rat-Liver-5d-dn	5/297	0,434581657	0,635257077	0	0	1,157045831	0,964248925	EEF1B2;RPS19;RPS6;RPSA;TPT1
Lomustine-560_uM_in_DMSO-Rat-Primary_rat_hepatocytes-1d-dn	5/297	0,434581657	0,635139197	0	0	1,157045831	0,964248925	RPS19;RPS8;RPS20;SEPP1;TPT1
Miconazole-200_mg/kg_in_Corn_Oil-Rat-Liver-1d-dn	5/297	0,434581657	0,63502136	0	0	1,157045831	0,964248925	C1QA;RPS8;RPSA;SEPP1;TPT1
Mitomycin_C-1.7_mg/kg_in_Saline-Rat-Liver-5d-dn	5/297	0,434581657	0,634903568	0	0	1,157045831	0,964248925	C1QA;RPL32;RPSA;RPL10A;SEPP1
Modafinil-17.5_mg/kg_in_Saline-Rat-Liver-1d-up	5/297	0,434581657	0,634785818	0	0	1,157045831	0,964248925	C1QA;RPS19;CLP1;RPS20;GOLGA7
Phenacemide-400_mg/kg_in_CMC-Rat-Kidney-1d-dn	5/297	0,434581657	0,634668113	0	0	1,157045831	0,964248925	EEF1B2;RPS19;ARL2BP;CLP1;RPS20
Rifabutin-1500_mg/kg_in_CMC-Rat-Liver-3d-dn	5/297	0,434581657	0,634550451	0	0	1,157045831	0,964248925	C1QA;RPS17;RPS19;SEPP1;RPL39
Ticrynafen-570_mg/kg_in_CMC-Rat-Liver-5d-dn	5/297	0,434581657	0,634432833	0	0	1,157045831	0,964248925	C1QA;RPS17;CLP1;RPS20;SEPP1
Vecuronium_Bromide-0.05_mg/kg_in_Saline-Rat-Liver-5d-up	5/297	0,434581657	0,634315258	0	0	1,157045831	0,964248925	EEF1B2;C1QA;RPS6;RPS20;RPL10A
Sulfathiazole-2629_mg/kg_in_Water-Rat-Kidney-5d-up	6/364	0,436691229	0,637276287	0	0	1,132887731	0,938630229	EEF1B2;RPS8;RPS3A;RAPGEF5;SEPP1;TPT1

Cefotaxime-1000_mg/kg_in_Saline-Rat-Kidney-1d-dn	4/232	0,437366481	0,638143461	0	0	1,184974523	0,979954741	EEF1B2;RPS19;CLP1;RPL39
1-Naphthyl_Isothiocyanate-30_mg/kg_in_Corn_Oil-Rat-Bone_marrow-0.25d-up	5/298	0,437430517	0,63811868	0	0	1,153163126	0,953478406	C1QA;RPS19;RPL10A;RPL39;SLC16A3
2-Acetylaminofluorene-30_mg/kg_in_CMC-Rat-Liver-1d-dn	5/298	0,437430517	0,638000509	0	0	1,153163126	0,953478406	RPL32;CLP1;RPS8;RPL10A;SEPP1
Alfacalcidol-0.043_mg/kg_in_CMC-Rat-Liver-3d-up	5/298	0,437430517	0,637882383	0	0	1,153163126	0,953478406	C1QA;CXCL11;RPS3A;RPL39;TPT1
Aspirin-375_mg/kg_in_Corn_Oil-Rat-Liver-3d-up	5/298	0,437430517	0,6377643	0	0	1,153163126	0,953478406	EEF1B2;RPS19;RPS3A;RPL10A;SEPP1
Azathioprine-54_mg/kg_in_Water-Rat-Bone_marrow-5d-up	5/298	0,437430517	0,637646261	0	0	1,153163126	0,953478406	C1QA;NKG7;RPS3A;SEPP1;TPT1
Beta-Estradiol_3-Benzoate-0.25_mg/kg_in_Corn_Oil-Rat-Liver-0.25d-up	5/298	0,437430517	0,637528266	0	0	1,153163126	0,953478406	C1QA;RPS19;RPS6;RPL10A;SEPP1
Carmustine-4_mg/kg_in_Corn_Oil-Rat-Bone_marrow-0.25d-up	5/298	0,437430517	0,637410315	0	0	1,153163126	0,953478406	C1QA;RPL10A;RPL39;GOLGA7;DUSP14
Choline_Chloride-2550_mg/kg_in_Water-Rat-Liver-1d-dn	5/298	0,437430517	0,637292407	0	0	1,153163126	0,953478406	EEF1B2;RPS19;RPL10A;SEPP1;RPL39
Cimetidine-2000_mg/kg_in_CMC-Rat-Heart-3d-up	5/298	0,437430517	0,637174542	0	0	1,153163126	0,953478406	RPS19;COL5A3;RPL10A;SEPP1;RPL39
Cyclosporin_A-350_mg/kg_in_Corn_Oil-Rat-Liver-1d-dn	5/298	0,437430517	0,637056722	0	0	1,153163126	0,953478406	EEF1B2;RPS17;RPS3A;RPL39;TPT1
Daunorubicin-1_mg/kg_in_Saline-Rat-Kidney-5d-up	5/298	0,437430517	0,636938944	0	0	1,153163126	0,953478406	EEF1B2;CLP1;RPS8;RPSA;RAPGEF5
Doxorubicin-3_mg/kg_in_Saline-Rat-Liver-0.25d-dn	5/298	0,437430517	0,636821211	0	0	1,153163126	0,953478406	EEF1B2;C1QA;RPS17;CLP1;RPL10A
Flurbiprofen-10_mg/kg_in_CMC-Rat-Kidney-1d-up	5/298	0,437430517	0,636703521	0	0	1,153163126	0,953478406	EEF1B2;RPL32;RPS19;RPS8;RPS6
Oxfendazole-1500_mg/kg_in_CMC-Rat-Kidney-5d-dn	5/298	0,437430517	0,636585874	0	0	1,153163126	0,953478406	ARL2BP;RPS8;RPS6;RPL10A;GOLGA7
Phenobarbital-54_mg/kg_in_Water-Rat-Liver-1d-dn	5/298	0,437430517	0,636468271	0	0	1,153163126	0,953478406	EEF1B2;C1QA;RPS8;SEPP1;TPT1
Rosiglitazone-1800_mg/kg_in_Corn_Oil-Rat-Liver-1d-up	5/298	0,437430517	0,636350711	0	0	1,153163126	0,953478406	RPS8;RPS20;RPSA;RPS3A;RPL10A
Tranilast-1100_mg/kg_in_CMC-Rat-Liver-1d-dn	5/298	0,437430517	0,636233195	0	0	1,153163126	0,953478406	EEF1B2;RPS17;RPS20;SEPP1;RPL39
Prednisolone-184_mg/kg_in_Water-Rat-Kidney-5d-up	6/365	0,439264517	0,638782743	0	0	1,129783929	0,929420707	EEF1B2;CXCL11;RPS6;SEPP1;RPL39;TPT1
11-Dichloroethene-600_mg/kg_in_Water-Rat-Liver-5d-dn	5/299	0,440276219	0,640135776	0	0	1,149306394	0,942836924	CLP1;RPS8;SEPP1;RPL39;TPT1
Acyclovir-330_mg/kg_in_Saline-Rat-Liver-3d-dn	5/299	0,440276219	0,640017626	0	0	1,149306394	0,942836924	EEF1B2;RPL32;RPS8;RPSA;TPT1
Atorvastatin-300_mg/kg_in_Corn_Oil-Rat-Skeletal_muscle-3d-dn	5/299	0,440276219	0,63989952	0	0	1,149306394	0,942836924	RPS8;FEM1B;COL5A3;ARL6IP5;SLC16A3
Bupropion-895_mg/kg_in_Water-Rat-Liver-5d-dn	5/299	0,440276219	0,639781458	0	0	1,149306394	0,942836924	C1QA;RPS17;RPSA;SEPP1;RPL39
Captopril-1750_mg/kg_in_Water-Rat-Kidney-5d-dn	5/299	0,440276219	0,639663439	0	0	1,149306394	0,942836924	ARL2BP;CLP1;RPS8;RPSA;SEPP1
Carboplatin-14_mg/kg_in_Saline-Rat-Kidney-3d-dn	5/299	0,440276219	0,639545463	0	0	1,149306394	0,942836924	RPS17;CLP1;FUNDC1;RPS3A;RPL39
Carboplatin-6_mg/kg_in_Saline-Rat-Kidney-3d-up	5/299	0,440276219	0,639427531	0	0	1,149306394	0,942836924	RPS6;ARL6IP5;RPS20;RPSA;TPT1
Dichlorvos-17_mg/kg_in_Water-Rat-Liver-1d-dn	5/299	0,440276219	0,639309642	0	0	1,149306394	0,942836924	RPS8;FEM1B;SEPP1;RPL39;TPT1
Digoxin-0.26_mg/kg_in_Corn_Oil-Rat-Liver-1d-up	5/299	0,440276219	0,639191797	0	0	1,149306394	0,942836924	EEF1B2;RPS17;RPS19;RPS20;RPL39
Disulfiram-500_mg/kg_in_Water-Rat-Liver-1d-dn	5/299	0,440276219	0,639073996	0	0	1,149306394	0,942836924	C1QA;CLP1;RPS8;SEPP1;TPT1
Fenofibrate-100_mg/kg_in_Corn_Oil-Rat-Liver-1d-dn	5/299	0,440276219	0,638956237	0	0	1,149306394	0,942836924	C1QA;RPSA;SEPP1;RPL39;TPT1
Fenofibrate-43_mg/kg_in_Corn_Oil-Rat-Liver-1d-up	5/299	0,440276219	0,638838523	0	0	1,149306394	0,942836924	RPS19;RPS6;RPS20;RPL10A;SEPP1
Ibuprofen-263_mg/kg_in_Corn_Oil-Rat-Liver-3d-dn	5/299	0,440276219	0,638720851	0	0	1,149306394	0,942836924	RPS17;RPL32;CLP1;RPSA;RPS3A
Isoeugenol-1560_mg/kg_in_CMC-Rat-Kidney-3d-up	5/299	0,440276219	0,638603223	0	0	1,149306394	0,942836924	EEF1B2;RPS17;CXCL11;RPS3A;SEPP1
Leucovorin-1500_mg/kg_in_CMC-Rat-Bone_marrow-3d-up	5/299	0,440276219	0,638485638	0	0	1,149306394	0,942836924	RPL32;RPS19;RPSA;RPL10A;SEPP1
Lomefloxacin-2000_mg/kg_in_CMC-Rat-Liver-3d-up	5/299	0,440276219	0,638368096	0	0	1,149306394	0,942836924	RPS19;RPS6;RPSA;RPL10A;RPL39
Meloxicam-33_mg/kg_in_Corn_Oil-Rat-Liver-1d-dn	5/299	0,440276219	0,638250598	0	0	1,149306394	0,942836924	RPSA;RPL10A;SEPP1;RPL39;TPT1
Olanzapine-23_mg/kg_in_CMC-Rat-Heart-5d-up	5/299	0,440276219	0,638133143	0	0	1,149306394	0,942836924	CXCL11;RPS8;RPSA;TOMM20;TPT1
Pirinixic_Acid-364_mg/kg_in_CMC-Rat-Liver-3d-dn	5/299	0,440276219	0,638015731	0	0	1,149306394	0,942836924	C1QA;RPS17;CLP1;ARL6IP5;SEPP1
Ticlopidine-223_mg/kg_in_CMC-Rat-Liver-3d-dn	5/299	0,440276219	0,637898363	0	0	1,149306394	0,942836924	EEF1B2;C1QA;RPS17;ARL6IP5;SEPP1
Tretinoin-7_mg/kg_in_Corn_Oil-Rat-Liver-5d-up	5/299	0,440276219	0,637781037	0	0	1,149306394	0,942836924	C1QA;RPS20;RPSA;SEPP1;TPT1
Trovafloxacin-600_mg/kg_in_CMC-Rat-Liver-1d-up	5/299	0,440276219	0,637663755	0	0	1,149306394	0,942836924	EEF1B2;RPS8;RPS6;RPS3A;TPT1
Ubenimex-1300_mg/kg_in_Water-Rat-Kidney-3d-dn	4/233	0,440596177	0,638009835	0	0	1,179888795	0,967068149	ARL2BP;CLP1;RPS8;TPT1
Acetaminophen-972_mg/kg_in_Corn_Oil-Rat-Liver-3d-dn	5/300	0,443118635	0,641544554	0	0	1,145475372	0,932322732	RPS17;RPL32;RPL35A;SEPP1;TPT1
Allyl_Alcohol-16_mg/kg_in_Saline-Rat-Kidney-0.25d-dn	5/300	0,443118635	0,641426644	0	0	1,145475372	0,932322732	RPS19;ARL2BP;RPS6;RPS20;RPL10A
Amikacin-20_mg/kg_in_Saline-Rat-Kidney-1d-up	5/300	0,443118635	0,641308778	0	0	1,145475372	0,932322732	RPS8;FEM1B;RPS6;RPSA;RPL39
Amiodarone-147_mg/kg_in_CMC-Rat-Kidney-3d-dn	5/300	0,443118635	0,641190956	0	0	1,145475372	0,932322732	RPS17;RPL32;ARL2BP;RPS8;SEPP1
Catechol-195_mg/kg_in_Saline-Rat-Kidney-3d-dn	5/300	0,443118635	0,641073176	0	0	1,145475372	0,932322732	RPS19;R3HCC1;RPS6;RPL10A;TPT1
Crotamiton-750_mg/kg_in_Corn_Oil-Rat-Liver-1d-dn	5/300	0,443118635	0,64095544	0	0	1,145475372	0,932322732	EEF1B2;C1QA;SEPP1;RPL39;TPT1
Ethanol-3000_mg/kg_in_Saline-Rat-Liver-3d-dn	5/300	0,443118635	0,640837747	0	0	1,145475372	0,932322732	CLP1;RPSA;SEPP1;RPL39;TPT1
Lithocholic_Acid-74_uM_in_DMSO-Rat-Primary_rat_hepatocytes-0.67d-dn	5/300	0,443118635	0,640720098	0	0	1,145475372	0,932322732	RPS19;RPS8;RPS20;RPSA;SLC16A3
Maprotiline-380_mg/kg_in_CMC-Rat-Heart-1d-dn	5/300	0,443118635	0,640602491	0	0	1,145475372	0,932322732	EEF1B2;RPS8;RPSA;RPL10A;RPL39
Maprotiline-380_mg/kg_in_CMC-Rat-Heart-1d-up	5/300	0,443118635	0,640484928	0	0	1,145475372	0,932322732	RPL32;FUNDC1;RPS6;RPS20;TPT1
Naloxone-76_mg/kg_in_Saline-Rat-Liver-3d-dn	5/300	0,443118635	0,640367408	0	0	1,145475372	0,932322732	RPS19;RPS8;RPS6;RPL39;TPT1
Penciclovir-1200_mg/kg_in_Corn_Oil-Rat-Bone_marrow-5d-up	5/300	0,443118635	0,640249931	0	0	1,145475372	0,932322732	EEF1B2;C1QA;ARL6IP5;NKG7;SLC16A3
Praziquantel-1200_mg/kg_in_CMC-Rat-Liver-5d-up	5/300	0,443118635	0,640132497	0	0	1,145475372	0,932322732	RPS19;RPS20;RPSA;RPL10A;SEPP1
Rifampin-99_mg/kg_in_CMC-Rat-Liver-5d-up	5/300	0,443118635	0,640015106	0	0	1,145475372	0,932322732	RPS17;RPS5;RPSA;SEPP1;RPL39
Sildenafil-420_mg/kg_in_Water-Rat-Liver-1d-dn	5/300	0,443118635	0,639897758	0	0	1,145475372	0,932322732	RPS19;CLP1;RPSA;RPL10A;SEPP1
Sulconazole-1200_mg/kg_in_Corn_Oil-Rat-Liver-5d-up	5/300	0,443118635	0,639780453	0	0	1,145475372	0,932322732	C1QA;RPS17;RPS20;RPSA;SEPP1
Tetracaine-24_mg/kg_in_Corn_Oil-Rat-Heart-5d-dn	5/300	0,443118635	0,639663191	0	0	1,145475372	0,932322732	RPL32;RPS19;RPS6;SEPP1;TPT1
Ascorbic_Acid-2000_mg/kg_in_Water-Rat-Kidney-3d-up	6/367	0,444404606	0,641401993	0	0	1,123627068	0,911283865	RPS17;RPSA;RAPGEF5;SEPP1;GOLGA7;TPT1
Bezafibrate-617_mg/kg_in_Corn_Oil-Rat-Heart-1d-up	5/301	0,44595764	0,643525535	0	0	1,141669806	0,921934114	RPS6;ARL6IP5;RPL10A;RPL39;TPT1
Carboplatin-14_mg/kg_in_Saline-Rat-Kidney-3d-up	5/301	0,44595764	0,643407652	0	0	1,141669806	0,921934114	RPS19;RPS8;RPS6;RPSA;TPT1
Chloroform-60_mg/kg_in_Water-Rat-Liver-1d-dn	5/301	0,44595764	0,643289812	0	0	1,141669806	0,921934114	C1QA;RPS19;RPS8;RPS20;SEPP1
Clobetasol_Propionate-17_mg/kg_in_Corn_Oil-Rat-Bone_marrow-1d-up	5/301	0,44595764	0,643172014	0	0	1,141669806	0,921934114	EEF1B2;C1QA;RPS17;NKG7;TPT1
Epirubicin-2.7_mg/kg_in_Saline-Rat-Kidney-3d-up	5/301	0,44595764	0,643054261	0	0	1,141669806	0,921934114	CXCL11;RPS8;RAPGEF5;RPL10A;TPT1
Ethylestrenol-390_mg/kg_in_CMC-Rat-Liver-3d-dn	5/301	0,44595764	0,64293655	0	0	1,141669806	0,921934114	C1QA;RPL32;CLP1;SEPP1;TPT1
Fludrocortisone_Acetate-125_mg/kg_in_CMC-Rat-Kidney-3d-dn	5/301	0,44595764	0,642818882	0	0	1,141669806	0,921934114	RPS17;RPL32;ARL2BP;CLP1;RPS3A
Indomethacin-9.6_mg/kg_in_Water-Rat-Heart-3d-dn	5/301	0,44595764	0,642701257	0	0	1,141669806	0,921934114	EEF1B2;RPL32;RPS8;FUNDC1;RPS6
Lovastatin-1500_mg/kg_in_Corn_Oil-Rat-Liver-1d-dn	5/301	0,44595764	0,642583676	0	0	1,141669806	0,921934114	EEF1B2;RPS17;CLP1;RPS8;SEPP1
Loxoprofen-18_mg/kg_in_Corn_Oil-Rat-Kidney-3d-dn	5/301	0,44595764	0,642466137	0	0	1,141669806	0,921934114	EEF1B2;RPS20;SEPP1;GOLGA7;TPT1
Mesna-755_mg/kg_in_Saline-Rat-Kidney-3d-dn	5/301	0,44595764	0,642348641	0	0	1,141669806	0,921934114	EEF1B2;RPS20;RPS3A;RPL39;TPT1
NN-Dimethylformamide-140_mg/kg_in_Saline-Rat-Liver-3d-dn	5/301	0,44595764	0,642231189	0	0	1,141669806	0,921934114	CLP1;RPS8;RPS20;RPL39;TPT1
Staurosporine-1.3_uM_in_DMSO-Rat-Primary_rat_hepatocytes-0.67d-up	5/301	0,44595764	0,642113779	0	0	1,141669806	0,921934114	EEF1B2;CXCL11;ARL6IP5;SEPP1;GOLGA7
Thioguanine-12_mg/kg_in_Corn_Oil-Rat-Spleen-1d-up	5/301	0,44595764	0,641996412	0	0	1,141669806	0,921934114	EEF1B2;C1QA;RPS17;RPL32;RPS6
Tranilast-300_mg/kg_in_CMC-Rat-Liver-3d-up	5/301	0,44595764	0,641879088	0	0	1,141669806	0,921934114	RAPGEF5;RPL10A;SEPP1;RPL39;TPT1
Tranilast-300_mg/kg_in_CMC-Rat-Spleen-5d-dn	5/301	0,44595764	0,641761807	0	0	1,141669806	0,921934114	RPS17;RPS8;RPL35A;RPS3A;TPT1
Valdecoxib-21_mg/kg_in_CMC-Rat-Heart-5d-up	5/301	0,44595764	0,641644569	0	0	1,141669806	0,921934114	EEF1B2;RPS8;ARL6IP5;RPSA;RPL39
Valproic_Acid-3168_uM_in_DMSO-Rat-Primary_rat_hepatocytes-0.67d-dn	5/301	0,44595764	0,641527374	0	0	1,141669806	0,921934114	ARL2BP;ARL6IP5;RPL10A;SEPP1;GOLGA7
Zomepirac-11_mg/kg_in_Saline-Rat-Kidney-3d-dn	5/301	0,44595764	0,641410221	0	0	1,141669806	0,921934114	EEF1B2;RPL32;R3HCC1;RPS6;RPL10A
Bisphenol_A-100_mg/kg_in_Corn_Oil-Rat-Liver-1d-up	4/235	0,447039264	0,642848501	0	0	1,169847189	0,941854325	RPS19;CLP1;RPSA;RPL39
Zidovudine-1540_mg/kg_in_Corn_Oil-Rat-Heart-3d-dn	4/235	0,447039264	0,64273115	0	0	1,169847189	0,941854325	RPS17;RPL32;RPS19;RPS3A
2-Acetylaminofluorene-30_mg/kg_in_CMC-Rat-Liver-1d-up	5/302	0,448793107	0,645134971	0	0	1,137889443	0,91166938	RPS19;RPS6;RPSA;RPL39;TPT1
33'5-Triiodo-L-Thyronine-0.1_mg/kg_in_PEG400-Rat-Heart-1d-dn	5/302	0,448793107	0,645017246	0	0	1,137889443	0,91166938	C1QA;CXCL11;FUNDC1;SEPP1;TPT1
Ampicillin-2000_mg/kg_in_CMC-Rat-Kidney-1d-dn	5/302	0,448793107	0,644899564	0	0	1,137889443	0,91166938	RPS17;CLP1;RPS20;SEPP1;TPT1
Azaribine-750_mg/kg_in_CMC-Rat-Heart-1d-up	5/302	0,448793107	0,644781924	0	0	1,137889443	0,91166938	EEF1B2;RPS17;RPL32;RPS20;RPL39
Beta-Estradiol_3-Benzoate-0.25_mg/kg_in_Corn_Oil-Rat-Liver-0.25d-dn	5/302	0,448793107	0,644664328	0	0	1,137889443	0,91166938	RPS17;RPS8;RPL35A;RPS20;RPSA
Carmustine-4_mg/kg_in_Corn_Oil-Rat-Bone_marrow-3d-up	5/302	0,448793107	0,644546774	0	0	1,137889443	0,91166938	C1QA;RPS17;RPS5;RPL35A;DUSP14
Cimetidine-100_mg/kg_in_CMC-Rat-Heart-5d-dn	5/302	0,448793107	0,644429263	0	0	1,137889443	0,91166938	EEF1B2;RPS19;FUNDC1;ARL6IP5;SEPP1
Colchicine-0.6_mg/kg_in_Saline-Rat-Kidney-3d-dn	5/302	0,448793107	0,644311795	0	0	1,137889443	0,91166938	EEF1B2;RPS17;FUNDC1;ARL6IP5;RPL39
Diisopropyl_Fluorophosphate-3.75_mg/kg_in_Water-Rat-Liver-1d-up	5/302	0,448793107	0,64419437	0	0	1,137889443	0,91166938	C1QA;RPS17;RPS20;RPSA;RPL10A

Ebastine-2_mg/kg_in_Water-Rat-Heart-5d-up	5/302	0,448793107	0,644076988	0	0	1,137889443	0,91166938	RPS19;FUNDC1;ARL6IP5;RPSA;RPL39
Fenoprofen-52_mg/kg_in_Corn_Oil-Rat-Kidney-1d-dn	5/302	0,448793107	0,643959648	0	0	1,137889443	0,91166938	EEF1B2;ARL2BP;CLP1;SEPP1;TPT1
Finasteride-800_mg/kg_in_Corn_Oil-Rat-Kidney-3d-dn	5/302	0,448793107	0,643842351	0	0	1,137889443	0,91166938	RPS17;RPS19;RPL39;GOLGA7;TPT1
Fludrocortisone_Acetate-125_mg/kg_in_CMC-Rat-Heart-5d-up	5/302	0,448793107	0,643725097	0	0	1,137889443	0,91166938	EEF1B2;ARL6IP5;RAPGEF5;SEPP1;RPL39
Ibuprofen-263_mg/kg_in_Corn_Oil-Rat-Liver-1d-dn	5/302	0,448793107	0,643607886	0	0	1,137889443	0,91166938	C1QA;RPS17;CLP1;RPS6;RPS3A
Methyl_Methanesulfonate-113_mg/kg_in_Water-Rat-Bone_marrow-1d-up	5/302	0,448793107	0,643490717	0	0	1,137889443	0,91166938	C1QA;RPS19;NKG7;RPL10A;SEPP1
NN-Dimethylformamide-1400_mg/kg_in_Saline-Rat-Spleen-3d-up	5/302	0,448793107	0,643373591	0	0	1,137889443	0,91166938	RPS19;RPS5;RPL13A;SEPP1;RPL39
Penicillamine-100_mg/kg_in_Water-Rat-Bone_marrow-1d-up	5/302	0,448793107	0,643256507	0	0	1,137889443	0,91166938	NKG7;RPL10A;SEPP1;GOLGA7;TPT1
Pentobarbital-70_mg/kg_in_Water-Rat-Liver-1d-dn	5/302	0,448793107	0,643139467	0	0	1,137889443	0,91166938	C1QA;FUNDC1;RPS6;SEPP1;TPT1
Sodium_Arsenite-10_mg/kg_in_Saline-Rat-Liver-3d-up	5/302	0,448793107	0,643022468	0	0	1,137889443	0,91166938	EEF1B2;RPS19;RPS6;RPS20;RPL10A
Tobramycin-40_mg/kg_in_Saline-Rat-Kidney-5d-up	5/302	0,448793107	0,642905513	0	0	1,137889443	0,91166938	CXCL11;RPS6;RPSA;RPL39;TPT1
Streptomycin-120_mg/kg_in_Saline-Rat-Bone_marrow-1d-up	6/369	0,449535398	0,643851754	0	0	1,117536949	0,893516249	RPS8;FEM1B;RPS6;RPS3A;RPL10A;TPT1
Finasteride-800_mg/kg_in_Corn_Oil-Rat-Liver-0.25d-dn	4/236	0,450252311	0,644761309	0	0	1,164890209	0,929520837	RPS6;RPS3A;SEPP1;TPT1
Propranolol-175_mg/kg_in_Water-Rat-Heart-3d-dn	4/236	0,450252311	0,644644101	0	0	1,164890209	0,929520837	RPS8;COL5A3;SEPP1;TPT1
17-Methyltestosterone-2000_mg/kg_in_CMC-Rat-Liver-1d-dn	5/303	0,451624912	0,646491786	0	0	1,134134032	0,901526867	EEF1B2;C1QA;CLP1;RPL35A;SEPP1
Acetazolamide-47_mg/kg_in_Corn_Oil-Rat-Kidney-0.25d-dn	5/303	0,451624912	0,646374306	0	0	1,134134032	0,901526867	EEF1B2;RPS8;FEM1B;RPS20;SEPP1
Acetazolamide-47_mg/kg_in_Corn_Oil-Rat-Liver-5d-up	5/303	0,451624912	0,646256869	0	0	1,134134032	0,901526867	RPS8;RPS5;RPS20;RPSA;TPT1
Amlodipine-0.2_mg/kg_in_Corn_Oil-Rat-Liver-3d-up	5/303	0,451624912	0,646139475	0	0	1,134134032	0,901526867	RPL35A;RPS20;RPSA;SEPP1;RPL39
Azathioprine-20_mg/kg_in_Corn_Oil-Rat-Bone_marrow-0.25d-up	5/303	0,451624912	0,646022123	0	0	1,134134032	0,901526867	C1QA;NKG7;RPL35A;RPL39;GOLGA7
Carbimazole-400_mg/kg_in_Water-Rat-Liver-3d-dn	5/303	0,451624912	0,645904814	0	0	1,134134032	0,901526867	EEF1B2;C1QA;CLP1;RPL35A;TPT1
Colchicine-0.6_mg/kg_in_Saline-Rat-Kidney-5d-dn	5/303	0,451624912	0,645787547	0	0	1,134134032	0,901526867	EEF1B2;ARL6IP5;RPSA;SEPP1;TPT1
Esmolol-27_mg/kg_in_Saline-Rat-Heart-5d-dn	5/303	0,451624912	0,645670323	0	0	1,134134032	0,901526867	EEF1B2;RPL32;RPS8;ARL6IP5;RAPGEF5
Ethylene_Glycol-3525_mg/kg_in_Water-Rat-Kidney-5d-up	5/303	0,451624912	0,645553141	0	0	1,134134032	0,901526867	EEF1B2;RPS17;FUNDC1;RPS20;RPSA
Lacidipine-1_mg/kg_in_Corn_Oil-Rat-Heart-0.25d-up	5/303	0,451624912	0,645436002	0	0	1,134134032	0,901526867	RPS20;RPSA;RPL10A;RPL39;TPT1
Quetiapine-500_mg/kg_in_CMC-Rat-Liver-3d-up	5/303	0,451624912	0,645318906	0	0	1,134134032	0,901526867	RPS19;RPS6;RPL10A;RPL39;GOLGA7
Rifabutin-1500_mg/kg_in_CMC-Rat-Liver-3d-up	5/303	0,451624912	0,645201852	0	0	1,134134032	0,901526867	EEF1B2;RPS8;RPS20;RPS3A;TPT1
Simvastatin-1200_mg/kg_in_Corn_Oil-Rat-Skeletal_muscle-3d-dn	5/303	0,451624912	0,64508484	0	0	1,134134032	0,901526867	EEF1B2;COL5A3;ARL6IP5;SLC16A3;TPT1
Timolol-10_mg/kg_in_Water-Rat-Heart-1d-dn	5/303	0,451624912	0,644967871	0	0	1,134134032	0,901526867	ARL2BP;RPS8;RPS6;RPSA;TPT1
Vinorelbine-1.5_mg/kg_in_Saline-Rat-Liver-3d-dn	5/303	0,451624912	0,644850944	0	0	1,134134032	0,901526867	C1QA;RPS6;RPL39;GOLGA7;TPT1
Netilmicin-40_mg/kg_in_Saline-Rat-Kidney-5d-up	6/370	0,452097069	0,645408105	0	0	1,114516578	0,884768312	CXCL11;CLP1;RPS6;RPS20;RPL10A;TPT1
Etoposide-188_mg/kg_in_Corn_Oil-Rat-Liver-3d-dn	4/237	0,453459469	0,647235734	0	0	1,159975061	0,917365565	RPS17;CLP1;RPS3A;SEPP1
1-Naphthyl_Isothiocyanate-166.67_uM_in_DMSO-Rat-Primary_rat_hepatocytes-0.67d-dn	5/304	0,454452933	0,648536203	0	0	1,130403328	0,891504937	RPS19;ARL6IP5;RPS20;SEPP1;SLC16A3
Acarbose-2000_mg/kg_in_Water-Rat-Kidney-1d-dn	5/304	0,454452933	0,648418714	0	0	1,130403328	0,891504937	ARL2BP;ARL6IP5;SEPP1;RPL39;TPT1
Carbamazepine-490_mg/kg_in_CMC-Rat-Liver-5d-dn	5/304	0,454452933	0,648301269	0	0	1,130403328	0,891504937	EEF1B2;C1QA;RPSA;RPL10A;SEPP1
Choline_Chloride-2550_mg/kg_in_Water-Rat-Liver-3d-dn	5/304	0,454452933	0,648183865	0	0	1,130403328	0,891504937	C1QA;RPS19;RPL10A;SEPP1;RPL39
Epalrestat-2500_mg/kg_in_CMC-Rat-Liver-3d-dn	5/304	0,454452933	0,648066504	0	0	1,130403328	0,891504937	C1QA;RPS17;CLP1;SEPP1;TPT1
Guanethidine-132_mg/kg_in_CMC-Rat-Heart-1d-up	5/304	0,454452933	0,647949186	0	0	1,130403328	0,891504937	FUNDC1;ARL6IP5;RPS20;SEPP1;TPT1
Letrozole-250_mg/kg_in_Corn_Oil-Rat-Liver-0.25d-dn	5/304	0,454452933	0,64783191	0	0	1,130403328	0,891504937	EEF1B2;RPL35A;RPS20;RPS3A;TPT1
Neomycin-877_mg/kg_in_Corn_Oil-Rat-Liver-0.25d-up	5/304	0,454452933	0,647714677	0	0	1,130403328	0,891504937	C1QA;RPS19;RPS6;RPS3A;GOLGA7
Oxcarbazepine-1600_mg/kg_in_CMC-Rat-Kidney-1d-up	5/304	0,454452933	0,647597486	0	0	1,130403328	0,891504937	RPS17;CLP1;RPS8;RPS3A;TPT1
Perhexiline-320_mg/kg_in_CMC-Rat-Liver-3d-up	5/304	0,454452933	0,647480337	0	0	1,130403328	0,891504937	EEF1B2;RPS8;RPS3A;RPL10A;SEPP1
Pioglitazone-1500_mg/kg_in_Corn_Oil-Rat-Liver-5d-dn	5/304	0,454452933	0,647363231	0	0	1,130403328	0,891504937	RPS17;RPS8;RPL35A;RPSA;RPS3A
Raloxifene-5_mg/kg_in_Corn_Oil-Rat-Liver-0.25d-up	5/304	0,454452933	0,647246167	0	0	1,130403328	0,891504937	EEF1B2;RPL32;RPS19;RPS6;RPSA
Sulfadiazine-1170_mg/kg_in_Water-Rat-Liver-3d-up	5/304	0,454452933	0,647129146	0	0	1,130403328	0,891504937	FUNDC1;RPS6;RPSA;RPL10A;SEPP1
Zomepirac-11_mg/kg_in_Saline-Rat-Liver-5d-dn	5/304	0,454452933	0,647012166	0	0	1,130403328	0,891504937	C1QA;CLP1;RPL10A;SEPP1;PDRG1
Catechol-40_mg/kg_in_Saline-Rat-Liver-1d-up	6/371	0,45465613	0,647184471	0	0	1,111512491	0,8761096	C1QA;RPS17;RPL32;RPS20;RPL10A;TPT1
Cyclosporin_A-70_mg/kg_in_Corn_Oil-Rat-Liver-3d-dn	4/238	0,456660573	0,649920252	0	0	1,155101216	0,905385535	RPS17;CLP1;RPS3A;SEPP1
Benoxaprofen-20_mg/kg_in_CMC-Rat-Liver-5d-up	5/305	0,457277049	0,650680043	0	0	1,126697087	0,88160198	RPS8;RPSA;RPS3A;GOLGA7;TPT1
Beta-Naphthoflavone-80_mg/kg_in_CMC-Rat-Liver-5d-dn	5/305	0,457277049	0,650562507	0	0	1,126697087	0,88160198	C1QA;RPL32;RPS6;RPL39;TPT1
Carboplatin-6_mg/kg_in_Saline-Rat-Bone_marrow-1d-dn	5/305	0,457277049	0,650445014	0	0	1,126697087	0,88160198	EEF1B2;RPS6;RPS3A;RPL10A;RPL39
Cobalt(II)_Acetylacetonate-100_mg/kg_in_Corn_Oil-Rat-Heart-3d-up	5/305	0,457277049	0,650327562	0	0	1,126697087	0,88160198	CLP1;ARL6IP5;RPS20;SEPP1;TPT1
Gentamicin-267_mg/kg_in_Corn_Oil-Rat-Liver-1d-dn	5/305	0,457277049	0,650210153	0	0	1,126697087	0,88160198	RPS17;RPL32;CLP1;RPS8;RPL39
Lansoprazole-600_mg/kg_in_CMC-Rat-Liver-5d-up	5/305	0,457277049	0,650092787	0	0	1,126697087	0,88160198	RPS17;RPS8;RPS5;RPL35A;RPSA
Lipopolysaccharide_E._Coli_O55:B5-1.25_mg/kg_in_Saline-Rat-Kidney-1d-dn	5/305	0,457277049	0,649975463	0	0	1,126697087	0,88160198	EEF1B2;RPS17;RPL32;RPL35A;RPS3A
Mifepristone-300_mg/kg_in_Corn_Oil-Rat-Liver-0.25d-up	5/305	0,457277049	0,649858181	0	0	1,126697087	0,88160198	EEF1B2;RPS6;RPS3A;RPL10A;TPT1
N-Nitrosodiethylamine-1.67_mg/kg_in_Saline-Rat-Bone_marrow-1d-dn	5/305	0,457277049	0,649740942	0	0	1,126697087	0,88160198	CLP1;FUNDC1;RPS20;RPL10A;RPL39
Risperidone-22_mg/kg_in_CMC-Rat-Heart-5d-dn	5/305	0,457277049	0,649623745	0	0	1,126697087	0,88160198	RPS19;FEM1B;COL5A3;RPS6;SEPP1
Safrole-488_mg/kg_in_Corn_Oil-Rat-Liver-5d-dn	5/305	0,457277049	0,64950659	0	0	1,126697087	0,88160198	EEF1B2;C1QA;RPSA;RPL10A;SEPP1
Sulfadoxine-380_mg/kg_in_Corn_Oil-Rat-Liver-1d-dn	5/305	0,457277049	0,649389477	0	0	1,126697087	0,88160198	RPS8;RPSA;RPS3A;SEPP1;TPT1
Diphenidol-300_mg/kg_in_Water-Rat-Heart-3d-dn	4/239	0,45985546	0,652933406	0	0	1,150268156	0,893577831	C1QA;RPL10A;RPL39;TPT1
2378-Tetrachlorodibenzo-P-Dioxin-0.02_mg/kg_in_CMC-Rat-Liver-3d-up	5/306	0,46009714	0,653158809	0	0	1,123015071	0,871816408	C1QA;RPS17;RPS3A;RPL10A;SEPP1
4-Chloro-2-Nitroaniline-1200_mg/kg_in_CMC-Rat-Kidney-1d-up	5/306	0,46009714	0,653041101	0	0	1,123015071	0,871816408	RPS17;RPS20;RPS3A;SEPP1;TPT1
Atorvastatin-300_mg/kg_in_Corn_Oil-Rat-Kidney-1d-up	5/306	0,46009714	0,652923436	0	0	1,123015071	0,871816408	RPS5;RPS20;RPSA;RAPGEF5;SEPP1
Benzethonium_Chloride-138_mg/kg_in_Water-Rat-Heart-3d-up	5/306	0,46009714	0,652805814	0	0	1,123015071	0,871816408	RPS19;ARL2BP;RPS8;RPS6;GOLGA7
Camptothecin-0.24_mg/kg_in_Saline-Rat-Bone_marrow-1d-dn	5/306	0,46009714	0,652688233	0	0	1,123015071	0,871816408	EEF1B2;CLP1;RPS8;FUNDC1;RPS3A
Carboplatin-14_mg/kg_in_Saline-Rat-Spleen-1d-up	5/306	0,46009714	0,652570695	0	0	1,123015071	0,871816408	RPS20;RPL10A;SEPP1;RPL39;TPT1
Cholic_Acid-1402_mg/kg_in_Water-Rat-Liver-1d-dn	5/306	0,46009714	0,6524532	0	0	1,123015071	0,871816408	C1QA;RPS19;RPL10A;SEPP1;TPT1
Cisplatin-2_mg/kg_in_Saline-Rat-Liver-5d-up	5/306	0,46009714	0,652335746	0	0	1,123015071	0,871816408	RPS19;RPS6;RPS20;RPL39;GOLGA7
Hydroquinone-800_mg/kg_in_CMC-Rat-Kidney-3d-dn	5/306	0,46009714	0,652218335	0	0	1,123015071	0,871816408	CLP1;RPS6;RPS20;RPSA;RPL39
Hydroxyurea-400_mg/kg_in_Saline-Rat-Liver-5d-up	5/306	0,46009714	0,652100967	0	0	1,123015071	0,871816408	RPS19;RPS6;SEPP1;RPL39;GOLGA7
Prednisolone-184_mg/kg_in_Water-Rat-Liver-5d-up	5/306	0,46009714	0,65198364	0	0	1,123015071	0,871816408	CXCL11;CXADR;RPS20;RPSA;SEPP1
Procarbazine-54_mg/kg_in_Water-Rat-Liver-5d-dn	5/306	0,46009714	0,651866356	0	0	1,123015071	0,871816408	C1QA;RPL32;RPS20;RPSA;RPL10A
Tazobactam-1500_mg/kg_in_CMC-Rat-Spleen-3d-dn	5/306	0,46009714	0,651749114	0	0	1,123015071	0,871816408	C1QA;ARL6IP5;RPS20;RPS3A;PDRG1
Thalidomide-113_mg/kg_in_CMC-Rat-Liver-3d-up	5/306	0,46009714	0,651631914	0	0	1,123015071	0,871816408	RPS17;RPL32;RPL35A;RPSA;RPL39
Warfarin-0.25_mg/kg_in_CMC-Rat-Liver-5d-dn	5/306	0,46009714	0,651514756	0	0	1,123015071	0,871816408	C1QA;RPS8;RPS6;RPS3A;TPT1
Zomepirac-11_mg/kg_in_Saline-Rat-Liver-3d-dn	5/306	0,46009714	0,65139764	0	0	1,123015071	0,871816408	RPS8;RPS6;RPL10A;RPL39;TPT1
Acetaminophen-486_mg/kg_in_Corn_Oil-Rat-Kidney-1d-up	5/307	0,462913086	0,655266618	0	0	1,119357041	0,862146661	RPS5;RPS20;RAPGEF5;SEPP1;RPL39
Aldosterone-0.065_mg/kg_in_Saline-Rat-Kidney-5d-up	5/307	0,462913086	0,65514887	0	0	1,119357041	0,862146661	RPS19;CLP1;RPS8;FUNDC1;ARL6IP5
Amoxapine-313_mg/kg_in_CMC-Rat-Heart-1d-up	5/307	0,462913086	0,655031165	0	0	1,119357041	0,862146661	EEF1B2;FUNDC1;RPS20;RPL10A;SEPP1
Cerivastatin-7_mg/kg_in_Corn_Oil-Rat-Liver-5d-up	5/307	0,462913086	0,654913502	0	0	1,119357041	0,862146661	RPS19;ARL2BP;RPS6;RPS20;RPL10A
Chloroform-600_mg/kg_in_Water-Rat-Liver-3d-up	5/307	0,462913086	0,654795881	0	0	1,119357041	0,862146661	RPS19;RPS6;RPSA;RPL10A;SEPP1
Clofibrate-500_mg/kg_in_Corn_Oil-Rat-Liver-3d-dn	5/307	0,462913086	0,654678302	0	0	1,119357041	0,862146661	EEF1B2;CLP1;RPSA;RPL10A;SEPP1
Furan-24_mg/kg_in_Corn_Oil-Rat-Kidney-5d-dn	5/307	0,462913086	0,654560766	0	0	1,119357041	0,862146661	C1QA;RPS17;RPL32;RPS3A;TPT1
Ifosfamide-143_mg/kg_in_Saline-Rat-Bone_marrow-1d-up	5/307	0,462913086	0,654443271	0	0	1,119357041	0,862146661	C1QA;NKG7;SEPP1;SLC16A3;TPT1
Lomustine-8.75_mg/kg_in_Corn_Oil-Rat-Bone_marrow-5d-up	5/307	0,462913086	0,654325819	0	0	1,119357041	0,862146661	C1QA;NKG7;RPL10A;SEPP1;TPT1
Methotrexate-27_mg/kg_in_Corn_Oil-Rat-Liver-0.25d-dn	5/307	0,462913086	0,654208409	0	0	1,119357041	0,862146661	RPS17;RPL32;RPS8;RPS20;RPSA
Sildenafil-2.5_mg/kg_in_Water-Rat-Liver-1d-dn	5/307	0,462913086	0,654091041	0	0	1,119357041	0,862146661	RPL32;RPS8;RPS6;SEPP1;TPT1
Procaine-2500_mg/kg_in_Water-Rat-Heart-3d-dn	4/240	0,463043968	0,654158618	0	0	1,145475372	0,881939594	EEF1B2;RPS3A;RPL39;TPT1
Azithromycin-225_mg/kg_in_Water-Rat-Liver-0.25d-up	6/375	0,464864413	0,656612646	0	0	1,099656357	0,842347218	EEF1B2;COL5A3;RPS6;RPL10A;SEPP1;RPL39
4-Chloroaniline-75_mg/kg_in_CMC-Rat-Kidney-1d-up	5/308	0,465724771	0,657709932	0	0	1,115722765	0,8525912	RPS17;RPS8;ARL6IP5;RPSA;RPS3A

Ethanol-3000_mg/kg_in_Saline-Rat-Bone_marrow-1d-up	5/308	0,465724771	0,657592021	0	0	1,115722765	0,8525912	RPS19;ARL2BP;RPS8;RPS20;GOLGA7
Ethisterone-1500_mg/kg_in_CMC-Rat-Liver-5d-up	5/308	0,465724771	0,657474152	0	0	1,115722765	0,8525912	C1QA;RPS17;ARL6IP5;RPSA;RPL39
Furosemide-375_mg/kg_in_CMC-Rat-Kidney-3d-dn	5/308	0,465724771	0,657356325	0	0	1,115722765	0,8525912	CLP1;RPS6;RPL10A;SEPP1;GOLGA7
Gentamicin-267_mg/kg_in_Corn_Oil-Rat-Liver-3d-up	5/308	0,465724771	0,65723854	0	0	1,115722765	0,8525912	C1QA;ARL2BP;RAPGEF5;SEPP1;GOLGA7
Idebenone-2500_mg/kg_in_CMC-Rat-Liver-5d-dn	5/308	0,465724771	0,657120798	0	0	1,115722765	0,8525912	EEF1B2;RPL32;RPS19;RPS3A;SEPP1
Lomefloxacin-2000_mg/kg_in_CMC-Rat-Liver-1d-dn	5/308	0,465724771	0,657003097	0	0	1,115722765	0,8525912	C1QA;RPL32;RPS19;RPS6;RPS3A
Mitomycin_C-0.5_mg/kg_in_Saline-Rat-Spleen-1d-up	5/308	0,465724771	0,656885439	0	0	1,115722765	0,8525912	RPS19;RPS6;RPL10A;SEPP1;RPL39
Nimesulide-162_mg/kg_in_Corn_Oil-Rat-Kidney-5d-dn	5/308	0,465724771	0,656767823	0	0	1,115722765	0,8525912	EEF1B2;RPS17;RPL32;SEPP1;TPT1
Nisoldipine-15_mg/kg_in_Water-Rat-Liver-1d-dn	5/308	0,465724771	0,656650249	0	0	1,115722765	0,8525912	EEF1B2;RPL32;RPS6;RPL10A;SEPP1
Perhexiline-12_uM_in_DMSO-Rat-Primary_rat_hepatocytes-0.67d-up	5/308	0,465724771	0,656532718	0	0	1,115722765	0,8525912	EEF1B2;RPS6;RPS20;RPL39;TPT1
Sildenafil-420_mg/kg_in_Water-Rat-Liver-5d-up	5/308	0,465724771	0,656415228	0	0	1,115722765	0,8525912	EEF1B2;RPS8;RPS20;RPS3A;TPT1
Sulfadoxine-380_mg/kg_in_Corn_Oil-Rat-Liver-5d-up	5/308	0,465724771	0,65629778	0	0	1,115722765	0,8525912	RPS17;RPS5;RPL35A;RPSA;RPL39
Sulpiride-1000_mg/kg_in_CMC-Rat-Heart-5d-dn	5/308	0,465724771	0,656180374	0	0	1,115722765	0,8525912	RPS19;RPS8;RPS20;SEPP1;TOMM20
Thalidomide-113_mg/kg_in_CMC-Rat-Liver-5d-dn	5/308	0,465724771	0,656063011	0	0	1,115722765	0,8525912	EEF1B2;RPS20;RPS3A;SEPP1;RPL39
Doxycycline-1000_mg/kg_in_Water-Rat-Liver-3d-dn	4/241	0,466225941	0,656651557	0	0	1,140722362	0,870468022	RPS20;RPL39;SEPP1;TPT1
Acarbose-2000_mg/kg_in_Water-Rat-Kidney-3d-up	5/309	0,468532078	0,659781628	0	0	1,112112012	0,843148512	CXCL11;RPS20;RAPGEF5;SEPP1;RPL39
Daunorubicin-3.25_mg/kg_in_Saline-Rat-Liver-3d-up	5/309	0,468532078	0,659663683	0	0	1,112112012	0,843148512	C1QA;CLP1;RPS8;SEPP1;TPT1
Fluvastatin-94_mg/kg_in_Corn_Oil-Rat-Kidney-1d-up	5/309	0,468532078	0,659545781	0	0	1,112112012	0,843148512	RPS5;RPS20;RAPGEF5;SEPP1;TPT1
Itraconazole-1093_mg/kg_in_Corn_Oil-Rat-Liver-3d-dn	5/309	0,468532078	0,659427921	0	0	1,112112012	0,843148512	C1QA;RPS17;RPL32;RPS6;TPT1
Methotrexate-0.3_mg/kg_in_Corn_Oil-Rat-Kidney-3d-dn	5/309	0,468532078	0,659310103	0	0	1,112112012	0,843148512	RPL32;RPS8;RPS6;RPL39;TPT1
Mitotane-5000_mg/kg_in_Water-Rat-Bone_marrow-3d-up	5/309	0,468532078	0,659192327	0	0	1,112112012	0,843148512	RPS19;NKG7;RPL10A;SEPP1;RPL39
Tamoxifen-32_mg/kg_in_Corn_Oil-Rat-Liver-5d-dn	5/309	0,468532078	0,659074593	0	0	1,112112012	0,843148512	C1QA;RPS20;SEPP1;RPL39;TPT1
Thioguanine-12_mg/kg_in_Corn_Oil-Rat-Bone_marrow-1d-up	5/309	0,468532078	0,658956901	0	0	1,112112012	0,843148512	C1QA;RPS17;NKG7;RPS20;SEPP1
Cetylpyridinium_Bromide-16.5_mg/kg_in_Water-Rat-Liver-0.25d-up	6/377	0,469950498	0,660833802	0	0	1,093822637	0,825976004	C1QA;RPS19;RPS6;RPSA;RPL10A;SEPP1
2378-Tetrachlorodibenzo-P-Dioxin-0.0007_mg/kg_in_CMC-Rat-Liver-5d-up	5/310	0,471334893	0,662662195	0	0	1,108524554	0,833817107	RPS17;RPS19;RPSA;RPL10A;SEPP1
Auranofin-33_mg/kg_in_CMC-Rat-Kidney-3d-dn	5/310	0,471334893	0,662543926	0	0	1,108524554	0,833817107	EEF1B2;RPS19;CLP1;RPS6;RPL39
Clofibrate-130_mg/kg_in_Corn_Oil-Rat-Liver-3d-up	5/310	0,471334893	0,662425699	0	0	1,108524554	0,833817107	RPS17;RPL32;RPS20;RPL39;TPT1
Diflunisal-207_mg/kg_in_Corn_Oil-Rat-Kidney-5d-dn	5/310	0,471334893	0,662307514	0	0	1,108524554	0,833817107	ARL2BP;CLP1;RPS8;SEPP1;TPT1
Diisopropyl_Fluorophosphate-3.75_mg/kg_in_Water-Rat-Heart-3d-up	5/310	0,471334893	0,662189372	0	0	1,108524554	0,833817107	RPS17;ARL2BP;FUNDC1;RPS3A;GOLGA7
Enrofloxacin-2000_mg/kg_in_CMC-Rat-Kidney-3d-dn	5/310	0,471334893	0,662071271	0	0	1,108524554	0,833817107	RPL32;RPS19;RPSA;RPL10A;SEPP1
Famciclovir-1200_mg/kg_in_Saline-Rat-Bone_marrow-3d-up	5/310	0,471334893	0,661953213	0	0	1,108524554	0,833817107	RPS17;RPS8;FEM1B;RPS5;RPS6
Finasteride-800_mg/kg_in_Corn_Oil-Rat-Liver-5d-up	5/310	0,471334893	0,661835197	0	0	1,108524554	0,833817107	EEF1B2;RPS17;RPL32;RPSA;TPT1
Ketoconazole-114_mg/kg_in_Corn_Oil-Rat-Liver-0.25d-dn	5/310	0,471334893	0,661717223	0	0	1,108524554	0,833817107	C1QA;RPS17;RPL32;RPS3A;RPL39
Lamotrigine-26_mg/kg_in_CMC-Rat-Heart-5d-dn	5/310	0,471334893	0,66159929	0	0	1,108524554	0,833817107	RPS19;RPS3A;SEPP1;RPL39;GOLGA7
Lead_(II)_Acetate-2_mg/kg_in_Saline-Rat-Kidney-5d-up	5/310	0,471334893	0,6614814	0	0	1,108524554	0,833817107	CXCL11;CLP1;RPS6;SEPP1;TPT1
Leflunomide-30_mg/kg_in_Corn_Oil-Rat-Spleen-1d-up	5/310	0,471334893	0,661363552	0	0	1,108524554	0,833817107	CLP1;ARL6IP5;RPSA;SEPP1;TPT1
Saquinavir-1500_mg/kg_in_CMC-Rat-Kidney-5d-dn	5/310	0,471334893	0,661245746	0	0	1,108524554	0,833817107	ARL2BP;RPSA;RPL10A;SEPP1;TPT1
Torsemide-3_mg/kg_in_Corn_Oil-Rat-Liver-5d-dn	5/310	0,471334893	0,661127982	0	0	1,108524554	0,833817107	C1QA;RPS6;RPS3A;RPL10A;SEPP1
Nimodipine-10_mg/kg_in_Corn_Oil-Rat-Heart-1d-dn	6/378	0,472488713	0,662628402	0	0	1,090928926	0,817914602	ARL2BP;RPS8;FUNDC1;ARL6IP5;RPSA;TOMM20
Dexchlorpheniramine-47_mg/kg_in_Water-Rat-Heart-3d-dn	4/243	0,472569656	0,66262393	0	0	1,131333701	0,84801394	RPS17;RPL32;RPS3A;TPT1
Pravastatin-1200_mg/kg_in_Corn_Oil-Rat-Liver-1d-up	4/243	0,472569656	0,662505983	0	0	1,131333701	0,84801394	C1QA;RPS19;GOLGA7;SEPP1
Dexfenfluramine-29_mg/kg_in_Water-Rat-Heart-3d-up	5/311	0,474133103	0,664579519	0	0	1,104960166	0,824595517	RPS17;ARL6IP5;RPS3A;SEPP1;GOLGA7
Doxorubicin-0.65_mg/kg_in_Saline-Rat-Spleen-0.25d-dn	5/311	0,474133103	0,664461267	0	0	1,104960166	0,824595517	CXCL11;FUNDC1;RPS6;RPS20;RPL39
Epalrestat-2500_mg/kg_in_CMC-Rat-Liver-1d-dn	5/311	0,474133103	0,664343056	0	0	1,104960166	0,824595517	EEF1B2;C1QA;RPL32;RPS20;SEPP1
Fenofibrate-215_mg/kg_in_Corn_Oil-Rat-Liver-0.25d-up	5/311	0,474133103	0,664224888	0	0	1,104960166	0,824595517	RPL32;RPS19;RPS6;RPL10A;SEPP1
Gliclazide-1500_mg/kg_in_CMC-Rat-Kidney-1d-up	5/311	0,474133103	0,664106761	0	0	1,104960166	0,824595517	RPS17;RPL32;RPS20;TOMM20;TPT1
Ivermectin-7.5_mg/kg_in_CMC-Rat-Liver-5d-up	5/311	0,474133103	0,663988677	0	0	1,104960166	0,824595517	RPS17;RPS20;RPSA;SEPP1;RPL39
Lead_(II)_Acetate-200_mg/kg_in_Saline-Rat-Kidney-3d-dn	5/311	0,474133103	0,663870635	0	0	1,104960166	0,824595517	EEF1B2;RPS19;RPS6;RPL10A;SEPP1
Metoprolol-1200_mg/kg_in_Water-Rat-Heart-1d-dn	5/311	0,474133103	0,663752634	0	0	1,104960166	0,824595517	EEF1B2;ARL2BP;RPS20;RPL39;TPT1
Nevirapine-200_mg/kg_in_Saline-Rat-Heart-3d-up	5/311	0,474133103	0,663634676	0	0	1,104960166	0,824595517	RPSA;RAPGEF5;RPL10A;RPL39;TPT1
Oxaliplatin-8_mg/kg_in_Saline-Rat-Kidney-5d-dn	5/311	0,474133103	0,663516759	0	0	1,104960166	0,824595517	RPS8;RPSA;RPS3A;RPL10A;SEPP1
Procarbazine-27_mg/kg_in_Water-Rat-Liver-5d-dn	5/311	0,474133103	0,663398884	0	0	1,104960166	0,824595517	EEF1B2;C1QA;RPL32;RPL10A;TPT1
Methotrexate-0.3_mg/kg_in_Corn_Oil-Rat-Kidney-1d-up	6/379	0,475023591	0,664526786	0	0	1,088050486	0,809934782	EEF1B2;RPS17;RPS5;RPS20;SEPP1;RPL39
Valproic_Acid-850_mg/kg_in_Water-Rat-Kidney-3d-dn	4/244	0,475731095	0,66539835	0	0	1,126697087	0,837026094	ARL2BP;RPS8;RPS6;RPL10A
Candesartan-1300_mg/kg_in_CMC-Rat-Kidney-3d-dn	5/312	0,476926596	0,666952036	0	0	1,101418627	0,815482297	RPS19;ARL2BP;RPS8;RPS6;ARL6IP5
Clofibric_Acid-448_mg/kg_in_Corn_Oil-Rat-Heart-5d-dn	5/312	0,476926596	0,666833635	0	0	1,101418627	0,815482297	RPS17;RPS6;RPS3A;RPL39;TPT1
Itraconazole-30_mg/kg_in_Corn_Oil-Rat-Liver-3d-dn	5/312	0,476926596	0,666715276	0	0	1,101418627	0,815482297	C1QA;RPS17;RPL32;RPS3A;TPT1
Lead_(II)_Acetate-600_mg/kg_in_Saline-Rat-Liver-5d-up	5/312	0,476926596	0,66659696	0	0	1,101418627	0,815482297	RPS8;RPS5;RPS6;RPS3A;TPT1
Nortriptyline-70_uM_in_DMSO-Rat-Primary_rat_hepatocytes-0.67d-up	5/312	0,476926596	0,666478685	0	0	1,101418627	0,815482297	EEF1B2;CXCL11;ARL6IP5;SEPP1;GOLGA7
Oxcarbazepine-1600_mg/kg_in_CMC-Rat-Heart-3d-up	5/312	0,476926596	0,666360452	0	0	1,101418627	0,815482297	EEF1B2;CLP1;RPS3A;RAPGEF5;TPT1
Bromfenac-5_mg/kg_in_CMC-Rat-Liver-5d-up	5/313	0,47971526	0,670137884	0	0	1,097899718	0,806476026	EEF1B2;RPS20;RPS3A;RPL10A;RPL39
Calcitriol-0.04_mg/kg_in_CMC-Rat-Kidney-1d-dn	5/313	0,47971526	0,670019044	0	0	1,097899718	0,806476026	ARL2BP;RPS8;RPSA;SEPP1;TPT1
Coumarin-111_mg/kg_in_Corn_Oil-Rat-Bone_marrow-1d-up	5/313	0,47971526	0,669900247	0	0	1,097899718	0,806476026	C1QA;RPL10A;SEPP1;RPL39;TPT1
Ifosfamide-17_mg/kg_in_Saline-Rat-Liver-5d-dn	5/313	0,47971526	0,669781491	0	0	1,097899718	0,806476026	RPS19;RPSA;RPL10A;SEPP1;TPT1
Itraconazole-10_uM_in_DMSO-Rat-Primary_rat_hepatocytes-0.67d-up	5/313	0,47971526	0,669662777	0	0	1,097899718	0,806476026	EEF1B2;ARL6IP5;RPSA;SEPP1;GOLGA7
Leucovorin-1500_mg/kg_in_CMC-Rat-Bone_marrow-5d-dn	5/313	0,47971526	0,669544106	0	0	1,097899718	0,806476026	RPS19;NKG7;RPL13A;RPL39;TPT1
Methotrexate-27_mg/kg_in_Corn_Oil-Rat-Spleen-3d-dn	5/313	0,47971526	0,669425477	0	0	1,097899718	0,806476026	C1QA;RPS19;NKG7;RPL39;TPT1
Nitrobenzene-225_mg/kg_in_CMC-Rat-Kidney-3d-dn	5/313	0,47971526	0,669306889	0	0	1,097899718	0,806476026	EEF1B2;ARL2BP;RPS8;RPS6;RPL39
Phenothiazine-386_mg/kg_in_Corn_Oil-Rat-Liver-1d-dn	5/313	0,47971526	0,669188344	0	0	1,097899718	0,806476026	C1QA;RPS17;RPS3A;SEPP1;TPT1
Pyrazinamide-1500_mg/kg_in_CMC-Rat-Liver-5d-dn	5/313	0,47971526	0,669069841	0	0	1,097899718	0,806476026	C1QA;RPSA;RPL10A;SEPP1;RPL39
Valproic_Acid-1340_mg/kg_in_Water-Rat-Liver-1d-dn	5/313	0,47971526	0,668951379	0	0	1,097899718	0,806476026	RPS19;RPS6;RPS20;RPL10A;SEPP1
Chlorambucil-4.5_mg/kg_in_Corn_Oil-Rat-Liver-3d-up	4/246	0,482032393	0,672063574	0	0	1,117536949	0,815515839	CLP1;RPS8;RPS20;SEPP1
Meloxicam-0.6_mg/kg_in_Corn_Oil-Rat-Kidney-0.25d-dn	4/246	0,482032393	0,671944625	0	0	1,117536949	0,815515839	C1QA;RPS17;RPSA;RPS3A
Phenothiazine-36_mg/kg_in_Corn_Oil-Rat-Liver-3d-dn	4/246	0,482032393	0,671825718	0	0	1,117536949	0,815515839	RPS17;CLP1;RPS3A;SEPP1
(+)-Pulegone-200_mg/kg_in_CMC-Rat-Kidney-5d-dn	5/314	0,482498988	0,672357047	0	0	1,094403222	0,797575301	RPS19;ARL2BP;RPS6;SEPP1;TPT1
3-Methylcholanthrene-12.5_mg/kg_in_CMC-Rat-Liver-5d-dn	5/314	0,482498988	0,672238109	0	0	1,094403222	0,797575301	RPS17;RPL32;RPS19;RPS8;TPT1
Acetaminophen-1950_uM_in_DMSO-Rat-Primary_rat_hepatocytes-0.67d-dn	5/314	0,482498988	0,672119213	0	0	1,094403222	0,797575301	EEF1B2;RPS17;SEPP1;RPL39;SLC16A3
Azithromycin-225_mg/kg_in_Water-Rat-Liver-1d-up	5/314	0,482498988	0,672000359	0	0	1,094403222	0,797575301	EEF1B2;C1QA;RPS17;RPS3A;RPL39
Bis(2-Ethylhexyl)Phthalate-1000_mg/kg_in_Corn_Oil-Rat-Liver-5d-up	5/314	0,482498988	0,671881547	0	0	1,094403222	0,797575301	C1QA;RPL32;RPS19;RPS5;TPT1
Diazepam-710_mg/kg_in_CMC-Rat-Kidney-3d-up	5/314	0,482498988	0,671762777	0	0	1,094403222	0,797575301	EEF1B2;RPS17;RPSA;RPS3A;SEPP1
Indomethacin-9.6_mg/kg_in_Water-Rat-Liver-1d-up	5/314	0,482498988	0,671644049	0	0	1,094403222	0,797575301	C1QA;RPS19;RPS6;RPL39;GOLGA7
Lead(IV)_Acetate-600_mg/kg_in_Saline-Rat-Liver-0.25d-dn	5/314	0,482498988	0,671525363	0	0	1,094403222	0,797575301	RPS19;RPS8;RPS20;RPL10A;TPT1
NN-Dimethylformamide-700_mg/kg_in_Saline-Rat-Liver-0.25d-dn	5/314	0,482498988	0,671406719	0	0	1,094403222	0,797575301	EEF1B2;RPS17;CLP1;RPS20;RPL39
Phenytoin-572_mg/kg_in_Water-Rat-Heart-5d-dn	5/314	0,482498988	0,671288117	0	0	1,094403222	0,797575301	ARL2BP;RPS8;FUNDC1;RPS20;RPSA
Rolipram-75_mg/kg_in_CMC-Rat-Liver-5d-dn	5/314	0,482498988	0,671169557	0	0	1,094403222	0,797575301	RPL32;RPS19;RPS6;RPS20;SEPP1
Venlafaxine-320_mg/kg_in_Water-Rat-Kidney-5d-up	5/314	0,482498988	0,671051038	0	0	1,094403222	0,797575301	EEF1B2;ARL2BP;CLP1;HOXA1;SEPP1
Etoposide-188_mg/kg_in_Corn_Oil-Rat-Liver-0.25d-up	6/383	0,485128015	0,67458832	0	0	1,076687034	0,778813463	EEF1B2;RPS8;RPS6;RPSA;RPL10A;SEPP1
Prednisolone-184_mg/kg_in_Water-Rat-Kidney-1d-up	6/383	0,485128015	0,67446924	0	0	1,076687034	0,778813463	EEF1B2;ARL2BP;RPS8;RPS6;RPL10A;TPT1
Ebastine-2_mg/kg_in_Water-Rat-Heart-3d-dn	4/247	0,485171963	0,674411292	0	0	1,113012507	0,804988398	EEF1B2;R3HCC1;RPS3A;RPL39
Emetine-1_mg/kg_in_Saline-Rat-Kidney-1d-up	4/247	0,485171963	0,674292285	0	0	1,113012507	0,804988398	RPS19;ARL2BP;RPS8;RPSA

Podophyllotoxin-4_mg/kg_in_Corn_Oil-Rat-Bone_marrow-5d-dn	4/247	0,485171963	0,67417332	0	0	1,113012507	0,804988398	RPS8;FUNDC1;RPS3A;TPT1
4-Nitrotoluene-750_mg/kg_in_CMC-Rat-Kidney-1d-dn	5/315	0,485277671	0,674201258	0	0	1,090928926	0,788778744	RPL32;RPS6;RPS20;RPSA;RPL39
Balsalazide-1100_mg/kg_in_Water-Rat-Liver-3d-up	5/315	0,485277671	0,674082351	0	0	1,090928926	0,788778744	EEF1B2;RPS19;RPS6;RPSA;RPL10A
Carvedilol-20_mg/kg_in_Corn_Oil-Rat-Heart-0.25d-dn	5/315	0,485277671	0,673963487	0	0	1,090928926	0,788778744	EEF1B2;RPS19;ARL2BP;FUNDC1;RPS6
Enoxacin-100_mg/kg_in_Corn_Oil-Rat-Liver-1d-dn	5/315	0,485277671	0,673844664	0	0	1,090928926	0,788778744	EEF1B2;RPL32;RPL10A;SEPP1;TPT1
Oxcarbazepine-1600_mg/kg_in_CMC-Rat-Heart-1d-up	5/315	0,485277671	0,673725883	0	0	1,090928926	0,788778744	EEF1B2;CLP1;RAPGEF5;SEPP1;TPT1
Ranitidine-1500_mg/kg_in_CMC-Rat-Heart-1d-up	5/315	0,485277671	0,673607144	0	0	1,090928926	0,788778744	CXCL11;RPS6;ARL6IP5;RAPGEF5;SEPP1
Roxithromycin-312_mg/kg_in_CMC-Rat-Kidney-1d-dn	5/315	0,485277671	0,673488446	0	0	1,090928926	0,788778744	RPL32;ARL6IP5;RPS20;RPL10A;SEPP1
1-Naphthyl_Isothiocyanate-15_mg/kg_in_Corn_Oil-Rat-Liver-1d-up	5/316	0,488051202	0,677218334	0	0	1,087476619	0,780084994	EEF1B2;C1QA;RPSA;RPS3A;SEPP1
Azathioprine-20_mg/kg_in_Corn_Oil-Rat-Liver-3d-up	5/316	0,488051202	0,677099042	0	0	1,087476619	0,780084994	EEF1B2;RPL32;RPS8;SEPP1;RPL39
Bromisovalum-250_mg/kg_in_Corn_Oil-Rat-Liver-1d-dn	5/316	0,488051202	0,676979793	0	0	1,087476619	0,780084994	EEF1B2;RPS6;RPL35A;RPS20;TPT1
L-Phenylephrine-0.44_mg/kg_in_Saline-Rat-Heart-5d-up	5/316	0,488051202	0,676860585	0	0	1,087476619	0,780084994	ARL2BP;CLP1;FUNDC1;ARL6IP5;RPS20
Oxymetazoline-0.5_mg/kg_in_Water-Rat-Liver-3d-dn	5/316	0,488051202	0,67674142	0	0	1,087476619	0,780084994	EEF1B2;RPS17;CLP1;RPS20;RPSA
Oxymetholone-1170_mg/kg_in_CMC-Rat-Liver-3d-up	5/316	0,488051202	0,676622296	0	0	1,087476619	0,780084994	C1QA;RPS17;RPS6;RPSA;RPS3A
Torsemide-110_mg/kg_in_Corn_Oil-Rat-Liver-1d-up	5/316	0,488051202	0,676503214	0	0	1,087476619	0,780084994	CXCL11;CLP1;RPS8;SEPP1;TPT1
Venlafaxine-1481_uM_in_DMSO-Rat-Primary_rat_hepatocytes-1d-dn	5/316	0,488051202	0,676384174	0	0	1,087476619	0,780084994	RPS8;ARL6IP5;RPS20;RPSA;SEPP1
Chlorpromazine-73_mg/kg_in_Water-Rat-Liver-0.25d-up	5/317	0,490819476	0,680101019	0	0	1,084046094	0,771492716	EEF1B2;RPS17;RPL32;RPS20;RPS3A
Clofibric_Acid-448_mg/kg_in_Corn_Oil-Rat-Liver-3d-up	5/317	0,490819476	0,679981388	0	0	1,084046094	0,771492716	RPS17;RPS19;RPS20;RPS3A;RPL39
Colchicine-2000_uM_in_DMSO-Rat-Primary_rat_hepatocytes-1d-dn	5/317	0,490819476	0,679861799	0	0	1,084046094	0,771492716	CXCL11;RPS19;RPS5;SEPP1;RPL39
Coumarin-111_mg/kg_in_Corn_Oil-Rat-Bone_marrow-5d-dn	5/317	0,490819476	0,679742253	0	0	1,084046094	0,771492716	EEF1B2;RPS19;NKG7;SEPP1;RPL39
Danazol-2000_mg/kg_in_CMC-Rat-Liver-3d-up	5/317	0,490819476	0,679622748	0	0	1,084046094	0,771492716	RPS19;RPS20;RPSA;RPL10A;RPL39
Ethylene_Glycol-352_mg/kg_in_Water-Rat-Liver-5d-dn	5/317	0,490819476	0,679503285	0	0	1,084046094	0,771492716	C1QA;RPS6;RPSA;RPS3A;SEPP1
Gemfibrozil-700_mg/kg_in_Corn_Oil-Rat-Kidney-1d-up	5/317	0,490819476	0,679383865	0	0	1,084046094	0,771492716	RPS19;RPS5;SEPP1;GOLGA7;TPT1
Insulin-0.56_mg/kg_in_Saline-Rat-Liver-1d-up	5/317	0,490819476	0,679264486	0	0	1,084046094	0,771492716	EEF1B2;RPS17;RPS6;RPS3A;SEPP1
Omeprazole-415_mg/kg_in_Corn_Oil-Rat-Liver-3d-up	5/317	0,490819476	0,679145149	0	0	1,084046094	0,771492716	RPS19;RPS20;RPSA;SEPP1;RPL39
Prochlorperazine-85_mg/kg_in_CMC-Rat-Heart-5d-dn	5/317	0,490819476	0,679025855	0	0	1,084046094	0,771492716	C1QA;RPS20;RPS3A;TOMM20;TPT1
Tamoxifen-32_mg/kg_in_Corn_Oil-Rat-Liver-1d-dn	5/317	0,490819476	0,678906602	0	0	1,084046094	0,771492716	RPS20;SEPP1;RPL39;GOLGA7;TPT1
Thioguanine-24_mg/kg_in_Corn_Oil-Rat-Kidney-5d-up	5/317	0,490819476	0,678787391	0	0	1,084046094	0,771492716	ARL2BP;CLP1;FUNDC1;RPS20;SEPP1
(R)-Bicalutamide-1400_mg/kg_in_CMC-Rat-Kidney-5d-dn	4/249	0,491428235	0,679509968	0	0	1,104072648	0,784376669	RPS17;ARL2BP;RPS20;RPS3A
Digitonin-75_mg/kg_in_Corn_Oil-Rat-Heart-3d-dn	4/249	0,491428235	0,679390693	0	0	1,104072648	0,784376669	ARL2BP;RPS8;FEM1B;TPT1
Loxoprofen-18_mg/kg_in_Corn_Oil-Rat-Kidney-5d-dn	4/249	0,491428235	0,67927146	0	0	1,104072648	0,784376669	EEF1B2;RPS17;CLP1;SEPP1
Melatonin-2000_mg/kg_in_Corn_Oil-Rat-Liver-0.25d-dn	4/249	0,491428235	0,679152268	0	0	1,104072648	0,784376669	EEF1B2;CLP1;RPS3A;TPT1
Niacin-2625_mg/kg_in_Water-Rat-Liver-5d-dn	4/249	0,491428235	0,679033119	0	0	1,104072648	0,784376669	EEF1B2;RPS20;RPL39;TPT1
Amitraz-75_mg/kg_in_CMC-Rat-Liver-5d-dn	5/318	0,493582389	0,681890001	0	0	1,080637144	0,763000589	C1QA;RPS17;RPL32;RPS19;RPL35A
Citalopram-40_mg/kg_in_Corn_Oil-Rat-Liver-1d-up	5/318	0,493582389	0,681770414	0	0	1,080637144	0,763000589	RPS17;RPS6;RPS20;RPL39;TPT1
Flubendazole-1200_mg/kg_in_Corn_Oil-Rat-Liver-1d-up	5/318	0,493582389	0,681650868	0	0	1,080637144	0,763000589	RPS17;RPL32;RPSA;RPL39;TPT1
Itraconazole-1093_mg/kg_in_Corn_Oil-Rat-Liver-5d-up	5/318	0,493582389	0,681531364	0	0	1,080637144	0,763000589	RPS19;RPS20;RPL10A;RPL39;GOLGA7
Lead_(II)_Acetate-600_mg/kg_in_Saline-Rat-Kidney-3d-up	5/318	0,493582389	0,681411901	0	0	1,080637144	0,763000589	CXCL11;RPS19;RPS8;RPS5;RPL10A
Lorazepam-2000_mg/kg_in_CMC-Rat-Liver-5d-up	5/318	0,493582389	0,681292481	0	0	1,080637144	0,763000589	RPS19;RPS20;RPSA;RPL10A;SEPP1
Naproxen-134_mg/kg_in_Corn_Oil-Rat-Kidney-1d-up	5/318	0,493582389	0,681173103	0	0	1,080637144	0,763000589	RPS5;RPS6;RPS20;SEPP1;TPT1
Nitrofurantoin-76_mg/kg_in_CMC-Rat-Liver-5d-up	5/318	0,493582389	0,681053766	0	0	1,080637144	0,763000589	C1QA;RPS20;RPL10A;SEPP1;RPL39
Rofecoxib-775_mg/kg_in_Corn_Oil-Rat-Liver-5d-dn	5/318	0,493582389	0,680934472	0	0	1,080637144	0,763000589	RPS17;RPS8;RPS6;RPS3A;SEPP1
Sulfaphenazole-1695_mg/kg_in_Water-Rat-Liver-1d-dn	5/318	0,493582389	0,680815219	0	0	1,080637144	0,763000589	EEF1B2;RPS19;CLP1;RPS20;SEPP1
Valsartan-1000_mg/kg_in_Corn_Oil-Rat-Kidney-3d-dn	5/318	0,493582389	0,680696007	0	0	1,080637144	0,763000589	RPS19;RPSA;RPL10A;SEPP1;RPL39
Atorvastatin-300_mg/kg_in_Corn_Oil-Rat-Liver-1d-up	4/250	0,494544662	0,681903669	0	0	1,099656357	0,774287631	C1QA;ARL2BP;RPSA;RPL39
Atropine-94_mg/kg_in_Water-Rat-Liver-3d-dn	4/250	0,494544662	0,681784309	0	0	1,099656357	0,774287631	EEF1B2;C1QA;RPL35A;TPT1
Beta-Naphthoflavone-1500_mg/kg_in_CMC-Rat-Liver-1d-up	5/319	0,49633984	0,684139408	0	0	1,077249566	0,754607317	C1QA;RPS17;RPSA;RPS3A;RPL10A
Cholecalciferol-8_mg/kg_in_CMC-Rat-Heart-1d-up	5/319	0,49633984	0,684019699	0	0	1,077249566	0,754607317	EEF1B2;RPS19;ARL6IP5;RAPGEF5;RPL39
Diazepam-710_mg/kg_in_CMC-Rat-Kidney-5d-up	5/319	0,49633984	0,683900031	0	0	1,077249566	0,754607317	EEF1B2;RPS17;RPS20;RPSA;RPS3A
Econazole-334_mg/kg_in_Corn_Oil-Rat-Liver-0.25d-dn	5/319	0,49633984	0,683780405	0	0	1,077249566	0,754607317	RPS8;RPS20;RPSA;SEPP1;TPT1
Ferrocene-330_mg/kg_in_CMC-Rat-Kidney-3d-dn	5/319	0,49633984	0,683660822	0	0	1,077249566	0,754607317	RPL32;ARL2BP;RPS6;RPL39;TPT1
Nimesulide-30_mg/kg_in_Corn_Oil-Rat-Liver-5d-dn	5/319	0,49633984	0,68354128	0	0	1,077249566	0,754607317	C1QA;RPS20;RPSA;RPL10A;SEPP1
Quinapril-2500_mg/kg_in_CMC-Rat-Kidney-0.25d-up	5/319	0,49633984	0,683421779	0	0	1,077249566	0,754607317	EEF1B2;RPS17;RPL32;SEPP1;RPL39
Roxarsone-11_mg/kg_in_Corn_Oil-Rat-Kidney-1d-dn	5/319	0,49633984	0,683302321	0	0	1,077249566	0,754607317	EEF1B2;ARL2BP;CLP1;RPS20;SEPP1
Tocainide-67_mg/kg_in_Corn_Oil-Rat-Liver-5d-dn	5/319	0,49633984	0,683182904	0	0	1,077249566	0,754607317	C1QA;RPL32;RPS6;RPS3A;SEPP1
1-Naphthyl_Isothiocyanate-30_mg/kg_in_Corn_Oil-Rat-Kidney-0.25d-dn	4/251	0,497653104	0,684870845	0	0	1,095275256	0,764340053	CLP1;RPS8;RPS20;RPSA
Camptothecin-2.5_mg/kg_in_Saline-Rat-Bone_marrow-3d-dn	4/251	0,497653104	0,684751196	0	0	1,095275256	0,764340053	RPS8;ARL6IP5;RPS3A;TPT1
3-Methylcholanthrene-12.5_mg/kg_in_CMC-Rat-Liver-28d-dn	5/320	0,499091726	0,686610731	0	0	1,073883162	0,74631162	RPL32;RPS8;RPS6;RPS20;RPS3A
Azithromycin-50_mg/kg_in_Water-Rat-Liver-1d-up	5/320	0,499091726	0,68649082	0	0	1,073883162	0,74631162	RPS6;RPS20;RPS3A;RPL10A;SEPP1
Cisplatin-1.17_mg/kg_in_Saline-Rat-Kidney-5d-dn	5/320	0,499091726	0,686370951	0	0	1,073883162	0,74631162	RPS17;CLP1;RPS8;RPS3A;SEPP1
Clotrimazole-52_mg/kg_in_Corn_Oil-Rat-Liver-1d-up	5/320	0,499091726	0,686251123	0	0	1,073883162	0,74631162	RPS19;RPS20;RPSA;RPL10A;RPL39
Ketamine-30_mg/kg_in_Saline-Rat-Heart-1d-up	5/320	0,499091726	0,686131338	0	0	1,073883162	0,74631162	RPS8;RPS5;RPL35A;RAPGEF5;TOMM20
Zidovudine-1540_mg/kg_in_Corn_Oil-Rat-Liver-0.25d-dn	5/320	0,499091726	0,686011594	0	0	1,073883162	0,74631162	EEF1B2;FUNDC1;RPS6;RPS20;RPL39
Aceclofenac-1_mg/kg_in_Corn_Oil-Rat-Kidney-1d-dn	4/252	0,500753428	0,688175537	0	0	1,090928926	0,754531673	RPS19;ARL2BP;RPS8;FUNDC1
Busulfan-9_mg/kg_in_Corn_Oil-Rat-Spleen-3d-up	4/252	0,500753428	0,688055478	0	0	1,090928926	0,754531673	CXCL11;RPS5;RPS6;RPS20
Nabumetone-2500_mg/kg_in_Corn_Oil-Rat-Kidney-3d-dn	4/252	0,500753428	0,687935462	0	0	1,090928926	0,754531673	CLP1;GOLGA7;SEPP1;TPT1
Penciclovir-1200_mg/kg_in_Corn_Oil-Rat-Bone_marrow-1d-dn	4/252	0,500753428	0,687815487	0	0	1,090928926	0,754531673	RPS6;RPSA;RPL10A;SEPP1
Carboplatin-14_mg/kg_in_Saline-Rat-Spleen-3d-up	5/321	0,501837948	0,689184949	0	0	1,070537731	0,738112239	RPS19;RPS20;RPL10A;SEPP1;RPL39
Dicumarol-13_mg/kg_in_Corn_Oil-Rat-Liver-1d-up	5/321	0,501837948	0,689064798	0	0	1,070537731	0,738112239	RPS17;CLP1;RPS20;SEPP1;TPT1
Enalapril-1500_mg/kg_in_CMC-Rat-Kidney-5d-dn	5/321	0,501837948	0,688944689	0	0	1,070537731	0,738112239	RPS17;RPS3A;RPL10A;SEPP1;TPT1
Epirubicin-2.7_mg/kg_in_Saline-Rat-Kidney-5d-dn	5/321	0,501837948	0,688824622	0	0	1,070537731	0,738112239	RPL32;ARL2BP;FEM1B;RPS3A;TPT1
Ethylene_Glycol-352_mg/kg_in_Water-Rat-Liver-0.25d-up	5/321	0,501837948	0,688704597	0	0	1,070537731	0,738112239	EEF1B2;RPS17;RPS20;RPS3A;RPL39
Lead(IV)_Acetate-200_mg/kg_in_Saline-Rat-Liver-1d-up	5/321	0,501837948	0,688584613	0	0	1,070537731	0,738112239	C1QA;CLP1;RPS20;RPL39;GOLGA7
Nifedipine-1022_mg/kg_in_CMC-Rat-Heart-1d-up	5/321	0,501837948	0,688464672	0	0	1,070537731	0,738112239	C1QA;RPS6;RPL10A;SEPP1;RPL39
Rabeprazole-1024_mg/kg_in_Water-Rat-Liver-1d-dn	5/321	0,501837948	0,688344772	0	0	1,070537731	0,738112239	C1QA;CLP1;RPS8;SEPP1;TPT1
Vinblastine-0.3_mg/kg_in_Saline-Rat-Bone_marrow-1d-up	5/321	0,501837948	0,688224914	0	0	1,070537731	0,738112239	C1QA;RPL32;RPS19;SEPP1;TPT1
Acrolein-20_mg/kg_in_Water-Rat-Kidney-5d-up	5/322	0,504578408	0,691862733	0	0	1,06721308	0,730007934	RPS3A;RPL10A;SEPP1;GOLGA7;TPT1
Bisphenol_A-100_mg/kg_in_Corn_Oil-Rat-Kidney-3d-dn	5/322	0,504578408	0,691742305	0	0	1,06721308	0,730007934	RPL32;RPS19;RPS6;RPL10A;RPL39
Gliclazide-1500_mg/kg_in_CMC-Rat-Kidney-5d-up	5/322	0,504578408	0,691621918	0	0	1,06721308	0,730007934	CXCL11;RPSA;SEPP1;RPL39;TPT1
4-Octylphenol-700_mg/kg_in_Corn_Oil-Rat-Kidney-1d-dn	5/323	0,507313008	0,695249217	0	0	1,063909015	0,721997482	RPS19;RPS8;RPS6;SEPP1;RPL39
Aspirin-35_mg/kg_in_Corn_Oil-Rat-Kidney-1d-up	5/323	0,507313008	0,695128262	0	0	1,063909015	0,721997482	RPS19;CLP1;RPS5;RAPGEF5;SEPP1
Esmolol-27_mg/kg_in_Saline-Rat-Heart-1d-up	5/323	0,507313008	0,695007349	0	0	1,063909015	0,721997482	RPS17;RPS19;RPS6;RPL39;GOLGA7
Fluphenazine-2.5_mg/kg_in_Corn_Oil-Rat-Liver-5d-up	5/323	0,507313008	0,694886478	0	0	1,063909015	0,721997482	EEF1B2;RPS20;SEPP1;RPL39;TPT1
Lamivudine-1300_mg/kg_in_Water-Rat-Liver-3d-up	5/323	0,507313008	0,694765649	0	0	1,063909015	0,721997482	EEF1B2;RPS17;RPS6;RPS20;SEPP1
Lead_(II)_Acetate-200_mg/kg_in_Saline-Rat-Spleen-1d-up	5/323	0,507313008	0,694644862	0	0	1,063909015	0,721997482	C1QA;RPS19;RPL10A;SEPP1;RPL39
Miconazole-200_mg/kg_in_Corn_Oil-Rat-Liver-0.25d-dn	5/323	0,507313008	0,694524118	0	0	1,063909015	0,721997482	RPS8;RPS20;RPSA;SEPP1;TPT1
Piclamilast-17_mg/kg_in_CMC-Rat-Liver-3d-dn	5/323	0,507313008	0,694403415	0	0	1,063909015	0,721997482	C1QA;RPL32;RPS19;RPSA;SEPP1
Carbon_Tetrachloride-1175_mg/kg_in_Corn_Oil-Rat-Liver-3d-dn	4/255	0,510004413	0,697966075	0	0	1,078094468	0,725919708	RPS17;CLP1;RPS3A;SEPP1
Clotrimazole-89_mg/kg_in_Corn_Oil-Rat-Liver-1d-up	4/255	0,510004413	0,697844816	0	0	1,078094468	0,725919708	RPS19;RPS8;RPS20;GOLGA7
NN-Dimethylformamide-140_mg/kg_in_Saline-Rat-Spleen-3d-up	4/255	0,510004413	0,697723599	0	0	1,078094468	0,725919708	CXCL11;RPS5;RPL13A;RPL35A

Bisphenol_A-610_mg/kg_in_Corn_Oil-Rat-Spleen-3d-dn	5/324	0,510041652	0,697653361	0	0	1,060625345	0,71407968	C1QA;RPS19;SEPP1;RPL39;TPT1
Doxorubicin-3_mg/kg_in_Saline-Rat-Bone_marrow-3d-up	5/324	0,510041652	0,69753222	0	0	1,060625345	0,71407968	C1QA;CLP1;NKG7;SEPP1;SLC16A3
Leflunomide-60_mg/kg_in_Corn_Oil-Rat-Liver-0.25d-up	5/324	0,510041652	0,69741112	0	0	1,060625345	0,71407968	EEF1B2;RPS6;RPS20;RPL10A;SEPP1
Methotrexate-0.3_mg/kg_in_Corn_Oil-Rat-Bone_marrow-0.25d-up	5/324	0,510041652	0,697290063	0	0	1,060625345	0,71407968	EEF1B2;C1QA;SEPP1;RPL39;TPT1
Niacin-2625_mg/kg_in_Water-Rat-Liver-0.25d-dn	5/324	0,510041652	0,697169048	0	0	1,060625345	0,71407968	RPS17;RPS3A;SEPP1;RPL39;TPT1
Tamoxifen-37.5_uM_in_DMSO-Rat-Primary_rat_hepatocytes-1d-dn	5/324	0,510041652	0,697048075	0	0	1,060625345	0,71407968	ARL6IP5;RPS20;SEPP1;RPL39;TPT1
Carbachol-15_mg/kg_in_Water-Rat-Brain-5d-dn	5/325	0,512764247	0,70064733	0	0	1,057361882	0,706253341	ARL2BP;RPS8;RPL35A;RPSA;TOMM20
Phenothiazine-36_mg/kg_in_Corn_Oil-Rat-Liver-1d-up	5/325	0,512764247	0,700525795	0	0	1,057361882	0,706253341	RPS19;RPSA;SEPP1;RPL39;TPT1
Salicylamide-1300_mg/kg_in_CMC-Rat-Kidney-3d-up	5/325	0,512764247	0,700404303	0	0	1,057361882	0,706253341	RPS17;RPS3A;RAPGEF5;SEPP1;TPT1
Valdecoxib-404_mg/kg_in_CMC-Rat-Heart-3d-dn	5/325	0,512764247	0,700282852	0	0	1,057361882	0,706253341	COL5A3;FUNDC1;RPS6;RPS3A;SEPP1
Carboplatin-14_mg/kg_in_Saline-Rat-Kidney-1d-dn	4/256	0,513070996	0,700580299	0	0	1,073883162	0,716646316	RPL32;RPS19;FEM1B;SEPP1
Carmustine-16_mg/kg_in_Corn_Oil-Rat-Liver-1d-dn	4/256	0,513070996	0,70045886	0	0	1,073883162	0,716646316	EEF1B2;RPS17;RPS19;RPSA
Rofecoxib-775_mg/kg_in_Corn_Oil-Rat-Liver-3d-up	5/326	0,515480699	0,703626687	0	0	1,054118441	0,698517296	EEF1B2;C1QA;RPS8;RPSA;TPT1
Diethylstilbestrol-2.8_mg/kg_in_Corn_Oil-Rat-Liver-3d-dn	4/257	0,516128837	0,704389312	0	0	1,069704628	0,707501423	RPS17;CLP1;RPS3A;SEPP1
Flurbiprofen-10_mg/kg_in_CMC-Rat-Kidney-5d-dn	4/257	0,516128837	0,704267276	0	0	1,069704628	0,707501423	CLP1;GOLGA7;SEPP1;TPT1
Tretinoin-156_uM_in_DMSO-Rat-Primary_rat_hepatocytes-1d-up	4/257	0,516128837	0,704145283	0	0	1,069704628	0,707501423	EEF1B2;ARL2BP;RPS20;TPT1
Atropine-94_mg/kg_in_Water-Rat-Liver-5d-up	5/327	0,518190916	0,706836102	0	0	1,050894837	0,690870395	RPS6;RPSA;RPS3A;SEPP1;RPL39
Busulfan-9_mg/kg_in_Corn_Oil-Rat-Spleen-5d-dn	5/327	0,518190916	0,706713706	0	0	1,050894837	0,690870395	RPS17;RPL32;RPS6;RPL35A;RPL39
Chloramphenicol-1500_mg/kg_in_CMC-Rat-Kidney-3d-up	5/327	0,518190916	0,706591352	0	0	1,050894837	0,690870395	RPS17;RPS19;RPS6;RPSA;RPL39
Indomethacin-9.6_mg/kg_in_Water-Rat-Liver-0.25d-up	5/327	0,518190916	0,706469041	0	0	1,050894837	0,690870395	RPS19;RPS20;RPS3A;RPL10A;GOLGA7
Mebendazole-714_mg/kg_in_Corn_Oil-Rat-Liver-3d-dn	5/327	0,518190916	0,706346772	0	0	1,050894837	0,690870395	C1QA;RPS17;RPS3A;GOLGA7;TPT1
Naloxone-76_mg/kg_in_Saline-Rat-Liver-1d-up	5/327	0,518190916	0,706224546	0	0	1,050894837	0,690870395	RPS17;RPL32;RPL35A;RPS20;RPSA
Rifabutin-1500_mg/kg_in_CMC-Rat-Kidney-5d-up	5/327	0,518190916	0,706102362	0	0	1,050894837	0,690870395	RPS6;RPS20;SEPP1;RPL39;TPT1
Zileuton-450_mg/kg_in_Corn_Oil-Rat-Liver-1d-dn	5/327	0,518190916	0,70598022	0	0	1,050894837	0,690870395	EEF1B2;RPS17;RPS8;RPL39;TPT1
Carboplatin-5_mg/kg_in_Saline-Rat-Kidney-14d-dn	4/258	0,519177817	0,707202437	0	0	1,065558486	0,698483006	RPS17;RPS8;RAPGEF5;SEPP1
Doxorubicin-3_mg/kg_in_Saline-Rat-Heart-1d-up	4/258	0,519177817	0,707080147	0	0	1,065558486	0,698483006	CXCL11;RPS19;GOLGA7;SEPP1
Naproxen-10_mg/kg_in_Corn_Oil-Rat-Liver-3d-dn	4/258	0,519177817	0,706957899	0	0	1,065558486	0,698483006	RPS17;CLP1;RPS3A;SEPP1
Benazepril-1750_mg/kg_in_CMC-Rat-Kidney-3d-up	5/328	0,520894807	0,709173293	0	0	1,047690889	0,683311503	RPS17;CXCL11;RPL32;SEPP1;RPL39
Phenacetin-619_mg/kg_in_Corn_Oil-Rat-Kidney-1d-dn	5/328	0,520894807	0,709050726	0	0	1,047690889	0,683311503	RPS6;RPSA;SEPP1;RPL39;PDRG1
Aminocaproic_Acid-2000_mg/kg_in_Water-Rat-Kidney-3d-dn	4/259	0,522217822	0,7107288	0	0	1,06144436	0,689589082	RPS17;RPL10A;RPL39;SEPP1
Emetine-1_mg/kg_in_Saline-Rat-Kidney-3d-up	4/260	0,525248738	0,714730314	0	0	1,057361882	0,680817703	RPS17;RPL32;RPS8;RPS3A
Meloxicam-6_mg/kg_in_Corn_Oil-Rat-Liver-1d-dn	4/260	0,525248738	0,714606851	0	0	1,057361882	0,680817703	RPS17;RPS8;RPS3A;RPL39
Prinomastat-500_uM_in_DMSO-Rat-Primary_rat_hepatocytes-1d-up	4/260	0,525248738	0,71448343	0	0	1,057361882	0,680817703	EEF1B2;RPL32;RPS8;RPS20
Cromolyn-1500_mg/kg_in_Water-Rat-Kidney-5d-up	5/330	0,52628326	0,715767045	0	0	1,041341248	0,668453289	RPS17;RPL32;FUNDC1;RPS6;RPL39
Hydroquinone-800_mg/kg_in_CMC-Rat-Kidney-1d-dn	5/330	0,52628326	0,715643466	0	0	1,041341248	0,668453289	RPS19;ARL2BP;RPS6;RPS20;RPL39
Nizatidine-1653_mg/kg_in_Corn_Oil-Rat-Heart-3d-up	5/330	0,52628326	0,715519931	0	0	1,041341248	0,668453289	CXCL11;RPS20;RPSA;RPL10A;TPT1
Rapamycin-60_mg/kg_in_CMC-Rat-Heart-5d-dn	5/330	0,52628326	0,715396437	0	0	1,041341248	0,668453289	COL5A3;ARL6IP5;RAPGEF5;SEPP1;RPL39
Cimetidine-100_mg/kg_in_CMC-Rat-Heart-3d-up	6/400	0,527338698	0,716707435	0	0	1,030927835	0,659703346	RPS17;RPL32;RPS3A;SEPP1;RPL39;TPT1
Pyrogallol-1000_mg/kg_in_Water-Rat-Liver-1d-dn	3/191	0,527792301	0,717200166	0	0	1,079505586	0,689860681	EEF1B2;C1QA;SEPP1
Hydroxyurea-400_mg/kg_in_Saline-Rat-Bone_marrow-3d-up	4/261	0,528270453	0,71772608	0	0	1,053310687	0,672166955	C1QA;RPS8;RPS20;SEPP1
Sotalol-2000_mg/kg_in_Water-Rat-Liver-5d-up	4/261	0,528270453	0,717602291	0	0	1,053310687	0,672166955	RPS8;RPS3A;RAPGEF5;SEPP1
Lorazepam-2000_mg/kg_in_CMC-Rat-Liver-3d-up	5/331	0,528967647	0,718425451	0	0	1,038195201	0,661151782	RPS19;RPS6;RPS20;RPSA;RPL39
Methyldopa-325_mg/kg_in_Water-Rat-Heart-1d-dn	5/331	0,528967647	0,718301584	0	0	1,038195201	0,661151782	EEF1B2;RPS19;RPS20;RPL10A;RPL39
Busulfan-36_mg/kg_in_Corn_Oil-Rat-Liver-0.25d-dn	4/262	0,531282858	0,721321115	0	0	1,049290417	0,663634963	EEF1B2;RPS17;RPS3A;TPT1
Celecoxib-400_mg/kg_in_Corn_Oil-Rat-Liver-3d-up	4/262	0,531282858	0,721196792	0	0	1,049290417	0,663634963	RPS17;RPS3A;GOLGA7;SEPP1
Genistein-20_mg/kg_in_Corn_Oil-Rat-Liver-5d-up	4/262	0,531282858	0,721072512	0	0	1,049290417	0,663634963	RPS19;RPS6;RPS20;RPSA
Methyl_Salicylate-444_mg/kg_in_Corn_Oil-Rat-Liver-5d-dn	4/262	0,531282858	0,720948275	0	0	1,049290417	0,663634963	EEF1B2;RPS17;RPL10A;SEPP1
Nimesulide-30_mg/kg_in_Corn_Oil-Rat-Liver-0.25d-up	4/262	0,531282858	0,720824081	0	0	1,049290417	0,663634963	RPS17;RPS19;RPS6;RPS20
Sodium_Arsenite-30_mg/kg_in_Saline-Rat-Liver-1d-dn	4/262	0,531282858	0,720699929	0	0	1,049290417	0,663634963	RPL32;RPS19;SEPP1;TPT1
Sulindac-64_mg/kg_in_Corn_Oil-Rat-Kidney-1d-dn	4/262	0,531282858	0,720575821	0	0	1,049290417	0,663634963	ARL2BP;RPS8;FUNDC1;SEPP1
Acetaminophen-100_mg/kg_in_Corn_Oil-Rat-Kidney-0.25d-up	5/332	0,53164536	0,720943329	0	0	1,035068107	0,653933909	RPS17;FUNDC1;RPS20;RPS3A;RPL39
Idarubicin-4_mg/kg_in_Saline-Rat-Heart-14d-dn	5/332	0,53164536	0,720819221	0	0	1,035068107	0,653933909	RPS17;COL5A3;R3HCC1;ARL6IP5;RPL39
Choline_Chloride-2550_mg/kg_in_Water-Rat-Liver-0.25d-dn	4/263	0,534285847	0,724274584	0	0	1,04530072	0,655219881	EEF1B2;CLP1;RPL39;TPT1
Eucalyptol-930_mg/kg_in_Corn_Oil-Rat-Kidney-5d-dn	4/263	0,534285847	0,724149945	0	0	1,04530072	0,655219881	CLP1;RPL10A;SEPP1;TPT1
Fenofibrate-430_mg/kg_in_Corn_Oil-Rat-Liver-5d-up	4/263	0,534285847	0,724025349	0	0	1,04530072	0,655219881	RPS8;RPS20;RPS3A;TPT1
Rifapentine-75_mg/kg_in_Corn_Oil-Rat-Kidney-1d-dn	4/263	0,534285847	0,723900797	0	0	1,04530072	0,655219881	CLP1;R3HCC1;RPL39;SEPP1
Rofecoxib-775_mg/kg_in_Corn_Oil-Rat-Liver-1d-dn	4/263	0,534285847	0,723776287	0	0	1,04530072	0,655219881	RPS17;RPS3A;RPL39;SEPP1
Sildenafil-300_mg/kg_in_Saline-Rat-Liver-1d-up	5/333	0,534316316	0,723693088	0	0	1,031959795	0,646798616	RPS17;RPS19;RPS8;RPS20;RPSA
Diethanolamine-620_mg/kg_in_Water-Rat-Kidney-3d-dn	3/193	0,534816907	0,724246554	0	0	1,068319	0,668586957	RPS17;RPL32;RPSA
Losartan-1000_mg/kg_in_Water-Rat-Kidney-1d-dn	3/193	0,534816907	0,724122049	0	0	1,068319	0,668586957	RPS17;RPS20;RPSA
Risperidone-400_uM_in_DMSO-Rat-Primary_rat_hepatocytes-1d-up	3/193	0,534816907	0,723997586	0	0	1,068319	0,668586957	FEM1B;RPS20;PDRG1
Daunorubicin-3.25_mg/kg_in_Saline-Rat-Heart-3d-up	5/334	0,536980432	0,726801492	0	0	1,028870095	0,639744866	CXCL11;RPS19;ARL6IP5;SEPP1;TPT1
Famotidine-1200_mg/kg_in_CMC-Rat-Heart-1d-up	5/334	0,536980432	0,726676612	0	0	1,028870095	0,639744866	RPS17;RPS6;ARL6IP5;RPS20;PDRG1
1-Naphthyl_Isothiocyanate-30_mg/kg_in_Corn_Oil-Rat-Liver-3d-dn	4/264	0,537279315	0,726956173	0	0	1,041341248	0,646919901	RPS17;CLP1;RPS3A;SEPP1
24-Diaminophenol-60_mg/kg_in_Water-Rat-Kidney-1d-dn	4/264	0,537279315	0,726831309	0	0	1,041341248	0,646919901	RPS17;RPL32;RPL35A;RPS20
Isoprenaline-15_mg/kg_in_Saline-Rat-Liver-5d-dn	4/264	0,537279315	0,726706488	0	0	1,041341248	0,646919901	EEF1B2;CLP1;SEPP1;TPT1
Tosufloxacin-2000_mg/kg_in_CMC-Rat-Liver-1d-up	4/264	0,537279315	0,726581711	0	0	1,041341248	0,646919901	ARL2BP;RPL39;GOLGA7;TPT1
Trans-Platinum(II)Diammine_Dichloride-0.5_mg/kg_in_Saline-Rat-Kidney-14d-dn	4/264	0,537279315	0,726456976	0	0	1,041341248	0,646919901	ARL2BP;CLP1;FUNDC1;RPSA
Venlafaxine-320_mg/kg_in_Water-Rat-Liver-1d-dn	4/264	0,537279315	0,726332283	0	0	1,041341248	0,646919901	CLP1;RPS8;RPL39;TPT1
Diethylstilbestrol-280_mg/kg_in_Corn_Oil-Rat-Spleen-3d-dn	5/335	0,539637626	0,72939522	0	0	1,025798841	0,632771634	C1QA;ARL2BP;SEPP1;RPL39;TPT1
Digoxin-11_mg/kg_in_Corn_Oil-Rat-Heart-5d-dn	5/335	0,539637626	0,729270066	0	0	1,025798841	0,632771634	COL5A3;RPSA;SEPP1;RPL39;TOMM20
Trihexyphenidyl-588_mg/kg_in_CMC-Rat-Heart-5d-dn	5/335	0,539637626	0,729144955	0	0	1,025798841	0,632771634	COL5A3;RPSA;RPL10A;SEPP1;RPL39
Amitraz-75_mg/kg_in_CMC-Rat-Heart-3d-up	4/265	0,540263159	0,729864947	0	0	1,037411658	0,638733244	RPS19;RPS6;GOLGA7;SEPP1
Azathioprine-20_mg/kg_in_Corn_Oil-Rat-Spleen-0.25d-dn	4/265	0,540263159	0,729739777	0	0	1,037411658	0,638733244	C1QA;RPS17;RPS8;TPT1
Lead_(II)_Acetate-2_mg/kg_in_Saline-Rat-Kidney-14d-dn	4/265	0,540263159	0,72961465	0	0	1,037411658	0,638733244	CLP1;RPSA;SEPP1;TOMM20
Phenylhydrazine-78_mg/kg_in_Water-Rat-Liver-3d-dn	4/265	0,540263159	0,729489566	0	0	1,037411658	0,638733244	CLP1;RPS20;RPL39;SEPP1
Zomepirac-11_mg/kg_in_Saline-Rat-Liver-0.25d-up	4/265	0,540263159	0,729364525	0	0	1,037411658	0,638733244	C1QA;RPL35A;RPS3A;SEPP1
Amikacin-160_mg/kg_in_Saline-Rat-Kidney-14d-dn	4/266	0,543237279	0,733253952	0	0	1,033511614	0,630658167	EEF1B2;RPS17;RPS19;FUNDC1
Methyldopa-325_mg/kg_in_Water-Rat-Heart-5d-up	4/266	0,543237279	0,733128309	0	0	1,033511614	0,630658167	C1QA;RPS17;RPL10A;SEPP1
Phalloidin-1_uM_in_DMSO-Rat-Primary_rat_hepatocytes-1d-up	4/266	0,543237279	0,733002709	0	0	1,033511614	0,630658167	EEF1B2;ARL2BP;ARL6IP5;RPS20
Tenidap-41_uM_in_DMSO-Rat-Primary_rat_hepatocytes-0.67d-up	4/266	0,543237279	0,732877152	0	0	1,033511614	0,630658167	RPS3A;RPL39;SLC16A3;TPT1
44'-Diethylaminoethoxyhexestrol-20_uM_in_DMSO-Rat-Primary_rat_hepatocytes-0.67d-dn	4/267	0,546201578	0,736750064	0	0	1,029640784	0,622692955	RPS17;RPL32;RPS3A;PDRG1
Celecoxib-400_mg/kg_in_Corn_Oil-Rat-Heart-3d-dn	4/267	0,546201578	0,736623909	0	0	1,029640784	0,622692955	RPS17;RPS6;RPS20;SEPP1
Chlorpyrifos-82_mg/kg_in_CMC-Rat-Kidney-5d-up	4/267	0,546201578	0,736497796	0	0	1,029640784	0,622692955	CLP1;FEM1B;RPS3A;TPT1
Cyclosporin_A-350_mg/kg_in_Corn_Oil-Rat-Kidney-3d-dn	4/267	0,546201578	0,736371726	0	0	1,029640784	0,622692955	ARL2BP;CLP1;SEPP1;TPT1
Ethosuximide-1200_mg/kg_in_Water-Rat-Brain-3d-up	4/267	0,546201578	0,7362457	0	0	1,029640784	0,622692955	RPS17;RPS8;ARL6IP5;TPT1
Harringtonine-23_mg/kg_in_Saline-Rat-Bone_marrow-1d-dn	4/267	0,546201578	0,736119717	0	0	1,029640784	0,622692955	RPS8;NKG7;RPS20;TPT1
Pralidoxime_Chloride-48_mg/kg_in_Saline-Rat-Heart-1d-up	4/267	0,546201578	0,735993777	0	0	1,029640784	0,622692955	C1QA;RPS6;RPL10A;RPL39
Sildenafil-300_mg/kg_in_Saline-Rat-Liver-1d-dn	4/267	0,546201578	0,73586788	0	0	1,029640784	0,622692955	C1QA;RPL39;SEPP1;TPT1
Venlafaxine-320_mg/kg_in_Water-Rat-Kidney-1d-up	6/408	0,546719201	0,736439272	0	0	1,010713564	0,610289016	EEF1B2;CLP1;RPS8;RPS5;SEPP1;TPT1

Nevirapine-29_mg/kg_in_Saline-Rat-Liver-3d-dn	5/338	0,54756689	0,737454998	0	0	1,016694117	0,612325028	C1QA;RPS19;RPS8;SEPP1;TPT1
Nimesulide-30_mg/kg_in_Corn_Oil-Rat-Liver-0.25d-dn	5/338	0,54756689	0,737328916	0	0	1,016694117	0,612325028	RPS8;RPSA;SEPP1;RPL39;TPT1
Ethylene_Glycol-352_mg/kg_in_Water-Rat-Kidney-3d-up	6/409	0,549117333	0,739290276	0	0	1,008242381	0,604383978	EEF1B2;RPS17;RPS20;RPS3A;SEPP1;GOLGA7
Ampicillin-2000_mg/kg_in_CMC-Rat-Kidney-5d-dn	4/268	0,549155958	0,739215916	0	0	1,025798841	0,614835925	RPS3A;RPL39;SEPP1;TPT1
Chlorambucil-0.6_mg/kg_in_Corn_Oil-Rat-Liver-0.25d-dn	4/268	0,549155958	0,739089598	0	0	1,025798841	0,614835925	RPS8;RPS20;RPSA;TPT1
Ethosuximide-1200_mg/kg_in_Water-Rat-Heart-5d-up	4/268	0,549155958	0,738963322	0	0	1,025798841	0,614835925	EEF1B2;RPS8;RPL10A;RPL39
Naloxone-76_mg/kg_in_Saline-Rat-Liver-5d-dn	4/268	0,549155958	0,73883709	0	0	1,025798841	0,614835925	CLP1;GOLGA7;SEPP1;TPT1
Zidovudine-1540_mg/kg_in_Corn_Oil-Rat-Liver-5d-dn	4/268	0,549155958	0,738710901	0	0	1,025798841	0,614835925	C1QA;RPS6;RPSA;RPL10A
Dipyridamole-750_mg/kg_in_CMC-Rat-Liver-3d-up	4/269	0,552100327	0,74254477	0	0	1,021985462	0,607085423	RPS19;RPS6;RPS20;RPL39
L-Buthionine_Sulfoximine-100000_uM_in_DMSO-Rat-Primary_rat_hepatocytes-0.67d-up	4/269	0,552100327	0,742417991	0	0	1,021985462	0,607085423	RPS17;RPS19;FEM1B;PDRG1
Metoclopramide-185_mg/kg_in_CMC-Rat-Intestine-5d-up	4/269	0,552100327	0,742291255	0	0	1,021985462	0,607085423	RPL32;FEM1B;RPS20;TPT1
Acetaminophen-100_mg/kg_in_Corn_Oil-Rat-Liver-1d-up	5/340	0,55281703	0,743127996	0	0	1,010713564	0,599078432	RPL32;RPS19;RPS20;RPSA;SEPP1
Fluocinolone_Acetonide-2.5_mg/kg_in_Corn_Oil-Rat-Kidney-5d-up	5/340	0,55281703	0,743001182	0	0	1,010713564	0,599078432	CXCL11;RPS6;RPS20;SEPP1;TPT1
Meloxicam-6_mg/kg_in_Corn_Oil-Rat-Liver-1d-up	5/340	0,55281703	0,742874412	0	0	1,010713564	0,599078432	EEF1B2;RPS19;RPS6;RPS20;TPT1
Altretamine-13_mg/kg_in_Corn_Oil-Rat-Liver-0.25d-dn	4/270	0,555034592	0,745727131	0	0	1,018200331	0,599439827	RPS6;RPS20;RPSA;RPL10A
Beta-Estradiol-150_mg/kg_in_Corn_Oil-Rat-Skeletal_muscle-3d-up	4/270	0,555034592	0,745599939	0	0	1,018200331	0,599439827	RPS19;RPS6;RPS20;RPL39
Clotrimazole-52_mg/kg_in_Corn_Oil-Rat-Liver-0.25d-dn	4/270	0,555034592	0,74547279	0	0	1,018200331	0,599439827	EEF1B2;RPS8;RPL39;SEPP1
Enrofloxacin-2000_mg/kg_in_CMC-Rat-Kidney-1d-dn	4/270	0,555034592	0,745345685	0	0	1,018200331	0,599439827	RPS8;RPS3A;SEPP1;TPT1
Epirubicin-2.7_mg/kg_in_Saline-Rat-Kidney-1d-dn	4/270	0,555034592	0,745218623	0	0	1,018200331	0,599439827	ARL2BP;RPS6;RPSA;TPT1
Fluphenazine-22_mg/kg_in_Corn_Oil-Rat-Liver-1d-dn	4/270	0,555034592	0,745091604	0	0	1,018200331	0,599439827	RPSA;RPL39;SEPP1;TPT1
Phenylhydrazine-78_mg/kg_in_Water-Rat-Liver-1d-dn	4/270	0,555034592	0,744964629	0	0	1,018200331	0,599439827	C1QA;RPS19;RPL10A;SEPP1
Tranexamic_Acid-2000_mg/kg_in_CMC-Rat-Heart-1d-up	5/341	0,555431065	0,74536975	0	0	1,007749594	0,592567619	CXCL11;RPS19;RAPGEF5;SEPP1;TPT1
Colchicine-2000_uM_in_DMSO-Rat-Primary_rat_hepatocytes-0.67d-dn	4/271	0,557958663	0,748634145	0	0	1,014443134	0,591897541	RPS17;RPL32;RPS3A;PDRG1
Cytarabine-487_mg/kg_in_Saline-Rat-Spleen-1d-dn	4/271	0,557958663	0,748506631	0	0	1,014443134	0,591897541	RPS8;FUNDC1;RPS3A;TPT1
Genistein-20_mg/kg_in_Corn_Oil-Rat-Liver-3d-dn	4/271	0,557958663	0,748379161	0	0	1,014443134	0,591897541	C1QA;RPS17;CLP1;RPS3A
Vincristine-0.05_mg/kg_in_Saline-Rat-Heart-5d-dn	4/271	0,557958663	0,748251734	0	0	1,014443134	0,591897541	ARL6IP5;RAPGEF5;GOLGA7;SEPP1
Zileuton-450_mg/kg_in_Corn_Oil-Rat-Kidney-5d-dn	4/271	0,557958663	0,74812435	0	0	1,014443134	0,591897541	EEF1B2;CLP1;SEPP1;TPT1
Tocainide-67_mg/kg_in_Corn_Oil-Rat-Liver-0.25d-dn	5/342	0,558037646	0,748102894	0	0	1,004802958	0,586130557	RPS17;RPL32;RPS19;RPS20;RPL10A
44'-Methylenedianiline-81_mg/kg_in_Corn_Oil-Rat-Kidney-1d-dn	5/343	0,560636703	0,751459271	0	0	1,001873503	0,579766334	RPS19;RPS8;RPS6;SEPP1;RPL39
Fluvastatin-5_mg/kg_in_Corn_Oil-Rat-Kidney-0.25d-up	4/272	0,560872454	0,751647345	0	0	1,010713564	0,584456998	RPS19;RPS6;GOLGA7;SEPP1
N-Nitrosodiethylamine-1.67_mg/kg_in_Saline-Rat-Spleen-1d-dn	4/272	0,560872454	0,751519471	0	0	1,010713564	0,584456998	RPL32;RPS8;FUNDC1;TPT1
Ofloxacin-3000_mg/kg_in_Water-Rat-Heart-5d-up	4/272	0,560872454	0,75139164	0	0	1,010713564	0,584456998	FUNDC1;RPS6;RAPGEF5;SEPP1
Phenytoin-572_mg/kg_in_Water-Rat-Heart-3d-up	4/272	0,560872454	0,751263852	0	0	1,010713564	0,584456998	RPS19;RPS6;GOLGA7;SEPP1
Tolazamide-1500_mg/kg_in_CMC-Rat-Heart-5d-up	4/272	0,560872454	0,751136108	0	0	1,010713564	0,584456998	EEF1B2;RPS19;CLP1;RPL39
Cloxacillin-1200_mg/kg_in_Water-Rat-Kidney-1d-dn	5/344	0,563228166	0,754162706	0	0	0,99896108	0,573474051	ARL2BP;FUNDC1;RPSA;SEPP1;TPT1
N-Nitrosodiethylamine-34_mg/kg_in_Saline-Rat-Liver-1d-up	5/344	0,563228166	0,754034513	0	0	0,99896108	0,573474051	RPS8;RPSA;SEPP1;RPL39;TPT1
2-Acetylaminofluorene-30_mg/kg_in_CMC-Rat-Kidney-3d-up	4/273	0,563775879	0,7546395	0	0	1,007011316	0,577116659	RPS17;RPS19;CLP1;RPS3A
Clofibrate-130_mg/kg_in_Corn_Oil-Rat-Kidney-3d-up	4/273	0,563775879	0,754511269	0	0	1,007011316	0,577116659	ARL2BP;CLP1;GOLGA7;SEPP1
Dipyrone-1636_mg/kg_in_Water-Rat-Liver-1d-dn	4/273	0,563775879	0,754383082	0	0	1,007011316	0,577116659	EEF1B2;C1QA;CLP1;SEPP1
Naproxen-10_mg/kg_in_Corn_Oil-Rat-Kidney-3d-dn	4/273	0,563775879	0,754254938	0	0	1,007011316	0,577116659	RPS19;RPS6;RPL10A;SEPP1
Stannous_Fluoride-90_mg/kg_in_Water-Rat-Kidney-3d-dn	4/273	0,563775879	0,754126838	0	0	1,007011316	0,577116659	RPS8;FEM1B;FUNDC1;RPSA
Vancomycin-160_mg/kg_in_Saline-Rat-Kidney-1d-dn	4/273	0,563775879	0,753998781	0	0	1,007011316	0,577116659	ARL2BP;CLP1;RPS20;TPT1
Vinblastine-0.3_mg/kg_in_Saline-Rat-Heart-3d-dn	4/273	0,563775879	0,753870767	0	0	1,007011316	0,577116659	RPS17;RPS3A;GOLGA7;SEPP1
4-Chloroaniline-75_mg/kg_in_CMC-Rat-Kidney-3d-dn	4/274	0,566668853	0,757610574	0	0	1,003336093	0,569875013	ARL2BP;CLP1;FUNDC1;SEPP1
Chlordiazepoxide-196_mg/kg_in_Water-Rat-Heart-1d-dn	4/274	0,566668853	0,757481991	0	0	1,003336093	0,569875013	RPL10A;RPL39;GOLGA7;SEPP1
Chlorpromazine-73_mg/kg_in_Water-Rat-Liver-5d-up	4/274	0,566668853	0,757353451	0	0	1,003336093	0,569875013	EEF1B2;RPS6;RPS3A;SEPP1
Clofibric_Acid-448_mg/kg_in_Corn_Oil-Rat-Heart-1d-up	4/274	0,566668853	0,757224956	0	0	1,003336093	0,569875013	RPS19;RPSA;RAPGEF5;TPT1
Cortisone-206_mg/kg_in_CMC-Rat-Liver-1d-up	4/274	0,566668853	0,757096504	0	0	1,003336093	0,569875013	RPS17;RPSA;RPL39;SEPP1
Digitonin-75_mg/kg_in_Corn_Oil-Rat-Heart-1d-dn	4/274	0,566668853	0,756968095	0	0	1,003336093	0,569875013	ARL2BP;RPS8;RAPGEF5;SEPP1
Fluocinolone_Acetonide-2.5_mg/kg_in_Corn_Oil-Rat-Kidney-3d-dn	4/274	0,566668853	0,75683973	0	0	1,003336093	0,569875013	RPS17;ARL2BP;RPSA;RPL39
Phenylbutazone-368_mg/kg_in_CMC-Rat-Kidney-5d-dn	4/274	0,566668853	0,756711409	0	0	1,003336093	0,569875013	EEF1B2;RPS17;RPS19;RPS3A
Valproic_Acid-1340_mg/kg_in_Water-Rat-Liver-0.25d-dn	4/274	0,566668853	0,756583131	0	0	1,003336093	0,569875013	RPS8;RPS6;RPL35A;TPT1
Daunorubicin-3.25_mg/kg_in_Saline-Rat-Kidney-1d-up	5/346	0,568388039	0,758749863	0	0	0,993186739	0,561101768	ARL2BP;RPS6;RPS20;RAPGEF5;SEPP1
Amoxapine-313_mg/kg_in_CMC-Rat-Liver-3d-dn	4/275	0,569551297	0,760173871	0	0	0,999687598	0,562730573	EEF1B2;C1QA;SEPP1;TPT1
Benzethonium_Chloride-30_mg/kg_in_Corn_Oil-Rat-Liver-5d-up	4/275	0,569551297	0,760045072	0	0	0,999687598	0,562730573	EEF1B2;C1QA;RPSA;SEPP1
Chlorpyrifos-82_mg/kg_in_CMC-Rat-Heart-5d-up	4/275	0,569551297	0,759916316	0	0	0,999687598	0,562730573	RPS3A;RAPGEF5;SEPP1;TPT1
Cisplatin-2_mg/kg_in_Saline-Rat-Spleen-1d-up	4/275	0,569551297	0,759787604	0	0	0,999687598	0,562730573	RPS6;RPS20;RPL10A;RPL39
Lomustine-8.75_mg/kg_in_Corn_Oil-Rat-Spleen-1d-up	4/275	0,569551297	0,759658936	0	0	0,999687598	0,562730573	CLP1;FUNDC1;RPS6;RPS20
Methotrexate-27_mg/kg_in_Corn_Oil-Rat-Liver-1d-up	4/275	0,569551297	0,759530311	0	0	0,999687598	0,562730573	RPS19;CLP1;RPS20;RPL39
Oxybutynin-230_mg/kg_in_Water-Rat-Heart-1d-up	4/275	0,569551297	0,759401729	0	0	0,999687598	0,562730573	RPS19;RPS6;RPSA;SEPP1
Simvastatin-1200_mg/kg_in_Corn_Oil-Rat-Liver-3d-dn	5/347	0,570956315	0,761146232	0	0	0,990324529	0,555020033	RPL32;CLP1;RPS8;SEPP1;TPT1
Troxipide-1200_mg/kg_in_CMC-Rat-Heart-5d-dn	5/347	0,570956315	0,76101742	0	0	0,990324529	0,555020033	RPS17;FEM1B;R3HCC1;SEPP1;RPL39
(R)-Bicalutamide-1400_mg/kg_in_CMC-Rat-Kidney-1d-dn	4/276	0,57242313	0,762843413	0	0	0,996065541	0,555681881	EEF1B2;ARL2BP;RPS3A;RPL39
Ampiroxicam-94_mg/kg_in_CMC-Rat-Kidney-1d-dn	4/276	0,57242313	0,762714358	0	0	0,996065541	0,555681881	ARL6IP5;RPS20;RPL39;GOLGA7
Cyclosporin_A-70_mg/kg_in_Corn_Oil-Rat-Liver-0.25d-dn	4/276	0,57242313	0,762585347	0	0	0,996065541	0,555681881	CLP1;RPS20;RPS3A;SEPP1
Flubendazole-1200_mg/kg_in_Corn_Oil-Rat-Liver-3d-up	4/276	0,57242313	0,762456379	0	0	0,996065541	0,555681881	RPS6;RAPGEF5;RPL10A;RPL39
Lead(IV)_Acetate-200_mg/kg_in_Saline-Rat-Liver-5d-dn	4/276	0,57242313	0,762327455	0	0	0,996065541	0,555681881	EEF1B2;RPL32;RPS19;SEPP1
Lipopolysaccharide_E._Coli_O55:B5-1.25_mg/kg_in_Saline-Rat-Liver-3d-up	4/276	0,57242313	0,762198575	0	0	0,996065541	0,555681881	C1QA;CLP1;RPS8;SEPP1
Marimastat-1000_uM_in_DMSO-Rat-Primary_rat_hepatocytes-0.67d-up	4/276	0,57242313	0,762069738	0	0	0,996065541	0,555681881	RPS6;RPL10A;RPL39;TPT1
Sildenafil-300_mg/kg_in_Saline-Rat-Liver-0.25d-up	4/276	0,57242313	0,761940945	0	0	0,996065541	0,555681881	CLP1;RPS8;RPS3A;SEPP1
Carboplatin-6_mg/kg_in_Saline-Rat-Kidney-1d-up	5/348	0,573516731	0,76326762	0	0	0,987478769	0,549006763	RPS8;FEM1B;RPS6;RPSA;RPL39
Cilostazol-1500_mg/kg_in_CMC-Rat-Heart-1d-up	5/348	0,573516731	0,763138668	0	0	0,987478769	0,549006763	RPS17;RPL32;RPS6;SEPP1;PDRG1
D-Galactosamine-470_uM_in_DMSO-Rat-Primary_rat_hepatocytes-0.67d-up	5/348	0,573516731	0,763009759	0	0	0,987478769	0,549006763	ARL6IP5;RPSA;RPL10A;SEPP1;GOLGA7
Dideoxycytidine-1200_mg/kg_in_CMC-Rat-Liver-3d-up	4/277	0,575284274	0,765232046	0	0	0,992469637	0,5487275	EEF1B2;RPS3A;RAPGEF5;TPT1
Dipyrone-1636_mg/kg_in_Water-Rat-Liver-5d-up	4/277	0,575284274	0,765102828	0	0	0,992469637	0,5487275	RPS6;RPS20;RPL10A;RPL39
Etoposide-188_mg/kg_in_Corn_Oil-Rat-Heart-5d-up	4/277	0,575284274	0,764973653	0	0	0,992469637	0,5487275	ARL2BP;FUNDC1;SEPP1;PDRG1
Famciclovir-1200_mg/kg_in_Saline-Rat-Liver-5d-dn	4/277	0,575284274	0,764844521	0	0	0,992469637	0,5487275	C1QA;RPL39;SEPP1;TPT1
Lead(IV)_Acetate-600_mg/kg_in_Saline-Rat-Spleen-5d-dn	4/277	0,575284274	0,764715434	0	0	0,992469637	0,5487275	EEF1B2;RPS8;RPL35A;RPSA
Lomustine-560_uM_in_DMSO-Rat-Primary_rat_hepatocytes-0.67d-dn	4/277	0,575284274	0,76458639	0	0	0,992469637	0,5487275	CXCL11;SEPP1;SLC16A3;TPT1
Phenacemide-400_mg/kg_in_CMC-Rat-Heart-3d-up	4/277	0,575284274	0,764457389	0	0	0,992469637	0,5487275	EEF1B2;CLP1;RAPGEF5;TPT1
Sulfisoxazole-2500_mg/kg_in_Corn_Oil-Rat-Liver-1d-up	4/277	0,575284274	0,764328432	0	0	0,992469637	0,5487275	CLP1;RPS3A;RPL39;SEPP1
Sulfaphenazole-1695_mg/kg_in_Water-Rat-Kidney-5d-up	5/349	0,576069224	0,765242235	0	0	0,984649317	0,543061122	EEF1B2;RPS8;RAPGEF5;SEPP1;TPT1
Amlodipine-19_mg/kg_in_Corn_Oil-Rat-Liver-5d-dn	4/278	0,578134656	0,767856416	0	0	0,988899602	0,541866023	CLP1;RPS6;RPL39;SEPP1
Busulfan-9_mg/kg_in_Corn_Oil-Rat-Bone_marrow-1d-dn	4/278	0,578134656	0,767726951	0	0	0,988899602	0,541866023	RPS8;RPS6;ARL6IP5;NKG7
Capsaicin-35_mg/kg_in_CMC-Rat-Liver-3d-up	4/278	0,578134656	0,76759753	0	0	0,988899602	0,541866023	RPS17;RPS3A;RPL39;SEPP1
Chlorobenzene-500_mg/kg_in_CMC-Rat-Heart-5d-dn	4/278	0,578134656	0,767468152	0	0	0,988899602	0,541866023	COL5A3;RPS6;SEPP1;TPT1
Etoposide-188_mg/kg_in_Corn_Oil-Rat-Liver-1d-dn	4/278	0,578134656	0,767338818	0	0	0,988899602	0,541866023	EEF1B2;RPS19;RPSA;SEPP1
Fenofibrate-43_mg/kg_in_Corn_Oil-Rat-Liver-3d-up	4/278	0,578134656	0,767209528	0	0	0,988899602	0,541866023	RPS17;RPL35A;RPS20;RPS3A
Pravastatin-1200_mg/kg_in_Corn_Oil-Rat-Kidney-1d-dn	4/278	0,578134656	0,767080281	0	0	0,988899602	0,541866023	RPS19;RPS6;RPL10A;RPL39
Sparteine-33_mg/kg_in_Water-Rat-Heart-1d-dn	4/278	0,578134656	0,766951078	0	0	0,988899602	0,541866023	EEF1B2;RPS19;ARL6IP5;RPL10A

Zopiclone-414_mg/kg_in_Water-Rat-Liver-3d-dn	4/278	0,578134656	0,766821918	0	0	0,988899602	0,541866023	EEF1B2;C1QA;SEPP1;TPT1
Ascorbic_Acid-2000_mg/kg_in_Water-Rat-Kidney-1d-up	4/279	0,5809742	0,770458461	0	0	0,985355159	0,535096064	RPS19;RPS8;RPS6;RPL10A
Benazepril-1750_mg/kg_in_CMC-Rat-Kidney-1d-dn	4/279	0,5809742	0,770328754	0	0	0,985355159	0,535096064	RPS8;RPSA;RPS3A;SEPP1
Camptothecin-2.5_mg/kg_in_Saline-Rat-Heart-5d-up	4/279	0,5809742	0,770199091	0	0	0,985355159	0,535096064	RPS8;HOXA1;RPSA;TPT1
Capsaicin-35_mg/kg_in_CMC-Rat-Liver-1d-up	4/279	0,5809742	0,770069471	0	0	0,985355159	0,535096064	RPS17;ARL2BP;RPS20;RPL39
Clarithromycin-56_mg/kg_in_Water-Rat-Liver-5d-dn	4/279	0,5809742	0,769939895	0	0	0,985355159	0,535096064	C1QA;RPSA;RPL39;TPT1
Leflunomide-30_mg/kg_in_Corn_Oil-Rat-Liver-1d-dn	4/279	0,5809742	0,769810363	0	0	0,985355159	0,535096064	C1QA;RPS8;RPS6;SEPP1
Oxymetholone-1170_mg/kg_in_CMC-Rat-Liver-1d-dn	4/279	0,5809742	0,769680874	0	0	0,985355159	0,535096064	C1QA;RPS19;CLP1;SEPP1
Oxytetracycline-1500_mg/kg_in_Water-Rat-Liver-3d-dn	4/279	0,5809742	0,769551429	0	0	0,985355159	0,535096064	C1QA;RPL39;SEPP1;TPT1
Procaine-2500_mg/kg_in_Water-Rat-Heart-5d-up	4/279	0,5809742	0,769422027	0	0	0,985355159	0,535096064	C1QA;RPS19;SEPP1;TPT1
Methotrexate-27_mg/kg_in_Corn_Oil-Rat-Kidney-1d-dn	3/207	0,582280274	0,771022098	0	0	0,996065541	0,538675608	EEF1B2;RPS19;SEPP1
Sulfadiazine-1170_mg/kg_in_Water-Rat-Kidney-1d-up	5/352	0,583678547	0,772743695	0	0	0,97625742	0,525621759	EEF1B2;CLP1;RPS8;SEPP1;TPT1
3-Methylcholanthrene-12.5_mg/kg_in_CMC-Rat-Liver-28d-up	4/280	0,583802835	0,772778342	0	0	0,981836033	0,528416263	EEF1B2;CLP1;RPSA;SEPP1
3-Methylcholanthrene-300_mg/kg_in_CMC-Rat-Liver-5d-up	4/280	0,583802835	0,772648485	0	0	0,981836033	0,528416263	RPS17;RPSA;RPS3A;SEPP1
Azithromycin-50_mg/kg_in_Water-Rat-Liver-1d-dn	4/280	0,583802835	0,772518671	0	0	0,981836033	0,528416263	RPS17;RPS8;RPSA;TPT1
Cyclosporin_A-70_mg/kg_in_Corn_Oil-Rat-Bone_marrow-3d-dn	4/280	0,583802835	0,772388902	0	0	0,981836033	0,528416263	RPS17;RPS19;FEM1B;ARL6IP5
Econazole-43_mg/kg_in_Corn_Oil-Rat-Liver-3d-up	4/280	0,583802835	0,772259176	0	0	0,981836033	0,528416263	C1QA;RPS20;RPL39;SEPP1
Kanamycin-6584_uM_in_DMSO-Rat-Primary_rat_hepatocytes-0.67d-dn	4/280	0,583802835	0,772129493	0	0	0,981836033	0,528416263	C1QA;RPS19;RPS8;ARL6IP5
Nateglinide-625_mg/kg_in_CMC-Rat-Liver-1d-dn	4/280	0,583802835	0,771999854	0	0	0,981836033	0,528416263	EEF1B2;C1QA;RPS19;CLP1
Norethindrone-125_uM_in_DMSO-Rat-Primary_rat_hepatocytes-1d-dn	4/280	0,583802835	0,771870259	0	0	0,981836033	0,528416263	RPS19;RPS20;RPL39;TPT1
Phenobarbital-80_mg/kg_in_Water-Rat-Liver-5d-up	4/280	0,583802835	0,771740707	0	0	0,981836033	0,528416263	RPS8;RPS3A;RPL10A;SEPP1
Piclamilast-17_mg/kg_in_CMC-Rat-Liver-1d-up	4/280	0,583802835	0,771611199	0	0	0,981836033	0,528416263	RPS8;RPS20;RPL39;TPT1
Procainamide-755_mg/kg_in_Water-Rat-Heart-5d-up	4/280	0,583802835	0,771481734	0	0	0,981836033	0,528416263	FEM1B;RPL10A;GOLGA7;SEPP1
Racepinephrine-0.77_mg/kg_in_Saline-Rat-Heart-3d-up	4/280	0,583802835	0,771352312	0	0	0,981836033	0,528416263	CXCL11;RPS8;FUNDC1;RAPGEF5
Emetine-1_mg/kg_in_Saline-Rat-Kidney-1d-dn	5/353	0,586198738	0,774388	0	0	0,973491818	0,51993848	RPL32;RPS6;RPS3A;SEPP1;TPT1
Azlocillin-800_mg/kg_in_Saline-Rat-Kidney-3d-dn	4/281	0,586620493	0,774815194	0	0	0,978341955	0,521825281	EEF1B2;CLP1;RPS20;TPT1
Cetylpyridinium_Bromide-119_mg/kg_in_Water-Rat-Liver-3d-dn	4/281	0,586620493	0,774685279	0	0	0,978341955	0,521825281	EEF1B2;RPS19;CLP1;SEPP1
Cytarabine-23_mg/kg_in_Saline-Rat-Bone_marrow-1d-up	4/281	0,586620493	0,774555407	0	0	0,978341955	0,521825281	RPS19;RPS6;RPS20;GOLGA7
Diazepam-710_mg/kg_in_CMC-Rat-Kidney-5d-dn	4/281	0,586620493	0,774425579	0	0	0,978341955	0,521825281	ARL2BP;RPS6;GOLGA7;SEPP1
Finasteride-800_mg/kg_in_Corn_Oil-Rat-Kidney-1d-dn	4/281	0,586620493	0,774295794	0	0	0,978341955	0,521825281	RPL32;RPS19;GOLGA7;TPT1
Gentamicin-40_mg/kg_in_Saline-Rat-Kidney-5d-dn	4/281	0,586620493	0,774166053	0	0	0,978341955	0,521825281	EEF1B2;RPS17;RPS19;ARL2BP
Isoprenaline-15_mg/kg_in_Saline-Rat-Liver-1d-dn	4/281	0,586620493	0,774036355	0	0	0,978341955	0,521825281	FEM1B;RPS3A;RPL10A;TPT1
Letrozole-250_mg/kg_in_Corn_Oil-Rat-Liver-5d-dn	4/281	0,586620493	0,773906701	0	0	0,978341955	0,521825281	EEF1B2;CLP1;RPS8;SEPP1
NN-Dimethylformamide-140_mg/kg_in_Saline-Rat-Liver-5d-dn	4/281	0,586620493	0,77377709	0	0	0,978341955	0,521825281	C1QA;RPS19;RPS8;GOLGA7
Nevirapine-250_uM_in_DMSO-Rat-Primary_rat_hepatocytes-1d-up	4/281	0,586620493	0,773647522	0	0	0,978341955	0,521825281	RPS5;ARL6IP5;RPS3A;SLC16A3
33'4'5-Tetrachlorosalicylanilide-50_mg/kg_in_Corn_Oil-Rat-Liver-3d-dn	4/282	0,589427105	0,777218798	0	0	0,974872657	0,515321803	C1QA;RPSA;RPL10A;TPT1
4-Methylpyrazole-155_mg/kg_in_Saline-Rat-Liver-5d-dn	4/282	0,589427105	0,777088697	0	0	0,974872657	0,515321803	EEF1B2;CLP1;SEPP1;TPT1
Amitriptyline-160_mg/kg_in_Water-Rat-Heart-5d-dn	4/282	0,589427105	0,776958641	0	0	0,974872657	0,515321803	EEF1B2;RPS19;RPS8;RPSA
Bromhexine-1200_mg/kg_in_CMC-Rat-Liver-1d-dn	4/282	0,589427105	0,776828628	0	0	0,974872657	0,515321803	EEF1B2;RPS20;RPL39;SEPP1
Fenofibrate-215_mg/kg_in_Corn_Oil-Rat-Liver-3d-dn	4/282	0,589427105	0,776698658	0	0	0,974872657	0,515321803	C1QA;CLP1;SEPP1;TPT1
Gadopentetate_Dimeglumine-125_mg/kg_in_Saline-Rat-Kidney-1d-dn	4/282	0,589427105	0,776568732	0	0	0,974872657	0,515321803	RPL10A;RPL39;SEPP1;TPT1
Hydroxyurea-59_mg/kg_in_Saline-Rat-Spleen-0.25d-up	4/282	0,589427105	0,776438849	0	0	0,974872657	0,515321803	RPS17;RPS8;NKG7;RPL35A
Isotretinoin-500_uM_in_DMSO-Rat-Primary_rat_hepatocytes-0.67d-dn	4/282	0,589427105	0,77630901	0	0	0,974872657	0,515321803	RPS17;RPL32;RPS3A;PDRG1
Lamotrigine-26_mg/kg_in_CMC-Rat-Heart-1d-up	4/282	0,589427105	0,776179214	0	0	0,974872657	0,515321803	CXCL11;RPS6;ARL6IP5;RAPGEF5
NN-Dimethylformamide-1400_mg/kg_in_Saline-Rat-Liver-0.25d-dn	4/282	0,589427105	0,776049461	0	0	0,974872657	0,515321803	RPS17;RPS19;RPS20;SEPP1
Sulfathiazole-2629_mg/kg_in_Water-Rat-Liver-1d-dn	4/282	0,589427105	0,775919752	0	0	0,974872657	0,515321803	EEF1B2;C1QA;CLP1;SEPP1
Thioacetamide-200_mg/kg_in_Saline-Rat-Liver-5d-dn	4/282	0,589427105	0,775790087	0	0	0,974872657	0,515321803	RPS17;RPL32;RPS6;RPL10A
Tocainide-224_mg/kg_in_Corn_Oil-Rat-Liver-5d-dn	4/282	0,589427105	0,775660464	0	0	0,974872657	0,515321803	C1QA;RPS17;RPS6;RPL10A
Tolazamide-1500_mg/kg_in_CMC-Rat-Heart-1d-up	4/282	0,589427105	0,775530885	0	0	0,974872657	0,515321803	EEF1B2;RPS17;RPS8;RPSA
Valproic_Acid-1340_mg/kg_in_Water-Rat-Liver-5d-dn	4/282	0,589427105	0,775401349	0	0	0,974872657	0,515321803	RPS19;RPS8;RPSA;SEPP1
Enoxacin-100_mg/kg_in_Corn_Oil-Rat-Liver-5d-dn	5/355	0,591214396	0,777622675	0	0	0,968007357	0,508761977	C1QA;RPL32;RPS6;RPS3A;RPL10A
Amantadine-58_mg/kg_in_Water-Rat-Liver-1d-dn	4/283	0,592222605	0,778818707	0	0	0,971427878	0,508904538	ARL2BP;RPS8;FEM1B;FUNDC1
Azasetron-580_mg/kg_in_CMC-Rat-Intestine-3d-up	4/283	0,592222605	0,778688688	0	0	0,971427878	0,508904538	RPL32;RPS6;RPL10A;SLC16A3
Carboplatin-6_mg/kg_in_Saline-Rat-Spleen-1d-up	4/283	0,592222605	0,778558711	0	0	0,971427878	0,508904538	RPL10A;RPL39;SEPP1;TPT1
Clofibric_Acid-448_mg/kg_in_Corn_Oil-Rat-Heart-3d-dn	4/283	0,592222605	0,778428778	0	0	0,971427878	0,508904538	FUNDC1;RPS6;RPS20;SEPP1
Diazepam-710_mg/kg_in_CMC-Rat-Liver-5d-dn	4/283	0,592222605	0,778298888	0	0	0,971427878	0,508904538	EEF1B2;C1QA;RPL32;SEPP1
Dichlorvos-17_mg/kg_in_Water-Rat-Heart-5d-up	4/283	0,592222605	0,778169042	0	0	0,971427878	0,508904538	ARL2BP;CLP1;FUNDC1;RAPGEF5
Ethylene_Glycol-352_mg/kg_in_Water-Rat-Liver-5d-up	4/283	0,592222605	0,778039239	0	0	0,971427878	0,508904538	RPS8;RPS5;RPL35A;RPS20
Mitomycin_C-1.7_mg/kg_in_Saline-Rat-Bone_marrow-5d-up	4/283	0,592222605	0,777909479	0	0	0,971427878	0,508904538	C1QA;ARL2BP;NKG7;RPS3A
NN-Dimethylformamide-65000_uM_in_DMSO-Rat-Primary_rat_hepatocytes-0.67d-dn	4/283	0,592222605	0,777779763	0	0	0,971427878	0,508904538	EEF1B2;RPS8;RPS5;RPS20
Nimesulide-30_mg/kg_in_Corn_Oil-Rat-Kidney-0.25d-dn	4/283	0,592222605	0,77765009	0	0	0,971427878	0,508904538	RPS8;RPS5;ARL6IP5;TOMM20
Omeprazole-415_mg/kg_in_Corn_Oil-Rat-Liver-3d-dn	4/283	0,592222605	0,77752046	0	0	0,971427878	0,508904538	C1QA;RPS17;RPS6;RPS3A
Oxcarbazepine-1600_mg/kg_in_CMC-Rat-Kidney-3d-up	4/283	0,592222605	0,777390873	0	0	0,971427878	0,508904538	EEF1B2;RPS17;RPS3A;SEPP1
Pyrazinamide-1500_mg/kg_in_CMC-Rat-Liver-1d-dn	4/283	0,592222605	0,77726133	0	0	0,971427878	0,508904538	RPSA;RPL39;GOLGA7;SEPP1
Sodium_Arsenite-30_mg/kg_in_Saline-Rat-Kidney-1d-dn	4/283	0,592222605	0,777131829	0	0	0,971427878	0,508904538	ARL2BP;RPL10A;RPL39;TPT1
Aflatoxin_B1-0.3_mg/kg_in_CMC-Rat-Liver-1d-dn	4/284	0,59500693	0,780655436	0	0	0,968007357	0,502572215	C1QA;CLP1;RPS6;RPL10A
Amoxapine-313_mg/kg_in_CMC-Rat-Liver-5d-dn	4/284	0,59500693	0,780525413	0	0	0,968007357	0,502572215	EEF1B2;RPL10A;RPL39;SEPP1
Ketoprofen-4_mg/kg_in_Corn_Oil-Rat-Kidney-5d-dn	4/284	0,59500693	0,780395434	0	0	0,968007357	0,502572215	ARL2BP;CLP1;SEPP1;TPT1
Omeprazole-30_mg/kg_in_Corn_Oil-Rat-Liver-5d-dn	4/284	0,59500693	0,780265498	0	0	0,968007357	0,502572215	RPS6;RPSA;RPL39;SEPP1
Phenacemide-400_mg/kg_in_CMC-Rat-Kidney-5d-dn	4/284	0,59500693	0,780135605	0	0	0,968007357	0,502572215	ARL2BP;CLP1;RPL39;TPT1
Quetiapine-500_mg/kg_in_CMC-Rat-Liver-1d-up	4/284	0,59500693	0,780005756	0	0	0,968007357	0,502572215	RPSA;RPS3A;RPL39;TPT1
Acetazolamide-47_mg/kg_in_Corn_Oil-Rat-Kidney-3d-dn	4/285	0,597780017	0,783510637	0	0	0,96461084	0,496323585	CLP1;RPL39;SEPP1;TPT1
Azaribine-750_mg/kg_in_CMC-Rat-Heart-3d-up	4/285	0,597780017	0,783380269	0	0	0,96461084	0,496323585	EEF1B2;RPL32;CLP1;RPS8
Carvedilol-2000_mg/kg_in_Corn_Oil-Rat-Liver-1d-dn	4/285	0,597780017	0,783249945	0	0	0,96461084	0,496323585	C1QA;RPS17;RPS8;RPS3A
Chlormadinone_Acetate-2000_mg/kg_in_CMC-Rat-Kidney-1d-dn	4/285	0,597780017	0,783119664	0	0	0,96461084	0,496323585	RPS17;RPL35A;SEPP1;TPT1
Furan-24_mg/kg_in_Corn_Oil-Rat-Heart-3d-up	4/285	0,597780017	0,782989426	0	0	0,96461084	0,496323585	ARL6IP5;RPL10A;SEPP1;PDRG1
Ifosfamide-17_mg/kg_in_Saline-Rat-Bone_marrow-0.25d-up	4/285	0,597780017	0,782859231	0	0	0,96461084	0,496323585	RPS19;RPS5;RPS20;RPSA
Loratadine-10_mg/kg_in_Water-Rat-Heart-3d-dn	4/285	0,597780017	0,78272908	0	0	0,96461084	0,496323585	R3HCC1;RPL39;SEPP1;TPT1
Mosapride-30_mg/kg_in_CMC-Rat-Intestine-3d-up	4/285	0,597780017	0,782598972	0	0	0,96461084	0,496323585	C1QA;RPS17;RPL32;RPS20
NN-Dimethylformamide-140_mg/kg_in_Saline-Rat-Kidney-1d-dn	4/285	0,597780017	0,782468908	0	0	0,96461084	0,496323585	EEF1B2;CLP1;FEM1B;RPL10A
Nateglinide-625_mg/kg_in_CMC-Rat-Liver-3d-dn	4/285	0,597780017	0,782338886	0	0	0,96461084	0,496323585	CLP1;RPS8;GOLGA7;TPT1
Teicoplanin-41_mg/kg_in_Saline-Rat-Bone_marrow-3d-up	4/285	0,597780017	0,782208908	0	0	0,96461084	0,496323585	EEF1B2;CLP1;RPS3A;RPL10A
Cyclosporin_A-350_mg/kg_in_Corn_Oil-Rat-Kidney-1d-dn	3/212	0,59847363	0,78298643	0	0	0,972573429	0,49929276	ARL2BP;CLP1;SEPP1
Benzocaine-1100_mg/kg_in_Corn_Oil-Rat-Liver-3d-dn	4/286	0,600541807	0,785561746	0	0	0,961238075	0,490157421	C1QA;RPS17;RPS3A;TPT1
Benzothiazyl_Disulfide-2600_mg/kg_in_Corn_Oil-Rat-Liver-1d-dn	4/286	0,600541807	0,785431298	0	0	0,961238075	0,490157421	EEF1B2;RPS20;GOLGA7;SEPP1
Carmustine-4_mg/kg_in_Corn_Oil-Rat-Liver-1d-dn	4/286	0,600541807	0,785300892	0	0	0,961238075	0,490157421	EEF1B2;RPS3A;RPL39;SEPP1
Diphenhydramine-100_mg/kg_in_Water-Rat-Heart-5d-dn	4/286	0,600541807	0,78517053	0	0	0,961238075	0,490157421	EEF1B2;ARL2BP;R3HCC1;RPS20
Ergocalciferol-15_mg/kg_in_CMC-Rat-Heart-1d-up	4/286	0,600541807	0,785040212	0	0	0,961238075	0,490157421	EEF1B2;RPS17;RPL32;RPSA
Etoposide-188_mg/kg_in_Corn_Oil-Rat-Bone_marrow-3d-dn	4/286	0,600541807	0,784909936	0	0	0,961238075	0,490157421	RPS19;FEM1B;RPS6;TPT1
Furosemide-375_mg/kg_in_CMC-Rat-Kidney-1d-dn	4/286	0,600541807	0,784779704	0	0	0,961238075	0,490157421	RPSA;RPL39;GOLGA7;SEPP1

Levodopa-1000_mg/kg_in_Water-Rat-Brain-3d-up	4/286	0,600541807	0,784649515	0	0	0,961238075	0,490157421	RPS17;RPS3A;RPL10A;TPT1
NN-Dimethylformamide-65000_uM_in_DMSO-Rat-Primary_rat_hepatocytes-1d-dn	4/286	0,600541807	0,784519369	0	0	0,961238075	0,490157421	RPS19;RPS20;RPS3A;TPT1
Nitrazepam-310_mg/kg_in_CMC-Rat-Liver-1d-up	4/286	0,600541807	0,784389266	0	0	0,961238075	0,490157421	RPS17;CXCL11;RPS8;RPS6
Zaleplon-100_mg/kg_in_Water-Rat-Liver-1d-dn	4/286	0,600541807	0,784259207	0	0	0,961238075	0,490157421	EEF1B2;RPS17;RPS19;SEPP1
Zomepirac-2.8_mg/kg_in_Saline-Rat-Liver-5d-dn	4/286	0,600541807	0,78412919	0	0	0,961238075	0,490157421	RPS6;RPS20;RPL10A;PDRG1
Atorvastatin-300_mg/kg_in_Corn_Oil-Rat-Liver-0.25d-dn	3/213	0,601662845	0,785462716	0	0	0,968007357	0,491803929	RPL32;SEPP1;TPT1
Azlocillin-800_mg/kg_in_Saline-Rat-Kidney-1d-dn	3/213	0,601662845	0,785332543	0	0	0,968007357	0,491803929	EEF1B2;CLP1;TPT1
Acetazolamide-250_mg/kg_in_Corn_Oil-Rat-Liver-0.25d-dn	4/287	0,603292241	0,787328864	0	0	0,957888813	0,484072516	C1QA;RPS17;RPL39;TPT1
Amiodarone-147_mg/kg_in_CMC-Rat-Heart-3d-dn	4/287	0,603292241	0,787198425	0	0	0,957888813	0,484072516	ARL6IP5;RPSA;RPL39;SEPP1
Clarithromycin-476_mg/kg_in_Water-Rat-Liver-3d-dn	4/287	0,603292241	0,787068029	0	0	0,957888813	0,484072516	C1QA;RPSA;RPL39;SEPP1
Cyclosporin_A-350_mg/kg_in_Corn_Oil-Rat-Heart-3d-dn	4/287	0,603292241	0,786937677	0	0	0,957888813	0,484072516	RPS17;RPL32;RPS19;RPS3A
Flavoxate-1255_mg/kg_in_CMC-Rat-Heart-1d-up	4/287	0,603292241	0,786807367	0	0	0,957888813	0,484072516	RPS17;RPL35A;RPS20;RPSA
Galantamine-110_mg/kg_in_CMC-Rat-Heart-3d-up	4/287	0,603292241	0,786677101	0	0	0,957888813	0,484072516	RPS17;RPL32;RPS6;SEPP1
Gatifloxacin-770_mg/kg_in_Corn_Oil-Rat-Kidney-3d-dn	4/287	0,603292241	0,786546878	0	0	0,957888813	0,484072516	EEF1B2;CLP1;RPL39;SEPP1
Hydralazine-280_mg/kg_in_Water-Rat-Heart-3d-up	4/287	0,603292241	0,786416698	0	0	0,957888813	0,484072516	ARL2BP;RPS8;FUNDC1;SEPP1
Ifosfamide-17_mg/kg_in_Saline-Rat-Spleen-0.25d-dn	4/287	0,603292241	0,786286562	0	0	0,957888813	0,484072516	FEM1B;RPS6;RPS20;TPT1
Ouabain-144_mg/kg_in_CMC-Rat-Heart-3d-dn	4/287	0,603292241	0,786156468	0	0	0,957888813	0,484072516	ARL2BP;RPS8;SEPP1;TPT1
Procarbazine-54_mg/kg_in_Water-Rat-Bone_marrow-5d-dn	4/287	0,603292241	0,786026417	0	0	0,957888813	0,484072516	FEM1B;RPS6;RPL39;SLC16A3
Tacrine-24_mg/kg_in_Water-Rat-Liver-1d-up	4/287	0,603292241	0,78589641	0	0	0,957888813	0,484072516	EEF1B2;RPS20;GOLGA7;SEPP1
Zidovudine-1540_mg/kg_in_Corn_Oil-Rat-Liver-3d-dn	4/287	0,603292241	0,785766445	0	0	0,957888813	0,484072516	EEF1B2;RPS17;RPS19;RPSA
2378-Tetrachlorodibenzo-P-Dioxin-0.0007_mg/kg_in_CMC-Rat-Liver-28d-up	4/288	0,606031263	0,78920341	0	0	0,95456281	0,478067684	EEF1B2;CLP1;RPSA;SEPP1
Azithromycin-50_mg/kg_in_Water-Rat-Liver-5d-dn	4/288	0,606031263	0,789072942	0	0	0,95456281	0,478067684	RPS19;RPS8;RPS20;RPSA
Benazepril-1750_mg/kg_in_CMC-Rat-Kidney-5d-dn	4/288	0,606031263	0,788942516	0	0	0,95456281	0,478067684	ARL2BP;RPS8;RPS3A;SEPP1
Camptothecin-5_mg/kg_in_Saline-Rat-Kidney-5d-up	4/288	0,606031263	0,788812134	0	0	0,95456281	0,478067684	CLP1;HOXA1;RPSA;SEPP1
Carbon_Tetrachloride-1175_mg/kg_in_Corn_Oil-Rat-Bone_marrow-3d-dn	4/288	0,606031263	0,788681795	0	0	0,95456281	0,478067684	EEF1B2;RPS17;RPS19;RPSA
Clofibric_Acid-448_mg/kg_in_Corn_Oil-Rat-Heart-5d-up	4/288	0,606031263	0,788551499	0	0	0,95456281	0,478067684	RPS19;CLP1;RPS8;RPS20
D-Tubocurarine_Chloride-0.033_mg/kg_in_Saline-Rat-Heart-5d-dn	4/288	0,606031263	0,788421246	0	0	0,95456281	0,478067684	EEF1B2;ARL2BP;ARL6IP5;RAPGEF5
Daunorubicin-3.25_mg/kg_in_Saline-Rat-Heart-1d-up	4/288	0,606031263	0,788291036	0	0	0,95456281	0,478067684	CXCL11;RPS19;GOLGA7;SEPP1
Erythromycin-1500_mg/kg_in_CMC-Rat-Liver-3d-dn	4/288	0,606031263	0,788160869	0	0	0,95456281	0,478067684	C1QA;RPS17;CLP1;SEPP1
Ethylestrenol-390_mg/kg_in_CMC-Rat-Liver-5d-up	4/288	0,606031263	0,788030745	0	0	0,95456281	0,478067684	RPSA;RPS3A;GOLGA7;SEPP1
Flubendazole-1200_mg/kg_in_Corn_Oil-Rat-Liver-5d-dn	4/288	0,606031263	0,787900664	0	0	0,95456281	0,478067684	EEF1B2;CLP1;RPS6;SEPP1
Griseofulvin-2500_mg/kg_in_Corn_Oil-Rat-Liver-5d-up	4/288	0,606031263	0,787770626	0	0	0,95456281	0,478067684	RPS19;ARL6IP5;RPSA;TPT1
Hydrocortisone-56_mg/kg_in_Corn_Oil-Rat-Kidney-3d-dn	4/288	0,606031263	0,787640631	0	0	0,95456281	0,478067684	ARL2BP;RPS3A;RPL39;TPT1
Indomethacin-4.5_mg/kg_in_Water-Rat-Liver-5d-dn	4/288	0,606031263	0,787510679	0	0	0,95456281	0,478067684	RPS19;RPS8;RPSA;SEPP1
Isoeugenol-1560_mg/kg_in_CMC-Rat-Liver-5d-up	4/288	0,606031263	0,787380769	0	0	0,95456281	0,478067684	RPS19;RPL39;GOLGA7;SEPP1
Propylthiouracil-625_mg/kg_in_CMC-Rat-Liver-5d-up	4/288	0,606031263	0,787250903	0	0	0,95456281	0,478067684	RPS17;RPS20;RPSA;SEPP1
Salicylamide-1300_mg/kg_in_CMC-Rat-Kidney-5d-up	4/288	0,606031263	0,787121079	0	0	0,95456281	0,478067684	CLP1;RPS3A;RAPGEF5;TPT1
Sodium_Orthovanadate-83_mg/kg_in_CMC-Rat-Kidney-5d-dn	4/288	0,606031263	0,786991298	0	0	0,95456281	0,478067684	RPS17;ARL2BP;RPS3A;SEPP1
Sulfadimethoxine-1100_mg/kg_in_CMC-Rat-Kidney-3d-dn	4/288	0,606031263	0,78686156	0	0	0,95456281	0,478067684	RPL32;RPS19;RPL35A;RPSA
Valproic_Acid-850_mg/kg_in_Water-Rat-Liver-1d-dn	4/288	0,606031263	0,786731865	0	0	0,95456281	0,478067684	CLP1;RPS8;RPS20;TPT1
TGF_Beta-1_Human_Recombinant-5_ng/ml_in_DMSO-Rat-Primary_rat_hepatocytes-1d-up	3/215	0,60799133	0,789146295	0	0	0,959002637	0,477194588	EEF1B2;RPS3A;SEPP1
Cinnarizine-750_mg/kg_in_CMC-Rat-Liver-3d-dn	4/289	0,608758817	0,790012266	0	0	0,951259825	0,472141758	EEF1B2;C1QA;SEPP1;TPT1
Cisplatin-0.5_mg/kg_in_Saline-Rat-Kidney-14d-dn	4/289	0,608758817	0,789882116	0	0	0,951259825	0,472141758	EEF1B2;RPS17;SEPP1;TOMM20
Danazol-95_uM_in_DMSO-Rat-Primary_rat_hepatocytes-0.67d-up	4/289	0,608758817	0,789752008	0	0	0,951259825	0,472141758	RPS17;RPS6;TPT1;PDRG1
Fenbendazole-375_mg/kg_in_Corn_Oil-Rat-Liver-1d-dn	4/289	0,608758817	0,789621944	0	0	0,951259825	0,472141758	EEF1B2;RPS17;RPL32;RPS8
Fluphenazine-22_mg/kg_in_Corn_Oil-Rat-Heart-3d-dn	4/289	0,608758817	0,789491922	0	0	0,951259825	0,472141758	RPS17;RPL32;ARL2BP;RPS20
Oxyquinoline-68_mg/kg_in_Corn_Oil-Rat-Liver-1d-dn	4/289	0,608758817	0,789361943	0	0	0,951259825	0,472141758	EEF1B2;FEM1B;RPL10A;SEPP1
Tobramycin-40_mg/kg_in_Saline-Rat-Kidney-28d-dn	4/289	0,608758817	0,789232007	0	0	0,951259825	0,472141758	CLP1;RPS6;RAPGEF5;SEPP1
2-Acetylaminofluorene-30_mg/kg_in_CMC-Rat-Kidney-1d-up	4/290	0,611474851	0,792622766	0	0	0,947979618	0,466293591	RPS17;CLP1;RPS8;RPS3A
2378-Tetrachlorodibenzo-P-Dioxin-0.0007_mg/kg_in_CMC-Rat-Liver-5d-dn	4/290	0,611474851	0,792492336	0	0	0,947979618	0,466293591	RPL32;RPS6;RPS20;RPL39
4-Nonylphenol-200_mg/kg_in_Corn_Oil-Rat-Liver-3d-dn	4/290	0,611474851	0,792361949	0	0	0,947979618	0,466293591	C1QA;RPL10A;RPL39;TPT1
Diazepam-710_mg/kg_in_CMC-Rat-Liver-1d-up	4/290	0,611474851	0,792231605	0	0	0,947979618	0,466293591	RPS17;RPS3A;RPL39;TPT1
Etoposide-100_mg/kg_in_Corn_Oil-Rat-Liver-0.25d-dn	4/290	0,611474851	0,792101303	0	0	0,947979618	0,466293591	C1QA;RPL35A;RPL39;TPT1
Mebendazole-50_mg/kg_in_Corn_Oil-Rat-Liver-0.25d-dn	4/290	0,611474851	0,791971045	0	0	0,947979618	0,466293591	RPS17;RPS20;RPS3A;RPL39
Neomycin-877_mg/kg_in_Corn_Oil-Rat-Liver-3d-dn	4/290	0,611474851	0,791840829	0	0	0,947979618	0,466293591	EEF1B2;RPS6;RPL10A;SEPP1
Noscapine-570_mg/kg_in_CMC-Rat-Spleen-1d-dn	4/290	0,611474851	0,791710657	0	0	0,947979618	0,466293591	C1QA;RPS17;RPS6;RPS3A
Phenothiazine-386_mg/kg_in_Corn_Oil-Rat-Liver-5d-up	4/290	0,611474851	0,791580527	0	0	0,947979618	0,466293591	EEF1B2;RPL32;RPL39;TPT1
Testosterone-375_mg/kg_in_CMC-Rat-Liver-3d-dn	4/290	0,611474851	0,79145044	0	0	0,947979618	0,466293591	C1QA;RPS19;FEM1B;RPL39
Tolazamide-1500_mg/kg_in_CMC-Rat-Liver-5d-dn	4/290	0,611474851	0,791320395	0	0	0,947979618	0,466293591	C1QA;RPL32;RPL39;TPT1
Trichloroacetic_Acid-474_mg/kg_in_Corn_Oil-Rat-Liver-3d-up	4/290	0,611474851	0,791190393	0	0	0,947979618	0,466293591	EEF1B2;RAPGEF5;RPL10A;RPL39
Zileuton-450_mg/kg_in_Corn_Oil-Rat-Kidney-1d-dn	4/290	0,611474851	0,791060434	0	0	0,947979618	0,466293591	ARL2BP;CLP1;RPL35A;SEPP1
Allyl_Alcohol-16_mg/kg_in_Saline-Rat-Bone_marrow-1d-up	5/364	0,613366413	0,793377216	0	0	0,944073109	0,461456124	RPS19;NKG7;RPL10A;RPL39;GOLGA7
Ofloxacin-3000_mg/kg_in_Water-Rat-Kidney-5d-up	5/364	0,613366413	0,79324694	0	0	0,944073109	0,461456124	CLP1;RPS8;SEPP1;TOMM20;TPT1
4-Octylphenol-450_mg/kg_in_Corn_Oil-Rat-Liver-5d-dn	4/291	0,614179313	0,794167833	0	0	0,944721957	0,460522055	EEF1B2;RPL32;RPSA;SEPP1
Bisacodyl-1750_mg/kg_in_CMC-Rat-Intestine-5d-up	4/291	0,614179313	0,794037471	0	0	0,944721957	0,460522055	RPL32;RPS20;RPL10A;TPT1
Bromisovalum-250_mg/kg_in_Corn_Oil-Rat-Heart-1d-up	4/291	0,614179313	0,793907151	0	0	0,944721957	0,460522055	RPS19;CLP1;RPSA;SEPP1
Chlorobenzene-500_mg/kg_in_CMC-Rat-Kidney-5d-dn	4/291	0,614179313	0,793776874	0	0	0,944721957	0,460522055	RPL32;ARL2BP;RPS6;SEPP1
Ciprofloxacin-450_mg/kg_in_Corn_Oil-Rat-Liver-0.25d-dn	4/291	0,614179313	0,79364664	0	0	0,944721957	0,460522055	RPS17;RPSA;RPL10A;TPT1
Diazepam-710_mg/kg_in_CMC-Rat-Heart-1d-up	4/291	0,614179313	0,793516449	0	0	0,944721957	0,460522055	EEF1B2;RPS17;RPSA;RAPGEF5
Doxazosin-5_mg/kg_in_Water-Rat-Heart-0.25d-dn	4/291	0,614179313	0,7933863	0	0	0,944721957	0,460522055	FUNDC1;RPS20;RAPGEF5;SEPP1
Fluocinolone_Acetonide-2.5_mg/kg_in_Corn_Oil-Rat-Heart-5d-up	4/291	0,614179313	0,793256194	0	0	0,944721957	0,460522055	C1QA;RAPGEF5;SEPP1;TPT1
Gentamicin-40_mg/kg_in_Saline-Rat-Kidney-14d-dn	4/291	0,614179313	0,793126131	0	0	0,944721957	0,460522055	ARL2BP;CLP1;RPSA;SEPP1
Hexachloro-13-Butadiene-50_mg/kg_in_Corn_Oil-Rat-Kidney-1d-dn	4/291	0,614179313	0,79299611	0	0	0,944721957	0,460522055	RPS19;RPS6;RPL39;SEPP1
Ibuprofen-263_mg/kg_in_Corn_Oil-Rat-Liver-5d-dn	4/291	0,614179313	0,792866132	0	0	0,944721957	0,460522055	RPS17;RPL32;RPS8;SEPP1
Lomustine-8.75_mg/kg_in_Corn_Oil-Rat-Liver-1d-dn	4/291	0,614179313	0,792736197	0	0	0,944721957	0,460522055	RPS17;ARL6IP5;RPSA;RPL10A
Methotrexate-0.3_mg/kg_in_Corn_Oil-Rat-Kidney-3d-up	4/291	0,614179313	0,792606304	0	0	0,944721957	0,460522055	RPS20;RPSA;RPS3A;SEPP1
Mevastatin-1200_mg/kg_in_CMC-Rat-Liver-1d-dn	4/291	0,614179313	0,792476454	0	0	0,944721957	0,460522055	C1QA;RPS19;CLP1;RPL39
Noscapine-570_mg/kg_in_CMC-Rat-Spleen-5d-dn	4/291	0,614179313	0,792346646	0	0	0,944721957	0,460522055	C1QA;RPS17;ARL6IP5;RPS3A
Ticrynafen-570_mg/kg_in_CMC-Rat-Liver-1d-dn	4/291	0,614179313	0,792216881	0	0	0,944721957	0,460522055	C1QA;RPS17;CLP1;SEPP1
Timolol-900_mg/kg_in_Water-Rat-Heart-5d-dn	4/291	0,614179313	0,792087158	0	0	0,944721957	0,460522055	RPS19;COL5A3;GOLGA7;TPT1
Tranilast-300_mg/kg_in_CMC-Rat-Liver-5d-up	4/291	0,614179313	0,791957478	0	0	0,944721957	0,460522055	EEF1B2;RPS3A;SEPP1;TPT1
Dexfenfluramine-29_mg/kg_in_Water-Rat-Kidney-3d-up	5/365	0,615784493	0,79389731	0	0	0,941486607	0,456487526	ARL2BP;CLP1;RPS3A;SEPP1;TPT1
Acetaminophen-486_mg/kg_in_Corn_Oil-Rat-Liver-5d-up	4/292	0,616872155	0,79516941	0	0	0,941486607	0,454826042	RPS8;RPS5;RPS20;RPSA
Allyl_Alcohol-16_mg/kg_in_Saline-Rat-Liver-1d-up	4/292	0,616872155	0,795039289	0	0	0,941486607	0,454826042	EEF1B2;RPS20;RPSA;SEPP1
Atorvastatin-300_mg/kg_in_Corn_Oil-Rat-Kidney-5d-dn	4/292	0,616872155	0,794909211	0	0	0,941486607	0,454826042	EEF1B2;RPL10A;SEPP1;RPL39
Bezafibrate-100_mg/kg_in_Corn_Oil-Rat-Liver-3d-up	4/292	0,616872155	0,794779175	0	0	0,941486607	0,454826042	RPS17;RPL32;RPS6;RPS3A
Caffeine-93_mg/kg_in_Water-Rat-Brain-5d-dn	4/292	0,616872155	0,794649181	0	0	0,941486607	0,454826042	FEM1B;RPS6;RPS20;SEPP1
Citalopram-90_mg/kg_in_Corn_Oil-Rat-Liver-1d-up	4/292	0,616872155	0,794519231	0	0	0,941486607	0,454826042	C1QA;RPS8;SEPP1;TPT1
Doxorubicin-1_mg/kg_in_Saline-Rat-Kidney-5d-up	4/292	0,616872155	0,794389322	0	0	0,941486607	0,454826042	CLP1;FUNDC1;RAPGEF5;RPL39
Isoniazid-50_mg/kg_in_Saline-Rat-Liver-5d-up	4/292	0,616872155	0,794259456	0	0	0,941486607	0,454826042	C1QA;SEPP1;GOLGA7;TPT1

Mevastatin-1200_mg/kg_in_CMC-Rat-Liver-3d-up	4/292	0,616872155	0,794129633	0	0	0,941486607	0,454826042	RPS17;RPS20;RPSA;RPL39
NN-Dimethylformamide-140_mg/kg_in_Saline-Rat-Liver-1d-dn	4/292	0,616872155	0,793999852	0	0	0,941486607	0,454826042	RPL32;RPS8;RPS20;TPT1
Nimesulide-30_mg/kg_in_Corn_Oil-Rat-Kidney-3d-up	4/292	0,616872155	0,793870114	0	0	0,941486607	0,454826042	RPL32;RPS8;RPS5;RPS3A
Phenytoin-46_mg/kg_in_Water-Rat-Heart-3d-dn	4/292	0,616872155	0,793740417	0	0	0,941486607	0,454826042	EEF1B2;CXCL11;FUNDC1;RAPGEF5
Pravastatin-1200_mg/kg_in_Corn_Oil-Rat-Kidney-3d-up	4/292	0,616872155	0,793610764	0	0	0,941486607	0,454826042	ARL2BP;RPS8;RAPGEF5;SEPP1
Safrole-488_mg/kg_in_Corn_Oil-Rat-Liver-3d-up	4/292	0,616872155	0,793481152	0	0	0,941486607	0,454826042	RPS19;RPS8;RPSA;TPT1
Tacrolimus-134_mg/kg_in_CMC-Rat-Kidney-5d-dn	4/292	0,616872155	0,793351583	0	0	0,941486607	0,454826042	RPS17;RPS3A;SEPP1;RPL39
Tinidazole-1200_mg/kg_in_CMC-Rat-Liver-1d-up	4/292	0,616872155	0,793222056	0	0	0,941486607	0,454826042	RPSA;RPS3A;RPL10A;RPL39
Tranilast-1100_mg/kg_in_CMC-Rat-Liver-3d-dn	4/292	0,616872155	0,793092572	0	0	0,941486607	0,454826042	C1QA;RPS17;RPL32;RPS20
Aspirin-35_mg/kg_in_Corn_Oil-Rat-Heart-3d-dn	3/218	0,617358414	0,793588194	0	0	0,945805353	0,456167149	CLP1;RPS3A;SEPP1
Candesartan-1300_mg/kg_in_CMC-Rat-Heart-1d-up	4/293	0,619553329	0,796279702	0	0	0,938273342	0,44920446	EEF1B2;CLP1;RPS20;RAPGEF5
Cetraxate-1503_mg/kg_in_CMC-Rat-Liver-1d-dn	4/293	0,619553329	0,796149782	0	0	0,938273342	0,44920446	RPS19;RPS6;RPL10A;SEPP1
Chlordiazepoxide-196_mg/kg_in_Water-Rat-Heart-3d-dn	4/293	0,619553329	0,796019905	0	0	0,938273342	0,44920446	EEF1B2;RPL10A;RPL39;TPT1
Dichlorvos-17_mg/kg_in_Water-Rat-Heart-3d-up	4/293	0,619553329	0,795890069	0	0	0,938273342	0,44920446	ARL2BP;RPS8;RAPGEF5;GOLGA7
Ergocalciferol-15_mg/kg_in_CMC-Rat-Liver-1d-up	4/293	0,619553329	0,795760277	0	0	0,938273342	0,44920446	CLP1;RPS8;RPS3A;TPT1
Gentian_Violet-18_mg/kg_in_Corn_Oil-Rat-Liver-5d-up	4/293	0,619553329	0,795630526	0	0	0,938273342	0,44920446	EEF1B2;C1QA;RPSA;SEPP1
Isoeugenol-1560_mg/kg_in_CMC-Rat-Kidney-1d-up	4/293	0,619553329	0,795500818	0	0	0,938273342	0,44920446	RPS17;ARL6IP5;RPS3A;SEPP1
Ramipril-620_uM_in_DMSO-Rat-Primary_rat_hepatocytes-0.67d-dn	4/293	0,619553329	0,795371152	0	0	0,938273342	0,44920446	RPS6;SEPP1;RPL39;TPT1
Roxarsone-11_mg/kg_in_Corn_Oil-Rat-Kidney-0.25d-dn	4/293	0,619553329	0,795241528	0	0	0,938273342	0,44920446	ARL2BP;CLP1;RPS8;TPT1
Terazosin-657_mg/kg_in_Water-Rat-Heart-3d-dn	4/293	0,619553329	0,795111947	0	0	0,938273342	0,44920446	EEF1B2;RPS20;SEPP1;RPL39
Tobramycin-40_mg/kg_in_Saline-Rat-Kidney-14d-dn	4/293	0,619553329	0,794982407	0	0	0,938273342	0,44920446	CLP1;FUNDC1;RAPGEF5;SEPP1
Eucalyptol-930_mg/kg_in_Corn_Oil-Rat-Kidney-1d-dn	3/219	0,620447081	0,795999545	0	0	0,941486607	0,449385645	ARL2BP;CLP1;RPL10A
1-Naphthyl_Isothiocyanate-60_mg/kg_in_Corn_Oil-Rat-Bone_marrow-3d-dn	4/294	0,622222787	0,798147667	0	0	0,935081937	0,443656237	RPS19;FEM1B;RPSA;SEPP1
2-Acetylaminofluorene-30_mg/kg_in_CMC-Rat-Liver-5d-dn	4/294	0,622222787	0,798017696	0	0	0,935081937	0,443656237	CLP1;RPS8;RPS3A;RPL10A
Alpha-Naphthoflavone-80_mg/kg_in_CMC-Rat-Liver-28d-up	4/294	0,622222787	0,797887768	0	0	0,935081937	0,443656237	CLP1;RPS20;SEPP1;RPL39
Azaribine-750_mg/kg_in_CMC-Rat-Heart-5d-up	4/294	0,622222787	0,797757883	0	0	0,935081937	0,443656237	EEF1B2;RPL32;RPS8;RPS3A
Bithionol-333_mg/kg_in_Corn_Oil-Rat-Bone_marrow-5d-dn	4/294	0,622222787	0,797628039	0	0	0,935081937	0,443656237	RPS19;RPS6;ARL6IP5;RPS3A
Butenafine-150_mg/kg_in_Corn_Oil-Rat-Liver-1d-up	4/294	0,622222787	0,797498238	0	0	0,935081937	0,443656237	C1QA;RPS17;RPS6;RPSA
Carbamazepine-490_mg/kg_in_CMC-Rat-Liver-3d-dn	4/294	0,622222787	0,797368479	0	0	0,935081937	0,443656237	EEF1B2;C1QA;SEPP1;TPT1
Catechol-40_mg/kg_in_Saline-Rat-Bone_marrow-0.25d-up	4/294	0,622222787	0,797238762	0	0	0,935081937	0,443656237	COL5A3;RPS5;NKG7;DUSP14
Cyclophosphamide-25_mg/kg_in_Water-Rat-Bone_marrow-3d-dn	4/294	0,622222787	0,797109088	0	0	0,935081937	0,443656237	RPS8;FEM1B;RPS3A;TPT1
Daunorubicin-4_mg/kg_in_Saline-Rat-Heart-28d-dn	4/294	0,622222787	0,796979456	0	0	0,935081937	0,443656237	ARL2BP;RPS8;RPS3A;TPT1
Deferiprone-500_mg/kg_in_Water-Rat-Liver-1d-dn	4/294	0,622222787	0,796849866	0	0	0,935081937	0,443656237	CLP1;RPS20;SEPP1;RPL39
Oxaprozin-1500_mg/kg_in_CMC-Rat-Kidney-5d-dn	4/294	0,622222787	0,796720317	0	0	0,935081937	0,443656237	EEF1B2;CLP1;RPS3A;SEPP1
Sotalol-2000_mg/kg_in_Water-Rat-Heart-3d-dn	4/294	0,622222787	0,796590812	0	0	0,935081937	0,443656237	EEF1B2;CXCL11;CLP1;SEPP1
Sparteine-2222_uM_in_DMSO-Rat-Primary_rat_hepatocytes-0.67d-up	4/294	0,622222787	0,796461348	0	0	0,935081937	0,443656237	RPS3A;RPL10A;GOLGA7;PDRG1
Theophylline-225_mg/kg_in_Water-Rat-Brain-5d-up	4/294	0,622222787	0,796331926	0	0	0,935081937	0,443656237	RPS19;RPS6;SEPP1;GOLGA7
Zomepirac-2.8_mg/kg_in_Saline-Rat-Kidney-3d-dn	4/294	0,622222787	0,796202546	0	0	0,935081937	0,443656237	RPS8;FUNDC1;RPS6;SEPP1
Allopurinol-175_mg/kg_in_Corn_Oil-Rat-Kidney-5d-dn	4/295	0,624880487	0,799473476	0	0	0,931912167	0,438180319	CLP1;RPS8;SEPP1;TPT1
Benoxaprofen-20_mg/kg_in_CMC-Rat-Liver-1d-dn	4/295	0,624880487	0,799343628	0	0	0,931912167	0,438180319	CLP1;RPS6;SEPP1;RPL39
Bezafibrate-617_mg/kg_in_Corn_Oil-Rat-Kidney-1d-up	4/295	0,624880487	0,799213822	0	0	0,931912167	0,438180319	RPS19;RPS6;RPL10A;PDRG1
Bithionol-59_mg/kg_in_Corn_Oil-Rat-Liver-1d-dn	4/295	0,624880487	0,799084059	0	0	0,931912167	0,438180319	RPS8;RPL10A;RPL39;TPT1
Chloramphenicol-1500_mg/kg_in_CMC-Rat-Kidney-5d-up	4/295	0,624880487	0,798954337	0	0	0,931912167	0,438180319	RPL32;RPS19;SEPP1;GOLGA7
Cyclosporin_A-350_mg/kg_in_Corn_Oil-Rat-Liver-5d-dn	4/295	0,624880487	0,798824658	0	0	0,931912167	0,438180319	CLP1;RPS8;RPS3A;SEPP1
Cyproheptadine-37_mg/kg_in_CMC-Rat-Heart-3d-up	4/295	0,624880487	0,798695021	0	0	0,931912167	0,438180319	RPS19;FUNDC1;SEPP1;TPT1
Dexamethasone-300_mg/kg_in_Corn_Oil-Rat-Kidney-1d-dn	4/295	0,624880487	0,798565425	0	0	0,931912167	0,438180319	RPS17;RPS19;ARL2BP;RPS3A
Etoposide-100_mg/kg_in_Corn_Oil-Rat-Spleen-0.25d-dn	4/295	0,624880487	0,798435872	0	0	0,931912167	0,438180319	RPS17;RPSA;RPL39;TPT1
Fenofibrate-430_mg/kg_in_Corn_Oil-Rat-Skeletal_muscle-3d-up	4/295	0,624880487	0,798306361	0	0	0,931912167	0,438180319	RPS19;ARL2BP;RPS6;RPS20
Gabapentin-100_mg/kg_in_Water-Rat-Heart-3d-dn	4/295	0,624880487	0,798176892	0	0	0,931912167	0,438180319	EEF1B2;RPS17;RPL32;RPL39
Gadopentetate_Dimeglumine-125_mg/kg_in_Saline-Rat-Kidney-3d-dn	4/295	0,624880487	0,798047465	0	0	0,931912167	0,438180319	RPS8;RPL10A;SEPP1;TPT1
Lamivudine-35_mg/kg_in_Water-Rat-Liver-1d-dn	4/295	0,624880487	0,79791808	0	0	0,931912167	0,438180319	RPS19;RPS8;RPSA;SEPP1
Lansoprazole-600_mg/kg_in_CMC-Rat-Liver-5d-dn	4/295	0,624880487	0,797788737	0	0	0,931912167	0,438180319	EEF1B2;RPS19;RPS6;SEPP1
Manganese_(II)_Chloride-700_mg/kg_in_Water-Rat-Liver-1d-up	4/295	0,624880487	0,797659435	0	0	0,931912167	0,438180319	C1QA;RPSA;RPL39;TPT1
Ondansetron-84_mg/kg_in_Water-Rat-Intestine-0.25d-up	4/295	0,624880487	0,797530176	0	0	0,931912167	0,438180319	EEF1B2;C1QA;RPS3A;RPL39
Carmofur-201_mg/kg_in_CMC-Rat-Bone_marrow-3d-dn	4/296	0,627526386	0,800777351	0	0	0,928763815	0,432775667	FEM1B;RPS6;RPS3A;RPL39
Cetraxate-1503_mg/kg_in_CMC-Rat-Liver-3d-dn	4/296	0,627526386	0,800647629	0	0	0,928763815	0,432775667	C1QA;CLP1;RPS8;TPT1
Clarithromycin-476_mg/kg_in_Water-Rat-Liver-1d-dn	4/296	0,627526386	0,800517948	0	0	0,928763815	0,432775667	RPL32;RPS19;RPL10A;SEPP1
Colchicine-1000_uM_in_DMSO-Rat-Primary_rat_hepatocytes-1d-up	4/296	0,627526386	0,80038831	0	0	0,928763815	0,432775667	ARL2BP;RPS8;RPS5;TPT1
Dobutamine-43_mg/kg_in_Saline-Rat-Heart-5d-dn	4/296	0,627526386	0,800258713	0	0	0,928763815	0,432775667	ARL2BP;RPS8;RPS20;RAPGEF5
Doxifluridine-1230_mg/kg_in_CMC-Rat-Bone_marrow-3d-up	4/296	0,627526386	0,800129159	0	0	0,928763815	0,432775667	C1QA;RPL32;CLP1;SLC16A3
NN-Dimethylformamide-140_mg/kg_in_Saline-Rat-Bone_marrow-3d-up	4/296	0,627526386	0,799999646	0	0	0,928763815	0,432775667	NKG7;RPS3A;SEPP1;RPL39
Niacinamide-750_mg/kg_in_Water-Rat-Liver-1d-up	4/296	0,627526386	0,799870175	0	0	0,928763815	0,432775667	RPS17;RPS6;RPL10A;RPL39
Nisoldipine-15_mg/kg_in_Water-Rat-Liver-3d-dn	4/296	0,627526386	0,799740746	0	0	0,928763815	0,432775667	EEF1B2;RPL32;SEPP1;RPL39
Phenytoin-46_mg/kg_in_Water-Rat-Heart-5d-up	4/296	0,627526386	0,799611359	0	0	0,928763815	0,432775667	RPL10A;SEPP1;RPL39;GOLGA7
Spironolactone-300_mg/kg_in_CMC-Rat-Heart-5d-dn	4/296	0,627526386	0,799482014	0	0	0,928763815	0,432775667	COL5A3;ARL6IP5;RPSA;SEPP1
Tacrine-24_mg/kg_in_Water-Rat-Liver-3d-up	4/296	0,627526386	0,799352711	0	0	0,928763815	0,432775667	EEF1B2;C1QA;RPL10A;SEPP1
Trichloroethane-300_mg/kg_in_Water-Rat-Liver-1d-up	4/296	0,627526386	0,79922345	0	0	0,928763815	0,432775667	CXCL11;RPL35A;RPSA;RPL39
Rosiglitazone-1800_mg/kg_in_Corn_Oil-Rat-Skeletal_muscle-5d-dn	5/370	0,627741787	0,799368523	0	0	0,928763815	0,432456918	FEM1B;COL5A3;ARL6IP5;SLC16A3;TPT1
Carboplatin-14_mg/kg_in_Saline-Rat-Bone_marrow-1d-dn	3/222	0,629611435	0,801619732	0	0	0,928763815	0,429694827	EEF1B2;RPS19;RPL39
4-Nonylphenol-200_mg/kg_in_Corn_Oil-Rat-Kidney-3d-dn	4/297	0,630160441	0,802189047	0	0	0,925636664	0,427441261	EEF1B2;RPL32;CLP1;SEPP1
Amoxapine-313_mg/kg_in_CMC-Rat-Liver-1d-dn	4/297	0,630160441	0,802059411	0	0	0,925636664	0,427441261	C1QA;RPS19;RPL10A;SEPP1
Buflomedil-615_mg/kg_in_CMC-Rat-Heart-5d-up	4/297	0,630160441	0,801929817	0	0	0,925636664	0,427441261	RPS17;RPL32;RPS6;TPT1
Carmustine-16_mg/kg_in_Corn_Oil-Rat-Bone_marrow-5d-dn	4/297	0,630160441	0,801800264	0	0	0,925636664	0,427441261	FEM1B;NKG7;RPL39;TPT1
Carmustine-16_mg/kg_in_Corn_Oil-Rat-Spleen-1d-dn	4/297	0,630160441	0,801670754	0	0	0,925636664	0,427441261	RPS19;RPS6;RPL35A;RPS20
Carvedilol-50_uM_in_DMSO-Rat-Primary_rat_hepatocytes-0.67d-dn	4/297	0,630160441	0,801541285	0	0	0,925636664	0,427441261	C1QA;ARL6IP5;SEPP1;SLC16A3
Clotrimazole-178_mg/kg_in_Corn_Oil-Rat-Liver-1d-up	4/297	0,630160441	0,801411858	0	0	0,925636664	0,427441261	RPS17;RPS19;RPS20;RPSA
Indinavir-1000_mg/kg_in_CMC-Rat-Kidney-1d-dn	4/297	0,630160441	0,801282473	0	0	0,925636664	0,427441261	SEPP1;RPL39;GOLGA7;TPT1
Pemoline-833_uM_in_DMSO-Rat-Primary_rat_hepatocytes-0.67d-up	4/297	0,630160441	0,801153129	0	0	0,925636664	0,427441261	CXCL11;ARL6IP5;SEPP1;GOLGA7
Simvastatin-1200_mg/kg_in_Corn_Oil-Rat-Skeletal_muscle-3d-up	4/297	0,630160441	0,801023828	0	0	0,925636664	0,427441261	RPS19;RPS20;RPL10A;SEPP1
Sodium_Arsenite-10_mg/kg_in_Saline-Rat-Kidney-1d-dn	4/297	0,630160441	0,800894568	0	0	0,925636664	0,427441261	RPS19;CLP1;RPL10A;TPT1
Valdecoxib-404_mg/kg_in_CMC-Rat-Heart-5d-dn	4/297	0,630160441	0,800765349	0	0	0,925636664	0,427441261	RPS19;COL5A3;RPS6;SEPP1
Cyclosporin_A-70_mg/kg_in_Corn_Oil-Rat-Kidney-1d-dn	3/223	0,632632221	0,803776636	0	0	0,924598955	0,423342458	EEF1B2;ARL2BP;SEPP1
Famciclovir-1200_mg/kg_in_Saline-Rat-Liver-3d-up	3/223	0,632632221	0,803646995	0	0	0,924598955	0,423342458	RPS19;RPS20;RPL10A
14-Dichlorobenzene-735_mg/kg_in_CMC-Rat-Kidney-1d-dn	4/298	0,632782615	0,803708414	0	0	0,922530501	0,422176098	RPS6;RPSA;SEPP1;RPL39
Aztreonam-1100_mg/kg_in_CMC-Rat-Spleen-3d-dn	4/298	0,632782615	0,803578826	0	0	0,922530501	0,422176098	EEF1B2;C1QA;RPL32;RPS3A
Carboplatin-6_mg/kg_in_Saline-Rat-Liver-5d-up	4/298	0,632782615	0,803449279	0	0	0,922530501	0,422176098	RPS19;RPL35A;RPS20;SEPP1
Chlorpromazine-73_mg/kg_in_Water-Rat-Liver-3d-dn	4/298	0,632782615	0,803319774	0	0	0,922530501	0,422176098	RPL32;RPL35A;RPL39;TPT1
Chlorpropamide-269_mg/kg_in_CMC-Rat-Heart-5d-dn	4/298	0,632782615	0,803190311	0	0	0,922530501	0,422176098	COL5A3;RPL10A;SEPP1;RPL39
Geraniol-1500_mg/kg_in_CMC-Rat-Liver-5d-dn	4/298	0,632782615	0,803060889	0	0	0,922530501	0,422176098	EEF1B2;RPS8;RPS20;RPSA
Gold_Sodium_Thiomalate-8_mg/kg_in_Saline-Rat-Kidney-28d-dn	4/298	0,632782615	0,802931509	0	0	0,922530501	0,422176098	CLP1;RPSA;RPL10A;SEPP1

Metronidazole-1500_mg/kg_in_Water-Rat-Liver-3d-dn	4/298	0,632782615	0,802802171	0	0	0,922530501	0,422176098	C1QA;CLP1;SEPP1;RPL39
Ritonavir-1200_mg/kg_in_CMC-Rat-Kidney-1d-up	4/298	0,632782615	0,802672875	0	0	0,922530501	0,422176098	C1QA;RPS19;ARL2BP;RPS6
Rosiglitazone-1800_mg/kg_in_Corn_Oil-Rat-Liver-5d-up	4/298	0,632782615	0,80254362	0	0	0,922530501	0,422176098	RPL32;RPS8;RPS20;RPSA
Sildenafil-300_mg/kg_in_Saline-Rat-Liver-3d-up	4/298	0,632782615	0,802414406	0	0	0,922530501	0,422176098	RPS17;RPS19;RPS20;RPL10A
Spironolactone-300_mg/kg_in_CMC-Rat-Kidney-3d-up	4/298	0,632782615	0,802285235	0	0	0,922530501	0,422176098	RPSA;RAPGEF5;RPL10A;RPL39
Amikacin-160_mg/kg_in_Saline-Rat-Kidney-5d-dn	4/299	0,63539287	0,805465031	0	0	0,919445115	0,41697919	RPS17;RPS19;ARL2BP;SEPP1
Bupropion-895_mg/kg_in_Water-Rat-Liver-1d-dn	4/299	0,63539287	0,80533541	0	0	0,919445115	0,41697919	C1QA;RPS8;SEPP1;TPT1
Carbon_Tetrachloride-400_mg/kg_in_Corn_Oil-Rat-Bone_marrow-3d-dn	4/299	0,63539287	0,805205831	0	0	0,919445115	0,41697919	RPS19;RPSA;RPL10A;SEPP1
Carvedilol-20_mg/kg_in_Corn_Oil-Rat-Heart-1d-up	4/299	0,63539287	0,805076294	0	0	0,919445115	0,41697919	RPS17;RPS3A;RPL10A;TPT1
Dexamethasone-1_mg/kg_in_Corn_Oil-Rat-Liver-0.25d-dn	4/299	0,63539287	0,804946798	0	0	0,919445115	0,41697919	C1QA;RPS20;SEPP1;RPL39
Diethylstilbestrol-280_mg/kg_in_Corn_Oil-Rat-Bone_marrow-5d-dn	4/299	0,63539287	0,804817343	0	0	0,919445115	0,41697919	RPS8;RPSA;RPS3A;TPT1
Fluocinolone_Acetonide-2.5_mg/kg_in_Corn_Oil-Rat-Heart-3d-up	4/299	0,63539287	0,804687931	0	0	0,919445115	0,41697919	C1QA;RPS20;RAPGEF5;SEPP1
Ketamine-30_mg/kg_in_Saline-Rat-Heart-5d-dn	4/299	0,63539287	0,80455856	0	0	0,919445115	0,41697919	ARL2BP;RPS8;ARL6IP5;RAPGEF5
Nevirapine-29_mg/kg_in_Saline-Rat-Liver-5d-up	4/299	0,63539287	0,80442923	0	0	0,919445115	0,41697919	RPS8;RPL35A;RPS20;RPSA
Pravastatin-1200_mg/kg_in_Corn_Oil-Rat-Skeletal_muscle-3d-up	4/299	0,63539287	0,804299942	0	0	0,919445115	0,41697919	RPS17;RPS19;RPS6;RPSA
Rapamycin-20_uM_in_DMSO-Rat-Primary_rat_hepatocytes-0.67d-dn	4/299	0,63539287	0,804170696	0	0	0,919445115	0,41697919	CXCL11;SEPP1;SLC16A3;TPT1
Tranilast-300_mg/kg_in_CMC-Rat-Heart-1d-up	4/299	0,63539287	0,804041491	0	0	0,919445115	0,41697919	EEF1B2;FUNDC1;RAPGEF5;RPL39
Oxymetazoline-0.5_mg/kg_in_Water-Rat-Liver-0.25d-dn	3/224	0,635635956	0,804219886	0	0	0,920471281	0,417092485	RPS17;RPS3A;RPL39
Sulfamethoxazole-1000_mg/kg_in_Water-Rat-Kidney-5d-dn	3/224	0,635635956	0,804090715	0	0	0,920471281	0,417092485	CLP1;SEPP1;TPT1
Bezafibrate-100_mg/kg_in_Corn_Oil-Rat-Kidney-5d-dn	4/300	0,637991168	0,806940491	0	0	0,916380298	0,411849566	CLP1;RAPGEF5;SEPP1;RPL39
Bromisovalum-250_mg/kg_in_Corn_Oil-Rat-Liver-5d-dn	4/300	0,637991168	0,806810925	0	0	0,916380298	0,411849566	EEF1B2;C1QA;RPL32;RPL10A
Celecoxib-35_mg/kg_in_Corn_Oil-Rat-Heart-5d-dn	4/300	0,637991168	0,8066814	0	0	0,916380298	0,411849566	RPL32;RPS6;RPS3A;SEPP1
Doxorubicin-4_mg/kg_in_Saline-Rat-Kidney-5d-up	4/300	0,637991168	0,806551916	0	0	0,916380298	0,411849566	RPS19;CLP1;RPS20;RPL10A
Econazole-334_mg/kg_in_Corn_Oil-Rat-Liver-5d-dn	4/300	0,637991168	0,806422475	0	0	0,916380298	0,411849566	C1QA;RPSA;RPL10A;SEPP1
Fluconazole-394_mg/kg_in_Corn_Oil-Rat-Liver-3d-dn	4/300	0,637991168	0,806293074	0	0	0,916380298	0,411849566	EEF1B2;RPL35A;SEPP1;TPT1
Geraniol-1500_mg/kg_in_CMC-Rat-Liver-3d-dn	4/300	0,637991168	0,806163716	0	0	0,916380298	0,411849566	C1QA;CLP1;SEPP1;TPT1
Harringtonine-23_mg/kg_in_Saline-Rat-Bone_marrow-5d-dn	4/300	0,637991168	0,806034398	0	0	0,916380298	0,411849566	NKG7;RPS3A;TPT1;DUSP14
Lead(IV)_Acetate-600_mg/kg_in_Saline-Rat-Liver-1d-up	4/300	0,637991168	0,805905123	0	0	0,916380298	0,411849566	C1QA;RPS19;CLP1;RPL39
Lead_(II)_Acetate-200_mg/kg_in_Saline-Rat-Spleen-3d-up	4/300	0,637991168	0,805775888	0	0	0,916380298	0,411849566	RPS19;RPS20;RPL10A;SEPP1
Lovastatin-1500_mg/kg_in_Corn_Oil-Rat-Skeletal_muscle-5d-dn	4/300	0,637991168	0,805646695	0	0	0,916380298	0,411849566	COL5A3;ARL6IP5;SEPP1;SLC16A3
Nimodipine-1100_mg/kg_in_Corn_Oil-Rat-Heart-3d-dn	4/300	0,637991168	0,805517544	0	0	0,916380298	0,411849566	ARL2BP;RPS8;FUNDC1;ARL6IP5
Roflumilast-17_mg/kg_in_CMC-Rat-Liver-1d-up	4/300	0,637991168	0,805388434	0	0	0,916380298	0,411849566	C1QA;RPS8;RPL39;TPT1
Sulfathiazole-2629_mg/kg_in_Water-Rat-Liver-3d-dn	4/300	0,637991168	0,805259365	0	0	0,916380298	0,411849566	C1QA;RPS19;RPL39;TPT1
Tenidap-75_mg/kg_in_Corn_Oil-Rat-Kidney-3d-dn	4/300	0,637991168	0,805130338	0	0	0,916380298	0,411849566	EEF1B2;RPS17;RPS8;TPT1
Tranexamic_Acid-2000_mg/kg_in_CMC-Rat-Heart-3d-up	4/300	0,637991168	0,805001352	0	0	0,916380298	0,411849566	RPS19;COL5A3;SEPP1;TPT1
Trimethadione-535_mg/kg_in_Water-Rat-Heart-3d-up	4/300	0,637991168	0,804872407	0	0	0,916380298	0,411849566	FUNDC1;ARL6IP5;SEPP1;GOLGA7
6-Mercaptopurine-25_mg/kg_in_Corn_Oil-Rat-Liver-0.25d-dn	4/301	0,640577476	0,808005798	0	0	0,913335845	0,406786271	EEF1B2;C1QA;RPL35A;RPL39
Acyclovir-330_mg/kg_in_Saline-Rat-Liver-3d-up	4/301	0,640577476	0,807876414	0	0	0,913335845	0,406786271	C1QA;RPS19;RPS6;RPL39
Allyl_Alcohol-25_mg/kg_in_Saline-Rat-Kidney-3d-dn	4/301	0,640577476	0,807747071	0	0	0,913335845	0,406786271	EEF1B2;RPS19;RPS6;RPL10A
Altretamine-40_mg/kg_in_Corn_Oil-Rat-Liver-0.25d-dn	4/301	0,640577476	0,807617769	0	0	0,913335845	0,406786271	RPS8;RPSA;RPL39;TPT1
Azathioprine-20_mg/kg_in_Corn_Oil-Rat-Liver-1d-up	4/301	0,640577476	0,807488509	0	0	0,913335845	0,406786271	RPS19;RPS20;RPL39;GOLGA7
Baclofen-6.6_mg/kg_in_CMC-Rat-Heart-5d-up	4/301	0,640577476	0,80735929	0	0	0,913335845	0,406786271	RPS19;RPS6;RPSA;RPL39
Benzothiazyl_Disulfide-2600_mg/kg_in_Corn_Oil-Rat-Liver-5d-dn	4/301	0,640577476	0,807230112	0	0	0,913335845	0,406786271	EEF1B2;RPS17;RPS20;SEPP1
Chlormadinone_Acetate-2000_mg/kg_in_CMC-Rat-Kidney-3d-up	4/301	0,640577476	0,807100976	0	0	0,913335845	0,406786271	RPS17;RPS20;RPS3A;RPL39
Econazole-334_mg/kg_in_Corn_Oil-Rat-Liver-1d-up	4/301	0,640577476	0,806971881	0	0	0,913335845	0,406786271	RPS19;RPS8;RPS20;RPL10A
Fenofibrate-215_mg/kg_in_Corn_Oil-Rat-Skeletal_muscle-3d-up	4/301	0,640577476	0,806842828	0	0	0,913335845	0,406786271	RPS19;FUNDC1;RPS6;SEPP1
Finasteride-800_mg/kg_in_Corn_Oil-Rat-Liver-3d-up	4/301	0,640577476	0,806713816	0	0	0,913335845	0,406786271	RPS8;RPS5;RPS20;RPSA
Flunarizine-126_mg/kg_in_CMC-Rat-Heart-5d-dn	4/301	0,640577476	0,806584845	0	0	0,913335845	0,406786271	RPL10A;SEPP1;RPL39;TPT1
Fluocinolone_Acetonide-2.5_mg/kg_in_Corn_Oil-Rat-Liver-1d-up	4/301	0,640577476	0,806455915	0	0	0,913335845	0,406786271	CLP1;RPS8;RPS20;TPT1
Itraconazole-1093_mg/kg_in_Corn_Oil-Rat-Liver-1d-dn	4/301	0,640577476	0,806327026	0	0	0,913335845	0,406786271	C1QA;RPS17;ARL2BP;SEPP1
Pioglitazone-3_mg/kg_in_Corn_Oil-Rat-Liver-0.25d-dn	4/301	0,640577476	0,806198179	0	0	0,913335845	0,406786271	RPS8;RPS5;RPS20;RPSA
Praziquantel-1200_mg/kg_in_CMC-Rat-Liver-3d-up	4/301	0,640577476	0,806069373	0	0	0,913335845	0,406786271	RPS19;RPS6;RPSA;RPL10A
Rofecoxib-1550_mg/kg_in_Corn_Oil-Rat-Liver-1d-dn	4/301	0,640577476	0,805940607	0	0	0,913335845	0,406786271	RPS8;RPS6;RPL39;TPT1
Rosiglitazone-10_mg/kg_in_Corn_Oil-Rat-Liver-0.25d-dn	4/301	0,640577476	0,805811884	0	0	0,913335845	0,406786271	RPS20;RPL10A;RPL39;TPT1
Cisplatin-1.17_mg/kg_in_Saline-Rat-Kidney-3d-dn	3/226	0,641592171	0,806959428	0	0	0,912325518	0,404892277	EEF1B2;RPS6;SEPP1
Cisplatin-1.17_mg/kg_in_Saline-Rat-Liver-1d-up	3/226	0,641592171	0,806830583	0	0	0,912325518	0,404892277	C1QA;RPS3A;TPT1
Altretamine-13_mg/kg_in_Corn_Oil-Rat-Bone_marrow-1d-up	4/302	0,643151761	0,808662719	0	0	0,910311554	0,401788364	C1QA;RPS17;RPS3A;RPL39
Bezafibrate-617_mg/kg_in_Corn_Oil-Rat-Liver-1d-dn	4/302	0,643151761	0,808533643	0	0	0,910311554	0,401788364	C1QA;RPSA;SEPP1;RPL39
Bisphenol_A-50_mg/kg_in_Corn_Oil-Rat-Liver-1d-dn	4/302	0,643151761	0,808404607	0	0	0,910311554	0,401788364	RPS19;RPS8;RPS6;RPS20
Chlorobenzene-500_mg/kg_in_CMC-Rat-Kidney-3d-up	4/302	0,643151761	0,808275614	0	0	0,910311554	0,401788364	CXCL11;CLP1;RPS3A;SEPP1
Diethylstilbestrol-2.8_mg/kg_in_Corn_Oil-Rat-Liver-0.25d-dn	4/302	0,643151761	0,808146661	0	0	0,910311554	0,401788364	RPL35A;SEPP1;RPL39;TPT1
Lomustine-4.2_mg/kg_in_Corn_Oil-Rat-Spleen-1d-up	4/302	0,643151761	0,808017749	0	0	0,910311554	0,401788364	ARL2BP;FUNDC1;RPS20;TPT1
Melphalan-12_mg/kg_in_CMC-Rat-Bone_marrow-1d-up	4/302	0,643151761	0,807888879	0	0	0,910311554	0,401788364	EEF1B2;RPS6;NKG7;RPS3A
Methyldopa-325_mg/kg_in_Water-Rat-Heart-3d-dn	4/302	0,643151761	0,80776005	0	0	0,910311554	0,401788364	EEF1B2;RPS19;SEPP1;RPL39
Rosiglitazone-1800_mg/kg_in_Corn_Oil-Rat-Liver-1d-dn	4/302	0,643151761	0,807631261	0	0	0,910311554	0,401788364	EEF1B2;C1QA;SEPP1;RPL39
Salicylic_Acid-223_mg/kg_in_Corn_Oil-Rat-Liver-3d-up	4/302	0,643151761	0,807502514	0	0	0,910311554	0,401788364	RPS19;FEM1B;RPS6;SEPP1
Valsartan-1000_mg/kg_in_Corn_Oil-Rat-Heart-3d-up	4/302	0,643151761	0,807373808	0	0	0,910311554	0,401788364	RPS19;FUNDC1;RPS6;SEPP1
Amantadine-1500_uM_in_DMSO-Rat-Primary_rat_hepatocytes-0.67d-dn	3/227	0,644544606	0,808993357	0	0	0,908306463	0,398938416	RPS17;CXADR;SLC16A3
4-Chloroaniline-75_mg/kg_in_CMC-Rat-Kidney-5d-dn	4/303	0,645713991	0,810331962	0	0	0,907307226	0,39685492	RPS19;RPS6;SEPP1;RPL39
Aconitine-0.9_mg/kg_in_CMC-Rat-Heart-3d-up	4/303	0,645713991	0,810202867	0	0	0,907307226	0,39685492	RPS17;RPS6;RPS3A;TPT1
Atorvastatin-300_mg/kg_in_Corn_Oil-Rat-Liver-3d-dn	4/303	0,645713991	0,810073812	0	0	0,907307226	0,39685492	RPL32;RPL35A;SEPP1;TPT1
Bithionol-59_mg/kg_in_Corn_Oil-Rat-Liver-3d-dn	4/303	0,645713991	0,809944799	0	0	0,907307226	0,39685492	C1QA;RPS8;SEPP1;TPT1
Cyclosporin_A-350_mg/kg_in_Corn_Oil-Rat-Heart-1d-up	4/303	0,645713991	0,809815827	0	0	0,907307226	0,39685492	CXCL11;COL5A3;SEPP1;RPL39
Diethylstilbestrol-2.8_mg/kg_in_Corn_Oil-Rat-Liver-1d-dn	4/303	0,645713991	0,809686896	0	0	0,907307226	0,39685492	C1QA;CLP1;SEPP1;TPT1
Doxepin-147_mg/kg_in_CMC-Rat-Heart-5d-dn	4/303	0,645713991	0,809558006	0	0	0,907307226	0,39685492	COL5A3;RPS20;RPSA;RPL10A
Lovastatin-450_mg/kg_in_Corn_Oil-Rat-Kidney-3d-dn	4/303	0,645713991	0,809429157	0	0	0,907307226	0,39685492	RPS19;RPL10A;SEPP1;TPT1
Methocarbamol-1125_mg/kg_in_CMC-Rat-Liver-1d-up	4/303	0,645713991	0,809300349	0	0	0,907307226	0,39685492	RPS17;RPSA;RPL39;TPT1
Norethindrone_Acetate-125_mg/kg_in_Corn_Oil-Rat-Liver-3d-dn	4/303	0,645713991	0,809171582	0	0	0,907307226	0,39685492	EEF1B2;C1QA;RPL35A;TPT1
Promethazine-113_mg/kg_in_Saline-Rat-Liver-3d-dn	4/303	0,645713991	0,809042856	0	0	0,907307226	0,39685492	EEF1B2;C1QA;SEPP1;TPT1
Propylthiouracil-625_mg/kg_in_CMC-Rat-Liver-1d-up	4/303	0,645713991	0,808914171	0	0	0,907307226	0,39685492	RPS19;RPS6;RPSA;RPL10A
SCH-351591-4.6_mg/kg_in_CMC-Rat-Liver-1d-up	4/303	0,645713991	0,808785527	0	0	0,907307226	0,39685492	C1QA;RPS17;RPSA;SEPP1
Simvastatin-1200_mg/kg_in_Corn_Oil-Rat-Liver-0.25d-dn	4/303	0,645713991	0,808656924	0	0	0,907307226	0,39685492	RPS20;RPSA;SEPP1;TPT1
Troglitazone-1200_mg/kg_in_Corn_Oil-Rat-Liver-3d-up	4/303	0,645713991	0,808528362	0	0	0,907307226	0,39685492	RPS8;RPS3A;SEPP1;TPT1
Vancomycin-160_mg/kg_in_Saline-Rat-Kidney-3d-dn	4/303	0,645713991	0,80839984	0	0	0,907307226	0,39685492	EEF1B2;RPS20;RPL39;TPT1
Zaleplon-100_mg/kg_in_Water-Rat-Liver-5d-up	4/303	0,645713991	0,80827136	0	0	0,907307226	0,39685492	CXCL11;ARL6IP5;SEPP1;RPL39
Idarubicin-1.5_uM_in_DMSO-Rat-Primary_rat_hepatocytes-0.67d-up	5/378	0,646402737	0,809004919	0	0	0,909107438	0,396673156	CXCL11;RPS19;RPL10A;SEPP1;GOLGA7
Promethazine-113_mg/kg_in_Saline-Rat-Liver-1d-dn	3/228	0,647479898	0,81022429	0	0	0,904322662	0,393079699	EEF1B2;SEPP1;PDRG1
Acetaminophen-486_mg/kg_in_Corn_Oil-Rat-Liver-0.25d-dn	4/304	0,648264137	0,811076782	0	0	0,904322662	0,391985031	C1QA;CLP1;RPL39;TPT1
Albendazole-62_mg/kg_in_Corn_Oil-Rat-Liver-1d-dn	4/304	0,648264137	0,810947957	0	0	0,904322662	0,391985031	EEF1B2;RPS17;SEPP1;RPL39
Carbon_Tetrachloride-400_mg/kg_in_Corn_Oil-Rat-Bone_marrow-0.25d-dn	4/304	0,648264137	0,810819174	0	0	0,904322662	0,391985031	RPS19;NKG7;RPL10A;SEPP1

Carmustine-16_mg/kg_in_Corn_Oil-Rat-Spleen-3d-dn	4/304	0,648264137	0,810690432	0	0	0,904322662	0,391985031	RPL10A;SEPP1;GOLGA7;TPT1
Disulfiram-100_mg/kg_in_Water-Rat-Liver-1d-dn	4/304	0,648264137	0,81056173	0	0	0,904322662	0,391985031	CLP1;SEPP1;GOLGA7;TPT1
Ergocalciferol-15_mg/kg_in_CMC-Rat-Kidney-3d-dn	4/304	0,648264137	0,81043307	0	0	0,904322662	0,391985031	RPSA;RPL10A;SEPP1;RPL39
Ketoconazole-25_mg/kg_in_Corn_Oil-Rat-Liver-5d-dn	4/304	0,648264137	0,81030445	0	0	0,904322662	0,391985031	C1QA;CLP1;RPS6;SEPP1
Leflunomide-60_mg/kg_in_Corn_Oil-Rat-Liver-5d-up	4/304	0,648264137	0,810175871	0	0	0,904322662	0,391985031	EEF1B2;RPL32;RPL39;TPT1
Levamisole-120_mg/kg_in_Water-Rat-Liver-1d-dn	4/304	0,648264137	0,810047333	0	0	0,904322662	0,391985031	EEF1B2;RPS19;RPS6;SEPP1
Levofloxacin-739_mg/kg_in_Water-Rat-Heart-5d-dn	4/304	0,648264137	0,809918836	0	0	0,904322662	0,391985031	RPS19;RPS8;FEM1B;RPS20
Methyl_Salicylate-444_mg/kg_in_Corn_Oil-Rat-Liver-1d-up	4/304	0,648264137	0,809790379	0	0	0,904322662	0,391985031	RPS19;RPS8;RPS6;RPSA
N-Nitrosodiethylamine-34_mg/kg_in_Saline-Rat-Kidney-5d-dn	4/304	0,648264137	0,809661963	0	0	0,904322662	0,391985031	EEF1B2;ARL2BP;CLP1;SEPP1
Oxymetholone-1170_mg/kg_in_CMC-Rat-Liver-5d-dn	4/304	0,648264137	0,809533588	0	0	0,904322662	0,391985031	RPL32;RPS8;RPS3A;TPT1
Penicillamine-852_mg/kg_in_Water-Rat-Liver-5d-dn	4/304	0,648264137	0,809405254	0	0	0,904322662	0,391985031	RPL32;RPSA;RPL10A;TPT1
Sildenafil-14.6_mg/kg_in_Saline-Rat-Liver-1d-up	4/304	0,648264137	0,80927696	0	0	0,904322662	0,391985031	C1QA;RPS19;RPL39;GOLGA7
Sparteine-33_mg/kg_in_Water-Rat-Heart-0.25d-dn	4/304	0,648264137	0,809148707	0	0	0,904322662	0,391985031	FUNDC1;ARL6IP5;RPSA;SEPP1
Doxorubicin-0.65_mg/kg_in_Saline-Rat-Bone_marrow-3d-up	5/379	0,6486939	0,809556831	0	0	0,906708738	0,392418393	RPS19;RPS6;RPL35A;RPS20;RPL10A
Thiamphenicol-1500_mg/kg_in_Water-Rat-Kidney-1d-dn	3/229	0,650398033	0,81155496	0	0	0,900373655	0,387314406	RPS20;GOLGA7;SEPP1
2-Nitroanisole-740_mg/kg_in_CMC-Rat-Kidney-3d-dn	4/305	0,650802169	0,811930601	0	0	0,90135767	0,3871778	RPS6;SEPP1;RPL39;TPT1
Bithionol-333_mg/kg_in_Corn_Oil-Rat-Kidney-3d-up	4/305	0,650802169	0,811802009	0	0	0,90135767	0,3871778	RPS19;RPS6;RPS3A;RAPGEF5
Citalopram-40_mg/kg_in_Corn_Oil-Rat-Liver-0.25d-up	4/305	0,650802169	0,811673457	0	0	0,90135767	0,3871778	C1QA;RPS6;RPL10A;RPL39
Ketoconazole-227_mg/kg_in_Corn_Oil-Rat-Liver-3d-dn	4/305	0,650802169	0,811544947	0	0	0,90135767	0,3871778	EEF1B2;RPS17;CLP1;RPS3A
N-Nitrosodimethylamine-10_mg/kg_in_Saline-Rat-Liver-5d-dn	4/305	0,650802169	0,811416477	0	0	0,90135767	0,3871778	EEF1B2;RPS17;RPL32;CLP1
NN-Dimethylformamide-1400_mg/kg_in_Saline-Rat-Spleen-5d-dn	4/305	0,650802169	0,811288047	0	0	0,90135767	0,3871778	RPS20;RPSA;RPL10A;SEPP1
Ofloxacin-30_mg/kg_in_Water-Rat-Heart-5d-dn	4/305	0,650802169	0,811159659	0	0	0,90135767	0,3871778	EEF1B2;RPS19;RPS8;RPL10A
Oxyquinoline-68_mg/kg_in_Corn_Oil-Rat-Liver-3d-dn	4/305	0,650802169	0,811031311	0	0	0,90135767	0,3871778	RPS19;FEM1B;RPSA;RPL39
Papaverine-69_mg/kg_in_CMC-Rat-Heart-5d-dn	4/305	0,650802169	0,810903003	0	0	0,90135767	0,3871778	COL5A3;SEPP1;RPL39;TPT1
Ramipril-1500_mg/kg_in_CMC-Rat-Kidney-1d-dn	4/305	0,650802169	0,810774736	0	0	0,90135767	0,3871778	FEM1B;SEPP1;GOLGA7;TPT1
TNF-alpha-0.25_mg/kg_in_RSA/Saline-Rat-Liver-0.25d-up	4/305	0,650802169	0,81064651	0	0	0,90135767	0,3871778	EEF1B2;RPS6;RPL10A;GOLGA7
Torsemide-110_mg/kg_in_Corn_Oil-Rat-Liver-3d-up	4/305	0,650802169	0,810518324	0	0	0,90135767	0,3871778	C1QA;SEPP1;RPL39;GOLGA7
D-Tubocurarine_Chloride-0.033_mg/kg_in_Saline-Rat-Heart-3d-dn	3/230	0,653298996	0,813499272	0	0	0,896458987	0,381640855	EEF1B2;RPS17;SEPP1
1-Naphthyl_Isothiocyanate-30_mg/kg_in_Corn_Oil-Rat-Bone_marrow-1d-up	4/306	0,653328061	0,813406862	0	0	0,898412057	0,382432347	C1QA;NKG7;RPL10A;RPL39
Cadmium_Chloride-8_uM_in_DMSO-Rat-Primary_rat_hepatocytes-0.67d-up	4/306	0,653328061	0,813278301	0	0	0,898412057	0,382432347	CXADR;RPS8;RPS5;RPSA
Digoxin-0.26_mg/kg_in_Corn_Oil-Rat-Liver-0.25d-up	4/306	0,653328061	0,813149781	0	0	0,898412057	0,382432347	RPS6;RPS20;RPL10A;RPL39
Irinotecan-5_mg/kg_in_Saline-Rat-Kidney-5d-up	4/306	0,653328061	0,813021301	0	0	0,898412057	0,382432347	C1QA;CXCL11;SEPP1;RPL39
Mevastatin-1200_mg/kg_in_CMC-Rat-Skeletal_muscle-3d-up	4/306	0,653328061	0,812892861	0	0	0,898412057	0,382432347	RPS6;RPS20;RPL10A;RPL39
NN-Dimethylformamide-1400_mg/kg_in_Saline-Rat-Liver-1d-up	4/306	0,653328061	0,812764462	0	0	0,898412057	0,382432347	RPS17;RPSA;RPL39;TPT1
Norethindrone-0.08_mg/kg_in_Corn_Oil-Rat-Liver-0.25d-dn	4/306	0,653328061	0,812636104	0	0	0,898412057	0,382432347	RPS8;RPSA;RPL39;TPT1
Phenylbutazone-368_mg/kg_in_CMC-Rat-Kidney-3d-dn	4/306	0,653328061	0,812507786	0	0	0,898412057	0,382432347	EEF1B2;ARL2BP;RPS8;RPS20
S(-)-Carbidopa-1200_mg/kg_in_CMC-Rat-Brain-3d-up	4/306	0,653328061	0,812379509	0	0	0,898412057	0,382432347	C1QA;RPS19;RAPGEF5;DUSP14
Saquinavir-1500_mg/kg_in_CMC-Rat-Kidney-1d-dn	4/306	0,653328061	0,812251273	0	0	0,898412057	0,382432347	RPSA;SEPP1;GOLGA7;TPT1
Tamoxifen-37.5_uM_in_DMSO-Rat-Primary_rat_hepatocytes-0.67d-dn	4/306	0,653328061	0,812123076	0	0	0,898412057	0,382432347	ARL6IP5;SEPP1;SLC16A3;TPT1
Tetracycline-1500_mg/kg_in_Water-Rat-Liver-1d-up	4/306	0,653328061	0,811994921	0	0	0,898412057	0,382432347	EEF1B2;RPL32;RPS6;RPL10A
Zileuton-450_mg/kg_in_Corn_Oil-Rat-Liver-3d-dn	4/306	0,653328061	0,811866805	0	0	0,898412057	0,382432347	EEF1B2;C1QA;SEPP1;RPL39
Bisphenol_A-100_mg/kg_in_Corn_Oil-Rat-Liver-5d-dn	4/307	0,655841789	0,814861954	0	0	0,895485633	0,377747805	RPS19;RPS6;RPS3A;RPL10A
Carbon_Tetrachloride-775_uM_in_DMSO-Rat-Primary_rat_hepatocytes-1d-up	4/307	0,655841789	0,814733427	0	0	0,895485633	0,377747805	RPS17;FUNDC1;SLC16A3;PDRG1
Chloroxylenol-1915_mg/kg_in_Corn_Oil-Rat-Liver-5d-up	4/307	0,655841789	0,81460494	0	0	0,895485633	0,377747805	EEF1B2;C1QA;RPSA;SEPP1
Ethambutol-998_mg/kg_in_CMC-Rat-Kidney-5d-dn	4/307	0,655841789	0,814476494	0	0	0,895485633	0,377747805	EEF1B2;RPS17;RPS20;RPS3A
Fenofibrate-100_mg/kg_in_Corn_Oil-Rat-Liver-0.25d-up	4/307	0,655841789	0,814348089	0	0	0,895485633	0,377747805	C1QA;RPS17;RPL32;RPS3A
Gemfibrozil-100_mg/kg_in_Corn_Oil-Rat-Liver-1d-dn	4/307	0,655841789	0,814219724	0	0	0,895485633	0,377747805	C1QA;RPS8;RPL10A;SEPP1
Hydrocortisone-56_mg/kg_in_Corn_Oil-Rat-Liver-3d-up	4/307	0,655841789	0,814091399	0	0	0,895485633	0,377747805	EEF1B2;CLP1;SEPP1;TPT1
Lisinopril-2000_mg/kg_in_CMC-Rat-Kidney-1d-dn	4/307	0,655841789	0,813963115	0	0	0,895485633	0,377747805	EEF1B2;RPS8;RPS6;SEPP1
Melatonin-2000_mg/kg_in_Corn_Oil-Rat-Liver-1d-dn	4/307	0,655841789	0,813834871	0	0	0,895485633	0,377747805	EEF1B2;C1QA;RPS17;SEPP1
Valsartan-1000_mg/kg_in_Corn_Oil-Rat-Kidney-1d-dn	4/307	0,655841789	0,813706668	0	0	0,895485633	0,377747805	RPS19;RPSA;RPL10A;SEPP1
Angiotensin_II_Human-0.72_mg/kg_in_Saline-Rat-Heart-1d-dn	4/308	0,658343326	0,816681688	0	0	0,892578212	0,37312332	RPS17;RPL32;SEPP1;TPT1
Bezafibrate-100_mg/kg_in_Corn_Oil-Rat-Liver-3d-dn	4/308	0,658343326	0,816553077	0	0	0,892578212	0,37312332	RPS8;SEPP1;RPL39;TPT1
Bisacodyl-1750_mg/kg_in_CMC-Rat-Intestine-3d-up	4/308	0,658343326	0,816424506	0	0	0,892578212	0,37312332	RPL32;RPS5;RPL10A;TPT1
Cephalexin-2500_mg/kg_in_CMC-Rat-Kidney-1d-up	4/308	0,658343326	0,816295976	0	0	0,892578212	0,37312332	RPS19;FUNDC1;ARL6IP5;RPSA
Cholecalciferol-8_mg/kg_in_CMC-Rat-Heart-5d-dn	4/308	0,658343326	0,816167486	0	0	0,892578212	0,37312332	COL5A3;FUNDC1;ARL6IP5;TPT1
Cisplatin-2_mg/kg_in_Saline-Rat-Bone_marrow-5d-dn	4/308	0,658343326	0,816039036	0	0	0,892578212	0,37312332	RPS17;RPL10A;RPL39;DUSP14
Diclofenac-10_mg/kg_in_Corn_Oil-Rat-Liver-1d-up	4/308	0,658343326	0,815910627	0	0	0,892578212	0,37312332	RPS6;RPS20;RPL10A;RPL39
Diethylstilbestrol-2.8_mg/kg_in_Corn_Oil-Rat-Kidney-1d-dn	4/308	0,658343326	0,815782259	0	0	0,892578212	0,37312332	RPL32;SEPP1;RPL39;TPT1
Digoxin-11_mg/kg_in_Corn_Oil-Rat-Heart-1d-up	4/308	0,658343326	0,815653931	0	0	0,892578212	0,37312332	RPS17;RPSA;RPS3A;SEPP1
Doxorubicin-0.65_mg/kg_in_Saline-Rat-Liver-0.25d-dn	4/308	0,658343326	0,815525643	0	0	0,892578212	0,37312332	C1QA;RPS19;CLP1;RPS6
Etidronate-1005_mg/kg_in_Water-Rat-Heart-1d-dn	4/308	0,658343326	0,815397395	0	0	0,892578212	0,37312332	RPSA;RPL10A;SEPP1;RPL39
Fluvastatin-5_mg/kg_in_Corn_Oil-Rat-Liver-5d-dn	4/308	0,658343326	0,815269188	0	0	0,892578212	0,37312332	RPL32;RPS8;RPL35A;RPSA
Lovastatin-1500_mg/kg_in_Corn_Oil-Rat-Liver-5d-dn	4/308	0,658343326	0,815141021	0	0	0,892578212	0,37312332	EEF1B2;CLP1;RPS3A;SEPP1
Ofloxacin-3000_mg/kg_in_Water-Rat-Heart-3d-dn	4/308	0,658343326	0,815012895	0	0	0,892578212	0,37312332	EEF1B2;RPL10A;RPL39;TPT1
Tetracycline-520_uM_in_DMSO-Rat-Primary_rat_hepatocytes-0.67d-dn	4/308	0,658343326	0,814884809	0	0	0,892578212	0,37312332	RPS19;RPSA;SEPP1;SLC16A3
Allyl_Alcohol-32_mg/kg_in_Saline-Rat-Liver-1d-dn	4/309	0,660832652	0,817837518	0	0	0,88968961	0,368558054	C1QA;CLP1;SEPP1;TPT1
Bromisovalum-250_mg/kg_in_Corn_Oil-Rat-Heart-1d-dn	4/309	0,660832652	0,817709029	0	0	0,88968961	0,368558054	RPL32;RPS6;RPS3A;TPT1
Cytarabine-487_mg/kg_in_Saline-Rat-Liver-5d-up	4/309	0,660832652	0,817580579	0	0	0,88968961	0,368558054	RPL32;SEPP1;RPL39;TPT1
Famciclovir-112_mg/kg_in_Saline-Rat-Bone_marrow-1d-up	4/309	0,660832652	0,81745217	0	0	0,88968961	0,368558054	EEF1B2;RPS19;RPS6;RPSA
Gentamicin-656.6_uM_in_DMSO-Rat-Primary_rat_hepatocytes-0.67d-up	4/309	0,660832652	0,817323801	0	0	0,88968961	0,368558054	RPL32;RPS3A;SEPP1;TPT1
Iproniazid-46_mg/kg_in_Water-Rat-Heart-3d-up	4/309	0,660832652	0,817195473	0	0	0,88968961	0,368558054	CLP1;RPS20;GOLGA7;SLC16A3
Mestranol-250_mg/kg_in_CMC-Rat-Liver-3d-dn	4/309	0,660832652	0,817067185	0	0	0,88968961	0,368558054	C1QA;CLP1;SEPP1;TPT1
Niacinamide-750_mg/kg_in_Water-Rat-Liver-3d-up	4/309	0,660832652	0,816938937	0	0	0,88968961	0,368558054	C1QA;CXCL11;RPSA;TPT1
Praziquantel-1200_mg/kg_in_CMC-Rat-Liver-1d-up	4/309	0,660832652	0,816810729	0	0	0,88968961	0,368558054	RPS19;RPS8;RPS20;RPL10A
Rabeprazole-333_uM_in_DMSO-Rat-Primary_rat_hepatocytes-0.67d-up	4/309	0,660832652	0,816682562	0	0	0,88968961	0,368558054	RPS6;RPL10A;RPL39;PDRG1
Raloxifene-650_mg/kg_in_Corn_Oil-Rat-Liver-0.25d-up	4/309	0,660832652	0,816554435	0	0	0,88968961	0,368558054	RPS19;RPSA;RPL10A;TPT1
Simvastatin-15_mg/kg_in_Corn_Oil-Rat-Liver-5d-dn	4/309	0,660832652	0,816426348	0	0	0,88968961	0,368558054	C1QA;RPS17;RPL35A;SEPP1
Sodium_Arsenite-10_mg/kg_in_Saline-Rat-Kidney-0.25d-dn	4/309	0,660832652	0,816298301	0	0	0,88968961	0,368558054	RPS19;ARL2BP;RPSA;RPL10A
Diclofenac-10_mg/kg_in_Corn_Oil-Rat-Kidney-3d-dn	3/233	0,661898749	0,817486992	0	0	0,884916597	0,365154358	KBTBD4;RPS19;SEPP1
Allyl_Alcohol-16_mg/kg_in_Saline-Rat-Liver-3d-dn	4/310	0,663309745	0,819101215	0	0	0,886819643	0,364051179	RPS17;RPS8;RPS3A;RPL39
Buspirone-196_mg/kg_in_CMC-Rat-Heart-3d-dn	4/310	0,663309745	0,818972809	0	0	0,886819643	0,364051179	RPS20;RPL10A;SEPP1;RPL39
Busulfan-36_mg/kg_in_Corn_Oil-Rat-Liver-1d-up	4/310	0,663309745	0,818844444	0	0	0,886819643	0,364051179	CXCL11;RPS6;RPS20;RPL39
Clarithromycin-476_mg/kg_in_Water-Rat-Liver-5d-dn	4/310	0,663309745	0,818716118	0	0	0,886819643	0,364051179	C1QA;RPS19;RPSA;SEPP1
Cortisone-206_mg/kg_in_CMC-Rat-Kidney-5d-dn	4/310	0,663309745	0,818587833	0	0	0,886819643	0,364051179	RPS6;RPL10A;SEPP1;GOLGA7
Dexamethasone-1_mg/kg_in_Corn_Oil-Rat-Liver-3d-dn	4/310	0,663309745	0,818459588	0	0	0,886819643	0,364051179	C1QA;RPS17;RPS3A;SEPP1
Diethylstilbestrol-280_mg/kg_in_Corn_Oil-Rat-Liver-1d-dn	4/310	0,663309745	0,818331383	0	0	0,886819643	0,364051179	C1QA;SEPP1;RPL39;TPT1
Gadopentetate_Dimeglumine-125_mg/kg_in_Saline-Rat-Kidney-5d-dn	4/310	0,663309745	0,818203219	0	0	0,886819643	0,364051179	RPS19;RPS6;RPL10A;SEPP1
Galantamine-110_mg/kg_in_CMC-Rat-Heart-1d-up	4/310	0,663309745	0,818075094	0	0	0,886819643	0,364051179	RPS6;RAPGEF5;SEPP1;PDRG1
Hydroquinone-800_mg/kg_in_CMC-Rat-Kidney-5d-up	4/310	0,663309745	0,81794701	0	0	0,886819643	0,364051179	EEF1B2;CLP1;RPS8;RPSA

Mestranol-250_mg/kg_in_CMC-Rat-Liver-1d-dn	4/310	0,663309745	0,817818965	0	0	0,886819643	0,364051179	C1QA;CLP1;SEPP1;TPT1
Methyl_Salicylate-444_mg/kg_in_Corn_Oil-Rat-Kidney-3d-dn	4/310	0,663309745	0,817690961	0	0	0,886819643	0,364051179	RPSA;RPL10A;SEPP1;PDRG1
Nortriptyline-63_mg/kg_in_Water-Rat-Heart-1d-dn	4/310	0,663309745	0,817562997	0	0	0,886819643	0,364051179	EEF1B2;FUNDC1;RPL35A;SEPP1
Nystatin-134_mg/kg_in_Corn_Oil-Rat-Liver-5d-dn	4/310	0,663309745	0,817435073	0	0	0,886819643	0,364051179	RPS6;RPS20;RPSA;RPL39
Pantoprazole-650_uM_in_DMSO-Rat-Primary_rat_hepatocytes-0.67d-up	4/310	0,663309745	0,817307189	0	0	0,886819643	0,364051179	RPS17;RPS6;RPL39;PDRG1
Sodium_Arsenite-10_mg/kg_in_Saline-Rat-Liver-0.25d-dn	4/310	0,663309745	0,817179345	0	0	0,886819643	0,364051179	EEF1B2;RPL10A;RPL39;TPT1
Clindamycin-161_mg/kg_in_Saline-Rat-Kidney-1d-dn	3/234	0,664730932	0,81880213	0	0	0,881134902	0,359831645	RPS19;RPL10A;GOLGA7
Nimodipine-10_mg/kg_in_Corn_Oil-Rat-Heart-0.25d-up	3/234	0,664730932	0,818674092	0	0	0,881134902	0,359831645	RPS20;RPSA;TPT1
Probenecid-600_mg/kg_in_Corn_Oil-Rat-Kidney-1d-dn	3/234	0,664730932	0,818546094	0	0	0,881134902	0,359831645	ARL2BP;CLP1;RPS8
Acyclovir-330_mg/kg_in_Saline-Rat-Liver-1d-up	4/311	0,665774585	0,819703085	0	0	0,883968133	0,359601884	C1QA;CXCL11;CLP1;RPSA
Aspirin-167_mg/kg_in_Corn_Oil-Rat-Liver-3d-up	4/311	0,665774585	0,819574966	0	0	0,883968133	0,359601884	RPS17;RPS19;SEPP1;TPT1
Bromfenac-5_mg/kg_in_CMC-Rat-Kidney-5d-dn	4/311	0,665774585	0,819446887	0	0	0,883968133	0,359601884	RPS6;SEPP1;RPL39;TPT1
Cadmium_Chloride-2_mg/kg_in_Saline-Rat-Kidney-28d-dn	4/311	0,665774585	0,819318849	0	0	0,883968133	0,359601884	CLP1;RPS6;SEPP1;RPL39
Chloroform-60_mg/kg_in_Water-Rat-Liver-0.25d-up	4/311	0,665774585	0,81919085	0	0	0,883968133	0,359601884	RPS19;RPS6;RPL10A;SEPP1
Cyclosporin_A-350_mg/kg_in_Corn_Oil-Rat-Heart-5d-dn	4/311	0,665774585	0,819062892	0	0	0,883968133	0,359601884	RPS17;RPL32;RPS19;RPL35A
Cyclosporin_A-350_mg/kg_in_Corn_Oil-Rat-Liver-3d-dn	4/311	0,665774585	0,818934973	0	0	0,883968133	0,359601884	RPS17;RPS6;RPL10A;RPL39
Didanosine-500_uM_in_DMSO-Rat-Primary_rat_hepatocytes-0.67d-up	4/311	0,665774585	0,818807094	0	0	0,883968133	0,359601884	RPL10A;RPL39;TPT1;PDRG1
Lipopolysaccharide_E._Coli_O55:B5-1.25_mg/kg_in_Saline-Rat-Kidney-5d-dn	4/311	0,665774585	0,818679256	0	0	0,883968133	0,359601884	RPS17;RPL32;CLP1;RPS3A
Mefenamic_Acid-93_mg/kg_in_Corn_Oil-Rat-Liver-1d-dn	4/311	0,665774585	0,818551457	0	0	0,883968133	0,359601884	EEF1B2;RPL32;RPSA;RPL10A
Ofloxacin-30_mg/kg_in_Water-Rat-Heart-3d-dn	4/311	0,665774585	0,818423698	0	0	0,883968133	0,359601884	EEF1B2;CXCL11;RPL10A;RPL39
Promethazine-113_mg/kg_in_Saline-Rat-Liver-0.25d-dn	4/311	0,665774585	0,818295979	0	0	0,883968133	0,359601884	EEF1B2;RPS20;RPL39;PDRG1
Ofloxacin-3000_mg/kg_in_Water-Rat-Kidney-1d-up	5/387	0,666685716	0,819287985	0	0	0,887965405	0,360013617	CLP1;RPS8;RPS3A;SEPP1;TPT1
(+)-Pulegone-200_mg/kg_in_CMC-Rat-Kidney-3d-dn	4/312	0,668227154	0,821054145	0	0	0,881134902	0,355209368	RPS19;FUNDC1;RPS6;RPS20
1-Naphthyl_Isothiocyanate-30_mg/kg_in_Corn_Oil-Rat-Kidney-1d-up	4/312	0,668227154	0,820926075	0	0	0,881134902	0,355209368	RPS5;RAPGEF5;SEPP1;TPT1
Acyclovir-980_mg/kg_in_Saline-Rat-Liver-3d-dn	4/312	0,668227154	0,820798045	0	0	0,881134902	0,355209368	RPL32;RPS8;RPL39;TPT1
Diazepam-710_mg/kg_in_CMC-Rat-Heart-3d-up	4/312	0,668227154	0,820670056	0	0	0,881134902	0,355209368	EEF1B2;CLP1;RPS8;TPT1
Gabapentin-500_uM_in_DMSO-Rat-Primary_rat_hepatocytes-0.67d-up	4/312	0,668227154	0,820542106	0	0	0,881134902	0,355209368	EEF1B2;RPS6;RPS20;RPL39
Pravastatin-1200_mg/kg_in_Corn_Oil-Rat-Liver-5d-dn	4/312	0,668227154	0,820414196	0	0	0,881134902	0,355209368	EEF1B2;RPL32;CLP1;RPS8
Temafloxacin-1000_mg/kg_in_CMC-Rat-Liver-5d-dn	4/312	0,668227154	0,820286326	0	0	0,881134902	0,355209368	RPS17;RPL32;RPS19;SEPP1
Urethane-453_mg/kg_in_Water-Rat-Bone_marrow-3d-dn	4/312	0,668227154	0,820158496	0	0	0,881134902	0,355209368	ARL2BP;RPS3A;RPL39;TPT1
Sulfathiazole-2629_mg/kg_in_Water-Rat-Kidney-1d-up	5/388	0,668892143	0,820846762	0	0	0,885676834	0,356159398	CLP1;RPS8;RPS3A;SEPP1;TPT1
Gentamicin-656.6_uM_in_DMSO-Rat-Primary_rat_hepatocytes-1d-up	3/236	0,670343677	0,822499891	0	0	0,873667657	0,349436264	EEF1B2;RPSA;SEPP1
123-Trichloropropane-108_mg/kg_in_CMC-Rat-Heart-1d-up	4/313	0,670667436	0,82276896	0	0	0,878319774	0,350872843	EEF1B2;RPS17;ARL6IP5;RPSA
Mitomycin_C-1.7_mg/kg_in_Saline-Rat-Spleen-3d-up	4/313	0,670667436	0,822640823	0	0	0,878319774	0,350872843	CXCL11;RPS19;RAPGEF5;SEPP1
NN-Dimethylformamide-2100_mg/kg_in_Saline-Rat-Liver-0.25d-dn	4/313	0,670667436	0,822512725	0	0	0,878319774	0,350872843	RPS19;RPL10A;RPL39;GOLGA7
Oxaliplatin-8_mg/kg_in_Saline-Rat-Bone_marrow-1d-up	4/313	0,670667436	0,822384668	0	0	0,878319774	0,350872843	C1QA;NKG7;SEPP1;SLC16A3
Prednisone-68_mg/kg_in_CMC-Rat-Kidney-3d-dn	4/313	0,670667436	0,822256651	0	0	0,878319774	0,350872843	RPS17;RPS19;CLP1;SEPP1
Amiodarone-147_mg/kg_in_CMC-Rat-Liver-3d-up	4/314	0,673095414	0,825104977	0	0	0,875522578	0,346591534	C1QA;RPSA;RPS3A;GOLGA7
Amoxicillin-1100_mg/kg_in_Water-Rat-Liver-1d-up	4/314	0,673095414	0,824976576	0	0	0,875522578	0,346591534	C1QA;ARL2BP;RPL39;TPT1
Ampiroxicam-94_mg/kg_in_CMC-Rat-Kidney-3d-dn	4/314	0,673095414	0,824848215	0	0	0,875522578	0,346591534	ARL2BP;RPSA;RPL39;GOLGA7
Clotrimazole-178_mg/kg_in_Corn_Oil-Rat-Liver-5d-dn	4/314	0,673095414	0,824719894	0	0	0,875522578	0,346591534	EEF1B2;RPS8;RPSA;SEPP1
Emetine-1_mg/kg_in_Saline-Rat-Heart-3d-dn	4/314	0,673095414	0,824591613	0	0	0,875522578	0,346591534	RPS17;RPL32;RPS19;RPS6
Ergocalciferol-15_mg/kg_in_CMC-Rat-Heart-1d-dn	4/314	0,673095414	0,824463371	0	0	0,875522578	0,346591534	FUNDC1;RPS6;RPS20;SEPP1
Fenoprofen-52_mg/kg_in_Corn_Oil-Rat-Kidney-5d-dn	4/314	0,673095414	0,82433517	0	0	0,875522578	0,346591534	EEF1B2;CLP1;SEPP1;RPL39
Hydralazine-280_mg/kg_in_Water-Rat-Liver-3d-up	4/314	0,673095414	0,824207008	0	0	0,875522578	0,346591534	RPS8;RPSA;RPS3A;TPT1
Lead(IV)_Acetate-200_mg/kg_in_Saline-Rat-Spleen-0.25d-up	4/314	0,673095414	0,824078887	0	0	0,875522578	0,346591534	C1QA;NKG7;RPL10A;SEPP1
Perhexiline-320_mg/kg_in_CMC-Rat-Liver-1d-dn	4/314	0,673095414	0,823950805	0	0	0,875522578	0,346591534	RPS19;RPS6;RPL39;TPT1
Sparfloxacin-29_mg/kg_in_Corn_Oil-Rat-Liver-0.25d-dn	4/314	0,673095414	0,823822763	0	0	0,875522578	0,346591534	C1QA;RPS5;RPSA;RPL39
Sulfathiazole-31_mg/kg_in_Water-Rat-Liver-0.25d-up	4/314	0,673095414	0,82369476	0	0	0,875522578	0,346591534	RPS6;RPS20;RPL10A;SEPP1
Acrolein-20_mg/kg_in_Water-Rat-Kidney-1d-dn	3/237	0,673124241	0,82360207	0	0	0,869981295	0,344360658	RPS17;RPL32;RPL35A
Omeprazole-400_uM_in_DMSO-Rat-Primary_rat_hepatocytes-1d-up	3/237	0,673124241	0,823474141	0	0	0,869981295	0,344360658	RPS17;RPS8;RPS3A
Ergocalciferol-15_mg/kg_in_CMC-Rat-Kidney-5d-dn	4/315	0,675511074	0,826265758	0	0	0,872743141	0,342364679	CLP1;SEPP1;GOLGA7;TPT1
Lacidipine-14_mg/kg_in_Corn_Oil-Rat-Heart-3d-dn	4/315	0,675511074	0,826137456	0	0	0,872743141	0,342364679	ARL2BP;RPS8;ARL6IP5;RPSA
Megestrol_Acetate-132_mg/kg_in_CMC-Rat-Liver-1d-up	4/315	0,675511074	0,826009194	0	0	0,872743141	0,342364679	RPSA;RPL10A;RPL39;TPT1
Methotrexate-27_mg/kg_in_Corn_Oil-Rat-Liver-3d-dn	4/315	0,675511074	0,825880971	0	0	0,872743141	0,342364679	RPS19;RPS6;RPL10A;RPL39
NN-Dimethylformamide-1400_mg/kg_in_Saline-Rat-Kidney-5d-dn	4/315	0,675511074	0,825752789	0	0	0,872743141	0,342364679	EEF1B2;ARL2BP;RPS8;SEPP1
Sulfathiazole-2629_mg/kg_in_Water-Rat-Kidney-3d-up	4/315	0,675511074	0,825624646	0	0	0,872743141	0,342364679	CLP1;RPS3A;SEPP1;TPT1
Trans-Platinum(II)Diammine_Dichloride-0.5_mg/kg_in_Saline-Rat-Kidney-5d-up	4/315	0,675511074	0,825496543	0	0	0,872743141	0,342364679	RPS6;RPL10A;RPL39;TPT1
Chlorpromazine-18_mg/kg_in_Water-Rat-Liver-0.25d-dn	3/238	0,675887604	0,825828541	0	0	0,866325912	0,339364535	RPL39;SEPP1;TPT1
Diphenhydramine-10.9_mg/kg_in_Water-Rat-Heart-3d-dn	3/238	0,675887604	0,825700446	0	0	0,866325912	0,339364535	EEF1B2;CXCL11;COL5A3
Fluvastatin-94_mg/kg_in_Corn_Oil-Rat-Kidney-3d-dn	3/238	0,675887604	0,82557239	0	0	0,866325912	0,339364535	CLP1;RPL10A;RPL39
Atorvastatin-2.5_mg/kg_in_Corn_Oil-Rat-Liver-0.25d-dn	4/316	0,677914403	0,827919653	0	0	0,869981295	0,338191524	CLP1;RPL35A;SEPP1;TPT1
Doxapram-2_mg/kg_in_Saline-Rat-Liver-3d-dn	4/316	0,677914403	0,827791293	0	0	0,869981295	0,338191524	RPS3A;RPL10A;SEPP1;TPT1
Ifosfamide-11000_uM_in_DMSO-Rat-Primary_rat_hepatocytes-0.67d-up	4/316	0,677914403	0,827662973	0	0	0,869981295	0,338191524	EEF1B2;ARL6IP5;SEPP1;GOLGA7
Nevirapine-200_mg/kg_in_Saline-Rat-Liver-0.25d-dn	4/316	0,677914403	0,827534693	0	0	0,869981295	0,338191524	EEF1B2;RPS8;RPS20;GOLGA7
Nitrazepam-310_mg/kg_in_CMC-Rat-Liver-5d-up	4/316	0,677914403	0,827406453	0	0	0,869981295	0,338191524	RPS17;RPS20;RPSA;RPL39
Phenothiazine-36_mg/kg_in_Corn_Oil-Rat-Liver-5d-up	4/316	0,677914403	0,827278252	0	0	0,869981295	0,338191524	RPL32;SEPP1;RPL39;TPT1
Piclamilast-17_mg/kg_in_CMC-Rat-Liver-5d-dn	4/316	0,677914403	0,827150092	0	0	0,869981295	0,338191524	RPL32;RPS19;RPS6;SEPP1
(+)-Pulegone-200_mg/kg_in_CMC-Rat-Kidney-1d-dn	4/317	0,680305391	0,829938856	0	0	0,867236875	0,334071332	EEF1B2;RPL32;RPS19;RPS6
4-Octylphenol-50_mg/kg_in_Corn_Oil-Rat-Liver-1d-up	4/317	0,680305391	0,829810323	0	0	0,867236875	0,334071332	CLP1;RPSA;SEPP1;RPL39
Diazepam-710_mg/kg_in_CMC-Rat-Liver-5d-up	4/317	0,680305391	0,82968183	0	0	0,867236875	0,334071332	RPS19;RPS20;RPSA;RPL10A
Doxorubicin-3_mg/kg_in_Saline-Rat-Spleen-5d-dn	4/317	0,680305391	0,829553376	0	0	0,867236875	0,334071332	C1QA;RPSA;RPL10A;RPL39
Fluorouracil-29_mg/kg_in_CMC-Rat-Spleen-5d-dn	4/317	0,680305391	0,829424962	0	0	0,867236875	0,334071332	RPS17;ARL6IP5;RPS3A;TPT1
Phalloidin-1_uM_in_DMSO-Rat-Primary_rat_hepatocytes-0.67d-dn	4/317	0,680305391	0,829296588	0	0	0,867236875	0,334071332	RPL32;FUNDC1;SEPP1;TPT1
Procarbazine-27_mg/kg_in_Water-Rat-Bone_marrow-3d-up	4/317	0,680305391	0,829168254	0	0	0,867236875	0,334071332	C1QA;NKG7;RPL10A;SEPP1
Streptozotocin-138_mg/kg_in_Saline-Rat-Liver-1d-up	4/317	0,680305391	0,829039959	0	0	0,867236875	0,334071332	RPS17;RPL32;RPS20;RPSA
Cadmium_Chloride-66_mg/kg_in_Corn_Oil-Rat-Kidney-5d-dn	3/240	0,681362756	0,830200041	0	0	0,859106529	0,329605184	CLP1;SEPP1;TPT1
Benzocaine-1100_mg/kg_in_Corn_Oil-Rat-Liver-5d-dn	4/318	0,682684026	0,831681266	0	0	0,864509715	0,330003375	EEF1B2;RPS19;ARL6IP5;RPSA
Clofibrate-130_mg/kg_in_Corn_Oil-Rat-Kidney-0.25d-up	4/318	0,682684026	0,831552642	0	0	0,864509715	0,330003375	FUNDC1;RPS20;SEPP1;RPL39
Dicyclomine-645_mg/kg_in_Water-Rat-Heart-5d-dn	4/318	0,682684026	0,831424058	0	0	0,864509715	0,330003375	RPS8;COL5A3;RPS20;RPSA
Meropenem-1500_mg/kg_in_CMC-Rat-Spleen-5d-dn	4/318	0,682684026	0,831295514	0	0	0,864509715	0,330003375	RPS17;ARL6IP5;RPS3A;TPT1
Pentoxifylline-1170_mg/kg_in_CMC-Rat-Heart-5d-dn	4/318	0,682684026	0,83116701	0	0	0,864509715	0,330003375	RPS19;RPS8;RPS6;SEPP1
Raloxifene-650_mg/kg_in_Corn_Oil-Rat-Liver-5d-dn	4/318	0,682684026	0,831038545	0	0	0,864509715	0,330003375	RPS17;FEM1B;RPL35A;SEPP1
Rifampin-125_uM_in_DMSO-Rat-Primary_rat_hepatocytes-0.67d-dn	3/241	0,684074562	0,832602573	0	0	0,855541772	0,324839251	RPS17;CLP1;ARL6IP5
Chlorambucil-0.6_mg/kg_in_Corn_Oil-Rat-Liver-1d-dn	4/319	0,685050299	0,833661334	0	0	0,861799653	0,325986935	RPS8;RPSA;RPL39;TPT1
Cytarabine-487_mg/kg_in_Saline-Rat-Kidney-5d-dn	4/319	0,685050299	0,833532543	0	0	0,861799653	0,325986935	RPL32;RPL10A;RPL39;GOLGA7
Pentobarbital-70_mg/kg_in_Water-Rat-Liver-3d-up	4/319	0,685050299	0,833403793	0	0	0,861799653	0,325986935	RPS19;CLP1;RPSA;RPS3A
Ouabain-144_mg/kg_in_CMC-Rat-Heart-1d-dn	3/242	0,686769204	0,835365907	0	0	0,852006475	0,32014739	RPS8;SEPP1;TPT1
33'4'5-Tetrachlorosalicylanilide-5_mg/kg_in_Corn_Oil-Rat-Liver-1d-dn	4/320	0,687404203	0,836009188	0	0	0,859106529	0,322021306	EEF1B2;RPS17;RPS20;RPS3A
Amitriptyline-160_mg/kg_in_Water-Rat-Kidney-3d-up	4/320	0,687404203	0,835880114	0	0	0,859106529	0,322021306	EEF1B2;CLP1;SEPP1;TPT1

Carboplatin-14_mg/kg_in_Saline-Rat-Liver-0.25d-dn	4/320	0,687404203	0,835751081	0	0	0,859106529	0,322021306	RPS19;RPSA;RPL10A;SEPP1
Chlorpromazine-18_mg/kg_in_Water-Rat-Liver-5d-up	4/320	0,687404203	0,835622087	0	0	0,859106529	0,322021306	C1QA;RPL10A;SEPP1;RPL39
Chlorzoxazone-763_mg/kg_in_CMC-Rat-Liver-5d-dn	4/320	0,687404203	0,835493133	0	0	0,859106529	0,322021306	EEF1B2;C1QA;SEPP1;RPL39
Terazosin-657_mg/kg_in_Water-Rat-Heart-1d-dn	4/320	0,687404203	0,835364219	0	0	0,859106529	0,322021306	EEF1B2;COL5A3;SEPP1;RPL39
Lead_(II)_Acetate-200_mg/kg_in_Saline-Rat-Liver-5d-up	3/243	0,689446695	0,837717089	0	0	0,848500276	0,315528313	C1QA;RPS8;RPS6
6-Methoxy-2-Naphthylacetic_Acid-360_mg/kg_in_Saline-Rat-Kidney-0.25d-dn	4/321	0,689745731	0,837951161	0	0	0,856430185	0,318105795	R3HCC1;RPS6;RPL39;TPT1
Eperisone-501_mg/kg_in_CMC-Rat-Liver-1d-dn	4/321	0,689745731	0,837821927	0	0	0,856430185	0,318105795	C1QA;RPL32;CLP1;RPS6
Fluorouracil-29_mg/kg_in_CMC-Rat-Spleen-3d-dn	4/321	0,689745731	0,837692733	0	0	0,856430185	0,318105795	NKG7;RPS20;TPT1;PDRG1
Sulindac-132_mg/kg_in_Corn_Oil-Rat-Liver-0.25d-dn	4/321	0,689745731	0,837563579	0	0	0,856430185	0,318105795	RPS17;RPL32;RPS8;TPT1
Warfarin-0.25_mg/kg_in_CMC-Rat-Liver-3d-dn	4/321	0,689745731	0,837434465	0	0	0,856430185	0,318105795	RPS19;CLP1;FEM1B;SEPP1
Aspirin-35_mg/kg_in_Corn_Oil-Rat-Kidney-0.25d-up	4/322	0,692074877	0,84013282	0	0	0,853770464	0,314239718	RPS20;SEPP1;RPL39;TPT1
Doxorubicin-1.5_uM_in_DMSO-Rat-Primary_rat_hepatocytes-1d-dn	4/322	0,692074877	0,840003349	0	0	0,853770464	0,314239718	ARL6IP5;RPS3A;RPL39;SLC16A3
Pioglitazone-1500_mg/kg_in_Corn_Oil-Rat-Heart-5d-up	4/322	0,692074877	0,839873919	0	0	0,853770464	0,314239718	RPSA;RAPGEF5;RPL39;TPT1
Promethazine-2.3_mg/kg_in_Saline-Rat-Liver-1d-up	4/322	0,692074877	0,839744528	0	0	0,853770464	0,314239718	C1QA;RPS19;RPSA;TPT1
Indomethacin-9.6_mg/kg_in_Water-Rat-Kidney-3d-dn	3/244	0,692107053	0,839654213	0	0	0,845022816	0,310980763	ARL6IP5;RPSA;SEPP1
Secobarbital-20_mg/kg_in_Water-Rat-Liver-0.25d-dn	3/244	0,692107053	0,839524896	0	0	0,845022816	0,310980763	RPS3A;RPL39;TPT1
Butenafine-150_mg/kg_in_Corn_Oil-Rat-Liver-3d-dn	4/323	0,694391638	0,842166391	0	0	0,851127212	0,310422399	C1QA;RPL35A;RPSA;TPT1
Ethacrynic_Acid-125_mg/kg_in_CMC-Rat-Kidney-3d-dn	4/323	0,694391638	0,842036727	0	0	0,851127212	0,310422399	RPL32;FEM1B;RPS20;SEPP1
Ethanol-6000_mg/kg_in_Saline-Rat-Bone_marrow-3d-up	4/323	0,694391638	0,841907103	0	0	0,851127212	0,310422399	RPS19;FEM1B;RPL10A;SEPP1
Indomethacin-12_mg/kg_in_Water-Rat-Liver-1d-dn	4/323	0,694391638	0,841777519	0	0	0,851127212	0,310422399	RPL32;CLP1;SEPP1;TPT1
Omeprazole-415_mg/kg_in_Corn_Oil-Rat-Liver-0.25d-dn	4/323	0,694391638	0,841647975	0	0	0,851127212	0,310422399	C1QA;RPS17;RPSA;SEPP1
Levofloxacin-739_mg/kg_in_Water-Rat-Kidney-1d-up	5/400	0,694621285	0,841796775	0	0	0,859106529	0,313048536	RPS8;FUNDC1;HOXA1;SEPP1;TPT1
Rifampin-125_uM_in_DMSO-Rat-Primary_rat_hepatocytes-1d-up	3/245	0,694750294	0,841823587	0	0	0,841573743	0,306503503	RPS20;RPS3A;SLC16A3
Aceclofenac-9_mg/kg_in_Corn_Oil-Rat-Kidney-1d-up	4/324	0,696696011	0,844051343	0	0	0,848500276	0,306653178	RPS19;RPSA;RPL39;TPT1
Gliclazide-1500_mg/kg_in_CMC-Rat-Kidney-3d-up	4/324	0,696696011	0,843921529	0	0	0,848500276	0,306653178	RPS17;RPL39;TOMM20;TPT1
Granisetron-175_mg/kg_in_Water-Rat-Liver-3d-dn	4/324	0,696696011	0,843791755	0	0	0,848500276	0,306653178	EEF1B2;C1QA;CLP1;SEPP1
Leflunomide-30_mg/kg_in_Corn_Oil-Rat-Spleen-3d-dn	4/324	0,696696011	0,843662021	0	0	0,848500276	0,306653178	C1QA;RPS17;RPS19;RPL10A
N-Nitrosodiethylamine-1.67_mg/kg_in_Saline-Rat-Liver-3d-dn	4/324	0,696696011	0,843532326	0	0	0,848500276	0,306653178	C1QA;RPL32;RPS8;TPT1
NN-Dimethylformamide-1400_mg/kg_in_Saline-Rat-Kidney-3d-dn	4/324	0,696696011	0,843402672	0	0	0,848500276	0,306653178	EEF1B2;RPS8;RPSA;RPL10A
Cytochalasin_B-167_uM_in_DMSO-Rat-Primary_rat_hepatocytes-0.67d-up	3/246	0,697376438	0,844096639	0	0	0,838152711	0,302095323	RPS17;RPS3A;TPT1
Gentamicin-267_mg/kg_in_Corn_Oil-Rat-Kidney-3d-dn	3/246	0,697376438	0,843966938	0	0	0,838152711	0,302095323	GOLGA7;SEPP1;TPT1
Angiotensin_II_Human-0.72_mg/kg_in_Saline-Rat-Heart-3d-up	4/325	0,698987993	0,845787285	0	0	0,845889506	0,302931399	RPS8;FUNDC1;RAPGEF5;RPL39
Beta-Estradiol-150_mg/kg_in_Corn_Oil-Rat-Kidney-3d-dn	4/325	0,698987993	0,845657363	0	0	0,845889506	0,302931399	RPS19;SEPP1;RPL39;GOLGA7
Beta-Estradiol-150_mg/kg_in_Corn_Oil-Rat-Kidney-5d-dn	4/325	0,698987993	0,845527482	0	0	0,845889506	0,302931399	FEM1B;SEPP1;GOLGA7;TPT1
Carbon_Tetrachloride-400_mg/kg_in_Corn_Oil-Rat-Kidney-1d-up	4/325	0,698987993	0,845397641	0	0	0,845889506	0,302931399	RPS19;RPS5;RPL10A;SEPP1
Norethindrone-375_mg/kg_in_Corn_Oil-Rat-Liver-3d-dn	4/325	0,698987993	0,845267839	0	0	0,845889506	0,302931399	C1QA;SEPP1;RPL39;TPT1
Piracetam-2500_mg/kg_in_CMC-Rat-Brain-5d-up	4/325	0,698987993	0,845138077	0	0	0,845889506	0,302931399	FEM1B;RPSA;SEPP1;RPL39
Lead_(II)_Acetate-200_mg/kg_in_Saline-Rat-Kidney-1d-dn	3/247	0,699985507	0,846214253	0	0	0,834759381	0,297755038	ARL2BP;RPS6;SEPP1
Benoxaprofen-20_mg/kg_in_CMC-Rat-Liver-3d-up	4/326	0,701267585	0,847634055	0	0	0,843294753	0,299256421	RPSA;SEPP1;GOLGA7;TPT1
Nevirapine-200_mg/kg_in_Saline-Rat-Liver-5d-up	4/326	0,701267585	0,847503989	0	0	0,843294753	0,299256421	RPS8;RPS20;RPSA;RPL39
Stanozolol-150_mg/kg_in_Water-Rat-Liver-3d-up	4/326	0,701267585	0,847373964	0	0	0,843294753	0,299256421	RPS19;RPS6;RPL10A;SEPP1
Sulindac-750_uM_in_DMSO-Rat-Primary_rat_hepatocytes-1d-up	3/248	0,702577524	0,848826596	0	0	0,831393415	0,293481485	CLP1;FEM1B;RPL39
Bisphenol_A-610_mg/kg_in_Corn_Oil-Rat-Liver-1d-dn	4/327	0,703534786	0,849852757	0	0	0,84071587	0,29562761	RPS6;RPS20;RPSA;RPS3A
Hydrocortisone-56_mg/kg_in_Corn_Oil-Rat-Heart-1d-up	4/327	0,703534786	0,849722431	0	0	0,84071587	0,29562761	C1QA;ARL6IP5;SEPP1;PDRG1
Lead_(II)_Acetate-600_mg/kg_in_Saline-Rat-Liver-1d-up	4/327	0,703534786	0,849592146	0	0	0,84071587	0,29562761	C1QA;CLP1;SEPP1;RPL39
Nimodipine-1100_mg/kg_in_Corn_Oil-Rat-Heart-1d-up	4/327	0,703534786	0,8494619	0	0	0,84071587	0,29562761	RPS3A;SEPP1;RPL39;TPT1
2378-Tetrachlorodibenzo-P-Dioxin-0.02_mg/kg_in_CMC-Rat-Liver-1d-up	4/328	0,705789599	0,852053783	0	0	0,838152711	0,292044342	C1QA;RPSA;RPL10A;SEPP1
Nevirapine-200_mg/kg_in_Saline-Rat-Liver-1d-up	4/328	0,705789599	0,8519232	0	0	0,838152711	0,292044342	CXCL11;RPS19;RPS20;RPL39
Nevirapine-29_mg/kg_in_Saline-Rat-Liver-1d-up	4/328	0,705789599	0,851792657	0	0	0,838152711	0,292044342	C1QA;RPS17;CLP1;RPL39
Aminocaproic_Acid-2000_mg/kg_in_Water-Rat-Kidney-5d-dn	3/250	0,7077105	0,853980067	0	0	0,824742268	0,285130035	RPS19;CLP1;TPT1
Camptothecin-2.5_mg/kg_in_Saline-Rat-Bone_marrow-1d-dn	3/250	0,7077105	0,853849249	0	0	0,824742268	0,285130035	RPS8;FUNDC1;RPS3A
Carbon_Tetrachloride-400_mg/kg_in_Corn_Oil-Rat-Kidney-0.25d-dn	3/250	0,7077105	0,853718471	0	0	0,824742268	0,285130035	ARL2BP;RPS8;TPT1
Glycine-2000_mg/kg_in_CMC-Rat-Kidney-1d-dn	3/250	0,7077105	0,853587733	0	0	0,824742268	0,285130035	RPS3A;RPL39;TPT1
Penicillamine-100_mg/kg_in_Water-Rat-Liver-3d-up	3/250	0,7077105	0,853457035	0	0	0,824742268	0,285130035	CLP1;RPS8;RPS3A
Carbon_Tetrachloride-1175_mg/kg_in_Corn_Oil-Rat-Kidney-3d-dn	4/329	0,708032025	0,853714058	0	0	0,835605135	0,288506004	RPSA;RPL10A;SEPP1;TPT1
Hydrochlorothiazide-1500_mg/kg_in_CMC-Rat-Kidney-1d-up	4/329	0,708032025	0,853583381	0	0	0,835605135	0,288506004	RPL32;RPS20;RAPGEF5;TPT1
Penciclovir-1200_mg/kg_in_Corn_Oil-Rat-Kidney-1d-dn	4/329	0,708032025	0,853452744	0	0	0,835605135	0,288506004	RPS6;RPS20;RPS3A;SEPP1
Diethylstilbestrol-120_uM_in_DMSO-Rat-Primary_rat_hepatocytes-1d-up	3/251	0,710251514	0,855997081	0	0	0,821456442	0,281049925	EEF1B2;RPS20;PDRG1
Leflunomide-30_mg/kg_in_Corn_Oil-Rat-Liver-0.25d-dn	3/251	0,710251514	0,855866115	0	0	0,821456442	0,281049925	C1QA;RPL39;TPT1
Pyrogallol-1000_mg/kg_in_Water-Rat-Liver-3d-dn	3/251	0,710251514	0,855735188	0	0	0,821456442	0,281049925	EEF1B2;C1QA;SEPP1
Zopiclone-414_mg/kg_in_Water-Rat-Liver-0.25d-dn	3/251	0,710251514	0,855604302	0	0	0,821456442	0,281049925	EEF1B2;RPL39;SEPP1
Clotrimazole-52_mg/kg_in_Corn_Oil-Rat-Liver-5d-dn	4/330	0,710262069	0,855486168	0	0	0,833072998	0,285011989	C1QA;CLP1;RPS6;SEPP1
Piracetam-2500_mg/kg_in_CMC-Rat-Brain-3d-up	4/330	0,710262069	0,85535536	0	0	0,833072998	0,285011989	ARL2BP;RPS8;RPSA;TPT1
Rifampin-99_mg/kg_in_CMC-Rat-Liver-1d-up	4/330	0,710262069	0,855224592	0	0	0,833072998	0,285011989	RPS19;RPL10A;SEPP1;GOLGA7
Trihexyphenidyl-588_mg/kg_in_CMC-Rat-Heart-3d-dn	4/330	0,710262069	0,855093863	0	0	0,833072998	0,285011989	ARL2BP;RPS8;RPS20;RPL39
Diethylstilbestrol-280_mg/kg_in_Corn_Oil-Rat-Liver-3d-dn	4/331	0,712479735	0,857632645	0	0	0,830556161	0,281561702	C1QA;RPL32;RPL35A;RPL39
Doxorubicin-0.65_mg/kg_in_Saline-Rat-Liver-5d-up	4/331	0,712479735	0,857501589	0	0	0,830556161	0,281561702	RPL32;RPS19;SEPP1;TPT1
Enoxacin-100_mg/kg_in_Corn_Oil-Rat-Liver-0.25d-dn	4/331	0,712479735	0,857370572	0	0	0,830556161	0,281561702	RPS17;RPSA;RPL39;TPT1
Indomethacin-4.5_mg/kg_in_Water-Rat-Kidney-3d-up	4/331	0,712479735	0,857239596	0	0	0,830556161	0,281561702	ARL2BP;CLP1;RPS20;GOLGA7
L-Buthionine_Sulfoximine-100000_uM_in_DMSO-Rat-Primary_rat_hepatocytes-0.67d-dn	4/331	0,712479735	0,85710866	0	0	0,830556161	0,281561702	RPS6;RPSA;SEPP1;TPT1
Azathioprine-160_mg/kg_in_Corn_Oil-Rat-Liver-3d-up	3/252	0,712775585	0,857333614	0	0	0,818196694	0,277032119	RPS8;RPS20;SEPP1
Isotretinoin-13_mg/kg_in_Corn_Oil-Rat-Liver-1d-up	3/252	0,712775585	0,857202704	0	0	0,818196694	0,277032119	RPS3A;RPL39;TPT1
Nimesulide-162_mg/kg_in_Corn_Oil-Rat-Liver-0.25d-up	3/252	0,712775585	0,857071833	0	0	0,818196694	0,277032119	C1QA;RPS17;RPS3A
Tamoxifen-2.5_mg/kg_in_Corn_Oil-Rat-Liver-0.25d-up	4/332	0,71468503	0,859236651	0	0	0,828054486	0,278154557	EEF1B2;RPS6;ARL6IP5;SEPP1
Clarithromycin-56_mg/kg_in_Water-Rat-Liver-3d-dn	3/253	0,715282743	0,859824006	0	0	0,814962715	0,273075563	RPSA;RPL39;TPT1
Fluocinolone_Acetonide-2.5_mg/kg_in_Corn_Oil-Rat-Liver-3d-dn	3/253	0,715282743	0,859692795	0	0	0,814962715	0,273075563	RPL39;GOLGA7;SEPP1
Neostigmine_Bromide-40_mg/kg_in_Water-Rat-Liver-1d-dn	3/253	0,715282743	0,859561624	0	0	0,814962715	0,273075563	CLP1;RPS8;RPL39
Pravastatin-1200_mg/kg_in_Corn_Oil-Rat-Skeletal_muscle-5d-up	3/253	0,715282743	0,859430494	0	0	0,814962715	0,273075563	RPS17;RPS6;RPS20
Leflunomide-60_mg/kg_in_Corn_Oil-Rat-Spleen-3d-dn	4/333	0,716877961	0,861215805	0	0	0,825567836	0,274789974	C1QA;RPS19;ARL2BP;GOLGA7
Pyridine-223_mg/kg_in_Water-Rat-Kidney-5d-up	4/333	0,716877961	0,861084462	0	0	0,825567836	0,274789974	CLP1;RAPGEF5;TOMM20;TPT1
Lead(IV)_Acetate-600_mg/kg_in_Saline-Rat-Spleen-1d-up	4/334	0,719058535	0,863571976	0	0	0,823096076	0,271467385	RPS19;RPL10A;SEPP1;RPL39
Leflunomide-30_mg/kg_in_Corn_Oil-Rat-Kidney-0.25d-dn	3/255	0,720246455	0,86486676	0	0	0,808570851	0,265342088	RPS19;CLP1;RPS20
Meloxicam-33_mg/kg_in_Corn_Oil-Rat-Kidney-3d-dn	4/335	0,721226761	0,865911886	0	0	0,820639073	0,268186229	RPS17;RPS19;CLP1;SEPP1
Azathioprine-54_mg/kg_in_Water-Rat-Kidney-5d-dn	3/256	0,722703078	0,867552118	0	0	0,805412371	0,261563161	RPL32;RPL10A;SEPP1
Gemcitabine-11_mg/kg_in_Saline-Rat-Bone_marrow-3d-dn	3/256	0,722703078	0,867419909	0	0	0,805412371	0,261563161	NKG7;RPS20;TPT1
Methotrexate-0.3_mg/kg_in_Corn_Oil-Rat-Liver-3d-dn	3/256	0,722703078	0,867287741	0	0	0,805412371	0,261563161	RPS17;CLP1;RPS3A
Neomycin-877_mg/kg_in_Corn_Oil-Rat-Kidney-1d-up	4/336	0,72338265	0,867971016	0	0	0,818196694	0,264945953	RPS6;RAPGEF5;RPL39;GOLGA7
44'-Methylenedianiline-81_mg/kg_in_Corn_Oil-Rat-Kidney-1d-up	3/257	0,725142929	0,869950603	0	0	0,802278471	0,25784147	RPS17;CLP1;RPS3A
Cyclosporin_A-8.5_uM_in_DMSO-Rat-Primary_rat_hepatocytes-0.67d-dn	3/257	0,725142929	0,86981811	0	0	0,802278471	0,25784147	RPS17;ARL6IP5;RPS3A
Doxorubicin-3_mg/kg_in_Saline-Rat-Kidney-1d-dn	3/257	0,725142929	0,869685657	0	0	0,802278471	0,25784147	ARL2BP;CLP1;RPS20

Lead_(II)_Acetate-600_mg/kg_in_Saline-Rat-Kidney-1d-dn	3/257	0,725142929	0,869553244	0	0	0,802278471	0,25784147	EEF1B2;RPL32;ARL2BP
Sparteine-2222_uM_in_DMSO-Rat-Primary_rat_hepatocytes-1d-up	3/257	0,725142929	0,869420872	0	0	0,802278471	0,25784147	RPL32;RPL10A;PDRG1
Sulfamethoxazole-1000_mg/kg_in_Water-Rat-Kidney-3d-dn	3/257	0,725142929	0,86928854	0	0	0,802278471	0,25784147	EEF1B2;ARL6IP5;SEPP1
Altretamine-40_mg/kg_in_Corn_Oil-Rat-Spleen-5d-dn	4/337	0,725526212	0,869615651	0	0	0,815768811	0,261746013	RPS17;RPS5;RPL13A;RPL35A
Amitriptyline-160_mg/kg_in_Water-Rat-Heart-3d-dn	3/258	0,727566045	0,871927902	0	0	0,799168864	0,254176057	EEF1B2;RPS8;TPT1
Bisphenol_A-100_mg/kg_in_Corn_Oil-Rat-Liver-3d-up	3/258	0,727566045	0,871795249	0	0	0,799168864	0,254176057	RPS8;RPS20;SEPP1
Cephalexin-2500_mg/kg_in_CMC-Rat-Kidney-5d-dn	3/258	0,727566045	0,871662636	0	0	0,799168864	0,254176057	EEF1B2;RPS17;RPS3A
Cobalt_(II)_Chloride-10_mg/kg_in_Saline-Rat-Kidney-14d-dn	4/338	0,727657459	0,871639566	0	0	0,813355294	0,258585872	C1QA;RPS19;SEPP1;GOLGA7
Isoniazid-50_mg/kg_in_Saline-Rat-Liver-1d-up	4/338	0,727657459	0,871507017	0	0	0,813355294	0,258585872	EEF1B2;RPS17;RPL32;SEPP1
Mitomycin_C-0.5_mg/kg_in_Saline-Rat-Liver-0.25d-dn	4/338	0,727657459	0,871374509	0	0	0,813355294	0,258585872	EEF1B2;RPS19;RPS6;RPS20
Beta-Estradiol_3-Benzoate-25_mg/kg_in_Corn_Oil-Rat-Liver-1d-dn	4/339	0,729776404	0,873779106	0	0	0,810956016	0,255465002	C1QA;RPS20;SEPP1;TPT1
Didanosine-500_uM_in_DMSO-Rat-Primary_rat_hepatocytes-1d-up	4/339	0,729776404	0,873646292	0	0	0,810956016	0,255465002	RPL32;RPS5;RPL39;TPT1
Lead_(II)_Acetate-600_mg/kg_in_Saline-Rat-Spleen-3d-up	4/339	0,729776404	0,873513519	0	0	0,810956016	0,255465002	RPS19;NKG7;SEPP1;RPL39
4-Methylpyrazole-155_mg/kg_in_Saline-Rat-Liver-1d-dn	3/259	0,729972467	0,873615431	0	0	0,79608327	0,250565985	CLP1;SEPP1;TPT1
Cobalt(II)_Acetylacetonate-100_mg/kg_in_Corn_Oil-Rat-Kidney-1d-dn	3/259	0,729972467	0,873482703	0	0	0,79608327	0,250565985	CLP1;SEPP1;TPT1
Hydrazine-45_mg/kg_in_Water-Rat-Liver-0.25d-dn	3/259	0,729972467	0,873350015	0	0	0,79608327	0,250565985	RPS17;RPL35A;RPL39
Amikacin-82_mg/kg_in_Saline-Rat-Kidney-1d-dn	3/260	0,732362235	0,876076088	0	0	0,793021412	0,247010333	RPS19;CLP1;GOLGA7
Ibufenac-500_mg/kg_in_Corn_Oil-Rat-Liver-3d-up	3/260	0,732362235	0,875943047	0	0	0,793021412	0,247010333	RPS8;RPS3A;TPT1
Ibuprofen-263_mg/kg_in_Corn_Oil-Rat-Kidney-5d-dn	3/260	0,732362235	0,875810046	0	0	0,793021412	0,247010333	ARL2BP;SEPP1;PDRG1
Famciclovir-112_mg/kg_in_Saline-Rat-Spleen-3d-up	4/341	0,733977444	0,877608373	0	0	0,806199676	0,249339001	RPS19;RPS20;SEPP1;RPL39
Ifosfamide-143_mg/kg_in_Saline-Rat-Liver-1d-up	4/341	0,733977444	0,87747516	0	0	0,806199676	0,249339001	C1QA;RPSA;RPL39;TPT1
Doxepin-147_mg/kg_in_CMC-Rat-Heart-1d-up	3/261	0,734735393	0,878247982	0	0	0,789983015	0,243508199	RPS19;RPS3A;TPT1
Indomethacin-5_mg/kg_in_Corn_Oil-Rat-Liver-1d-up	3/261	0,734735393	0,878114712	0	0	0,789983015	0,243508199	RPS19;RPS8;SEPP1
Vincristine-0.05_mg/kg_in_Saline-Rat-Heart-3d-dn	3/261	0,734735393	0,877981483	0	0	0,789983015	0,243508199	RPS3A;GOLGA7;SEPP1
Fenoprofen-52_mg/kg_in_Corn_Oil-Rat-Kidney-0.25d-up	4/342	0,73605957	0,879430396	0	0	0,803842367	0,246332852	RPL32;RPS20;SEPP1;TPT1
Pravastatin-1200_mg/kg_in_Corn_Oil-Rat-Kidney-5d-up	4/342	0,73605957	0,879297008	0	0	0,803842367	0,246332852	FUNDC1;RPS20;RAPGEF5;SEPP1
Ethylene_Glycol-3525_mg/kg_in_Water-Rat-Liver-3d-dn	3/262	0,737091983	0,880396794	0	0	0,786967813	0,240058697	EEF1B2;RPL32;SEPP1
Nortriptyline-63_mg/kg_in_Water-Rat-Heart-3d-up	3/262	0,737091983	0,880263299	0	0	0,786967813	0,240058697	RPS19;RPS6;HOXA1
Simvastatin-160_uM_in_DMSO-Rat-Primary_rat_hepatocytes-1d-up	3/262	0,737091983	0,880129845	0	0	0,786967813	0,240058697	EEF1B2;RPL39;SEPP1
Sulfadoxine-380_mg/kg_in_Corn_Oil-Rat-Liver-3d-up	3/262	0,737091983	0,879996432	0	0	0,786967813	0,240058697	RPS20;RPL39;SEPP1
Fluoxetine-52_mg/kg_in_CMC-Rat-Kidney-3d-up	3/263	0,739432051	0,882656386	0	0	0,78397554	0,236660958	RPL32;RPS3A;TPT1
Fluvastatin-94_mg/kg_in_Corn_Oil-Rat-Kidney-5d-dn	3/263	0,739432051	0,88252263	0	0	0,78397554	0,236660958	CLP1;RPSA;TPT1
Papaverine-69_mg/kg_in_CMC-Rat-Heart-1d-up	3/263	0,739432051	0,882388915	0	0	0,78397554	0,236660958	CXCL11;FUNDC1;SEPP1
Cyproheptadine-37_mg/kg_in_CMC-Rat-Heart-1d-up	5/423	0,740028576	0,882966984	0	0	0,812393881	0,244584564	CXCL11;RPS19;RPSA;SEPP1;TPT1
Methotrexate-0.3_mg/kg_in_Corn_Oil-Rat-Spleen-3d-dn	4/344	0,740187114	0,883022373	0	0	0,799168864	0,240431766	RPS19;RPL10A;RPL39;TPT1
Doxorubicin-4_mg/kg_in_Saline-Rat-Heart-28d-dn	3/264	0,741755643	0,884759571	0	0	0,781005936	0,23331413	ARL2BP;SEPP1;TPT1
Indomethacin-4.5_mg/kg_in_Water-Rat-Liver-3d-dn	3/264	0,741755643	0,884625598	0	0	0,781005936	0,23331413	RPS19;RPL39;SEPP1
Ketorolac-48_mg/kg_in_Water-Rat-Liver-3d-dn	3/264	0,741755643	0,884491665	0	0	0,781005936	0,23331413	C1QA;RPL39;SEPP1
Candesartan-1300_mg/kg_in_CMC-Rat-Kidney-1d-dn	3/265	0,744062806	0,887108487	0	0	0,778058743	0,230017374	ARL2BP;SEPP1;TPT1
D-Tubocurarine_Chloride-0.0033_mg/kg_in_Saline-Rat-Heart-3d-dn	3/265	0,744062806	0,886974219	0	0	0,778058743	0,230017374	EEF1B2;FUNDC1;ARL6IP5
Daunorubicin-3.25_mg/kg_in_Saline-Rat-Liver-5d-up	3/265	0,744062806	0,886839992	0	0	0,778058743	0,230017374	GOLGA7;SEPP1;TPT1
Mitomycin_C-0.5_mg/kg_in_Saline-Rat-Liver-1d-up	3/265	0,744062806	0,886705805	0	0	0,778058743	0,230017374	RPS8;RPSA;TPT1
Piclamilast-17_mg/kg_in_CMC-Rat-Heart-5d-dn	3/265	0,744062806	0,886571659	0	0	0,778058743	0,230017374	COL5A3;RPS6;SEPP1
Rolipram-75_mg/kg_in_CMC-Rat-Liver-1d-up	3/265	0,744062806	0,886437553	0	0	0,778058743	0,230017374	CLP1;RPS3A;TPT1
Azithromycin-50_mg/kg_in_Water-Rat-Liver-3d-up	4/347	0,746286935	0,888952798	0	0	0,792259623	0,231850914	RPS6;RPS3A;RPL10A;SEPP1
Desloratadine-57_uM_in_DMSO-Rat-Primary_rat_hepatocytes-1d-up	4/347	0,746286935	0,888818373	0	0	0,792259623	0,231850914	RPL32;RPS8;RPS5;RPS3A
Bis(2-Ethylhexyl)Phthalate-1000_mg/kg_in_Corn_Oil-Rat-Liver-1d-dn	3/266	0,746353589	0,888763361	0	0	0,775133711	0,226769871	EEF1B2;C1QA;RPS3A
Doxorubicin-3_mg/kg_in_Saline-Rat-Liver-5d-dn	3/266	0,746353589	0,888629005	0	0	0,775133711	0,226769871	C1QA;RPS17;RPL32
Famotidine-1200_mg/kg_in_CMC-Rat-Heart-1d-dn	3/266	0,746353589	0,88849469	0	0	0,775133711	0,226769871	ARL2BP;RPS8;RPS3A
Sparfloxacin-450_mg/kg_in_Corn_Oil-Rat-Liver-5d-dn	3/266	0,746353589	0,888360415	0	0	0,775133711	0,226769871	RPL32;RPS8;RPL35A
4-Nonylphenol-200_mg/kg_in_Corn_Oil-Rat-Liver-5d-up	3/267	0,748628041	0,890932979	0	0	0,772230588	0,223570814	RPS19;RPS6;TPT1
Busulfan-3_mg/kg_in_Corn_Oil-Rat-Liver-5d-dn	3/267	0,748628041	0,890798376	0	0	0,772230588	0,223570814	RPL10A;RPL39;SEPP1
Econazole-43_mg/kg_in_Corn_Oil-Rat-Liver-0.25d-dn	3/267	0,748628041	0,890663815	0	0	0,772230588	0,223570814	RPS19;SEPP1;TPT1
Hexachloro-13-Butadiene-50_mg/kg_in_Corn_Oil-Rat-Kidney-5d-dn	3/267	0,748628041	0,890529293	0	0	0,772230588	0,223570814	EEF1B2;RPL39;SEPP1
Meloxicam-6_mg/kg_in_Corn_Oil-Rat-Liver-5d-up	3/267	0,748628041	0,890394813	0	0	0,772230588	0,223570814	RPS19;CLP1;TPT1
1-Naphthyl_Isothiocyanate-60_mg/kg_in_Corn_Oil-Rat-Kidney-1d-dn	3/268	0,750886213	0,892945767	0	0	0,769349131	0,220419413	ARL2BP;CLP1;TPT1
Allyl_Alcohol-25_mg/kg_in_Saline-Rat-Kidney-1d-dn	3/268	0,750886213	0,892810962	0	0	0,769349131	0,220419413	EEF1B2;RPL10A;SEPP1
Amantadine-220_mg/kg_in_Water-Rat-Liver-3d-dn	3/268	0,750886213	0,892676198	0	0	0,769349131	0,220419413	C1QA;CLP1;SEPP1
Clozapine-95_mg/kg_in_CMC-Rat-Heart-1d-up	3/268	0,750886213	0,892541475	0	0	0,769349131	0,220419413	FUNDC1;SEPP1;TPT1
Cyclosporin_A-70_mg/kg_in_Corn_Oil-Rat-Kidney-5d-dn	3/268	0,750886213	0,892406792	0	0	0,769349131	0,220419413	CLP1;R3HCC1;RAPGEF5
Hydroxyurea-59_mg/kg_in_Saline-Rat-Spleen-1d-up	3/268	0,750886213	0,89227215	0	0	0,769349131	0,220419413	RPS19;RPL35A;RPL39
Sulindac-64_mg/kg_in_Corn_Oil-Rat-Kidney-5d-dn	3/268	0,750886213	0,892137549	0	0	0,769349131	0,220419413	RPSA;RPS3A;TPT1
Tamoxifen-64_mg/kg_in_Corn_Oil-Rat-Heart-5d-up	3/268	0,750886213	0,892002988	0	0	0,769349131	0,220419413	RPS8;SEPP1;TPT1
Troglitazone-100_mg/kg_in_Corn_Oil-Rat-Liver-1d-up	3/268	0,750886213	0,891868468	0	0	0,769349131	0,220419413	CLP1;GOLGA7;TPT1
Beta-Estradiol-0.3_mg/kg_in_Corn_Oil-Rat-Liver-3d-dn	3/269	0,753128156	0,894396465	0	0	0,766489097	0,21731489	RPS17;RPS8;RPS3A
Cisapride-250_mg/kg_in_Corn_Oil-Rat-Heart-5d-up	3/269	0,753128156	0,894261625	0	0	0,766489097	0,21731489	RPS17;CLP1;PDRG1
Coumarin-111_mg/kg_in_Corn_Oil-Rat-Bone_marrow-0.25d-up	3/269	0,753128156	0,894126825	0	0	0,766489097	0,21731489	C1QA;RPS3A;RPL39
Finasteride-25_mg/kg_in_Corn_Oil-Rat-Liver-0.25d-dn	3/269	0,753128156	0,893992066	0	0	0,766489097	0,21731489	C1QA;RPL39;SEPP1
Sparfloxacin-450_mg/kg_in_Corn_Oil-Rat-Liver-3d-dn	3/269	0,753128156	0,893857348	0	0	0,766489097	0,21731489	RPS17;CLP1;RPS3A
Acyclovir-330_mg/kg_in_Saline-Rat-Liver-0.25d-up	3/270	0,755353924	0,896363945	0	0	0,763650248	0,214256484	C1QA;RPSA;RPL39
Bezafibrate-100_mg/kg_in_Corn_Oil-Rat-Liver-0.25d-up	3/270	0,755353924	0,89622891	0	0	0,763650248	0,214256484	EEF1B2;RPS19;RPS6
Carboplatin-6_mg/kg_in_Saline-Rat-Liver-1d-up	3/270	0,755353924	0,896093915	0	0	0,763650248	0,214256484	RPS20;RPL39;TPT1
Clindamycin-161_mg/kg_in_Saline-Rat-Kidney-3d-dn	3/270	0,755353924	0,895958961	0	0	0,763650248	0,214256484	RPS19;ARL2BP;RPS8
Fenofibrate-215_mg/kg_in_Corn_Oil-Rat-Liver-1d-dn	3/270	0,755353924	0,895824048	0	0	0,763650248	0,214256484	C1QA;CLP1;SEPP1
Fluoxetine-52_mg/kg_in_CMC-Rat-Liver-1d-dn	3/270	0,755353924	0,895689176	0	0	0,763650248	0,214256484	C1QA;RPS6;RPL39
Lead(IV)_Acetate-200_mg/kg_in_Saline-Rat-Kidney-1d-dn	3/270	0,755353924	0,895554343	0	0	0,763650248	0,214256484	CLP1;SEPP1;TPT1
Maprotiline-380_mg/kg_in_CMC-Rat-Heart-5d-up	3/270	0,755353924	0,895419552	0	0	0,763650248	0,214256484	C1QA;RPL32;RPS8
Norethindrone-75_mg/kg_in_Corn_Oil-Rat-Liver-5d-dn	3/270	0,755353924	0,895284801	0	0	0,763650248	0,214256484	C1QA;SEPP1;TPT1
Piracetam-2500_mg/kg_in_CMC-Rat-Brain-3d-dn	3/270	0,755353924	0,895150091	0	0	0,763650248	0,214256484	EEF1B2;RPS6;SEPP1
Valproic_Acid-850_mg/kg_in_Water-Rat-Kidney-5d-dn	3/270	0,755353924	0,895015421	0	0	0,763650248	0,214256484	RPS19;CLP1;SEPP1
N-Nitrosodiethylamine-1.67_mg/kg_in_Saline-Rat-Spleen-3d-up	4/352	0,756210679	0,895895804	0	0	0,781005936	0,218240601	CXCL11;RPS19;NKG7;RPS20
Valproic_Acid-1500_mg/kg_in_Water-Rat-Liver-5d-up	4/352	0,756210679	0,895761063	0	0	0,781005936	0,218240601	RPS8;RPS3A;RPL10A;TPT1
Ampiroxicam-94_mg/kg_in_CMC-Rat-Kidney-5d-dn	3/271	0,757563569	0,897228673	0	0	0,760832351	0,211243447	ARL2BP;RPL39;SEPP1
Bithionol-333_mg/kg_in_Corn_Oil-Rat-Kidney-5d-dn	3/271	0,757563569	0,897093771	0	0	0,760832351	0,211243447	RPSA;SEPP1;TPT1
Diethylstilbestrol-2.8_mg/kg_in_Corn_Oil-Rat-Spleen-1d-dn	3/271	0,757563569	0,896958911	0	0	0,760832351	0,211243447	RPS19;RPS5;RPS6
Levodopa-1000_mg/kg_in_Water-Rat-Brain-5d-up	3/271	0,757563569	0,89682409	0	0	0,760832351	0,211243447	EEF1B2;RPS8;ARL6IP5
Streptomycin-120_mg/kg_in_Saline-Rat-Bone_marrow-5d-dn	3/271	0,757563569	0,896689311	0	0	0,760832351	0,211243447	RPL35A;RPL39;DUSP14
Hydroxyurea-59_mg/kg_in_Saline-Rat-Liver-0.25d-dn	4/353	0,758159238	0,897259528	0	0	0,778793454	0,215618188	RPL32;SEPP1;RPL39;TPT1
11-Dichloroethene-600_mg/kg_in_Water-Rat-Liver-3d-dn	3/272	0,759757148	0,899015519	0	0	0,758035173	0,208275045	CLP1;SEPP1;TPT1
Doxapram-2_mg/kg_in_Saline-Rat-Liver-5d-dn	3/272	0,759757148	0,898880471	0	0	0,758035173	0,208275045	CLP1;SEPP1;TPT1

Irinotecan-5_mg/kg_in_Saline-Rat-Heart-1d-up	3/272	0,759757148	0,898745464	0	0	0,758035173	0,208275045	C1QA;RPS3A;RPL39
17-Methyltestosterone-2000_mg/kg_in_CMC-Rat-Liver-3d-dn	3/273	0,761934715	0,901186036	0	0	0,755258487	0,205350556	C1QA;SEPP1;TPT1
4-Nonylphenol-20_mg/kg_in_Corn_Oil-Rat-Liver-1d-dn	3/273	0,761934715	0,901050723	0	0	0,755258487	0,205350556	RPS17;RPS19;RPS20
Atropine-94_mg/kg_in_Water-Rat-Liver-0.25d-dn	3/273	0,761934715	0,90091545	0	0	0,755258487	0,205350556	C1QA;RPL35A;TPT1
Benzethonium_Chloride-5_mg/kg_in_Water-Rat-Heart-3d-dn	3/273	0,761934715	0,900780218	0	0	0,755258487	0,205350556	R3HCC1;RPL39;SEPP1
Bithionol-333_mg/kg_in_Corn_Oil-Rat-Bone_marrow-3d-dn	3/273	0,761934715	0,900645027	0	0	0,755258487	0,205350556	RPS19;RPSA;RPL10A
Cytarabine-487_mg/kg_in_Saline-Rat-Kidney-3d-up	3/273	0,761934715	0,900509876	0	0	0,755258487	0,205350556	RPS19;CLP1;RPSA
Fludrocortisone_Acetate-125_mg/kg_in_CMC-Rat-Liver-1d-up	3/273	0,761934715	0,900374766	0	0	0,755258487	0,205350556	CXCL11;RPS20;RPL39
Fluvastatin-94_mg/kg_in_Corn_Oil-Rat-Liver-5d-up	3/273	0,761934715	0,900239696	0	0	0,755258487	0,205350556	RPS6;RPS20;RPL39
Glipizide-2500_mg/kg_in_CMC-Rat-Liver-0.25d-dn	3/273	0,761934715	0,900104667	0	0	0,755258487	0,205350556	CLP1;SEPP1;TPT1
Ibuprofen-54_mg/kg_in_Corn_Oil-Rat-Kidney-3d-dn	3/273	0,761934715	0,899969678	0	0	0,755258487	0,205350556	RPS19;SEPP1;TPT1
Indomethacin-4.5_mg/kg_in_Water-Rat-Kidney-1d-dn	3/273	0,761934715	0,89983473	0	0	0,755258487	0,205350556	RPS20;RPL10A;RPL39
Oxymetazoline-0.5_mg/kg_in_Water-Rat-Liver-1d-dn	3/273	0,761934715	0,899699822	0	0	0,755258487	0,205350556	RPS6;SEPP1;TPT1
Tocainide-224_mg/kg_in_Corn_Oil-Rat-Liver-0.25d-dn	3/273	0,761934715	0,899564955	0	0	0,755258487	0,205350556	RPSA;RPL39;TPT1
Ifosfamide-17_mg/kg_in_Saline-Rat-Liver-1d-up	4/356	0,76393298	0,901788991	0	0	0,772230588	0,207942558	C1QA;RPSA;RPL39;TPT1
Stanozolol-150_mg/kg_in_Water-Rat-Liver-1d-dn	4/356	0,76393298	0,901653852	0	0	0,772230588	0,207942558	C1QA;RPS17;FEM1B;SEPP1
123-Trichloropropane-108_mg/kg_in_CMC-Rat-Kidney-1d-up	3/274	0,764096327	0,901711518	0	0	0,752502069	0,202469272	RPS17;RPS3A;SEPP1
3-Chloroaniline-200_mg/kg_in_CMC-Rat-Kidney-1d-up	3/274	0,764096327	0,90157643	0	0	0,752502069	0,202469272	RPS17;RPS19;SEPP1
Azathioprine-160_mg/kg_in_Corn_Oil-Rat-Liver-1d-dn	3/274	0,764096327	0,901441382	0	0	0,752502069	0,202469272	C1QA;RPS3A;TPT1
Cisplatin-63_uM_in_DMSO-Rat-Primary_rat_hepatocytes-0.67d-dn	3/274	0,764096327	0,901306375	0	0	0,752502069	0,202469272	SEPP1;SLC16A3;TPT1
Clotrimazole-60_uM_in_DMSO-Rat-Primary_rat_hepatocytes-1d-up	3/274	0,764096327	0,901171409	0	0	0,752502069	0,202469272	FEM1B;RPS3A;PDRG1
Dimenhydrinate-165_mg/kg_in_CMC-Rat-Liver-1d-dn	3/274	0,764096327	0,901036483	0	0	0,752502069	0,202469272	RPS20;RPL10A;SEPP1
Fenofibrate-250_uM_in_DMSO-Rat-Primary_rat_hepatocytes-0.67d-up	3/274	0,764096327	0,900901597	0	0	0,752502069	0,202469272	RPS17;RPS3A;TPT1
Lamivudine-1300_mg/kg_in_Water-Rat-Liver-1d-dn	3/274	0,764096327	0,900766752	0	0	0,752502069	0,202469272	RPS19;RPS8;RPSA
Methotrexate-0.3_mg/kg_in_Corn_Oil-Rat-Kidney-0.25d-dn	3/274	0,764096327	0,900631947	0	0	0,752502069	0,202469272	ARL2BP;RPS8;TPT1
Probenecid-600_mg/kg_in_Corn_Oil-Rat-Kidney-3d-dn	3/274	0,764096327	0,900497182	0	0	0,752502069	0,202469272	EEF1B2;RPS17;RPS20
Rifampin-99_mg/kg_in_CMC-Rat-Kidney-3d-up	3/274	0,764096327	0,900362458	0	0	0,752502069	0,202469272	RPS17;RPS19;RPS3A
Rofecoxib-3_mg/kg_in_Corn_Oil-Rat-Liver-3d-dn	3/274	0,764096327	0,900227774	0	0	0,752502069	0,202469272	RPS17;RPS8;RPS3A
Zileuton-450_mg/kg_in_Corn_Oil-Rat-Liver-0.25d-up	3/274	0,764096327	0,90009313	0	0	0,752502069	0,202469272	RPS8;RPS6;RPS3A
Acemetacin-2_mg/kg_in_Corn_Oil-Rat-Liver-5d-dn	3/275	0,766242041	0,902485766	0	0	0,749765698	0,199630499	RPS19;FEM1B;RPL10A
Alendronic_Acid-138_mg/kg_in_CMC-Rat-Heart-1d-up	3/275	0,766242041	0,902350825	0	0	0,749765698	0,199630499	CXCL11;ARL2BP;RPS20
Bromfenac-5_mg/kg_in_CMC-Rat-Liver-3d-dn	3/275	0,766242041	0,902215924	0	0	0,749765698	0,199630499	C1QA;CLP1;SEPP1
Ciprofloxacin-72_mg/kg_in_Corn_Oil-Rat-Liver-3d-dn	3/275	0,766242041	0,902081064	0	0	0,749765698	0,199630499	EEF1B2;CLP1;RPL10A
Lomustine-4.2_mg/kg_in_Corn_Oil-Rat-Liver-5d-dn	3/275	0,766242041	0,901946244	0	0	0,749765698	0,199630499	C1QA;RPL32;RPS8
Norethindrone_Acetate-125_mg/kg_in_Corn_Oil-Rat-Liver-5d-dn	3/275	0,766242041	0,901811464	0	0	0,749765698	0,199630499	EEF1B2;C1QA;SEPP1
Tacrine-24_mg/kg_in_Water-Rat-Liver-5d-up	3/275	0,766242041	0,901676724	0	0	0,749765698	0,199630499	CLP1;GOLGA7;SEPP1
Troglitazone-1200_mg/kg_in_Corn_Oil-Rat-Liver-1d-up	3/275	0,766242041	0,901542025	0	0	0,749765698	0,199630499	GOLGA7;SEPP1;TPT1
Enoxacin-100_mg/kg_in_Corn_Oil-Rat-Liver-3d-up	2/190	0,766513508	0,90172672	0	0	0,72345813	0,192369657	RPS20;SEPP1
Auranofin-33_mg/kg_in_CMC-Rat-Kidney-1d-up	3/276	0,768371916	0,903777959	0	0	0,747049156	0,196833555	RPS8;RPS3A;TPT1
Daunorubicin-3.25_mg/kg_in_Saline-Rat-Liver-1d-up	3/276	0,768371916	0,903643006	0	0	0,747049156	0,196833555	RPS8;SEPP1;TPT1
Ibuprofen-275_mg/kg_in_Corn_Oil-Rat-Liver-1d-up	3/276	0,768371916	0,903508094	0	0	0,747049156	0,196833555	EEF1B2;RPS8;SEPP1
Mevastatin-1200_mg/kg_in_CMC-Rat-Kidney-5d-dn	3/276	0,768371916	0,903373222	0	0	0,747049156	0,196833555	RPS19;RPS6;TPT1
Penicillamine-852_mg/kg_in_Water-Rat-Bone_marrow-5d-dn	3/276	0,768371916	0,90323839	0	0	0,747049156	0,196833555	RPS19;RPS8;RPSA
Secobarbital-20_mg/kg_in_Water-Rat-Liver-1d-dn	3/276	0,768371916	0,903103599	0	0	0,747049156	0,196833555	RPS8;SEPP1;TPT1
Trimethadione-535_mg/kg_in_Water-Rat-Kidney-5d-up	4/359	0,769599389	0,904411338	0	0	0,765777408	0,20054575	EEF1B2;RPS8;SEPP1;TPT1
4-Octylphenol-700_mg/kg_in_Corn_Oil-Rat-Kidney-1d-up	3/277	0,770486012	0,90531819	0	0	0,744352227	0,194077769	RPS17;CLP1;RPSA
Eucalyptol-930_mg/kg_in_Corn_Oil-Rat-Kidney-3d-dn	3/277	0,770486012	0,905183149	0	0	0,744352227	0,194077769	CLP1;RPL39;TPT1
Hydroxyurea-59_mg/kg_in_Saline-Rat-Bone_marrow-1d-up	3/277	0,770486012	0,905048148	0	0	0,744352227	0,194077769	EEF1B2;NKG7;RPS3A
Simvastatin-15_mg/kg_in_Corn_Oil-Rat-Kidney-0.25d-dn	3/277	0,770486012	0,904913187	0	0	0,744352227	0,194077769	RPS6;ARL6IP5;SEPP1
Ticlopidine-223_mg/kg_in_CMC-Rat-Liver-1d-dn	3/277	0,770486012	0,904778266	0	0	0,744352227	0,194077769	EEF1B2;FEM1B;SEPP1
Tretinoin-7_mg/kg_in_Corn_Oil-Rat-Liver-3d-dn	3/277	0,770486012	0,904643386	0	0	0,744352227	0,194077769	RPS6;RPL10A;RPL39
Trichloroacetic_Acid-474_mg/kg_in_Corn_Oil-Rat-Liver-5d-dn	3/277	0,770486012	0,904508545	0	0	0,744352227	0,194077769	CLP1;RPS3A;SEPP1
Catechol-40_mg/kg_in_Saline-Rat-Liver-5d-dn	3/278	0,772584388	0,906836757	0	0	0,741674701	0,191362484	C1QA;RPL39;SEPP1
Famciclovir-112_mg/kg_in_Saline-Rat-Liver-0.25d-dn	3/278	0,772584388	0,90670163	0	0	0,741674701	0,191362484	RPS19;RPL10A;TPT1
Ketoprofen-4_mg/kg_in_Corn_Oil-Rat-Kidney-0.25d-dn	3/278	0,772584388	0,906566543	0	0	0,741674701	0,191362484	ARL2BP;FUNDC1;ARL6IP5
Rolipram-75_mg/kg_in_CMC-Rat-Heart-5d-dn	3/278	0,772584388	0,906431497	0	0	0,741674701	0,191362484	RPS19;COL5A3;SEPP1
Sparfloxacin-450_mg/kg_in_Corn_Oil-Rat-Liver-1d-dn	3/278	0,772584388	0,90629649	0	0	0,741674701	0,191362484	RPL32;RPS8;RPL10A
Temafloxacin-1000_mg/kg_in_CMC-Rat-Liver-1d-up	3/278	0,772584388	0,906161524	0	0	0,741674701	0,191362484	RPSA;RPL10A;TPT1
2-Amino-4-Nitrophenol-625_mg/kg_in_CMC-Rat-Heart-1d-up	3/279	0,774667104	0,908469046	0	0	0,739016369	0,188687052	EEF1B2;RPS17;TOMM20
Chlorambucil-0.6_mg/kg_in_Corn_Oil-Rat-Liver-3d-up	3/279	0,774667104	0,908333797	0	0	0,739016369	0,188687052	RPS8;RPS20;SEPP1
Daunorubicin-4_mg/kg_in_Saline-Rat-Kidney-14d-dn	3/279	0,774667104	0,908198588	0	0	0,739016369	0,188687052	CLP1;SEPP1;TPT1
Gabapentin-100_mg/kg_in_Water-Rat-Heart-5d-dn	3/279	0,774667104	0,908063419	0	0	0,739016369	0,188687052	ARL2BP;RPS8;RPS6
Idebenone-2500_mg/kg_in_CMC-Rat-Liver-0.25d-dn	3/279	0,774667104	0,907928291	0	0	0,739016369	0,188687052	EEF1B2;CLP1;SEPP1
Iproniazid-46_mg/kg_in_Water-Rat-Brain-3d-up	3/279	0,774667104	0,907793202	0	0	0,739016369	0,188687052	ARL2BP;FEM1B;TPT1
Mycophenolate_Mofetil-63_mg/kg_in_CMC-Rat-Bone_marrow-3d-dn	3/279	0,774667104	0,907658154	0	0	0,739016369	0,188687052	FEM1B;RPL10A;TPT1
Rofecoxib-775_mg/kg_in_Corn_Oil-Rat-Kidney-5d-dn	3/279	0,774667104	0,907523146	0	0	0,739016369	0,188687052	RPL10A;RPL39;SEPP1
Rosiglitazone-10_mg/kg_in_Corn_Oil-Rat-Liver-3d-dn	3/279	0,774667104	0,907388179	0	0	0,739016369	0,188687052	RPS17;RPS6;RPS3A
Triacetin-1200_mg/kg_in_CMC-Rat-Liver-1d-dn	3/279	0,774667104	0,907253251	0	0	0,739016369	0,188687052	RPS17;RPS20;SEPP1
Valproic_Acid-850_mg/kg_in_Water-Rat-Bone_marrow-3d-dn	3/279	0,774667104	0,907118363	0	0	0,739016369	0,188687052	ARL2BP;FUNDC1;TPT1
Carboplatin-14_mg/kg_in_Saline-Rat-Liver-3d-up	3/280	0,776734223	0,909403708	0	0	0,736377025	0,186050841	RPS19;RPS6;RPL10A
Carmustine-16_mg/kg_in_Corn_Oil-Rat-Liver-5d-dn	3/280	0,776734223	0,909268541	0	0	0,736377025	0,186050841	RPS17;RPL32;RPS3A
Dexamethasone-300_mg/kg_in_Corn_Oil-Rat-Kidney-3d-dn	3/280	0,776734223	0,909133414	0	0	0,736377025	0,186050841	RPS17;CLP1;RPS3A
Digitoxin-1_mg/kg_in_Corn_Oil-Rat-Heart-1d-up	3/280	0,776734223	0,908998327	0	0	0,736377025	0,186050841	RPS19;COL5A3;RAPGEF5
Doxapram-2_mg/kg_in_Saline-Rat-Liver-1d-dn	3/280	0,776734223	0,908863281	0	0	0,736377025	0,186050841	RPS20;SEPP1;TPT1
Losartan-1000_mg/kg_in_Water-Rat-Heart-1d-dn	3/280	0,776734223	0,908728274	0	0	0,736377025	0,186050841	RPS8;COL5A3;RPL39
Rolipram-75_mg/kg_in_CMC-Rat-Heart-3d-dn	3/280	0,776734223	0,908593308	0	0	0,736377025	0,186050841	RPL32;COL5A3;RPS6
Tegafur-175_mg/kg_in_Water-Rat-Bone_marrow-1d-up	3/280	0,776734223	0,908458382	0	0	0,736377025	0,186050841	RPS19;RPS6;RPL10A
Acrolein-20_mg/kg_in_Water-Rat-Heart-1d-up	3/281	0,778785808	0,910722646	0	0	0,733756466	0,183453226	RPS8;SEPP1;TPT1
Aspirin-35_mg/kg_in_Corn_Oil-Rat-Liver-0.25d-dn	3/281	0,778785808	0,910587444	0	0	0,733756466	0,183453226	C1QA;RPL39;TPT1
Catechol-40_mg/kg_in_Saline-Rat-Kidney-0.25d-up	3/281	0,778785808	0,910452281	0	0	0,733756466	0,183453226	RPS19;CLP1;GOLGA7
Fluphenazine-22_mg/kg_in_Corn_Oil-Rat-Liver-0.25d-dn	3/281	0,778785808	0,910317159	0	0	0,733756466	0,183453226	RPS17;FUNDC1;TPT1
Isoprenaline-15_mg/kg_in_Saline-Rat-Heart-5d-up	3/281	0,778785808	0,910182078	0	0	0,733756466	0,183453226	RPS19;RPSA;RPL39
Lovastatin-1500_mg/kg_in_Corn_Oil-Rat-Kidney-3d-up	3/281	0,778785808	0,910047036	0	0	0,733756466	0,183453226	RPL32;RPS20;RPL39
Penicillin_G-243_mg/kg_in_Saline-Rat-Kidney-3d-dn	3/281	0,778785808	0,909912034	0	0	0,733756466	0,183453226	ARL2BP;RPL39;TPT1
Prednisolone-184_mg/kg_in_Water-Rat-Heart-1d-up	3/281	0,778785808	0,909777072	0	0	0,733756466	0,183453226	RPS6;ARL6IP5;TPT1
Sodium_Selenate-1.6_mg/kg_in_Water-Rat-Heart-1d-up	3/281	0,778785808	0,90964215	0	0	0,733756466	0,183453226	RPS8;SEPP1;TPT1
Sulfaphenazole-1695_mg/kg_in_Water-Rat-Liver-0.25d-dn	3/281	0,778785808	0,909507269	0	0	0,733756466	0,183453226	EEF1B2;RPS19;RPL39
Sulfinpyrazone-269_mg/kg_in_CMC-Rat-Kidney-5d-dn	3/281	0,778785808	0,909372427	0	0	0,733756466	0,183453226	ARL2BP;RPS8;RPS3A
Valproic_Acid-850_mg/kg_in_Water-Rat-Bone_marrow-5d-dn	3/281	0,778785808	0,909237625	0	0	0,733756466	0,183453226	RPS8;FEM1B;SLC16A3
Zidovudine-1473_uM_in_DMSO-Rat-Primary_rat_hepatocytes-1d-up	3/281	0,778785808	0,909102864	0	0	0,733756466	0,183453226	EEF1B2;CLP1;FUNDC1

Oxaliplatin-8_mg/kg_in_Saline-Rat-Kidney-1d-up	4/364	0,778807006	0,908992884	0	0	0,755258487	0,188808587	CLP1;RPS8;FUNDC1;SEPP1
Sulfaphenazole-1695_mg/kg_in_Water-Rat-Kidney-1d-up	4/364	0,778807006	0,908858198	0	0	0,755258487	0,188808587	EEF1B2;CLP1;SEPP1;TPT1
Aldosterone-0.065_mg/kg_in_Saline-Rat-Kidney-1d-up	3/282	0,78082192	0,911074584	0	0	0,731154493	0,180893596	RPS19;ARL6IP5;GOLGA7
Carbachol-15_mg/kg_in_Water-Rat-Brain-3d-dn	3/282	0,78082192	0,91093963	0	0	0,731154493	0,180893596	ARL2BP;RPS6;SEPP1
Cisplatin-1.17_mg/kg_in_Saline-Rat-Kidney-1d-dn	3/282	0,78082192	0,910804716	0	0	0,731154493	0,180893596	RPL32;RPL10A;SEPP1
Citric_Acid-3000_mg/kg_in_Water-Rat-Liver-3d-dn	3/282	0,78082192	0,910669842	0	0	0,731154493	0,180893596	GOLGA7;SEPP1;TPT1
Ipriflavone-1500_mg/kg_in_CMC-Rat-Liver-3d-dn	3/282	0,78082192	0,910535007	0	0	0,731154493	0,180893596	EEF1B2;CLP1;RPS8
Lomustine-4.2_mg/kg_in_Corn_Oil-Rat-Bone_marrow-3d-dn	3/282	0,78082192	0,910400213	0	0	0,731154493	0,180893596	FEM1B;RPS6;ARL6IP5
Methapyrilene-100_mg/kg_in_CMC-Rat-Liver-3d-dn	3/282	0,78082192	0,910265459	0	0	0,731154493	0,180893596	C1QA;CLP1;TPT1
N-Nitrosodiethylamine-34_mg/kg_in_Saline-Rat-Kidney-1d-dn	3/282	0,78082192	0,910130744	0	0	0,731154493	0,180893596	ARL2BP;RPL39;TPT1
Naproxen-10_mg/kg_in_Corn_Oil-Rat-Liver-0.25d-dn	3/282	0,78082192	0,90999607	0	0	0,731154493	0,180893596	RPL32;RPL39;TPT1
Nateglinide-625_mg/kg_in_CMC-Rat-Kidney-1d-up	3/282	0,78082192	0,909861435	0	0	0,731154493	0,180893596	RPS17;RPS3A;TPT1
Sparfloxacin-29_mg/kg_in_Corn_Oil-Rat-Liver-1d-dn	3/282	0,78082192	0,90972684	0	0	0,731154493	0,180893596	C1QA;RPS17;RPL35A
Alfacalcidol-0.043_mg/kg_in_CMC-Rat-Heart-1d-up	3/283	0,782842624	0,911946237	0	0	0,728570908	0,178371348	ARL6IP5;RPL10A;RPL39
Alfacalcidol-0.043_mg/kg_in_CMC-Rat-Heart-3d-up	3/283	0,782842624	0,911811373	0	0	0,728570908	0,178371348	C1QA;CXCL11;RPL10A
Beta-Naphthoflavone-1500_mg/kg_in_CMC-Rat-Liver-3d-dn	3/283	0,782842624	0,91167655	0	0	0,728570908	0,178371348	CLP1;RPL39;TPT1
Doxorubicin-3_mg/kg_in_Saline-Rat-Liver-3d-dn	3/283	0,782842624	0,911541766	0	0	0,728570908	0,178371348	C1QA;RPL32;TPT1
Ethanol-3000_mg/kg_in_Saline-Rat-Bone_marrow-0.25d-up	3/283	0,782842624	0,911407022	0	0	0,728570908	0,178371348	RPS19;FUNDC1;RPS20
Fluoxetine-52_mg/kg_in_CMC-Rat-Liver-3d-dn	3/283	0,782842624	0,911272318	0	0	0,728570908	0,178371348	RPL39;SEPP1;TPT1
Foscarnet-350_mg/kg_in_Saline-Rat-Kidney-5d-up	3/283	0,782842624	0,911137654	0	0	0,728570908	0,178371348	CLP1;RPSA;TPT1
Lamotrigine-26_mg/kg_in_CMC-Rat-Kidney-1d-up	3/283	0,782842624	0,91100303	0	0	0,728570908	0,178371348	RPS17;RPS3A;RAPGEF5
Mitoxantrone-2_mg/kg_in_Saline-Rat-Heart-1d-dn	3/283	0,782842624	0,910868445	0	0	0,728570908	0,178371348	FEM1B;ARL6IP5;SEPP1
Penicillamine-100_mg/kg_in_Water-Rat-Liver-0.25d-dn	3/283	0,782842624	0,9107339	0	0	0,728570908	0,178371348	RPL32;RPS19;RPSA
Probenecid-600_mg/kg_in_Corn_Oil-Rat-Kidney-0.25d-dn	3/283	0,782842624	0,910599395	0	0	0,728570908	0,178371348	EEF1B2;RPS8;RPS6
Sotalol-2000_mg/kg_in_Water-Rat-Liver-1d-up	3/283	0,782842624	0,91046493	0	0	0,728570908	0,178371348	C1QA;RPSA;TPT1
Beta-Naphthoflavone-80_mg/kg_in_CMC-Rat-Liver-91d-up	3/284	0,784847984	0,912662443	0	0	0,726005518	0,175885894	RPS8;RPSA;SEPP1
Citric_Acid-3000_mg/kg_in_Water-Rat-Liver-5d-dn	3/284	0,784847984	0,912527713	0	0	0,726005518	0,175885894	CLP1;RPL39;SEPP1
Diisopropyl_Fluorophosphate-3.75_mg/kg_in_Water-Rat-Liver-5d-up	3/284	0,784847984	0,912393022	0	0	0,726005518	0,175885894	RPS17;ARL6IP5;RPSA
Dimenhydrinate-165_mg/kg_in_CMC-Rat-Liver-5d-dn	3/284	0,784847984	0,912258371	0	0	0,726005518	0,175885894	EEF1B2;C1QA;RPL39
Doxycycline-14_mg/kg_in_Water-Rat-Liver-0.25d-up	3/284	0,784847984	0,91212376	0	0	0,726005518	0,175885894	RPSA;RPL10A;SEPP1
Ibuprofen-54_mg/kg_in_Corn_Oil-Rat-Liver-3d-up	3/284	0,784847984	0,911989189	0	0	0,726005518	0,175885894	RPS8;RPS20;RPSA
Phenacemide-400_mg/kg_in_CMC-Rat-Kidney-3d-dn	3/284	0,784847984	0,911854658	0	0	0,726005518	0,175885894	RPS8;RPL39;TPT1
Simvastatin-15_mg/kg_in_Corn_Oil-Rat-Liver-1d-dn	3/284	0,784847984	0,911720166	0	0	0,726005518	0,175885894	C1QA;RPS19;SEPP1
Thalidomide-113_mg/kg_in_CMC-Rat-Kidney-5d-up	3/284	0,784847984	0,911585714	0	0	0,726005518	0,175885894	ARL2BP;CLP1;SEPP1
Thimerosal-10_mg/kg_in_Water-Rat-Brain-3d-up	3/284	0,784847984	0,911451301	0	0	0,726005518	0,175885894	EEF1B2;SEPP1;TPT1
Thioridazine-63_mg/kg_in_Corn_Oil-Rat-Heart-3d-up	3/284	0,784847984	0,911316928	0	0	0,726005518	0,175885894	RPS20;RPL10A;SEPP1
Tropisetron-100_mg/kg_in_CMC-Rat-Liver-5d-dn	3/284	0,784847984	0,911182595	0	0	0,726005518	0,175885894	RPS17;SEPP1;TPT1
Cobalt(II)_Acetylacetonate-100_mg/kg_in_Corn_Oil-Rat-Heart-5d-up	3/285	0,786838066	0,91335838	0	0	0,72345813	0,173436652	CLP1;COL5A3;SEPP1
Doxifluridine-1230_mg/kg_in_CMC-Rat-Bone_marrow-1d-up	3/285	0,786838066	0,913223786	0	0	0,72345813	0,173436652	C1QA;SEPP1;TPT1
Eperisone-501_mg/kg_in_CMC-Rat-Liver-5d-up	3/285	0,786838066	0,913089231	0	0	0,72345813	0,173436652	RPS19;RPSA;RPL39
Ivermectin-7.5_mg/kg_in_CMC-Rat-Liver-3d-dn	3/285	0,786838066	0,912954716	0	0	0,72345813	0,173436652	CLP1;RPS8;TPT1
Omeprazole-400_uM_in_DMSO-Rat-Primary_rat_hepatocytes-0.67d-up	3/285	0,786838066	0,91282024	0	0	0,72345813	0,173436652	EEF1B2;RPL10A;TOMM20
Thalidomide-113_mg/kg_in_CMC-Rat-Kidney-1d-up	3/285	0,786838066	0,912685804	0	0	0,72345813	0,173436652	RPS17;RPSA;RPS3A
Tramadol-114_mg/kg_in_Water-Rat-Brain-5d-up	3/285	0,786838066	0,912551408	0	0	0,72345813	0,173436652	RPS19;FUNDC1;RPS6
Bithionol-59_mg/kg_in_Corn_Oil-Rat-Kidney-1d-up	3/286	0,788812935	0,914707108	0	0	0,720928556	0,171023053	RPS19;RPS5;RPS20
Carmustine-4_mg/kg_in_Corn_Oil-Rat-Liver-0.25d-up	3/286	0,788812935	0,914572454	0	0	0,720928556	0,171023053	RPS6;RPL10A;SEPP1
Cetylpyridinium_Bromide-16.5_mg/kg_in_Water-Rat-Liver-1d-dn	3/286	0,788812935	0,914437839	0	0	0,720928556	0,171023053	ARL2BP;RPS20;SEPP1
Clarithromycin-476_mg/kg_in_Water-Rat-Liver-0.25d-dn	3/286	0,788812935	0,914303264	0	0	0,720928556	0,171023053	RPSA;RPL10A;SEPP1
Disulfiram-500_mg/kg_in_Water-Rat-Liver-3d-dn	3/286	0,788812935	0,914168728	0	0	0,720928556	0,171023053	CLP1;RPS8;TPT1
Finasteride-800_mg/kg_in_Corn_Oil-Rat-Liver-1d-up	3/286	0,788812935	0,914034233	0	0	0,720928556	0,171023053	RPS19;RPS6;RPS3A
Fluconazole-10_mg/kg_in_Corn_Oil-Rat-Liver-1d-dn	3/286	0,788812935	0,913899776	0	0	0,720928556	0,171023053	RPL39;SEPP1;TPT1
Ketoconazole-25_mg/kg_in_Corn_Oil-Rat-Liver-0.25d-dn	3/286	0,788812935	0,913765359	0	0	0,720928556	0,171023053	EEF1B2;CLP1;SEPP1
Melatonin-2000_mg/kg_in_Corn_Oil-Rat-Liver-5d-up	3/286	0,788812935	0,913630982	0	0	0,720928556	0,171023053	RPS19;RPSA;SEPP1
NN'-Diphenyl-P-Phenylenediamine-1000_mg/kg_in_CMC-Rat-Liver-3d-dn	3/286	0,788812935	0,913496644	0	0	0,720928556	0,171023053	C1QA;CLP1;RPS8
Netilmicin-40_mg/kg_in_Saline-Rat-Kidney-28d-dn	3/286	0,788812935	0,913362346	0	0	0,720928556	0,171023053	ARL2BP;CLP1;SEPP1
Valacyclovir-2000_mg/kg_in_CMC-Rat-Kidney-1d-dn	3/286	0,788812935	0,913228087	0	0	0,720928556	0,171023053	RPL35A;SEPP1;TOMM20
Dexfenfluramine-29_mg/kg_in_Water-Rat-Kidney-5d-up	4/370	0,78947095	0,913855556	0	0	0,743011052	0,175642048	ARL2BP;CLP1;FUNDC1;TPT1
Troglitazone-100_mg/kg_in_Corn_Oil-Rat-Liver-5d-up	2/200	0,790724612	0,915172232	0	0	0,687285223	0,161378367	RPS8;TPT1
4-Octylphenol-450_mg/kg_in_Corn_Oil-Rat-Liver-3d-up	3/287	0,790772658	0,915093367	0	0	0,71841661	0,168644537	EEF1B2;C1QA;RPSA
Bezafibrate-100_mg/kg_in_Corn_Oil-Rat-Kidney-3d-dn	3/287	0,790772658	0,914958933	0	0	0,71841661	0,168644537	ARL2BP;RPSA;SEPP1
Clofibric_Acid-448_mg/kg_in_Corn_Oil-Rat-Liver-5d-dn	3/287	0,790772658	0,914824538	0	0	0,71841661	0,168644537	RPS19;CLP1;SEPP1
Dimenhydrinate-165_mg/kg_in_CMC-Rat-Liver-3d-dn	3/287	0,790772658	0,914690183	0	0	0,71841661	0,168644537	C1QA;SEPP1;TPT1
Levamisole-120_mg/kg_in_Water-Rat-Liver-5d-dn	3/287	0,790772658	0,914555867	0	0	0,71841661	0,168644537	C1QA;CLP1;RPL39
Secobarbital-70_mg/kg_in_Water-Rat-Liver-1d-dn	3/287	0,790772658	0,914421591	0	0	0,71841661	0,168644537	RPS20;SEPP1;TPT1
1-Naphthyl_Isothiocyanate-60_mg/kg_in_Corn_Oil-Rat-Liver-3d-dn	3/288	0,792717302	0,916535741	0	0	0,715922108	0,166300554	RPL32;RPL35A;TPT1
Allyl_Alcohol-25_mg/kg_in_Saline-Rat-Liver-3d-dn	3/288	0,792717302	0,916401213	0	0	0,715922108	0,166300554	RPS3A;RPL39;SEPP1
Alpha-Naphthoflavone-80_mg/kg_in_CMC-Rat-Liver-5d-up	3/288	0,792717302	0,916266725	0	0	0,715922108	0,166300554	RPSA;RPS3A;SEPP1
Calcitriol-0.04_mg/kg_in_CMC-Rat-Kidney-3d-dn	3/288	0,792717302	0,916132277	0	0	0,715922108	0,166300554	RPS17;ARL2BP;SEPP1
Candesartan-1300_mg/kg_in_CMC-Rat-Kidney-3d-up	3/288	0,792717302	0,915997868	0	0	0,715922108	0,166300554	RPS17;RPL39;SEPP1
Captopril-1750_mg/kg_in_Water-Rat-Kidney-3d-dn	3/288	0,792717302	0,915863498	0	0	0,715922108	0,166300554	CLP1;RPS6;SEPP1
Cyclosporin_A-350_mg/kg_in_Corn_Oil-Rat-Bone_marrow-3d-dn	3/288	0,792717302	0,915729168	0	0	0,715922108	0,166300554	RPS19;FEM1B;RPS3A
Cytarabine-487_mg/kg_in_Saline-Rat-Bone_marrow-3d-up	3/288	0,792717302	0,915594877	0	0	0,715922108	0,166300554	C1QA;RPS19;SEPP1
Diflunisal-207_mg/kg_in_Corn_Oil-Rat-Kidney-0.25d-dn	3/288	0,792717302	0,915460626	0	0	0,715922108	0,166300554	EEF1B2;FUNDC1;ARL6IP5
Nimesulide-30_mg/kg_in_Corn_Oil-Rat-Kidney-1d-up	3/288	0,792717302	0,915326414	0	0	0,715922108	0,166300554	ARL2BP;RPSA;TPT1
Sparfloxacin-29_mg/kg_in_Corn_Oil-Rat-Liver-3d-up	3/288	0,792717302	0,915192241	0	0	0,715922108	0,166300554	RPS20;RPL39;SEPP1
Stanozolol-39_mg/kg_in_Water-Rat-Liver-1d-up	3/288	0,792717302	0,915058107	0	0	0,715922108	0,166300554	RPSA;SEPP1;TPT1
Zomepirac-2.8_mg/kg_in_Saline-Rat-Kidney-0.25d-up	3/288	0,792717302	0,914924013	0	0	0,715922108	0,166300554	C1QA;RPS3A;RPL39
Diethanolamine-620_mg/kg_in_Water-Rat-Kidney-1d-dn	2/201	0,793020194	0,915139494	0	0	0,683865894	0,158593009	RPS6;RPSA
Azauridine-1500_mg/kg_in_CMC-Rat-Bone_marrow-3d-dn	3/289	0,794646933	0,916882397	0	0	0,713444869	0,163990563	EEF1B2;NKG7;TPT1
Flurbiprofen-10_mg/kg_in_CMC-Rat-Kidney-3d-dn	3/289	0,794646933	0,916748095	0	0	0,713444869	0,163990563	ARL2BP;CLP1;SEPP1
Gallamine_Triethiodide-2.5_mg/kg_in_Saline-Rat-Heart-1d-up	3/289	0,794646933	0,916613832	0	0	0,713444869	0,163990563	RPS19;RPS6;RPL10A
Idarubicin-4_mg/kg_in_Saline-Rat-Kidney-5d-dn	3/289	0,794646933	0,916479608	0	0	0,713444869	0,163990563	C1QA;RPS17;RPS6
Nitrofurantoin-76_mg/kg_in_CMC-Rat-Liver-1d-up	3/289	0,794646933	0,916345424	0	0	0,713444869	0,163990563	CLP1;RPS6;TPT1
Atropine-2.3_mg/kg_in_Water-Rat-Liver-3d-up	4/373	0,794647481	0,916211911	0	0	0,737035092	0,169412441	RPS8;RPS5;RPSA;RPL39
Hydroxyurea-59_mg/kg_in_Saline-Rat-Liver-1d-dn	2/202	0,795293579	0,916822633	0	0	0,680480419	0,155859924	RPS8;SEPP1
Acetone-4000_mg/kg_in_Water-Rat-Kidney-1d-up	4/374	0,796350202	0,917906365	0	0	0,73506441	0,167386101	CLP1;RPS8;SEPP1;TPT1
Acyclovir-980_mg/kg_in_Saline-Rat-Liver-5d-up	3/290	0,796561621	0,918015705	0	0	0,710984714	0,161714033	CLP1;RPS20;GOLGA7
Catechol-195_mg/kg_in_Saline-Rat-Bone_marrow-5d-up	3/290	0,796561621	0,917881394	0	0	0,710984714	0,161714033	NKG7;SEPP1;TPT1
Clofibrate-500_mg/kg_in_Corn_Oil-Rat-Liver-1d-up	3/290	0,796561621	0,917747122	0	0	0,710984714	0,161714033	RPS6;RPS20;RPS3A
Digitoxin-1_mg/kg_in_Corn_Oil-Rat-Heart-0.25d-up	3/290	0,796561621	0,91761289	0	0	0,710984714	0,161714033	RPS5;SEPP1;TPT1

Ethanol-3000_mg/kg_in_Saline-Rat-Bone_marrow-3d-up	3/290	0,796561621	0,917478696	0	0	0,710984714	0,161714033	RPS19;ARL2BP;RPS20
Etoposide-100_mg/kg_in_Corn_Oil-Rat-Bone_marrow-0.25d-up	3/290	0,796561621	0,917344543	0	0	0,710984714	0,161714033	C1QA;NKG7;SLC16A3
Finasteride-800_mg/kg_in_Corn_Oil-Rat-Liver-5d-dn	3/290	0,796561621	0,917210428	0	0	0,710984714	0,161714033	RPS6;RPL10A;RPL39
Hydralazine-280_mg/kg_in_Water-Rat-Heart-1d-up	3/290	0,796561621	0,917076352	0	0	0,710984714	0,161714033	RPS6;ARL6IP5;RAPGEF5
Idarubicin-0.625_mg/kg_in_Saline-Rat-Heart-1d-up	3/290	0,796561621	0,916942316	0	0	0,710984714	0,161714033	RPL10A;RPL39;PDRG1
Olanzapine-23_mg/kg_in_CMC-Rat-Liver-1d-dn	3/290	0,796561621	0,916808319	0	0	0,710984714	0,161714033	C1QA;RPL10A;SEPP1
Perhexiline-320_mg/kg_in_CMC-Rat-Liver-5d-up	3/290	0,796561621	0,916674361	0	0	0,710984714	0,161714033	RPSA;RPS3A;SEPP1
Streptozotocin-138_mg/kg_in_Saline-Rat-Liver-5d-up	3/290	0,796561621	0,916540442	0	0	0,710984714	0,161714033	C1QA;RPS3A;RPL39
Vinblastine-0.3_mg/kg_in_Saline-Rat-Liver-3d-dn	3/290	0,796561621	0,916406562	0	0	0,710984714	0,161714033	CLP1;RPL39;SEPP1
Bisphenol_A-50_mg/kg_in_Corn_Oil-Rat-Liver-0.25d-dn	3/291	0,798461434	0,918458048	0	0	0,708541467	0,159470444	RPS8;RPS6;TPT1
Bromfenac-5_mg/kg_in_CMC-Rat-Liver-1d-dn	3/291	0,798461434	0,918323927	0	0	0,708541467	0,159470444	C1QA;CLP1;SEPP1
Cadmium_Chloride-2_mg/kg_in_Saline-Rat-Kidney-14d-dn	3/291	0,798461434	0,918189846	0	0	0,708541467	0,159470444	RPS17;RPS19;SEPP1
Cyproheptadine-37_mg/kg_in_CMC-Rat-Heart-5d-dn	3/291	0,798461434	0,918055803	0	0	0,708541467	0,159470444	COL5A3;RPL35A;RPS3A
Estriol-313_mg/kg_in_CMC-Rat-Liver-3d-dn	3/291	0,798461434	0,9179218	0	0	0,708541467	0,159470444	C1QA;CLP1;SEPP1
Etoposide-188_mg/kg_in_Corn_Oil-Rat-Kidney-5d-dn	3/291	0,798461434	0,917787836	0	0	0,708541467	0,159470444	RPS17;RAPGEF5;SEPP1
Lansoprazole-240_uM_in_DMSO-Rat-Primary_rat_hepatocytes-0.67d-up	3/291	0,798461434	0,917653911	0	0	0,708541467	0,159470444	EEF1B2;RPL39;PDRG1
Meloxicam-33_mg/kg_in_Corn_Oil-Rat-Liver-0.25d-dn	3/291	0,798461434	0,917520025	0	0	0,708541467	0,159470444	RPS8;RPS3A;TPT1
Neomycin-56_mg/kg_in_Corn_Oil-Rat-Liver-3d-up	3/291	0,798461434	0,917386178	0	0	0,708541467	0,159470444	RPL39;GOLGA7;SEPP1
Sulfadoxine-380_mg/kg_in_Corn_Oil-Rat-Liver-0.25d-dn	3/291	0,798461434	0,917252371	0	0	0,708541467	0,159470444	RPL32;RPS20;SEPP1
Trichloroethylene-1230_mg/kg_in_CMC-Rat-Kidney-5d-dn	3/291	0,798461434	0,917118602	0	0	0,708541467	0,159470444	ARL2BP;RPS6;SEPP1
Carvedilol-2000_mg/kg_in_Corn_Oil-Rat-Liver-5d-dn	3/292	0,800346441	0,919149689	0	0	0,706114956	0,157259281	C1QA;RPS17;RPL35A
Erythromycin-1500_mg/kg_in_CMC-Rat-Kidney-1d-up	3/292	0,800346441	0,919015683	0	0	0,706114956	0,157259281	RPS17;RPS3A;TPT1
Ethylene_Glycol-3525_mg/kg_in_Water-Rat-Bone_marrow-1d-up	3/292	0,800346441	0,918881715	0	0	0,706114956	0,157259281	NKG7;RPL35A;DUSP14
Fludrocortisone_Acetate-125_mg/kg_in_CMC-Rat-Heart-3d-dn	3/292	0,800346441	0,918747787	0	0	0,706114956	0,157259281	ARL2BP;FUNDC1;RPS20
Furan-24_mg/kg_in_Corn_Oil-Rat-Heart-1d-up	3/292	0,800346441	0,918613898	0	0	0,706114956	0,157259281	C1QA;RPS17;SEPP1
Lomustine-8.75_mg/kg_in_Corn_Oil-Rat-Liver-3d-up	3/292	0,800346441	0,918480048	0	0	0,706114956	0,157259281	RPS20;SEPP1;RPL39
Methotrexate-0.3_mg/kg_in_Corn_Oil-Rat-Liver-1d-dn	3/292	0,800346441	0,918346236	0	0	0,706114956	0,157259281	CXCL11;SEPP1;TPT1
NN-Dimethylformamide-1400_mg/kg_in_Saline-Rat-Bone_marrow-1d-up	3/292	0,800346441	0,918212464	0	0	0,706114956	0,157259281	C1QA;NKG7;DUSP14
Naproxen-10_mg/kg_in_Corn_Oil-Rat-Kidney-1d-up	4/377	0,801390524	0,919276401	0	0	0,729215091	0,161453256	RPS20;RPSA;SEPP1;RPL39
Tacrine-24_mg/kg_in_Water-Rat-Heart-3d-up	4/377	0,801390524	0,919142532	0	0	0,729215091	0,161453256	ARL2BP;RPS8;FUNDC1;GOLGA7
1-Naphthyl_Isothiocyanate-30_mg/kg_in_Corn_Oil-Rat-Bone_marrow-3d-dn	3/293	0,802216711	0,919956147	0	0	0,703705007	0,155080042	RPS19;FEM1B;SEPP1
Azathioprine-54_mg/kg_in_Water-Rat-Liver-1d-dn	3/293	0,802216711	0,919822218	0	0	0,703705007	0,155080042	CLP1;RPS8;RPSA
Catechol-40_mg/kg_in_Saline-Rat-Bone_marrow-1d-up	3/293	0,802216711	0,919688329	0	0	0,703705007	0,155080042	COL5A3;NKG7;DUSP14
Cobalt_(II)_Chloride-10_mg/kg_in_Saline-Rat-Heart-5d-up	3/293	0,802216711	0,919554478	0	0	0,703705007	0,155080042	RPS20;SEPP1;RPL39
Cytarabine-23_mg/kg_in_Saline-Rat-Liver-5d-up	3/293	0,802216711	0,919420666	0	0	0,703705007	0,155080042	RPSA;RPL39;TPT1
Doxorubicin-3_mg/kg_in_Saline-Rat-Liver-1d-up	3/293	0,802216711	0,919286893	0	0	0,703705007	0,155080042	ARL2BP;RPS8;RPS20
Ethanol-3000_mg/kg_in_Saline-Rat-Kidney-1d-dn	3/293	0,802216711	0,91915316	0	0	0,703705007	0,155080042	CLP1;RPS3A;RPL10A
Etoposide-188_mg/kg_in_Corn_Oil-Rat-Spleen-1d-up	3/293	0,802216711	0,919019465	0	0	0,703705007	0,155080042	CXCL11;NKG7;SEPP1
Ifosfamide-143_mg/kg_in_Saline-Rat-Liver-5d-dn	3/293	0,802216711	0,918885808	0	0	0,703705007	0,155080042	RPS19;RPL10A;SEPP1
Lipopolysaccharide_E._Coli_O55:B5-1.25_mg/kg_in_Saline-Rat-Liver-1d-dn	3/293	0,802216711	0,918752191	0	0	0,703705007	0,155080042	EEF1B2;C1QA;RPS3A
Lisinopril-2000_mg/kg_in_CMC-Rat-Kidney-1d-up	3/293	0,802216711	0,918618613	0	0	0,703705007	0,155080042	RPS19;FEM1B;RPS3A
Suxamethonium_Chloride-0.125_mg/kg_in_Saline-Rat-Heart-1d-dn	3/293	0,802216711	0,918485073	0	0	0,703705007	0,155080042	RPS8;FEM1B;ARL6IP5
Thioguanine-12_mg/kg_in_Corn_Oil-Rat-Liver-1d-dn	3/293	0,802216711	0,918351572	0	0	0,703705007	0,155080042	RPS17;RPS8;SEPP1
Valproic_Acid-1500_mg/kg_in_Water-Rat-Liver-3d-up	3/293	0,802216711	0,918218111	0	0	0,703705007	0,155080042	RPS3A;RPL10A;RPL39
Benzyl_Acetate-1868_mg/kg_in_CMC-Rat-Kidney-1d-dn	3/294	0,804072316	0,920208306	0	0	0,701311452	0,152932231	RPS19;FEM1B;SEPP1
Citalopram-40_mg/kg_in_Corn_Oil-Rat-Liver-3d-up	3/294	0,804072316	0,920074613	0	0	0,701311452	0,152932231	SEPP1;RPL39;GOLGA7
Clomiphene-250_mg/kg_in_CMC-Rat-Liver-3d-dn	3/294	0,804072316	0,919940959	0	0	0,701311452	0,152932231	CLP1;RPS8;SEPP1
Fluvastatin-94_mg/kg_in_Corn_Oil-Rat-Liver-0.25d-up	3/294	0,804072316	0,919807344	0	0	0,701311452	0,152932231	RPS19;RPS6;SEPP1
Metoclopramide-185_mg/kg_in_CMC-Rat-Intestine-1d-up	3/294	0,804072316	0,919673767	0	0	0,701311452	0,152932231	RPS8;SLC16A3;TPT1
NN'-Diphenyl-P-Phenylenediamine-1000_mg/kg_in_CMC-Rat-Kidney-3d-up	3/294	0,804072316	0,919540229	0	0	0,701311452	0,152932231	RPS17;RPS3A;SEPP1
Penicillamine-852_mg/kg_in_Water-Rat-Bone_marrow-1d-up	3/294	0,804072316	0,919406731	0	0	0,701311452	0,152932231	NKG7;RPL10A;SEPP1
Primidone-750_mg/kg_in_CMC-Rat-Liver-5d-up	3/294	0,804072316	0,91927327	0	0	0,701311452	0,152932231	RPS19;RPSA;RPL10A
Pyrilamine-688_mg/kg_in_Water-Rat-Heart-3d-up	3/294	0,804072316	0,919139849	0	0	0,701311452	0,152932231	RAPGEF5;SEPP1;GOLGA7
Rofecoxib-1550_mg/kg_in_Corn_Oil-Rat-Liver-5d-up	3/294	0,804072316	0,919006466	0	0	0,701311452	0,152932231	EEF1B2;RPSA;SEPP1
Saquinavir-1500_mg/kg_in_CMC-Rat-Kidney-1d-up	3/294	0,804072316	0,918873123	0	0	0,701311452	0,152932231	EEF1B2;RPS6;RPS20
Streptozotocin-138_mg/kg_in_Saline-Rat-Liver-3d-up	3/294	0,804072316	0,918739817	0	0	0,701311452	0,152932231	RPS17;RPL32;RPL10A
Thioguanine-12_mg/kg_in_Corn_Oil-Rat-Liver-3d-up	3/294	0,804072316	0,918606551	0	0	0,701311452	0,152932231	RPS8;RPSA;RPL39
1-Naphthyl_Isothiocyanate-30_mg/kg_in_Corn_Oil-Rat-Kidney-3d-dn	3/295	0,805913325	0,920576265	0	0	0,698934125	0,150815362	CLP1;RPSA;SEPP1
Acetazolamide-250_mg/kg_in_Corn_Oil-Rat-Kidney-5d-dn	3/295	0,805913325	0,920442771	0	0	0,698934125	0,150815362	CLP1;SEPP1;TPT1
Bromobenzene-439_mg/kg_in_CMC-Rat-Kidney-1d-dn	3/295	0,805913325	0,920309315	0	0	0,698934125	0,150815362	EEF1B2;RPS19;ARL6IP5
Catechol-195_mg/kg_in_Saline-Rat-Bone_marrow-1d-up	3/295	0,805913325	0,920175898	0	0	0,698934125	0,150815362	COL5A3;RPL35A;DUSP14
Clomipramine-7_uM_in_DMSO-Rat-Primary_rat_hepatocytes-1d-up	3/295	0,805913325	0,92004252	0	0	0,698934125	0,150815362	ARL2BP;RPS8;RPS5
Crotamiton-750_mg/kg_in_Corn_Oil-Rat-Liver-5d-dn	3/295	0,805913325	0,919909181	0	0	0,698934125	0,150815362	EEF1B2;SEPP1;RPL39
Fluoxetine-52_mg/kg_in_CMC-Rat-Kidney-5d-dn	3/295	0,805913325	0,91977588	0	0	0,698934125	0,150815362	RPL32;RPL35A;RPS20
Gallamine_Triethiodide-2.5_mg/kg_in_Saline-Rat-Heart-5d-dn	3/295	0,805913325	0,919642618	0	0	0,698934125	0,150815362	EEF1B2;RAPGEF5;SEPP1
Genistein-375_mg/kg_in_Corn_Oil-Rat-Liver-1d-dn	3/295	0,805913325	0,919509394	0	0	0,698934125	0,150815362	RPS17;RPL39;TPT1
Hydrocortisone-56_mg/kg_in_Corn_Oil-Rat-Bone_marrow-5d-up	3/295	0,805913325	0,919376209	0	0	0,698934125	0,150815362	RPL32;RPS5;SEPP1
Lomustine-8.75_mg/kg_in_Corn_Oil-Rat-Bone_marrow-3d-dn	3/295	0,805913325	0,919243063	0	0	0,698934125	0,150815362	RPS19;FEM1B;NKG7
Mitomycin_C-0.5_mg/kg_in_Saline-Rat-Liver-5d-dn	3/295	0,805913325	0,919109955	0	0	0,698934125	0,150815362	C1QA;CLP1;RPSA
Norepinephrine-1.75_mg/kg_in_Saline-Rat-Heart-3d-dn	3/295	0,805913325	0,918976885	0	0	0,698934125	0,150815362	RPL32;RPS6;RPS20
Terbinafine-2000_mg/kg_in_CMC-Rat-Liver-5d-up	3/295	0,805913325	0,918843855	0	0	0,698934125	0,150815362	RPS17;RPSA;RPL39
4-Methylpyrazole-155_mg/kg_in_Saline-Rat-Kidney-1d-up	3/296	0,80773981	0,920792986	0	0	0,696572862	0,148728957	CLP1;RPS8;SEPP1
4-Nitrobenzoic_Acid-1470_mg/kg_in_CMC-Rat-Kidney-3d-dn	3/296	0,80773981	0,920659731	0	0	0,696572862	0,148728957	RPL32;ARL2BP;CLP1
44'-Methylenedianiline-296_uM_in_DMSO-Rat-Primary_rat_hepatocytes-0.67d-dn	3/296	0,80773981	0,920526514	0	0	0,696572862	0,148728957	CXCL11;RPS6;SEPP1
Acetaminophen-486_mg/kg_in_Corn_Oil-Rat-Liver-3d-dn	3/296	0,80773981	0,920393336	0	0	0,696572862	0,148728957	EEF1B2;C1QA;TPT1
Allyl_Alcohol-16_mg/kg_in_Saline-Rat-Kidney-1d-dn	3/296	0,80773981	0,920260197	0	0	0,696572862	0,148728957	EEF1B2;RPS19;ARL2BP
Azithromycin-225_mg/kg_in_Water-Rat-Liver-5d-dn	3/296	0,80773981	0,920127096	0	0	0,696572862	0,148728957	RPS8;RPS6;RPL39
Beta-Naphthoflavone-1500_mg/kg_in_CMC-Rat-Liver-5d-dn	3/296	0,80773981	0,919994033	0	0	0,696572862	0,148728957	RPS20;RPL39;TPT1
Celecoxib-400_mg/kg_in_Corn_Oil-Rat-Liver-5d-up	3/296	0,80773981	0,919861009	0	0	0,696572862	0,148728957	C1QA;RPS8;SEPP1
Clomiphene-250_mg/kg_in_CMC-Rat-Liver-1d-dn	3/296	0,80773981	0,919728024	0	0	0,696572862	0,148728957	RPL35A;RPS20;SEPP1
Daunorubicin-3.25_mg/kg_in_Saline-Rat-Bone_marrow-1d-up	3/296	0,80773981	0,919595077	0	0	0,696572862	0,148728957	C1QA;RPS19;SEPP1
Diphenidol-300_mg/kg_in_Water-Rat-Heart-1d-dn	3/296	0,80773981	0,919462168	0	0	0,696572862	0,148728957	EEF1B2;FUNDC1;RPL39
Doxorubicin-1.5_uM_in_DMSO-Rat-Primary_rat_hepatocytes-0.67d-dn	3/296	0,80773981	0,919329298	0	0	0,696572862	0,148728957	RPSA;SLC16A3;TPT1
Etoposide-100_mg/kg_in_Corn_Oil-Rat-Spleen-1d-up	3/296	0,80773981	0,919196466	0	0	0,696572862	0,148728957	C1QA;NKG7;SEPP1
Fluconazole-394_mg/kg_in_Corn_Oil-Rat-Liver-5d-dn	3/296	0,80773981	0,919063672	0	0	0,696572862	0,148728957	C1QA;RPS17;SEPP1
Gemfibrozil-700_mg/kg_in_Corn_Oil-Rat-Liver-1d-dn	3/296	0,80773981	0,918930917	0	0	0,696572862	0,148728957	C1QA;RPS17;CLP1
Granisetron-175_mg/kg_in_Water-Rat-Liver-1d-dn	3/296	0,80773981	0,9187982	0	0	0,696572862	0,148728957	C1QA;CLP1;SEPP1
Idarubicin-0.625_mg/kg_in_Saline-Rat-Heart-5d-up	3/296	0,80773981	0,918665522	0	0	0,696572862	0,148728957	RPS17;RPL32;TPT1
Idarubicin-1_mg/kg_in_Saline-Rat-Kidney-5d-up	3/296	0,80773981	0,918532882	0	0	0,696572862	0,148728957	CLP1;RPS20;RPL39
Ifosfamide-143_mg/kg_in_Saline-Rat-Liver-3d-up	3/296	0,80773981	0,91840028	0	0	0,696572862	0,148728957	RPS19;RPSA;SEPP1

Isoprenaline-4.2_mg/kg_in_Saline-Rat-Heart-5d-dn	3/296	0,80773981	0,918267716	0	0	0,696572862	0,148728957	ARL2BP;FUNDC1;RAPGEF5
Lead_(II)_Acetate-2_mg/kg_in_Saline-Rat-Kidney-28d-dn	3/296	0,80773981	0,918135191	0	0	0,696572862	0,148728957	RPS6;RPSA;SEPP1
Nisoldipine-15_mg/kg_in_Water-Rat-Liver-5d-up	3/296	0,80773981	0,918002704	0	0	0,696572862	0,148728957	RPS20;SEPP1;GOLGA7
Prednisolone-184_mg/kg_in_Water-Rat-Kidney-3d-up	3/296	0,80773981	0,917870255	0	0	0,696572862	0,148728957	SEPP1;RPL39;TPT1
Acyclovir-980_mg/kg_in_Saline-Rat-Spleen-3d-up	3/297	0,809551841	0,919796639	0	0	0,694227498	0,146672545	RPS19;RPS6;RPL39
Allyl_Alcohol-16_mg/kg_in_Saline-Rat-Liver-5d-up	3/297	0,809551841	0,919663969	0	0	0,694227498	0,146672545	C1QA;SEPP1;GOLGA7
Artemisinin-2000_mg/kg_in_CMC-Rat-Liver-5d-dn	3/297	0,809551841	0,919531338	0	0	0,694227498	0,146672545	EEF1B2;C1QA;RPL32
Aspirin-375_mg/kg_in_Corn_Oil-Rat-Liver-5d-dn	3/297	0,809551841	0,919398745	0	0	0,694227498	0,146672545	C1QA;RPSA;SEPP1
Bezafibrate-617_mg/kg_in_Corn_Oil-Rat-Kidney-3d-dn	3/297	0,809551841	0,919266191	0	0	0,694227498	0,146672545	EEF1B2;RPS17;SEPP1
Choline_Chloride-2550_mg/kg_in_Water-Rat-Liver-5d-dn	3/297	0,809551841	0,919133674	0	0	0,694227498	0,146672545	EEF1B2;RPL39;TPT1
Cortisone-206_mg/kg_in_CMC-Rat-Liver-5d-up	3/297	0,809551841	0,919001196	0	0	0,694227498	0,146672545	RPSA;RPL10A;TPT1
Daunorubicin-4_mg/kg_in_Saline-Rat-Heart-14d-dn	3/297	0,809551841	0,918868756	0	0	0,694227498	0,146672545	FUNDC1;ARL6IP5;RPSA
Etoposide-188_mg/kg_in_Corn_Oil-Rat-Kidney-3d-dn	3/297	0,809551841	0,918736354	0	0	0,694227498	0,146672545	EEF1B2;RPS6;RPL39
Megestrol_Acetate-132_mg/kg_in_CMC-Rat-Liver-5d-up	3/297	0,809551841	0,918603991	0	0	0,694227498	0,146672545	C1QA;RPSA;RPL39
Nimetazepam-122_mg/kg_in_CMC-Rat-Liver-5d-up	3/297	0,809551841	0,918471665	0	0	0,694227498	0,146672545	C1QA;RPSA;RPL10A
Prednisone-68_mg/kg_in_CMC-Rat-Heart-5d-dn	3/297	0,809551841	0,918339378	0	0	0,694227498	0,146672545	COL5A3;SEPP1;GOLGA7
Simvastatin-160_uM_in_DMSO-Rat-Primary_rat_hepatocytes-0.67d-up	3/297	0,809551841	0,918207128	0	0	0,694227498	0,146672545	RPS6;RPL39;TPT1
Allyl_Alcohol-16_mg/kg_in_Saline-Rat-Bone_marrow-3d-dn	2/209	0,81059896	0,919262405	0	0	0,657689209	0,138102796	RPL32;NKG7
123-Trichloropropane-108_mg/kg_in_CMC-Rat-Kidney-5d-dn	3/298	0,811349491	0,919981082	0	0	0,691897876	0,144645665	RPS6;RPS20;SEPP1
Alpha-Naphthoflavone-80_mg/kg_in_CMC-Rat-Liver-91d-up	3/298	0,811349491	0,919848653	0	0	0,691897876	0,144645665	RPS8;SEPP1;TPT1
Granisetron-175_mg/kg_in_Water-Rat-Liver-5d-dn	3/298	0,811349491	0,919716263	0	0	0,691897876	0,144645665	EEF1B2;CLP1;TPT1
Ibuprofen-54_mg/kg_in_Corn_Oil-Rat-Liver-0.25d-up	3/298	0,811349491	0,91958391	0	0	0,691897876	0,144645665	EEF1B2;RPS6;RPSA
Norethindrone-0.08_mg/kg_in_Corn_Oil-Rat-Liver-1d-dn	3/298	0,811349491	0,919451596	0	0	0,691897876	0,144645665	RPS3A;RPL39;TPT1
Terfenadine-750_mg/kg_in_CMC-Rat-Heart-5d-dn	3/298	0,811349491	0,91931932	0	0	0,691897876	0,144645665	COL5A3;SEPP1;TPT1
Testosterone-375_mg/kg_in_CMC-Rat-Liver-5d-dn	3/298	0,811349491	0,919187082	0	0	0,691897876	0,144645665	C1QA;RPS6;RPL39
Allyl_Alcohol-32_mg/kg_in_Saline-Rat-Liver-3d-dn	3/299	0,813132832	0,921074959	0	0	0,689583836	0,142647862	C1QA;SEPP1;TPT1
Bromfenac-5_mg/kg_in_CMC-Rat-Kidney-3d-up	3/299	0,813132832	0,920942506	0	0	0,689583836	0,142647862	RPS17;RPS3A;SEPP1
Busulfan-3_mg/kg_in_Corn_Oil-Rat-Liver-0.25d-up	3/299	0,813132832	0,920810092	0	0	0,689583836	0,142647862	RPL10A;SEPP1;RPL39
Cyclopropane_Carboxylic_Acid-375_mg/kg_in_Water-Rat-Liver-3d-up	3/299	0,813132832	0,920677715	0	0	0,689583836	0,142647862	RPS8;RPS3A;TPT1
DActinomycin-0.06_mg/kg_in_Saline-Rat-Bone_marrow-5d-dn	3/299	0,813132832	0,920545377	0	0	0,689583836	0,142647862	SEPP1;RPL39;DUSP14
Finasteride-25_mg/kg_in_Corn_Oil-Rat-Liver-1d-up	3/299	0,813132832	0,920413077	0	0	0,689583836	0,142647862	RPS19;RPS8;FEM1B
Nateglinide-625_mg/kg_in_CMC-Rat-Liver-5d-dn	3/299	0,813132832	0,920280815	0	0	0,689583836	0,142647862	EEF1B2;CLP1;RPL39
Norethindrone-375_mg/kg_in_Corn_Oil-Rat-Liver-1d-dn	3/299	0,813132832	0,92014859	0	0	0,689583836	0,142647862	C1QA;SEPP1;TPT1
Pirinixic_Acid-364_mg/kg_in_CMC-Rat-Liver-5d-dn	3/299	0,813132832	0,920016404	0	0	0,689583836	0,142647862	C1QA;CLP1;SEPP1
Sodium_Arsenite-30_mg/kg_in_Saline-Rat-Liver-3d-dn	3/299	0,813132832	0,919884256	0	0	0,689583836	0,142647862	C1QA;CLP1;SEPP1
Dexfenfluramine-29_mg/kg_in_Water-Rat-Kidney-1d-up	4/385	0,814340751	0,921118448	0	0	0,71406257	0,146651591	EEF1B2;CLP1;SEPP1;TPT1
Cinnarizine-750_mg/kg_in_CMC-Rat-Liver-1d-dn	3/300	0,814901937	0,921620858	0	0	0,687285223	0,140678691	EEF1B2;RPS20;SEPP1
Fenofibrate-215_mg/kg_in_Corn_Oil-Rat-Liver-5d-dn	3/300	0,814901937	0,921488537	0	0	0,687285223	0,140678691	EEF1B2;RPSA;SEPP1
Fluvastatin-94_mg/kg_in_Corn_Oil-Rat-Skeletal_muscle-5d-dn	3/300	0,814901937	0,921356253	0	0	0,687285223	0,140678691	COL5A3;ARL6IP5;SLC16A3
Methotrexate-0.3_mg/kg_in_Corn_Oil-Rat-Liver-5d-dn	3/300	0,814901937	0,921224007	0	0	0,687285223	0,140678691	C1QA;CLP1;TPT1
N-Nitrosodiethylamine-100_mg/kg_in_Saline-Rat-Liver-1d-dn	3/300	0,814901937	0,921091799	0	0	0,687285223	0,140678691	RPS17;RPL32;RPL10A
Nitrazepam-310_mg/kg_in_CMC-Rat-Liver-3d-up	3/300	0,814901937	0,920959629	0	0	0,687285223	0,140678691	RPS6;RPS3A;RPL39
Penicillamine-852_mg/kg_in_Water-Rat-Liver-1d-dn	3/300	0,814901937	0,920827498	0	0	0,687285223	0,140678691	RPS17;RPL10A;SEPP1
Racepinephrine-0.77_mg/kg_in_Saline-Rat-Heart-1d-up	3/300	0,814901937	0,920695403	0	0	0,687285223	0,140678691	RPS8;ARL6IP5;RAPGEF5
Tocainide-67_mg/kg_in_Corn_Oil-Rat-Liver-1d-dn	3/300	0,814901937	0,920563347	0	0	0,687285223	0,140678691	EEF1B2;RPL32;RPL10A
Bisphenol_A-610_mg/kg_in_Corn_Oil-Rat-Liver-0.25d-dn	3/301	0,816656879	0,922413534	0	0	0,685001884	0,138737713	RPS20;RPL39;GOLGA7
Clomipramine-115_mg/kg_in_Water-Rat-Liver-1d-dn	3/301	0,816656879	0,92228127	0	0	0,685001884	0,138737713	RPS19;RPS20;TPT1
Diethylstilbestrol-280_mg/kg_in_Corn_Oil-Rat-Kidney-1d-up	3/301	0,816656879	0,922149043	0	0	0,685001884	0,138737713	RPS19;RPS20;TPT1
Galantamine-110_mg/kg_in_CMC-Rat-Heart-5d-dn	3/301	0,816656879	0,922016854	0	0	0,685001884	0,138737713	ARL2BP;RPS8;FUNDC1
Haloperidol-21_mg/kg_in_Corn_Oil-Rat-Heart-3d-dn	3/301	0,816656879	0,921884703	0	0	0,685001884	0,138737713	RPS6;RPL13A;TPT1
Sparfloxacin-29_mg/kg_in_Corn_Oil-Rat-Liver-5d-dn	3/301	0,816656879	0,92175259	0	0	0,685001884	0,138737713	RPSA;RPL10A;SEPP1
Sulfisoxazole-250_mg/kg_in_Corn_Oil-Rat-Liver-1d-dn	3/301	0,816656879	0,921620515	0	0	0,685001884	0,138737713	RPS6;RPSA;RPL39
Thioguanine-24_mg/kg_in_Corn_Oil-Rat-Kidney-3d-up	3/301	0,816656879	0,921488478	0	0	0,685001884	0,138737713	RPS8;RPS3A;SEPP1
Tocainide-224_mg/kg_in_Corn_Oil-Rat-Liver-3d-dn	3/301	0,816656879	0,921356478	0	0	0,685001884	0,138737713	RPSA;RPL10A;TPT1
Bromhexine-1200_mg/kg_in_CMC-Rat-Liver-3d-dn	3/302	0,81839773	0,923188273	0	0	0,682733666	0,136824495	C1QA;RPL39;TPT1
Ebastine-2000_mg/kg_in_Water-Rat-Heart-3d-up	3/302	0,81839773	0,923056067	0	0	0,682733666	0,136824495	RPS8;RPS6;GOLGA7
Etodolac-24_mg/kg_in_CMC-Rat-Liver-5d-dn	3/302	0,81839773	0,9229239	0	0	0,682733666	0,136824495	RPS8;RPS20;SEPP1
Gentamicin-267_mg/kg_in_Corn_Oil-Rat-Liver-5d-up	3/302	0,81839773	0,922791771	0	0	0,682733666	0,136824495	C1QA;RAPGEF5;SEPP1
Isotretinoin-125_mg/kg_in_Corn_Oil-Rat-Liver-0.25d-dn	3/302	0,81839773	0,922659679	0	0	0,682733666	0,136824495	EEF1B2;SEPP1;TPT1
Sodium_Arsenite-10_mg/kg_in_Saline-Rat-Kidney-3d-dn	3/302	0,81839773	0,922527625	0	0	0,682733666	0,136824495	CLP1;RPS6;RPL10A
Tranexamic_Acid-2000_mg/kg_in_CMC-Rat-Heart-5d-dn	3/302	0,81839773	0,922395609	0	0	0,682733666	0,136824495	RPS8;RPL35A;RPS3A
Vinblastine-45_uM_in_DMSO-Rat-Primary_rat_hepatocytes-0.67d-dn	3/302	0,81839773	0,922263631	0	0	0,682733666	0,136824495	CXCL11;SEPP1;TPT1
33'5-Triiodo-L-Thyronine-0.001_mg/kg_in_PEG400-Rat-Heart-1d-up	3/303	0,820124564	0,924077406	0	0	0,680480419	0,134938616	ARL2BP;FUNDC1;RAPGEF5
Altretamine-13_mg/kg_in_Corn_Oil-Rat-Liver-3d-up	3/303	0,820124564	0,923945225	0	0	0,680480419	0,134938616	RPS20;RPSA;SEPP1
Carbimazole-400_mg/kg_in_Water-Rat-Liver-5d-dn	3/303	0,820124564	0,923813082	0	0	0,680480419	0,134938616	EEF1B2;C1QA;TPT1
Cisplatin-1.17_mg/kg_in_Saline-Rat-Bone_marrow-3d-up	3/303	0,820124564	0,923680976	0	0	0,680480419	0,134938616	C1QA;RPS19;SEPP1
Diethylstilbestrol-2.8_mg/kg_in_Corn_Oil-Rat-Kidney-0.25d-up	3/303	0,820124564	0,923548909	0	0	0,680480419	0,134938616	FUNDC1;RPL10A;SEPP1
Irinotecan-5_mg/kg_in_Saline-Rat-Bone_marrow-5d-dn	3/303	0,820124564	0,923416879	0	0	0,680480419	0,134938616	RPS3A;RPL39;DUSP14
Methyl_Salicylate-444_mg/kg_in_Corn_Oil-Rat-Kidney-1d-dn	3/303	0,820124564	0,923284887	0	0	0,680480419	0,134938616	RPL10A;SEPP1;PDRG1
Piracetam-2500_mg/kg_in_CMC-Rat-Brain-1d-up	3/303	0,820124564	0,923152932	0	0	0,680480419	0,134938616	ARL2BP;RPS8;FUNDC1
Sulindac-64_mg/kg_in_Corn_Oil-Rat-Liver-5d-dn	3/303	0,820124564	0,923021016	0	0	0,680480419	0,134938616	ARL2BP;RPS8;SEPP1
Amineptine-1667_uM_in_DMSO-Rat-Primary_rat_hepatocytes-0.67d-dn	3/304	0,821837456	0,92481666	0	0	0,678241997	0,133079657	CXCL11;ARL6IP5;SEPP1
Bis(2-Ethylhexyl)Phthalate-100_mg/kg_in_Corn_Oil-Rat-Liver-0.25d-dn	3/304	0,821837456	0,924684543	0	0	0,678241997	0,133079657	RPS17;CLP1;TPT1
Fenbendazole-375_mg/kg_in_Corn_Oil-Rat-Liver-3d-up	3/304	0,821837456	0,924552464	0	0	0,678241997	0,133079657	RPS19;RPS6;RPL10A
Flunarizine-126_mg/kg_in_CMC-Rat-Heart-3d-dn	3/304	0,821837456	0,924420423	0	0	0,678241997	0,133079657	SEPP1;GOLGA7;TPT1
Fluocinolone_Acetonide-2.5_mg/kg_in_Corn_Oil-Rat-Heart-1d-up	3/304	0,821837456	0,92428842	0	0	0,678241997	0,133079657	RPS3A;SEPP1;PDRG1
Leflunomide-30_mg/kg_in_Corn_Oil-Rat-Spleen-0.25d-dn	3/304	0,821837456	0,924156454	0	0	0,678241997	0,133079657	RPS17;RPS19;RPS5
Methyldopa-325_mg/kg_in_Water-Rat-Liver-1d-up	3/304	0,821837456	0,924024526	0	0	0,678241997	0,133079657	EEF1B2;CLP1;RPS20
Mifepristone-3_mg/kg_in_Corn_Oil-Rat-Liver-1d-up	3/304	0,821837456	0,923892635	0	0	0,678241997	0,133079657	RPS19;RPL39;GOLGA7
Phenacetin-619_mg/kg_in_Corn_Oil-Rat-Liver-0.25d-dn	3/304	0,821837456	0,923760782	0	0	0,678241997	0,133079657	C1QA;RPL39;TPT1
Sertraline-210_mg/kg_in_Corn_Oil-Rat-Liver-5d-up	3/304	0,821837456	0,923628967	0	0	0,678241997	0,133079657	RPS20;RPSA;SEPP1
Thioguanine-12_mg/kg_in_Corn_Oil-Rat-Liver-0.25d-dn	3/304	0,821837456	0,92349719	0	0	0,678241997	0,133079657	C1QA;RPS8;SEPP1
Altretamine-13_mg/kg_in_Corn_Oil-Rat-Bone_marrow-3d-up	3/305	0,823536479	0,925274366	0	0	0,676018252	0,131247208	C1QA;RPS19;SEPP1
Aspirin-375_mg/kg_in_Corn_Oil-Rat-Liver-1d-dn	3/305	0,823536479	0,925142392	0	0	0,676018252	0,131247208	C1QA;RPL39;TPT1
Cholecalciferol-8_mg/kg_in_CMC-Rat-Kidney-1d-up	3/305	0,823536479	0,925010455	0	0	0,676018252	0,131247208	EEF1B2;RPS17;CLP1
Cisplatin-1.17_mg/kg_in_Saline-Rat-Liver-3d-dn	3/305	0,823536479	0,924878555	0	0	0,676018252	0,131247208	CLP1;RPS3A;SEPP1
Labetalol-2500_mg/kg_in_Water-Rat-Heart-1d-dn	3/305	0,823536479	0,924746693	0	0	0,676018252	0,131247208	EEF1B2;RPS3A;TPT1
Labetalol-60_mg/kg_in_Water-Rat-Heart-5d-dn	3/305	0,823536479	0,924614869	0	0	0,676018252	0,131247208	EEF1B2;RPS8;TPT1
Lipopolysaccharide_E._Coli_O55:B5-1.25_mg/kg_in_Saline-Rat-Kidney-3d-dn	3/305	0,823536479	0,924483083	0	0	0,676018252	0,131247208	EEF1B2;RPS17;RPL35A
Prednisone-68_mg/kg_in_CMC-Rat-Kidney-5d-dn	3/305	0,823536479	0,924351334	0	0	0,676018252	0,131247208	RPS19;CLP1;SEPP1

Sulindac-64_mg/kg_in_Corn_Oil-Rat-Liver-3d-up	3/305	0,823536479	0,924219622	0	0	0,676018252	0,131247208	RPL10A;SEPP1;RPL39
Valsartan-1000_mg/kg_in_Corn_Oil-Rat-Kidney-5d-up	3/305	0,823536479	0,924087948	0	0	0,676018252	0,131247208	ARL2BP;RPS6;RPS20
Vincristine-0.05_mg/kg_in_Saline-Rat-Bone_marrow-5d-dn	3/305	0,823536479	0,923956312	0	0	0,676018252	0,131247208	RPL39;TPT1;DUSP14
Dimethyl_Methylphosphonate-1500_mg/kg_in_Water-Rat-Kidney-1d-up	4/391	0,82359481	0,923890147	0	0	0,703105088	0,136456249	RPS3A;SEPP1;TOMM20;TPT1
Trimethadione-535_mg/kg_in_Water-Rat-Kidney-1d-up	4/391	0,82359481	0,923758576	0	0	0,703105088	0,136456249	CLP1;RPS8;SEPP1;TPT1
Acetaminophen-486_mg/kg_in_Corn_Oil-Rat-Kidney-5d-dn	3/306	0,825221708	0,925451541	0	0	0,673809043	0,129440868	RPSA;RPL10A;TPT1
Azathioprine-54_mg/kg_in_Water-Rat-Liver-5d-dn	3/306	0,825221708	0,925319785	0	0	0,673809043	0,129440868	C1QA;RPS8;RPSA
Cholecalciferol-8_mg/kg_in_CMC-Rat-Liver-3d-dn	3/306	0,825221708	0,925188067	0	0	0,673809043	0,129440868	CLP1;RPS20;TPT1
Clobetasol_Propionate-17_mg/kg_in_Corn_Oil-Rat-Bone_marrow-3d-up	3/306	0,825221708	0,925056386	0	0	0,673809043	0,129440868	C1QA;NKG7;TPT1
Doxorubicin-0.65_mg/kg_in_Saline-Rat-Spleen-3d-up	3/306	0,825221708	0,924924743	0	0	0,673809043	0,129440868	RPS19;RPL10A;SEPP1
Ethinylestradiol-1480_mg/kg_in_Corn_Oil-Rat-Liver-1d-dn	3/306	0,825221708	0,924793137	0	0	0,673809043	0,129440868	C1QA;SEPP1;TPT1
Progesterone-11.3_mg/kg_in_Corn_Oil-Rat-Liver-3d-dn	3/306	0,825221708	0,924661569	0	0	0,673809043	0,129440868	EEF1B2;RPS17;RPSA
Propylene_Glycol-2000_mg/kg_in_Water-Rat-Liver-3d-dn	3/306	0,825221708	0,924530038	0	0	0,673809043	0,129440868	RPS19;RPL39;TPT1
Risperidone-22_mg/kg_in_CMC-Rat-Heart-1d-up	3/306	0,825221708	0,924398545	0	0	0,673809043	0,129440868	EEF1B2;RPS20;RPS3A
Safrole-488_mg/kg_in_Corn_Oil-Rat-Liver-0.25d-dn	3/306	0,825221708	0,924267089	0	0	0,673809043	0,129440868	EEF1B2;RPL39;TPT1
Tocainide-224_mg/kg_in_Corn_Oil-Rat-Liver-1d-dn	3/306	0,825221708	0,92413567	0	0	0,673809043	0,129440868	RPS17;RPSA;RPS3A
Propylene_Glycol-2000_mg/kg_in_Water-Rat-Kidney-1d-up	4/393	0,826593955	0,9255408	0	0	0,699526945	0,133219094	CLP1;RPS8;SEPP1;TPT1
33'4'5-Tetrachlorosalicylanilide-50_mg/kg_in_Corn_Oil-Rat-Liver-1d-up	3/307	0,826893217	0,925744276	0	0	0,671614225	0,127660239	CLP1;RPSA;TPT1
Azathioprine-160_mg/kg_in_Corn_Oil-Rat-Liver-0.25d-dn	3/307	0,826893217	0,925612703	0	0	0,671614225	0,127660239	RPS20;SEPP1;TPT1
Cyclophosphamide-25_mg/kg_in_Water-Rat-Heart-5d-dn	3/307	0,826893217	0,925481168	0	0	0,671614225	0,127660239	RPS8;RPL35A;TPT1
Methimazole-28_mg/kg_in_Water-Rat-Liver-1d-dn	3/307	0,826893217	0,92534967	0	0	0,671614225	0,127660239	RPS19;CLP1;SEPP1
Nevirapine-29_mg/kg_in_Saline-Rat-Liver-0.25d-dn	3/307	0,826893217	0,92521821	0	0	0,671614225	0,127660239	RPS8;RPS20;RPL39
Sulfadiazine-1170_mg/kg_in_Water-Rat-Liver-5d-dn	3/307	0,826893217	0,925086787	0	0	0,671614225	0,127660239	EEF1B2;RPL39;TPT1
Tacrolimus-134_mg/kg_in_CMC-Rat-Kidney-3d-dn	3/307	0,826893217	0,924955401	0	0	0,671614225	0,127660239	RPSA;SEPP1;RPL39
Vinorelbine-1.5_mg/kg_in_Saline-Rat-Liver-5d-up	3/307	0,826893217	0,924824053	0	0	0,671614225	0,127660239	RPS20;RPL39;GOLGA7
2378-Tetrachlorodibenzo-P-Dioxin-0.02_mg/kg_in_CMC-Rat-Liver-5d-dn	3/308	0,828551082	0,926546688	0	0	0,669433659	0,125904932	C1QA;RPL39;TPT1
Bacitracin-380_mg/kg_in_Corn_Oil-Rat-Kidney-5d-dn	3/308	0,828551082	0,926415151	0	0	0,669433659	0,125904932	EEF1B2;CLP1;SEPP1
Famciclovir-112_mg/kg_in_Saline-Rat-Liver-3d-dn	3/308	0,828551082	0,926283651	0	0	0,669433659	0,125904932	SEPP1;RPL39;TPT1
Nafenopin-338_mg/kg_in_Corn_Oil-Rat-Liver-5d-dn	3/308	0,828551082	0,926152189	0	0	0,669433659	0,125904932	RPS6;SEPP1;RPL39
Pemoline-70_mg/kg_in_CMC-Rat-Liver-3d-up	3/308	0,828551082	0,926020764	0	0	0,669433659	0,125904932	RPS6;RAPGEF5;RPL39
Valproic_Acid-1500_mg/kg_in_Water-Rat-Liver-1d-dn	3/308	0,828551082	0,925889376	0	0	0,669433659	0,125904932	C1QA;CLP1;SEPP1
Alprazolam-115_mg/kg_in_Corn_Oil-Rat-Liver-1d-dn	3/309	0,830195377	0,927595232	0	0	0,667267207	0,124174565	C1QA;RPS8;RPL35A
Phenylbutazone-368_mg/kg_in_CMC-Rat-Kidney-1d-dn	3/309	0,830195377	0,927463658	0	0	0,667267207	0,124174565	EEF1B2;RPL39;TPT1
Progesterone-164_mg/kg_in_Corn_Oil-Rat-Liver-0.25d-dn	3/309	0,830195377	0,927332122	0	0	0,667267207	0,124174565	RPS17;SEPP1;RPL39
Salicylamide-1300_mg/kg_in_CMC-Rat-Kidney-1d-up	3/309	0,830195377	0,927200623	0	0	0,667267207	0,124174565	CLP1;RPS3A;SEPP1
Sildenafil-300_mg/kg_in_Saline-Rat-Liver-5d-up	3/309	0,830195377	0,927069161	0	0	0,667267207	0,124174565	RPS19;RPS20;GOLGA7
Tamoxifen-2.5_mg/kg_in_Corn_Oil-Rat-Liver-5d-dn	3/309	0,830195377	0,926937736	0	0	0,667267207	0,124174565	RPSA;RPL10A;SEPP1
Hydroxyurea-59_mg/kg_in_Saline-Rat-Liver-3d-dn	2/219	0,830697379	0,927366769	0	0	0,627657738	0,116424055	RPS8;SEPP1
Beta-Estradiol_3-Benzoate-0.25_mg/kg_in_Corn_Oil-Rat-Liver-1d-dn	3/310	0,831826179	0,928495321	0	0	0,665114732	0,122468759	ARL6IP5;SEPP1;GOLGA7
Bromhexine-1200_mg/kg_in_CMC-Rat-Liver-5d-dn	3/310	0,831826179	0,92836375	0	0	0,665114732	0,122468759	EEF1B2;C1QA;TPT1
Cilostazol-1500_mg/kg_in_CMC-Rat-Heart-3d-up	3/310	0,831826179	0,928232216	0	0	0,665114732	0,122468759	C1QA;RPS6;PDRG1
Closantel-22_mg/kg_in_CMC-Rat-Liver-5d-up	3/310	0,831826179	0,92810072	0	0	0,665114732	0,122468759	RPS6;RPL35A;SEPP1
Dexchlorpheniramine-3_mg/kg_in_Water-Rat-Heart-5d-up	3/310	0,831826179	0,927969261	0	0	0,665114732	0,122468759	EEF1B2;SEPP1;RPL39
Doxazosin-500_mg/kg_in_Water-Rat-Heart-3d-up	3/310	0,831826179	0,927837839	0	0	0,665114732	0,122468759	C1QA;RPL10A;SEPP1
Indomethacin-5_mg/kg_in_Corn_Oil-Rat-Liver-3d-dn	3/310	0,831826179	0,927706455	0	0	0,665114732	0,122468759	RPL35A;RPS3A;TPT1
Lamotrigine-26_mg/kg_in_CMC-Rat-Kidney-3d-up	3/310	0,831826179	0,927575107	0	0	0,665114732	0,122468759	RPS17;RPS3A;TPT1
Sildenafil-14.6_mg/kg_in_Saline-Rat-Liver-0.25d-dn	3/310	0,831826179	0,927443797	0	0	0,665114732	0,122468759	RPS19;RPS8;RPS20
Cycloheximide-0.25_mg/kg_in_Water-Rat-Liver-5d-dn	2/220	0,832598096	0,92817305	0	0	0,624804749	0,114466874	SEPP1;TPT1
1-Naphthyl_Isothiocyanate-30_mg/kg_in_Corn_Oil-Rat-Liver-0.25d-dn	3/311	0,833443561	0,928984077	0	0	0,6629761	0,120787146	RPS20;RPSA;TPT1
Acetaminophen-100_mg/kg_in_Corn_Oil-Rat-Liver-5d-up	3/311	0,833443561	0,928852623	0	0	0,6629761	0,120787146	EEF1B2;RPS19;SEPP1
Altretamine-40_mg/kg_in_Corn_Oil-Rat-Liver-3d-up	3/311	0,833443561	0,928721207	0	0	0,6629761	0,120787146	RPS3A;RPL39;TPT1
BW-723C86-50_mg/kg_in_Saline-Rat-Heart-1d-dn	3/311	0,833443561	0,928589827	0	0	0,6629761	0,120787146	RPS17;RPL32;SEPP1
Carboplatin-14_mg/kg_in_Saline-Rat-Liver-1d-up	3/311	0,833443561	0,928458485	0	0	0,6629761	0,120787146	C1QA;SEPP1;RPL39
Nateglinide-625_mg/kg_in_CMC-Rat-Kidney-3d-up	3/311	0,833443561	0,92832718	0	0	0,6629761	0,120787146	RPS17;RPS3A;TPT1
Nitrendipine-1100_mg/kg_in_Corn_Oil-Rat-Heart-3d-dn	3/311	0,833443561	0,928195912	0	0	0,6629761	0,120787146	RPS8;COL5A3;ARL6IP5
Zomepirac-2.8_mg/kg_in_Saline-Rat-Kidney-1d-up	3/311	0,833443561	0,928064681	0	0	0,6629761	0,120787146	RPS19;SEPP1;GOLGA7
Rofecoxib-775_mg/kg_in_Corn_Oil-Rat-Heart-3d-dn	2/221	0,834479594	0,929086978	0	0	0,621977578	0,11254497	RPS17;SEPP1
Clotrimazole-89_mg/kg_in_Corn_Oil-Rat-Liver-3d-dn	3/312	0,835047601	0,929587973	0	0	0,660851176	0,119129361	EEF1B2;RPL35A;SEPP1
Loratadine-2000_mg/kg_in_Water-Rat-Heart-5d-dn	3/312	0,835047601	0,929456601	0	0	0,660851176	0,119129361	COL5A3;ARL6IP5;SEPP1
Nimesulide-30_mg/kg_in_Corn_Oil-Rat-Liver-1d-dn	3/312	0,835047601	0,929325266	0	0	0,660851176	0,119129361	C1QA;SEPP1;TPT1
Procarbazine-54_mg/kg_in_Water-Rat-Liver-0.25d-up	3/312	0,835047601	0,929193968	0	0	0,660851176	0,119129361	C1QA;RPS6;SEPP1
Diethanolamine-620_mg/kg_in_Water-Rat-Kidney-1d-up	4/399	0,835339709	0,929387702	0	0	0,689007743	0,123964068	EEF1B2;RPS3A;SEPP1;TPT1
Benzocaine-1100_mg/kg_in_Corn_Oil-Rat-Liver-1d-up	3/313	0,836638374	0,930701106	0	0	0,658739831	0,117495044	RPS20;RPSA;SEPP1
Catechol-40_mg/kg_in_Saline-Rat-Liver-3d-dn	3/313	0,836638374	0,93056967	0	0	0,658739831	0,117495044	RPS8;SEPP1;RPL39
Chlorambucil-4.5_mg/kg_in_Corn_Oil-Rat-Liver-1d-dn	3/313	0,836638374	0,930438271	0	0	0,658739831	0,117495044	C1QA;RPS17;RPL10A
Ethacrynic_Acid-125_mg/kg_in_CMC-Rat-Kidney-1d-dn	3/313	0,836638374	0,930306909	0	0	0,658739831	0,117495044	RPS17;SEPP1;RPL39
Ethinylestradiol-10_mg/kg_in_Corn_Oil-Rat-Liver-1d-dn	3/313	0,836638374	0,930175584	0	0	0,658739831	0,117495044	C1QA;SEPP1;TPT1
Geraniol-1500_mg/kg_in_CMC-Rat-Liver-1d-dn	3/313	0,836638374	0,930044296	0	0	0,658739831	0,117495044	C1QA;CLP1;ARL6IP5
Oxytetracycline-1500_mg/kg_in_Water-Rat-Liver-5d-up	3/313	0,836638374	0,929913045	0	0	0,658739831	0,117495044	C1QA;ARL2BP;TPT1
Prazosin-2000_mg/kg_in_CMC-Rat-Heart-5d-dn	3/313	0,836638374	0,929781831	0	0	0,658739831	0,117495044	EEF1B2;ARL6IP5;TPT1
Saquinavir-1500_mg/kg_in_CMC-Rat-Kidney-3d-dn	3/313	0,836638374	0,929650654	0	0	0,658739831	0,117495044	RPSA;SEPP1;TPT1
Levofloxacin-739_mg/kg_in_Water-Rat-Kidney-3d-up	4/401	0,83817227	0,931223699	0	0	0,685571295	0,121025016	CLP1;RPS8;RAPGEF5;SEPP1
Allyl_Alcohol-16_mg/kg_in_Saline-Rat-Bone_marrow-0.25d-up	3/314	0,838215956	0,931140884	0	0	0,656641933	0,115883845	RPS19;RPS6;NKG7
Clemastine-2500_mg/kg_in_CMC-Rat-Heart-3d-dn	3/314	0,838215956	0,931009571	0	0	0,656641933	0,115883845	RPS8;COL5A3;SEPP1
Lead_(II)_Acetate-200_mg/kg_in_Saline-Rat-Liver-0.25d-dn	3/314	0,838215956	0,930878295	0	0	0,656641933	0,115883845	SEPP1;RPL39;TPT1
Sulfisoxazole-2500_mg/kg_in_Corn_Oil-Rat-Liver-3d-dn	3/314	0,838215956	0,930747056	0	0	0,656641933	0,115883845	RPS17;RPS3A;TPT1
Aspirin-375_mg/kg_in_Corn_Oil-Rat-Liver-0.25d-dn	3/315	0,839780423	0,932352779	0	0	0,654557356	0,114295416	C1QA;RPL39;TPT1
Fluconazole-394_mg/kg_in_Corn_Oil-Rat-Liver-0.25d-dn	3/315	0,839780423	0,932221369	0	0	0,654557356	0,114295416	C1QA;CLP1;SEPP1
Rapamycin-60_mg/kg_in_CMC-Rat-Heart-3d-dn	3/315	0,839780423	0,932089996	0	0	0,654557356	0,114295416	ARL6IP5;SEPP1;RPL39
Tosufloxacin-2000_mg/kg_in_CMC-Rat-Liver-5d-dn	3/315	0,839780423	0,93195866	0	0	0,654557356	0,114295416	EEF1B2;RPL32;RPS3A
Ifosfamide-17_mg/kg_in_Saline-Rat-Liver-3d-dn	2/224	0,840010319	0,932082456	0	0	0,613647521	0,106983985	RPL39;SEPP1
Carboplatin-5_mg/kg_in_Saline-Rat-Kidney-28d-dn	3/316	0,841331852	0,933417335	0	0	0,652485972	0,112729417	C1QA;RPS6;RAPGEF5
Cisplatin-2_mg/kg_in_Saline-Rat-Liver-0.25d-dn	3/316	0,841331852	0,933285868	0	0	0,652485972	0,112729417	EEF1B2;RPS19;SEPP1
Cyclopropane_Carboxylic_Acid-375_mg/kg_in_Water-Rat-Liver-5d-dn	3/316	0,841331852	0,933154438	0	0	0,652485972	0,112729417	C1QA;RPS17;SEPP1
Isoprenaline-4.2_mg/kg_in_Saline-Rat-Heart-1d-dn	3/316	0,841331852	0,933023045	0	0	0,652485972	0,112729417	FUNDC1;SEPP1;TPT1
Theophylline-225_mg/kg_in_Water-Rat-Heart-1d-dn	3/316	0,841331852	0,932891688	0	0	0,652485972	0,112729417	RPL32;RPL10A;SEPP1
Zidovudine-1540_mg/kg_in_Corn_Oil-Rat-Liver-1d-dn	3/316	0,841331852	0,932760369	0	0	0,652485972	0,112729417	EEF1B2;C1QA;RPSA
Promazine-100_mg/kg_in_Corn_Oil-Rat-Liver-0.25d-dn	2/225	0,841816483	0,933166309	0	0	0,610920199	0,105196329	CLP1;SEPP1
Catechol-195_mg/kg_in_Saline-Rat-Liver-3d-dn	3/317	0,842870318	0,934203015	0	0	0,650427656	0,111185513	RPS8;RPL10A;TPT1
Isotretinoin-13_mg/kg_in_Corn_Oil-Rat-Liver-5d-dn	3/317	0,842870318	0,934071567	0	0	0,650427656	0,111185513	RPS6;RPSA;SEPP1

Nifedipine-1022_mg/kg_in_CMC-Rat-Heart-3d-dn	3/317	0,842870318	0,933940155	0	0	0,650427656	0,111185513	ARL2BP;RPS8;ARL6IP5
Vinblastine-45_uM_in_DMSO-Rat-Primary_rat_hepatocytes-1d-dn	3/318	0,844395899	0,935498959	0	0	0,648382286	0,109663373	RPS20;SEPP1;RPL39
Kanamycin-225_mg/kg_in_Saline-Rat-Kidney-1d-dn	2/227	0,845373612	0,936450431	0	0	0,605537642	0,101716158	RPS19;CLP1
Alprazolam-115_mg/kg_in_Corn_Oil-Rat-Liver-5d-up	3/319	0,84590867	0,936911361	0	0	0,64634974	0,108162673	C1QA;RPS17;SEPP1
Bisphenol_A-100_mg/kg_in_Corn_Oil-Rat-Liver-0.25d-dn	3/319	0,84590867	0,936779624	0	0	0,64634974	0,108162673	RPS6;RPS20;RPL39
Ethinylestradiol-1480_mg/kg_in_Corn_Oil-Rat-Liver-0.25d-dn	3/319	0,84590867	0,936647925	0	0	0,64634974	0,108162673	C1QA;RPL32;SEPP1
Mifepristone-3_mg/kg_in_Corn_Oil-Rat-Liver-3d-dn	3/319	0,84590867	0,936516262	0	0	0,64634974	0,108162673	C1QA;RPS6;RPL10A
Progesterone-164_mg/kg_in_Corn_Oil-Rat-Liver-1d-dn	3/319	0,84590867	0,936384636	0	0	0,64634974	0,108162673	RPS17;SEPP1;RPL39
Promethazine-2.3_mg/kg_in_Saline-Rat-Liver-0.25d-dn	3/319	0,84590867	0,936253048	0	0	0,64634974	0,108162673	RPS19;RPS20;RPL39
Azithromycin-225_mg/kg_in_Water-Rat-Kidney-1d-up	3/320	0,84740871	0,937781509	0	0	0,644329897	0,106683095	ARL2BP;RPL10A;TPT1
Benzothiazyl_Disulfide-2600_mg/kg_in_Corn_Oil-Rat-Kidney-5d-up	3/320	0,84740871	0,937649761	0	0	0,644329897	0,106683095	RPS5;SEPP1;TPT1
Carbon_Tetrachloride-400_mg/kg_in_Corn_Oil-Rat-Liver-0.25d-dn	3/320	0,84740871	0,93751805	0	0	0,644329897	0,106683095	RPL32;RPS8;TPT1
Ethylene_Glycol-352_mg/kg_in_Water-Rat-Liver-1d-up	3/320	0,84740871	0,937386376	0	0	0,644329897	0,106683095	EEF1B2;C1QA;RPS17
Nystatin-134_mg/kg_in_Corn_Oil-Rat-Liver-1d-dn	3/320	0,84740871	0,937254739	0	0	0,644329897	0,106683095	RPS6;RPS3A;RPL10A
Oxybutynin-230_mg/kg_in_Water-Rat-Heart-5d-dn	3/320	0,84740871	0,937123139	0	0	0,644329897	0,106683095	ARL2BP;RPS8;RPS20
Phenacetin-619_mg/kg_in_Corn_Oil-Rat-Liver-5d-dn	3/320	0,84740871	0,936991576	0	0	0,644329897	0,106683095	EEF1B2;C1QA;RPL39
Ketoprofen-4_mg/kg_in_Corn_Oil-Rat-Kidney-3d-dn	2/229	0,848858152	0,938462494	0	0	0,600249103	0,098358729	RPL39;SEPP1
Lead(IV)_Acetate-600_mg/kg_in_Saline-Rat-Kidney-1d-dn	2/229	0,848858152	0,93833078	0	0	0,600249103	0,098358729	ARL2BP;SEPP1
Bromfenac-5_mg/kg_in_CMC-Rat-Kidney-1d-dn	3/321	0,848896093	0,938241037	0	0	0,642322639	0,105224323	ARL2BP;RPS6;RPS20
Levofloxacin-739_mg/kg_in_Water-Rat-Heart-1d-dn	3/321	0,848896093	0,938109391	0	0	0,642322639	0,105224323	RPSA;RPL10A;SEPP1
Mitomycin_C-1.7_mg/kg_in_Saline-Rat-Liver-0.25d-dn	3/321	0,848896093	0,937977782	0	0	0,642322639	0,105224323	EEF1B2;RPS8;RPSA
Sulindac-23_mg/kg_in_Corn_Oil-Rat-Liver-5d-dn	3/321	0,848896093	0,93784621	0	0	0,642322639	0,105224323	RPSA;RPL10A;SEPP1
Isoeugenol-1560_mg/kg_in_CMC-Rat-Liver-1d-dn	3/322	0,850370898	0,939343786	0	0	0,640327848	0,10378605	C1QA;CLP1;SEPP1
Meropenem-1500_mg/kg_in_CMC-Rat-Spleen-3d-dn	3/322	0,850370898	0,939212059	0	0	0,640327848	0,10378605	EEF1B2;NKG7;RPS20
Naproxen-134_mg/kg_in_Corn_Oil-Rat-Kidney-3d-dn	3/322	0,850370898	0,939080369	0	0	0,640327848	0,10378605	RPS19;RPSA;SEPP1
Roxithromycin-312_mg/kg_in_CMC-Rat-Kidney-3d-dn	3/322	0,850370898	0,938948716	0	0	0,640327848	0,10378605	RPL32;RPSA;SEPP1
Clarithromycin-56_mg/kg_in_Water-Rat-Liver-0.25d-dn	2/230	0,85057358	0,939040862	0	0	0,597639325	0,096724553	RPS19;CLP1
Ethinylestradiol-1480_mg/kg_in_Corn_Oil-Rat-Liver-5d-dn	3/323	0,851833201	0,94029969	0	0	0,638345409	0,102367971	EEF1B2;C1QA;SEPP1
Zomepirac-2.8_mg/kg_in_Saline-Rat-Liver-3d-dn	3/323	0,851833201	0,940167921	0	0	0,638345409	0,102367971	RPL10A;SEPP1;RPL39
Beta-Estradiol-150_mg/kg_in_Corn_Oil-Rat-Liver-0.25d-dn	2/231	0,852271312	0,940519666	0	0	0,595052141	0,0951193	RPS8;TPT1
Benoxaprofen-89_mg/kg_in_CMC-Rat-Liver-1d-dn	3/324	0,853283078	0,941504276	0	0	0,636375207	0,100969788	C1QA;RPL39;TPT1
Doxorubicin-0.65_mg/kg_in_Saline-Rat-Bone_marrow-1d-up	3/324	0,853283078	0,941372394	0	0	0,636375207	0,100969788	RPS19;RPS6;RPS20
Hydrazine-45_mg/kg_in_Water-Rat-Kidney-3d-up	3/324	0,853283078	0,941240549	0	0	0,636375207	0,100969788	RPS8;SEPP1;TPT1
Lansoprazole-240_uM_in_DMSO-Rat-Primary_rat_hepatocytes-1d-up	2/232	0,853951499	0,941845961	0	0	0,592487262	0,09354241	EEF1B2;PDRG1
Ethanol-6000_mg/kg_in_Saline-Rat-Liver-0.25d-dn	3/325	0,854720607	0,942562237	0	0	0,634417129	0,099591206	C1QA;RPS19;SEPP1
Lamotrigine-26_mg/kg_in_CMC-Rat-Kidney-5d-up	3/325	0,854720607	0,942430281	0	0	0,634417129	0,099591206	EEF1B2;RPS17;RAPGEF5
Pramoxine-526_mg/kg_in_Corn_Oil-Rat-Liver-3d-dn	3/325	0,854720607	0,942298362	0	0	0,634417129	0,099591206	RPS19;RPS6;RPL39
Rosiglitazone-1800_mg/kg_in_Corn_Oil-Rat-Liver-0.25d-up	3/325	0,854720607	0,94216648	0	0	0,634417129	0,099591206	RPS17;RPS19;RPS8
Sotalol-2000_mg/kg_in_Water-Rat-Liver-3d-up	3/325	0,854720607	0,942034635	0	0	0,634417129	0,099591206	RPS8;RAPGEF5;SEPP1
Cerivastatin-7_mg/kg_in_Corn_Oil-Rat-Skeletal_muscle-5d-dn	3/326	0,856145864	0,943473461	0	0	0,632471064	0,098231937	COL5A3;SEPP1;SLC16A3
Sulindac-132_mg/kg_in_Corn_Oil-Rat-Liver-3d-dn	3/326	0,856145864	0,94334147	0	0	0,632471064	0,098231937	RPL32;RPL35A;SEPP1
Hydroxyurea-400_mg/kg_in_Saline-Rat-Liver-0.25d-dn	2/234	0,857259832	0,944436766	0	0	0,587423268	0,090471535	RPS19;RPS20
1-Naphthyl_Isothiocyanate-60_mg/kg_in_Corn_Oil-Rat-Liver-1d-dn	3/327	0,857558927	0,944634141	0	0	0,630536902	0,096891695	RPL35A;SEPP1;TPT1
6-Mercaptopurine-25_mg/kg_in_Corn_Oil-Rat-Liver-5d-up	3/327	0,857558927	0,944502042	0	0	0,630536902	0,096891695	RPS19;RPS20;GOLGA7
Atropine-2.3_mg/kg_in_Water-Rat-Liver-5d-up	3/327	0,857558927	0,944369981	0	0	0,630536902	0,096891695	RPSA;RPL39;TPT1
Etoposide-188_mg/kg_in_Corn_Oil-Rat-Kidney-1d-up	3/327	0,857558927	0,944237957	0	0	0,630536902	0,096891695	ARL2BP;SEPP1;TPT1
Lead_(II)_Acetate-600_mg/kg_in_Saline-Rat-Liver-3d-dn	3/327	0,857558927	0,944105969	0	0	0,630536902	0,096891695	RPS19;SEPP1;RPL39
Methotrexate-27_mg/kg_in_Corn_Oil-Rat-Kidney-3d-dn	3/327	0,857558927	0,943974019	0	0	0,630536902	0,096891695	EEF1B2;RPS19;SEPP1
Tramadol-114_mg/kg_in_Water-Rat-Brain-3d-dn	3/327	0,857558927	0,943842105	0	0	0,630536902	0,096891695	ARL2BP;RPS6;SEPP1
Aspirin-375_mg/kg_in_Corn_Oil-Rat-Kidney-1d-dn	2/235	0,858888274	0,945173124	0	0	0,584923594	0,088976489	CLP1;SEPP1
Beta-Estradiol_3-Benzoate-25_mg/kg_in_Corn_Oil-Rat-Liver-3d-dn	3/328	0,858959871	0,945119858	0	0	0,628614534	0,0955702	C1QA;RPL35A;SEPP1
Zomepirac-2.8_mg/kg_in_Saline-Rat-Liver-1d-up	3/328	0,858959871	0,94498784	0	0	0,628614534	0,0955702	C1QA;RPS17;RPSA
Chlorambucil-4.5_mg/kg_in_Corn_Oil-Rat-Liver-5d-dn	3/329	0,860348775	0,946383652	0	0	0,626703851	0,094267176	C1QA;SEPP1;TPT1
Diclofenac-3.5_mg/kg_in_Corn_Oil-Rat-Liver-3d-up	3/330	0,861725714	0,947765916	0	0	0,624804749	0,092982353	RPS19;RPL39;GOLGA7
Etoposide-100_mg/kg_in_Corn_Oil-Rat-Liver-1d-up	3/330	0,861725714	0,947633583	0	0	0,624804749	0,092982353	RPS19;RPSA;SEPP1
Tamoxifen-2.5_mg/kg_in_Corn_Oil-Rat-Liver-3d-up	3/330	0,861725714	0,947501288	0	0	0,624804749	0,092982353	RPS19;SEPP1;GOLGA7
Acyclovir-980_mg/kg_in_Saline-Rat-Bone_marrow-3d-up	3/331	0,863090766	0,948869747	0	0	0,622917121	0,091715463	NKG7;RPL35A;RPS3A
Azathioprine-20_mg/kg_in_Corn_Oil-Rat-Liver-5d-up	3/331	0,863090766	0,948737316	0	0	0,622917121	0,091715463	CLP1;RPL39;TPT1
Mitomycin_C-0.5_mg/kg_in_Saline-Rat-Bone_marrow-3d-up	3/331	0,863090766	0,948604922	0	0	0,622917121	0,091715463	RPS5;RPL10A;SEPP1
Sildenafil-14.6_mg/kg_in_Saline-Rat-Liver-5d-up	3/331	0,863090766	0,948472565	0	0	0,622917121	0,091715463	CLP1;RPS20;SEPP1
Acetaminophen-100_mg/kg_in_Corn_Oil-Rat-Kidney-1d-dn	2/238	0,863672465	0,948979399	0	0	0,577550608	0,084646784	ARL2BP;TPT1
Lead_(II)_Acetate-200_mg/kg_in_Saline-Rat-Liver-3d-dn	3/332	0,864444006	0,949694657	0	0	0,621040864	0,090466244	RPS8;RPS3A;SEPP1
Gentian_Violet-18_mg/kg_in_Corn_Oil-Rat-Liver-1d-dn	3/333	0,865785513	0,951035802	0	0	0,619175877	0,089234436	RPS19;RPL10A;RPL39
Amiloride-22_mg/kg_in_CMC-Rat-Heart-1d-up	2/240	0,866779101	0,95199445	0	0	0,572737686	0,081884948	FUNDC1;TPT1
Fenofibrate-215_mg/kg_in_Corn_Oil-Rat-Kidney-1d-dn	2/241	0,868308006	0,953540694	0	0	0,570361181	0,080540008	RPL32;SEPP1
S(-)-Carbidopa-1200_mg/kg_in_CMC-Rat-Brain-1d-up	2/241	0,868308006	0,953407759	0	0	0,570361181	0,080540008	C1QA;RPL10A
Sulbactam-671_mg/kg_in_Saline-Rat-Kidney-1d-dn	2/242	0,869820826	0,954935715	0	0	0,568004317	0,079218447	ARL2BP;RPS8
Papaverine-69_mg/kg_in_CMC-Rat-Heart-3d-up	2/243	0,871317703	0,956445746	0	0	0,565666851	0,077919824	FUNDC1;SEPP1
Ascorbic_Acid-2000_mg/kg_in_Water-Rat-Kidney-5d-up	3/339	0,873592412	0,958809064	0	0	0,608217012	0,082195274	RPS17;RAPGEF5;SEPP1
Clofibrate-130_mg/kg_in_Corn_Oil-Rat-Liver-0.25d-dn	3/339	0,873592412	0,958675469	0	0	0,608217012	0,082195274	CLP1;RPL39;TPT1
Furan-24_mg/kg_in_Corn_Oil-Rat-Kidney-3d-dn	3/339	0,873592412	0,958541911	0	0	0,608217012	0,082195274	CLP1;SEPP1;RPL39
Camptothecin-5_mg/kg_in_Saline-Rat-Kidney-3d-up	2/245	0,874264194	0,959145396	0	0	0,561049162	0,075389672	CLP1;SEPP1
Gatifloxacin-770_mg/kg_in_Corn_Oil-Rat-Kidney-5d-dn	2/245	0,874264194	0,95901181	0	0	0,561049162	0,075389672	CLP1;SEPP1
Tiapride-1200_mg/kg_in_Water-Rat-Heart-3d-dn	2/245	0,874264194	0,958878261	0	0	0,561049162	0,075389672	RPS20;RPSA
Amitriptyline-160_mg/kg_in_Water-Rat-Kidney-5d-up	3/340	0,874853917	0,959391458	0	0	0,606428138	0,081078447	EEF1B2;CLP1;RPL10A
Cadmium_Chloride-8_uM_in_DMSO-Rat-Primary_rat_hepatocytes-1d-up	2/246	0,87571409	0,960201055	0	0	0,558768474	0,074157306	RPS20;SLC16A3
Fluvastatin-5_mg/kg_in_Corn_Oil-Rat-Kidney-3d-up	2/246	0,87571409	0,960067396	0	0	0,558768474	0,074157306	ARL2BP;RPS20
Nimesulide-162_mg/kg_in_Corn_Oil-Rat-Liver-3d-dn	3/341	0,876104297	0,960361509	0	0	0,604649757	0,079977105	RPL35A;SEPP1;TPT1
Ethylene_Glycol-352_mg/kg_in_Water-Rat-Liver-3d-up	3/344	0,879789444	0,964266861	0	0	0,599376648	0,076763766	CLP1;RPSA;RPL39
Cytochalasin_B-167_uM_in_DMSO-Rat-Primary_rat_hepatocytes-1d-up	2/249	0,879972048	0,964332803	0	0	0,552036324	0,070586199	RPS19;RPS20
Raloxifene-650_mg/kg_in_Corn_Oil-Rat-Liver-3d-dn	2/249	0,879972048	0,964198644	0	0	0,552036324	0,070586199	GOLGA7;SEPP1
Tranilast-1100_mg/kg_in_CMC-Rat-Heart-1d-dn	2/249	0,879972048	0,964064522	0	0	0,552036324	0,070586199	RPS19;COL5A3
Sulindac-23_mg/kg_in_Corn_Oil-Rat-Liver-1d-up	2/250	0,881361251	0,965452185	0	0	0,549828179	0,069436531	RPS20;RPL39
Caffeine-93_mg/kg_in_Water-Rat-Brain-3d-dn	3/346	0,882191969	0,966227777	0	0	0,595912043	0,07469495	RPS20;SEPP1;DUSP14
Diethylstilbestrol-2.8_mg/kg_in_Corn_Oil-Rat-Spleen-0.25d-dn	2/251	0,882735622	0,966688788	0	0	0,547637628	0,068306585	RPS5;RPS6
Fenofibrate-215_mg/kg_in_Corn_Oil-Rat-Kidney-5d-dn	2/251	0,882735622	0,966554395	0	0	0,547637628	0,068306585	CLP1;SEPP1
6-Methoxy-2-Naphthylacetic_Acid-360_mg/kg_in_Saline-Rat-Kidney-1d-dn	2/252	0,884095299	0,967908614	0	0	0,545464463	0,067195995	R3HCC1;RPL39
44'-Methylenedianiline-81_mg/kg_in_Corn_Oil-Rat-Liver-1d-dn	2/253	0,885440415	0,96924652	0	0	0,543308477	0,066104404	EEF1B2;SEPP1
Camptothecin-5_mg/kg_in_Saline-Rat-Kidney-1d-up	2/253	0,885440415	0,969111827	0	0	0,543308477	0,066104404	RPS8;SEPP1
Cyclosporin_A-70_mg/kg_in_Corn_Oil-Rat-Spleen-0.25d-dn	2/253	0,885440415	0,968977172	0	0	0,543308477	0,066104404	RPS19;RPS5

Ubenimex-1300_mg/kg_in_Water-Rat-Kidney-5d-dn	2/253	0,885440415	0,968842555	0	0	0,543308477	0,066104404	CLP1;SEPP1
Podophyllotoxin-4_mg/kg_in_Corn_Oil-Rat-Bone_marrow-3d-dn	2/254	0,886771105	0,970163803	0	0	0,541169467	0,065031461	RPS17;RPS19
Mercuric_Chloride-2.5_mg/kg_in_Water-Rat-Kidney-5d-up	3/350	0,886869538	0,970136733	0	0	0,58910162	0,070726003	CLP1;RAPGEF5;TOMM20
Caffeine-93_mg/kg_in_Water-Rat-Heart-3d-up	2/255	0,888087501	0,971334143	0	0	0,539047234	0,063976823	RAPGEF5;GOLGA7
Gemfibrozil-100_mg/kg_in_Corn_Oil-Rat-Kidney-3d-up	2/255	0,888087501	0,971199273	0	0	0,539047234	0,063976823	ARL2BP;RPSA
Valproic_Acid-850_mg/kg_in_Water-Rat-Liver-3d-dn	2/255	0,888087501	0,97106444	0	0	0,539047234	0,063976823	RPS8;SEPP1
Aspirin-375_mg/kg_in_Corn_Oil-Rat-Kidney-5d-dn	2/256	0,889389737	0,972353355	0	0	0,536941581	0,062940152	CLP1;SEPP1
Atorvastatin-300_mg/kg_in_Corn_Oil-Rat-Skeletal_muscle-5d-up	2/256	0,889389737	0,9722184	0	0	0,536941581	0,062940152	RPS8;RPS20
Indomethacin-12_mg/kg_in_Water-Rat-Liver-5d-dn	2/256	0,889389737	0,972083482	0	0	0,536941581	0,062940152	SEPP1;TPT1
Loratadine-2000_mg/kg_in_Water-Rat-Heart-3d-dn	2/256	0,889389737	0,971948601	0	0	0,536941581	0,062940152	COL5A3;SEPP1
Azithromycin-50_mg/kg_in_Water-Rat-Liver-0.25d-dn	2/257	0,890677944	0,973221349	0	0	0,534852314	0,061921121	RPS8;RPL35A
Citalopram-90_mg/kg_in_Corn_Oil-Rat-Heart-3d-dn	2/257	0,890677944	0,973086348	0	0	0,534852314	0,061921121	RPS17;RPL35A
Ibufenac-500_mg/kg_in_Corn_Oil-Rat-Liver-5d-up	2/257	0,890677944	0,972951385	0	0	0,534852314	0,061921121	RPS8;TPT1
Crotamiton-750_mg/kg_in_Corn_Oil-Rat-Liver-3d-up	2/258	0,891952252	0,974208284	0	0	0,532779243	0,060919405	RPSA;SEPP1
Ethanol-6000_mg/kg_in_Saline-Rat-Kidney-1d-dn	2/258	0,891952252	0,974073202	0	0	0,532779243	0,060919405	RPL10A;SEPP1
Imatinib-150_mg/kg_in_Water-Rat-Liver-3d-up	2/258	0,891952252	0,973938158	0	0	0,532779243	0,060919405	RPS8;TPT1
Acyclovir-980_mg/kg_in_Saline-Rat-Bone_marrow-1d-up	3/355	0,892483599	0,974383258	0	0	0,580804414	0,066064842	RPS6;GOLGA7;TPT1
Beta-Naphthoflavone-80_mg/kg_in_CMC-Rat-Liver-28d-up	2/259	0,893212791	0,975044205	0	0	0,53072218	0,059934689	CLP1;SEPP1
Doxazosin-5_mg/kg_in_Water-Rat-Heart-1d-up	2/259	0,893212791	0,974909083	0	0	0,53072218	0,059934689	ARL6IP5;SEPP1
Gentian_Violet-18_mg/kg_in_Corn_Oil-Rat-Liver-3d-up	2/259	0,893212791	0,974773998	0	0	0,53072218	0,059934689	GOLGA7;SEPP1
Netilmicin-40_mg/kg_in_Saline-Rat-Kidney-14d-dn	2/259	0,893212791	0,97463895	0	0	0,53072218	0,059934689	CLP1;SEPP1
Diisopropyl_Fluorophosphate-3.75_mg/kg_in_Water-Rat-Liver-3d-up	3/356	0,893576094	0,974900307	0	0	0,579172941	0,065170731	RPS8;RPSA;TPT1
Interleukin_1_Beta-0.01_mg/kg_in_RSA/Saline-Rat-Liver-0.25d-dn	2/260	0,894459691	0,975729159	0	0	0,528680941	0,058966662	CLP1;SEPP1
Meloxicam-6_mg/kg_in_Corn_Oil-Rat-Liver-0.25d-dn	2/261	0,895693079	0,976939301	0	0	0,526655344	0,05801502	RPS3A;TPT1
Pramoxine-526_mg/kg_in_Corn_Oil-Rat-Heart-1d-up	2/261	0,895693079	0,976804028	0	0	0,526655344	0,05801502	RPS19;KCNJ12
Enoxacin-750_mg/kg_in_Corn_Oil-Rat-Liver-5d-dn	3/359	0,896794258	0,977869525	0	0	0,574333056	0,062561416	RPSA;RPL10A;SEPP1
6-Mercaptopurine-25_mg/kg_in_Corn_Oil-Rat-Kidney-1d-up	2/262	0,896913084	0,977863711	0	0	0,524645209	0,057079467	RPS17;TPT1
Penicillin_G-243_mg/kg_in_Saline-Rat-Kidney-1d-dn	2/262	0,896913084	0,977728367	0	0	0,524645209	0,057079467	ARL2BP;RPS3A
4-Methylpyrazole-155_mg/kg_in_Saline-Rat-Kidney-3d-up	2/263	0,898119832	0,978908358	0	0	0,52265036	0,05615971	RPS19;CLP1
Allopurinol-175_mg/kg_in_Corn_Oil-Rat-Kidney-1d-dn	2/263	0,898119832	0,978772906	0	0	0,52265036	0,05615971	CLP1;SEPP1
Chlorpropamide-269_mg/kg_in_CMC-Rat-Heart-1d-up	2/263	0,898119832	0,978637492	0	0	0,52265036	0,05615971	FUNDC1;SEPP1
Cisplatin-2_mg/kg_in_Saline-Rat-Kidney-3d-dn	2/263	0,898119832	0,978502116	0	0	0,52265036	0,05615971	EEF1B2;SEPP1
Digoxin-11_mg/kg_in_Corn_Oil-Rat-Liver-5d-up	2/263	0,898119832	0,978366776	0	0	0,52265036	0,05615971	RPL39;TPT1
Meloxicam-33_mg/kg_in_Corn_Oil-Rat-Kidney-1d-dn	2/263	0,898119832	0,978231475	0	0	0,52265036	0,05615971	CLP1;SEPP1
Monocrotaline-333_uM_in_DMSO-Rat-Primary_rat_hepatocytes-0.67d-dn	2/263	0,898119832	0,97809621	0	0	0,52265036	0,05615971	SEPP1;SLC16A3
Penicillamine-852_mg/kg_in_Water-Rat-Liver-0.25d-dn	2/263	0,898119832	0,977960984	0	0	0,52265036	0,05615971	RPS17;RPSA
Bisphenol_A-610_mg/kg_in_Corn_Oil-Rat-Kidney-5d-dn	2/264	0,899313448	0,97912534	0	0	0,520670624	0,055255463	RPS19;RPL10A
Modafinil-325_mg/kg_in_Saline-Rat-Liver-1d-dn	2/264	0,899313448	0,978990009	0	0	0,520670624	0,055255463	RPS8;TPT1
Pralidoxime_Chloride-48_mg/kg_in_Saline-Rat-Liver-3d-up	2/264	0,899313448	0,978854714	0	0	0,520670624	0,055255463	C1QA;RPS8
Amiodarone-147_mg/kg_in_CMC-Rat-Heart-1d-up	2/265	0,900494057	0,98000431	0	0	0,518705829	0,054366447	FUNDC1;SEPP1
44'-Methylenedianiline-81_mg/kg_in_Corn_Oil-Rat-Liver-5d-dn	2/266	0,901661784	0,981139571	0	0	0,516755807	0,053492386	EEF1B2;SEPP1
Cyproterone_Acetate-2500_mg/kg_in_CMC-Rat-Liver-5d-up	2/266	0,901661784	0,981004035	0	0	0,516755807	0,053492386	RPL39;TPT1
Sulindac-132_mg/kg_in_Corn_Oil-Rat-Heart-3d-dn	2/266	0,901661784	0,980868538	0	0	0,516755807	0,053492386	FUNDC1;SEPP1
44'-Methylenedianiline-81_mg/kg_in_Corn_Oil-Rat-Liver-3d-dn	2/267	0,902816752	0,98198933	0	0	0,514820392	0,052633012	EEF1B2;RPL35A
Hydrazine-45_mg/kg_in_Water-Rat-Liver-3d-dn	2/267	0,902816752	0,981853734	0	0	0,514820392	0,052633012	EEF1B2;CLP1
Lovastatin-450_mg/kg_in_Corn_Oil-Rat-Kidney-0.25d-dn	2/267	0,902816752	0,981718175	0	0	0,514820392	0,052633012	CLP1;RPS8
Pentobarbital-20_mg/kg_in_Water-Rat-Liver-1d-dn	2/267	0,902816752	0,981582653	0	0	0,514820392	0,052633012	RPS20;TPT1
Anisindione-75_mg/kg_in_CMC-Rat-Liver-1d-dn	2/268	0,903959083	0,982688991	0	0	0,51289942	0,05178806	RPSA;SEPP1
Isoprenaline-15_mg/kg_in_Saline-Rat-Liver-3d-dn	2/268	0,903959083	0,982553373	0	0	0,51289942	0,05178806	CLP1;RPL39
Methimazole-28_mg/kg_in_Water-Rat-Liver-0.25d-dn	2/268	0,903959083	0,982417792	0	0	0,51289942	0,05178806	RPL39;TPT1
2-Amino-4-Nitrophenol-625_mg/kg_in_CMC-Rat-Heart-3d-up	2/269	0,905088899	0,983509957	0	0	0,510992731	0,050957273	EEF1B2;RPS8
Dicyclomine-645_mg/kg_in_Water-Rat-Heart-3d-up	2/269	0,905088899	0,983374282	0	0	0,510992731	0,050957273	FUNDC1;SEPP1
Levamisole-120_mg/kg_in_Water-Rat-Liver-3d-dn	2/269	0,905088899	0,983238644	0	0	0,510992731	0,050957273	EEF1B2;CLP1
NN-Dimethylformamide-140_mg/kg_in_Saline-Rat-Bone_marrow-1d-dn	3/368	0,90593166	0,984018446	0	0	0,560286867	0,055351528	RPSA;RPL10A;SEPP1
Cisplatin-0.5_mg/kg_in_Saline-Rat-Kidney-28d-dn	2/270	0,906206321	0,984181051	0	0	0,509100165	0,050140395	CLP1;SEPP1
Oxymetazoline-0.5_mg/kg_in_Water-Rat-Heart-3d-up	2/270	0,906206321	0,984045358	0	0	0,509100165	0,050140395	RPL10A;RPL39
Procarbazine-54_mg/kg_in_Water-Rat-Liver-3d-up	2/270	0,906206321	0,983909702	0	0	0,509100165	0,050140395	EEF1B2;RPS19
Allopurinol-175_mg/kg_in_Corn_Oil-Rat-Kidney-3d-dn	2/271	0,907311468	0,984973828	0	0	0,507221567	0,04933718	CLP1;SEPP1
Idarubicin-4_mg/kg_in_Saline-Rat-Heart-5d-up	2/271	0,907311468	0,984838081	0	0	0,507221567	0,04933718	RPSA;SEPP1
Lomustine-8.75_mg/kg_in_Corn_Oil-Rat-Liver-5d-dn	2/271	0,907311468	0,984702373	0	0	0,507221567	0,04933718	RPS6;RPL35A
Neomycin-877_mg/kg_in_Corn_Oil-Rat-Kidney-5d-dn	2/271	0,907311468	0,984566701	0	0	0,507221567	0,04933718	CLP1;SEPP1
Rofecoxib-250_mg/kg_in_Corn_Oil-Rat-Liver-1d-up	2/271	0,907311468	0,984431067	0	0	0,507221567	0,04933718	RPS8;SEPP1
Zirconium(IV)_Chloride-633_mg/kg_in_Corn_Oil-Rat-Liver-3d-up	2/271	0,907311468	0,984295471	0	0	0,507221567	0,04933718	RPS19;RPS6
Cadmium_Chloride-66_mg/kg_in_Corn_Oil-Rat-Kidney-1d-dn	2/272	0,908404459	0,985345478	0	0	0,505356782	0,048547382	ARL2BP;RPL39
Chlorambucil-0.6_mg/kg_in_Corn_Oil-Rat-Liver-5d-up	2/272	0,908404459	0,985209793	0	0	0,505356782	0,048547382	RPL39;SEPP1
Enoxacin-750_mg/kg_in_Corn_Oil-Rat-Liver-3d-up	2/272	0,908404459	0,985074145	0	0	0,505356782	0,048547382	RPS20;SEPP1
Glimepiride-2500_mg/kg_in_CMC-Rat-Liver-0.25d-dn	2/272	0,908404459	0,984938535	0	0	0,505356782	0,048547382	CLP1;SEPP1
Lead(IV)_Acetate-600_mg/kg_in_Saline-Rat-Bone_marrow-3d-dn	2/272	0,908404459	0,984802962	0	0	0,505356782	0,048547382	NKG7;SEPP1
Omeprazole-415_mg/kg_in_Corn_Oil-Rat-Liver-1d-dn	2/272	0,908404459	0,984667426	0	0	0,505356782	0,048547382	C1QA;RPS6
Theophylline-225_mg/kg_in_Water-Rat-Heart-5d-up	2/272	0,908404459	0,984531928	0	0	0,505356782	0,048547382	SEPP1;TPT1
Troglitazone-1200_mg/kg_in_Corn_Oil-Rat-Liver-5d-up	2/272	0,908404459	0,984396466	0	0	0,505356782	0,048547382	RPS8;SEPP1
Vinorelbine-1.5_mg/kg_in_Saline-Rat-Bone_marrow-1d-up	2/272	0,908404459	0,984261042	0	0	0,505356782	0,048547382	C1QA;SEPP1
Allyl_Alcohol-16_mg/kg_in_Saline-Rat-Liver-0.25d-dn	2/273	0,909485413	0,985296714	0	0	0,503505658	0,047770764	RPS17;RPL10A
Chlortetracycline-1500_mg/kg_in_CMC-Rat-Liver-5d-dn	2/273	0,909485413	0,985161203	0	0	0,503505658	0,047770764	EEF1B2;SEPP1
Cycloheximide-0.25_mg/kg_in_Water-Rat-Liver-1d-dn	2/273	0,909485413	0,98502573	0	0	0,503505658	0,047770764	C1QA;SEPP1
Sparfloxacin-450_mg/kg_in_Corn_Oil-Rat-Liver-0.25d-dn	2/273	0,909485413	0,984890294	0	0	0,503505658	0,047770764	RPL32;RPL35A
Streptomycin-120_mg/kg_in_Saline-Rat-Kidney-5d-up	2/273	0,909485413	0,984754895	0	0	0,503505658	0,047770764	RPS17;RPSA
Pyridine-223_mg/kg_in_Water-Rat-Kidney-1d-up	3/372	0,909753561	0,984909834	0	0	0,554262277	0,052422973	RPS8;SEPP1;TPT1
Azathioprine-20_mg/kg_in_Corn_Oil-Rat-Kidney-3d-up	2/274	0,910554446	0,985641399	0	0	0,501668046	0,04700709	C1QA;RPS3A
Benzyl_Acetate-1868_mg/kg_in_CMC-Rat-Kidney-5d-dn	2/274	0,910554446	0,985505953	0	0	0,501668046	0,04700709	ARL2BP;SEPP1
Cytarabine-487_mg/kg_in_Saline-Rat-Bone_marrow-1d-dn	2/274	0,910554446	0,985370544	0	0	0,501668046	0,04700709	FEM1B;RPL39
Guanethidine-132_mg/kg_in_CMC-Rat-Heart-3d-dn	2/274	0,910554446	0,985235172	0	0	0,501668046	0,04700709	ARL2BP;COL5A3
Mycophenolate_Mofetil-63_mg/kg_in_CMC-Rat-Bone_marrow-5d-dn	2/274	0,910554446	0,985099838	0	0	0,501668046	0,04700709	FEM1B;TPT1
Atenolol-1000_mg/kg_in_CMC-Rat-Heart-5d-dn	2/275	0,911611676	0,986108167	0	0	0,499843799	0,046256131	COL5A3;RPS20
Indomethacin-9.6_mg/kg_in_Water-Rat-Kidney-5d-dn	2/275	0,911611676	0,98597275	0	0	0,499843799	0,046256131	CLP1;SEPP1
Isoprenaline-4.2_mg/kg_in_Saline-Rat-Heart-3d-dn	2/275	0,911611676	0,98583737	0	0	0,499843799	0,046256131	FUNDC1;TPT1
Oxcarbazepine-1600_mg/kg_in_CMC-Rat-Heart-5d-up	2/275	0,911611676	0,985702027	0	0	0,499843799	0,046256131	EEF1B2;RAPGEF5
Alfacalcidol-0.043_mg/kg_in_CMC-Rat-Heart-5d-up	2/276	0,912657218	0,986697083	0	0	0,498032771	0,045517662	CLP1;RPL39
Busulfan-9_mg/kg_in_Corn_Oil-Rat-Liver-5d-dn	2/276	0,912657218	0,98656166	0	0	0,498032771	0,045517662	RPSA;TPT1
Lomustine-4.2_mg/kg_in_Corn_Oil-Rat-Liver-0.25d-dn	2/276	0,912657218	0,986426273	0	0	0,498032771	0,045517662	CLP1;RPS20

Nortriptyline-63_mg/kg_in_Water-Rat-Heart-0.25d-dn	2/276	0,912657218	0,986290924	0	0	0,498032771	0,045517662	RAPGEF5;SEPP1
Phenobarbital-25_mg/kg_in_Water-Rat-Liver-0.25d-dn	2/276	0,912657218	0,986155611	0	0	0,498032771	0,045517662	EEF1B2;TPT1
Sulpiride-1000_mg/kg_in_CMC-Rat-Heart-3d-dn	2/276	0,912657218	0,986020336	0	0	0,498032771	0,045517662	C1QA;RPL39
Tetracaine-24_mg/kg_in_Corn_Oil-Rat-Heart-3d-dn	2/276	0,912657218	0,985885098	0	0	0,498032771	0,045517662	GOLGA7;SEPP1
Thalidomide-113_mg/kg_in_CMC-Rat-Kidney-3d-up	2/276	0,912657218	0,985749897	0	0	0,498032771	0,045517662	RPS17;CLP1
Buspirone-196_mg/kg_in_CMC-Rat-Heart-1d-up	2/277	0,913691187	0,986731357	0	0	0,496234818	0,044791462	FUNDC1;RAPGEF5
Digoxin-11_mg/kg_in_Corn_Oil-Rat-Liver-1d-up	2/277	0,913691187	0,986596078	0	0	0,496234818	0,044791462	RPS20;RPL39
Ethanol-6000_mg/kg_in_Saline-Rat-Liver-3d-dn	2/277	0,913691187	0,986460835	0	0	0,496234818	0,044791462	CLP1;SEPP1
Neomycin-877_mg/kg_in_Corn_Oil-Rat-Liver-5d-dn	2/277	0,913691187	0,986325629	0	0	0,496234818	0,044791462	RPL10A;SEPP1
Tetracycline-520_uM_in_DMSO-Rat-Primary_rat_hepatocytes-1d-dn	2/277	0,913691187	0,98619046	0	0	0,496234818	0,044791462	RPS20;SEPP1
Mercuric_Chloride-2.5_mg/kg_in_Water-Rat-Kidney-3d-up	3/377	0,914333312	0,98674831	0	0	0,546911318	0,048981433	ARL2BP;CLP1;RPL10A
Azathioprine-20_mg/kg_in_Corn_Oil-Rat-Liver-0.25d-dn	2/278	0,914713696	0,987023575	0	0	0,494449801	0,044077314	RPL32;RPL35A
Carbon_Tetrachloride-400_mg/kg_in_Corn_Oil-Rat-Kidney-3d-dn	2/278	0,914713696	0,986888366	0	0	0,494449801	0,044077314	RPL39;TPT1
Ciprofloxacin-450_mg/kg_in_Corn_Oil-Rat-Liver-5d-dn	2/278	0,914713696	0,986753195	0	0	0,494449801	0,044077314	RPS6;RPL10A
Cisplatin-2_mg/kg_in_Saline-Rat-Kidney-5d-dn	2/278	0,914713696	0,98661806	0	0	0,494449801	0,044077314	ARL2BP;SEPP1
Mifepristone-300_mg/kg_in_Corn_Oil-Rat-Liver-1d-dn	2/278	0,914713696	0,986482962	0	0	0,494449801	0,044077314	C1QA;SEPP1
Progesterone-11.3_mg/kg_in_Corn_Oil-Rat-Liver-1d-dn	2/278	0,914713696	0,986347902	0	0	0,494449801	0,044077314	C1QA;SEPP1
Rifampin-99_mg/kg_in_CMC-Rat-Liver-3d-dn	2/278	0,914713696	0,986212878	0	0	0,494449801	0,044077314	C1QA;TPT1
Rapamycin-60_mg/kg_in_CMC-Rat-Kidney-5d-dn	1/168	0,915647775	0,987084845	0	0	0,409098347	0,036051184	SEPP1
Aldosterone-0.065_mg/kg_in_Saline-Rat-Heart-3d-up	2/279	0,915724859	0,987032844	0	0	0,492677579	0,043375005	ARL2BP;CLP1
Cholic_Acid-1402_mg/kg_in_Water-Rat-Liver-5d-dn	2/279	0,915724859	0,986897782	0	0	0,492677579	0,043375005	CLP1;TPT1
Dexchlorpheniramine-3_mg/kg_in_Water-Rat-Heart-3d-dn	2/279	0,915724859	0,986762757	0	0	0,492677579	0,043375005	EEF1B2;SEPP1
Leflunomide-60_mg/kg_in_Corn_Oil-Rat-Spleen-5d-dn	2/279	0,915724859	0,986627769	0	0	0,492677579	0,043375005	RPS5;RPS6
Miconazole-920_mg/kg_in_Corn_Oil-Rat-Liver-1d-up	2/279	0,915724859	0,986492818	0	0	0,492677579	0,043375005	RPS17;RPS3A
Terbutaline-130_mg/kg_in_Corn_Oil-Rat-Heart-3d-dn	2/279	0,915724859	0,986357904	0	0	0,492677579	0,043375005	CLP1;ARL6IP5
Dicyclomine-645_mg/kg_in_Water-Rat-Heart-1d-up	2/280	0,916724788	0,987299936	0	0	0,490918017	0,042684327	SEPP1;TPT1
Erythromycin-1500_mg/kg_in_CMC-Rat-Liver-5d-dn	2/280	0,916724788	0,987164949	0	0	0,490918017	0,042684327	RPL39;SEPP1
Gabapentin-500_uM_in_DMSO-Rat-Primary_rat_hepatocytes-1d-up	2/280	0,916724788	0,987029998	0	0	0,490918017	0,042684327	EEF1B2;RPS20
Rosiglitazone-10_mg/kg_in_Corn_Oil-Rat-Liver-1d-dn	2/280	0,916724788	0,986895084	0	0	0,490918017	0,042684327	RPS17;RPL39
3-Methylcholanthrene-12.5_mg/kg_in_CMC-Rat-Liver-91d-up	2/281	0,917713595	0,987824555	0	0	0,489170977	0,042005075	RPL39;SEPP1
Amitraz-75_mg/kg_in_CMC-Rat-Heart-1d-up	2/281	0,917713595	0,98768957	0	0	0,489170977	0,042005075	FUNDC1;RAPGEF5
Catechol-195_mg/kg_in_Saline-Rat-Kidney-5d-dn	2/281	0,917713595	0,987554621	0	0	0,489170977	0,042005075	R3HCC1;RPL10A
Clemastine-5.4_mg/kg_in_CMC-Rat-Heart-5d-dn	2/281	0,917713595	0,987419709	0	0	0,489170977	0,042005075	ARL2BP;FUNDC1
Phenacetin-619_mg/kg_in_Corn_Oil-Rat-Kidney-3d-up	2/281	0,917713595	0,987284834	0	0	0,489170977	0,042005075	CLP1;RPS8
Stavudine-1400_mg/kg_in_Water-Rat-Liver-3d-dn	2/281	0,917713595	0,987149996	0	0	0,489170977	0,042005075	CLP1;SEPP1
Tolazamide-1500_mg/kg_in_CMC-Rat-Heart-3d-up	2/281	0,917713595	0,987015195	0	0	0,489170977	0,042005075	CLP1;RPS8
Pyridine-223_mg/kg_in_Water-Rat-Kidney-3d-up	3/381	0,917844284	0,98702097	0	0	0,541169467	0,04639312	CLP1;RAPGEF5;SEPP1
Bisphenol_A-610_mg/kg_in_Corn_Oil-Rat-Liver-5d-dn	2/282	0,918691389	0,987797048	0	0	0,487436329	0,04133705	RPS6;RPL10A
Busulfan-3_mg/kg_in_Corn_Oil-Rat-Liver-3d-up	2/282	0,918691389	0,987662214	0	0	0,487436329	0,04133705	RPS20;SEPP1
Carmustine-4_mg/kg_in_Corn_Oil-Rat-Spleen-0.25d-dn	2/282	0,918691389	0,987527416	0	0	0,487436329	0,04133705	EEF1B2;TPT1
Cholecalciferol-8_mg/kg_in_CMC-Rat-Liver-1d-dn	2/282	0,918691389	0,987392655	0	0	0,487436329	0,04133705	RPSA;SEPP1
Cyclosporin_A-70_mg/kg_in_Corn_Oil-Rat-Kidney-3d-dn	2/282	0,918691389	0,987257931	0	0	0,487436329	0,04133705	CLP1;SEPP1
Fluvastatin-5_mg/kg_in_Corn_Oil-Rat-Liver-0.25d-dn	2/282	0,918691389	0,987123244	0	0	0,487436329	0,04133705	RPS17;RPS8
Hexachlorophene-8_mg/kg_in_Corn_Oil-Rat-Liver-5d-dn	2/282	0,918691389	0,986988593	0	0	0,487436329	0,04133705	RPS6;RPL10A
Naproxen-10_mg/kg_in_Corn_Oil-Rat-Liver-1d-dn	2/282	0,918691389	0,986853979	0	0	0,487436329	0,04133705	GOLGA7;SEPP1
Sodium_Selenite-2_mg/kg_in_Water-Rat-Kidney-1d-up	3/382	0,918701356	0,986730107	0	0	0,539752793	0,045767893	CLP1;RPS8;TPT1
Clindamycin-161_mg/kg_in_Saline-Rat-Kidney-5d-dn	2/283	0,91965828	0,987623209	0	0	0,485713939	0,040680054	RPS8;RPS6
Cortisone-206_mg/kg_in_CMC-Rat-Heart-3d-dn	2/283	0,91965828	0,987488564	0	0	0,485713939	0,040680054	COL5A3;RPS6
Digoxin-11_mg/kg_in_Corn_Oil-Rat-Liver-3d-up	2/283	0,91965828	0,987353955	0	0	0,485713939	0,040680054	RPL39;SEPP1
Gabapentin-2500_mg/kg_in_Water-Rat-Heart-3d-dn	2/283	0,91965828	0,987219383	0	0	0,485713939	0,040680054	EEF1B2;RPS8
Rifampin-99_mg/kg_in_CMC-Rat-Kidney-5d-up	2/283	0,91965828	0,987084848	0	0	0,485713939	0,040680054	CLP1;RPSA
Tolazamide-1500_mg/kg_in_CMC-Rat-Liver-1d-dn	2/283	0,91965828	0,986950349	0	0	0,485713939	0,040680054	C1QA;RPL39
4-Octylphenol-700_mg/kg_in_Corn_Oil-Rat-Kidney-3d-dn	2/284	0,920614378	0,987841804	0	0	0,484003678	0,040033895	CLP1;SEPP1
Acetaminophen-100_mg/kg_in_Corn_Oil-Rat-Liver-3d-up	2/284	0,920614378	0,987707239	0	0	0,484003678	0,040033895	RPS20;SEPP1
Fenofibrate-250_uM_in_DMSO-Rat-Primary_rat_hepatocytes-1d-up	2/284	0,920614378	0,98757271	0	0	0,484003678	0,040033895	RPS19;TPT1
Fluocinolone_Acetonide-2.5_mg/kg_in_Corn_Oil-Rat-Liver-5d-up	2/284	0,920614378	0,987438219	0	0	0,484003678	0,040033895	CLP1;TPT1
Hydrocortisone-56_mg/kg_in_Corn_Oil-Rat-Liver-5d-up	2/284	0,920614378	0,987303764	0	0	0,484003678	0,040033895	CLP1;SEPP1
Ifosfamide-17_mg/kg_in_Saline-Rat-Liver-0.25d-up	2/284	0,920614378	0,987169345	0	0	0,484003678	0,040033895	RPS8;SEPP1
Paroxetine-104_mg/kg_in_Water-Rat-Brain-5d-dn	2/284	0,920614378	0,987034963	0	0	0,484003678	0,040033895	RPS8;SEPP1
Penciclovir-1200_mg/kg_in_Corn_Oil-Rat-Kidney-3d-up	2/284	0,920614378	0,986900618	0	0	0,484003678	0,040033895	C1QA;CLP1
Pirinixic_Acid-364_mg/kg_in_CMC-Rat-Liver-1d-dn	2/284	0,920614378	0,986766309	0	0	0,484003678	0,040033895	CLP1;SEPP1
Primaquine-45_mg/kg_in_CMC-Rat-Liver-3d-dn	2/284	0,920614378	0,986632037	0	0	0,484003678	0,040033895	CLP1;SEPP1
Progesterone-11.3_mg/kg_in_Corn_Oil-Rat-Liver-5d-dn	2/284	0,920614378	0,986497801	0	0	0,484003678	0,040033895	EEF1B2;C1QA
Sildenafil-2.5_mg/kg_in_Water-Rat-Liver-3d-dn	2/284	0,920614378	0,986363602	0	0	0,484003678	0,040033895	RPL39;TPT1
Sotalol-2000_mg/kg_in_Water-Rat-Heart-1d-dn	2/284	0,920614378	0,98622944	0	0	0,484003678	0,040033895	EEF1B2;SEPP1
Spironolactone-300_mg/kg_in_CMC-Rat-Liver-5d-up	2/284	0,920614378	0,986095313	0	0	0,484003678	0,040033895	FEM1B;SEPP1
2-Acetylaminofluorene-30_mg/kg_in_CMC-Rat-Kidney-5d-up	2/285	0,92155979	0,986973743	0	0	0,48230542	0,039398382	RPS17;CLP1
4-Chloro-2-Nitroaniline-1200_mg/kg_in_CMC-Rat-Kidney-5d-dn	2/285	0,92155979	0,986839552	0	0	0,48230542	0,039398382	ARL2BP;SEPP1
Clotrimazole-178_mg/kg_in_Corn_Oil-Rat-Liver-0.25d-up	2/285	0,92155979	0,986705397	0	0	0,48230542	0,039398382	HOXA1;RPL10A
Furan-24_mg/kg_in_Corn_Oil-Rat-Heart-5d-dn	2/285	0,92155979	0,98657128	0	0	0,48230542	0,039398382	RPS19;RPS6
Gatifloxacin-770_mg/kg_in_Corn_Oil-Rat-Heart-1d-up	2/285	0,92155979	0,986437198	0	0	0,48230542	0,039398382	RPS19;CLP1
Itraconazole-30_mg/kg_in_Corn_Oil-Rat-Liver-1d-dn	2/285	0,92155979	0,986303153	0	0	0,48230542	0,039398382	C1QA;SEPP1
Mevastatin-1200_mg/kg_in_CMC-Rat-Liver-5d-dn	2/285	0,92155979	0,986169144	0	0	0,48230542	0,039398382	EEF1B2;RPL39
Phenobarbital-80_mg/kg_in_Water-Rat-Liver-1d-dn	2/285	0,92155979	0,986035172	0	0	0,48230542	0,039398382	RPL39;TPT1
Sumatriptan-1100_mg/kg_in_Water-Rat-Brain-5d-dn	2/285	0,92155979	0,985901237	0	0	0,48230542	0,039398382	RPS20;SEPP1
Amantadine-220_mg/kg_in_Water-Rat-Liver-1d-dn	2/286	0,922494623	0,986767303	0	0	0,480619037	0,038773331	RPL10A;SEPP1
Cefotaxime-1000_mg/kg_in_Saline-Rat-Kidney-5d-dn	2/286	0,922494623	0,986633304	0	0	0,480619037	0,038773331	EEF1B2;RPS19
Chlorambucil-0.6_mg/kg_in_Corn_Oil-Rat-Spleen-0.25d-dn	2/286	0,922494623	0,986499342	0	0	0,480619037	0,038773331	RPS19;RPS8
Cyclophosphamide-25_mg/kg_in_Water-Rat-Heart-3d-dn	2/286	0,922494623	0,986365416	0	0	0,480619037	0,038773331	SEPP1;TPT1
Idarubicin-4_mg/kg_in_Saline-Rat-Kidney-14d-dn	2/286	0,922494623	0,986231526	0	0	0,480619037	0,038773331	RPL13A;TPT1
Isotretinoin-125_mg/kg_in_Corn_Oil-Rat-Liver-5d-dn	2/286	0,922494623	0,986097673	0	0	0,480619037	0,038773331	RPS6;RPL10A
Phenothiazine-386_mg/kg_in_Corn_Oil-Rat-Liver-0.25d-dn	2/286	0,922494623	0,985963856	0	0	0,480619037	0,038773331	C1QA;RPSA
Theophylline-225_mg/kg_in_Water-Rat-Brain-3d-up	2/286	0,922494623	0,985830075	0	0	0,480619037	0,038773331	ARL2BP;RPS8
Acetazolamide-250_mg/kg_in_Corn_Oil-Rat-Liver-3d-dn	2/287	0,923418984	0,986684021	0	0	0,478944407	0,038158559	C1QA;SEPP1
Doxorubicin-4_mg/kg_in_Saline-Rat-Kidney-14d-dn	2/287	0,923418984	0,986550179	0	0	0,478944407	0,038158559	C1QA;TPT1
Fluvastatin-94_mg/kg_in_Corn_Oil-Rat-Liver-1d-up	2/287	0,923418984	0,986416373	0	0	0,478944407	0,038158559	RPS19;SEPP1
Methotrexate-0.3_mg/kg_in_Corn_Oil-Rat-Liver-0.25d-dn	2/287	0,923418984	0,986282603	0	0	0,478944407	0,038158559	RPSA;RPL39
Thiabendazole-92_mg/kg_in_Corn_Oil-Rat-Liver-3d-dn	2/287	0,923418984	0,98614887	0	0	0,478944407	0,038158559	RPS8;TPT1
Epalrestat-2500_mg/kg_in_CMC-Rat-Liver-0.25d-dn	2/288	0,924332978	0,986991125	0	0	0,477281405	0,037553888	SEPP1;TPT1
Estriol-313_mg/kg_in_CMC-Rat-Liver-1d-dn	2/288	0,924332978	0,986857332	0	0	0,477281405	0,037553888	C1QA;SEPP1

Griseofulvin-75_uM_in_DMSO-Rat-Primary_rat_hepatocytes-0.67d-up	2/288	0,924332978	0,986723575	0	0	0,477281405	0,037553888	ARL2BP;RPS20
Minoxidil-83_mg/kg_in_CMC-Rat-Heart-5d-dn	2/288	0,924332978	0,986589854	0	0	0,477281405	0,037553888	RPS8;ARL6IP5
1-Naphthyl_Isothiocyanate-15_mg/kg_in_Corn_Oil-Rat-Liver-5d-dn	2/289	0,92523671	0,987420641	0	0	0,475629912	0,036959142	RPS6;TPT1
Abamectin-10_mg/kg_in_Water-Rat-Liver-1d-dn	2/289	0,92523671	0,987286862	0	0	0,475629912	0,036959142	GOLGA7;SEPP1
Azathioprine-54_mg/kg_in_Water-Rat-Liver-0.25d-dn	2/289	0,92523671	0,98715312	0	0	0,475629912	0,036959142	RPSA;RPL39
Chlorzoxazone-763_mg/kg_in_CMC-Rat-Liver-1d-dn	2/289	0,92523671	0,987019413	0	0	0,475629912	0,036959142	C1QA;SEPP1
Gentamicin-267_mg/kg_in_Corn_Oil-Rat-Kidney-5d-dn	2/289	0,92523671	0,986885743	0	0	0,475629912	0,036959142	EEF1B2;SEPP1
Progesterone-11.3_mg/kg_in_Corn_Oil-Rat-Liver-0.25d-dn	2/289	0,92523671	0,986752109	0	0	0,475629912	0,036959142	RPSA;RPL39
Propylene_Glycol-2000_mg/kg_in_Water-Rat-Liver-1d-dn	2/289	0,92523671	0,986618512	0	0	0,475629912	0,036959142	GOLGA7;SEPP1
Tamoxifen-2.5_mg/kg_in_Corn_Oil-Rat-Liver-1d-dn	2/289	0,92523671	0,98648495	0	0	0,475629912	0,036959142	RPL39;TPT1
Triacetin-1200_mg/kg_in_CMC-Rat-Liver-3d-dn	2/289	0,92523671	0,986351425	0	0	0,475629912	0,036959142	GOLGA7;SEPP1
Vinblastine-0.3_mg/kg_in_Saline-Rat-Liver-5d-dn	2/289	0,92523671	0,986217936	0	0	0,475629912	0,036959142	CLP1;SEPP1
2-Nitroanisole-740_mg/kg_in_CMC-Rat-Kidney-5d-dn	2/290	0,926130283	0,987036822	0	0	0,473989809	0,036374148	RPL32;RPS6
Beta-Estradiol-150_mg/kg_in_Corn_Oil-Rat-Liver-1d-dn	2/290	0,926130283	0,986903276	0	0	0,473989809	0,036374148	C1QA;SEPP1
Chlortetracycline-1500_mg/kg_in_CMC-Rat-Liver-1d-dn	2/290	0,926130283	0,986769766	0	0	0,473989809	0,036374148	RPL35A;SEPP1
Glyburide-1500_mg/kg_in_CMC-Rat-Heart-3d-dn	2/290	0,926130283	0,986636293	0	0	0,473989809	0,036374148	EEF1B2;SEPP1
Lead_(II)_Acetate-600_mg/kg_in_Saline-Rat-Liver-0.25d-dn	2/290	0,926130283	0,986502855	0	0	0,473989809	0,036374148	CLP1;TPT1
Terfenadine-750_mg/kg_in_CMC-Rat-Heart-3d-dn	2/290	0,926130283	0,986369454	0	0	0,473989809	0,036374148	COL5A3;RPSA
2-Amino-4-Nitrophenol-625_mg/kg_in_CMC-Rat-Kidney-3d-dn	2/291	0,927013801	0,987176947	0	0	0,472360978	0,035798739	ARL2BP;CLP1
Betamethasone-79_mg/kg_in_Corn_Oil-Rat-Liver-1d-up	2/291	0,927013801	0,98704349	0	0	0,472360978	0,035798739	RPS20;RPL39
Cholecalciferol-8_mg/kg_in_CMC-Rat-Liver-5d-dn	2/291	0,927013801	0,98691007	0	0	0,472360978	0,035798739	RPL39;SEPP1
Clotrimazole-89_mg/kg_in_Corn_Oil-Rat-Liver-0.25d-dn	2/291	0,927013801	0,986776686	0	0	0,472360978	0,035798739	EEF1B2;SEPP1
Diclofenac-3.5_mg/kg_in_Corn_Oil-Rat-Liver-0.25d-up	2/291	0,927013801	0,986643338	0	0	0,472360978	0,035798739	RPS19;RPS6
Erythromycin-1500_mg/kg_in_CMC-Rat-Kidney-5d-dn	2/291	0,927013801	0,986510025	0	0	0,472360978	0,035798739	ARL2BP;RPSA
Ethosuximide-1200_mg/kg_in_Water-Rat-Brain-5d-dn	2/291	0,927013801	0,986376749	0	0	0,472360978	0,035798739	RPS8;FEM1B
Fludrocortisone_Acetate-125_mg/kg_in_CMC-Rat-Heart-1d-up	2/291	0,927013801	0,986243509	0	0	0,472360978	0,035798739	ARL6IP5;SEPP1
Imatinib-150_mg/kg_in_Water-Rat-Liver-5d-up	2/291	0,927013801	0,986110305	0	0	0,472360978	0,035798739	RPS8;TPT1
Irinotecan-5_mg/kg_in_Saline-Rat-Heart-3d-up	2/291	0,927013801	0,985977137	0	0	0,472360978	0,035798739	RAPGEF5;SEPP1
Itraconazole-30_mg/kg_in_Corn_Oil-Rat-Liver-0.25d-dn	2/291	0,927013801	0,985844005	0	0	0,472360978	0,035798739	C1QA;SEPP1
Lead(IV)_Acetate-600_mg/kg_in_Saline-Rat-Kidney-5d-dn	2/291	0,927013801	0,985710908	0	0	0,472360978	0,035798739	ARL2BP;SEPP1
Microcystin-LR-0.07_uM_in_DMSO-Rat-Primary_rat_hepatocytes-0.67d-dn	2/291	0,927013801	0,985577848	0	0	0,472360978	0,035798739	SEPP1;TPT1
Buflomedil-615_mg/kg_in_CMC-Rat-Heart-1d-up	2/292	0,927887366	0,98637345	0	0	0,470743304	0,035232748	RAPGEF5;TPT1
Cisplatin-2_mg/kg_in_Saline-Rat-Liver-3d-dn	2/292	0,927887366	0,986240336	0	0	0,470743304	0,035232748	SEPP1;TPT1
Hydrocortisone-56_mg/kg_in_Corn_Oil-Rat-Kidney-5d-up	2/292	0,927887366	0,986107258	0	0	0,470743304	0,035232748	EEF1B2;RPS20
Irinotecan-5_mg/kg_in_Saline-Rat-Kidney-3d-up	2/292	0,927887366	0,985974216	0	0	0,470743304	0,035232748	RAPGEF5;RPL10A
Phenelzine-27_mg/kg_in_Water-Rat-Brain-5d-dn	2/292	0,927887366	0,98584121	0	0	0,470743304	0,035232748	RPS8;TOMM20
Rosiglitazone-1800_mg/kg_in_Corn_Oil-Rat-Skeletal_muscle-3d-up	2/292	0,927887366	0,98570824	0	0	0,470743304	0,035232748	RPS19;TPT1
TNF-alpha-0.25_mg/kg_in_RSA/Saline-Rat-Liver-1d-dn	2/292	0,927887366	0,985575306	0	0	0,470743304	0,035232748	C1QA;CLP1
Tranilast-300_mg/kg_in_CMC-Rat-Heart-3d-up	2/292	0,927887366	0,985442407	0	0	0,470743304	0,035232748	RAPGEF5;TOMM20
Cholecalciferol-8_mg/kg_in_CMC-Rat-Kidney-5d-dn	2/293	0,928751078	0,986226707	0	0	0,469136671	0,034676013	RPS3A;SEPP1
Cyclandelate-1500_mg/kg_in_CMC-Rat-Liver-1d-dn	2/293	0,928751078	0,986093757	0	0	0,469136671	0,034676013	C1QA;RPS19
Cytarabine-487_mg/kg_in_Saline-Rat-Bone_marrow-5d-dn	2/293	0,928751078	0,985960842	0	0	0,469136671	0,034676013	NKG7;DUSP14
Hydroxyurea-59_mg/kg_in_Saline-Rat-Spleen-3d-up	2/293	0,928751078	0,985827963	0	0	0,469136671	0,034676013	RPS19;SEPP1
Melphalan-12_mg/kg_in_CMC-Rat-Bone_marrow-5d-up	2/293	0,928751078	0,98569512	0	0	0,469136671	0,034676013	EEF1B2;RPS3A
Mitomycin_C-1.7_mg/kg_in_Saline-Rat-Liver-3d-dn	2/293	0,928751078	0,985562313	0	0	0,469136671	0,034676013	SEPP1;TPT1
Sildenafil-2.5_mg/kg_in_Water-Rat-Liver-5d-dn	2/293	0,928751078	0,985429542	0	0	0,469136671	0,034676013	RPSA;SEPP1
Tenidap-75_mg/kg_in_Corn_Oil-Rat-Kidney-5d-dn	2/293	0,928751078	0,985296806	0	0	0,469136671	0,034676013	EEF1B2;RPS8
4-Nonylphenol-20_mg/kg_in_Corn_Oil-Rat-Liver-0.25d-dn	2/294	0,929605038	0,986069937	0	0	0,467540968	0,034128374	RPS3A;TPT1
Bromodichloromethane-430_mg/kg_in_CMC-Rat-Kidney-5d-dn	2/294	0,929605038	0,985937151	0	0	0,467540968	0,034128374	ARL2BP;SEPP1
Cortisone-206_mg/kg_in_CMC-Rat-Liver-3d-up	2/294	0,929605038	0,9858044	0	0	0,467540968	0,034128374	RPSA;RPL39
Dexamethasone-1_mg/kg_in_Corn_Oil-Rat-Liver-1d-dn	2/294	0,929605038	0,985671686	0	0	0,467540968	0,034128374	C1QA;SEPP1
Dobutamine-43_mg/kg_in_Saline-Rat-Heart-3d-dn	2/294	0,929605038	0,985539007	0	0	0,467540968	0,034128374	ARL2BP;RPS8
Omeprazole-415_mg/kg_in_Corn_Oil-Rat-Liver-5d-dn	2/294	0,929605038	0,985406363	0	0	0,467540968	0,034128374	C1QA;RPL39
Rapamycin-60_mg/kg_in_CMC-Rat-Heart-1d-dn	2/294	0,929605038	0,985273756	0	0	0,467540968	0,034128374	COL5A3;SEPP1
Albendazole-62_mg/kg_in_Corn_Oil-Rat-Liver-0.25d-dn	2/295	0,930449347	0,986035933	0	0	0,465956084	0,033589675	EEF1B2;RPL39
Altretamine-40_mg/kg_in_Corn_Oil-Rat-Liver-1d-dn	2/295	0,930449347	0,985903276	0	0	0,465956084	0,033589675	RPS6;SEPP1
Amoxapine-313_mg/kg_in_CMC-Rat-Heart-5d-dn	2/295	0,930449347	0,985770656	0	0	0,465956084	0,033589675	RPS19;COL5A3
Benoxaprofen-89_mg/kg_in_CMC-Rat-Liver-5d-dn	2/295	0,930449347	0,98563807	0	0	0,465956084	0,033589675	C1QA;SEPP1
Clonazepam-2500_mg/kg_in_CMC-Rat-Liver-0.25d-dn	2/295	0,930449347	0,985505521	0	0	0,465956084	0,033589675	EEF1B2;SEPP1
Clonidine-6_mg/kg_in_Water-Rat-Heart-5d-dn	2/295	0,930449347	0,985373007	0	0	0,465956084	0,033589675	ARL2BP;COL5A3
Famciclovir-112_mg/kg_in_Saline-Rat-Bone_marrow-3d-up	2/295	0,930449347	0,985240529	0	0	0,465956084	0,033589675	RPS17;RPS8
Foscarnet-350_mg/kg_in_Saline-Rat-Bone_marrow-3d-up	2/295	0,930449347	0,985108086	0	0	0,465956084	0,033589675	EEF1B2;RPL10A
Indomethacin-9.6_mg/kg_in_Water-Rat-Liver-5d-dn	2/295	0,930449347	0,984975679	0	0	0,465956084	0,033589675	RPS8;RPSA
Interleukin_1_Beta-0.01_mg/kg_in_RSA/Saline-Rat-Liver-1d-dn	2/295	0,930449347	0,984843308	0	0	0,465956084	0,033589675	RPL10A;TPT1
Penciclovir-1200_mg/kg_in_Corn_Oil-Rat-Kidney-5d-up	2/295	0,930449347	0,984710972	0	0	0,465956084	0,033589675	C1QA;RPS19
Sulindac-64_mg/kg_in_Corn_Oil-Rat-Liver-1d-dn	2/295	0,930449347	0,984578672	0	0	0,465956084	0,033589675	RPSA;SEPP1
Amiodarone-147_mg/kg_in_CMC-Rat-Liver-1d-up	2/296	0,931284102	0,985329606	0	0	0,464381908	0,033059762	RPSA;TPT1
Bacitracin-380_mg/kg_in_Corn_Oil-Rat-Kidney-3d-dn	2/296	0,931284102	0,985197258	0	0	0,464381908	0,033059762	CLP1;SEPP1
Beta-Estradiol-150_mg/kg_in_Corn_Oil-Rat-Liver-3d-dn	2/296	0,931284102	0,985064946	0	0	0,464381908	0,033059762	C1QA;RPL35A
Nafenopin-338_mg/kg_in_Corn_Oil-Rat-Liver-3d-dn	2/296	0,931284102	0,984932669	0	0	0,464381908	0,033059762	C1QA;SEPP1
Penicillin_G-243_mg/kg_in_Saline-Rat-Kidney-5d-dn	2/296	0,931284102	0,984800428	0	0	0,464381908	0,033059762	EEF1B2;TPT1
Pioglitazone-300_mg/kg_in_Corn_Oil-Rat-Liver-1d-up	2/296	0,931284102	0,984668222	0	0	0,464381908	0,033059762	RPL32;SEPP1
Pyrogallol-1000_mg/kg_in_Water-Rat-Liver-5d-dn	2/296	0,931284102	0,984536052	0	0	0,464381908	0,033059762	RPL39;TPT1
Raloxifene-5_mg/kg_in_Corn_Oil-Rat-Liver-0.25d-dn	2/296	0,931284102	0,984403917	0	0	0,464381908	0,033059762	RPS17;SEPP1
DActinomycin-0.06_mg/kg_in_Saline-Rat-Bone_marrow-0.25d-dn	2/297	0,932109402	0,985144075	0	0	0,462818332	0,032538483	NKG7;SEPP1
Interleukin_6-0.125_mg/kg_in_RSA/Saline-Rat-Liver-1d-dn	2/297	0,932109402	0,985011894	0	0	0,462818332	0,032538483	CLP1;TPT1
Lead(IV)_Acetate-200_mg/kg_in_Saline-Rat-Liver-0.25d-dn	2/297	0,932109402	0,984879749	0	0	0,462818332	0,032538483	EEF1B2;TPT1
Mianserin-116_mg/kg_in_Corn_Oil-Rat-Heart-1d-dn	2/297	0,932109402	0,984747639	0	0	0,462818332	0,032538483	ARL2BP;FUNDC1
Sertraline-210_mg/kg_in_Corn_Oil-Rat-Liver-3d-up	2/297	0,932109402	0,984615564	0	0	0,462818332	0,032538483	RPSA;SEPP1
Stavudine-58_mg/kg_in_Water-Rat-Liver-1d-dn	2/297	0,932109402	0,984483525	0	0	0,462818332	0,032538483	RPS8;TPT1
Timolol-10_mg/kg_in_Water-Rat-Heart-0.25d-dn	2/297	0,932109402	0,984351521	0	0	0,462818332	0,032538483	COL5A3;SEPP1
Propylene_Glycol-2000_mg/kg_in_Water-Rat-Kidney-3d-up	3/400	0,932798892	0,98494759	0	0	0,515463918	0,035858583	CLP1;SEPP1;TPT1
Chloroform-600_mg/kg_in_Water-Rat-Liver-1d-dn	2/298	0,932925343	0,984949062	0	0	0,461265251	0,032025692	RPS19;SEPP1
Ibuprofen-263_mg/kg_in_Corn_Oil-Rat-Liver-0.25d-dn	2/298	0,932925343	0,984817049	0	0	0,461265251	0,032025692	RPS8;TPT1
Mestranol-250_mg/kg_in_CMC-Rat-Liver-5d-dn	2/298	0,932925343	0,984685072	0	0	0,461265251	0,032025692	EEF1B2;C1QA
Methotrexate-0.3_mg/kg_in_Corn_Oil-Rat-Spleen-0.25d-dn	2/298	0,932925343	0,984553129	0	0	0,461265251	0,032025692	RPS19;RPS6
NN'-Diphenyl-P-Phenylenediamine-1000_mg/kg_in_CMC-Rat-Liver-5d-dn	2/298	0,932925343	0,984421222	0	0	0,461265251	0,032025692	RPS20;RPSA
Phenobarbital-54_mg/kg_in_Water-Rat-Liver-5d-up	2/298	0,932925343	0,984289351	0	0	0,461265251	0,032025692	RPS19;PDRG1
Stavudine-1400_mg/kg_in_Water-Rat-Liver-1d-dn	2/298	0,932925343	0,984157515	0	0	0,461265251	0,032025692	CLP1;SEPP1
Valproic_Acid-1500_mg/kg_in_Water-Rat-Heart-1d-up	2/298	0,932925343	0,984025714	0	0	0,461265251	0,032025692	RPS8;TPT1

Pentamidine-18_mg/kg_in_Saline-Rat-Bone_marrow-3d-up	1/184	0,933419894	0,984415517	0	0	0,373524578	0,025735893	TPT1
3-Methylcholanthrene-300_mg/kg_in_CMC-Rat-Liver-1d-up	2/299	0,933732023	0,984612855	0	0	0,459722557	0,031521243	RPL32;RPSA
Busulfan-9_mg/kg_in_Corn_Oil-Rat-Liver-3d-dn	2/299	0,933732023	0,984481046	0	0	0,459722557	0,031521243	RPSA;RPL10A
Chlortetracycline-1500_mg/kg_in_CMC-Rat-Liver-3d-dn	2/299	0,933732023	0,984349273	0	0	0,459722557	0,031521243	C1QA;SEPP1
Digoxin-0.26_mg/kg_in_Corn_Oil-Rat-Liver-3d-up	2/299	0,933732023	0,984217534	0	0	0,459722557	0,031521243	RPS3A;RPL39
Ethanol-3000_mg/kg_in_Saline-Rat-Kidney-0.25d-dn	2/299	0,933732023	0,984085831	0	0	0,459722557	0,031521243	RPL10A;SEPP1
Hexachloro-13-Butadiene-50_mg/kg_in_Corn_Oil-Rat-Kidney-3d-dn	2/299	0,933732023	0,983954163	0	0	0,459722557	0,031521243	CLP1;SEPP1
Mycophenolate_Mofetil-63_mg/kg_in_CMC-Rat-Bone_marrow-1d-dn	2/299	0,933732023	0,98382253	0	0	0,459722557	0,031521243	RPS8;RPS3A
Oxfendazole-1500_mg/kg_in_CMC-Rat-Liver-1d-up	2/299	0,933732023	0,983690933	0	0	0,459722557	0,031521243	RPS19;RPSA
Oxfendazole-1500_mg/kg_in_CMC-Rat-Liver-5d-up	2/299	0,933732023	0,983559371	0	0	0,459722557	0,031521243	RPS6;RPL39
Paroxetine-104_mg/kg_in_Water-Rat-Brain-3d-dn	2/299	0,933732023	0,983427844	0	0	0,459722557	0,031521243	RPL39;PDRG1
Terbutaline-130_mg/kg_in_Corn_Oil-Rat-Heart-1d-dn	2/299	0,933732023	0,983296352	0	0	0,459722557	0,031521243	FUNDC1;SEPP1
Verapamil-108_mg/kg_in_Water-Rat-Heart-3d-dn	2/299	0,933732023	0,983164895	0	0	0,459722557	0,031521243	EEF1B2;SEPP1
2-Amino-4-Nitrophenol-625_mg/kg_in_CMC-Rat-Kidney-1d-dn	2/300	0,934529537	0,983873096	0	0	0,458190149	0,031024992	ARL2BP;SEPP1
Benzoic_Acid-1700_mg/kg_in_CMC-Rat-Liver-5d-dn	2/300	0,934529537	0,983741598	0	0	0,458190149	0,031024992	CLP1;SEPP1
Chloroform-600_mg/kg_in_Corn_Oil-Rat-Liver-1d-dn	2/300	0,934529537	0,983610134	0	0	0,458190149	0,031024992	C1QA;SEPP1
Ciprofloxacin-72_mg/kg_in_Corn_Oil-Rat-Liver-0.25d-dn	2/300	0,934529537	0,983478706	0	0	0,458190149	0,031024992	RPS6;SEPP1
Clonidine-6_mg/kg_in_Water-Rat-Heart-3d-dn	2/300	0,934529537	0,983347312	0	0	0,458190149	0,031024992	EEF1B2;CXCL11
Ferulic_Acid-1500_mg/kg_in_CMC-Rat-Liver-1d-dn	2/300	0,934529537	0,983215954	0	0	0,458190149	0,031024992	C1QA;SEPP1
Lovastatin-450_mg/kg_in_Corn_Oil-Rat-Liver-5d-dn	2/300	0,934529537	0,983084631	0	0	0,458190149	0,031024992	CLP1;SEPP1
Modafinil-325_mg/kg_in_Saline-Rat-Liver-3d-dn	2/300	0,934529537	0,982953343	0	0	0,458190149	0,031024992	SEPP1;TPT1
N-Nitrosodiethylamine-34_mg/kg_in_Saline-Rat-Kidney-3d-dn	2/300	0,934529537	0,98282209	0	0	0,458190149	0,031024992	RPSA;TPT1
NN-Dimethylformamide-140_mg/kg_in_Saline-Rat-Liver-0.25d-dn	2/300	0,934529537	0,982690872	0	0	0,458190149	0,031024992	RPS19;RPS20
Norethindrone-375_mg/kg_in_Corn_Oil-Rat-Liver-5d-dn	2/300	0,934529537	0,982559689	0	0	0,458190149	0,031024992	C1QA;SEPP1
Carboplatin-14_mg/kg_in_Saline-Rat-Liver-5d-up	2/301	0,935317979	0,983257395	0	0	0,456667923	0,030536801	RPS8;SEPP1
Diethylstilbestrol-280_mg/kg_in_Corn_Oil-Rat-Kidney-5d-dn	2/301	0,935317979	0,983126172	0	0	0,456667923	0,030536801	EEF1B2;SEPP1
Promethazine-113_mg/kg_in_Saline-Rat-Liver-5d-up	2/301	0,935317979	0,982994983	0	0	0,456667923	0,030536801	RPS19;RPSA
Atorvastatin-2.5_mg/kg_in_Corn_Oil-Rat-Liver-5d-dn	2/302	0,936097444	0,983682918	0	0	0,455155777	0,030056531	C1QA;SEPP1
Bezafibrate-617_mg/kg_in_Corn_Oil-Rat-Liver-0.25d-dn	2/302	0,936097444	0,98355169	0	0	0,455155777	0,030056531	CLP1;SEPP1
Bezafibrate-617_mg/kg_in_Corn_Oil-Rat-Skeletal_muscle-3d-up	2/302	0,936097444	0,983420497	0	0	0,455155777	0,030056531	ARL2BP;RPS20
Catechol-195_mg/kg_in_Saline-Rat-Liver-5d-up	2/302	0,936097444	0,98328934	0	0	0,455155777	0,030056531	RPSA;GOLGA7
Hydrazine-45_mg/kg_in_Water-Rat-Liver-1d-dn	2/302	0,936097444	0,983158217	0	0	0,455155777	0,030056531	EEF1B2;TPT1
Ketoconazole-114_mg/kg_in_Corn_Oil-Rat-Liver-1d-up	2/302	0,936097444	0,983027129	0	0	0,455155777	0,030056531	RPS19;RPL39
Ketoconazole-25_mg/kg_in_Corn_Oil-Rat-Liver-3d-up	2/302	0,936097444	0,982896076	0	0	0,455155777	0,030056531	RPSA;RPL39
Miconazole-920_mg/kg_in_Corn_Oil-Rat-Liver-3d-dn	2/302	0,936097444	0,982765058	0	0	0,455155777	0,030056531	C1QA;SEPP1
Miconazole-920_mg/kg_in_Corn_Oil-Rat-Liver-5d-dn	2/302	0,936097444	0,982634076	0	0	0,455155777	0,030056531	C1QA;SEPP1
N-Nitrosodiethylamine-1.67_mg/kg_in_Saline-Rat-Spleen-0.25d-dn	2/302	0,936097444	0,982503127	0	0	0,455155777	0,030056531	RPS6;RPL39
Oxaprozin-1500_mg/kg_in_CMC-Rat-Kidney-3d-dn	2/302	0,936097444	0,982372214	0	0	0,455155777	0,030056531	CLP1;SEPP1
Oxiconazole-1500_mg/kg_in_Corn_Oil-Rat-Liver-1d-dn	2/302	0,936097444	0,982241336	0	0	0,455155777	0,030056531	SEPP1;GOLGA7
Gatifloxacin-770_mg/kg_in_Corn_Oil-Rat-Heart-3d-up	2/303	0,936868024	0,98291895	0	0	0,453653613	0,029584048	RPS20;PDRG1
Lomefloxacin-2000_mg/kg_in_CMC-Rat-Liver-5d-dn	2/303	0,936868024	0,982788034	0	0	0,453653613	0,029584048	EEF1B2;ARL6IP5
Sisomicin-34_mg/kg_in_Saline-Rat-Kidney-3d-dn	2/303	0,936868024	0,982657153	0	0	0,453653613	0,029584048	RPL10A;RPL39
Tegafur-175_mg/kg_in_Water-Rat-Bone_marrow-5d-dn	2/303	0,936868024	0,982526306	0	0	0,453653613	0,029584048	RPS17;RPL10A
Vinorelbine-1.5_mg/kg_in_Saline-Rat-Liver-1d-up	2/303	0,936868024	0,982395494	0	0	0,453653613	0,029584048	SEPP1;GOLGA7
Acetaminophen-972_mg/kg_in_Corn_Oil-Rat-Liver-1d-dn	2/304	0,937629813	0,983063419	0	0	0,452161331	0,029119219	C1QA;SEPP1
Chlorpyrifos-82_mg/kg_in_CMC-Rat-Heart-3d-up	2/304	0,937629813	0,982932571	0	0	0,452161331	0,029119219	ARL6IP5;SEPP1
Diisopropyl_Fluorophosphate-3.75_mg/kg_in_Water-Rat-Heart-1d-up	2/304	0,937629813	0,982801757	0	0	0,452161331	0,029119219	RPS8;TPT1
Doxorubicin-3_mg/kg_in_Saline-Rat-Heart-5d-dn	2/304	0,937629813	0,982670978	0	0	0,452161331	0,029119219	RPS8;COL5A3
Epirubicin-2.7_mg/kg_in_Saline-Rat-Liver-5d-up	2/304	0,937629813	0,982540235	0	0	0,452161331	0,029119219	C1QA;SEPP1
Etoposide-188_mg/kg_in_Corn_Oil-Rat-Liver-5d-up	2/304	0,937629813	0,982409526	0	0	0,452161331	0,029119219	RPS6;SEPP1
Fluconazole-394_mg/kg_in_Corn_Oil-Rat-Liver-1d-dn	2/304	0,937629813	0,982278851	0	0	0,452161331	0,029119219	EEF1B2;SEPP1
Lovastatin-1500_mg/kg_in_Corn_Oil-Rat-Skeletal_muscle-3d-up	2/304	0,937629813	0,982148212	0	0	0,452161331	0,029119219	RPSA;SEPP1
N-Nitrosodiethylamine-34_mg/kg_in_Saline-Rat-Spleen-5d-dn	2/304	0,937629813	0,982017607	0	0	0,452161331	0,029119219	RPSA;SEPP1
Raloxifene-5_mg/kg_in_Corn_Oil-Rat-Liver-1d-dn	2/304	0,937629813	0,981887037	0	0	0,452161331	0,029119219	C1QA;SEPP1
Valproic_Acid-850_mg/kg_in_Water-Rat-Liver-5d-dn	2/304	0,937629813	0,981756501	0	0	0,452161331	0,029119219	RPSA;SEPP1
Warfarin-0.25_mg/kg_in_CMC-Rat-Liver-1d-dn	2/304	0,937629813	0,981626001	0	0	0,452161331	0,029119219	RPS8;RPL39
Amprenavir-600_mg/kg_in_CMC-Rat-Kidney-1d-up	2/305	0,9383829	0,982283855	0	0	0,450678835	0,028661914	EEF1B2;PDRG1
Anisindione-75_mg/kg_in_CMC-Rat-Liver-5d-dn	2/305	0,9383829	0,982153319	0	0	0,450678835	0,028661914	SEPP1;RPL39
Benzethonium_Chloride-30_mg/kg_in_Corn_Oil-Rat-Heart-5d-dn	2/305	0,9383829	0,982022817	0	0	0,450678835	0,028661914	COL5A3;ARL6IP5
Famciclovir-112_mg/kg_in_Saline-Rat-Liver-1d-up	2/305	0,9383829	0,98189235	0	0	0,450678835	0,028661914	RPS20;RPL39
Fluconazole-10_mg/kg_in_Corn_Oil-Rat-Liver-5d-dn	2/305	0,9383829	0,981761918	0	0	0,450678835	0,028661914	C1QA;RPL10A
Nicotine-75_mg/kg_in_Water-Rat-Brain-5d-dn	2/305	0,9383829	0,981631521	0	0	0,450678835	0,028661914	TOMM20;PDRG1
Azathioprine-20_mg/kg_in_Corn_Oil-Rat-Spleen-3d-dn	2/306	0,939127378	0,982279845	0	0	0,449206028	0,028212005	RPS19;TPT1
Beta-Estradiol-0.3_mg/kg_in_Corn_Oil-Rat-Liver-5d-dn	2/306	0,939127378	0,982149413	0	0	0,449206028	0,028212005	C1QA;RPS8
Clotrimazole-89_mg/kg_in_Corn_Oil-Rat-Liver-5d-dn	2/306	0,939127378	0,982019016	0	0	0,449206028	0,028212005	EEF1B2;SEPP1
Digoxin-11_mg/kg_in_Corn_Oil-Rat-Liver-0.25d-up	2/306	0,939127378	0,981888654	0	0	0,449206028	0,028212005	ARL6IP5;SEPP1
Norethindrone-0.08_mg/kg_in_Corn_Oil-Rat-Liver-5d-dn	2/306	0,939127378	0,981758326	0	0	0,449206028	0,028212005	SEPP1;TPT1
Aminoglutethimide-350_mg/kg_in_CMC-Rat-Liver-1d-up	2/307	0,939863336	0,982397297	0	0	0,447742817	0,027769368	RPSA;TPT1
Benoxaprofen-89_mg/kg_in_CMC-Rat-Liver-3d-dn	2/307	0,939863336	0,982266937	0	0	0,447742817	0,027769368	C1QA;SEPP1
Calcitriol-0.04_mg/kg_in_CMC-Rat-Heart-5d-dn	2/307	0,939863336	0,982136611	0	0	0,447742817	0,027769368	ARL6IP5;RPSA
Lovastatin-450_mg/kg_in_Corn_Oil-Rat-Liver-1d-up	2/307	0,939863336	0,98200632	0	0	0,447742817	0,027769368	RPS19;RPSA
Zileuton-450_mg/kg_in_Corn_Oil-Rat-Kidney-3d-dn	2/307	0,939863336	0,981876063	0	0	0,447742817	0,027769368	RPSA;SEPP1
Fenofibrate-43_mg/kg_in_Corn_Oil-Rat-Liver-0.25d-up	2/308	0,940590864	0,982505788	0	0	0,446289106	0,027333879	RPSA;TPT1
Nifedipine-1022_mg/kg_in_CMC-Rat-Heart-5d-dn	2/308	0,940590864	0,9823755	0	0	0,446289106	0,027333879	ARL2BP;COL5A3
6-Methoxy-2-Naphthylacetic_Acid-360_mg/kg_in_Saline-Rat-Kidney-5d-dn	2/309	0,941310049	0,98299628	0	0	0,444844805	0,026905417	ARL2BP;RPS6
Acrolein-20_mg/kg_in_Water-Rat-Heart-5d-dn	2/309	0,941310049	0,982865961	0	0	0,444844805	0,026905417	ARL2BP;COL5A3
Benzethonium_Chloride-30_mg/kg_in_Corn_Oil-Rat-Liver-1d-dn	2/309	0,941310049	0,982735677	0	0	0,444844805	0,026905417	SEPP1;RPL39
Beta-Estradiol_3-Benzoate-25_mg/kg_in_Corn_Oil-Rat-Liver-5d-dn	2/309	0,941310049	0,982605427	0	0	0,444844805	0,026905417	C1QA;SEPP1
Cisplatin-1.17_mg/kg_in_Saline-Rat-Liver-0.25d-dn	2/309	0,941310049	0,982475212	0	0	0,444844805	0,026905417	RPS8;RPSA
Famciclovir-112_mg/kg_in_Saline-Rat-Bone_marrow-0.25d-dn	1/193	0,941721268	0,982774176	0	0	0,356106333	0,02138274	RPS6
Ethylestrenol-390_mg/kg_in_CMC-Rat-Liver-1d-dn	2/310	0,94202098	0,982956709	0	0	0,443409822	0,026483864	FUNDC1;RPS6
Norepinephrine-1.75_mg/kg_in_Saline-Rat-Heart-1d-dn	2/310	0,94202098	0,982826498	0	0	0,443409822	0,026483864	RPL32;SEPP1
Vancomycin-160_mg/kg_in_Saline-Rat-Kidney-5d-dn	2/310	0,94202098	0,982696323	0	0	0,443409822	0,026483864	EEF1B2;RPS20
Sodium_Arsenite-30_mg/kg_in_Saline-Rat-Liver-0.25d-dn	2/311	0,942723743	0,983299192	0	0	0,441984066	0,026069102	RPS19;RPL39
Spironolactone-300_mg/kg_in_CMC-Rat-Heart-3d-dn	2/311	0,942723743	0,983168988	0	0	0,441984066	0,026069102	RPS8;ARL6IP5
Amitraz-75_mg/kg_in_CMC-Rat-Heart-5d-dn	2/312	0,943418424	0,983763207	0	0	0,440567451	0,025661018	ARL6IP5;RPL39
Diclofenac-3.5_mg/kg_in_Corn_Oil-Rat-Liver-1d-up	2/312	0,943418424	0,983632977	0	0	0,440567451	0,025661018	RPS19;GOLGA7
Lovastatin-450_mg/kg_in_Corn_Oil-Rat-Liver-0.25d-dn	2/312	0,943418424	0,98350278	0	0	0,440567451	0,025661018	RPL39;TPT1
N-Nitrosodiethylamine-34_mg/kg_in_Saline-Rat-Liver-3d-up	2/312	0,943418424	0,983372619	0	0	0,440567451	0,025661018	RPSA;SEPP1
6-Methoxy-2-Naphthylacetic_Acid-360_mg/kg_in_Saline-Rat-Kidney-3d-dn	2/313	0,944105109	0,983958163	0	0	0,439159887	0,0252595	EEF1B2;RPS8

Chloroform-600_mg/kg_in_Corn_Oil-Rat-Liver-3d-dn	2/313	0,944105109	0,983827975	0	0	0,439159887	0,0252595	C1QA;RPL35A
Cisplatin-1.17_mg/kg_in_Saline-Rat-Liver-5d-up	2/313	0,944105109	0,983697822	0	0	0,439159887	0,0252595	RPS19;SEPP1
Gemfibrozil-700_mg/kg_in_Corn_Oil-Rat-Liver-0.25d-dn	2/313	0,944105109	0,983567703	0	0	0,439159887	0,0252595	C1QA;RPL39
Isoniazid-79_mg/kg_in_Water-Rat-Liver-1d-dn	2/313	0,944105109	0,983437619	0	0	0,439159887	0,0252595	C1QA;TPT1
Lead_(II)_Acetate-200_mg/kg_in_Saline-Rat-Liver-1d-up	2/313	0,944105109	0,983307569	0	0	0,439159887	0,0252595	C1QA;RPL10A
Urethane-453_mg/kg_in_Water-Rat-Bone_marrow-5d-dn	2/313	0,944105109	0,983177553	0	0	0,439159887	0,0252595	RPS6;RPL39
Tacrolimus-134_mg/kg_in_CMC-Rat-Heart-3d-dn	2/314	0,944783882	0,983754343	0	0	0,437761289	0,024864436	ARL6IP5;RPSA
Valproic_Acid-1500_mg/kg_in_Water-Rat-Heart-3d-up	2/314	0,944783882	0,983624303	0	0	0,437761289	0,024864436	RPS19;SEPP1
123-Trichloropropane-108_mg/kg_in_CMC-Rat-Kidney-3d-dn	2/315	0,945454827	0,984192733	0	0	0,43637157	0,024475719	ARL2BP;CLP1
Acetaminophen-972_mg/kg_in_Corn_Oil-Rat-Liver-5d-dn	2/315	0,945454827	0,984062669	0	0	0,43637157	0,024475719	C1QA;SEPP1
Bisphenol_A-100_mg/kg_in_Corn_Oil-Rat-Spleen-0.25d-dn	2/315	0,945454827	0,98393264	0	0	0,43637157	0,024475719	RPS17;RPL39
Phenelzine-27_mg/kg_in_Water-Rat-Brain-3d-dn	2/315	0,945454827	0,983802645	0	0	0,43637157	0,024475719	SEPP1;GOLGA7
Thalidomide-113_mg/kg_in_CMC-Rat-Kidney-1d-dn	2/315	0,945454827	0,983672684	0	0	0,43637157	0,024475719	SEPP1;TPT1
Thioguanine-12_mg/kg_in_Corn_Oil-Rat-Spleen-0.25d-dn	2/315	0,945454827	0,983542757	0	0	0,43637157	0,024475719	RPS17;RPS19
Cyclosporin_A-70_mg/kg_in_Corn_Oil-Rat-Spleen-3d-dn	2/316	0,946118027	0,984102692	0	0	0,434990648	0,024093242	RPS19;TPT1
Daunorubicin-3.25_mg/kg_in_Saline-Rat-Kidney-3d-up	2/316	0,946118027	0,983972743	0	0	0,434990648	0,024093242	RPL10A;SEPP1
Epirubicin-2.7_mg/kg_in_Saline-Rat-Liver-1d-up	2/316	0,946118027	0,983842828	0	0	0,434990648	0,024093242	RPSA;SEPP1
Famciclovir-1200_mg/kg_in_Saline-Rat-Liver-0.25d-dn	2/316	0,946118027	0,983712948	0	0	0,434990648	0,024093242	RPS19;RPL39
Isoeugenol-1560_mg/kg_in_CMC-Rat-Liver-3d-dn	2/316	0,946118027	0,983583102	0	0	0,434990648	0,024093242	C1QA;RPL32
1-Naphthyl_Isothiocyanate-30_mg/kg_in_Corn_Oil-Rat-Liver-1d-dn	2/317	0,946773564	0,984134696	0	0	0,433618437	0,0237169	RPL10A;TPT1
Cetylpyridinium_Bromide-119_mg/kg_in_Water-Rat-Liver-1d-up	2/317	0,946773564	0,984004829	0	0	0,433618437	0,0237169	RPS20;RPL39
Doxycycline-14_mg/kg_in_Water-Rat-Liver-1d-up	2/317	0,946773564	0,983874996	0	0	0,433618437	0,0237169	RPS17;RPL39
Neomycin-56_mg/kg_in_Corn_Oil-Rat-Liver-0.25d-up	2/318	0,947421521	0,984418456	0	0	0,432254857	0,023346592	RPS6;SEPP1
Enalapril-1500_mg/kg_in_CMC-Rat-Kidney-1d-dn	2/319	0,948061977	0,984953981	0	0	0,430899827	0,022982216	RPS17;SEPP1
Ferrocene-330_mg/kg_in_CMC-Rat-Kidney-5d-dn	2/319	0,948061977	0,984824074	0	0	0,430899827	0,022982216	RPS19;RPS6
Mesna-755_mg/kg_in_Saline-Rat-Kidney-5d-dn	2/319	0,948061977	0,984694201	0	0	0,430899827	0,022982216	EEF1B2;TPT1
Cadmium_Acetate-167_mg/kg_in_Water-Rat-Kidney-5d-dn	2/320	0,948695013	0,985221772	0	0	0,429553265	0,022623672	EEF1B2;RPS8
Cyclosporin_A-350_mg/kg_in_Corn_Oil-Rat-Spleen-3d-dn	2/320	0,948695013	0,985091881	0	0	0,429553265	0,022623672	RPS19;TPT1
Carmustine-16_mg/kg_in_Corn_Oil-Rat-Liver-0.25d-up	2/321	0,949320708	0,985611639	0	0	0,428215092	0,022270865	RPS6;RPL10A
Hydrocortisone-56_mg/kg_in_Corn_Oil-Rat-Heart-3d-up	2/321	0,949320708	0,985481731	0	0	0,428215092	0,022270865	ARL6IP5;SEPP1
Sulindac-132_mg/kg_in_Corn_Oil-Rat-Liver-5d-dn	2/321	0,949320708	0,985351857	0	0	0,428215092	0,022270865	SEPP1;TPT1
Carvedilol-2000_mg/kg_in_Corn_Oil-Rat-Heart-3d-dn	2/322	0,949939141	0,985863839	0	0	0,426885232	0,021923698	ARL2BP;COL5A3
Dexamethasone-150_mg/kg_in_Corn_Oil-Rat-Liver-1d-up	2/322	0,949939141	0,985733949	0	0	0,426885232	0,021923698	RPS19;TPT1
Indomethacin-12_mg/kg_in_Water-Rat-Liver-3d-dn	2/323	0,950550389	0,986238291	0	0	0,425563606	0,021582077	SEPP1;TPT1
L-Buthionine_Sulfoximine-100000_uM_in_DMSO-Rat-Primary_rat_hepatocytes-1d-dn	2/323	0,950550389	0,986108386	0	0	0,425563606	0,021582077	RPS19;SEPP1
Atorvastatin-300_mg/kg_in_Corn_Oil-Rat-Liver-5d-dn	2/324	0,95115453	0,986605173	0	0	0,424250138	0,021245911	CLP1;SEPP1
Clemastine-2500_mg/kg_in_CMC-Rat-Heart-5d-dn	2/324	0,95115453	0,986475254	0	0	0,424250138	0,021245911	COL5A3;ARL6IP5
Diethylstilbestrol-280_mg/kg_in_Corn_Oil-Rat-Liver-5d-dn	2/324	0,95115453	0,98634537	0	0	0,424250138	0,021245911	C1QA;SEPP1
Indomethacin-9.6_mg/kg_in_Water-Rat-Heart-5d-dn	2/324	0,95115453	0,986215519	0	0	0,424250138	0,021245911	COL5A3;TPT1
Phenothiazine-386_mg/kg_in_Corn_Oil-Rat-Liver-3d-up	2/324	0,95115453	0,986085702	0	0	0,424250138	0,021245911	RPS20;SEPP1
Rofecoxib-775_mg/kg_in_Corn_Oil-Rat-Kidney-1d-up	2/324	0,95115453	0,98595592	0	0	0,424250138	0,021245911	RPS19;RAPGEF5
Rifampin-99_mg/kg_in_CMC-Rat-Kidney-1d-dn	2/325	0,951751641	0,986445049	0	0	0,422944753	0,020915108	RPS6;SEPP1
4-Octylphenol-450_mg/kg_in_Corn_Oil-Rat-Liver-1d-dn	2/326	0,952341797	0,986926841	0	0	0,421647376	0,02058958	RPS6;SEPP1
Allyl_Alcohol-25_mg/kg_in_Saline-Rat-Liver-0.25d-dn	2/326	0,952341797	0,986796999	0	0	0,421647376	0,02058958	RPS20;RPL10A
Atropine-2.3_mg/kg_in_Water-Rat-Liver-1d-up	2/326	0,952341797	0,986667192	0	0	0,421647376	0,02058958	RPL39;TPT1
Disulfiram-100_mg/kg_in_Water-Rat-Liver-0.25d-up	2/326	0,952341797	0,986537418	0	0	0,421647376	0,02058958	RPL10A;SEPP1
Lead(IV)_Acetate-200_mg/kg_in_Saline-Rat-Liver-3d-dn	2/326	0,952341797	0,986407679	0	0	0,421647376	0,02058958	RPS19;ARL2BP
Sulindac-132_mg/kg_in_Corn_Oil-Rat-Heart-5d-dn	2/328	0,953501542	0,987479046	0	0	0,419076356	0,019954001	COL5A3;SEPP1
Lovastatin-450_mg/kg_in_Corn_Oil-Rat-Liver-3d-dn	2/329	0,954071281	0,98793918	0	0	0,417802567	0,019643778	CLP1;SEPP1
Clofibrate-500_mg/kg_in_Corn_Oil-Rat-Liver-0.25d-dn	2/330	0,95463436	0,988392299	0	0	0,416536499	0,01933849	CLP1;RPS8
Esmolol-27_mg/kg_in_Saline-Rat-Heart-3d-up	2/330	0,95463436	0,988262384	0	0	0,416536499	0,01933849	RPS20;TPT1
Diclofenac-3.5_mg/kg_in_Corn_Oil-Rat-Liver-5d-up	2/331	0,955190853	0,988708523	0	0	0,415278081	0,019038055	SEPP1;GOLGA7
Bithionol-333_mg/kg_in_Corn_Oil-Rat-Liver-0.25d-dn	1/211	0,955356024	0,988749546	0	0	0,325727594	0,014876373	RPS20
Phenobarbital-80_mg/kg_in_Water-Rat-Liver-3d-up	2/332	0,95574083	0,989017839	0	0	0,414027243	0,018742393	RPS8;SEPP1
Ethinylestradiol-10_mg/kg_in_Corn_Oil-Rat-Liver-3d-dn	2/333	0,956284363	0,989450295	0	0	0,412783918	0,018451424	RPS17;RPS3A
Foscarnet-350_mg/kg_in_Saline-Rat-Kidney-1d-up	2/333	0,956284363	0,989320326	0	0	0,412783918	0,018451424	EEF1B2;RPS8
Methyl_Salicylate-444_mg/kg_in_Corn_Oil-Rat-Kidney-5d-up	2/333	0,956284363	0,989190392	0	0	0,412783918	0,018451424	RPS19;TPT1
Nisoldipine-1125_mg/kg_in_Water-Rat-Liver-3d-up	2/333	0,956284363	0,989060492	0	0	0,412783918	0,018451424	RPS19;RPL39
Torsemide-3_mg/kg_in_Corn_Oil-Rat-Liver-1d-dn	2/333	0,956284363	0,988930625	0	0	0,412783918	0,018451424	RPS17;RPSA
Cytarabine-487_mg/kg_in_Saline-Rat-Liver-3d-dn	1/214	0,957296693	0,989847545	0	0	0,321161319	0,014016094	SEPP1
Alendronic_Acid-138_mg/kg_in_CMC-Rat-Heart-5d-dn	2/335	0,957352377	0,98977518	0	0	0,410319536	0,017883262	FEM1B;SEPP1
Lead(IV)_Acetate-600_mg/kg_in_Saline-Rat-Liver-5d-up	2/336	0,957876996	0,990187587	0	0	0,409098347	0,017605918	RPS20;RPL39
Modafinil-325_mg/kg_in_Saline-Rat-Liver-0.25d-dn	2/337	0,958395448	0,990593511	0	0	0,407884406	0,017332967	RPS20;TPT1
Meloxicam-33_mg/kg_in_Corn_Oil-Rat-Kidney-5d-dn	2/339	0,95941412	0,991516285	0	0	0,405478008	0,016799956	ARL2BP;CLP1
Neomycin-56_mg/kg_in_Corn_Oil-Rat-Liver-1d-up	2/341	0,960408925	0,992414155	0	0	0,403099838	0,01628367	C1QA;RPL39
Sodium_Nitroprusside-6_mg/kg_in_Saline-Rat-Heart-3d-up	1/220	0,960929398	0,992821716	0	0	0,312402374	0,01245059	RAPGEF5
Ethanol-3000_mg/kg_in_Saline-Rat-Liver-1d-up	2/343	0,961380385	0,993157386	0	0	0,400749401	0,015783566	C1QA;SEPP1
Raloxifene-5_mg/kg_in_Corn_Oil-Rat-Liver-5d-dn	2/343	0,961380385	0,993027136	0	0	0,400749401	0,015783566	RPS8;SEPP1
NN-Dimethylformamide-1400_mg/kg_in_Saline-Rat-Bone_marrow-3d-dn	1/221	0,961504165	0,993024758	0	0	0,310988789	0,012208295	EEF1B2
N-Nitrosodiethylamine-1.67_mg/kg_in_Saline-Rat-Liver-0.25d-dn	1/223	0,962628541	0,994055643	0	0	0,308199652	0,011738608	RPL39
Leflunomide-60_mg/kg_in_Corn_Oil-Rat-Liver-1d-up	1/227	0,964780042	0,996146777	0	0	0,302768821	0,010855818	RPS19
Busulfan-9_mg/kg_in_Corn_Oil-Rat-Liver-0.25d-dn	1/231	0,966808073	0,998109894	0	0	0,297526071	0,010043076	RPS8
Oxcarbazepine-1600_mg/kg_in_CMC-Rat-Brain-3d-up	1/231	0,966808073	0,99797908	0	0	0,297526071	0,010043076	RPS6
Cisplatin-2_mg/kg_in_Saline-Rat-Kidney-1d-dn	1/233	0,96777801	0,998849378	0	0	0,294972199	0,009661091	SEPP1
Allylamine-75_mg/kg_in_Water-Rat-Heart-3d-up	1/236	0,969180201	1	0	0	0,291222552	0,00911664	SEPP1
Indomethacin-9.6_mg/kg_in_Water-Rat-Liver-3d-dn	1/239	0,97052157	1	0	0	0,287567039	0,008604481	RPSA
Moclobemide-266_mg/kg_in_Water-Rat-Brain-3d-up	1/239	0,97052157	1	0	0	0,287567039	0,008604481	TPT1
Haloperidol-21_mg/kg_in_Corn_Oil-Rat-Heart-5d-dn	1/241	0,971383328	1	0	0	0,285180591	0,008279965	RPS17
Levosulpiride-1200_mg/kg_in_CMC-Rat-Heart-1d-up	2/367	0,971393856	1	0	0	0,374542356	0,010870445	RPS8;SEPP1
Doxorubicin-3_mg/kg_in_Saline-Rat-Heart-3d-dn	1/242	0,971804745	1	0	0	0,284002158	0,008122568	COL5A3
Atropine-94_mg/kg_in_Water-Rat-Heart-5d-up	1/247	0,973820837	1	0	0	0,278253127	0,007381482	RPS17
Trimethadione-535_mg/kg_in_Water-Rat-Kidney-3d-up	2/376	0,974462331	1	0	0	0,365577246	0,00945727	CLP1;SEPP1
Vinorelbine-1.5_mg/kg_in_Saline-Rat-Heart-1d-up	1/249	0,974586445	1	0	0	0,276018162	0,007105275	CLP1
Gentamicin-267_mg/kg_in_Corn_Oil-Rat-Kidney-1d-dn	1/250	0,974960839	1	0	0	0,274914089	0,006971264	ARL2BP
Oxybutynin-230_mg/kg_in_Water-Rat-Heart-3d-dn	1/250	0,974960839	1	0	0	0,274914089	0,006971264	RPS8
Lead(IV)_Acetate-600_mg/kg_in_Saline-Rat-Kidney-3d-dn	1/253	0,976051357	1	0	0	0,271654238	0,006584919	SEPP1
Sulindac-23_mg/kg_in_Corn_Oil-Rat-Kidney-3d-up	1/254	0,976404239	1	0	0	0,270584734	0,006461184	GOLGA7
Atorvastatin-250_uM_in_DMSO-Rat-Primary_rat_hepatocytes-1d-up	1/255	0,976751939	1	0	0	0,269523617	0,006339886	RPS20
Mesna-755_mg/kg_in_Saline-Rat-Kidney-1d-dn	1/256	0,977094531	1	0	0	0,26847079	0,006220972	TPT1
Insulin-0.56_mg/kg_in_Saline-Rat-Liver-3d-dn	1/257	0,977432092	1	0	0	0,267426157	0,006104393	RPS8

N-Nitrosodiethylamine-1.67_mg/kg_in_Saline-Rat-Liver-1d-dn	1/257	0,977432092	1	0	0	0,267426157	0,006104393	RPL10A
Naproxen-134_mg/kg_in_Corn_Oil-Rat-Liver-0.25d-dn	1/258	0,977764694	1	0	0	0,266389621	0,0059901	RPS17
Sodium_Selenate-1.6_mg/kg_in_Water-Rat-Kidney-1d-up	2/387	0,97778352	1	0	0	0,355186162	0,007979961	SEPP1;TPT1
Prednisolone-184_mg/kg_in_Water-Rat-Heart-3d-up	1/260	0,978415312	1	0	0	0,264340471	0,005768185	ARL6IP5
44'-Methylenedianiline-81_mg/kg_in_Corn_Oil-Rat-Kidney-5d-up	1/261	0,97873347	1	0	0	0,263327672	0,005660471	RPS5
Dexfenfluramine-29_mg/kg_in_Water-Rat-Heart-5d-up	1/261	0,97873347	1	0	0	0,263327672	0,005660471	SEPP1
Zileuton-450_mg/kg_in_Corn_Oil-Rat-Kidney-0.25d-dn	1/261	0,97873347	1	0	0	0,263327672	0,005660471	RPL35A
Azasetron-580_mg/kg_in_CMC-Rat-Intestine-1d-up	1/263	0,979355832	1	0	0	0,26132518	0,005451305	RPS8
Tamoxifen-32_mg/kg_in_Corn_Oil-Rat-Liver-3d-dn	1/263	0,979355832	1	0	0	0,26132518	0,005451305	SEPP1
Tenidap-75_mg/kg_in_Corn_Oil-Rat-Kidney-0.25d-dn	1/263	0,979355832	1	0	0	0,26132518	0,005451305	SEPP1
Trimethadione-535_mg/kg_in_Water-Rat-Heart-1d-up	1/263	0,979355832	1	0	0	0,26132518	0,005451305	SEPP1
Fluvastatin-94_mg/kg_in_Corn_Oil-Rat-Liver-3d-dn	1/266	0,9802555	1	0	0	0,258377904	0,005152579	SEPP1
3-Acetamidophenol-700_mg/kg_in_Corn_Oil-Rat-Kidney-5d-up	1/267	0,980546619	1	0	0	0,257410196	0,005056846	CLP1
Irinotecan-5_mg/kg_in_Saline-Rat-Kidney-1d-up	1/267	0,980546619	1	0	0	0,257410196	0,005056846	SEPP1
Mefenamic_Acid-93_mg/kg_in_Corn_Oil-Rat-Kidney-5d-up	1/267	0,980546619	1	0	0	0,257410196	0,005056846	ARL2BP
CerIVastatin-7_mg/kg_in_Corn_Oil-Rat-Kidney-1d-up	1/268	0,98083346	1	0	0	0,25644971	0,004962969	CLP1
6-Mercaptopurine-25_mg/kg_in_Corn_Oil-Rat-Kidney-5d-up	1/269	0,981116084	1	0	0	0,255496366	0,004870909	CLP1
Azathioprine-54_mg/kg_in_Water-Rat-Liver-3d-dn	1/269	0,981116084	1	0	0	0,255496366	0,004870909	RPSA
Gatifloxacin-770_mg/kg_in_Corn_Oil-Rat-Heart-5d-up	1/269	0,981116084	1	0	0	0,255496366	0,004870909	CLP1
Benzyl_Acetate-1868_mg/kg_in_CMC-Rat-Kidney-3d-dn	1/270	0,981394555	1	0	0	0,254550083	0,00478063	ARL2BP
CerIVastatin-7_mg/kg_in_Corn_Oil-Rat-Kidney-3d-up	1/270	0,981394555	1	0	0	0,254550083	0,00478063	CLP1
Atorvastatin-2.5_mg/kg_in_Corn_Oil-Rat-Liver-1d-dn	1/271	0,981668933	1	0	0	0,253610784	0,004692094	SEPP1
Bithionol-59_mg/kg_in_Corn_Oil-Rat-Liver-0.25d-dn	1/271	0,981668933	1	0	0	0,253610784	0,004692094	CLP1
Hydralazine-280_mg/kg_in_Water-Rat-Liver-1d-dn	1/271	0,981668933	1	0	0	0,253610784	0,004692094	SEPP1
Acetazolamide-47_mg/kg_in_Corn_Oil-Rat-Liver-3d-dn	1/272	0,981939277	1	0	0	0,252678391	0,004605268	SEPP1
Bromisovalum-250_mg/kg_in_Corn_Oil-Rat-Kidney-1d-up	1/272	0,981939277	1	0	0	0,252678391	0,004605268	CLP1
Dexamethasone-1_mg/kg_in_Corn_Oil-Rat-Liver-5d-up	1/272	0,981939277	1	0	0	0,252678391	0,004605268	RPL39
Lead_(II)_Acetate-600_mg/kg_in_Saline-Rat-Kidney-5d-dn	1/272	0,981939277	1	0	0	0,252678391	0,004605268	ARL2BP
Tacrolimus-134_mg/kg_in_CMC-Rat-Heart-1d-dn	1/272	0,981939277	1	0	0	0,252678391	0,004605268	TPT1
3-Acetamidophenol-700_mg/kg_in_Corn_Oil-Rat-Kidney-1d-up	1/273	0,982205648	1	0	0	0,251752829	0,004520115	CLP1
Bromisovalum-250_mg/kg_in_Corn_Oil-Rat-Heart-5d-up	1/273	0,982205648	1	0	0	0,251752829	0,004520115	CLP1
Clomipramine-115_mg/kg_in_Water-Rat-Heart-5d-up	1/273	0,982205648	1	0	0	0,251752829	0,004520115	RPL39
Dexfenfluramine-29_mg/kg_in_Water-Rat-Heart-1d-up	1/273	0,982205648	1	0	0	0,251752829	0,004520115	SEPP1
Foscarnet-350_mg/kg_in_Saline-Rat-Kidney-3d-up	1/273	0,982205648	1	0	0	0,251752829	0,004520115	CLP1
Carbon_Tetrachloride-3178_mg/kg_in_Saline-Rat-Liver-1d-dn	1/274	0,982468102	1	0	0	0,250834023	0,004436602	SEPP1
Clotrimazole-52_mg/kg_in_Corn_Oil-Rat-Liver-3d-up	1/274	0,982468102	1	0	0	0,250834023	0,004436602	RPSA
Cadmium_Acetate-167_mg/kg_in_Water-Rat-Kidney-3d-dn	1/275	0,982726698	1	0	0	0,249921899	0,004354696	SEPP1
Cerivastatin-7_mg/kg_in_Corn_Oil-Rat-Skeletal_muscle-3d-up	1/275	0,982726698	1	0	0	0,249921899	0,004354696	TPT1
Cyclopropane_Carboxylic_Acid-375_mg/kg_in_Water-Rat-Liver-1d-up	1/275	0,982726698	1	0	0	0,249921899	0,004354696	CXCL11
Neostigmine_Bromide-11_mg/kg_in_Water-Rat-Liver-1d-dn	1/275	0,982726698	1	0	0	0,249921899	0,004354696	CLP1
Olanzapine-23_mg/kg_in_CMC-Rat-Liver-5d-dn	1/275	0,982726698	1	0	0	0,249921899	0,004354696	C1QA
Oxymetazoline-0.5_mg/kg_in_Water-Rat-Heart-5d-up	1/275	0,982726698	1	0	0	0,249921899	0,004354696	CLP1
Artemether-74_mg/kg_in_Corn_Oil-Rat-Liver-5d-dn	1/276	0,982981492	1	0	0	0,249016385	0,004274363	FEM1B
Isotretinoin-13_mg/kg_in_Corn_Oil-Rat-Liver-0.25d-up	1/276	0,982981492	1	0	0	0,249016385	0,004274363	RPS19
Lipopolysaccharide_E._Coli_O55:B5-1.25_mg/kg_in_Saline-Rat-Liver-5d-dn	1/276	0,982981492	1	0	0	0,249016385	0,004274363	TPT1
Atorvastatin-2.5_mg/kg_in_Corn_Oil-Rat-Kidney-3d-up	1/278	0,983479897	1	0	0	0,2472249	0,004118293	ARL2BP
Beta-Estradiol-150_mg/kg_in_Corn_Oil-Rat-Liver-5d-dn	1/278	0,983479897	1	0	0	0,2472249	0,004118293	C1QA
Diclofenac-10_mg/kg_in_Corn_Oil-Rat-Kidney-5d-dn	1/278	0,983479897	1	0	0	0,2472249	0,004118293	RPL10A
Diphenhydramine-100_mg/kg_in_Water-Rat-Heart-3d-dn	1/279	0,983723616	1	0	0	0,24633879	0,004042493	R3HCC1
Ergocalciferol-15_mg/kg_in_CMC-Rat-Heart-5d-up	1/279	0,983723616	1	0	0	0,24633879	0,004042493	RAPGEF5
Rapamycin-60_mg/kg_in_CMC-Rat-Kidney-3d-dn	1/279	0,983723616	1	0	0	0,24633879	0,004042493	SEPP1
BW-723C86-50_mg/kg_in_Saline-Rat-Heart-5d-dn	1/280	0,983963751	1	0	0	0,245459008	0,003968145	COL5A3
Pralidoxime_Chloride-48_mg/kg_in_Saline-Rat-Liver-1d-up	1/280	0,983963751	1	0	0	0,245459008	0,003968145	C1QA
Sumatriptan-1100_mg/kg_in_Water-Rat-Brain-3d-dn	1/280	0,983963751	1	0	0	0,245459008	0,003968145	ARL2BP
Atropine-94_mg/kg_in_Water-Rat-Liver-1d-dn	1/281	0,984200355	1	0	0	0,244585489	0,003895217	TPT1
Betamethasone-79_mg/kg_in_Corn_Oil-Rat-Liver-3d-up	1/281	0,984200355	1	0	0	0,244585489	0,003895217	CLP1
Bithionol-333_mg/kg_in_Corn_Oil-Rat-Spleen-5d-dn	1/281	0,984200355	1	0	0	0,244585489	0,003895217	RPS19
Ethosuximide-1200_mg/kg_in_Water-Rat-Heart-3d-up	1/281	0,984200355	1	0	0	0,244585489	0,003895217	RAPGEF5
Glyburide-1500_mg/kg_in_CMC-Rat-Heart-1d-up	1/281	0,984200355	1	0	0	0,244585489	0,003895217	RPS19
Letrozole-250_mg/kg_in_Corn_Oil-Rat-Liver-1d-up	1/282	0,984433479	1	0	0	0,243718164	0,003823683	SEPP1
Spironolactone-300_mg/kg_in_CMC-Rat-Liver-1d-up	1/282	0,984433479	1	0	0	0,243718164	0,003823683	TPT1
Streptomycin-120_mg/kg_in_Saline-Rat-Kidney-3d-up	1/282	0,984433479	1	0	0	0,243718164	0,003823683	RAPGEF5
Verapamil-108_mg/kg_in_Water-Rat-Heart-1d-dn	1/282	0,984433479	1	0	0	0,243718164	0,003823683	SEPP1
Calcitriol-0.04_mg/kg_in_CMC-Rat-Kidney-5d-dn	1/283	0,984663174	1	0	0	0,242856969	0,003753513	SEPP1
Loperamide-47_mg/kg_in_CMC-Rat-Intestine-3d-up	1/284	0,984889491	1	0	0	0,242001839	0,00368468	RPL39
Nevirapine-200_mg/kg_in_Saline-Rat-Liver-3d-up	1/284	0,984889491	1	0	0	0,242001839	0,00368468	RPSA
4-Methylpyrazole-155_mg/kg_in_Saline-Rat-Kidney-5d-up	1/285	0,985112479	1	0	0	0,24115271	0,003617159	RPSA
Thioguanine-24_mg/kg_in_Corn_Oil-Rat-Spleen-5d-dn	1/285	0,985112479	1	0	0	0,24115271	0,003617159	RPS19
Citalopram-40_mg/kg_in_Corn_Oil-Rat-Liver-5d-dn	1/286	0,985332187	1	0	0	0,240309519	0,003550921	SEPP1
Methapyrilene-100_mg/kg_in_CMC-Rat-Liver-1d-dn	1/286	0,985332187	1	0	0	0,240309519	0,003550921	CLP1
1-Naphthyl_Isothiocyanate-60_mg/kg_in_Corn_Oil-Rat-Liver-0.25d-dn	1/287	0,985548662	1	0	0	0,239472203	0,003485943	TPT1
Estriol-313_mg/kg_in_CMC-Rat-Liver-5d-dn	1/287	0,985548662	1	0	0	0,239472203	0,003485943	C1QA
Nortriptyline-63_mg/kg_in_Water-Rat-Heart-5d-up	1/287	0,985548662	1	0	0	0,239472203	0,003485943	RPL39
Aspirin-35_mg/kg_in_Corn_Oil-Rat-Liver-3d-dn	1/288	0,985761954	1	0	0	0,238640703	0,003422198	RPL39
Beta-Estradiol-0.3_mg/kg_in_Corn_Oil-Rat-Liver-1d-dn	1/288	0,985761954	1	0	0	0,238640703	0,003422198	C1QA
Betamethasone-79_mg/kg_in_Corn_Oil-Rat-Liver-5d-up	1/288	0,985761954	1	0	0	0,238640703	0,003422198	CLP1
Dibromochloromethane-325_mg/kg_in_CMC-Rat-Kidney-3d-dn	1/288	0,985761954	1	0	0	0,238640703	0,003422198	ARL2BP
Ethosuximide-1200_mg/kg_in_Water-Rat-Heart-1d-up	1/288	0,985761954	1	0	0	0,238640703	0,003422198	RPS6
2378-Tetrachlorodibenzo-P-Dioxin-0.0007_mg/kg_in_CMC-Rat-Liver-91d-up	1/289	0,985972107	1	0	0	0,237814956	0,003359663	SEPP1
Hydrazine-45_mg/kg_in_Water-Rat-Liver-5d-dn	1/289	0,985972107	1	0	0	0,237814956	0,003359663	EEF1B2
Mitomycin_C-0.5_mg/kg_in_Saline-Rat-Bone_marrow-0.25d-up	1/289	0,985972107	1	0	0	0,237814956	0,003359663	RPL35A
Streptomycin-120_mg/kg_in_Saline-Rat-Kidney-1d-up	1/289	0,985972107	1	0	0	0,237814956	0,003359663	CLP1
4-Nitrobenzoic_Acid-1470_mg/kg_in_CMC-Rat-Kidney-1d-dn	1/290	0,986179168	1	0	0	0,236994905	0,003298312	SEPP1
Carbon_Tetrachloride-3178_mg/kg_in_Saline-Rat-Liver-5d-dn	1/290	0,986179168	1	0	0	0,236994905	0,003298312	SEPP1
Fludrocortisone_Acetate-125_mg/kg_in_CMC-Rat-Liver-5d-up	1/290	0,986179168	1	0	0	0,236994905	0,003298312	RPL39
Ketoconazole-227_mg/kg_in_Corn_Oil-Rat-Liver-1d-dn	1/290	0,986179168	1	0	0	0,236994905	0,003298312	TPT1
Sodium_Arsenite-10_mg/kg_in_Saline-Rat-Liver-1d-up	1/290	0,986179168	1	0	0	0,236994905	0,003298312	RPS17
Thioguanine-12_mg/kg_in_Corn_Oil-Rat-Liver-5d-dn	1/290	0,986179168	1	0	0	0,236994905	0,003298312	RPL10A
Thioguanine-24_mg/kg_in_Corn_Oil-Rat-Liver-5d-dn	1/290	0,986179168	1	0	0	0,236994905	0,003298312	SEPP1
Citalopram-90_mg/kg_in_Corn_Oil-Rat-Liver-0.25d-dn	1/291	0,986383183	1	0	0	0,236180489	0,003238123	RPL39

Pergolide-1.1_mg/kg_in_CMC-Rat-Liver-5d-dn	1/291	0,986383183	1	0	0	0,236180489	0,003238123	EEF1B2
Phenobarbital-25_mg/kg_in_Water-Rat-Liver-1d-dn	1/291	0,986383183	1	0	0	0,236180489	0,003238123	C1QA
Tolazamide-1500_mg/kg_in_CMC-Rat-Kidney-5d-dn	1/291	0,986383183	1	0	0	0,236180489	0,003238123	RPS6
Aminosalicylic_Acid-2337_mg/kg_in_CMC-Rat-Liver-5d-dn	1/293	0,98678225	1	0	0	0,234568336	0,003121139	C1QA
Carbon_Tetrachloride-1175_mg/kg_in_Corn_Oil-Rat-Liver-0.25d-dn	1/293	0,98678225	1	0	0	0,234568336	0,003121139	SEPP1
Hydroxyurea-400_mg/kg_in_Saline-Rat-Bone_marrow-5d-dn	1/293	0,98678225	1	0	0	0,234568336	0,003121139	DUSP14
Thioacetamide-200_mg/kg_in_Saline-Rat-Liver-1d-dn	1/293	0,98678225	1	0	0	0,234568336	0,003121139	SEPP1
Doxofylline-483_mg/kg_in_Water-Rat-Heart-1d-dn	1/294	0,98697739	1	0	0	0,233770484	0,003064298	EEF1B2
Indomethacin-4.5_mg/kg_in_Water-Rat-Liver-1d-dn	1/294	0,98697739	1	0	0	0,233770484	0,003064298	SEPP1
Manganese_(II)_Chloride-700_mg/kg_in_Water-Rat-Liver-5d-up	1/294	0,98697739	1	0	0	0,233770484	0,003064298	RPL10A
Tacrolimus-134_mg/kg_in_CMC-Rat-Heart-5d-dn	1/294	0,98697739	1	0	0	0,233770484	0,003064298	ARL6IP5
Vinorelbine-1.5_mg/kg_in_Saline-Rat-Heart-5d-up	1/294	0,98697739	1	0	0	0,233770484	0,003064298	CLP1
Amiodarone-147_mg/kg_in_CMC-Rat-Liver-5d-up	1/295	0,987169659	1	0	0	0,232978042	0,00300853	RPSA
Dibromochloromethane-325_mg/kg_in_CMC-Rat-Kidney-5d-dn	1/295	0,987169659	1	0	0	0,232978042	0,00300853	SEPP1
Procainamide-755_mg/kg_in_Water-Rat-Heart-1d-dn	1/295	0,987169659	1	0	0	0,232978042	0,00300853	RPL10A
Vinorelbine-1.5_mg/kg_in_Saline-Rat-Heart-3d-up	1/295	0,987169659	1	0	0	0,232978042	0,00300853	CLP1
Aminosalicylic_Acid-2337_mg/kg_in_CMC-Rat-Liver-1d-dn	1/296	0,987359097	1	0	0	0,232190954	0,002953812	SEPP1
Bis(2-Ethylhexyl)Phthalate-1000_mg/kg_in_Corn_Oil-Rat-Liver-3d-up	1/296	0,987359097	1	0	0	0,232190954	0,002953812	RPS20
Cyclosporin_A-350_mg/kg_in_Corn_Oil-Rat-Kidney-5d-dn	1/296	0,987359097	1	0	0	0,232190954	0,002953812	SEPP1
Promethazine-2.3_mg/kg_in_Saline-Rat-Liver-3d-dn	1/296	0,987359097	1	0	0	0,232190954	0,002953812	SEPP1
Rifabutin-1500_mg/kg_in_CMC-Rat-Liver-1d-up	1/296	0,987359097	1	0	0	0,232190954	0,002953812	SEPP1
Roflumilast-17_mg/kg_in_CMC-Rat-Liver-4d-dn	1/296	0,987359097	1	0	0	0,232190954	0,002953812	SEPP1
SCH-351591-4.6_mg/kg_in_CMC-Rat-Liver-4d-dn	1/296	0,987359097	1	0	0	0,232190954	0,002953812	SEPP1
4-Nitrobenzoic_Acid-1470_mg/kg_in_CMC-Rat-Kidney-5d-dn	1/297	0,987545748	1	0	0	0,231409166	0,002900125	SEPP1
Mitomycin_C-0.5_mg/kg_in_Saline-Rat-Liver-3d-dn	1/297	0,987545748	1	0	0	0,231409166	0,002900125	SEPP1
NN-Dimethylformamide-2100_mg/kg_in_Saline-Rat-Liver-1d-up	1/297	0,987545748	1	0	0	0,231409166	0,002900125	RPSA
Naproxen-10_mg/kg_in_Corn_Oil-Rat-Liver-5d-dn	1/298	0,987729651	1	0	0	0,230632625	0,002847448	RPL32
Carbon_Tetrachloride-400_mg/kg_in_Corn_Oil-Rat-Liver-1d-dn	1/300	0,988089377	1	0	0	0,229095074	0,002745045	TPT1
Cobalt_(II)_Chloride-10_mg/kg_in_Saline-Rat-Heart-14d-dn	1/300	0,988089377	1	0	0	0,229095074	0,002745045	COL5A3
Cyclosporin_A-350_mg/kg_in_Corn_Oil-Rat-Spleen-5d-dn	1/300	0,988089377	1	0	0	0,229095074	0,002745045	RPS6
Cytarabine-487_mg/kg_in_Saline-Rat-Liver-0.25d-dn	1/300	0,988089377	1	0	0	0,229095074	0,002745045	RPS19
Enrofloxacin-2000_mg/kg_in_CMC-Rat-Kidney-5d-dn	1/300	0,988089377	1	0	0	0,229095074	0,002745045	SEPP1
Roflumilast-17_mg/kg_in_CMC-Rat-Liver-3d-dn	1/300	0,988089377	1	0	0	0,229095074	0,002745045	SEPP1
Tranilast-300_mg/kg_in_CMC-Rat-Liver-1d-up	1/300	0,988089377	1	0	0	0,229095074	0,002745045	TPT1
Leflunomide-30_mg/kg_in_Corn_Oil-Rat-Liver-3d-up	1/301	0,988265277	1	0	0	0,228333961	0,002695281	TPT1
Valproic_Acid-1500_mg/kg_in_Water-Rat-Heart-5d-dn	1/301	0,988265277	1	0	0	0,228333961	0,002695281	COL5A3
Benzocaine-1100_mg/kg_in_Corn_Oil-Rat-Liver-0.25d-dn	1/302	0,988438589	1	0	0	0,227577889	0,00264645	ARL6IP5
Cobalt_(II)_Chloride-10_mg/kg_in_Saline-Rat-Kidney-28d-dn	1/302	0,988438589	1	0	0	0,227577889	0,00264645	RPS6
Norethindrone_Acetate-125_mg/kg_in_Corn_Oil-Rat-Liver-1d-dn	1/302	0,988438589	1	0	0	0,227577889	0,00264645	C1QA
Bromodichloromethane-430_mg/kg_in_CMC-Rat-Kidney-1d-dn	1/304	0,988777594	1	0	0	0,226080666	0,002551513	SEPP1
Lead_(II)_Acetate-600_mg/kg_in_Saline-Rat-Spleen-5d-dn	1/304	0,988777594	1	0	0	0,226080666	0,002551513	RPL10A
Sulindac-132_mg/kg_in_Corn_Oil-Rat-Liver-1d-dn	1/304	0,988777594	1	0	0	0,226080666	0,002551513	SEPP1
Clonidine-6_mg/kg_in_Water-Rat-Heart-1d-dn	1/305	0,988943362	1	0	0	0,225339417	0,002505372	EEF1B2
Dicumarol-5_mg/kg_in_Corn_Oil-Rat-Liver-0.25d-dn	1/305	0,988943362	1	0	0	0,225339417	0,002505372	RPS8
Fluvastatin-94_mg/kg_in_Corn_Oil-Rat-Skeletal_muscle-3d-up	1/305	0,988943362	1	0	0	0,225339417	0,002505372	RPS17
Tranilast-1100_mg/kg_in_CMC-Rat-Heart-3d-up	1/305	0,988943362	1	0	0	0,225339417	0,002505372	TPT1
Idarubicin-4_mg/kg_in_Saline-Rat-Heart-28d-dn	1/307	0,989267612	1	0	0	0,223871408	0,002415661	ARL2BP
Pravastatin-1200_mg/kg_in_Corn_Oil-Rat-Liver-3d-dn	1/308	0,989426164	1	0	0	0,223144553	0,002372057	RPL32
Ethinylestradiol-10_mg/kg_in_Corn_Oil-Rat-Liver-0.25d-dn	1/309	0,989582382	1	0	0	0,222422402	0,002329266	TPT1
Norethindrone-75_mg/kg_in_Corn_Oil-Rat-Liver-3d-dn	1/309	0,989582382	1	0	0	0,222422402	0,002329266	C1QA
Catechol-195_mg/kg_in_Saline-Rat-Liver-0.25d-dn	1/310	0,989736298	1	0	0	0,221704911	0,002287271	RPS19
Mitomycin_C-1.7_mg/kg_in_Saline-Rat-Bone_marrow-1d-up	1/310	0,989736298	1	0	0	0,221704911	0,002287271	NKG7
Nimesulide-162_mg/kg_in_Corn_Oil-Rat-Liver-5d-dn	1/310	0,989736298	1	0	0	0,221704911	0,002287271	SEPP1
Secobarbital-70_mg/kg_in_Water-Rat-Liver-3d-dn	1/310	0,989736298	1	0	0	0,221704911	0,002287271	RPL35A
Acyclovir-980_mg/kg_in_Saline-Rat-Liver-0.25d-dn	1/311	0,989887948	1	0	0	0,220992033	0,002246058	SEPP1
Roflumilast-17_mg/kg_in_CMC-Rat-Heart-3d-dn	1/311	0,989887948	1	0	0	0,220992033	0,002246058	COL5A3
Spironolactone-300_mg/kg_in_CMC-Rat-Liver-3d-up	1/311	0,989887948	1	0	0	0,220992033	0,002246058	RPL10A
Indomethacin-10_mg/kg_in_Corn_Oil-Rat-Liver-1d-dn	1/312	0,990037364	1	0	0	0,220283725	0,002205612	GOLGA7
Lipopolysaccharide_E._Coli_O55:B5-1.25_mg/kg_in_Saline-Rat-Liver-0.25d-dn	1/312	0,990037364	1	0	0	0,220283725	0,002205612	SEPP1
Megestrol_Acetate-132_mg/kg_in_CMC-Rat-Liver-3d-up	1/312	0,990037364	1	0	0	0,220283725	0,002205612	RPSA
Ouabain-144_mg/kg_in_CMC-Rat-Heart-5d-up	1/312	0,990037364	0,999940277	0	0	0,220283725	0,002205612	RPL32
Idarubicin-0.625_mg/kg_in_Saline-Rat-Heart-3d-up	1/314	0,990329626	1	0	0	0,218880644	0,002126959	SEPP1
Carboplatin-6_mg/kg_in_Saline-Rat-Liver-0.25d-dn	1/315	0,990472536	1	0	0	0,218185785	0,002088723	SEPP1
Diethylstilbestrol-2.8_mg/kg_in_Corn_Oil-Rat-Liver-5d-dn	1/315	0,990472536	0,999995089	0	0	0,218185785	0,002088723	SEPP1
Diethylstilbestrol-280_mg/kg_in_Corn_Oil-Rat-Liver-0.25d-dn	1/315	0,990472536	0,999866917	0	0	0,218185785	0,002088723	SEPP1
Beta-Estradiol_3-Benzoate-2_mg/kg_in_Corn_Oil-Rat-Liver-3d-dn	1/316	0,99061334	0,9998809	0	0	0,217495324	0,002051197	EEF1B2
Chloroform-600_mg/kg_in_Corn_Oil-Rat-Liver-5d-dn	1/317	0,99075207	0,999892786	0	0	0,216809219	0,002014365	SEPP1
Naproxen-134_mg/kg_in_Corn_Oil-Rat-Liver-1d-dn	1/317	0,99075207	0,999764677	0	0	0,216809219	0,002014365	SEPP1
Thiabendazole-10_mg/kg_in_Corn_Oil-Rat-Liver-1d-dn	1/317	0,99075207	0,9996366	0	0	0,216809219	0,002014365	RPS8
Pramoxine-526_mg/kg_in_Corn_Oil-Rat-Liver-1d-dn	1/318	0,990888755	0,99964645	0	0	0,216127429	0,001978216	RPS6
Modafinil-17.5_mg/kg_in_Saline-Rat-Liver-3d-dn	1/319	0,991023427	0,999654267	0	0	0,215449913	0,001942734	C1QA
Carbon_Tetrachloride-400_mg/kg_in_Corn_Oil-Rat-Liver-3d-dn	1/320	0,991156115	0,999660079	0	0	0,214776632	0,001907909	TPT1
Ethinylestradiol-10_mg/kg_in_Corn_Oil-Rat-Liver-5d-dn	1/320	0,991156115	0,999532082	0	0	0,214776632	0,001907909	C1QA
Sulfaphenazole-1695_mg/kg_in_Water-Rat-Liver-5d-up	1/321	0,991286847	0,999535937	0	0	0,214107546	0,001873727	TPT1
Mitomycin_C-1.7_mg/kg_in_Saline-Rat-Bone_marrow-3d-up	1/322	0,991415652	0,999537849	0	0	0,213442616	0,001840175	SEPP1
Fenofibrate-430_mg/kg_in_Corn_Oil-Rat-Skeletal_muscle-5d-dn	1/326	0,991912168	0,999910436	0	0	0,210823688	0,001712039	PDRG1
Modafinil-17.5_mg/kg_in_Saline-Rat-Liver-0.25d-dn	1/329	0,992265672	1	0	0	0,208901284	0,001621992	RPS20
Norethindrone-375_mg/kg_in_Corn_Oil-Rat-Liver-0.25d-dn	1/336	0,993031755	1	0	0	0,204549174	0,001430338	TPT1
Prochlorperazine-85_mg/kg_in_CMC-Rat-Heart-3d-dn	1/336	0,993031755	1	0	0	0,204549174	0,001430338	RPS20
Prednisolone-184_mg/kg_in_Water-Rat-Liver-3d-up	1/337	0,993134836	1	0	0	0,203942203	0,001404925	RPSA
Hexachlorophene-8_mg/kg_in_Corn_Oil-Rat-Liver-1d-dn	1/346	0,993996762	1	0	0	0,198637348	0,001196061	TPT1
Lead(IV)_Acetate-200_mg/kg_in_Saline-Rat-Bone_marrow-3d-up	1/350	0,994344352	1	0	0	0,196367207	0,001113736	FEM1B
TGF_Beta-1_Human_Recombinant-5_ng/ml_in_DMSO-Rat-Primary_rat_hepatocytes-0.67d-up	1/355	0,994750752	1	0	0	0,193601471	0,001018939	SLC16A3
Norethindrone-75_mg/kg_in_Corn_Oil-Rat-Liver-1d-dn	1/357	0,994905037	1	0	0	0,192516869	0,000983374	TPT1
Propylene_Glycol-2000_mg/kg_in_Water-Rat-Kidney-5d-up	1/411	0,997725235	1	0	0	0,167222682	0,000380826	SEPP1

L35A;SNHG6;EFNA1;CXCL11;RPS27;RPS29;WT1;RPS20;RPS21;ITM2A;HSPA1B;SLC26A6;TPT1

ARL2BP;RPS29;ITM2B
APGEF5;MIR210HG;UBA52;BEX1;IFI6;MARC2;ADGRG5;APBB2;NPTX1;RPS27A;VAT1L;NTNG2;LINC00472;RPL41;GAD1;SNHG6;DOLK;GNG12;POU4F1;EFNA1;KBTBD4;RPS27;ARL2BP;COL5A3;R3HCC1;ITM2A;ITM2B

Supplementary Table 4

Description	ber_of_datasets_MDS	MDS BM	MDS CD34+	MDS 812	MDS 748	MDS 747	MDS GMP	MDS SC	MDS 749	MDS 750	MDS 206	MDS HSC	MDS MEP
ribosomal protein S19	12	TRUE	TRUE	TRUE	TRUE	TRUE	TRUE	TRUE	TRUE	TRUE	TRUE	TRUE	TRUE
ribosomal protein SA	11	TRUE	TRUE	TRUE	TRUE	TRUE	TRUE	TRUE	FALSE	TRUE	TRUE	TRUE	TRUE
hemoglobin subunit gamma 2	9	TRUE	TRUE	TRUE	TRUE	TRUE	FALSE	FALSE	TRUE	TRUE	TRUE	FALSE	TRUE
integral membrane protein 2A	9	FALSE	FALSE	TRUE	TRUE	TRUE	TRUE	FALSE	TRUE	TRUE	TRUE	TRUE	TRUE
ribosomal protein S21	8	TRUE	TRUE	FALSE	TRUE	TRUE	FALSE	FALSE	TRUE	TRUE	TRUE	FALSE	TRUE
ribosomal protein S20	8	TRUE	FALSE	TRUE	TRUE	TRUE	TRUE	TRUE	TRUE	TRUE	FALSE	FALSE	FALSE
ribosomal protein L11	7	TRUE	TRUE	TRUE	TRUE	TRUE	FALSE	FALSE	TRUE	FALSE	TRUE	FALSE	FALSE
interferon induced transmembrane |	7	FALSE	FALSE	TRUE	TRUE	TRUE	FALSE	FALSE	TRUE	FALSE	TRUE	TRUE	TRUE
ribosomal protein S29	7	TRUE	TRUE	TRUE	TRUE	TRUE	FALSE	TRUE	FALSE	FALSE	FALSE	TRUE	FALSE
brain expressed X-linked 1	7	TRUE	FALSE	TRUE	FALSE	TRUE	TRUE	FALSE	TRUE	TRUE	FALSE	TRUE	FALSE
ribosomal protein S18	7	TRUE	TRUE	TRUE	TRUE	TRUE	FALSE	FALSE	TRUE	TRUE	FALSE	FALSE	FALSE
ribosomal protein L36	7	TRUE	TRUE	FALSE	TRUE	TRUE	FALSE	TRUE	TRUE	TRUE	FALSE	FALSE	FALSE
ribosomal protein lateral stalk subu	7	TRUE	TRUE	TRUE	FALSE	FALSE	TRUE	TRUE	FALSE	FALSE	TRUE	TRUE	FALSE
ribosomal protein L27	6	TRUE	FALSE	TRUE	TRUE	TRUE	TRUE	FALSE	FALSE	FALSE	FALSE	TRUE	FALSE
ribosomal protein L12	6	TRUE	TRUE	FALSE	TRUE	TRUE	TRUE	FALSE	FALSE	TRUE	FALSE	FALSE	FALSE
ribosomal protein L19	6	TRUE	FALSE	TRUE	TRUE	TRUE	FALSE	FALSE	FALSE	TRUE	FALSE	TRUE	FALSE
ribosomal protein L34	6	TRUE	FALSE	TRUE	TRUE	TRUE	FALSE	FALSE	TRUE	FALSE	TRUE	FALSE	FALSE
ribosomal protein S7	5	TRUE	FALSE	FALSE	FALSE	FALSE	TRUE	TRUE	FALSE	FALSE	TRUE	TRUE	FALSE
ribosomal protein S5	5	TRUE	TRUE	TRUE	TRUE	FALSE	FALSE	TRUE	FALSE	FALSE	FALSE	FALSE	FALSE
small nucleolar RNA host gene 6	5	FALSE	FALSE	FALSE	TRUE	TRUE	FALSE	FALSE	TRUE	TRUE	TRUE	FALSE	FALSE
ribosomal protein L30	5	TRUE	FALSE	FALSE	TRUE	TRUE	TRUE	FALSE	TRUE	FALSE	FALSE	FALSE	FALSE
ribosomal protein L32	5	TRUE	FALSE	FALSE	TRUE	TRUE	FALSE	TRUE	FALSE	TRUE	FALSE	FALSE	FALSE
ribosomal protein S17	5	TRUE	TRUE	FALSE	FALSE	FALSE	TRUE	TRUE	FALSE	FALSE	FALSE	TRUE	FALSE
ADP ribosylation factor like GTPase (	5	FALSE	FALSE	FALSE	TRUE	FALSE	FALSE	FALSE	TRUE	FALSE	TRUE	TRUE	TRUE
ribosomal protein L39	5	TRUE	TRUE	TRUE	TRUE	TRUE	FALSE	FALSE	FALSE	FALSE	FALSE	FALSE	FALSE
ubiquitin A-52 residue ribosomal prc	5	TRUE	TRUE	FALSE	TRUE	FALSE	TRUE	FALSE	FALSE	FALSE	FALSE	TRUE	FALSE
ribosomal protein L41	4	TRUE	TRUE	TRUE	FALSE	FALSE	FALSE	TRUE	FALSE	FALSE	FALSE	FALSE	FALSE
ribosomal protein S27	4	TRUE	FALSE	FALSE	TRUE	FALSE	TRUE	TRUE	FALSE	FALSE	FALSE	FALSE	FALSE
ribosomal protein L35	4	TRUE	TRUE	FALSE	FALSE	FALSE	TRUE	TRUE	FALSE	FALSE	FALSE	FALSE	FALSE
ribosomal protein S8	3	TRUE	FALSE	FALSE	FALSE	FALSE	TRUE	FALSE	FALSE	FALSE	FALSE	TRUE	FALSE
interferon alpha inducible protein 6	3	FALSE	TRUE	FALSE	FALSE	FALSE	FALSE	FALSE	TRUE	FALSE	TRUE	FALSE	FALSE
ribosomal protein S3	3	TRUE	TRUE	FALSE	FALSE	FALSE	FALSE	TRUE	FALSE	FALSE	FALSE	FALSE	FALSE
ribosomal protein S23	3	TRUE	FALSE	FALSE	FALSE	TRUE	FALSE	FALSE	FALSE	TRUE	FALSE	FALSE	FALSE
ribosomal protein S25	3	TRUE	FALSE	TRUE	FALSE	FALSE	FALSE	TRUE	FALSE	FALSE	FALSE	FALSE	FALSE
ribosomal protein L9	3	FALSE	TRUE	FALSE	FALSE	FALSE	FALSE	TRUE	FALSE	FALSE	FALSE	FALSE	TRUE
eukaryotic translation elongation fa	3	TRUE	FALSE	TRUE	FALSE	FALSE	TRUE	FALSE	FALSE	FALSE	FALSE	FALSE	FALSE
ribosomal protein L37	3	TRUE	TRUE	FALSE	FALSE	FALSE	TRUE	FALSE	FALSE	FALSE	FALSE	FALSE	FALSE
ribosomal protein S12	3	TRUE	TRUE	FALSE	FALSE	FALSE	TRUE	FALSE	FALSE	FALSE	FALSE	FALSE	FALSE
serglycin	3	TRUE	FALSE	TRUE	FALSE	FALSE	TRUE	FALSE	FALSE	FALSE	FALSE	FALSE	FALSE
ribosomal protein S3A	3	TRUE	FALSE	TRUE	FALSE	FALSE	TRUE	FALSE	FALSE	FALSE	FALSE	FALSE	FALSE
cathepsin G	3	TRUE	FALSE	TRUE	FALSE	TRUE	FALSE	FALSE	FALSE	FALSE	FALSE	FALSE	FALSE
ribosomal protein L10a	3	TRUE	FALSE	FALSE	TRUE	FALSE	TRUE	FALSE	FALSE	FALSE	FALSE	FALSE	FALSE
ribosomal protein S6	2	TRUE	FALSE	FALSE	FALSE	FALSE	FALSE	TRUE	FALSE	FALSE	FALSE	FALSE	FALSE

NA	2	TRUE	TRUE	FALSE	FALSE	FALSE	FALSE	FALSE	FALSE	FALSE	FALSE	FALSE	FALSE
eukaryotic translation elongation fa	2	TRUE	FALSE	FALSE	FALSE	FALSE	FALSE	TRUE	FALSE	FALSE	FALSE	FALSE	FALSE
lymphocyte antigen 6 family membe	2	FALSE	TRUE	FALSE	FALSE	FALSE	FALSE	TRUE	FALSE	FALSE	FALSE	FALSE	FALSE
Charcot-Leyden crystal galectin	2	FALSE	FALSE	TRUE	FALSE	FALSE	FALSE	FALSE	FALSE	TRUE	FALSE	FALSE	FALSE
translocase of outer mitochondrial r	2	FALSE	FALSE	FALSE	FALSE	FALSE	FALSE	FALSE	TRUE	FALSE	FALSE	FALSE	TRUE
integral membrane protein 2B	2	FALSE	TRUE	FALSE	FALSE	FALSE	FALSE	TRUE	FALSE	FALSE	FALSE	FALSE	FALSE
ribosomal protein L7	2	TRUE	FALSE	FALSE	FALSE	FALSE	TRUE	FALSE	FALSE	FALSE	FALSE	FALSE	FALSE
ribosomal protein L13a	2	TRUE	TRUE	FALSE	FALSE	FALSE	FALSE	FALSE	FALSE	FALSE	FALSE	FALSE	FALSE
ribosomal protein L37a	2	TRUE	TRUE	FALSE	FALSE	FALSE	FALSE	FALSE	FALSE	FALSE	FALSE	FALSE	FALSE
ribosomal protein S27a	2	TRUE	FALSE	FALSE	FALSE	FALSE	TRUE	FALSE	FALSE	FALSE	FALSE	FALSE	FALSE
tumor protein, translationally-contr	2	FALSE	FALSE	FALSE	FALSE	TRUE	TRUE	FALSE	FALSE	FALSE	FALSE	FALSE	FALSE
cystatin F	2	TRUE	FALSE	FALSE	FALSE	FALSE	TRUE	FALSE	FALSE	FALSE	FALSE	FALSE	FALSE
family with sequence similarity 129	1	FALSE	TRUE	FALSE	FALSE	FALSE	FALSE	FALSE	FALSE	FALSE	FALSE	FALSE	FALSE
aldehyde oxidase 2 pseudogene	1	FALSE	TRUE	FALSE	FALSE	FALSE	FALSE	FALSE	FALSE	FALSE	FALSE	FALSE	FALSE
paired box 5	1	FALSE	FALSE	FALSE	FALSE	FALSE	FALSE	TRUE	FALSE	FALSE	FALSE	FALSE	FALSE
neuron navigator 2	1	FALSE	FALSE	FALSE	FALSE	FALSE	FALSE	TRUE	FALSE	FALSE	FALSE	FALSE	FALSE
immunoglobulin lambda like polype	1	FALSE	TRUE	FALSE	FALSE	FALSE	FALSE	FALSE	FALSE	FALSE	FALSE	FALSE	FALSE
defensin alpha 3	1	TRUE	FALSE	FALSE	FALSE	FALSE	FALSE	FALSE	FALSE	FALSE	FALSE	FALSE	FALSE
hemoglobin subunit gamma 1	1	FALSE	FALSE	FALSE	FALSE	FALSE	FALSE	FALSE	FALSE	FALSE	FALSE	FALSE	TRUE
natural killer cell granule protein 7	1	TRUE	FALSE	FALSE	FALSE	FALSE	FALSE	FALSE	FALSE	FALSE	FALSE	FALSE	FALSE
ribosomal protein L3	1	TRUE	FALSE	FALSE	FALSE	FALSE	FALSE	FALSE	FALSE	FALSE	FALSE	FALSE	FALSE
ribosomal protein L31	1	TRUE	FALSE	FALSE	FALSE	FALSE	FALSE	FALSE	FALSE	FALSE	FALSE	FALSE	FALSE
interleukin 12A	1	FALSE	FALSE	FALSE	FALSE	FALSE	FALSE	TRUE	FALSE	FALSE	FALSE	FALSE	FALSE
ribosomal protein S14	1	TRUE	FALSE	FALSE	FALSE	FALSE	FALSE	FALSE	FALSE	FALSE	FALSE	FALSE	FALSE
ribosomal protein L35a	1	TRUE	FALSE	FALSE	FALSE	FALSE	FALSE	FALSE	FALSE	FALSE	FALSE	FALSE	FALSE
ribonuclease A family member 3	1	FALSE	FALSE	TRUE	FALSE	FALSE	FALSE	FALSE	FALSE	FALSE	FALSE	FALSE	FALSE
cytidine/uridine monophosphate kin	1	FALSE	FALSE	FALSE	FALSE	FALSE	FALSE	FALSE	FALSE	FALSE	TRUE	FALSE	FALSE
UDP-GlcNAc:betaGal beta-1,3-N-ace	1	FALSE	FALSE	FALSE	FALSE	FALSE	FALSE	FALSE	FALSE	FALSE	TRUE	FALSE	FALSE
complement C1q A chain	1	FALSE	TRUE	FALSE	FALSE	FALSE	FALSE	FALSE	FALSE	FALSE	FALSE	FALSE	FALSE
cilia and flagella associated protein	0	FALSE	FALSE	FALSE	FALSE	FALSE	FALSE	FALSE	FALSE	FALSE	FALSE	FALSE	FALSE
microRNA 6075	0	FALSE	FALSE	FALSE	FALSE	FALSE	FALSE	FALSE	FALSE	FALSE	FALSE	FALSE	FALSE
POU class 2 associating factor 1	0	FALSE	FALSE	FALSE	FALSE	FALSE	FALSE	FALSE	FALSE	FALSE	FALSE	FALSE	FALSE
NA	0	FALSE	FALSE	FALSE	FALSE	FALSE	FALSE	FALSE	FALSE	FALSE	FALSE	FALSE	FALSE
POU class 4 homeobox 1	0	FALSE	FALSE	FALSE	FALSE	FALSE	FALSE	FALSE	FALSE	FALSE	FALSE	FALSE	FALSE
NA	0	FALSE	FALSE	FALSE	FALSE	FALSE	FALSE	FALSE	FALSE	FALSE	FALSE	FALSE	FALSE
family with sequence similarity 157	0	FALSE	FALSE	FALSE	FALSE	FALSE	FALSE	FALSE	FALSE	FALSE	FALSE	FALSE	FALSE
NA	0	FALSE	FALSE	FALSE	FALSE	FALSE	FALSE	FALSE	FALSE	FALSE	FALSE	FALSE	FALSE
microRNA 7848	0	FALSE	FALSE	FALSE	FALSE	FALSE	FALSE	FALSE	FALSE	FALSE	FALSE	FALSE	FALSE
ASH2 like histone lysine methyltrans	0	FALSE	FALSE	FALSE	FALSE	FALSE	FALSE	FALSE	FALSE	FALSE	FALSE	FALSE	FALSE
NA	0	FALSE	FALSE	FALSE	FALSE	FALSE	FALSE	FALSE	FALSE	FALSE	FALSE	FALSE	FALSE
microRNA 3155a	0	FALSE	FALSE	FALSE	FALSE	FALSE	FALSE	FALSE	FALSE	FALSE	FALSE	FALSE	FALSE
NA	0	FALSE	FALSE	FALSE	FALSE	FALSE	FALSE	FALSE	FALSE	FALSE	FALSE	FALSE	FALSE
RNA, 7SL, cytoplasmic 683, pseudog	0	FALSE	FALSE	FALSE	FALSE	FALSE	FALSE	FALSE	FALSE	FALSE	FALSE	FALSE	FALSE
NA	0	FALSE	FALSE	FALSE	FALSE	FALSE	FALSE	FALSE	FALSE	FALSE	FALSE	FALSE	FALSE
receptor tyrosine kinase like orphan	0	FALSE	FALSE	FALSE	FALSE	FALSE	FALSE	FALSE	FALSE	FALSE	FALSE	FALSE	FALSE
MIR210 host gene	0	FALSE	FALSE	FALSE	FALSE	FALSE	FALSE	FALSE	FALSE	FALSE	FALSE	FALSE	FALSE

heat shock protein family A (Hsp70)	0	FALSE	FALSE	FALSE	FALSE	FALSE	FALSE	FALSE	FALSE	FALSE	FALSE	FALSE	FALSE
transmembrane protein 63A	0	FALSE	FALSE	FALSE	FALSE	FALSE	FALSE	FALSE	FALSE	FALSE	FALSE	FALSE	FALSE
NA	0	FALSE	FALSE	FALSE	FALSE	FALSE	FALSE	FALSE	FALSE	FALSE	FALSE	FALSE	FALSE
microRNA 3909	0	FALSE	FALSE	FALSE	FALSE	FALSE	FALSE	FALSE	FALSE	FALSE	FALSE	FALSE	FALSE
NA	0	FALSE	FALSE	FALSE	FALSE	FALSE	FALSE	FALSE	FALSE	FALSE	FALSE	FALSE	FALSE
immunoglobulin kappa variable 1D-	0	FALSE	FALSE	FALSE	FALSE	FALSE	FALSE	FALSE	FALSE	FALSE	FALSE	FALSE	FALSE
lysosomal associated membrane prc	0	FALSE	FALSE	FALSE	FALSE	FALSE	FALSE	FALSE	FALSE	FALSE	FALSE	FALSE	FALSE
V-set pre-B cell surrogate light chair	0	FALSE	FALSE	FALSE	FALSE	FALSE	FALSE	FALSE	FALSE	FALSE	FALSE	FALSE	FALSE
NA	0	FALSE	FALSE	FALSE	FALSE	FALSE	FALSE	FALSE	FALSE	FALSE	FALSE	FALSE	FALSE
NA	0	FALSE	FALSE	FALSE	FALSE	FALSE	FALSE	FALSE	FALSE	FALSE	FALSE	FALSE	FALSE
V-set pre-B cell surrogate light chair	0	FALSE	FALSE	FALSE	FALSE	FALSE	FALSE	FALSE	FALSE	FALSE	FALSE	FALSE	FALSE
NA	0	FALSE	FALSE	FALSE	FALSE	FALSE	FALSE	FALSE	FALSE	FALSE	FALSE	FALSE	FALSE
neuronal pentraxin 1	0	FALSE	FALSE	FALSE	FALSE	FALSE	FALSE	FALSE	FALSE	FALSE	FALSE	FALSE	FALSE
amyloid beta precursor protein bind	0	FALSE	FALSE	FALSE	FALSE	FALSE	FALSE	FALSE	FALSE	FALSE	FALSE	FALSE	FALSE
gap junction protein alpha 6 pseudo	0	FALSE	FALSE	FALSE	FALSE	FALSE	FALSE	FALSE	FALSE	FALSE	FALSE	FALSE	FALSE
solute carrier family 16 member 3	0	FALSE	FALSE	FALSE	FALSE	FALSE	FALSE	FALSE	FALSE	FALSE	FALSE	FALSE	FALSE
NA	0	FALSE	FALSE	FALSE	FALSE	FALSE	FALSE	FALSE	FALSE	FALSE	FALSE	FALSE	FALSE
NA	0	FALSE	FALSE	FALSE	FALSE	FALSE	FALSE	FALSE	FALSE	FALSE	FALSE	FALSE	FALSE
netrin G2	0	FALSE	FALSE	FALSE	FALSE	FALSE	FALSE	FALSE	FALSE	FALSE	FALSE	FALSE	FALSE
NA	0	FALSE	FALSE	FALSE	FALSE	FALSE	FALSE	FALSE	FALSE	FALSE	FALSE	FALSE	FALSE
long intergenic non-protein coding F	0	FALSE	FALSE	FALSE	FALSE	FALSE	FALSE	FALSE	FALSE	FALSE	FALSE	FALSE	FALSE
transient receptor potential cation c	0	FALSE	FALSE	FALSE	FALSE	FALSE	FALSE	FALSE	FALSE	FALSE	FALSE	FALSE	FALSE
cytochrome P450 family 4 subfamily	0	FALSE	FALSE	FALSE	FALSE	FALSE	FALSE	FALSE	FALSE	FALSE	FALSE	FALSE	FALSE
microRNA 27a	0	FALSE	FALSE	FALSE	FALSE	FALSE	FALSE	FALSE	FALSE	FALSE	FALSE	FALSE	FALSE
NA	0	FALSE	FALSE	FALSE	FALSE	FALSE	FALSE	FALSE	FALSE	FALSE	FALSE	FALSE	FALSE
RNA, 7SK small nuclear pseudogene	0	FALSE	FALSE	FALSE	FALSE	FALSE	FALSE	FALSE	FALSE	FALSE	FALSE	FALSE	FALSE
NA	0	FALSE	FALSE	FALSE	FALSE	FALSE	FALSE	FALSE	FALSE	FALSE	FALSE	FALSE	FALSE
NA	0	FALSE	FALSE	FALSE	FALSE	FALSE	FALSE	FALSE	FALSE	FALSE	FALSE	FALSE	FALSE
NA	0	FALSE	FALSE	FALSE	FALSE	FALSE	FALSE	FALSE	FALSE	FALSE	FALSE	FALSE	FALSE
cadherin related family member 2	0	FALSE	FALSE	FALSE	FALSE	FALSE	FALSE	FALSE	FALSE	FALSE	FALSE	FALSE	FALSE
HIF1A antisense RNA 2	0	FALSE	FALSE	FALSE	FALSE	FALSE	FALSE	FALSE	FALSE	FALSE	FALSE	FALSE	FALSE
immunoglobulin kappa variable 2D-	0	FALSE	FALSE	FALSE	FALSE	FALSE	FALSE	FALSE	FALSE	FALSE	FALSE	FALSE	FALSE
NA	0	FALSE	FALSE	FALSE	FALSE	FALSE	FALSE	FALSE	FALSE	FALSE	FALSE	FALSE	FALSE
olfactory receptor family 10 subfam	0	FALSE	FALSE	FALSE	FALSE	FALSE	FALSE	FALSE	FALSE	FALSE	FALSE	FALSE	FALSE
NA	0	FALSE	FALSE	FALSE	FALSE	FALSE	FALSE	FALSE	FALSE	FALSE	FALSE	FALSE	FALSE
cell migration inducing hyaluronan l	0	FALSE	FALSE	FALSE	FALSE	FALSE	FALSE	FALSE	FALSE	FALSE	FALSE	FALSE	FALSE
dipeptidyl peptidase like 10	0	FALSE	FALSE	FALSE	FALSE	FALSE	FALSE	FALSE	FALSE	FALSE	FALSE	FALSE	FALSE
RNA, 7SL, cytoplasmic 600, pseudog	0	FALSE	FALSE	FALSE	FALSE	FALSE	FALSE	FALSE	FALSE	FALSE	FALSE	FALSE	FALSE
NA	0	FALSE	FALSE	FALSE	FALSE	FALSE	FALSE	FALSE	FALSE	FALSE	FALSE	FALSE	FALSE
hedgehog interacting protein	0	FALSE	FALSE	FALSE	FALSE	FALSE	FALSE	FALSE	FALSE	FALSE	FALSE	FALSE	FALSE
NA	0	FALSE	FALSE	FALSE	FALSE	FALSE	FALSE	FALSE	FALSE	FALSE	FALSE	FALSE	FALSE
tropomyosin 3 pseudogene 7	0	FALSE	FALSE	FALSE	FALSE	FALSE	FALSE	FALSE	FALSE	FALSE	FALSE	FALSE	FALSE
ATP binding cassette subfamily A me	0	FALSE	FALSE	FALSE	FALSE	FALSE	FALSE	FALSE	FALSE	FALSE	FALSE	FALSE	FALSE
NA	0	FALSE	FALSE	FALSE	FALSE	FALSE	FALSE	FALSE	FALSE	FALSE	FALSE	FALSE	FALSE
Fc receptor like A	0	FALSE	FALSE	FALSE	FALSE	FALSE	FALSE	FALSE	FALSE	FALSE	FALSE	FALSE	FALSE
La ribonucleoprotein domain family	0	FALSE	FALSE	FALSE	FALSE	FALSE	FALSE	FALSE	FALSE	FALSE	FALSE	FALSE	FALSE

transmembrane protein 104	0	FALSE	FALSE	FALSE	FALSE	FALSE	FALSE	FALSE	FALSE	FALSE	FALSE	FALSE	FALSE
NA	0	FALSE	FALSE	FALSE	FALSE	FALSE	FALSE	FALSE	FALSE	FALSE	FALSE	FALSE	FALSE
amidohydrolase domain containing	0	FALSE	FALSE	FALSE	FALSE	FALSE	FALSE	FALSE	FALSE	FALSE	FALSE	FALSE	FALSE
RNA, U6 small nuclear 1003, pseudo	0	FALSE	FALSE	FALSE	FALSE	FALSE	FALSE	FALSE	FALSE	FALSE	FALSE	FALSE	FALSE
NA	0	FALSE	FALSE	FALSE	FALSE	FALSE	FALSE	FALSE	FALSE	FALSE	FALSE	FALSE	FALSE
NA	0	FALSE	FALSE	FALSE	FALSE	FALSE	FALSE	FALSE	FALSE	FALSE	FALSE	FALSE	FALSE
NA	0	FALSE	FALSE	FALSE	FALSE	FALSE	FALSE	FALSE	FALSE	FALSE	FALSE	FALSE	FALSE
NA	0	FALSE	FALSE	FALSE	FALSE	FALSE	FALSE	FALSE	FALSE	FALSE	FALSE	FALSE	FALSE
recombination activating 1	0	FALSE	FALSE	FALSE	FALSE	FALSE	FALSE	FALSE	FALSE	FALSE	FALSE	FALSE	FALSE
recombination activating 2	0	FALSE	FALSE	FALSE	FALSE	FALSE	FALSE	FALSE	FALSE	FALSE	FALSE	FALSE	FALSE
MIR646 host gene	0	FALSE	FALSE	FALSE	FALSE	FALSE	FALSE	FALSE	FALSE	FALSE	FALSE	FALSE	FALSE
NA	0	FALSE	FALSE	FALSE	FALSE	FALSE	FALSE	FALSE	FALSE	FALSE	FALSE	FALSE	FALSE
Fc receptor like 1	0	FALSE	FALSE	FALSE	FALSE	FALSE	FALSE	FALSE	FALSE	FALSE	FALSE	FALSE	FALSE
potassium voltage-gated channel su	0	FALSE	FALSE	FALSE	FALSE	FALSE	FALSE	FALSE	FALSE	FALSE	FALSE	FALSE	FALSE
NA	0	FALSE	FALSE	FALSE	FALSE	FALSE	FALSE	FALSE	FALSE	FALSE	FALSE	FALSE	FALSE
NA	0	FALSE	FALSE	FALSE	FALSE	FALSE	FALSE	FALSE	FALSE	FALSE	FALSE	FALSE	FALSE
DNA nucleotidylexotransferase	0	FALSE	FALSE	FALSE	FALSE	FALSE	FALSE	FALSE	FALSE	FALSE	FALSE	FALSE	FALSE
germinal center associated signalinc	0	FALSE	FALSE	FALSE	FALSE	FALSE	FALSE	FALSE	FALSE	FALSE	FALSE	FALSE	FALSE
NA	0	FALSE	FALSE	FALSE	FALSE	FALSE	FALSE	FALSE	FALSE	FALSE	FALSE	FALSE	FALSE
NA	0	FALSE	FALSE	FALSE	FALSE	FALSE	FALSE	FALSE	FALSE	FALSE	FALSE	FALSE	FALSE
NA	0	FALSE	FALSE	FALSE	FALSE	FALSE	FALSE	FALSE	FALSE	FALSE	FALSE	FALSE	FALSE
bone morphogenetic protein 3	0	FALSE	FALSE	FALSE	FALSE	FALSE	FALSE	FALSE	FALSE	FALSE	FALSE	FALSE	FALSE
NME/NM23 family member 8	0	FALSE	FALSE	FALSE	FALSE	FALSE	FALSE	FALSE	FALSE	FALSE	FALSE	FALSE	FALSE
myosin IIIA	0	FALSE	FALSE	FALSE	FALSE	FALSE	FALSE	FALSE	FALSE	FALSE	FALSE	FALSE	FALSE
NA	0	FALSE	FALSE	FALSE	FALSE	FALSE	FALSE	FALSE	FALSE	FALSE	FALSE	FALSE	FALSE
coiled-coil domain containing 191	0	FALSE	FALSE	FALSE	FALSE	FALSE	FALSE	FALSE	FALSE	FALSE	FALSE	FALSE	FALSE
cAMP regulated phosphoprotein 21	0	FALSE	FALSE	FALSE	FALSE	FALSE	FALSE	FALSE	FALSE	FALSE	FALSE	FALSE	FALSE
solute carrier family 26 member 6	0	FALSE	FALSE	FALSE	FALSE	FALSE	FALSE	FALSE	FALSE	FALSE	FALSE	FALSE	FALSE
Rap guanine nucleotide exchange fa	0	FALSE	FALSE	FALSE	FALSE	FALSE	FALSE	FALSE	FALSE	FALSE	FALSE	FALSE	FALSE
mitochondrially encoded NADH:ubiq	0	FALSE	FALSE	FALSE	FALSE	FALSE	FALSE	FALSE	FALSE	FALSE	FALSE	FALSE	FALSE
harakiri, BCL2 interacting protein	0	FALSE	FALSE	FALSE	FALSE	FALSE	FALSE	FALSE	FALSE	FALSE	FALSE	FALSE	FALSE
long intergenic non-protein coding R	0	FALSE	FALSE	FALSE	FALSE	FALSE	FALSE	FALSE	FALSE	FALSE	FALSE	FALSE	FALSE
NA	0	FALSE	FALSE	FALSE	FALSE	FALSE	FALSE	FALSE	FALSE	FALSE	FALSE	FALSE	FALSE
hypoxia inducible lipid droplet assoc	0	FALSE	FALSE	FALSE	FALSE	FALSE	FALSE	FALSE	FALSE	FALSE	FALSE	FALSE	FALSE
NA	0	FALSE	FALSE	FALSE	FALSE	FALSE	FALSE	FALSE	FALSE	FALSE	FALSE	FALSE	FALSE
NA	0	FALSE	FALSE	FALSE	FALSE	FALSE	FALSE	FALSE	FALSE	FALSE	FALSE	FALSE	FALSE
deltex E3 ubiquitin ligase 1	0	FALSE	FALSE	FALSE	FALSE	FALSE	FALSE	FALSE	FALSE	FALSE	FALSE	FALSE	FALSE
AE binding protein 1	0	FALSE	FALSE	FALSE	FALSE	FALSE	FALSE	FALSE	FALSE	FALSE	FALSE	FALSE	FALSE
RPS6KA2 intronic transcript 1	0	FALSE	FALSE	FALSE	FALSE	FALSE	FALSE	FALSE	FALSE	FALSE	FALSE	FALSE	FALSE
hexokinase 2	0	FALSE	FALSE	FALSE	FALSE	FALSE	FALSE	FALSE	FALSE	FALSE	FALSE	FALSE	FALSE
zinc finger protein 415 pseudogene	0	FALSE	FALSE	FALSE	FALSE	FALSE	FALSE	FALSE	FALSE	FALSE	FALSE	FALSE	FALSE
NA	0	FALSE	FALSE	FALSE	FALSE	FALSE	FALSE	FALSE	FALSE	FALSE	FALSE	FALSE	FALSE
NA	0	FALSE	FALSE	FALSE	FALSE	FALSE	FALSE	FALSE	FALSE	FALSE	FALSE	FALSE	FALSE
NA	0	FALSE	FALSE	FALSE	FALSE	FALSE	FALSE	FALSE	FALSE	FALSE	FALSE	FALSE	FALSE
NA	0	FALSE	FALSE	FALSE	FALSE	FALSE	FALSE	FALSE	FALSE	FALSE	FALSE	FALSE	FALSE
RNA, 7SL, cytoplasmic 472, pseudog	0	FALSE	FALSE	FALSE	FALSE	FALSE	FALSE	FALSE	FALSE	FALSE	FALSE	FALSE	FALSE

NA	0	FALSE	FALSE	FALSE	FALSE	FALSE	FALSE	FALSE	FALSE	FALSE	FALSE	FALSE	FALSE
NA	0	FALSE	FALSE	FALSE	FALSE	FALSE	FALSE	FALSE	FALSE	FALSE	FALSE	FALSE	FALSE
CPB2 antisense RNA 1	0	FALSE	FALSE	FALSE	FALSE	FALSE	FALSE	FALSE	FALSE	FALSE	FALSE	FALSE	FALSE
SET pseudogene 21	0	FALSE	FALSE	FALSE	FALSE	FALSE	FALSE	FALSE	FALSE	FALSE	FALSE	FALSE	FALSE
NA	0	FALSE	FALSE	FALSE	FALSE	FALSE	FALSE	FALSE	FALSE	FALSE	FALSE	FALSE	FALSE
aldehyde oxidase 3, pseudogene	0	FALSE	FALSE	FALSE	FALSE	FALSE	FALSE	FALSE	FALSE	FALSE	FALSE	FALSE	FALSE
CXADR, Ig-like cell adhesion molecul	0	FALSE	FALSE	FALSE	FALSE	FALSE	FALSE	FALSE	FALSE	FALSE	FALSE	FALSE	FALSE
RNA polymerase III subunit D pseud	0	FALSE	FALSE	FALSE	FALSE	FALSE	FALSE	FALSE	FALSE	FALSE	FALSE	FALSE	FALSE
long intergenic non-protein coding	0	FALSE	FALSE	FALSE	FALSE	FALSE	FALSE	FALSE	FALSE	FALSE	FALSE	FALSE	FALSE
microRNA 23a	0	FALSE	FALSE	FALSE	FALSE	FALSE	FALSE	FALSE	FALSE	FALSE	FALSE	FALSE	FALSE
glutamate decarboxylase 1	0	FALSE	FALSE	FALSE	FALSE	FALSE	FALSE	FALSE	FALSE	FALSE	FALSE	FALSE	FALSE
olfactory receptor family 6 subfamil	0	FALSE	FALSE	FALSE	FALSE	FALSE	FALSE	FALSE	FALSE	FALSE	FALSE	FALSE	FALSE
NA	0	FALSE	FALSE	FALSE	FALSE	FALSE	FALSE	FALSE	FALSE	FALSE	FALSE	FALSE	FALSE
NA	0	FALSE	FALSE	FALSE	FALSE	FALSE	FALSE	FALSE	FALSE	FALSE	FALSE	FALSE	FALSE
NA	0	FALSE	FALSE	FALSE	FALSE	FALSE	FALSE	FALSE	FALSE	FALSE	FALSE	FALSE	FALSE
NA	0	FALSE	FALSE	FALSE	FALSE	FALSE	FALSE	FALSE	FALSE	FALSE	FALSE	FALSE	FALSE
microRNA 24-2	0	FALSE	FALSE	FALSE	FALSE	FALSE	FALSE	FALSE	FALSE	FALSE	FALSE	FALSE	FALSE
NA	0	FALSE	FALSE	FALSE	FALSE	FALSE	FALSE	FALSE	FALSE	FALSE	FALSE	FALSE	FALSE
dishevelled associated activator of	0	FALSE	FALSE	FALSE	FALSE	FALSE	FALSE	FALSE	FALSE	FALSE	FALSE	FALSE	FALSE
claudin 9	0	FALSE	FALSE	FALSE	FALSE	FALSE	FALSE	FALSE	FALSE	FALSE	FALSE	FALSE	FALSE
NA	0	FALSE	FALSE	FALSE	FALSE	FALSE	FALSE	FALSE	FALSE	FALSE	FALSE	FALSE	FALSE
RAB19, member RAS oncogene fami	0	FALSE	FALSE	FALSE	FALSE	FALSE	FALSE	FALSE	FALSE	FALSE	FALSE	FALSE	FALSE
small nuclear ribonucleoprotein D2	0	FALSE	FALSE	FALSE	FALSE	FALSE	FALSE	FALSE	FALSE	FALSE	FALSE	FALSE	FALSE
NA	0	FALSE	FALSE	FALSE	FALSE	FALSE	FALSE	FALSE	FALSE	FALSE	FALSE	FALSE	FALSE
C-type lectin domain family 12 mem	0	FALSE	FALSE	FALSE	FALSE	FALSE	FALSE	FALSE	FALSE	FALSE	FALSE	FALSE	FALSE
NA	0	FALSE	FALSE	FALSE	FALSE	FALSE	FALSE	FALSE	FALSE	FALSE	FALSE	FALSE	FALSE
CEP295 N-terminal like	0	FALSE	FALSE	FALSE	FALSE	FALSE	FALSE	FALSE	FALSE	FALSE	FALSE	FALSE	FALSE
brain enriched guanylate kinase ass	0	FALSE	FALSE	FALSE	FALSE	FALSE	FALSE	FALSE	FALSE	FALSE	FALSE	FALSE	FALSE
high mobility group box 1 pseudoge	0	FALSE	FALSE	FALSE	FALSE	FALSE	FALSE	FALSE	FALSE	FALSE	FALSE	FALSE	FALSE
FAR1 intronic transcript 1	0	FALSE	FALSE	FALSE	FALSE	FALSE	FALSE	FALSE	FALSE	FALSE	FALSE	FALSE	FALSE
NA	0	FALSE	FALSE	FALSE	FALSE	FALSE	FALSE	FALSE	FALSE	FALSE	FALSE	FALSE	FALSE
NA	0	FALSE	FALSE	FALSE	FALSE	FALSE	FALSE	FALSE	FALSE	FALSE	FALSE	FALSE	FALSE
CECR2, histone acetyl-lysine reader	0	FALSE	FALSE	FALSE	FALSE	FALSE	FALSE	FALSE	FALSE	FALSE	FALSE	FALSE	FALSE
sphingomyelin phosphodiesterase 5	0	FALSE	FALSE	FALSE	FALSE	FALSE	FALSE	FALSE	FALSE	FALSE	FALSE	FALSE	FALSE
5'-nucleotidase domain containing 3	0	FALSE	FALSE	FALSE	FALSE	FALSE	FALSE	FALSE	FALSE	FALSE	FALSE	FALSE	FALSE
RNA, 7SL, cytoplasmic 288, pseudog	0	FALSE	FALSE	FALSE	FALSE	FALSE	FALSE	FALSE	FALSE	FALSE	FALSE	FALSE	FALSE
ribosomal protein S6 kinase A2	0	FALSE	FALSE	FALSE	FALSE	FALSE	FALSE	FALSE	FALSE	FALSE	FALSE	FALSE	FALSE
vesicle amine transport 1 like	0	FALSE	FALSE	FALSE	FALSE	FALSE	FALSE	FALSE	FALSE	FALSE	FALSE	FALSE	FALSE
NA	0	FALSE	FALSE	FALSE	FALSE	FALSE	FALSE	FALSE	FALSE	FALSE	FALSE	FALSE	FALSE
NA	0	FALSE	FALSE	FALSE	FALSE	FALSE	FALSE	FALSE	FALSE	FALSE	FALSE	FALSE	FALSE
EPH receptor B4	0	FALSE	FALSE	FALSE	FALSE	FALSE	FALSE	FALSE	FALSE	FALSE	FALSE	FALSE	FALSE
transmembrane 4 L six family memb	0	FALSE	FALSE	FALSE	FALSE	FALSE	FALSE	FALSE	FALSE	FALSE	FALSE	FALSE	FALSE
KIAA1217	0	FALSE	FALSE	FALSE	FALSE	FALSE	FALSE	FALSE	FALSE	FALSE	FALSE	FALSE	FALSE
C-X-C motif chemokine ligand 11	0	FALSE	FALSE	FALSE	FALSE	FALSE	FALSE	FALSE	FALSE	FALSE	FALSE	FALSE	FALSE
Wilms tumor 1	0	FALSE	FALSE	FALSE	FALSE	FALSE	FALSE	FALSE	FALSE	FALSE	FALSE	FALSE	FALSE
docking protein 4	0	FALSE	FALSE	FALSE	FALSE	FALSE	FALSE	FALSE	FALSE	FALSE	FALSE	FALSE	FALSE

collagen type V alpha 3 chain	0	FALSE	FALSE	FALSE	FALSE	FALSE	FALSE	FALSE	FALSE	FALSE	FALSE	FALSE	FALSE
long intergenic non-protein coding F	0	FALSE	FALSE	FALSE	FALSE	FALSE	FALSE	FALSE	FALSE	FALSE	FALSE	FALSE	FALSE
NA	0	FALSE	FALSE	FALSE	FALSE	FALSE	FALSE	FALSE	FALSE	FALSE	FALSE	FALSE	FALSE
zinc finger protein 792	0	FALSE	FALSE	FALSE	FALSE	FALSE	FALSE	FALSE	FALSE	FALSE	FALSE	FALSE	FALSE
sterile alpha motif domain containir	0	FALSE	FALSE	FALSE	FALSE	FALSE	FALSE	FALSE	FALSE	FALSE	FALSE	FALSE	FALSE
RNA, U6 small nuclear 8	0	FALSE	FALSE	FALSE	FALSE	FALSE	FALSE	FALSE	FALSE	FALSE	FALSE	FALSE	FALSE
coiled-coil domain containing 102A	0	FALSE	FALSE	FALSE	FALSE	FALSE	FALSE	FALSE	FALSE	FALSE	FALSE	FALSE	FALSE
PIGY upstream reading frame	0	FALSE	FALSE	FALSE	FALSE	FALSE	FALSE	FALSE	FALSE	FALSE	FALSE	FALSE	FALSE
cleavage and polyadenylation factor	0	FALSE	FALSE	FALSE	FALSE	FALSE	FALSE	FALSE	FALSE	FALSE	FALSE	FALSE	FALSE
nuclear receptor binding factor 2	0	FALSE	FALSE	FALSE	FALSE	FALSE	FALSE	FALSE	FALSE	FALSE	FALSE	FALSE	FALSE
homeobox A1	0	FALSE	FALSE	FALSE	FALSE	FALSE	FALSE	FALSE	FALSE	FALSE	FALSE	FALSE	FALSE
solute carrier family 6 member 19	0	FALSE	FALSE	FALSE	FALSE	FALSE	FALSE	FALSE	FALSE	FALSE	FALSE	FALSE	FALSE
dolichol kinase	0	FALSE	FALSE	FALSE	FALSE	FALSE	FALSE	FALSE	FALSE	FALSE	FALSE	FALSE	FALSE
COMM domain containing 6	0	FALSE	FALSE	FALSE	FALSE	FALSE	FALSE	FALSE	FALSE	FALSE	FALSE	FALSE	FALSE
ephrin A1	0	FALSE	FALSE	FALSE	FALSE	FALSE	FALSE	FALSE	FALSE	FALSE	FALSE	FALSE	FALSE
homeobox B5	0	FALSE	FALSE	FALSE	FALSE	FALSE	FALSE	FALSE	FALSE	FALSE	FALSE	FALSE	FALSE
CD274 molecule	0	FALSE	FALSE	FALSE	FALSE	FALSE	FALSE	FALSE	FALSE	FALSE	FALSE	FALSE	FALSE
desert hedgehog	0	FALSE	FALSE	FALSE	FALSE	FALSE	FALSE	FALSE	FALSE	FALSE	FALSE	FALSE	FALSE
glycerophosphodiester phosphodies	0	FALSE	FALSE	FALSE	FALSE	FALSE	FALSE	FALSE	FALSE	FALSE	FALSE	FALSE	FALSE
adhesion G protein-coupled recepto	0	FALSE	FALSE	FALSE	FALSE	FALSE	FALSE	FALSE	FALSE	FALSE	FALSE	FALSE	FALSE
NA	0	FALSE	FALSE	FALSE	FALSE	FALSE	FALSE	FALSE	FALSE	FALSE	FALSE	FALSE	FALSE
eukaryotic translation elongation fa	0	FALSE	FALSE	FALSE	FALSE	FALSE	FALSE	FALSE	FALSE	FALSE	FALSE	FALSE	FALSE
eukaryotic translation elongation fa	0	FALSE	FALSE	FALSE	FALSE	FALSE	FALSE	FALSE	FALSE	FALSE	FALSE	FALSE	FALSE
membrane associated ring-CH-type	0	FALSE	FALSE	FALSE	FALSE	FALSE	FALSE	FALSE	FALSE	FALSE	FALSE	FALSE	FALSE
TNF superfamily member 10	0	FALSE	FALSE	FALSE	FALSE	FALSE	FALSE	FALSE	FALSE	FALSE	FALSE	FALSE	FALSE
golgin A7	0	FALSE	FALSE	FALSE	FALSE	FALSE	FALSE	FALSE	FALSE	FALSE	FALSE	FALSE	FALSE
family with sequence similarity 81 m	0	FALSE	FALSE	FALSE	FALSE	FALSE	FALSE	FALSE	FALSE	FALSE	FALSE	FALSE	FALSE
NA	0	FALSE	FALSE	FALSE	FALSE	FALSE	FALSE	FALSE	FALSE	FALSE	FALSE	FALSE	FALSE
family with sequence similarity 32 m	0	FALSE	FALSE	FALSE	FALSE	FALSE	FALSE	FALSE	FALSE	FALSE	FALSE	FALSE	FALSE
G protein subunit gamma 12	0	FALSE	FALSE	FALSE	FALSE	FALSE	FALSE	FALSE	FALSE	FALSE	FALSE	FALSE	FALSE
kelch repeat and BTB domain contai	0	FALSE	FALSE	FALSE	FALSE	FALSE	FALSE	FALSE	FALSE	FALSE	FALSE	FALSE	FALSE
R3H domain and coiled-coil containi	0	FALSE	FALSE	FALSE	FALSE	FALSE	FALSE	FALSE	FALSE	FALSE	FALSE	FALSE	FALSE
ADP ribosylation factor like GTPase	0	FALSE	FALSE	FALSE	FALSE	FALSE	FALSE	FALSE	FALSE	FALSE	FALSE	FALSE	FALSE
NA	0	FALSE	FALSE	FALSE	FALSE	FALSE	FALSE	FALSE	FALSE	FALSE	FALSE	FALSE	FALSE
hemoglobin subunit beta pseudoger	0	FALSE	FALSE	FALSE	FALSE	FALSE	FALSE	FALSE	FALSE	FALSE	FALSE	FALSE	FALSE
MyoD family inhibitor	0	FALSE	FALSE	FALSE	FALSE	FALSE	FALSE	FALSE	FALSE	FALSE	FALSE	FALSE	FALSE
zinc finger protein 597	0	FALSE	FALSE	FALSE	FALSE	FALSE	FALSE	FALSE	FALSE	FALSE	FALSE	FALSE	FALSE
CD244 molecule	0	FALSE	FALSE	FALSE	FALSE	FALSE	FALSE	FALSE	FALSE	FALSE	FALSE	FALSE	FALSE
prostaglandin D2 receptor 2	0	FALSE	FALSE	FALSE	FALSE	FALSE	FALSE	FALSE	FALSE	FALSE	FALSE	FALSE	FALSE
beta globin locus transcript 3 (non-p	0	FALSE	FALSE	FALSE	FALSE	FALSE	FALSE	FALSE	FALSE	FALSE	FALSE	FALSE	FALSE
NA	0	FALSE	FALSE	FALSE	FALSE	FALSE	FALSE	FALSE	FALSE	FALSE	FALSE	FALSE	FALSE
eukaryotic translation initiation fact	0	FALSE	FALSE	FALSE	FALSE	FALSE	FALSE	FALSE	FALSE	FALSE	FALSE	FALSE	FALSE
NA	0	FALSE	FALSE	FALSE	FALSE	FALSE	FALSE	FALSE	FALSE	FALSE	FALSE	FALSE	FALSE
dual specificity phosphatase 14	0	FALSE	FALSE	FALSE	FALSE	FALSE	FALSE	FALSE	FALSE	FALSE	FALSE	FALSE	FALSE
Fc fragment of IgG receptor IIIb	0	FALSE	FALSE	FALSE	FALSE	FALSE	FALSE	FALSE	FALSE	FALSE	FALSE	FALSE	FALSE
adhesion G protein-coupled recepto	0	FALSE	FALSE	FALSE	FALSE	FALSE	FALSE	FALSE	FALSE	FALSE	FALSE	FALSE	FALSE

major facilitator superfamily domai	0	FALSE	FALSE	FALSE	FALSE	FALSE	FALSE	FALSE	FALSE	FALSE	FALSE	FALSE	FALSE
NA	0	FALSE	FALSE	FALSE	FALSE	FALSE	FALSE	FALSE	FALSE	FALSE	FALSE	FALSE	FALSE
FUN14 domain containing 1	0	FALSE	FALSE	FALSE	FALSE	FALSE	FALSE	FALSE	FALSE	FALSE	FALSE	FALSE	FALSE
p53 and DNA damage regulated 1	0	FALSE	FALSE	FALSE	FALSE	FALSE	FALSE	FALSE	FALSE	FALSE	FALSE	FALSE	FALSE
nucleophosmin/nucleoplasmin 2	0	FALSE	FALSE	FALSE	FALSE	FALSE	FALSE	FALSE	FALSE	FALSE	FALSE	FALSE	FALSE
fem-1 homolog B	0	FALSE	FALSE	FALSE	FALSE	FALSE	FALSE	FALSE	FALSE	FALSE	FALSE	FALSE	FALSE
vault RNA 1-3	0	FALSE	FALSE	FALSE	FALSE	FALSE	FALSE	FALSE	FALSE	FALSE	FALSE	FALSE	FALSE
angiopoietin like 6	0	FALSE	FALSE	FALSE	FALSE	FALSE	FALSE	FALSE	FALSE	FALSE	FALSE	FALSE	FALSE
eukaryotic translation elongation fa	0	FALSE	FALSE	FALSE	FALSE	FALSE	FALSE	FALSE	FALSE	FALSE	FALSE	FALSE	FALSE
NA	0	FALSE	FALSE	FALSE	FALSE	FALSE	FALSE	FALSE	FALSE	FALSE	FALSE	FALSE	FALSE
NA	0	FALSE	FALSE	FALSE	FALSE	FALSE	FALSE	FALSE	FALSE	FALSE	FALSE	FALSE	FALSE
receptor transporter protein 4	0	FALSE	FALSE	FALSE	FALSE	FALSE	FALSE	FALSE	FALSE	FALSE	FALSE	FALSE	FALSE
eukaryotic translation elongation fa	0	FALSE	FALSE	FALSE	FALSE	FALSE	FALSE	FALSE	FALSE	FALSE	FALSE	FALSE	FALSE
NA	0	FALSE	FALSE	FALSE	FALSE	FALSE	FALSE	FALSE	FALSE	FALSE	FALSE	FALSE	FALSE
NA	0	FALSE	FALSE	FALSE	FALSE	FALSE	FALSE	FALSE	FALSE	FALSE	FALSE	FALSE	FALSE
interferon induced with helicase C d	0	FALSE	FALSE	FALSE	FALSE	FALSE	FALSE	FALSE	FALSE	FALSE	FALSE	FALSE	FALSE
interferon beta 1	0	FALSE	FALSE	FALSE	FALSE	FALSE	FALSE	FALSE	FALSE	FALSE	FALSE	FALSE	FALSE
zinc finger protein 689	0	FALSE	FALSE	FALSE	FALSE	FALSE	FALSE	FALSE	FALSE	FALSE	FALSE	FALSE	FALSE

Zubovic et al., Supplementary Figure S1

E

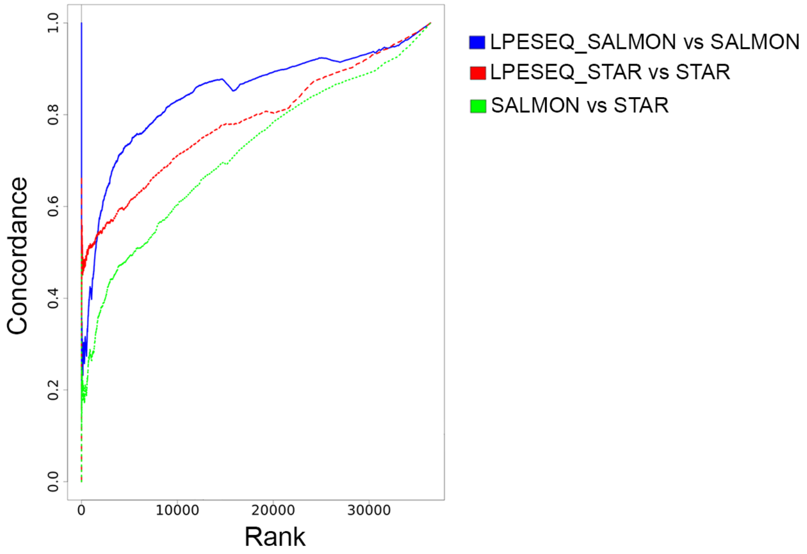

F

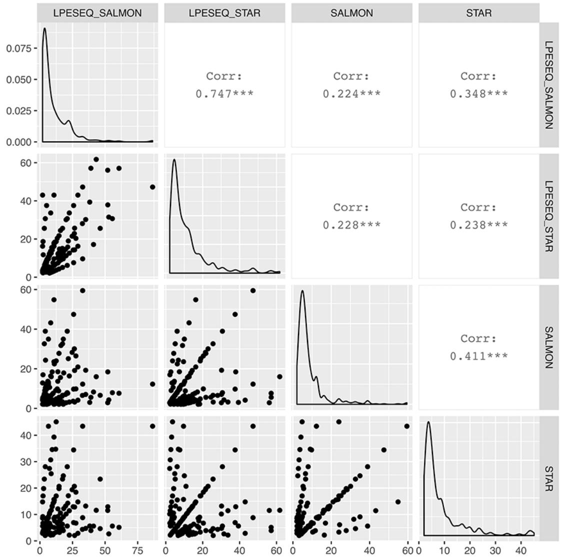

G

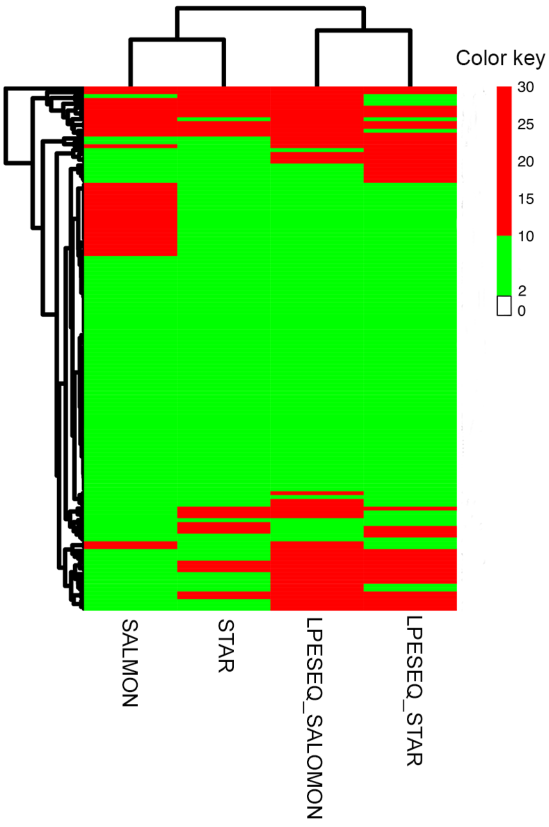

Zubovic et al., Supplementary Figure S2

A

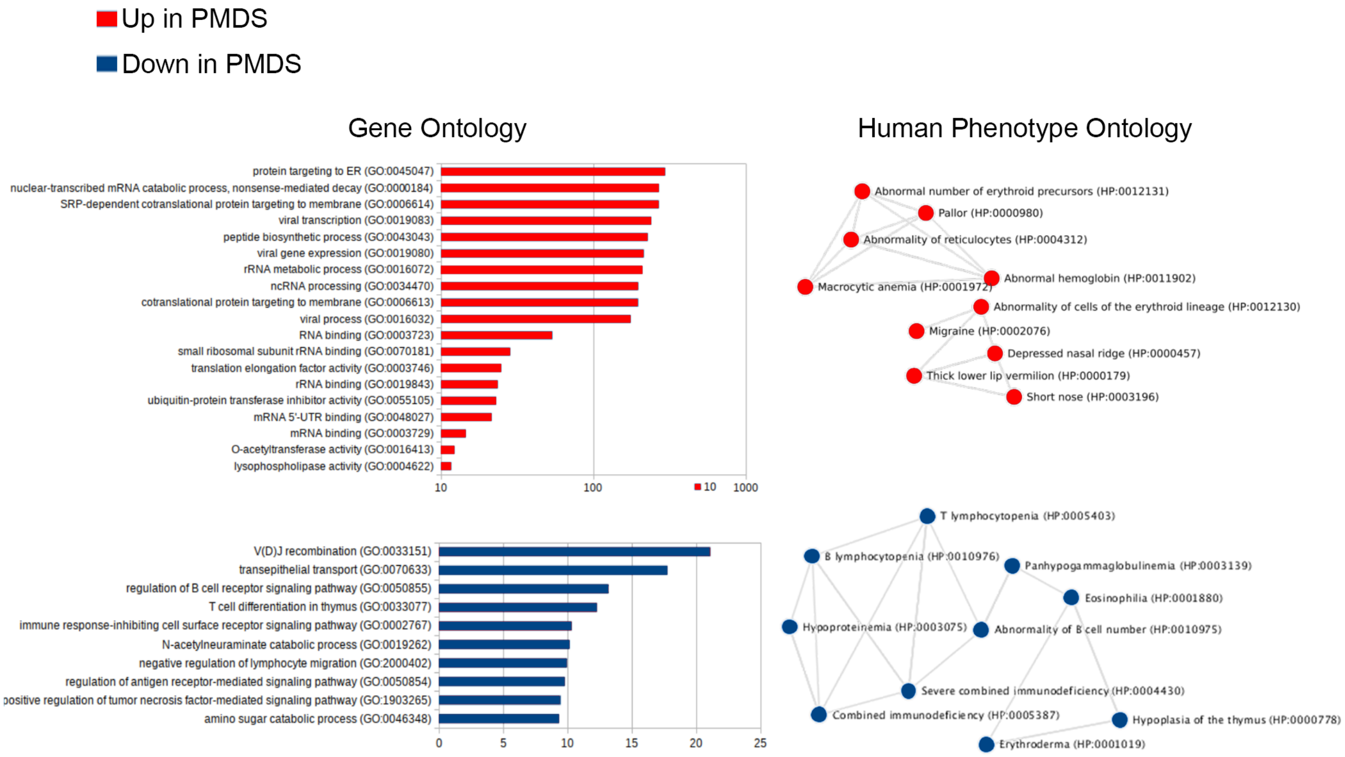

B

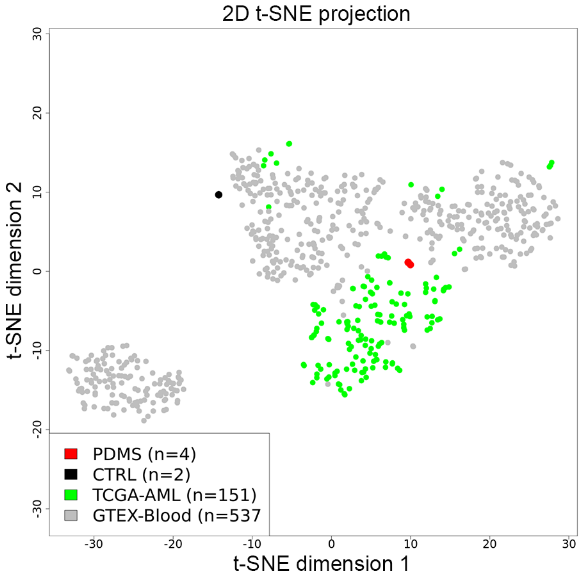

Zubovic et al., Supplementary Figure S3

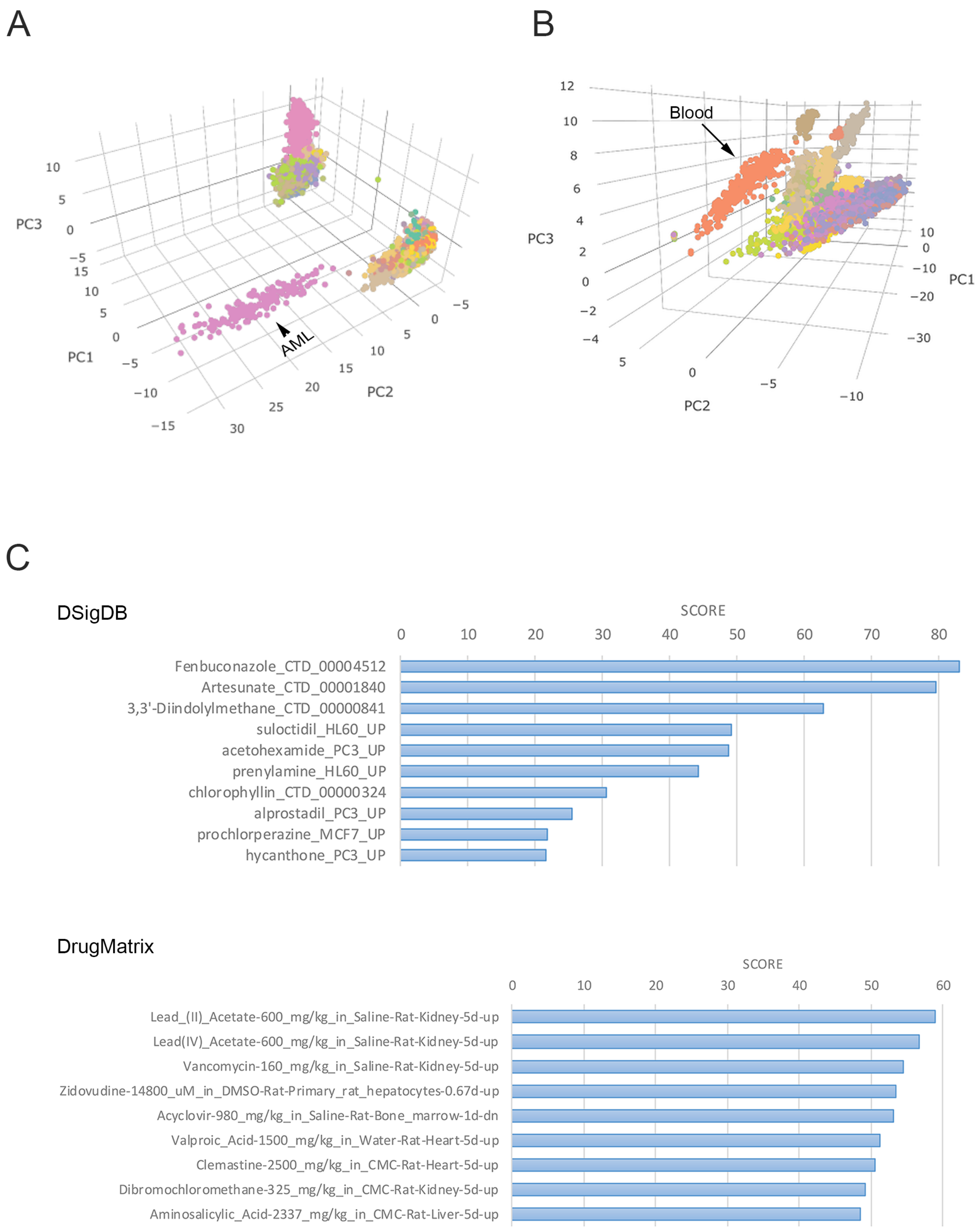

Zubovic et al., Supplementary Figure S4

A

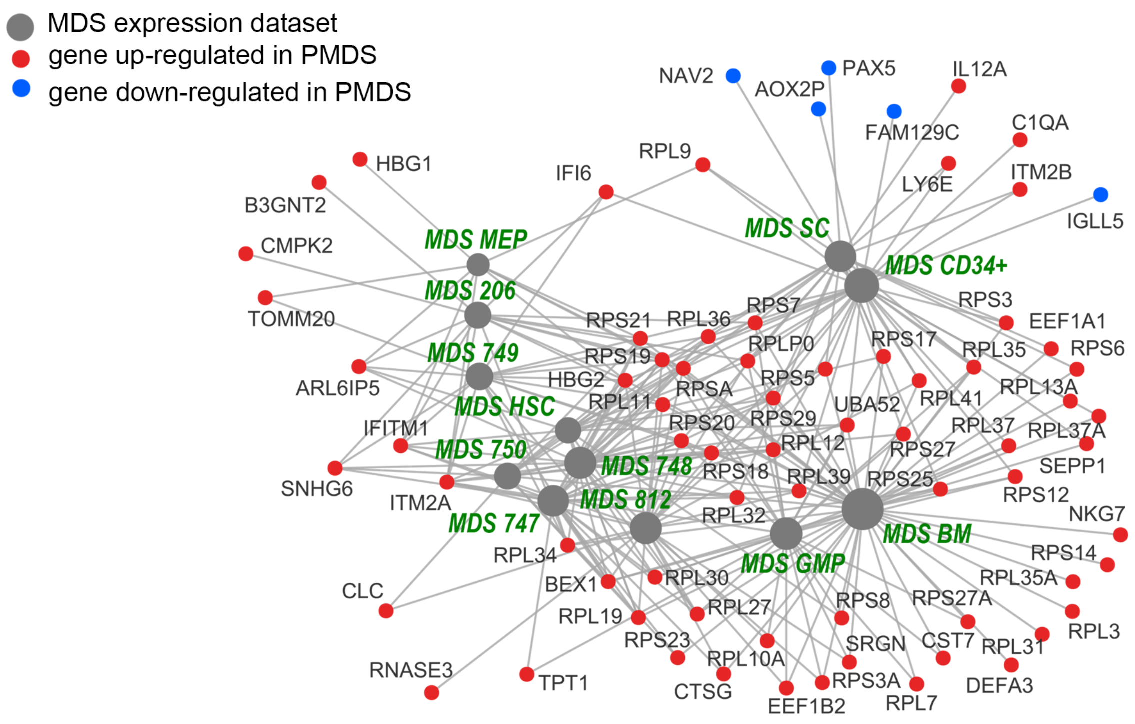

B

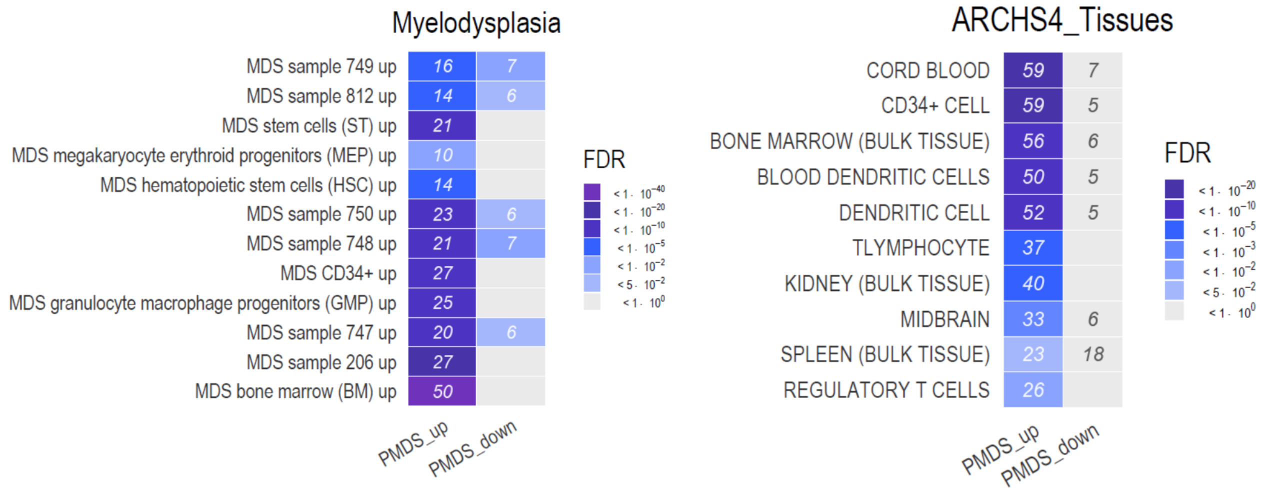

Zubovic et al., Supplementary Figure S5

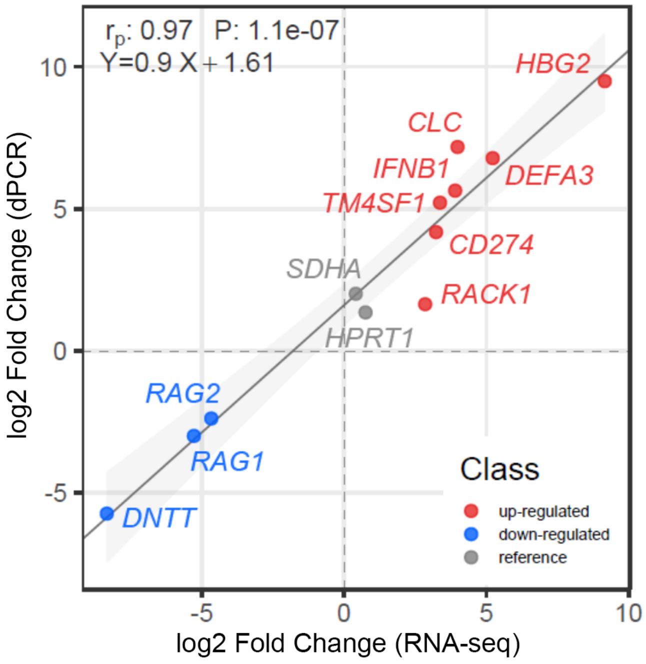

Zubovic et al., Supplementary Figure S6

A

Samples	Age of diagnosis (years)	Sex	Year of birth	Caryotype	Diagnosis
**P5**	14	Female	2006	45 XX, -7	MDS EB-1
**P6**	10	Female	2010	46 XX	MDS EB-1
**B1**	5	Female	2011	46 XX	MDS EB-1
**B2**	7	Male	2011	46 XY	MDS EB-1
**B3**	12	Male	2006	46 XY	MDS EB-2
**B4**	11	Female	2009	46 XX	MDS EB-1

B

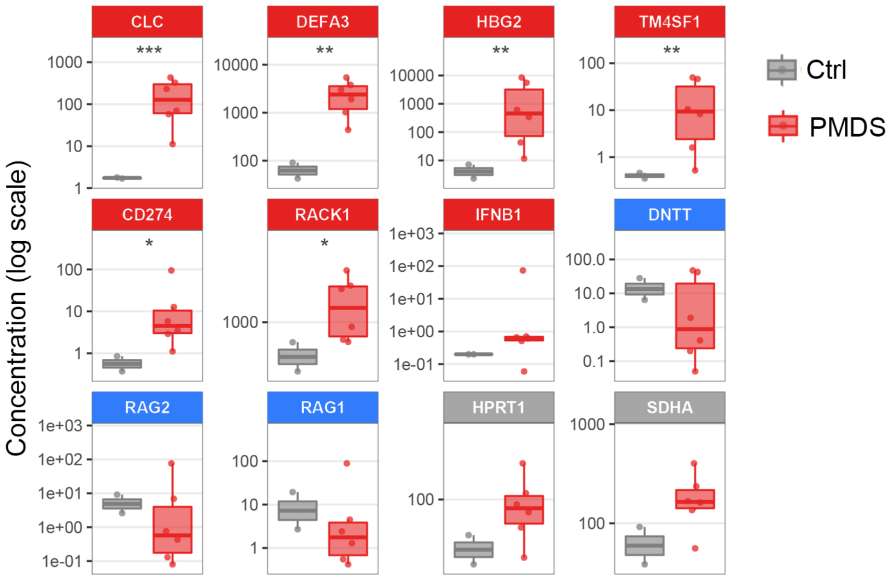

C

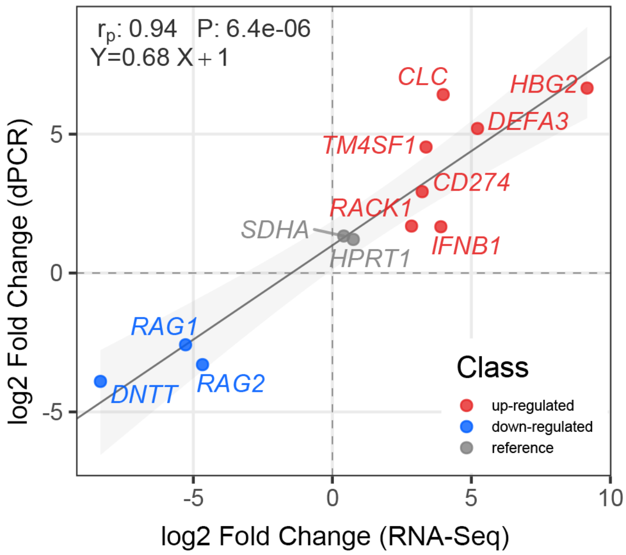

Zubovic et al., Supplementary Figure S7

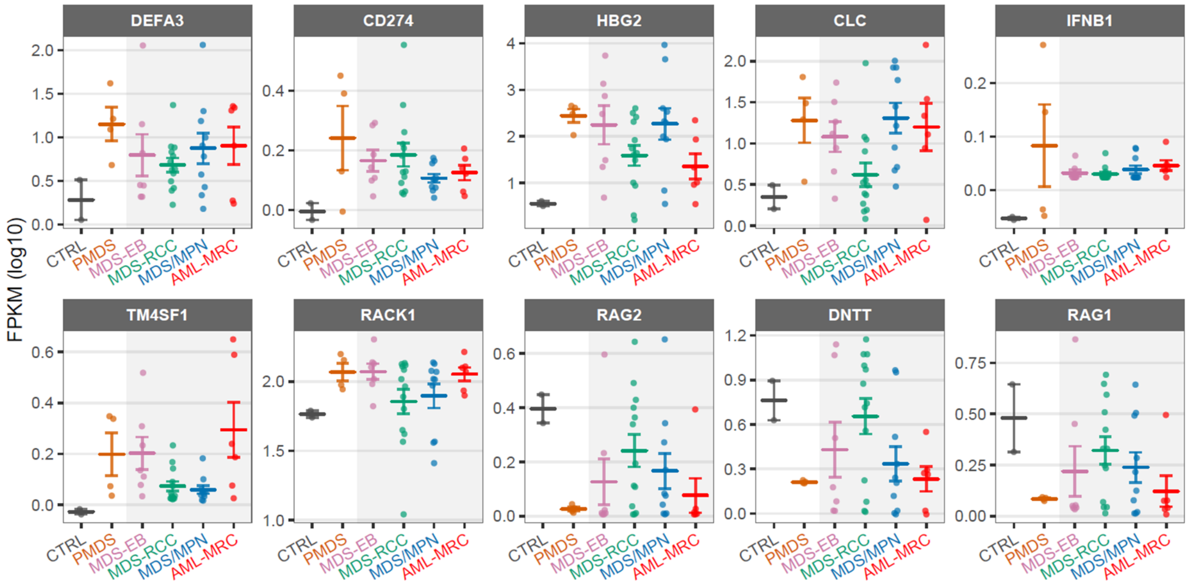

Supplementary Table 1.

Upregulated DEGs	Downregulated DEGs
RPS8	CFAP73
RPS6	MIR6075
RPS7	POU2AF1
DEFA3	RP11-206L10.2
SEPP1	POU4F1
RPSA	TSSC1-IT1
TM4SF1	FAM157C
KIAA1217	RP11-563J2.2
EEF1A1	MIR7848
CXCL11	ASH2LP1
WT1	RP4-666F24.3
DOK4	MIR3155A
COL5A3	AC084117.3
LY6E	RN7SL683P
CLC	RP4-669L17.10
LINC01218	ROR1
RPL27	MIR210HG
RP11-521I2.3	HSPA1B
ZNF792	TMEM63A
SAMD9	RP1-161N10.1
RPL11	MIR3909
IFITM1	C6ORF99
RNU6-8	IGKV1D-22
RPL12	FAM129C
IFI6	LAMP5
CCDC102A	VPREB3
TOMM20	RP11-723D22.2
PYURF	RP1-168P16.2
CLP1	VPREB1
NRBF2	RP11-452K12.6
RPS5	NPTX1
HOXA1	APBB2
RPS3	GJA6P
RPL19	SLC16A3
HBG1	RP11-553K8.2
SLC6A19	RP11-10J21.4
RPL41	NTNG2
HBG2	CTD-3137H5.4
NKG7	LINC00671

Supplementary Table 1.

DOLK	TRPC6
SNHG6	CYP4F3
COMMD6	MIR27A
RPS27	RP13-554M15.7
EFNA1	RN7SKP134
RPS29	CTC-436K13.1
HOXB5	RP11-605P22.1
RPS21	CTC-436K13.2
RPS20	CDHR2
ITM2A	HIF1A-AS2
RPS23	IGKV2D-24
ITM2B	RP11-804M7.2
RPS25	OR10AA1P
RPL3	AOX2P
RPL31	RP11-5L12.1
CD274	CEMIP
DHH	DPP10
RPL30	RN7SL600P
BEX1	RP11-164J13.1
RPL32	HHIP
RPL35	KB-208E9.1
RPL34	TPM3P7
GDE1	ABCA13
RPL9	RP11-295K3.1
RPL7	FCRLA
EEF1B2	LARP1P1
ADGRG5	TMEM104
RPS18	RP11-147L13.14
RPS17	AMDHD2
C6ORF106	RNU6-1003P
RPS19	RP11-196B3.1
EEF1A1P12	RP11-426C22.5
EEF1A1P13	RP11-123C21.3
MARC2	RP3-416J7.4
RPL37	RAG1
ARL6IP5	RAG2
RPL36	MIR646HG
RPL39	RP11-769N19.2
TNFSF10	FCRL1
IL12A	KCNJ12
GOLGA7	RP11-426C22.1

Supplementary Table 1.

RPS12	RP11-35J23.1
FAM81B	PAX5
RPS14	DNTT
RP11-561B11.1	GCSAM
SRGN	NAV2
FAM32A	RP11-87G24.3
RPL13A	RP11-87G24.2
RPS3A	METAZOA_SRP
GNG12	BMP3
KBTBD4	NME8
R3HCC1	MYO3A
ARL2BP	RP13-516M14.8
RP11-386I8.4	CCDC191
RPL37A	ARPP21
HBBP1	SLC26A6
MDFI	RAPGEF5
ZNF597	MTND5P32
CD244	HRK
PTGDR2	LINC01013
BGLT3	RP11-147L13.8
RP11-372K14.2	HILPDA
EIF4BP6	C2ORF48
RP11-152H18.3	C3ORF52
DUSP14	DTX1
FCGR3B	AEBP1
ADGRE1	RPS6KA2-IT1
MFSD5	HK2
CTD-2651B20.6	ZNF415P1
FUNDC1	RP11-16C1.2
CTSG	AC011899.9
RPS27A	RP11-87G24.6
PDRG1	C17ORF107
NPM2	RN7SL472P
FEM1B	RP11-391L3.3
VTRNA1-3	RP11-34P13.16
RPL35A	CPB2-AS1
RNASE3	SETP21
CMPK2	RP11-53B2.2
ANGPTL6	AOX3P

Supplementary Table 1.

B3GNT2	CXADR
UBA52	POLR3DP1
EEF1A1P5	LINC00472
CTD-2553L13.10	MIR23A
TPT1	GAD1
RP11-384O8.1	OR6N2
C1QA	AC130469.1
RTP4	RP11-618M23.5
EEF1A1P6	RP11-139H15.5
RPLP0	RP4-647J21.1
CTB-58E17.1	MIR24-2
RPL10A	IGLL5
RP11-217B1.2	RP13-516M14.10
CST7	DAAM2
IFIH1	CLDN9
IFNB1	RP11-631M6.2
ZNF689	RAB19
	SNRPD2P1
	RP11-216N14.7
	CLEC12B
	CTD-2179L22.1
	CEP295NL
	BEGAIN
	HMGB1P30
	FAR1-IT1
	AC068580.5
	RP4-673D20.3
	CECR2
	SMPD5
	NT5DC3
	RN7SL288P
	RPS6KA2
	VAT1L
	AL928654.7
	RP11-504I13.2
	EPHB4

SUPPLEMENTARY TABLE 2. DSigDB TABLE

Term	Overlap	P-value	Adjusted P-value	Old P-value	Old Adjusted P-value	Odds Ratio	Combined Score
3,3'-Diindolylmethane_CTD_00000841	9/105	2,2898E-05	0,092187548	0	0	5,8910162	62,94231997
suloctidil_HL60_UP	10/141	4,2074E-05	0,084694946	0	0	4,874363286	49,11447818
Fenbuconazole_CTD_00004512	5/36	0,000164121	0,220249745	0	0	9,545628102	83,18928492
Artesunate_CTD_00001840	5/37	0,000187525	0,188743686	0	0	9,287638154	79,70279177
prenylamine_HL60_UP	4/38	0,002195283	1	0	0	7,234581299	44,28608523
acetohexamide_PC3_UP	3/23	0,00435185	1	0	0	8,96458987	48,74185878
alprostadil_PC3_UP	4/53	0,007348174	1	0	0	5,18705829	25,48559148
prochlorperazine_MCF7_UP	4/58	0,010063486	1	0	0	4,739898092	21,79804059
chlorophyllin_CTD_00000324	3/31	0,010141135	1	0	0	6,651147323	30,53645065
Beryllium sulfate_CTD_00001005	3/39	0,01894004	1	0	0	5,286809411	20,97000822
LY-294002_PC3_DOWN	3/41	0,02162999	1	0	0	5,028916269	19,2792281
deferoxamine_PC3_UP	3/44	0,026035779	1	0	0	4,686035614	17,09598672
puromycin_MCF7_DOWN	5/116	0,027378506	1	0	0	2,962436308	10,65883702
2,2',4,4',5,5'-Hexachlorobiphenyl_CTD_00000731	7/209	0,033681487	1	0	0	2,301912231	7,805339959
hydralazine_CTD_00006108	18/777	0,035856216	1	0	0	1,592166541	5,2991097
hycanthone_PC3_UP	2/21	0,036935773	1	0	0	6,545573556	21,59106355
securinine_PC3_UP	3/52	0,039943221	1	0	0	3,965107058	12,7688196
2,6-DICHLORO-4-NITROPHENOL_CTD_00000815	2/22	0,040246859	1	0	0	6,248047485	20,07324784
triprolidine_PC3_UP	2/22	0,040246859	1	0	0	6,248047485	20,07324784
corbadrine_PC3_UP	2/23	0,043665437	1	0	0	5,976393247	18,71327297
propofol_MCF7_UP	3/54	0,043901983	1	0	0	3,818251241	11,93507367
protriptyline_MCF7_UP	2/24	0,047187703	1	0	0	5,727376861	17,48924374
rescinnamine_MCF7_UP	2/24	0,047187703	1	0	0	5,727376861	17,48924374
(3s)-3-Cyclopentyl-6-Methyl-7-[(4-Methylpiperazin-1-Yl)sulfonyl]-3,4-Dihy	3/57	0,05019171	1	0	0	3,617290649	10,82259146
lycorine_MCF7_DOWN	2/25	0,050809937	1	0	0	5,498281787	16,38302862
montelukast_CTD_00003205	2/25	0,050809937	1	0	0	5,498281787	16,38302862
nifuroxazide_HL60_DOWN	2/25	0,050809937	1	0	0	5,498281787	16,38302862
ampyrone_HL60_UP	10/380	0,052218424	1	0	0	1,808645325	5,339699576
celastrol_MCF7_DOWN	2/26	0,054528508	1	0	0	5,286809411	15,37949579
isoetarine_PC3_UP	2/26	0,054528508	1	0	0	5,286809411	15,37949579
3-Butylidenephthalide_CTD_00001227	3/59	0,054614772	1	0	0	3,494670627	10,16058319
dobutamine_PC3_UP	2/27	0,058339867	1	0	0	5,091001655	14,46592641
ciclopirox_HL60_DOWN	3/61	0,059217795	1	0	0	3,380091262	9,553940124
nocodazole_MCF7_UP	3/62	0,061585647	1	0	0	3,325573661	9,269459416
deferoxamine_MCF7_UP	2/29	0,066227163	1	0	0	4,739898092	12,86723347
colforsin_MCF7_UP	4/106	0,069154586	1	0	0	2,593529145	6,928382042
mebendazole_HL60_DOWN	5/151	0,07001291	1	0	0	2,275778885	6,05146816
ICN 1229_CTD_00006697	2/30	0,070296408	1	0	0	4,581901489	12,16510688
anisomycin_MCF7_DOWN	8/307	0,080797376	1	0	0	1,790971266	4,50574483
MG-262_PC3_DOWN	9/360	0,081362614	1	0	0	1,718213058	4,310720615
Cobalt sulfate_CTD_00001238	2/33	0,082968001	1	0	0	4,16536499	10,36884424
alexidine_PC3_DOWN	2/34	0,087336219	1	0	0	4,042854255	9,856438325
ritodrine_PC3_UP	2/35	0,091771664	1	0	0	3,927344134	9,380271764
benzethonium chloride_HL60_DOWN	2/36	0,096271471	1	0	0	3,818251241	8,936934929
ketoconazole_HL60_DOWN	2/38	0,105453052	1	0	0	3,617290649	8,137057087
Selenium methyl cysteine_CTD_00000103	2/38	0,105453052	1	0	0	3,617290649	8,137057087
2-Naphthoxyacetic acid_BOSS	2/38	0,105453052	1	0	0	3,617290649	8,137057087
thapsigargin_PC3_DOWN	8/329	0,108779668	1	0	0	1,67121027	3,707464395
mebendazole_MCF7_UP	2/40	0,114859393	1	0	0	3,436426117	7,436586154
aspirin_CTD_00005447	12/562	0,120423119	1	0	0	1,467512932	3,106348818
menadione_HL60_DOWN	3/84	0,123730797	1	0	0	2,454590083	5,129226974
clenbuterol_PC3_UP	2/42	0,124469957	1	0	0	3,272786778	6,819476037
IRON_CTD_00006166	5/181	0,125010906	1	0	0	1,898577965	3,94781625
NICKEL CHLORIDE_CTD_00001064	9/400	0,131039632	1	0	0	1,546391753	3,14266309
ellipticine_HL60_UP	3/87	0,133501358	1	0	0	2,369949046	4,772232792
etynodiol_HL60_DOWN	2/44	0,134265199	1	0	0	3,124023743	6,272847039
ajmaline_HL60_UP	7/294	0,138546336	1	0	0	1,636393389	3,234414095
puromycin_HL60_DOWN	6/240	0,138655017	1	0	0	1,718213058	3,394787491
chrysin_HL60_UP	2/45	0,139226228	1	0	0	3,054600993	6,022619713
fenoterol_PC3_UP	2/45	0,139226228	1	0	0	3,054600993	6,022619713
thiostrepton_PC3_UP	2/45	0,139226228	1	0	0	3,054600993	6,022619713
thapsigargin_MCF7_DOWN	4/137	0,13971036	1	0	0	2,006672185	3,949499796
harmine_MCF7_UP	3/90	0,143530018	1	0	0	2,290950745	4,447218974
Inosinic acid_BOSS	2/46	0,144226526	1	0	0	2,988196623	5,786254639
cephaeline_HL60_DOWN	9/411	0,147010105	1	0	0	1,505004139	2,885475131
5,6-Dichloro-1H-benzimidazole_CTD_00003559	1/11	0,148934309	1	0	0	6,248047485	11,89784409
IB-MECA_CTD_00003042	1/11	0,148934309	1	0	0	6,248047485	11,89784409
PRIMAQUINE_CTD_00006613	1/11	0,148934309	1	0	0	6,248047485	11,89784409
Bosentan_CTD_00003071	1/11	0,148934309	1	0	0	6,248047485	11,89784409
betulinic acid_HL60_DOWN	2/47	0,149263913	1	0	0	2,924617972	5,562738363
phenoxybenzamine_MCF7_DOWN	2/47	0,149263913	1	0	0	2,924617972	5,562738363
Bexarotene_CTD_00003225	3/92	0,150350566	1	0	0	2,241147468	4,246493959
cicloheximide_MCF7_DOWN	4/141	0,150369409	1	0	0	1,949745315	3,694105019
diltiazem_PC3_UP	2/48	0,154336259	1	0	0	2,863688431	5,351149929
dimethyloxalylglycine_PC3_UP	2/48	0,154336259	1	0	0	2,863688431	5,351149929
R-atenolol_PC3_DOWN	4/143	0,155812774	1	0	0	1,922476149	3,574075718
4-Methylhistamine_CTD_00001201	1/12	0,161324112	1	0	0	5,727376861	10,44868168
1,1-Bis(4-hydroxyphenyl)-2-phenylbut-1-ene_CTD_00002234	1/12	0,161324112	1	0	0	5,727376861	10,44868168
2-Naphthalenamine_CTD_00007026	3/97	0,167836074	1	0	0	2,125624402	3,793745411
(17S)-17-hydroxy-13,17-dimethyl-1,2,6,7,8,14,15,16-octahydrocyclopent	8/368	0,169637195	1	0	0	1,494098312	2,650669762
scopolamine_PC3_DOWN	8/368	0,169637195	1	0	0	1,494098312	2,650669762
ciclopirox_PC3_UP	3/98	0,171402475	1	0	0	2,103934357	3,710794938
amlexanox_CTD_00002035	1/13	0,173534154	1	0	0	5,286809411	9,259216728

15(R)-Prostaglandin D2_CTD_00007048	1/13	0,173534154	1	0	0	5,286809411	9,259216728
126643-37-6_CTD_00002813	1/13	0,173534154	1	0	0	5,286809411	9,259216728
MS-275_PC3_UP	9/431	0,178401246	1	0	0	1,43516636	2,473825061
piperlongumine_MCF7_DOWN	6/262	0,183752179	1	0	0	1,573935626	2,666510241
MG-132_MCF7_DOWN	5/207	0,184616136	1	0	0	1,660109235	2,804715628
HELENALIN_CTD_00000035	2/54	0,185392029	1	0	0	2,545500827	4,289888298
10'(Z),13'(E),15'(E)-Heptadecatrienylhydroquinone_CTD_00004147	1/14	0,185567037	1	0	0	4,909180167	8,268724001
bepridil_CTD_00007094	1/14	0,185567037	1	0	0	4,909180167	8,268724001
celastrol_CTD_00002186	3/103	0,18954614	1	0	0	2,001801621	3,329241919
Tanespimycin_CTD_00003582	2/56	0,195935592	1	0	0	2,454590083	4,000906447
Dimaprit_CTD_00007150	1/15	0,19742532	1	0	0	4,581901489	7,433653572
propanil_CTD_00004257	1/15	0,19742532	1	0	0	4,581901489	7,433653572
alexidine_MCF7_DOWN	3/107	0,204397713	1	0	0	1,926967916	3,059423085
metoprolol_HL60_DOWN	3/107	0,204397713	1	0	0	1,926967916	3,059423085
chrysin_HL60_DOWN	2/58	0,206551617	1	0	0	2,369949046	3,737895338
lomustine_PC3_DOWN	3/109	0,211922254	1	0	0	1,891610707	2,93490173
mometasone_PC3_UP	2/60	0,217227175	1	0	0	2,290950745	3,497850132
terfenadine_HL60_UP	2/60	0,217227175	1	0	0	2,290950745	3,497850132
curcumin II_CTD_00002181	1/17	0,220628154	1	0	0	4,042854255	6,109870857
Diethylcarbamodithioic acid_CTD_00005815	1/17	0,220628154	1	0	0	4,042854255	6,109870857
allopurinol_CTD_00005353	1/17	0,220628154	1	0	0	4,042854255	6,109870857
Phorbol 12,13-dibutyrate_CTD_00007051	1/17	0,220628154	1	0	0	4,042854255	6,109870857
leukotriene D4_CTD_00007224	1/17	0,220628154	1	0	0	4,042854255	6,109870857
Temsirolimus_CTD_00003745	1/17	0,220628154	1	0	0	4,042854255	6,109870857
cyclophosphamide_CTD_00005734	6/281	0,226727864	1	0	0	1,467512932	2,17779626
Ro 41-5253_CTD_00003040	1/18	0,231977645	1	0	0	3,818251241	5,578901374
diltiazem_CTD_00005836	1/18	0,231977645	1	0	0	3,818251241	5,578901374
N-ethylmaleimide_CTD_00005942	1/18	0,231977645	1	0	0	3,818251241	5,578901374
tyrphostin AG-825_MCF7_DOWN	9/462	0,232385291	1	0	0	1,338867318	1,953887464
dequalinium chloride_HL60_DOWN	2/63	0,233325527	1	0	0	2,181857852	3,175302872
orciprenaline_PC3_UP	2/63	0,233325527	1	0	0	2,181857852	3,175302872
Chromium(III) oxide_CTD_00001091	2/63	0,233325527	1	0	0	2,181857852	3,175302872
Glycidamide_CTD_00002776	3/115	0,23483386	1	0	0	1,792917974	2,597717606
oxygen_CTD_00006454	10/525	0,236327697	1	0	0	1,309114711	1,888444955
mycophenolate mofetil_BOSS	2/64	0,238708558	1	0	0	2,147766323	3,076700807
3-Phenyl-5H-indeno[1,2-c]pyridazin-5-one_TTD_00000814	1/19	0,24316242	1	0	0	3,617290649	5,114941807
Zinc sulfate_CTD_00007264	6/288	0,243330382	1	0	0	1,431844215	2,023675775
FCCP_CTD_00005590	2/65	0,24409776	1	0	0	2,114723764	2,982154859
ambroxol_PC3_UP	4/175	0,251180846	1	0	0	1,570937653	2,170379341
0317956-0000_MCF7_DOWN	1/20	0,254184862	1	0	0	3,436426117	4,70685042
AR-A014418_PC3_UP	1/20	0,254184862	1	0	0	3,436426117	4,70685042
TTNPB_MCF7_UP	1/20	0,254184862	1	0	0	3,436426117	4,70685042
diflorasone_PC3_UP	1/20	0,254184862	1	0	0	3,436426117	4,70685042
DIBUTYLTIN DICHLORIDE_CTD_00001008	1/20	0,254184862	1	0	0	3,436426117	4,70685042
NVP-BEZ235_CTD_00004861	1/20	0,254184862	1	0	0	3,436426117	4,70685042
pentetrazol_HL60_DOWN	2/67	0,25488946	1	0	0	2,051597682	2,804380813
luteolin_HL60_DOWN	6/293	0,255404034	1	0	0	1,407410014	1,920985946
5-azacytidine_CTD_00005455	7/353	0,255839174	1	0	0	1,362888545	1,857898196
Chromium(II) chloride_CTD_00000877	2/68	0,260289439	1	0	0	2,021427128	2,720762159
UNII-9XX54M675G_CTD_00004634	2/68	0,260289439	1	0	0	2,021427128	2,720762159
Dasatinib_MRC	1/21	0,265047319	1	0	0	3,272786778	4,345759799
GW559768X_GSK	1/21	0,265047319	1	0	0	3,272786778	4,345759799
formoterol_CTD_00000665	1/21	0,265047319	1	0	0	3,272786778	4,345759799
CP-690334-01_MCF7_DOWN	1/21	0,265047319	1	0	0	3,272786778	4,345759799
metixene_MCF7_UP	1/21	0,265047319	1	0	0	3,272786778	4,345759799
norcyclobenzaprine_MCF7_UP	1/21	0,265047319	1	0	0	3,272786778	4,345759799
securinine_MCF7_UP	1/21	0,265047319	1	0	0	3,272786778	4,345759799
TEPROTIDE_BOSS	1/21	0,265047319	1	0	0	3,272786778	4,345759799
securinine_HL60_DOWN	2/69	0,26569055	1	0	0	1,992131082	2,640416341
parthenolide_HL60_DOWN	4/181	0,270338064	1	0	0	1,518862372	1,986796551
digoxigenin_PC3_DOWN	2/70	0,271091622	1	0	0	1,963672067	2,563178063
tolnaftate_PC3_DOWN	5/240	0,271733382	1	0	0	1,431844215	1,865598376
levonorgestrel_HL60_DOWN	6/300	0,272573587	1	0	0	1,374570447	1,786730798
Dovitinib_RBC	1/22	0,275752105	1	0	0	3,124023743	4,024532923
cinchocaine_PC3_UP	1/22	0,275752105	1	0	0	3,124023743	4,024532923
24939-16-0_CTD_00001660	1/22	0,275752105	1	0	0	3,124023743	4,024532923
labetalol_HL60_UP	1/22	0,275752105	1	0	0	3,124023743	4,024532923
lasalocid_HL60_DOWN	1/22	0,275752105	1	0	0	3,124023743	4,024532923
oxyphenbutazone_MCF7_UP	1/22	0,275752105	1	0	0	3,124023743	4,024532923
4-aminobutyric acid_CTD_00006014	1/22	0,275752105	1	0	0	3,124023743	4,024532923
calyculin A_CTD_00002466	1/22	0,275752105	1	0	0	3,124023743	4,024532923
Muramyl Dipeptide_CTD_00005307	1/22	0,275752105	1	0	0	3,124023743	4,024532923
solanine_HL60_UP	3/127	0,281771426	1	0	0	1,623508402	2,056431662
bucladesine_HL60_DOWN	2/72	0,281889124	1	0	0	1,90912562	2,417414018
emetine_MCF7_DOWN	5/244	0,282883237	1	0	0	1,408371359	1,778380172
Copper(II) chloride_CTD_00001408	4/185	0,283250176	1	0	0	1,486022105	1,874505072
Dinoprostone_CTD_00007049	3/128	0,285727876	1	0	0	1,610824742	2,01790497
indomethacin	1/23	0,2863015	1	0	0	2,988196623	3,737366885
MLN-8054_Kinome Scan	1/23	0,2863015	1	0	0	2,988196623	3,737366885
XMD14-99_LINCS	1/23	0,2863015	1	0	0	2,988196623	3,737366885
BI-2536_LINCS	1/23	0,2863015	1	0	0	2,988196623	3,737366885
AY 9944_CTD_00005454	1/23	0,2863015	1	0	0	2,988196623	3,737366885
carmustine_MCF7_UP	1/23	0,2863015	1	0	0	2,988196623	3,737366885
ethaverine_PC3_UP	1/23	0,2863015	1	0	0	2,988196623	3,737366885
4,5,6,7-tetrabromobenzotriazole_CTD_00003398	1/23	0,2863015	1	0	0	2,988196623	3,737366885

IN1541_CTD_00001481	1/23	0,2863015	1	0	0	2,988196623	3,737366885
Flavopiridol hydrochloride_BOSS	1/23	0,2863015	1	0	0	2,988196623	3,737366885
lonidamine_CTD_00000839	1/23	0,2863015	1	0	0	2,988196623	3,737366885
biotin_CTD_00005513	1/23	0,2863015	1	0	0	2,988196623	3,737366885
Cladribine_BOSS	1/23	0,2863015	1	0	0	2,988196623	3,737366885
flufenamic acid_BOSS	1/23	0,2863015	1	0	0	2,988196623	3,737366885
630-60-4_TTD_00001253	1/23	0,2863015	1	0	0	2,988196623	3,737366885
GNF-PF-3832_TTD_00008267	1/23	0,2863015	1	0	0	2,988196623	3,737366885
benzene_CTD_00005481	11/616	0,286727571	1	0	0	1,227295042	1,533164881
rosiglitazone_PC3_UP	2/73	0,287283376	1	0	0	1,882973215	2,348606461
2-Bromo-3-hydroxy-4-methoxybenzaldehyde_CTD_00004641	3/129	0,289688876	1	0	0	1,598337729	1,980256972
1,5-isoquinolinediol_HL60_DOWN	2/74	0,29267323	1	0	0	1,857527631	2,282341504
perhexiline_HL60_UP	2/74	0,29267323	1	0	0	1,857527631	2,282341504
STOCK1N-35874_PC3_UP	1/24	0,296697753	1	0	0	2,863688431	3,479499787
ifosfamide_MCF7_UP	1/24	0,296697753	1	0	0	2,863688431	3,479499787
minocycline_HL60_DOWN	1/24	0,296697753	1	0	0	2,863688431	3,479499787
papaverine_HL60_UP	1/24	0,296697753	1	0	0	2,863688431	3,479499787
methylprednisolone_BOSS	2/75	0,298057674	1	0	0	1,832760596	2,218498558
Fulvestrant_CTD_00002740	6/311	0,300084382	1	0	0	1,325952199	1,596037487
emetine_HL60_DOWN	8/435	0,300638532	1	0	0	1,263972825	1,519101475
actinomycin D_CTD_00005748	4/191	0,302783842	1	0	0	1,439340782	1,719632424
GW768505A_GSK	1/25	0,306943079	1	0	0	2,749140893	3,246990952
ALW-II-49-7_LINCS	1/25	0,306943079	1	0	0	2,749140893	3,246990952
5194442_MCF7_UP	1/25	0,306943079	1	0	0	2,749140893	3,246990952
5253409_MCF7_UP	1/25	0,306943079	1	0	0	2,749140893	3,246990952
disulfiram_PC3_UP	1/25	0,306943079	1	0	0	2,749140893	3,246990952
metanephrine_PC3_UP	1/25	0,306943079	1	0	0	2,749140893	3,246990952
salbutamol_PC3_UP	1/25	0,306943079	1	0	0	2,749140893	3,246990952
Everolimus_CTD_00003479	1/25	0,306943079	1	0	0	2,749140893	3,246990952
STOCK1N-35215_PC3_UP	2/77	0,308806437	1	0	0	1,785156424	2,097631302
pyrvinium_HL60_DOWN	2/77	0,308806437	1	0	0	1,785156424	2,097631302
tetryzoline_HL60_DOWN	3/135	0,313523676	1	0	0	1,527300496	1,771485909
folic acid_CTD_00005997	4/195	0,315890867	1	0	0	1,409815843	1,624613245
ebselen_MCF7_UP	1/26	0,317039662	1	0	0	2,643404705	3,036554048
ionomycin_MCF7_DOWN	1/26	0,317039662	1	0	0	2,643404705	3,036554048
letrozole_CTD_00002692	1/26	0,317039662	1	0	0	2,643404705	3,036554048
Salbutamol sulfate_BOSS	1/26	0,317039662	1	0	0	2,643404705	3,036554048
GNF-PF-2812_TTD_00008260	1/26	0,317039662	1	0	0	2,643404705	3,036554048
apigenin_HL60_UP	4/196	0,31917573	1	0	0	1,402622905	1,601814225
morphine_CTD_00006352	2/80	0,324865434	1	0	0	1,718213058	1,931862944
withaferin A_PC3_UP	2/80	0,324865434	1	0	0	1,718213058	1,931862944
wortmannin_MCF7_UP	2/80	0,324865434	1	0	0	1,718213058	1,931862944
Bosutinib_MRC	1/27	0,326989654	1	0	0	2,545500827	2,845428911
LY-317615_Kinome Scan	1/27	0,326989654	1	0	0	2,545500827	2,845428911
alexidine_HL60_DOWN	1/27	0,326989654	1	0	0	2,545500827	2,845428911
niclosamide_MCF7_DOWN	1/27	0,326989654	1	0	0	2,545500827	2,845428911
nicotinamide_CTD_00006391	1/27	0,326989654	1	0	0	2,545500827	2,845428911
zuclopenthixol_PC3_DOWN	5/260	0,328239132	1	0	0	1,321702353	1,47239344
ursodiol_CTD_00006973	3/139	0,329445363	1	0	0	1,483349403	1,647029231
hyoscyamine_HL60_DOWN	2/81	0,33019783	1	0	0	1,697000552	1,880384062
ALW-II-38-3_LINCS	1/28	0,336795176	1	0	0	2,454590083	2,671282081
5155877_PC3_UP	1/28	0,336795176	1	0	0	2,454590083	2,671282081
bisindolylmaleimide IX_CTD_00002617	1/28	0,336795176	1	0	0	2,454590083	2,671282081
AC1L1KON_CTD_00000061	1/28	0,336795176	1	0	0	2,454590083	2,671282081
nutlin-3B_BOSS	1/28	0,336795176	1	0	0	2,454590083	2,671282081
dacarbazine_CTD_00005747	1/28	0,336795176	1	0	0	2,454590083	2,671282081
Pentadecafluorooctanoic acid_CTD_00001078	4/204	0,345530837	1	0	0	1,347618085	1,432077873
staurosporine_MCF7_DOWN	11/649	0,345939089	1	0	0	1,164890209	1,236522294
triamterene	1/29	0,346458319	1	0	0	2,369949046	2,512128831
XMD8-85_LINCS	1/29	0,346458319	1	0	0	2,369949046	2,512128831
PEPCID_CTD_00007086	1/29	0,346458319	1	0	0	2,369949046	2,512128831
Compound C (Dorsomorphin)_MRC	1/29	0,346458319	1	0	0	2,369949046	2,512128831
benfluorex_MCF7_UP	1/29	0,346458319	1	0	0	2,369949046	2,512128831
isoxsuprine_PC3_UP	1/29	0,346458319	1	0	0	2,369949046	2,512128831
mefloquine_HL60_UP	1/29	0,346458319	1	0	0	2,369949046	2,512128831
naloxone_PC3_DOWN	1/29	0,346458319	1	0	0	2,369949046	2,512128831
deltamethrin_CTD_00000882	1/29	0,346458319	1	0	0	2,369949046	2,512128831
thiotepa_CTD_00006869	1/29	0,346458319	1	0	0	2,369949046	2,512128831
IMIQUIMOD_BOSS	1/29	0,346458319	1	0	0	2,369949046	2,512128831
Disodium selenite_CTD_00007229	22/1371	0,347948823	1	0	0	1,102864691	1,164294111
ouabain_PC3_DOWN	3/144	0,349334343	1	0	0	1,431844215	1,505907521
0179445-0000_PC3_DOWN	2/85	0,351402882	1	0	0	1,617141702	1,69124221
Lead(II) acetate_CTD_00000394	2/85	0,351402882	1	0	0	1,617141702	1,69124221
SILVER_CTD_00006742	2/85	0,351402882	1	0	0	1,617141702	1,69124221
(-)-isoprenaline_HL60_DOWN	3/145	0,353306287	1	0	0	1,421969428	1,479445332
HC toxin_MCF7_UP	6/332	0,353845117	1	0	0	1,242081729	1,29039372
BI-2536_Kinome Scan	1/30	0,355981142	1	0	0	2,290950745	2,366271525
Indirubin Derivative E804_RBC	1/30	0,355981142	1	0	0	2,290950745	2,366271525
amantadine_PC3_UP	1/30	0,355981142	1	0	0	2,290950745	2,366271525
atovaquone_MCF7_UP	1/30	0,355981142	1	0	0	2,290950745	2,366271525
harmol_MCF7_UP	1/30	0,355981142	1	0	0	2,290950745	2,366271525
monorden_PC3_UP	1/30	0,355981142	1	0	0	2,290950745	2,366271525
tanespimycin_ssMCF7_UP	1/30	0,355981142	1	0	0	2,290950745	2,366271525
tonzonium bromide_PC3_UP	3/146	0,357275364	1	0	0	1,412229911	1,453535472
wortmannin_CTD_00000504	3/147	0,361241191	1	0	0	1,402622905	1,42816386

Gly-His-Lys_PC3_UP	1/31	0,365365677	1	0	0	2,217049108	2,232250463
lasalocid_HL60_UP	1/31	0,365365677	1	0	0	2,217049108	2,232250463
PI-103_CTD_00004750	1/31	0,365365677	1	0	0	2,217049108	2,232250463
vigabatrin_HL60_UP	1/31	0,365365677	1	0	0	2,217049108	2,232250463
vorinostat_HL60_UP	12/727	0,369118391	1	0	0	1,134446036	1,130631851
microcystin RR_CTD_00002594	2/89	0,372373532	1	0	0	1,544461176	1,525708036
Zinc acetate dihydrate_CTD_00007284	3/150	0,373115468	1	0	0	1,374570447	1,355144114
colforsin_PC3_UP	1/32	0,374613925	1	0	0	2,147766323	2,108804372
HMN-176_CTD_00004250	1/32	0,374613925	1	0	0	2,147766323	2,108804372
2,2',3,4,4',5'-HEXACHLOROBIPHENYL_CTD_00001404	1/32	0,374613925	1	0	0	2,147766323	2,108804372
simvastatin_PC3_UP	1/32	0,374613925	1	0	0	2,147766323	2,108804372
ranitidine_CTD_00006691	1/32	0,374613925	1	0	0	2,147766323	2,108804372
GNF-Pf-78_TTD_00008278	1/32	0,374613925	1	0	0	2,147766323	2,108804372
testosterone enanthate_CTD_00000155	9/534	0,374626377	1	0	0	1,158345882	1,137294195
gedunin_CTD_00003449	2/90	0,37757452	1	0	0	1,527300496	1,487571328
helveticoside_PC3_DOWN	2/91	0,382757603	1	0	0	1,510516974	1,450630082
digitoxin	1/33	0,383727859	1	0	0	2,082682495	1,994838443
chloroquine_CTD_00005645	1/33	0,383727859	1	0	0	2,082682495	1,994838443
Pulmicort Nebuamp_BOSS	1/33	0,383727859	1	0	0	2,082682495	1,994838443
HG-9-91-01_LINCS	3/153	0,384947386	1	0	0	1,347618085	1,286501738
tolazoline_PC3_DOWN	3/153	0,384947386	1	0	0	1,347618085	1,286501738
tamoxifen_CTD_00006827	13/803	0,386835066	1	0	0	1,112665991	1,056762161
5-Fluorouracil_CTD_00005987	19/1203	0,38952603	1	0	0	1,085487884	1,023424665
danthron_CTD_00000135	1/34	0,392709422	1	0	0	2,021427128	1,889398271
(+)-isoprenaline_PC3_UP	1/34	0,392709422	1	0	0	2,021427128	1,889398271
bisacodyl_PC3_UP	1/34	0,392709422	1	0	0	2,021427128	1,889398271
3-Aminofluoranthene_CTD_00002071	1/34	0,392709422	1	0	0	2,021427128	1,889398271
flufenamic acid_CTD_00005976	1/34	0,392709422	1	0	0	2,021427128	1,889398271
rosavin_CTD_00004837	1/34	0,392709422	1	0	0	2,021427128	1,889398271
EGTA_CTD_00005889	1/34	0,392709422	1	0	0	2,021427128	1,889398271
terbutaline_PC3_UP	1/34	0,392709422	1	0	0	2,021427128	1,889398271
Salmeterol_CTD_00002421	1/34	0,392709422	1	0	0	2,021427128	1,889398271
Inositol 1,4,5-triphosphate_BOSS	1/34	0,392709422	1	0	0	2,021427128	1,889398271
uridine triphosphate_BOSS	1/34	0,392709422	1	0	0	2,021427128	1,889398271
pyrithyldione_HL60_DOWN	3/155	0,392807188	1	0	0	1,330229465	1,243014835
KIN001-111/A770041_LINCS	2/93	0,393067759	1	0	0	1,478032738	1,380147459
dexibuprofen_PC3_DOWN	4/219	0,394974206	1	0	0	1,255315476	1,166106254
Caspan_CTD_00000180	13/809	0,397061865	1	0	0	1,104413832	1,020106392
Bortezomib	1/35	0,401560531	1	0	0	1,963672067	1,791648495
POLY I-C_CTD_00006579	1/35	0,401560531	1	0	0	1,963672067	1,791648495
Geranylgeranyl pyrophosphate_CTD_00000102	1/35	0,401560531	1	0	0	1,963672067	1,791648495
dipivefrine_HL60_UP	1/35	0,401560531	1	0	0	1,963672067	1,791648495
luteolin_PC3_UP	1/35	0,401560531	1	0	0	1,963672067	1,791648495
pyrantel_HL60_DOWN	1/35	0,401560531	1	0	0	1,963672067	1,791648495
tanespimycin_SKMEL5_UP	1/35	0,401560531	1	0	0	1,963672067	1,791648495
vanillin_CTD_00003324	1/35	0,401560531	1	0	0	1,963672067	1,791648495
beta-D-Glucose 6-phosphate_BOSS	1/35	0,401560531	1	0	0	1,963672067	1,791648495
Pentabromodiphenyl ether_CTD_00003077	7/416	0,402295272	1	0	0	1,156489559	1,053063486
methylene blue_BOSS	2/95	0,403299604	1	0	0	1,44691626	1,313909292
parthenolide_MCF7_UP	4/223	0,408086965	1	0	0	1,232798607	1,104926543
tretinoin_MCF7_UP	2/96	0,408384872	1	0	0	1,431844215	1,282281264
R547_Kinome Scan	1/36	0,410283073	1	0	0	1,90912562	1,700855165
WZ3105_LINCS	1/36	0,410283073	1	0	0	1,90912562	1,700855165
prednisone_CTD_00006606	1/36	0,410283073	1	0	0	1,90912562	1,700855165
leucovorin_CTD_00005678	1/36	0,410283073	1	0	0	1,90912562	1,700855165
Tributyltin_CTD_00000610	1/36	0,410283073	1	0	0	1,90912562	1,700855165
hyoscyamine_HL60_UP	1/36	0,410283073	1	0	0	1,90912562	1,700855165
oxolinic acid_HL60_UP	1/36	0,410283073	1	0	0	1,90912562	1,700855165
primaquine_MCF7_UP	1/36	0,410283073	1	0	0	1,90912562	1,700855165
Vandetanib_CTD_00004046	1/36	0,410283073	1	0	0	1,90912562	1,700855165
9-Anthroic acid_BOSS	1/36	0,410283073	1	0	0	1,90912562	1,700855165
EINECS 250-892-2_CTD_00001193	4/224	0,411356875	1	0	0	1,227295042	1,090198985
ellipticine_HL60_DOWN	3/161	0,41622292	1	0	0	1,280655696	1,122538639
SELENIUM_CTD_00006731	24/1560	0,418510364	1	0	0	1,057361882	0,921018899
Dasatinib_RBC	1/37	0,41887891	1	0	0	1,857527631	1,616371129
IKK Inhibitor VII_MRC	1/37	0,41887891	1	0	0	1,857527631	1,616371129
STOCK1N-35696_PC3_UP	1/37	0,41887891	1	0	0	1,857527631	1,616371129
beclometasone_HL60_UP	1/37	0,41887891	1	0	0	1,857527631	1,616371129
trolox_CTD_00000557	1/37	0,41887891	1	0	0	1,857527631	1,616371129
cloperastine_PC3_DOWN	1/37	0,41887891	1	0	0	1,857527631	1,616371129
disulfiram_HL60_UP	1/37	0,41887891	1	0	0	1,857527631	1,616371129
securinine_PC3_DOWN	1/37	0,41887891	1	0	0	1,857527631	1,616371129
semustine_MCF7_UP	1/37	0,41887891	1	0	0	1,857527631	1,616371129
edaravone_BOSS	1/37	0,41887891	1	0	0	1,857527631	1,616371129
Tenofovir_BOSS	1/37	0,41887891	1	0	0	1,857527631	1,616371129
ambroxol_PC3_DOWN	10/622	0,419301508	1	0	0	1,104960166	0,960392733
POTASSIUM DICHROMATE_CTD_00006598	9/557	0,422288839	1	0	0	1,110514724	0,957336705
alexidine_MCF7_UP	2/99	0,423512333	1	0	0	1,388454997	1,192922549
Vorinostat_CTD_00003560	7/426	0,426058866	1	0	0	1,129341916	0,963529405
Nilotinib_Kinome Scan	1/38	0,427349877	1	0	0	1,808645325	1,537623832
15-delta prostaglandin J2_MCF7_DOWN	1/38	0,427349877	1	0	0	1,808645325	1,537623832
2,2',5,5'-TETRACHLOROBIPHENYL_CTD_00000481	1/38	0,427349877	1	0	0	1,808645325	1,537623832
etilefrine_PC3_UP	1/38	0,427349877	1	0	0	1,808645325	1,537623832
verteporfin_HL60_UP	1/38	0,427349877	1	0	0	1,808645325	1,537623832
HELENALIN_TTD_00008402	1/38	0,427349877	1	0	0	1,808645325	1,537623832

strophanthidin_PC3_DOWN	3/164	0,427823651	1	0	0	1,257229067	1,067443045
okadaic acid_CTD_00007275	2/100	0,428510553	1	0	0	1,374570447	1,164865861
ACMC-20mvek_CTD_00002629	2/101	0,433485939	1	0	0	1,360960838	1,137621611
nitrofural_PC3_DOWN	4/231	0,434131583	1	0	0	1,190104283	0,993032064
Bosutinib_RBC	1/39	0,435697781	1	0	0	1,762269804	1,4641051
Nilotinib_FDA	1/39	0,435697781	1	0	0	1,762269804	1,4641051
naringin_HL60_DOWN	1/39	0,435697781	1	0	0	1,762269804	1,4641051
pyrantel_HL60_UP	1/39	0,435697781	1	0	0	1,762269804	1,4641051
Ephedrone_CTD_00002112	1/39	0,435697781	1	0	0	1,762269804	1,4641051
mitoxantrone_MCF7_DOWN	8/497	0,436366411	1	0	0	1,106294122	0,917419843
sanguinarine_HL60_DOWN	7/431	0,437907509	1	0	0	1,116240502	0,921732868
METHAMPHETAMINE_CTD_00006286	2/102	0,438438097	1	0	0	1,347618085	1,111160496
1,2,4-BENZENETRIOL_CTD_00000682	2/102	0,438438097	1	0	0	1,347618085	1,111160496
MERCURY_CTD_00006274	6/365	0,439264517	1	0	0	1,129783929	0,929420707
Clorgiline_CTD_00005690	3/168	0,443163026	1	0	0	1,227295042	0,99879427
ciclopirox_MCF7_UP	2/103	0,443366649	1	0	0	1,334534414	1,08545451
baclofen_HL60_DOWN	4/234	0,443820496	1	0	0	1,174846536	0,954369063
pentoxifylline_CTD_00006487	1/40	0,443924404	1	0	0	1,718213058	1,395362531
dexverapamil_MCF7_UP	1/40	0,443924404	1	0	0	1,718213058	1,395362531
Clofop [ISO]_CTD_00000269	1/40	0,443924404	1	0	0	1,718213058	1,395362531
1,6,7,8,9,11a,12,13,14,14a-Decahydro-1,13-dihydroxy-6-methyl-4H-cycl	1/40	0,443924404	1	0	0	1,718213058	1,395362531
trifluoperazine_MCF7_UP	1/40	0,443924404	1	0	0	1,718213058	1,395362531
Silica_BOSS	1/40	0,443924404	1	0	0	1,718213058	1,395362531
lycorine_PC3_DOWN	5/302	0,448793107	1	0	0	1,137889443	0,91166938
captopril_HL60_DOWN	3/170	0,450773028	1	0	0	1,212856277	0,966393366
MLN8054_LINCS	1/41	0,452031501	1	0	0	1,676305423	1,33099222
F0447-0125_PC3_UP	1/41	0,452031501	1	0	0	1,676305423	1,33099222
alvespimycin_HL60_UP	1/41	0,452031501	1	0	0	1,676305423	1,33099222
furazolidone_HL60_UP	1/41	0,452031501	1	0	0	1,676305423	1,33099222
mephentermine_HL60_DOWN	1/41	0,452031501	1	0	0	1,676305423	1,33099222
hesperetin_PC3_DOWN	4/237	0,453459469	1	0	0	1,159975061	0,917365565
staurosporine_CTD_00007273	3/171	0,454562238	1	0	0	1,20576355	0,950648625
flunixin_HL60_DOWN	4/238	0,456660573	1	0	0	1,155101216	0,905385535
3,3',4,4',5-Pentachlorobiphenyl_CTD_00001077	4/238	0,456660573	1	0	0	1,155101216	0,905385535
buflomedil_PC3_DOWN	2/106	0,458007118	1	0	0	1,296764572	1,01260527
Imatinib_CTD_00003267	2/106	0,458007118	1	0	0	1,296764572	1,01260527
amantadine_PC3_DOWN	1/42	0,460020804	1	0	0	1,636393389	1,270632571
monorden_HL60_UP	1/42	0,460020804	1	0	0	1,636393389	1,270632571
CHLOROGENIC ACID_CTD_00005640	1/42	0,460020804	1	0	0	1,636393389	1,270632571
rimexolone_HL60_DOWN	1/42	0,460020804	1	0	0	1,636393389	1,270632571
3'-Azido-3'-deoxythymidine_CTD_00007047	6/375	0,464864413	1	0	0	1,099656357	0,842347218
1,10-phenanthroline_CTD_00001181	2/108	0,467643156	1	0	0	1,272750414	0,967353647
calmidazolium_MCF7_DOWN	2/108	0,467643156	1	0	0	1,272750414	0,967353647
5182598_MCF7_UP	1/43	0,467894018	1	0	0	1,598337729	1,213959027
tert-Butylhydroquinone_CTD_00000961	1/43	0,467894018	1	0	0	1,598337729	1,213959027
isotretinoin_HL60_DOWN	1/43	0,467894018	1	0	0	1,598337729	1,213959027
GADOLINIUM_BOSS	1/43	0,467894018	1	0	0	1,598337729	1,213959027
zafirlukast_CTD_00002560	1/44	0,475652825	1	0	0	1,562011871	1,160679553
isoflupredone_PC3_UP	1/44	0,475652825	1	0	0	1,562011871	1,160679553
mevalolactone_HL60_UP	1/44	0,475652825	1	0	0	1,562011871	1,160679553
minocycline_HL60_UP	1/44	0,475652825	1	0	0	1,562011871	1,160679553
mycophenolic acid_MCF7_UP	1/44	0,475652825	1	0	0	1,562011871	1,160679553
Gadodiamide hydrate_CTD_00002623	1/44	0,475652825	1	0	0	1,562011871	1,160679553
oxolinic acid_HL60_DOWN	1/44	0,475652825	1	0	0	1,562011871	1,160679553
pentetrazol_HL60_UP	1/44	0,475652825	1	0	0	1,562011871	1,160679553
BARIUM_CTD_00005464	1/44	0,475652825	1	0	0	1,562011871	1,160679553
loratadine_CTD_00007174	1/44	0,475652825	1	0	0	1,562011871	1,160679553
securinine_HL60_UP	1/44	0,475652825	1	0	0	1,562011871	1,160679553
Lysergide_BOSS	1/44	0,475652825	1	0	0	1,562011871	1,160679553
POTASSIUM_CTD_00006595	1/44	0,475652825	1	0	0	1,562011871	1,160679553
sertraline_CTD_00007358	2/110	0,47717698	1	0	0	1,249609497	0,924545867
puromycin_PC3_DOWN	11/722	0,481467183	1	0	0	1,047110451	0,765351045
kojic acid_CTD_00000624	5/314	0,482498988	1	0	0	1,094403222	0,797575301
PKR Inhibitor_RBC	1/45	0,483298881	1	0	0	1,527300496	1,110530761
HG-5-88-01_LINCS	1/45	0,483298881	1	0	0	1,527300496	1,110530761
SU11652_RBC	1/45	0,483298881	1	0	0	1,527300496	1,110530761
GSK461364_LINCS	1/45	0,483298881	1	0	0	1,527300496	1,110530761
STYRENE_CTD_00001125	1/45	0,483298881	1	0	0	1,527300496	1,110530761
metoprolol_HL60_UP	1/45	0,483298881	1	0	0	1,527300496	1,110530761
semustine_PC3_UP	1/45	0,483298881	1	0	0	1,527300496	1,110530761
strophanthidin_MCF7_UP	1/45	0,483298881	1	0	0	1,527300496	1,110530761
Acetovanillone_CTD_00002374	1/45	0,483298881	1	0	0	1,527300496	1,110530761
Acridine orange_BOSS	1/45	0,483298881	1	0	0	1,527300496	1,110530761
okadaic acid_BOSS	1/45	0,483298881	1	0	0	1,527300496	1,110530761
cicloheximide_PC3_DOWN	4/247	0,485171963	1	0	0	1,113012507	0,804988398
22-Hydroxycholesterol_CTD_00000121	1/46	0,490833819	1	0	0	1,494098312	1,06327456
dorsomorphin_CTD_00004649	1/46	0,490833819	1	0	0	1,494098312	1,06327456
Bromoacetate_CTD_00000832	1/46	0,490833819	1	0	0	1,494098312	1,06327456
Org-2058_CTD_00000993	1/46	0,490833819	1	0	0	1,494098312	1,06327456
colchicine_SKMEL5_UP	1/46	0,490833819	1	0	0	1,494098312	1,06327456
geldanamycin_PC3_UP	1/46	0,490833819	1	0	0	1,494098312	1,06327456
Diquat dication_CTD_00005858	1/46	0,490833819	1	0	0	1,494098312	1,06327456
Bazedoxifene_CTD_00004022	1/46	0,490833819	1	0	0	1,494098312	1,06327456
2-deoxy-D-glucose_BOSS	1/46	0,490833819	1	0	0	1,494098312	1,06327456
montelukast_BOSS	1/46	0,490833819	1	0	0	1,494098312	1,06327456

bilirubin_BOSS	3/182	0,495479593	1	0	0	1,132887731	0,795546743
Sorafenib_CTD_00004146	2/114	0,495929594	1	0	0	1,20576355	0,845627671
pomiferin_CTD_00004186	1/47	0,498259248	1	0	0	1,462308986	1,018695268
1,4-chrysenequinone_HL60_UP	1/47	0,498259248	1	0	0	1,462308986	1,018695268
benserazide_PC3_UP	1/47	0,498259248	1	0	0	1,462308986	1,018695268
Ferriheme chloride_CTD_00006079	1/47	0,498259248	1	0	0	1,462308986	1,018695268
indometacin_PC3_UP	1/47	0,498259248	1	0	0	1,462308986	1,018695268
methylergometrine_PC3_DOWN	1/47	0,498259248	1	0	0	1,462308986	1,018695268
alpha-Neu5Ac_BOSS	1/47	0,498259248	1	0	0	1,462308986	1,018695268
Erlotinib_CTD_00003738	1/47	0,498259248	1	0	0	1,462308986	1,018695268
FURA-2_BOSS	1/47	0,498259248	1	0	0	1,462308986	1,018695268
5,6-BENZOFLAVONE_CTD_00007276	3/183	0,499124558	1	0	0	1,126697087	0,782941354
azacitidine_PC3_DOWN	9/595	0,500392726	1	0	0	1,039591094	0,719773407
cytarabine_CTD_00005743	9/595	0,500392726	1	0	0	1,039591094	0,719773407
LITHOCHOLIC ACID_CTD_00006221	2/116	0,505144702	1	0	0	1,184974523	0,809231368
Cardidigin_CTD_00005822	1/48	0,505576755	1	0	0	1,431844215	0,976597097
mitotane_CTD_00006344	1/48	0,505576755	1	0	0	1,431844215	0,976597097
primidone_PC3_UP	1/48	0,505576755	1	0	0	1,431844215	0,976597097
AMILORIDE_CTD_00005369	1/48	0,505576755	1	0	0	1,431844215	0,976597097
Crizotinib_CTD_00004714	1/48	0,505576755	1	0	0	1,431844215	0,976597097
Sildenafil citrate_BOSS	1/48	0,505576755	1	0	0	1,431844215	0,976597097
disulfiram_CTD_00005860	2/117	0,509711246	1	0	0	1,174846536	0,791741884
fendiline_MCF7_UP	2/117	0,509711246	1	0	0	1,174846536	0,791741884
mitoxantrone_PC3_DOWN	7/463	0,512503213	1	0	0	1,03909213	0,694579367
LY-333531_Kinome Scan	1/49	0,512787903	1	0	0	1,402622905	0,93680197
Staurosporine, N-benzoyl-_RBC	1/49	0,512787903	1	0	0	1,402622905	0,93680197
Go 6976_RBC	1/49	0,512787903	1	0	0	1,402622905	0,93680197
etoposide_HL60_DOWN	1/49	0,512787903	1	0	0	1,402622905	0,93680197
pioglitazone_PC3_UP	1/49	0,512787903	1	0	0	1,402622905	0,93680197
progesterone_HL60_DOWN	1/49	0,512787903	1	0	0	1,402622905	0,93680197
carboplatin_CTD_00007106	1/49	0,512787903	1	0	0	1,402622905	0,93680197
mycophenolic acid_BOSS	1/49	0,512787903	1	0	0	1,402622905	0,93680197
GDC-0941_CTD_00004868	8/534	0,516739953	1	0	0	1,029640784	0,679784828
primaquine_PC3_DOWN	8/535	0,518865911	1	0	0	1,027716222	0,674294674
Cabozantinib_FDA	1/50	0,519894232	1	0	0	1,374570447	0,899147613
lactacystin_CTD_00002698	1/50	0,519894232	1	0	0	1,374570447	0,899147613
(+)-chelidonine_HL60_DOWN	1/50	0,519894232	1	0	0	1,374570447	0,899147613
CP-863187_PC3_DOWN	1/50	0,519894232	1	0	0	1,374570447	0,899147613
STOCK1N-35696_MCF7_UP	1/50	0,519894232	1	0	0	1,374570447	0,899147613
Osajin_CTD_00004306	1/50	0,519894232	1	0	0	1,374570447	0,899147613
monorden_MCF7_UP	1/50	0,519894232	1	0	0	1,374570447	0,899147613
perphenazine_PC3_UP	1/50	0,519894232	1	0	0	1,374570447	0,899147613
Dasatinib_TTD_00007441	1/50	0,519894232	1	0	0	1,374570447	0,899147613
methacholine_BOSS	1/50	0,519894232	1	0	0	1,374570447	0,899147613
ellipticine_PC3_DOWN	6/399	0,524892684	1	0	0	1,033511614	0,666161743
Elesclomol_CTD_00004602	1/51	0,526897261	1	0	0	1,347618085	0,863485884
Leptomycin B_CTD_00001805	1/51	0,526897261	1	0	0	1,347618085	0,863485884
5155877_MCF7_UP	1/51	0,526897261	1	0	0	1,347618085	0,863485884
apigenin_MCF7_UP	1/51	0,526897261	1	0	0	1,347618085	0,863485884
mycophenolic acid_CTD_00006358	1/51	0,526897261	1	0	0	1,347618085	0,863485884
estradiol_CTD_00005920	63/4337	0,529234909	1	0	0	0,998362211	0,63528072
cyclosporin A_BOSS	3/192	0,531311936	1	0	0	1,073883162	0,679130133
staurosporine_TTD_00011086	2/122	0,53212709	1	0	0	1,126697087	0,710802691
SP600125_LINCS	1/52	0,533798486	1	0	0	1,321702353	0,829681308
(-)-isoprenaline_PC3_UP	1/52	0,533798486	1	0	0	1,321702353	0,829681308
dinoprostone_PC3_UP	1/52	0,533798486	1	0	0	1,321702353	0,829681308
isoetarine_HL60_UP	1/52	0,533798486	1	0	0	1,321702353	0,829681308
pararosaniline_MCF7_UP	1/52	0,533798486	1	0	0	1,321702353	0,829681308
budesonide_CTD_00007317	1/52	0,533798486	1	0	0	1,321702353	0,829681308
suloctidil_PC3_UP	1/52	0,533798486	1	0	0	1,321702353	0,829681308
cicloheximide_HL60_DOWN	10/684	0,539022435	1	0	0	1,004802958	0,620966305
amikacin_PC3_DOWN	5/335	0,539637626	1	0	0	1,025798841	0,632771634
LY-294002_HL60_DOWN	1/53	0,540599383	1	0	0	1,296764572	0,797609786
ellipticine_MCF7_UP	1/53	0,540599383	1	0	0	1,296764572	0,797609786
benserazide_PC3_DOWN	2/125	0,545238035	1	0	0	1,099656357	0,66697767
picrotoxinin_PC3_DOWN	3/196	0,545242537	1	0	0	1,051967179	0,638043931
sanguinarine_MCF7_UP	3/196	0,545242537	1	0	0	1,051967179	0,638043931
niclosamide_HL60_DOWN	4/267	0,546201578	1	0	0	1,029640784	0,622692955
Decitabine_CTD_00000750	26/1801	0,547068314	1	0	0	0,992194104	0,598473222
GW-5074_LINCS	1/54	0,547301405	1	0	0	1,272750414	0,767157457
acacetin_HL60_DOWN	1/54	0,547301405	1	0	0	1,272750414	0,767157457
etacrynic acid_MCF7_UP	1/54	0,547301405	1	0	0	1,272750414	0,767157457
indole_BOSS	1/54	0,547301405	1	0	0	1,272750414	0,767157457
Cyclohexanecarboxamide, 4-(1-aminoethyl)-N-4-pyridinyl-, trans-_CTD_[illegible]	1/54	0,547301405	1	0	0	1,272750414	0,767157457
metronidazole_PC3_UP	19/1316	0,548221534	1	0	0	0,992281098	0,596436168
MANGANESE CHLORIDE_CTD_00001187	3/197	0,548687575	1	0	0	1,046627244	0,628212967
clozapine_CTD_00005693	2/126	0,549551226	1	0	0	1,090928926	0,653088187
methotrexate_CTD_00006299	9/620	0,550196622	1	0	0	0,997672098	0,596088697
p-Phenylenediamine_CTD_00001400	1/55	0,553905984	1	0	0	1,249609497	0,738219695
CHEBI:18224_CTD_00001728	1/55	0,553905984	1	0	0	1,249609497	0,738219695
JAK3 Inhibitor VI_RBC	1/55	0,553905984	1	0	0	1,249609497	0,738219695
Vandetanib_Kinome Scan	1/55	0,553905984	1	0	0	1,249609497	0,738219695
Vandetanib_FDA	1/55	0,553905984	1	0	0	1,249609497	0,738219695
3,3',4,4'-Tetrachlorobiphenyl_CTD_00001342	1/55	0,553905984	1	0	0	1,249609497	0,738219695
atovaquone_HL60_DOWN	1/55	0,553905984	1	0	0	1,249609497	0,738219695

verapamil_MCF7_DOWN	1/55	0,553905984	1	0	0	1,249609497	0,738219695
azacitidine_MCF7_DOWN	7/482	0,555170091	1	0	0	0,998132067	0,587381499
Bosutinib	3/199	0,555531692	1	0	0	1,036108377	0,609055193
azathioprine_CTD_00005457	1/56	0,560414533	1	0	0	1,227295042	0,710700211
AZ-628_LINCS	1/56	0,560414533	1	0	0	1,227295042	0,710700211
fluoride_CTD_00005982	1/56	0,560414533	1	0	0	1,227295042	0,710700211
nilutamide_PC3_UP	1/56	0,560414533	1	0	0	1,227295042	0,710700211
cephaeline_MCF7_DOWN	3/201	0,562313807	1	0	0	1,025798841	0,590547479
trichostatin A_PC3_UP	6/415	0,563385623	1	0	0	0,993665383	0,570156196
acetaldehyde_CTD_00005293	2/130	0,566516062	1	0	0	1,057361882	0,600845727
acacetin_HL60_UP	1/57	0,566828442	1	0	0	1,20576355	0,68451027
terfenadine_CTD_00007125	1/57	0,566828442	1	0	0	1,20576355	0,68451027
LITHIUM_CTD_00006220	1/57	0,566828442	1	0	0	1,20576355	0,68451027
parthenolide_PC3_UP	3/203	0,569033103	1	0	0	1,015692448	0,572664333
NSC25485_CTD_00006443	2/131	0,57068495	1	0	0	1,049290417	0,588565853
TAE-684_Kinome Scan	3/204	0,572368956	1	0	0	1,010713564	0,563949331
monobenzone_PC3_DOWN	3/204	0,572368956	1	0	0	1,010713564	0,563949331
digoxin	1/58	0,573149084	1	0	0	1,184974523	0,659567975
0297417-0002B_MCF7_UP	1/58	0,573149084	1	0	0	1,184974523	0,659567975
FERROUS SULFATE_CTD_00001009	1/58	0,573149084	1	0	0	1,184974523	0,659567975
orlistat_CTD_00002339	1/58	0,573149084	1	0	0	1,184974523	0,659567975
taurine_CTD_00006829	1/58	0,573149084	1	0	0	1,184974523	0,659567975
hydroxyurea_BOSS	1/58	0,573149084	1	0	0	1,184974523	0,659567975
co-dergocrine mesilate_HL60_UP	2/132	0,574824803	1	0	0	1,041341248	0,576580209
lanatoside C_PC3_DOWN	2/133	0,57893558	1	0	0	1,033511614	0,564880313
1-Methyl-3-nitro-1-nitrosoguanidine_CTD_00006318	2/133	0,57893558	1	0	0	1,033511614	0,564880313
Tyrphostin B42_CTD_00003234	1/59	0,57937781	1	0	0	1,164890209	0,63579765
Puerarin_CTD_00001616	1/59	0,57937781	1	0	0	1,164890209	0,63579765
GW9662_CTD_00004071	1/59	0,57937781	1	0	0	1,164890209	0,63579765
Everolimus_BOSS	1/59	0,57937781	1	0	0	1,164890209	0,63579765
alpha-Tocopherol_CTD_00007387	1/59	0,57937781	1	0	0	1,164890209	0,63579765
Alvocidib_CTD_00002892	1/59	0,57937781	1	0	0	1,164890209	0,63579765
methylene blue_TTD_00009259	1/59	0,57937781	1	0	0	1,164890209	0,63579765
Caffeic acid phenethyl ester_CTD_00002352	1/60	0,585515952	1	0	0	1,145475372	0,613129268
H-89_PC3_DOWN	1/60	0,585515952	1	0	0	1,145475372	0,613129268
STOCK1N-35215_MCF7_UP	1/60	0,585515952	1	0	0	1,145475372	0,613129268
PONATINIB_CTD_00004976	1/60	0,585515952	1	0	0	1,145475372	0,613129268
zardaverine_HL60_DOWN	1/60	0,585515952	1	0	0	1,145475372	0,613129268
dihydroergotamine_HL60_UP	2/136	0,591093157	1	0	0	1,010713564	0,531414643
isotretinoin_HL60_UP	2/136	0,591093157	1	0	0	1,010713564	0,531414643
INCB18424_Kinome Scan	1/61	0,591564823	1	0	0	1,126697087	0,591497955
F0447-0125_MCF7_UP	1/61	0,591564823	1	0	0	1,126697087	0,591497955
tamoxifen_MCF7_UP	1/61	0,591564823	1	0	0	1,126697087	0,591497955
azathioprine_BOSS	1/61	0,591564823	1	0	0	1,126697087	0,591497955
ouabain_BOSS	1/61	0,591564823	1	0	0	1,126697087	0,591497955
taurine_BOSS	1/61	0,591564823	1	0	0	1,126697087	0,591497955
pyrvinium_HL60_UP	2/137	0,595087368	1	0	0	1,003336093	0,520778636
irinotecan_PC3_UP	7/501	0,596147577	1	0	0	0,960278755	0,49672054
Nickelous acetate_CTD_00003684	1/62	0,597525718	1	0	0	1,108524554	0,570843536
alvespimycin_MCF7_UP	1/62	0,597525718	1	0	0	1,108524554	0,570843536
cefotiam_PC3_UP	1/62	0,597525718	1	0	0	1,108524554	0,570843536
methylene blue_CTD_00006312	1/62	0,597525718	1	0	0	1,108524554	0,570843536
Electrocorundum_CTD_00005364	1/62	0,597525718	1	0	0	1,108524554	0,570843536
Iniprol_BOSS	1/62	0,597525718	1	0	0	1,108524554	0,570843536
Ruxolitinib_FDA	1/63	0,60339991	1	0	0	1,090928926	0,551110131
MANGANESE_CTD_00006240	3/214	0,60483543	1	0	0	0,963483958	0,48443865
Mustard gas_CTD_00006356	3/215	0,60799133	1	0	0	0,959002637	0,477194588
valproic acid_HL60_UP	4/289	0,608758817	1	0	0	0,951259825	0,472141758
Dibenziodolium_CTD_00000351	1/64	0,609188657	1	0	0	1,073883162	0,532245787
digoxin_CTD_00005825	1/64	0,609188657	1	0	0	1,073883162	0,532245787
thiostrepton_MCF7_UP	1/64	0,609188657	1	0	0	1,073883162	0,532245787
valsartan_CTD_00002971	1/64	0,609188657	1	0	0	1,073883162	0,532245787
thalidomide_CTD_00006858	2/142	0,614620935	1	0	0	0,968007357	0,471177162
arecoline_CTD_00005439	1/65	0,614893198	1	0	0	1,057361882	0,514202155
15-delta prostaglandin J2_PC3_UP	1/65	0,614893198	1	0	0	1,057361882	0,514202155
papaverine_HL60_DOWN	1/65	0,614893198	1	0	0	1,057361882	0,514202155
thioridazine_HL60_UP	1/65	0,614893198	1	0	0	1,057361882	0,514202155
Calcium phosphate_BOSS	1/65	0,614893198	1	0	0	1,057361882	0,514202155
sulfaguanidine_PC3_DOWN	8/583	0,616408938	1	0	0	0,943101507	0,456314643
glibenclamide_HL60_UP	5/366	0,618193755	1	0	0	0,93891424	0,45157395
Nilotinib	1/66	0,620514753	1	0	0	1,041341248	0,496934185
DL-Mevalonic acid_CTD_00006329	1/66	0,620514753	1	0	0	1,041341248	0,496934185
corbadrine_HL60_UP	1/66	0,620514753	1	0	0	1,041341248	0,496934185
carvedilol_CTD_00001961	1/66	0,620514753	1	0	0	1,041341248	0,496934185
VANADIUM PENTOXIDE_CTD_00002655	1/66	0,620514753	1	0	0	1,041341248	0,496934185
ellipticine_MCF7_DOWN	7/515	0,625082669	1	0	0	0,93417409	0,438941657
HG-6-64-01_LINCS	2/145	0,625991632	1	0	0	0,947979618	0,444050978
chlortetracycline_PC3_UP	1/67	0,626054526	1	0	0	1,025798841	0,480399866
ciclopirox_MCF7_DOWN	1/67	0,626054526	1	0	0	1,025798841	0,480399866
Masoprocol_CTD_00006416	1/67	0,626054526	1	0	0	1,025798841	0,480399866
FITC_BOSS	1/67	0,626054526	1	0	0	1,025798841	0,480399866
TITANIUM DIOXIDE_CTD_00000489	2/146	0,6297238	1	0	0	0,941486607	0,435413048
minoxidil_PC3_UP	5/371	0,630106344	1	0	0	0,926260409	0,427808815
5109870_MCF7_DOWN	1/68	0,631513702	1	0	0	1,010713564	0,464559976
beclomethasone_CTD_00005468	1/68	0,631513702	1	0	0	1,010713564	0,464559976

lomustine_MCF7_UP	1/68	0,631513702	1	0	0	1,010713564	0,464559976
Calcifediol_BOSS	1/68	0,631513702	1	0	0	1,010713564	0,464559976
3-methylcholanthrene_CTD_00006310	4/298	0,632782615	1	0	0	0,922530501	0,422176098
etoposide_HL60_UP	2/147	0,633427005	1	0	0	0,935081937	0,426968241
Mehp_CTD_00000849	1/69	0,63689345	1	0	0	0,996065541	0,449377864
histamine_CTD_00006100	1/69	0,63689345	1	0	0	0,996065541	0,449377864
disulfiram_MCF7_UP	1/69	0,63689345	1	0	0	0,996065541	0,449377864
glycocholic acid_PC3_DOWN	1/69	0,63689345	1	0	0	0,996065541	0,449377864
gossypol_CTD_00006052	1/69	0,63689345	1	0	0	0,996065541	0,449377864
spiperone_PC3_DOWN	1/69	0,63689345	1	0	0	0,996065541	0,449377864
trichostatin A_HL60_UP	9/667	0,637917324	1	0	0	0,927371366	0,416896635
[6-[6-(butanoylamino)purin-9-yl]-2-hydroxy-2-oxo-4a,6,7,7a-tetrahydro-4l	3/225	0,638622614	1	0	0	0,916380298	0,410943035
CHROMIUM_CTD_00005668	2/149	0,640746733	1	0	0	0,922530501	0,410637711
lomustine_PC3_UP	2/149	0,640746733	1	0	0	0,922530501	0,410637711
quercetin_HL60_UP	1/70	0,642194921	1	0	0	0,981836033	0,43481925
tonzonium bromide_PC3_DOWN	1/70	0,642194921	1	0	0	0,981836033	0,43481925
tretinoin_HL60_DOWN	1/70	0,642194921	1	0	0	0,981836033	0,43481925
brefeldin A_BOSS	1/70	0,642194921	1	0	0	0,981836033	0,43481925
digitoxigenin_MCF7_DOWN	5/377	0,644102289	1	0	0	0,911518864	0,400975081
thioguanosine_MCF7_DOWN	2/150	0,644363368	1	0	0	0,916380298	0,402742245
GW-8510_MCF7_UP	1/71	0,647419251	1	0	0	0,968007357	0,420852043
clotrimazole_CTD_00005691	1/71	0,647419251	1	0	0	0,968007357	0,420852043
nitrofural_PC3_UP	1/71	0,647419251	1	0	0	0,968007357	0,420852043
pipemidic acid_PC3_DOWN	1/71	0,647419251	1	0	0	0,968007357	0,420852043
Fludarabine_CTD_00001135	1/71	0,647419251	1	0	0	0,968007357	0,420852043
camptothecin_BOSS	1/71	0,647419251	1	0	0	0,968007357	0,420852043
HC toxin_MCF7_DOWN	2/151	0,647951268	1	0	0	0,910311554	0,395020404
rifabutin_PC3_UP	2/151	0,647951268	1	0	0	0,910311554	0,395020404
chlorzoxazone_HL60_UP	6/455	0,652501324	1	0	0	0,906310185	0,386941984
0297417-0002B_PC3_DOWN	1/72	0,652567558	1	0	0	0,95456281	0,407446171
fluoxetine_CTD_00005988	1/72	0,652567558	1	0	0	0,95456281	0,407446171
gossypol_HL60_UP	1/72	0,652567558	1	0	0	0,95456281	0,407446171
harmine_PC3_UP	1/72	0,652567558	1	0	0	0,95456281	0,407446171
levonorgestrel_HL60_UP	3/231	0,656182775	1	0	0	0,892578212	0,3760574
TBTO_CTD_00000264	1/73	0,657640944	1	0	0	0,941486607	0,394573435
PHA-00767505E_MCF7_DOWN	1/73	0,657640944	1	0	0	0,941486607	0,394573435
benzocaine_PC3_DOWN	1/73	0,657640944	1	0	0	0,941486607	0,394573435
phenoxybenzamine_PC3_UP	2/154	0,658543246	1	0	0	0,892578212	0,372852311
sanguinarine_HL60_UP	5/384	0,660009474	1	0	0	0,894902635	0,37183302
thioridazine_MCF7_UP	2/155	0,662016917	1	0	0	0,886819643	0,365781327
imidurea_PC3_DOWN	1/74	0,662640498	1	0	0	0,928763815	0,382207367
carbachol_BOSS	1/74	0,662640498	1	0	0	0,928763815	0,382207367
diethylstilbestrol_CTD_00005818	3/234	0,664730932	1	0	0	0,881134902	0,359831645
ns-398_CTD_00002958	1/75	0,667567288	1	0	0	0,916380298	0,370323105
piperlongumine_HL60_DOWN	5/389	0,671089042	1	0	0	0,88340003	0,35234715
vorinostat_MCF7_UP	8/613	0,671786963	1	0	0	0,896946458	0,356817865
N-NITROSODIETHYLAMINE_CTD_00005817	2/158	0,672268127	1	0	0	0,869981295	0,345467849
canavanine_MCF7_DOWN	1/76	0,672422371	1	0	0	0,904322662	0,358897276
tanespimycin_HL60_UP	1/76	0,672422371	1	0	0	0,904322662	0,358897276
withaferin A_MCF7_UP	1/76	0,672422371	1	0	0	0,904322662	0,358897276
Medroxyprogesterone acetate_CTD_00006623	5/390	0,673276394	1	0	0	0,881134902	0,348576389
luteolin_HL60_UP	2/159	0,675628897	1	0	0	0,864509715	0,338984048
Panobinostat_CTD_00004412	1/77	0,677206786	1	0	0	0,892578212	0,347907893
vanoxerine_PC3_UP	1/77	0,677206786	1	0	0	0,892578212	0,347907893
Pyrrolidine dithiocarbamate_CTD_00001021	2/160	0,678961675	1	0	0	0,859106529	0,332637969
8-HYDROXYQUINOLINE_CTD_00007045	3/240	0,681362756	1	0	0	0,859106529	0,329605184
mesalazine_MCF7_DOWN	1/78	0,681921559	1	0	0	0,881134902	0,337334253
primidone_PC3_DOWN	1/78	0,681921559	1	0	0	0,881134902	0,337334253
quercetin_PC3_UP	1/78	0,681921559	1	0	0	0,881134902	0,337334253
ellagic acid_TTD_00007809	1/78	0,681921559	1	0	0	0,881134902	0,337334253
digitoxigenin_PC3_DOWN	9/694	0,68386136	1	0	0	0,891292076	0,338691053
apigenin_HL60_DOWN	5/396	0,686199071	1	0	0	0,867784373	0,32679675
gefitinib_HL60_DOWN	1/79	0,686567701	1	0	0	0,869981295	0,32715685
puromycin_MCF7_UP	3/242	0,686769204	1	0	0	0,852006475	0,32014739
geldanamycin_HL60_UP	1/80	0,691146205	1	0	0	0,859106529	0,317357296
tanespimycin_PC3_UP	1/80	0,691146205	1	0	0	0,859106529	0,317357296
ANTIMONY_CTD_00005424	1/80	0,691146205	1	0	0	0,859106529	0,317357296
CARBON TETRACHLORIDE_BOSS	1/80	0,691146205	1	0	0	0,859106529	0,317357296
quinpirole_HL60_DOWN	3/245	0,694750294	1	0	0	0,841573743	0,306503503
bromocriptine_HL60_UP	1/81	0,695658055	1	0	0	0,848500276	0,307918238
Bathocuproine disulfonate_CTD_00001350	1/81	0,695658055	1	0	0	0,848500276	0,307918238
biotin_BOSS	1/81	0,695658055	1	0	0	0,848500276	0,307918238
gabexate_PC3_DOWN	2/166	0,698376326	1	0	0	0,828054486	0,297269219
cinnamaldehyde_CTD_00000671	2/166	0,698376326	1	0	0	0,828054486	0,297269219
pregnenolone_PC3_DOWN	4/325	0,698987993	1	0	0	0,845889506	0,302931399
TRICHLOROETHYLENE_CTD_00006932	1/82	0,700104217	1	0	0	0,838152711	0,298823295
Dasatinib_Kinome Scan	1/82	0,700104217	1	0	0	0,838152711	0,298823295
Dasatinib_FDA	1/82	0,700104217	1	0	0	0,838152711	0,298823295
dihydroergocristine_HL60_UP	1/82	0,700104217	1	0	0	0,838152711	0,298823295
rapamycin_CTD_00007350	3/248	0,702577524	1	0	0	0,831393415	0,293481485
Palbociclib_FDA	1/83	0,704485644	1	0	0	0,828054486	0,290056991
PD0332991_LINCS	1/83	0,704485644	1	0	0	0,828054486	0,290056991
SB 218078_RBC	1/83	0,704485644	1	0	0	0,828054486	0,290056991
Staurosporine_MRC	1/83	0,704485644	1	0	0	0,828054486	0,290056991
Apocarotenal_CTD_00000185	1/83	0,704485644	1	0	0	0,828054486	0,290056991

Vorinostat_BOSS	1/83	0,704485644	1	0	0	0,828054486	0,290056991
diphenylpyraline_BOSS	2/168	0,704628809	1	0	0	0,818196694	0,286437676
staurosporine_MCF7_UP	1/84	0,708803276	1	0	0	0,818196694	0,281604695
doxorubicin_MCF7_DOWN	13/1006	0,70903079	1	0	0	0,888141939	0,305393224
NSC94017_CTD_00005320	1/85	0,713058038	1	0	0	0,808570851	0,273452568
Rubidomycin hydrochloride	1/85	0,713058038	1	0	0	0,808570851	0,273452568
acetoacetic acid_BOSS	1/85	0,713058038	1	0	0	0,808570851	0,273452568
ouabain_MCF7_DOWN	6/486	0,713384897	1	0	0	0,848500276	0,286567543
5109870_MCF7_UP	2/171	0,713805405	1	0	0	0,803842367	0,27101135
bicalutamide_CTD_00002279	2/171	0,713805405	1	0	0	0,803842367	0,27101135
WH-4-025_LINCS	1/86	0,717250842	1	0	0	0,799168864	0,265587509
GSK1059615_LINCS	1/86	0,717250842	1	0	0	0,799168864	0,265587509
rifabutin_PC3_DOWN	1/86	0,717250842	1	0	0	0,799168864	0,265587509
Adenosine triphosphate_CTD_00005324	1/86	0,717250842	1	0	0	0,799168864	0,265587509
Edetate sodium_BOSS	1/86	0,717250842	1	0	0	0,799168864	0,265587509
Vincristine sulfate_BOSS	1/86	0,717250842	1	0	0	0,799168864	0,265587509
AZD7762_LINCS	3/254	0,717773022	1	0	0	0,811754201	0,269179224
cantharidin_HL60_DOWN	3/254	0,717773022	1	0	0	0,811754201	0,269179224
TPEN_CTD_00001994	3/254	0,717773022	1	0	0	0,811754201	0,269179224
Irinotecan hydrochloride_CTD_00002224	7/566	0,720074342	1	0	0	0,849999393	0,279140497
minoxidil_PC3_DOWN	3/255	0,720246455	1	0	0	0,808570851	0,265342088
mifepristone_CTD_00007083	4/335	0,721226761	1	0	0	0,820639073	0,268186229
chitosamine_CTD_00006030	1/87	0,721382588	1	0	0	0,789983015	0,257997115
alsterpaullone_PC3_UP	1/87	0,721382588	1	0	0	0,789983015	0,257997115
adenine_CTD_00005317	1/87	0,721382588	1	0	0	0,789983015	0,257997115
felodipine_MCF7_UP	1/87	0,721382588	1	0	0	0,789983015	0,257997115
Spectrum_001666_CTD_00000796	1/87	0,721382588	1	0	0	0,789983015	0,257997115
tanespimycin_MCF7_UP	1/87	0,721382588	1	0	0	0,789983015	0,257997115
calcitriol_BOSS	1/87	0,721382588	1	0	0	0,789983015	0,257997115
alsterpaullone_MCF7_UP	3/256	0,722703078	1	0	0	0,805412371	0,261563161
Gefitinib	1/88	0,725454161	1	0	0	0,781005936	0,250669627
GW-8510_PC3_UP	1/88	0,725454161	1	0	0	0,781005936	0,250669627
ANTIMONY POTASSIUM TARTRATE_CTD_00005425	1/88	0,725454161	1	0	0	0,781005936	0,250669627
Sunitinib	3/258	0,727566045	1	0	0	0,799168864	0,254176057
paclitaxel_CTD_00007144	6/494	0,727853963	1	0	0	0,834759381	0,265165367
midecamycin_HL60_UP	2/176	0,728569192	1	0	0	0,781005936	0,247323243
ALDOSTERONE_BOSS	2/176	0,728569192	1	0	0	0,781005936	0,247323243
paclitaxel_PC3_DOWN	5/417	0,728680137	1	0	0	0,824083002	0,260839092
IN1152_CTD_00001901	1/89	0,729466436	1	0	0	0,772230588	0,2435939
IBMX_CTD_00007018	1/89	0,729466436	1	0	0	0,772230588	0,2435939
metoclopramide_HL60_DOWN	1/89	0,729466436	1	0	0	0,772230588	0,2435939
emetine_PC3_DOWN	3/260	0,732362235	1	0	0	0,793021412	0,247010333
vincristine_CTD_00006988	16/1246	0,732922363	1	0	0	0,882549243	0,274221728
Ponatinib_FDA	1/90	0,733420273	1	0	0	0,763650248	0,236759359
azacyclonol_PC3_UP	1/90	0,733420273	1	0	0	0,763650248	0,236759359
Octa-2,4,6-trienoic acid_CTD_00002551	1/90	0,733420273	1	0	0	0,763650248	0,236759359
Dichloromercury_CTD_00006273	1/90	0,733420273	1	0	0	0,763650248	0,236759359
pergolide_HL60_DOWN	4/341	0,733977444	1	0	0	0,806199676	0,249339001
niclosamide_HL60_UP	2/178	0,734292111	1	0	0	0,772230588	0,238502149
CALCIUM_CTD_00005559	2/179	0,737114993	1	0	0	0,767916451	0,234223249
GEMCITABINE HYDROCHLORIDE_BOSS	2/179	0,737114993	1	0	0	0,767916451	0,234223249
pimozide_MCF7_UP	1/91	0,73731652	1	0	0	0,755258487	0,230155967
chrysin_CTD_00001976	1/91	0,73731652	1	0	0	0,755258487	0,230155967
lanatoside C_MCF7_DOWN	8/653	0,737654683	1	0	0	0,842003336	0,256204332
digoxin_PC3_DOWN	2/180	0,739912343	1	0	0	0,763650248	0,230029443
PhIP_CTD_00002156	2/180	0,739912343	1	0	0	0,763650248	0,230029443
histamine_BOSS	2/180	0,739912343	1	0	0	0,763650248	0,230029443
clotrimazole_PC3_UP	1/92	0,741156013	1	0	0	0,747049156	0,223774191
pyrvinium_MCF7_DOWN	1/92	0,741156013	1	0	0	0,747049156	0,223774191
9,10-Phenanthrenequinone_TTD_00001360	1/92	0,741156013	1	0	0	0,747049156	0,223774191
hemin_BOSS	2/181	0,742684304	1	0	0	0,759431186	0,225918793
Diallyl trisulfide_CTD_00001934	1/93	0,744939576	1	0	0	0,739016369	0,217604974
hexachlorophene_CTD_00006092	1/93	0,744939576	1	0	0	0,739016369	0,217604974
(-)-isoprenaline_HL60_UP	2/182	0,745431019	1	0	0	0,755258487	0,221889414
PROPIDIUM_BOSS	2/182	0,745431019	1	0	0	0,755258487	0,221889414
tolnaftate_PC3_UP	3/266	0,746353589	1	0	0	0,775133711	0,226769871
rifabutin_MCF7_UP	3/267	0,748628041	1	0	0	0,772230588	0,223570814
Deguelin_CTD_00003487	1/94	0,748668021	1	0	0	0,731154493	0,211639704
valinomycin_MCF7_UP	1/94	0,748668021	1	0	0	0,731154493	0,211639704
KW-2449_Kinome Scan	2/184	0,750849295	1	0	0	0,747049156	0,214067175
Cdk1/2 Inhibitor III_RBC	1/95	0,752342148	1	0	0	0,72345813	0,205870193
chlortetracycline_PC3_DOWN	1/95	0,752342148	1	0	0	0,72345813	0,205870193
geldanamycin_MCF7_UP	1/95	0,752342148	1	0	0	0,72345813	0,205870193
thioguanosine_MCF7_UP	1/95	0,752342148	1	0	0	0,72345813	0,205870193
valinomycin_PC3_UP	1/95	0,752342148	1	0	0	0,72345813	0,205870193
UNII-768N7QO4KH_BOSS	1/95	0,752342148	1	0	0	0,72345813	0,205870193
15-delta prostaglandin J2_HL60_UP	2/185	0,753521147	1	0	0	0,743011052	0,210270788
5224221_MCF7_UP	1/96	0,755962746	1	0	0	0,715922108	0,200288647
Gefitinib_CTD_00003879	1/96	0,755962746	1	0	0	0,715922108	0,200288647
thapsigargin_BOSS	1/96	0,755962746	1	0	0	0,715922108	0,200288647
GNF-Pf-2272_TTD_00008257	1/96	0,755962746	1	0	0	0,715922108	0,200288647
carbon monoxide_BOSS	1/96	0,755962746	1	0	0	0,715922108	0,200288647
TERT-BUTYL HYDROPEROXIDE_CTD_00007349	17/1342	0,757378602	1	0	0	0,870629568	0,241941006
Quercetin dihydrate_BOSS	2/187	0,758791022	1	0	0	0,73506441	0,202899001
15-delta prostaglandin J2_HL60_DOWN	1/97	0,759530592	1	0	0	0,708541467	0,194887646

apigenin_PC3_DOWN	1/97	0,759530592	1	0	0	0,708541467	0,194887646
phenylephrine_BOSS	1/97	0,759530592	1	0	0	0,708541467	0,194887646
beta-D-allopyranose_BOSS	1/97	0,759530592	1	0	0	0,708541467	0,194887646
hydrocortisone_CTD_00006117	1/98	0,763046452	1	0	0	0,701311452	0,189660123
lovastatin_CTD_00006225	1/98	0,763046452	1	0	0	0,701311452	0,189660123
chloroquine_BOSS	1/98	0,763046452	1	0	0	0,701311452	0,189660123
prednisolone_BOSS	1/98	0,763046452	1	0	0	0,701311452	0,189660123
camptothecin_PC3_UP	5/437	0,765152238	1	0	0	0,786367532	0,210495224
azacyclonol_HL60_UP	3/275	0,766242041	1	0	0	0,749765698	0,199630499
0297417-0002B_MCF7_DOWN	1/99	0,76651108	1	0	0	0,694227498	0,184599344
33258 Hoechst_BOSS	1/99	0,76651108	1	0	0	0,694227498	0,184599344
doxorubicin_CTD_00005874	9/751	0,76847582	1	0	0	0,823644076	0,216903521
ciglitazone_CTD_00001835	2/191	0,769039655	1	0	0	0,719670391	0,188994616
phenoxybenzamine_MCF7_UP	2/191	0,769039655	1	0	0	0,719670391	0,188994616
AGN-PC-0JHFVD_BOSS	2/191	0,769039655	1	0	0	0,719670391	0,188994616
nitric oxide_BOSS	2/191	0,769039655	1	0	0	0,719670391	0,188994616
trimethoprim_BOSS	2/191	0,769039655	1	0	0	0,719670391	0,188994616
alexidine_HL60_UP	1/100	0,769925222	1	0	0	0,687285223	0,179698889
hesperidin_PC3_DOWN	1/100	0,769925222	1	0	0	0,687285223	0,179698889
Hydroxyzine dihydrochloride_BOSS	1/100	0,769925222	1	0	0	0,687285223	0,179698889
Toxoflavin_TTD_00011503	1/100	0,769925222	1	0	0	0,687285223	0,179698889
Calcimycin_CTD_00005287	1/100	0,769925222	1	0	0	0,687285223	0,179698889
PHA-767491_LINCS	1/101	0,77328961	1	0	0	0,680480419	0,174952634
Crizotinib_Kinome Scan	1/101	0,77328961	1	0	0	0,680480419	0,174952634
cerivastatin_BOSS	2/193	0,774020842	1	0	0	0,712212667	0,182437888
Angiotensin II, human_BOSS	2/193	0,774020842	1	0	0	0,712212667	0,182437888
ethosuximide_HL60_DOWN	2/194	0,77647619	1	0	0	0,708541467	0,17925341
furosemide_BOSS	2/194	0,77647619	1	0	0	0,708541467	0,17925341
Aizen uranine_BOSS	2/194	0,77647619	1	0	0	0,708541467	0,17925341
losartan_BOSS	1/102	0,776604966	1	0	0	0,673809043	0,170354738
Phenethyl isothiocyanate_CTD_00002443	1/102	0,776604966	1	0	0	0,673809043	0,170354738
melphalan_CTD_00006262	3/280	0,776734223	1	0	0	0,736377025	0,186050841
CHIR-258/TKI-258_Kinome Scan	1/103	0,779872003	1	0	0	0,667267207	0,165899624
R-atenolol_PC3_UP	2/196	0,781317169	1	0	0	0,701311452	0,173065507
phenol_CTD_00007305	1/104	0,783091423	1	0	0	0,660851176	0,161581965
Doxorubicin Hydrochloride	1/104	0,783091423	1	0	0	0,660851176	0,161581965
phenoxybenzamine_PC3_DOWN	1/104	0,783091423	1	0	0	0,660851176	0,161581965
curcumin_CTD_00000663	6/529	0,785099864	1	0	0	0,779529554	0,188602775
0175029-0000_MCF7_UP	4/368	0,785962593	1	0	0	0,747049156	0,17992386
cyclophosphamide_BOSS	2/198	0,786066226	1	0	0	0,694227498	0,16711044
rapamycin_BOSS	2/198	0,786066226	1	0	0	0,694227498	0,16711044
raloxifene_CTD_00007367	8/687	0,786088313	1	0	0	0,800332138	0,192628849
Axitinib	1/105	0,786263917	1	0	0	0,654557356	0,157396675
Decabromodiphenyl oxide_CTD_00000574	1/105	0,786263917	1	0	0	0,654557356	0,157396675
bromocriptine_HL60_DOWN	1/105	0,786263917	1	0	0	0,654557356	0,157396675
cholecalciferol_CTD_00005655	1/105	0,786263917	1	0	0	0,654557356	0,157396675
picrotoxinin_HL60_DOWN	1/105	0,786263917	1	0	0	0,654557356	0,157396675
tyrphostin AG-1478_MCF7_UP	1/105	0,786263917	1	0	0	0,654557356	0,157396675
beta-carotene_CTD_00007253	1/105	0,786263917	1	0	0	0,654557356	0,157396675
MAGNESIUM_BOSS	1/105	0,786263917	1	0	0	0,654557356	0,157396675
Dasatinib_BOSS	1/106	0,789390167	1	0	0	0,648382286	0,153338891
doxorubicin_BOSS	2/200	0,790724612	1	0	0	0,687285223	0,161378367
chlortetracycline_HL60_UP	3/289	0,794646933	1	0	0	0,713444869	0,163990563
Chlorpyrifos oxon_CTD_00000500	1/108	0,795506613	1	0	0	0,636375207	0,14558745
OTSSP167_MRC	1/108	0,795506613	1	0	0	0,636375207	0,14558745
CHOLINE HYDROXIDE_BOSS	1/108	0,795506613	1	0	0	0,636375207	0,14558745
SODIUM BICARBONATE_BOSS	1/108	0,795506613	1	0	0	0,636375207	0,14558745
9,10-Phenanthrenequinone_CTD_00000222	1/108	0,795506613	1	0	0	0,636375207	0,14558745
co-dergocrine mesilate_HL60_DOWN	2/203	0,797544923	1	0	0	0,677128299	0,153178011
Allococaine_CTD_00005697	3/291	0,798461434	1	0	0	0,708541467	0,159470444
clonidine_HL60_DOWN	1/109	0,798498123	1	0	0	0,630536902	0,141885092
HG-14-10-04_LINCS	2/204	0,799774385	1	0	0	0,673809043	0,150546197
phenytoin_CTD_00006527	1/110	0,801446018	1	0	0	0,624804749	0,138292821
temozolomide_CTD_00002088	2/205	0,80198212	1	0	0	0,670522169	0,147963433
cicloheximide_PC3_UP	4/378	0,803048145	1	0	0	0,727285951	0,159523344
hexachlorophene	1/111	0,804350932	1	0	0	0,619175877	0,134806738
docetaxel_CTD_00002688	1/111	0,804350932	1	0	0	0,619175877	0,134806738
prenylamine_PC3_UP	1/111	0,804350932	1	0	0	0,619175877	0,134806738
etoposide_CTD_00005948	5/462	0,805393854	1	0	0	0,743815177	0,160979353
8-azaguanine_PC3_DOWN	2/207	0,806333046	1	0	0	0,664043694	0,142940992
Pioglitazone_CTD_00002515	1/112	0,80721349	1	0	0	0,613647521	0,131423109
sulforaphane_CTD_00000857	1/112	0,80721349	1	0	0	0,613647521	0,131423109
dimethyl sulfoxide_CTD_00005842	2/208	0,80847655	1	0	0	0,660851176	0,140499342
amiodarone_CTD_00005381	7/626	0,809220379	1	0	0	0,768529802	0,162685455
ionomycin_CTD_00007090	1/113	0,810034307	1	0	0	0,608217012	0,128138356
menadione_PC3_UP	1/113	0,810034307	1	0	0	0,608217012	0,128138356
Sodium dichromate_CTD_00000827	2/209	0,81059896	1	0	0	0,657689209	0,138102796
NVP-TAE684_LINCS	2/212	0,81684119	1	0	0	0,648382286	0,1311746
(-)-Epigallocatechin gallate_BOSS	2/212	0,81684119	1	0	0	0,648382286	0,1311746
puromycin_PC3_UP	6/552	0,817464404	1	0	0	0,747049156	0,150566203
Sorafenib	1/116	0,818252334	1	0	0	0,592487262	0,118843769
PD-173955_Kinome Scan	1/116	0,818252334	1	0	0	0,592487262	0,118843769
AM580_CTD_00002706	1/116	0,818252334	1	0	0	0,592487262	0,118843769
caffeine_CTD_00005556	1/116	0,818252334	1	0	0	0,592487262	0,118843769
Alitretinoin_CTD_00003402	2/213	0,818880791	1	0	0	0,645338238	0,128949396

PARAQUAT_CTD_00006471	2/214	0,820900083	1	0	0	0,642322639	0,126764864
Vandetanib	1/117	0,820912166	1	0	0	0,587423268	0,115921614
SKI-606_Kinome Scan	1/117	0,820912166	1	0	0	0,587423268	0,115921614
celecoxib_CTD_00003448	1/117	0,820912166	1	0	0	0,587423268	0,115921614
suloctidil_MCF7_UP	1/117	0,820912166	1	0	0	0,587423268	0,115921614
helveticoside_MCF7_DOWN	6/555	0,8213897	1	0	0	0,743011052	0,146193084
HG-5-113-01_LINCS	1/118	0,823533203	1	0	0	0,582445105	0,113082539
anisomycin_PC3_DOWN	6/558	0,825248267	1	0	0	0,739016369	0,141943619
1,4-chrysenequinone_PC3_UP	1/119	0,826116009	1	0	0	0,577550608	0,110323757
Nandrolone phenpropionate_BOSS	1/119	0,826116009	1	0	0	0,577550608	0,110323757
Tamibarotene_CTD_00002527	6/560	0,827783845	1	0	0	0,736377025	0,139177625
Deoxycorticosterone acetate_BOSS	1/120	0,828661141	1	0	0	0,572737686	0,107642591
mitomycin C_CTD_00007136	3/309	0,830195377	1	0	0	0,667267207	0,124174565
PP-242_Kinome Scan	1/121	0,831169145	1	0	0	0,568004317	0,105036472
luteolin_CTD_00007430	1/121	0,831169145	1	0	0	0,568004317	0,105036472
zalcitabine_PC3_DOWN	8/726	0,833186171	1	0	0	0,757339089	0,138212995
15-delta prostaglandin J2_MCF7_UP	1/122	0,833640562	1	0	0	0,563348544	0,10250293
perhexiline_MCF7_UP	1/123	0,836075923	1	0	0	0,558768474	0,100039591
deferoxamine_CTD_00005759	1/124	0,838475753	1	0	0	0,554262277	0,097644172
tolazoline_PC3_UP	2/224	0,840010319	1	0	0	0,613647521	0,106983985
PKC-412_Kinome Scan	1/125	0,840840568	1	0	0	0,549828179	0,095314481
CGP74514A_LINCS	1/125	0,840840568	1	0	0	0,549828179	0,095314481
Dronabinol_CTD_00006853	4/403	0,840964099	1	0	0	0,682168956	0,118155967
N-Acetyl-L-cysteine_CTD_00005305	3/316	0,841331852	1	0	0	0,652485972	0,112729417
methazolamide_HL60_UP	3/316	0,841331852	1	0	0	0,652485972	0,112729417
K-252a, Nocardiopsis sp._RBC	1/126	0,843170877	1	0	0	0,545464463	0,093048405
azacyclonol_MCF7_UP	1/126	0,843170877	1	0	0	0,545464463	0,093048405
Arsenenous acid_CTD_00000922	15/1284	0,843724266	1	0	0	0,802903298	0,136436986
PHENCYCLIDINE_CTD_00005881	2/227	0,845373612	1	0	0	0,605537642	0,101716158
clidinium bromide_HL60_UP	2/227	0,845373612	1	0	0	0,605537642	0,101716158
N-NITROSODIMETHYLAMINE_CTD_00005845	1/127	0,845467183	1	0	0	0,541169467	0,090843913
methyprylon_BOSS	1/127	0,845467183	1	0	0	0,541169467	0,090843913
MG-262_MCF7_DOWN	4/407	0,846427044	1	0	0	0,675464593	0,112621067
LY 294002_CTD_00003061	2/228	0,84712488	1	0	0	0,602881775	0,100022402
NICKEL SULFATE_CTD_00001417	6/577	0,848173238	1	0	0	0,714681341	0,117686844
FENRETINIDE_CTD_00007166	2/229	0,848858152	1	0	0	0,600249103	0,098358729
paclitaxel_PC3_UP	1/129	0,849959754	1	0	0	0,532779243	0,086611939
8-azaguanine_PC3_UP	3/323	0,851833201	1	0	0	0,638345409	0,102367971
colchicine_HL60_DOWN	1/130	0,852156986	1	0	0	0,528680941	0,084580763
rosiglitazone_CTD_00003139	4/412	0,853033276	1	0	0	0,667267207	0,106066607
scriptaid_PC3_UP	7/664	0,853336405	1	0	0	0,724547675	0,114914298
LITHIUM CHLORIDE_CTD_00007226	2/232	0,853951499	1	0	0	0,592487262	0,09354241
Retinoic acid_BOSS	2/232	0,853951499	1	0	0	0,592487262	0,09354241
Oxazolone_CTD_00006449	1/131	0,854322151	1	0	0	0,524645209	0,082603778
methazolamide_HL60_DOWN	1/131	0,854322151	1	0	0	0,524645209	0,082603778
bucladesine_HL60_UP	2/233	0,85561429	1	0	0	0,589944398	0,091993334
thimerosal_CTD_00006868	1/132	0,856455713	1	0	0	0,520670624	0,080679303
STOCK1N-28457_MCF7_DOWN	1/132	0,856455713	1	0	0	0,520670624	0,080679303
thiostrepton_HL60_UP	1/132	0,856455713	1	0	0	0,520670624	0,080679303
cephaeline_PC3_DOWN	2/234	0,857259832	1	0	0	0,587423268	0,090471535
vorinostat_PC3_UP	4/416	0,858144017	1	0	0	0,660851176	0,101099221
flunixin_HL60_UP	1/133	0,858558133	1	0	0	0,516755807	0,078805719
harmine_PC3_DOWN	1/133	0,858558133	1	0	0	0,516755807	0,078805719
tacrolimus_BOSS	1/133	0,858558133	1	0	0	0,516755807	0,078805719
1-NITROPYRENE_CTD_00001569	2/235	0,858888274	1	0	0	0,584923594	0,088976489
Torcetrapib_CTD_00004291	3/328	0,858959871	1	0	0	0,628614534	0,0955702
Dasatinib_CTD_00004330	5/503	0,859382043	1	0	0	0,683186107	0,103531185
irinotecan_MCF7_UP	7/671	0,860494363	1	0	0	0,716989056	0,107726325
Prestwick-983_HL60_DOWN	2/236	0,860499763	1	0	0	0,582445105	0,087507682
gemcitabine_CTD_00002382	2/236	0,860499763	1	0	0	0,582445105	0,087507682
Adenosine triphosphate_BOSS	1/134	0,860629864	1	0	0	0,51289942	0,076981463
OTSSP167_LINCS	2/237	0,862094444	1	0	0	0,57998753	0,086064611
WH-4-023_LINCS	1/135	0,862671351	1	0	0	0,509100165	0,075205031
AG-012559_PC3_DOWN	1/135	0,862671351	1	0	0	0,509100165	0,075205031
retinol_BOSS	1/135	0,862671351	1	0	0	0,509100165	0,075205031
alsterpaullone_PC3_DOWN	13/1153	0,862727147	1	0	0	0,774909619	0,114420679
dorzolamide_HL60_DOWN	2/238	0,863672465	1	0	0	0,577550608	0,084646784
midecamycin_HL60_DOWN	2/238	0,863672465	1	0	0	0,577550608	0,084646784
Crizotinib_LINCS	1/136	0,864683037	1	0	0	0,505356782	0,07347497
methylergometrine_HL60_UP	1/136	0,864683037	1	0	0	0,505356782	0,07347497
3,4-DICHLOROANILINE_CTD_00000754	1/136	0,864683037	1	0	0	0,505356782	0,07347497
cimetidine_PC3_UP	4/422	0,865526946	1	0	0	0,651455188	0,094081055
dexamethasone_CTD_00005779	4/422	0,865526946	1	0	0	0,651455188	0,094081055
proscillaridin_MCF7_DOWN	4/423	0,866725018	1	0	0	0,649915105	0,092959643
menadione_HL60_UP	3/334	0,867115362	1	0	0	0,617322057	0,088019787
medrysone_HL60_DOWN	8/760	0,867299116	1	0	0	0,72345813	0,102999718
cyclopenthiazide_PC3_UP	3/335	0,868433629	1	0	0	0,615479305	0,086822044
Crizotinib_FDA	1/138	0,868618726	1	0	0	0,498032771	0,070148414
1-chloro-2,4-dinitrobenzene_CTD_00005848	2/243	0,871317703	1	0	0	0,565666851	0,077919824
iohexol_HL60_DOWN	1/140	0,872440327	1	0	0	0,490918017	0,066991174
Uranium acetate_CTD_00000229	2/244	0,872798779	1	0	0	0,563348544	0,076643707
Bosutinib_FDA	1/141	0,874309377	1	0	0	0,487436329	0,065472929
colchicine_CTD_00005701	1/141	0,874309377	1	0	0	0,487436329	0,065472929
strophanthidin_HL60_DOWN	3/341	0,876104297	1	0	0	0,604649757	0,079977105
docetaxel_BOSS	1/142	0,876151134	1	0	0	0,484003678	0,063993357

scriptaid_MCF7_UP	7/688	0,876702055	1	0	0	0,699272756	0,092015957
URANIUM_CTD_00006964	2/247	0,877148606	1	0	0	0,556506254	0,072946202
mefloquine_MCF7_UP	1/143	0,877965994	1	0	0	0,480619037	0,062551326
ARSENIC_CTD_00005442	9/854	0,878150249	1	0	0	0,724305271	0,09411447
cefadroxil_HL60_DOWN	1/144	0,879754349	1	0	0	0,477281405	0,061145742
diuron_CTD_00005864	1/144	0,879754349	1	0	0	0,477281405	0,061145742
Decitabine_BOSS	1/144	0,879754349	1	0	0	0,477281405	0,061145742
Dasatinib	1/145	0,881516585	1	0	0	0,473989809	0,059775548
prenylamine_MCF7_UP	1/145	0,881516585	1	0	0	0,473989809	0,059775548
Cadmium sulfate_CTD_00001745	1/145	0,881516585	1	0	0	0,473989809	0,059775548
SU-14813_Kinome Scan	1/146	0,883253082	1	0	0	0,470743304	0,058439723
fulvestrant_PC3_DOWN	1/146	0,883253082	1	0	0	0,470743304	0,058439723
doxorubicin_PC3_DOWN	11/1023	0,883992097	1	0	0	0,739016369	0,091126007
lycorine_HL60_DOWN	9/862	0,884530639	1	0	0	0,71758318	0,088046111
Crizotinib	1/147	0,884964214	1	0	0	0,467540968	0,05713728
ellagic acid_CTD_00005891	1/147	0,884964214	1	0	0	0,467540968	0,05713728
daunorubicin_CTD_00005752	1/147	0,884964214	1	0	0	0,467540968	0,05713728
AST-487_Kinome Scan	1/148	0,886650351	1	0	0	0,464381908	0,055867264
EXEL-2880/GSK-1363089_Kinome Scan	1/149	0,888311857	1	0	0	0,461265251	0,054628754
mitoxantrone_CTD_00006345	2/256	0,889389737	1	0	0	0,536941581	0,062940152
tetrahydropalmatine_CTD_00000745	1/150	0,88994909	1	0	0	0,458190149	0,053420857
Cianidanol_CTD_00005600	9/871	0,891379483	1	0	0	0,710168428	0,081658742
parthenolide_HL60_UP	1/151	0,891562403	1	0	0	0,455155777	0,05224271
anisomycin_HL60_DOWN	11/1034	0,891752882	1	0	0	0,731154493	0,083765608
635-65-4_CTD_00005510	1/152	0,893152146	1	0	0	0,452161331	0,051093478
nitrate_BOSS	1/152	0,893152146	1	0	0	0,452161331	0,051093478
digoxin_MCF7_DOWN	4/449	0,894812767	1	0	0	0,612280823	0,068049369
cyclopenthiazide_PC3_DOWN	2/261	0,895693079	1	0	0	0,526655344	0,05801502
ascorbic acid_CTD_00005445	2/262	0,896913084	1	0	0	0,524645209	0,057079467
Bortezomib_CTD_00003736	9/879	0,897183226	1	0	0	0,703705007	0,076348596
meclofenoxate_HL60_DOWN	7/713	0,897687495	1	0	0	0,674754076	0,072828416
PD 98059_CTD_00003206	2/263	0,898119832	1	0	0	0,52265036	0,05615971
scopolamine_PC3_UP	4/454	0,899578488	1	0	0	0,605537642	0,064083426
Premarin_CTD_00005926	2/266	0,901661784	1	0	0	0,516755807	0,053492386
daunorubicin_MCF7_DOWN	14/1290	0,901911227	1	0	0	0,74589094	0,07700517
diltiazem_PC3_DOWN	3/364	0,90196445	1	0	0	0,566443865	0,058445775
Nintedanib_FDA	1/158	0,902214483	1	0	0	0,434990648	0,044761843
BIBF-1120 (derivative)_Kinome Scan	1/158	0,902214483	1	0	0	0,434990648	0,044761843
Vanadic sulfate_CTD_00001628	1/158	0,902214483	1	0	0	0,434990648	0,044761843
dmnq_CTD_00002569	1/158	0,902214483	1	0	0	0,434990648	0,044761843
PHOSPHINE_BOSS	1/158	0,902214483	1	0	0	0,434990648	0,044761843
raloxifene_HL60_DOWN	1/160	0,905061684	1	0	0	0,429553265	0,042848874
Sorafenib tosylate_BOSS	1/160	0,905061684	1	0	0	0,429553265	0,042848874
MG-132_MCF7_UP	2/270	0,906206321	1	0	0	0,509100165	0,050140395
parthenolide_MCF7_DOWN	1/162	0,907826258	1	0	0	0,424250138	0,041025949
CEP-701_Kinome Scan	2/272	0,908404459	1	0	0	0,505356782	0,048547382
liothyronine_CTD_00006943	1/163	0,909178309	1	0	0	0,421647376	0,040146752
Adehl_CTD_00006002	1/164	0,910510595	1	0	0	0,419076356	0,039288301
puromycin_HL60_UP	2/274	0,910554446	1	0	0	0,501668046	0,04700709
zalcitabine_PC3_UP	9/902	0,912451538	1	0	0	0,685761309	0,06282966
sanguinarine_MCF7_DOWN	3/376	0,913434566	1	0	0	0,54836587	0,049650985
GW-8510_PC3_DOWN	14/1312	0,913970826	1	0	0	0,733383622	0,065972717
niclosamide_PC3_UP	1/168	0,915647775	1	0	0	0,409098347	0,036051184
troglitazone_CTD_00002415	6/652	0,915950579	1	0	0	0,632471064	0,055526449
theophylline_CTD_00006862	4/473	0,91600054	1	0	0	0,58121372	0,050994718
methylergometrine_HL60_DOWN	1/169	0,916885468	1	0	0	0,406677647	0,035288523
tobramycin_PC3_DOWN	1/169	0,916885468	1	0	0	0,406677647	0,035288523
daunorubicin_PC3_DOWN	3/381	0,917844284	1	0	0	0,541169467	0,04639312
Coxistac_CTD_00000539	1/170	0,918105061	1	0	0	0,404285426	0,034543541
pyrvinium_MCF7_UP	1/170	0,918105061	1	0	0	0,404285426	0,034543541
carmustine_PC3_UP	1/171	0,919306819	1	0	0	0,401921183	0,03381578
dirithromycin_HL60_UP	1/171	0,919306819	1	0	0	0,401921183	0,03381578
arbutin_CTD_00005438	2/283	0,91965828	1	0	0	0,485713939	0,040680054
verteporfin_HL60_DOWN	2/283	0,91965828	1	0	0	0,485713939	0,040680054
14-Deoxy-11,12-didehydroandrographolide_CTD_00004394	1/173	0,921657863	1	0	0	0,397274696	0,032410149
astemizole_MCF7_UP	1/173	0,921657863	1	0	0	0,397274696	0,032410149
L-aspartic acid_BOSS	1/173	0,921657863	1	0	0	0,397274696	0,032410149
digoxigenin_HL60_DOWN	6/662	0,922636929	1	0	0	0,622917121	0,050156964
COBALT_CTD_00005696	1/174	0,922807657	1	0	0	0,394991508	0,031731427
meclofenoxate_HL60_UP	3/387	0,922865946	1	0	0	0,532779243	0,042766879
H-7_MCF7_DOWN	14/1332	0,923847156	1	0	0	0,722371856	0,05721809
menadione_PC3_DOWN	1/175	0,923940634	1	0	0	0,392734413	0,031068221
Phorbol 12-myristate 13-acetate_CTD_00006852	4/484	0,924376283	1	0	0	0,568004317	0,04466562
ciclopirox_HL60_UP	1/177	0,926157108	1	0	0	0,388296736	0,029786784
dimethyl sulfoxide_BOSS	1/177	0,926157108	1	0	0	0,388296736	0,029786784
dUTP_BOSS	1/179	0,928309205	1	0	0	0,383958225	0,028562808
O,P'-DDT_CTD_00000838	1/179	0,928309205	1	0	0	0,383958225	0,028562808
dihydroergotamine_HL60_DOWN	2/293	0,928751078	1	0	0	0,469136671	0,034676013
clopamide_HL60_UP	1/180	0,929361697	1	0	0	0,381825124	0,027971468
doxycycline_HL60_DOWN	1/180	0,929361697	1	0	0	0,381825124	0,027971468
4-aminobutyric acid_BOSS	1/180	0,929361697	1	0	0	0,381825124	0,027971468
L-threonine_BOSS	1/181	0,930398788	1	0	0	0,379715593	0,027393435
folic acid_BOSS	1/181	0,930398788	1	0	0	0,379715593	0,027393435
hydrogen sulfide_BOSS	1/181	0,930398788	1	0	0	0,379715593	0,027393435
digoxigenin_PC3_UP	2/296	0,931284102	1	0	0	0,464381908	0,033059762

danazol_HL60_UP	3/398	0,931351254	1	0	0	0,518054188	0,036843385
calmidazolium_MCF7_UP	1/182	0,931420705	1	0	0	0,377629244	0,026828375
1,9-Pyrazoloanthrone_CTD_00003948	1/182	0,931420705	1	0	0	0,377629244	0,026828375
methotrexate_BOSS	1/182	0,931420705	1	0	0	0,377629244	0,026828375
L-leucine_BOSS	1/182	0,931420705	1	0	0	0,377629244	0,026828375
VITAMIN E_CTD_00006994	16/1511	0,931915833	1	0	0	0,727767278	0,051316892
Velcade(Bortezomib)_BOSS	1/183	0,932427667	1	0	0	0,375565696	0,026275965
cephaeline_HL60_UP	7/767	0,93290793	1	0	0	0,627248574	0,043561639
piperlongumine_HL60_UP	1/184	0,933419894	1	0	0	0,373524578	0,025735893
terfenadine_MCF7_UP	1/184	0,933419894	1	0	0	0,373524578	0,025735893
ellagic acid_BOSS	1/184	0,933419894	1	0	0	0,373524578	0,025735893
COPPER_BOSS	1/184	0,933419894	1	0	0	0,373524578	0,025735893
Sunitinib_Kinome Scan	1/185	0,934397599	1	0	0	0,371505526	0,025207852
ZEARALENONE_CTD_00007008	1/185	0,934397599	1	0	0	0,371505526	0,025207852
IRON_BOSS	1/185	0,934397599	1	0	0	0,371505526	0,025207852
diazinon_CTD_00005790	10/1028	0,935204321	1	0	0	0,668565392	0,044787362
3-(1-methylpyrrolidin-2-yl)pyridine_CTD_00006393	4/500	0,935225726	1	0	0	0,549828179	0,036820542
zuclopenthixol_PC3_UP	2/301	0,935317979	1	0	0	0,456667923	0,030536801
lactic acid_BOSS	1/186	0,935360996	1	0	0	0,369508185	0,024691547
Sunitinib_FDA	1/187	0,936310291	1	0	0	0,367532205	0,024186688
acetic acid_BOSS	1/187	0,936310291	1	0	0	0,367532205	0,024186688
ZINC_BOSS	1/187	0,936310291	1	0	0	0,367532205	0,024186688
Imatinib mesylate_BOSS	1/187	0,936310291	1	0	0	0,367532205	0,024186688
Erythorbic acid_BOSS	1/187	0,936310291	1	0	0	0,367532205	0,024186688
17-Hydroxyandrostan-3-one_CTD_00006776	1/188	0,937245693	1	0	0	0,365577246	0,023692995
parathion_CTD_00006472	7/777	0,938118104	1	0	0	0,619175877	0,0395526
Prestwick-983_HL60_UP	1/189	0,938167402	1	0	0	0,363642975	0,023210196
metformin_BOSS	1/189	0,938167402	1	0	0	0,363642975	0,023210196
17-Ethynyl estradiol_CTD_00005932	2/305	0,9383829	1	0	0	0,450678835	0,028661914
NICKEL_CTD_00006389	1/190	0,939075618	1	0	0	0,361729065	0,022738026
L-glutamic acid_BOSS	1/190	0,939075618	1	0	0	0,361729065	0,022738026
L-glutamine_BOSS	1/190	0,939075618	1	0	0	0,361729065	0,022738026
HEXACHLOROETHANE_BOSS	1/190	0,939075618	1	0	0	0,361729065	0,022738026
cholesterol_BOSS	1/190	0,939075618	1	0	0	0,361729065	0,022738026
streptozocin_BOSS	1/191	0,93997054	1	0	0	0,359835195	0,022276226
N-Acetyl-L-cysteine_BOSS	1/191	0,93997054	1	0	0	0,359835195	0,022276226
Insulin_BOSS	1/191	0,93997054	1	0	0	0,359835195	0,022276226
GW-8510_MCF7_DOWN	13/1290	0,940713937	1	0	0	0,692613016	0,042329865
dihydroergocristine_HL60_DOWN	1/192	0,940852359	1	0	0	0,357961054	0,021824545
Isoguanine_BOSS	1/192	0,940852359	1	0	0	0,357961054	0,021824545
iopromide_HL60_UP	1/193	0,941721268	1	0	0	0,356106333	0,02138274
DICHLOROMETHANE_BOSS	1/193	0,941721268	1	0	0	0,356106333	0,02138274
Simvastatin and niacin_BOSS	1/193	0,941721268	1	0	0	0,356106333	0,02138274
17-Hydroxy-17-methylandrost-4-en-3-one_BOSS	1/194	0,942577456	1	0	0	0,354270734	0,020950573
PLATINUM_BOSS	1/194	0,942577456	1	0	0	0,354270734	0,020950573
trichostatin A_MCF7_UP	8/874	0,942752588	1	0	0	0,629094026	0,037085972
MG-262_MCF7_UP	2/312	0,943418424	1	0	0	0,440567451	0,025661018
pergolide_HL60_UP	2/312	0,943418424	1	0	0	0,440567451	0,025661018
valproic acid_HL60_DOWN	1/195	0,943421106	1	0	0	0,352453961	0,020527812
cephaeline_PC3_UP	4/514	0,943543737	1	0	0	0,534852314	0,031081637
Demecolcine_CTD_00005762	8/877	0,944084265	1	0	0	0,626942051	0,036074153
thioridazine_PC3_UP	1/196	0,944252404	1	0	0	0,350655726	0,020114234
2-Butanone_BOSS	1/196	0,944252404	1	0	0	0,350655726	0,020114234
Staurosporine, Streptomyces sp._RBC	1/197	0,945071528	1	0	0	0,348875748	0,019709618
tamoxifen_BOSS	1/197	0,945071528	1	0	0	0,348875748	0,019709618
11-cis-Retinal_BOSS	1/197	0,945071528	1	0	0	0,348875748	0,019709618
5-Fluorouracil_BOSS	1/197	0,945071528	1	0	0	0,348875748	0,019709618
Staurosporine_Kinome Scan	2/315	0,945454827	1	0	0	0,43637157	0,024475719
Acid red 87_BOSS	1/198	0,945878656	1	0	0	0,347113749	0,019313752
Bisulfite_BOSS	1/198	0,945878656	1	0	0	0,347113749	0,019313752
Andriol_CTD_00000567	1/199	0,946673964	1	0	0	0,345369459	0,018926429
MG-262_PC3_UP	2/320	0,948695013	1	0	0	0,429553265	0,022623672
Zoledronic acid_CTD_00003127	3/426	0,949229453	1	0	0	0,484003678	0,025218879
proscillaridin_HL60_DOWN	5/620	0,949518145	1	0	0	0,554262277	0,02871114
R406_Kinome Scan	1/203	0,949740406	1	0	0	0,338564149	0,017458598
Vitamin D3_BOSS	1/203	0,949740406	1	0	0	0,338564149	0,017458598
Fonofos_CTD_00005884	6/713	0,949987583	1	0	0	0,578360637	0,029673582
0179445-0000_MCF7_UP	1/204	0,95047915	1	0	0	0,336904521	0,017111062
helveticoside_PC3_UP	2/324	0,95115453	1	0	0	0,424250138	0,021245911
TERBUFOS_CTD_00000658	6/716	0,951287358	1	0	0	0,575937338	0,028761791
carmustine_CTD_00005595	3/430	0,951398325	1	0	0	0,479501319	0,023889933
cycloheximide_CTD_00005731	2/325	0,951751641	1	0	0	0,422944753	0,020915108
BX-912_LINCS	1/206	0,951924329	1	0	0	0,333633604	0,016438039
norgestrel_CTD_00006422	1/206	0,951924329	1	0	0	0,333633604	0,016438039
dexverapamil_MCF7_DOWN	2/326	0,952341797	1	0	0	0,421647376	0,02058958
Capsaicin_CTD_00005570	1/207	0,952631078	1	0	0	0,332021847	0,016112212
sulfamonomethoxine_HL60_UP	2/328	0,953501542	1	0	0	0,419076356	0,019954001
helveticoside_HL60_DOWN	4/534	0,953746942	1	0	0	0,514820392	0,024380299
deptropine_HL60_DOWN	3/435	0,953988269	1	0	0	0,473989809	0,022326771
clindamycin_HL60_UP	2/332	0,95574083	1	0	0	0,414027243	0,018742393
nocodazole_HL60_DOWN	1/212	0,956012487	1	0	0	0,324191143	0,014583513
fisetin_PC3_DOWN	4/541	0,956898467	1	0	0	0,50815913	0,022388469
trichostatin A_HL60_DOWN	4/543	0,957761938	1	0	0	0,506287457	0,021849357
piperlongumine_MCF7_UP	1/216	0,958543529	1	0	0	0,318187603	0,01347216
medrysone_HL60_UP	2/341	0,960408925	1	0	0	0,403099838	0,01628367

camptothecin_MCF7_UP	1/222	0,962070505	1	0	0	0,309587938	0,011971004
clopamide_HL60_DOWN	2/345	0,962329014	1	0	0	0,398426216	0,015299119
8-Bromo-cAMP, Na_CTD_00007044	5/653	0,963016062	1	0	0	0,526252085	0,019831909
H-7_PC3_DOWN	13/1361	0,963403909	1	0	0	0,65648111	0,024475275
pregnenolone_PC3_UP	3/456	0,963522864	1	0	0	0,452161331	0,016801891
resveratrol_BOSS	1/225	0,963720183	1	0	0	0,305460099	0,011288062
latamoxef_HL60_UP	11/1196	0,964639413	1	0	0	0,632118516	0,022756844
strophanthidin_PC3_UP	3/459	0,964723032	1	0	0	0,449206028	0,01613289
vinblastine_CTD_00006986	1/228	0,965298342	1	0	0	0,301440887	0,010646308
lanatoside C_HL60_DOWN	4/563	0,965561127	1	0	0	0,488302113	0,017112971
celastrol_MCF7_UP	1/229	0,96580904	1	0	0	0,300124552	0,010441077
SARIN_CTD_00006722	1/230	0,966312247	1	0	0	0,298819662	0,01024003
atovaquone_MCF7_DOWN	2/354	0,966328538	1	0	0	0,388296736	0,013299707
cicloheximide_MCF7_UP	2/354	0,966328538	1	0	0	0,388296736	0,013299707
vorinostat_PC3_DOWN	2/355	0,96674692	1	0	0	0,387202943	0,013094636
daunorubicin_MCF7_UP	1/232	0,967296625	1	0	0	0,296243631	0,009850125
ethanol_CTD_00005337	1/232	0,967296625	1	0	0	0,296243631	0,009850125
vorinostat_HL60_DOWN	4/569	0,967626706	1	0	0	0,483153057	0,015900036
Aflodac_CTD_00006817	1/235	0,968719696	1	0	0	0,292461797	0,00929443
tretinoin_HL60_UP	1/241	0,971383328	1	0	0	0,285180591	0,008279965
digoxigenin_HL60_UP	1/244	0,972629113	1	0	0	0,281674272	0,007817151
quinpirole_HL60_UP	1/245	0,973032243	1	0	0	0,280524581	0,007668998
digitoxigenin_PC3_UP	3/483	0,973071772	1	0	0	0,426885232	0,011652872
digoxin_PC3_UP	3/483	0,973071772	1	0	0	0,426885232	0,011652872
4-Hydroxytamoxifen_CTD_00000850	4/587	0,973153155	1	0	0	0,468337461	0,012745244
Cylindrospermopsin_CTD_00003136	3/484	0,973375483	1	0	0	0,426003238	0,011495854
ouabain_MCF7_UP	1/246	0,973429456	1	0	0	0,279384237	0,007523796
bisacodyl_MCF7_DOWN	2/373	0,973476527	1	0	0	0,368517546	0,009906329
cicloheximide_HL60_UP	3/485	0,973675954	1	0	0	0,42512488	0,01134094
harmine_MCF7_DOWN	1/247	0,973820837	1	0	0	0,278253127	0,007381482
progesterone_CTD_00006624	19/1916	0,975149739	1	0	0	0,68154589	0,017150585
topotecan_CTD_00007293	1/251	0,975329736	1	0	0	0,273818814	0,006839905
HEMATOXYLIN_BOSS	1/251	0,975329736	1	0	0	0,273818814	0,006839905
PNU-0293363_MCF7_UP	1/255	0,976751939	1	0	0	0,269523617	0,006339886
digoxigenin_MCF7_UP	1/255	0,976751939	1	0	0	0,269523617	0,006339886
emetine_PC3_UP	6/801	0,977544857	1	0	0	0,514820392	0,011692137
alprostadil_HL60_DOWN	3/500	0,977815174	1	0	0	0,412371134	0,009251386
anisomycin_PC3_UP	7/899	0,97828356	1	0	0	0,535149785	0,011749595
CUPRIC OXIDE_CTD_00001471	2/394	0,979675583	1	0	0	0,348875748	0,007163745
lycorine_PC3_UP	3/508	0,979762275	1	0	0	0,4058771	0,008298285
hydrogen peroxide_CTD_00006118	28/2674	0,979818206	1	0	0	0,719670391	0,014672805
sulpiride_PC3_DOWN	4/617	0,980449817	1	0	0	0,445565785	0,008797169
digoxin_HL60_UP	1/267	0,980546619	1	0	0	0,257410196	0,005056846
Tetradioxin_CTD_00006848	42/3769	0,980662178	1	0	0	0,765878997	0,014955506
2-Nonenal, 4-hydroxy-, (2E,4R)-_CTD_00001295	1/275	0,982726698	1	0	0	0,249921899	0,004354696
thapsigargin_MCF7_UP	2/407	0,9827844	1	0	0	0,337732296	0,005864894
alprostadil_HL60_UP	2/409	0,983219831	1	0	0	0,336080794	0,005687344
lanatoside C_PC3_UP	2/411	0,983644584	1	0	0	0,334445364	0,005515219
Retinoic acid_CTD_00006918	48/4259	0,983756575	1	0	0	0,774587714	0,012685265
PHA-00665752_MCF7_DOWN	1/280	0,983963751	1	0	0	0,245459008	0,003968145
ouabain_PC3_UP	2/416	0,984661384	1	0	0	0,330425588	0,005107543
digoxigenin_MCF7_DOWN	1/283	0,984663174	1	0	0	0,242856969	0,003753513
metronidazole_PC3_DOWN	5/745	0,985115511	1	0	0	0,461265251	0,006917307
emetine_HL60_UP	6/849	0,98581776	1	0	0	0,485713939	0,006937826
neostigmine bromide_PC3_DOWN	4/650	0,986304076	1	0	0	0,422944753	0,005832653
chlorhexidine_PC3_UP	1/293	0,98678225	1	0	0	0,234568336	0,003121139
ouabain_HL60_DOWN	3/549	0,987441416	1	0	0	0,375565696	0,004746441
piroxicam_CTD_00006571	3/550	0,987588294	1	0	0	0,374882849	0,004682052
OCHRATOXIN A_CTD_00001202	1/298	0,987729651	1	0	0	0,230632625	0,002847448
primaquine_PC3_UP	2/434	0,987838371	1	0	0	0,316721301	0,003875461
carbamazepine_CTD_00005574	2/437	0,988301577	1	0	0	0,314547013	0,003701397
testosterone_CTD_00006844	10/1248	0,988470164	1	0	0	0,550709314	0,006386477
simvastatin_CTD_00007319	1/305	0,988943362	1	0	0	0,225339417	0,002505372
scriptaid_MCF7_DOWN	4/670	0,988998106	1	0	0	0,410319536	0,004539309
Vitinoin_CTD_00007069	5/781	0,989733618	1	0	0	0,440003344	0,00454059
Bisphenol A_CTD_00000312	10/1263	0,989857216	1	0	0	0,544168823	0,005547568
digoxin_HL60_DOWN	2/452	0,990371989	1	0	0	0,304108506	0,002942146
LEAD_CTD_00006203	5/792	0,990849413	1	0	0	0,433892186	0,003988645
Silica_CTD_00006678	6/899	0,991345684	1	0	0	0,458699815	0,003987011
scriptaid_PC3_DOWN	4/692	0,991377408	1	0	0	0,397274696	0,003440392
digoxin_MCF7_UP	1/324	0,991667596	1	0	0	0,212125069	0,001774917
thiostrepton_HL60_DOWN	1/325	0,99179079	1	0	0	0,211472376	0,001743186
trichostatin A_MCF7_DOWN	3/588	0,992095398	1	0	0	0,350655726	0,002782807
digitoxigenin_MCF7_UP	1/328	0,992149583	1	0	0	0,209538178	0,001651453
indomethacin_CTD_00006147	1/336	0,993031755	1	0	0	0,204549174	0,001430338
anisomycin_MCF7_UP	4/718	0,993557774	1	0	0	0,382888704	0,002474635
strophanthidin_HL60_UP	1/344	0,993815061	1	0	0	0,199792216	0,00123954
trichostatin A_CTD_00000660	37/3585	0,993824936	1	0	0	0,70933203	0,004393751
etifenin_PC3_DOWN	3/609	0,993860058	1	0	0	0,338564149	0,002085172
digitoxigenin_HL60_DOWN	4/723	0,993911549	1	0	0	0,380240787	0,002322154
mebendazole_HL60_UP	1/346	0,993996762	1	0	0	0,198637348	0,001196061
genistein_CTD_00007324	9/1232	0,994164346	1	0	0	0,502075244	0,00293852
resveratrol_CTD_00002483	13/1602	0,994228419	1	0	0	0,557722091	0,003228263
vorinostat_MCF7_DOWN	2/492	0,994299539	1	0	0	0,279384237	0,001597175
calcitriol_CTD_00005558	17/1959	0,994424085	1	0	0	0,59641903	0,003334888

CADMIUM_CTD_00005555	8/1143	0,994532742	1	0	0	0,481039526	0,002637183
ajmaline_HL60_DOWN	2/496	0,994592536	1	0	0	0,277131138	0,001502643
helveticoside_MCF7_UP	1/355	0,994750752	1	0	0	0,193601471	0,001018939
cimetidine_PC3_DOWN	6/953	0,995004179	1	0	0	0,43270843	0,002167152
67526-95-8_CTD_00007263	6/954	0,995055483	1	0	0	0,432254857	0,002142593
captopril_PC3_DOWN	5/856	0,995377531	1	0	0	0,401451649	0,00186
POTASSIUM CHROMATE_CTD_00001284	16/1898	0,995455763	1	0	0	0,579376374	0,002638824
ZINC_CTD_00007011	13/1643	0,995904579	1	0	0	0,543804498	0,002231681
nocodazole_HL60_UP	1/373	0,995987148	1	0	0	0,184258773	0,000740891
alsterpaullone_MCF7_DOWN	11/1469	0,996244772	1	0	0	0,514645164	0,001936248
proscillaridin_MCF7_UP	1/378	0,996275767	1	0	0	0,181821488	0,00067841
0175029-0000_MCF7_DOWN	11/1488	0,996832411	1	0	0	0,508073754	0,001611923
doxycycline_HL60_UP	1/390	0,996886766	1	0	0	0,17622698	0,000549492
irinotecan_PC3_DOWN	6/999	0,996908921	1	0	0	0,412783918	0,001277924
COPPER_CTD_00005706	13/1689	0,997237956	1	0	0	0,528993955	0,001463126
lanatoside C_MCF7_UP	1/405	0,997511817	1	0	0	0,169700055	0,000422771
dirithromycin_HL60_DOWN	2/556	0,997566338	1	0	0	0,2472249	0,000602395
phenobarbital_CTD_00006510	7/1130	0,997682122	1	0	0	0,425751908	0,000987986
sulfaguanidine_PC3_UP	4/809	0,997739742	1	0	0	0,339819641	0,000768949
menadione_CTD_00007386	7/1141	0,997930591	1	0	0	0,421647376	0,000873465
digitoxigenin_HL60_UP	1/421	0,998041106	1	0	0	0,163250647	0,000320104
8-azaguanine_HL60_UP	1/428	0,998235782	1	0	0	0,16058066	0,000283549
diclofenac_CTD_00005804	1/428	0,998235782	1	0	0	0,16058066	0,000283549
lanatoside C_HL60_UP	1/432	0,998338226	1	0	0	0,159093802	0,000264598
(-)-Epigallocatechin gallate_CTD_00002033	17/2115	0,998396362	1	0	0	0,552427839	0,000886605
latamoxef_HL60_DOWN	11/1578	0,998615463	1	0	0	0,479096163	0,000663786
Enterolactone_CTD_00001393	5/972	0,998726854	1	0	0	0,353541782	0,000450397
benzo[a]pyrene_CTD_00005488	44/4425	0,999000978	1	0	0	0,683402256	0,000683075
8-azaguanine_HL60_DOWN	4/897	0,999206565	1	0	0	0,306481705	0,00024327
thapsigargin_PC3_UP	1/484	0,999236713	1	0	0	0,142001079	0,000108429
0179445-0000_MCF7_DOWN	4/911	0,999329986	1	0	0	0,301771778	0,000202259
anisomycin_HL60_UP	6/1142	0,999345933	1	0	0	0,361095564	0,000236258
chlorzoxazone_HL60_DOWN	7/1286	0,999556465	1	0	0	0,374105487	0,000165966
camptothecin_MCF7_DOWN	9/1513	0,999628892	1	0	0	0,408827958	0,000151747
chlorhexidine_PC3_DOWN	1/534	0,999638566	1	0	0	0,128705098	4,65268E-05
azacyclonol_HL60_DOWN	2/703	0,999669633	1	0	0	0,195529224	6,46071E-05
glibenclamide_HL60_DOWN	7/1333	0,999734646	1	0	0	0,360914971	9,57829E-05
lycorine_HL60_UP	3/862	0,999744645	1	0	0	0,239194393	6,10873E-05
7646-79-9_CTD_00000928	25/3095	0,999832072	1	0	0	0,555157693	9,32341E-05
formaldehyde_CTD_00006001	26/3193	0,999846103	1	0	0	0,559643464	8,61343E-05
Pirinixic acid_CTD_00000280	7/1403	0,999877247	1	0	0	0,342907809	4,20957E-05
chlortetracycline_HL60_DOWN	4/1050	0,999877542	1	0	0	0,261822942	3,20642E-05
ETHYL METHANESULFONATE_CTD_00005938	16/2316	0,999878648	1	0	0	0,474808444	5,76225E-05
clindamycin_HL60_DOWN	9/1626	0,999885558	1	0	0	0,380416175	4,35381E-05
ampyrone_HL60_DOWN	2/781	0,999886269	1	0	0	0,176001338	2,00179E-05
arsenite_CTD_00000779	6/1301	0,999891623	1	0	0	0,316964746	3,43534E-05
quercetin_CTD_00006679	25/3159	0,999900647	1	0	0	0,543910433	5,40418E-05
emetine_MCF7_UP	1/637	0,999921128	1	0	0	0,10789407	8,51012E-06
cephaeline_MCF7_UP	2/811	0,999924168	1	0	0	0,169490807	1,28533E-05
2-Methylcholine_CTD_00002006	9/1667	0,999925277	1	0	0	0,371059809	2,77277E-05
danazol_HL60_DOWN	5/1233	0,999939237	1	0	0	0,27870447	1,69353E-05
atrazine_CTD_00005450	22/2969	0,999947921	1	0	0	0,509271637	2,6523E-05
camptothecin_PC3_DOWN	7/1494	0,999954341	1	0	0	0,322021189	1,47036E-05
0175029-0000_PC3_DOWN	25/3326	0,999973445	1	0	0	0,516600438	1,37183E-05
lobeline_HL60_DOWN	6/1510	0,999987451	1	0	0	0,273093466	3,42718E-06
trichostatin A_PC3_DOWN	3/1133	0,999989495	1	0	0	0,181981966	1,91176E-06
cyclosporin A_CTD_00007121	40/4826	0,99999079	1	0	0	0,569652071	5,24666E-06
COUMESTROL_CTD_00005717	8/1813	0,99999184	1	0	0	0,303269817	2,47458E-06
AFLATOXIN B1_CTD_00007128	20/3082	0,999992218	1	0	0	0,445999496	3,47062E-06
acetaminophen_CTD_00005295	30/4136	0,999993749	1	0	0	0,498514427	3,11603E-06
Copper sulfate_CTD_00007279	51/6017	0,999993756	1	0	0	0,582541904	3,63716E-06
VALPROIC ACID_CTD_00006977	75/8313	0,999993779	1	0	0	0,620069671	3,85745E-06
METHYL METHANESULFONATE_CTD_00006307	27/3865	0,999993828	1	0	0	0,480121631	2,9635E-06
irinotecan_MCF7_DOWN	2/1272	0,999995843	1	0	0	0,108063714	4,49192E-07

Genes
HRK;RPS17;RPL41;RPL31;RPS6;RPLP0;RPS3;RPL13A;RPL7
LAMP5;RTP4;IFIH1;IFITM1;CXCL11;SAMD9;IFNB1;TNFSF10;IFI6;LY6E
EEF1A1;RPS19;RPS7;RPS3A;RPL27
RPL41;RPS6;TPT1;RPL7;RPS12
IFIH1;SAMD9;IFI6;TNFSF10
IFIH1;IFITM1;IFI6
CEMIP;B3GNT2;SLC16A3;TM4SF1
IFIH1;IFITM1;CEMIP;IFI6
IFITM1;BEX1;IFI6
RPL30;RPS7;RPL37A
WT1;HK2;HSPA1B
HILPDA;RPL37A;HK2
KBTBD4;RPL31;MFSD5;HILPDA;B3GNT2
CEMIP;HBG1;TNFSF10;SEPP1;MDFI;HSPA1B;HK2
RPL41;RPL31;RPL34;COMMD6;RPL10A;TOMM20;RPL7;KIAA1217;RPS27;MFSD5;RPL37A;ARL6IP5;RPL36;RPL27;RPS27A;RPL39;UBA52;RPS12
IFITM1;IFI6
C3ORF52;IL12A;HSPA1B
RPS3;RPL13A
IFITM1;IFI6
CEMIP;TM4SF1
IFIH1;IFITM1;IFI6
CEMIP;IFI6
IFITM1;IFI6
IFIH1;IFNB1;RNASE3
B3GNT2;HSPA1B
IL12A;RNASE3
HILPDA;SLC16A3
CXADR;DOK4;PTGDR2;C6ORF106;MARC2;AMDHD2;APBB2;AEBP1;CLC;HSPA1B
MFSD5;B3GNT2
CEMIP;TM4SF1
EEF1A1;RPSA;TPT1
CEMIP;TM4SF1
TMEM63A;B3GNT2;CD244
CEMIP;RPL31;NAV2
HILPDA;HK2
CEMIP;RPS6KA2;NAV2;TM4SF1
LAMP5;HILPDA;CTSG;HK2;HSPA1B
TNFSF10;IL12A
CLP1;RPL31;MFSD5;HILPDA;RPL37A;B3GNT2;DOLK;HSPA1B
EFNA1;TMEM63A;CXADR;MFSD5;HILPDA;ARL6IP5;B3GNT2;ROR1;HK2
HILPDA;HK2
HILPDA;B3GNT2
CEMIP;SLC16A3
HILPDA;SLC16A3
HILPDA;HK2
TNFSF10;SEPP1
IFIH1;TNFSF10
TMEM63A;CXADR;MFSD5;ARL6IP5;B3GNT2;DOLK;SLC16A3;HSPA1B
CEMIP;NAV2
EEF1A1;CD274;RPS19;HBG2;PTGDR2;RPL34;ARL6IP5;TNFSF10;NKG7;IL12A;CLC;RPS27A
RPS6KA2;HILPDA;HK2
CEMIP;TM4SF1
LAMP5;TNFSF10;DEFA3;IL12A;CLC
IFIH1;EFNA1;CXADR;FEM1B;HILPDA;TNFSF10;NAV2;HSPA1B;HK2
IFIH1;KBTBD4;CLP1
HILPDA;HK2
DOK4;PTGDR2;MARC2;GAD1;AMDHD2;APBB2;AEBP1
WT1;HILPDA;RPL37A;CTSG;SLC16A3;HK2
RPL31;RPS20
CEMIP;TM4SF1
C3ORF52;HSPA1B
KBTBD4;MFSD5;B3GNT2;HSPA1B
RPL31;CLP1;RPS20
IFIH1;CXCL11
TMEM63A;RPS6KA2;HILPDA;RPL37A;CTSG;RPS21;TOMM20;SLC16A3;HK2
TNFSF10
IL12A
HBG2
CXADR
HILPDA;HK2
B3GNT2;APBB2
ARL6IP5;ITM2A;TM4SF1
RPL31;RPL37A;APBB2;HSPA1B
CEMIP;RPL37A
HILPDA;HK2
BEX1;RPL31;B3GNT2;HOXA1
IL12A
TNFSF10
RPS25;POU2AF1;IFI6
RPL3;RPS8;RPL11;RPS6;RPS3;RPS20;RPSA;SEPP1
BEX1;CXADR;TMEM104;RPL31;WT1;GAD1;C3ORF52;HOXA1
HILPDA;SLC16A3;HK2
TNFSF10

PTGDR2
TRPC6
CEMIP;TMEM63A;BEX1;CXADR;GAD1;C3ORF52;HOXA1;IL12A;SEPP1
MFSD5;HILPDA;RPL37A;C3ORF52;B3GNT2;DOLK
EFNA1;MFSD5;HILPDA;ARL6IP5;B3GNT2
TNFSF10;IL12A
TNFSF10
TNFSF10
HILPDA;TNFSF10;HSPA1B
RPS6;TNFSF10
IL12A
HSPA1B
B3GNT2;DOLK;HSPA1B
MFSD5;RPL37A;HSPA1B
TNFSF10;CD244
CXADR;TMEM63A;ROR1
NAV2;HSPA1B
IFIH1;IFI6
WT1
HBG2
IFNB1
IL12A
IL12A
RPS6
RPS6KA2;HOXA1;GNG12;CST7;TM4SF1;HSPA1B
IL12A
IL12A
GAD1
EFNA1;MFSD5;FEM1B;HILPDA;RPL37A;B3GNT2;APBB2;DOLK;HSPA1B
HILPDA;HK2
CEMIP;TM4SF1
TNFSF10;TM4SF1
IFITM1;SEPP1;TM4SF1
EFNA1;CD274;WT1;HILPDA;RPS6;IL12A;NPTX1;SLC16A3;TM4SF1;HK2
IFNB1;RAPGEF5
TNFSF10
EFNA1;RPS6KA2;HILPDA;IFI6;ROR1;HSPA1B
RPL13A;HSPA1B
CEMIP;CXADR;RPL37A;HOXA1
NAV2
ROR1
HOXA1
NAV2
HSPA1B
RPS6
R3HCC1;SLC16A3
WT1;HILPDA;TNFSF10;HSPA1B;CD244;HK2
HRK;EEF1A1;CLDN9;RPS6;TNFSF10;HOXA1;RPSA
TNFSF10;TM4SF1
CD274;HSPA1B
EPHB4
EPHB4
RNASE3
SLC16A3
CEMIP
CEMIP
HSPA1B
RAPGEF5
IFIH1;HILPDA
IFIH1;WT1;HILPDA;SLC16A3
HILPDA;HK2
BEX1;RPL31;RPL37A;C3ORF52;HOXA1
LAMP5;ARL2BP;MFSD5;RPL37A;SLC16A3;HSPA1B
RPS6KA2
RPL37A
WT1
IFI6
SLC16A3
HSPA1B
GAD1
RPS6
IL12A
CXADR;RPL37A;RPL35A
LAMP5;RPS6KA2
RPL31;RPL37A;B3GNT2;TOMM20;HSPA1B
RPL32;COMMD6;SEPP1;HSPA1B
WT1;RPS6;IL12A
PTGDR2
EPHB4
RPS6KA2
RPS6KA2
IL12A
HSPA1B
CEMIP
TNFSF10

RPS6
CLP1
RPS6
CXCL11
IFNB1
TRPC6
TNFSF10
TNFSF10
FCRLA;IFITM1;MFSD5;AMDHD2;CYP4F3;IFI6;IL12A;DOLK;RPS21;SLC16A3;HSPA1B
WT1;HK2
EEF1B2;EEF1A1;HSPA1B
SLC16A3;HSPA1B
IFIH1;RPS6KA2
HSPA1B
RPL37A
HSPA1B
BEX1
IFNB1;DNTT
EFNA1;CXCL11;WT1;C6ORF106;IFI6;PAX5
RPL31;RPS6KA2;HILPDA;RPL37A;B3GNT2;CTSG;TOMM20;HK2
IFNB1;RPL34;TNFSF10;NAV2
EPHB4
EPHB4
HSPA1B
HSPA1B
HSPA1B
CEMIP
TM4SF1
RPS6
C3ORF52;HSPA1B
HILPDA;HK2
LAMP5;WT1;HSPA1B
HRK;IFITM1;RPS5;TM4SF1
HSPA1B
HSPA1B
RPS6
RNASE3
TNFSF10
KBTBD4;RPL31;CLP1;FEM1B
MIR23A;ARL6IP5
C3ORF52;HSPA1B
CEMIP;TM4SF1
EPHB4
RPS6KA2
HILPDA
HSPA1B
RPS6
CXADR;BEX1;RPL31;WT1;HOXA1
RPL32;RPL35A;RPL27
RPL36;SLC16A3
EPHB4
HSPA1B
TNFSF10
IL12A
TNFSF10
TNFSF10
MARC2;GAD1;MDFI;TPT1
KBTBD4;CLP1;MFSD5;FEM1B;CLDN9;HILPDA;RPL37A;B3GNT2;DOLK;NRBF2;HSPA1B
CTSG
RPS6KA2
IL12A
EPHB4
IFIH1
CEMIP
IFIH1
C6ORF106
UBA52
HBG2
IFNB1
RPL30;RPL32;RPS7;HBG1;RPL12;RPS5;RPLP0;DEFA3;RPL35A;RPSA;RPS3A;RPL10A;SEPP1;RPL9;RPL7;ARL2BP;RPL37A;TNFSF10;RPS3;B3GNT2;GOLGA7;UBA52
WT1;HILPDA;HK2
DOLK;HK2
TNFSF10;TM4SF1
IFNB1;TNFSF10
LAMP5;WT1;CTSG
IFITM1;CXADR;IFI6;HOXA1;NAV2;HSPA1B
RPS6KA2
RPS6KA2
IFI6
SAMD9
RPL31
HSPA1B
HSPA1B
EFNA1;C3ORF52;HSPA1B
IFNB1;RPS6;IL12A

SLC16A3
IFIH1
RPS6
TNFSF10
CXADR;ARL2BP;CLP1;RPS6KA2;R3HCC1;GDE1;B3GNT2;HOXA1;APBB2;GOLGA7;POU4F1;HSPA1B
ZNF792;HSPA1B
HRK;HILPDA;HSPA1B
CEMIP
EEF1A1
IL12A
SLC16A3
IL12A
TNFSF10
C1QA;RPL30;HBG2;HBG1;KCNJ12;TNFSF10;IFI6;HSPA1B;EPHB4
HILPDA;HSPA1B
WT1;HK2
TNFSF10
HSPA1B
RNASE3
RPS6KA2;MYO3A;EPHB4
RPL31;C3ORF52;HOXA1
HRK;IFITM1;CXADR;IFNB1;GAD1;IFI6;NAV2;TM4SF1;EEF1A1;WT1;RPS6KA2;TNFSF10;ITM2B
CD274;IFITM1;RPL3;IFNB1;IFI6;CST7;RPL10A;SEPP1;HK2;DUSP14;VPREB3;EEF1A1;ARL6IP5;TNFSF10;APBB2;RPL37;HSPA1B;MDFI;TPT1
TNFSF10
CEMIP
EFNA1
CXCL11
TRPC6
TPT1
TNFSF10
CEMIP
RNASE3
TRPC6
DNTT
LAMP5;MFSD5;TNFSF10
RPS6KA2;MYO3A
SRGN;RPL11;RPS6;SLC16A3
IFITM1;RPL31;GAD1;NAV2;SEPP1;HK2;DOK4;ARL6IP5;B3GNT2;APBB2;ITM2A;SLC16A3;HSPA1B
CTSG
IFNB1
TNFSF10
BEX1
RPL31
SLC16A3
HSPA1B
HSPA1B
HK2
CEMIP;CXADR;RPL31;FEM1B;DOLK;RPL37;HK2
HBG2;TNFSF10
EFNA1;KBTBD4;HSPA1B;DUSP14
HOXA1;NAV2
RPS6KA2
RPS6KA2
IL12A
ARL6IP5
VPREB1
RPL37A
RPL37A
RPL37A
HILPDA
CTSG
IFITM1;CLP1;TNFSF10;HSPA1B
HILPDA;SLC16A3;HK2
IFITM1;TMEM63A;RPS7;RPL31;HHIP;IFI6;NAV2;RPL10A;SEPP1;RPL7;RPS25;RPS27;RPS29;C6ORF106;RPS6KA2;RPL37A;TNFSF10;RPL27;RPS20;FAM32A;RPS21;RPL39;UBA52;LY6E
EPHB4
EPHB4
HSPA1B
RPL37A
TNFSF10
HSPA1B
HSPA1B
IFIH1
HSPA1B
DNTT
RAPGEF5
SRGN;RPS7;MFSD5;RPL11;RPS6;GDE1;RPL13A;SLC16A3;HSPA1B;LY6E
RPL41;RPS17;RPS27;RPL34;TNFSF10;RPS20;RPS27A;UBA52;TPT1
CEMIP;NAV2
EEF1A1;RPL12;HHIP;RPS6;TNFSF10;RPSA;DUSP14
EPHB4
CLDN9
IL12A
CEMIP
HSPA1B
TNFSF10

CLP1;HILPDA;HK2
TNFSF10;RPS12
TNFSF10;IL12A
SRGN;RPL11;RPS6;LY6E
EPHB4
EPHB4
SLC16A3
RPL37A
C1QA
EFNA1;MFSD5;FEM1B;HILPDA;RPL37A;DOLK;NRBF2;HSPA1B
WT1;HILPDA;RPL37A;TNFSF10;TOMM20;SLC16A3;HK2
MARC2;RAPGEF5
ARL6IP5;HSPA1B
FCRLA;RPS5;TNFSF10;SEPP1;HSPA1B;RPL19
SAMD9;HBBP1;SEPP1
HILPDA;HK2
RPS17;RPL41;RPL35;TOMM20
TNFSF10
NT5DC3
GNG12
TNFSF10
CEMIP
ARPP21
CXADR;ARL6IP5;B3GNT2;HSPA1B;EPHB4
RPL3;GDE1;SLC16A3
EPHB4
HSPA1B
HSPA1B
DOK4
LAMP5
SRGN;C6ORF106;RPL11;RPS6
WT1;RPS6KA2;TNFSF10
LAMP5;GDE1;SLC16A3;HSPA1B
C1QA;NPTX1;SEPP1;MDFI
BEX1;RPL31
WT1;RPS6
HOXA1
HSPA1B
TPT1
HILPDA
IFIH1;IFITM1;TNFSF10;IFI6;SLC16A3;LY6E
HILPDA;HK2
RPL37A;B3GNT2
HSPA1B
HBG1
LAMP5
IFNB1
RNASE3
NAV2
RPL37A
RPS6KA2
RPL31
CXCL11
SLC16A3
RPL37A
KCNJ12
RPS6
HSPA1B
DNTT
KCNJ12
HSPA1B;DUSP14
SRGN;RPS7;MFSD5;RPL11;HILPDA;RPS6;B3GNT2;RPL13A;SLC16A3;TM4SF1;LY6E
CD274;DOK4;ARL6IP5;ABCA13;MDFI
RPS6KA2
MYO3A
RPS6KA2
RPS6KA2
TNFSF10
RPL37A
HSPA1B
EFNA1
IFNB1
DNTT
DTX1
HILPDA;B3GNT2;HSPA1B;HK2
CXCL11
RPS6
TNFSF10
TM4SF1
RPL37A
HSPA1B
HSPA1B
MDFI
TNFSF10
RNASE3

HBG2;DNTT;RAPGEF5
RPS6;TNFSF10
TPT1
HSPA1B
RPL37A
ARL6IP5
GDE1
SRGN
ARL2BP
RPS6
TRPC6
DEFA3;NAV2;HSPA1B
CLP1;WT1;MFSD5;HILPDA;RPL37A;HOXA1;DOLK;HSPA1B;HOXB5
EFNA1;SRGN;CEMIP;DOK4;ARL6IP5;TNFSF10;NAV2;CCDC102A;SLC16A3
EFNA1;EPHB4
TNFSF10
IFNB1
RPL37A
TNFSF10
RPS6
TRPC6
HBG2;HSPA1B
EFNA1;IFIH1
KBTBD4;CLP1;HILPDA;RPL37A;B3GNT2;DOLK;HSPA1B
RPS6KA2
RPS6KA2
RPS6KA2
HK2
WT1
HILPDA
CD274
RAPGEF5
WT1;HILPDA;RPS6;TNFSF10;SEPP1;TM4SF1;HSPA1B;HK2
TMEM63A;BEX1;CXADR;RPL31;WT1;HILPDA;HOXA1;HSPA1B
EPHB4
TOMM20
WT1
GDE1
HSPA1B
TPT1
HSPA1B
TM4SF1
EPHB4
RNASE3
MFSD5;FEM1B;HILPDA;RPL37A;C3ORF52;HSPA1B
HSPA1B
HK2
HSPA1B
RPL31
RPS6
IFITM1;RPL32;RPL31;RPL34;RPLP0;HK2;RPL7;IFIH1;RPS14;RPS17;C6ORF106;MFSD5;RPS6KA2;ARL6IP5;RPL36;TNFSF10;HOXA1;IL12A;SLC16A3;EPHB4;SRGN;TRPC6;CXADR;RPS7;RPS6;RPL13A;RPSA;PAX5;NAV2;COMMD6;SEPP1;TM4SF1;EEF1A1;CLDN9;RPL37A;CMPK2;B3GNT2;ROR1;ZNF597;LY6E;CEMIP;SAMD9;IFI6;RPS3;RPS27A;RPL41;HBG2;HBG1;FEM1B;CYP4F3;RP
WT1;DNTT;RAPGEF5
RPS6KA2;TNFSF10
RPS6KA2
CEMIP
CEMIP
TNFSF10
NAV2
RNASE3
IFIH1
RPS14;TMEM63A;RPL31;RPS6KA2;HILPDA;RPL37A;NT5DC3;TOMM20;HSPA1B;HK2
SRGN;RPL11;RPS6;RPL13A;SLC16A3
CTSG
RPL37A
RPL11;LY6E
BEX1;NT5DC3;HOXA1
SAMD9;RPL35A;HSPA1B
WT1;HILPDA;SLC16A3;HSPA1B
HRK;ZNF792;IFITM1;HHIP;RPLP0;IFI6;AEBP1;CST7;RPS6KA2;NT5DC3;ARL6IP5;TNFSF10;NPTX1;SLC16A3;VAT1L;RAG1;SRGN;NPM2;CXADR;IFNB1;RPSA;SEPP1;EFNA1;DOK4;ZNF597;TPT1
MYO3A
CD244
HSPA1B
PTGDR2
TNFSF10
SAMD9;PTGDR2;IFNB1;RPS8;FEM1B;DEFA3;DNTT;AEBP1;POU4F1;RPS14;CXCL11;DOK4;WT1;COL5A3;AMDHD2;RPS6KA2;TNFSF10;ROR1;CLC
WT1;IL12A;SLC16A3
FCGR3B;GAD1
CXCL11;FCGR3B;GAD1;CYP4F3;CTSG;CST7;SEPP1;HSPA1B;DUSP14
CD274
CD274
RPS6KA2
EPHB4
EPHB4
RPL35A
HILPDA

HSPA1B
KBTBD4;CLP1;MFSD5;RPL37A;B3GNT2;DOLK;HSPA1B
RPS6KA2;MYO3A;EPHB4
IL12A
EPHB4
TNFSF10
RPL37A
RPL31;RPL37A;HSPA1B
CXADR;RPS6KA2;GDE1;HOXA1;TM4SF1;HSPA1B
IFNB1;HILPDA
RPL31
RPS6
HILPDA
KBTBD4;HSPA1B;DUSP14
TNFSF10;TPT1
RPS6KA2;MYO3A;EPHB4
SRGN;RPL11;RPS6
TNFSF10
IFIH1
CXCL11
TNFSF10
RPS6KA2
HBG1
BEX1;TNFSF10
HILPDA;HK2
IFITM1;RPSA
IFNB1
TPT1
IL12A
RAPGEF5
HBG2
TNFSF10
TNFSF10
RPS6KA2
GDE1
HSPA1B
EPHB4
WT1
SRGN;BEX1
HOXA1;CD244
RPS6KA2
HSPA1B
EFNA1
IFNB1
TNFSF10
GAD1
RPL37A;HSPA1B
EFNA1;CLP1;RPL31;MFSD5;HSPA1B;HOXB5;DUSP14
TPT1
HSPA1B
RPL37A
TNFSF10
TNFSF10
WT1
RPS6KA2
WT1;IL12A;SLC16A3
RPS6;TOMM20;RPS12
RPS6KA2;GDE1;POU4F1;HSPA1B
TRPC6
TNFSF10
HSPA1B
CXADR
TNFSF10;IL12A
MIR23A
HSPA1B
WT1
IFIH1
RAPGEF5
BEX1;RPL31;WT1;RPL37A;NT5DC3;C3ORF52;B3GNT2;HOXA1
DOK4;MARC2;GAD1;APBB2;ITM2A
EPHB4
TNFSF10
TNFSF10
IL12A
IFNB1
MFSD5;FEM1B;HILPDA;RPL37A;C3ORF52;B3GNT2;HSPA1B
RPS6KA2;EPHB4
RPL37A
HSPA1B
TNFSF10
DNTT
CXCL11;IL12A
IFIH1;SAMD9;CYP4F3;RPL37A;RPS3A
HSPA1B
RNASE3

HSPA1B
RAPGEF5
RPL11;IL12A;LY6E;DUSP14
IFIH1;IFI6
HSPA1B
IL12A
HSPA1B
SRGN
EPHB4
HSPA1B
CXADR;CLP1;RPS6KA2;R3HCC1;GDE1;APBB2;GOLGA7;POU4F1;HSPA1B
EEF1A1;HOXA1;RPSA
HHIP;TNFSF10
C3ORF52;HSPA1B
RPS20
WT1
WT1
SLC6A19
IFIH1;FEM1B;B3GNT2;DOLK;HSPA1B
B3GNT2;HSPA1B
C6ORF106
HK2
RPL37A
RPS6
TNFSF10
TNFSF10
KBTBD4;HK2
CXADR;HOXA1
CXADR;DOK4;PTGDR2;MARC2;GAD1;CLC
HSPA1B
HBG2
RPS6KA2
RPL31
RPL37A;RPS3A;TM4SF1
VPREB1
GDE1
HOXA1
RPL31;HSPA1B
CXADR;IFNB1;RPL37A;RPL35A;HSPA1B
CEMIP;IFI6
SAMD9
TRPC6
RPS19;DOK4;RPS3A
TNFSF10
WT1;HILPDA;RPL37A;SLC16A3;HK2
CXADR;DOK4;CLP1;RPS6KA2;HOXA1;NAV2;SEPP1;HSPA1B
IFI6;HSPA1B
GDE1
HSPA1B
HSPA1B
EEF1A1;C1QA;HOXA1;RPSA;SEPP1
RPL31;RPS20
TNFSF10
IFIH1
SRGN;NKG7
HILPDA;TNFSF10;APBB2
HSPA1B
LY6E
SAMD9
HSPA1B
KBTBD4;BEX1;CXADR;WT1;C6ORF106;FEM1B;HILPDA;RPL37A;HOXA1
WT1;HILPDA;TNFSF10;CD244;HK2
HSPA1B
IFIH1;EFNA1;HSPA1B
HSPA1B
HSPA1B
TNFSF10
DNTT
WT1;CTSG;HK2
BEX1
SRGN
DNTT
BEX1;HOXA1
CD274;HSPA1B
BEX1;RPL37A;C3ORF52;HOXA1
EEF1A1
EPHB4
EPHB4
BEX1
WT1;RPS6;HSPA1B
RPS6KA2
RPS6KA2
RPS6KA2
EPHB4
TNFSF10

TNFSF10
PTGDR2;RNASE3
NAV2
FEM1B;HILPDA;DOLK;EFNA1;KBTBD4;CLP1;MFSD5;RPL37A;C3ORF52;B3GNT2;APBB2;NRBF2;HSPA1B
IL12A
TNFSF10
DNTT
FEM1B;RPL37A;B3GNT2;APBB2;DOLK;HSPA1B
HILPDA;HK2
KIAA1217;RPS6
EPHB4
RPS6KA2
WT1
HSPA1B
RAPGEF5
DNTT
RPS6KA2;MYO3A;EPHB4
TMEM63A;MFSD5;RPL37A
RPS6KA2;IFI6;ROR1
IFITM1;SAMD9;HBG1;TNFSF10;IFI6;PAX5;COMMD6
KBTBD4;CXADR;WT1
TNFSF10;DEFA3;CST7;RPL39
RPS6
RPL31
DUSP14
EFNA1
SEPP1
HSPA1B
RAPGEF5
VPREB3;TMEM104;RPL31
EPHB4
RPL37A
TNFSF10
RPS6KA2;MYO3A;EPHB4
EFNA1;SRGN;CD274;TNFSF10;RAPGEF5;ITM2B
PTGDR2;IFI6
DNTT;RAPGEF5
SRGN;RPL11;RPS6;GDE1;LY6E
TNFSF10
ROR1
SLC16A3
RPL37A;TOMM20;HSPA1B
SRGN;CD274;RPL31;CYP4F3;HILPDA;RPL7;IFIH1;CXCL11;DOK4;MFSD5;POU2AF1;RPL37A;MYO3A;TNFSF10;HBBP1;ITM2A
EPHB4
IFIH1
HOXA1
IFNB1
LAMP5;WT1;CTSG;HSPA1B
IFIH1;FEM1B
TRPC6;PTGDR2
WT1;TNFSF10
EFNA1
TNFSF10
IFIH1;FEM1B;CLDN9;RPL37A;B3GNT2;APBB2;DOLK;HSPA1B
HILPDA;HK2
NKG7;NAV2
RNASE3;TPT1
HSPA1B
HSPA1B
TNFSF10
HBG2;CD244
TPT1
HSPA1B
BEX1;RPS6KA2
TNFSF10;DNTT
IFIH1;BMP3;RPS6
CXADR;HOXA1;SEPP1
RPS6
NAV2
RPS6KA2;MYO3A
RPS6KA2
RPS6
HSPA1B
RPL31
RPS6KA2
DNTT
HOXA1;HSPA1B
HSPA1B
IFI6
TRPC6
TNFSF10
HBG2
SRGN;IFITM1;BEX1;CXADR;HILPDA;GDE1;RPL13A;SEPP1;TM4SF1;IFIH1;RPS19;ARL6IP5;TNFSF10;NRBF2;HSPA1B;ITM2B;EPHB4
TNFSF10;DNTT
WT1

WT1
DNTT
PAX5
IFNB1
TNFSF10
IFIH1
RAPGEF5
RPL31;HOXA1;SLC16A3;HOXB5;LY6E
PTGDR2;AMDHD2;APBB2
HSPA1B
DNTT
SRGN;HBG2;GAD1;HILPDA;ARL6IP5;TNFSF10;CTSG;TOMM20;HK2
EFNA1;DOK4
RPL31;HSPA1B
PAX5;RAG1
HBG2;RNASE3
EEF1A1;RNASE3
RPS6KA2
SLC16A3
IFNB1
TNFSF10
TNFSF10
RPS6KA2
EPHB4
IFNB1;RAPGEF5
TRPC6;RAPGEF5
WT1;NRBF2
WT1;RAPGEF5
IFNB1;DNTT
RAPGEF5
RPS21
B3GNT2;IL12A;ROR1
RPS6KA2
NME8;CYP4F3
CYP4F3
TNFSF10
ROR1
RPL31;IFNB1;WT1;RPS6;TNFSF10;HOXB5
CLP1;C6ORF106;IFI6;B3GNT2
IFNB1;RAPGEF5
RPS6;RAPGEF5
CXADR;C6ORF106;GAD1;RPS6KA2;IL12A;PAX5;NAV2;HSPA1B
MYO3A
HRK
WT1
TNFSF10
WT1
EFNA1
TNFSF10
RAPGEF5
EPHB4
TNFSF10;DNTT
PTGDR2;MARC2;AEBP1
HK2
EPHB4
EEF1A1
RAPGEF5
TNFSF10
WT1;CTSG
GAD1;RPSA;EPHB4
HSPA1B
RPS6KA2;MYO3A
RPSA
TNFSF10;VAT1L
EFNA1;GAD1;C3ORF52;ROR1
HSPA1B
TNFSF10
IFIH1
CD274;IFITM1;ARL6IP5;TPT1;LY6E
HILPDA;HSPA1B
HILPDA
RPSA
CLDN9;GAD1
FCRL1;HRK;LINC00472;NME8;MYO3A;TNFSF10;CST7
NKG7
HSPA1B
RPL31;RPS3A
RPS6KA2;MYO3A
TNFSF10;DNTT
EFNA1;GAD1;RPL37A;C3ORF52;HOXA1;HSPA1B
EPHB4
EPHB4
COL5A3
RPS6
TNFSF10;IL12A

HRK;CXCL11
EPHB4
EPHB4
TNFSF10
EFNA1
IFIH1;FEM1B;B3GNT2;APBB2;DOLK;HSPA1B
RPS6KA2
MFSD5;HILPDA;ARL6IP5;DOLK;RAPGEF5;LY6E
HSPA1B
BMP3
EEF1A1;ADGRE1;TNFSF10;NKG7;SLC16A3;LY6E
RAPGEF5
SRGN;TNFSF10;RPS3A
MYO3A
TNFSF10
BEX1;CXADR;RPL31;WT1;RPL37A;NT5DC3;C3ORF52;HOXA1
HSPA1B
EFNA1
WT1
RTP4;CYP4F3
RPS6KA2
RPS6KA2
SEPP1;POU4F1;HSPA1B;TPT1
TNFSF10;HSPA1B;HK2
RPS3A;RAG2;TM4SF1
RPS6KA2
EFNA1
SRGN;IFITM1;HHIP;IFI6;TM4SF1;DUSP14;EEF1A1;RPS25;WT1;TNFSF10;C3ORF52;RAPGEF5;HSPA1B;EPHB4;LY6E
RNASE3;HSPA1B
CDHR2;TM4SF1
TNFSF10
PAX5
EFNA1;MFSD5;HILPDA;B3GNT2
RPS6;TNFSF10
EFNA1;IFNB1;HILPDA;TNFSF10;CMPK2;MDFI
ARL6IP5;TNFSF10
RPL37A
RPL37A;C3ORF52;TM4SF1
HSPA1B
COL5A3;HILPDA;IL12A;HK2
CXADR;RPS6KA2;C3ORF52;HOXA1;SLC16A3;HSPA1B;DUSP14
C6ORF106;TM4SF1
IFIH1;WT1
CMPK2
B3GNT2
RTP4;RPS3A
HSPA1B
GDE1
HSPA1B
RPL37A;HSPA1B
CXADR;HOXA1;HSPA1B;DUSP14
RPL37A
HILPDA
RAPGEF5
IFITM1;CXCL11
CXADR;APBB2;GNG12
CD274;RPS6;ROR1;SEPP1;EPHB4
CLP1;RPL31;MFSD5;CLDN9;B3GNT2;RPS20;DUSP14
WT1;CTSG
TNFSF10;RPL37
HSPA1B
RPS6KA2;EPHB4
EPHB4
GDE1
IFIH1
FEM1B;HILPDA;DOLK;NAV2;EFNA1;CLP1;WT1;MFSD5;RPL37A;C3ORF52;B3GNT2;NRBF2;HSPA1B
GDE1;SLC16A3
RPL30;GDE1
EPHB4
BEX1
CYP4F3
DOK4;PAX5;AEBP1;ITM2A
SRGN;TNFSF10;ROR1;RAG1
IFIH1;FEM1B;DOLK;HSPA1B
C6ORF106;SLC16A3;HSPA1B
LAMP5;WT1;MFSD5;RPL37A;GDE1;ARL6IP5;SLC16A3;HSPA1B
IFIH1;NME8;CYP4F3
EPHB4
CD274;CMPK2
TMEM63A
EEF1A1;TOMM20
EPHB4
DUSP14
FEM1B;HILPDA;HSPA1B
TNFSF10

CXADR;DOK4;CLP1;RPS6KA2;SEPP1;GOLGA7;HSPA1B
EEF1A1;TOMM20
EFNA1
FCRL1;ADGRE1;CLP1;PTGDR2;DAAM2;GAD1;TNFSF10;HSPA1B;HOXB5
B3GNT2
CYP4F3
WT1
EPHB4
EFNA1
FEM1B
RPS6KA2
BEX1
EFNA1;KBTBD4;CLP1;WT1;MFSD5;FEM1B;HILPDA;RPL37A;NRBF2;NAV2;HSPA1B
TMEM63A;WT1;RPS6KA2;HILPDA;TNFSF10;CTSG;HSPA1B;CD244;HK2
EPHB4
HSPA1B
TNFSF10
MYO3A
EPHB4
IFITM1;CXADR
EEF1A1
EEF1B2;AOX2P;RPL30;TRPC6;CLP1;FUNDC1;SEPP1;CD244;EPHB4
HSPA1B
WT1;RPS6KA2;R3HCC1;HILPDA;RPL37A;TNFSF10;CTSG;CST7;TOMM20;CD244;HK2
CXCL11
HBG2
FEM1B;DOLK;HSPA1B;HK2
CXADR;BEX1
TNFSF10;TM4SF1
IFITM1;RPS19;WT1;GAD1;TNFSF10;B3GNT2;NPTX1;CST7;HSPA1B
LAMP5;ARL2BP;MFSD5;RPS6KA2;ARL6IP5;SLC16A3;HSPA1B
RPS6;IL12A
RTP4;DOK4;NME8;RPS3A
EFNA1;TM4SF1
FEM1B;HILPDA;DOLK;NAV2;HK2;EFNA1;KBTBD4;CLP1;MFSD5;RPL37A;C3ORF52;APBB2;NRBF2;HSPA1B
SRGN;RPL11;RPS6
RPS6KA2
RPS6KA2
CTSG
TNFSF10
RAPGEF5
NT5DC3
TNFSF10
HRK;HSPA1B
B3GNT2
RPS6KA2;MYO3A
SLC16A3
WT1
IFIH1;HSPA1B
IFIH1;BMP3;CXCL11;FCGR3B;SAMD9;DOK4;NME8;CYP4F3;RAG2
CEMIP;RPL37A;B3GNT2
FEM1B;HILPDA;DOLK;NAV2;EFNA1;KBTBD4;CLP1;WT1;MFSD5;RPL37A;B3GNT2;HOXA1;NRBF2;HSPA1B
NRBF2
EFNA1;IFITM1;HILPDA;RPLP0;TNFSF10;RPL13A
TMEM63A;IL12A;DOLK;ITM2B
WT1
SAMD9
ROR1;TM4SF1;HK2
AEBP1
NAV2
HSPA1B
APBB2
ABCA13;MDFI
ARL6IP5;TNFSF10
WT1
EFNA1
GAD1
WT1;FEM1B;RPS6KA2;HILPDA;HSPA1B;HK2
HK2
RTP4;FCGR3B;RPL37A
TMEM63A;FEM1B;HILPDA;DOLK;NAV2;EFNA1;KBTBD4;CLP1;MFSD5;RPL37A;B3GNT2;APBB2;NRBF2;HSPA1B
NAV2
RPS6;ARL6IP5;TNFSF10;NKG7
HILPDA
DNTT
DNTT
HRK
WT1;CTSG
APBB2
B3GNT2
GAD1
EEF1A1
IFI6
HBG2
EFNA1;C3ORF52

SRGN;PTGDR2;CLC
EFNA1
TNFSF10
IFNB1
RPS6
IFITM1;TMEM63A;RPS7;IFI6;AEBP1;RPL10A;RPL7;RPS27;RPS19;DAAM2;RPS6KA2;TNFSF10;RPL27;APBB2;UBA52;LY6E
TNFSF10
IFIH1;LAMP5;TMEM63A;TNFSF10;HOXA1;NRBF2;CD244
HSPA1B
EFNA1
HSPA1B
HBG2
RPS6KA2
DOK4
HBG2
EFNA1;NTNG2;VPREB1;DOK4;DHH;RPL12;RPS5;NKG7;IL12A;HSPA1B
RPS25;POU2AF1;CYP4F3;IFI6
NME8;CYP4F3
HK2
RPS6KA2
PTGDR2
WT1
WT1
HBG2
KIAA1217
NTNG2;RPL31;HHIP;GAD1;CYP4F3;ROR1;ZNF597
BEX1
RAPGEF5
HHIP;IFI6
TRPC6
GAD1
LY6E
RAPGEF5
RAPGEF5
DNTT
TNFSF10
TNFSF10
FEM1B;HILPDA;DOLK;DUSP14;EFNA1;KBTBD4;CLP1;MFSD5;RPL37A;B3GNT2;APBB2;NRBF2;HSPA1B
WT1
RAPGEF5
TM4SF1
RAPGEF5
RAPGEF5
DNTT
TNFSF10
CXADR;DOK4;CLP1;RPS6KA2;HOXA1;NAV2;SEPP1;HSPA1B
HRK;HSPA1B
BEX1;RPS6KA2
WT1
EFNA1;GAD1;HOXA1;ROR1
SRGN;CD274;CXCL11;RPL31;CYP4F3;RPL37A;TNFSF10;HOXA1
TM4SF1
CD274
RPS6KA2
DNTT
DNTT
TNFSF10
RPS6KA2;MYO3A
DNTT
WT1
SRGN
C3ORF52;HSPA1B
FEM1B;EPHB4;RPL7
SAMD9;WT1;FEM1B;HILPDA;HK2
EPHB4
RAPGEF5
NTNG2;RPL31;HHIP;GAD1;ROR1;ZNF597
FEM1B
EFNA1;C3ORF52
NTNG2;RPL31;HHIP;GAD1;ROR1;ZNF597
WT1;TNFSF10;VAT1L
TNFSF10;IFI6
RPS6KA2
SRGN
TOMM20;HSPA1B
TNFSF10
RPS3A;RAG2
FEM1B;RPS6KA2;HILPDA;HK2
GDE1;B3GNT2;SLC16A3
PTGDR2;MARC2
HK2
CLP1;MFSD5;NRBF2;HSPA1B
WT1;CTSG;CD244;HK2
HSPA1B
SAMD9;RPL37A

CLP1
ARL6IP5;SLC16A3
PTGDR2;HBG1;ROR1;GCSAM;CD244
FEM1B;HILPDA;DOLK;NAV2;EFNA1;KBTBD4;CLP1;WT1;MFSD5;RPL37A;C3ORF52;NRBF2;HSPA1B
SAMD9;NME8;CYP4F3
TNFSF10
IFIH1;RTP4;DOK4;NME8;RPS6;APBB2;ROR1;RPS3A;HBBP1;TM4SF1;MDFI
EFNA1;C3ORF52;DUSP14
SRGN
WT1;FEM1B;HILPDA;HK2
HSPA1B
ITM2B
RPL37A;GDE1
EFNA1;CLDN9
KBTBD4;WT1
NPTX1
MIR27A
WT1;CTSG;CD244;HK2
TNFSF10
HOXA1
RPL37A
BEX1
EFNA1;NME8;CYP4F3
EFNA1;C3ORF52;B3GNT2
EFNA1;C6ORF106;B3GNT2;TM4SF1
TNFSF10;ROR1;DUSP14
EFNA1
B3GNT2;DOLK
IFIH1;TNFSF10;CD244
HSPA1B
SRGN;CEMIP;TRPC6;SAMD9;HBG2;HBG1;DEFA3;NKG7;PAX5;KIAA1217;EFNA1;CXCL11;WT1;TNFSF10;CMPK2;RPS20;IL12A;ITM2A;HSPA1B
SRGN
DNTT
GDE1
EFNA1
EFNA1;GAD1;HOXA1;ROR1;NPTX1;SLC26A6
WT1;CTSG;HK2
EFNA1;CEMIP;GAD1;C3ORF52;TM4SF1;SLC26A6;DUSP14
KBTBD4;ROR1
C3ORF52;IL12A;DUSP14
RTP4;IFITM1;BEX1;HILPDA;RPL9;HK2;RPL7;DUSP14;FCGR3B;ADGRG5;ARL6IP5;HOXA1;RPL39;SLC16A3;IFNB1;GAD1;RPL13A;DOLK;RPS3A;NAV2;SEPP1;EFNA1;MIR24-2;RPS27;CLDN9;HSPA1B;ITM2B;TPT1
SRGN;RPL37A;GDE1;TM4SF1
IFIH1
C1QA;IFITM1;CEMIP;SAMD9;RPL31;PTGDR2;RPLP0;IFI6;TOMM20;HK2;RPL7;IFIH1;VPREB1;RPS17;MARC2;FUNDC1;TNFSF10;RPS3;APBB2;NPTX1;RPL41;NPM2;CXADR;HBG1;GAD1;RPS6;RPL13A;NAV2;COMMD6;SEPP1;TM4SF1;EEF1A1;EFNA1;CXCL11;RPS27;DOK4;DAAM2;POU2AF1;FAM32A;ROR1;RAPGEF5;SLC26A6
RPS6KA2
NRBF2;NAV2
BEX1;RPS6KA2
EFNA1;C3ORF52
IFITM1;RPL30;BEX1;IFI6;DTX1;HK2;BEGAIN;LAMP5;EEF1B2;ADGRE1;RPS14;TMEM104;C6ORF106;ARL6IP5;TNFSF10;RPS3;HOXA1;APBB2;IL12A;CTSG;NPTX1;SLC16A3;VAT1L;NTNG2;ARPP21;IFNB1;KCNJ12;MIR23A;GAD1;CYP4F3;DEFA3;NKG7;RPSA;PAX5;NAV2;KIAA1217;EEF1A1;EFNA1;CXCL11;DOK4;WT1;POU2AF1;ROR1;ITM2A;ITM2B;TPT1;HOXB5;LY6E
GDE1
EFNA1;C3ORF52
DOLK
BEX1;WT1;RPLP0;GOLGA7;HSPA1B
IFIH1;LAMP5;TNFSF10;HOXA1;NRBF2;CD244
CXADR;C6ORF106;ARL6IP5;SLC16A3
CLC
FEM1B;HILPDA;HK2
ARL6IP5;TM4SF1;HSPA1B
B3GNT2
CYP4F3;RPS6
CYP4F3;CMPK2
NTNG2;CD274;CXCL11;CXADR;SAMD9;RPS19;DOK4;RPS6;B3GNT2;TM4SF1
TNFSF10
EFNA1;MFSD5;RPL37A;B3GNT2
IFITM1;CYP4F3;HILPDA;TNFSF10;ROR1
EEF1A1;HHIP;RPS6;GDE1;APBB2;RPSA;RPL37;TOMM20;SLC16A3;MDFI
FEM1B;SLC16A3
HRK;RPL31;RPL35A;SLC26A6;HK2
CXCL11;KCNJ12;NPTX1;ITM2A;POU4F1;VAT1L
KBTBD4;WT1;MFSD5;HK2
EFNA1
RPS6KA2
EFNA1;KBTBD4;MFSD5
EFNA1
PTGDR2
IFIH1;EFNA1;CEMIP;NRBF2
SLC16A3
HRK;RPL31;HHIP;RPLP0;IFI6;ABCA13;HK2;IFIH1;CECR2;CLP1;C3ORF52;APBB2;IL12A;RPL37;SLC16A3;VAT1L;RAG1;NTNG2;TRPC6;CXADR;GAD1;NAV2;GNG12;SEPP1;POU4F1;KIAA1217;EFNA1;KBTBD4;RPS27;WT1;DAAM2;CLDN9;COL5A3;ROR1;ITM2A;TPT1;RPS23
CXADR;RPL37A;SLC16A3
WT1;FEM1B;HILPDA;HK2
RPL31
RPS25;RPS19;DOK4;RPS29;RPS18;TNFSF10;SLC16A3;HK2;EPHB4
MIR23A;HHIP;RPS6;RPS3A;SEPP1;HK2;EFNA1;DOK4;TNFSF10;HOXA1;IL12A;HSPA1B;TPT1
EFNA1;KBTBD4
NTNG2;CD274;CEMIP;CXADR;SAMD9;HHIP;SEPP1;TM4SF1;EEF1A1;CXCL11;RPS19;DOK4;ARL2BP;POU2AF1;B3GNT2;ROR1;RAG1

ADGRE1;PYURF;FEM1B;AMDHD2;TNFSF10;TM4SF1;HSPA1B;HK2
GDE1;GOLGA7
EFNA1
SRGN;MFSD5;GDE1;NRBF2;GOLGA7;TM4SF1
MARC2;CYP4F3;RPL37;HSPA1B;HK2;BEGAIN
TMEM63A;CXADR;C6ORF106;ARL6IP5;SLC16A3
SAMD9;RPL34;RPS3A;TOMM20;TM4SF1;HK2;PDRG1;IFIH1;EEF1A1;EFNA1;CLP1;RPL37A;TNFSF10;APBB2;RPL37;SLC16A3
HRK;CXADR;GAD1;PDRG1;EEF1A1;RPS19;POU2AF1;RPL37A;ARL6IP5;RPL35;B3GNT2;NRBF2;HSPA1B
NRBF2
EFNA1;KBTBD4;MFSD5;FEM1B;HILPDA;RPL37A;B3GNT2;APBB2;DOLK;NRBF2;HSPA1B
EFNA1
EFNA1;KBTBD4;CLP1;MFSD5;FEM1B;HILPDA;RPL37A;DOLK;NRBF2;HSPA1B;DUSP14
RPS3A
CXADR;HILPDA;APBB2;ROR1;NAV2;HK2
SRGN;CEMIP;RPSA;ABCA13;EEF1A1;WT1;MARC2;RPS6KA2;B3GNT2;HOXA1;RPL37;RAPGEF5;LY6E
EFNA1
ARL6IP5;NRBF2
FEM1B;CYP4F3;TNFSF10;B3GNT2;DOLK;NRBF2;LY6E
FCGR3B;SAMD9;CYP4F3;CDHR2
MFSD5;C3ORF52;APBB2;SEPP1;SLC16A3;HSPA1B;PDRG1
IFIH1
CXADR
CMPK2
IFIH1
SAMD9;RPL34;RPS3A;TOMM20;TM4SF1;HK2;PDRG1;IFIH1;EEF1A1;EFNA1;CLP1;RPL37A;TNFSF10;APBB2;RPL37;SLC16A3;HSPA1B
LAMP5;CLP1;MFSD5;FEM1B;RPS6KA2;NT5DC3;B3GNT2;FAM32A;CST7;SLC16A3;HSPA1B
EEF1A1;EFNA1;APBB2;SEPP1;TM4SF1
IFITM1;CEMIP;RPL3;BEX1;SAMD9;GDE1;IFI6;CST7;ABCA13;HK2;RPL7;DUSP14;VPREB3;IFIH1;TMEM104;MFSD5;RPS6KA2;TNFSF10;C3ORF52;APBB2;NPTX1;SLC16A3;SRGN;LINC00472;NPM2;TRPC6;CXADR;GAD1;MIR27A;NAV2;SEPP1;POU4F1;TM4SF1;EEF1A1;EFNA1;RPS25;ANGPTL6;POU2AF1;ROR1;RAPGEF5;UBA52;HSPA1B;ITM2B;SLC26A6
WT1;HILPDA;HSPA1B;HK2
ROR1
APBB2;DOLK;NAV2;HSPA1B
IFIH1;C6ORF106;FEM1B;B3GNT2;NRBF2;DUSP14
WT1;GDE1;B3GNT2;NRBF2;CST7;GOLGA7;SLC16A3
KBTBD4;FEM1B;HILPDA;RPL37A;C3ORF52;B3GNT2;APBB2;DOLK;NRBF2
WT1
WT1;CST7
SRGN;RPL3;RPL37A;ARL6IP5;IFI6;CST7;SLC16A3
FEM1B;NRBF2;DUSP14
HHIP;HILPDA;HK2;RPS19;C6ORF106;RPS6KA2;APBB2;NRBF2;GOLGA7;SLC16A3;EPHB4;FEM1B;RPL35A;NAV2;GNG12;EEF1A1;WT1;RPL37A;B3GNT2;RPL27;UBA52;HSPA1B;ITM2B;MDFI;LY6E
RTP4;CD274;RPL31;HILPDA;GDE1;HK2;RPL7;DUSP14;FCRLA;IFIH1;CLP1;TNFSF10;HOXA1;FEM1B;MIR23A;CYP4F3;MIR27A;RPL35A;GCSAM;CXCL11;DOK4;RPL37A;ROR1;HBBP1;ITM2A;HSPA1B
EFNA1;C1QA;TRPC6;ARPP21;R3HCC1;TM4SF1;EPHB4
WT1;RPL37A;NRBF2;HSPA1B
RTP4;CD274;TMEM63A;CYP4F3;HK2;RPL7;DUSP14;FCRLA;IFIH1;CXCL11;CLP1;TNFSF10;IL12A;ROR1;RPL37;ITM2A
SRGN;WT1;RPL37A;GDE1;ARL6IP5;NRBF2;CST7;GOLGA7;SLC16A3
GDE1;SLC16A3
WT1;KCNJ12;MYO3A;B3GNT2;PAX5;HSPA1B
CEMIP;BEX1;SAMD9;HILPDA;IFI6;HK2;IFIH1;CLP1;ARL6IP5;TNFSF10;C3ORF52;APBB2;SLC16A3;HBG2;GAD1;RPSA;DOLK;NAV2;TM4SF1;EFNA1;KBTBD4;POU2AF1;CMPK2;HSPA1B;RPS23
EFNA1
EFNA1;GAD1
EFNA1;EEF1B2;ARL6IP5;CCDC102A;RPL9;HSPA1B;SLC26A6;EPHB4;BEGAIN
WT1;RPL37A;GDE1;ARL6IP5;SLC16A3
NTNG2;IFITM1;RPL32;RPL34;HILPDA;RPLP0;IFI6;CCDC102A;TOMM20;VPREB3;RPS17;DOK4;CLP1;RPS29;MFSD5;RPS6KA2;FAM32A;ZNF689;RPS27A;SLC16A3;HSPA1B;LY6E
WT1;HILPDA;RPL37A;C3ORF52;NRBF2;NAV2;HK2
HILPDA;HK2;DUSP14;CLP1;MFSD5;NT5DC3;C3ORF52;HOXA1;APBB2;NRBF2;SLC16A3;KCNJ12;FEM1B;GAD1;RPL35A;DOLK;NAV2;EFNA1;KBTBD4;WT1;RPL37A;B3GNT2;ROR1;HSPA1B;SLC26A6
RPL31;RPL37A;GDE1;ARL6IP5;NRBF2;SLC16A3
KBTBD4;WT1;MFSD5
RTP4;RPL30;SAMD9;HILPDA;IFI6;RPS14;RPS19;MARC2;FUNDC1;RPS6KA2;ARL6IP5;RPL36;TNFSF10;RPS3;C3ORF52;IL12A;NRBF2;NPTX1;SLC16A3;SRGN;CXADR;FEM1B;RPS6;SNHG6;NAV2;SEPP1;POU4F1;TM4SF1;PDRG1;EEF1A1;KBTBD4;DOK4;DAAM2;CDHR2;B3GNT2;RAPGEF5;RPS21;HSPA1B;SLC26A6;LY6E
EEF1A1;EFNA1;RPL31;RPL37A;C3ORF52;APBB2;SEPP1;TM4SF1
SRGN;NTNG2;SAMD9;IFI6;GNG12;SEPP1;TM4SF1;DUSP14;FCRLA;ADGRE1;CXCL11;CLP1;MARC2;RPS6KA2;C3ORF52;IL12A;ROR1;FAM81B;MDFI;SLC26A6
RTP4;IFITM1;CEMIP;DHH;RPL12;IFI6;RPL9;RPL7;IFIH1;TMEM104;MFSD5;FUNDC1;ARL6IP5;TNFSF10;APBB2;ZNF689;EPHB4;NTNG2;CYP4F3;RPS3A;NAV2;GNG12;TM4SF1;EFNA1;RPS25;CLDN9;CMPK2;B3GNT2;SLC26A6;RPS23
HRK;RPL3;RPL31;RPL34;HILPDA;RPLP0;GDE1;RPL10A;TOMM20;HK2;RPL7;EEF1B2;C6ORF106;MFSD5;RPS6KA2;ARL6IP5;TNFSF10;C3ORF52;ZNF689;RPL39;GOLGA7;SLC16A3;EPHB4;SRGN;CXADR;RPS3A;NAV2;SEPP1;PDRG1;EEF1A1;CLDN9;MYO3A;B3GNT2;FAM32A;ROR1;RAPGEF5;ZNF792;BEX1;AMDHD2;RPS3;APBB2;NPTX1;NTNG2;RPL41;NPM2;CYP4F3;DOLK;EFNA1;[illegible]
HRK;DPP10;IFITM1;RPL31;RPL34;HHIP;HILPDA;GDE1;RPL10A;CCDC102A;ABCA13;TOMM20;HK2;RPL7;IFIH1;LAMP5;EEF1B2;CLP1;MFSD5;RPS6KA2;ARL6IP5;RPL36;TNFSF10;C3ORF52;HOXA1;ZNF689;RPL37;FAM81B;RPL39;SLC16A3;RPS12;RAG1;TRPC6;CXADR;RPS3A;NAV2;COMMD6;SEPP1;TM4SF1;KIAA1217;EEF1A1;NME8;RPL37A;MYO3A;CMPK2;B3GNT2;RPL27;ROR1;[illegible]
CD274;IFITM1;TMEM63A;RPL34;GDE1;CCDC102A;TOMM20;HK2;RPL7;DUSP14;IFIH1;CLP1;TNFSF10;IL12A;ZNF689;RPL37;CYP4F3;SNHG6;PDRG1;POU2AF1;COL5A3;R3HCC1;RPL37A;ROR1;ITM2A;ZNF597;LY6E
APBB2;HK2

SUPPLEMENTARY TABLE 3: DRUG MATRIX TABLE

Term	Overlap	P-value	Adjusted P-value	Old P-value	Old Adjusted P-value	Odds Ratio	Combined Score	Genes
Lead_(II)_Acetate-600_mg/kg_in_Saline-Rat-Kidney-5d-up	19/328	3,74559E-07	0,002950024	0	0	3,981225379	58,91225243	C1QA;RPL32;RPS8;RPS5;RPL13A;RPL35A;RPSA;RPS3A;RPL10A;TOMM20;EEF1B2;RPS17;CXCL11;RPS19;ARL6IP5;RPS20;RPL39;GOLGA7;TPT1
Lead(IV)_Acetate-600_mg/kg_in_Saline-Rat-Kidney-5d-up	18/309	7,04103E-07	0,002772756	0	0	4,003603243	56,71641214	C1QA;RPL32;RPS8;RPS5;RPS6;RPL13A;RPL35A;RPSA;RPS3A;RPL10A;TOMM20;RPS17;CXCL11;RPS19;ARL6IP5;RPS20;RPL39;TPT1
Vancomycin-160_mg/kg_in_Saline-Rat-Kidney-5d-up	17/290	1,3217E-06	0,003469901	0	0	4,028913378	54,53775834	C1QA;RPL32;RPS5;RPS6;RPL13A;RPL35A;RPSA;RPS3A;RPL10A;SEPP1;RPS17;CXCL11;RPS19;ARL6IP5;RPL39;GOLGA7;TPT1
Zidovudine-14800_uM_in_DMSO-Rat-Primary_rat_hepatocytes-0.67d-up	17/293	1,52165E-06	0,002996125	0	0	3,987661706	53,41758663	RPL32;RPS8;FEM1B;RPS5;RPS6;RPL13A;RPL35A;RPS3A;PDRG1;EEF1B2;RPS17;RPS19;ARL2BP;CLP1;RPS20;RPL39;TPT1
Valproic_Acid-1500_mg/kg_in_Water-Rat-Heart-5d-up	17/299	2,00558E-06	0,003159188	0	0	3,907641738	51,26660933	C1QA;RPL32;RPS8;RPS5;RPS6;RPL35A;RPSA;RPS3A;RPL10A;SEPP1;RPS17;RPS19;FUNDC1;RPS20;RAPGEF5;RPL39;TPT1
Dibromochloromethane-325_mg/kg_in_CMC-Rat-Kidney-5d-up	17/305	2,62438E-06	0,003444933	0	0	3,830770097	49,22794991	RPL32;RPS8;RPS5;RPS6;RPL35A;RPSA;RPS3A;RPL10A;TOMM20;EEF1B2;RPS17;RPS19;ARL2BP;FUNDC1;RPS20;RPL39;TPT1
Aminosalicylic_Acid-2337_mg/kg_in_CMC-Rat-Liver-5d-up	17/307	2,86602E-06	0,003224685	0	0	3,80581394	48,57202543	RPL32;RPS8;FEM1B;RPS5;RPS6;RPL13A;RPL35A;RPSA;RPS3A;RPL10A;SEPP1;EEF1B2;RPS17;RPS19;RPS20;RPL39;TPT1
Clemastine-2500_mg/kg_in_CMC-Rat-Heart-5d-up	16/276	3,13639E-06	0,003087778	0	0	3,984262164	50,4903135	C1QA;RPL32;RPS8;RPS6;RPL35A;RPSA;RPS3A;RPL10A;SEPP1;EEF1B2;RPS17;RPS19;RPS20;RAPGEF5;RPL39;TPT1
Acyclovir-980_mg/kg_in_Saline-Rat-Bone_marrow-1d-dn	15/245	3,28747E-06	0,002876905	0	0	4,207868714	53,1259888	RPL32;RPS8;FEM1B;RPS5;NKG7;RPL13A;RPL35A;RPSA;RPS3A;RPL10A;EEF1B2;RPS17;RPS19;RPS20;RPL39
Dibromochloromethane-325_mg/kg_in_CMC-Rat-Kidney-3d-up	17/312	3,56022E-06	0,002804033	0	0	3,744823332	46,98138141	RPL32;RPS8;RPS5;RPS6;RPSA;RPS3A;RPL10A;SEPP1;EEF1B2;RPS17;CXCL11;RPS19;ARL6IP5;RPS20;RPL39;GOLGA7;TPT1
Estriol-313_mg/kg_in_CMC-Rat-Liver-1d-up	17/312	3,56022E-06	0,002549121	0	0	3,744823332	46,98138141	RPL32;RPS8;FEM1B;RPS5;RPS6;RPL13A;RPL35A;RPSA;RPS3A;RPL10A;EEF1B2;RPS17;RPS19;RPS20;RPL39;GOLGA7;TPT1
Lead(IV)_Acetate-600_mg/kg_in_Saline-Rat-Kidney-3d-up	18/347	3,66119E-06	0,002402962	0	0	3,565168306	44,62778523	RPL32;RPS8;RPS5;RPS6;RPL13A;RPL35A;RPSA;RPS3A;RPL10A;TOMM20;EEF1B2;RPS17;CXCL11;RPS19;ARL6IP5;RPS20;RPL39;TPT1
Cytarabine-23_mg/kg_in_Saline-Rat-Liver-3d-up	16/286	4,94446E-06	0,002995581	0	0	3,844952299	46,97471702	RPL32;RPS8;RPS5;RPS6;RPL35A;RPSA;RPS3A;RPL10A;SEPP1;TOMM20;EEF1B2;RPS17;RPS19;RPS20;RPL39;TPT1
Idarubicin-0.625_mg/kg_in_Saline-Rat-Heart-3d-dn	16/286	4,94446E-06	0,002781611	0	0	3,844952299	46,97471702	RPL32;RPS8;RPS5;RPS6;RPL35A;RPSA;RPS3A;TOMM20;EEF1B2;RPS17;RPS19;ARL2BP;FUNDC1;ARL6IP5;RPS20;TPT1
Cisplatin-2_mg/kg_in_Saline-Rat-Kidney-5d-up	17/322	5,41934E-06	0,002845517	0	0	3,628524471	43,99780345	RPL32;RPS8;RPS5;RPS6;RPL13A;RPL35A;RPSA;RPS3A;RPL10A;TOMM20;RPS17;CXCL11;RPS19;ARL6IP5;RPS20;RPL39;TPT1
Rolipram-75_mg/kg_in_CMC-Rat-Heart-5d-up	17/322	5,41934E-06	0,002667672	0	0	3,628524471	43,99780345	RPL32;RPS8;RPS5;RPL35A;RPS3A;RPL10A;TOMM20;EEF1B2;RPS17;ARL2BP;CLP1;FUNDC1;ARL6IP5;RPS20;RAPGEF5;RPL39;TPT1
Ketoconazole-114_mg/kg_in_Corn_Oil-Rat-Liver-3d-dn	16/292	6,43266E-06	0,002980213	0	0	3,765946429	45,01858528	C1QA;RPL32;RPS8;RPS5;RPS6;RPL35A;RPSA;RPS3A;RPL10A;SEPP1;EEF1B2;RPS17;RPS19;RPS20;RPL39;TPT1
Bromodichloromethane-430_mg/kg_in_CMC-Rat-Kidney-3d-up	16/302	9,81842E-06	0,004296106	0	0	3,641246217	41,98812019	RPL32;RPS8;RPS5;RPS6;RPSA;RPS3A;RPL10A;SEPP1;EEF1B2;RPS17;CXCL11;RPS19;CLP1;RPS20;RPL39;TPT1
Timolol-10_mg/kg_in_Water-Rat-Heart-0.25d-up	16/303	1,02319E-05	0,004241403	0	0	3,629228902	41,69983143	RPL32;RPS8;RPS5;RPL35A;RPSA;RPS3A;RPL10A;PDRG1;EEF1B2;RPS17;RPS19;ARL6IP5;RPS20;RAPGEF5;RPL39;TPT1
Aminosalicylic_Acid-2337_mg/kg_in_CMC-Rat-Liver-1d-up	16/304	1,06609E-05	0,00419826	0	0	3,617290649	41,41410147	RPL32;RPS8;RPS5;RPS6;RPSA;RPS3A;RPL10A;PDRG1;EEF1B2;RPS17;RPS19;ARL2BP;RPS20;RPL39;GOLGA7;TPT1
Cyclosporin_A-350_mg/kg_in_Corn_Oil-Rat-Kidney-5d-up	16/304	1,06609E-05	0,003998343	0	0	3,617290649	41,41410147	C1QA;RPL32;RPS8;RPS5;RPS6;RPL35A;RPSA;RPS3A;RPL10A;EEF1B2;RPS17;RPS19;ARL2BP;RPS20;RPL39;TPT1
Penicillin_G-243_mg/kg_in_Saline-Rat-Kidney-5d-up	16/304	1,06609E-05	0,0038166	0	0	3,617290649	41,41410147	C1QA;RPL32;RPS5;RPS6;RPL13A;RPL35A;RPSA;RPL10A;SEPP1;TOMM20;RPS17;RPS19;ARL6IP5;RPS20;RAPGEF5;RPL39
Tacrolimus-134_mg/kg_in_CMC-Rat-Heart-5d-up	16/306	1,15672E-05	0,003961024	0	0	3,593648227	40,85019985	RPL32;RPS8;RPS6;RPSA;RPS3A;RPL10A;SEPP1;EEF1B2;RPS17;CXCL11;RPS19;ARL2BP;RPS20;RAPGEF5;RPL39;TPT1
Dibromochloromethane-325_mg/kg_in_CMC-Rat-Kidney-1d-up	16/310	1,35885E-05	0,004459285	0	0	3,547278572	39,75182703	RPL32;RPS8;RPS5;RPS6;RPSA;RPS3A;RPL10A;SEPP1;EEF1B2;RPS17;RPS19;CLP1;ARL6IP5;RPS20;RPL39;TPT1
Idarubicin-0.625_mg/kg_in_Saline-Rat-Heart-1d-dn	16/310	1,35885E-05	0,004280913	0	0	3,547278572	39,75182703	RPL32;RPS8;RPS6;RPL35A;RPSA;RPS3A;SEPP1;TOMM20;EEF1B2;RPS17;RPS19;ARL2BP;FUNDC1;ARL6IP5;RPS20;TPT1
Diethylstilbestrol-280_mg/kg_in_Corn_Oil-Rat-Liver-5d-up	15/276	1,3803E-05	0,004181246	0	0	3,735245779	41,79973483	RPL32;RPS8;FEM1B;RPS5;RPS6;RPL35A;RPSA;RPS3A;RPL10A;EEF1B2;RPS17;RPS19;RPS20;RPL39;TPT1
Tobramycin-40_mg/kg_in_Saline-Rat-Kidney-28d-up	16/311	1,41405E-05	0,00412484	0	0	3,535872532	39,4832027	C1QA;RPL32;RPS8;FEM1B;RPS5;RPSA;RPS3A;RPL10A;EEF1B2;RPS17;CXCL11;RPS19;ARL6IP5;RPS20;RPL39;TPT1
Cytarabine-23_mg/kg_in_Saline-Rat-Liver-0.25d-up	16/313	1,53048E-05	0,004305029	0	0	3,513279097	38,95292799	RPL32;RPS8;RPS5;RPS6;RPL35A;RPSA;RPS3A;RPL10A;SEPP1;TOMM20;EEF1B2;RPS17;RPS19;RPS20;RPL39;TPT1
Estriol-313_mg/kg_in_CMC-Rat-Liver-5d-up	16/313	1,53048E-05	0,00415658	0	0	3,513279097	38,95292799	RPL32;RPS8;FEM1B;RPS6;RPSA;RPS3A;RPL10A;SEPP1;RPS17;RPS19;ARL2BP;ARL6IP5;RPS20;RPL39;GOLGA7;TPT1
Primaquine-45_mg/kg_in_CMC-Rat-Liver-5d-up	16/315	1,65537E-05	0,00434589	0	0	3,490972563	38,43177791	C1QA;RPL32;RPS8;RPS5;RPS6;RPSA;RPS3A;RPL10A;SEPP1;EEF1B2;RPS17;RPS19;ARL2BP;RPS20;RPL39;TPT1
Thioguanine-24_mg/kg_in_Corn_Oil-Rat-Spleen-5d-up	16/315	1,65537E-05	0,0042057	0	0	3,490972563	38,43177791	C1QA;RPL32;RPS8;RPS5;RPL35A;RPSA;RPS3A;RPL10A;SEPP1;EEF1B2;RPS17;CXCL11;RPS20;RPL39;GOLGA7;TPT1
Beta-Estradiol_3-Benzoate-2_mg/kg_in_Corn_Oil-Rat-Liver-3d-up	15/284	1,93136E-05	0,004753559	0	0	3,630027588	39,40286501	RPL32;RPS8;RPS5;RPS6;RPL13A;RPL35A;RPSA;RPL10A;SEPP1;TOMM20;RPS17;RPS19;RPS20;RPL39;TPT1
Betamethasone-79_mg/kg_in_Corn_Oil-Rat-Liver-3d-dn	16/319	1,93263E-05	0,004612546	0	0	3,447198613	37,41604273	C1QA;RPL32;RPS8;RPS5;RPS6;RPL35A;RPSA;RPS3A;RPL10A;SEPP1;EEF1B2;RPS17;RPS19;RPS20;RPL39;TPT1
Gabapentin-500_uM_in_DMSO-Rat-Primary_rat_hepatocytes-1d-dn	16/320	2,00809E-05	0,004651676	0	0	3,436426117	37,16750049	RPL32;RPS8;RPS5;RPS6;RPL13A;RPL35A;RPSA;RPS3A;RPL10A;SEPP1;RPS17;RPS19;ARL2BP;ARL6IP5;RPL39;GOLGA7
Diethylstilbestrol-2.8_mg/kg_in_Corn_Oil-Rat-Liver-5d-up	15/285	2,01239E-05	0,004528443	0	0	3,617290649	39,11595017	RPL32;RPS8;FEM1B;RPS5;RPS6;RPL35A;RPSA;RPS3A;RPL10A;EEF1B2;RPS17;RPS19;RPS20;RPL39;TPT1
Dexchlorpheniramine-3_mg/kg_in_Water-Rat-Heart-3d-up	16/321	2,08615E-05	0,00456403	0	0	3,42572074	36,92106728	RPL32;RPS8;RPS5;RPS6;RPL35A;RPSA;RPL10A;TOMM20;RPS17;RPS19;ARL2BP;RPS20;RAPGEF5;RPL39;GOLGA7;TPT1
Diethylstilbestrol-280_mg/kg_in_Corn_Oil-Rat-Spleen-5d-up	15/286	2,09641E-05	0,00446251	0	0	3,60464278	38,83174031	C1QA;RPL32;RPS8;RPS5;RPL35A;RPSA;RPS3A;RPL10A;SEPP1;EEF1B2;RPS17;RPS19;RPS20;GOLGA7;TPT1
Diazepam-710_mg/kg_in_CMC-Rat-Heart-3d-dn	15/288	2,2738E-05	0,004712758	0	0	3,579610538	38,27130298	RPL32;RPS6;RPSA;RPS3A;RPL10A;SEPP1;PDRG1;RPS17;RPS19;ARL2BP;COL5A3;FUNDC1;ARL6IP5;RPS20;RPL39
Hexachlorophene-8_mg/kg_in_Corn_Oil-Rat-Liver-1d-up	14/254	2,28619E-05	0,004616941	0	0	3,788186271	40,48069793	C1QA;RPL32;RPS8;RPS5;RPS6;RPL35A;RPSA;RPS3A;RPL10A;SEPP1;RPS17;RPS19;RPS20;RPL39
Fludrocortisone_Acetate-125_mg/kg_in_CMC-Rat-Liver-3d-dn	16/324	2,33675E-05	0,004601053	0	0	3,394001103	36,19419112	C1QA;RPL32;RPS8;RPS5;RPS6;RPL35A;RPSA;RPS3A;RPL10A;SEPP1;EEF1B2;RPS17;RPS19;CLP1;RPS20;TPT1
Cyclopropane_Carboxylic_Acid-375_mg/kg_in_Water-Rat-Liver-1d-dn	16/325	2,42602E-05	0,004660327	0	0	3,383558023	35,95596493	C1QA;RPL32;RPS8;RPS6;RPSA;RPS3A;RPL10A;SEPP1;EEF1B2;RPS17;RPS19;ARL2BP;CLP1;RPS20;RPL39;TPT1
Neostigmine_Bromide-11_mg/kg_in_Water-Rat-Liver-1d-up	16/325	2,42602E-05	0,004549367	0	0	3,383558023	35,95596493	RPL32;RPS8;RPS5;RPS6;RPL35A;RPSA;RPS3A;RPL10A;SEPP1;TOMM20;EEF1B2;RPS17;RPS19;RPS20;RPL39;TPT1
6-Methoxy-2-Naphthylacetic_Acid-360_mg/kg_in_Saline-Rat-Kidney-5d-up	15/291	2,5648E-05	0,004697759	0	0	3,542707337	37,45011901	C1QA;RPL32;RPS8;RPS5;RPL13A;RPSA;RPS3A;RPL10A;SEPP1;EEF1B2;RPS17;RPS19;RPS20;RPL39;TPT1
Norethindrone-75_mg/kg_in_Corn_Oil-Rat-Liver-3d-up	15/291	2,5648E-05	0,004590991	0	0	3,542707337	37,45011901	RPL32;RPS8;FEM1B;RPS5;RPS6;RPSA;RPS3A;RPL10A;SEPP1;EEF1B2;RPS17;RPS19;ARL6IP5;RPS20;RPL39
33'4'5-Tetrachlorosalicylanilide-50_mg/kg_in_Corn_Oil-Rat-Liver-1d-dn	15/293	2,77662E-05	0,0048597	0	0	3,518525034	36,91527945	RPL32;RPS8;RPS5;RPS6;RPS3A;RPL10A;SEPP1;EEF1B2;RPS17;RPS19;FUNDC1;ARL6IP5;RPS20;RPL39;GOLGA7
Doxorubicin-0.65_mg/kg_in_Saline-Rat-Spleen-3d-dn	15/294	2,88821E-05	0,00494511	0	0	3,506557262	36,65155362	C1QA;RPL32;RPS8;RPS5;RPS6;RPL13A;RPL35A;RPSA;RPS3A;EEF1B2;RPS17;ARL6IP5;RPS20;RPL39;TPT1
Meloxicam-33_mg/kg_in_Corn_Oil-Rat-Kidney-5d-up	14/261	3,08601E-05	0,005171373	0	0	3,686587405	38,2890626	C1QA;RPL32;RPS8;RPS5;RPS6;RPL35A;RPSA;RPS3A;RPL10A;RPS17;RPS19;RPS20;RPL39;TPT1
Cisplatin-1.17_mg/kg_in_Saline-Rat-Bone_marrow-3d-dn	15/297	3,24707E-05	0,005327907	0	0	3,471137492	35,87480022	RPL32;RPS8;RPS5;RPS6;NKG7;RPL13A;RPL35A;RPSA;RPS3A;RPL10A;EEF1B2;RPS17;RPS20;RPL39;TPT1
Gemfibrozil-100_mg/kg_in_Corn_Oil-Rat-Liver-0.25d-up	15/297	3,24707E-05	0,005219174	0	0	3,471137492	35,87480022	C1QA;RPL32;RPS8;RPS6;RPSA;RPS3A;RPL10A;SEPP1;EEF1B2;RPS17;CXCL11;RPS19;RPL39;GOLGA7;TPT1
Sisomicin-34_mg/kg_in_Saline-Rat-Kidney-3d-up	15/297	3,24707E-05	0,005114791	0	0	3,471137492	35,87480022	C1QA;RPL32;RPS8;RPS5;RPS6;RPL13A;RPL35A;RPSA;RPS3A;SEPP1;EEF1B2;RPS17;RPS19;ARL6IP5;RPS20
Lead(IV)_Acetate-600_mg/kg_in_Saline-Rat-Kidney-1d-up	17/371	3,37182E-05	0,005207152	0	0	3,14928539	32,42967813	RPL32;RPS8;RPS5;RPS6;RPL13A;RPL35A;RPSA;RPS3A;TOMM20;EEF1B2;RPS17;CXCL11;RPS19;ARL6IP5;RPS20;RPL39;TPT1
Benzocaine-1100_mg/kg_in_Corn_Oil-Rat-Liver-0.25d-up	15/298	3,37514E-05	0,005112036	0	0	3,459489379	35,62059534	RPL32;RPS8;RPS5;RPS6;RPL35A;RPSA;RPS3A;RPL10A;SEPP1;EEF1B2;RPS17;RPS19;CLP1;RPS20;RPL39
Lomustine-4.2_mg/kg_in_Corn_Oil-Rat-Spleen-1d-dn	15/298	3,37514E-05	0,005015582	0	0	3,459489379	35,62059534	C1QA;RPL32;RPS8;RPS5;RPS6;RPL13A;RPL35A;RPSA;RPS3A;RPL10A;TOMM20;EEF1B2;RPS17;RPS19;RPL39
Miconazole-920_mg/kg_in_Corn_Oil-Rat-Liver-5d-up	15/298	3,37514E-05	0,004922701	0	0	3,459489379	35,62059534	RPL32;RPS8;RPS5;RPS6;RPL13A;RPL35A;RPSA;RPS3A;RPL10A;EEF1B2;RPS17;RPS19;RPS20;RPL39;TPT1
N-Nitrosodiethylamine-1.67_mg/kg_in_Saline-Rat-Spleen-0.25d-up	15/298	3,37514E-05	0,004833197	0	0	3,459489379	35,62059534	C1QA;RPL32;RPS8;RPS5;RPL13A;RPL35A;RPSA;RPS3A;RPL10A;SEPP1;TOMM20;EEF1B2;RPS17;RPS19;RPS20
Norethindrone_Acetate-125_mg/kg_in_Corn_Oil-Rat-Liver-1d-up	15/298	3,37514E-05	0,00474689	0	0	3,459489379	35,62059534	RPL32;RPS8;FEM1B;RPS6;RPSA;RPS3A;RPL10A;SEPP1;EEF1B2;RPS17;RPS19;ARL6IP5;RPS20;RPL39;TPT1
44'-Methylenedianiline-81_mg/kg_in_Corn_Oil-Rat-Liver-5d-up	16/334	3,37594E-05	0,004664726	0	0	3,292384304	33,8992128	C1QA;CXADR;RPL32;RPS8;FEM1B;RPS6;RPSA;RPS3A;RPL10A;RPS17;RPS19;ARL2BP;RPS20;RPL39;GOLGA7;TPT1
Hexachloro-13-Butadiene-50_mg/kg_in_Corn_Oil-Rat-Kidney-3d-up	15/301	3,78644E-05	0,005141723	0	0	3,425009419	34,87173178	RPL32;RPS8;RPS5;RPS6;RPL35A;RPSA;RPS3A;RPL10A;TOMM20;EEF1B2;RPS17;RPS19;ARL6IP5;RPS20;TPT1
Verapamil-108_mg/kg_in_Water-Rat-Heart-3d-up	15/301	3,78644E-05	0,005054575	0	0	3,425009419	34,87173178	RPL32;RPS5;RPS6;RPL35A;RPSA;RPS3A;RPL10A;RPS17;RPS19;ARL2BP;FUNDC1;ARL6IP5;RPS20;RAPGEF5;RPL39
Chlorpropamide-269_mg/kg_in_CMC-Rat-Heart-5d-up	15/302	3,93302E-05	0,005162746	0	0	3,413668328	34,62660472	RPL32;RPS8;RPS5;RPS6;RPL35A;RPSA;RPS3A;TOMM20;EEF1B2;RPS17;RPS19;ARL2BP;FUNDC1;RPS20;TPT1
Mestranol-250_mg/kg_in_CMC-Rat-Liver-5d-up	15/302	3,93302E-05	0,005078111	0	0	3,413668328	34,62660472	RPL32;RPS8;FEM1B;RPS5;RPS6;RPSA;RPS3A;RPL10A;SEPP1;RPS17;RPS19;ARL2BP;RPS20;RPL39;TPT1
4-Nitrobenzoic_Acid-1470_mg/kg_in_CMC-Rat-Kidney-5d-up	15/303	4,08458E-05	0,005188728	0	0	3,402402096	34,38368093	RPL32;RPS8;RPS5;RPS6;RPSA;RPS3A;RPL10A;EEF1B2;RPS17;RPS19;CLP1;RPS20;RAPGEF5;RPL39;TPT1
Cadmium_Chloride-66_mg/kg_in_Corn_Oil-Rat-Kidney-3d-up	15/303	4,08458E-05	0,005106368	0	0	3,402402096	34,38368093	RPL32;RPS8;RPS5;RPS6;RPL35A;RPSA;RPS3A;RPL10A;TOMM20;RPS17;CXCL11;RPS19;ARL2BP;RAPGEF5;TPT1
Bacitracin-380_mg/kg_in_Corn_Oil-Rat-Kidney-3d-up	15/304	4,24125E-05	0,005219382	0	0	3,391209984	34,14293471	C1QA;RPL32;RPS8;RPS5;RPS6;RPL35A;RPSA;RPS3A;RPL10A;EEF1B2;RPS17;CXCL11;RPS19;RPS20;RPL39
Leflunomide-30_mg/kg_in_Corn_Oil-Rat-Liver-5d-dn	15/304	4,24125E-05	0,005139084	0	0	3,391209984	34,14293471	C1QA;RPL32;RPS8;RPS5;RPS6;RPL35A;RPSA;RPS3A;RPL10A;SEPP1;EEF1B2;RPS17;RPS19;RPS20;RPL39
Rapamycin-60_mg/kg_in_CMC-Rat-Heart-1d-up	15/306	4,57052E-05	0,005454154	0	0	3,369045213	33,66787407	RPL32;RPS8;RPS6;RPSA;RPS3A;RPL10A;EEF1B2;RPS17;RPS19;ARL2BP;FUNDC1;ARL6IP5;RPS20;RPL39;TPT1
Modafinil-17.5_mg/kg_in_Saline-Rat-Liver-0.25d-up	14/271	4,65171E-05	0,005468186	0	0	3,550550969	35,41920001	RPL32;RPS8;RPS5;RPS6;RPL35A;RPSA;RPS3A;RPL10A;SEPP1;EEF1B2;RPS17;RPS19;RPL39;TPT1
Carbon_Tetrachloride-1175_mg/kg_in_Corn_Oil-Rat-Liver-0.25d-up	15/307	4,74343E-05	0,005494008	0	0	3,358071124	33,43351011	RPL32;RPS8;RPS5;RPS6;RPL35A;RPSA;RPS3A;RPL10A;PDRG1;EEF1B2;RPS17;RPS19;RPS20;RPL39;TPT1
Gemcitabine-11_mg/kg_in_Saline-Rat-Bone_marrow-3d-up	16/344	4,80474E-05	0,005484363	0	0	3,196675458	31,78557722	C1QA;RPL32;RPS8;RPS5;RPS6;RPL35A;RPSA;RPS3A;RPL10A;SEPP1;EEF1B2;RPS17;RPS19;ARL2BP;COL5A3;RPL39
Gentamicin-40_mg/kg_in_Saline-Rat-Kidney-14d-up	15/309	5,10658E-05	0,00574563	0	0	3,336336036	32,97099377	C1QA;RPL32;RPS8;RPS5;RPS6;RPS3A;RPL10A;EEF1B2;RPS17;CXCL11;RPS19;RPS20;RPL39;GOLGA7;TPT1
Itraconazole-30_mg/kg_in_Corn_Oil-Rat-Liver-0.25d-up	15/309	5,10658E-05	0,005664705	0	0	3,336336036	32,97099377	RPL32;RPS8;RPS5;RPS6;RPSA;RPS3A;RPL10A;TOMM20;EEF1B2;RPS17;RPS19;ARL6IP5;RPS20;RPL39;TPT1
Pergolide-1.1_mg/kg_in_CMC-Rat-Liver-5d-up	15/309	5,10658E-05	0,005586029	0	0	3,336336036	32,97099377	C1QA;RPL32;RPS8;RPS5;RPS6;RPL35A;RPSA;RPS3A;RPL10A;SEPP1;RPS17;RPS19;RPS20;RPL39;TPT1
Lead_(II)_Acetate-600_mg/kg_in_Saline-Rat-Liver-0.25d-up	15/310	5,29714E-05	0,005715108	0	0	3,325573661	32,74279397	C1QA;RPL32;RPS8;RPS5;RPS6;RPL35A;RPSA;RPS3A;RPL10A;SEPP1;EEF1B2;RPS17;RPS19;RPS20;RPL39
Chlorzoxazone-763_mg/kg_in_CMC-Rat-Liver-1d-up	15/311	5,49392E-05	0,005847313	0	0	3,314880499	32,51660205	RPL32;RPS8;RPS5;RPS6;RPL13A;RPL35A;RPSA;RPS3A;RPL10A;EEF1B2;RPS17;RPS19;RPS20;RPL39;TPT1
Propylene_Glycol-2000_mg/kg_in_Water-Rat-Liver-1d-up	15/311	5,49392E-05	0,005769349	0	0	3,314880499	32,51660205	C1QA;RPL32;RPS8;RPS5;RPS6;RPL35A;RPSA;RPS3A;RPL10A;EEF1B2;RPS17;RPS19;RPS20;RPL39;TPT1
Enoxacin-750_mg/kg_in_Corn_Oil-Rat-Liver-5d-up	13/241	5,58337E-05	0,005786132	0	0	3,707347678	36,30655031	C1QA;RPL32;RPS8;RPS6;RPL35A;RPS3A;EEF1B2;RPS17;RPS19;ARL2BP;RPS20;RPL39;TPT1
Granisetron-175_mg/kg_in_Water-Rat-Liver-3d-up	14/276	5,66793E-05	0,005797484	0	0	3,486229394	34,08870424	RPL32;RPS8;RPS5;RPS6;RPL35A;RPSA;RPS3A;RPL10A;BMP3;RPS17;RPS19;RPS20;RPL39;TPT1
Diethylstilbestrol-2.8_mg/kg_in_Corn_Oil-Rat-Spleen-0.25d-up	16/349	5,70147E-05	0,005757022	0	0	3,150877815	30,79101326	C1QA;RPL32;RPS8;RPL13A;RPL35A;RPSA;RPS3A;RPL10A;SEPP1;EEF1B2;RPS17;RPS19;ARL6IP5;RPS20;RPL39;TPT1
Fenofibrate-215_mg/kg_in_Corn_Oil-Rat-Kidney-5d-up	16/349	5,70147E-05	0,005684148	0	0	3,150877815	30,79101326	RPL32;RPS8;RPS5;RPS6;RPL35A;RPSA;RPS3A;RPL10A;EEF1B2;RPS17;RPS19;ARL2BP;RPS20;RPL39;GOLGA7;TPT1
Gentamicin-267_mg/kg_in_Corn_Oil-Rat-Kidney-1d-up	16/350	5,89753E-05	0,005806122	0	0	3,141875307	30,59681083	C1QA;RPL32;RPS8;RPS5;RPS6;RPL35A;RPSA;RPS3A;SEPP1;EEF1B2;RPS17;RPS19;ARL6IP5;RPS20;RPL39;TPT1
Vinorelbine-1.5_mg/kg_in_Saline-Rat-Heart-1d-dn	16/351	6,09951E-05	0,005930833	0	0	3,132924095	30,40414128	RPL32;RPS8;RPS6;RPSA;RPS3A;RPL10A;SEPP1;EEF1B2;RPS17;RPS19;ARL2BP;FUNDC1;ARL6IP5;RPS20;RPL39;TPT1
Idarubicin-4_mg/kg_in_Saline-Rat-Kidney-14d-up	15/314	6,12329E-05	0,005881343	0	0	3,283209666	31,84984652	RPL32;RPS8;RPS6;RPSA;RPS3A;RPL10A;SEPP1;EEF1B2;RPS17;CXCL11;RPS19;RPS20;RAPGEF5;RPL39;GOLGA7
4-Chloro-2-Nitroaniline-1200_mg/kg_in_CMC-Rat-Kidney-5d-up	15/315	6,34669E-05	0,006022469	0	0	3,272786778	31,6314607	RPL32;RPS8;RPS5;RPSA;RPS3A;TOMM20;EEF1B2;RPS17;CXCL11;RPS19;ARL6IP5;RPS20;RAPGEF5;RPL39;TPT1
Sumatriptan-1100_mg/kg_in_Water-Rat-Brain-5d-up	15/315	6,34669E-05	0,005950773	0	0	3,272786778	31,6314607	RPL32;RPS8;RPS5;RPS6;RPL13A;RPL35A;RPSA;RPS3A;EEF1B2;RPS17;RPS19;ARL2BP;FUNDC1;ARL6IP5;TPT1
Venlafaxine-320_mg/kg_in_Water-Rat-Heart-3d-up	15/315	6,34669E-05	0,005880764	0	0	3,272786778	31,6314607	RPL32;RPS8;RPS6;RPSA;RPS3A;RPL10A;SEPP1;TOMM20;RPS17;RPS19;RPS20;RAPGEF5;RPL39;GOLGA7;TPT1
Cisplatin-63_uM_in_DMSO-Rat-Primary_rat_hepatocytes-1d-up	14/279	6,36656E-05	0,005830581	0	0	3,448743056	33,32129615	RPL32;RPS8;RPS5;RPS6;RPL35A;RPSA;PDRG1;EEF1B2;RPS17;CXCL11;ARL2BP;CLP1;RPS20;GOLGA7
4-Octylphenol-700_mg/kg_in_Corn_Oil-Rat-Kidney-3d-up	15/316	6,57719E-05	0,005954251	0	0	3,262429858	31,41497165	RPL32;RPS8;RPS5;RPS6;RPSA;RPS3A;RPL10A;EEF1B2;RPS17;CXCL11;RPS19;ARL6IP5;RPS20;RPL39;TPT1

Fenofibrate-250_uM_in_DMSO-Rat-Primary_rat_hepatocytes-1d-dn	15/316	6,57719E-05	0,005886589	0	0	3,262429858	31,41497165	RPL32;RPS8;RPS5;RPS6;RPL35A;RPSA;RPS3A;RPL10A;SEPP1;EEF1B2;RPS17;CXCL11;ARL2BP;ARL6IP5;RPS20
Ketoprofen-4_mg/kg_in_Corn_Oil-Rat-Kidney-5d-up	15/316	6,57719E-05	0,005820448	0	0	3,262429858	31,41497165	C1QA;RPL32;RPS8;RPS5;RPS6;RPL35A;RPSA;RPS3A;RPL10A;PDRG1;RPS17;CXCL11;RPS19;RPS20;RPL39
Modafinil-17.5_mg/kg_in_Saline-Rat-Liver-3d-up	14/281	6,87303E-05	0,006014668	0	0	3,424196842	32,82202209	RPL32;RPS8;RPS5;RPS6;RPL35A;RPSA;RPS3A;RPL10A;EEF1B2;RPS17;RPS19;RPS20;RPL39;TPT1
Cyclosporin_A-70_mg/kg_in_Corn_Oil-Rat-Kidney-3d-up	15/318	7,06032E-05	0,00611067	0	0	3,241911431	30,98759904	C1QA;RPL32;RPS8;RPS5;RPS6;RPL35A;RPSA;RPS3A;RPL10A;RPS17;RPS19;RPS20;RPL39;GOLGA7;TPT1
DActinomycin-0.06_mg/kg_in_Saline-Rat-Bone_marrow-3d-up	14/282	7,1392E-05	0,006111774	0	0	3,4120543	32,57599162	C1QA;RPL32;RPS5;NKG7;RPL35A;RPSA;RPS3A;RPL10A;SEPP1;EEF1B2;RPS17;RPS19;ARL6IP5;RPL39
Bithionol-333_mg/kg_in_Corn_Oil-Rat-Spleen-5d-up	15/319	7,31333E-05	0,006193529	0	0	3,231748699	30,7766738	C1QA;RPL32;RPS8;RPS5;NKG7;RPL35A;RPSA;RPS3A;RPL10A;EEF1B2;RPS17;CXCL11;RPS20;RPL39;TPT1
Catechol-40_mg/kg_in_Saline-Rat-Kidney-0.25d-dn	15/319	7,31333E-05	0,00612764	0	0	3,231748699	30,7766738	RPL32;RPS8;RPS6;RPL35A;RPSA;RPS3A;RPL10A;SEPP1;EEF1B2;RPS17;R3HCC1;ARL6IP5;RPS20;RPL39;TPT1
Penicillin_G-243_mg/kg_in_Saline-Rat-Kidney-3d-up	15/319	7,31333E-05	0,006063139	0	0	3,231748699	30,7766738	RPL32;RPS8;RPS5;RPS6;RPL13A;RPL35A;RPSA;RPL10A;SEPP1;TOMM20;EEF1B2;RPS17;RPS19;ARL6IP5;RPS20
Stavudine-1400_mg/kg_in_Water-Rat-Liver-3d-up	15/319	7,31333E-05	0,005999981	0	0	3,231748699	30,7766738	RPL32;RPS8;FEM1B;RPS5;RPS6;RPL35A;RPSA;RPS3A;RPL10A;EEF1B2;RPS17;RPS19;RPS20;RPL39;TPT1
Chloroform-600_mg/kg_in_Corn_Oil-Rat-Liver-5d-up	14/283	7,4143E-05	0,00602011	0	0	3,399997571	32,3323254	RPL32;RPS8;RPS5;RPS6;RPSA;RPS3A;RPL10A;EEF1B2;RPS17;RPS19;ARL2BP;RPS20;RPL39;TPT1
Naproxen-134_mg/kg_in_Corn_Oil-Rat-Liver-1d-up	14/283	7,4143E-05	0,00595868	0	0	3,399997571	32,3323254	C1QA;RPL32;RPS8;RPS5;RPS6;RPL13A;RPL35A;RPS3A;EEF1B2;RPS17;RPS19;RPS20;RPL39;TPT1
N-Nitrosodiethylamine-1.67_mg/kg_in_Saline-Rat-Spleen-3d-dn	13/248	7,47253E-05	0,005944816	0	0	3,6027048	34,23178908	C1QA;RPL32;RPS8;RPS5;RPS6;RPL13A;RPL35A;RPSA;RPS3A;RPL10A;RPS17;RPL39;TPT1
Mycophenolate_Mofetil-63_mg/kg_in_CMC-Rat-Bone_marrow-3d-up	15/321	7,84327E-05	0,006177363	0	0	3,211613193	30,36024345	C1QA;RPL32;RPS8;RPS6;NKG7;RPL35A;RPSA;RPS3A;EEF1B2;RPS17;RPS19;ARL2BP;ARL6IP5;RPS20;RPL39
Thioguanine-12_mg/kg_in_Corn_Oil-Rat-Spleen-0.25d-up	14/285	7,99236E-05	0,006232459	0	0	3,376137939	31,85196818	C1QA;RPL32;RPS8;RPS5;RPS6;RPL35A;RPSA;RPS3A;RPL10A;SEPP1;EEF1B2;RAPGEF5;RPL39;TPT1
Beta-Estradiol-150_mg/kg_in_Corn_Oil-Rat-Liver-5d-up	15/322	8,12062E-05	0,006270395	0	0	3,201639239	30,15469839	RPL32;RPS8;FEM1B;RPS5;RPS6;RPSA;RPS3A;RPL10A;EEF1B2;RPS17;RPS19;ARL6IP5;RPS20;RPL39;TPT1
Imatinib-150_mg/kg_in_Water-Rat-Liver-1d-dn	15/322	8,12062E-05	0,006209518	0	0	3,201639239	30,15469839	C1QA;RPL32;RPS8;RPS5;RPS6;RPL35A;RPSA;RPS3A;RPL10A;SEPP1;EEF1B2;RPS17;RPS19;RPS20;RPL39
6-Methoxy-2-Naphthylacetic_Acid-360_mg/kg_in_Saline-Rat-Kidney-3d-up	14/287	8,60928E-05	0,006519877	0	0	3,352610846	31,38071992	C1QA;RPL32;RPS5;RPS6;RPL13A;RPL35A;RPSA;RPS3A;RPL10A;SEPP1;RPS17;RPS19;RPS20;RPL39;TPT1
Chloroform-600_mg/kg_in_Corn_Oil-Rat-Liver-3d-up	14/287	8,60928E-05	0,006457783	0	0	3,352610846	31,38071992	RPL32;RPS8;FEM1B;RPS5;RPS6;RPSA;RPS3A;RPL10A;SEPP1;EEF1B2;RPS19;RPS20;RPL39;TPT1
Geraniol-1500_mg/kg_in_CMC-Rat-Liver-1d-up	14/287	8,60928E-05	0,006396861	0	0	3,352610846	31,38071992	RPL32;RPS8;RPS5;RPS6;RPL35A;RPSA;RPS3A;RPL10A;SEPP1;EEF1B2;RPS17;RPS19;RPS20;RPL39
Urethane-453_mg/kg_in_Water-Rat-Bone_marrow-5d-up	14/287	8,60928E-05	0,006337077	0	0	3,352610846	31,38071992	C1QA;RPL32;RPS8;RPS5;RPL13A;RPL35A;RPSA;RPS3A;RPL10A;EEF1B2;RPS17;RPS19;RPS20;TPT1
Tetracaine-24_mg/kg_in_Corn_Oil-Rat-Heart-3d-up	15/324	8,70116E-05	0,006345404	0	0	3,181876034	29,7488507	C1QA;RPL32;RPS8;RPS5;RPS6;RPL35A;RPSA;RPS3A;RPL10A;EEF1B2;RPS17;RPS19;ARL6IP5;RPS20;TPT1
Indomethacin-10_mg/kg_in_Corn_Oil-Rat-Liver-1d-up	14/288	8,933E-05	0,006454705	0	0	3,340969836	31,14844157	C1QA;RPL32;RPS8;RPS5;RPS6;RPSA;RPL10A;SEPP1;EEF1B2;RPS17;ARL2BP;RPS20;RPL39;TPT1
Lipopolysaccharide_E._Coli_O55:B5-1.25_mg/kg_in_Saline-Rat-Liver-0.25d-up	14/288	8,933E-05	0,006396026	0	0	3,340969836	31,14844157	RPL32;RPS8;RPS6;RPSA;RPS3A;RPL10A;EEF1B2;RPS17;CXCL11;RPS19;ARL2BP;RPS20;RPL39;TPT1
Lomustine-8.75_mg/kg_in_Corn_Oil-Rat-Spleen-1d-dn	15/325	9,0048E-05	0,006389352	0	0	3,172085646	29,54850975	C1QA;RPL32;RPS5;RPL13A;RPL35A;RPSA;RPS3A;RPL10A;SEPP1;TOMM20;EEF1B2;RPS17;RPS19;RPL39;TPT1
Roflumilast-17_mg/kg_in_CMC-Rat-Heart-3d-up	14/289	9,26725E-05	0,006516861	0	0	3,329409387	30,91835725	C1QA;RPS8;RPS6;RPSA;RPS3A;RPL10A;EEF1B2;RPS17;RPS19;ARL2BP;RPS20;RAPGEF5;RPL39;TPT1
Benzyl_Acetate-1868_mg/kg_in_CMC-Rat-Kidney-5d-up	15/326	9,31765E-05	0,006494323	0	0	3,162355322	29,34986564	RPL32;RPS8;RPS5;RPSA;RPS3A;RPL10A;TOMM20;EEF1B2;RPS17;CXCL11;RPS19;ARL6IP5;RPS20;RPL39;TPT1
Carbon_Tetrachloride-3178_mg/kg_in_Saline-Rat-Liver-1d-up	15/326	9,31765E-05	0,006437355	0	0	3,162355322	29,34986564	RPL32;RPS8;RPS5;RPS6;RPSA;RPS3A;RPL10A;PDRG1;EEF1B2;RPS17;RPS19;RPS20;RPL39;GOLGA7;TPT1
Guanethidine-132_mg/kg_in_CMC-Rat-Heart-3d-up	15/326	9,31765E-05	0,006381378	0	0	3,162355322	29,34986564	RPL32;RPS8;RPS6;RPL35A;RPSA;RPS3A;RPL10A;EEF1B2;RPS17;CXCL11;RPS19;ARL6IP5;RPS20;RAPGEF5;TPT1
Caffeine-93_mg/kg_in_Water-Rat-Brain-3d-up	13/254	9,51034E-05	0,00645719	0	0	3,517601537	32,57491198	RPL32;RPS8;RPS5;RPS6;RPL35A;RPSA;RPS3A;RPL10A;EEF1B2;RPS17;RPS19;ARL2BP;TPT1
Mitomycin_C-1.7_mg/kg_in_Saline-Rat-Bone_marrow-1d-dn	14/290	9,61232E-05	0,006470655	0	0	3,317928665	30,69044009	RPL32;RPS8;RPS5;RPS6;RPL35A;RPSA;RPS3A;RPL10A;EEF1B2;RPS17;RPS19;RPS20;RPL39;TPT1
Nimesulide-162_mg/kg_in_Corn_Oil-Rat-Liver-5d-up	14/290	9,61232E-05	0,006415819	0	0	3,317928665	30,69044009	RPL32;RPS8;RPS5;RPS6;RPL35A;RPSA;RPS3A;RPL10A;TOMM20;EEF1B2;RPS17;RPS19;RPS20;RPL39
Beta-Estradiol_3-Benzoate-25_mg/kg_in_Corn_Oil-Rat-Liver-5d-up	14/291	9,96852E-05	0,006597651	0	0	3,306526848	30,46466366	RPL32;RPS8;FEM1B;RPS5;RPS6;RPSA;RPS3A;RPL10A;EEF1B2;RPS19;ARL2BP;RPS20;RPL39;TPT1
Ethinylestradiol-10_mg/kg_in_Corn_Oil-Rat-Liver-0.25d-up	14/291	9,96852E-05	0,006542671	0	0	3,306526848	30,46466366	RPL32;RPS8;FEM1B;RPS5;RPS6;RPSA;RPS3A;RPL10A;SEPP1;EEF1B2;RPS17;RPS19;RPS20;RPL39
Sodium_Arsenite-10_mg/kg_in_Saline-Rat-Kidney-0.25d-up	14/291	9,96852E-05	0,006488599	0	0	3,306526848	30,46466366	RPL32;RPS8;RPS5;RPL13A;RPL35A;RPS3A;SEPP1;TOMM20;EEF1B2;RPS17;CXCL11;RPS20;RPL39;TPT1
Idarubicin-1.5_uM_in_DMSO-Rat-Primary_rat_hepatocytes-0.67d-dn	12/222	0,000105427	0,006806103	0	0	3,715055261	34,02058154	RPS17;CXADR;ARL2BP;RPS8;RPS6;RPS20;RPSA;RPS3A;RPL39;SLC16A3;TPT1;PDRG1
Benzyl_Acetate-1868_mg/kg_in_CMC-Rat-Kidney-3d-up	15/330	0,00010666	0,006829722	0	0	3,124023743	28,57189118	RPL32;RPS8;RPS5;RPS6;RPSA;RPS3A;RPL10A;SEPP1;EEF1B2;RPS17;CXCL11;RPS19;RPS20;RPL39;TPT1
Norethindrone-75_mg/kg_in_Corn_Oil-Rat-Liver-5d-up	15/330	0,00010666	0,006774644	0	0	3,124023743	28,57189118	RPL32;RPS8;FEM1B;RPS5;RPS6;RPL13A;RPL35A;RPSA;RPS3A;RPL10A;EEF1B2;RPS17;RPS19;RPS20;RPL39
Lovastatin-450_mg/kg_in_Corn_Oil-Rat-Liver-1d-dn	14/293	0,000107155	0,006751598	0	0	3,283956699	30,01942923	C1QA;RPL32;RPS8;RPS6;RPL35A;RPS3A;RPL10A;SEPP1;EEF1B2;RPS17;CLP1;RPS20;RPL39;TPT1
Oxcarbazepine-1600_mg/kg_in_CMC-Rat-Brain-3d-dn	16/369	0,000109348	0,00683513	0	0	2,98009853	27,18139915	C1QA;RPL32;RPS8;RPSA;RPS3A;RPL10A;SEPP1;TOMM20;EEF1B2;RPS17;RPS19;CLP1;FUNDC1;RPS20;RPL39;TPT1
Dicyclomine-645_mg/kg_in_Water-Rat-Heart-3d-dn	15/331	0,000110286	0,006839459	0	0	3,114585604	28,38145866	RPL32;RPS8;RPS5;RPS6;RPL35A;RPSA;RPS3A;EEF1B2;RPS17;RPS19;COL5A3;ARL6IP5;RPS20;RPL39;TPT1
Camptothecin-0.24_mg/kg_in_Saline-Rat-Bone_marrow-1d-up	14/294	0,000111068	0,006834168	0	0	3,272786778	29,79992036	C1QA;RPL32;FEM1B;RPS5;RPS6;NKG7;RPL35A;RPSA;RPL10A;RPS17;RPS19;RPS20;RPL39;TPT1
Trimethadione-535_mg/kg_in_Water-Rat-Kidney-3d-dn	12/224	0,000114746	0,007005757	0	0	3,681885125	33,40495615	RPS17;RPL32;RPS19;RPS8;RPS5;RPS6;RPL35A;RPS20;RPSA;RPS3A;RPL10A;RPL39
Dicumarol-5_mg/kg_in_Corn_Oil-Rat-Liver-0.25d-up	14/295	0,000115105	0,006973616	0	0	3,261692585	29,58245043	C1QA;RPL32;FEM1B;RPS6;RPL35A;RPSA;RPS3A;RPL10A;SEPP1;EEF1B2;RPS17;RPS19;RPL39;TPT1
Cyclosporin_A-350_mg/kg_in_Corn_Oil-Rat-Kidney-3d-up	15/333	0,000117861	0,007086042	0	0	3,095879385	28,00534512	C1QA;RPL32;RPS8;RPS5;RPS6;RPL35A;RPSA;RPS3A;RPL10A;EEF1B2;RPS17;RPS19;RPS20;RAPGEF5;RPL39
Hydrazine-45_mg/kg_in_Water-Rat-Liver-3d-up	15/333	0,000117861	0,007032359	0	0	3,095879385	28,00534512	C1QA;RPL32;RPS8;RPS5;RPS6;RPL35A;RPSA;RPS3A;RPL10A;SEPP1;RPS17;RPS19;RPS20;RPL39;TPT1
Irinotecan-5_mg/kg_in_Saline-Rat-Kidney-1d-dn	15/333	0,000117861	0,006979485	0	0	3,095879385	28,00534512	RPL32;RPS8;RPS5;RPS6;RPL13A;RPL35A;RPSA;RPS3A;EEF1B2;RPS17;RPS19;ARL2BP;RPS20;RPL39;TPT1
Pralidoxime_Chloride-48_mg/kg_in_Saline-Rat-Heart-1d-dn	15/333	0,000117861	0,006927399	0	0	3,095879385	28,00534512	RPL32;RPS8;RPS5;RPL35A;RPSA;RPS3A;SEPP1;TOMM20;EEF1B2;RPS17;RPS19;FUNDC1;ARL6IP5;RPS20;TPT1
Doxorubicin-3_mg/kg_in_Saline-Rat-Heart-5d-up	14/296	0,000119269	0,006958259	0	0	3,250673354	29,36699496	RPL32;RPS6;RPSA;RPS3A;RPL10A;SEPP1;EEF1B2;RPS17;CXCL11;RPS19;FUNDC1;RPS20;RPL39;TPT1
Lead_(II)_Acetate-600_mg/kg_in_Saline-Rat-Spleen-5d-up	14/296	0,000119269	0,006907095	0	0	3,250673354	29,36699496	C1QA;RPL32;RPS8;RPS5;RPS6;RPSA;RPS3A;SEPP1;EEF1B2;RPS17;RPS19;RPS20;RAPGEF5;RPL39
Leflunomide-30_mg/kg_in_Corn_Oil-Rat-Spleen-0.25d-up	14/296	0,000119269	0,006856678	0	0	3,250673354	29,36699496	C1QA;RPL32;RPS8;RPSA;RPS3A;RPL10A;SEPP1;EEF1B2;CXCL11;CLP1;FUNDC1;RPS20;RPL39;TPT1
Gatifloxacin-770_mg/kg_in_Corn_Oil-Rat-Heart-3d-dn	14/297	0,000123563	0,007052058	0	0	3,239728326	29,15352983	RPL32;RPS8;RPS6;RPSA;RPS3A;RPL10A;SEPP1;EEF1B2;RPS17;RPS19;ARL2BP;FUNDC1;RPL39;TPT1
Betahistine-1500_mg/kg_in_Water-Rat-Kidney-5d-up	15/335	0,000125885	0,007132886	0	0	3,077396523	27,63545442	C1QA;RPL32;RPS6;RPL35A;RPSA;RPS3A;RPL10A;PDRG1;EEF1B2;RPS17;RPS19;ARL2BP;RPS20;RAPGEF5;TPT1
Piclamilast-17_mg/kg_in_CMC-Rat-Heart-5d-up	15/335	0,000125885	0,007081936	0	0	3,077396523	27,63545442	RPL32;RPS8;RPL35A;RPSA;RPS3A;RPL10A;TOMM20;EEF1B2;RPS17;ARL2BP;ARL6IP5;RPS20;RAPGEF5;RPL39;TPT1
Bezafibrate-617_mg/kg_in_Corn_Oil-Rat-Skeletal_muscle-3d-dn	14/298	0,000127991	0,007149318	0	0	3,228856754	28,94203129	RPL32;FEM1B;RPS6;RPSA;RPS3A;RPL10A;EEF1B2;RPS17;RPS19;COL5A3;ARL6IP5;RPL39;SLC16A3;TPT1
Catechol-195_mg/kg_in_Saline-Rat-Liver-5d-dn	14/298	0,000127991	0,007098971	0	0	3,228856754	28,94203129	C1QA;RPL32;RPS8;RPS5;RPS6;RPS3A;SEPP1;EEF1B2;RPS17;RPS19;ARL6IP5;RPS20;RPL39;TPT1
Hydrazine-45_mg/kg_in_Water-Rat-Liver-1d-up	14/298	0,000127991	0,007049328	0	0	3,228856754	28,94203129	RPL32;RPS8;FEM1B;RPS5;RPS6;RPL35A;RPSA;RPS3A;RPL10A;SEPP1;RPS17;RPS19;RPS20;RPL39
Allyl_Alcohol-25_mg/kg_in_Saline-Rat-Kidney-3d-up	14/299	0,000132555	0,007250012	0	0	3,218057902	28,73247593	C1QA;RPL32;RPS8;RPS5;RPL13A;RPL35A;RPSA;RPS3A;SEPP1;TOMM20;RPS17;RPS20;RPL39;TPT1
Eucalyptol-930_mg/kg_in_Corn_Oil-Rat-Kidney-5d-up	15/337	0,000134381	0,007299207	0	0	3,059133042	27,27165471	RPL32;RPS8;RPS5;RPS6;RPL13A;RPL35A;RPSA;RPS3A;EEF1B2;RPS17;RPS19;ARL2BP;ARL6IP5;RPS20;RPL39
Monocrotaline-333_uM_in_DMSO-Rat-Primary_rat_hepatocytes-0.67d-up	15/337	0,000134381	0,007249213	0	0	3,059133042	27,27165471	RPL32;RPS8;RPS5;RPL35A;RPSA;RPS3A;TOMM20;PDRG1;EEF1B2;RPS17;RPS19;ARL2BP;RPS20;RPL39;GOLGA7
Papaverine-69_mg/kg_in_CMC-Rat-Heart-1d-dn	15/337	0,000134381	0,007199898	0	0	3,059133042	27,27165471	RPL32;RPS8;RPS6;RPL35A;RPSA;RPS3A;RPL10A;EEF1B2;RPS17;RPS19;ARL2BP;ARL6IP5;RPS20;RPL39;TPT1
Tenidap-75_mg/kg_in_Corn_Oil-Rat-Kidney-0.25d-up	15/337	0,000134381	0,007151251	0	0	3,059133042	27,27165471	RPL32;RPS8;RPS5;RPS6;RPL13A;RPL35A;RPSA;RPS3A;RPL10A;RPS17;RPS19;ARL6IP5;RPS20;RPL39;TPT1
Trimethadione-535_mg/kg_in_Water-Rat-Heart-1d-dn	15/337	0,000134381	0,007103256	0	0	3,059133042	27,27165471	RPL32;RPS8;RPS5;RPS6;RPL35A;RPSA;RPS3A;RPL10A;TOMM20;EEF1B2;RPS17;RPS19;FUNDC1;RPS20;RPL39
2-Amino-4-Nitrophenol-625_mg/kg_in_CMC-Rat-Kidney-1d-up	14/300	0,000137259	0,007207035	0	0	3,207331042	28,52484071	RPL32;RPS8;RPS6;RPSA;RPS3A;RPL10A;EEF1B2;RPS17;CXCL11;RPS19;ARL6IP5;RPS20;RPL39;TPT1
Cytarabine-487_mg/kg_in_Saline-Rat-Liver-0.25d-up	14/300	0,000137259	0,007159306	0	0	3,207331042	28,52484071	C1QA;RPL32;RPS8;RPS5;RPS6;RPL35A;RPSA;RPS3A;RPL10A;TOMM20;EEF1B2;RPS17;RPL39;TPT1
Enrofloxacin-2000_mg/kg_in_CMC-Rat-Kidney-5d-up	14/300	0,000137259	0,007112205	0	0	3,207331042	28,52484071	RPL32;RPS8;RPS5;RPS6;RPL13A;RPL35A;RPSA;RPS3A;RPL10A;EEF1B2;RPS17;RPS19;RPL39;TPT1
Geraniol-1500_mg/kg_in_CMC-Rat-Liver-3d-up	14/300	0,000137259	0,00706572	0	0	3,207331042	28,52484071	RPL32;RPS8;RPS5;RPS6;RPL13A;RPL35A;RPSA;RPS3A;RPL10A;EEF1B2;RPS17;RPS19;RPS20;RPL39
Methotrexate-0.3_mg/kg_in_Corn_Oil-Rat-Liver-5d-up	14/300	0,000137259	0,007019839	0	0	3,207331042	28,52484071	RPL32;RPS8;FEM1B;RPS5;RPS6;RPSA;RPS3A;RPL10A;SEPP1;EEF1B2;RPS17;RPS19;RPS20;RPL39
Modafinil-325_mg/kg_in_Saline-Rat-Liver-3d-up	14/300	0,000137259	0,00697455	0	0	3,207331042	28,52484071	RPL32;RPS8;RPS5;RPS6;RPL13A;RPL35A;RPSA;RPS3A;RPL10A;EEF1B2;RPS17;RPS19;RPS20;RPL39
Neomycin-877_mg/kg_in_Corn_Oil-Rat-Kidney-1d-dn	13/264	0,000139796	0,007057895	0	0	3,384359054	30,03729643	RPL32;RPS8;RPL13A;RPL35A;RPSA;RPS3A;SEPP1;TOMM20;EEF1B2;RPS17;RPS19;ARL2BP;TPT1
Carbon_Tetrachloride-400_mg/kg_in_Corn_Oil-Rat-Bone_marrow-3d-up	14/301	0,000142108	0,007128933	0	0	3,196675458	28,31910291	C1QA;RPL32;RPS8;FEM1B;RPS5;RPS6;RPL13A;RPL35A;RPS3A;RPS17;FUNDC1;RPS20;RPL39;TPT1
Mycophenolate_Mofetil-63_mg/kg_in_CMC-Rat-Bone_marrow-1d-up	14/301	0,000142108	0,007083813	0	0	3,196675458	28,31910291	C1QA;RPL32;RPS6;NKG7;RPL35A;RPSA;SEPP1;EEF1B2;RPS19;FUNDC1;ARL6IP5;RPS20;RPL39;TPT1
Catechol-40_mg/kg_in_Saline-Rat-Spleen-0.25d-up	15/339	0,000143372	0,007101854	0	0	3,041085059	26,91381759	C1QA;RPL32;RPS5;RPS6;NKG7;RPL13A;RPL35A;RPSA;RPL10A;SEPP1;TOMM20;RPS17;RPS19;ARL6IP5;RPL39
Stavudine-1400_mg/kg_in_Water-Rat-Liver-1d-up	14/302	0,000147104	0,007241201	0	0	3,186090439	28,11524018	RPL32;RPS8;RPS5;RPS6;RPL35A;RPSA;RPS3A;RPL10A;EEF1B2;RPS17;RPS19;RPS20;RPL39;TPT1
Ibuprofen-263_mg/kg_in_Corn_Oil-Rat-Kidney-5d-up	15/340	0,00014806	0,007242976	0	0	3,032140691	26,73709582	C1QA;RPL32;RPS8;RPS5;RPS6;RPL35A;RPSA;RPS3A;RPL10A;EEF1B2;RPS17;CXCL11;RPS19;RPS20;RPL39
Vecuronium_Bromide-0.05_mg/kg_in_Saline-Rat-Liver-1d-dn	15/340	0,00014806	0,007198266	0	0	3,032140691	26,73709582	RPL32;RPS8;RPS5;RPS6;RPL35A;RPSA;RPS3A;RPL10A;SEPP1;EEF1B2;RPS17;RPS19;RPS20;RPL39;TPT1
NN-Dimethylformamide-1400_mg/kg_in_Saline-Rat-Bone_marrow-3d-up	16/379	0,000148633	0,007181795	0	0	2,901467961	25,5736292	C1QA;RPL32;RPS8;FEM1B;RPS5;RPS6;NKG7;RPSA;RPS3A;RPL10A;SEPP1;RPS17;RPS19;RPS20;RPL39;GOLGA7
Zidovudine-14800_uM_in_DMSO-Rat-Primary_rat_hepatocytes-1d-up	13/266	0,000150632	0,007234013	0	0	3,358912746	29,56068367	RPL32;RPS8;FEM1B;RPS5;RPL35A;RPSA;RPS3A;PDRG1;EEF1B2;RPS17;ARL2BP;CLP1;RPS20
Acyclovir-980_mg/kg_in_Saline-Rat-Spleen-3d-dn	14/303	0,000152252	0,007267478	0	0	3,175575289	27,91323046	RPL32;RPS8;RPS5;RPL13A;RPL35A;RPSA;RPS3A;RPL10A;EEF1B2;RPS17;ARL2BP;ARL6IP5;RPS20;TPT1
Interleukin_6-0.125_mg/kg_in_RSA/Saline-Rat-Liver-1d-up	14/303	0,000152252	0,007223698	0	0	3,175575289	27,91323046	C1QA;RPL32;RPS8;RPS6;RPL35A;RPSA;RPS3A;SEPP1;EEF1B2;RPS17;RPS19;RPS20;RAPGEF5;RPL39
Nisoldipine-1125_mg/kg_in_Water-Rat-Liver-3d-dn	13/267	0,000156316	0,007372134	0	0	3,346332548	29,32601943	RPL32;RPS8;RPS5;RPS6;RPL35A;RPSA;RPS3A;RPL10A;SEPP1;EEF1B2;RPS17;RPS20;TPT1
Torsemide-3_mg/kg_in_Corn_Oil-Rat-Liver-1d-up	13/267	0,000156316	0,007328252	0	0	3,346332548	29,32601943	C1QA;RPL32;RPS8;RPS5;RPS6;RPL35A;RPS3A;SEPP1;RPS19;RPS20;RPL39;GOLGA7;TPT1
Eucalyptol-930_mg/kg_in_Corn_Oil-Rat-Kidney-1d-up	16/381	0,000157821	0,007355014	0	0	2,886237158	25,26626376	RPL32;RPS8;RPS6;RPL13A;RPL35A;RPSA;RPS3A;SEPP1;TOMM20;EEF1B2;RPS17;RPS19;ARL6IP5;RPS20;RPL39;TPT1
Acetazolamide-250_mg/kg_in_Corn_Oil-Rat-Kidney-5d-up	14/305	0,000163016	0,007552427	0	0	3,154751845	27,51468353	RPL32;RPS8;RPS5;RPS6;RPL35A;RPSA;RPS3A;EEF1B2;RPS17;RPS19;ARL2BP;RPS20;RAPGEF5;RPL39
Albendazole-62_mg/kg_in_Corn_Oil-Rat-Liver-0.25d-up	14/305	0,000163016	0,007508261	0	0	3,154751845	27,51468353	C1QA;RPL32;RPS5;RPS6;RPS3A;RPL10A;SEPP1;RPS17;CXCL11;RPS19;CLP1;RPS20;GOLGA7;TPT1
Amiodarone-147_mg/kg_in_CMC-Rat-Liver-5d-dn	14/305	0,000163016	0,007464608	0	0	3,154751845	27,51468353	RPL32;RPS8;RPS5;RPS6;RPL35A;RPS3A;SEPP1;EEF1B2;RPS17;RPS19;CLP1;RPS20;RPL39;TPT1
Clomipramine-7_uM_in_DMSO-Rat-Primary_rat_hepatocytes-1d-dn	14/305	0,000163016	0,00742146	0	0	3,154751845	27,51468353	RPL32;RPS6;RPSA;RPS3A;RPL10A;SEPP1;RPS17;CXCL11;RPS19;ARL6IP5;RPS20;RPL39;GOLGA7;TPT1
Clonidine-6_mg/kg_in_Water-Rat-Heart-5d-up	14/305	0,000163016	0,007378808	0	0	3,154751845	27,51468353	C1QA;RPL32;RPS5;RPS6;RPL35A;RPSA;RPS3A;RPL10A;RPS17;RPS19;RPS20;RAPGEF5;RPL39;TPT1
Lomustine-8.75_mg/kg_in_Corn_Oil-Rat-Bone_marrow-3d-up	14/305	0,000163016	0,007336643	0	0	3,154751845	27,51468353	C1QA;RPL32;RPS8;RPS5;RPS6;RPL35A;RPS3A;RPL10A;DUSP14;EEF1B2;RPS17;RPS20;RPL39;TPT1
Aspirin-375_mg/kg_in_Corn_Oil-Rat-Kidney-5d-up	15/344	0,000168171	0,007525658	0	0	2,996883241	26,04449896	RPL32;RPS8;RPS5;RPS6;RPL13A;RPL35A;RPSA;RPS3A;RPL10A;TOMM20;EEF1B2;RPS17;RPS19;RPS20;RPL39
Diclofenac-3.5_mg/kg_in_Corn_Oil-Rat-Liver-5d-dn	13/269	0,00016824	0,007486215	0	0	3,321452752	28,86381533	C1QA;RPL32;RPS8;RPS5;RPSA;RPS3A;RPL10A;EEF1B2;RPS17;RPS19;RPS20;RPL39;TPT1

Mitomycin_C-0.5_mg/kg_in_Saline-Rat-Bone_marrow-3d-dn	13/269	0,00016824	0,007444157	0	0	3,321452752	28,86381533	RPL32;RPS8;RPS6;RPSA;RPS3A;EEF1B2;RPS19;ARL2BP;FUNDC1;ARL6IP5;RPS20;RPL39;TPT1
Bromodichloromethane-430_mg/kg_in_CMC-Rat-Kidney-5d-up	14/306	0,00016864	0,007420174	0	0	3,144442198	27,31810385	RPL32;RPS8;RPS5;RPS6;RPSA;RPS3A;RPL10A;EEF1B2;RPS17;RPS19;ARL6IP5;RPS20;RPL39;TPT1
Raloxifene-5_mg/kg_in_Corn_Oil-Rat-Liver-3d-up	14/306	0,00016864	0,007378951	0	0	3,144442198	27,31810385	C1QA;CXADR;RPL32;RPS8;RPS5;RPS6;RPL13A;RPL35A;TOMM20;EEF1B2;RPS19;RPS20;RPL39;TPT1
Catechol-40_mg/kg_in_Saline-Rat-Bone_marrow-1d-dn	14/307	0,000174432	0,007590191	0	0	3,134199716	27,12329221	RPL32;RPS8;RPS5;RPS6;RPSA;RPS3A;RPL10A;EEF1B2;RPS17;RPS19;ARL2BP;RPS20;RPL39;TPT1
Ethanol-3000_mg/kg_in_Saline-Rat-Kidney-1d-up	14/307	0,000174432	0,007548486	0	0	3,134199716	27,12329221	RPL32;RPS5;RPS6;RPL13A;RPL35A;RPSA;SEPP1;EEF1B2;RPS17;RPS19;ARL6IP5;RPS20;RPL39;TPT1
Mitomycin_C-1.7_mg/kg_in_Saline-Rat-Liver-3d-up	14/307	0,000174432	0,007507238	0	0	3,134199716	27,12329221	RPL32;RPS8;RPS5;RPS6;RPL35A;RPSA;RPS3A;RPL10A;EEF1B2;RPS17;RPS19;RPS20;RAPGEF5;RPL39
Ramipril-620_uM_in_DMSO-Rat-Primary_rat_hepatocytes-0.67d-up	14/307	0,000174432	0,007466437	0	0	3,134199716	27,12329221	RPL32;RPS8;RPS5;RPL35A;RPSA;RPS3A;RPL10A;PDRG1;EEF1B2;RPS17;RPS19;ARL2BP;ARL6IP5;RPS20
Terazosin-657_mg/kg_in_Water-Rat-Heart-3d-up	14/307	0,000174432	0,007426078	0	0	3,134199716	27,12329221	C1QA;RPL32;RPS8;RPS5;RPS6;RPL35A;RPSA;RPS3A;RPL10A;RPS17;RPS19;ARL2BP;FUNDC1;ARL6IP5
Propylene_Glycol-2000_mg/kg_in_Water-Rat-Kidney-3d-dn	11/200	0,000175024	0,007411233	0	0	3,780068729	32,69981427	RPS17;RPL32;RPS19;RPS8;RPS6;RPL35A;RPS20;RPSA;RPS3A;RPL10A;RPL39
Cisplatin-2_mg/kg_in_Saline-Rat-Liver-3d-up	14/308	0,000180394	0,00759778	0	0	3,124023743	26,93022816	C1QA;RPL32;RPS8;RPS5;RPS6;RPL35A;RPSA;RPS3A;RPL10A;EEF1B2;RPS17;RPS19;RPS20;RPL39
Tranilast-300_mg/kg_in_CMC-Rat-Heart-3d-dn	14/308	0,000180394	0,007557366	0	0	3,124023743	26,93022816	C1QA;RPL32;RPS8;RPS6;RPSA;RPS3A;RPL10A;SEPP1;RPS17;RPS19;ARL2BP;ARL6IP5;RPS20;RPL39
44'-Methylenedianiline-81_mg/kg_in_Corn_Oil-Rat-Liver-1d-up	15/347	0,000184772	0,007699805	0	0	2,970973588	25,53964379	C1QA;CXADR;RPL32;RPS8;RPS5;RPS6;RPSA;RPS3A;RPL10A;RPS17;RPS19;RPS20;RPL39;GOLGA7;TPT1
2-Amino-4-Nitrophenol-625_mg/kg_in_CMC-Rat-Kidney-3d-up	14/309	0,000186532	0,007732236	0	0	3,113913633	26,73889151	C1QA;RPL32;RPS8;RPS6;RPSA;RPS3A;RPL10A;SEPP1;EEF1B2;RPS17;RPS19;RPS20;RPL39;TPT1
Cholecalciferol-8_mg/kg_in_CMC-Rat-Liver-5d-up	14/309	0,000186532	0,007691753	0	0	3,113913633	26,73889151	C1QA;RPL32;RPS8;RPS5;RPS6;RPL35A;RPSA;RPS3A;RPL10A;EEF1B2;RPS17;RPS19;RPS20;TPT1
Estriol-313_mg/kg_in_CMC-Rat-Liver-3d-up	14/309	0,000186532	0,007651692	0	0	3,113913633	26,73889151	RPL32;RPS8;FEM1B;RPS5;RPS6;RPSA;RPS3A;RPL10A;EEF1B2;RPS17;RPS19;RPS20;RPL39;TPT1
Mevastatin-1200_mg/kg_in_CMC-Rat-Liver-1d-up	14/309	0,000186532	0,007612046	0	0	3,113913633	26,73889151	RPL32;RPS8;RPS5;RPS6;RPL35A;RPSA;RPS3A;RPL10A;SEPP1;EEF1B2;RPS17;CXCL11;RPS20;TPT1
Phenobarbital-25_mg/kg_in_Water-Rat-Liver-1d-up	14/309	0,000186532	0,007572809	0	0	3,113913633	26,73889151	RPL32;RPS8;RPS5;RPS6;RPL35A;RPSA;RPS3A;RPL10A;SEPP1;EEF1B2;RPS17;RPS19;RPS20;RPL39
Timolol-900_mg/kg_in_Water-Rat-Heart-5d-up	14/309	0,000186532	0,007533974	0	0	3,113913633	26,73889151	RPL32;RPS8;RPS5;RPS6;RPSA;RPS3A;RPL10A;SEPP1;EEF1B2;RPS17;ARL2BP;FUNDC1;ARL6IP5;RPL39
Nabumetone-2500_mg/kg_in_Corn_Oil-Rat-Kidney-3d-up	15/348	0,000190612	0,007659508	0	0	2,962436308	25,374062	C1QA;RPL32;RPS8;RPS5;RPS6;RPL35A;RPSA;RPS3A;RPL10A;PDRG1;EEF1B2;RPS17;RPS19;RPS20;RPL39
Chlortetracycline-1500_mg/kg_in_CMC-Rat-Liver-1d-up	14/310	0,000192849	0,007710041	0	0	3,103868751	26,54926237	RPL32;RPS8;RPS5;RPS6;RPSA;RPS3A;RPL10A;EEF1B2;RPS17;RPS19;ARL2BP;RPS20;RPL39;TPT1
Digitoxin-1_mg/kg_in_Corn_Oil-Rat-Heart-0.25d-dn	14/310	0,000192849	0,007671102	0	0	3,103868751	26,54926237	RPL32;RPS8;RPS6;RPL35A;RPSA;TOMM20;PDRG1;EEF1B2;RPS17;RPS19;ARL2BP;FUNDC1;RPS20;RPL39
Cyclosporin_A-350_mg/kg_in_Corn_Oil-Rat-Kidney-1d-up	16/388	0,000193994	0,007677874	0	0	2,83416587	24,22555211	C1QA;RPL32;RPS8;RPS5;RPS6;RPL35A;RPSA;RPS3A;RPL10A;EEF1B2;RPS17;CXCL11;RPS19;RPS20;RPL39;TPT1
Abamectin-10_mg/kg_in_Water-Rat-Liver-1d-up	14/311	0,00019935	0,007850393	0	0	3,093888465	26,36132117	C1QA;RPL32;RPS8;RPS5;RPS6;RPL35A;RPSA;RPS3A;EEF1B2;RPS17;RPS19;RPS20;RPL39;TPT1
Hydrazine-45_mg/kg_in_Water-Rat-Liver-5d-up	14/311	0,00019935	0,007811336	0	0	3,093888465	26,36132117	C1QA;RPL32;RPS8;RPS5;RPS6;RPSA;RPS3A;RPL10A;SEPP1;RPS17;RPS19;RPS20;RAPGEF5;RPL39
Risperidone-22_mg/kg_in_CMC-Rat-Heart-3d-up	14/311	0,00019935	0,007772666	0	0	3,093888465	26,36132117	RPL32;RPS8;RPS5;RPS3A;SEPP1;EEF1B2;RPS17;RPS19;ARL2BP;FUNDC1;RPS20;RAPGEF5;GOLGA7;TPT1
Vinblastine-0.3_mg/kg_in_Saline-Rat-Liver-5d-up	14/311	0,00019935	0,007734377	0	0	3,093888465	26,36132117	C1QA;RPL32;RPS8;RPS5;RPS6;RPSA;RPS3A;RPL10A;EEF1B2;RPS17;RPS19;RPS20;RPL39;TPT1
Fenofibrate-430_mg/kg_in_Corn_Oil-Rat-Skeletal_muscle-5d-up	13/274	0,000201526	0,007780498	0	0	3,260842301	27,74843238	C1QA;RPL32;RPS8;RPS6;RPSA;RPS3A;RPL10A;EEF1B2;RPS17;RPS19;RPS20;RPL39;TPT1
Lead(IV)_Acetate-200_mg/kg_in_Saline-Rat-Liver-3d-up	13/274	0,000201526	0,007742544	0	0	3,260842301	27,74843238	RPL32;RPS8;RPS5;RPS6;RPL35A;RPSA;RPL10A;SEPP1;EEF1B2;RPS17;RPS20;RPL39;TPT1
1-Naphthyl_Isothiocyanate-60_mg/kg_in_Corn_Oil-Rat-Liver-3d-up	14/312	0,000206039	0,00787748	0	0	3,083972156	26,17504858	C1QA;RPS8;FEM1B;RPS5;RPS6;RPSA;RPS3A;RPL10A;SEPP1;EEF1B2;RPS17;RPS19;RPS20;RPL39
Digitonin-75_mg/kg_in_Corn_Oil-Rat-Heart-3d-up	15/351	0,000209105	0,007956081	0	0	2,937116339	24,88523336	RPL32;RPS5;RPS6;RPL35A;RPSA;RPS3A;RPL10A;SEPP1;TOMM20;EEF1B2;RPS17;RPS19;ARL6IP5;RPS20;RPL39
Dimenhydrinate-165_mg/kg_in_CMC-Rat-Liver-3d-up	14/313	0,00021292	0,008062309	0	0	3,07411921	25,99042555	RPL32;RPS8;RPS5;RPS6;RPL13A;RPL35A;RPSA;RPS3A;RPL10A;EEF1B2;RPS17;RPS19;RPS20;RPL39
Procarbazine-54_mg/kg_in_Water-Rat-Bone_marrow-5d-up	14/313	0,00021292	0,008023734	0	0	3,07411921	25,99042555	C1QA;RPL32;RPS8;RPS5;RPL13A;RPL35A;RPSA;RPS3A;SEPP1;EEF1B2;RPS17;ARL2BP;RPS20;TPT1
Sulindac-23_mg/kg_in_Corn_Oil-Rat-Liver-3d-dn	14/313	0,00021292	0,007985525	0	0	3,07411921	25,99042555	C1QA;RPL32;RPS8;RPS6;RPL35A;RPSA;RPS3A;RPL10A;SEPP1;RPS17;RPS19;RPS20;RPL39;TPT1
Rofecoxib-775_mg/kg_in_Corn_Oil-Rat-Kidney-1d-dn	13/276	0,000216342	0,00807539	0	0	3,237213009	27,31771197	RPL32;RPS8;RPS6;RPL35A;RPSA;RPS3A;SEPP1;TOMM20;EEF1B2;RPS17;ARL6IP5;RPL39;TPT1
Cyclophosphamide-25_mg/kg_in_Water-Rat-Heart-3d-up	14/314	0,000219999	0,008173173	0	0	3,064329021	25,80743334	RPL32;RPS8;RPS6;RPL35A;RPSA;RPS3A;RPL10A;EEF1B2;RPS17;RPS19;ARL2BP;ARL6IP5;RPS20;RAPGEF5
Etoposide-188_mg/kg_in_Corn_Oil-Rat-Bone_marrow-3d-up	14/314	0,000219999	0,008134802	0	0	3,064329021	25,80743334	C1QA;RPL32;RPS8;RPS5;NKG7;RPL35A;RPSA;RPS3A;RPL10A;SEPP1;EEF1B2;RPS17;RPS20;RPL39
Methapyrilene-100_mg/kg_in_CMC-Rat-Liver-1d-up	14/314	0,000219999	0,008096789	0	0	3,064329021	25,80743334	RPL32;RPS8;RPS5;RPS6;RPSA;RPS3A;RPL10A;SEPP1;EEF1B2;RPS17;RPS19;RPS20;RPL39;TPT1
Aspirin-35_mg/kg_in_Corn_Oil-Rat-Kidney-1d-dn	13/277	0,000224093	0,008209104	0	0	3,225526319	27,10554579	RPL32;RPS8;RPL35A;RPSA;RPS3A;EEF1B2;RPS17;ARL2BP;R3HCC1;ARL6IP5;RPS20;RPL39;TPT1
L-Buthionine_Sulfoximine-100000_uM_in_DMSO-Rat-Primary_rat_hepatocytes-1d-up	13/277	0,000224093	0,008171098	0	0	3,225526319	27,10554579	RPL32;RPS8;FEM1B;RPS5;RPL13A;RPL35A;RPS3A;PDRG1;EEF1B2;RPS17;ARL6IP5;RPS20;TPT1
Mevastatin-1200_mg/kg_in_CMC-Rat-Liver-5d-up	14/315	0,00022728	0,0082491	0	0	3,054600993	25,62605344	RPL32;RPS8;RPS5;RPS6;RPL35A;RPSA;RPS3A;RPL10A;SEPP1;RPS17;RPS19;ARL6IP5;RPS20;TPT1
Oxcarbazepine-1600_mg/kg_in_CMC-Rat-Brain-5d-up	14/315	0,00022728	0,00821126	0	0	3,054600993	25,62605344	RPL32;RPS8;RPS6;RPL35A;RPSA;RPS3A;RPL10A;SEPP1;TOMM20;RPS17;RPS20;RAPGEF5;RPL39;GOLGA7
Teicoplanin-41_mg/kg_in_Saline-Rat-Bone_marrow-3d-dn	14/315	0,00022728	0,008173766	0	0	3,054600993	25,62605344	C1QA;RPL32;RPS8;RPS5;RPS6;NKG7;RPL13A;RPL35A;RPSA;RPS17;ARL6IP5;RPS20;RPL39;TPT1
Mitomycin_C-1.7_mg/kg_in_Saline-Rat-Bone_marrow-3d-dn	13/278	0,000232081	0,00830851	0	0	3,213923706	26,89547266	RPL32;RPS8;FEM1B;RPS6;NKG7;RPSA;RPS3A;EEF1B2;RPS17;ARL2BP;FUNDC1;RPS20;TPT1
Dimenhydrinate-165_mg/kg_in_CMC-Rat-Liver-5d-up	14/316	0,000234767	0,008366622	0	0	3,044934534	25,44626762	RPL32;RPS8;RPS5;RPS6;RPL13A;RPL35A;RPSA;RPS3A;SEPP1;RPS17;RPS19;ARL6IP5;RPS20;TPT1
Phenacemide-400_mg/kg_in_CMC-Rat-Kidney-5d-up	14/316	0,000234767	0,008328934	0	0	3,044934534	25,44626762	RPL32;RPS8;RPS5;RPS6;RPL35A;RPSA;RPS3A;RPL10A;SEPP1;EEF1B2;RPS17;CXCL11;RPS19;RPS20
Pirinixic_Acid-364_mg/kg_in_CMC-Rat-Liver-1d-up	14/316	0,000234767	0,008291585	0	0	3,044934534	25,44626762	RPL32;RPS8;RPS5;RPS6;RPL35A;RPSA;RPS3A;RPL10A;EEF1B2;RPS17;RPS19;RPS20;RPL39;TPT1
Primaquine-45_mg/kg_in_CMC-Rat-Liver-3d-up	14/316	0,000234767	0,008254569	0	0	3,044934534	25,44626762	C1QA;RPL32;RPS8;RPS5;RPS6;RPSA;RPS3A;RPL10A;EEF1B2;RPS19;ARL2BP;RPS20;RPL39;TPT1
Rifampin-125_uM_in_DMSO-Rat-Primary_rat_hepatocytes-1d-dn	15/355	0,000236164	0,008266804	0	0	2,904022071	24,25143717	RPL32;RPS8;RPS5;RPL35A;RPSA;RPL10A;SEPP1;EEF1B2;RPS17;CXCL11;RPS19;ARL2BP;ARL6IP5;RPL39;TPT1
Calcitriol-0.04_mg/kg_in_CMC-Rat-Kidney-5d-up	14/317	0,000242465	0,008449809	0	0	3,035329062	25,26805789	RPL32;RPS8;RPS6;RPSA;RPS3A;RPL10A;EEF1B2;CXCL11;RPS19;CLP1;RPS20;RAPGEF5;RPL39;TPT1
Catechol-195_mg/kg_in_Saline-Rat-Kidney-1d-up	14/317	0,000242465	0,008412585	0	0	3,035329062	25,26805789	RPL32;RPS8;RPS5;RPL13A;RPL35A;RPSA;RPS3A;SEPP1;TOMM20;EEF1B2;RPS17;ARL6IP5;RPS20;RPL39
Ethylene_Glycol-3525_mg/kg_in_Water-Rat-Bone_marrow-5d-up	14/317	0,000242465	0,008375688	0	0	3,035329062	25,26805789	C1QA;RPL32;RPS5;NKG7;RPL35A;RPSA;RPS3A;RPL10A;SEPP1;EEF1B2;RPS17;RPS20;RPL39;TPT1
Foscarnet-350_mg/kg_in_Saline-Rat-Kidney-5d-dn	14/317	0,000242465	0,008339113	0	0	3,035329062	25,26805789	RPL32;RPS5;RPS6;RPL35A;RPS3A;RPL10A;SEPP1;EEF1B2;RPS17;RPS19;ARL6IP5;RPS20;RAPGEF5;RPL39
Gabapentin-2500_mg/kg_in_Water-Rat-Heart-3d-up	14/317	0,000242465	0,008302856	0	0	3,035329062	25,26805789	RPL32;RPS6;RPSA;RPS3A;RPL10A;SEPP1;RPS17;RPS19;COL5A3;ARL6IP5;RPS20;RAPGEF5;RPL39;GOLGA7
Lamotrigine-26_mg/kg_in_CMC-Rat-Kidney-1d-dn	14/317	0,000242465	0,008266912	0	0	3,035329062	25,26805789	RPL32;RPS8;RPS6;RPSA;RPL10A;SEPP1;PDRG1;EEF1B2;RPS19;ARL2BP;ARL6IP5;RPS20;RPL39;TPT1
Ondansetron-84_mg/kg_in_Water-Rat-Intestine-3d-dn	14/317	0,000242465	0,008231279	0	0	3,035329062	25,26805789	RPL32;RPS8;RPS5;RPS6;RPL13A;RPL35A;RPSA;RPS3A;RPL10A;EEF1B2;RPS17;RPS20;RPL39;TPT1
Norethindrone-75_mg/kg_in_Corn_Oil-Rat-Liver-1d-up	12/243	0,000244657	0,008270028	0	0	3,394001103	28,2233403	EEF1B2;C1QA;RPS17;RPS19;RPS8;FEM1B;RPS6;RPS20;RPSA;RPS3A;RPL10A;RPL39
Phenacetin-619_mg/kg_in_Corn_Oil-Rat-Liver-5d-up	13/280	0,000248792	0,008373862	0	0	3,190967108	26,48149925	RPL32;RPS8;RPS5;RPS6;RPL35A;RPSA;RPS3A;RPL10A;SEPP1;RPS17;RPS19;RPS20;TPT1
Aldosterone-0.065_mg/kg_in_Saline-Rat-Kidney-1d-dn	14/318	0,00025038	0,00839146	0	0	3,025784002	25,09140654	RPL32;RPS5;RPS6;RPL35A;RPSA;RPS3A;RPL10A;EEF1B2;RPS17;ARL2BP;FUNDC1;RPS20;RPL39;TPT1
Citric_Acid-3000_mg/kg_in_Water-Rat-Liver-3d-up	14/318	0,00025038	0,008355903	0	0	3,025784002	25,09140654	RPL32;RPS8;RPS5;RPS6;RPL35A;RPSA;RPS3A;RPL10A;EEF1B2;RPS17;RPS19;ARL6IP5;RPS20;RPL39
Kanamycin-225_mg/kg_in_Saline-Rat-Kidney-3d-up	14/318	0,00025038	0,008320646	0	0	3,025784002	25,09140654	C1QA;RPL32;RPS8;RPS5;RPL13A;RPL35A;RPSA;RPS3A;RPS17;CXCL11;ARL6IP5;RPS20;RPL39;TPT1
Zidovudine-1473_uM_in_DMSO-Rat-Primary_rat_hepatocytes-1d-dn	14/319	0,000258516	0,008554924	0	0	3,016298786	24,91629609	RPL32;RPS8;RPS6;RPL35A;RPS3A;RPL10A;RPS17;CXCL11;RPS19;ARL6IP5;RPS20;RPL39;GOLGA7;TPT1
Clomipramine-115_mg/kg_in_Water-Rat-Heart-3d-dn	14/320	0,000266878	0,008794697	0	0	3,006872852	24,74270932	RPL32;RPS8;RPS6;RPSA;RPS3A;RPL10A;SEPP1;EEF1B2;RPS17;RPS19;FUNDC1;RPS20;RPL39;TPT1
Cyclosporin_A-70_mg/kg_in_Corn_Oil-Rat-Bone_marrow-3d-up	14/320	0,000266878	0,008758053	0	0	3,006872852	24,74270932	C1QA;RPL32;RPS8;RPS5;RPS6;RPL35A;RPSA;RPL10A;EEF1B2;CLP1;RPS20;RPL39;SLC16A3;TPT1
Pralidoxime_Chloride-48_mg/kg_in_Saline-Rat-Liver-1d-dn	14/320	0,000266878	0,008721712	0	0	3,006872852	24,74270932	RPL32;RPS8;RPS5;RPS6;RPL35A;RPSA;RPS3A;RPL10A;EEF1B2;RPS17;RPS19;RPS20;RPL39;TPT1
Rolipram-75_mg/kg_in_CMC-Rat-Heart-3d-up	14/320	0,000266878	0,008685672	0	0	3,006872852	24,74270932	RPS8;RPSA;RPS3A;RPL10A;TOMM20;PDRG1;EEF1B2;ARL2BP;FUNDC1;ARL6IP5;RPS20;RAPGEF5;RPL39;TPT1
Cadmium_Chloride-66_mg/kg_in_Corn_Oil-Rat-Kidney-5d-up	15/360	0,000274204	0,008887385	0	0	2,863688431	23,48693127	RPL32;RPS8;RPS5;RPS6;RPL35A;RPSA;RPS3A;RPL10A;EEF1B2;RPS17;CXCL11;RPS19;ARL6IP5;RPS20;RPL39
Diphenhydramine-100_mg/kg_in_Water-Rat-Heart-3d-up	14/321	0,000275472	0,00889187	0	0	2,997505647	24,57062923	RPL32;RPS8;RPS5;RPS6;RPL35A;RPSA;RPS3A;SEPP1;RPS17;RPS19;ARL2BP;RPS20;GOLGA7;TPT1
Leflunomide-60_mg/kg_in_Corn_Oil-Rat-Spleen-5d-up	14/321	0,000275472	0,008855576	0	0	2,997505647	24,57062923	C1QA;RPL32;RPS8;RPSA;RPS3A;RPL10A;SEPP1;EEF1B2;RPS17;CXCL11;RPS20;RAPGEF5;RPL39;TPT1
Terbutaline-130_mg/kg_in_Corn_Oil-Rat-Heart-3d-up	14/321	0,000275472	0,008819578	0	0	2,997505647	24,57062923	C1QA;RPL32;RPS8;RPS5;RPS6;RPSA;RPS3A;RPL10A;SEPP1;EEF1B2;RPS19;RPS20;RPL39;TPT1
Coumarin-111_mg/kg_in_Corn_Oil-Rat-Bone_marrow-5d-up	13/283	0,000275784	0,008793829	0	0	3,157140602	25,87558372	C1QA;RPL32;RPS8;FEM1B;RPS5;RPS6;RPL35A;RPSA;RPS3A;RPS17;CLP1;RPS20;TPT1
4-Octylphenol-50_mg/kg_in_Corn_Oil-Rat-Liver-0.25d-up	15/361	0,000282416	0,008968984	0	0	2,855755776	23,33760611	RPL32;RPS8;RPS5;RPS6;RPL35A;RPSA;RPS3A;RPL10A;SEPP1;EEF1B2;RPS17;RPS19;RPS20;RPL39;TPT1
Diclofenac-10_mg/kg_in_Corn_Oil-Rat-Kidney-5d-up	14/322	0,000284302	0,008992625	0	0	2,988196623	24,40003908	C1QA;RPL32;RPS8;RPS5;RPS6;RPL35A;RPSA;RPS3A;RPS17;RPS19;RPS20;RPL39;GOLGA7;TPT1
Sparteine-33_mg/kg_in_Water-Rat-Heart-1d-up	14/322	0,000284302	0,008956654	0	0	2,988196623	24,40003908	C1QA;RPL32;RPS8;RPS5;RPS6;RPL13A;RPL35A;RPSA;RPS3A;TOMM20;RPS17;RPS20;RPL39;TPT1
Carbon_Tetrachloride-1175_mg/kg_in_Corn_Oil-Rat-Liver-1d-up	13/284	0,000285321	0,008952929	0	0	3,14602391	25,67752326	RPL32;RPS8;RPS5;RPS6;RPSA;RPS3A;RPL10A;EEF1B2;RPS17;RPS19;RPS20;RPL39;TPT1
Diphenhydramine-10.9_mg/kg_in_Water-Rat-Heart-3d-up	15/362	0,000290839	0,009089863	0	0	2,847866948	23,18944534	RPL32;RPS8;RPS5;RPS6;RPL35A;RPSA;RPS3A;RPL10A;RPS17;RPS19;ARL2BP;RPS20;RPL39;GOLGA7;TPT1
Eucalyptol-930_mg/kg_in_Corn_Oil-Rat-Kidney-3d-up	14/323	0,000293374	0,009132872	0	0	2,978945241	24,23092236	RPL32;RPS8;RPS5;RPS6;RPL13A;RPL35A;RPSA;RPS3A;EEF1B2;RPS17;RPS19;ARL2BP;ARL6IP5;RPS20
Lomustine-560_uM_in_DMSO-Rat-Primary_rat_hepatocytes-0.67d-up	14/323	0,000293374	0,009096916	0	0	2,978945241	24,23092236	RPL32;RPS8;FEM1B;RPS5;RPL13A;PDRG1;EEF1B2;RPS17;RPS19;ARL2BP;CLP1;ARL6IP5;RPS20;GOLGA7
Carboplatin-6_mg/kg_in_Saline-Rat-Liver-0.25d-up	13/285	0,000295137	0,009115695	0	0	3,134985229	25,48137777	RPL32;RPS8;RPS5;RPS6;RPL35A;RPS3A;RPL10A;EEF1B2;RPS17;RPS19;RPS20;RPL39;TPT1
Ergocalciferol-15_mg/kg_in_CMC-Rat-Kidney-5d-up	13/285	0,000295137	0,009080087	0	0	3,134985229	25,48137777	C1QA;RPL32;RPS8;RPS6;RPL35A;RPSA;RPS3A;RPL10A;EEF1B2;RPS17;RPS19;RPS20;RAPGEF5
Lacidipine-14_mg/kg_in_Corn_Oil-Rat-Heart-3d-up	13/285	0,000295137	0,009044756	0	0	3,134985229	25,48137777	C1QA;RPL32;RPS6;RPL35A;RPS3A;RPL10A;SEPP1;RPS17;RPS19;RPS20;RAPGEF5;RPL39;TPT1
Phenelzine-27_mg/kg_in_Water-Rat-Brain-3d-up	13/285	0,000295137	0,009009699	0	0	3,134985229	25,48137777	RPL32;RPL35A;RPSA;RPS3A;RPL10A;EEF1B2;RPS17;RPS19;ARL2BP;ARL6IP5;RPS20;RPL39;TPT1
Ranitidine-1500_mg/kg_in_CMC-Rat-Heart-1d-dn	13/285	0,000295137	0,008974912	0	0	3,134985229	25,48137777	C1QA;RPL32;RPS8;RPS5;RPSA;RPS3A;TOMM20;EEF1B2;RPS17;RPS19;ARL2BP;RPL39;TPT1
Artemether-74_mg/kg_in_Corn_Oil-Rat-Liver-5d-up	14/324	0,000302694	0,0091693	0	0	2,969750965	24,06326276	RPL32;RPS8;RPS5;RPS6;RPL35A;RPSA;RPS3A;RPL10A;SEPP1;EEF1B2;RPS17;RPS19;RPS20;TPT1
Chlorambucil-4.5_mg/kg_in_Corn_Oil-Rat-Spleen-5d-dn	14/324	0,000302694	0,009134169	0	0	2,969750965	24,06326276	RPS8;RPS5;RPS6;RPL13A;RPL35A;RPSA;RPS3A;RPL10A;RPS17;RPS19;FUNDC1;RPS20;RPL39;TPT1
Penicillamine-852_mg/kg_in_Water-Rat-Bone_marrow-5d-up	14/324	0,000302694	0,009099306	0	0	2,969750965	24,06326276	C1QA;RPL32;RPS5;RPS6;NKG7;RPL35A;RPS3A;RPL10A;SEPP1;EEF1B2;RPS17;RPS20;RPL39;TPT1
Atenolol-1000_mg/kg_in_CMC-Rat-Heart-5d-up	14/325	0,000312267	0,009351374	0	0	2,96061327	23,89704424	C1QA;RPL32;RPS8;RPS5;RPS6;RPL35A;RPSA;RPS3A;TOMM20;EEF1B2;RPS17;RPS19;FUNDC1;TPT1
Bromfenac-5_mg/kg_in_CMC-Rat-Liver-3d-up	14/325	0,000312267	0,009315952	0	0	2,96061327	23,89704424	RPL32;RPS8;RPS5;RPS6;RPSA;RPS3A;RPL10A;EEF1B2;RPS17;RPS19;ARL2BP;RPS20;RPL39;TPT1
Norethindrone_Acetate-125_mg/kg_in_Corn_Oil-Rat-Liver-5d-up	14/325	0,000312267	0,009280797	0	0	2,96061327	23,89704424	RPL32;RPS8;RPS5;RPS6;RPL13A;RPL35A;RPSA;RPS3A;RPL10A;RPS17;RPS19;RPS20;RPL39;TPT1
Ethinylestradiol-10_mg/kg_in_Corn_Oil-Rat-Liver-1d-up	13/287	0,000315639	0,009345757	0	0	3,113138642	25,09473664	RPL32;RPS8;FEM1B;RPS5;RPS6;RPSA;RPS3A;RPL10A;EEF1B2;RPS17;RPS19;RPS20;RPL39
Lead_(II)_Acetate-200_mg/kg_in_Saline-Rat-Liver-1d-dn	13/287	0,000315639	0,009310754	0	0	3,113138642	25,09473664	RPL32;RPS8;RPS5;RPS6;RPSA;RPS3A;SEPP1;EEF1B2;RPS17;RPS19;RPS20;RPL39;TPT1

Oxaliplatin-8_mg/kg_in_Saline-Rat-Bone_marrow-1d-dn	13/287	0,000315639	0,009276012	0	0	3,113138642	25,09473664	RPL32;RPS8;RPS5;RPS6;RPL35A;RPSA;RPS3A;EEF1B2;RPS17;RPS19;RPS20;RPL39;TPT1
Oxytetracycline-1500_mg/kg_in_Water-Rat-Liver-5d-dn	13/287	0,000315639	0,009241529	0	0	3,113138642	25,09473664	RPL32;RPS8;RPS5;RPS6;RPL35A;RPSA;RPS3A;RPL10A;SEPP1;EEF1B2;RPS17;RPS19;RPL39
Propranolol-175_mg/kg_in_Water-Rat-Heart-1d-up	15/365	0,000317419	0,009259238	0	0	2,824459822	22,75183461	RPL32;RPS8;RPS5;RPS6;RPL35A;RPSA;RPS3A;EEF1B2;RPS17;CXCL11;RPS19;ARL6IP5;RPS20;RPL39;TPT1
Cytarabine-487_mg/kg_in_Saline-Rat-Bone_marrow-1d-up	14/326	0,000322098	0,009361034	0	0	2,951531634	23,73225095	C1QA;RPL32;RPS8;RPS5;RPS6;RPL35A;RPSA;RPS3A;RPL10A;EEF1B2;RPS17;RPS19;RPS20;TPT1
Famciclovir-1200_mg/kg_in_Saline-Rat-Liver-1d-dn	14/326	0,000322098	0,009326618	0	0	2,951531634	23,73225095	RPL32;RPS8;RPS5;RPS6;RPL35A;RPS3A;RPL10A;SEPP1;EEF1B2;RPS17;RPS19;RPS20;RPL39;TPT1
Dexamethasone-150_mg/kg_in_Corn_Oil-Rat-Liver-3d-dn	13/288	0,000326337	0,009414765	0	0	3,102329133	24,90419438	C1QA;RPL32;RPS8;RPS6;RPSA;RPS3A;RPL10A;SEPP1;EEF1B2;RPS17;RPS19;RPS20;RPL39
Megestrol_Acetate-132_mg/kg_in_CMC-Rat-Liver-3d-dn	13/288	0,000326337	0,009380405	0	0	3,102329133	24,90419438	C1QA;RPL32;RPS8;FEM1B;RPS6;RPL35A;RPS3A;SEPP1;RPS17;RPS19;CLP1;RPS20;TPT1
Dexfenfluramine-29_mg/kg_in_Water-Rat-Kidney-1d-dn	11/215	0,000326663	0,009355624	0	0	3,516343003	28,22421538	RPS17;RPL32;RPS19;RPS6;ARL6IP5;RPL35A;RPS20;RPSA;RPS3A;RPL10A;RPL39
Risperidone-400_uM_in_DMSO-Rat-Primary_rat_hepatocytes-1d-dn	16/407	0,000330736	0,009437968	0	0	2,701858372	21,65320395	RPL32;RPS8;RPS5;RPS6;RPS3A;RPL10A;SEPP1;EEF1B2;RPS17;CXCL11;RPS19;ARL2BP;ARL6IP5;RPL39;SLC16A3;TPT1
Amlodipine-19_mg/kg_in_Corn_Oil-Rat-Liver-0.25d-up	14/327	0,000332193	0,009445306	0	0	2,942505543	23,56886726	RPL32;RPS8;FEM1B;RPS5;RPL13A;RPL35A;RPSA;RPS3A;SEPP1;TOMM20;EEF1B2;RPS17;RPS19;TPT1
Cycloheximide-0.25_mg/kg_in_Water-Rat-Liver-1d-up	14/327	0,000332193	0,00941133	0	0	2,942505543	23,56886726	RPL32;RPS8;RPS5;RPS6;RPL35A;RPSA;RPS3A;RPL10A;EEF1B2;RPS17;RPS19;RPS20;RPL39;TPT1
Cyclosporin_A-350_mg/kg_in_Corn_Oil-Rat-Heart-5d-up	13/289	0,000337343	0,009522989	0	0	3,09159443	24,71547386	C1QA;RPS8;RPS6;RPSA;RPS3A;RPL10A;SEPP1;CXCL11;FUNDC1;RPS20;RAPGEF5;RPL39;TPT1
Nitrendipine-1100_mg/kg_in_Corn_Oil-Rat-Heart-3d-up	13/289	0,000337343	0,009488979	0	0	3,09159443	24,71547386	C1QA;RPL32;RPS5;RPS6;RPL35A;RPSA;RPS3A;RPL10A;SEPP1;RPS19;RPS20;RPL39;TPT1
Sodium_Arsenite-30_mg/kg_in_Saline-Rat-Liver-0.25d-up	13/289	0,000337343	0,00945521	0	0	3,09159443	24,71547386	C1QA;RPL32;RPS8;RPS5;RPS6;RPL35A;RPSA;RPS3A;SEPP1;EEF1B2;RPS17;RPS20;TPT1
Zomepirac-2.8_mg/kg_in_Saline-Rat-Kidney-1d-dn	13/289	0,000337343	0,009421681	0	0	3,09159443	24,71547386	RPL32;RPS8;RPS6;RPSA;RPS3A;TOMM20;EEF1B2;RPS17;ARL2BP;R3HCC1;RPS20;RPL39;TPT1
11-Dichloroethene-600_mg/kg_in_Water-Rat-Liver-3d-up	14/328	0,000342558	0,009533516	0	0	2,93353449	23,40687777	C1QA;RPL32;RPS8;RPS5;RPS6;RPL35A;RPSA;RPS3A;RPL10A;EEF1B2;RPS17;RPS19;RPS20;RPL39
Cadmium_Chloride-66_mg/kg_in_Corn_Oil-Rat-Kidney-1d-up	14/328	0,000342558	0,009499947	0	0	2,93353449	23,40687777	RPL32;RPS8;RPS5;RPS6;RPSA;RPS3A;RPL10A;SEPP1;TOMM20;RPS17;RPS19;RPS20;RAPGEF5;TPT1
Tacrolimus-134_mg/kg_in_CMC-Rat-Heart-1d-up	14/328	0,000342558	0,009466614	0	0	2,93353449	23,40687777	RPL32;RPS6;RPSA;RPS3A;RPL10A;SEPP1;EEF1B2;RPS17;CXCL11;RPS19;ARL6IP5;RPS20;RAPGEF5;RPL39
Vinorelbine-1.5_mg/kg_in_Saline-Rat-Bone_marrow-1d-dn	14/328	0,000342558	0,009433514	0	0	2,93353449	23,40687777	RPL32;RPS8;RPS5;RPS6;NKG7;RPSA;RPS3A;RPL10A;EEF1B2;RPS17;RPS19;RPS20;RPL39;TPT1
Mestranol-250_mg/kg_in_CMC-Rat-Liver-1d-up	13/290	0,000348664	0,009568212	0	0	3,08093376	24,52855266	RPL32;RPS8;FEM1B;RPS5;RPS6;RPSA;RPS3A;RPL10A;EEF1B2;RPS17;RPS19;RPS20;RPL39
Allopurinol-175_mg/kg_in_Corn_Oil-Rat-Kidney-3d-up	14/329	0,000353199	0,009659002	0	0	2,924617972	23,24626728	C1QA;RPL32;RPS8;RPS5;RPS6;RPSA;RPS3A;RPL10A;EEF1B2;RPS17;RPS19;RPS20;RPL39;TPT1
Rofecoxib-250_mg/kg_in_Corn_Oil-Rat-Liver-1d-dn	14/329	0,000353199	0,00962558	0	0	2,924617972	23,24626728	C1QA;RPL32;RPS6;RPL35A;RPSA;RPS3A;RPL10A;EEF1B2;RPS17;RPS19;RPS20;RPL39;GOLGA7;TPT1
Vincristine-0.05_mg/kg_in_Saline-Rat-Heart-5d-up	14/329	0,000353199	0,009592388	0	0	2,924617972	23,24626728	RPL32;RPS8;RPS6;RPL35A;RPSA;RPS3A;RPL10A;EEF1B2;RPS17;RPS19;COL5A3;RPS20;RPL39;TPT1
Mestranol-250_mg/kg_in_CMC-Rat-Liver-3d-up	13/291	0,000360307	0,009751809	0	0	3,070346359	24,34340873	RPL32;RPS8;FEM1B;RPS5;RPS6;RPSA;RPS3A;RPL10A;EEF1B2;RPS17;RPS19;RPS20;RPL39
Cisplatin-0.5_mg/kg_in_Saline-Rat-Kidney-28d-up	14/330	0,000364121	0,009821298	0	0	2,915755493	23,0870208	C1QA;RPL32;RPS8;FEM1B;RPS6;RPSA;RPS3A;RPL10A;EEF1B2;CXCL11;RPS19;ARL6IP5;RPS20;RPL39
Ofloxacin-3000_mg/kg_in_Water-Rat-Heart-3d-up	13/292	0,000372279	0,010007069	0	0	3,059831474	24,16002033	RPL32;RPS8;RPS5;RPS6;RPS3A;SEPP1;RPS17;RPS19;ARL2BP;FUNDC1;ARL6IP5;RPS20;RAPGEF5
Acetaminophen-486_mg/kg_in_Corn_Oil-Rat-Kidney-1d-dn	13/293	0,000384589	0,01030279	0	0	3,049388363	23,97836608	RPL32;RPS8;RPS6;RPL35A;RPSA;RPS3A;RPL10A;TOMM20;EEF1B2;RPS17;ARL2BP;CLP1;TPT1
Calcitriol-0.04_mg/kg_in_CMC-Rat-Heart-5d-up	13/293	0,000384589	0,010267865	0	0	3,049388363	23,97836608	RPL32;RPS8;RPS6;RPS3A;RPL10A;SEPP1;EEF1B2;RPS17;RPS19;ARL2BP;RPS20;RPL39;TPT1
Vinorelbine-1.5_mg/kg_in_Saline-Rat-Liver-5d-dn	13/293	0,000384589	0,010233176	0	0	3,049388363	23,97836608	C1QA;RPL32;RPS8;RPS5;RPS6;RPL35A;RPSA;RPS3A;SEPP1;EEF1B2;RPS17;RPS19;TPT1
Amantadine-220_mg/kg_in_Water-Rat-Liver-3d-up	14/332	0,000386836	0,01025832	0	0	2,898190701	22,77256091	RPL32;RPS8;RPS5;RPS6;RPL35A;RPSA;RPS3A;RPL10A;RPS17;RPS19;RPS20;RAPGEF5;RPL39;TPT1
1-Naphthyl_Isothiocyanate-30_mg/kg_in_Corn_Oil-Rat-Bone_marrow-1d-dn	13/294	0,000397243	0,010498937	0	0	3,039016294	23,79842489	RPL32;RPS8;RPS5;RPS6;RPL35A;RPSA;RPS3A;EEF1B2;RPS17;RPS19;ARL6IP5;RPS20;TPT1
Bisphenol_A-100_mg/kg_in_Corn_Oil-Rat-Spleen-1d-up	13/294	0,000397243	0,010463823	0	0	3,039016294	23,79842489	C1QA;RPL32;RPS8;RPL35A;RPS3A;SEPP1;RPS17;ARL2BP;FUNDC1;ARL6IP5;RPS20;GOLGA7;TPT1
Cholecalciferol-8_mg/kg_in_CMC-Rat-Liver-3d-up	13/294	0,000397243	0,010428944	0	0	3,039016294	23,79842489	C1QA;RPL32;RPS8;RPS5;RPS6;RPSA;RPS3A;RPL10A;SEPP1;EEF1B2;RPS17;RPS19;RPL39
S(-)-Carbidopa-1200_mg/kg_in_CMC-Rat-Brain-3d-dn	13/294	0,000397243	0,010394296	0	0	3,039016294	23,79842489	RPL32;RPS8;RPS6;RPL35A;RPSA;RPS3A;SEPP1;EEF1B2;RPS17;ARL6IP5;RPS20;RPL39;TPT1
44'-Methylenedianiline-81_mg/kg_in_Corn_Oil-Rat-Liver-3d-up	14/333	0,000398641	0,010396338	0	0	2,889487426	22,61731852	C1QA;RPL32;RPS8;RPS5;RPS6;RPSA;RPS3A;RPL10A;SEPP1;RPS17;RPS19;RPS20;RPL39;TPT1
Mefenamic_Acid-93_mg/kg_in_Corn_Oil-Rat-Kidney-5d-dn	14/333	0,000398641	0,010362027	0	0	2,889487426	22,61731852	RPL32;RPS8;RPS6;RPSA;RPS3A;RPL10A;SEPP1;EEF1B2;RPS17;RPS19;ARL6IP5;RPS20;RAPGEF5;RPL39
Ethanol-3000_mg/kg_in_Saline-Rat-Liver-1d-dn	12/257	0,000406655	0,010535572	0	0	3,209113883	25,05530333	RPS17;RPL32;RPS19;RPS8;RPS5;RPS6;RPL35A;RPS20;RPS3A;RPL10A;RPL39;TPT1
Ifosfamide-17_mg/kg_in_Saline-Rat-Bone_marrow-3d-dn	12/257	0,000406655	0,010501029	0	0	3,209113883	25,05530333	EEF1B2;RPS17;RPL32;RPS19;RPS6;ARL6IP5;RPL35A;RPS20;RPSA;RPS3A;RPL39;TPT1
Raloxifene-5_mg/kg_in_Corn_Oil-Rat-Liver-5d-up	12/257	0,000406655	0,010466712	0	0	3,209113883	25,05530333	EEF1B2;RPS17;RPL32;RPS19;RPS6;RPL35A;RPS20;RPSA;RPS3A;RPL10A;RPL39;TPT1
Anisindione-75_mg/kg_in_CMC-Rat-Liver-5d-up	13/295	0,000410249	0,010524825	0	0	3,028714544	23,62017604	RPL32;RPS8;RPS5;RPS6;RPL35A;RPSA;RPS3A;RPL10A;RPS17;RPS19;ARL2BP;RPS20;TPT1
Famciclovir-112_mg/kg_in_Saline-Rat-Liver-1d-dn	13/295	0,000410249	0,010490654	0	0	3,028714544	23,62017604	RPL32;RPS8;RPS5;RPS6;RPL35A;RPSA;RPS3A;RPL10A;SEPP1;EEF1B2;RPS17;RPS19;TPT1
Lipopolysaccharide_E._Coli_O55:B5-1.25_mg/kg_in_Saline-Rat-Kidney-3d-up	13/295	0,000410249	0,010456703	0	0	3,028714544	23,62017604	C1QA;RPS8;FEM1B;RPS6;RPSA;RPS3A;RPL10A;SEPP1;CXCL11;RPS19;RPS20;RPL39;TPT1
Tranilast-1100_mg/kg_in_CMC-Rat-Heart-3d-dn	13/295	0,000410249	0,010422972	0	0	3,028714544	23,62017604	RPL32;RPS6;RPSA;RPS3A;SEPP1;RPS17;CXCL11;RPS19;FUNDC1;ARL6IP5;RPS20;RAPGEF5;RPL39
Pravastatin-1200_mg/kg_in_Corn_Oil-Rat-Kidney-5d-dn	12/258	0,000421067	0,010663408	0	0	3,196675458	24,84686255	EEF1B2;RPS17;RPL32;RPS19;CLP1;RPS8;RPS6;RPSA;RPS3A;RPL10A;RPL39;TPT1
Busulfan-9_mg/kg_in_Corn_Oil-Rat-Bone_marrow-3d-up	14/335	0,000423176	0,010682481	0	0	2,872236754	22,31073789	C1QA;RPL32;RPS8;RPS5;RPL35A;RPSA;RPS3A;RPL10A;SEPP1;EEF1B2;RPS17;RPS20;RPL39;TPT1
Bromodichloromethane-430_mg/kg_in_CMC-Rat-Kidney-1d-up	13/296	0,000423616	0,010659414	0	0	3,0184824	23,44359907	RPS8;RPS5;RPS6;RPSA;RPS3A;RPL10A;EEF1B2;RPS17;RPS19;ARL6IP5;RPS20;RAPGEF5;TPT1
Carmustine-16_mg/kg_in_Corn_Oil-Rat-Spleen-3d-up	13/296	0,000423616	0,010625467	0	0	3,0184824	23,44359907	C1QA;RPL32;RPS8;RPS5;RPS6;RPL35A;RPS3A;EEF1B2;RPS17;CXCL11;RPS20;RAPGEF5;RPL39
Disulfiram-100_mg/kg_in_Water-Rat-Liver-1d-up	13/296	0,000423616	0,010591736	0	0	3,0184824	23,44359907	RPL32;RPS8;RPS5;RPS6;RPL35A;RPSA;RPS3A;RPL10A;EEF1B2;RPS17;RPS19;RPS20;RPL39
Lovastatin-1500_mg/kg_in_Corn_Oil-Rat-Skeletal_muscle-3d-dn	13/296	0,000423616	0,010558218	0	0	3,0184824	23,44359907	RPL32;RPS8;RPS5;RPS6;RPL35A;RPS3A;RPL10A;EEF1B2;RPS17;RPS19;ARL6IP5;RPS20;RPL39
Raloxifene-5_mg/kg_in_Corn_Oil-Rat-Liver-1d-up	13/296	0,000423616	0,010524911	0	0	3,0184824	23,44359907	RPL32;RPS8;RPS6;RPSA;RPS3A;RPL10A;TOMM20;EEF1B2;RPS17;RPS19;RPS20;RPL39;TPT1
Famciclovir-112_mg/kg_in_Saline-Rat-Spleen-3d-dn	12/259	0,000435907	0,010796243	0	0	3,184333081	24,64062785	EEF1B2;C1QA;RPS17;RPL32;ARL2BP;RPS8;RPS5;FUNDC1;ARL6IP5;RPL13A;RPL35A;RPSA
33'5-Triiodo-L-Thyronine-0.001_mg/kg_in_PEG400-Rat-Heart-1d-dn	13/297	0,00043735	0,010798032	0	0	3,00831916	23,26867389	C1QA;RPL32;RPS8;RPS6;RPSA;RPS3A;RPL10A;RPS17;CXCL11;RPS19;ARL6IP5;RPL39;TPT1
Carbimazole-400_mg/kg_in_Water-Rat-Liver-5d-up	13/297	0,00043735	0,010764289	0	0	3,00831916	23,26867389	RPL32;RPS8;FEM1B;RPS5;RPS6;RPSA;RPS3A;RPL10A;SEPP1;RPS17;RPS19;RPS20;RPL39
Diethylstilbestrol-2.8_mg/kg_in_Corn_Oil-Rat-Liver-1d-up	13/297	0,00043735	0,010730755	0	0	3,00831916	23,26867389	RPL32;RPS8;FEM1B;RPS5;RPS6;RPSA;RPS3A;RPL10A;EEF1B2;RPS17;RPS19;RPS20;RPL39
N-Nitrosodiethylamine-1.67_mg/kg_in_Saline-Rat-Liver-0.25d-up	15/377	0,000445738	0,010902573	0	0	2,734556592	21,09923796	C1QA;RPL32;RPS8;RPS5;RPS6;RPL35A;RPSA;RPS3A;RPL10A;SEPP1;EEF1B2;RPS17;RPS19;RPS20;TPT1
Allopurinol-175_mg/kg_in_Corn_Oil-Rat-Kidney-1d-up	14/337	0,000448989	0,010948115	0	0	2,855190839	22,00927035	RPL32;RPS8;RPS5;RPS6;RPSA;RPS3A;RPL10A;EEF1B2;RPS17;RPS19;ARL2BP;ARL6IP5;RPS20;RPL39
Fluvastatin-94_mg/kg_in_Corn_Oil-Rat-Kidney-5d-up	14/337	0,000448989	0,010914324	0	0	2,855190839	22,00927035	RPL32;RPS8;RPS5;RPS6;RPL13A;RPL35A;RPS3A;RPL10A;SEPP1;TOMM20;EEF1B2;RPS17;RPS19;RPS20
Meloxicam-33_mg/kg_in_Corn_Oil-Rat-Kidney-1d-up	14/337	0,000448989	0,010880742	0	0	2,855190839	22,00927035	RPL32;RPS8;RPS5;RPS6;RPL13A;RPL35A;RPSA;RPS3A;EEF1B2;RPS17;RPS19;RPS20;RPL39;TPT1
Rifapentine-75_mg/kg_in_Corn_Oil-Rat-Kidney-1d-up	14/337	0,000448989	0,010847365	0	0	2,855190839	22,00927035	RPL32;RPS8;RPS6;RPL35A;RPSA;RPS3A;RPL10A;EEF1B2;RPS17;RPS19;ARL2BP;ARL6IP5;RPS20;TPT1
Tamoxifen-32_mg/kg_in_Corn_Oil-Rat-Liver-3d-up	14/337	0,000448989	0,010814193	0	0	2,855190839	22,00927035	RPL32;RPS8;RPS5;RPS6;RPL35A;RPSA;RPS3A;RPL10A;EEF1B2;RPS17;RPS19;RPS20;RPL39;TPT1
Amitriptyline-160_mg/kg_in_Water-Rat-Kidney-5d-dn	12/260	0,000451187	0,010833993	0	0	3,172085646	24,43656906	RPS17;RPL32;RPS19;RPS8;RPS5;RPS6;RPL35A;RPS20;RPSA;RPS3A;RPL39;SEPP1
Atenolol-1000_mg/kg_in_CMC-Rat-Heart-3d-up	13/298	0,000451462	0,010807636	0	0	2,998224129	23,09538065	RPL32;RPS8;RPS5;RPL13A;RPL35A;RPSA;RPS3A;TOMM20;RPS17;RPS19;FUNDC1;ARL6IP5;RAPGEF5
Lead_(II)_Acetate-600_mg/kg_in_Saline-Rat-Bone_marrow-5d-up	13/298	0,000451462	0,010774885	0	0	2,998224129	23,09538065	RPL32;RPS8;FEM1B;RPS5;RPS6;RPL35A;RPSA;RPS3A;RPS17;RPS19;FUNDC1;RPS20;TPT1
Oxiconazole-1500_mg/kg_in_Corn_Oil-Rat-Liver-1d-up	13/298	0,000451462	0,010742333	0	0	2,998224129	23,09538065	C1QA;RPL32;RPS8;RPS6;RPSA;RPS3A;EEF1B2;RPS17;RPS19;ARL2BP;RPS20;RPL39;TPT1
Ramipril-1500_mg/kg_in_CMC-Rat-Kidney-5d-up	15/378	0,000458197	0,01086976	0	0	2,727322315	20,96822979	RPL32;RPS5;RPS6;RPL13A;RPL35A;RPSA;RPS3A;SEPP1;TOMM20;EEF1B2;RPS17;CXCL11;ARL6IP5;RPS20;RPL39
Fenofibrate-215_mg/kg_in_Corn_Oil-Rat-Skeletal_muscle-3d-dn	13/299	0,000465957	0,011020664	0	0	2,988196623	22,92369984	RPL32;RPS8;FEM1B;RPS5;RPL35A;RPS3A;TOMM20;EEF1B2;RPS17;ARL6IP5;RPS20;RPL39;SLC16A3
Propylene_Glycol-2000_mg/kg_in_Water-Rat-Kidney-5d-dn	10/189	0,000465975	0,010988075	0	0	3,636429753	27,89643124	RPS17;RPL32;RPS19;RPS6;RPL35A;RPSA;RPS20;RPS3A;RPL10A;RPL39
Beta-Estradiol_3-Benzoate-25_mg/kg_in_Corn_Oil-Rat-Liver-1d-up	12/261	0,000466917	0,010977417	0	0	3,159932061	24,23465653	EEF1B2;RPS17;RPL32;RPS19;RPS8;FEM1B;RPS5;RPS6;RPL35A;RPSA;RPS3A;RPL10A
Vecuronium_Bromide-0.05_mg/kg_in_Saline-Rat-Liver-3d-dn	14/339	0,000476135	0,011160823	0	0	2,838346055	21,71280754	RPL32;RPS8;RPS6;RPL35A;RPSA;RPS3A;RPL10A;SEPP1;EEF1B2;RPS17;RPS19;RPS20;RPL39;TPT1
Zileuton-450_mg/kg_in_Corn_Oil-Rat-Kidney-0.25d-up	14/339	0,000476135	0,011127705	0	0	2,838346055	21,71280754	RPL32;RPS8;RPS5;RPL13A;RPL35A;RPSA;RPS3A;SEPP1;TOMM20;RPS17;RPS19;FUNDC1;RPS20;RPL39;TPT1
Chlortetracycline-1500_mg/kg_in_CMC-Rat-Liver-3d-up	13/301	0,000496137	0,011560864	0	0	2,968341496	22,58509888	RPL32;RPS8;RPS5;RPS6;RPL13A;RPL35A;RPSA;RPS3A;RPL10A;RPS17;RPS19;RPS20;RPL39
Cytarabine-23_mg/kg_in_Saline-Rat-Spleen-3d-dn	13/301	0,000496137	0,011526761	0	0	2,968341496	22,58509888	C1QA;RPL32;RPS8;RPS5;RPS6;RPL35A;RPSA;RPS3A;EEF1B2;RPS17;ARL2BP;ARL6IP5;RPL39
DActinomycin-0.06_mg/kg_in_Saline-Rat-Bone_marrow-5d-up	13/301	0,000496137	0,011492859	0	0	2,968341496	22,58509888	RPL32;RPS8;RPS5;RPS6;NKG7;RPSA;RPS3A;RPS17;RPS19;ARL2BP;ARL6IP5;RPS20;TPT1
Ethanol-3000_mg/kg_in_Saline-Rat-Kidney-0.25d-up	13/301	0,000496137	0,011459156	0	0	2,968341496	22,58509888	RPL32;RPS8;RPS5;RPL13A;RPL35A;RPS3A;TOMM20;EEF1B2;RPS17;RPS19;CLP1;RPS20;TPT1
Norethindrone-375_mg/kg_in_Corn_Oil-Rat-Liver-1d-up	13/301	0,000496137	0,011425649	0	0	2,968341496	22,58509888	RPL32;RPS8;FEM1B;RPS5;RPS6;RPSA;RPS3A;RPL10A;EEF1B2;RPS17;RPS19;RPS20;RPL39
Oxfendazole-1500_mg/kg_in_CMC-Rat-Liver-1d-dn	13/301	0,000496137	0,011392338	0	0	2,968341496	22,58509888	C1QA;RPL32;RPS8;RPS6;RPL35A;RPS3A;RPL10A;SEPP1;RPS17;CLP1;RPS20;RPL39;TPT1
Oxfendazole-1500_mg/kg_in_CMC-Rat-Liver-5d-dn	13/301	0,000496137	0,011359221	0	0	2,968341496	22,58509888	C1QA;RPL32;RPS8;RPL35A;RPSA;RPS3A;RPL10A;SEPP1;EEF1B2;RPS17;RPS19;ARL6IP5;TPT1
Pirinixic_Acid-364_mg/kg_in_CMC-Rat-Liver-5d-up	13/301	0,000496137	0,011326296	0	0	2,968341496	22,58509888	RPL32;RPS8;RPS5;RPS6;RPL35A;RPSA;RPS3A;RPL10A;EEF1B2;RPS17;RPS19;RPL39;TPT1
Terbutaline-130_mg/kg_in_Corn_Oil-Rat-Heart-1d-up	13/301	0,000496137	0,011293561	0	0	2,968341496	22,58509888	C1QA;RPL32;RPS8;RPS6;RPSA;RPS3A;RPL10A;EEF1B2;RPS17;RPS19;RPS20;RPL39;TPT1
Acetone-4000_mg/kg_in_Water-Rat-Kidney-1d-dn	11/226	0,000497938	0,011301896	0	0	3,345193565	25,44031566	EEF1B2;RPS17;RPL32;RPS19;RPS6;RPL35A;RPS20;RPSA;RPS3A;RPL10A;RPL39
Prednisolone-184_mg/kg_in_Water-Rat-Liver-3d-dn	12/263	0,000499766	0,011310797	0	0	3,13590216	23,83715414	EEF1B2;C1QA;RPS17;RPL32;RPS19;RPS8;RPS6;RPS20;RPS3A;RPL10A;RPL39;TPT1
4-Methylpyrazole-155_mg/kg_in_Saline-Rat-Liver-1d-up	14/341	0,000504667	0,011388982	0	0	2,821698864	21,421244	C1QA;RPL32;RPS8;RPS5;RPS6;RPL35A;RPSA;RPS3A;EEF1B2;RPS17;RPS19;ARL6IP5;RPS20;RPL39
Gentian_Violet-18_mg/kg_in_Corn_Oil-Rat-Liver-3d-dn	14/341	0,000504667	0,011356442	0	0	2,821698864	21,421244	RPL32;RPS8;RPS5;RPS6;RPL35A;RPSA;RPS3A;RPL10A;EEF1B2;RPS17;RPS19;RPS20;RPL39;TPT1
Netilmicin-40_mg/kg_in_Saline-Rat-Kidney-14d-up	14/341	0,000504667	0,011324088	0	0	2,821698864	21,421244	C1QA;RPL32;RPS8;RPS6;RPSA;RPS3A;RPL10A;RPS17;CXCL11;RPS19;ARL6IP5;RPS20;RPL39;TPT1
Carmustine-4_mg/kg_in_Corn_Oil-Rat-Bone_marrow-0.25d-dn	13/302	0,000511837	0,011452355	0	0	2,958512551	22,41814113	RPL32;RPS8;FEM1B;RPS5;RPS6;RPL35A;RPSA;RPS3A;EEF1B2;RPS17;ARL6IP5;RPS20;TPT1
Terfenadine-750_mg/kg_in_CMC-Rat-Heart-5d-up	13/302	0,000511837	0,011419912	0	0	2,958512551	22,41814113	RPL32;RPS8;RPS6;RPSA;RPS3A;RPL10A;EEF1B2;RPS19;ARL2BP;CLP1;RPS20;RAPGEF5;RPL39
Norethindrone-375_mg/kg_in_Corn_Oil-Rat-Liver-0.25d-up	12/264	0,000516908	0,011500463	0	0	3,124023743	23,64150747	EEF1B2;RPS17;RPS19;RPS8;FEM1B;RPS6;RPS20;RPSA;RPS3A;RPL10A;RPL39;GOLGA7
Ethanol-6000_mg/kg_in_Saline-Rat-Kidney-1d-up	14/342	0,00051947	0,011524927	0	0	2,813448283	21,27726725	RPL32;RPS5;RPS6;RPL13A;RPL35A;RPSA;RPS3A;EEF1B2;RPS17;RPS19;ARL6IP5;RPS20;RPL39;TPT1
Allyl_Alcohol-16_mg/kg_in_Saline-Rat-Liver-5d-dn	13/303	0,000527957	0,011680296	0	0	2,948748483	22,25272058	RPL32;RPS8;RPS5;RPS6;RPL35A;RPSA;RPS3A;RPL10A;EEF1B2;RPS17;RPS19;RPS20;RPL39
Carbon_Tetrachloride-400_mg/kg_in_Corn_Oil-Rat-Bone_marrow-1d-up	13/303	0,000527957	0,011647578	0	0	2,948748483	22,25272058	C1QA;RPL32;RPS8;RPS5;RPS6;RPL13A;RPL35A;RPS3A;RPS17;FUNDC1;RPS20;RPL39;TPT1

Megestrol_Acetate-132_mg/kg_in_CMC-Rat-Liver-5d-dn	13/303	0,000527957	0,011615043	0	0	2,948748483	22,25272058	RPL32;RPS8;RPS5;RPS6;RPL35A;RPS3A;RPL10A;SEPP1;EEF1B2;RPS17;RPS19;RPS20;TPT1
Simvastatin-160_uM_in_DMSO-Rat-Primary_rat_hepatocytes-0.67d-dn	13/303	0,000527957	0,011582689	0	0	2,948748483	22,25272058	RPS8;RPS5;RPSA;RPS3A;RPL10A;SEPP1;RPS17;CXCL11;RPS19;ARL6IP5;RPS20;GOLGA7;SLC16A3
Citalopram-90_mg/kg_in_Corn_Oil-Rat-Heart-3d-up	14/343	0,000534642	0,011696785	0	0	2,80524581	21,134477	RPS8;RPS6;RPSA;RPS3A;RPL10A;SEPP1;EEF1B2;RPS19;FUNDC1;ARL6IP5;RPS20;RAPGEF5;RPL39;TPT1
Ergocalciferol-15_mg/kg_in_CMC-Rat-Heart-3d-dn	14/343	0,000534642	0,011664384	0	0	2,80524581	21,134477	RPL32;RPS8;RPS6;RPS3A;RPL10A;SEPP1;RPS17;RPS19;ARL2BP;FUNDC1;ARL6IP5;RPS20;RPL39;TPT1
Prednisolone-184_mg/kg_in_Water-Rat-Heart-5d-dn	14/343	0,000534642	0,011632162	0	0	2,80524581	21,134477	RPL32;RPS8;RPS6;RPSA;RPS3A;RPL10A;SEPP1;RPS17;RPS19;ARL2BP;FUNDC1;RPS20;RPL39;TPT1
4-Nitrobenzoic_Acid-1470_mg/kg_in_CMC-Rat-Kidney-3d-up	13/304	0,000544504	0,011814081	0	0	2,939048653	22,08881914	RPS8;RPS5;RPS6;RPSA;RPS3A;RPL10A;SEPP1;EEF1B2;RPS17;RPS19;RPS20;RPL39;TPT1
Acetaminophen-486_mg/kg_in_Corn_Oil-Rat-Liver-3d-up	13/304	0,000544504	0,011781625	0	0	2,939048653	22,08881914	RPL32;RPS8;RPS5;RPS6;RPSA;RPS3A;RPL10A;SEPP1;RPS17;RPS19;ARL6IP5;RPS20;RPL39
Amiodarone-147_mg/kg_in_CMC-Rat-Liver-1d-dn	13/304	0,000544504	0,011749346	0	0	2,939048653	22,08881914	RPL32;RPS8;FEM1B;RPS6;RPL35A;RPS3A;RPL10A;SEPP1;EEF1B2;RPS17;RPS19;RPS20;RPL39
Clomiphene-250_mg/kg_in_CMC-Rat-Liver-1d-up	13/304	0,000544504	0,011717244	0	0	2,939048653	22,08881914	C1QA;RPL32;RPS8;RPS6;RPSA;RPS3A;RPL10A;PDRG1;EEF1B2;RPS17;RPS19;RPL39;TPT1
Daunorubicin-3.25_mg/kg_in_Saline-Rat-Bone_marrow-1d-dn	13/304	0,000544504	0,011685317	0	0	2,939048653	22,08881914	RPL32;RPS8;RPS5;RPS6;RPSA;RPS3A;RPL10A;EEF1B2;RPS17;FUNDC1;RPS20;RPL39;TPT1
Dobutamine-43_mg/kg_in_Saline-Rat-Heart-5d-up	13/304	0,000544504	0,011653564	0	0	2,939048653	22,08881914	RPL32;RPS5;RPS6;RPL35A;RPSA;RPS3A;RPL10A;SEPP1;EEF1B2;RPS17;RPS19;RPL39;TPT1
Fluconazole-394_mg/kg_in_Corn_Oil-Rat-Liver-5d-up	13/304	0,000544504	0,011621982	0	0	2,939048653	22,08881914	RPL32;RPS8;RPS5;RPS6;RPSA;RPS3A;RPL10A;RPS19;ARL2BP;RPS20;RPL39;GOLGA7;TPT1
Granisetron-175_mg/kg_in_Water-Rat-Liver-1d-up	13/304	0,000544504	0,011590571	0	0	2,939048653	22,08881914	RPL32;RPS8;RPS5;RPS6;RPL35A;RPSA;RPS3A;RPL10A;RPS17;RPS19;RPS20;RPL39;TPT1
SCH-351591-4.6_mg/kg_in_CMC-Rat-Liver-4d-up	13/304	0,000544504	0,01155933	0	0	2,939048653	22,08881914	RPL32;RPS8;RPS6;RPL35A;RPSA;RPS3A;RPL10A;EEF1B2;RPS17;RPS19;ARL6IP5;RPS20;RPL39
Indomethacin-12_mg/kg_in_Water-Rat-Liver-5d-up	14/344	0,00055019	0,011648637	0	0	2,797091025	20,99286084	RPL32;RPS8;RPS5;RPS6;RPSA;RPS3A;RPL10A;EEF1B2;RPS17;RPS19;ARL2BP;RPS20;RPL39;GOLGA7
Bezafibrate-100_mg/kg_in_Corn_Oil-Rat-Kidney-0.25d-dn	12/266	0,000552679	0,011669972	0	0	3,100534842	23,25628473	RPS17;RPL32;ARL2BP;RPS8;RPS5;RPS6;ARL6IP5;RPL35A;RPSA;SEPP1;TOMM20;TPT1
Procainamide-755_mg/kg_in_Water-Rat-Heart-1d-up	13/305	0,000561488	0,011824273	0	0	2,929412427	21,92641894	RPL32;RPS8;RPS5;RPL35A;RPSA;RPS3A;SEPP1;EEF1B2;RPS17;RPS19;ARL6IP5;RPS20;TPT1
Atorvastatin-250_uM_in_DMSO-Rat-Primary_rat_hepatocytes-1d-dn	14/345	0,00056612	0,011890026	0	0	2,788983515	20,85240652	RPL32;RPS8;RPS5;RPSA;RPL10A;SEPP1;EEF1B2;RPS17;CXCL11;RPS19;ARL6IP5;RPL39;SLC16A3;TPT1
NN-Dimethylformamide-140_mg/kg_in_Saline-Rat-Spleen-3d-dn	14/345	0,00056612	0,011858403	0	0	2,788983515	20,85240652	C1QA;RPL32;RPS8;RPS6;RPSA;RPS3A;RPL10A;EEF1B2;RPS17;ARL2BP;ARL6IP5;RPS20;RPL39;TPT1
Neostigmine_Bromide-40_mg/kg_in_Water-Rat-Liver-5d-dn	14/345	0,00056612	0,011826949	0	0	2,788983515	20,85240652	C1QA;RPL32;RPS8;RPL35A;RPSA;RPS3A;RPL10A;SEPP1;EEF1B2;RPS17;RPS19;RPS20;RPL39;TPT1
Bithionol-333_mg/kg_in_Corn_Oil-Rat-Bone_marrow-5d-up	13/306	0,000578918	0,012062323	0	0	2,919839184	21,76550242	C1QA;RPL32;FEM1B;RPS5;NKG7;RPL35A;RPSA;RPL10A;SEPP1;EEF1B2;RPS17;RPS20;TPT1
Primidone-750_mg/kg_in_CMC-Rat-Liver-5d-dn	13/306	0,000578918	0,012030496	0	0	2,919839184	21,76550242	C1QA;RPL32;RPS6;RPL35A;RPS3A;SEPP1;EEF1B2;RPS17;CLP1;ARL6IP5;RPS20;RPL39;TPT1
Sotalol-2000_mg/kg_in_Water-Rat-Heart-3d-up	13/306	0,000578918	0,011998837	0	0	2,919839184	21,76550242	RPS8;RPS6;RPSA;RPS3A;RPL10A;RPS17;RPS19;FUNDC1;ARL6IP5;RPS20;RAPGEF5;GOLGA7;TPT1
Sulindac-23_mg/kg_in_Corn_Oil-Rat-Kidney-3d-dn	14/346	0,000582441	0,012040161	0	0	2,780922869	20,71310192	RPL32;RPS8;RPS6;RPSA;RPS3A;RPL10A;SEPP1;TOMM20;EEF1B2;RPS17;RPS19;RPS20;RPL39;TPT1
Phenobarbital-80_mg/kg_in_Water-Rat-Liver-3d-dn	12/268	0,00059051	0,012175023	0	0	3,077396523	22,87897691	EEF1B2;C1QA;RPS17;RPL32;RPS19;RPS6;RPS20;RPSA;RPS3A;RPL10A;RPL39;TPT1
Chlordiazepoxide-196_mg/kg_in_Water-Rat-Heart-3d-up	13/307	0,000596803	0,012272648	0	0	2,910328307	21,60605224	RPL32;RPS8;RPS5;RPS6;RPL35A;RPSA;RPS3A;RPS17;RPS19;ARL2BP;FUNDC1;ARL6IP5;GOLGA7
Doxorubicin-3_mg/kg_in_Saline-Rat-Liver-1d-dn	13/307	0,000596803	0,012240688	0	0	2,910328307	21,60605224	C1QA;RPL32;RPS6;RPL35A;RPSA;RPS3A;RPL10A;EEF1B2;RPS17;RPS19;CLP1;RPL39;TPT1
Etoposide-188_mg/kg_in_Corn_Oil-Rat-Spleen-1d-dn	13/307	0,000596803	0,012208894	0	0	2,910328307	21,60605224	C1QA;RPL32;RPS8;RPS6;RPL35A;RPSA;RPS3A;RPL10A;EEF1B2;RPS17;RPS20;RPL39;TPT1
Melphalan-12_mg/kg_in_CMC-Rat-Bone_marrow-5d-dn	13/307	0,000596803	0,012177265	0	0	2,910328307	21,60605224	RPL32;RPS8;FEM1B;RPS5;RPS6;NKG7;RPSA;RPL10A;RPS17;RPS19;RPS20;RPL39;TPT1
Sildenafil-14.6_mg/kg_in_Saline-Rat-Liver-5d-dn	12/269	0,000610227	0,012418991	0	0	3,065956387	22,69322558	EEF1B2;C1QA;RPS17;RPL32;RPS19;RPS8;RPS6;RPSA;RPS3A;RPL10A;RPL39;TPT1
Buflomedil-615_mg/kg_in_CMC-Rat-Heart-1d-dn	13/308	0,000615154	0,012486988	0	0	2,90087919	21,44805133	C1QA;RPL32;RPS8;RPS6;RPSA;RPS3A;RPL10A;SEPP1;RPS17;RPS19;ARL2BP;RPS20;RPL39
Ethylene_Glycol-3525_mg/kg_in_Water-Rat-Bone_marrow-1d-dn	13/308	0,000615154	0,012454888	0	0	2,90087919	21,44805133	C1QA;RPL32;RPS8;RPS6;RPSA;RPS3A;RPL10A;SEPP1;RPS19;ARL6IP5;RPS20;RPL39;TPT1
Isoniazid-50_mg/kg_in_Saline-Rat-Liver-5d-dn	13/308	0,000615154	0,012422952	0	0	2,90087919	21,44805133	RPL32;RPS8;RPS5;RPS6;RPL35A;RPSA;RPS3A;RPL10A;EEF1B2;RPS17;RPS19;RPS20;RPL39
NN-Dimethylformamide-1400_mg/kg_in_Saline-Rat-Bone_marrow-1d-dn	13/308	0,000615154	0,01239118	0	0	2,90087919	21,44805133	RPL32;RPS8;FEM1B;RPS5;RPS6;RPSA;RPL10A;EEF1B2;RPS17;RPS19;RPS20;RPL39;TPT1
Phenytoin-46_mg/kg_in_Water-Rat-Heart-3d-up	13/308	0,000615154	0,01235957	0	0	2,90087919	21,44805133	RPL32;FEM1B;RPS6;RPL35A;RPSA;RPS3A;RPL10A;RPS17;RPS19;COL5A3;ARL6IP5;RPL39;TPT1
6-Methoxy-2-Naphthylacetic_Acid-360_mg/kg_in_Saline-Rat-Kidney-1d-up	14/348	0,000616284	0,012350773	0	0	2,764940554	20,4378943	RPL32;RPS8;RPS5;RPS6;RPL13A;RPL35A;RPSA;RPS3A;SEPP1;TOMM20;RPS17;RPS19;ARL6IP5;RPS20
Diethylstilbestrol-120_uM_in_DMSO-Rat-Primary_rat_hepatocytes-1d-dn	14/349	0,000633823	0,01267002	0	0	2,757018088	20,30196786	RPL32;RPS8;RPS5;RPS6;RPL35A;RPSA;RPL10A;SEPP1;RPS17;CXCL11;RPS19;ARL6IP5;RPL39;TPT1
Leflunomide-30_mg/kg_in_Corn_Oil-Rat-Bone_marrow-3d-up	13/309	0,000633978	0,012641049	0	0	2,891491231	21,29148287	C1QA;RPL32;RPS8;RPS5;NKG7;RPL35A;RPS3A;SEPP1;EEF1B2;RPS17;RPS20;RPL39;TPT1
Allyl_Alcohol-16_mg/kg_in_Saline-Rat-Bone_marrow-3d-up	15/391	0,000649396	0,012915772	0	0	2,636644079	19,35156298	C1QA;RPS8;FEM1B;RPS5;RPS6;RPL35A;RPSA;RPS3A;RPL10A;EEF1B2;RPS17;RPS19;RPS20;RPL39;TPT1
Labetalol-250_uM_in_DMSO-Rat-Primary_rat_hepatocytes-1d-up	12/271	0,000651323	0,012921462	0	0	3,043329402	22,32740082	EEF1B2;RPS17;RPL32;ARL2BP;RPS8;RPS5;RPS6;RPL35A;RPS20;RPS3A;RPL10A;PDRG1
Oxybutynin-230_mg/kg_in_Water-Rat-Heart-3d-up	14/350	0,000651783	0,012898099	0	0	2,749140893	20,16714431	RPL32;RPS5;RPS6;RPL35A;RPSA;RPS3A;RPL10A;SEPP1;TOMM20;RPS17;RPS19;ARL6IP5;RPS20;TPT1
Beta-Estradiol-150_mg/kg_in_Corn_Oil-Rat-Liver-1d-up	13/310	0,000653287	0,012895464	0	0	2,88216384	21,13633027	RPL32;RPS8;FEM1B;RPS5;RPS6;RPSA;RPS3A;RPL10A;EEF1B2;RPS17;RPS19;RPS20;RPL39
Carbon_Tetrachloride-3178_mg/kg_in_Saline-Rat-Liver-5d-up	13/310	0,000653287	0,012863226	0	0	2,88216384	21,13633027	C1QA;RPL32;RPS8;RPS6;RPSA;RPS3A;RPL10A;EEF1B2;RPS17;RPS19;RPS20;RPL39;TPT1
Fludrocortisone_Acetate-125_mg/kg_in_CMC-Rat-Liver-5d-dn	13/310	0,000653287	0,012831148	0	0	2,88216384	21,13633027	C1QA;RPL32;RPS8;RPS5;RPS6;RPL35A;RPS3A;RPL10A;SEPP1;EEF1B2;RPS17;RPS19;TPT1
NN'-Diphenyl-P-Phenylenediamine-1000_mg/kg_in_CMC-Rat-Kidney-1d-dn	13/310	0,000653287	0,012799229	0	0	2,88216384	21,13633027	RPL32;RPS8;RPS6;RPSA;RPL10A;SEPP1;TOMM20;PDRG1;RPS19;ARL2BP;ARL6IP5;RPS20;RPL39
Lead_(II)_Acetate-600_mg/kg_in_Saline-Rat-Bone_marrow-1d-dn	12/272	0,000672726	0,013147373	0	0	3,032140691	22,14727774	EEF1B2;RPS17;RPL32;RPS19;RPS5;NKG7;RPSA;RPS3A;RPL10A;RPL39;SEPP1;TPT1
Azauridine-1500_mg/kg_in_CMC-Rat-Bone_marrow-3d-up	13/311	0,00067309	0,013121922	0	0	2,872896432	20,9825772	C1QA;RPL32;RPS8;FEM1B;RPS6;RPSA;RPS3A;SEPP1;RPS17;RPS19;ARL2BP;RPS20;RPL39
Dexamethasone-1_mg/kg_in_Corn_Oil-Rat-Heart-5d-dn	13/311	0,00067309	0,013089522	0	0	2,872896432	20,9825772	RPL32;RPS8;RPS6;RPSA;RPS3A;RPL10A;RPS17;RPS19;ARL2BP;COL5A3;RPL39;GOLGA7;TPT1
Betamethasone-79_mg/kg_in_Corn_Oil-Rat-Liver-5d-dn	13/312	0,000693397	0,01345121	0	0	2,863688431	20,83020754	C1QA;RPL32;RPS8;RPS6;RPL35A;RPS3A;RPL10A;SEPP1;EEF1B2;RPS17;RPS19;RPS20;RPL39
Indomethacin-9.6_mg/kg_in_Water-Rat-Liver-0.25d-dn	12/273	0,000694716	0,013443691	0	0	3,021033949	21,96898179	EEF1B2;C1QA;RPS17;RPL32;RPS8;RPS5;RPS6;RPL35A;RPSA;RPL39;SEPP1;TPT1
Lead_(II)_Acetate-600_mg/kg_in_Saline-Rat-Liver-3d-up	12/273	0,000694716	0,013410741	0	0	3,021033949	21,96898179	EEF1B2;RPS17;RPL32;RPS8;RPS5;RPS6;RPL35A;RPS20;RPSA;RPL10A;TOMM20;TPT1
Methotrexate-27_mg/kg_in_Corn_Oil-Rat-Kidney-3d-up	12/273	0,000694716	0,013377952	0	0	3,021033949	21,96898179	RPS17;RPL32;RPS8;RPS5;RPS6;RPL35A;RPS20;RPSA;RPS3A;RAPGEF5;RPL10A;TPT1
Allyl_Alcohol-16_mg/kg_in_Saline-Rat-Bone_marrow-1d-dn	11/236	0,000713415	0,013704535	0	0	3,203448075	23,21041246	EEF1B2;RPS17;RPL32;RPS8;RPS5;RPS6;RPL13A;RPL35A;RPS20;RPSA;TPT1
Oxaliplatin-8_mg/kg_in_Saline-Rat-Kidney-1d-dn	11/236	0,000713415	0,013671191	0	0	3,203448075	23,21041246	EEF1B2;RPS17;RPL32;RPS19;RPL35A;RPS20;RPSA;RPS3A;RPL10A;RPL39;TPT1
Sulfaphenazole-1695_mg/kg_in_Water-Rat-Kidney-1d-dn	11/236	0,000713415	0,013638008	0	0	3,203448075	23,21041246	RPS17;RPL32;RPS19;ARL2BP;RPS6;ARL6IP5;RPL35A;RPS20;RPSA;RPS3A;RPL39
Levamisole-120_mg/kg_in_Water-Rat-Liver-5d-up	13/313	0,000714217	0,013620279	0	0	2,854539266	20,67920544	RPL32;RPS8;RPS5;RPS6;RPL13A;RPL35A;RPSA;RPS3A;RPL10A;SEPP1;RPS17;RPS19;RPS20
Thiabendazole-92_mg/kg_in_Corn_Oil-Rat-Liver-3d-up	13/313	0,000714217	0,01358738	0	0	2,854539266	20,67920544	RPL32;RPS6;RPL35A;RPS3A;RPL10A;SEPP1;EEF1B2;RPS17;RPS19;CLP1;COL5A3;RPS20;RPL39
Amantadine-220_mg/kg_in_Water-Rat-Liver-1d-up	13/314	0,000735562	0,013959732	0	0	2,845448377	20,52955523	C1QA;RPL32;RPS8;FEM1B;RPS6;RPL35A;RPSA;RPS3A;RPS17;RPS19;RPS20;RPL39;TPT1
Cefotaxime-1000_mg/kg_in_Saline-Rat-Kidney-5d-up	13/314	0,000735562	0,013926175	0	0	2,845448377	20,52955523	RPL32;RPS8;RPS5;RPL13A;RPL35A;RPSA;TOMM20;RPS17;CXCL11;ARL6IP5;RPS20;RPL39;TPT1
Nitrazepam-310_mg/kg_in_CMC-Rat-Liver-1d-dn	13/314	0,000735562	0,013892779	0	0	2,845448377	20,52955523	C1QA;RPL32;RPL35A;RPSA;RPL10A;SEPP1;EEF1B2;RPS19;CLP1;ARL6IP5;RPS20;RPL39;TPT1
Gatifloxacin-770_mg/kg_in_Corn_Oil-Rat-Heart-1d-dn	13/315	0,000757442	0,014271804	0	0	2,836415208	20,38124151	RPL32;RPS8;RPS5;RPS6;RPL35A;RPSA;RPS3A;EEF1B2;RPS17;ARL2BP;RPS20;RPL39;TPT1
Itraconazole-30_mg/kg_in_Corn_Oil-Rat-Liver-1d-up	13/315	0,000757442	0,014237742	0	0	2,836415208	20,38124151	RPL32;RPS8;RPS5;RPS6;RPSA;RPS3A;RPL10A;EEF1B2;RPS17;RPS19;RPS20;RPL39;TPT1
Loratadine-10_mg/kg_in_Water-Rat-Heart-3d-up	13/315	0,000757442	0,014203843	0	0	2,836415208	20,38124151	RPL32;RPS8;RPS5;RPS6;RPL35A;RPSA;RPS3A;RPS17;RPS19;ARL2BP;RPS20;RAPGEF5;GOLGA7
NN-Dimethylformamide-140_mg/kg_in_Saline-Rat-Kidney-1d-up	13/315	0,000757442	0,014170104	0	0	2,836415208	20,38124151	RPL32;RPS8;RPS5;RPS6;RPL13A;RPL35A;RPSA;RPS3A;RPS17;RPS19;RPS20;RPL39;TPT1
Atorvastatin-300_mg/kg_in_Corn_Oil-Rat-Liver-5d-up	12/276	0,000764331	0,014265103	0	0	2,988196623	21,44482046	EEF1B2;RPS17;CXADR;RPS19;RPS8;RPS5;RPS6;RPS20;RPSA;RPS3A;RPL10A;RPL39
6-Mercaptopurine-25_mg/kg_in_Corn_Oil-Rat-Kidney-3d-dn	13/316	0,000779867	0,014520647	0	0	2,82743921	20,23424906	RPL32;RPS8;RPS6;RPSA;RPS3A;SEPP1;EEF1B2;RPS17;RPS19;ARL2BP;RPS20;RPL39;TPT1
Bithionol-333_mg/kg_in_Corn_Oil-Rat-Bone_marrow-1d-up	13/316	0,000779867	0,014486401	0	0	2,82743921	20,23424906	C1QA;RPL32;RPS5;NKG7;RPSA;RPL10A;SEPP1;EEF1B2;RPS17;RPS19;RPS20;RPL39;TPT1
N-Nitrosodiethylamine-7000_uM_in_DMSO-Rat-Primary_rat_hepatocytes-0.67d-up	13/316	0,000779867	0,014452315	0	0	2,82743921	20,23424906	RPL32;RPS8;FEM1B;RPS5;RPL35A;RPSA;PDRG1;EEF1B2;CXCL11;CLP1;RPS20;RPL39;GOLGA7
Spironolactone-300_mg/kg_in_CMC-Rat-Liver-5d-dn	13/316	0,000779867	0,014418389	0	0	2,82743921	20,23424906	C1QA;RPL32;RPS5;RPS6;RPL35A;RPSA;RPS3A;EEF1B2;RPS17;RPS19;RPS20;RPL39;TPT1
Thioridazine-63_mg/kg_in_Corn_Oil-Rat-Heart-3d-dn	13/316	0,000779867	0,014384623	0	0	2,82743921	20,23424906	RPL32;RPS8;RPS6;RPSA;RPS3A;EEF1B2;RPS17;RPS19;CLP1;R3HCC1;ARL6IP5;RPL39;TPT1
Indomethacin-12_mg/kg_in_Water-Rat-Liver-3d-up	12/277	0,000788795	0,014515303	0	0	2,97740891	21,2735987	EEF1B2;RPS17;RPL32;RPS19;RPS8;RPS5;RPS6;RPS20;RPSA;RPS3A;RPL10A;RPL39
Zomepirac-2.8_mg/kg_in_Saline-Rat-Liver-3d-up	12/277	0,000788795	0,014481468	0	0	2,97740891	21,2735987	EEF1B2;RPS17;CXADR;RPL32;RPS19;RPS8;RPS5;RPL35A;RPS20;RPSA;RPS3A;TOMM20
2-Amino-4-Nitrophenol-625_mg/kg_in_CMC-Rat-Kidney-5d-up	13/317	0,000802848	0,014705192	0	0	2,818519843	20,08856291	C1QA;RPL32;RPS8;RPS6;RPSA;RPS3A;RPL10A;SEPP1;EEF1B2;RPS17;RPS19;RPL39;TPT1
Cholecalciferol-8_mg/kg_in_CMC-Rat-Heart-3d-dn	13/317	0,000802848	0,014671074	0	0	2,818519843	20,08856291	RPL32;RPS8;RPS6;RPSA;RPS3A;SEPP1;RPS17;RPS19;FUNDC1;ARL6IP5;RPS20;RPL39;TPT1
Sodium_Arsenite-30_mg/kg_in_Saline-Rat-Kidney-1d-up	13/317	0,000802848	0,014637113	0	0	2,818519843	20,08856291	RPL32;RPS8;RPS5;RPS6;RPL13A;RPL35A;RPS3A;SEPP1;EEF1B2;RPS17;RPS19;ARL6IP5;RPS20
Tolazamide-1500_mg/kg_in_CMC-Rat-Liver-1d-up	13/317	0,000802848	0,014603309	0	0	2,818519843	20,08856291	RPL32;RPS8;RPS5;RPS6;RPL35A;RPSA;RPS3A;RPL10A;EEF1B2;RPS17;RPS19;RPS20;TPT1
Amiloride-22_mg/kg_in_CMC-Rat-Heart-5d-up	12/278	0,00081391	0,014770404	0	0	2,966698806	21,10408881	EEF1B2;RPS17;RPL32;ARL2BP;RPS8;RPS5;FUNDC1;RPS6;RPL35A;RPS3A;TOMM20;TPT1
Naproxen-134_mg/kg_in_Corn_Oil-Rat-Kidney-3d-up	12/278	0,00081391	0,014736449	0	0	2,966698806	21,10408881	EEF1B2;C1QA;RPS17;RPL32;RPS8;RPS6;RPL13A;RPL35A;RPS20;RPS3A;RAPGEF5;RPL39
Carmustine-4_mg/kg_in_Corn_Oil-Rat-Spleen-0.25d-up	13/318	0,000826396	0,014928202	0	0	2,809656574	19,94416827	RPL32;RPS8;RPS6;RPL13A;RPL35A;RPSA;RPS3A;RPL10A;RPS17;FUNDC1;ARL6IP5;RPS20;RPL39
Cholecalciferol-8_mg/kg_in_CMC-Rat-Liver-1d-up	13/318	0,000826396	0,014894041	0	0	2,809656574	19,94416827	C1QA;RPL32;RPS8;RPS5;RPS6;RPL35A;RPS3A;RPL10A;EEF1B2;RPS17;RPS19;RPS20;TPT1
Letrozole-250_mg/kg_in_Corn_Oil-Rat-Liver-1d-dn	13/318	0,000826396	0,014860036	0	0	2,809656574	19,94416827	RPL32;RPS8;RPS6;RPL35A;RPSA;RPS3A;RPL10A;EEF1B2;RPS17;RPS19;RPS20;RPL39;TPT1
Lomustine-4.2_mg/kg_in_Corn_Oil-Rat-Bone_marrow-3d-up	13/318	0,000826396	0,014826187	0	0	2,809656574	19,94416827	C1QA;RPL32;RPS8;RPS5;RPL13A;RPL35A;RPS3A;SEPP1;EEF1B2;RPS17;RPS20;RPL39;TPT1
Methapyrilene-100_mg/kg_in_CMC-Rat-Liver-3d-up	13/318	0,000826396	0,014792491	0	0	2,809656574	19,94416827	RPL32;RPS8;RPS5;RPS6;RPSA;RPS3A;RPL10A;SEPP1;EEF1B2;RPS17;RPS19;RPS20;RPL39
N-Nitrosodiethylamine-34_mg/kg_in_Saline-Rat-Kidney-1d-up	13/318	0,000826396	0,014758948	0	0	2,809656574	19,94416827	RPL32;RPS8;RPS5;RPS6;RPL13A;RPL35A;RPSA;RPS3A;SEPP1;TOMM20;EEF1B2;RPS17;RPS20
Verapamil-108_mg/kg_in_Water-Rat-Heart-1d-up	13/318	0,000826396	0,014725556	0	0	2,809656574	19,94416827	RPL32;RPS8;RPS5;RPS6;RPL35A;RPSA;RPS3A;RPL10A;RPS17;RPS19;ARL6IP5;RPS20;TPT1
Haloperidol-21_mg/kg_in_Corn_Oil-Rat-Heart-5d-up	14/359	0,000833779	0,01482358	0	0	2,680220927	19,00153833	RPL32;RPS8;RPS3A;RPL10A;SEPP1;EEF1B2;RPS19;ARL2BP;FUNDC1;ARL6IP5;RPS20;RAPGEF5;RPL39;TPT1
Dicumarol-13_mg/kg_in_Corn_Oil-Rat-Liver-1d-dn	12/279	0,00083969	0,014895038	0	0	2,956065477	20,93626884	EEF1B2;RPL32;RPS19;RPS8;RPS5;RPS6;RPL35A;RPSA;RPS3A;RPL10A;RPL39;GOLGA7
Amitraz-75_mg/kg_in_CMC-Rat-Heart-1d-dn	13/319	0,000850522	0,015053281	0	0	2,800848873	19,80105058	C1QA;RPL32;RPS8;FEM1B;RPSA;RPS3A;RPL10A;EEF1B2;RPS17;RPS19;ARL2BP;RPS20;RPL39
Ethosuximide-1200_mg/kg_in_Water-Rat-Heart-3d-dn	13/319	0,000850522	0,015019529	0	0	2,800848873	19,80105058	RPL32;RPS8;RPS6;RPSA;RPS3A;RPL10A;SEPP1;EEF1B2;RPS17;RPS19;RPS20;RPL39;TPT1
Sulfinpyrazone-269_mg/kg_in_CMC-Rat-Kidney-5d-up	13/319	0,000850522	0,014985928	0	0	2,800848873	19,80105058	RPL32;RPS6;RPSA;RPL10A;SEPP1;RPS17;CXCL11;RPS19;ARL6IP5;RPS20;RAPGEF5;RPL39;GOLGA7

Valproic_Acid-850_mg/kg_in_Water-Rat-Bone_marrow-5d-up	13/319	0,000850522	0,014952478	0	0	2,800848873	19,80105058	RPL32;RPS5;NKG7;RPL35A;RPS3A;RPL10A;SEPP1;EEF1B2;RPS17;RPS19;RPS20;RPL39;TPT1
Cadmium_Acetate-167_mg/kg_in_Water-Rat-Kidney-5d-up	12/280	0,000866148	0,015193277	0	0	2,9455081	20,77011718	RPS17;RPL32;ARL2BP;RPS6;RPS20;RPSA;RPS3A;RAPGEF5;RPL10A;RPL39;SEPP1;TPT1
Carbon_Tetrachloride-400_mg/kg_in_Corn_Oil-Rat-Liver-3d-up	12/280	0,000866148	0,015159514	0	0	2,9455081	20,77011718	EEF1B2;C1QA;RPS17;RPL32;RPS19;RPS8;RPS5;RPS20;RPSA;RPS3A;RPL39;SEPP1
Nystatin-134_mg/kg_in_Corn_Oil-Rat-Liver-1d-up	12/280	0,000866148	0,015125901	0	0	2,9455081	20,77011718	EEF1B2;C1QA;RPS17;RPL32;RPS19;ARL2BP;RPS8;RPS20;RPSA;RPL39;SEPP1;TPT1
BW-723C86-50_mg/kg_in_Saline-Rat-Heart-5d-up	13/320	0,000875236	0,015250802	0	0	2,79209622	19,65919548	C1QA;RPS8;RPS6;RPS3A;RPL10A;SEPP1;EEF1B2;RPS17;RPS19;ARL2BP;ARL6IP5;RPS20;RPL39
Cobalt(II)_Acetylacetonate-100_mg/kg_in_Corn_Oil-Rat-Kidney-5d-up	13/320	0,000875236	0,015217135	0	0	2,79209622	19,65919548	RPL32;RPS8;RPS5;RPS6;RPL35A;RPSA;RPS3A;PDRG1;EEF1B2;RPS17;ARL6IP5;RPS20;RPL39
Dicyclomine-645_mg/kg_in_Water-Rat-Heart-1d-dn	13/320	0,000875236	0,015183617	0	0	2,79209622	19,65919548	RPL32;RPS8;RPS6;RPSA;RPS3A;RPL10A;EEF1B2;RPS17;RPS19;ARL2BP;FUNDC1;RPS20;RPL39
Erythromycin-1500_mg/kg_in_CMC-Rat-Liver-5d-up	13/320	0,000875236	0,015150247	0	0	2,79209622	19,65919548	C1QA;RPL32;RPS8;RPS5;RPS6;RPSA;RPS3A;RPL10A;EEF1B2;RPS19;ARL2BP;RPS20;TPT1
Kanamycin-6584_uM_in_DMSO-Rat-Primary_rat_hepatocytes-0.67d-up	13/320	0,000875236	0,015117023	0	0	2,79209622	19,65919548	RPL32;FEM1B;RPS6;RPL35A;RPS3A;RPL10A;EEF1B2;RPS17;CXCL11;CLP1;RPS20;RPL39;TPT1
Sulindac-750_uM_in_DMSO-Rat-Primary_rat_hepatocytes-0.67d-dn	13/320	0,000875236	0,015083944	0	0	2,79209622	19,65919548	RPL32;RPS8;RPS5;RPSA;RPL10A;SEPP1;CXCL11;RPS19;FUNDC1;ARL6IP5;RPS20;GOLGA7;TPT1
Sumatriptan-1100_mg/kg_in_Water-Rat-Brain-3d-up	13/320	0,000875236	0,015051009	0	0	2,79209622	19,65919548	C1QA;RPL32;RPS8;FEM1B;RPL35A;RPS3A;RPL10A;SEPP1;EEF1B2;RPS17;RPS19;RPL39;TPT1
Tegafur-175_mg/kg_in_Water-Rat-Bone_marrow-1d-dn	13/320	0,000875236	0,015018219	0	0	2,79209622	19,65919548	C1QA;RPL32;RPS8;RPS5;RPL35A;RPSA;RPS3A;EEF1B2;RPS17;ARL6IP5;RPS20;RPL39;TPT1
Netilmicin-41_mg/kg_in_Saline-Rat-Kidney-5d-up	14/361	0,00087958	0,015059938	0	0	2,665372057	18,75373411	RPL32;RPS5;RPS6;RPL35A;RPSA;SEPP1;TOMM20;RPS17;RPS19;CLP1;FUNDC1;ARL6IP5;RAPGEF5;TPT1
Enalapril-1500_mg/kg_in_CMC-Rat-Kidney-1d-up	12/281	0,000893298	0,015261646	0	0	2,935025865	20,6056126	EEF1B2;RPL32;RPS19;ARL2BP;RPS8;RPS6;RPS20;RPSA;RPS3A;RPL10A;RPL39;TPT1
Mifepristone-3_mg/kg_in_Corn_Oil-Rat-Liver-3d-up	12/281	0,000893298	0,015228612	0	0	2,935025865	20,6056126	EEF1B2;RPS17;RPL32;RPS19;RPS8;RPS5;RPL35A;RPS20;RPS3A;RPL39;SEPP1;TPT1
Nimesulide-162_mg/kg_in_Corn_Oil-Rat-Liver-1d-up	12/281	0,000893298	0,015195721	0	0	2,935025865	20,6056126	EEF1B2;RPS17;RPS19;RPS8;RPS5;RPS20;RPSA;RPS3A;RPL10A;RPL39;SEPP1;TPT1
Roxarsone-11_mg/kg_in_Corn_Oil-Rat-Kidney-1d-up	12/281	0,000893298	0,015162972	0	0	2,935025865	20,6056126	RPS17;RPL32;RPS19;RPS8;RPS5;RPS6;RPL35A;RPSA;RPS3A;RPL10A;RPL39;TPT1
Ascorbic_Acid-2000_mg/kg_in_Water-Rat-Kidney-1d-dn	13/321	0,000900551	0,015253208	0	0	2,783398101	19,51858881	RPL32;RPL35A;RPSA;RPS3A;SEPP1;TOMM20;EEF1B2;ARL2BP;FUNDC1;ARL6IP5;RPS20;RPL39;TPT1
Cholic_Acid-1402_mg/kg_in_Water-Rat-Liver-5d-up	13/321	0,000900551	0,015220476	0	0	2,783398101	19,51858881	RPL32;RPS8;RPS6;RPL35A;RPS3A;RPL10A;SEPP1;EEF1B2;RPS17;RPS19;ARL6IP5;RPS20;RPL39
Rapamycin-60_mg/kg_in_CMC-Rat-Kidney-3d-up	13/321	0,000900551	0,015187884	0	0	2,783398101	19,51858881	RPS8;RPS5;RPS6;RPL13A;RPSA;RPS3A;EEF1B2;RPS17;RPS19;ARL2BP;RPS20;RPL39;TPT1
Clofibrate-130_mg/kg_in_Corn_Oil-Rat-Kidney-0.25d-dn	12/282	0,000921155	0,015502178	0	0	2,924617972	20,44273419	EEF1B2;RPS17;RPL32;ARL2BP;RPS8;RPS5;RPS6;ARL6IP5;RPL35A;RPSA;TOMM20;TPT1
Noscapine-570_mg/kg_in_CMC-Rat-Bone_marrow-1d-dn	12/282	0,000921155	0,015469124	0	0	2,924617972	20,44273419	RPS17;RPL32;RPS19;ARL2BP;RPS8;FEM1B;RPS5;FUNDC1;RPS6;RPL35A;RPS20;RPS3A
Pramoxine-526_mg/kg_in_Corn_Oil-Rat-Liver-1d-up	12/282	0,000921155	0,015436211	0	0	2,924617972	20,44273419	EEF1B2;C1QA;RPS17;RPL32;RPS19;ARL2BP;RPS8;RPS20;RPSA;RPL10A;RPL39;SEPP1
Vinblastine-45_uM_in_DMSO-Rat-Primary_rat_hepatocytes-1d-up	12/282	0,000921155	0,015403438	0	0	2,924617972	20,44273419	EEF1B2;RPS17;ARL2BP;RPS8;FEM1B;RPS5;RPS6;RPS3A;RPL10A;GOLGA7;TPT1;PDRG1
Chlorobenzene-500_mg/kg_in_CMC-Rat-Heart-5d-up	13/322	0,000926478	0,015459615	0	0	2,774754007	19,37921662	RPL32;RPS8;RPS5;RPSA;RPS3A;RPL10A;TOMM20;PDRG1;EEF1B2;RPS17;ARL2BP;RPS20;RAPGEF5
Famciclovir-112_mg/kg_in_Saline-Rat-Liver-0.25d-up	13/322	0,000926478	0,015426931	0	0	2,774754007	19,37921662	C1QA;RPL32;RPS8;RPS5;RPS6;RPL35A;RPSA;RPS3A;SEPP1;EEF1B2;RPS17;RPS20;RPL39
Ketoprofen-4_mg/kg_in_Corn_Oil-Rat-Kidney-0.25d-up	13/322	0,000926478	0,015394384	0	0	2,774754007	19,37921662	C1QA;RPL32;RPS8;RPS6;RPSA;RPS3A;RPL10A;SEPP1;RPS17;RPS19;RPS20;RAPGEF5;TPT1
4-Octylphenol-50_mg/kg_in_Corn_Oil-Rat-Liver-1d-dn	12/283	0,000949733	0,015747568	0	0	2,914283633	20,28146136	EEF1B2;RPS17;RPL32;RPS19;RPS8;FEM1B;RPS6;RPS20;RPS3A;RPL10A;GOLGA7;TPT1
Gemfibrozil-700_mg/kg_in_Corn_Oil-Rat-Kidney-1d-dn	12/283	0,000949733	0,015714485	0	0	2,914283633	20,28146136	EEF1B2;RPS17;RPL32;RPS8;RPS6;RPL35A;RPS20;RPSA;RPS3A;RPL10A;RPL39;TOMM20
Phenacemide-400_mg/kg_in_CMC-Rat-Heart-3d-dn	13/323	0,000953027	0,015735938	0	0	2,766163438	19,24106513	RPL32;RPS8;RPS6;RPSA;RPS3A;RPL10A;SEPP1;PDRG1;RPS17;RPS19;ARL6IP5;RPS20;RPL39
Tetracycline-520_uM_in_DMSO-Rat-Primary_rat_hepatocytes-1d-up	13/323	0,000953027	0,015703018	0	0	2,766163438	19,24106513	RPL32;RPS8;RPS6;RPL35A;RPSA;RPS3A;RPL10A;PDRG1;EEF1B2;RPS17;ARL2BP;RPL39;TPT1
Daunorubicin-3.25_mg/kg_in_Saline-Rat-Kidney-3d-dn	12/284	0,000979045	0,016098036	0	0	2,904022071	20,12177386	EEF1B2;RPS17;RPL32;RPS8;RPS5;FUNDC1;ARL6IP5;RPL35A;RPS20;RPSA;RPS3A;RPL39
Doxapram-2_mg/kg_in_Saline-Rat-Liver-3d-up	12/284	0,000979045	0,016064499	0	0	2,904022071	20,12177386	EEF1B2;C1QA;RPS17;RPL32;RPS19;RPS5;RPS6;ARL6IP5;RPL35A;RPS20;RPSA;RPL39
Theophylline-225_mg/kg_in_Water-Rat-Heart-1d-up	12/284	0,000979045	0,016031101	0	0	2,904022071	20,12177386	EEF1B2;C1QA;RPS17;RPS19;ARL2BP;RPS8;RPS6;RPS20;RPS3A;RAPGEF5;RPL39;TPT1
Busulfan-9_mg/kg_in_Corn_Oil-Rat-Liver-5d-up	13/324	0,000980212	0,016016903	0	0	2,757625896	19,10412077	C1QA;RPL32;RPS8;RPS5;RPS6;RPL35A;RPS3A;RPL10A;EEF1B2;RPS17;RPS19;RPS20;RPL39
Daunorubicin-3.25_mg/kg_in_Saline-Rat-Liver-1d-dn	13/324	0,000980212	0,015983742	0	0	2,757625896	19,10412077	C1QA;RPL32;RPS6;RPL35A;RPSA;RPS3A;RPL10A;EEF1B2;RPS17;RPS19;ARL6IP5;RPS20;RPL39
Lipopolysaccharide_E._Coli_O55:B5-1.25_mg/kg_in_Saline-Rat-Liver-5d-up	13/324	0,000980212	0,015950717	0	0	2,757625896	19,10412077	C1QA;RPL32;RPS8;RPS6;RPL35A;RPSA;RPS3A;RPL10A;SEPP1;RPS17;RPS19;RPS20;RPL39
Phenobarbital-25_mg/kg_in_Water-Rat-Liver-0.25d-up	13/324	0,000980212	0,015917829	0	0	2,757625896	19,10412077	RPL32;RPS8;RPS5;RPS6;RPL35A;RPSA;RPS3A;RPL10A;SEPP1;RPS17;RPS19;RPS20;RPL39
Clindamycin-161_mg/kg_in_Saline-Rat-Kidney-1d-up	14/366	0,001003459	0,016261811	0	0	2,628959871	18,15113456	RPL32;RPS8;RPS5;RPS6;RPL13A;RPL35A;RPSA;RPS3A;SEPP1;TOMM20;RPS17;ARL6IP5;RPS20;TPT1
Amoxapine-313_mg/kg_in_CMC-Rat-Liver-3d-up	13/325	0,001008043	0,016302557	0	0	2,749140893	18,96837016	RPL32;RPS8;RPS5;RPS6;RPL13A;RPL35A;RPSA;RPS3A;RPL10A;RPS17;RPS19;RPS20;RPL39
Cerivastatin-7_mg/kg_in_Corn_Oil-Rat-Skeletal_muscle-3d-dn	13/325	0,001008043	0,01626915	0	0	2,749140893	18,96837016	RPL32;RPS6;RPSA;RPS3A;RPL10A;EEF1B2;RPS17;RPS19;COL5A3;ARL6IP5;RPS20;RPL39;SLC16A3
Indomethacin-9.6_mg/kg_in_Water-Rat-Kidney-5d-up	13/325	0,001008043	0,01623588	0	0	2,749140893	18,96837016	C1QA;RPL32;RPS8;RPS5;RPS6;RPL13A;RPL35A;RPS3A;EEF1B2;RPS17;RPS20;RPL39;TPT1
Valdecoxib-404_mg/kg_in_CMC-Rat-Kidney-1d-up	13/325	0,001008043	0,016202745	0	0	2,749140893	18,96837016	RPL32;RPS8;RPS5;RPS6;RPL13A;RPL35A;RPSA;RPS3A;EEF1B2;RPS17;RPS19;ARL6IP5;RPS20
Acetaminophen-972_mg/kg_in_Corn_Oil-Rat-Liver-5d-up	12/285	0,001009107	0,016186818	0	0	2,893832519	19,96365177	EEF1B2;RPS17;RPL32;RPS19;RPS8;RPS5;RPS6;RPS20;RPSA;RPS3A;RPL10A;TPT1
Fluconazole-394_mg/kg_in_Corn_Oil-Rat-Liver-0.25d-up	12/285	0,001009107	0,016153917	0	0	2,893832519	19,96365177	EEF1B2;RPS17;RPL32;RPS19;RPS8;RPS6;RPS20;RPSA;RPS3A;RPL10A;RPL39;TPT1
Diclofenac-10_mg/kg_in_Corn_Oil-Rat-Kidney-3d-up	14/367	0,001029919	0,016453636	0	0	2,621796492	18,03343748	C1QA;RPL32;RPS8;RPS5;RPS6;RPL13A;RPL35A;TOMM20;EEF1B2;RPS17;RPS20;RPL39;GOLGA7;TPT1
Cisplatin-63_uM_in_DMSO-Rat-Primary_rat_hepatocytes-0.67d-up	13/326	0,001036533	0,016525771	0	0	2,740707946	18,83380009	RPL32;RPS6;RPSA;RPL10A;PDRG1;EEF1B2;RPS17;CXCL11;ARL2BP;CLP1;ARL6IP5;RPL39;GOLGA7
Cytarabine-487_mg/kg_in_Saline-Rat-Spleen-5d-up	13/326	0,001036533	0,016492386	0	0	2,740707946	18,83380009	RPL32;RPS8;RPS5;RPL13A;RPL35A;RPSA;SEPP1;EEF1B2;RPS17;CXCL11;RPS19;FUNDC1;ARL6IP5
Mifepristone-300_mg/kg_in_Corn_Oil-Rat-Liver-5d-dn	13/326	0,001036533	0,016459135	0	0	2,740707946	18,83380009	C1QA;RPL32;RPS8;RPS6;RPSA;RPS3A;RPL10A;SEPP1;EEF1B2;RPS17;RPS19;RPS20;RPL39
Clemastine-2500_mg/kg_in_CMC-Rat-Heart-3d-up	12/286	0,001039933	0,016479911	0	0	2,883714224	19,80707545	C1QA;RPS17;RPL32;RPS19;RPS5;RPS6;RPL35A;RPSA;RPS3A;RPL10A;RPL39;TPT1
Valproic_Acid-1500_mg/kg_in_Water-Rat-Heart-3d-dn	12/286	0,001039933	0,016446818	0	0	2,883714224	19,80707545	EEF1B2;RPS17;RPL32;RPS8;COL5A3;RPS6;RPL35A;RPS20;RPSA;RPL39;TOMM20;TPT1
Cefotaxime-1000_mg/kg_in_Saline-Rat-Kidney-1d-up	14/368	0,001056964	0,016682665	0	0	2,614672045	17,91665974	C1QA;RPL32;RPS8;RPS5;RPS6;RPL13A;RPL35A;RPSA;RPS3A;TOMM20;RPS17;ARL6IP5;RPS20;TPT1
Chlortetracycline-1500_mg/kg_in_CMC-Rat-Liver-5d-up	13/327	0,001065693	0,016786801	0	0	2,732326576	18,70039754	C1QA;RPL32;RPS8;RPS6;RPL35A;RPSA;RPS3A;RPL10A;RPS17;RPS19;RPS20;RPL39;TPT1
Dexfenfluramine-29_mg/kg_in_Water-Rat-Heart-1d-dn	13/327	0,001065693	0,016753294	0	0	2,732326576	18,70039754	RPL32;RPS8;RPS6;RPSA;RPS3A;RPL10A;EEF1B2;RPS17;RPS19;COL5A3;RPS20;RPL39;TPT1
Procainamide-755_mg/kg_in_Water-Rat-Heart-3d-up	13/327	0,001065693	0,016719921	0	0	2,732326576	18,70039754	RPL32;RPS8;RPS5;RPL35A;RPSA;RPL10A;SEPP1;TOMM20;EEF1B2;RPS17;COL5A3;ARL6IP5;RPS20
Coumarin-111_mg/kg_in_Corn_Oil-Rat-Bone_marrow-1d-dn	12/287	0,001071539	0,016778214	0	0	2,873666439	19,65202561	EEF1B2;RPS17;RPL32;ARL2BP;RPS8;RPS5;RPS6;ARL6IP5;RPL35A;RPS20;RPSA;RPS3A
Isoniazid-79_mg/kg_in_Water-Rat-Liver-1d-up	12/287	0,001071539	0,016744924	0	0	2,873666439	19,65202561	EEF1B2;RPS17;RPL32;RPS19;RPS5;RPS6;RPL35A;RPS20;RPSA;RPS3A;RPL10A;RPL39
Lamotrigine-26_mg/kg_in_CMC-Rat-Heart-3d-up	12/287	0,001071539	0,016711766	0	0	2,873666439	19,65202561	RPS17;CXCL11;RPL32;RPS8;RPS5;RPL35A;RPSA;RPS3A;RAPGEF5;RPL10A;TOMM20;TPT1
N-Nitrosodiethylamine-34_mg/kg_in_Saline-Rat-Bone_marrow-3d-dn	12/287	0,001071539	0,016678739	0	0	2,873666439	19,65202561	EEF1B2;RPS17;RPL32;RPS8;RPS5;RPL13A;RPL35A;RPSA;RPS3A;RPL10A;RPL39;TPT1
Bromisovalum-250_mg/kg_in_Corn_Oil-Rat-Kidney-1d-dn	13/328	0,001095537	0,017018639	0	0	2,723996312	18,56814968	RPL32;RPS8;RPS6;RPSA;RPS3A;SEPP1;EEF1B2;RPS17;RPS19;ARL2BP;RPS20;RPL39;TPT1
Diclofenac-3.5_mg/kg_in_Corn_Oil-Rat-Liver-1d-dn	12/288	0,001103939	0,017115405	0	0	2,863688431	19,49848322	EEF1B2;C1QA;RPS17;RPL32;RPS8;RPS6;RPSA;RPS3A;RPL10A;RPL39;SEPP1;TPT1
Ouabain-144_mg/kg_in_CMC-Rat-Heart-5d-dn	12/288	0,001103939	0,017081779	0	0	2,863688431	19,49848322	RPS17;RPS19;ARL2BP;RPS8;FUNDC1;ARL6IP5;RPS20;RPSA;RPS3A;RPL39;SEPP1;TPT1
Ampiroxicam-94_mg/kg_in_CMC-Rat-Kidney-5d-up	13/329	0,001126076	0,017390152	0	0	2,715716688	18,43704385	C1QA;RPL32;RPS8;RPS6;RPSA;RPS3A;RPL10A;EEF1B2;RPS17;CXCL11;RPS19;RPS20;TPT1
Cytarabine-487_mg/kg_in_Saline-Rat-Spleen-1d-up	13/329	0,001126076	0,01735612	0	0	2,715716688	18,43704385	C1QA;RPL32;RPS5;RPL35A;RPSA;RPL10A;SEPP1;EEF1B2;RPS17;RPS19;ARL6IP5;RPS20;RPL39
Hydralazine-280_mg/kg_in_Water-Rat-Liver-1d-up	13/329	0,001126076	0,017322222	0	0	2,715716688	18,43704385	RPL32;RPS8;RPS5;RPS6;RPL35A;RPSA;RPS3A;RPS17;CXCL11;RPS19;RPS20;RPL39;TPT1
Acyclovir-330_mg/kg_in_Saline-Rat-Liver-1d-dn	12/289	0,001137149	0,017458456	0	0	2,853779474	19,34642957	EEF1B2;RPS17;RPL32;RPS19;RPS8;RPS6;RPS20;RPS3A;RPL10A;RPL39;SEPP1;TPT1
Doxorubicin-0.65_mg/kg_in_Saline-Rat-Spleen-0.25d-up	12/289	0,001137149	0,01742449	0	0	2,853779474	19,34642957	C1QA;RPS17;RPL32;RPS19;RPS8;RPS5;RPL13A;RPL35A;RPSA;RPS3A;RPL10A;TPT1
Ofloxacin-30_mg/kg_in_Water-Rat-Heart-3d-up	12/289	0,001137149	0,017390656	0	0	2,853779474	19,34642957	RPS17;RPL32;RPS19;ARL2BP;RPS8;RPS5;RPS6;ARL6IP5;RPS20;RPSA;RPS3A;RAPGEF5
Spironolactone-300_mg/kg_in_CMC-Rat-Liver-3d-dn	12/289	0,001137149	0,017356953	0	0	2,853779474	19,34642957	C1QA;RPS17;RPL32;RPS19;CLP1;RPS8;RPS6;RPL35A;RPSA;RPS3A;RPL39;SEPP1
Mercuric_Chloride-2.5_mg/kg_in_Water-Rat-Kidney-5d-dn	11/250	0,001140698	0,017377447	0	0	3,024054983	20,49134322	RPS17;RPL32;RPS19;RPS6;ARL6IP5;RPS20;RPSA;RPS3A;RPL39;SEPP1;TPT1
Ketoprofen-4_mg/kg_in_Corn_Oil-Rat-Kidney-3d-up	14/371	0,00114171	0,017359275	0	0	2,593529145	17,57175272	RPL32;RPS8;RPS5;RPS6;RPL35A;RPSA;RPS3A;RPL10A;RPS17;RPS19;ARL2BP;RPS20;RAPGEF5;TPT1
Sulindac-64_mg/kg_in_Corn_Oil-Rat-Kidney-3d-up	14/371	0,00114171	0,017325827	0	0	2,593529145	17,57175272	RPL32;RPS8;RPS5;RPS6;RPL13A;RPL35A;RPSA;RPS3A;RPL10A;TOMM20;EEF1B2;RPS17;RPS19;RPS20
Clindamycin-161_mg/kg_in_Saline-Rat-Kidney-3d-up	13/330	0,001157324	0,017529005	0	0	2,707487244	18,30706754	RPL32;RPS5;RPS6;RPL13A;RPL35A;RPSA;RPS3A;SEPP1;TOMM20;EEF1B2;RPS17;RPS20;TPT1
Methapyrilene-100_mg/kg_in_CMC-Rat-Liver-5d-up	13/330	0,001157324	0,01749536	0	0	2,707487244	18,30706754	RPL32;RPS8;RPS6;RPSA;RPS3A;RPL10A;SEPP1;EEF1B2;RPS17;RPS19;R3HCC1;RPS20;RPL39
Diethylstilbestrol-280_mg/kg_in_Corn_Oil-Rat-Liver-1d-up	12/290	0,001171185	0,017670979	0	0	2,843938855	19,19584625	EEF1B2;RPS17;RPL32;RPS19;RPS8;FEM1B;RPS5;RPS6;RPS20;RPSA;RPS3A;RPL10A
Galantamine-110_mg/kg_in_CMC-Rat-Heart-1d-dn	12/290	0,001171185	0,017637191	0	0	2,843938855	19,19584625	EEF1B2;RPS17;RPL32;RPS8;COL5A3;ARL6IP5;RPL35A;RPS20;RPSA;RPS3A;RPL39;TPT1
Indomethacin-5_mg/kg_in_Corn_Oil-Rat-Liver-3d-up	12/290	0,001171185	0,017603533	0	0	2,843938855	19,19584625	EEF1B2;C1QA;RPS17;RPS19;ARL2BP;RPS8;RPS6;RPS20;RPSA;RPL10A;RPL39;SEPP1
Norepinephrine-1.75_mg/kg_in_Saline-Rat-Heart-1d-up	12/290	0,001171185	0,017570002	0	0	2,843938855	19,19584625	C1QA;RPS17;CXCL11;RPS19;RPS8;RPS6;ARL6IP5;RPS20;RPSA;RPS3A;RPL39;TPT1
Nortriptyline-63_mg/kg_in_Water-Rat-Heart-1d-up	12/290	0,001171185	0,017536599	0	0	2,843938855	19,19584625	C1QA;RPS17;RPL32;ARL2BP;RPS8;RPS6;ARL6IP5;HOXA1;RPSA;RPS3A;RPL39;TPT1
Levamisole-120_mg/kg_in_Water-Rat-Liver-3d-up	13/331	0,001189292	0,017773937	0	0	2,699307524	18,17820845	RPL32;RPS8;RPS5;RPS6;RPL35A;RPSA;RPS3A;RPL10A;SEPP1;RPS17;RPS19;RPL39;TPT1
Metoclopramide-185_mg/kg_in_CMC-Rat-Intestine-5d-dn	13/331	0,001189292	0,017740275	0	0	2,699307524	18,17820845	RPS8;RPS6;RPL13A;RPL35A;RPSA;RPS3A;RPL10A;SEPP1;EEF1B2;RPS17;RPS19;ARL6IP5;RPL39
Kanamycin-225_mg/kg_in_Saline-Rat-Kidney-1d-up	14/373	0,001201319	0,017885799	0	0	2,579622822	17,34624867	RPL32;RPS8;RPS5;RPL13A;RPL35A;RPSA;RPS3A;SEPP1;TOMM20;RPS17;ARL6IP5;RPS20;RPL39;TPT1
6-Mercaptopurine-5_uM_in_DMSO-Rat-Primary_rat_hepatocytes-1d-dn	18/549	0,001205607	0,017915777	0	0	2,253394175	15,1445484	RPL32;RPS8;RPS5;RPS6;RPL35A;RPSA;RPS3A;RPL10A;SEPP1;EEF1B2;RPS17;RPS19;ARL2BP;FUNDC1;RPS20;RPL39;GOLGA7;TPT1
Labetalol-250_uM_in_DMSO-Rat-Primary_rat_hepatocytes-0.67d-up	12/291	0,001206061	0,017888774	0	0	2,83416587	19,0467151	EEF1B2;RPS17;RPL32;RPS19;ARL2BP;RPS5;RPS6;RPS20;RPS3A;RPL10A;RPL39;PDRG1
Allyl_Alcohol-25_mg/kg_in_Saline-Rat-Kidney-1d-up	13/332	0,001221994	0,018091027	0	0	2,691177079	18,05045441	RPL32;RPS8;RPS5;RPS6;RPL13A;RPL35A;RPSA;RPS3A;TOMM20;RPS17;RPS20;RPL39;TPT1
Cyclosporin_A-70_mg/kg_in_Corn_Oil-Rat-Kidney-5d-up	13/332	0,001221994	0,018057085	0	0	2,691177079	18,05045441	RPS8;RPS6;RPSA;RPS3A;RPL10A;SEPP1;EEF1B2;RPS17;CXCL11;RPS19;ARL6IP5;RPS20;TPT1
Bacitracin-380_mg/kg_in_Corn_Oil-Rat-Kidney-5d-up	12/292	0,001241795	0,018315313	0	0	2,824459822	18,89901827	C1QA;RPS17;RPL32;RPS19;RPS8;RPS5;RPS6;RPSA;RPS3A;RPL10A;RPL39;TPT1
Doxorubicin-0.65_mg/kg_in_Saline-Rat-Liver-0.25d-up	12/292	0,001241795	0,018281078	0	0	2,824459822	18,89901827	EEF1B2;RPS17;RPL32;RPS8;RPS5;RPL35A;RPSA;RPS3A;RPL10A;SEPP1;RPL39;TPT1
Etidronate-1005_mg/kg_in_Water-Rat-Heart-1d-up	12/292	0,001241795	0,018246972	0	0	2,824459822	18,89901827	EEF1B2;RPS17;RPL32;RPS19;ARL2BP;RPS8;RPS5;RPS6;ARL6IP5;RPL35A;RPS20;RPS3A
Furosemide-375_mg/kg_in_CMC-Rat-Kidney-3d-up	12/292	0,001241795	0,018212992	0	0	2,824459822	18,89901827	EEF1B2;C1QA;RPS17;RPL32;RPS19;RPS8;RPS5;ARL6IP5;RPL13A;RPL35A;RPS20;RPS3A

Nifedipine-1022_mg/kg_in_CMC-Rat-Heart-5d-up	12/292	0,001241795	0,018179139	0	0	2,824459822	18,89901827	C1QA;RPS17;RPL32;RPS19;RPS8;RPS6;RPS20;RPSA;RPS3A;RPL10A;SEPP1;RPL39
Pentobarbital-20_mg/kg_in_Water-Rat-Liver-1d-up	13/333	0,001255443	0,018344841	0	0	2,683095467	17,92379345	RPL32;RPS8;RPS5;RPS6;RPL35A;RPSA;RPS3A;RPL10A;SEPP1;EEF1B2;RPS17;RPS19;RPL39
Valproic_Acid-235_mg/kg_in_Water-Rat-Kidney-3d-up	14/375	0,001263518	0,018428638	0	0	2,565864834	17,12421174	RPL32;RPS8;RPS5;RPS6;RPL13A;RPL35A;RPSA;RPS3A;EEF1B2;RPS17;RPS19;ARL6IP5;RPS20;RPL39
Amoxapine-313_mg/kg_in_CMC-Rat-Heart-1d-dn	12/293	0,001278402	0,01861126	0	0	2,814820027	18,75273817	RPS17;RPL32;RPS19;ARL2BP;RPS8;COL5A3;RPS6;RPL35A;RPSA;RPS3A;GOLGA7;TPT1
Ethambutol-998_mg/kg_in_CMC-Rat-Kidney-5d-up	12/293	0,001278402	0,018576922	0	0	2,814820027	18,75273817	RPL32;RPS19;RPS8;RPS5;RPS6;RPSA;RPL10A;SEPP1;RPL39;GOLGA7;TOMM20;TPT1
Ifosfamide-143_mg/kg_in_Saline-Rat-Bone_marrow-1d-dn	12/293	0,001278402	0,018542711	0	0	2,814820027	18,75273817	EEF1B2;RPS17;RPL32;RPS19;RPS8;FEM1B;RPS6;RPS20;RPSA;RPS3A;RPL10A;RPL39
Methimazole-28_mg/kg_in_Water-Rat-Liver-1d-up	12/293	0,001278402	0,018508625	0	0	2,814820027	18,75273817	EEF1B2;RPS17;RPL32;RPS8;RPS5;RPS6;RPL13A;RPL35A;RPS20;RPSA;RPS3A;RPL39
Tacrolimus-134_mg/kg_in_CMC-Rat-Kidney-3d-up	12/293	0,001278402	0,018474664	0	0	2,814820027	18,75273817	EEF1B2;C1QA;RPL32;RPS19;ARL2BP;RPS8;RPS5;RPS6;RPL35A;RPS3A;TPT1;PDRG1
Zileuton-450_mg/kg_in_Corn_Oil-Rat-Kidney-3d-up	12/293	0,001278402	0,018440828	0	0	2,814820027	18,75273817	RPS17;RPL32;RPS19;ARL2BP;RPS8;RPS5;RPS6;RPL13A;RPL35A;RPS20;RPS3A;RPL39
Fluvastatin-94_mg/kg_in_Corn_Oil-Rat-Liver-3d-up	13/334	0,001289652	0,0185691	0	0	2,675062247	17,79821372	RPL32;RPS8;RPS5;RPS6;RPL35A;RPSA;RPS3A;RPL10A;EEF1B2;RPS17;RPS19;RPS20;TPT1
Valproic_Acid-235_mg/kg_in_Water-Rat-Bone_marrow-3d-up	13/334	0,001289652	0,018535215	0	0	2,675062247	17,79821372	C1QA;RPL32;RPS5;NKG7;RPL35A;RPSA;RPS3A;RPL10A;SEPP1;EEF1B2;RPS17;RPS20;RPL39
Sulfamethoxazole-1000_mg/kg_in_Water-Rat-Kidney-5d-up	14/376	0,001295615	0,018587002	0	0	2,559040725	17,01447252	C1QA;RPL32;RPS6;RPL13A;RPL35A;RPSA;RPS3A;RPL10A;EEF1B2;RPS17;CXCL11;RPS19;ARL6IP5;RPS20
Azathioprine-20_mg/kg_in_Corn_Oil-Rat-Spleen-3d-up	12/294	0,001315898	0,018843659	0	0	2,80524581	18,60785749	EEF1B2;C1QA;RPS17;RPL32;RPS8;RPS5;RPS6;RPL13A;RPL35A;RPS20;RPSA;RPS3A
Cadmium_Chloride-8_uM_in_DMSO-Rat-Primary_rat_hepatocytes-0.67d-dn	12/294	0,001315898	0,01880946	0	0	2,80524581	18,60785749	EEF1B2;RPS17;CXCL11;RPS19;ARL2BP;FEM1B;RPS20;RPS3A;RPL10A;SEPP1;RPL39;TPT1
Carboplatin-14_mg/kg_in_Saline-Rat-Spleen-1d-dn	12/294	0,001315898	0,018775384	0	0	2,80524581	18,60785749	EEF1B2;RPS17;RPL32;RPS19;RPS8;RPS5;FUNDC1;RPS6;RPL13A;RPL35A;RPSA;RPS3A
Ethinylestradiol-1480_mg/kg_in_Corn_Oil-Rat-Liver-1d-up	12/294	0,001315898	0,018741433	0	0	2,80524581	18,60785749	EEF1B2;RPS17;RPL32;RPS19;RPS8;FEM1B;RPS6;RPS20;RPSA;RPS3A;RPL10A;RPL39
Norethindrone-0.08_mg/kg_in_Corn_Oil-Rat-Liver-5d-up	12/294	0,001315898	0,018707603	0	0	2,80524581	18,60785749	EEF1B2;C1QA;RPS17;RPL32;RPS19;RPS8;RPS5;RPS6;RPL35A;RPS20;RPSA;RPL39
Phenylbutazone-368_mg/kg_in_CMC-Rat-Kidney-3d-up	12/294	0,001315898	0,018673896	0	0	2,80524581	18,60785749	C1QA;RPL32;RPS19;FEM1B;RPS5;RPS6;RPL35A;RPS3A;RPL10A;SEPP1;RPL39;TPT1
Candesartan-1300_mg/kg_in_CMC-Rat-Kidney-1d-up	13/335	0,001324634	0,018764058	0	0	2,667076986	17,67370358	RPL32;RPS8;RPS6;RPL35A;RPSA;RPL10A;EEF1B2;RPS17;RPS19;FUNDC1;ARL6IP5;RPS20;RPL39
Daunorubicin-3.25_mg/kg_in_Saline-Rat-Liver-5d-dn	13/335	0,001324634	0,01873037	0	0	2,667076986	17,67370358	C1QA;RPL32;RPS6;RPL35A;RPSA;RPS3A;RPL10A;EEF1B2;RPS17;RPS19;ARL6IP5;RPS20;RPL39
Lead_(II)_Acetate-2_mg/kg_in_Saline-Rat-Kidney-14d-up	13/335	0,001324634	0,018696803	0	0	2,667076986	17,67370358	RPL32;RPS8;RPS5;RPS6;RPS3A;RPL10A;EEF1B2;RPS17;CXCL11;RPS19;ARL6IP5;RPS20;RPL39
Fluconazole-10_mg/kg_in_Corn_Oil-Rat-Liver-5d-up	12/295	0,0013543	0,019081338	0	0	2,795736502	18,46435917	EEF1B2;RPS17;RPL32;RPS8;RPS5;RPS6;RPL35A;RPS20;RPSA;RPS3A;SEPP1;RPL39
Ramipril-1500_mg/kg_in_CMC-Rat-Kidney-1d-up	12/295	0,0013543	0,019047264	0	0	2,795736502	18,46435917	EEF1B2;C1QA;RPS17;RPL32;RPS8;RPS5;RPS6;ARL6IP5;RPL13A;RPL35A;RPS20;RPL10A
Torsemide-110_mg/kg_in_Corn_Oil-Rat-Liver-3d-dn	12/295	0,0013543	0,019013312	0	0	2,795736502	18,46435917	EEF1B2;RPS17;RPL32;RPS19;RPS8;RPS5;RPL35A;RPS20;RPSA;RPS3A;RPL10A;TPT1
1-Naphthyl_Isothiocyanate-30_mg/kg_in_Corn_Oil-Rat-Liver-3d-up	13/336	0,001360403	0,019065002	0	0	2,659139257	17,55025151	C1QA;RPL32;RPS8;RPS5;RPS6;RPL35A;RPSA;RPL10A;EEF1B2;RPS19;RPS20;RPL39;TPT1
Bisphenol_A-610_mg/kg_in_Corn_Oil-Rat-Kidney-5d-up	13/336	0,001360403	0,019031139	0	0	2,659139257	17,55025151	RPL32;RPS8;RPS5;RPL35A;RPSA;RPS3A;SEPP1;TOMM20;EEF1B2;RPS17;ARL2BP;RPS20;TPT1
Acetaminophen-1950_uM_in_DMSO-Rat-Primary_rat_hepatocytes-1d-up	11/256	0,00137934	0,019261847	0	0	2,953178694	19,45007828	C1QA;CXCL11;RPL32;ARL2BP;CLP1;RPS8;FEM1B;RPS5;RPS6;RPS20;RPS3A
Ethylene_Glycol-352_mg/kg_in_Water-Rat-Liver-3d-dn	11/256	0,00137934	0,019227756	0	0	2,953178694	19,45007828	RPS17;RPL32;RPS19;RPS8;RPS6;ARL6IP5;RPL35A;RPS20;RPS3A;SEPP1;TPT1
Carbon_Tetrachloride-400_mg/kg_in_Corn_Oil-Rat-Bone_marrow-0.25d-up	12/296	0,001393625	0,019392562	0	0	2,786291446	18,32222643	C1QA;RPL32;RPS8;FEM1B;RPS5;FUNDC1;RPS6;RPL35A;RPS20;RPS3A;RPL39;TPT1
Carmustine-16_mg/kg_in_Corn_Oil-Rat-Bone_marrow-3d-up	12/296	0,001393625	0,01935836	0	0	2,786291446	18,32222643	EEF1B2;C1QA;RPS17;RPL32;RPS8;RPS5;RPL13A;RPL35A;RPS20;SEPP1;TPT1;DUSP14
N-Nitrosodiethylamine-34_mg/kg_in_Saline-Rat-Kidney-5d-up	12/296	0,001393625	0,019324278	0	0	2,786291446	18,32222643	RPS17;RPL32;RPS19;RPS5;RPS6;RPL13A;RPL35A;RPS20;RPSA;RPS3A;RPL39;TPT1
Sildenafil-14.6_mg/kg_in_Saline-Rat-Liver-1d-dn	12/296	0,001393625	0,019290316	0	0	2,786291446	18,32222643	EEF1B2;RPS17;RPL32;RPS8;RPS5;RPS6;RPL35A;RPSA;RPS3A;RPL10A;SEPP1;TPT1
Valproic_Acid-850_mg/kg_in_Water-Rat-Liver-5d-up	12/296	0,001393625	0,019256474	0	0	2,786291446	18,32222643	EEF1B2;RPS17;RPL32;RPS19;RPS8;RPS5;RPS6;RPL35A;RPS20;RPS3A;RPL10A;RPL39
Zomepirac-11_mg/kg_in_Saline-Rat-Liver-5d-up	12/296	0,001393625	0,019222749	0	0	2,786291446	18,32222643	EEF1B2;RPS17;RPL32;RPS19;RPS8;RPS6;RPL35A;RPS20;RPSA;RPS3A;RPL39;TPT1
Rofecoxib-775_mg/kg_in_Corn_Oil-Rat-Heart-3d-up	14/379	0,001396034	0,019222312	0	0	2,538784466	16,69027377	C1QA;RPS6;RPSA;RPS3A;RPL10A;PDRG1;EEF1B2;RPS19;ARL2BP;ARL6IP5;RPS20;RAPGEF5;RPL39;TPT1
Sodium_Nitroprusside-6_mg/kg_in_Saline-Rat-Heart-3d-dn	14/380	0,00143092	0,019668282	0	0	2,532103455	16,58385384	RPL32;FEM1B;RPS6;RPSA;RPS3A;RPL10A;SEPP1;EEF1B2;RPS17;RPS19;COL5A3;RPS20;RPL39;TPT1
4-Chloroaniline-75_mg/kg_in_CMC-Rat-Kidney-5d-up	12/297	0,001433889	0,019674759	0	0	2,776909993	18,18144271	EEF1B2;RPS17;CXCL11;CLP1;RPS8;ARL6IP5;RPS20;RPSA;RPS3A;RAPGEF5;TOMM20;TPT1
Atorvastatin-300_mg/kg_in_Corn_Oil-Rat-Liver-3d-up	12/297	0,001433889	0,019640542	0	0	2,776909993	18,18144271	C1QA;RPS17;CXADR;RPS19;ARL2BP;RPS8;RPS6;RPS20;RPSA;RPS3A;RPL10A;RPL39
Busulfan-3_mg/kg_in_Corn_Oil-Rat-Bone_marrow-3d-up	12/297	0,001433889	0,019606444	0	0	2,776909993	18,18144271	EEF1B2;C1QA;RPS17;RPL32;RPS8;RPS5;RPL13A;RPL35A;RPS20;RPS3A;TPT1;DUSP14
Lead_(II)_Acetate-200_mg/kg_in_Saline-Rat-Kidney-0.25d-up	12/297	0,001433889	0,019572464	0	0	2,776909993	18,18144271	RPS17;RPL32;RPS8;RPS5;ARL6IP5;RPL13A;RPL35A;RPS20;RPSA;RPS3A;TOMM20;TPT1
Lomefloxacin-2000_mg/kg_in_CMC-Rat-Liver-5d-up	12/297	0,001433889	0,019538601	0	0	2,776909993	18,18144271	RPS17;RPL32;RPS19;ARL2BP;RPS8;RPS6;RPS20;RPSA;RPL10A;SEPP1;RPL39;TPT1
Quetiapine-500_mg/kg_in_CMC-Rat-Liver-3d-dn	12/297	0,001433889	0,019504856	0	0	2,776909993	18,18144271	EEF1B2;C1QA;RPS17;RPL32;RPS8;RPS5;RPL35A;RPS20;RPSA;RPS3A;SEPP1;TPT1
Vinorelbine-1.5_mg/kg_in_Saline-Rat-Liver-1d-dn	12/297	0,001433889	0,019471227	0	0	2,776909993	18,18144271	EEF1B2;RPS17;RPL32;RPS19;RPS8;RPS6;RPL35A;RPS20;RPSA;RPS3A;RPL10A;RPL39
Pyridine-223_mg/kg_in_Water-Rat-Kidney-3d-dn	10/219	0,00143901	0,019507137	0	0	3,138288691	20,53633221	RPS17;RPL32;RPS19;ARL2BP;RPS8;RPS6;RPL35A;RPSA;RPS3A;RPL39
Phenelzine-27_mg/kg_in_Water-Rat-Heart-3d-dn	11/258	0,001467447	0,019858447	0	0	2,930285836	19,11786115	EEF1B2;RPS17;RPL32;RPS19;COL5A3;RPS6;RPSA;RPS3A;RPL10A;RPL39;SEPP1
Melphalan-12_mg/kg_in_CMC-Rat-Bone_marrow-1d-dn	12/298	0,00147511	0,019927902	0	0	2,767591503	18,04199174	C1QA;RPS17;RPL32;RPS19;ARL2BP;RPS8;RPS5;RPL13A;RPL35A;RPSA;RPL10A;SEPP1
Methyl_Methanesulfonate-113_mg/kg_in_Water-Rat-Bone_marrow-1d-dn	12/298	0,00147511	0,019893779	0	0	2,767591503	18,04199174	EEF1B2;RPS17;RPL32;RPS8;RPS5;RPS6;ARL6IP5;RPL35A;RPS20;RPS3A;RPL39;TPT1
Penicillamine-100_mg/kg_in_Water-Rat-Bone_marrow-1d-dn	12/298	0,00147511	0,019859773	0	0	2,767591503	18,04199174	EEF1B2;RPS17;RPL32;RPS19;RPS8;RPS5;RPS6;ARL6IP5;RPL35A;RPS20;RPSA;DUSP14
Sodium_Arsenite-10_mg/kg_in_Saline-Rat-Kidney-3d-up	12/298	0,00147511	0,019825882	0	0	2,767591503	18,04199174	RPS17;RPL32;ARL2BP;RPS5;ARL6IP5;RPL13A;RPL35A;RPS20;RPS3A;SEPP1;RPL39;TOMM20
Prednisolone-184_mg/kg_in_Water-Rat-Heart-3d-dn	13/340	0,00151162	0,020281973	0	0	2,627855266	17,06679905	RPL32;RPS8;RPS5;RPS6;RPL35A;RPSA;RPS3A;EEF1B2;RPS17;ARL2BP;RPS20;RPL39;TPT1
Carboplatin-14_mg/kg_in_Saline-Rat-Liver-5d-dn	12/299	0,001517305	0,020323626	0	0	2,758335345	17,90385748	EEF1B2;RPS17;RPL32;RPS19;CLP1;RPS6;RPS20;RPSA;RPS3A;RPL10A;RPL39;TPT1
Loxoprofen-18_mg/kg_in_Corn_Oil-Rat-Kidney-3d-up	12/299	0,001517305	0,02028912	0	0	2,758335345	17,90385748	RPS17;RPL32;RPS19;ARL2BP;RPS5;RPS6;RPL35A;RPSA;RPS3A;RPL10A;RPL39;TOMM20
Sparfloxacin-29_mg/kg_in_Corn_Oil-Rat-Liver-5d-up	12/299	0,001517305	0,020254732	0	0	2,758335345	17,90385748	EEF1B2;C1QA;RPS17;RPL32;RPS19;CLP1;RPS8;RPS6;RPS20;RPS3A;RPL39;TPT1
Tocainide-224_mg/kg_in_Corn_Oil-Rat-Liver-3d-up	12/299	0,001517305	0,02022046	0	0	2,758335345	17,90385748	EEF1B2;RPS17;RPL32;RPS19;CLP1;RPS8;RPS5;RPS6;RPL35A;RPS20;RPS3A;RPL39
Valproic_Acid-3168_uM_in_DMSO-Rat-Primary_rat_hepatocytes-0.67d-up	12/299	0,001517305	0,020186304	0	0	2,758335345	17,90385748	EEF1B2;RPS17;RPL32;RPS19;RPS8;RPS5;RPS6;RPL35A;RPS20;RPS3A;TPT1;PDRG1
Doxazosin-5_mg/kg_in_Water-Rat-Heart-1d-dn	13/341	0,00155153	0,020606826	0	0	2,620148945	16,94846998	RPL32;RPS8;FEM1B;RPS6;RPS3A;RPL10A;EEF1B2;RPS17;RPS19;ARL2BP;RPS20;RPL39;TPT1
Hydrazine-45_mg/kg_in_Water-Rat-Liver-0.25d-up	13/341	0,00155153	0,020572134	0	0	2,620148945	16,94846998	C1QA;RPL32;RPS8;RPS5;RPS6;RPL13A;RPSA;RPS3A;RPL10A;SEPP1;RPS19;RPS20;TPT1
Catechol-195_mg/kg_in_Saline-Rat-Kidney-3d-up	12/300	0,001560491	0,020656175	0	0	2,749140893	17,76702411	RPS17;RPL32;RPS8;RPS5;ARL6IP5;RPL13A;RPL35A;RPS20;RPSA;RPS3A;RPL39;TOMM20
Clonidine-6_mg/kg_in_Water-Rat-Heart-3d-up	12/300	0,001560491	0,020621517	0	0	2,749140893	17,76702411	C1QA;RPS17;RPL32;RPS8;RPS5;RPS6;RPS20;RPS3A;RAPGEF5;RPL10A;SEPP1;TPT1
Cyclosporin_A-350_mg/kg_in_Corn_Oil-Rat-Spleen-5d-up	12/300	0,001560491	0,020586975	0	0	2,749140893	17,76702411	EEF1B2;C1QA;RPS17;RPL32;RPS19;RPS8;RPS5;RPS20;RPSA;RPS3A;RPL10A;TPT1
Diethylstilbestrol-280_mg/kg_in_Corn_Oil-Rat-Bone_marrow-1d-dn	12/300	0,001560491	0,020552548	0	0	2,749140893	17,76702411	EEF1B2;RPS17;RPL32;RPS8;FEM1B;RPS5;RPS6;ARL6IP5;RPL13A;RPL35A;RPSA;RPS3A
Ferulic_Acid-1500_mg/kg_in_CMC-Rat-Liver-1d-up	12/300	0,001560491	0,020518237	0	0	2,749140893	17,76702411	EEF1B2;RPS17;RPL32;RPS19;RPS8;RPS5;RPS6;RPSA;RPS3A;RPL10A;RPL39;TPT1
Fluvastatin-94_mg/kg_in_Corn_Oil-Rat-Skeletal_muscle-5d-up	12/300	0,001560491	0,02048404	0	0	2,749140893	17,76702411	EEF1B2;RPS17;RPL32;RPS19;RPS8;RPS6;RPS20;RPSA;RPS3A;RPL10A;RPL39;TPT1
Lead_(II)_Acetate-200_mg/kg_in_Saline-Rat-Spleen-3d-dn	12/300	0,001560491	0,020449957	0	0	2,749140893	17,76702411	EEF1B2;C1QA;RPS17;ARL2BP;RPS8;FUNDC1;RPS6;ARL6IP5;RPL35A;RPS3A;RPL39;TPT1
Methotrexate-27_mg/kg_in_Corn_Oil-Rat-Bone_marrow-1d-dn	12/300	0,001560491	0,020415987	0	0	2,749140893	17,76702411	EEF1B2;RPS17;RPL32;RPS8;FEM1B;RPS5;RPS6;ARL6IP5;RPL35A;RPS20;RPSA;RPS3A
NN-Dimethylformamide-140_mg/kg_in_Saline-Rat-Spleen-1d-dn	12/300	0,001560491	0,020382129	0	0	2,749140893	17,76702411	EEF1B2;RPS17;RPL32;RPS8;RPS5;ARL6IP5;RPL13A;RPL35A;RPS20;RPSA;RPS3A;TPT1
Norethindrone-375_mg/kg_in_Corn_Oil-Rat-Liver-5d-up	12/300	0,001560491	0,020348384	0	0	2,749140893	17,76702411	EEF1B2;RPS17;RPL32;RPS19;RPS8;RPS5;RPS6;RPL35A;RPS20;RPSA;RPS3A;RPL39
Sulfathiazole-2629_mg/kg_in_Water-Rat-Liver-3d-up	12/300	0,001560491	0,02031475	0	0	2,749140893	17,76702411	EEF1B2;RPS17;RPL32;RPS8;RPS5;RPS6;RPL35A;RPS20;RPSA;RPS3A;SEPP1;TOMM20
Trimethadione-535_mg/kg_in_Water-Rat-Heart-3d-dn	12/300	0,001560491	0,020281228	0	0	2,749140893	17,76702411	RPS17;RPL32;RPS8;COL5A3;RPS5;RPS6;RPL35A;RPS20;RPSA;RPS3A;RPL39;TPT1
Doxorubicin-3_mg/kg_in_Saline-Rat-Heart-1d-dn	13/342	0,00159231	0,020660684	0	0	2,612487691	16,83113308	RPL32;RPS8;RPS5;RPL35A;RPSA;RPS3A;TOMM20;EEF1B2;RPS17;ARL6IP5;RPS20;RPL39;TPT1
11-Dichloroethene-600_mg/kg_in_Water-Rat-Liver-5d-up	12/301	0,001604685	0,020787009	0	0	2,740007535	17,63147608	EEF1B2;C1QA;RPS17;RPL32;RPS19;RPS5;RPS6;ARL6IP5;RPL35A;RPS20;RPSA;RPS3A
Altretamine-40_mg/kg_in_Corn_Oil-Rat-Bone_marrow-5d-up	12/301	0,001604685	0,020752876	0	0	2,740007535	17,63147608	EEF1B2;C1QA;RPS17;RPL32;FEM1B;RPS5;RPL35A;RPS20;RPS3A;SEPP1;RPL39;TPT1
Atorvastatin-300_mg/kg_in_Corn_Oil-Rat-Skeletal_muscle-3d-up	12/301	0,001604685	0,020718855	0	0	2,740007535	17,63147608	EEF1B2;RPS17;RPL32;RPS19;RPS5;FUNDC1;RPL35A;RPS20;RPSA;RPS3A;RPL39;TPT1
Captopril-1750_mg/kg_in_Water-Rat-Kidney-5d-up	12/301	0,001604685	0,020684946	0	0	2,740007535	17,63147608	EEF1B2;RPS17;CXCL11;RPL32;RPS19;RPS6;RPL35A;RPS20;RPS3A;RAPGEF5;RPL10A;RPL39
Diethylstilbestrol-2.8_mg/kg_in_Corn_Oil-Rat-Liver-3d-up	13/343	0,001633976	0,021028097	0	0	2,604871109	16,71477787	C1QA;RPL32;RPS8;RPS5;RPS6;RPL35A;RPSA;RPL10A;EEF1B2;RPS19;RPS20;RPL39;TPT1
Mercuric_Chloride-2.5_mg/kg_in_Water-Rat-Kidney-3d-dn	10/223	0,00164588	0,021146741	0	0	3,081996517	19,75399502	RPS17;RPL32;RPS19;RPS6;ARL6IP5;RPL35A;RPS20;RPSA;RPS3A;TPT1
Methotrexate-0.3_mg/kg_in_Corn_Oil-Rat-Spleen-0.25d-up	12/302	0,001649907	0,021163951	0	0	2,730934662	17,49719805	EEF1B2;C1QA;RPS17;RPS8;RPS5;RPL13A;RPL35A;RPS20;RPS3A;RAPGEF5;SEPP1;TPT1
Mesna-755_mg/kg_in_Saline-Rat-Kidney-1d-up	13/344	0,001676542	0,021470639	0	0	2,597298809	16,59939399	C1QA;RPL32;RPS6;RPL13A;RPL35A;RPSA;RPS3A;SEPP1;RPS17;RPS19;ARL6IP5;RPS20;RPL39
Methotrexate-0.3_mg/kg_in_Corn_Oil-Rat-Liver-3d-up	13/344	0,001676542	0,021435784	0	0	2,597298809	16,59939399	C1QA;RPL32;RPS8;RPS5;RPS6;RPL35A;RPSA;SEPP1;EEF1B2;RPS19;RPS20;RPL39;TPT1
DActinomycin-0.06_mg/kg_in_Saline-Rat-Bone_marrow-0.25d-up	12/303	0,001696173	0,021651633	0	0	2,721921677	17,36417492	RPS17;RPL32;RPS19;FEM1B;RPS5;RPS6;ARL6IP5;RPSA;RPS3A;RPL10A;RPL39;TPT1
Lomustine-560_uM_in_DMSO-Rat-Primary_rat_hepatocytes-1d-up	12/303	0,001696173	0,021616598	0	0	2,721921677	17,36417492	EEF1B2;RPS17;RPL32;CLP1;RPS5;RPS6;ARL6IP5;RPL35A;RPSA;RPL10A;GOLGA7;PDRG1
Sertraline-210_mg/kg_in_Corn_Oil-Rat-Liver-3d-dn	12/303	0,001696173	0,021581676	0	0	2,721921677	17,36417492	EEF1B2;RPS17;RPL32;RPS19;CLP1;RPS8;RPS6;RPL35A;RPS3A;RPL10A;RPL39;TPT1
Sodium_Arsenite-10_mg/kg_in_Saline-Rat-Kidney-1d-up	12/303	0,001696173	0,021546867	0	0	2,721921677	17,36417492	RPS17;RPL32;RPS8;RPS6;ARL6IP5;RPL13A;RPL35A;RPS20;RPSA;RPS3A;SEPP1;RPL39
Caffeine-93_mg/kg_in_Water-Rat-Heart-3d-dn	13/345	0,001720022	0,021814647	0	0	2,589770407	16,48497125	RPL32;RPS8;RPS6;RPL35A;RPSA;RPS3A;RPL10A;PDRG1;EEF1B2;RPS17;RPS19;ARL6IP5;RPL39
Azathioprine-0.5_uM_in_DMSO-Rat-Primary_rat_hepatocytes-0.67d-up	12/304	0,001743502	0,022076882	0	0	2,712967987	17,23239181	EEF1B2;RPS17;RPL32;RPS19;RPS5;ARL6IP5;RPL35A;RPS3A;SEPP1;RPL39;TPT1;PDRG1
Beta-Estradiol-150_mg/kg_in_Corn_Oil-Rat-Liver-3d-up	12/304	0,001743502	0,022041446	0	0	2,712967987	17,23239181	EEF1B2;RPS17;RPL32;RPS19;FEM1B;RPS6;RPS20;RPSA;RPS3A;RPL10A;SEPP1;RPL39
Bleomycin_A2-12_mg/kg_in_Saline-Rat-Bone_marrow-3d-up	12/304	0,001743502	0,022006123	0	0	2,712967987	17,23239181	EEF1B2;RPL32;RPS8;RPS5;FUNDC1;RPL13A;RPL35A;RPS20;RPSA;RPL10A;TOMM20;TPT1
Doxorubicin-1.5_uM_in_DMSO-Rat-Primary_rat_hepatocytes-0.67d-up	12/304	0,001743502	0,021970913	0	0	2,712967987	17,23239181	EEF1B2;RPS17;CXCL11;RPL32;RPS19;RPS8;RPS6;RPS20;RPS3A;SEPP1;RPL39;GOLGA7
Ifosfamide-143_mg/kg_in_Saline-Rat-Liver-3d-dn	12/304	0,001743502	0,021935816	0	0	2,712967987	17,23239181	EEF1B2;RPS17;RPL32;RPS8;RPS6;ARL6IP5;RPL35A;RPS20;RPS3A;RPL10A;RPL39;TPT1
Lomustine-4.2_mg/kg_in_Corn_Oil-Rat-Spleen-0.25d-up	12/304	0,001743502	0,02190083	0	0	2,712967987	17,23239181	EEF1B2;CXCL11;RPS19;ARL2BP;CLP1;RPS8;FUNDC1;RPS6;RPS20;RPSA;RPL10A;RPL39

NN-Dimethylformamide-140_mg/kg_in_Saline-Rat-Bone_marrow-3d-dn	12/304	0,001743502	0,021865957	0	0	2,712967987	17,23239181	EEF1B2;RPL32;RPS19;ARL2BP;RPS8;RPS5;FUNDC1;RPL13A;RPL35A;RPS20;RPSA;TPT1
Pyrogallol-1000_mg/kg_in_Water-Rat-Liver-5d-up	12/304	0,001743502	0,021831194	0	0	2,712967987	17,23239181	EEF1B2;RPS17;RPL32;RPS19;RPS8;RPS5;RPS6;RPL35A;RPS20;RPS3A;RPL10A;SEPP1
Rifabutin-1500_mg/kg_in_CMC-Rat-Liver-1d-dn	12/304	0,001743502	0,021796541	0	0	2,712967987	17,23239181	EEF1B2;C1QA;RPS17;RPL32;RPS19;RPS6;RPS20;RPSA;RPS3A;RPL10A;RPL39;TPT1
Spironolactone-300_mg/kg_in_CMC-Rat-Heart-5d-up	12/304	0,001743502	0,021761998	0	0	2,712967987	17,23239181	EEF1B2;RPS17;RPL32;RPS19;RPS8;RPS5;RPS6;RPL35A;RPS20;RPS3A;RPL10A;RPL39
Daunorubicin-3.25_mg/kg_in_Saline-Rat-Bone_marrow-3d-dn	11/264	0,001759854	0,021931339	0	0	2,863688431	18,16301463	EEF1B2;RPS17;RPL32;RPS8;FEM1B;RPS6;NKG7;RPS20;RPSA;RPS3A;RPL39
Bezafibrate-617_mg/kg_in_Corn_Oil-Rat-Kidney-1d-dn	12/305	0,001791912	0,022295576	0	0	2,70407301	17,10183405	EEF1B2;RPS17;RPL32;ARL2BP;RPS8;FUNDC1;RPS20;RPSA;SEPP1;RPL39;TOMM20;TPT1
Crotamiton-750_mg/kg_in_Corn_Oil-Rat-Liver-5d-up	12/305	0,001791912	0,02226041	0	0	2,70407301	17,10183405	RPS17;RPL32;RPS19;ARL2BP;RPS8;RPS5;RPS6;RPS20;RPSA;RPS3A;RPL10A;TPT1
Fenofibrate-430_mg/kg_in_Corn_Oil-Rat-Skeletal_muscle-3d-dn	12/305	0,001791912	0,022225354	0	0	2,70407301	17,10183405	EEF1B2;RPS17;RPL32;RPS8;FEM1B;COL5A3;ARL6IP5;RPL35A;RPSA;RPL10A;RPL39;TPT1
Hydroxyurea-400_mg/kg_in_Saline-Rat-Spleen-3d-dn	12/305	0,001791912	0,022190409	0	0	2,70407301	17,10183405	EEF1B2;C1QA;RPS17;RPL32;RPS8;RPS5;RPS6;ARL6IP5;RPL35A;RPSA;RPS3A;TPT1
Interleukin_1_Beta-0.01_mg/kg_in_RSA/Saline-Rat-Liver-1d-up	12/305	0,001791912	0,022155573	0	0	2,70407301	17,10183405	EEF1B2;RPS17;RPL32;RPS19;RPS8;RPS6;ARL6IP5;RPL35A;RPS20;RPSA;RPS3A;RPL39
Mitomycin_C-0.5_mg/kg_in_Saline-Rat-Liver-5d-up	12/305	0,001791912	0,022120846	0	0	2,70407301	17,10183405	EEF1B2;RPS17;RPL32;RPS19;RPS8;RPS6;RPS20;RPS3A;SEPP1;RPL39;GOLGA7;TPT1
Pantoprazole-1100_mg/kg_in_Water-Rat-Liver-1d-up	12/305	0,001791912	0,022086228	0	0	2,70407301	17,10183405	EEF1B2;RPS17;RPL32;RPS19;RPS8;RPS5;RPS6;RPL35A;RPS20;RPSA;RPS3A;RPL39
Vinorelbine-1.5_mg/kg_in_Saline-Rat-Heart-3d-dn	12/305	0,001791912	0,022051719	0	0	2,70407301	17,10183405	RPS17;RPL32;RPS19;RPS8;FUNDC1;RPS6;ARL6IP5;RPS20;RPSA;RPS3A;RPL39;TPT1
Camptothecin-5_mg/kg_in_Saline-Rat-Kidney-1d-dn	13/347	0,001809788	0,022236953	0	0	2,574843776	16,258969	RPL32;RPS6;RPL35A;RPSA;RPS3A;RPL10A;EEF1B2;RPS17;CLP1;FUNDC1;RPS20;RPL39;TPT1
Cyclosporin_A-70_mg/kg_in_Corn_Oil-Rat-Spleen-0.25d-up	13/347	0,001809788	0,022202316	0	0	2,574843776	16,258969	C1QA;RPL32;RPS8;RPS6;RPL13A;RPSA;RPL10A;SEPP1;EEF1B2;RPS17;RPS20;RPL39;TPT1
Pravastatin-1200_mg/kg_in_Corn_Oil-Rat-Skeletal_muscle-5d-dn	13/347	0,001809788	0,022167786	0	0	2,574843776	16,258969	RPL32;RPS8;FEM1B;RPSA;RPS3A;RPL10A;EEF1B2;RPS19;COL5A3;ARL6IP5;RPL39;SLC16A3;TPT1
Alendronic_Acid-138_mg/kg_in_CMC-Rat-Heart-5d-up	11/265	0,001812918	0,022171653	0	0	2,852882059	18,00972348	EEF1B2;RPS17;RPL32;RPS19;ARL2BP;RPS8;RPL35A;RPS20;RPSA;RPS3A;TPT1
Azathioprine-54_mg/kg_in_Water-Rat-Bone_marrow-1d-dn	12/306	0,001841422	0,022485335	0	0	2,69523617	16,9724872	EEF1B2;RPS17;RPL32;RPS8;RPS5;RPS6;ARL6IP5;RPL35A;RPS20;RPSA;RPS3A;DUSP14
Cortisone-206_mg/kg_in_CMC-Rat-Liver-3d-dn	12/306	0,001841422	0,022450528	0	0	2,69523617	16,9724872	EEF1B2;RPL32;RPS19;RPS8;RPS5;RPS6;ARL6IP5;RPS20;RPS3A;RPL10A;SEPP1;TPT1
Coumarin-111_mg/kg_in_Corn_Oil-Rat-Bone_marrow-3d-up	12/306	0,001841422	0,022415829	0	0	2,69523617	16,9724872	C1QA;RPS19;CLP1;RPS8;RPS5;RPS6;NKG7;RPL35A;RPS20;RPS3A;RPL10A;RPL39
Cyclophosphamide-25_mg/kg_in_Water-Rat-Bone_marrow-3d-up	12/306	0,001841422	0,022381237	0	0	2,69523617	16,9724872	EEF1B2;C1QA;RPS17;RPL32;RPS19;RPS5;RPS6;NKG7;RPL35A;RPS20;RPL10A;SEPP1
Dexamethasone-1_mg/kg_in_Corn_Oil-Rat-Liver-1d-up	12/306	0,001841422	0,022346751	0	0	2,69523617	16,9724872	EEF1B2;RPL32;RPS19;RPS8;FEM1B;RPS6;RPS20;RPSA;RPS3A;RPL10A;RPL39;TPT1
Doxofylline-483_mg/kg_in_Water-Rat-Heart-1d-up	12/306	0,001841422	0,022312371	0	0	2,69523617	16,9724872	RPS17;RPL32;RPS19;ARL2BP;RPS6;ARL6IP5;RPS20;RPSA;RAPGEF5;RPL10A;SEPP1;TPT1
Metoclopramide-185_mg/kg_in_CMC-Rat-Intestine-1d-dn	12/306	0,001841422	0,022278097	0	0	2,69523617	16,9724872	EEF1B2;C1QA;RPS17;RPL32;RPS19;RPL35A;RPS20;RPSA;RPS3A;RPL10A;SEPP1;RPL39
NN'-Diphenyl-P-Phenylenediamine-1000_mg/kg_in_CMC-Rat-Kidney-3d-dn	12/306	0,001841422	0,022243928	0	0	2,69523617	16,9724872	RPS19;ARL2BP;RPS8;FEM1B;RPS6;ARL6IP5;RPS20;RPSA;RPL10A;RPL39;TPT1;PDRG1
Penicillamine-852_mg/kg_in_Water-Rat-Bone_marrow-1d-dn	12/306	0,001841422	0,022209864	0	0	2,69523617	16,9724872	EEF1B2;RPS17;RPL32;RPS8;RPS5;RPS6;ARL6IP5;RPL35A;RPS20;RPSA;RPS3A;DUSP14
Phenelzine-27_mg/kg_in_Water-Rat-Heart-5d-up	12/306	0,001841422	0,022175904	0	0	2,69523617	16,9724872	EEF1B2;RPS17;RPL32;ARL2BP;CLP1;RPS8;RPS20;RPS3A;RAPGEF5;RPL39;GOLGA7;TPT1
Pyrilamine-688_mg/kg_in_Water-Rat-Heart-3d-dn	12/306	0,001841422	0,022142048	0	0	2,69523617	16,9724872	EEF1B2;RPS17;RPL32;RPS19;RPS8;RPS6;RPS20;RPSA;RPS3A;RPL10A;RPL39;TPT1
1-Naphthyl_Isothiocyanate-30_mg/kg_in_Corn_Oil-Rat-Bone_marrow-3d-up	12/307	0,001892051	0,022716148	0	0	2,686456899	16,84433703	EEF1B2;C1QA;RPS17;RPL32;RPS8;RPS5;RPS6;RPL35A;RPS20;RPL39;SLC16A3;TPT1
4-Methylpyrazole-155_mg/kg_in_Saline-Rat-Liver-3d-up	12/307	0,001892051	0,022681572	0	0	2,686456899	16,84433703	C1QA;RPS17;RPL32;RPS19;RPS5;RPS6;ARL6IP5;RPL35A;RPS20;RPSA;RPL10A;RPL39
Bacitracin-380_mg/kg_in_Corn_Oil-Rat-Kidney-1d-up	12/307	0,001892051	0,022647102	0	0	2,686456899	16,84433703	EEF1B2;RPS17;CXCL11;RPL32;RPS8;RPS5;RPS6;RPSA;RPS3A;RAPGEF5;SEPP1;RPL39
Candesartan-1300_mg/kg_in_CMC-Rat-Heart-1d-dn	12/307	0,001892051	0,022612736	0	0	2,686456899	16,84433703	RPS17;RPL32;RPS19;RPS8;COL5A3;RPS5;RPS6;ARL6IP5;RPSA;RPS3A;SEPP1;TPT1
Cyclandelate-1500_mg/kg_in_CMC-Rat-Liver-1d-up	12/307	0,001892051	0,022578474	0	0	2,686456899	16,84433703	EEF1B2;RPS17;RPL32;RPS6;RPL35A;RPS20;RPSA;RPS3A;RPL10A;SEPP1;RPL39;TPT1
Hydrocortisone-56_mg/kg_in_Corn_Oil-Rat-Bone_marrow-3d-dn	12/307	0,001892051	0,022544316	0	0	2,686456899	16,84433703	RPS17;RPS19;FEM1B;RPS6;NKG7;RPS20;RPSA;RPS3A;RPL10A;SEPP1;RPL39;TPT1
Hydroxyurea-400_mg/kg_in_Saline-Rat-Bone_marrow-5d-up	12/307	0,001892051	0,022510261	0	0	2,686456899	16,84433703	EEF1B2;C1QA;RPL32;RPS19;ARL2BP;RPS8;RPS6;RPS20;RPS3A;SEPP1;RPL39;TPT1
Hydroxyurea-59_mg/kg_in_Saline-Rat-Spleen-3d-dn	12/307	0,001892051	0,022476309	0	0	2,686456899	16,84433703	EEF1B2;C1QA;RPS17;RPL32;ARL2BP;RPS8;RPS6;ARL6IP5;RPSA;RPS3A;RPL39;TPT1
Leflunomide-60_mg/kg_in_Corn_Oil-Rat-Bone_marrow-5d-up	12/307	0,001892051	0,022442459	0	0	2,686456899	16,84433703	EEF1B2;C1QA;RPL32;RPS19;RPS5;NKG7;RPL35A;RPS20;RPL10A;SEPP1;RPL39;GOLGA7
Oxfendazole-1500_mg/kg_in_CMC-Rat-Liver-3d-up	12/307	0,001892051	0,022408711	0	0	2,686456899	16,84433703	EEF1B2;RPS17;CXADR;RPL32;RPS19;RPS5;RPS6;RPL35A;RPS20;RPSA;RPL10A;RPL39
Tenidap-75_mg/kg_in_Corn_Oil-Rat-Kidney-5d-up	12/307	0,001892051	0,022375064	0	0	2,686456899	16,84433703	RPS17;RPL32;RPS19;ARL2BP;RPS5;RPS6;RPL35A;RPS20;RPSA;RPS3A;RPL10A;RPL39
Oxaliplatin-8_mg/kg_in_Saline-Rat-Bone_marrow-3d-dn	11/267	0,001922967	0,022706582	0	0	2,831512156	17,70795378	EEF1B2;RPS17;RPL32;RPS8;FEM1B;FUNDC1;RPS6;RPS20;RPS3A;RPL39;TPT1
Doxorubicin-1_mg/kg_in_Saline-Rat-Kidney-5d-dn	12/308	0,001943817	0,022918419	0	0	2,677734636	16,71736952	EEF1B2;RPS17;RPL32;RPS19;RPS8;RPS5;RPS6;ARL6IP5;RPSA;RPS3A;SEPP1;TPT1
TNF-alpha-0.25_mg/kg_in_RSA/Saline-Rat-Liver-1d-up	12/308	0,001943817	0,022884161	0	0	2,677734636	16,71736952	EEF1B2;RPS17;RPL32;RPS19;RPS8;RPS6;RPSA;RPS3A;RPL10A;SEPP1;RPL39;TPT1
Aminocaproic_Acid-2000_mg/kg_in_Water-Rat-Kidney-5d-up	13/350	0,001951675	0,022942379	0	0	2,552773687	15,92692651	RPL32;RPS5;RPL13A;RPL35A;RPSA;RPS3A;SEPP1;TOMM20;EEF1B2;RPS17;ARL6IP5;RPS20;RPL39
Camptothecin-2.5_mg/kg_in_Saline-Rat-Bone_marrow-1d-up	13/350	0,001951675	0,022908188	0	0	2,552773687	15,92692651	C1QA;RPL32;RPS5;NKG7;RPL35A;RPSA;RPL10A;SEPP1;EEF1B2;RPS17;RPS19;RPL39;TPT1
Idarubicin-4_mg/kg_in_Saline-Rat-Heart-14d-up	11/268	0,001979999	0,023206055	0	0	2,820946812	17,55943231	EEF1B2;C1QA;RPS19;ARL2BP;RPS8;RPS6;RPS3A;RAPGEF5;RPL10A;GOLGA7;TPT1
Ethylene_Glycol-352_mg/kg_in_Water-Rat-Kidney-3d-dn	9/191	0,001987352	0,023257633	0	0	3,238516759	20,14665737	RPL32;RPS19;ARL2BP;RPS8;RPS6;RPSA;RPL10A;TPT1;PDRG1
Bromfenac-5_mg/kg_in_CMC-Rat-Liver-1d-up	12/309	0,00199674	0,023332826	0	0	2,669068829	16,59157085	EEF1B2;RPL32;RPS19;RPS8;RPS5;RPS6;RPS20;RPSA;RPS3A;RPL10A;RPL39;TPT1
Diclofenac-3.5_mg/kg_in_Corn_Oil-Rat-Liver-0.25d-dn	12/309	0,00199674	0,023298259	0	0	2,669068829	16,59157085	EEF1B2;C1QA;RPS17;RPL32;RPS8;RPL35A;RPS20;RPSA;RPS3A;SEPP1;RPL39;TPT1
Doxorubicin-0.65_mg/kg_in_Saline-Rat-Liver-3d-up	12/309	0,00199674	0,023263794	0	0	2,669068829	16,59157085	EEF1B2;RPS17;RPL32;RPS19;RPS5;RPS6;RPL35A;RPS20;RPS3A;RPL10A;SEPP1;RPL39
Ethanol-3000_mg/kg_in_Saline-Rat-Liver-5d-dn	12/309	0,00199674	0,023229431	0	0	2,669068829	16,59157085	EEF1B2;RPS17;RPL32;RPS19;CLP1;RPS8;RPS6;RPL35A;RPS20;RPSA;RPS3A;TPT1
Ethosuximide-1200_mg/kg_in_Water-Rat-Brain-5d-up	12/309	0,00199674	0,023195169	0	0	2,669068829	16,59157085	EEF1B2;C1QA;RPS17;RPL32;RPS19;ARL2BP;RPS6;ARL6IP5;RPS20;RPS3A;GOLGA7;TPT1
Methylparaben-1500_mg/kg_in_CMC-Rat-Liver-3d-up	12/309	0,00199674	0,023161008	0	0	2,669068829	16,59157085	RPL32;RPS19;RPS5;RPS6;RPL35A;RPS20;RPSA;RPS3A;RPL10A;SEPP1;RPL39;GOLGA7
Raloxifene-650_mg/kg_in_Corn_Oil-Rat-Liver-3d-up	13/351	0,002000964	0,02317587	0	0	2,545500827	15,81806355	RPL32;RPS8;RPS5;RPS6;RPL35A;RPSA;RPS3A;RPL10A;EEF1B2;RPS17;RPS19;RPL39;TPT1
Acyclovir-980_mg/kg_in_Saline-Rat-Bone_marrow-3d-dn	11/269	0,0020384	0,023574798	0	0	2,810460021	17,41245841	EEF1B2;C1QA;RPL32;RPS19;RPS8;RPS6;ARL6IP5;RPS20;RPSA;RPL10A;SEPP1
Azathioprine-20_mg/kg_in_Corn_Oil-Rat-Liver-5d-dn	11/269	0,0020384	0,023540231	0	0	2,810460021	17,41245841	C1QA;RPS17;RPL32;RPS8;RPS6;ARL6IP5;RPS20;RPSA;RPS3A;RPL10A;SEPP1
Diethylstilbestrol-280_mg/kg_in_Corn_Oil-Rat-Liver-3d-up	11/269	0,0020384	0,023505765	0	0	2,810460021	17,41245841	EEF1B2;RPS17;RPS19;RPS8;FEM1B;RPS6;RPS20;RPSA;RPS3A;RPL10A;TPT1
Indomethacin-4.5_mg/kg_in_Water-Rat-Kidney-3d-dn	11/269	0,0020384	0,0234714	0	0	2,810460021	17,41245841	EEF1B2;RPS17;RPL32;RPS19;RPS8;FUNDC1;RPS6;RPSA;RPL10A;RPL39;SEPP1
Ethylene_Glycol-3525_mg/kg_in_Water-Rat-Bone_marrow-3d-dn	12/310	0,002050839	0,023580157	0	0	2,660458929	16,46692739	RPS19;ARL2BP;CLP1;RPS8;FEM1B;FUNDC1;RPS6;RPS20;RPSA;RPS3A;RPL39;TPT1
Ketoconazole-227_mg/kg_in_Corn_Oil-Rat-Liver-1d-up	12/310	0,002050839	0,023545783	0	0	2,660458929	16,46692739	EEF1B2;RPS17;RPL32;RPS19;RPS8;RPS5;RPS6;RPSA;RPS3A;RPL10A;SEPP1;RPL39
Olanzapine-23_mg/kg_in_CMC-Rat-Liver-1d-up	12/310	0,002050839	0,02351151	0	0	2,660458929	16,46692739	EEF1B2;RPS17;RPL32;RPS19;RPS5;RPS6;RPL35A;RPS20;RPSA;RPS3A;RPL39;TPT1
Terfenadine-750_mg/kg_in_CMC-Rat-Heart-3d-up	12/310	0,002050839	0,023477336	0	0	2,660458929	16,46692739	EEF1B2;RPL32;ARL2BP;RPS8;RPS6;RPS20;RPS3A;RAPGEF5;RPL10A;SEPP1;RPL39;TPT1
Zileuton-450_mg/kg_in_Corn_Oil-Rat-Kidney-1d-up	12/310	0,002050839	0,023443262	0	0	2,660458929	16,46692739	RPS17;RPL32;RPS19;RPS8;RPS5;RPS6;RPS20;RPSA;RPS3A;RAPGEF5;RPL10A;TOMM20
Dexfenfluramine-29_mg/kg_in_Water-Rat-Kidney-5d-dn	10/230	0,002065552	0,023577231	0	0	2,988196623	18,47410029	RPS17;RPL32;RPS8;RPS6;ARL6IP5;RPL35A;RPS20;RPSA;RPS3A;SEPP1
Rapamycin-60_mg/kg_in_CMC-Rat-Heart-5d-up	11/270	0,002098195	0,023915175	0	0	2,80005091	17,2670115	EEF1B2;RPL32;RPS19;ARL2BP;RPS8;RPS6;RPS20;RPSA;RPS3A;RPL10A;TPT1
Lead_(II)_Acetate-200_mg/kg_in_Saline-Rat-Kidney-1d-up	13/353	0,002102625	0,023931036	0	0	2,531078726	15,60300834	RPL32;RPS8;RPS5;RPL13A;RPL35A;RPSA;RPS3A;TOMM20;RPS17;ARL6IP5;RPS20;RPL39;TPT1
123-Trichloropropane-108_mg/kg_in_CMC-Rat-Heart-5d-up	12/311	0,002106133	0,023936373	0	0	2,651904399	16,34342573	EEF1B2;RPS17;RPL32;ARL2BP;RPS8;ARL6IP5;RPS20;RPSA;RPS3A;RPL10A;RPL39;TOMM20
Ceramide_C2-44_uM_in_DMSO-Rat-Primary_rat_hepatocytes-0.67d-up	12/311	0,002106133	0,023901882	0	0	2,651904399	16,34342573	ARL2BP;RPS8;RPS5;RPS6;ARL6IP5;RPL35A;RPS3A;RPL10A;RPL39;GOLGA7;TOMM20;PDRG1
Gentamicin-267_mg/kg_in_Corn_Oil-Rat-Kidney-5d-up	12/311	0,002106133	0,023867491	0	0	2,651904399	16,34342573	C1QA;RPS17;CXCL11;RPL32;RPS19;RPS8;FEM1B;RPS6;RPS3A;RPL10A;RPL39;PDRG1
Nitrofurantoin-76_mg/kg_in_CMC-Rat-Liver-1d-dn	12/311	0,002106133	0,023833199	0	0	2,651904399	16,34342573	EEF1B2;RPS17;RPL32;RPS19;RPS8;RPS5;ARL6IP5;RPL35A;RPS20;RPSA;RPS3A;RPL10A
Progesterone-11.3_mg/kg_in_Corn_Oil-Rat-Liver-0.25d-up	12/311	0,002106133	0,023799005	0	0	2,651904399	16,34342573	EEF1B2;C1QA;RPS17;RPL32;RPS19;RPS8;FEM1B;RPS6;RPS20;RPS3A;RPL10A;SEPP1
Streptomycin-120_mg/kg_in_Saline-Rat-Bone_marrow-1d-dn	10/231	0,002131975	0,024056496	0	0	2,975260707	18,29995556	EEF1B2;RPS17;RPL32;RPS19;FUNDC1;NKG7;RPL35A;RPS20;RPSA;RPL39
Cadmium_Chloride-8_uM_in_DMSO-Rat-Primary_rat_hepatocytes-1d-dn	13/354	0,00215503	0,024281858	0	0	2,523928786	15,49679781	RPL32;FEM1B;RPS5;RPS6;RPS3A;RPL10A;SEPP1;EEF1B2;RPS17;RPS19;ARL2BP;RPL39;TPT1
Carmustine-16_mg/kg_in_Corn_Oil-Rat-Spleen-5d-up	11/271	0,002159409	0,024296439	0	0	2,789718619	17,12307135	C1QA;CXCL11;RPL32;RPS19;CLP1;RPS8;FUNDC1;RPS20;RPS3A;SEPP1;TPT1
Chlorambucil-4.5_mg/kg_in_Corn_Oil-Rat-Liver-5d-up	11/271	0,002159409	0,024261779	0	0	2,789718619	17,12307135	EEF1B2;RPS17;RPL32;RPS19;RPS8;RPS6;RPS20;RPSA;RPS3A;RPL10A;RPL39
Beta-Estradiol-0.3_mg/kg_in_Corn_Oil-Rat-Liver-1d-up	12/312	0,002162643	0,024263498	0	0	2,643404705	16,22105264	EEF1B2;RPS17;RPL32;RPS19;RPS8;RPS6;RPS20;RPSA;RPL10A;SEPP1;RPL39;TPT1
Bithionol-59_mg/kg_in_Corn_Oil-Rat-Bone_marrow-3d-up	12/312	0,002162643	0,024228984	0	0	2,643404705	16,22105264	EEF1B2;C1QA;RPS17;RPL32;ARL2BP;RPS8;RPS5;RPL35A;RPS20;RPS3A;RPL39;TPT1
Calcitriol-0.04_mg/kg_in_CMC-Rat-Kidney-3d-up	12/312	0,002162643	0,024194568	0	0	2,643404705	16,22105264	EEF1B2;C1QA;CXCL11;RPS19;RPS8;RPS6;RPS20;RPSA;RPS3A;RPL10A;RPL39;TPT1
Citric_Acid-3000_mg/kg_in_Water-Rat-Liver-1d-up	12/312	0,002162643	0,02416025	0	0	2,643404705	16,22105264	EEF1B2;C1QA;RPS17;RPL32;RPS19;RPS8;RPS5;RPS6;RPL35A;RPS20;RPSA;RPS3A
Cyclosporin_A-350_mg/kg_in_Corn_Oil-Rat-Bone_marrow-3d-up	12/312	0,002162643	0,024126028	0	0	2,643404705	16,22105264	EEF1B2;C1QA;RPL32;RPS8;RPS5;RPS6;NKG7;RPL35A;RPS20;RPL10A;RPL39;TPT1
Cytarabine-487_mg/kg_in_Saline-Rat-Bone_marrow-3d-dn	12/312	0,002162643	0,024091904	0	0	2,643404705	16,22105264	EEF1B2;RPS17;RPL32;RPS8;FEM1B;RPS6;NKG7;RPS20;RPSA;RPS3A;RPL39;TPT1
Erythromycin-1500_mg/kg_in_CMC-Rat-Liver-3d-up	12/312	0,002162643	0,024057876	0	0	2,643404705	16,22105264	RPL32;RPS19;RPS8;RPS5;RPS6;RPL35A;RPS20;RPSA;RPS3A;RPL10A;RPL39;TPT1
Gemfibrozil-100_mg/kg_in_Corn_Oil-Rat-Kidney-1d-dn	12/312	0,002162643	0,024023943	0	0	2,643404705	16,22105264	EEF1B2;RPS17;RPL32;RPS19;RPS6;RPL35A;RPS20;RPSA;RPS3A;RPL10A;SEPP1;RPL39
Isoeugenol-1560_mg/kg_in_CMC-Rat-Liver-5d-dn	12/312	0,002162643	0,023990107	0	0	2,643404705	16,22105264	EEF1B2;C1QA;RPS17;RPL32;RPS8;RPS5;RPL35A;RPS20;RPSA;RPS3A;RPL10A;TPT1
Mevastatin-1200_mg/kg_in_CMC-Rat-Kidney-3d-dn	12/312	0,002162643	0,023956366	0	0	2,643404705	16,22105264	RPL32;RPS19;ARL2BP;CLP1;RPS8;FEM1B;RPS6;RPS20;RPSA;RPL10A;RPL39;TPT1
Salicylamide-1300_mg/kg_in_CMC-Rat-Kidney-5d-dn	12/312	0,002162643	0,023922719	0	0	2,643404705	16,22105264	EEF1B2;RPS17;RPL32;RPS19;RPS8;RPS5;RPS6;RPL35A;RPS20;RPSA;RPL10A;SEPP1
Sodium_Orthovanadate-83_mg/kg_in_CMC-Rat-Kidney-5d-up	12/312	0,002162643	0,023889167	0	0	2,643404705	16,22105264	EEF1B2;C1QA;RPL32;RPS19;RPS8;RPS6;RPS20;RPSA;RAPGEF5;RPL10A;RPL39;TPT1
NN-Dimethylformamide-140_mg/kg_in_Saline-Rat-Bone_marrow-1d-up	10/232	0,002200108	0,024268977	0	0	2,962436308	18,12788505	EEF1B2;C1QA;RPS17;RPS19;RPS8;RPS6;RPS20;RPS3A;RPL39;TPT1
1-Naphthyl_Isothiocyanate-60_mg/kg_in_Corn_Oil-Rat-Liver-0.25d-up	12/313	0,002220388	0,024458424	0	0	2,634959323	16,0997951	EEF1B2;RPS17;RPL32;RPS19;RPS8;RPS6;RPL35A;RPS20;RPS3A;RPL10A;SEPP1;RPL39
Bezafibrate-100_mg/kg_in_Corn_Oil-Rat-Kidney-3d-up	12/313	0,002220388	0,024424264	0	0	2,634959323	16,0997951	EEF1B2;RPS17;RPL32;RPS19;RPS8;RPS5;RPL35A;RPS20;RPS3A;RPL39;TPT1;PDRG1
Clarithromycin-476_mg/kg_in_Water-Rat-Liver-3d-up	12/313	0,002220388	0,024390199	0	0	2,634959323	16,0997951	EEF1B2;RPS17;RPL32;RPS19;RPS8;RPS5;RPS6;RPL35A;RPS20;RPS3A;RPL10A;TPT1

Epirubicin-2.7_mg/kg_in_Saline-Rat-Heart-1d-dn	12/313	0,002220388	0,02435623	0	0	2,634959323	16,0997951	EEF1B2;RPS17;RPL32;RPS8;COL5A3;ARL6IP5;RPS20;RPSA;RPS3A;RPL39;GOLGA7;TPT1
Methotrexate-0.3_mg/kg_in_Corn_Oil-Rat-Liver-0.25d-up	12/313	0,002220388	0,024322355	0	0	2,634959323	16,0997951	EEF1B2;RPS17;CXADR;RPL32;RPS19;RPS6;ARL6IP5;RPS20;RPS3A;RPL10A;SEPP1;TPT1
Piclamilast-17_mg/kg_in_CMC-Rat-Heart-3d-up	12/313	0,002220388	0,024288574	0	0	2,634959323	16,0997951	EEF1B2;RPS8;RPS5;FUNDC1;ARL6IP5;RPS20;RPSA;RPS3A;RAPGEF5;RPL10A;RPL39;TOMM20
Secobarbital-70_mg/kg_in_Water-Rat-Liver-1d-up	12/313	0,002220388	0,024254886	0	0	2,634959323	16,0997951	EEF1B2;RPS17;RPL32;RPS19;RPS8;RPS5;RPS6;RPL35A;RPSA;RPS3A;RPL39;TOMM20
Beta-Estradiol_3-Benzoate-25_mg/kg_in_Corn_Oil-Rat-Liver-3d-up	11/272	0,002222067	0,024239607	0	0	2,7794623	16,98061805	EEF1B2;RPS17;RPS19;RPS8;RPS6;RPS20;RPSA;RPS3A;RPL10A;RPL39;TPT1
Staurosporine-1.3_uM_in_DMSO-Rat-Primary_rat_hepatocytes-1d-up	11/272	0,002222067	0,02420608	0	0	2,7794623	16,98061805	EEF1B2;RPS17;CXCL11;RPL32;RPS5;RPS20;RPSA;RPS3A;RPL10A;GOLGA7;SEPP1
Sulindac-132_mg/kg_in_Corn_Oil-Rat-Heart-5d-up	11/272	0,002222067	0,024172647	0	0	2,7794623	16,98061805	EEF1B2;C1QA;RPS17;RPS19;RPS8;RPS6;RPS20;RPS3A;RPL10A;RPL39;TPT1
Chlorambucil-0.6_mg/kg_in_Corn_Oil-Rat-Spleen-0.25d-up	12/314	0,002279387	0,024762008	0	0	2,626567733	15,97964024	EEF1B2;C1QA;RPS17;RPL32;RPS5;RPS6;RPSA;RPS3A;RPL10A;SEPP1;RPL39;TPT1
Citalopram-40_mg/kg_in_Corn_Oil-Rat-Liver-5d-up	12/314	0,002279387	0,0247279	0	0	2,626567733	15,97964024	EEF1B2;RPS17;RPL32;RPS8;RPS5;RPS6;RPL35A;RPS20;RPSA;RPL10A;RPL39;TPT1
Disulfiram-500_mg/kg_in_Water-Rat-Liver-3d-up	12/314	0,002279387	0,024693887	0	0	2,626567733	15,97964024	EEF1B2;RPS17;RPL32;RPS19;RPS5;RPS6;RPS20;RPSA;RPS3A;RPL10A;SEPP1;RPL39
NN-Dimethylformamide-1400_mg/kg_in_Saline-Rat-Liver-3d-up	12/314	0,002279387	0,024659967	0	0	2,626567733	15,97964024	EEF1B2;RPS17;RPL32;RPS19;RPS6;RPS20;RPSA;RPS3A;RPL10A;SEPP1;RPL39;TPT1
Netilmicin-40_mg/kg_in_Saline-Rat-Kidney-28d-up	12/314	0,002279387	0,024626139	0	0	2,626567733	15,97964024	EEF1B2;C1QA;RPS17;RPL32;RPS19;RPS8;RPS20;RPSA;RPS3A;RPL10A;RPL39;TPT1
Vinblastine-0.3_mg/kg_in_Saline-Rat-Heart-5d-up	12/314	0,002279387	0,024592405	0	0	2,626567733	15,97964024	EEF1B2;C1QA;RPL32;RPS19;FUNDC1;RPS6;RPS20;RPSA;RPL10A;RPL39;TPT1;PDRG1
6-Mercaptopurine-25_mg/kg_in_Corn_Oil-Rat-Liver-5d-dn	11/273	0,002286192	0,024632078	0	0	2,76928112	16,83963204	EEF1B2;RPS17;RPL32;RPS8;RPS6;RPSA;RPS3A;RPL10A;RPL39;SEPP1;TPT1
Hydrocortisone-56_mg/kg_in_Corn_Oil-Rat-Heart-1d-dn	11/273	0,002286192	0,024598428	0	0	2,76928112	16,83963204	RPS17;RPL32;RPS19;ARL2BP;RPS8;RPS6;RPS20;RPS3A;RPL10A;RPL39;TPT1
Lead_(II)_Acetate-600_mg/kg_in_Saline-Rat-Liver-1d-dn	11/273	0,002286192	0,024564869	0	0	2,76928112	16,83963204	EEF1B2;RPL32;RPS19;RPS8;RPS5;RPS6;RPS20;RPSA;RPS3A;RPL10A;TPT1
4-Methylpyrazole-155_mg/kg_in_Saline-Rat-Kidney-5d-dn	12/315	0,002339663	0,025105154	0	0	2,618229422	15,8605754	EEF1B2;RPS17;RPS19;ARL2BP;RPS6;ARL6IP5;RPS20;RPS3A;RPL10A;SEPP1;RPL39;TPT1
Acetazolamide-47_mg/kg_in_Corn_Oil-Rat-Kidney-3d-up	12/315	0,002339663	0,025070998	0	0	2,618229422	15,8605754	EEF1B2;C1QA;RPS17;RPL32;RPS19;RPS8;RPS5;RPS6;RPL13A;RPL35A;RPSA;RPS3A
Guanethidine-132_mg/kg_in_CMC-Rat-Heart-5d-up	12/315	0,002339663	0,025036934	0	0	2,618229422	15,8605754	RPS17;CXCL11;RPL32;RPS5;ARL6IP5;RPL35A;RPS20;RPSA;RPS3A;RAPGEF5;TOMM20;TPT1
Ivermectin-7.5_mg/kg_in_CMC-Rat-Liver-3d-up	12/315	0,002339663	0,025002962	0	0	2,618229422	15,8605754	EEF1B2;RPS17;RPL32;RPS19;RPS6;RPL35A;RPS20;RPSA;RPS3A;RPL10A;SEPP1;RPL39
Nateglinide-625_mg/kg_in_CMC-Rat-Liver-3d-up	12/315	0,002339663	0,024969083	0	0	2,618229422	15,8605754	C1QA;RPS17;RPL32;RPS19;RPS6;RPL35A;RPS20;RPSA;RPS3A;RPL10A;SEPP1;RPL39
Sulbactam-671_mg/kg_in_Saline-Rat-Kidney-1d-up	13/358	0,002375528	0,02531753	0	0	2,495728465	15,08052844	RPL32;RPS6;RPL35A;RPSA;RPS3A;RPL10A;SEPP1;EEF1B2;RPS17;RPS19;ARL6IP5;RPL39;GOLGA7
Citric_Acid-3000_mg/kg_in_Water-Rat-Liver-5d-up	12/316	0,002401234	0,025556914	0	0	2,609943886	15,74258812	C1QA;RPS17;RPL32;RPS19;RPS8;RPS5;RPS6;RPL35A;RPSA;RPS3A;RPL10A;TPT1
Ergocalciferol-15_mg/kg_in_CMC-Rat-Liver-5d-dn	12/316	0,002401234	0,025522424	0	0	2,609943886	15,74258812	C1QA;RPS17;RPL32;RPS19;RPS8;RPS6;RPL35A;RPS20;RPSA;RPL10A;SEPP1;TPT1
Fluocinolone_Acetonide-2.5_mg/kg_in_Corn_Oil-Rat-Liver-5d-dn	12/316	0,002401234	0,025488028	0	0	2,609943886	15,74258812	EEF1B2;C1QA;RPS17;RPL32;RPS19;RPS8;RPS6;RPSA;RPS3A;RPL10A;SEPP1;RPL39
Ibuprofen-54_mg/kg_in_Corn_Oil-Rat-Liver-3d-dn	12/316	0,002401234	0,025453723	0	0	2,609943886	15,74258812	EEF1B2;C1QA;RPS17;RPL32;RPS19;RPS6;RPS3A;RPL10A;SEPP1;RPL39;GOLGA7;TPT1
Indomethacin-9.6_mg/kg_in_Water-Rat-Kidney-1d-up	12/316	0,002401234	0,025419511	0	0	2,609943886	15,74258812	C1QA;RPS17;RPL32;RPS19;RPS8;RPS5;RPS6;RPS20;RPS3A;RPL10A;GOLGA7;TPT1
Loperamide-47_mg/kg_in_CMC-Rat-Intestine-3d-dn	12/316	0,002401234	0,025385391	0	0	2,609943886	15,74258812	EEF1B2;C1QA;RPS17;RPS19;RPS8;RPS5;RPS20;RPSA;RPS3A;RPL10A;SEPP1;TPT1
Sildenafil-2.5_mg/kg_in_Water-Rat-Liver-3d-up	12/316	0,002401234	0,025351363	0	0	2,609943886	15,74258812	EEF1B2;C1QA;RPS17;RPL32;RPS19;RPS8;RPS6;ARL6IP5;RPS20;RPSA;RPS3A;SEPP1
Sotalol-2000_mg/kg_in_Water-Rat-Heart-1d-up	12/316	0,002401234	0,025317425	0	0	2,609943886	15,74258812	RPS17;CXCL11;RPL32;RPS19;RPS8;RPS6;ARL6IP5;RPS20;RPSA;RPS3A;RPL39;TPT1
Dexfenfluramine-29_mg/kg_in_Water-Rat-Kidney-3d-dn	10/235	0,002415089	0,025429464	0	0	2,924617972	17,62380421	RPS17;RPL32;RPS19;RPS6;ARL6IP5;RPL35A;RPS20;RPSA;RPL10A;RPL39
Amikacin-82_mg/kg_in_Saline-Rat-Bone_marrow-3d-dn	11/275	0,002418949	0,025436105	0	0	2,749140893	16,5619852	EEF1B2;RPS17;RPS19;ARL2BP;RPS8;FUNDC1;RPS6;RPL13A;RPS20;RPSA;RPL10A
Benzoic_Acid-1700_mg/kg_in_CMC-Rat-Liver-3d-dn	11/275	0,002418949	0,02540219	0	0	2,749140893	16,5619852	EEF1B2;C1QA;RPS17;RPL32;CLP1;RPS8;RPS5;RPL35A;RPS3A;SEPP1;TPT1
Alfacalcidol-0.043_mg/kg_in_CMC-Rat-Heart-3d-dn	12/317	0,002464121	0,025842101	0	0	2,601710625	15,62566607	EEF1B2;RPS17;RPL32;RPS19;RPS8;FUNDC1;RPS6;RPS20;RPSA;RPS3A;SEPP1;TPT1
Clindamycin-161_mg/kg_in_Saline-Rat-Kidney-5d-up	12/317	0,002464121	0,025807737	0	0	2,601710625	15,62566607	EEF1B2;RPS17;RPL32;RPS19;ARL6IP5;RPL13A;RPL35A;RPS20;RPSA;RPS3A;RPL39;TPT1
Cortisone-206_mg/kg_in_CMC-Rat-Heart-3d-up	12/317	0,002464121	0,025773464	0	0	2,601710625	15,62566607	EEF1B2;RPL32;RPS19;ARL2BP;CLP1;RPS8;RPL35A;RPS20;RPSA;RPS3A;RPL10A;SEPP1
Mitoxantrone-2_mg/kg_in_Saline-Rat-Heart-1d-up	12/317	0,002464121	0,025739281	0	0	2,601710625	15,62566607	C1QA;RPS17;RPL32;RPS19;ARL2BP;RPS8;RPS6;RPS20;RAPGEF5;RPL10A;TPT1;PDRG1
NN-Dimethylformamide-65000_uM_in_DMSO-Rat-Primary_rat_hepatocytes-0.67d-up	12/317	0,002464121	0,025705189	0	0	2,601710625	15,62566607	CXCL11;RPS19;ARL2BP;CLP1;FEM1B;ARL6IP5;RPSA;RPL10A;SEPP1;RPL39;TPT1;PDRG1
Pyrazinamide-1500_mg/kg_in_CMC-Rat-Liver-1d-up	12/317	0,002464121	0,025671188	0	0	2,601710625	15,62566607	EEF1B2;RPS17;RPL32;RPS19;RPS8;RPS5;RPS6;RPL35A;RPS20;RPS3A;RPL10A;TPT1
Sotalol-2000_mg/kg_in_Water-Rat-Liver-1d-dn	12/317	0,002464121	0,025637276	0	0	2,601710625	15,62566607	RPS17;RPL32;RPS19;RPS8;RPS5;RPS6;RPL35A;RPS20;RPS3A;RPL10A;SEPP1;RPL39
Doxorubicin-3_mg/kg_in_Saline-Rat-Bone_marrow-3d-dn	11/276	0,002487631	0,025847738	0	0	2,739180238	16,42528679	EEF1B2;RPS17;RPL32;RPS8;FEM1B;RPS6;RPSA;RPS3A;RPL10A;RPL39;TPT1
Indomethacin-9.6_mg/kg_in_Water-Rat-Heart-5d-up	11/276	0,002487631	0,025813683	0	0	2,739180238	16,42528679	EEF1B2;C1QA;RPL32;RPS19;RPS8;RPS6;ARL6IP5;RPS20;RPSA;RPL10A;RPL39
4-Methylpyrazole-155_mg/kg_in_Saline-Rat-Liver-5d-up	12/318	0,002528346	0,026201647	0	0	2,593529145	15,50979713	C1QA;RPS17;RPL32;RPS19;RPS8;RPS5;RPS6;RPL35A;RPS20;RPSA;RPS3A;RPL39
Cytarabine-487_mg/kg_in_Saline-Rat-Kidney-1d-dn	12/318	0,002528346	0,026167216	0	0	2,593529145	15,50979713	EEF1B2;C1QA;RPS17;RPL32;RPS5;RPS6;RPL35A;RPS20;RPS3A;RPL10A;RPL39;GOLGA7
DActinomycin-0.06_mg/kg_in_Saline-Rat-Bone_marrow-1d-up	12/318	0,002528346	0,026132876	0	0	2,593529145	15,50979713	C1QA;RPS17;RPL32;RPS19;FEM1B;RPS5;RPS6;ARL6IP5;RPSA;RPS3A;RPL10A;RPL39
Hydroxyurea-59_mg/kg_in_Saline-Rat-Spleen-0.25d-dn	12/318	0,002528346	0,026098626	0	0	2,593529145	15,50979713	EEF1B2;CXCL11;RPL32;RPS19;RPS6;RPS20;RPSA;RPS3A;RPL10A;SEPP1;RPL39;TPT1
Spironolactone-300_mg/kg_in_CMC-Rat-Liver-1d-dn	12/318	0,002528346	0,026064465	0	0	2,593529145	15,50979713	EEF1B2;RPS17;RPL32;RPS19;RPS8;RPS6;RPL35A;RPS20;RPS3A;RPL10A;SEPP1;RPL39
Diphenidol-300_mg/kg_in_Water-Rat-Heart-3d-up	13/361	0,002552791	0,026282068	0	0	2,474988339	14,77708621	RPL32;RPS8;RPS6;RPS3A;EEF1B2;RPS17;RPS19;ARL2BP;FUNDC1;ARL6IP5;RPS20;RAPGEF5;GOLGA7
Ethanol-6000_mg/kg_in_Saline-Rat-Bone_marrow-3d-dn	11/277	0,002557884	0,026300126	0	0	2,729291501	16,28998054	EEF1B2;C1QA;RPS17;RPL32;ARL2BP;RPS8;RPS6;NKG7;RPL35A;RPS3A;TPT1
Ethinylestradiol-1480_mg/kg_in_Corn_Oil-Rat-Liver-5d-up	11/277	0,002557884	0,026265836	0	0	2,729291501	16,28998054	RPS17;RPL32;RPS19;RPS8;FEM1B;RPS5;RPS6;ARL6IP5;RPS20;RPSA;RPS3A
Mitomycin_C-0.5_mg/kg_in_Saline-Rat-Spleen-3d-dn	11/277	0,002557884	0,026231636	0	0	2,729291501	16,28998054	EEF1B2;RPS17;RPL32;ARL2BP;RPS8;RPS5;FUNDC1;RPS6;ARL6IP5;RPSA;RPL10A
N-Nitrosodiethylamine-1.67_mg/kg_in_Saline-Rat-Kidney-3d-up	11/277	0,002557884	0,026197525	0	0	2,729291501	16,28998054	C1QA;RPS17;RPL32;RPS19;ARL2BP;ARL6IP5;RPL35A;RPS20;RPS3A;GOLGA7;SEPP1
Clemastine-5.4_mg/kg_in_CMC-Rat-Heart-5d-up	12/319	0,002593929	0,026532184	0	0	2,585398959	15,39496934	EEF1B2;RPS17;RPL32;RPS19;RPS6;RPL35A;RPS20;RPSA;RPS3A;RPL10A;RPL39;TPT1
Fludrocortisone_Acetate-125_mg/kg_in_CMC-Rat-Kidney-1d-dn	12/319	0,002593929	0,026497772	0	0	2,585398959	15,39496934	RPS17;RPL32;RPS19;ARL2BP;CLP1;RPS8;RPS5;RPL35A;RPS20;RPSA;RPS3A;SEPP1
Trihexyphenidyl-588_mg/kg_in_CMC-Rat-Heart-1d-dn	12/319	0,002593929	0,026463448	0	0	2,585398959	15,39496934	EEF1B2;RPS17;RPL32;RPS8;ARL6IP5;RPS20;RPSA;RPS3A;RPL10A;RPL39;TPT1;PDRG1
Cyclosporin_A-70_mg/kg_in_Corn_Oil-Rat-Liver-3d-up	13/362	0,002614235	0,02663611	0	0	2,468151355	14,67756261	C1QA;RPL32;RPS8;RPS5;RPS6;RPL35A;RPSA;RPL10A;EEF1B2;RPS19;RPS20;RPL39;TPT1
Fluvastatin-94_mg/kg_in_Corn_Oil-Rat-Kidney-3d-up	13/362	0,002614235	0,026601697	0	0	2,468151355	14,67756261	RPL32;RPS8;RPS5;RPS6;RPL13A;RPL35A;RPS3A;TOMM20;EEF1B2;RPS17;RPS19;ARL6IP5;RPS20
Levofloxacin-739_mg/kg_in_Water-Rat-Kidney-3d-dn	9/199	0,002621779	0,026644036	0	0	3,108325131	18,47558102	RPS17;RPL32;RPS19;RPS6;RPL35A;RPSA;RPS3A;RPL10A;RPL39
Promethazine-2.3_mg/kg_in_Saline-Rat-Liver-1d-dn	11/278	0,002629734	0,02669044	0	0	2,719473905	16,15604842	EEF1B2;RPS17;RPL32;RPS8;RPS5;RPS6;RPS20;RPS3A;RPL10A;RPL39;SEPP1
Nateglinide-625_mg/kg_in_CMC-Rat-Liver-1d-up	12/320	0,002660891	0,026971913	0	0	2,577319588	15,28117092	RPS17;RPL32;RPS8;RPS5;RPS6;RPL35A;RPS20;RPSA;RPS3A;RPL10A;SEPP1;RPL39
Norethindrone-125_uM_in_DMSO-Rat-Primary_rat_hepatocytes-1d-up	12/320	0,002660891	0,026937244	0	0	2,577319588	15,28117092	EEF1B2;RPS17;CXCL11;RPL32;RPS8;RPS5;RPS6;RPL35A;RPS3A;RPL10A;SEPP1;PDRG1
Rosiglitazone-10_mg/kg_in_Corn_Oil-Rat-Liver-1d-up	12/320	0,002660891	0,026902665	0	0	2,577319588	15,28117092	EEF1B2;C1QA;RPL32;RPS19;RPS8;RPS5;RPS20;RPSA;RPS3A;RPL10A;SEPP1;GOLGA7
Etoposide-188_mg/kg_in_Corn_Oil-Rat-Liver-3d-up	13/363	0,002676886	0,027029681	0	0	2,46135204	14,57883733	C1QA;RPL32;RPS8;RPS5;RPS6;RPL35A;RPSA;RPL10A;EEF1B2;RPS19;RPS20;RPL39;TPT1
6-Methoxy-2-Naphthylacetic_Acid-360_mg/kg_in_Saline-Rat-Kidney-0.25d-up	11/279	0,002703206	0,027260497	0	0	2,709726687	16,02347268	EEF1B2;RPS17;RPL32;RPS19;RPS8;RPS5;RPL35A;RPS20;RPSA;RPS3A;TOMM20
Carboplatin-14_mg/kg_in_Saline-Rat-Spleen-3d-dn	11/279	0,002703206	0,027225637	0	0	2,709726687	16,02347268	EEF1B2;C1QA;RPS17;RPL32;ARL2BP;RPS8;RPS6;ARL6IP5;RPSA;RPS3A;TPT1
Hydrocortisone-56_mg/kg_in_Corn_Oil-Rat-Heart-3d-dn	11/279	0,002703206	0,027190866	0	0	2,709726687	16,02347268	RPS17;RPL32;RPS19;ARL2BP;RPS8;RPS6;RPS20;RPS3A;RPL39;GOLGA7;TPT1
Levofloxacin-739_mg/kg_in_Water-Rat-Heart-1d-up	11/279	0,002703206	0,027156183	0	0	2,709726687	16,02347268	EEF1B2;RPS17;RPL32;RPS19;ARL2BP;RPS8;RPS6;RPS20;RPS3A;RPL39;TPT1
Sulindac-132_mg/kg_in_Corn_Oil-Rat-Liver-5d-up	11/279	0,002703206	0,02712159	0	0	2,709726687	16,02347268	EEF1B2;RPS17;RPL32;RPS19;RPS5;RPS6;RPS20;RPSA;RPS3A;RPL10A;RPL39
Warfarin-0.25_mg/kg_in_CMC-Rat-Liver-3d-up	11/279	0,002703206	0,027087084	0	0	2,709726687	16,02347268	C1QA;RPS17;RPL32;RPS6;RPL35A;RPS20;RPSA;RPS3A;RAPGEF5;RPL10A;RPL39
Diflunisal-207_mg/kg_in_Corn_Oil-Rat-Kidney-1d-up	12/321	0,002729254	0,027313344	0	0	2,569290555	15,16839023	C1QA;CXCL11;RPS19;RPS8;RPS5;RPS6;RPS20;RPSA;RPS3A;RAPGEF5;RPL10A;TPT1
Famciclovir-112_mg/kg_in_Saline-Rat-Spleen-0.25d-up	12/321	0,002729254	0,027278683	0	0	2,569290555	15,16839023	C1QA;RPS17;RPL32;RPS5;NKG7;RPSA;RPS3A;RAPGEF5;RPL10A;SEPP1;RPL39;TPT1
Gabapentin-100_mg/kg_in_Water-Rat-Heart-5d-up	12/321	0,002729254	0,027244109	0	0	2,569290555	15,16839023	RPS17;RPS19;FEM1B;COL5A3;ARL6IP5;RPS3A;RAPGEF5;RPL10A;RPL39;GOLGA7;TPT1;PDRG1
Idebenone-2500_mg/kg_in_CMC-Rat-Liver-0.25d-up	12/321	0,002729254	0,027209623	0	0	2,569290555	15,16839023	C1QA;RPS17;RPL32;RPS19;RPS8;RPS6;RPS20;RPSA;RPS3A;RPL10A;RPL39;TPT1
Sporidesmin_A-0.15_uM_in_DMSO-Rat-Primary_rat_hepatocytes-0.67d-dn	12/321	0,002729254	0,027175224	0	0	2,569290555	15,16839023	EEF1B2;RPS17;RPL32;ARL2BP;RPS8;FUNDC1;RPSA;RPS3A;RPL10A;SEPP1;RPL39;SLC16A3
Gentamicin-656.6_uM_in_DMSO-Rat-Primary_rat_hepatocytes-1d-dn	13/364	0,002740761	0,027255346	0	0	2,454590083	14,48090239	RPL32;RPS8;RPS5;RPS6;RPL35A;RPS3A;PDRG1;RPS17;RPS19;ARL2BP;RPS20;RPL39;TPT1
Amitriptyline-160_mg/kg_in_Water-Rat-Kidney-3d-dn	11/280	0,002778327	0,027594079	0	0	2,700049092	15,89223588	RPS17;RPL32;RPS19;RPS8;RPS6;ARL6IP5;RPL35A;RPSA;RPS3A;RPL10A;RPL39
Cyclosporin_A-350_mg/kg_in_Corn_Oil-Rat-Spleen-3d-up	11/280	0,002778327	0,027559326	0	0	2,700049092	15,89223588	EEF1B2;RPS17;RPL32;RPS8;RPS5;RPS6;RPL35A;RPS20;RPSA;RPS3A;RAPGEF5
Ethinylestradiol-10_mg/kg_in_Corn_Oil-Rat-Liver-5d-up	11/280	0,002778327	0,02752466	0	0	2,700049092	15,89223588	EEF1B2;RPS17;RPL32;RPS19;RPS8;RPS6;RPS20;RPSA;RPS3A;RPL10A;RPL39
Terazosin-657_mg/kg_in_Water-Rat-Heart-1d-up	11/280	0,002778327	0,027490081	0	0	2,700049092	15,89223588	RPS17;RPL32;RPS19;RPS8;FEM1B;RPS6;ARL6IP5;RPS20;RPSA;RAPGEF5;TPT1
Amlodipine-19_mg/kg_in_Corn_Oil-Rat-Liver-5d-up	12/322	0,002799039	0,027660262	0	0	2,561311391	15,05661583	EEF1B2;RPS17;RPL32;RPS19;RPS8;RPS5;RPL35A;RPS20;RPSA;RPS3A;TOMM20;TPT1
Busulfan-9_mg/kg_in_Corn_Oil-Rat-Bone_marrow-1d-up	12/322	0,002799039	0,0276256	0	0	2,561311391	15,05661583	EEF1B2;C1QA;RPS17;RPL32;RPS5;RPL35A;RPS20;RPSA;RPS3A;RPL10A;RPL39;TPT1
Candesartan-1300_mg/kg_in_CMC-Rat-Kidney-5d-up	12/322	0,002799039	0,027591025	0	0	2,561311391	15,05661583	RPL32;RPS19;FEM1B;RPS6;RPL35A;RPSA;RAPGEF5;RPL10A;SEPP1;RPL39;TOMM20;TPT1
Mifepristone-300_mg/kg_in_Corn_Oil-Rat-Liver-1d-up	12/322	0,002799039	0,027556536	0	0	2,561311391	15,05661583	EEF1B2;RPS17;RPL32;RPS19;RPS8;RPS5;RPS6;RPS20;RPSA;RPS3A;RPL10A;TPT1
Progesterone-11.3_mg/kg_in_Corn_Oil-Rat-Liver-1d-up	12/322	0,002799039	0,027522134	0	0	2,561311391	15,05661583	EEF1B2;RPS17;RPL32;RPS19;RPS8;RPS6;RPS20;RPSA;RPS3A;RPL10A;RPL39;TPT1
Rifampin-99_mg/kg_in_CMC-Rat-Liver-3d-up	12/322	0,002799039	0,027487817	0	0	2,561311391	15,05661583	EEF1B2;RPS17;RPL32;RPS19;RPS8;RPS6;RPS20;RPSA;RPS3A;RPL10A;SEPP1;RPL39
Aspirin-375_mg/kg_in_Corn_Oil-Rat-Kidney-1d-up	13/365	0,002805879	0,027520676	0	0	2,447865179	14,38374987	RPL32;RPS8;RPS5;RPS6;RPL13A;RPL35A;RPSA;RPS3A;RPS17;RPS19;RPS20;RPL39;TPT1
Gentamicin-2900_uM_in_DMSO-Rat-Primary_rat_hepatocytes-0.67d-dn	13/365	0,002805879	0,027486446	0	0	2,447865179	14,38374987	RPL32;RPS8;RPS5;RPS6;RPL35A;RPSA;RPL10A;SEPP1;TOMM20;RPS17;ARL6IP5;RPL39;TPT1
Azathioprine-20_mg/kg_in_Corn_Oil-Rat-Spleen-1d-dn	11/281	0,002855125	0,027934116	0	0	2,690440376	15,76232085	EEF1B2;RPS19;RPS8;RPS5;RPL13A;RPL35A;RPS20;RPSA;RPL10A;TOMM20;TPT1
Pentobarbital-70_mg/kg_in_Water-Rat-Liver-3d-dn	11/281	0,002855125	0,027899458	0	0	2,690440376	15,76232085	EEF1B2;C1QA;RPS17;RPL32;ARL2BP;RPS6;RPS20;RPL10A;RPL39;SEPP1;TPT1
Valeric_Acid-23000_uM_in_DMSO-Rat-Primary_rat_hepatocytes-1d-up	11/281	0,002855125	0,027864886	0	0	2,690440376	15,76232085	EEF1B2;RPS17;RPL32;RPS5;RPL35A;RPS20;RPSA;RPS3A;RPL10A;TOMM20;PDRG1

Dexamethasone-456_uM_in_DMSO-Rat-Primary_rat_hepatocytes-0.67d-dn	12/323	0,002870268	0,027978005	0	0	2,553381635	14,94583641	RPS17;CXCL11;RPS19;RPS8;ARL6IP5;RPSA;RPS3A;SEPP1;RPL39;GOLGA7;TPT1;PDRG1
Ethanol-6000_mg/kg_in_Saline-Rat-Liver-3d-up	12/323	0,002870268	0,027943421	0	0	2,553381635	14,94583641	EEF1B2;RPS17;RPL32;RPS19;RPS8;RPS5;RPS6;RPL35A;RPS20;RPSA;RPS3A;RPL10A
Carvedilol-50_uM_in_DMSO-Rat-Primary_rat_hepatocytes-1d-up	11/282	0,002933626	0,028524982	0	0	2,680899807	15,6337107	EEF1B2;RPS17;RPL32;ARL2BP;RPS5;RPS6;RPS20;RPSA;RPS3A;RPL10A;PDRG1
Insulin-0.56_mg/kg_in_Saline-Rat-Liver-5d-dn	11/282	0,002933626	0,028489809	0	0	2,680899807	15,6337107	RPS17;RPL32;RPS19;CLP1;RPS8;RPS5;RPL35A;RPSA;RPS3A;SEPP1;TPT1
Cisplatin-2_mg/kg_in_Saline-Rat-Kidney-1d-up	13/367	0,002939914	0,028515716	0	0	2,434525314	14,19176103	RPL32;RPS8;RPS6;RPL13A;RPL35A;RPSA;RPS3A;RPS17;RPS19;FUNDC1;ARL6IP5;RPS20;RPL39
Secobarbital-20_mg/kg_in_Water-Rat-Liver-1d-up	12/324	0,002942962	0,028510173	0	0	2,545500827	14,83604083	EEF1B2;RPS17;RPL32;RPS19;RPS5;RPS6;RPL13A;RPL35A;RPS20;RPSA;RPS3A;RPL39
Carboplatin-6_mg/kg_in_Saline-Rat-Spleen-3d-dn	11/283	0,003013857	0,0291611	0	0	2,671426663	15,50638879	EEF1B2;C1QA;RPS17;RPL32;ARL2BP;RPS8;FUNDC1;RPS6;ARL6IP5;RPSA;RPS3A
Fluorouracil-29_mg/kg_in_CMC-Rat-Spleen-5d-up	11/283	0,003013857	0,02912532	0	0	2,671426663	15,50638879	EEF1B2;RPL32;RPS19;RPS5;FUNDC1;RPS6;RPS20;RPSA;RPL10A;RPL39;SEPP1
Leflunomide-30_mg/kg_in_Corn_Oil-Rat-Kidney-3d-up	11/283	0,003013857	0,029089627	0	0	2,671426663	15,50638879	RPS17;RPL32;ARL2BP;RPS8;RPS5;FUNDC1;RPL35A;RPS20;RAPGEF5;GOLGA7;TPT1
Procarbazine-27_mg/kg_in_Water-Rat-Bone_marrow-3d-dn	11/283	0,003013857	0,029054022	0	0	2,671426663	15,50638879	RPS17;RPL32;ARL2BP;RPS8;FEM1B;FUNDC1;RPS6;RPS20;RPSA;RPS3A;TPT1
Acemetacin-2_mg/kg_in_Corn_Oil-Rat-Liver-5d-up	12/325	0,003017145	0,02905016	0	0	2,537668517	14,72721811	EEF1B2;C1QA;RPS17;RPL32;RPS8;RPS5;RPL35A;RPSA;RPS3A;SEPP1;RPL39;TPT1
Bromisovalum-250_mg/kg_in_Corn_Oil-Rat-Kidney-3d-dn	12/325	0,003017145	0,02901469	0	0	2,537668517	14,72721811	EEF1B2;RPS17;RPL32;RPS19;RPS8;RPS6;RPS20;RPSA;RPS3A;SEPP1;RPL39;TPT1
Cadmium_Acetate-167_mg/kg_in_Water-Rat-Kidney-3d-up	12/325	0,003017145	0,028979306	0	0	2,537668517	14,72721811	RPS17;RPL32;RPS19;RPS8;RPS5;RPS6;RPL13A;RPL35A;RPS20;RPS3A;RPL39;TPT1
Cisplatin-2_mg/kg_in_Saline-Rat-Spleen-1d-dn	12/325	0,003017145	0,028944009	0	0	2,537668517	14,72721811	EEF1B2;C1QA;RPS17;RPL32;RPS8;RPS5;FUNDC1;RPL13A;RPL35A;RPSA;RPS3A;SEPP1
Isoprenaline-4.2_mg/kg_in_Saline-Rat-Heart-3d-up	12/325	0,003017145	0,028908797	0	0	2,537668517	14,72721811	EEF1B2;C1QA;RPS19;RPS8;RPS5;RPS6;RPS20;RPSA;RPS3A;RPL10A;SEPP1;RPL39
Clotrimazole-52_mg/kg_in_Corn_Oil-Rat-Liver-3d-dn	12/326	0,003092837	0,029598036	0	0	2,529884258	14,61935741	EEF1B2;C1QA;RPL32;RPS19;RPS8;RPS6;RPL35A;RPS20;RPL10A;SEPP1;RPL39;TPT1
Methotrexate-0.3_mg/kg_in_Corn_Oil-Rat-Kidney-0.25d-up	12/326	0,003092837	0,029562116	0	0	2,529884258	14,61935741	EEF1B2;RPS17;RPS19;FUNDC1;RPS6;RPS20;RPS3A;RAPGEF5;RPL10A;SEPP1;RPL39;GOLGA7
NN-Dimethylformamide-700_mg/kg_in_Saline-Rat-Liver-3d-up	12/326	0,003092837	0,029526284	0	0	2,529884258	14,61935741	EEF1B2;RPS17;RPL32;RPS19;RPS8;RPS6;RPS20;RPS3A;RPL10A;SEPP1;RPL39;TPT1
Sulfadimethoxine-1100_mg/kg_in_CMC-Rat-Kidney-5d-up	12/326	0,003092837	0,029490537	0	0	2,529884258	14,61935741	EEF1B2;RPS17;CXCL11;RPL32;ARL2BP;ARL6IP5;RPL13A;RPL35A;RPSA;SEPP1;RPL39;TPT1
Cyclopropane_Carboxylic_Acid-375_mg/kg_in_Water-Rat-Liver-5d-up	11/284	0,003095846	0,029483532	0	0	2,662020231	15,38033877	EEF1B2;RPL32;RPS19;RPS8;RPS5;RPS6;RPS20;RPS3A;RPL10A;RPL39;TPT1
Losartan-1000_mg/kg_in_Water-Rat-Kidney-3d-dn	11/284	0,003095846	0,029447923	0	0	2,662020231	15,38033877	RPS17;RPL32;RPS19;RPS8;RPS6;ARL6IP5;RPL35A;RPSA;RPL10A;RPL39;SEPP1
Zidovudine-1540_mg/kg_in_Corn_Oil-Rat-Liver-1d-up	11/284	0,003095846	0,029412401	0	0	2,662020231	15,38033877	RPL32;RPS19;ARL2BP;RPS8;FEM1B;RPS6;RPS20;RPS3A;RPL10A;RPL39;SEPP1
Stanozolol-150_mg/kg_in_Water-Rat-Liver-1d-up	10/244	0,003162982	0,030014034	0	0	2,816742719	16,21384692	RPL32;RPS19;RPS5;RPS6;RPL35A;RPS20;RPSA;RPS3A;RPL10A;RPL39
Bithionol-333_mg/kg_in_Corn_Oil-Rat-Bone_marrow-3d-up	12/327	0,003170062	0,030045013	0	0	2,522147609	14,51244804	EEF1B2;C1QA;RPS17;RPL32;RPS8;RPS5;NKG7;RPL35A;RPS20;RPS3A;RPL39;TPT1
Clofibrate-130_mg/kg_in_Corn_Oil-Rat-Kidney-3d-dn	12/327	0,003170062	0,030008901	0	0	2,522147609	14,51244804	EEF1B2;RPS17;RPL32;RPS19;RPS6;ARL6IP5;RPL35A;RPS20;RPSA;RPL10A;RPL39;TPT1
Fludrocortisone_Acetate-125_mg/kg_in_CMC-Rat-Liver-1d-dn	12/327	0,003170062	0,029972876	0	0	2,522147609	14,51244804	EEF1B2;C1QA;RPS17;RPL32;RPS19;RPS8;RPS5;RPS6;RPL35A;RPS3A;RPL10A;SEPP1
Foscarnet-350_mg/kg_in_Saline-Rat-Kidney-3d-dn	12/327	0,003170062	0,029936937	0	0	2,522147609	14,51244804	RPS17;RPL32;ARL2BP;RPS5;RPS6;RPS20;RPSA;RPS3A;RPL10A;SEPP1;RPL39;TPT1
Ibuprofen-54_mg/kg_in_Corn_Oil-Rat-Kidney-3d-up	12/327	0,003170062	0,029901084	0	0	2,522147609	14,51244804	C1QA;RPS17;RPL32;ARL2BP;RPS8;RPS5;RPS6;RPL13A;RPL35A;RPS20;RPSA;RPS3A
Naproxen-10_mg/kg_in_Corn_Oil-Rat-Kidney-3d-up	12/327	0,003170062	0,029865318	0	0	2,522147609	14,51244804	EEF1B2;RPS17;RPS8;RPS5;RPL13A;RPL35A;RPS20;RPSA;RPS3A;RPL39;TOMM20;TPT1
Stannous_Fluoride-90_mg/kg_in_Water-Rat-Kidney-3d-up	12/327	0,003170062	0,029829636	0	0	2,522147609	14,51244804	EEF1B2;C1QA;RPS17;RPL32;RPS19;RPS6;RPS20;RPS3A;RAPGEF5;SEPP1;RPL39;TPT1
Vancomycin-160_mg/kg_in_Saline-Rat-Kidney-1d-up	12/327	0,003170062	0,02979404	0	0	2,522147609	14,51244804	EEF1B2;RPS17;RPL32;RPS19;RPS8;RPS6;ARL6IP5;RPL13A;RPL35A;RPSA;RPS3A;RPL39
Aspirin-375_mg/kg_in_Corn_Oil-Rat-Liver-0.25d-up	11/285	0,00317962	0,029848257	0	0	2,652679809	15,25554453	EEF1B2;RPS17;RPL32;RPS19;RPS8;RPS6;RPL35A;RPS20;RPSA;RPL10A;SEPP1
Desloratadine-57_uM_in_DMSO-Rat-Primary_rat_hepatocytes-0.67d-up	11/285	0,00317962	0,029812723	0	0	2,652679809	15,25554453	EEF1B2;CXCL11;RPS8;FEM1B;RPS6;ARL6IP5;RPSA;RPL10A;RPL39;GOLGA7;TPT1
Famciclovir-1200_mg/kg_in_Saline-Rat-Bone_marrow-5d-up	11/285	0,00317962	0,029777274	0	0	2,652679809	15,25554453	C1QA;RPS17;RPS19;ARL2BP;RPS8;FUNDC1;RPS6;RPS20;RPSA;RPS3A;TPT1
Megestrol_Acetate-132_mg/kg_in_CMC-Rat-Liver-1d-dn	11/285	0,00317962	0,029741909	0	0	2,652679809	15,25554453	C1QA;RPS17;RPL32;RPS19;RPS8;FEM1B;RPS6;RPL35A;RPS20;RPS3A;SEPP1
Oxcarbazepine-1600_mg/kg_in_CMC-Rat-Heart-1d-dn	11/285	0,00317962	0,029706628	0	0	2,652679809	15,25554453	RPL32;RPS19;ARL2BP;COL5A3;RPS6;ARL6IP5;RPSA;RPS3A;RPL10A;RPL39;PDRG1
Rapamycin-60_mg/kg_in_CMC-Rat-Heart-3d-up	11/285	0,00317962	0,029671431	0	0	2,652679809	15,25554453	EEF1B2;CXCL11;RPL32;ARL2BP;RPS8;FUNDC1;RPS6;RPS20;RPSA;RPS3A;TPT1
Doxapram-2_mg/kg_in_Saline-Rat-Liver-5d-up	12/328	0,003248841	0,030281507	0	0	2,514458134	14,40647948	C1QA;RPS17;RPL32;RPS8;RPS5;ARL6IP5;RPL13A;RPL35A;RPS20;RPSA;RPS3A;RPL39
Fluvastatin-5_mg/kg_in_Corn_Oil-Rat-Kidney-0.25d-dn	12/328	0,003248841	0,030245713	0	0	2,514458134	14,40647948	EEF1B2;RPS17;RPL32;RPS8;ARL6IP5;RPL35A;RPS20;RPSA;RPS3A;RPL39;TOMM20;TPT1
Glimepiride-2500_mg/kg_in_CMC-Rat-Liver-0.25d-up	12/328	0,003248841	0,030210004	0	0	2,514458134	14,40647948	C1QA;RPS17;RPL32;RPS19;RPS8;RPS6;RPS20;RPSA;RPS3A;RPL10A;RPL39;TPT1
Lead(IV)_Acetate-600_mg/kg_in_Saline-Rat-Bone_marrow-3d-up	12/328	0,003248841	0,030174379	0	0	2,514458134	14,40647948	EEF1B2;RPS17;RPL32;RPS19;RPS8;RPS6;RPL35A;RPS20;RPS3A;RPL10A;RPL39;TPT1
N-Nitrosodiethylamine-1.67_mg/kg_in_Saline-Rat-Spleen-1d-up	12/328	0,003248841	0,030138838	0	0	2,514458134	14,40647948	EEF1B2;C1QA;RPS17;RPS19;RPS5;RPL35A;RPS20;RPSA;RPS3A;RPL10A;SEPP1;RPL39
Sodium_Nitroprusside-6_mg/kg_in_Saline-Rat-Heart-5d-up	12/328	0,003248841	0,030103381	0	0	2,514458134	14,40647948	C1QA;RPS17;RPL32;RPS19;RPS8;RPS5;FUNDC1;RPS6;ARL6IP5;RPL35A;RPSA;RPS3A
TGF_Beta-1_Human_Recombinant-5_ng/ml_in_DMSO-Rat-Primary_rat_hepatocytes-0.67d-dn	10/245	0,00325635	0,030137503	0	0	2,80524581	16,06605861	RPS17;RPS19;RPS8;RPS5;RPS20;RPS3A;RPL10A;RPL39;SEPP1;PDRG1
Acetaminophen-1950_uM_in_DMSO-Rat-Primary_rat_hepatocytes-0.67d-up	11/286	0,003265208	0,030184009	0	0	2,643404705	15,13199023	C1QA;RPL32;RPS19;ARL2BP;CLP1;RPS8;FEM1B;RPS6;RPS20;RPSA;TPT1
Azithromycin-225_mg/kg_in_Water-Rat-Kidney-3d-dn	11/286	0,003265208	0,030148624	0	0	2,643404705	15,13199023	RPS17;RPL32;RPS19;ARL6IP5;RPL35A;RPS20;RPSA;RPL10A;RPL39;SEPP1;TPT1
Clopidogrel-400_mg/kg_in_Corn_Oil-Rat-Bone_marrow-5d-up	11/286	0,003265208	0,030113321	0	0	2,643404705	15,13199023	EEF1B2;C1QA;RPS17;RPL32;RPS8;FEM1B;RPS6;RPL35A;RPS3A;RPL39;SLC16A3
Lead(IV)_Acetate-200_mg/kg_in_Saline-Rat-Spleen-0.25d-dn	11/286	0,003265208	0,0300781	0	0	2,643404705	15,13199023	EEF1B2;CXCL11;RPL32;RPS19;RPS8;FUNDC1;RPL35A;RPS20;RPSA;RPL39;TPT1
Tacrolimus-134_mg/kg_in_CMC-Rat-Heart-3d-up	11/286	0,003265208	0,030042962	0	0	2,643404705	15,13199023	RPS17;CXCL11;RPL32;RPS19;ARL2BP;RPS6;RPS20;RPS3A;RAPGEF5;SEPP1;TPT1
Venlafaxine-320_mg/kg_in_Water-Rat-Kidney-5d-dn	11/286	0,003265208	0,030007906	0	0	2,643404705	15,13199023	RPS17;RPL32;RPS19;RPS6;RPL35A;RPS20;RPSA;RPS3A;RPL10A;RPL39;TPT1
Neomycin-877_mg/kg_in_Corn_Oil-Rat-Kidney-5d-up	12/329	0,003329198	0,030560334	0	0	2,506815404	14,30144133	EEF1B2;RPS17;RPL32;RPS19;RPS8;RPS5;RPS6;RPL35A;RPS20;RPS3A;RPL10A;RPL39
Streptomycin-120_mg/kg_in_Saline-Rat-Bone_marrow-5d-up	12/329	0,003329198	0,030524758	0	0	2,506815404	14,30144133	EEF1B2;RPL32;RPS19;ARL2BP;RPS8;FEM1B;RPS6;RPS20;RPSA;RPS3A;RPL10A;TPT1
Dichlorvos-17_mg/kg_in_Water-Rat-Heart-1d-up	11/287	0,003352637	0,030703915	0	0	2,634194236	15,00966026	RPS17;RPL32;ARL2BP;CLP1;RPS8;FUNDC1;RPS6;RPS20;RPS3A;RPL39;TPT1
Ifosfamide-17_mg/kg_in_Saline-Rat-Spleen-1d-dn	11/287	0,003352637	0,030668254	0	0	2,634194236	15,00966026	EEF1B2;RPS17;RPL32;ARL2BP;RPS8;RPS5;FUNDC1;RPL13A;RPL35A;RPS3A;TPT1
Leucovorin-1500_mg/kg_in_CMC-Rat-Bone_marrow-5d-up	11/287	0,003352637	0,030632676	0	0	2,634194236	15,00966026	C1QA;RPS17;RPL32;RPS8;RPS5;RPS6;RPS20;RPSA;RPS3A;RPL10A;SEPP1
Methotrexate-27_mg/kg_in_Corn_Oil-Rat-Spleen-3d-up	11/287	0,003352637	0,030597181	0	0	2,634194236	15,00966026	EEF1B2;RPL32;RPS8;RPS5;RPS6;RPL35A;RPS20;RPSA;RPS3A;RAPGEF5;SEPP1
Progesterone-148_uM_in_DMSO-Rat-Primary_rat_hepatocytes-1d-up	11/287	0,003352637	0,030561767	0	0	2,634194236	15,00966026	EEF1B2;RPS17;RPL32;CLP1;RPL35A;RPS20;RPS3A;RPL10A;RPL39;TOMM20;TPT1
Thioguanine-24_mg/kg_in_Corn_Oil-Rat-Spleen-1d-up	11/287	0,003352637	0,030526436	0	0	2,634194236	15,00966026	C1QA;CXCL11;RPS8;FUNDC1;RPS6;RPS20;RPSA;RPS3A;RPL10A;SEPP1;TPT1
Amantadine-1500_uM_in_DMSO-Rat-Primary_rat_hepatocytes-0.67d-up	13/373	0,003373729	0,030683015	0	0	2,395364049	13,63378116	RPL32;RPS8;RPS5;RPS6;RPL35A;RPSA;SEPP1;PDRG1;EEF1B2;RPS19;RPS20;RPL39;TPT1
Leflunomide-60_mg/kg_in_Corn_Oil-Rat-Liver-1d-dn	13/373	0,003373729	0,030647625	0	0	2,395364049	13,63378116	C1QA;RPL32;RPS8;RPS6;RPSA;RPS3A;RPL10A;SEPP1;EEF1B2;RPS17;RPS20;RPL39;TPT1
Propylene_Glycol-2000_mg/kg_in_Water-Rat-Kidney-1d-dn	9/207	0,003407086	0,030914985	0	0	2,988196623	16,97862838	RPS17;RPL32;RPS19;RPS6;RPL35A;RPS20;RPSA;RPS3A;RPL39
Aspirin-500_mg/kg_in_Corn_Oil-Rat-Liver-3d-dn	12/330	0,003411156	0,030916301	0	0	2,499218994	14,19732334	EEF1B2;RPL32;RPS19;RPS8;RPS5;RPS6;RPL35A;RPS20;RPSA;RPL10A;RPL39;TPT1
Carboplatin-6_mg/kg_in_Saline-Rat-Liver-1d-dn	12/330	0,003411156	0,030880765	0	0	2,499218994	14,19732334	EEF1B2;RPS17;RPL32;RPS19;RPS8;RPS5;RPS6;RPL35A;RPSA;RPS3A;RPL10A;SEPP1
Cisplatin-2_mg/kg_in_Saline-Rat-Spleen-3d-dn	12/330	0,003411156	0,030845311	0	0	2,499218994	14,19732334	EEF1B2;C1QA;RPS17;RPL32;RPS6;ARL6IP5;RPL35A;RPSA;RPS3A;RPL10A;SEPP1;TPT1
Enrofloxacin-2000_mg/kg_in_CMC-Rat-Kidney-1d-up	12/330	0,003411156	0,030809938	0	0	2,499218994	14,19732334	EEF1B2;RPS17;RPL32;RPS19;RPS6;ARL6IP5;RPL13A;RPL35A;RPS20;RPSA;RPL10A;RPL39
Epirubicin-2.7_mg/kg_in_Saline-Rat-Kidney-1d-up	12/330	0,003411156	0,030774646	0	0	2,499218994	14,19732334	EEF1B2;RPS17;RPL32;RPS19;RPS8;ARL6IP5;RPL13A;RPL35A;RPS20;RPS3A;RPL10A;SEPP1
Fluoxetine-52_mg/kg_in_CMC-Rat-Liver-1d-up	12/330	0,003411156	0,030739435	0	0	2,499218994	14,19732334	EEF1B2;RPS17;RPL32;RPS8;RPS5;RPL13A;RPL35A;RPS20;RPSA;RPS3A;SEPP1;TPT1
Lead(IV)_Acetate-200_mg/kg_in_Saline-Rat-Kidney-1d-up	12/330	0,003411156	0,030704304	0	0	2,499218994	14,19732334	RPS17;CXCL11;RPS19;RPS8;RPS6;ARL6IP5;RPL35A;RPS20;RPSA;RPL10A;RPL39;GOLGA7
Valproic_Acid-850_mg/kg_in_Water-Rat-Kidney-5d-up	12/330	0,003411156	0,030669253	0	0	2,499218994	14,19732334	EEF1B2;C1QA;RPS17;RPL32;RPS8;RPS6;RPL35A;RPS20;RPSA;RPS3A;RPL39;TPT1
Amitraz-75_mg/kg_in_CMC-Rat-Heart-5d-up	11/288	0,003441935	0,030910699	0	0	2,625047728	14,88853927	RPS17;RPL32;RPS19;RPS8;RPS5;RPS6;RPS20;RPSA;RPS3A;SEPP1;TOMM20
Gabapentin-500_uM_in_DMSO-Rat-Primary_rat_hepatocytes-0.67d-dn	11/288	0,003441935	0,030875493	0	0	2,625047728	14,88853927	CXCL11;RPL32;RPS19;ARL2BP;RPS8;RPS5;ARL6IP5;RPSA;RPS3A;GOLGA7;SEPP1
Leflunomide-60_mg/kg_in_Corn_Oil-Rat-Bone_marrow-3d-dn	11/288	0,003441935	0,030840367	0	0	2,625047728	14,88853927	RPS17;RPL32;RPS19;RPS8;FEM1B;RPS6;ARL6IP5;RPS20;RPSA;RPS3A;RPL10A
Mitomycin_C-0.5_mg/kg_in_Saline-Rat-Bone_marrow-1d-dn	11/288	0,003441935	0,030805322	0	0	2,625047728	14,88853927	EEF1B2;RPL32;RPS19;ARL2BP;RPS8;RPS6;RPS20;RPSA;RPS3A;RPL39;TPT1
Urethane-453_mg/kg_in_Water-Rat-Bone_marrow-3d-up	11/288	0,003441935	0,030770355	0	0	2,625047728	14,88853927	C1QA;RPS17;RPL32;RPS19;RPS8;RPS5;RPS6;RPL35A;RPSA;RPL10A;SEPP1
Cisplatin-1.17_mg/kg_in_Saline-Rat-Liver-1d-dn	13/374	0,003450852	0,030815092	0	0	2,388959333	13,54333081	RPL32;RPS8;RPS5;RPS6;RPL35A;RPSA;RPL10A;SEPP1;EEF1B2;RPS17;RPS19;RPS20;RPL39
Promazine-100_mg/kg_in_Corn_Oil-Rat-Liver-0.25d-up	13/375	0,003529403	0,031480835	0	0	2,382588774	13,45358906	C1QA;RPL32;RPS8;RPS5;RPS6;RPSA;RPS3A;RPL10A;RPS17;RPS19;RPS20;RPL39;TPT1
Altretamine-40_mg/kg_in_Corn_Oil-Rat-Liver-3d-dn	11/289	0,003533132	0,031478449	0	0	2,615964518	14,76861214	EEF1B2;RPS17;RPL32;RPS19;ARL2BP;RPS8;RPS6;RPL35A;RPSA;RPL10A;SEPP1
BW-723C86-50_mg/kg_in_Saline-Rat-Heart-1d-up	11/289	0,003533132	0,03144288	0	0	2,615964518	14,76861214	EEF1B2;C1QA;RPS19;RPS8;RPS6;RPS20;RPSA;RPS3A;RAPGEF5;RPL10A;RPL39
Lead_(II)_Acetate-200_mg/kg_in_Saline-Rat-Kidney-3d-up	11/289	0,003533132	0,031407391	0	0	2,615964518	14,76861214	RPS17;CXCL11;RPS8;RPS5;ARL6IP5;RPL13A;RPL35A;RPS20;RPSA;RPL39;TOMM20
Lipopolysaccharide_E._Coli_O55:B5-1.25_mg/kg_in_Saline-Rat-Kidney-5d-up	11/289	0,003533132	0,031371983	0	0	2,615964518	14,76861214	C1QA;RPS19;RPS6;RPS20;RPSA;RAPGEF5;RPL10A;RPL39;GOLGA7;SEPP1;TPT1
Ampicillin-2000_mg/kg_in_CMC-Rat-Kidney-5d-up	12/332	0,003579965	0,031752036	0	0	2,484163458	13,99180754	EEF1B2;C1QA;CXCL11;CXADR;RPL32;RPS19;RPS8;RPS6;ARL6IP5;RPS20;RPSA;RPL10A
Isoprenaline-15_mg/kg_in_Saline-Rat-Liver-3d-up	12/332	0,003579965	0,031716319	0	0	2,484163458	13,99180754	EEF1B2;C1QA;RPS17;RPL32;RPS19;RPS6;RPS20;RPSA;RPS3A;RPL10A;SEPP1;TPT1
Naloxone-76_mg/kg_in_Saline-Rat-Liver-5d-up	12/332	0,003579965	0,031680683	0	0	2,484163458	13,99180754	EEF1B2;C1QA;RPS17;RPL32;RPS19;RPS8;RPL35A;RPS20;RPSA;RPS3A;RPL10A;RPL39
Beta-Estradiol_3-Benzoate-0.25_mg/kg_in_Corn_Oil-Rat-Liver-1d-up	11/290	0,003626256	0,03205431	0	0	2,606943951	14,64986399	RPS17;RPL32;RPS5;RPS6;RPL35A;RPS20;RPSA;RPS3A;RPL39;TOMM20;TPT1
Bisphenol_A-100_mg/kg_in_Corn_Oil-Rat-Bone_marrow-3d-up	11/290	0,003626256	0,032018374	0	0	2,606943951	14,64986399	C1QA;RPS17;ARL2BP;CLP1;RPS8;RPS5;RPS6;RPL35A;RPS20;RPS3A;TPT1
Bromhexine-1200_mg/kg_in_CMC-Rat-Liver-5d-up	11/290	0,003626256	0,03198252	0	0	2,606943951	14,64986399	RPS17;RPL32;RPS19;RPS8;RPS6;RPS20;RPSA;RPS3A;RPL10A;RPL39;SEPP1
Catechol-195_mg/kg_in_Saline-Rat-Liver-0.25d-up	11/290	0,003626256	0,031946745	0	0	2,606943951	14,64986399	EEF1B2;RPS17;RPL32;RPS8;RPS5;RPS6;RPSA;RPS3A;RPL10A;RPL39;TPT1
Closantel-22_mg/kg_in_CMC-Rat-Liver-5d-dn	11/290	0,003626256	0,03191105	0	0	2,606943951	14,64986399	EEF1B2;C1QA;RPS17;RPL32;RPS19;RPS8;RPSA;RPS3A;RPL10A;RPL39;TPT1
Ethylestrenol-390_mg/kg_in_CMC-Rat-Liver-1d-up	11/290	0,003626256	0,031875435	0	0	2,606943951	14,64986399	RPS17;RPL32;RPS19;RPS8;RPS5;ARL6IP5;RPSA;RPS3A;RPL10A;RPL39;SEPP1
Leflunomide-30_mg/kg_in_Corn_Oil-Rat-Spleen-1d-dn	11/290	0,003626256	0,0318399	0	0	2,606943951	14,64986399	EEF1B2;RPS17;RPL32;RPS19;RPS8;RPS5;RPS6;RPL35A;RPS20;RPL10A;RPL39

Procarbazine-54_mg/kg_in_Water-Rat-Bone_marrow-3d-dn	11/290	0,003626256	0,031804443	0	0	2,606943951	14,64986399	RPS17;RPL32;RPS8;FEM1B;FUNDC1;RPS6;ARL6IP5;RPS20;RPS3A;RPL39;TPT1
Sildenafil-14.6_mg/kg_in_Saline-Rat-Liver-0.25d-up	11/290	0,003626256	0,031769066	0	0	2,606943951	14,64986399	EEF1B2;C1QA;RPS17;RPL32;RPS6;RPSA;RPS3A;RPL10A;RPL39;SEPP1;TPT1
Torsemide-3_mg/kg_in_Corn_Oil-Rat-Liver-5d-up	11/290	0,003626256	0,031733767	0	0	2,606943951	14,64986399	EEF1B2;RPS17;RPL32;RPS19;RPS8;RPS5;RPL35A;RPS20;RPSA;RPL39;TPT1
Amitriptyline-160_mg/kg_in_Water-Rat-Kidney-1d-dn	10/249	0,003652001	0,031923592	0	0	2,76018162	15,49146457	RPS17;RPL32;RPS19;RPS6;RPL35A;RPS20;RPSA;RPL10A;RPL39;GOLGA7
Streptozotocin-3000_uM_in_DMSO-Rat-Primary_rat_hepatocytes-0.67d-dn	10/249	0,003652001	0,0318882	0	0	2,76018162	15,49146457	CXCL11;RPS19;RPS5;ARL6IP5;RPS20;RPSA;RPL10A;RPL39;GOLGA7;SEPP1
3-Acetamidophenol-700_mg/kg_in_Corn_Oil-Rat-Kidney-5d-dn	12/333	0,003666864	0,031982523	0	0	2,476703508	13,89038993	EEF1B2;RPS17;RPL32;RPS19;RPS8;RPS6;RPS20;RPSA;RPS3A;SEPP1;RPL39;TPT1
Bromisovalum-250_mg/kg_in_Corn_Oil-Rat-Kidney-5d-dn	12/333	0,003666864	0,031947144	0	0	2,476703508	13,89038993	EEF1B2;RPS17;RPL32;RPS19;RPS8;RPS6;RPS20;RPSA;RPS3A;SEPP1;RPL39;TPT1
Lovastatin-450_mg/kg_in_Corn_Oil-Rat-Kidney-0.25d-up	12/333	0,003666864	0,031911843	0	0	2,476703508	13,89038993	EEF1B2;RPS17;RPL32;RPS5;RPS6;RPL13A;RPS20;RPSA;RPS3A;SEPP1;RPL39;TPT1
Acrolein-20_mg/kg_in_Water-Rat-Heart-5d-up	11/291	0,003721335	0,032350148	0	0	2,59798538	14,53228018	EEF1B2;RPS17;RPL32;RPS8;ARL6IP5;RPS20;RPS3A;RPL10A;RPL39;SEPP1;TPT1
Allyl_Alcohol-32_mg/kg_in_Saline-Rat-Liver-1d-up	11/291	0,003721335	0,032314481	0	0	2,59798538	14,53228018	EEF1B2;RPS17;RPL32;RPS19;RPS8;RPS6;RPS20;RPSA;RPS3A;RPL10A;RPL39
Bleomycin_A2-12_mg/kg_in_Saline-Rat-Bone_marrow-0.25d-up	11/291	0,003721335	0,032278892	0	0	2,59798538	14,53228018	RPL32;RPS19;RPS8;FEM1B;RPS5;FUNDC1;RPS6;RPS20;RPSA;RPL10A;TPT1
Dyphylline-3855_mg/kg_in_CMC-Rat-Heart-3d-up	11/291	0,003721335	0,032243382	0	0	2,59798538	14,53228018	RPS17;RPL32;RPS19;RPS5;RPS6;RPL35A;RPS20;RPSA;RPS3A;TOMM20;TPT1
Sildenafil-300_mg/kg_in_Saline-Rat-Liver-5d-dn	11/291	0,003721335	0,03220795	0	0	2,59798538	14,53228018	EEF1B2;C1QA;RPS17;RPL32;RPS8;RPS5;RPS6;RPSA;RPS3A;RPL10A;RPL39
Lead(IV)_Acetate-200_mg/kg_in_Saline-Rat-Bone_marrow-3d-dn	10/250	0,003756638	0,032477809	0	0	2,749140893	15,35183714	EEF1B2;RPS17;RPL32;RPS8;RPS5;RPS6;NKG7;RPSA;RPS3A;TPT1
Carboplatin-14_mg/kg_in_Saline-Rat-Bone_marrow-1d-up	13/378	0,003773817	0,032590549	0	0	2,36367934	13,1885469	C1QA;RPS8;RPS5;RPS6;NKG7;RPL35A;RPSA;RPS3A;RPL10A;SEPP1;RPS17;RPS20;TPT1
Bisacodyl-1750_mg/kg_in_CMC-Rat-Intestine-3d-dn	11/292	0,003818399	0,032939442	0	0	2,58908817	14,41584627	EEF1B2;C1QA;RPS17;RPS19;ARL2BP;RPS8;RPS6;ARL6IP5;RPSA;RPS3A;SEPP1
Cholecalciferol-8_mg/kg_in_CMC-Rat-Heart-5d-up	11/292	0,003818399	0,032903404	0	0	2,58908817	14,41584627	EEF1B2;C1QA;RPS17;CXCL11;RPS19;RPS8;RPS20;RPSA;RPL10A;SEPP1;RPL39
Famciclovir-112_mg/kg_in_Saline-Rat-Liver-3d-up	11/292	0,003818399	0,032867444	0	0	2,58908817	14,41584627	EEF1B2;RPS17;RPS19;CLP1;RPS8;RPS6;RPL35A;RPS20;RPSA;RPS3A;RPL10A
Pralidoxime_Chloride-48_mg/kg_in_Saline-Rat-Heart-5d-up	11/292	0,003818399	0,032831562	0	0	2,58908817	14,41584627	C1QA;RPS17;RPL32;RPS19;NKG7;RPS20;RPS3A;RPL10A;SEPP1;RPL39;TPT1
Pravastatin-1200_mg/kg_in_Corn_Oil-Rat-Liver-3d-up	11/292	0,003818399	0,032795759	0	0	2,58908817	14,41584627	CXADR;RPS19;RPS8;FEM1B;RPS6;RPS20;RPSA;RPS3A;RPL10A;SEPP1;TPT1
Valproic_Acid-1500_mg/kg_in_Water-Rat-Liver-1d-up	11/292	0,003818399	0,032760034	0	0	2,58908817	14,41584627	RPS17;RPL32;RPS19;RPS8;RPS6;RPS20;RPSA;RPS3A;RPL10A;RPL39;TPT1
D-Tubocurarine_Chloride-0.0033_mg/kg_in_Saline-Rat-Heart-3d-up	12/335	0,003845765	0,032958917	0	0	2,461917218	13,69018679	RPS17;RPL32;RPS19;ARL2BP;RPS8;RPS6;RPS20;RPSA;RPS3A;RPL10A;RPL39;TPT1
Irinotecan-5_mg/kg_in_Saline-Rat-Bone_marrow-1d-dn	12/335	0,003845765	0,032923092	0	0	2,461917218	13,69018679	EEF1B2;RPS17;RPL32;RPS8;FUNDC1;RPS6;RPL35A;RPS20;RPSA;RPS3A;RPL39;TPT1
Mannitol-1000_mg/kg_in_Saline-Rat-Kidney-3d-up	12/335	0,003845765	0,032887345	0	0	2,461917218	13,69018679	C1QA;RPS17;CXCL11;RPL32;RPS8;RPS5;ARL6IP5;RPL13A;RPL35A;RPSA;RPS3A;SEPP1
Carbon_Tetrachloride-775_uM_in_DMSO-Rat-Primary_rat_hepatocytes-1d-dn	11/293	0,003917477	0,033464263	0	0	2,580251692	14,30054808	EEF1B2;RPL32;RPS8;RPS5;RPS6;RPS20;RPS3A;RPL10A;SEPP1;RPL39;TPT1
Iproniazid-46_mg/kg_in_Water-Rat-Brain-5d-up	11/293	0,003917477	0,033428007	0	0	2,580251692	14,30054808	EEF1B2;RPS19;ARL2BP;CLP1;RPS6;ARL6IP5;RPS20;RPL10A;SEPP1;RPL39;GOLGA7
Lomustine-8.75_mg/kg_in_Corn_Oil-Rat-Bone_marrow-5d-dn	11/293	0,003917477	0,033391829	0	0	2,580251692	14,30054808	EEF1B2;RPS17;RPL32;RPS19;RPS8;FUNDC1;RPS6;ARL6IP5;RPS20;RPSA;RPS3A
Mitoxantrone-2_mg/kg_in_Saline-Rat-Heart-5d-up	11/293	0,003917477	0,03335573	0	0	2,580251692	14,30054808	RPS17;RPL32;RPS19;CLP1;RPS8;FUNDC1;RPS6;RPS20;RPS3A;RPL10A;TPT1
Atorvastatin-300_mg/kg_in_Corn_Oil-Rat-Kidney-1d-dn	11/294	0,004018599	0,034179789	0	0	2,571475326	14,18637161	EEF1B2;RPS17;RPL32;RPS19;ARL2BP;RPS8;RPS6;RPS3A;RPL10A;RPL39;TPT1
CerlVastatin-7_mg/kg_in_Corn_Oil-Rat-Kidney-5d-dn	11/294	0,004018599	0,034142917	0	0	2,571475326	14,18637161	EEF1B2;RPS17;RPL32;RPS19;ARL2BP;RPS6;RPS20;RPSA;RPS3A;SEPP1;RPL39
Cholic_Acid-1402_mg/kg_in_Water-Rat-Liver-1d-up	11/294	0,004018599	0,034106125	0	0	2,571475326	14,18637161	EEF1B2;RPS17;RPL32;RPS8;RPS5;RPS6;RPL13A;RPL35A;RPS20;RPSA;RPL39
Clobetasol_Propionate-17_mg/kg_in_Corn_Oil-Rat-Bone_marrow-3d-dn	11/294	0,004018599	0,034069413	0	0	2,571475326	14,18637161	RPS17;RPL32;RPS19;FEM1B;RPS6;RPS20;RPSA;RPS3A;RPL10A;SEPP1;RPL39
Ethanol-3000_mg/kg_in_Saline-Rat-Kidney-3d-up	11/294	0,004018599	0,034032779	0	0	2,571475326	14,18637161	RPS17;ARL2BP;RPS5;ARL6IP5;RPL13A;RPL35A;RPS20;RPSA;RPL39;TOMM20;TPT1
Lidocaine-71_mg/kg_in_Water-Rat-Heart-3d-dn	11/294	0,004018599	0,033996224	0	0	2,571475326	14,18637161	EEF1B2;RPS17;RPL32;RPS8;RPS20;RPSA;RPS3A;RPL10A;SEPP1;RPL39;TPT1
Propylene_Glycol-2000_mg/kg_in_Water-Rat-Liver-3d-up	11/294	0,004018599	0,033959747	0	0	2,571475326	14,18637161	EEF1B2;RPS17;RPL32;RPS8;RPS5;RPS6;ARL6IP5;RPS20;RPSA;RPS3A;SEPP1
Tamoxifen-37.5_uM_in_DMSO-Rat-Primary_rat_hepatocytes-0.67d-up	11/294	0,004018599	0,033923349	0	0	2,571475326	14,18637161	EEF1B2;RPS17;RPS19;ARL2BP;RPS8;RPS5;RPS20;RPS3A;RPL10A;RPL39;PDRG1
4-Methylpyrazole-155_mg/kg_in_Saline-Rat-Kidney-3d-dn	12/337	0,004031632	0,033996927	0	0	2,447306433	13,49342991	EEF1B2;RPS17;RPL32;ARL2BP;RPS8;RPS6;RPS20;RPSA;RPS3A;SEPP1;RPL39;TPT1
Mannitol-1000_mg/kg_in_Saline-Rat-Kidney-1d-up	12/337	0,004031632	0,033960567	0	0	2,447306433	13,49342991	RPS17;RPL32;RPS19;RPS8;RPS6;ARL6IP5;RPL35A;RPS20;RPSA;RPS3A;SEPP1;TOMM20
Clotrimazole-60_uM_in_DMSO-Rat-Primary_rat_hepatocytes-0.67d-dn	13/381	0,004031785	0,033925574	0	0	2,345067691	12,92963883	RPL32;RPS8;RPS5;RPS6;RPSA;RPS3A;SEPP1;RPS17;CXCL11;CLP1;ARL6IP5;RPS20;TPT1
Buflomedil-615_mg/kg_in_CMC-Rat-Heart-3d-up	11/295	0,004121794	0,034645942	0	0	2,56275846	14,07330311	EEF1B2;RPS17;RPL32;RPS19;RPS8;RPS5;FUNDC1;RPS6;RPL35A;RPS20;RPS3A
Clonidine-6_mg/kg_in_Water-Rat-Heart-1d-up	11/295	0,004121794	0,034609006	0	0	2,56275846	14,07330311	C1QA;RPS17;RPL32;RPS19;RPS8;RPS6;RPS20;RPS3A;RAPGEF5;SEPP1;TPT1
Lipopolysaccharide_E._Coli_O55:B5-1.25_mg/kg_in_Saline-Rat-Kidney-1d-up	11/295	0,004121794	0,034572148	0	0	2,56275846	14,07330311	C1QA;CXCL11;RPS19;RPS6;RPS20;RPSA;RAPGEF5;RPL10A;SEPP1;RPL39;TPT1
N-Nitrosodiethylamine-1.67_mg/kg_in_Saline-Rat-Bone_marrow-1d-up	11/295	0,004121794	0,034535369	0	0	2,56275846	14,07330311	C1QA;RPS17;RPL32;RPS19;RPS8;RPS5;RPS6;ARL6IP5;RPL35A;RPS3A;TPT1
Oxyquinoline-68_mg/kg_in_Corn_Oil-Rat-Liver-3d-up	11/295	0,004121794	0,034498669	0	0	2,56275846	14,07330311	EEF1B2;C1QA;RPS17;RPL32;RPS8;RPS6;RPS20;RPS3A;RAPGEF5;SEPP1;TPT1
Paraquat_Dichloride-650_uM_in_DMSO-Rat-Primary_rat_hepatocytes-0.67d-dn	11/295	0,004121794	0,034462046	0	0	2,56275846	14,07330311	RPS17;CXCL11;RPL32;RPS8;RPS5;ARL6IP5;RPL35A;RPSA;RPS3A;TOMM20;TPT1
Acyclovir-980_mg/kg_in_Saline-Rat-Liver-1d-dn	12/338	0,004127237	0,034470964	0	0	2,440065882	13,39632061	EEF1B2;RPS17;RPL32;RPS19;RPS6;RPL35A;RPS20;RPS3A;RPL10A;SEPP1;RPL39;TPT1
Celecoxib-400_mg/kg_in_Corn_Oil-Rat-Liver-3d-dn	12/338	0,004127237	0,034434448	0	0	2,440065882	13,39632061	EEF1B2;RPL32;RPS19;RPS8;RPS5;RPS6;RPL35A;RPS20;RPSA;RPL10A;RPL39;TPT1
Penicillin_G-243_mg/kg_in_Saline-Rat-Kidney-1d-up	12/338	0,004127237	0,034398009	0	0	2,440065882	13,39632061	RPS17;RPL32;RPS19;RPS8;RPS5;RPS6;ARL6IP5;RPL35A;RPS20;RPSA;RPL10A;SEPP1
Daunorubicin-3.25_mg/kg_in_Saline-Rat-Kidney-1d-dn	10/254	0,004199194	0,034960731	0	0	2,705847336	14,80873093	RPS17;RPL32;RPS8;RPS5;ARL6IP5;RPL35A;RPSA;RPS3A;TOMM20;TPT1
44'-Methylenedianiline-81_mg/kg_in_Corn_Oil-Rat-Kidney-5d-dn	12/339	0,004224656	0,035135579	0	0	2,432868047	13,30004528	EEF1B2;RPS17;RPL32;RPS19;ARL2BP;RPS8;RPS6;RPS20;RPSA;RPS3A;SEPP1;RPL39
Dexfenfluramine-29_mg/kg_in_Water-Rat-Heart-5d-dn	12/339	0,004224656	0,035098516	0	0	2,432868047	13,30004528	RPS17;RPL32;RPS19;RPS8;COL5A3;RPS6;RPL35A;RPS20;RPSA;RPS3A;RPL39;TPT1
Rofecoxib-250_mg/kg_in_Corn_Oil-Rat-Liver-3d-dn	12/339	0,004224656	0,035061531	0	0	2,432868047	13,30004528	EEF1B2;RPL32;RPS19;RPS8;RPS5;RPS6;RPL35A;RPS20;RPSA;RPL10A;RPL39;TPT1
Acetaminophen-972_mg/kg_in_Corn_Oil-Rat-Liver-1d-up	11/296	0,004227092	0,035044815	0	0	2,554100492	13,96132901	EEF1B2;RPS17;RPL32;RPS19;RPS8;RPS6;RPS20;RPSA;RPS3A;RPL10A;RPL39
Amineptine-1667_uM_in_DMSO-Rat-Primary_rat_hepatocytes-0.67d-up	11/296	0,004227092	0,035007964	0	0	2,554100492	13,96132901	EEF1B2;RPS19;ARL2BP;CLP1;RPS8;FEM1B;RPS6;RPS20;RPL10A;RPL39;PDRG1
Aztreonam-1100_mg/kg_in_CMC-Rat-Spleen-5d-up	11/296	0,004227092	0,034971191	0	0	2,554100492	13,96132901	EEF1B2;C1QA;RPS19;RPS5;FUNDC1;RPS6;RPS20;RPSA;RPL10A;SEPP1;RPL39
Fluconazole-394_mg/kg_in_Corn_Oil-Rat-Liver-1d-up	11/296	0,004227092	0,034934495	0	0	2,554100492	13,96132901	RPS17;RPL32;RPS19;RPS8;RPS6;RPS20;RPSA;RPS3A;RPL10A;RPL39;TPT1
Flunarizine-126_mg/kg_in_CMC-Rat-Heart-3d-up	11/296	0,004227092	0,034897876	0	0	2,554100492	13,96132901	EEF1B2;RPS17;RPL32;RPS19;RPS8;RPS5;RPS6;RPL35A;RPSA;RPS3A;RPL39
Guanethidine-132_mg/kg_in_CMC-Rat-Heart-1d-dn	11/296	0,004227092	0,034861334	0	0	2,554100492	13,96132901	EEF1B2;RPS17;RPL32;RPS19;RPS8;COL5A3;RPS6;RPSA;RPS3A;RPL10A;RPL39
Lead_(II)_Acetate-200_mg/kg_in_Saline-Rat-Bone_marrow-0.25d-up	11/296	0,004227092	0,034824868	0	0	2,554100492	13,96132901	RPS17;RPL32;RPS8;RPS5;FUNDC1;RPL13A;RPL35A;RPS20;RPSA;RPS3A;TPT1
Clomipramine-115_mg/kg_in_Water-Rat-Heart-1d-dn	12/340	0,004323914	0,035585315	0	0	2,425712553	13,20459497	EEF1B2;RPS17;RPL32;RPS19;FUNDC1;RPS6;RPSA;RPS3A;RPL10A;SEPP1;RPL39;TPT1
17-Methyltestosterone-2000_mg/kg_in_CMC-Rat-Liver-1d-up	11/297	0,004334523	0,035635388	0	0	2,545500827	13,85043595	RPS17;RPL32;RPS19;RPS8;RPS6;RPS20;RPSA;RPS3A;RPL10A;RPL39;TPT1
Carbimazole-400_mg/kg_in_Water-Rat-Liver-3d-up	11/297	0,004334523	0,035598229	0	0	2,545500827	13,85043595	RPS17;RPL32;RPS19;RPS8;RPS5;RPS6;RPSA;RPS3A;RPL10A;SEPP1;RPL39
Cyclosporin_A-350_mg/kg_in_Corn_Oil-Rat-Heart-1d-dn	11/297	0,004334523	0,035561147	0	0	2,545500827	13,85043595	EEF1B2;RPS17;RPL32;RPS19;ARL2BP;RPS8;RPS6;RPL35A;RPS20;RPS3A;RPL10A
Doxepin-147_mg/kg_in_CMC-Rat-Heart-5d-up	11/297	0,004334523	0,035524143	0	0	2,545500827	13,85043595	EEF1B2;RPS17;CXCL11;RPL32;RPS8;RPS5;RPS6;RPL35A;RPS3A;RPL39;TPT1
Norethindrone_Acetate-125_mg/kg_in_Corn_Oil-Rat-Liver-3d-up	11/297	0,004334523	0,035487215	0	0	2,545500827	13,85043595	RPS17;RPS19;RPS8;FEM1B;RPS6;RPS20;RPSA;RPS3A;RPL10A;SEPP1;RPL39
Sulindac-64_mg/kg_in_Corn_Oil-Rat-Liver-5d-up	11/297	0,004334523	0,035450365	0	0	2,545500827	13,85043595	EEF1B2;RPS17;RPL32;RPS19;RPS6;RPS20;RPSA;RPS3A;RPL10A;RPL39;TPT1
Cycloheximide-0.25_mg/kg_in_Water-Rat-Liver-3d-up	13/385	0,00439776	0,035930249	0	0	2,320703352	12,59366781	RPL32;RPS8;RPS5;RPS6;RPL35A;RPSA;RPS3A;RPL10A;EEF1B2;RPS17;RPS19;RPS20;RPL39
Rofecoxib-800_mg/kg_in_Corn_Oil-Rat-Liver-3d-dn	12/341	0,004425035	0,036115619	0	0	2,418599027	13,10996085	EEF1B2;RPL32;RPS19;RPS8;RPS5;RPS6;RPL35A;RPS20;RPSA;RPL10A;RPL39;TPT1
Carmustine-4_mg/kg_in_Corn_Oil-Rat-Bone_marrow-3d-dn	11/298	0,004444117	0,036233817	0	0	2,536958878	13,7406108	RPS19;RPS8;FEM1B;RPS6;RPS20;RPSA;RPS3A;RPL10A;SEPP1;RPL39;GOLGA7
Clozapine-69_uM_in_DMSO-Rat-Primary_rat_hepatocytes-0.67d-up	11/298	0,004444117	0,036196347	0	0	2,536958878	13,7406108	EEF1B2;RPS17;RPL32;RPS19;RPS5;RPS6;RPL35A;RPS20;RPS3A;RPL39;PDRG1
Diethylstilbestrol-2.8_mg/kg_in_Corn_Oil-Rat-Liver-0.25d-up	11/298	0,004444117	0,036158954	0	0	2,536958878	13,7406108	EEF1B2;RPS17;RPS19;RPS8;FEM1B;RPS5;RPS6;RPS20;RPSA;RPS3A;RPL10A
Ketoconazole-114_mg/kg_in_Corn_Oil-Rat-Liver-1d-dn	11/298	0,004444117	0,036121638	0	0	2,536958878	13,7406108	EEF1B2;C1QA;RPS17;RPL32;RPS8;RPS6;RPSA;RPS3A;RPL10A;SEPP1;TPT1
Methyldopa-325_mg/kg_in_Water-Rat-Heart-3d-up	11/298	0,004444117	0,036084399	0	0	2,536958878	13,7406108	RPS17;RPL32;ARL2BP;RPS8;RPS5;RPS6;RPL35A;RPS20;RPSA;RPS3A;TPT1
Miconazole-920_mg/kg_in_Corn_Oil-Rat-Liver-3d-up	11/298	0,004444117	0,036047237	0	0	2,536958878	13,7406108	RPS17;RPL32;RPS19;RPS8;RPS5;RPS6;RPS20;RPSA;RPS3A;RPL10A;TPT1
Bisphenol_A-100_mg/kg_in_Corn_Oil-Rat-Liver-3d-dn	12/342	0,004528043	0,036690191	0	0	2,4115271	13,01613422	EEF1B2;C1QA;RPS17;RPL32;RPS19;RPS6;RPSA;RPS3A;RPL10A;RPL39;GOLGA7;TPT1
Cephalexin-2500_mg/kg_in_CMC-Rat-Kidney-5d-up	12/342	0,004528043	0,036652483	0	0	2,4115271	13,01613422	CXCL11;RPL32;RPS19;ARL2BP;RPS8;RPS6;RPS20;RPSA;RAPGEF5;RPL10A;SEPP1;TPT1
Ibuprofen-90_mg/kg_in_Corn_Oil-Rat-Liver-3d-dn	12/342	0,004528043	0,036614852	0	0	2,4115271	13,01613422	EEF1B2;RPL32;RPS19;RPS8;RPS5;RPS6;RPL35A;RPS20;RPSA;RPL10A;RPL39;TPT1
Naproxen-134_mg/kg_in_Corn_Oil-Rat-Liver-0.25d-up	12/342	0,004528043	0,036577299	0	0	2,4115271	13,01613422	EEF1B2;RPL32;RPS19;ARL2BP;RPS6;RPS20;RPS3A;RPL10A;SEPP1;RPL39;TPT1;PDRG1
6-Mercaptopurine-25_mg/kg_in_Corn_Oil-Rat-Liver-0.25d-up	11/299	0,004555905	0,036764663	0	0	2,528474066	13,63184061	RPS17;RPL32;RPS19;RPS8;RPS6;RPS20;RPSA;RPS3A;RPL10A;SEPP1;TPT1
Antipyrine-1500_mg/kg_in_CMC-Rat-Kidney-5d-up	11/299	0,004555905	0,036727033	0	0	2,528474066	13,63184061	EEF1B2;RPS17;RPL32;RPS8;RPS5;ARL6IP5;RPL13A;RPL35A;RPSA;SEPP1;TOMM20
Fludrocortisone_Acetate-125_mg/kg_in_CMC-Rat-Kidney-3d-up	11/299	0,004555905	0,03668948	0	0	2,528474066	13,63184061	EEF1B2;CXCL11;RPS19;RPS6;ARL6IP5;RPS20;RPSA;RPL10A;SEPP1;RPL39;TPT1
Praziquantel-1200_mg/kg_in_CMC-Rat-Liver-3d-dn	11/299	0,004555905	0,036652003	0	0	2,528474066	13,63184061	C1QA;RPS17;RPL32;RPS8;ARL6IP5;RPL35A;RPS20;RPS3A;SEPP1;RPL39;TPT1
Flurbiprofen-10_mg/kg_in_CMC-Rat-Kidney-5d-up	12/343	0,004632964	0,037233902	0	0	2,404496408	12,92310648	EEF1B2;C1QA;RPS17;RPL32;RPS19;RPS8;RPS6;RPSA;RPS3A;RPL10A;RPL39;PDRG1
Insulin-0.56_mg/kg_in_Saline-Rat-Liver-3d-up	12/343	0,004632964	0,037195947	0	0	2,404496408	12,92310648	EEF1B2;C1QA;RPS17;RPL32;RPS19;RPS6;RPS20;RPS3A;RPL10A;SEPP1;RPL39;TPT1
Sparteine-2222_uM_in_DMSO-Rat-Primary_rat_hepatocytes-1d-dn	12/343	0,004632964	0,03715807	0	0	2,404496408	12,92310648	EEF1B2;RPS17;RPS19;RPS6;ARL6IP5;RPS20;RPSA;RPS3A;SEPP1;RPL39;SLC16A3;TPT1
Sulfamethoxazole-1000_mg/kg_in_Water-Rat-Kidney-3d-up	12/343	0,004632964	0,037120269	0	0	2,404496408	12,92310648	RPS17;RPL32;RPS19;ARL2BP;RPS8;FEM1B;RPS6;RPS20;RPSA;RPS3A;RAPGEF5;TPT1
Allyl_Alcohol-16_mg/kg_in_Saline-Rat-Kidney-0.25d-up	11/300	0,004669918	0,037378327	0	0	2,520045819	13,5241126	EEF1B2;RPS17;RPL32;RPS8;RPS5;RPL35A;RPSA;RPS3A;RPL39;TOMM20;TPT1
Benzoic_Acid-1700_mg/kg_in_CMC-Rat-Liver-5d-up	11/300	0,004669918	0,03734038	0	0	2,520045819	13,5241126	EEF1B2;RPS17;RPL32;RPS19;RPS8;RPS6;RPS20;RPSA;RPL10A;RPL39;TPT1
Carbon_Tetrachloride-400_mg/kg_in_Corn_Oil-Rat-Liver-1d-up	11/300	0,004669918	0,037302509	0	0	2,520045819	13,5241126	EEF1B2;RPS17;RPL32;RPS19;RPS8;RPS6;RPS20;RPSA;RPS3A;RPL10A;SEPP1
Chloroform-600_mg/kg_in_Corn_Oil-Rat-Liver-1d-up	11/300	0,004669918	0,037264715	0	0	2,520045819	13,5241126	EEF1B2;RPS19;ARL2BP;RPS8;RPS6;RPS20;RPSA;RPS3A;RPL10A;RPL39;TPT1

Ciprofloxacin-72_mg/kg_in_Corn_Oil-Rat-Liver-0.25d-up	11/300	0,004669918	0,037226998	0	0	2,520045819	13,5241126	RPS17;RPL32;RPS19;RPS8;RPS5;RPL35A;RPSA;RPS3A;RPL39;TOMM20;TPT1
Crotamiton-750_mg/kg_in_Corn_Oil-Rat-Liver-1d-up	11/300	0,004669918	0,037189357	0	0	2,520045819	13,5241126	RPS17;RPL32;RPS8;RPS5;RPS6;RPL13A;RPL35A;RPS20;RPSA;RPS3A;RPL10A
Daunorubicin-1.5_uM_in_DMSO-Rat-Primary_rat_hepatocytes-0.67d-up	11/300	0,004669918	0,037151792	0	0	2,520045819	13,5241126	EEF1B2;RPS17;CXCL11;RPL32;CLP1;RPS8;RPS6;ARL6IP5;RPL35A;RPS3A;GOLGA7
Harringtonine-23_mg/kg_in_Saline-Rat-Bone_marrow-5d-up	11/300	0,004669918	0,037114303	0	0	2,520045819	13,5241126	EEF1B2;RPL32;RPS19;ARL2BP;RPS8;RPS6;ARL6IP5;RPS20;RPSA;RPL10A;RPL39
Lead(IV)_Acetate-600_mg/kg_in_Saline-Rat-Liver-1d-dn	11/300	0,004669918	0,037076889	0	0	2,520045819	13,5241126	EEF1B2;RPL32;RPS8;RPS5;RPS6;RPL35A;RPSA;RPS3A;RPL10A;SEPP1;TPT1
Lovastatin-1500_mg/kg_in_Corn_Oil-Rat-Skeletal_muscle-5d-up	11/300	0,004669918	0,037039551	0	0	2,520045819	13,5241126	RPS17;RPL32;RPS19;RPS8;RPS5;RPS6;RPS20;RPS3A;RPL10A;RPL39;TPT1
Mitotane-5000_mg/kg_in_Water-Rat-Bone_marrow-1d-dn	11/300	0,004669918	0,037002288	0	0	2,520045819	13,5241126	EEF1B2;C1QA;RPS17;RPL32;RPS8;RPS5;ARL6IP5;RPS20;RPS3A;RPL39;DUSP14
Nimodipine-1100_mg/kg_in_Corn_Oil-Rat-Heart-3d-up	11/300	0,004669918	0,036965099	0	0	2,520045819	13,5241126	C1QA;RPS17;RPL32;RPS19;RPS6;RPS20;RPS3A;RPL10A;SEPP1;RPL39;TPT1
Penicillamine-852_mg/kg_in_Water-Rat-Liver-1d-up	11/300	0,004669918	0,036927986	0	0	2,520045819	13,5241126	EEF1B2;C1QA;RPL32;RPS19;RPS8;RPS6;RPL35A;RPS20;RPS3A;RPL39;TPT1
Roflumilast-17_mg/kg_in_CMC-Rat-Liver-3d-up	11/300	0,004669918	0,036890947	0	0	2,520045819	13,5241126	EEF1B2;RPS17;RPS8;RPS5;RPS6;RPS20;RPSA;RPS3A;RPL10A;RPL39;TPT1
Carboplatin-6_mg/kg_in_Saline-Rat-Kidney-3d-dn	11/301	0,004786186	0,037771541	0	0	2,511673574	13,41741424	EEF1B2;RPS17;RPS19;ARL2BP;CLP1;RPS5;FUNDC1;RPS3A;RPL10A;SEPP1;RPL39
Diethylstilbestrol-280_mg/kg_in_Corn_Oil-Rat-Bone_marrow-5d-up	11/301	0,004786186	0,037733732	0	0	2,511673574	13,41741424	EEF1B2;C1QA;RPL32;RPS5;RPS6;NKG7;RPL35A;RPS20;RPL10A;SEPP1;RPL39
Disulfiram-500_mg/kg_in_Water-Rat-Liver-1d-up	11/301	0,004786186	0,037695998	0	0	2,511673574	13,41741424	EEF1B2;RPS17;RPL32;RPS19;RPS6;RPL35A;RPS20;RPSA;RPS3A;RPL10A;RPL39
Finasteride-25_mg/kg_in_Corn_Oil-Rat-Liver-1d-dn	11/301	0,004786186	0,03765834	0	0	2,511673574	13,41741424	EEF1B2;RPS17;RPL32;RPS6;RPL35A;RPSA;RPS3A;RPL10A;SEPP1;RPL39;TPT1
Lead(IV)_Acetate-600_mg/kg_in_Saline-Rat-Bone_marrow-5d-up	11/301	0,004786186	0,037620757	0	0	2,511673574	13,41741424	RPL32;RPS8;FEM1B;RPS5;FUNDC1;RPS6;RPL35A;RPS20;RPSA;RPS3A;TPT1
Leucovorin-1500_mg/kg_in_CMC-Rat-Bone_marrow-3d-dn	11/301	0,004786186	0,037583248	0	0	2,511673574	13,41741424	EEF1B2;RPS17;RPS8;RPS5;RPS6;NKG7;RPL13A;RPL35A;RPS3A;RPL39;TPT1
Nevirapine-29_mg/kg_in_Saline-Rat-Liver-5d-dn	11/301	0,004786186	0,037545815	0	0	2,511673574	13,41741424	EEF1B2;C1QA;RPS17;RPL32;RPS19;RPS6;RPS3A;RPL10A;SEPP1;RPL39;TPT1
Paroxetine-104_mg/kg_in_Water-Rat-Brain-3d-up	11/301	0,004786186	0,037508456	0	0	2,511673574	13,41741424	EEF1B2;RPS17;RPL32;RPS19;RPS8;RPL35A;RPS20;RPSA;RPS3A;RPL10A;TPT1
Sodium_Arsenite-30_mg/kg_in_Saline-Rat-Liver-3d-up	11/301	0,004786186	0,037471171	0	0	2,511673574	13,41741424	EEF1B2;RPS17;RPL32;RPS19;RPS8;RPS5;RPL35A;RPS20;RPSA;RPS3A;RPL10A
Nimesulide-162_mg/kg_in_Corn_Oil-Rat-Liver-3d-up	10/259	0,004809218	0,037614102	0	0	2,653610901	14,16290729	EEF1B2;RPS19;RPS8;RPS5;RPS6;RPS20;RPSA;RPS3A;RPL10A;RPL39
Tranexamic_Acid-2000_mg/kg_in_CMC-Rat-Heart-1d-dn	10/259	0,004809218	0,037576786	0	0	2,653610901	14,16290729	EEF1B2;RPS17;RPL32;RPS8;RPS5;RPS6;RPL35A;RPSA;RPS3A;TOMM20
Carbon_Tetrachloride-1175_mg/kg_in_Corn_Oil-Rat-Liver-3d-up	12/345	0,004848645	0,0378473	0	0	2,390557299	12,73941387	EEF1B2;RPL32;RPS19;RPS8;RPS5;RPS6;RPL35A;RPS20;RPSA;RPL10A;RPL39;TPT1
Aldosterone-0.065_mg/kg_in_Saline-Rat-Heart-1d-dn	11/302	0,004904739	0,038247255	0	0	2,503356774	13,31173313	EEF1B2;RPS17;RPL32;RPS19;RPS6;RPS20;RPSA;RPS3A;RPL10A;SEPP1;RPL39
Betamethasone-79_mg/kg_in_Corn_Oil-Rat-Bone_marrow-3d-dn	11/302	0,004904739	0,038209424	0	0	2,503356774	13,31173313	RPS17;RPL32;RPS19;FEM1B;RPS6;NKG7;RPSA;RPS3A;RPL10A;RPL39;GOLGA7
Bithionol-59_mg/kg_in_Corn_Oil-Rat-Kidney-3d-up	11/302	0,004904739	0,038171667	0	0	2,503356774	13,31173313	RPS17;RPL32;ARL2BP;RPS8;RPS5;ARL6IP5;RPL13A;RPL35A;RPS20;RPSA;RPS3A
Busulfan-3_mg/kg_in_Corn_Oil-Rat-Bone_marrow-0.25d-up	11/302	0,004904739	0,038133985	0	0	2,503356774	13,31173313	EEF1B2;C1QA;RPS17;RPL32;RPS5;RPL13A;RPL35A;RPS20;RPS3A;RPL39;DUSP14
Metronidazole-1500_mg/kg_in_Water-Rat-Liver-3d-up	11/302	0,004904739	0,038096378	0	0	2,503356774	13,31173313	EEF1B2;RPS17;RPL32;RPS19;RPS8;RPS5;RPS6;RPL35A;RPSA;RPS3A;TPT1
NN'-Diphenyl-P-Phenylenediamine-1000_mg/kg_in_CMC-Rat-Liver-5d-up	11/302	0,004904739	0,038058845	0	0	2,503356774	13,31173313	EEF1B2;RPS17;RPL32;RPS19;RPS8;FEM1B;RPS6;RPS3A;RPL10A;SEPP1;RPL39
Naproxen-10_mg/kg_in_Corn_Oil-Rat-Liver-5d-up	11/302	0,004904739	0,038021385	0	0	2,503356774	13,31173313	EEF1B2;RPS17;RPS19;RPS8;RPS6;RPL35A;RPS20;RPS3A;SEPP1;RPL39;GOLGA7
Phenobarbital-54_mg/kg_in_Water-Rat-Liver-5d-dn	11/302	0,004904739	0,037983999	0	0	2,503356774	13,31173313	EEF1B2;RPL32;RPS8;RPS6;RPS20;RPSA;RPS3A;RPL10A;SEPP1;RPL39;TPT1
Troxipide-1200_mg/kg_in_CMC-Rat-Heart-1d-dn	11/302	0,004904739	0,037946687	0	0	2,503356774	13,31173313	RPS17;RPL32;RPS8;FEM1B;COL5A3;RPS6;ARL6IP5;RPL35A;RPSA;RPL39;TPT1
Carmustine-16_mg/kg_in_Corn_Oil-Rat-Bone_marrow-5d-up	11/303	0,00502561	0,038843677	0	0	2,49509487	13,20705709	EEF1B2;C1QA;RPS17;RPL32;RPS19;RPS8;RPS5;RPL35A;RPS20;RPSA;SEPP1
Cimetidine-2000_mg/kg_in_CMC-Rat-Heart-5d-up	11/303	0,00502561	0,038805595	0	0	2,49509487	13,20705709	C1QA;RPS17;RPL32;RPS6;RPL35A;RPS20;RPSA;RPS3A;RPL39;TOMM20;TPT1
Lead(IV)_Acetate-200_mg/kg_in_Saline-Rat-Liver-0.25d-up	11/303	0,00502561	0,038767587	0	0	2,49509487	13,20705709	RPS17;RPL32;RPS8;RPS6;RPL35A;RPS20;RPSA;RPS3A;RPL10A;SEPP1;RPL39
Mitomycin_C-0.5_mg/kg_in_Saline-Rat-Liver-3d-up	11/303	0,00502561	0,038729654	0	0	2,49509487	13,20705709	EEF1B2;RPS17;RPL32;RPS19;RPS8;RPS5;RPS6;RPL35A;RPS20;RPS3A;RPL10A
Modafinil-17.5_mg/kg_in_Saline-Rat-Liver-1d-dn	11/303	0,00502561	0,038691795	0	0	2,49509487	13,20705709	EEF1B2;RPL32;RPS8;RPS5;RPS6;RPL35A;RPS3A;RPL10A;SEPP1;RPL39;TPT1
NN-Dimethylformamide-2100_mg/kg_in_Saline-Rat-Liver-1d-dn	11/303	0,00502561	0,03865401	0	0	2,49509487	13,20705709	EEF1B2;RPS17;RPL32;RPS19;RPS8;RPS6;RPL35A;RPS20;RPS3A;SEPP1;TPT1
Ascorbic_Acid-2000_mg/kg_in_Water-Rat-Kidney-5d-dn	10/261	0,00507189	0,03897191	0	0	2,633276718	13,91434401	EEF1B2;RPL32;RPS19;CLP1;RPS8;ARL6IP5;RPL35A;RPS20;RPL39;TPT1
Neostigmine_Bromide-40_mg/kg_in_Water-Rat-Liver-1d-up	12/347	0,005072279	0,03893691	0	0	2,37677887	12,55881649	EEF1B2;RPS17;RPL32;RPS19;RPS5;RPS6;RPL35A;RPS20;RPSA;RPS3A;SEPP1;TPT1
44'-Methylenedianiline-296_uM_in_DMSO-Rat-Primary_rat_hepatocytes-0.67d-up	11/304	0,00514883	0,039486058	0	0	2,486887321	13,10337412	EEF1B2;RPS17;RPL32;RPS8;RPS5;ARL6IP5;RPS3A;RPL10A;RPL39;TPT1;PDRG1
Beta-Naphthoflavone-1500_mg/kg_in_CMC-Rat-Liver-5d-up	11/304	0,00514883	0,039447648	0	0	2,486887321	13,10337412	C1QA;RPS17;RPL32;RPS19;CLP1;RPS8;RPS5;RPSA;RPS3A;RPL10A;SEPP1
Bis(2-Ethylhexyl)Phthalate-1000_mg/kg_in_Corn_Oil-Rat-Liver-3d-dn	11/304	0,00514883	0,039409312	0	0	2,486887321	13,10337412	EEF1B2;C1QA;RPS17;RPL32;CLP1;RPS8;RPS6;RPS3A;RPL10A;SEPP1;TPT1
Cetraxate-1503_mg/kg_in_CMC-Rat-Liver-3d-up	11/304	0,00514883	0,03937105	0	0	2,486887321	13,10337412	EEF1B2;RPS17;RPL32;RPS19;RPS6;RPS20;RPSA;RPS3A;RPL10A;SEPP1;RPL39
Colchicine-1000_uM_in_DMSO-Rat-Primary_rat_hepatocytes-1d-dn	11/304	0,00514883	0,039332863	0	0	2,486887321	13,10337412	EEF1B2;CXCL11;RPL32;RPS19;RPS6;RPS20;RPSA;RPS3A;RPL10A;SEPP1;RPL39
Etoposide-100_mg/kg_in_Corn_Oil-Rat-Spleen-1d-dn	11/304	0,00514883	0,03929475	0	0	2,486887321	13,10337412	EEF1B2;RPS17;RPL32;RPS8;RPS5;RPL13A;RPL35A;RPS20;RPSA;RPL39;TPT1
Isoprenaline-4.2_mg/kg_in_Saline-Rat-Heart-5d-up	11/304	0,00514883	0,03925671	0	0	2,486887321	13,10337412	EEF1B2;C1QA;RPS17;RPS19;RPS8;RPS6;RPS20;RPSA;RPL10A;SEPP1;RPL39
Pioglitazone-300_mg/kg_in_Corn_Oil-Rat-Liver-1d-dn	11/304	0,00514883	0,039218745	0	0	2,486887321	13,10337412	C1QA;RPS17;RPS19;RPS8;FUNDC1;RPS6;RPS20;RPS3A;RPL10A;RPL39;TPT1
Promethazine-2.3_mg/kg_in_Saline-Rat-Liver-3d-up	11/304	0,00514883	0,039180852	0	0	2,486887321	13,10337412	EEF1B2;C1QA;RPS17;RPL32;RPS19;RPS6;RPL35A;RPSA;RPS3A;RPL39;TPT1
Roflumilast-17_mg/kg_in_CMC-Rat-Liver-4d-up	11/304	0,00514883	0,039143033	0	0	2,486887321	13,10337412	EEF1B2;RPS17;RPL32;RPS19;RPS8;RPS5;RPS6;RPS20;RPS3A;RPL39;TPT1
Azathioprine-160_mg/kg_in_Corn_Oil-Rat-Liver-3d-dn	12/348	0,005187143	0,039396274	0	0	2,369949046	12,4696582	EEF1B2;C1QA;RPS17;RPL32;RPS19;RPS6;RPSA;RPS3A;RPL10A;RPL39;GOLGA7;TPT1
1-Naphthyl_Isothiocyanate-30_mg/kg_in_Corn_Oil-Rat-Kidney-3d-up	11/305	0,005274429	0,040020618	0	0	2,478733592	13,00067237	EEF1B2;RPS17;RPL32;RPS19;RPS8;RPS5;RPS6;RPL13A;RPL35A;RPS20;RPS3A
Artemether-74_mg/kg_in_Corn_Oil-Rat-Liver-1d-up	11/305	0,005274429	0,039982099	0	0	2,478733592	13,00067237	C1QA;RPS17;RPL32;RPS5;RPL35A;RPSA;RPS3A;SEPP1;RPL39;GOLGA7;TPT1
Benoxaprofen-89_mg/kg_in_CMC-Rat-Liver-5d-up	11/305	0,005274429	0,039943655	0	0	2,478733592	13,00067237	EEF1B2;RPS19;RPS8;RPS5;RPS6;RPS20;RPSA;RPS3A;RPL10A;RPL39;TPT1
Catechol-195_mg/kg_in_Saline-Rat-Bone_marrow-1d-dn	11/305	0,005274429	0,039905284	0	0	2,478733592	13,00067237	RPS17;RPL32;RPS19;RPS8;RPS6;RPS20;RPSA;RPS3A;RPL10A;RPL39;TPT1
Doxorubicin-3_mg/kg_in_Saline-Rat-Spleen-3d-up	11/305	0,005274429	0,039866988	0	0	2,478733592	13,00067237	CXCL11;RPS19;RPS8;RPS5;RPL35A;RPS20;RAPGEF5;RPL10A;SEPP1;GOLGA7;TPT1
Etoposide-100_mg/kg_in_Corn_Oil-Rat-Spleen-0.25d-up	11/305	0,005274429	0,039828764	0	0	2,478733592	13,00067237	EEF1B2;C1QA;RPL32;RPS19;RPS8;RPS5;RPS6;RPL35A;RPS3A;RPL10A;SEPP1
Famciclovir-112_mg/kg_in_Saline-Rat-Bone_marrow-3d-dn	11/305	0,005274429	0,039790614	0	0	2,478733592	13,00067237	C1QA;RPL32;RPS19;NKG7;RPS20;RPSA;RPS3A;RPL10A;SEPP1;RPL39;TPT1
Foscarnet-350_mg/kg_in_Saline-Rat-Bone_marrow-3d-dn	11/305	0,005274429	0,039752537	0	0	2,478733592	13,00067237	RPS17;RPL32;RPS19;RPS8;RPS6;NKG7;RPS20;RPSA;SEPP1;RPL39;TPT1
Losartan-1000_mg/kg_in_Water-Rat-Heart-3d-up	11/305	0,005274429	0,039714533	0	0	2,478733592	13,00067237	RPS17;RPL32;RPS19;ARL2BP;RPS8;RPS5;RPS6;RPSA;RPS3A;RAPGEF5;RPL10A
Leflunomide-30_mg/kg_in_Corn_Oil-Rat-Liver-0.25d-up	12/349	0,005304072	0,039899588	0	0	2,363158361	12,38124956	EEF1B2;RPS17;RPL32;RPS19;RPS8;RPS6;RPL35A;RPS20;RPSA;RPS3A;RPL10A;SEPP1
Pyrogallol-1000_mg/kg_in_Water-Rat-Liver-3d-up	12/349	0,005304072	0,039861516	0	0	2,363158361	12,38124956	RPS17;CXCL11;RPL32;RPS19;ARL2BP;RPS8;RPS6;RPS20;RPSA;RPS3A;RPL10A;TPT1
Leflunomide-30_mg/kg_in_Corn_Oil-Rat-Kidney-1d-up	9/222	0,005368555	0,040307663	0	0	2,786291446	14,56449294	RPS17;RPL32;RPS8;RPS5;FUNDC1;RPL35A;RPS20;RPS3A;TPT1
1-Naphthyl_Isothiocyanate-60_mg/kg_in_Corn_Oil-Rat-Bone_marrow-3d-up	11/306	0,005402439	0,040523441	0	0	2,470633156	12,8989402	EEF1B2;C1QA;RPL32;RPS5;RPS6;RPL35A;RPS20;RPS3A;RPL39;SLC16A3;TPT1
Citalopram-40_mg/kg_in_Corn_Oil-Rat-Liver-3d-dn	11/306	0,005402439	0,040484884	0	0	2,470633156	12,8989402	EEF1B2;RPS17;RPL32;RPS19;RPS8;RPS6;RPS20;RPSA;RPS3A;RPL10A;TPT1
Manganese_(II)_Chloride-700_mg/kg_in_Water-Rat-Liver-5d-dn	11/306	0,005402439	0,0404464	0	0	2,470633156	12,8989402	RPS17;RPL32;RPS19;RPS8;RPS6;RPL35A;RPS20;RPSA;SEPP1;RPL39;TPT1
Rofecoxib-1550_mg/kg_in_Corn_Oil-Rat-Liver-5d-dn	11/306	0,005402439	0,04040799	0	0	2,470633156	12,8989402	C1QA;RPS17;RPL32;RPS19;RPS8;FEM1B;RPS6;RPS3A;RPL10A;RPL39;TPT1
Zomepirac-2.8_mg/kg_in_Saline-Rat-Kidney-3d-up	11/306	0,005402439	0,040369652	0	0	2,470633156	12,8989402	EEF1B2;C1QA;RPS17;RPL32;RPS5;ARL6IP5;RPL35A;RPS20;RPSA;RPS3A;RPL39
Penicillamine-100_mg/kg_in_Water-Rat-Liver-3d-dn	12/350	0,005423092	0,040485565	0	0	2,35640648	12,29358273	EEF1B2;C1QA;RPS17;RPL32;RPS19;RPS6;RPL35A;RPS20;RPSA;RPL10A;RPL39;TPT1
Prochlorperazine-85_mg/kg_in_CMC-Rat-Heart-3d-up	10/264	0,005486869	0,040922901	0	0	2,603353119	13,55148761	RPS17;RPL32;RPS19;RPS8;FUNDC1;RPS6;RPS3A;RAPGEF5;SEPP1;TPT1
Naproxen-10_mg/kg_in_Corn_Oil-Rat-Kidney-1d-dn	9/223	0,005525116	0,041169173	0	0	2,773796866	14,41944704	EEF1B2;RPS17;RPL32;ARL2BP;RPS8;RPS6;RPL35A;RPS3A;TPT1
Cholecalciferol-8_mg/kg_in_CMC-Rat-Kidney-5d-up	11/307	0,005532893	0,041188155	0	0	2,462585491	12,79816612	EEF1B2;C1QA;RPS17;CXCL11;RPS19;CLP1;RPS8;RPS6;RPS20;RPSA;TPT1
Cytarabine-23_mg/kg_in_Saline-Rat-Liver-5d-dn	11/307	0,005532893	0,041149261	0	0	2,462585491	12,79816612	EEF1B2;C1QA;RPS17;RPL32;RPS19;RPS8;RPS6;RPS20;RPS3A;SEPP1;GOLGA7
Cytarabine-487_mg/kg_in_Saline-Rat-Bone_marrow-5d-up	11/307	0,005532893	0,041110441	0	0	2,462585491	12,79816612	RPS17;RPL32;RPS19;ARL2BP;RPS8;RPS6;RPS20;RPSA;RPS3A;RPL10A;TPT1
Gentian_Violet-18_mg/kg_in_Corn_Oil-Rat-Liver-5d-dn	11/307	0,005532893	0,041071694	0	0	2,462585491	12,79816612	RPS17;RPL32;RPS19;RPS8;RPS6;ARL6IP5;RPL35A;RPS3A;RPL10A;RPL39;TPT1
Melphalan-12_mg/kg_in_CMC-Rat-Spleen-5d-up	11/307	0,005532893	0,041033021	0	0	2,462585491	12,79816612	EEF1B2;RPL32;RPS19;RPS5;FUNDC1;RPS6;RPS20;RPSA;RPL10A;SEPP1;RPL39
Tobramycin-40_mg/kg_in_Saline-Rat-Kidney-14d-up	11/307	0,005532893	0,040994419	0	0	2,462585491	12,79816612	EEF1B2;RPS17;CXCL11;RPL32;RPS19;RPS8;RPS6;RPS3A;RPL10A;RPL39;TPT1
Troglitazone-100_mg/kg_in_Corn_Oil-Rat-Liver-3d-dn	13/397	0,005659246	0,041891184	0	0	2,250556147	11,64542324	RPL32;RPS5;RPS6;RPL35A;RPSA;RPS3A;RPL10A;SEPP1;EEF1B2;RPS17;RPS19;RPL39;TPT1
Atorvastatin-300_mg/kg_in_Corn_Oil-Rat-Kidney-5d-up	11/308	0,005665822	0,041900485	0	0	2,454590083	12,69833882	RPS17;RPL32;RPS19;ARL2BP;RPS8;RPS5;RPS6;RPL35A;RPS20;RPS3A;TPT1
Bezafibrate-617_mg/kg_in_Corn_Oil-Rat-Liver-3d-up	11/308	0,005665822	0,041861179	0	0	2,454590083	12,69833882	EEF1B2;RPL32;RPS19;RPS8;RPS5;RPS6;RPL35A;RPS20;RPSA;RPL10A;TPT1
Ethylene_Glycol-352_mg/kg_in_Water-Rat-Bone_marrow-3d-dn	11/308	0,005665822	0,041821946	0	0	2,454590083	12,69833882	RPS19;ARL2BP;RPS8;FEM1B;FUNDC1;RPS6;RPS20;RPSA;RPS3A;RPL39;TPT1
Fluconazole-10_mg/kg_in_Corn_Oil-Rat-Liver-0.25d-up	11/308	0,005665822	0,041782787	0	0	2,454590083	12,69833882	EEF1B2;RPS17;RPL32;RPS19;RPS8;RPS6;RPS20;RPS3A;RPL10A;SEPP1;TPT1
Phenelzine-27_mg/kg_in_Water-Rat-Brain-5d-up	11/308	0,005665822	0,041743701	0	0	2,454590083	12,69833882	RPS19;ARL2BP;FUNDC1;RPS6;ARL6IP5;RPSA;RPL10A;SEPP1;RPL39;GOLGA7;TPT1
Sulfanilamide-636_mg/kg_in_Corn_Oil-Rat-Kidney-1d-up	11/308	0,005665822	0,041704688	0	0	2,454590083	12,69833882	EEF1B2;RPS17;RPL32;RPS19;ARL2BP;RPS8;RPS6;RPL35A;RPS20;RPS3A;RPL39
Tacrolimus-134_mg/kg_in_CMC-Rat-Kidney-5d-up	11/308	0,005665822	0,041665748	0	0	2,454590083	12,69833882	RPL32;RPS19;ARL2BP;RPS8;RPS5;RPL35A;RPS20;RPSA;RAPGEF5;TOMM20;TPT1
Sulindac-750_uM_in_DMSO-Rat-Primary_rat_hepatocytes-1d-dn	12/352	0,00566751	0,041639283	0	0	2,343017807	12,12044367	EEF1B2;CXCL11;RPS19;RPS8;RPS5;FUNDC1;RPS6;ARL6IP5;RPS3A;SEPP1;GOLGA7;TPT1
Lead(IV)_Acetate-600_mg/kg_in_Saline-Rat-Spleen-1d-dn	10/266	0,005777965	0,04241123	0	0	2,583779035	13,31603182	EEF1B2;ARL2BP;RPS8;RPS5;FUNDC1;RPL13A;RPL35A;RPSA;RPS3A;TPT1
Venlafaxine-320_mg/kg_in_Water-Rat-Kidney-3d-dn	10/266	0,005777965	0,042371741	0	0	2,583779035	13,31603182	RPS17;RPL32;RPS19;RPS6;ARL6IP5;RPL35A;RPS20;RPSA;RPL10A;RPL39
Podophyllotoxin-4_mg/kg_in_Corn_Oil-Rat-Bone_marrow-5d-up	12/353	0,005792961	0,042442196	0	0	2,336380363	12,03495625	EEF1B2;C1QA;RPS17;RPL32;RPS19;FEM1B;RPS6;NKG7;RPS20;RPL10A;SEPP1;GOLGA7
Betamethasone-79_mg/kg_in_Corn_Oil-Rat-Liver-1d-dn	11/309	0,005801259	0,042463487	0	0	2,446646426	12,59944715	EEF1B2;C1QA;RPS17;RPL32;RPS19;RPS8;RPS6;RPSA;RPS3A;RPL10A;SEPP1
Bisacodyl-1750_mg/kg_in_CMC-Rat-Intestine-5d-dn	11/309	0,005801259	0,042424059	0	0	2,446646426	12,59944715	EEF1B2;RPS17;RPS19;RPS8;RPS6;ARL6IP5;RPL13A;RPL35A;RPS3A;SEPP1;RPL39

Ciprofloxacin-450_mg/kg_in_Corn_Oil-Rat-Liver-0.25d-up	11/309	0,005801259	0,042384705	0	0	2,446646426	12,59944715	EEF1B2;C1QA;RPL32;RPS19;RPS8;FEM1B;RPS6;RPS20;RPS3A;SEPP1;RPL39
Citalopram-90_mg/kg_in_Corn_Oil-Rat-Liver-0.25d-up	11/309	0,005801259	0,042345424	0	0	2,446646426	12,59944715	EEF1B2;RPS17;RPL32;RPS8;RPS5;RPS6;RPS20;RPSA;RPS3A;RPL10A;SEPP1
Doxazosin-5_mg/kg_in_Water-Rat-Heart-0.25d-up	11/309	0,005801259	0,042306215	0	0	2,446646426	12,59944715	RPS17;RPL32;RPS19;RPS8;RPS5;RPS6;ARL6IP5;RPL35A;RPSA;RPS3A;TPT1
Erythromycin-1500_mg/kg_in_CMC-Rat-Kidney-5d-up	11/309	0,005801259	0,042267079	0	0	2,446646426	12,59944715	EEF1B2;RPS17;RPS19;CLP1;RPS8;RPS6;RPS20;RPS3A;SEPP1;RPL39;TPT1
Fludrocortisone_Acetate-125_mg/kg_in_CMC-Rat-Heart-1d-dn	11/309	0,005801259	0,042228015	0	0	2,446646426	12,59944715	RPS17;RPL32;RPS19;ARL2BP;RPS8;RPS6;RPS20;RPSA;RPS3A;RPL10A;RPL39
Fluocinolone_Acetonide-2.5_mg/kg_in_Corn_Oil-Rat-Heart-5d-dn	11/309	0,005801259	0,042189023	0	0	2,446646426	12,59944715	EEF1B2;RPS17;RPL32;RPS19;ARL2BP;RPS8;COL5A3;RPSA;RPS3A;RPL10A;RPL39
Hydralazine-280_mg/kg_in_Water-Rat-Liver-0.25d-up	11/309	0,005801259	0,042150103	0	0	2,446646426	12,59944715	RPL32;RPS19;ARL2BP;CLP1;RPS6;RPS20;RPSA;RPS3A;RPL10A;SEPP1;TPT1
Ibuprofen-263_mg/kg_in_Corn_Oil-Rat-Liver-5d-up	11/309	0,005801259	0,042111255	0	0	2,446646426	12,59944715	EEF1B2;C1QA;RPS19;RPS5;RPS6;RPS20;RPSA;RPS3A;RPL10A;RPL39;TPT1
Imatinib-150_mg/kg_in_Water-Rat-Liver-5d-dn	11/309	0,005801259	0,042072479	0	0	2,446646426	12,59944715	EEF1B2;RPS17;RPL32;RPS19;RPS6;RPL35A;RPS20;RPSA;RPS3A;SEPP1;RPL39
Lansoprazole-240_uM_in_DMSO-Rat-Primary_rat_hepatocytes-0.67d-dn	11/309	0,005801259	0,042033774	0	0	2,446646426	12,59944715	CXCL11;RPL32;RPS19;RPS8;ARL6IP5;RPS20;RPSA;RPS3A;RPL10A;SEPP1;TPT1
Noscapine-570_mg/kg_in_CMC-Rat-Spleen-5d-up	11/309	0,005801259	0,04199514	0	0	2,446646426	12,59944715	EEF1B2;RPL32;RPS19;RPS5;FUNDC1;RPS6;RPS20;RPSA;RPL10A;SEPP1;RPL39
Ticrynafen-570_mg/kg_in_CMC-Rat-Liver-1d-up	11/309	0,005801259	0,041956577	0	0	2,446646426	12,59944715	EEF1B2;RPL32;RPS8;RPS5;RPS6;RPS20;RPSA;RPS3A;RPL10A;RPL39;TPT1
Tolazamide-1500_mg/kg_in_CMC-Rat-Kidney-5d-up	11/309	0,005801259	0,041918084	0	0	2,446646426	12,59944715	EEF1B2;RPS17;RPS8;RPS5;ARL6IP5;RPSA;RPS3A;RAPGEF5;RPL10A;SEPP1;TPT1
Trichloroethylene-1230_mg/kg_in_CMC-Rat-Kidney-5d-up	11/309	0,005801259	0,041879663	0	0	2,446646426	12,59944715	EEF1B2;RPS17;CXCL11;RPS8;ARL6IP5;RPSA;RPS3A;RAPGEF5;RPL10A;TOMM20;TPT1
Zopiclone-414_mg/kg_in_Water-Rat-Liver-5d-up	11/309	0,005801259	0,041841311	0	0	2,446646426	12,59944715	RPS17;RPL32;RPS8;RPS5;RPS6;RPL35A;RPS20;RPSA;RPS3A;RPL10A;RPL39
Ethinylestradiol-10_mg/kg_in_Corn_Oil-Rat-Liver-3d-up	10/267	0,005927965	0,042716058	0	0	2,57410196	13,20018616	EEF1B2;RPL32;RPS19;RPS8;RPS6;RPS20;RPSA;RPL10A;RPL39;TPT1
Leflunomide-60_mg/kg_in_Corn_Oil-Rat-Spleen-3d-up	10/267	0,005927965	0,042677012	0	0	2,57410196	13,20018616	EEF1B2;RPS17;RPL32;RPS8;RPS5;RPS6;RPS20;RPSA;RPS3A;RAPGEF5
2-Nitroanisole-740_mg/kg_in_CMC-Rat-Kidney-5d-up	11/310	0,005939234	0,042719094	0	0	2,438754018	12,50148012	EEF1B2;RPS17;CLP1;ARL6IP5;RPSA;RPS3A;RAPGEF5;SEPP1;RPL39;TOMM20;TPT1
Amlodipine-19_mg/kg_in_Corn_Oil-Rat-Liver-3d-up	11/310	0,005939234	0,042680116	0	0	2,438754018	12,50148012	EEF1B2;C1QA;RPS17;RPL32;RPS5;RPL13A;RPL35A;RPS20;RPSA;RPS3A;TPT1
Ethanol-3000_mg/kg_in_Saline-Rat-Bone_marrow-3d-dn	11/310	0,005939234	0,04264121	0	0	2,438754018	12,50148012	EEF1B2;C1QA;RPS17;RPL32;RPS8;RPS6;NKG7;RPSA;RPS3A;RPL10A;TPT1
Methimazole-100_mg/kg_in_Water-Rat-Liver-1d-up	11/310	0,005939234	0,042602375	0	0	2,438754018	12,50148012	EEF1B2;RPS17;RPL32;RPS19;RPS8;RPS5;RPS20;RPSA;RPS3A;RPL10A;RPL39
Procarbazine-27_mg/kg_in_Water-Rat-Bone_marrow-1d-dn	11/310	0,005939234	0,04256361	0	0	2,438754018	12,50148012	EEF1B2;RPS17;RPL32;RPS19;RPS8;RPS6;ARL6IP5;RPS20;RPSA;RPL39;DUSP14
Sodium_Arsenite-10_mg/kg_in_Saline-Rat-Liver-1d-dn	11/310	0,005939234	0,042524916	0	0	2,438754018	12,50148012	C1QA;RPL32;RPS19;CLP1;RPS6;RPS20;RPS3A;RPL10A;SEPP1;RPL39;TPT1
Thioguanine-12_mg/kg_in_Corn_Oil-Rat-Liver-5d-up	11/310	0,005939234	0,042486292	0	0	2,438754018	12,50148012	EEF1B2;RPS17;CLP1;RPS8;RPS6;RPS20;RPSA;RPS3A;SEPP1;RPL39;TPT1
Trichloroethane-3000_mg/kg_in_Water-Rat-Liver-5d-up	11/310	0,005939234	0,042447738	0	0	2,438754018	12,50148012	EEF1B2;C1QA;RPS17;RPL32;RPS8;RPS5;RPS6;ARL6IP5;RPL35A;RPS20;RPS3A
Allyl_Alcohol-25_mg/kg_in_Saline-Rat-Bone_marrow-3d-dn	9/226	0,006016067	0,042957881	0	0	2,736976553	13,99504128	RPL32;RPS19;ARL2BP;RPS8;FUNDC1;RPS6;RPS20;RPSA;RPL10A
Camptothecin-5_mg/kg_in_Saline-Rat-Kidney-3d-dn	12/355	0,006050478	0,043164458	0	0	2,323217656	11,86610847	EEF1B2;RPS17;RPL32;RPS19;ARL2BP;RPS6;RPL35A;RPS20;RPSA;RPS3A;RPL10A;RPL39
Gatifloxacin-770_mg/kg_in_Corn_Oil-Rat-Kidney-5d-up	12/355	0,006050478	0,043125395	0	0	2,323217656	11,86610847	EEF1B2;RPS17;RPL32;RPS19;RPS8;RPS6;RPL35A;RPS20;RPSA;RPS3A;RPL10A;RPL39
Betamethasone-79_mg/kg_in_Corn_Oil-Rat-Bone_marrow-1d-up	11/311	0,006079781	0,043295079	0	0	2,430912366	12,40442691	C1QA;RPS17;RPL32;RPS19;RPS8;RPS5;RPS6;RPL13A;RPL35A;RPS3A;SEPP1
Cisplatin-0.5_mg/kg_in_Saline-Rat-Kidney-14d-up	11/311	0,006079781	0,043255969	0	0	2,430912366	12,40442691	CXCL11;RPL32;RPS19;RPS8;RPS6;RPS20;RPSA;RPS3A;RPL10A;RPL39;TPT1
Ethisterone-1500_mg/kg_in_CMC-Rat-Liver-1d-up	11/311	0,006079781	0,04321693	0	0	2,430912366	12,40442691	C1QA;RPS17;RPL32;RPS6;ARL6IP5;RPL35A;RPS20;RPSA;RPS3A;RPL10A;RPL39
Flurbiprofen-10_mg/kg_in_CMC-Rat-Kidney-3d-up	11/311	0,006079781	0,04317796	0	0	2,430912366	12,40442691	EEF1B2;C1QA;RPL32;RPS5;RPS6;RPS20;RPS3A;RPL10A;RPL39;TPT1;PDRG1
Leucovorin-1500_mg/kg_in_CMC-Rat-Bone_marrow-1d-dn	11/311	0,006079781	0,043139061	0	0	2,430912366	12,40442691	EEF1B2;C1QA;RPS17;RPL32;RPS19;ARL2BP;RPS8;RPS6;RPL35A;RPS3A;RPL39
Mitomycin_C-0.5_mg/kg_in_Saline-Rat-Bone_marrow-0.25d-dn	11/311	0,006079781	0,043100232	0	0	2,430912366	12,40442691	EEF1B2;RPS19;RPS8;RPS6;NKG7;RPS20;RPSA;RPS3A;RPL10A;RPL39;TPT1
Nadolol-1500_mg/kg_in_CMC-Rat-Heart-3d-up	11/311	0,006079781	0,043061473	0	0	2,430912366	12,40442691	EEF1B2;RPS17;RPL32;RPS8;RPS5;RPS6;RPSA;RPS3A;RPL10A;RPL39;PDRG1
Neomycin-877_mg/kg_in_Corn_Oil-Rat-Kidney-3d-up	11/311	0,006079781	0,043022783	0	0	2,430912366	12,40442691	RPS17;RPL32;ARL2BP;RPS8;RPS6;ARL6IP5;RPL35A;RPS20;RPS3A;RPL39;GOLGA7
Stavudine-1400_mg/kg_in_Water-Rat-Heart-3d-up	11/311	0,006079781	0,042984163	0	0	2,430912366	12,40442691	RPS17;RPS8;RPS6;ARL6IP5;RPSA;RAPGEF5;RPL10A;SEPP1;RPL39;GOLGA7;TPT1
Streptomycin-120_mg/kg_in_Saline-Rat-Kidney-1d-dn	11/311	0,006079781	0,042945612	0	0	2,430912366	12,40442691	EEF1B2;RPS17;RPL32;RPS8;RPS6;RPL35A;RPS20;RPS3A;SEPP1;RPL39;TPT1
Triacetin-1200_mg/kg_in_CMC-Rat-Liver-3d-up	11/311	0,006079781	0,042907131	0	0	2,430912366	12,40442691	EEF1B2;C1QA;RPS17;RPL32;RPS19;RPS8;RPS6;RPS20;RPSA;RPS3A;RPL39
Finasteride-25_mg/kg_in_Corn_Oil-Rat-Liver-5d-dn	10/268	0,006080987	0,04287722	0	0	2,564497102	13,08557277	EEF1B2;C1QA;RPS17;RPL32;RPS8;RPS6;ARL6IP5;RPL35A;RPS3A;SEPP1
Indomethacin-9.6_mg/kg_in_Water-Rat-Kidney-3d-up	12/356	0,006182597	0,04355468	0	0	2,316691764	11,7827335	RPS17;RPL32;RPS19;RPS8;RPS5;RPS6;RPL35A;RPS20;RPS3A;RPL10A;RPL39;TPT1
Valproic_Acid-850_mg/kg_in_Water-Rat-Kidney-3d-up	12/356	0,006182597	0,043515757	0	0	2,316691764	11,7827335	EEF1B2;RPS17;RPL32;RPS5;RPL13A;RPL35A;RPS20;RPSA;SEPP1;RPL39;TOMM20;TPT1
Aspirin-35_mg/kg_in_Corn_Oil-Rat-Liver-3d-up	11/312	0,006222933	0,043760553	0	0	2,42312098	12,30827685	RPS17;RPL32;RPS19;RPS8;RPS5;RPS6;RPS20;RPSA;RPS3A;SEPP1;GOLGA7
Bisphenol_A-610_mg/kg_in_Corn_Oil-Rat-Bone_marrow-3d-up	11/312	0,006222933	0,043721516	0	0	2,42312098	12,30827685	EEF1B2;C1QA;RPL32;ARL2BP;RPS8;RPS5;FUNDC1;RPS6;RPL35A;RPS3A;TPT1
Camptothecin-5_mg/kg_in_Saline-Rat-Kidney-5d-dn	11/312	0,006222933	0,043682548	0	0	2,42312098	12,30827685	EEF1B2;RPS19;ARL2BP;RPS8;RPS6;ARL6IP5;RPS20;RPS3A;RAPGEF5;RPL10A;TPT1
Captopril-1750_mg/kg_in_Water-Rat-Kidney-3d-up	11/312	0,006222933	0,04364365	0	0	2,42312098	12,30827685	RPS17;RPL32;ARL2BP;RPS5;RPL35A;RPS20;RPSA;RPS3A;RPL10A;RPL39;TPT1
Flubendazole-1200_mg/kg_in_Corn_Oil-Rat-Liver-5d-up	11/312	0,006222933	0,043604821	0	0	2,42312098	12,30827685	C1QA;RPS17;RPL32;RPS19;RPS8;RPS20;RPSA;RPS3A;RPL10A;RPL39;TPT1
Hydrocortisone-56_mg/kg_in_Corn_Oil-Rat-Bone_marrow-1d-dn	11/312	0,006222933	0,043566061	0	0	2,42312098	12,30827685	RPL32;RPS8;FEM1B;RPS6;RPL35A;RPS20;RPSA;RPS3A;RPL10A;RPL39;TPT1
Hydrocortisone-56_mg/kg_in_Corn_Oil-Rat-Kidney-3d-up	11/312	0,006222933	0,04352737	0	0	2,42312098	12,30827685	EEF1B2;RPS17;CXCL11;RPL32;RPS19;RPS6;ARL6IP5;RPL35A;RPS20;RPSA;SEPP1
Nimesulide-30_mg/kg_in_Corn_Oil-Rat-Kidney-1d-dn	11/312	0,006222933	0,043488748	0	0	2,42312098	12,30827685	EEF1B2;RPS17;RPL32;RPS19;RPS6;RPL35A;RPS20;RPS3A;RPL10A;SEPP1;RPL39
Famciclovir-112_mg/kg_in_Saline-Rat-Spleen-1d-dn	10/269	0,006237072	0,043548916	0	0	2,554963656	12,97217508	EEF1B2;C1QA;RPS17;ARL2BP;RPS8;RPS5;FUNDC1;RPL13A;RPL35A;TPT1
Pravastatin-1200_mg/kg_in_Corn_Oil-Rat-Liver-1d-dn	12/357	0,006316992	0,044067874	0	0	2,310202431	11,70004823	EEF1B2;RPS17;RPL32;CLP1;RPS8;FEM1B;RPS20;RPSA;RPS3A;RPL10A;RPL39;TPT1
Acetazolamide-250_mg/kg_in_Corn_Oil-Rat-Liver-0.25d-up	11/313	0,006368721	0,044389423	0	0	2,415379379	12,21301943	EEF1B2;RPL32;RPS19;RPS8;FEM1B;RPS6;RPS20;RPSA;RPS3A;RPL10A;SEPP1
Acetazolamide-250_mg/kg_in_Corn_Oil-Rat-Liver-3d-up	11/313	0,006368721	0,044350175	0	0	2,415379379	12,21301943	EEF1B2;RPS17;RPL32;RPS19;RPS8;RPS5;RPS20;RPSA;RPS3A;RPL10A;RPL39
Clofibric_Acid-448_mg/kg_in_Corn_Oil-Rat-Liver-5d-up	11/313	0,006368721	0,044310997	0	0	2,415379379	12,21301943	RPS17;RPL32;RPS5;RPS6;RPL35A;RPS20;RPSA;RPS3A;RPL10A;RPL39;TPT1
Doxorubicin-4_mg/kg_in_Saline-Rat-Kidney-14d-up	11/313	0,006368721	0,044271887	0	0	2,415379379	12,21301943	EEF1B2;CXCL11;RPL32;RPS19;RPS8;RPS6;RPS20;RPSA;RPL10A;RPL39;GOLGA7
Galantamine-110_mg/kg_in_CMC-Rat-Heart-3d-dn	11/313	0,006368721	0,044232847	0	0	2,415379379	12,21301943	EEF1B2;RPS19;ARL2BP;RPS8;COL5A3;FUNDC1;ARL6IP5;RPS20;RPSA;RPL39;TPT1
Ifosfamide-17_mg/kg_in_Saline-Rat-Spleen-0.25d-up	11/313	0,006368721	0,044193875	0	0	2,415379379	12,21301943	EEF1B2;C1QA;RPS17;RPL32;RPS8;RPS5;RPL13A;RPL35A;RPSA;RPL10A;SEPP1
Clotrimazole-52_mg/kg_in_Corn_Oil-Rat-Liver-5d-up	10/270	0,00639626	0,044345899	0	0	2,545500827	12,85997679	RPS17;RPL32;RPS19;RPS8;RPS5;RPL35A;RPS20;RPSA;RPS3A;RPL39
Diclofenac-3.5_mg/kg_in_Corn_Oil-Rat-Liver-3d-dn	10/270	0,00639626	0,044306897	0	0	2,545500827	12,85997679	EEF1B2;C1QA;RPS17;RPL32;RPS8;RPS6;RPS20;RPSA;RPS3A;TPT1
Esmolol-27_mg/kg_in_Saline-Rat-Heart-3d-dn	10/270	0,00639626	0,044267963	0	0	2,545500827	12,85997679	EEF1B2;RPS17;RPL32;RPS19;RPS8;RPS6;ARL6IP5;RPS3A;RPL10A;RPL39
Etoposide-100_mg/kg_in_Corn_Oil-Rat-Liver-1d-dn	10/270	0,00639626	0,044229097	0	0	2,545500827	12,85997679	EEF1B2;RPS17;RPL32;RPS8;RPS6;RPL35A;RPS3A;RPL10A;RPL39;TPT1
Etoposide-188_mg/kg_in_Corn_Oil-Rat-Heart-3d-dn	10/270	0,00639626	0,044190299	0	0	2,545500827	12,85997679	EEF1B2;RPS17;RPL32;RPS19;RPS8;RPS6;RPS20;RPS3A;RPL39;TPT1
Tacrolimus-50_uM_in_DMSO-Rat-Primary_rat_hepatocytes-1d-up	10/270	0,00639626	0,04415157	0	0	2,545500827	12,85997679	EEF1B2;RPS17;ARL2BP;RPS8;RPS5;RPS20;RPSA;RPS3A;RPL10A;PDRG1
Benzocaine-1100_mg/kg_in_Corn_Oil-Rat-Liver-3d-up	11/314	0,006517179	0,04494685	0	0	2,407687088	12,11864428	RPL32;RPS19;RPS8;RPS5;RPS6;RPL35A;RPS20;RPSA;RPL10A;SEPP1;RPL39
Carmustine-4_mg/kg_in_Corn_Oil-Rat-Liver-0.25d-dn	11/314	0,006517179	0,044907526	0	0	2,407687088	12,11864428	EEF1B2;RPS17;RPL32;RPS19;CLP1;RPS8;RPS20;RPSA;RPS3A;RPL39;TPT1
Cetylpyridinium_Bromide-16.5_mg/kg_in_Water-Rat-Liver-1d-up	11/314	0,006517179	0,044868272	0	0	2,407687088	12,11864428	EEF1B2;RPS17;RPL32;RPS19;RPS6;RPL35A;RPSA;RPS3A;RPL10A;RPL39;TPT1
Isotretinoin-125_mg/kg_in_Corn_Oil-Rat-Liver-5d-up	11/314	0,006517179	0,044829085	0	0	2,407687088	12,11864428	EEF1B2;C1QA;RPS17;RPL32;RPS19;CLP1;RPS8;RPL35A;RPS20;RPS3A;TPT1
NN-Dimethylformamide-65000_uM_in_DMSO-Rat-Primary_rat_hepatocytes-1d-up	11/314	0,006517179	0,044789967	0	0	2,407687088	12,11864428	RPS17;CXCL11;RPL32;RPS8;RPS5;RPS6;RPL35A;RPSA;RPL10A;SEPP1;PDRG1
Valacyclovir-2000_mg/kg_in_CMC-Rat-Kidney-1d-up	11/314	0,006517179	0,044750918	0	0	2,407687088	12,11864428	C1QA;RPS17;CXCL11;RPL32;RPS19;RPS8;RPS6;ARL6IP5;RPS20;RPL10A;RPL39
Zomepirac-2.8_mg/kg_in_Saline-Rat-Liver-5d-up	11/314	0,006517179	0,044711936	0	0	2,407687088	12,11864428	EEF1B2;C1QA;RPS17;RPL32;ARL6IP5;RPL35A;RPSA;RPS3A;SEPP1;RPL39;TPT1
Carboplatin-6_mg/kg_in_Saline-Rat-Bone_marrow-0.25d-up	10/271	0,006558591	0,04495689	0	0	2,536107835	12,74896189	RPL32;RPS19;RPS8;RPS5;FUNDC1;RPL13A;RPL35A;RPS20;RPSA;TPT1
Fenofibrate-215_mg/kg_in_Corn_Oil-Rat-Kidney-1d-up	12/359	0,006592719	0,045151526	0	0	2,297332223	11,53671864	RPS17;RPS19;RPS8;RPS5;RPS6;RPS20;RPSA;RPS3A;RAPGEF5;RPL39;GOLGA7;TPT1
Bisphenol_A-610_mg/kg_in_Corn_Oil-Rat-Bone_marrow-5d-dn	11/315	0,00666834	0,04562975	0	0	2,400043637	12,02514119	EEF1B2;RPS17;RPL32;RPS8;RPS5;ARL6IP5;RPL13A;RPL35A;RPSA;RPS3A;TPT1
Cobalt(II)_Acetylacetonate-100_mg/kg_in_Corn_Oil-Rat-Heart-5d-dn	11/315	0,00666834	0,045590141	0	0	2,400043637	12,02514119	EEF1B2;RPS17;RPL32;RPS19;RPS8;RPS6;RPS20;RPSA;RPS3A;RPL10A;RPL39
Lovastatin-1500_mg/kg_in_Corn_Oil-Rat-Liver-3d-up	11/315	0,00666834	0,0455506	0	0	2,400043637	12,02514119	EEF1B2;RPL32;RPS8;RPS5;RPS6;RPL35A;RPS20;RPSA;RPL10A;RPL39;TPT1
Netilmicin-40_mg/kg_in_Saline-Rat-Kidney-5d-dn	9/230	0,00672222	0,045878859	0	0	2,689376961	13,4531695	RPS17;RPS19;ARL2BP;RPS8;FUNDC1;RPSA;RPS3A;RPL39;SEPP1
2378-Tetrachlorodibenzo-P-Dioxin-0.02_mg/kg_in_CMC-Rat-Liver-1d-dn	10/272	0,006724108	0,045852012	0	0	2,526783909	12,63911464	EEF1B2;RPS17;RPL32;RPS19;CLP1;RPS8;RPS6;RPS20;RPL39;TPT1
Nevirapine-29_mg/kg_in_Saline-Rat-Liver-1d-dn	10/272	0,006724108	0,045812348	0	0	2,526783909	12,63911464	EEF1B2;RPL32;RPS19;RPS8;RPS5;RPS6;RPSA;RPL10A;SEPP1;TPT1
Nizatidine-1653_mg/kg_in_Corn_Oil-Rat-Heart-5d-dn	10/272	0,006724108	0,045772752	0	0	2,526783909	12,63911464	EEF1B2;RPS17;RPL32;ARL2BP;RPS8;RPS6;RPL35A;RPSA;RPS3A;RPL39
Aflatoxin_B1-0.3_mg/kg_in_CMC-Rat-Liver-1d-up	11/316	0,006822235	0,046400627	0	0	2,392448562	11,9325001	EEF1B2;RPS17;RPL32;RPS19;RPS8;RPS20;RPSA;RPS3A;SEPP1;RPL39;TPT1
Diethylstilbestrol-280_mg/kg_in_Corn_Oil-Rat-Bone_marrow-3d-up	11/316	0,006822235	0,046360592	0	0	2,392448562	11,9325001	EEF1B2;C1QA;RPS17;RPL32;RPS5;NKG7;RPL35A;RPS3A;SEPP1;RPL39;TPT1
Ifosfamide-17_mg/kg_in_Saline-Rat-Liver-0.25d-dn	11/316	0,006822235	0,046320626	0	0	2,392448562	11,9325001	EEF1B2;RPS17;RPL32;RPS19;RPS6;RPL35A;RPS20;RPSA;RPL10A;RPL39;TPT1
Irinotecan-5_mg/kg_in_Saline-Rat-Bone_marrow-3d-dn	11/316	0,006822235	0,046280729	0	0	2,392448562	11,9325001	CLP1;RPS8;FEM1B;FUNDC1;RPS6;NKG7;RPS20;RPSA;RPS3A;GOLGA7;TPT1
Nimesulide-162_mg/kg_in_Corn_Oil-Rat-Kidney-1d-up	11/316	0,006822235	0,0462409	0	0	2,392448562	11,9325001	RPS17;RPS19;RPS8;RPS6;RPS20;RPSA;RPS3A;RAPGEF5;RPL10A;GOLGA7;TPT1
Paroxetine-104_mg/kg_in_Water-Rat-Brain-5d-up	11/316	0,006822235	0,04620114	0	0	2,392448562	11,9325001	EEF1B2;RPS19;CLP1;RPS6;RPS20;RPSA;RPS3A;RAPGEF5;RPL10A;RPL39;TPT1
Quetiapine-500_mg/kg_in_CMC-Rat-Liver-1d-dn	11/316	0,006822235	0,046161449	0	0	2,392448562	11,9325001	EEF1B2;C1QA;RPS17;RPL32;RPS19;RPS8;RPS6;RPL35A;RPS20;RPL10A;SEPP1
1-Naphthyl_Isothiocyanate-60_mg/kg_in_Corn_Oil-Rat-Liver-1d-up	10/273	0,00689285	0,046599218	0	0	2,517528291	12,53041952	EEF1B2;RPS17;RPS19;ARL2BP;RPS6;RPS20;RPSA;RPS3A;RPL10A;RPL39
Busulfan-9_mg/kg_in_Corn_Oil-Rat-Spleen-5d-up	10/273	0,00689285	0,046559253	0	0	2,517528291	12,53041952	EEF1B2;CXCL11;RPS19;CLP1;RPS8;FUNDC1;RPS20;RPSA;GOLGA7;SEPP1
Mebendazole-714_mg/kg_in_Corn_Oil-Rat-Liver-3d-up	10/273	0,00689285	0,046519357	0	0	2,517528291	12,53041952	EEF1B2;RPL32;RPS19;RPS8;RPS5;RPL35A;RPS20;RPSA;RPL39;SEPP1

Dichlorvos-17_mg/kg_in_Water-Rat-Heart-5d-dn	11/317	0,0069789	0,047059774	0	0	2,384901406	11,84071108	RPS17;RPL32;RPS19;RPS6;RPS20;RPSA;RPS3A;RPL10A;RPL39;TPT1;PDRG1
Ethanol-3000_mg/kg_in_Saline-Rat-Bone_marrow-0.25d-dn	11/317	0,0069789	0,047019517	0	0	2,384901406	11,84071108	EEF1B2;C1QA;RPS17;RPL32;RPS8;RPL35A;RPSA;RPS3A;RPL10A;RPL39;TPT1
Lidocaine-71_mg/kg_in_Water-Rat-Heart-1d-up	11/317	0,0069789	0,04697933	0	0	2,384901406	11,84071108	C1QA;RPS17;RPL32;RPS19;ARL2BP;RPS8;RPS6;RPSA;RPS3A;RAPGEF5;TPT1
Mitomycin_C-1.7_mg/kg_in_Saline-Rat-Bone_marrow-5d-dn	11/317	0,0069789	0,046939211	0	0	2,384901406	11,84071108	EEF1B2;RPS17;RPL32;FEM1B;RPS5;RPL35A;RPSA;RPL10A;RPL39;TPT1;DUSP14
Nitrofurantoin-76_mg/kg_in_CMC-Rat-Liver-3d-up	11/317	0,0069789	0,04689916	0	0	2,384901406	11,84071108	RPS17;RPS19;RPS8;RPS6;RPS20;RPSA;RPS3A;RAPGEF5;RPL10A;SEPP1;RPL39
Allyl_Alcohol-25_mg/kg_in_Saline-Rat-Liver-0.25d-up	10/274	0,00706486	0,047436347	0	0	2,508340231	12,42286132	EEF1B2;RPS17;RPL32;RPS8;RPS5;RPS6;RPSA;RPS3A;RPL39;SEPP1
Atropine-2.3_mg/kg_in_Water-Rat-Liver-1d-dn	10/274	0,00706486	0,047395941	0	0	2,508340231	12,42286132	EEF1B2;RPS17;RPL32;RPS19;RPS8;RPS6;RPS20;RPSA;RPS3A;SEPP1
Bromhexine-1200_mg/kg_in_CMC-Rat-Liver-1d-up	11/318	0,007138366	0,047848318	0	0	2,377401716	11,74976436	C1QA;RPS17;RPL32;RPS19;RPS8;RPS5;RPS6;RPSA;RPS3A;TPT1;PDRG1
Chlorambucil-0.6_mg/kg_in_Corn_Oil-Rat-Bone_marrow-3d-up	11/318	0,007138366	0,04780763	0	0	2,377401716	11,74976436	EEF1B2;C1QA;RPL32;RPS8;RPS5;RPL35A;RPS20;RPL10A;SEPP1;RPL39;TPT1
Cisplatin-1.17_mg/kg_in_Saline-Rat-Kidney-1d-up	11/318	0,007138366	0,047767012	0	0	2,377401716	11,74976436	EEF1B2;RPS17;RPS19;RPS8;RPS6;RPL13A;RPL35A;RPS20;RPSA;RPS3A;RPL39
Deferiprone-500_mg/kg_in_Water-Rat-Liver-5d-up	11/318	0,007138366	0,047726463	0	0	2,377401716	11,74976436	EEF1B2;RPS17;RPL32;RPS19;RPS5;RPS6;RPL35A;RPSA;RPS3A;RAPGEF5;RPL10A
Gadopentetate_Dimeglumine-125_mg/kg_in_Saline-Rat-Kidney-1d-up	11/318	0,007138366	0,047685982	0	0	2,377401716	11,74976436	EEF1B2;RPS17;RPL32;RPS19;RPS8;RPS6;ARL6IP5;RPL35A;RPS20;RPSA;RPS3A
Isotretinoin-500_uM_in_DMSO-Rat-Primary_rat_hepatocytes-0.67d-up	11/318	0,007138366	0,04764557	0	0	2,377401716	11,74976436	EEF1B2;RPS19;RPS8;RPS6;ARL6IP5;RPSA;RPL10A;SEPP1;RPL39;GOLGA7;TPT1
NN-Dimethylformamide-1400_mg/kg_in_Saline-Rat-Spleen-1d-dn	11/318	0,007138366	0,047605227	0	0	2,377401716	11,74976436	EEF1B2;RPS17;RPL32;RPS8;RPS5;ARL6IP5;RPL35A;RPS20;RPSA;RPS3A;TPT1
Nateglinide-625_mg/kg_in_CMC-Rat-Kidney-1d-dn	11/318	0,007138366	0,047564952	0	0	2,377401716	11,74976436	RPS19;ARL2BP;RPS8;RPS6;ARL6IP5;RPS20;RPSA;RPL10A;SEPP1;RPL39;PDRG1
Perhexiline-12_uM_in_DMSO-Rat-Primary_rat_hepatocytes-1d-dn	11/318	0,007138366	0,047524745	0	0	2,377401716	11,74976436	RPS17;CXCL11;RPL32;RPS19;ARL2BP;RPS8;RPS5;ARL6IP5;RPL35A;SEPP1;TPT1
Sparfloxacin-29_mg/kg_in_Corn_Oil-Rat-Liver-1d-up	11/318	0,007138366	0,047484606	0	0	2,377401716	11,74976436	EEF1B2;RPL32;RPS19;RPS8;FEM1B;RPS6;RPS20;RPS3A;SEPP1;GOLGA7;TPT1
Valdecoxib-404_mg/kg_in_CMC-Rat-Kidney-5d-up	11/318	0,007138366	0,047444534	0	0	2,377401716	11,74976436	C1QA;RPL32;RPS5;RPS6;ARL6IP5;RPL13A;RPL35A;RPS20;RPSA;RPL39;TOMM20
Acrolein-20_mg/kg_in_Water-Rat-Kidney-1d-up	12/363	0,007172679	0,047632394	0	0	2,272017267	11,21803087	EEF1B2;RPS19;ARL2BP;CLP1;RPS8;RPS6;RPS20;RPS3A;SEPP1;RPL39;TOMM20;TPT1
Ramipril-1500_mg/kg_in_CMC-Rat-Kidney-3d-dn	13/409	0,007195092	0,047740983	0	0	2,18452516	10,77922508	RPL32;RPS8;RPS6;RPS3A;RPL10A;SEPP1;EEF1B2;RPS17;RPS19;CLP1;RPS20;RPL39;TPT1
Venlafaxine-320_mg/kg_in_Water-Rat-Kidney-1d-dn	8/192	0,007229517	0,04792902	0	0	2,863688431	14,11678997	RPS17;RPL32;RPS19;RPS6;RPSA;RPS20;RPL10A;RPL39
Beta-Estradiol-150_mg/kg_in_Corn_Oil-Rat-Kidney-5d-up	10/275	0,007240177	0,047959325	0	0	2,499218994	12,31642504	EEF1B2;RPL32;RPS19;ARL2BP;RPS8;RPS6;RPS20;RPSA;RPS3A;RPL10A
Carbon_Tetrachloride-400_mg/kg_in_Corn_Oil-Rat-Kidney-1d-dn	10/275	0,007240177	0,047919023	0	0	2,499218994	12,31642504	EEF1B2;RPS17;RPL32;ARL2BP;RPS8;ARL6IP5;RPL35A;RPSA;RPS3A;RPL39
Cephalexin-2500_mg/kg_in_CMC-Rat-Kidney-3d-dn	10/275	0,007240177	0,047878789	0	0	2,499218994	12,31642504	EEF1B2;RPL32;RPS19;ARL2BP;CLP1;RPS8;RPS6;RPL35A;RPS20;RPL10A
Lamotrigine-26_mg/kg_in_CMC-Rat-Kidney-5d-dn	10/275	0,007240177	0,047838622	0	0	2,499218994	12,31642504	RPL32;RPS19;ARL2BP;RPS8;RPS6;RPS20;RPSA;RPL10A;SEPP1;TPT1
Norethindrone-375_mg/kg_in_Corn_Oil-Rat-Liver-3d-up	10/275	0,007240177	0,047798522	0	0	2,499218994	12,31642504	EEF1B2;RPS17;RPL32;RPS19;RPS8;RPS5;RPS6;RPS20;RPSA;RPL10A
Piracetam-2500_mg/kg_in_CMC-Rat-Brain-5d-dn	10/275	0,007240177	0,04775849	0	0	2,499218994	12,31642504	EEF1B2;RPS17;RPL32;RPS19;ARL2BP;RPS8;ARL6IP5;RPS20;RPS3A;GOLGA7
Ascorbic_Acid-2000_mg/kg_in_Water-Rat-Kidney-3d-dn	9/233	0,007292325	0,048062219	0	0	2,65474979	13,06384544	EEF1B2;RPL32;RPS19;CLP1;RPS8;ARL6IP5;RPL35A;RPL10A;RPL39
Neomycin-56_mg/kg_in_Corn_Oil-Rat-Kidney-3d-dn	9/233	0,007292325	0,048022034	0	0	2,65474979	13,06384544	EEF1B2;RPL32;RPS19;ARL2BP;CLP1;RPS3A;RPL10A;SEPP1;TPT1
3-Methylcholanthrene-12.5_mg/kg_in_CMC-Rat-Liver-91d-dn	11/319	0,007300668	0,048036809	0	0	2,369949046	11,65965028	EEF1B2;RPS17;RPL32;RPS19;RPS8;RPS6;RPS20;RPSA;RPS3A;RPL10A;TPT1
Cytarabine-23_mg/kg_in_Saline-Rat-Bone_marrow-1d-dn	11/319	0,007300668	0,047996712	0	0	2,369949046	11,65965028	RPS17;RPL32;RPS8;FEM1B;RPS5;RPL13A;RPL35A;RPSA;RPS3A;RPL10A;TPT1
Fenofibrate-215_mg/kg_in_Corn_Oil-Rat-Heart-3d-up	11/319	0,007300668	0,047956681	0	0	2,369949046	11,65965028	RPS19;ARL2BP;RPS6;ARL6IP5;RPS20;RPSA;RAPGEF5;RPL10A;SEPP1;RPL39;TPT1
Finasteride-800_mg/kg_in_Corn_Oil-Rat-Liver-0.25d-up	12/364	0,007323767	0,048068326	0	0	2,265775462	11,13998057	EEF1B2;C1QA;RPS17;RPL32;RPS19;RPS8;RPS5;RPL35A;RPS20;RPSA;RPL10A;RPL39
Aceclofenac-9_mg/kg_in_Corn_Oil-Rat-Kidney-1d-dn	10/276	0,007418845	0,048651809	0	0	2,490163853	12,21109596	EEF1B2;RPS17;RPL32;ARL2BP;RPS8;RPS6;RPL13A;RPL35A;RPS3A;TOMM20
Benoxaprofen-89_mg/kg_in_CMC-Rat-Liver-1d-up	10/276	0,007418845	0,048611333	0	0	2,490163853	12,21109596	EEF1B2;RPS17;RPL32;RPS8;RPS6;RPL35A;RPS20;RPS3A;RPL10A;SEPP1
Hydrazine-45_mg/kg_in_Water-Rat-Kidney-3d-dn	10/276	0,007418845	0,048570924	0	0	2,490163853	12,21109596	RPS17;RPL32;RPS19;FEM1B;RPS6;ARL6IP5;RPL35A;RPSA;RPS3A;RPL10A
Ofloxacin-3000_mg/kg_in_Water-Rat-Kidney-3d-dn	10/276	0,007418845	0,048530583	0	0	2,490163853	12,21109596	RPS17;RPL32;RPS19;RPS6;RPL35A;RPSA;RPS3A;RPL10A;RPL39;TPT1
Dexamethasone-300_mg/kg_in_Corn_Oil-Rat-Kidney-3d-up	11/320	0,007465838	0,048797463	0	0	2,362542955	11,57035935	C1QA;RPS19;RPS8;RPS6;ARL6IP5;RPS20;RPSA;RPL10A;SEPP1;GOLGA7;TPT1
Dobutamine-43_mg/kg_in_Saline-Rat-Heart-1d-up	11/320	0,007465838	0,048757	0	0	2,362542955	11,57035935	EEF1B2;RPS17;RPL32;RPS19;ARL6IP5;RPL35A;RPS20;RPSA;RPL10A;RPL39;TPT1
Butenafine-150_mg/kg_in_Corn_Oil-Rat-Liver-3d-up	10/277	0,007600904	0,049597943	0	0	2,481174092	12,10685955	EEF1B2;RPS17;RPL32;RPS19;CLP1;RPS8;RPS6;RPS20;RPL10A;SEPP1
Esmolol-27_mg/kg_in_Saline-Rat-Heart-1d-dn	10/277	0,007600904	0,049556885	0	0	2,481174092	12,10685955	EEF1B2;RPL32;ARL2BP;RPS8;RPS5;FUNDC1;RPS20;RPS3A;SEPP1;TPT1
Fluphenazine-2.5_mg/kg_in_Corn_Oil-Rat-Liver-5d-dn	10/277	0,007600904	0,049515895	0	0	2,481174092	12,10685955	C1QA;RPS17;RPL32;RPS19;RPS8;RPS6;RPL35A;RPSA;RPS3A;RPL10A
Acrolein-20_mg/kg_in_Water-Rat-Heart-3d-dn	11/321	0,007633911	0,04968982	0	0	2,355183008	11,4818822	EEF1B2;RPS17;CXCL11;RPL32;RPS8;COL5A3;RPS6;RPSA;RPS3A;RPL39;TPT1
Aldosterone-0.065_mg/kg_in_Saline-Rat-Heart-3d-dn	11/321	0,007633911	0,049648788	0	0	2,355183008	11,4818822	EEF1B2;RPS17;RPL32;RPS19;RPS5;RPS6;RPS20;RPS3A;RPL10A;SEPP1;RPL39
Artemisinin-2000_mg/kg_in_CMC-Rat-Liver-1d-up	11/321	0,007633911	0,049607824	0	0	2,355183008	11,4818822	RPS17;RPS19;RPS8;RPS5;RPS6;RPL35A;RPSA;RPS3A;RPL10A;RPL39;TPT1
Chlorambucil-0.6_mg/kg_in_Corn_Oil-Rat-Liver-3d-dn	11/321	0,007633911	0,049566927	0	0	2,355183008	11,4818822	C1QA;RPS17;RPL32;RPS19;RPS6;RPSA;RPS3A;RPL10A;RPL39;GOLGA7;TPT1
Oxymetholone-1170_mg/kg_in_CMC-Rat-Liver-1d-up	11/321	0,007633911	0,049526098	0	0	2,355183008	11,4818822	RPS17;RPL32;RPS5;ARL6IP5;RPL35A;RPS20;RPSA;RPS3A;RPL10A;RPL39;TPT1
Oxytetracycline-1500_mg/kg_in_Water-Rat-Liver-3d-up	11/321	0,007633911	0,049485335	0	0	2,355183008	11,4818822	RPS17;RPL32;RPS19;ARL2BP;RPS8;RPS6;ARL6IP5;RPS20;RPSA;RPS3A;RPL10A
Procaine-2500_mg/kg_in_Water-Rat-Heart-1d-dn	11/321	0,007633911	0,04944464	0	0	2,355183008	11,4818822	EEF1B2;RPS17;RPL32;RPS8;RPS5;RPL35A;RPS20;RPS3A;RPL39;TOMM20;TPT1
Rofecoxib-775_mg/kg_in_Corn_Oil-Rat-Kidney-5d-up	11/321	0,007633911	0,049404012	0	0	2,355183008	11,4818822	RPS17;RPS19;ARL2BP;CLP1;RPS8;RPS5;RPS6;RPL35A;RPS20;GOLGA7;TPT1
Valproic_Acid-850_mg/kg_in_Water-Rat-Bone_marrow-3d-up	11/321	0,007633911	0,04936345	0	0	2,355183008	11,4818822	C1QA;RPS17;RPL32;RPS19;RPS8;RPS5;RPS6;NKG7;RPL35A;RPSA;RPL10A
Bisacodyl-1750_mg/kg_in_CMC-Rat-Intestine-1d-dn	10/278	0,007786395	0,050308162	0	0	2,472249005	12,00370158	EEF1B2;C1QA;RPS19;ARL2BP;RPS6;RPS20;RPSA;RPS3A;RPL39;SEPP1
Carvedilol-2000_mg/kg_in_Corn_Oil-Rat-Heart-3d-up	10/278	0,007786395	0,050266926	0	0	2,472249005	12,00370158	RPS17;RPL32;RPS19;RPS6;RPL35A;RPS20;RAPGEF5;RPL10A;RPL39;SEPP1
Chloroquine-21.95_uM_in_DMSO-Rat-Primary_rat_hepatocytes-1d-up	10/278	0,007786395	0,050225758	0	0	2,472249005	12,00370158	EEF1B2;RPS17;CXCL11;RPL32;RPL35A;RPSA;RPL10A;GOLGA7;SEPP1;SLC16A3
Clozapine-95_mg/kg_in_CMC-Rat-Heart-5d-up	10/278	0,007786395	0,050184656	0	0	2,472249005	12,00370158	RPS17;RPL32;ARL2BP;RPS8;FUNDC1;RPS6;RPL35A;RPS3A;RAPGEF5;TOMM20
Doxorubicin-1.5_uM_in_DMSO-Rat-Primary_rat_hepatocytes-1d-up	10/278	0,007786395	0,050143622	0	0	2,472249005	12,00370158	EEF1B2;RPS17;CXCL11;RPL32;FEM1B;RPS5;RPS20;RPSA;SEPP1;TOMM20
Isoeugenol-1560_mg/kg_in_CMC-Rat-Liver-1d-up	10/278	0,007786395	0,050102655	0	0	2,472249005	12,00370158	EEF1B2;RPS19;RPS8;RPS5;RPS6;RPSA;RPS3A;RPL10A;RPL39;TPT1
Atorvastatin-2.5_mg/kg_in_Corn_Oil-Rat-Kidney-3d-dn	11/322	0,00780492	0,050180854	0	0	2,347868775	11,39420959	EEF1B2;RPS17;RPL32;RPS19;RPS8;RPS6;RPSA;RPL10A;SEPP1;RPL39;TPT1
Azathioprine-20_mg/kg_in_Corn_Oil-Rat-Liver-0.25d-up	11/322	0,00780492	0,050139924	0	0	2,347868775	11,39420959	EEF1B2;RPS19;RPS8;RPS6;RPS20;RPSA;RPS3A;RPL10A;SEPP1;RPL39;TPT1
Capsaicin-35_mg/kg_in_CMC-Rat-Liver-3d-dn	11/322	0,00780492	0,05009906	0	0	2,347868775	11,39420959	C1QA;RPL32;RPS19;CLP1;FEM1B;RPS5;RPS6;RPS20;RPSA;RPL10A;TPT1
Ofloxacin-3000_mg/kg_in_Water-Rat-Kidney-5d-dn	9/236	0,007898716	0,050659844	0	0	2,62100297	12,68841966	RPS17;RPL32;RPS19;RPS6;RPS20;RPSA;RPS3A;RPL10A;RPL39
44'-Diethylaminoethoxyhexestrol-20_uM_in_DMSO-Rat-Primary_rat_hepatocytes-1d-up	10/279	0,007975362	0,051109805	0	0	2,463387897	11,90160799	EEF1B2;RPL32;RPS8;RPS5;RPL35A;RPS20;RPSA;RPS3A;RPL10A;PDRG1
Carmustine-16_mg/kg_in_Corn_Oil-Rat-Liver-0.25d-dn	10/279	0,007975362	0,051068252	0	0	2,463387897	11,90160799	EEF1B2;RPS17;RPL32;RPS19;RPS8;RPL35A;RPS20;RPS3A;RPL39;TPT1
Lead(IV)_Acetate-200_mg/kg_in_Saline-Rat-Liver-1d-dn	10/279	0,007975362	0,051026767	0	0	2,463387897	11,90160799	EEF1B2;RPL32;RPS19;RPS8;RPS5;RPS6;RPSA;RPS3A;RPL10A;TPT1
Rabeprazole-1024_mg/kg_in_Water-Rat-Liver-1d-up	10/279	0,007975362	0,050985349	0	0	2,463387897	11,90160799	EEF1B2;RPS17;RPL32;RPS19;RPS6;RPS20;RPSA;RPS3A;RPL10A;RPL39
Sulfaphenazole-1695_mg/kg_in_Water-Rat-Liver-5d-dn	10/279	0,007975362	0,050943998	0	0	2,463387897	11,90160799	EEF1B2;RPS17;RPL32;RPS19;CLP1;RPS8;RPS6;RPS20;RPL39;SEPP1
Vinblastine-0.3_mg/kg_in_Saline-Rat-Bone_marrow-1d-dn	10/279	0,007975362	0,050902715	0	0	2,463387897	11,90160799	EEF1B2;RPS17;RPS8;RPS6;NKG7;RPS20;RPSA;RPS3A;RPL10A;RPL39
Buspirone-196_mg/kg_in_CMC-Rat-Heart-1d-dn	11/323	0,007978898	0,050884049	0	0	2,340599832	11,30733241	RPS17;RPL32;RPS19;RPS8;RPS20;RPSA;RPS3A;RPL10A;SEPP1;RPL39;TPT1
Digoxin-11_mg/kg_in_Corn_Oil-Rat-Liver-1d-dn	11/323	0,007978898	0,050842881	0	0	2,340599832	11,30733241	EEF1B2;C1QA;RPS17;RPL32;RPS8;RPS5;RPS6;RPS3A;RPL10A;GOLGA7;TPT1
Hydroxyurea-59_mg/kg_in_Saline-Rat-Bone_marrow-1d-dn	11/323	0,007978898	0,050801779	0	0	2,340599832	11,30733241	RPS17;RPL32;RPS19;RPS8;RPS5;RPL35A;RPS20;RPSA;RPL10A;SEPP1;TPT1
Proglumide-1125_mg/kg_in_CMC-Rat-Liver-3d-dn	11/323	0,007978898	0,050760743	0	0	2,340599832	11,30733241	EEF1B2;RPL32;RPS19;CLP1;RPS8;FEM1B;RPS5;RPS20;RPSA;RPL10A;SEPP1
Beta-Estradiol-150_mg/kg_in_Corn_Oil-Rat-Liver-0.25d-up	12/369	0,008117183	0,051598815	0	0	2,235073897	10,75913643	EEF1B2;RPS17;RPL32;RPS19;FEM1B;RPS6;RPSA;RPS3A;RPL10A;SEPP1;RPL39;PDRG1
Isotretinoin-13_mg/kg_in_Corn_Oil-Rat-Liver-0.25d-dn	11/324	0,00815588	0,051802993	0	0	2,333375758	11,22124167	EEF1B2;RPS17;RPL32;RPS8;RPS5;RPL35A;RPS20;RPSA;RPS3A;RPL39;TPT1
Lomustine-4.2_mg/kg_in_Corn_Oil-Rat-Liver-0.25d-up	11/324	0,00815588	0,05176125	0	0	2,333375758	11,22124167	EEF1B2;RPS17;RPL32;RPS19;RPS6;RPSA;RPS3A;RPL10A;SEPP1;RPL39;TPT1
Nortriptyline-63_mg/kg_in_Water-Rat-Heart-0.25d-up	11/324	0,00815588	0,051719574	0	0	2,333375758	11,22124167	RPL32;RPS19;ARL2BP;RPS8;RPS6;ARL6IP5;RPL35A;RPS20;RPSA;RPL39;TPT1
Carboplatin-14_mg/kg_in_Saline-Rat-Liver-0.25d-up	10/280	0,008167845	0,051753779	0	0	2,454590083	11,80056499	EEF1B2;C1QA;RPS17;RPS8;RPS6;RPL35A;RPS20;RPS3A;RPL39;TPT1
Chlorpromazine-18_mg/kg_in_Water-Rat-Liver-5d-dn	10/280	0,008167845	0,051712176	0	0	2,454590083	11,80056499	EEF1B2;RPS17;RPL32;RPS8;RPS5;RPL35A;RPS20;RPSA;RPS3A;TPT1
Letrozole-250_mg/kg_in_Corn_Oil-Rat-Liver-3d-up	10/280	0,008167845	0,05167064	0	0	2,454590083	11,80056499	RPL32;RPS19;RPS8;RPS5;RPL35A;RPS20;RPSA;RPL10A;RPL39;SEPP1
Rofecoxib-3_mg/kg_in_Corn_Oil-Rat-Liver-0.25d-dn	10/280	0,008167845	0,051629171	0	0	2,454590083	11,80056499	EEF1B2;RPS17;RPL32;RPS8;RPS5;RPS20;RPSA;RPS3A;RPL39;TPT1
Sulconazole-40_uM_in_DMSO-Rat-Primary_rat_hepatocytes-1d-up	10/280	0,008167845	0,051587768	0	0	2,454590083	11,80056499	EEF1B2;RPS17;RPL32;RPS8;RPS5;RPL35A;RPS20;RPSA;RPS3A;RPL10A
Pentamidine-18_mg/kg_in_Saline-Rat-Bone_marrow-3d-dn	13/416	0,008233176	0,05195873	0	0	2,147766323	10,30838362	C1QA;RPL32;RPS8;FEM1B;RPS5;RPSA;RPL10A;EEF1B2;RPS17;RPS19;ARL6IP5;RPS20;RPL39
Clarithromycin-56_mg/kg_in_Water-Rat-Liver-0.25d-up	12/370	0,008283656	0,052235449	0	0	2,229033157	10,68480545	EEF1B2;RPS17;RPL32;RPS6;RPL35A;RPS20;RPSA;RPS3A;RPL10A;SEPP1;RPL39;TPT1
D-Tubocurarine_Chloride-0.033_mg/kg_in_Saline-Rat-Heart-3d-up	12/370	0,008283656	0,052193661	0	0	2,229033157	10,68480545	RPL32;RPS19;ARL2BP;RPS8;FEM1B;RPS6;RPS20;RPSA;RPS3A;RPL10A;RPL39;TPT1
Gemfibrozil-700_mg/kg_in_Corn_Oil-Rat-Liver-3d-dn	11/325	0,0083359	0,052480852	0	0	2,326196141	11,13592853	EEF1B2;C1QA;RPS17;RPL32;RPS8;RPS6;RPL35A;RPSA;RPL10A;SEPP1;RPL39
Hydralazine-280_mg/kg_in_Water-Rat-Heart-5d-dn	11/325	0,0083359	0,052438935	0	0	2,326196141	11,13592853	C1QA;RPS17;RPL32;RPS19;RPS8;COL5A3;RPS6;RPS20;RPSA;TOMM20;TPT1
Olanzapine-23_mg/kg_in_CMC-Rat-Liver-5d-up	11/325	0,0083359	0,052397084	0	0	2,326196141	11,13592853	EEF1B2;RPS17;RPL32;RPS19;RPS6;RPL35A;RPS20;RPL10A;SEPP1;RPL39;TPT1
Oxcarbazepine-1600_mg/kg_in_CMC-Rat-Heart-5d-dn	11/325	0,0083359	0,0523553	0	0	2,326196141	11,13592853	C1QA;RPS17;RPS19;RPS6;ARL6IP5;RPS3A;RPL10A;SEPP1;GOLGA7;TPT1;PDRG1
Ethinylestradiol-1480_mg/kg_in_Corn_Oil-Rat-Liver-0.25d-up	10/281	0,008363887	0,05248922	0	0	2,445854887	11,700559	EEF1B2;RPS17;RPS19;RPS8;RPS6;RPS20;RPSA;RPS3A;RPL10A;RPL39
Mesna-755_mg/kg_in_Saline-Rat-Kidney-5d-up	10/281	0,008363887	0,052447429	0	0	2,445854887	11,700559	C1QA;RPS17;CXCL11;RPL32;RPS19;RPS5;RPS20;RPSA;RAPGEF5;SEPP1
Progesterone-164_mg/kg_in_Corn_Oil-Rat-Liver-1d-up	10/281	0,008363887	0,052405705	0	0	2,445854887	11,700559	RPL32;RPS19;RPS8;RPS5;RPS6;RPS20;RPSA;RPS3A;RPL10A;TPT1

3-Chloroaniline-200_mg/kg_in_CMC-Rat-Kidney-1d-dn	11/326	0,008518991	0,053335114	0	0	2,31906057	11,05138426	EEF1B2;RPL32;ARL2BP;RPS8;RPS6;RPS20;RPSA;RPL10A;RPL39;TOMM20;TPT1
4-Chloroaniline-75_mg/kg_in_CMC-Rat-Kidney-3d-up	11/326	0,008518991	0,053292751	0	0	2,31906057	11,05138426	EEF1B2;RPS17;CXCL11;RPS19;RPS8;ARL6IP5;RPS20;RPS3A;RAPGEF5;GOLGA7;TPT1
Azathioprine-160_mg/kg_in_Corn_Oil-Rat-Liver-1d-up	11/326	0,008518991	0,053250456	0	0	2,31906057	11,05138426	EEF1B2;RPS17;RPL32;RPS19;RPS8;RPS6;RPS20;RPSA;RPL10A;SEPP1;RPL39
Cortisone-206_mg/kg_in_CMC-Rat-Liver-1d-dn	11/326	0,008518991	0,053208227	0	0	2,31906057	11,05138426	EEF1B2;C1QA;RPL32;RPS19;RPS8;RPS6;ARL6IP5;RPS20;RPS3A;RPL10A;TPT1
Digitonin-75_mg/kg_in_Corn_Oil-Rat-Heart-1d-up	11/326	0,008518991	0,053166065	0	0	2,31906057	11,05138426	EEF1B2;RPS17;RPL32;RPS19;RPS6;RPS20;RPSA;RPS3A;RPL39;TPT1;PDRG1
Dimenhydrinate-165_mg/kg_in_CMC-Rat-Liver-1d-up	11/326	0,008518991	0,05312397	0	0	2,31906057	11,05138426	C1QA;RPS17;RPL32;RPS19;RPS8;RPS6;RPSA;RPS3A;RPL39;TPT1;PDRG1
Itraconazole-1093_mg/kg_in_Corn_Oil-Rat-Liver-5d-dn	10/282	0,008563529	0,053359457	0	0	2,437181643	11,60157665	EEF1B2;RPS17;RPL32;RPS8;ARL6IP5;RPL35A;RPSA;RPS3A;SEPP1;TPT1
Meropenem-1500_mg/kg_in_CMC-Rat-Spleen-5d-up	10/282	0,008563529	0,053317276	0	0	2,437181643	11,60157665	EEF1B2;RPL32;RPS19;RPS5;RPS6;RPS20;RPSA;RPL10A;RPL39;SEPP1
Naproxen-134_mg/kg_in_Corn_Oil-Rat-Kidney-1d-dn	10/282	0,008563529	0,053275161	0	0	2,437181643	11,60157665	EEF1B2;RPS17;RPL32;ARL2BP;RPS8;ARL6IP5;RPL35A;RPSA;RPS3A;RPL39
Sulfaphenazole-1695_mg/kg_in_Water-Rat-Liver-1d-up	10/282	0,008563529	0,053233113	0	0	2,437181643	11,60157665	RPS17;RPL32;RPS8;RPS5;RPL35A;RPSA;RPS3A;RPL10A;RPL39;TPT1
Valproic_Acid-850_mg/kg_in_Water-Rat-Bone_marrow-1d-up	10/282	0,008563529	0,053191131	0	0	2,437181643	11,60157665	EEF1B2;RPS17;RPL32;RPS8;RPS5;NKG7;RPL13A;RPL35A;RPS20;TOMM20
Allyl_Alcohol-16_mg/kg_in_Saline-Rat-Liver-0.25d-up	11/327	0,008705188	0,054028419	0	0	2,311968641	10,96760024	EEF1B2;C1QA;RPL32;RPS19;RPS8;RPS5;RPS20;RPSA;RPS3A;SEPP1;RPL39
Benzethonium_Chloride-5_mg/kg_in_Water-Rat-Heart-3d-up	11/327	0,008705188	0,053985877	0	0	2,311968641	10,96760024	RPS17;RPL32;RPS19;ARL2BP;RPS8;RPS6;RPL35A;RPS20;RPSA;GOLGA7;TPT1
Famotidine-1200_mg/kg_in_CMC-Rat-Heart-5d-up	11/327	0,008705188	0,053943401	0	0	2,311968641	10,96760024	EEF1B2;RPS17;RPL32;ARL6IP5;RPS20;RPSA;RAPGEF5;RPL10A;SEPP1;RPL39;PDRG1
Indomethacin-4.5_mg/kg_in_Water-Rat-Kidney-1d-up	11/327	0,008705188	0,053900993	0	0	2,311968641	10,96760024	EEF1B2;RPS17;RPL32;RPS8;RPS5;RPS6;RPL13A;RPL35A;RPS3A;SEPP1;TOMM20
Streptomycin-120_mg/kg_in_Saline-Rat-Kidney-5d-dn	11/327	0,008705188	0,053858651	0	0	2,311968641	10,96760024	EEF1B2;ARL2BP;RPS8;FUNDC1;RPS6;RPS20;RPS3A;RPL10A;SEPP1;RPL39;TPT1
Acyclovir-330_mg/kg_in_Saline-Rat-Spleen-1d-dn	10/283	0,008766814	0,054197352	0	0	2,428569694	11,50360479	EEF1B2;RPS17;CXCL11;RPS8;RPS5;FUNDC1;RPL13A;RPL35A;RPSA;TPT1
Lomustine-4.2_mg/kg_in_Corn_Oil-Rat-Bone_marrow-0.25d-up	10/283	0,008766814	0,054154845	0	0	2,428569694	11,50360479	EEF1B2;C1QA;RPS17;RPL32;RPL35A;RPS20;RPS3A;RPL10A;RPL39;TPT1
N-Nitrosodiethylamine-34_mg/kg_in_Saline-Rat-Spleen-1d-dn	10/283	0,008766814	0,054112404	0	0	2,428569694	11,50360479	RPS17;RPL32;RPS8;RPS5;FUNDC1;RPS6;RPL13A;RPL35A;RPS3A;TPT1
Tamoxifen-32_mg/kg_in_Corn_Oil-Rat-Liver-1d-up	10/283	0,008766814	0,054070029	0	0	2,428569694	11,50360479	EEF1B2;RPS17;RPL32;RPS19;RPS8;RPS6;ARL6IP5;RPSA;RPS3A;RPL10A
Thioguanine-24_mg/kg_in_Corn_Oil-Rat-Kidney-5d-dn	10/283	0,008766814	0,054027721	0	0	2,428569694	11,50360479	EEF1B2;RPS17;RPL32;RPS19;RPS6;RPSA;RPS3A;RPL10A;RPL39;TPT1
Omeprazole-415_mg/kg_in_Corn_Oil-Rat-Liver-1d-up	11/328	0,008894526	0,054771918	0	0	2,304919956	10,88456798	EEF1B2;RPS17;RPL32;RPS19;RPS8;RPS20;RPSA;RPS3A;SEPP1;RPL39;TPT1
Troglitazone-1200_mg/kg_in_Corn_Oil-Rat-Liver-5d-dn	11/328	0,008894526	0,054729128	0	0	2,304919956	10,88456798	RPS17;RPL32;RPS19;RPS6;RPL35A;RPS20;RPSA;RPS3A;RPL10A;RPL39;TPT1
Famciclovir-1200_mg/kg_in_Saline-Rat-Liver-0.25d-up	10/284	0,008973784	0,05517371	0	0	2,420018392	11,40663047	C1QA;RPS17;RPL32;RPS8;RPS6;RPL35A;RPS20;RPSA;RPS3A;SEPP1
Pralidoxime_Chloride-48_mg/kg_in_Saline-Rat-Heart-3d-dn	10/284	0,008973784	0,055130673	0	0	2,420018392	11,40663047	EEF1B2;RPS17;RPL32;ARL2BP;RPS8;RPS5;FUNDC1;RPSA;RPL39;TOMM20
Sulfadiazine-1170_mg/kg_in_Water-Rat-Kidney-3d-dn	10/284	0,008973784	0,055087703	0	0	2,420018392	11,40663047	RPS17;RPL32;RPS19;RPS6;RPL35A;RPS20;RPSA;RPS3A;RPL10A;RPL39
Zomepirac-2.8_mg/kg_in_Saline-Rat-Liver-0.25d-up	10/284	0,008973784	0,0550448	0	0	2,420018392	11,40663047	C1QA;RPS17;RPL32;RPS8;RPS5;RPL35A;RPSA;RPS3A;TOMM20;TPT1
Trimethadione-535_mg/kg_in_Water-Rat-Kidney-5d-dn	9/241	0,008993894	0,055125223	0	0	2,566625315	12,09190914	RPS17;RPL32;RPS19;RPS6;RPL35A;RPS20;RPSA;RPS3A;RPL39
Bithionol-333_mg/kg_in_Corn_Oil-Rat-Kidney-5d-up	11/329	0,009087037	0,055652803	0	0	2,297914121	10,80227911	EEF1B2;RPS17;RPL32;RPS19;ARL2BP;RPS5;RPS6;ARL6IP5;RPL35A;RPS20;RPS3A
Bithionol-59_mg/kg_in_Corn_Oil-Rat-Liver-0.25d-up	11/329	0,009087037	0,055609561	0	0	2,297914121	10,80227911	EEF1B2;RPS17;RPL32;RPS19;RPS6;RPL35A;RPS20;RPS3A;SEPP1;RPL39;TPT1
Idarubicin-4_mg/kg_in_Saline-Rat-Heart-5d-dn	11/329	0,009087037	0,055566386	0	0	2,297914121	10,80227911	EEF1B2;RPS17;CXCL11;RPL32;RPS8;RPS6;ARL6IP5;RPS20;RPS3A;RPL39;TPT1
Lomustine-8.75_mg/kg_in_Corn_Oil-Rat-Liver-5d-up	11/329	0,009087037	0,055523278	0	0	2,297914121	10,80227911	EEF1B2;RPS17;RPL32;RPS19;RPS20;RPSA;RPS3A;RPL10A;SEPP1;RPL39;TPT1
Lomustine-8.75_mg/kg_in_Corn_Oil-Rat-Spleen-5d-dn	11/329	0,009087037	0,055480237	0	0	2,297914121	10,80227911	EEF1B2;RPS17;RPL32;RPS8;RPS5;RPL13A;RPL35A;RPSA;RPS3A;RPL39;TPT1
Methocarbamol-1125_mg/kg_in_CMC-Rat-Liver-5d-up	11/329	0,009087037	0,055437262	0	0	2,297914121	10,80227911	C1QA;RPS17;RPS19;FEM1B;RPS6;RPS20;RPSA;RPL10A;RPL39;GOLGA7;TPT1
Zaleplon-100_mg/kg_in_Water-Rat-Liver-3d-dn	11/329	0,009087037	0,055394354	0	0	2,297914121	10,80227911	EEF1B2;RPL32;RPS19;RPS8;RPL35A;RPS20;RPS3A;RPL10A;SEPP1;RPL39;TPT1
Zileuton-450_mg/kg_in_Corn_Oil-Rat-Kidney-5d-up	11/329	0,009087037	0,055351512	0	0	2,297914121	10,80227911	RPS17;RPL32;RPS19;RPS8;RPS5;RPS6;RPL35A;RPS20;RPSA;RPS3A;RPL39
Levofloxacin-739_mg/kg_in_Water-Rat-Kidney-1d-dn	8/200	0,009137289	0,055614594	0	0	2,749140893	12,90829304	RPS17;RPL32;RPS19;RPS6;RPSA;RPS3A;RPL10A;RPL39
123-Trichloropropane-108_mg/kg_in_CMC-Rat-Kidney-3d-up	10/285	0,009184481	0,055858667	0	0	2,4115271	11,31064097	EEF1B2;RPS17;RPL32;RPS19;RPS8;RPS3A;RPL10A;RPL39;SEPP1;TPT1
Diethylstilbestrol-280_mg/kg_in_Corn_Oil-Rat-Liver-0.25d-up	10/285	0,009184481	0,055815566	0	0	2,4115271	11,31064097	EEF1B2;RPS17;RPL32;RPS19;RPS8;RPS6;RPSA;RPS3A;RPL10A;RPL39
Sulfathiazole-2629_mg/kg_in_Water-Rat-Kidney-3d-dn	10/285	0,009184481	0,055772532	0	0	2,4115271	11,31064097	RPS17;RPL32;RPS19;RPS6;ARL6IP5;RPL35A;RPS20;RPSA;RPL10A;RPL39
Diclofenac-10_mg/kg_in_Corn_Oil-Rat-Liver-0.25d-up	11/330	0,009282758	0,056325885	0	0	2,290950745	10,72072537	CXADR;RPS19;RPS8;FEM1B;RPS5;RPS6;ARL6IP5;RPL35A;RPS20;RPL10A;TOMM20
Neomycin-56_mg/kg_in_Corn_Oil-Rat-Kidney-5d-up	11/330	0,009282758	0,056282524	0	0	2,290950745	10,72072537	RPS17;CXCL11;RPL32;RPS19;ARL2BP;RPS8;R3HCC1;RPS6;RPS20;RPS3A;TPT1
Ifosfamide-17_mg/kg_in_Saline-Rat-Liver-3d-up	12/376	0,009339396	0,056582371	0	0	2,193463479	10,25118164	C1QA;RPS17;RPL32;RPS19;RPS8;RPS5;RPS6;RPL35A;RPS20;RPSA;RPS3A;TPT1
Amiodarone-147_mg/kg_in_CMC-Rat-Liver-3d-dn	10/286	0,009398948	0,056899396	0	0	2,403095187	11,21562375	RPS17;RPL32;RPS19;RPS8;RPS6;RPL35A;RPS20;RPL39;SEPP1;TPT1
Diethylstilbestrol-2.8_mg/kg_in_Corn_Oil-Rat-Bone_marrow-1d-dn	10/286	0,009398948	0,056855695	0	0	2,403095187	11,21562375	RPL32;RPS19;RPS8;FEM1B;RPS6;ARL6IP5;RPL35A;RPS20;RPSA;RPS3A
Fenoprofen-52_mg/kg_in_Corn_Oil-Rat-Kidney-5d-up	10/286	0,009398948	0,05681206	0	0	2,403095187	11,21562375	RPS17;RPL32;RPS8;RPS5;RPS6;RPL35A;RPSA;RPS3A;RAPGEF5;TPT1
Indomethacin-9.6_mg/kg_in_Water-Rat-Liver-1d-dn	10/286	0,009398948	0,056768493	0	0	2,403095187	11,21562375	EEF1B2;RPS17;RPL32;RPS8;RPS5;RPL35A;RPS20;RPSA;RPS3A;TPT1
Isoniazid-79_mg/kg_in_Water-Rat-Liver-5d-dn	10/286	0,009398948	0,056724992	0	0	2,403095187	11,21562375	EEF1B2;RPL32;RPS19;RPS8;RPS6;RPS3A;RPL10A;RPL39;SEPP1;TPT1
NN-Dimethylformamide-700_mg/kg_in_Saline-Rat-Liver-0.25d-up	10/286	0,009398948	0,056681558	0	0	2,403095187	11,21562375	RPL32;RPS19;RPS8;RPS6;RPSA;RPS3A;RAPGEF5;RPL10A;SEPP1;TPT1
Sulfisoxazole-2500_mg/kg_in_Corn_Oil-Rat-Liver-3d-up	10/286	0,009398948	0,05663819	0	0	2,403095187	11,21562375	EEF1B2;RPL32;RPS19;RPS8;RPS5;RPS6;RPL35A;RPSA;SEPP1;TOMM20
2-Amino-4-Nitrophenol-625_mg/kg_in_CMC-Rat-Heart-3d-dn	11/331	0,009481721	0,057093299	0	0	2,284029443	10,63989862	RPS17;RPL32;RPS19;ARL2BP;COL5A3;RPS6;RPS20;RPSA;RPS3A;RPL39;TPT1
6-Mercaptopurine-25_mg/kg_in_Corn_Oil-Rat-Kidney-5d-dn	11/331	0,009481721	0,057049683	0	0	2,284029443	10,63989862	EEF1B2;RPS17;RPL32;RPS8;RPS6;RPS20;RPSA;RPS3A;SEPP1;RPL39;TPT1
Beta-Estradiol-0.3_mg/kg_in_Corn_Oil-Rat-Liver-3d-up	11/331	0,009481721	0,057006134	0	0	2,284029443	10,63989862	EEF1B2;C1QA;RPL32;RPS19;RPS6;RPL35A;RPS20;RPL10A;SEPP1;RPL39;TPT1
Coumarin-111_mg/kg_in_Corn_Oil-Rat-Bone_marrow-0.25d-dn	11/331	0,009481721	0,056962651	0	0	2,284029443	10,63989862	RPS17;RPL32;RPS19;RPS8;FEM1B;RPS5;RPS6;RPL35A;RPS20;RPSA;RPL10A
Gemcitabine-11_mg/kg_in_Saline-Rat-Bone_marrow-1d-up	11/331	0,009481721	0,056919234	0	0	2,284029443	10,63989862	EEF1B2;C1QA;RPS17;RPL32;ARL2BP;RPS8;RPS6;ARL6IP5;NKG7;RPL35A;RPS3A
Carboplatin-6_mg/kg_in_Saline-Rat-Bone_marrow-3d-up	12/377	0,009525101	0,057136099	0	0	2,187645273	10,18091767	EEF1B2;RPS17;RPL32;RPS8;RPS5;RPS6;RPL13A;RPS20;RPSA;RPS3A;RPL10A;SEPP1
Benzocaine-1100_mg/kg_in_Corn_Oil-Rat-Liver-1d-dn	10/287	0,009617227	0,057644808	0	0	2,394722033	11,12156648	EEF1B2;C1QA;RPS17;RPL32;RPS8;RPS6;RPS3A;RPL10A;RPL39;TPT1
Cisplatin-1.17_mg/kg_in_Saline-Rat-Liver-5d-dn	10/287	0,009617227	0,057600972	0	0	2,394722033	11,12156648	EEF1B2;C1QA;RPS17;RPL32;CLP1;RPS20;RPSA;RPS3A;RPL39;TPT1
Ethacrynic_Acid-125_mg/kg_in_CMC-Rat-Kidney-1d-up	10/287	0,009617227	0,057557202	0	0	2,394722033	11,12156648	EEF1B2;RPL32;RPS19;ARL2BP;RPS8;RPS5;RPS6;RPS20;RPL10A;TPT1
Mitomycin_C-1.7_mg/kg_in_Saline-Rat-Spleen-3d-dn	10/287	0,009617227	0,057513499	0	0	2,394722033	11,12156648	EEF1B2;C1QA;RPS17;RPL32;RPS8;RPS5;RPS6;ARL6IP5;RPSA;RPL10A
1-Naphthyl_Isothiocyanate-60_mg/kg_in_Corn_Oil-Rat-Kidney-1d-up	11/332	0,009683962	0,057868654	0	0	2,277149836	10,55979082	EEF1B2;RPL32;RPS19;RPS5;RPS6;RPS20;RPSA;RPS3A;RAPGEF5;RPL10A;SEPP1
Clozapine-95_mg/kg_in_CMC-Rat-Heart-1d-dn	11/332	0,009683962	0,057824781	0	0	2,277149836	10,55979082	RPS17;RPL32;RPS19;RPS8;COL5A3;RPS6;RPS20;RPSA;RPS3A;RPL10A;RPL39
Hydroxyurea-59_mg/kg_in_Saline-Rat-Spleen-1d-dn	11/332	0,009683962	0,057780975	0	0	2,277149836	10,55979082	EEF1B2;RPS17;RPL32;RPS8;RPS5;RPS6;RPS20;RPSA;RPS3A;RPL10A;TPT1
Ibuprofen-275_mg/kg_in_Corn_Oil-Rat-Liver-3d-dn	11/332	0,009683962	0,057737234	0	0	2,277149836	10,55979082	EEF1B2;RPL32;RPS19;RPS8;RPS6;RPL35A;RPS20;RPSA;RPL10A;RPL39;TPT1
Sulindac-64_mg/kg_in_Corn_Oil-Rat-Kidney-5d-up	11/332	0,009683962	0,05769356	0	0	2,277149836	10,55979082	EEF1B2;RPS17;RPL32;RPS19;RPS8;RPS5;RPS6;RPL35A;RPS20;RPL10A;SEPP1
Troglitazone-100_mg/kg_in_Corn_Oil-Rat-Liver-1d-dn	11/332	0,009683962	0,057649952	0	0	2,277149836	10,55979082	EEF1B2;RPS17;RPL32;RPS8;RPS6;RPS20;RPSA;RPS3A;RPL10A;SEPP1;RPL39
Carboplatin-14_mg/kg_in_Saline-Rat-Bone_marrow-5d-up	9/244	0,009704064	0,057725987	0	0	2,535068447	11,75057594	C1QA;RPS19;ARL2BP;RPS8;RPS6;NKG7;RPS20;RPSA;TPT1
1-Naphthyl_Isothiocyanate-30_mg/kg_in_Corn_Oil-Rat-Kidney-1d-dn	10/288	0,00983936	0,05848664	0	0	2,386407026	11,02845701	EEF1B2;RPS17;RPL32;ARL2BP;CLP1;RPS8;RPS6;RPL35A;RPSA;RPS3A
Altretamine-40_mg/kg_in_Corn_Oil-Rat-Spleen-1d-dn	10/288	0,00983936	0,058442533	0	0	2,386407026	11,02845701	EEF1B2;C1QA;RPL32;RPS19;RPS5;RPS6;RPL13A;RPL35A;RPL39;TOMM20
Amitraz-75_mg/kg_in_CMC-Rat-Liver-3d-dn	10/288	0,00983936	0,058398492	0	0	2,386407026	11,02845701	C1QA;RPS17;RPL32;RPS19;CLP1;RPS8;ARL6IP5;RPL35A;RPS20;SEPP1
Amlodipine-19_mg/kg_in_Corn_Oil-Rat-Liver-1d-dn	10/288	0,00983936	0,058354517	0	0	2,386407026	11,02845701	RPS17;CLP1;RPS8;RPS6;RPS20;RPS3A;RPL10A;RPL39;SEPP1;TPT1
Loratadine-2000_mg/kg_in_Water-Rat-Heart-5d-up	10/288	0,00983936	0,058310608	0	0	2,386407026	11,02845701	C1QA;RPS17;RPL32;RPS19;RPS8;RPS6;RPS20;RPS3A;RPL39;TPT1
Nimesulide-30_mg/kg_in_Corn_Oil-Rat-Liver-1d-up	10/288	0,00983936	0,058266766	0	0	2,386407026	11,02845701	EEF1B2;RPS17;RPL32;RPS19;RPS5;RPS6;RPS20;RPSA;RPS3A;RPL10A
Procarbazine-54_mg/kg_in_Water-Rat-Liver-0.25d-dn	10/288	0,00983936	0,058222989	0	0	2,386407026	11,02845701	EEF1B2;RPS17;RPL32;RPS8;RPS5;RPL35A;RPSA;RPS3A;RPL39;TPT1
Sulbactam-671_mg/kg_in_Saline-Rat-Kidney-5d-up	10/288	0,00983936	0,058179278	0	0	2,386407026	11,02845701	EEF1B2;RPS17;RPL32;RPS19;ARL6IP5;RPL35A;RPSA;RPS3A;RAPGEF5;RPL39
Chlorpyrifos-82_mg/kg_in_CMC-Rat-Kidney-5d-dn	11/333	0,009889516	0,058431977	0	0	2,270311549	10,48039405	EEF1B2;RPS17;RPL32;RPS19;ARL2BP;RPS6;RPL35A;RPS20;RPSA;SEPP1;RPL39
Harringtonine-23_mg/kg_in_Saline-Rat-Bone_marrow-1d-up	11/333	0,009889516	0,058388175	0	0	2,270311549	10,48039405	EEF1B2;RPS17;RPL32;RPS19;RPS5;RPS6;RPL35A;RPSA;RPL10A;SEPP1;RPL39
1-Naphthyl_Isothiocyanate-30_mg/kg_in_Corn_Oil-Rat-Liver-0.25d-up	10/289	0,01006539	0,059382031	0	0	2,378149562	10,93628339	EEF1B2;RPS17;CXCL11;RPS19;RPS8;RPS6;RPS3A;RPL10A;RPL39;SEPP1
Acyclovir-980_mg/kg_in_Saline-Rat-Liver-0.25d-up	10/289	0,01006539	0,059337583	0	0	2,378149562	10,93628339	C1QA;RPS17;RPL32;RPS8;RPS6;RPL35A;RPS20;RPSA;RPS3A;RPL39
Carboplatin-14_mg/kg_in_Saline-Rat-Liver-1d-dn	10/289	0,01006539	0,059293202	0	0	2,378149562	10,93628339	EEF1B2;RPL32;RPS19;RPS8;RPS5;RPS6;RPSA;RPS3A;RPL10A;TPT1
Diethylstilbestrol-2.8_mg/kg_in_Corn_Oil-Rat-Bone_marrow-3d-dn	10/289	0,01006539	0,059248887	0	0	2,378149562	10,93628339	EEF1B2;RPS17;RPL32;RPS8;FEM1B;RPS6;ARL6IP5;RPS20;RPSA;RPL10A
Fludrocortisone_Acetate-125_mg/kg_in_CMC-Rat-Kidney-5d-up	10/289	0,01006539	0,059204638	0	0	2,378149562	10,93628339	EEF1B2;RPS19;RPS6;RPS20;RPSA;RPL10A;RPL39;GOLGA7;SEPP1;TPT1
Leflunomide-30_mg/kg_in_Corn_Oil-Rat-Bone_marrow-1d-dn	10/289	0,01006539	0,059160456	0	0	2,378149562	10,93628339	EEF1B2;RPL32;RPS8;FEM1B;RPS6;ARL6IP5;RPL35A;RPS20;RPSA;RPS3A
Promethazine-113_mg/kg_in_Saline-Rat-Liver-0.25d-up	10/289	0,01006539	0,059116339	0	0	2,378149562	10,93628339	RPL32;RPS19;RPS8;RPS5;RPS6;RPSA;RPS3A;RPL10A;SEPP1;TPT1
Tazobactam-1500_mg/kg_in_CMC-Rat-Spleen-5d-up	10/289	0,01006539	0,059072288	0	0	2,378149562	10,93628339	CXCL11;RPS19;RPS5;RPS6;RPS20;RPSA;RAPGEF5;RPL10A;RPL39;SEPP1
Cisplatin-1.17_mg/kg_in_Saline-Rat-Spleen-3d-dn	11/334	0,010098416	0,059221983	0	0	2,263514209	10,4017005	EEF1B2;C1QA;RPS17;RPL32;RPS8;RPS6;ARL6IP5;RPSA;RPS3A;RPL10A;TPT1
Sulindac-132_mg/kg_in_Corn_Oil-Rat-Heart-3d-up	11/334	0,010098416	0,059177919	0	0	2,263514209	10,4017005	EEF1B2;C1QA;RPS19;RPS8;RPS6;ARL6IP5;RPSA;RPS3A;RPL10A;RPL39;PDRG1
Cycloheximide-0.25_mg/kg_in_Water-Rat-Liver-5d-up	12/380	0,010099502	0,059140281	0	0	2,17037439	9,973454497	EEF1B2;RPS17;RPL32;RPS19;ARL2BP;RPS8;RPS5;RPS6;RPS20;RPSA;RPS3A;RPL10A
Buspirone-196_mg/kg_in_CMC-Rat-Heart-3d-up	10/290	0,010295359	0,060242385	0	0	2,369949046	10,84503385	RPS17;RPL32;RPS19;RPS5;RPS6;ARL6IP5;RPL35A;RPSA;RPS3A;TPT1
Cortisone-206_mg/kg_in_CMC-Rat-Kidney-5d-up	10/290	0,010295359	0,060197662	0	0	2,369949046	10,84503385	EEF1B2;RPS17;RPS19;ARL2BP;CLP1;RPS8;RPS20;RPSA;RPS3A;TPT1

Doxazosin-500_mg/kg_in_Water-Rat-Heart-3d-dn	10/290	0,010295359	0,060153005	0	0	2,369949046	10,84503385	EEF1B2;RPS17;RPL32;RPS19;RPS8;COL5A3;ARL6IP5;RPSA;RPL39;TPT1
Lamotrigine-26_mg/kg_in_CMC-Rat-Kidney-3d-dn	10/290	0,010295359	0,060108414	0	0	2,369949046	10,84503385	RPL32;RPS19;ARL2BP;RPS6;RPS20;RPSA;RPL10A;RPL39;SEPP1;PDRG1
Lead(IV)_Acetate-200_mg/kg_in_Saline-Rat-Spleen-1d-dn	10/290	0,010295359	0,060063889	0	0	2,369949046	10,84503385	EEF1B2;CXCL11;ARL2BP;RPS8;RPS5;FUNDC1;RPL13A;RPL35A;RPSA;TPT1
Nystatin-134_mg/kg_in_Corn_Oil-Rat-Liver-5d-up	10/290	0,010295359	0,06001943	0	0	2,369949046	10,84503385	EEF1B2;C1QA;RPL32;RPS19;RPS8;RPS5;RPS3A;RPL10A;SEPP1;TPT1
Amitraz-75_mg/kg_in_CMC-Rat-Heart-3d-dn	11/335	0,010310697	0,060064389	0	0	2,25675745	10,32370245	C1QA;RPS17;RPL32;RPS8;COL5A3;ARL6IP5;RPS20;RPSA;RPS3A;RPL39;TPT1
Azathioprine-20_mg/kg_in_Corn_Oil-Rat-Spleen-0.25d-up	11/335	0,010310697	0,060019995	0	0	2,25675745	10,32370245	EEF1B2;RPL32;RPS19;RPS5;RPS6;RPL13A;RPL35A;RPSA;RPL10A;SEPP1;RPL39
Valacyclovir-88_mg/kg_in_CMC-Rat-Kidney-5d-up	11/335	0,010310697	0,059975667	0	0	2,25675745	10,32370245	RPS17;RPL32;RPS19;FEM1B;ARL6IP5;RPS20;RPSA;SEPP1;RPL39;GOLGA7;TPT1
Hydroxyurea-59_mg/kg_in_Saline-Rat-Liver-0.25d-up	9/247	0,010455795	0,060774793	0	0	2,504278142	11,42100805	EEF1B2;RPS17;RPS19;RPS8;RPS6;ARL6IP5;RPS20;RPSA;RPS3A
Busulfan-500_uM_in_DMSO-Rat-Primary_rat_hepatocytes-1d-up	11/336	0,010526395	0,061140037	0	0	2,25004091	10,2463923	EEF1B2;RPS17;CXCL11;RPL32;RPS19;ARL2BP;RPS6;ARL6IP5;RPS3A;RPL39;SLC16A3
Doxorubicin-4_mg/kg_in_Saline-Rat-Heart-28d-up	11/336	0,010526395	0,061094981	0	0	2,25004091	10,2463923	EEF1B2;RPS17;RPL32;RPS19;RPS6;RPL35A;RPS20;RPSA;RPS3A;RPL10A;RPL39
Clopidogrel-400_mg/kg_in_Corn_Oil-Rat-Bone_marrow-3d-dn	10/291	0,010529311	0,061066901	0	0	2,361804891	10,75469683	EEF1B2;RPS17;RPS19;FEM1B;RPSA;RPS3A;RPL10A;RPL39;SEPP1;TPT1
Daunorubicin-3.25_mg/kg_in_Saline-Rat-Liver-3d-dn	10/291	0,010529311	0,061021966	0	0	2,361804891	10,75469683	EEF1B2;RPS17;RPL32;RPS19;RPS6;RPL35A;RPSA;RPS3A;RPL10A;RPL39
Famciclovir-112_mg/kg_in_Saline-Rat-Bone_marrow-1d-dn	10/291	0,010529311	0,060977097	0	0	2,361804891	10,75469683	C1QA;RPS17;RPL32;RPS8;FEM1B;RPS20;RPS3A;RPL10A;RPL39;TPT1
Fluvastatin-94_mg/kg_in_Corn_Oil-Rat-Kidney-1d-dn	10/291	0,010529311	0,060932294	0	0	2,361804891	10,75469683	EEF1B2;RPS17;RPL32;RPS19;RPS8;RPL35A;RPSA;RPS3A;RPL10A;RPL39
Iproniazid-46_mg/kg_in_Water-Rat-Heart-3d-dn	10/291	0,010529311	0,060887556	0	0	2,361804891	10,75469683	RPS17;CXCL11;RPL32;RPS19;RPS8;RPS6;RPSA;RPS3A;RPL39;PDRG1
Mitotane-5000_mg/kg_in_Water-Rat-Bone_marrow-3d-dn	10/291	0,010529311	0,060842884	0	0	2,361804891	10,75469683	EEF1B2;RPS17;RPL32;ARL2BP;RPS8;RPS5;RPS20;RPSA;RPS3A;TPT1
Niacinamide-750_mg/kg_in_Water-Rat-Liver-3d-dn	10/291	0,010529311	0,060798278	0	0	2,361804891	10,75469683	EEF1B2;RPS17;RPL32;RPS19;CLP1;RPS8;RPS20;RPL10A;RPL39;SEPP1
Phenylbutazone-368_mg/kg_in_CMC-Rat-Kidney-1d-up	10/291	0,010529311	0,060753737	0	0	2,361804891	10,75469683	RPS17;RPL32;RPS19;RPS8;FEM1B;RPS6;RPSA;RPL10A;GOLGA7;SEPP1
Tamoxifen-2.5_mg/kg_in_Corn_Oil-Rat-Liver-5d-up	10/291	0,010529311	0,060709262	0	0	2,361804891	10,75469683	RPS17;RPL32;RPS19;RPS8;RPS6;RPL35A;RPS20;RPS3A;RPL39;TPT1
Thioguanine-12_mg/kg_in_Corn_Oil-Rat-Bone_marrow-1d-dn	10/291	0,010529311	0,060664851	0	0	2,361804891	10,75469683	EEF1B2;RPL32;RPS19;RPS8;FUNDC1;RPS6;ARL6IP5;RPSA;RPS3A;RPL39
Diethanolamine-620_mg/kg_in_Water-Rat-Kidney-5d-dn	9/248	0,010715858	0,061694518	0	0	2,494180246	11,31367776	RPS17;RPL32;ARL2BP;RPS8;RPS6;RPL35A;RPS20;RPSA;RPS3A
Lithocholic_Acid-74_uM_in_DMSO-Rat-Primary_rat_hepatocytes-1d-up	9/248	0,010715858	0,061649453	0	0	2,494180246	11,31367776	EEF1B2;RPS17;RPL32;CLP1;RPS8;RPS5;ARL6IP5;RPS20;PDRG1
Sulfadiazine-1170_mg/kg_in_Water-Rat-Kidney-1d-dn	9/248	0,010715858	0,061604453	0	0	2,494180246	11,31367776	RPS17;RPL32;RPS19;RPS6;RPL35A;RPS20;RPSA;RPS3A;RPL39
Valproic_Acid-1500_mg/kg_in_Water-Rat-Liver-5d-dn	9/248	0,010715858	0,061559519	0	0	2,494180246	11,31367776	EEF1B2;C1QA;RPS17;RPL32;RPS19;RPL35A;RPS20;RPL39;SEPP1
Catechol-195_mg/kg_in_Saline-Rat-Bone_marrow-3d-up	11/337	0,010745544	0,061685063	0	0	2,243364231	10,16976255	C1QA;RPS17;RPL32;RPS19;RPS8;FEM1B;RPS6;RPL35A;RPSA;RPS3A;RPL39
Chlorpropamide-269_mg/kg_in_CMC-Rat-Heart-1d-dn	11/337	0,010745544	0,061640135	0	0	2,243364231	10,16976255	EEF1B2;RPS17;RPL32;RPS19;RPS8;RPS5;RPS6;RPS20;RPSA;RPL10A;RPL39
Choline_Chloride-2550_mg/kg_in_Water-Rat-Liver-0.25d-up	11/337	0,010745544	0,061595274	0	0	2,243364231	10,16976255	RPS17;RPL32;RPS19;RPS8;RPS6;ARL6IP5;RPS20;RPSA;RPS3A;RPL10A;SEPP1
Cisplatin-2_mg/kg_in_Saline-Rat-Kidney-3d-up	11/337	0,010745544	0,061550477	0	0	2,243364231	10,16976255	RPS17;CXCL11;RPS19;ARL2BP;CLP1;RPS8;RPS6;RPS20;RPSA;RPS3A;TPT1
Streptozotocin-3000_uM_in_DMSO-Rat-Primary_rat_hepatocytes-1d-up	6/127	0,010760208	0,061589679	0	0	3,247016803	14,71515672	EEF1B2;CLP1;RPS6;RPS20;GOLGA7;PDRG1
Cephalexin-2500_mg/kg_in_CMC-Rat-Kidney-1d-dn	10/292	0,010767287	0,06158544	0	0	2,353716518	10,66526091	RPL32;ARL2BP;CLP1;RPS8;RPS6;RPL35A;RPS20;RPS3A;SEPP1;TPT1
Diclofenac-10_mg/kg_in_Corn_Oil-Rat-Liver-1d-dn	10/292	0,010767287	0,061540748	0	0	2,353716518	10,66526091	EEF1B2;RPS17;RPL32;RPS19;RPS8;RPS5;RPSA;RPS3A;SEPP1;TPT1
Diclofenac-10_mg/kg_in_Corn_Oil-Rat-Liver-3d-dn	10/292	0,010767287	0,061496121	0	0	2,353716518	10,66526091	RPS17;RPL32;RPS19;CLP1;RPS8;RPS5;RPL35A;RPSA;SEPP1;TPT1
Diethylstilbestrol-2.8_mg/kg_in_Corn_Oil-Rat-Bone_marrow-0.25d-dn	10/292	0,010767287	0,061451558	0	0	2,353716518	10,66526091	RPS17;RPL32;RPS19;CLP1;RPS8;FEM1B;RPS6;NKG7;RPS3A;TPT1
Digoxin-11_mg/kg_in_Corn_Oil-Rat-Heart-1d-dn	10/292	0,010767287	0,06140706	0	0	2,353716518	10,66526091	EEF1B2;RPL32;RPS8;RPS6;RPL35A;RPS20;RPL10A;RPL39;TPT1;PDRG1
Gentamicin-267_mg/kg_in_Corn_Oil-Rat-Liver-3d-dn	10/292	0,010767287	0,061362627	0	0	2,353716518	10,66526091	EEF1B2;RPS17;RPL32;RPS19;RPS8;RPS6;RPS20;RPS3A;RPL39;TPT1
Ibuprofen-263_mg/kg_in_Corn_Oil-Rat-Kidney-3d-up	10/292	0,010767287	0,061318258	0	0	2,353716518	10,66526091	C1QA;CXCL11;RPL32;RPS19;RPS8;RPS5;RPS6;RPS20;RPS3A;RPL10A
Mitomycin_C-0.5_mg/kg_in_Saline-Rat-Spleen-1d-dn	10/292	0,010767287	0,061273952	0	0	2,353716518	10,66526091	EEF1B2;RPS17;RPS8;RPS5;FUNDC1;RPL13A;RPL35A;RPSA;RPS3A;TPT1
Sulpiride-1000_mg/kg_in_CMC-Rat-Heart-5d-up	10/292	0,010767287	0,061229711	0	0	2,353716518	10,66526091	RPS17;RPL32;COL5A3;RPS6;RPSA;RPS3A;RAPGEF5;RPL10A;RPL39;TPT1
Busulfan-9_mg/kg_in_Corn_Oil-Rat-Spleen-1d-up	11/338	0,010968179	0,062327111	0	0	2,236727058	10,09380579	C1QA;RPL32;ARL2BP;CLP1;RPS8;RPS6;NKG7;RPS20;RPSA;RPL39;TPT1
Ethylene_Glycol-3525_mg/kg_in_Water-Rat-Liver-3d-up	11/338	0,010968179	0,062282174	0	0	2,236727058	10,09380579	RPS17;CXCL11;RPS19;RPS8;FUNDC1;RPS6;RPS20;RPS3A;RPL10A;RPL39;TPT1
Sulindac-64_mg/kg_in_Corn_Oil-Rat-Kidney-1d-up	11/338	0,010968179	0,062237302	0	0	2,236727058	10,09380579	EEF1B2;RPS17;RPL32;RPS19;RPS5;RPS6;RPS20;RPS3A;RPL10A;RPL39;TPT1
Cyclophosphamide-25_mg/kg_in_Water-Rat-Heart-5d-up	10/293	0,01100933	0,062425833	0	0	2,345683356	10,57671487	EEF1B2;RPS17;CXCL11;RPL32;RPS19;ARL2BP;RPS6;RPS3A;RPL10A;SEPP1
Hydrocortisone-56_mg/kg_in_Corn_Oil-Rat-Liver-3d-dn	10/293	0,01100933	0,062380922	0	0	2,345683356	10,57671487	C1QA;RPS17;RPL32;RPS19;RPS8;FEM1B;RPS6;RPS3A;RPL10A;RPL39
Idarubicin-4_mg/kg_in_Saline-Rat-Heart-28d-up	10/293	0,01100933	0,062336076	0	0	2,345683356	10,57671487	EEF1B2;C1QA;RPS19;RPS6;RPS20;RPSA;RPL10A;SEPP1;RPL39;GOLGA7
Rapamycin-60_mg/kg_in_CMC-Rat-Kidney-5d-up	13/432	0,011049872	0,062520685	0	0	2,068219422	9,31802427	RPL32;RPS8;RPS6;RPL35A;RPSA;RPS3A;EEF1B2;RPS17;RPS19;ARL2BP;RPS20;RPL39;TPT1
Cisplatin-2_mg/kg_in_Saline-Rat-Spleen-5d-dn	11/339	0,011194334	0,063292586	0	0	2,230129043	10,01851474	EEF1B2;C1QA;RPS17;RPL32;RPS19;RPS8;RPS6;RPS20;RPSA;RPS3A;RPL10A
Indomethacin-5_mg/kg_in_Corn_Oil-Rat-Liver-1d-dn	11/339	0,011194334	0,063247183	0	0	2,230129043	10,01851474	EEF1B2;RPS17;RPL32;RPS6;RPL35A;RPS20;RPS3A;RPL10A;RPL39;GOLGA7;TPT1
BW-723C86-10_mg/kg_in_Saline-Rat-Heart-5d-dn	10/294	0,011255483	0,063547084	0	0	2,337704841	10,48904766	RPS17;RPL32;RPS8;COL5A3;RPS6;ARL6IP5;RPL35A;RPS3A;TPT1
Beta-Estradiol-0.3_mg/kg_in_Corn_Oil-Rat-Liver-5d-up	10/294	0,011255483	0,063501563	0	0	2,337704841	10,48904766	EEF1B2;RPS17;RPL32;RPS19;RPS5;RPS6;RPL35A;RPSA;RPL10A;RPL39
Carmustine-4_mg/kg_in_Corn_Oil-Rat-Spleen-1d-dn	10/294	0,011255483	0,063456107	0	0	2,337704841	10,48904766	EEF1B2;RPS17;RPS19;RPS8;RPS5;RPL13A;RPL35A;RPS20;RPSA;TOMM20
Digoxin-11_mg/kg_in_Corn_Oil-Rat-Liver-0.25d-dn	10/294	0,011255483	0,063410717	0	0	2,337704841	10,48904766	EEF1B2;RPS17;RPL32;RPS19;RPS8;RPS6;RPL35A;RPS20;RPS3A;TPT1
Methotrexate-0.3_mg/kg_in_Corn_Oil-Rat-Bone_marrow-1d-dn	10/294	0,011255483	0,063365391	0	0	2,337704841	10,48904766	EEF1B2;RPL32;RPS19;RPS8;FEM1B;RPS6;ARL6IP5;RPL35A;RPS20;RPSA
Mitomycin_C-1.7_mg/kg_in_Saline-Rat-Spleen-1d-dn	10/294	0,011255483	0,06332013	0	0	2,337704841	10,48904766	EEF1B2;RPS17;RPS8;RPS5;FUNDC1;RPL13A;RPL35A;RPSA;GOLGA7;TPT1
Noscapine-570_mg/kg_in_CMC-Rat-Bone_marrow-5d-dn	10/294	0,011255483	0,063274934	0	0	2,337704841	10,48904766	RPS17;RPS19;RPS8;FEM1B;RPS5;RPSA;RPL10A;SEPP1;GOLGA7;TPT1
Progesterone-11.3_mg/kg_in_Corn_Oil-Rat-Liver-3d-up	10/294	0,011255483	0,063229802	0	0	2,337704841	10,48904766	RPL32;RPS19;RPS8;RPS6;RPS20;RPS3A;RPL10A;SEPP1;RPL39;TPT1
Warfarin-0.25_mg/kg_in_CMC-Rat-Liver-5d-up	10/294	0,011255483	0,063184734	0	0	2,337704841	10,48904766	EEF1B2;RPS17;RPL32;RPS19;ARL6IP5;RPS20;RPSA;RAPGEF5;SEPP1;RPL39
Acyclovir-330_mg/kg_in_Saline-Rat-Bone_marrow-1d-up	12/386	0,011328681	0,063550349	0	0	2,136638	9,573030588	EEF1B2;C1QA;RPL32;RPS8;FEM1B;RPS5;RPS6;RPL13A;RPL35A;RPSA;TOMM20;TPT1
Cytarabine-487_mg/kg_in_Saline-Rat-Liver-3d-up	12/386	0,011328681	0,063505118	0	0	2,136638	9,573030588	EEF1B2;C1QA;RPS17;RPL32;RPS19;RPS8;RPS5;RPS6;RPS3A;RPL10A;RPL39;TPT1
Amikacin-82_mg/kg_in_Saline-Rat-Kidney-1d-up	11/340	0,011424044	0,063994148	0	0	2,22356984	9,943882171	EEF1B2;RPS17;RPL32;ARL2BP;RPS8;RPS6;RPSA;RPS3A;RPL10A;SEPP1;TPT1
Interleukin_1_Beta-0.01_mg/kg_in_RSA/Saline-Rat-Liver-0.25d-up	11/340	0,011424044	0,063948665	0	0	2,22356984	9,943882171	C1QA;RPS17;RPS19;RPS8;RPS6;RPS20;RPS3A;RPL10A;RPL39;GOLGA7;TPT1
Altretamine-13_mg/kg_in_Corn_Oil-Rat-Bone_marrow-3d-dn	10/295	0,011505788	0,064360502	0	0	2,329780418	10,40224839	EEF1B2;RPS17;RPL32;RPS8;ARL6IP5;NKG7;RPS20;RPSA;RPS3A;TPT1
Aspirin-375_mg/kg_in_Corn_Oil-Rat-Liver-1d-up	10/295	0,011505788	0,064314823	0	0	2,329780418	10,40224839	EEF1B2;RPS17;RPL32;RPS19;RPS8;RPS6;RPS20;RPSA;RPS3A;SEPP1
Atorvastatin-2.5_mg/kg_in_Corn_Oil-Rat-Kidney-1d-dn	10/295	0,011505788	0,06426921	0	0	2,329780418	10,40224839	EEF1B2;RPS17;RPL32;RPS8;RPSA;RPS3A;RPL10A;RPL39;TOMM20;TPT1
Carboplatin-6_mg/kg_in_Saline-Rat-Bone_marrow-1d-up	10/295	0,011505788	0,064223661	0	0	2,329780418	10,40224839	C1QA;RPS17;RPS19;ARL2BP;RPS8;FEM1B;RPS5;RPL35A;RPS20;TPT1
Cholecalciferol-8_mg/kg_in_CMC-Rat-Kidney-1d-dn	10/295	0,011505788	0,064178177	0	0	2,329780418	10,40224839	RPS19;ARL2BP;RPS8;FUNDC1;RPS6;RPS20;RPS3A;RPL10A;SEPP1;RPL39
Cisplatin-1.17_mg/kg_in_Saline-Rat-Liver-3d-up	10/295	0,011505788	0,064132757	0	0	2,329780418	10,40224839	EEF1B2;RPS17;RPS19;RPS8;RPS6;RPS20;RPSA;RPL10A;RPL39;TPT1
Diazepam-710_mg/kg_in_CMC-Rat-Liver-3d-dn	10/295	0,011505788	0,064087402	0	0	2,329780418	10,40224839	EEF1B2;C1QA;RPS17;RPL32;CLP1;ARL6IP5;RPL35A;RPS3A;SEPP1;TPT1
NN-Dimethylformamide-1400_mg/kg_in_Saline-Rat-Spleen-5d-up	10/295	0,011505788	0,06404211	0	0	2,329780418	10,40224839	RPS17;CXCL11;RPL32;RPS8;RPS5;FUNDC1;RPL35A;RPS3A;RPL39;TPT1
Ofloxacin-30_mg/kg_in_Water-Rat-Heart-5d-up	10/295	0,011505788	0,063996883	0	0	2,329780418	10,40224839	RPS17;RPL32;ARL2BP;RPS6;ARL6IP5;RPS20;RPS3A;RAPGEF5;GOLGA7;TPT1
Vincristine-0.05_mg/kg_in_Saline-Rat-Bone_marrow-5d-up	10/295	0,011505788	0,063951719	0	0	2,329780418	10,40224839	C1QA;RPL32;RPS19;ARL2BP;RPS8;RPS6;NKG7;RPS20;RPSA;RPS3A
Sulfaphenazole-1695_mg/kg_in_Water-Rat-Kidney-5d-dn	9/251	0,011525254	0,064014739	0	0	2,464369327	10,99900931	RPS17;RPL32;RPS19;RPS6;RPL35A;RPS20;RPSA;RPS3A;RPL39
Azlocillin-800_mg/kg_in_Saline-Rat-Kidney-1d-up	12/387	0,011544301	0,064075347	0	0	2,131116972	9,508113392	RPS17;RPL32;RPS8;RPS5;RPS6;RPL13A;RPL35A;RPS20;RPSA;RPS3A;SEPP1;RPL39
Trimethadione-535_mg/kg_in_Water-Rat-Kidney-1d-dn	8/209	0,011707237	0,064933942	0	0	2,630756836	11,70041755	RPS17;RPL32;RPS19;RPS6;RPS20;RPSA;RPS3A;RPL39
Acetaminophen-486_mg/kg_in_Corn_Oil-Rat-Liver-0.25d-up	10/296	0,011760288	0,065182287	0	0	2,321909538	10,31630637	EEF1B2;RPS17;RPL32;RPS19;RPS8;RPS6;RPS3A;RPL10A;SEPP1;PDRG1
Baclofen-6.6_mg/kg_in_CMC-Rat-Heart-3d-dn	10/296	0,011760288	0,065136449	0	0	2,321909538	10,31630637	EEF1B2;RPS17;RPS8;ARL6IP5;RPS20;RPSA;RPS3A;RPL10A;SEPP1;TOMM20
Chlorpyrifos-82_mg/kg_in_CMC-Rat-Heart-3d-dn	10/296	0,011760288	0,065090675	0	0	2,321909538	10,31630637	RPS17;RPL32;RPS19;ARL2BP;RPS6;RPS20;RPS3A;RPL10A;RPL39;TPT1
Diisopropyl_Fluorophosphate-3.75_mg/kg_in_Water-Rat-Heart-1d-dn	10/296	0,011760288	0,065044965	0	0	2,321909538	10,31630637	EEF1B2;RPS17;RPL32;RPS19;FUNDC1;RPS6;RPS20;RPSA;RPL10A;RPL39
Epalrestat-2500_mg/kg_in_CMC-Rat-Liver-3d-up	10/296	0,011760288	0,064999319	0	0	2,321909538	10,31630637	RPL32;RPS19;RPS8;RPS5;RPS6;RPS20;RPSA;RPS3A;RPL10A;RPL39
Epirubicin-2.7_mg/kg_in_Saline-Rat-Liver-5d-dn	10/296	0,011760288	0,064953738	0	0	2,321909538	10,31630637	EEF1B2;RPS17;RPL32;RPS19;RPS8;RPS6;RPS20;RPS3A;RPL10A;RPL39
Etoposide-188_mg/kg_in_Corn_Oil-Rat-Liver-5d-dn	10/296	0,011760288	0,06490822	0	0	2,321909538	10,31630637	RPS17;RPL32;RPS8;RPS5;RPL35A;RPSA;RPS3A;RPL10A;RPL39;TPT1
Fenbendazole-375_mg/kg_in_Corn_Oil-Rat-Liver-3d-dn	10/296	0,011760288	0,064862766	0	0	2,321909538	10,31630637	EEF1B2;RPS17;RPL32;RPS8;RPL35A;RPS20;RPS3A;SEPP1;RPL39;TPT1
Levamisole-120_mg/kg_in_Water-Rat-Liver-1d-up	10/296	0,011760288	0,064817376	0	0	2,321909538	10,31630637	RPS17;RPL32;RPS8;RPS5;RPL35A;RPSA;RPS3A;RPL10A;RPL39;TPT1
Levofloxacin-739_mg/kg_in_Water-Rat-Heart-5d-up	10/296	0,011760288	0,064772049	0	0	2,321909538	10,31630637	EEF1B2;RPS17;RPL32;ARL2BP;RPL35A;RPS3A;RAPGEF5;SEPP1;GOLGA7;TPT1
Mifepristone-3_mg/kg_in_Corn_Oil-Rat-Liver-1d-dn	10/296	0,011760288	0,064726786	0	0	2,321909538	10,31630637	EEF1B2;C1QA;RPS17;RPL32;RPS8;RPS20;RPSA;RPS3A;SEPP1;TPT1
Penicillamine-852_mg/kg_in_Water-Rat-Liver-5d-up	10/296	0,011760288	0,064681585	0	0	2,321909538	10,31630637	EEF1B2;RPS17;CLP1;RPS8;RPS5;RPS6;RPL35A;RPS3A;SEPP1;GOLGA7
Phenacetin-619_mg/kg_in_Corn_Oil-Rat-Liver-0.25d-up	10/296	0,011760288	0,064636448	0	0	2,321909538	10,31630637	RPS17;RPL32;RPS19;CLP1;RPS8;RPS6;ARL6IP5;RPL35A;RPSA;SEPP1
Sertraline-210_mg/kg_in_Corn_Oil-Rat-Liver-5d-dn	10/296	0,011760288	0,064591374	0	0	2,321909538	10,31630637	EEF1B2;RPS17;RPL32;RPS19;CLP1;RPS6;RPL35A;RPS3A;RPL10A;TPT1
Simvastatin-1200_mg/kg_in_Corn_Oil-Rat-Kidney-3d-up	10/296	0,011760288	0,064546362	0	0	2,321909538	10,31630637	RPS17;RPL32;RPS8;RPS5;ARL6IP5;RPL35A;RPS20;RPSA;RPS3A;SEPP1
Sparteine-33_mg/kg_in_Water-Rat-Heart-0.25d-up	10/296	0,011760288	0,064501414	0	0	2,321909538	10,31630637	EEF1B2;RPS17;RPL32;RPS19;RPS6;RPL35A;RPS20;RPS3A;RPL39;TPT1
Sulindac-132_mg/kg_in_Corn_Oil-Rat-Liver-1d-up	10/296	0,011760288	0,064456528	0	0	2,321909538	10,31630637	EEF1B2;RPS17;RPL32;RPS19;RPS6;RPS20;RPSA;RPS3A;RPL10A;RPL39

Warfarin-0.25_mg/kg_in_CMC-Rat-Liver-1d-up	10/296	0,011760288	0,064411704	0	0	2,321909538	10,31630637	EEF1B2;RPS17;RPL32;RPS6;ARL6IP5;RPS20;RPSA;RPS3A;SEPP1;TPT1
Carboplatin-6_mg/kg_in_Saline-Rat-Kidney-1d-dn	9/252	0,011804966	0,064611474	0	0	2,454590083	10,8965022	EEF1B2;RPS17;RPL32;RPS19;RPS5;RPL35A;RPS3A;TPT1;PDRG1
Acetazolamide-47_mg/kg_in_Corn_Oil-Rat-Liver-5d-dn	10/297	0,012019026	0,065737396	0	0	2,314091661	10,23121102	EEF1B2;RPS17;RPL32;RPS19;RPS6;RPL35A;RPS3A;RPL10A;SEPP1;RPL39
Azathioprine-20_mg/kg_in_Corn_Oil-Rat-Bone_marrow-0.25d-dn	10/297	0,012019026	0,065691776	0	0	2,314091661	10,23121102	RPS17;RPL32;RPS19;RPS8;FEM1B;RPS5;RPS6;ARL6IP5;RPS20;RPSA
Benzothiazyl_Disulfide-2600_mg/kg_in_Corn_Oil-Rat-Kidney-3d-up	10/297	0,012019026	0,06564622	0	0	2,314091661	10,23121102	RPS17;CXCL11;RPL32;ARL2BP;CLP1;RPS8;RPS6;RPS20;RPS3A;TPT1
Busulfan-500_uM_in_DMSO-Rat-Primary_rat_hepatocytes-0.67d-up	10/297	0,012019026	0,065600727	0	0	2,314091661	10,23121102	EEF1B2;RPS17;CXCL11;RPL32;ARL2BP;FEM1B;RPS6;ARL6IP5;RPL39;GOLGA7
Cyclophosphamide-1320_uM_in_DMSO-Rat-Primary_rat_hepatocytes-0.67d-up	10/297	0,012019026	0,065555297	0	0	2,314091661	10,23121102	EEF1B2;RPL32;RPS19;ARL2BP;CLP1;RPS8;FEM1B;RPS5;RPS6;PDRG1
Diethylstilbestrol-2.8_mg/kg_in_Corn_Oil-Rat-Kidney-0.25d-dn	10/297	0,012019026	0,065509931	0	0	2,314091661	10,23121102	EEF1B2;RPS17;RPL32;RPS19;RPS8;RPS6;ARL6IP5;RPS20;RPSA;RPS3A
Ethambutol-998_mg/kg_in_CMC-Rat-Kidney-1d-up	10/297	0,012019026	0,065464626	0	0	2,314091661	10,23121102	EEF1B2;RPS17;RPL32;RPS19;RPS8;ARL6IP5;RPL13A;RPL35A;RPS20;RPS3A
Irinotecan-5_mg/kg_in_Saline-Rat-Bone_marrow-5d-up	10/297	0,012019026	0,065419385	0	0	2,314091661	10,23121102	C1QA;RPL32;RPS19;RPS8;RPS6;NKG7;RPS20;RPSA;RPL10A;TPT1
Lacidipine-1_mg/kg_in_Corn_Oil-Rat-Heart-0.25d-dn	10/297	0,012019026	0,065374206	0	0	2,314091661	10,23121102	RPS17;RPL32;ARL2BP;RPS8;FUNDC1;RPS6;ARL6IP5;RPL13A;RPS3A;SEPP1
Progesterone-148_uM_in_DMSO-Rat-Primary_rat_hepatocytes-0.67d-up	10/297	0,012019026	0,065329089	0	0	2,314091661	10,23121102	EEF1B2;RPL32;RPS19;RPS6;RPL35A;RPSA;RPS3A;RPL10A;RPL39;TOMM20
Tegafur-175_mg/kg_in_Water-Rat-Bone_marrow-5d-up	10/297	0,012019026	0,065284034	0	0	2,314091661	10,23121102	EEF1B2;C1QA;RPL32;ARL2BP;RPS8;RPS6;ARL6IP5;RPS3A;RPL39;TPT1
Vancomycin-160_mg/kg_in_Saline-Rat-Kidney-3d-up	10/297	0,012019026	0,065239042	0	0	2,314091661	10,23121102	RPS17;RPL32;RPS19;RPS8;RPS5;RPS6;RPL13A;RPL35A;RPSA;SEPP1
Verapamil-108_mg/kg_in_Water-Rat-Heart-5d-up	10/297	0,012019026	0,065194111	0	0	2,314091661	10,23121102	RPS17;RPL32;RPS19;RPS5;RPS6;RPSA;RAPGEF5;RPL10A;RPL39;TPT1
Vinorelbine-1.5_mg/kg_in_Saline-Rat-Liver-3d-up	10/297	0,012019026	0,065149243	0	0	2,314091661	10,23121102	EEF1B2;RPS17;RPL32;RPS19;ARL6IP5;RPS20;RPSA;RPS3A;RPL10A;SEPP1
Zaleplon-100_mg/kg_in_Water-Rat-Liver-5d-dn	10/297	0,012019026	0,065104436	0	0	2,314091661	10,23121102	EEF1B2;RPL32;RPS19;CLP1;RPS8;RPS6;RPL35A;RPS3A;RPL10A;TPT1
Famciclovir-1200_mg/kg_in_Saline-Rat-Spleen-3d-dn	9/253	0,012089724	0,06544238	0	0	2,444888146	10,79515783	EEF1B2;C1QA;RPL32;ARL2BP;RPS8;FUNDC1;RPS6;ARL6IP5;RPSA
Doxorubicin-3_mg/kg_in_Saline-Rat-Kidney-1d-up	11/343	0,012134856	0,065641572	0	0	2,204121708	9,72386483	EEF1B2;RPS17;RPL32;RPS19;RPS8;RPS6;RPSA;RPS3A;RPL10A;RPL39;TPT1
Atorvastatin-2.5_mg/kg_in_Corn_Oil-Rat-Liver-5d-up	10/298	0,012282044	0,066392162	0	0	2,306326253	10,14695198	EEF1B2;RPS17;RPL32;RPS19;RPS20;RPSA;RPS3A;RPL10A;RPL39;TPT1
Azathioprine-20_mg/kg_in_Corn_Oil-Rat-Bone_marrow-3d-up	10/298	0,012282044	0,066346625	0	0	2,306326253	10,14695198	EEF1B2;C1QA;CLP1;RPS5;NKG7;RPL35A;RPS20;SEPP1;RPL39;GOLGA7
Bezafibrate-617_mg/kg_in_Corn_Oil-Rat-Liver-0.25d-up	10/298	0,012282044	0,066301151	0	0	2,306326253	10,14695198	EEF1B2;C1QA;RPS17;RPL32;RPS19;RPS6;RPS3A;RPL10A;RPL39;TPT1
Bromhexine-1200_mg/kg_in_CMC-Rat-Liver-3d-up	10/298	0,012282044	0,06625574	0	0	2,306326253	10,14695198	RPS17;RPL32;RPS19;RPS8;RPS5;RPS6;RPS20;RPS3A;RPL10A;SEPP1
Carmustine-16_mg/kg_in_Corn_Oil-Rat-Bone_marrow-1d-up	10/298	0,012282044	0,06621039	0	0	2,306326253	10,14695198	EEF1B2;C1QA;RPS17;COL5A3;RPS5;NKG7;RPL35A;RPL10A;RPL39;DUSP14
Ethylene_Glycol-3525_mg/kg_in_Water-Rat-Liver-1d-up	10/298	0,012282044	0,066165102	0	0	2,306326253	10,14695198	C1QA;RPS17;CXCL11;RPS8;FUNDC1;RPL35A;RPS20;RPSA;RPS3A;TPT1
Etodolac-24_mg/kg_in_CMC-Rat-Liver-5d-up	10/298	0,012282044	0,066119877	0	0	2,306326253	10,14695198	EEF1B2;C1QA;RPS17;RPS19;ARL2BP;RPS6;RPSA;RPS3A;RPL10A;RPL39
Fenoprofen-52_mg/kg_in_Corn_Oil-Rat-Kidney-1d-up	10/298	0,012282044	0,066074713	0	0	2,306326253	10,14695198	RPL32;RPS19;RPS8;RPS6;RPS20;RPSA;RPS3A;RPL10A;RPL39;PDRG1
Gentamicin-267_mg/kg_in_Corn_Oil-Rat-Liver-5d-dn	10/298	0,012282044	0,066029611	0	0	2,306326253	10,14695198	EEF1B2;RPS17;RPL32;RPS19;RPS8;RPS6;RPS20;RPS3A;RPL39;TPT1
Oxaprozin-1500_mg/kg_in_CMC-Rat-Kidney-3d-up	10/298	0,012282044	0,06598457	0	0	2,306326253	10,14695198	RPS17;RPL32;RPS19;RPS8;RPS6;RPS20;RPS3A;RAPGEF5;RPL10A;RPL39
Diclofenac-3.5_mg/kg_in_Corn_Oil-Rat-Kidney-3d-up	11/344	0,012379137	0,066460862	0	0	2,197714377	9,6517961	C1QA;RPL32;ARL2BP;RPS8;RPS5;RPL35A;RPS20;RPSA;RPS3A;TOMM20;TPT1
Gentamicin-2900_uM_in_DMSO-Rat-Primary_rat_hepatocytes-1d-dn	11/344	0,012379137	0,066415589	0	0	2,197714377	9,6517961	RPS17;CXCL11;RPL32;ARL2BP;RPS8;RPS5;RPS6;ARL6IP5;RPSA;RPL10A;SEPP1
Isoprenaline-15_mg/kg_in_Saline-Rat-Heart-1d-dn	11/344	0,012379137	0,066370377	0	0	2,197714377	9,6517961	RPS17;RPL32;RPS8;FUNDC1;RPS6;RPS20;RPS3A;RPL10A;SEPP1;RPL39;TPT1
Loratadine-2000_mg/kg_in_Water-Rat-Heart-3d-up	11/344	0,012379137	0,066325227	0	0	2,197714377	9,6517961	RPS17;RPL32;RPS19;RPS5;RPS6;RPL35A;RPS20;RPSA;RPS3A;RPL10A;GOLGA7
Rifapentine-75_mg/kg_in_Corn_Oil-Rat-Kidney-5d-up	11/344	0,012379137	0,066280139	0	0	2,197714377	9,6517961	EEF1B2;RPS17;RPL32;RPS19;RPS6;RPL35A;RPS20;RPSA;RPS3A;RPL10A;RPL39
Nystatin-134_mg/kg_in_Corn_Oil-Rat-Kidney-5d-dn	9/254	0,01237958	0,066237483	0	0	2,435262602	10,69495961	EEF1B2;RPS17;RPL32;CLP1;RPS8;RPS5;RPL35A;RPS3A;TOMM20
Chlormadinone_Acetate-2000_mg/kg_in_CMC-Rat-Kidney-3d-dn	10/299	0,012549385	0,067100446	0	0	2,298612787	10,06351899	EEF1B2;RPL32;RPS8;FEM1B;RPS6;ARL6IP5;RPL35A;RPSA;RPL10A;SEPP1
Chloroform-60_mg/kg_in_Water-Rat-Liver-1d-up	10/299	0,012549385	0,067054923	0	0	2,298612787	10,06351899	EEF1B2;RPS17;RPL32;RPS5;RPS6;RPL35A;RPS3A;RPL10A;RPL39;TPT1
Clomipramine-115_mg/kg_in_Water-Rat-Liver-1d-up	10/299	0,012549385	0,067009462	0	0	2,298612787	10,06351899	RPS17;RPL32;RPS8;RPS5;RPS6;RPL35A;RPSA;RPS3A;RPL39;GOLGA7
Fluocinolone_Acetonide-2.5_mg/kg_in_Corn_Oil-Rat-Liver-1d-dn	10/299	0,012549385	0,066964063	0	0	2,298612787	10,06351899	C1QA;RPS17;RPL32;RPS19;RPS6;RPSA;RPS3A;RPL10A;SEPP1;RPL39
Galantamine-110_mg/kg_in_CMC-Rat-Heart-5d-up	10/299	0,012549385	0,066918725	0	0	2,298612787	10,06351899	RPS17;CXCL11;RPL32;RPS19;RPS6;RPSA;RPS3A;RPL10A;RPL39;TPT1
Itraconazole-1093_mg/kg_in_Corn_Oil-Rat-Liver-1d-up	10/299	0,012549385	0,066873448	0	0	2,298612787	10,06351899	EEF1B2;RPL32;RPS19;RPS8;RPS6;RPS20;RPS3A;RPL10A;RPL39;TPT1
Promethazine-113_mg/kg_in_Saline-Rat-Liver-5d-dn	10/299	0,012549385	0,066828233	0	0	2,298612787	10,06351899	EEF1B2;C1QA;RPS17;RPL32;RPS8;RPS6;RPS3A;SEPP1;RPL39;TPT1
Staurosporine-1.3_uM_in_DMSO-Rat-Primary_rat_hepatocytes-0.67d-dn	10/299	0,012549385	0,066783079	0	0	2,298612787	10,06351899	RPS17;RPL32;RPS19;RPS8;RPS5;RPL35A;RPS20;RPS3A;TPT1;PDRG1
Sulfisoxazole-250_mg/kg_in_Corn_Oil-Rat-Liver-1d-up	10/299	0,012549385	0,066737985	0	0	2,298612787	10,06351899	EEF1B2;C1QA;RPS17;RPL32;RPS19;RPS8;RPL35A;RPS20;RPS3A;TPT1
Terazosin-657_mg/kg_in_Water-Rat-Heart-5d-dn	10/299	0,012549385	0,066692953	0	0	2,298612787	10,06351899	EEF1B2;RPS17;RPL32;ARL2BP;RPS8;RPS20;RPSA;RPS3A;TOMM20;TPT1
Valdecoxib-21_mg/kg_in_CMC-Rat-Heart-5d-dn	10/299	0,012549385	0,066647981	0	0	2,298612787	10,06351899	C1QA;RPS17;RPS19;ARL2BP;COL5A3;FUNDC1;RPS6;RPL10A;SEPP1;GOLGA7
Clotrimazole-89_mg/kg_in_Corn_Oil-Rat-Liver-1d-dn	11/345	0,012627148	0,067015779	0	0	2,19134419	9,580351258	EEF1B2;C1QA;RPS17;RPL32;RPS6;RPSA;RPS3A;RPL10A;SEPP1;RPL39;TPT1
Valproic_Acid-850_mg/kg_in_Water-Rat-Liver-3d-up	11/345	0,012627148	0,06697065	0	0	2,19134419	9,580351258	EEF1B2;C1QA;RPS17;RPL32;RPS5;RPS6;RPL35A;RPSA;RPS3A;RPL10A;RPL39
Sulfathiazole-2629_mg/kg_in_Water-Rat-Kidney-1d-dn	8/212	0,012671918	0,067162869	0	0	2,593529145	11,32948696	RPS17;RPL32;RPS19;RPS6;ARL6IP5;RPS20;RPSA;RPL39
Amantadine-58_mg/kg_in_Water-Rat-Liver-0.25d-dn	9/255	0,012674589	0,067131851	0	0	2,425712553	10,59589122	EEF1B2;RPS17;RPL32;RPS8;RPL35A;RPS20;RPS3A;RPL39;TPT1
1-Naphthyl_Isothiocyanate-60_mg/kg_in_Corn_Oil-Rat-Kidney-3d-up	10/300	0,012821091	0,067862173	0	0	2,290950745	9,980901987	EEF1B2;RPS17;RPL32;RPS8;RPS5;RPS6;RPL13A;RPL35A;RPS20;RPS3A
Aflatoxin_B1-0.3_mg/kg_in_CMC-Rat-Liver-5d-dn	10/300	0,012821091	0,067816598	0	0	2,290950745	9,980901987	C1QA;RPL32;CLP1;RPS8;RPS5;RPS6;RPS20;RPSA;RPS3A;RPL10A
Amikacin-20_mg/kg_in_Saline-Rat-Kidney-1d-dn	10/300	0,012821091	0,067771083	0	0	2,290950745	9,980901987	EEF1B2;RPS17;RPL32;CLP1;RPS5;FUNDC1;RPL35A;RPS20;RPS3A;SEPP1
Bezafibrate-100_mg/kg_in_Corn_Oil-Rat-Kidney-5d-up	10/300	0,012821091	0,06772563	0	0	2,290950745	9,980901987	RPS17;RPL32;RPS19;ARL2BP;RPS8;ARL6IP5;RPS20;RPS3A;RPL10A;TPT1
Bromisovalum-250_mg/kg_in_Corn_Oil-Rat-Liver-5d-up	10/300	0,012821091	0,067680237	0	0	2,290950745	9,980901987	RPS17;RPS19;RPS8;RPS6;RPS20;RPSA;RPS3A;SEPP1;RPL39;TPT1
Carbon_Tetrachloride-1175_mg/kg_in_Corn_Oil-Rat-Kidney-1d-dn	10/300	0,012821091	0,067634906	0	0	2,290950745	9,980901987	EEF1B2;RPS17;RPL32;ARL2BP;ARL6IP5;RPL35A;RPSA;RPS3A;RPL39;TOMM20
Carbon_Tetrachloride-1175_mg/kg_in_Corn_Oil-Rat-Kidney-1d-up	10/300	0,012821091	0,067589635	0	0	2,290950745	9,980901987	C1QA;RPS19;RPS8;RPS5;RPS6;RPS20;RAPGEF5;SEPP1;GOLGA7;TPT1
Cinnarizine-750_mg/kg_in_CMC-Rat-Liver-1d-up	10/300	0,012821091	0,067544424	0	0	2,290950745	9,980901987	RPS17;RPL32;RPS19;RPS8;RPS5;RPS6;RPSA;RPS3A;RPL10A;TPT1
Econazole-334_mg/kg_in_Corn_Oil-Rat-Liver-5d-up	10/300	0,012821091	0,067499274	0	0	2,290950745	9,980901987	EEF1B2;RPS17;RPL32;RPS8;RPS6;RPL35A;RPS20;RPS3A;RPL39;TPT1
Ethanol-3000_mg/kg_in_Saline-Rat-Liver-3d-up	10/300	0,012821091	0,067454184	0	0	2,290950745	9,980901987	EEF1B2;RPS17;RPL32;RPS19;RPS8;RPS6;RPL35A;RPS20;RPS3A;RPL10A
Etoposide-188_mg/kg_in_Corn_Oil-Rat-Bone_marrow-1d-up	10/300	0,012821091	0,067409155	0	0	2,290950745	9,980901987	EEF1B2;C1QA;RPS17;RPS19;NKG7;RPL35A;RPS20;RPS3A;SEPP1;RPL39
Hydroxyurea-400_mg/kg_in_Saline-Rat-Spleen-1d-dn	10/300	0,012821091	0,067364185	0	0	2,290950745	9,980901987	EEF1B2;RPS17;RPL32;RPS8;RPS5;RPL13A;RPL35A;RPS20;RPS3A;TPT1
Lovastatin-450_mg/kg_in_Corn_Oil-Rat-Liver-5d-up	10/300	0,012821091	0,067319276	0	0	2,290950745	9,980901987	RPS17;CXADR;RPL32;RPS19;RPS6;RPS20;RPSA;RPL10A;RPL39;TPT1
Methylparaben-1500_mg/kg_in_CMC-Rat-Liver-1d-up	10/300	0,012821091	0,067274426	0	0	2,290950745	9,980901987	RPS17;RPS19;RPS8;RPS5;RPS6;RPSA;RPS3A;RPL10A;RPL39;TPT1
N-Nitrosodiethylamine-100_mg/kg_in_Saline-Rat-Liver-1d-up	10/300	0,012821091	0,067229636	0	0	2,290950745	9,980901987	EEF1B2;CXCL11;RPS19;RPS8;RPS6;RPS20;SEPP1;RPL39;TPT1;PDRG1
NN-Dimethylformamide-140_mg/kg_in_Saline-Rat-Liver-0.25d-up	10/300	0,012821091	0,067184906	0	0	2,290950745	9,980901987	EEF1B2;C1QA;RPS17;RPL32;RPS5;RPL35A;RPSA;RPS3A;RPL39;TPT1
Nitrazepam-310_mg/kg_in_CMC-Rat-Liver-3d-dn	10/300	0,012821091	0,067140235	0	0	2,290950745	9,980901987	RPL32;CLP1;RPS8;ARL6IP5;RPL35A;RPS20;RPSA;RPL10A;SEPP1;TPT1
Sildenafil-420_mg/kg_in_Water-Rat-Liver-1d-up	10/300	0,012821091	0,067095624	0	0	2,290950745	9,980901987	EEF1B2;RPS17;RPL32;RPS8;RPL13A;RPL35A;RPS20;RPS3A;RPL39;TPT1
Tetracycline-1500_mg/kg_in_Water-Rat-Liver-5d-up	10/300	0,012821091	0,067051072	0	0	2,290950745	9,980901987	RPS17;RPL32;RPS5;RPS6;ARL6IP5;RPS20;RPSA;RAPGEF5;SEPP1;RPL39
Tranexamic_Acid-2000_mg/kg_in_CMC-Rat-Heart-3d-dn	10/300	0,012821091	0,067006579	0	0	2,290950745	9,980901987	EEF1B2;RPS17;RPL32;RPS8;RPS6;ARL6IP5;RPL35A;RPS20;RPS3A;RPL39
Methotrexate-27_mg/kg_in_Corn_Oil-Rat-Kidney-1d-up	12/393	0,012905218	0,067401521	0	0	2,098580835	9,129085985	RPS17;RPL32;RPS8;RPS5;RPS6;RPL35A;RPS20;RPSA;RPS3A;RPL39;TOMM20;TPT1
Cloxacillin-1200_mg/kg_in_Water-Rat-Kidney-1d-up	9/256	0,012974803	0,067720045	0	0	2,416237113	10,49793663	EEF1B2;RPS17;RPL32;RPS19;RPS8;RPS6;RPS20;RPL10A;RPL39
Ofloxacin-3000_mg/kg_in_Water-Rat-Kidney-1d-dn	8/213	0,013006078	0,067838323	0	0	2,581352952	11,20910835	RPS17;RPL32;RPS19;RPS6;RPL35A;RPS20;RPSA;RPL39
Sodium_Selenate-1.6_mg/kg_in_Water-Rat-Kidney-1d-dn	8/213	0,013006078	0,067793427	0	0	2,581352952	11,20910835	EEF1B2;RPS17;RPL32;RPS19;RPS6;RPSA;RPS3A;RPL39
Allyl_Alcohol-32_mg/kg_in_Saline-Rat-Liver-3d-up	10/301	0,013097205	0,068223272	0	0	2,283339613	9,899091045	EEF1B2;RPS17;RPS19;RPS8;RPS5;RPS6;RPS20;RPSA;RPS3A;RPL10A
Azauridine-1500_mg/kg_in_CMC-Rat-Bone_marrow-5d-dn	10/301	0,013097205	0,068178181	0	0	2,283339613	9,899091045	RPS17;RPL32;RPS8;RPS5;RPS6;ARL6IP5;RPL13A;RPL35A;RPS3A;TPT1
Carvedilol-20_mg/kg_in_Corn_Oil-Rat-Heart-1d-dn	10/301	0,013097205	0,068133149	0	0	2,283339613	9,899091045	RPL32;RPS19;ARL2BP;RPS8;FEM1B;FUNDC1;RPS6;ARL6IP5;RPS20;RPSA
Dexamethasone-1_mg/kg_in_Corn_Oil-Rat-Liver-0.25d-up	10/301	0,013097205	0,068088177	0	0	2,283339613	9,899091045	EEF1B2;RPS17;RPL32;RPS19;RPS6;ARL6IP5;RPSA;RPS3A;RPL10A;TPT1
Digoxin-0.26_mg/kg_in_Corn_Oil-Rat-Liver-3d-dn	10/301	0,013097205	0,068043264	0	0	2,283339613	9,899091045	EEF1B2;C1QA;RPS17;RPL32;RPS8;RPS5;RPS6;RPSA;RPL10A;TPT1
Fluocinolone_Acetonide-2.5_mg/kg_in_Corn_Oil-Rat-Heart-3d-dn	10/301	0,013097205	0,06799841	0	0	2,283339613	9,899091045	RPS17;RPL32;RPS19;ARL2BP;RPS8;COL5A3;RPS6;RPSA;RPS3A;TPT1
Ketamine-30_mg/kg_in_Saline-Rat-Heart-5d-up	10/301	0,013097205	0,067953615	0	0	2,283339613	9,899091045	C1QA;RPS17;RPL32;RPS19;RPL35A;RPS20;RPSA;RPS3A;SEPP1;RPL39
Pravastatin-1200_mg/kg_in_Corn_Oil-Rat-Skeletal_muscle-3d-dn	10/301	0,013097205	0,067908879	0	0	2,283339613	9,899091045	RPS8;FEM1B;COL5A3;ARL6IP5;RPS20;RPL10A;SEPP1;RPL39;SLC16A3;TPT1
Tranilast-300_mg/kg_in_CMC-Rat-Heart-1d-dn	10/301	0,013097205	0,067864202	0	0	2,283339613	9,899091045	RPL32;RPS19;RPS8;COL5A3;RPS6;ARL6IP5;RPSA;RPL10A;SEPP1;GOLGA7
Amitriptyline-72_uM_in_DMSO-Rat-Primary_rat_hepatocytes-1d-up	11/347	0,013134498	0,068012693	0	0	2,178713965	9,439306728	RPS17;RPL32;RPS19;ARL2BP;RPS8;RPS5;RPS3A;RPL10A;RPL39;TPT1;PDRG1
Clarithromycin-56_mg/kg_in_Water-Rat-Liver-3d-up	11/347	0,013134498	0,067968007	0	0	2,178713965	9,439306728	EEF1B2;RPS17;RPL32;RPS19;RPS8;RPS5;RPS6;RPL35A;RPS20;RPS3A;RPL10A
Fluocinolone_Acetonide-2.5_mg/kg_in_Corn_Oil-Rat-Liver-3d-up	11/347	0,013134498	0,067923379	0	0	2,178713965	9,439306728	EEF1B2;RPS17;RPL32;RPS19;RPS8;RPS5;RPS6;RPL35A;RPS3A;RPL10A;TPT1
Ubenimex-1300_mg/kg_in_Water-Rat-Kidney-5d-up	11/347	0,013134498	0,06787881	0	0	2,178713965	9,439306728	C1QA;RPS17;RPL32;RPS19;RPS6;RPS20;RPSA;RPS3A;RPL10A;RPL39;PDRG1
Fluoxetine-39.6_uM_in_DMSO-Rat-Primary_rat_hepatocytes-1d-up	9/257	0,013280275	0,068587177	0	0	2,406835413	10,40108011	EEF1B2;RPS17;RPL32;RPS8;RPS5;RPS6;RPSA;RPS3A;RPL10A
123-Trichloropropane-108_mg/kg_in_CMC-Rat-Kidney-5d-up	10/302	0,013377769	0,069045421	0	0	2,275778885	9,818076388	EEF1B2;RPS17;RPS19;CLP1;RPS8;RPSA;RPS3A;RPL10A;RPL39;TPT1
Acetazolamide-47_mg/kg_in_Corn_Oil-Rat-Liver-0.25d-dn	10/302	0,013377769	0,069000204	0	0	2,275778885	9,818076388	EEF1B2;C1QA;RPS17;RPL32;RPL35A;RPS20;RPS3A;SEPP1;RPL39;TPT1

Aspirin-375_mg/kg_in_Corn_Oil-Rat-Liver-3d-dn	10/302	0,013377769	0,068955047	0	0	2,275778885	9,818076388	C1QA;RPS17;RPL32;CLP1;RPS8;RPS6;RPL35A;RPS20;RPL39;TPT1
Azathioprine-20_mg/kg_in_Corn_Oil-Rat-Bone_marrow-3d-dn	10/302	0,013377769	0,068909949	0	0	2,275778885	9,818076388	RPS17;RPL32;RPS8;FEM1B;RPS6;ARL6IP5;RPSA;RPS3A;RPL10A;TPT1
Carmustine-16_mg/kg_in_Corn_Oil-Rat-Bone_marrow-1d-dn	10/302	0,013377769	0,06886491	0	0	2,275778885	9,818076388	RPL32;RPS19;RPS8;FEM1B;RPS6;ARL6IP5;RPS20;RPSA;RPS3A;TPT1
Chloroform-600_mg/kg_in_Water-Rat-Liver-1d-up	10/302	0,013377769	0,06881993	0	0	2,275778885	9,818076388	RPS17;RPL32;RPS8;RPS5;RPS6;RPL35A;RPS20;RPSA;RPL39;TPT1
Cimetidine-2000_mg/kg_in_CMC-Rat-Heart-3d-dn	10/302	0,013377769	0,068775008	0	0	2,275778885	9,818076388	EEF1B2;RPS17;ARL2BP;RPS8;FUNDC1;RPS6;ARL6IP5;RPS20;RPSA;RPS3A
Cloxacillin-1200_mg/kg_in_Water-Rat-Kidney-5d-up	10/302	0,013377769	0,068730145	0	0	2,275778885	9,818076388	EEF1B2;RPS17;RPL32;RPS19;RPS5;ARL6IP5;RPL13A;RPL35A;RPS20;RPS3A
Daunorubicin-1_mg/kg_in_Saline-Rat-Kidney-5d-dn	10/302	0,013377769	0,06868534	0	0	2,275778885	9,818076388	RPS17;RPL32;RPS19;ARL2BP;ARL6IP5;RPS20;RPS3A;RPL10A;SEPP1;TPT1
Ethisterone-1500_mg/kg_in_CMC-Rat-Liver-3d-up	10/302	0,013377769	0,068640594	0	0	2,275778885	9,818076388	C1QA;RPS17;RPL32;RPS6;ARL6IP5;RPL35A;RPS20;RPSA;RPS3A;RPL10A
Granisetron-175_mg/kg_in_Water-Rat-Liver-5d-up	10/302	0,013377769	0,068595906	0	0	2,275778885	9,818076388	RPS17;RPL32;RPS19;RPS8;RPS5;RPS6;RPS20;RPL10A;SEPP1;RPL39
Lead(IV)_Acetate-200_mg/kg_in_Saline-Rat-Bone_marrow-0.25d-up	10/302	0,013377769	0,068551277	0	0	2,275778885	9,818076388	RPS17;RPL32;RPS19;RPS8;RPS5;RPL13A;RPL35A;RPS20;RPSA;TPT1
N-Nitrosodiethylamine-1.67_mg/kg_in_Saline-Rat-Bone_marrow-3d-up	10/302	0,013377769	0,068506705	0	0	2,275778885	9,818076388	RPL32;RPS19;RPS8;RPS5;RPL35A;RPSA;RPS3A;RPL10A;SEPP1;TPT1
Flutamide-15_mg/kg_in_Corn_Oil-Rat-Kidney-0.25d-up	11/348	0,013393908	0,06854478	0	0	2,172453292	9,369694003	RPS17;RPL32;RPS19;RPS6;RPS20;RPSA;RPS3A;RPL10A;SEPP1;RPL39;TPT1
Penciclovir-1200_mg/kg_in_Corn_Oil-Rat-Bone_marrow-1d-up	11/348	0,013393908	0,068500271	0	0	2,172453292	9,369694003	EEF1B2;C1QA;RPS17;RPL32;RPS19;RPS8;NKG7;RPS20;RPS3A;RPL39;TPT1
Zopiclone-414_mg/kg_in_Water-Rat-Liver-0.25d-up	11/349	0,013657187	0,069801429	0	0	2,166228498	9,300679089	C1QA;RPS17;RPL32;RPS19;RPS8;RPS6;RPL35A;RPS20;RPSA;RPS3A;RPL10A
Acrolein-20_mg/kg_in_Water-Rat-Kidney-3d-dn	10/303	0,013662826	0,069784968	0	0	2,268268064	9,737848387	EEF1B2;RPS17;RPL32;RPS19;ARL2BP;RPS8;RPS6;RPL35A;RPSA;RPL39
Amiodarone-147_mg/kg_in_CMC-Rat-Kidney-5d-up	10/303	0,013662826	0,069739742	0	0	2,268268064	9,737848387	RPS19;FEM1B;RPS6;RPS20;RPSA;RPS3A;RPL10A;SEPP1;RPL39;TPT1
Amlodipine-0.2_mg/kg_in_Corn_Oil-Rat-Liver-1d-up	10/303	0,013662826	0,069694573	0	0	2,268268064	9,737848387	EEF1B2;RPS17;RPL32;RPS19;RPS8;COL5A3;RPS5;RPL35A;RPS3A;SEPP1
Amoxapine-313_mg/kg_in_CMC-Rat-Liver-1d-up	10/303	0,013662826	0,069649464	0	0	2,268268064	9,737848387	EEF1B2;RPS17;RPL32;RPS5;RPS6;RPL35A;RPSA;RPS3A;RPL39;TPT1
Artemisinin-2000_mg/kg_in_CMC-Rat-Liver-5d-up	10/303	0,013662826	0,069604412	0	0	2,268268064	9,737848387	RPS17;RPS19;RPS8;FEM1B;RPS6;RPS20;RPSA;RPS3A;SEPP1;RPL39
Carvedilol-50_uM_in_DMSO-Rat-Primary_rat_hepatocytes-0.67d-up	10/303	0,013662826	0,069559419	0	0	2,268268064	9,737848387	RPS17;RPL32;RPS19;ARL2BP;RPS6;RPS20;RPS3A;RPL10A;RPL39;PDRG1
Clonazepam-2500_mg/kg_in_CMC-Rat-Heart-3d-dn	10/303	0,013662826	0,069514484	0	0	2,268268064	9,737848387	RPS17;RPL32;RPS19;RPS6;ARL6IP5;RPSA;RPS3A;RPL10A;SEPP1;PDRG1
Cortisone-206_mg/kg_in_CMC-Rat-Liver-5d-dn	10/303	0,013662826	0,069469607	0	0	2,268268064	9,737848387	EEF1B2;RPS17;RPL32;RPS19;RPS6;ARL6IP5;RPS20;RPS3A;SEPP1;RPL39
Erythromycin-1500_mg/kg_in_CMC-Rat-Liver-1d-dn	10/303	0,013662826	0,069424788	0	0	2,268268064	9,737848387	EEF1B2;RPL32;RPS19;CLP1;RPS6;RPS20;RPS3A;RPL10A;SEPP1;RPL39
Lacidipine-1_mg/kg_in_Corn_Oil-Rat-Heart-1d-up	10/303	0,013662826	0,069380027	0	0	2,268268064	9,737848387	C1QA;RPS17;RPL32;RPS19;RPS20;RPS3A;RAPGEF5;RPL39;TPT1;PDRG1
Mianserin-116_mg/kg_in_Corn_Oil-Rat-Heart-1d-up	10/303	0,013662826	0,069335323	0	0	2,268268064	9,737848387	C1QA;RPS17;RPL32;RPS19;RPS6;ARL6IP5;RPS20;RPS3A;RPL39;TPT1
Nimetazepam-122_mg/kg_in_CMC-Rat-Liver-5d-dn	10/303	0,013662826	0,069290677	0	0	2,268268064	9,737848387	EEF1B2;RPS17;RPL32;RPS8;RPS6;ARL6IP5;RPL35A;RPS20;SEPP1;TPT1
Stavudine-58_mg/kg_in_Water-Rat-Liver-1d-up	10/303	0,013662826	0,069246088	0	0	2,268268064	9,737848387	EEF1B2;RPS17;RPL32;RPS19;RPS6;RPS20;RPSA;RPS3A;SEPP1;RPL39
Sulpiride-667_uM_in_DMSO-Rat-Primary_rat_hepatocytes-1d-dn	10/303	0,013662826	0,069201557	0	0	2,268268064	9,737848387	CXCL11;RPS19;RPS8;RPS5;ARL6IP5;RPL35A;RPSA;SEPP1;GOLGA7;TPT1
Isotretinoin-500_uM_in_DMSO-Rat-Primary_rat_hepatocytes-1d-up	9/259	0,013907205	0,070394051	0	0	2,388249811	10,21059966	EEF1B2;ARL2BP;RPS6;ARL6IP5;RPS20;RPSA;RPS3A;RPL10A;PDRG1
Loxoprofen-18_mg/kg_in_Corn_Oil-Rat-Kidney-0.25d-dn	9/259	0,013907205	0,07034884	0	0	2,388249811	10,21059966	EEF1B2;RPS17;RPL32;CLP1;RPS8;FUNDC1;ARL6IP5;RPL35A;SEPP1
Acetaminophen-486_mg/kg_in_Corn_Oil-Rat-Liver-1d-up	10/304	0,013952419	0,070532252	0	0	2,260806656	9,658397557	RPS17;RPL32;RPS19;RPS8;RPS5;RPS6;RPS20;RPSA;RPS3A;RPL10A
Allyl_Alcohol-16_mg/kg_in_Saline-Rat-Kidney-1d-up	10/304	0,013952419	0,07048701	0	0	2,260806656	9,658397557	RPS17;RPL32;RPS6;RPL35A;RPS20;RPSA;RPS3A;SEPP1;RPL39;TPT1
Aspirin-35_mg/kg_in_Corn_Oil-Rat-Kidney-3d-up	10/304	0,013952419	0,070441826	0	0	2,260806656	9,658397557	C1QA;RPS17;RPL32;ARL2BP;RPL35A;RPS20;RPSA;RPS3A;RAPGEF5;RPL39
Daunorubicin-3.25_mg/kg_in_Saline-Rat-Heart-5d-up	10/304	0,013952419	0,0703967	0	0	2,260806656	9,658397557	EEF1B2;CXCL11;RPL32;RPS19;FUNDC1;RPS6;RPS20;RPL10A;SEPP1;RPL39
Doxifluridine-1230_mg/kg_in_CMC-Rat-Bone_marrow-3d-dn	10/304	0,013952419	0,070351632	0	0	2,260806656	9,658397557	EEF1B2;RPS17;FEM1B;RPS5;RPS6;NKG7;RPSA;RPS3A;RPL10A;RPL39
Idarubicin-0.625_mg/kg_in_Saline-Rat-Heart-5d-dn	10/304	0,013952419	0,070306621	0	0	2,260806656	9,658397557	EEF1B2;RPS19;RPS8;RPS6;ARL6IP5;RPS20;RAPGEF5;RPL10A;SEPP1;RPL39
Lead_(II)_Acetate-2_mg/kg_in_Saline-Rat-Kidney-28d-up	10/304	0,013952419	0,070261668	0	0	2,260806656	9,658397557	EEF1B2;RPS17;CXCL11;RPL32;RPS19;RPS8;RPS5;RPS20;RPS3A;RPL39
Nafenopin-338_mg/kg_in_Corn_Oil-Rat-Liver-3d-up	10/304	0,013952419	0,070216773	0	0	2,260806656	9,658397557	EEF1B2;RPS17;RPL32;RPS19;RPS8;RPS6;RPSA;RPS3A;RPL10A;TPT1
Nisoldipine-15_mg/kg_in_Water-Rat-Liver-5d-dn	10/304	0,013952419	0,070171934	0	0	2,260806656	9,658397557	EEF1B2;C1QA;RPS17;RPL32;RPS8;RPS6;RPSA;RPS3A;RPL10A;TPT1
Penciclovir-1200_mg/kg_in_Corn_Oil-Rat-Bone_marrow-3d-up	10/304	0,013952419	0,070127153	0	0	2,260806656	9,658397557	EEF1B2;C1QA;RPL32;RPS8;ARL6IP5;NKG7;RPL35A;RPS20;RPS3A;SLC16A3
Phenytoin-46_mg/kg_in_Water-Rat-Heart-5d-dn	10/304	0,013952419	0,070082429	0	0	2,260806656	9,658397557	RPS17;RPL32;RPS19;ARL2BP;RPS8;RPS6;RPS20;RPSA;RPS3A;TPT1
Clonazepam-2500_mg/kg_in_CMC-Rat-Liver-0.25d-up	10/305	0,014246588	0,071514422	0	0	2,253394175	9,579714555	RPS17;RPL32;RPS19;RPS8;RPS6;RPS20;RPSA;RPS3A;RPL10A;RPL39
Cyclosporin_A-350_mg/kg_in_Corn_Oil-Rat-Liver-5d-up	10/305	0,014246588	0,071468872	0	0	2,253394175	9,579714555	EEF1B2;RPS17;RPL32;RPS19;RPS5;RPS6;RPS20;RPSA;RPL39;TPT1
Cyproheptadine-37_mg/kg_in_CMC-Rat-Heart-3d-dn	10/305	0,014246588	0,071423379	0	0	2,253394175	9,579714555	EEF1B2;RPS17;RPL32;RPS8;RPS6;RPL35A;RPS20;RPS3A;RPL10A;RPL39
Gabapentin-100_mg/kg_in_Water-Rat-Heart-3d-up	10/305	0,014246588	0,071377944	0	0	2,253394175	9,579714555	RPS19;ARL2BP;FEM1B;FUNDC1;RPS6;RPSA;RAPGEF5;RPL10A;GOLGA7;TPT1
Gallamine_Triethiodide-2.5_mg/kg_in_Saline-Rat-Heart-5d-up	10/305	0,014246588	0,071332567	0	0	2,253394175	9,579714555	C1QA;RPS17;RPL32;RPS8;RPS6;RPL35A;RPS20;RPS3A;RPL39;TPT1
Hydrocortisone-56_mg/kg_in_Corn_Oil-Rat-Bone_marrow-5d-dn	10/305	0,014246588	0,071287248	0	0	2,253394175	9,579714555	EEF1B2;C1QA;RPS17;CLP1;RPS8;FUNDC1;RPS3A;RPL10A;RPL39;TPT1
Lacidipine-14_mg/kg_in_Corn_Oil-Rat-Heart-1d-up	10/305	0,014246588	0,071241986	0	0	2,253394175	9,579714555	EEF1B2;C1QA;RPS17;CXCL11;RPL32;RPS19;RPS6;RPS3A;RPL10A;RPL39
Lamivudine-35_mg/kg_in_Water-Rat-Liver-1d-up	10/305	0,014246588	0,071196782	0	0	2,253394175	9,579714555	EEF1B2;RPS17;RPL32;RPS6;RPL35A;RPS20;RPS3A;RPL10A;RPL39;TPT1
Mefenamic_Acid-93_mg/kg_in_Corn_Oil-Rat-Liver-5d-up	10/305	0,014246588	0,071151635	0	0	2,253394175	9,579714555	C1QA;RPS17;RPS8;RPS5;RPL35A;RPS20;RPSA;SEPP1;RPL39;TPT1
Norepinephrine-1.75_mg/kg_in_Saline-Rat-Heart-3d-up	10/305	0,014246588	0,071106545	0	0	2,253394175	9,579714555	C1QA;RPS19;RPS8;RPSA;RPS3A;RAPGEF5;RPL10A;SEPP1;RPL39;TPT1
Sulbactam-671_mg/kg_in_Saline-Rat-Kidney-3d-up	10/305	0,014246588	0,071061513	0	0	2,253394175	9,579714555	EEF1B2;RPS17;CXCL11;RPL32;RPS19;ARL6IP5;RPS20;RPSA;RAPGEF5;SEPP1
Sulindac-64_mg/kg_in_Corn_Oil-Rat-Liver-1d-up	10/305	0,014246588	0,071016537	0	0	2,253394175	9,579714555	EEF1B2;RPS17;RPL32;RPS19;RPS8;RPS20;RPS3A;RPL10A;RPL39;TPT1
Terbinafine-2000_mg/kg_in_CMC-Rat-Liver-5d-dn	10/305	0,014246588	0,070971618	0	0	2,253394175	9,579714555	EEF1B2;C1QA;RPL32;RPS19;RPS6;ARL6IP5;RPL35A;RPS3A;SEPP1;TPT1
Vinblastine-0.08_mg/kg_in_Saline-Rat-Bone_marrow-0.25d-up	10/305	0,014246588	0,070926756	0	0	2,253394175	9,579714555	RPL32;RPS19;RPS8;RPS5;FUNDC1;RPS6;RPL13A;RPL35A;RPS20;RPSA
Prednisolone-184_mg/kg_in_Water-Rat-Kidney-1d-dn	8/217	0,014407527	0,071682678	0	0	2,533770409	10,74319802	RPS17;RPL32;RPS19;RPL35A;RPSA;RPS3A;RPL39;SEPP1
Benzyl_Acetate-1868_mg/kg_in_CMC-Rat-Kidney-1d-up	10/306	0,014545377	0,072322849	0	0	2,246030142	9,501790175	RPS17;CXCL11;CLP1;RPS8;RPS6;RPS20;RPSA;RPS3A;TOMM20;TPT1
Carbamazepine-490_mg/kg_in_CMC-Rat-Liver-3d-up	10/306	0,014545377	0,072277219	0	0	2,246030142	9,501790175	RPS17;RPL32;RPS19;RPS8;RPS6;RPS20;RPSA;RPS3A;RPL10A;RPL39
Catechol-40_mg/kg_in_Saline-Rat-Bone_marrow-0.25d-dn	10/306	0,014545377	0,072231647	0	0	2,246030142	9,501790175	RPL32;RPS19;RPS8;RPS6;RPS20;RPSA;RPS3A;RPL10A;RPL39;TPT1
Chlorambucil-4.5_mg/kg_in_Corn_Oil-Rat-Bone_marrow-3d-up	10/306	0,014545377	0,072186133	0	0	2,246030142	9,501790175	EEF1B2;C1QA;RPS8;RPS5;RPL35A;RPS20;SEPP1;RPL39;SLC16A3;TPT1
Clomiphene-250_mg/kg_in_CMC-Rat-Liver-3d-up	10/306	0,014545377	0,072140675	0	0	2,246030142	9,501790175	C1QA;RPS17;RPS19;RPS6;RPS20;RPSA;RPS3A;RPL10A;RPL39;TPT1
Diethylstilbestrol-280_mg/kg_in_Corn_Oil-Rat-Kidney-3d-up	10/306	0,014545377	0,072095275	0	0	2,246030142	9,501790175	CXCL11;RPS19;ARL2BP;RPS6;RPS20;RPS3A;RPL10A;RPL39;GOLGA7;TPT1
Indomethacin-4.5_mg/kg_in_Water-Rat-Liver-1d-up	10/306	0,014545377	0,072049932	0	0	2,246030142	9,501790175	EEF1B2;RPS17;RPL32;RPS5;RPL35A;RPS20;RPS3A;RPL10A;RPL39;TPT1
Oxaprozin-1500_mg/kg_in_CMC-Rat-Kidney-5d-up	10/306	0,014545377	0,072004646	0	0	2,246030142	9,501790175	RPS17;RPL32;RPS19;RPS8;RPS6;RPS20;RAPGEF5;RPL10A;RPL39;TPT1
Vinorelbine-1.5_mg/kg_in_Saline-Rat-Heart-5d-dn	10/306	0,014545377	0,071959417	0	0	2,246030142	9,501790175	RPL32;RPS8;FUNDC1;RPS6;ARL6IP5;RPSA;RPS3A;SEPP1;RPL39;TPT1
Lead_(II)_Acetate-600_mg/kg_in_Saline-Rat-Spleen-3d-dn	9/261	0,014555799	0,071965773	0	0	2,369949046	10,02432937	EEF1B2;RPS17;RPL32;RPS8;RPS5;RPL35A;RPSA;RPS3A;TPT1
Tenidap-41_uM_in_DMSO-Rat-Primary_rat_hepatocytes-1d-dn	11/353	0,014749695	0,072878667	0	0	2,141681999	9,030472617	CXCL11;RPS19;RPS8;RPS6;ARL6IP5;RPS20;RPSA;RPL10A;SEPP1;RPL39;TPT1
Sodium_Selenite-2_mg/kg_in_Water-Rat-Kidney-1d-dn	8/218	0,014774428	0,072955104	0	0	2,522147609	10,63049266	RPS17;RPL32;RPS19;RPS6;RPL35A;RPS20;RPSA;RPL39
Azathioprine-54_mg/kg_in_Water-Rat-Liver-1d-up	10/307	0,014848828	0,07327655	0	0	2,238714083	9,42461535	EEF1B2;RPS17;RPL32;RPS19;RPS6;RPS20;RPS3A;RPL10A;RPL39;TPT1
Beta-Estradiol-150_mg/kg_in_Corn_Oil-Rat-Kidney-1d-dn	10/307	0,014848828	0,073230666	0	0	2,238714083	9,42461535	RPS17;RPL32;RPS19;RPS6;RPSA;RPL10A;SEPP1;RPL39;GOLGA7;TPT1
Doxifluridine-1230_mg/kg_in_CMC-Rat-Bone_marrow-0.25d-up	10/307	0,014848828	0,073184839	0	0	2,238714083	9,42461535	RPS17;RPL32;RPS19;RPS8;FUNDC1;RPS6;RPL35A;RPS20;RPL39;TPT1
Furosemide-375_mg/kg_in_CMC-Rat-Kidney-5d-up	10/307	0,014848828	0,07313907	0	0	2,238714083	9,42461535	EEF1B2;RPL32;RPS8;ARL6IP5;RPL13A;RPL35A;RPS20;RPSA;RPL39;TPT1
Ifosfamide-143_mg/kg_in_Saline-Rat-Liver-5d-up	10/307	0,014848828	0,073093358	0	0	2,238714083	9,42461535	RPS17;RPL32;ARL2BP;RPS8;RPS6;RPL35A;RPS20;RPS3A;RPL39;TPT1
Lipopolysaccharide_E._Coli_O55:B5-1.25_mg/kg_in_Saline-Rat-Liver-1d-up	10/307	0,014848828	0,073047703	0	0	2,238714083	9,42461535	RPS17;RPL32;RPS19;RPS8;RPS20;RPSA;RPL10A;SEPP1;RPL39;TPT1
Sildenafil-2.5_mg/kg_in_Water-Rat-Liver-5d-up	10/307	0,014848828	0,073002106	0	0	2,238714083	9,42461535	RPS17;RPL32;RPS19;RPS8;FEM1B;RPS6;RPS20;RPS3A;RPL39;TPT1
Tetracaine-24_mg/kg_in_Corn_Oil-Rat-Heart-1d-up	10/307	0,014848828	0,072956565	0	0	2,238714083	9,42461535	EEF1B2;RPS17;RPL32;RPS8;RPS5;ARL6IP5;RPL35A;RPS20;RPS3A;TPT1
Thioacetamide-200_mg/kg_in_Saline-Rat-Liver-1d-up	10/307	0,014848828	0,07291108	0	0	2,238714083	9,42461535	EEF1B2;RPS19;RPS8;RPS6;RPS20;RPSA;RPS3A;RPL10A;RPL39;TPT1
Chloroxylenol-1915_mg/kg_in_Corn_Oil-Rat-Liver-1d-up	9/262	0,014888352	0,073059603	0	0	2,360903439	9,932736506	EEF1B2;C1QA;RPS17;ARL2BP;RPS8;RPS5;RPS20;RPSA;SEPP1
Ramipril-620_uM_in_DMSO-Rat-Primary_rat_hepatocytes-1d-up	9/262	0,014888352	0,073014112	0	0	2,360903439	9,932736506	EEF1B2;RPS17;RPL32;RPS8;RPS5;RPL35A;RPS20;SEPP1;PDRG1
Azathioprine-0.5_uM_in_DMSO-Rat-Primary_rat_hepatocytes-1d-dn	11/354	0,015032844	0,073676836	0	0	2,13563205	8,964353767	RPS17;RPL32;RPS19;ARL2BP;CLP1;RPS8;RPS5;RPS20;RPS3A;RPL39;TPT1
Chlorambucil-4.5_mg/kg_in_Corn_Oil-Rat-Liver-3d-dn	11/354	0,015032844	0,073631017	0	0	2,13563205	8,964353767	EEF1B2;C1QA;RPS17;RPL32;RPS19;RPS6;RPSA;RPS3A;RPL10A;RPL39;TPT1
Phenothiazine-36_mg/kg_in_Corn_Oil-Rat-Liver-3d-up	11/354	0,015032844	0,073585255	0	0	2,13563205	8,964353767	EEF1B2;C1QA;RPL32;RPS8;RPS5;RPS6;RPL35A;RPS20;RPSA;RPL39;TPT1
Epinephrine-0.0375_mg/kg_in_Saline-Rat-Heart-3d-dn	8/219	0,015148078	0,074103267	0	0	2,510630953	10,51924654	RPS19;ARL2BP;RPS8;FUNDC1;ARL6IP5;RPS20;SEPP1;TPT1
Acetaminophen-486_mg/kg_in_Corn_Oil-Rat-Liver-5d-dn	10/308	0,015156983	0,074100808	0	0	2,23144553	9,348181146	EEF1B2;C1QA;RPS17;RPL32;RPS19;RPS6;RPS3A;SEPP1;RPL39;TPT1
Azauridine-1500_mg/kg_in_CMC-Rat-Bone_marrow-1d-dn	10/308	0,015156983	0,07405484	0	0	2,23144553	9,348181146	EEF1B2;C1QA;RPS17;RPL32;RPS8;FEM1B;RPS6;RPS3A;RPL39;TPT1
Caffeine-93_mg/kg_in_Water-Rat-Brain-5d-up	10/308	0,015156983	0,074008929	0	0	2,23144553	9,348181146	EEF1B2;RPS17;RPS19;ARL2BP;CLP1;RPS8;ARL6IP5;RPSA;RPS3A;TPT1
Carvedilol-2000_mg/kg_in_Corn_Oil-Rat-Liver-5d-up	10/308	0,015156983	0,073963074	0	0	2,23144553	9,348181146	EEF1B2;RPL32;RPS19;ARL2BP;RPS6;RPS20;RPL10A;SEPP1;RPL39;TPT1
Cyproterone_Acetate-2500_mg/kg_in_CMC-Rat-Liver-0.25d-up	10/308	0,015156983	0,073917277	0	0	2,23144553	9,348181146	RPS17;RPL32;RPS19;RPS6;ARL6IP5;RPS20;RPSA;RPL10A;RPL39;GOLGA7
Erythromycin-1500_mg/kg_in_CMC-Rat-Kidney-1d-dn	10/308	0,015156983	0,073871536	0	0	2,23144553	9,348181146	RPL32;ARL2BP;RPS6;ARL6IP5;RPS20;RPSA;RPL10A;SEPP1;RPL39;TOMM20
Hydrocortisone-56_mg/kg_in_Corn_Oil-Rat-Kidney-5d-dn	10/308	0,015156983	0,073825851	0	0	2,23144553	9,348181146	C1QA;RPS17;RPL32;RPS8;RPSA;RPS3A;RAPGEF5;RPL10A;SEPP1;RPL39

Ritonavir-1200_mg/kg_in_CMC-Rat-Kidney-5d-up	10/308	0,015156983	0,073780224	0	0	2,23144553	9,348181146	C1QA;CXCL11;RPS19;RPS6;RPSA;RPS3A;RAPGEF5;RPL10A;RPL39;TPT1
Thalidomide-113_mg/kg_in_CMC-Rat-Liver-1d-up	10/308	0,015156983	0,073734652	0	0	2,23144553	9,348181146	EEF1B2;RPS17;RPL32;RPS8;RPS5;RPL35A;RPSA;RPS3A;RPL10A;TPT1
Diclofenac-3.5_mg/kg_in_Corn_Oil-Rat-Kidney-1d-dn	9/263	0,015226479	0,074027003	0	0	2,35192662	9,842152856	EEF1B2;RPS17;RPL32;CLP1;RPS8;RPL35A;RPS3A;SEPP1;TOMM20
Leflunomide-60_mg/kg_in_Corn_Oil-Rat-Kidney-3d-up	9/263	0,015226479	0,073981336	0	0	2,35192662	9,842152856	RPS17;RPL32;ARL2BP;RPS8;FUNDC1;RPL35A;RPS20;RPS3A;RAPGEF5
Modafinil-325_mg/kg_in_Saline-Rat-Liver-0.25d-up	9/263	0,015226479	0,073935724	0	0	2,35192662	9,842152856	RPS17;RPL32;RPS19;RPS8;RPS6;RPSA;RPS3A;RPL10A;SEPP1
N-Nitrosodiethylamine-34_mg/kg_in_Saline-Rat-Bone_marrow-1d-dn	9/263	0,015226479	0,073890169	0	0	2,35192662	9,842152856	EEF1B2;RPS17;RPL32;RPS8;RPS5;RPL35A;RPL10A;RPL39;TPT1
Bisphenol_A-50_mg/kg_in_Corn_Oil-Rat-Liver-0.25d-up	10/309	0,015469884	0,075025129	0	0	2,224224024	9,27247876	RPS17;RPL32;RPS19;RPL35A;RPS20;RPSA;RPS3A;RPL10A;SEPP1;RPL39
Cadmium_Chloride-2_mg/kg_in_Saline-Rat-Kidney-14d-up	10/309	0,015469884	0,07497896	0	0	2,224224024	9,27247876	RPL32;ARL2BP;RPS8;RPS6;RPS20;RPSA;RPS3A;RPL10A;RPL39;TPT1
Clopidogrel-400_mg/kg_in_Corn_Oil-Rat-Bone_marrow-3d-up	10/309	0,015469884	0,074932847	0	0	2,224224024	9,27247876	C1QA;RPL32;ARL2BP;CLP1;RPS8;RPS5;RPS6;RPL35A;RPS20;SLC16A3
Closantel-22_mg/kg_in_CMC-Rat-Liver-1d-up	10/309	0,015469884	0,074886791	0	0	2,224224024	9,27247876	EEF1B2;RPS17;CXCL11;RPL32;RPS6;RPS20;RPSA;RPS3A;RPL10A;TPT1
Etoposide-188_mg/kg_in_Corn_Oil-Rat-Kidney-5d-up	10/309	0,015469884	0,074840792	0	0	2,224224024	9,27247876	ARL2BP;CLP1;RPS8;RPS6;RPS20;RPSA;RPS3A;RPL10A;RPL39;TPT1
Hexachloro-13-Butadiene-50_mg/kg_in_Corn_Oil-Rat-Kidney-1d-up	10/309	0,015469884	0,074794849	0	0	2,224224024	9,27247876	RPS17;RPL32;RPS8;RPS5;ARL6IP5;RPL35A;RPSA;RPS3A;TOMM20;TPT1
Irinotecan-5_mg/kg_in_Saline-Rat-Heart-3d-dn	10/309	0,015469884	0,074748963	0	0	2,224224024	9,27247876	EEF1B2;RPS17;RPL32;RPS19;ARL2BP;RPS8;RPS6;ARL6IP5;RPS3A;TPT1
Meloxicam-33_mg/kg_in_Corn_Oil-Rat-Liver-0.25d-up	10/309	0,015469884	0,074703133	0	0	2,224224024	9,27247876	EEF1B2;RPS17;RPL32;RPS19;RPS6;ARL6IP5;RPL35A;RPS20;RPSA;RPL39
Microcystin-LR-0.07_uM_in_DMSO-Rat-Primary_rat_hepatocytes-0.67d-up	10/309	0,015469884	0,074657359	0	0	2,224224024	9,27247876	EEF1B2;RPS19;ARL2BP;CLP1;RPS6;ARL6IP5;RPS20;RPSA;RPL39;PDRG1
Sulconazole-40_uM_in_DMSO-Rat-Primary_rat_hepatocytes-0.67d-up	10/309	0,015469884	0,074611641	0	0	2,224224024	9,27247876	EEF1B2;RPS17;RPL32;RPS19;RPS5;RPS6;RPS20;RPSA;RPL10A;RPL39
Tranilast-300_mg/kg_in_CMC-Rat-Liver-5d-dn	10/309	0,015469884	0,074565979	0	0	2,224224024	9,27247876	RPS17;RPL32;RPS19;RPS8;RPS5;RPS6;RPL35A;RPS20;RPSA;RPL10A
Lead(IV)_Acetate-600_mg/kg_in_Saline-Rat-Liver-5d-dn	9/264	0,01557023	0,07500375	0	0	2,343017807	9,752564495	EEF1B2;RPS17;RPL32;RPS19;RPS8;RPS6;RPSA;RPS3A;SEPP1
Secobarbital-20_mg/kg_in_Water-Rat-Liver-0.25d-up	11/356	0,015611411	0,075156156	0	0	2,123634117	8,833793749	EEF1B2;RPS17;RPL32;RPS19;RPS8;RPS6;RPL35A;RPS20;RPSA;SEPP1;GOLGA7
4-Nitrobenzoic_Acid-1470_mg/kg_in_CMC-Rat-Kidney-1d-up	10/310	0,015787573	0,075957805	0	0	2,217049108	9,197499518	RPS17;RPS19;RPS8;RPS6;RPS20;RPSA;RPS3A;RAPGEF5;RPL10A;RPL39
Acyclovir-980_mg/kg_in_Saline-Rat-Liver-5d-dn	10/310	0,015787573	0,075911432	0	0	2,217049108	9,197499518	EEF1B2;RPL32;RPS19;RPS5;RPS6;RPSA;RPS3A;RPL10A;SEPP1;RPL39
Bromobenzene-439_mg/kg_in_CMC-Rat-Kidney-5d-up	10/310	0,015787573	0,075865117	0	0	2,217049108	9,197499518	RPL32;RPS8;RPS5;RPS6;RPSA;RPS3A;RAPGEF5;RPL10A;SEPP1;TPT1
Gemfibrozil-700_mg/kg_in_Corn_Oil-Rat-Skeletal_muscle-3d-up	10/310	0,015787573	0,075818857	0	0	2,217049108	9,197499518	RPS17;RPL32;ARL2BP;RPS8;RPS5;FUNDC1;RPS6;RPL35A;RPS20;RPS3A
Hydralazine-280_mg/kg_in_Water-Rat-Heart-1d-dn	10/310	0,015787573	0,075772655	0	0	2,217049108	9,197499518	EEF1B2;RPS17;RPL32;RPS19;RPS5;RPL35A;RPS20;RPS3A;RPL10A;RPL39
Hydrocortisone-56_mg/kg_in_Corn_Oil-Rat-Heart-5d-dn	10/310	0,015787573	0,075726508	0	0	2,217049108	9,197499518	RPS17;RPL32;RPS19;ARL2BP;RPS8;COL5A3;RPS6;RPSA;RPS3A;RPL39
Neomycin-877_mg/kg_in_Corn_Oil-Rat-Liver-3d-up	10/310	0,015787573	0,075680418	0	0	2,217049108	9,197499518	RPS17;RPL32;RPS19;RPS8;RPL35A;RPS20;RPS3A;RPL39;GOLGA7;TPT1
Nystatin-134_mg/kg_in_Corn_Oil-Rat-Kidney-1d-up	10/310	0,015787573	0,075634383	0	0	2,217049108	9,197499518	C1QA;RPS19;ARL2BP;FEM1B;RPS6;ARL6IP5;RPS20;RPSA;SEPP1;GOLGA7
Testosterone-375_mg/kg_in_CMC-Rat-Liver-3d-up	10/310	0,015787573	0,075588405	0	0	2,217049108	9,197499518	EEF1B2;RPS17;RPL32;RPS8;RPS5;RPSA;RPS3A;RPL10A;SEPP1;TPT1
Thioguanine-24_mg/kg_in_Corn_Oil-Rat-Liver-5d-up	10/310	0,015787573	0,075542482	0	0	2,217049108	9,197499518	EEF1B2;RPS17;RPL32;RPS19;RPS8;RPS20;RPSA;RPS3A;RPL39;TPT1
Tolazamide-1500_mg/kg_in_CMC-Rat-Liver-5d-up	10/310	0,015787573	0,075496616	0	0	2,217049108	9,197499518	EEF1B2;RPS17;RPS19;RPS8;RPS6;RPS20;RPSA;RPS3A;RPL10A;SEPP1
Vinblastine-0.3_mg/kg_in_Saline-Rat-Liver-3d-up	10/310	0,015787573	0,075450805	0	0	2,217049108	9,197499518	RPS17;RPL32;RPS19;RPS8;RPS6;RPS20;RPSA;RPS3A;RPL10A;TPT1
Dexchlorpheniramine-47_mg/kg_in_Water-Rat-Heart-3d-up	11/357	0,015906899	0,075974974	0	0	2,117685562	8,769340986	RPS19;ARL2BP;RPS8;FUNDC1;RPS6;ARL6IP5;RPS20;RPSA;RAPGEF5;RPL39;GOLGA7
[truncated: 1,041,432 more chars]
